# Targeting Mitochondrial Metabolism in Clear Cell Carcinoma of the Ovaries

**DOI:** 10.3390/ijms22094750

**Published:** 2021-04-29

**Authors:** Xiaonan Zhang, Mihir Shetty, Valentino Clemente, Stig Linder, Martina Bazzaro

**Affiliations:** 1 Department of Immunology, Genetics and Pathology, Uppsala University, 75185 Uppsala, Sweden; xiaonan.zhang@igp.uu.se; 2 Masonic Cancer Center and Department of Obstetrics, Gynecology and Women’s Health, University of Minnesota, Minneapolis, MN 55455, USA; shett036@umn.edu (M.S.); vclement@umn.edu (V.C.); 3 Department of Biomedical and Clinical Sciences (BKV), Linköping University, 58183 Linköping, Sweden; stig.linder@liu.se; 4 Department of Oncology and Pathology, Karolinska Institute, 17176 Stockholm, Sweden

**Keywords:** ARID1A, OCCC, mitochondria, ovarian cancer

## Abstract

Ovarian clear cell carcinoma (OCCC) is a rare but chemorefractory tumor. About 50% of all OCCC patients have inactivating mutations of *ARID1A*, a member of the SWI/SNF chromatin-remodeling complex. Members of the SWI/SNF remodeling have emerged as regulators of the energetic metabolism of mammalian cells; however, the role of ARID1A as a modulator of the mitochondrial metabolism in OCCCs is yet to be defined. Here, we show that ARID1A loss results in increased mitochondrial metabolism and renders *ARID1A*-mutated cells increasingly and selectively dependent on it. The increase in mitochondrial activity following ARID1A loss is associated with increase in c-Myc expression and increased mitochondrial number and reduction of their size consistent with a higher mitochondrial cristae/outer membrane ratio. Significantly, preclinical testing of the complex I mitochondrial inhibitor IACS-010759 showed it extends overall survival in a preclinical model of *ARID1A*-mutated OCCC. These findings provide for the targeting mitochondrial activity in *ARID1A*-mutated OCCCs.

## 1. Introduction

Ovarian clear cell carcinoma (OCCC) has a 5-year overall survival of less than 10% and is often associated with inactivating mutations of ARID1A, a component of the SWI/SNF nucleosome remodeling complex. Over half of the patients with OCCC harbor loss-of-function mutations in ARID1A [1]. Mutations of members of the SWI/SNF remodeling complex are found in approximately 20% of human cancers, including another untreatable though rare ovarian cancer histotype, the cell carcinoma of the ovary, hypercalcemic type (SCCOHT), where inactivating mutations of SMARCA4 are found in 100% of the patients [2].

The SWI/SNF remodeling complex has been shown to control the energetic metabolism of mammalian cells including cancer cells. For instance: (a) a study conducted in lung cancer shows that mutations in SMARCA4, a SWI/SNF complex member, induced targetable dependence upon mitochondrial inhibitors [1]; (b) a study conducted in ovarian cancer shows that OCCC-derived cells have higher mitochondrial respiration as compared to ovarian cancer cell lines derived from other histotypes [3]; (c) a study conducted in ovarian cancer shows that loss of ARID1A leads to sensitivity to ROS-inducing agents [4]; and (d) a study shows that targeting the vulnerability to glutathione metabolism can be a strategy to treat ARID1A-deficient human cancers [5]. Today, the role of ARID1A as a modulator of specific mitochondrial metabolism in OCCCs remains elusive.

Mitochondrial dynamics is the process through which mitochondria, once formed, undergo the cycling process of fusion and fission. Tight regulation of mitochondrial dynamics is fundamental for cell functions, and its disruption has been associated with aging, neurogenerative diseases, and cancer. [6,7,8,9,10]. For instance, several of studies show that a fragmented mitochondrial phenotype is essential in many human tumors [11,12,13,14,15]. Many recent reports also show that increased mitochondrial fission is associated with higher mitochondrial activity in cancer cells [6,16,17]. This seems to be due to a higher ratio of cristae to the outer membrane surface of the mitochondria [18,19,20,21]. 

c-Myc has been shown to be involved in regulating mitochondrial biogenesis and trafficking. [22,23]. In prostate cancer, c-Myc has been shown to regulate cancer metastasis via regulating mitochondrial trafficking [24] and to control mitochondrial fission via upregulating the mitochondrial fission factor (MFF) [25]. In neuroblastomas, n-Myc has been shown to regulate both mitochondrial trafficking and dynamics [26]. c-Myc amplification is common in ovarian cancer [27] but is particularly common in the OCCC subtype, where over 40% of patients have increased c-Myc [28]. In mouse fibroblasts, c-Myc has been shown to be a direct target of ARID1A [29]. In OCCCs, ARID1A has been shown to control global transcription [30]. In another highly chemoresistant ovarian cancer histotype, high-grade serous carcinoma (HGSC), active c-Myc transcription has been shown to be dependent upon continuous transcription [27].

Inhibition of mitochondrial bioenergetics has raised increasing interest in the field of cancer therapeutics [31,32,33,34,35,36]. A number of different inhibitors are being evaluated, including inhibitors of the respiratory chain, the tricarboxylic acid cycle, and mtDNA [36]. IACS-010759 is a clinical-grade small-molecule inhibitor of complex I of the electron transport chain [32]. IACS-010759 has been shown to selectively kill tumor cells that depend on OXPHOS, both in vitro and in preclinical models of human cancers with no cytotoxicity at tolerated doses in normal cells [32,37,38,39,40,41,42,43,44,45].

Here, we show that ARID1A-deficient cells upregulate OXPHOS genes and pathways and this corresponds to an upregulation of mitochondrial membrane potential and respiration and an increase in mitochondrial number and their cristae to outer membrane surface ratio. This mechanism could be, at least in part, due to upregulation of c-Myc protein and targets. We also show that the sensitivity of ARID1A-deficient cells to the complex I mitochondrial inhibitor IACS-010759 is exclusively dependent upon ARID1A, and that IACS-010759 severely compromises mitochondrial respiration of ARID1A-deficient cells in a dose-dependent manner in both monolayer culture and multicellular spheroids. Lastly, we show that in a preclinical model of ARID1A-deficient OCCCs, IACS-010759-treated mice have twice the overall survival as compared to a vehicle-treated group with no observable toxicity to the host.

## 2. Results

### 2.1. ARID1A-Deficient Cells Have Increased Expression of OXPHOS Genes and Pathways

**Figure 1:**
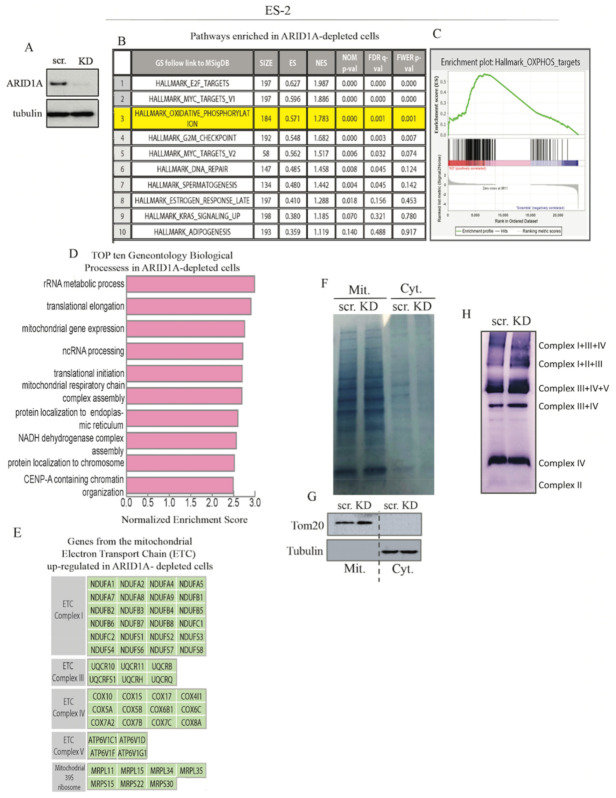
ARID1A-deficient cells upregulate OXPHOX pathways and increase mitochondrial respiration. (**A**) Western blot analysis for levels of ARID1A in scramble (scr.) versus ARID1A knockdown (KD) in *ARID1A* WT ES-2 cells. Tubulin was used as loading control. (**B**) GSEA data analysis of microarray transcriptomic profiling in ARID1A KD versus scramble ES-2 cells revealed that the OXPHOS pathway is among the top-most upregulated pathways following ARID1A loss. RNA-sequencing data were obtained using 3 independent samples for each cohort (scramble and KD). (**C**) Representative graph for enriched mitochondrial OXPHOS pathway (*p* = 0.000, *q* = 0.001). (**D**) Left, Top ten gene ontology (GO) biological process (*p* < 0.001) in ARID1A KD versus scramble ES-2 cells. (**E**) Genes coding for mitochondrial electron transport chain (ETC) components are upregulated in ARID1A KD versus scramble ES-2 cells. Results are from three independent experiments and are expressed as mean ± SD. (**F**) Amido black staining of mitochondrial (Mit.) and cytoplasmic (Cyt.) fractions isolated from scramble (scr.) and ARID1A KD (KD) ES-2 cells. (**G**) Western blot analysis for levels of Tom20 in scramble (scr.) versus ARID1A knockdown (KD) *ARID1A* WT ES-2 isolated mitochondria. Tubulin was used as a loading control. (**H**) Isolated mitochondrial pellets were processed in native gels following the protocol described in [47]. Shown are complexes and supercomplexes of the mitochondrial ETC.

To begin investigating the possible effects of ARID1A loss on mitochondrial metabolism, ARID1A was knocked down via siRNA in *ARID1A* WT ES-2 cells (Figure 1A, and Supplementary Figure S1A) and control and knockdown cells were subjected to transcriptomic profiling using RNA-sequencing. Gene set enrichment analysis (GSEA) revealed that *E2F* and *MYC* targets were upregulated in ARID1A knockdown cells, suggesting effects on cell cycle progression. Furthermore, and interesting considering previous findings in the literature [3], the oxidative phosphorylation pathway is among the most prominently enriched pathways in *ARID1A*-mutated cells (Figure 1B–D). This includes the upregulation of several genes encoding for components of the mitochondrial electron transport chain (ETC) complexes I, III, IV, and V (Figure 1E). The full list of the genes differentially expressed in scramble vs. ARID1A knock-down (KD) *ARID1A* WT ES-2 cells is available in Supplementary Table S1. Next, mitochondrial and cytoplasmic fractions were isolated from Scrambled (scr.) and ARID1A KD ES-2 cells (Figure 1F) and the levels in the outer membrane of mitochondria (OMM) marker TOM20 and the mitochondrial complexes were analyzed via Western blot and native polyacrylamide gel electrophoresis (BN-PAGE) respectively [46,47,48]. As shown in Figure 1G, the levels of TOM20 were higher in the mitochondrial fraction of ARID1A KD ES-2 cells as compared to scramble. Furthermore, an increased proportion of several mitochondrial complexes and supercomplexes, including complex III and IV, was found in ARID1A KD ES-2 cells as compared to scramble control (Figure 1H). Importantly, mitochondrial supercomplexes constitute the higher-order organization of the mitochondrial electron transport chain that promotes efficient and rapid catalysis of respiration [49,50,51]. Taken together, our results show that loss of ARID1A in ARID1A WT cells results in upregulation of OXPHOS pathway by GSEA analysis, biological processes regulating mitochondrial gene expression by GO analysis, and increase in the overall mitochondrial complex and supercomplex levels.

### 2.2. ARID1A Regulates Mitochondrial Respiration in OCCC Cells

**Figure 2:**
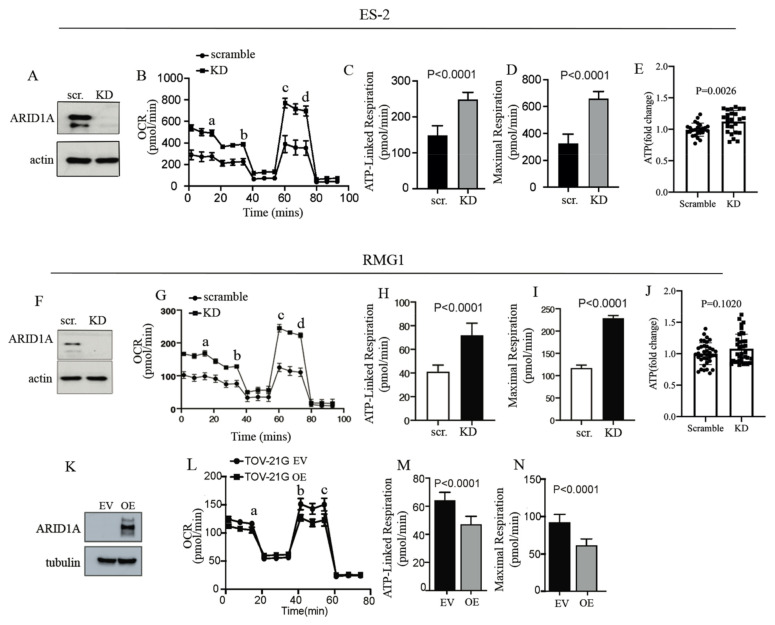
ARID1A controls mitochondria metabolism in OCCC cells. (**A**) Western blot analysis for levels of ARID1A in scramble (scr.) versus ARID1A knockdown (KD) *ARID1A* WT ES-2 cells. Actin was used as a loading control. (**B**) Real-time oxygen consumption rate (OCR) in scramble versus ARID1A KD ES-2 cells as measured by Seahorse. The injection order: a. glucose, b. oligomycin, c. FCCP, and d. rotenone and antimycin. (**C**) ATP-linked respiration in ARID1A in scramble (scr.) versus ARID1A knockdown (KD) *ARID1A* WT ES-2 cells. (**D**) Maximal respiration in scramble versus ARID1A KD ES-2 cells. (**E**) Fold change of basal ATP total amount in scramble (scr.) versus ARID1A knockdown (KD) *ARID1A* WT ES-2 cells. (**F**) Western blot analysis for levels of ARID1A in scramble (scr.) versus ARID1A knockdown (KD) *ARID1A* WT RMG1 cells. Actin was used as a loading control. (**G**) Real-time oxygen consumption rate (OCR) in scramble versus ARID1A KD RMG1 cells as measured by Seahorse. The injection order: a. glucose, b. oligomycin, c. FCCP, and d. rotenone and antimycin (**H**) ATP-linked respiration in ARID1A in scramble (scr.) versus ARID1A knockdown (KD) *ARID1A* WT RMG1 cells. (**I**) Maximal respiration in scramble versus ARID1A KD RMG1 cells. (**J**) Fold change of basal ATP total amount in scramble (scr.) versus ARID1A knockdown (KD) *ARID1A* WT RMG1 cells. (**K**) Western blot analysis for levels of ARID1A empty vector (EV) versus ARID1A overexpressing (OE) ARID1A-mutated TOV-21G cells. Tubulin was used as a loading control. (**L**) Real-time oxygen consumption rate (OCR) in ARID1A empty vector (EV) versus ARID1A overexpressing (OE) ARID1A-mutated TOV-21G cells. (**M**) ATP-linked respiration in ARID1A empty vector (EV) versus ARID1A overexpressing (OE) *ARID1A*-mutated TOV-21G cells. (**N**) Maximal respiration in ARID1A empty vector (EV) versus ARID1A overexpressing (OE) ARID1A-mutated TOV-21G cells. The injection order: a. oligomycin, b. FCCP, and c. rotenone and antimycin. Results are from three independent experiments and are expressed as mean ± SD.

To determine whether the enrichment in OXPHOS pathways following ARID1A loss is associated with a functional increase in mitochondrial activity, we measured the mitochondrial oxygen consumption rate (OCR) in the presence and absence of ARID1A. To this end, ARID1A was knocked down via siRNA in ARID1A WT ES-2 cells (Figure 2A, and Supplementary Figure S1B, left) and ATP-linked respiration and maximal respiration were measured in scramble versus ARID1A KD equal amounts of cells (Supplementary Figure S1C) and as previously described [52]. We found that loss of ARID1A resulted in increases in OCR (Figure 2B), ATP-linked respiration (Figure 2C), and maximal respiration (Figure 2D). The same results were confirmed when ARID1A was knocked down in an additional *ARID1A* WT cell line, RMG1 (Figure 2F–I and Supplementary Figure S1B, right, and D). When we measured the ATP levels in scramble versus ARID1A KD cells, we found that the loss of ARID1A in ES-2 cells resulted in a significant increase in the ATP levels (Figure 2E). In RGM1 cells, however, loss of ARID1A resulted in a slight but not significant increase in the ATP levels (Figure 2J). This apparent discrepancy could be explained by the fact that different cells may consume the produced ATP at different rates. To further establish that ARID1A is responsible for increases in mitochondrial respiration, we conducted complementary experiments [39] and tested whether ectopic expression of ARID1A in the *ARID1A*-mutated TOV-21G ovarian cancer cell line would result in decreases in mitochondrial activity. As shown in Figure 2K–N (and supplementary Figure S1E,F), we found that cells expressing ARID1A had significantly lower mitochondrial activity as compared to vector-alone expressing cells. Noteworthy, the decrease in mitochondrial activity in TOV-21 ARID1A overexpressing cells is less dramatic as compared to the increase in mitochondrial activity following ARID1A KD. Taken together, this indicates that ARID1A is responsible for controlling mitochondrial activity in OCCC cells and that its loss results in increases in mitochondrial respiration.

### 2.3. ARID1A Controls Mitochondrial Membrane Potential, Mitochondrial Number, and Size

**Figure 3:**
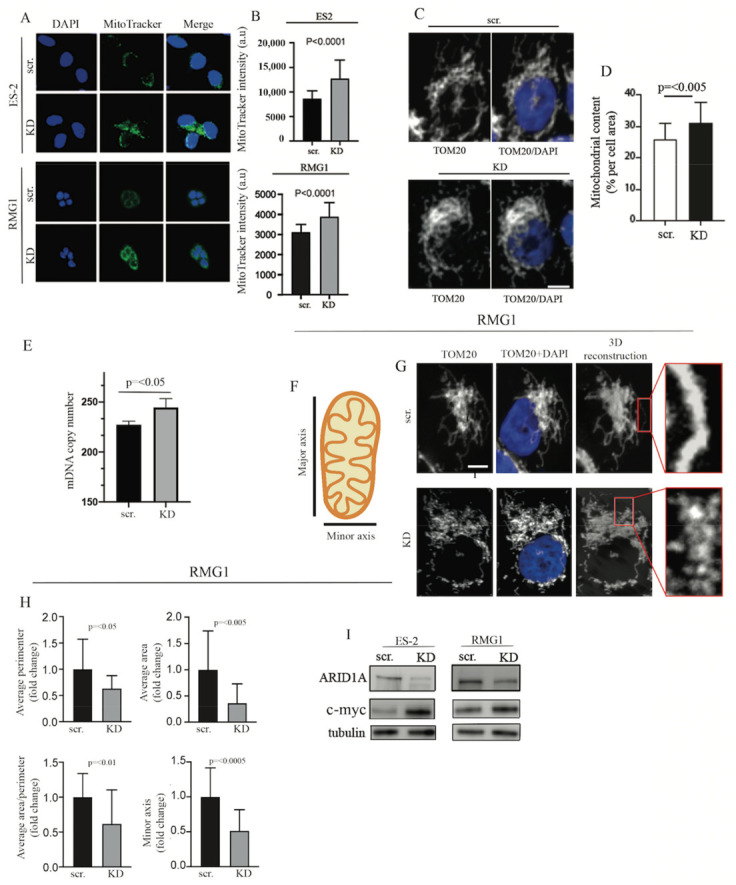
Loss of ARID1A results in increased mitochondrial membrane potential and increased mitochondrial content and fragmentation. (**A**) Representative images (original magnification 60×) of scramble (scr.) and ARID1A KD (KD) ES-2 (top four panels) and RMG1 (bottom four panels) *ARID1A* WT cells stained with DAPI (blue) and MitoTracker green. Merge image is also shown. (**B**) Quantification of MitoTracker fluorescence intensity in ES-2 (top panel) or RMG1 (bottom panel) cells under the abovementioned conditions expressed as arbitrary units (a.u). (**C**) Representative image of mitochondrial content in scramble (scr.) versus ARID1A knockdown (KD) *ARID1A* WT RMG1 cells evaluated via TOM20 staining. (**D**) Quantification of mitochondrial content expressed per each condition. (**E**) Mitochondrial DNA content in scr. versus ARID1A knockdown (KD) *ARID1A* WT RMG1 cells. (**F**) Representation of parameters used to evaluate mitochondrial morphology. (**G**) Representative images of TOM20 and DAPI staining in scr. versus ARID1A knockdown (KD) *ARID1A* WT RMG1 cells followed by 3D reconstruction of mitochondria. (**H**) Average mitochondrial perimeter in scr. vs. KD cells (top left), average mitochondrial area in scr. vs. KD cells (top right), average mitochondrial area/perimeter in scr. vs. KD cells (bottom left). Minor axis in scr. vs. KD cells (bottom right). Results are from three independent experiments and are expressed as mean ± SD. (**I**) Left, Western blot analysis for levels of ARID1A and c-Myc in scramble (scr.) versus ARID1A knockdown (KD) *ARID1A* WT ES-2 cells. Tubulin was used as a loading control. Right, Western blot analysis for levels of ARID1A and c-Myc in scramble (scr.) versus ARID1A knockdown (KD) ARID1A WT RMG1 cells. Tubulin was used as a loading control.

The finding that ARID1A regulates mitochondrial respiration in OCCC cells prompted us to examine alterations in mitochondrial membrane potential. To this end, scramble and KD RMG1 and ES-2 *ARID1A* WT cells were stained with the MitoTracker Deep Red dye, which is known to accumulate in mitochondria in a membrane-potential-dependent manner [53]. As shown in Figure 3A,B, loss of ARID1A resulted in an increase in the uptake of MitoTracker Deep Red in both ES2 and RMG1 OCCC cells. We next determined whether the loss of ARID1A would result in an increase in mitochondrial content. To this end, scramble versus ARID1A KD *ARID1A* WT RMG1 cells were subjected to immunostaining using an antibody to the mitochondrial marker TOM20. Mitochondrial content was visualized and evaluated via immunofluorescence analysis of reconstructed mitochondria. As shown in Figure 3C,D, the loss of ARID1A resulted in a statistically significant increase in the mitochondrial mass in RMG1 cells. Furthermore, we observed an increase of mtDNA content in ARID1A KD RMG1 cells, which further supports the finding of an increase in mitochondrial content following ARID1A loss (Figure 3E). A higher ratio of mitochondrial cristae to the outer membrane surface has been associated with increased mitochondrial activity [18,19,20,21]. Thus, we investigated whether the loss of ARID1A resulted in changes in mitochondrial shape and size in ARID1A scramble versus ARID1A WT RMG1 cells. To this end, we measured both minor and major mitochondrial axes (Figure 3F) as well as in cell perimeter and area in TOM20+DAPI-stained cells (Figure 3G) per each condition and as previously described [54]. As shown in Figure 3H, loss of ARID1A led to an overall decrease in mitochondrial size. Taken together, this suggests that the increase in mitochondrial activity following ARID1A loss is, at least in part, due to both an increase in mitochondrial biogenesis and a decrease in mitochondrial size. This is consistent with previous reports showing that an increase in mitochondrial fragmentation is associated with more aggressive cancer cell behavior [55]. The increase in mitochondrial content followed by the knockdown of ARID1A was accompanied by an increase in the expression levels of c-Myc in both ES-2 and RMG1 ovarian cancer cell lines (Figure 3I). This is consistent with a functional antagonism between c-Myc and the SWI/SNF complex [56] and is also consistent with our RNA-seq data showing that c-Myc targets are upregulated following ARID1A loss (Figure 1B).

### 2.4. ARID1A Loss Results in Selective Sensitivity to the Mitochondrial Inhibitor IACS-010759 in 2D

**Figure 4:**
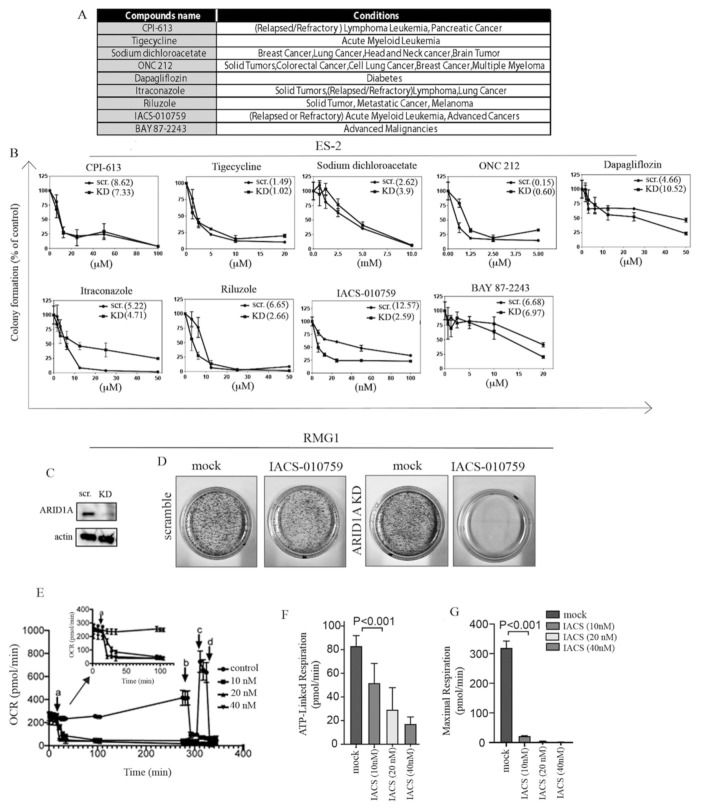
Loss of ARID1A results in selective sensitivity to mitochondrial inhibition in 2D. (**A**) Table of compounds with mitochondrial inhibitions properties in clinical trials. Compound names and conditions for which they are used are indicated [57]. (**B**) Dose-dependent sensitivity of scramble (scr.) and ARID1A KD (KD) ES-2 WT cells to the panel of mitochondrial inhibitors. Results are expressed as residual colony formation as compared to control. Drug treatment was conducted over a period of 7 days. (**C**) Western blot analysis for expression levels of ARID1A in scramble and ARID1A KD RMG1 cells obtained by lentiviral-mediated delivery of shRNA-ARID1A. Actin was used as a loading control. (**D**) Residual colony formation in scramble and ARID1A KD RMG1 cells exposed to 10 nM of the mitochondrial inhibitor IACS-010759 over a period of 10 days. (**E**) Real-time oxygen consumption rate (OCR) in scramble versus ARID1A KD RMG1 cells exposed to increasing concentrations of the mitochondrial inhibitor IACS-010759. The injection order: a. IACS-010759, b. oligomycin, c. FCCP, and d. rotenone and antimycin. Inset shows the OCR alterations after IACS-010759 injection during the first 100 min. (**F**) ATP-linked respiration in ARID1A in scramble (scr.) versus ARID1A knockdown (KD) *ARID1A* WT RMG1-2 cells exposed to increasing concentrations on the mitochondrial inhibitor IACS-010759. (**G**) Maximal respiration in ARID1A in scramble (scr.) versus ARID1A knockdown (KD) *ARID1A* WT RMG1-2 cells exposed to increasing concentrations on the mitochondrial inhibitor IACS-010759.

We next tested whether the increase in mitochondrial activity following ARID1A loss in OCCCs would render these cells selectively dependent upon mitochondrial energy production. To this end, we selected a panel of nine mitochondrial inhibitors (Figure 4A) that were in clinical trials at the time when this study was initiated [57] and subjected scramble and ARID1A KD ES-2 *ARID1A* WT cells to a colony formation assay in the absence (mock) and presence of an increasing concentration of the mitochondrial inhibitors indicated in Figure 4A. As shown in Figure 4B, all nine inhibitors induced a dose-dependent decrease in the clonogenicity of ES-2 cells. However, only IACS-010759 [43] and dapagliflozin [58] displayed selectivity for ES-2 cells where ARID1A has been knocked down. Of these two, IACS-010759 showed a much higher potency. These results were confirmed using the OCCC-derived *ARID1A* WT RMG1 cell line. Specifically, using the same colony formation assay, ARID1A KD RMG1 cells (Figure 4C) were more sensitive to the mitochondrial inhibitor IACS-010750 as compared to *ARID1A* WT (scramble) RMG1 cells (Figure 4D). To confirm that the selective sensitivity to IACS-010759 is dependent upon ARID1A, we performed a complementary experiment using the *ARID1A*-mutated OCCC-derived TOV-21G cell line. Specifically, ARID1A was overexpressed in TOV-21 cells (Figure 2I) and sensitivity to IACS-010759 was determined via colony formation assay. As shown in Supplementary Figure S3, the expression of ARID1A in the *ARID1A*-mutated TOV21G cell line results in the loss of sensitivity to the mitochondrial inhibitor IACS-010759. Lastly, we confirmed that treatment with IACS-010759 results in a dose-dependent decrease in mitochondrial activity in ARID1A KD RMG1 cells as determined via measuring OCR (Figure 4E), ATP-linked respiration (Figure 4F), and maximal respiration (Figure 4G) in the scramble and ARID1A KD cells exposed to increasing concentrations of IACS-010759 and as we previously described [59]. Taken together, this suggests that IACS-010759 selectively affects the cell viability of ARID1A-mutated cells via inhibiting their greater demand for mitochondrial activity.

### 2.5. ARID1A Loss Results in Selective Sensitivity to the Mitochondrial Inhibitor IACS-010759 in Three-Dimensional Culture

**Figure 5:**
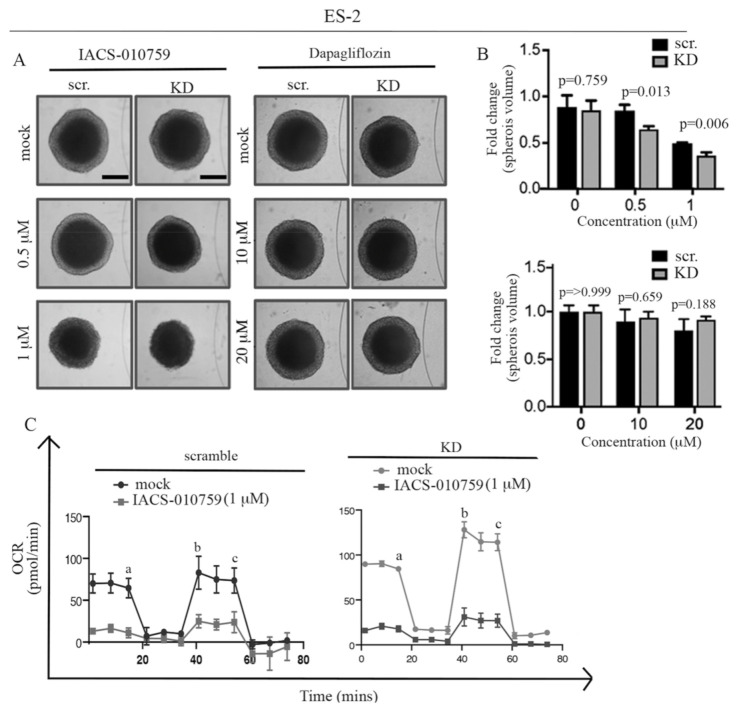
Loss of ARID1A results in selective sensitivity of spheroids to mitochondrial inhibition. (**A**) ES-2 derived spheroids exposed to the indicated concentrations of either IACS-010759 (left) or dapagliflozin (right) over a period of 48 h. (**B**) Quantification of residual spheroid volume in cells exposed to IACS-010759 (top) or dapagliflozin (bottom). (**C**) Real-time oxygen consumption rate (OCR) in scramble versus ARID1A KD spheroids derived from ES-2 cells exposed to the indicated concentrations of IACS-010759. The injection order: a. oligomycin, b. FCCP, and c. rotenone and antimycin.

Here, we sought to determine whether IACS-010759 retains selectivity toward ARID1A-deficient cells in a three-dimensional culture system. To this end, ES-2-derived spheroids were either mock-treated or treated with increasing concentrations (500 nM and 1 µM) of IACS-010759 over a period of 10 days. Dapagliflozin, which displayed selectivity for ES-2 cells where ARID1A has been knocked down although at micromolar concentrations, was also used. As shown in Figure 5A (left panel) and Figure 5B (top panel), IACS-010759 treatment led to a decrease in the spheroid volume that was greater in the *ARID1A*-mutated cells as compared to ARID1A WT cells. Dapagliflozin treatment, on the other end, had minimal effect on the spheroid volume in both *ARID1A*-mutated and ARID1A WT cells (Figure 5A, right panel, and Figure 5B, bottom panel). Lastly, we determined the effect of IACS-010759 treatment on mitochondrial respiration in spheroids. As shown in Figure 5C, IACS-010759 treatment led to a significant reduction of mitochondrial activity as determined via measuring OCR. Taken together, this suggests that IACS-010759 selectively affects the cell viability of ARID1A-mutated cells in 3D cultures.

### 2.6. IACS-010759 Treatment Prolongs Survival in a Model of ARID1A-Mutated OCCCs

**Figure 6:**
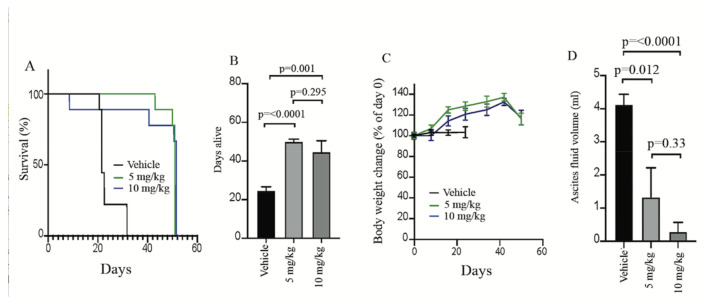
Preclinical testing of IACS-010759 in a xenograft model of OCCC-mutated tumor. (**A**) The percentage survival of mice. (**B**) Days the mice are alive. (**C**) Percentage body weight overtime. (**D**). Ascitic fluid volume after intraperitoneal injection of TOV21G cells followed by oral dosing of either vehicle control or indicated dose of IACS-010759 (N = 9 mice/group).

To address the clinical relevance of targeting mitochondrial function as a potential therapeutic strategy for the treatment of ARID1A-deficient OCCC cancers, we tested the ability of IACS-010759 to suppress the growth of ARID1A-deficient OCCC tumors in vivo. Specifically, ARID1A-deficient TOV-21G cells were intraperitoneally injected in athymic nude female mice which were randomly assigned to three treatment arms: vehicle alone, IACS-010759 5 mg/kg, and IACS-010759 10 mg/kg. These doses were chosen because they were previously described as well tolerated in preclinical studies [32,39,45]. Treatment was performed via oral gavage on a 5 day on/2 day off schedule. As shown in Figure 6A, while mice in the control group (treated with vehicle) had to be sacrificed by week 3 according to the IACUC guidelines, mice in both IACS-010750-treated arms survived almost as twice longer and until the experiments were terminated because their reached significance at month two. More specifically and as shown in Figure 6B, the mean days alive in treated groups (50.1 ± 0.7 days in the 5 mg/kg group and 44.8 ± 4.9 days in the 10 mg/kg group) are more than two times that in the control group (24.8 ± 1.3 days). More importantly, during the two-month experiment, mice kept a stable growing weight in the treated groups (mice weight increased more than 20%, even dropped but still higher than the starting weight before sacrifice, Figure 6C) which is not due to the increased volume of fluids in the abdomen (4.25 ± 0.31 mL in the control group, 1.3 ± 0.88 and 0.29 ± 0.25 mL in groups of 5 and 10 mg/kg, respectively, Figure 6D) which is a characteristic of ovarian cancer [60,61]. Taken together, this suggests that targeting mitochondrial function by IACS-010759 could represent an effective strategy for the treatment of *ARID1A*-mutated OCCCs.

## 3. Discussion

Here, we report that knockdown of ARID1A expression in ovarian cancer cells expressing this protein results in increases in oxidative phosphorylation (OXPHOS). We also show that restoration of ARID1A in previously defective cells results in decreases in OXPHOS. These findings are consistent with those reporting that lung cancer cells with mutations in another component of the SWI/SNF complex (SMARCA4) display enhanced oxygen consumption [39]. Both studies demonstrate a vulnerability of cells with ARID1A/SMARCA4 mutations/defects to the effects of mitochondrial inhibitors, raising the possibility for therapeutic interventions. The underlying mechanisms are not entirely clear but may be related to c-Myc activity. c-Myc has been shown to be antagonized by the SWI/SNF complex [56] and c-Myc is well known to play a major role in metabolic dysregulation of tumors [62,63,64]. Whereas SMARCA4 mutations were not reported to result in an increase in c-Myc expression, gene set enrichment analyses (GSEA) showed an increase in c-Myc target genes (Lissanu Deribe et al., 2018). We observed both a large increase in c-Myc expression in ES-2 cells transfected with ARID1A siRNA and also increases in c-Myc targets genes in GSEA (Figure 1). The issue of whether the effect of ARID1A on OXPHOS is mediated by c-Myc could theoretically be addressed by knocking down c-Myc, but the results would be confounded by the ubiquitous effects of C-myc on cell proliferation and other processes.

The efficacy of glycolysis in terms of ATP production is too low to meet the energy demands of highly active tumor cells, resulting in dependence on mitochondrial energy production [65]. Cells display a high degree of metabolic plasticity, shifting between different energy sources and metabolic pathways. Inhibition of OXPHOS may therefore enable the upregulation of compensatory pathways, such as glycolysis, to support cancer cell survival. It has occasionally been suggested that inhibition of both mitochondrial metabolism and glycolysis may overcome such resistance mechanisms. Such strategies are, however, expected to generate general toxicity. The potential for compensatory glycolysis may in fact be limited in solid tumors due to the low availability of glucose [66,67]. Cells in solid tumor may therefore be more vulnerable to inhibition of OXPHOS compared to normal cells situated in well-vascularized regions [34,59]. The increasing understanding of the important role of mitochondrial energy production for tumor cell viability has resulted in considerable interest in this process as a target for the development of antineoplastic agents [31,59,68,69,70].

There are several unanswered questions with regard to the optimal characteristics of cancer drugs acting on mitochondrial energetics. Which degree of inhibition of energy production will inhibit tumor cell survival without resulting in general toxicity? Which part of the respiratory chain (or other components) should be inhibited for optimal anti-neoplastic effects? Furthermore, factors related to the pharmacological properties of drugs, such as their lipophilicity and ability to penetrate solid tumors, are likely to be important [71]. The first reports of mitochondrial inhibitors showing antitumor effects used the cell-penetrating dye rhodamine 123 [72]. Although the mechanism of tumor cell toxicity by rhodamine 123 was shown to be the impairment of ATP synthesis [72], the larger degree of retention of the dye in carcinoma cells compared to normal cells was believed as an important factor for tumor-specificity [73]. It is possible that the specific mechanism of mitochondrial inhibition may not be critical. Inhibitors of the electron transport chain, inhibitors of the F1F0-ATPase, and mitochondrial uncouplers were all reported to be effective in inducing cell death in the core regions of multicellular tumor spheroids [74]. We, here, tested a number of different mitochondrial inhibitors belonging to different mechanistic classes and found that the complex I inhibitor IACS-010759 showed the best activity in our in vitro models. IACS-010759 has been shown to induce apoptosis of OXPHOS-dependent brain cancer and acute myeloid leukemia cells [32], lung cancer cell lines and tumors [39], and mantle cell lymphoma cells [45]. An important factor to be considered in clinical development is the occurrence of acidosis. Lactate levels were increased in patient plasma consistent with inhibition of complex I, but acidosis was not induced (NCT03291938). IACS-010759 is being evaluated in phase I clinical trials in relapsed/refractory AML and solid tumors (NCT02882321, NCT03291938). It is unclear why IACS-010759 showed selectivity to ARID1A-deficient ovarian cancer cells, whereas other inhibitors that were tested showed less or no such selectivity. Further understanding of this question is necessary to develop improved compounds.

The field of mitochondrial bioenergetics as a target for antineoplastic agents is expanding rapidly [33,35,36]. The identification of metabolic vulnerabilities will be important in expanding this field and is expected to broaden the arsenal of clinically available therapeutics for the treatment of advanced disease.

## 4. Materials and Methods

### 4.1. Chemicals and Antibodies

IACS-010759 (S8731) was obtained from Selleck Chemicals (Houston, TX, US). The following antibodies were from Santa Cruz (Dallas, TX, US): anti-ARID1A (sc-32761), anti-actin (sc-8432), anti-tubulin (sc-5286), anti-Tom20 (sc-17764). Anti c-Myc antibody (ab32072) was obtained from Abcam (Cambridge, MA, US). CPI-613 (s2776), tigecycline (s1403), sodium dichloroacetate (DCA, s8615), Onc212 (s8673), dapagliflozin (s1548), itraconazole (s2467), riluzole (s1614), and Bay87-2243 (S7309) were all obtained from Selleck Chemicals (Houston, TX, USA).

### 4.2. Cell Culture

ES2, RMG1, and TOV-21G human clear cell carcinoma were maintained in DMEM (Dulbecco’s modified Eagle’s medium; 12320032) with 10% FBS and 1% penicillin.

### 4.3. siRNA

ES2 or RMG1 cells were plated and left to attach for 24 h in 6-well plates. Cells were then transfected with ON-TARGET plus human non-targeting control pool (D-001810-10-05) or ON-TARGET plus human ARID1A siRNAs (accessions Hit: NM_006015, NM_139135; L-017263-00-0005) followed the general protocol provided by Dharmacon (Cambridge, UK) for 48 h. Different post-transfection experiments were then performed.

### 4.4. Western Blot

Cells were collected and lysed in ice-cold lysis buffer and electrophoresed on 4–15% SDS-PAGE gels. Proteins were then transferred to nitrocellulose membranes and incubated at room temperature in 5% milk in 1× PBST buffer for 1 h. Membranes were then incubated overnight with primary antibodies at a dilution of 1:1000 in 1× PBST buffer. The next day, membranes were washed with 1× PBST buffer and incubated with anti-rabbit or anti-mouse secondary antibodies at a 1:5000 dilution for 1 h. Immunoreactive bands were detected by FluorChem Fluorescent Western Imaging System from ProteinSimple (San Jose, CA, USA).

### 4.5. RNA-SEQ Experiments

ES2 human clear cell carcinoma cells present (wild type) or absent (siRNA) ARID1A were collected and extracted using an RNeasy Plus Micro Kit (Qiagen, Hilden, Germany). Barcoded TruSeq RNA v2 libraries (Illumina, San Diego, CA, USA) were created, and libraries were sequenced on a HiSeq 2500 (Illumina, San Diego, CA, USA) as paired-end 100 bp. STAR version 2.4.0d was used to align the RNA-seq reads to the human genome reference build GRCh37 (hg19). Ensembl gene annotation version 75 was provided as the gene transfer format for exon junction support. For each sample, reads were assigned to genes and summarized using featureCounts (subread package version 1.4.5-p1). Raw read counts were read into R (version 3.2.0) and subjected to normalization by the trimmed mean of M-values normalization method implemented in the R/bioconductor edgeR package and variance normalized using voom from the R/bioconductor limma package. All genes with at least one count per million (CPM) mapped reads in at least two samples were analyzed further. Differential gene expression was performed using the R/bioconductor limma package. RNA-seq data can be found under the GEO accession no. GSE117614.

### 4.6. Isolation of Mitochondria

Mitochondria were isolated using cell mitochondria isolation kit (Sigma Aldrich, St. Louis, MO, USA; MITOISO2). Briefly, ~90% confluent cells were collected by trypsinization and centrifuged for 5 min at 600× *g*. Cell pellets were then resuspended in ice-cold PBS and centrifuged again for 5 min at 600× *g* at 2–8 °C and the supernatant discarded. This wash step was repeated and 0.5 mL 1 × volume extraction buffer with cell lysis solution (1:200 (*v*/*v*)) was added to resuspend the cell pellet. Following incubation on ice for 5 min, 2 × 1 mL volume extraction buffer was added, and the homogenate was centrifuged at 600× *g* for 10 min at 4° C. The supernatant was carefully transferred to a fresh tube followed by centrifugation at 11,000× *g* for 10 min at 4 °C. Pellets were resuspended in ice-cold PBS and centrifuged again for 5 min at 11,000× *g*. The pellet was suspended in CelLytic M Cell Lysis Reagent with Protease Inhibitor Cocktail (1:100 *v*/*v*)). For digestion with trypsin, isolated mitochondria pellets were resuspended with trypsin (0.05%, #25300054, ThermoFisher, Waltham, MA, USA) and kept on a rotator at a slow speed at 2–8 °C for 30 min. For permeabilization by digitonin, isolated mitochondria pellets were resuspended with digitonin (0%, 0.2% and 0.4% [*w*/*v*]) and kept on ice for 15 min. Then mitochondria were collected by centrifugation at 11,000× *g* for 10 min at 4 °C, washed in cold PBS and centrifuged again at 11,000× *g* for 10 min at 4 °C. Pellets were then suspended in CelLytic M Cell Lysis Reagent with Protease Inhibitor Cocktail (1:100 (*v*/*v*)).

### 4.7. Native Gel

Native gels were performed and immunoreactive bands were detected based a previously described protocol [75].

### 4.8. Measurements of Oxygen Consumption

The Seahorse XF analyzer was used as recommended by the manufacturer (Seahorse Bioscience, North Billerica, MA, USA). Seahorse Cell Mito Stress Test Kit (Agilent Technologies) was used to measure mitochondrial OxPHOS. Briefly, 60,000 cells per condition were plated in 100 µL culture medium in XF24-well cell plates with blank control wells. Prior to measurements of OCR, the medium was replaced with 500 µL Seahorse assay media (1 mM pyruvate and 2 mM glutamine) at 37 °C without CO_2_ for 1 h. ATP-linked respiration OCR were calculated as average values of basal OCR (after the injection of glucose) minus average postoligomycin OCR values. Maximum mitochondrial respiration capacity was calculated as average maximal OCR values minus average postoligomycin OCR values. ATP-linked respiration OCR were calculated as average values of basal OCR minus average postoligomycin OCR values.

The concentration of oligomycin is 0.5 µM, the concentration of FCCP is 0.5 µM, the concentrations of rotenone and antimycin are 1.0 µM. Injection detail for each seahorse measurement was described in each corresponding figure legend. For all Seahorse-related experiments, an equal cell number per condition was confirmed post-analysis in cell lysates via amido black staining.

### 4.9. ATP Quantification Assay

Around 20,000 cells per well under conditions of scramble and knockdown were plated in 96-well plates one day before the assay. Next day, the amount of total ATP was determined using the ATP Bioluminescence Assay Kit HS II (Sigma catalogue number 11699709001) and analyzed on an Infinite M200 PRO Luminometer (Tecan). Values obtained from the Infinite M200 PRO Luminometer were further normalized by the protein amount under each condition.

### 4.10. Confocal Microscopy

For mitochondrial membrane potential assay, cells were stained with MitoTracker Deep Red dye (Cell signaling catalogue number 8778) according to the manufacturer’s instructions. Briefly, 500 nM MitoTracker Deep Red dye solution was added into cells and incubated for 30 min at 37 °C. After staining, medium with MitoTracker solution was removed and cells were then fixed in ice-cold, 100% methanol for 15 min at −20 °C and rinse 3 times with PBS for 5 min. Cells were fixed and mounted with DAPI staining dye Hoechst 33342 (ThermoScientific catalogue number H3570). Images were acquired with an Olympus BX upright microscope equipped with a Fluoview 1000 confocal scan head with a 60× oil immersion objective (Olympus, Shinjuku, Japan). Image processing and fluorescence intensity quantification was performed using Icy open platform software.

### 4.11. Analysis of Mitochondrial Morphology

For studies of mitochondrial morphology, cells were fixed with 2% paraformaldehyde (PFA) for 20 min in PBS at RT. Then samples were washed with PBS 10 min ×3 on shaker. For permeabilization, samples were incubated with 0.1% Triton X-100 in PBS for 15 min. After blocking with 5% BSA in PBST, cells were stained with anti-Tom20 followed by Alexa Fluor 594-conjugated secondary antibodies and analyzed via confocal fluorescence microscopy. Images were acquired with an Olympus FluoView BX2 Upright confocal with a 60× oil immersion objective (Olympus, Shinjuku, Japan). To ensure that mitochondria present through multiple planes of the cell could be assayed, z-stacks with a thickness ~3 μm were acquired for each cell (~15 captures for each figure). Images from z-stack were constructed by Imaris (Oxford Instruments) under the function of volume and 3D view. Procedures in detail could be obtained from the website of Imaris (Imaris for Cell Biologists—Imaris—Oxford Instruments) and morphology of mitochondria was assessed using the Fiji software mitochondrial morphology plugins and calculated as described in the mitochondrial morphology tutorial [76] (https://imagejdocu.tudor.lu/plugin/morphology/mitochondrial_morphology_macro_plug-in/start) (accessed on 1 April 2021).

### 4.12. Measurement of mDNA Content

DNA was isolated using PureLink^®^ Genomic DNA Mini Kit (Thermo Fisher Scientific; Waltham, MA, USA) and mtDNA and nDNA were amplified by Human Mitochondrial DNA (mtDNA) Monitoring Primer Set (catalogue number 7246, Takara, Maebashi, Japan) using the 7500/7500 Fast Real-Time PCR System (Applied Biosystems; Foster City, CA, USA). Mitochondria DNA copy number was calculated based on the mtDNA copy number calculation tool supported by Takara.

### 4.13. Plasmid Transfection

TOV-21G cells were seeded in Dulbecco’s modified Eagle’s medium one day before the transfection in 6-well plates and incubated at 37 °C, 5% CO_2_. Transfection was performed with control vector (pcDNA/GW-40/LacZ) or 4 µg pARID1A (Addgene: 39311; Watertown, MA, USA) using FuGENE 6 Transfection Reagent (Promega, Madison, WI, USA) according to manufacturer’s protocol. Further experiments were performed 48 h after transfection.

### 4.14. Generation of Spheroids

Spheroids were prepared using a modification of our previously described method [77]. A cell suspension containing 5000 cells/200 µL was added to each well in the 96-well plates. Wells were overfilled by adding 180 µL media to acquire a convex surface curvature and plates inverted in order to allow cells to sediment at the liquid–air interface. Plates were returned to normal position after 24 h incubation with excess media removed by aspiration, and then incubated for 4 days before drug exposure. Images for each spheroid were taken by microscopy camera and diameters were then measured and volume of spheroids were calculated by equation (1) where r = radius.

Volume = 4/3 × π × r^3^ (1)

### 4.15. Crystal Violet Staining

Cells were fixed with ice-cold methanol for 15 min, then methanol was removed and crystal violet staining solution (0.05% (*w*/*v*)) was added to each well for 30 min (the volume should cover the whole surface of each well). Then, staining solution was removed, and plates were washed with water three times.

### 4.16. Colony Formation Assay

For colony formation assay 10,000 or 20,000 cells were plated in 10 cm plates and treated with the indicated drugs at the indicated concentration over a period of 10 days. Medium was changed every 3 days. At the end of the 10-day period, cells were stained with crystal violet and colony were visualized and quantified using ImageJ.

### 4.17. Preclinical Testing of IACS-010759

TOV21G cells (10 million) infected with GFP lentivirus were injected intraperitoneally into athymic nude female mice at 6 weeks of age (Charles River Laboratories, Crl:NU(NCr)-Foxn1nu). Control mice were treated with vehicle (8% DMSO) and the experimental mice were treated with IACS-010759 (5 and 10 mg/kg) orally daily on a 5 day on/ 2 day off schedule until the time the mice were either dead or euthanized due to signs of distress or moribundity followed by collection of ascitic fluid/blood from the peritoneal cavity. IACS-010759 was dissolved in DMSO and administered in milk. Mice were weighed and imaged once a week using IVIS. The IACS-010759 administered was started from the same day TOV21G cells were injected. The animal experiments were approved by the Institutional Animal Care and Use Committee at the University of Minnesota (Protocol ID:1807-36121A).

### 4.18. Statistical Analysis

Statistics were calculated using GraphPad (v8.0) by Prism (San Diego, CA, USA). Data are expressed as the mean ± SD. Differences between groups were determined by Student’s *t*-test. *p* values < 0.05 were considered statistically significant (* *p* < 0.05, ** *p* < 0.01).

## Supplementary Materials

The following are available online at https://www.mdpi.com/article/10.3390/ijms22094750/s1, Figure S1: Original WBs. Figure S2: Original WBs. Figure S3: Colony formation assay. Table S1: Comparison of gene expression profile between scramble and siARID1A in ES2 cells.

## References

[1] Fukumoto T., Magno E., Zhang R. (2018). SWI/SNF Complexes in Ovarian Cancer: Mechanistic Insights and Therapeutic Implications. Mol. Cancer Res..

[2] Jelinic W., Mueller J.J., Olvera N., Dao F., Scott S.N., Shah R., Gao J.J., Schultz N., Gonen M., Soslow R.A. (2014). Recurrent SMARCA4 mutations in small cell carcinoma of the ovary. Nat. Gen..

[3] Dier U., Shin D.H., Hemachandra L.P., Uusitalo L.M., Hempel N. (2014). Bioenergetic analysis of ovarian cancer cell lines: Profiling of histological subtypes and identification of a mitochondria-defective cell line. PLoS ONE.

[4] Kwan S.Y., Cheng X., Tsang Y.T., Choi J.S., Kwan S.Y., Izaguirre D.I., Kwan H.S., Gershenson D.M., Wong K.K. (2016). Loss of ARID1A expression leads to sensitivity to ROS-inducing agent elesclomol in gynecologic cancer cells. Oncotarget.

[5] Ogiwara H., Takahashi K., Sasaki M., Kuroda T., Yoshida H., Watanabe R., Maruyama A., Makinoshima H., Chiwaki F., Sasaki H. (2019). Targeting the Vulnerability of Glutathione Metabolism in ARID1A-Deficient Cancers. Cancer Cell.

[6] Anderson G.R., Wardell S.E., Cakir M., Yip C., Ahn Y.R., Ali M., Yllanes A.P., Chao C.A., McDonnell D.P., Wood K.C. (2018). Dysregulation of mitochondrial dynamics proteins are a targetable feature of human tumors. Nat. Commun..

[7] Archer S.L. (2013). Mitochondrial dynamics--mitochondrial fission and fusion in human diseases. N. Engl. J. Med..

[8] Maycotte P., Marin-Hernandez A., Goyri-Aguirre M., Anaya-Ruiz M., Reyes-Leyva J., Cortes-Hernandez P. (2017). Mitochondrial dynamics and cancer. Tumour Biol..

[9] Samant S.A., Zhang H.J., Hong Z., Pillai V.B., Sundaresan N.R., Wolfgeher D., Archer S.L., Chan D.C., Gupta M.P. (2014). SIRT3 deacetylates and activates OPA1 to regulate mitochondrial dynamics during stress. Mol. Cell. Biol..

[10] Trotta A.P., Chipuk J.E. (2017). Mitochondrial dynamics as regulators of cancer biology. Cell. Mol. Life Sci..

[11] Kashatus J.A., Nascimento A., Myers L.J., Sher A., Byrne F.L., Hoehn K.L., Counter C.M., Kashatus D.F. (2015). Erk2 phosphorylation of Drp1 promotes mitochondrial fission and MAPK-driven tumor growth. Mol. Cell.

[12] Malhotra A., Dey A., Prasad N., Kenney A.M. (2016). Sonic Hedgehog Signaling Drives Mitochondrial Fragmentation by Suppressing Mitofusins in Cerebellar Granule Neuron Precursors and Medulloblastoma. Mol. Cancer Res. MCR.

[13] Wieder S.Y., Serasinghe M.N., Sung J.C., Choi D.C., Birge M.B., Yao J.L., Bernstein E., Celebi J.T., Chipuk J.E. (2015). Activation of the Mitochondrial Fragmentation Protein DRP1 Correlates with BRAF(V600E) Melanoma. J. Investig. Derm..

[14] Zhang G.E., Jin H.L., Lin X.K., Chen C., Liu X.S., Zhang Q., Yu J.R. (2013). Anti-tumor effects of Mfn2 in gastric cancer. Int. J. Mol. Sci..

[15] Zhao X., Tian C., Puszyk W.M., Ogunwobi O.O., Cao M., Wang T., Cabrera R., Nelson D.R., Liu C. (2013). OPA1 downregulation is involved in sorafenib-induced apoptosis in hepatocellular carcinoma. Lab. Investig..

[16] Peiris-Pages M., Bonuccelli G., Sotgia F., Lisanti M.P. (2018). Mitochondrial fission as a driver of stemness in tumor cells: mDIVI1 inhibits mitochondrial function, cell migration and cancer stem cell (CSC) signalling. Oncotarget.

[17] Rao V.A. (2019). Targeting Mitochondrial Fission to Trigger Cancer Cell Death. Cancer Res..

[18] Devine M.J., Kittler J.T. (2018). Mitochondria at the neuronal presynapse in health and disease. Nat. Rev. Neurosci..

[19] Perkins G.A., Renken C.W., Frey T.G., Ellisman M.H. (2001). Membrane architecture of mitochondria in neurons of the central nervous system. J. Neurosci. Res..

[20] Perkins G.A., Tjong J., Brown J.M., Poquiz P.H., Scott R.T., Kolson D.R., Ellisman M.H., Spirou G.A. (2010). The micro-architecture of mitochondria at active zones: Electron tomography reveals novel anchoring scaffolds and cristae structured for high-rate metabolism. J. Neurosci..

[21] Picard M., Shirihai O.S., Gentil B.J., Burelle Y. (2013). Mitochondrial morphology transitions and functions: Implications for retrograde signaling?. Am. J. Physiol. R. Integr. Comp Physiol..

[22] Li F., Wang Y., Zeller K.I., Potter J.J., Wonsey D.R., O’Donnell K.A., Kim J.W., Yustein J.T., Lee L.A., Dang C.V. (2005). Myc stimulates nuclearly encoded mitochondrial genes and mitochondrial biogenesis. Mol. Cell. Biol..

[23] Morrish F., Hockenbery D. (2014). MYC and mitochondrial biogenesis. Cold Spring Harb Perspect. Med..

[24] Agarwal E., Altman B.J., Ho Seo J., Bertolini I., Ghosh J.C., Kaur A., Kossenkov A.V., Languino L.R., Gabrilovich D.I., Speicher D.W. (2019). Myc Regulation of a Mitochondrial Trafficking Network Mediates Tumor Cell Invasion and Metastasis. Mol. Cell. Biol..

[25] Seo J.H., Agarwal E., Chae Y.C., Lee Y.G., Garlick D.S., Storaci A.M., Ferrero S., Gaudioso G., Gianelli U., Vaira V. (2019). Mitochondrial fission factor is a novel Myc-dependent regulator of mitochondrial permeability in cancer. EBioMedicine.

[26] Casinelli G., LaRosa J., Sharma M., Cherok E., Banerjee S., Branca M., Edmunds L., Wang Y., Sims-Lucas S., Churley L. (2016). n-Myc overexpression increases cisplatin resistance in neuroblastoma via deregulation of mitochondrial dynamics. Cell Death Discov..

[27] Zeng M., Kwiatkowski N.P., Zhang T., Nabet B., Xu M., Liang Y., Quan C., Wang J., Hao M., Palakurthi S. (2018). Targeting MYC dependency in ovarian cancer through inhibition of CDK7 and CDK12/13. eLife.

[28] Dimova I., Raitcheva S., Dimitrov R., Doganov N., Toncheva D. (2006). Correlations between c-myc gene copy-number and clinicopathological parameters of ovarian tumours. Eur. J. Cancer.

[29] Nagl N.G., Zweitzig D.R., Thimmapaya B., Beck G.R., Moran E. (2006). The c-myc gene is a direct target of mammalian SWI/SNF-related complexes during differentiation-associated cell cycle arrest. Cancer Res..

[30] Trizzino M., Barbieri E., Petracovici A., Wu S., Welsh S.A., Owens T.A., Licciulli S., Zhang R., Gardini A. (2018). The Tumor Suppressor ARID1A Controls Global Transcription via Pausing of RNA Polymerase II. Cell Rep..

[31] Emmings E., Mullany S., Chang Z., Landen C.N., Linder S., Bazzaro M. (2019). Targeting Mitochondria for Treatment of Chemoresistant Ovarian Cancer. Int. J. Mol. Sci..

[32] Molina J.R., Sun Y., Protopopova M., Gera S., Bandi M., Bristow C., McAfoos T., Morlacchi P., Ackroyd J., Agip A.A. (2018). An inhibitor of oxidative phosphorylation exploits cancer vulnerability. Nat. Med..

[33] Sica V., Bravo-San Pedro J.M., Stoll G., Kroemer G. (2020). Oxidative phosphorylation as a potential therapeutic target for cancer therapy. Int. J. Cancer.

[34] Zhang X., De Milito A., Demiroglu-Zergeroglu A., Gullbo J., D’Arcy P., Linder S. (2016). Eradicating Quiescent Tumor Cells by Targeting Mitochondrial Bioenergetics. Trends Cancer.

[35] Frattaruolo L., Brindisi M., Curcio R., Marra F., Dolce V., Cappello A.R. (2020). Targeting the Mitochondrial Metabolic Network: A Promising Strategy in Cancer Treatment. Int. J. Mol. Sci..

[36] Oliveira G.L., Coelho A.R., Marques R., Oliveira P.J. (2021). Cancer cell metabolism: Rewiring the mitochondrial hub. Biochim. Biophys. Acta Mol. Basis Dis..

[37] Bajpai R., Sharma A., Achreja A., Edgar C.L., Wei C., Siddiqa A.A., Gupta V.A., Matulis S.M., McBrayer S.K., Mittal A. (2020). Electron transport chain activity is a predictor and target for venetoclax sensitivity in multiple myeloma. Nat. Commun..

[38] Fischer G.M., Jalali A., Kircher D.A., Lee W.C., McQuade J.L., Haydu L.E., Joon A.Y., Reuben A., de Macedo M.P., Carapeto F.C.L. (2019). Molecular Profiling Reveals Unique Immune and Metabolic Features of Melanoma Brain Metastases. Cancer Discov..

[39] Lissanu Deribe Y., Sun Y., Terranova C., Khan F., Martinez-Ledesma J., Gay J., Gao G., Mullinax R.A., Khor T., Feng N. (2018). Mutations in the SWI/SNF complex induce a targetable dependence on oxidative phosphorylation in lung cancer. Nat. Med..

[40] Panina S.B., Pei J., Baran N., Konopleva M., Kirienko N.V. (2020). Utilizing Synergistic Potential of Mitochondria-Targeting Drugs for Leukemia Therapy. Front. Oncol..

[41] Sun Y., Bandi M., Lofton T., Smith M., Bristow C.A., Carugo A., Rogers N., Leonard P., Chang Q., Mullinax R. (2019). Functional Genomics Reveals Synthetic Lethality between Phosphogluconate Dehydrogenase and Oxidative Phosphorylation. Cell Rep..

[42] Teh J.L.F., Purwin T.J., Han A., Chua V., Patel P., Baqai U., Liao C., Bechtel N., Sato T., Davies M.A. (2020). Metabolic Adaptations to MEK and CDK4/6 Cotargeting in Uveal Melanoma. Mol. Cancer.

[43] Tsuji A., Akao T., Masuya T., Murai M., Miyoshi H. (2020). IACS-010759, a potent inhibitor of glycolysis-deficient hypoxic tumor cells, inhibits mitochondrial respiratory complex I through a unique mechanism. J. Biol. Chem..

[44] Vashisht Gopal Y.N., Gammon S., Prasad R., Knighton B., Pisaneschi F., Roszik J., Feng N., Johnson S., Pramanik S., Sudderth J. (2019). A Novel Mitochondrial Inhibitor Blocks MAPK Pathway and Overcomes MAPK Inhibitor Resistance in Melanoma. Clin. Cancer Res..

[45] Zhang L., Yao Y., Zhang S., Liu Y., Guo H., Ahmed M., Bell T., Zhang H., Han G., Lorence E. (2019). Metabolic reprogramming toward oxidative phosphorylation identifies a therapeutic target for mantle cell lymphoma. Sci. Transl. Med..

[46] Konovalova S. (2019). Analysis of Mitochondrial Respiratory Chain Complexes in Cultured Human Cells using Blue Native Polyacrylamide Gel Electrophoresis and Immunoblotting. J. Vis. Exp. Jove.

[47] McKenzie M., Lazarou M., Thorburn D.R., Ryan M.T. (2007). Analysis of mitochondrial subunit assembly into respiratory chain complexes using Blue Native polyacrylamide gel electrophoresis. Anal. Biochem..

[48] Van Coster R., Smet J., George E., De Meirleir L., Seneca S., Van Hove J., Sebire G., Verhelst H., De Bleecker J., Van Vlem B. (2001). Blue native polyacrylamide gel electrophoresis: A powerful tool in diagnosis of oxidative phosphorylation defects. Pediatr. Res..

[49] Lenaz G., Genova M.L. (2010). Structure and organization of mitochondrial respiratory complexes: A new understanding of an old subject. Antioxid Redox Signal.

[50] Hirst J. (2018). Open questions: Respiratory chain supercomplexes-why are they there and what do they do?. BMC Biol..

[51] Letts J.A., Sazanov L.A. (2017). Clarifying the supercomplex: The higher-order organization of the mitochondrial electron transport chain. Nat. Struct. Mol. Biol..

[52] Cichocki F., Wu C.Y., Zhang B., Felices M., Tesi B., Tuininga K., Dougherty P., Taras E., Hinderlie P., Blazar B.R. (2018). ARID5B regulates metabolic programming in human adaptive NK cells. J. Exp. Med..

[53] Xiao B., Deng X., Zhou W., Tan E.K. (2016). Flow Cytometry-Based Assessment of Mitophagy Using MitoTracker. Front. Cell Neurosci..

[54] Lee J.E., Westrate L.M., Wu H., Page C., Voeltz G.K. (2016). Multiple dynamin family members collaborate to drive mitochondrial division. Nature.

[55] Yu M., Nguyen N.D., Huang Y., Lin D., Fujimoto T.N., Molkentine J.M., Deorukhkar A., Kang Y., San Lucas F.A., Fernandes C.J. (2019). Mitochondrial fusion exploits a therapeutic vulnerability of pancreatic cancer. Jci Insight.

[56] Romero O.A., Setien F., John S., Gimenez-Xavier P., Gomez-Lopez G., Pisano D., Condom E., Villanueva A., Hager G.L., Sanchez-Cespedes M. (2012). The tumour suppressor and chromatin-remodelling factor BRG1 antagonizes Myc activity and promotes cell differentiation in human cancer. Embo Mol. Med..

[57] https://www.clinicaltrials.gov.

[58] Nasiri A.R., Rodrigues M.R., Li Z., Leitner B.P., Perry R.J. (2019). SGLT2 inhibition slows tumor growth in mice by reversing hyperinsulinemia. Cancer Metab..

[59] Zhang X., Fryknäs M., Hernlund E., Fayad W., De Milito A., Olofsson M.H., Gogvadze V., Dang L., Påhlman S., Schughart L.A. (2014). Induction of mitochondrial dysfunction as a strategy for targeting tumour cells in metabolically compromised microenvironments. Nat. Commun..

[60] Penet M.F., Krishnamachary B., Wildes F.B., Mironchik Y., Hung C.F., Wu T.C., Bhujwalla Z.M. (2018). Ascites Volumes and the Ovarian Cancer Microenvironment. Front. Oncol..

[61] Ahmed N., Stenvers K.L. (2013). Getting to know ovarian cancer ascites: Opportunities for targeted therapy-based translational research. Front. Oncol..

[62] Dang C.V., O’Donnell K.A., Zeller K.I., Nguyen T., Osthus R.C., Li F. (2006). The c-Myc target gene network. Semin. Cancer Biol..

[63] Hsieh A.L., Walton Z.E., Altman B.J., Stine Z.E., Dang C.V. (2015). MYC and metabolism on the path to cancer. Semin. Cell Dev. Biol..

[64] Stine Z.E., Walton Z.E., Altman B.J., Hsieh A.L., Dang C.V. (2015). MYC, Metabolism, and Cancer. Cancer Discov..

[65] Wallace D.C. (2012). Mitochondria and cancer. Nat. Rev. Cancer.

[66] Hirayama A., Kami K., Sugimoto M., Sugawara M., Toki N., Onozuka H., Kinoshita T., Saito N., Ochiai A., Tomita M. (2009). Quantitative metabolome profiling of colon and stomach cancer microenvironment by capillary electrophoresis time-of-flight mass spectrometry. Cancer Res..

[67] Yaromina A., Quennet V., Zips D., Meyer S., Shakirin G., Walenta S., Mueller-Klieser W., Baumann M. (2009). Co-localisation of hypoxia and perfusion markers with parameters of glucose metabolism in human squamous cell carcinoma (hSCC) xenografts. Int. J. Radiat Biol..

[68] Weinberg S.E., Chandel N.S. (2015). Targeting mitochondria metabolism for cancer therapy. Nat. Chem. Biol..

[69] Pavlova N.N., Thompson C.B. (2016). The Emerging Hallmarks of Cancer Metabolism. Cell Metab..

[70] Zong W.X., Rabinowitz J.D., White E. (2016). Mitochondria and Cancer. Mol. Cell.

[71] Minchinton A.I., Tannock I.F. (2006). Drug penetration in solid tumours. Nat. Rev. Cancer.

[72] Bernal S.D., Lampidis T.J., McIsaac R.M., Chen L.B. (1983). Anticarcinoma activity in vivo of rhodamine 123, a mitochondrial-specific dye. Science.

[73] Lampidis T.J., Bernal S.D., Summerhayes I.C., Chen L.B. (1983). Selective toxicity of rhodamine 123 in carcinoma cells in vitro. Cancer Res..

[74] Wenzel C., Riefke B., Grundemann S., Krebs A., Christian S., Prinz F., Osterland M., Golfier S., Rase S., Ansari N. (2014). 3D high-content screening for the identification of compounds that target cells in dormant tumor spheroid regions. Exp. Cell Res..

[75] Jha P., Wang X., Auwerx J. (2016). Analysis of Mitochondrial Respiratory Chain Supercomplexes Using Blue Native Polyacrylamide Gel Electrophoresis (BN-PAGE). Curr. Protoc. Mouse Biol..

[76] ImageJ Documentation Wiki. https://imagejdocu.tudor.lu/plugin/morphology/mitochondrial_morphology_macro_plug-in/start.

[77] Fayad W., Rickardson L., Haglund C., Olofsson M.H., D’Arcy P., Larsson R., Linder S., Fryknas M. (2011). Identification of agents that induce apoptosis of multicellular tumour spheroids: Enrichment for mitotic inhibitors with hydrophobic properties. Chem. Biol. Drug Des..

