# Supplementary material for: Targeting Mitochondrial Metabolism in Clear Cell Carcinoma of the Ovaries
Source: Int J Mol Sci. 2021 Apr 29;22(9):4750. doi: 10.3390/ijms22094750 (PMC8124918; doi:10.3390/ijms22094750)
Supplement: Supplementary file 1 [file ijms-22-04750-s001.zip › ijms-1196285-supplementary.pdf]

Supplementary Figure 1

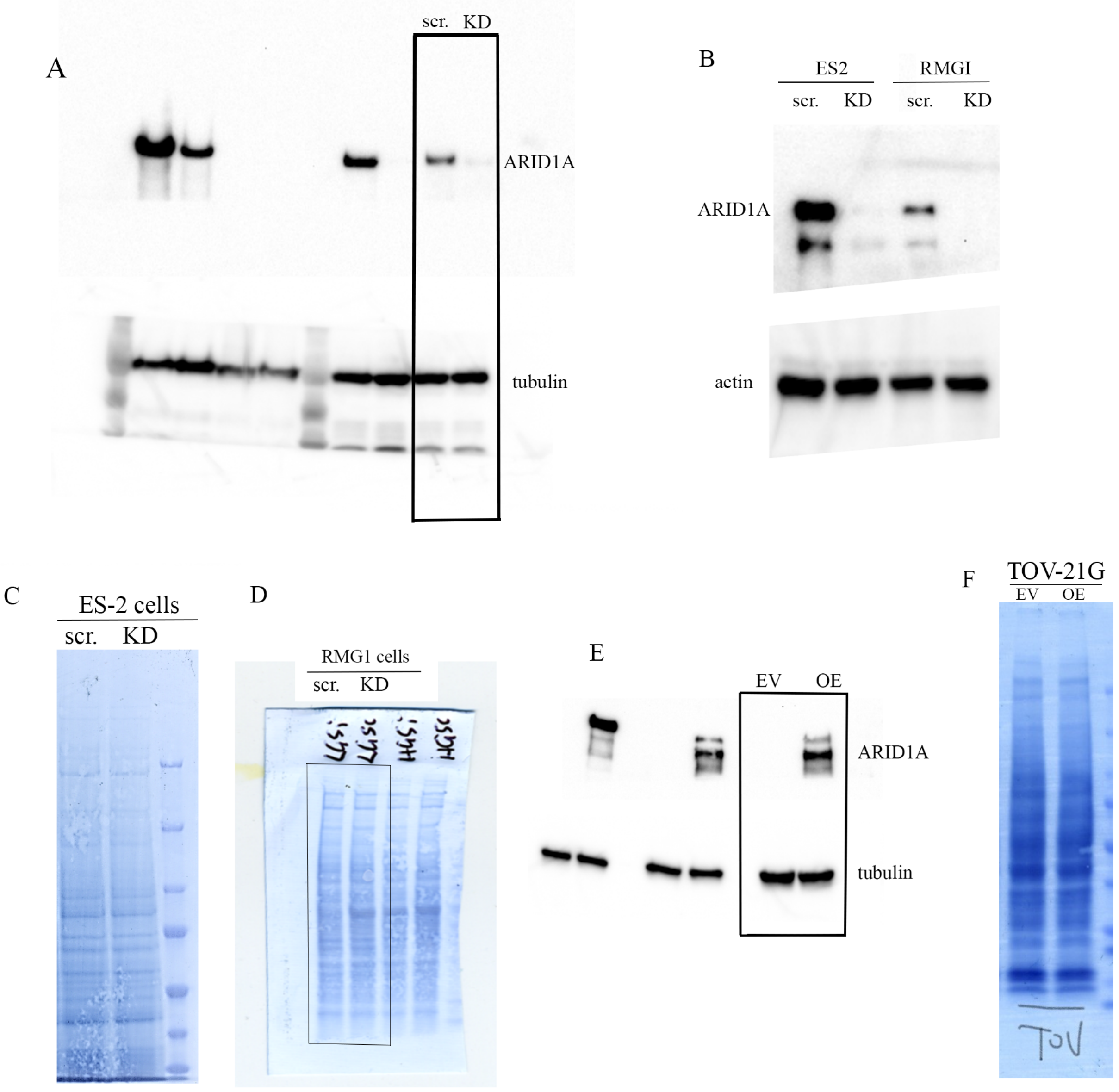

Supplementary Figure 2

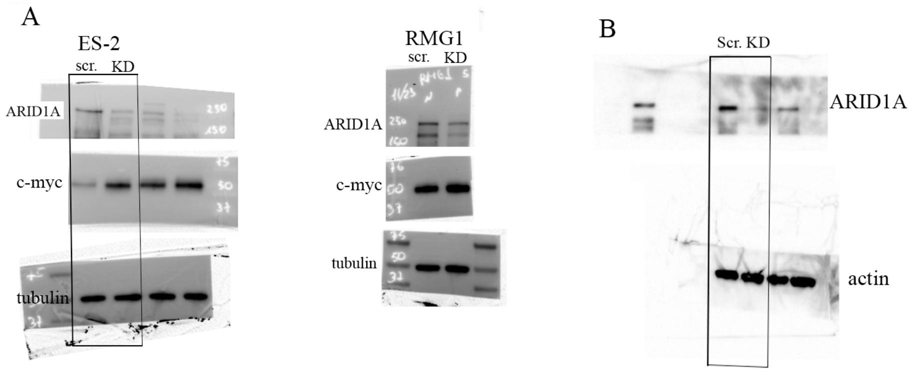

Supplementary Figure 3

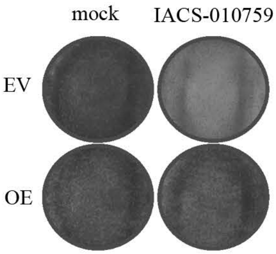

Feature ID	ES2 Scramble				
	Expression value 1	Expression value 2	Expression value 3	Average (Expression value)	Expression value 1
A1BG	89.00	76.00	87.00	84.00	69.00
A1BG-AS1	163.00	157.00	153.00	157.67	134.00
A2M	6.00	6.00	4.00	5.33	2.00
A2M-AS1	9.00	14.00	11.00	11.33	16.00
AAAS	1566.00	1587.00	1706.00	1619.67	1866.00
AACS	554.00	584.00	598.00	578.67	481.00
AACSP1	82.00	68.00	107.00	85.67	92.00
AADAC	6.00	4.00	1.00	3.67	2.00
AADAT	383.00	310.00	397.00	363.33	465.00
AAED1	107.00	108.00	115.00	110.00	143.00
AAGAB	1147.00	1109.00	1211.00	1155.67	1622.00
AAK1	1773.00	1610.00	1931.00	1771.33	1519.00
AAMDC	48.00	53.00	59.00	53.33	79.00
AAMP	2969.00	3063.00	3196.00	3076.00	3491.00
AANAT	5.00	7.00	5.00	5.67	5.00
AAR2	830.00	827.00	900.00	852.33	878.00
AARD	15.00	10.00	21.00	15.33	20.00
AARS	10691.00	10101.00	11170.00	10654.00	10259.00
AARS2	1364.00	1380.00	1406.00	1383.33	1358.00
AARSD1	7.00	17.00	11.00	11.67	20.00
AASDH	427.00	405.00	474.00	435.33	404.00
AASDHPPT	710.00	686.00	832.00	742.67	890.00
AASS	1932.00	1959.00	2217.00	2036.00	1919.00
AATK	4.00	3.00	4.00	3.67	1.00
ABAT	7.00	7.00	8.00	7.33	2.00
ABCA1	851.00	825.00	994.00	890.00	517.00
ABCA10	10.00	11.00	5.00	8.67	22.00
ABCA11P	137.00	105.00	160.00	134.00	178.00
ABCA13	4.00	7.00	7.00	6.00	8.00
ABCA2	1793.00	1838.00	2081.00	1904.00	1902.00
ABCA3	8.00	16.00	22.00	15.33	17.00
ABCA4	4.00	6.00	3.00	4.33	4.00
ABCA5	252.00	253.00	281.00	262.00	264.00
ABCA7	302.00	342.00	330.00	324.67	257.00
ABCA8	4.00	3.00	1.00	2.67	3.00
ABCB1	193.00	155.00	158.00	168.67	185.00
ABCB10	437.00	405.00	461.00	434.33	494.00
ABCB4	188.00	174.00	181.00	181.00	128.00
ABCB6	646.00	724.00	693.00	687.67	645.00
ABCB7	727.00	668.00	770.00	721.67	757.00
ABCB8	1736.00	1753.00	1764.00	1751.00	1852.00
ABCB9	384.00	335.00	306.00	341.67	244.00
ABCC1	364.00	355.00	367.00	362.00	339.00

ABCC10	1155.00	1104.00	1130.00	1129.67	1005.00
ABCC11	8.00	4.00	9.00	7.00	4.00
ABCC12	4.00	2.00	0.00	2.00	2.00
ABCC2	817.00	829.00	833.00	826.33	861.00
ABCC3	699.00	774.00	931.00	801.33	166.00
ABCC4	1761.00	1734.00	2020.00	1838.33	1863.00
ABCC5	3057.00	2982.00	3263.00	3100.67	2678.00
ABCC9	447.00	383.00	408.00	412.67	449.00
ABCD1	218.00	175.00	195.00	196.00	191.00
ABCD3	981.00	910.00	1048.00	979.67	1097.00
ABCD4	1344.00	1300.00	1314.00	1319.33	1194.00
ABCE1	2943.00	2798.00	3184.00	2975.00	3441.00
ABCF1	4.00	3.00	2.00	3.00	0.00
ABCF2	3563.00	3577.00	3726.00	3622.00	3920.00
ABCF3	1152.00	1220.00	1138.00	1170.00	1260.00
ABCG2	306.00	227.00	240.00	257.67	236.00
ABHD10	852.00	702.00	808.00	787.33	886.00
ABHD11	263.00	267.00	271.00	267.00	408.00
ABHD12	1178.00	1255.00	1203.00	1212.00	1151.00
ABHD13	363.00	328.00	388.00	359.67	439.00
ABHD14A	178.00	200.00	175.00	184.33	193.00
ABHD14B	3099.00	2884.00	3147.00	3043.33	3547.00
ABHD15	228.00	243.00	253.00	241.33	251.00
ABHD17A	392.00	470.00	528.00	463.33	541.00
ABHD17B	194.00	159.00	213.00	188.67	201.00
ABHD17C	254.00	245.00	265.00	254.67	297.00
ABHD2	4449.00	4138.00	4289.00	4292.00	3014.00
ABHD3	210.00	215.00	209.00	211.33	271.00
ABHD4	908.00	848.00	998.00	918.00	675.00
ABHD5	1089.00	1064.00	1199.00	1117.33	1016.00
ABHD6	271.00	243.00	229.00	247.67	333.00
ABHD8	123.00	125.00	173.00	140.33	132.00
ABI1	1361.00	1205.00	1515.00	1360.33	1548.00
ABI2	2292.00	2327.00	2493.00	2370.67	2540.00
ABI3	72.00	75.00	81.00	76.00	70.00
ABI3BP	421.00	378.00	459.00	419.33	293.00
ABL1	4897.00	4562.00	4902.00	4787.00	6225.00
ABL2	6297.00	5713.00	6780.00	6263.33	6395.00
ABLIM1	11.00	19.00	15.00	15.00	20.00
ABLIM2	4.00	4.00	5.00	4.33	11.00
ABLIM3	2225.00	2281.00	2362.00	2289.33	1396.00
ABRACL	958.00	902.00	970.00	943.33	1161.00
ABT1	699.00	730.00	795.00	741.33	945.00
ABTB1	1013.00	1110.00	1154.00	1092.33	1136.00
ABTB2	161.00	154.00	187.00	167.33	102.00
ACAA1	747.00	817.00	801.00	788.33	903.00
ACAA2	176.00	157.00	183.00	172.00	314.00
ACACA	26.00	19.00	19.00	21.33	16.00

ACACB	30.00	27.00	27.00	28.00	30.00
ACAD10	1311.00	1297.00	1286.00	1298.00	1246.00
ACAD11	182.00	238.00	254.00	224.67	194.00
ACAD8	465.00	520.00	571.00	518.67	452.00
ACAD9	804.00	868.00	877.00	849.67	905.00
ACADL	14.00	7.00	6.00	9.00	11.00
ACADM	860.00	758.00	864.00	827.33	1119.00
ACADS	104.00	122.00	109.00	111.67	124.00
ACADSB	612.00	557.00	673.00	614.00	587.00
ACADVL	6792.00	6583.00	7372.00	6915.67	7301.00
ACAP1	9.00	7.00	4.00	6.67	11.00
ACAP2	1416.00	1189.00	1506.00	1370.33	1621.00
ACAP3	813.00	897.00	916.00	875.33	755.00
ACAT1	1207.00	1096.00	1186.00	1163.00	1643.00
ACAT2	298.00	266.00	304.00	289.33	398.00
ACBD3	922.00	896.00	969.00	929.00	942.00
ACBD4	214.00	205.00	226.00	215.00	225.00
ACBD5	794.00	750.00	857.00	800.33	909.00
ACBD6	754.00	759.00	836.00	783.00	843.00
ACBD7	35.00	48.00	45.00	42.67	63.00
ACCS	377.00	374.00	365.00	372.00	433.00
ACD	601.00	552.00	556.00	569.67	700.00
ACER2	21.00	10.00	27.00	19.33	38.00
ACER3	782.00	836.00	826.00	814.67	824.00
ACIN1	6503.00	6554.00	6951.00	6669.33	6151.00
ACLY	6580.00	6019.00	6704.00	6434.33	6542.00
ACO1	2611.00	2348.00	2849.00	2602.67	2402.00
ACO2	2181.00	2296.00	2165.00	2214.00	2543.00
ACOT1	7.00	9.00	8.00	8.00	18.00
ACOT13	469.00	478.00	535.00	494.00	780.00
ACOT2	119.00	140.00	153.00	137.33	161.00
ACOT7	1062.00	1070.00	1092.00	1074.67	1645.00
ACOT8	420.00	396.00	392.00	402.67	448.00
ACOT9	589.00	561.00	589.00	579.67	659.00
ACOX1	559.00	516.00	587.00	554.00	550.00
ACOX2	29.00	30.00	35.00	31.33	82.00
ACOX3	317.00	272.00	301.00	296.67	290.00
ACP1	1556.00	1425.00	1837.00	1606.00	2087.00
ACP2	764.00	793.00	703.00	753.33	727.00
ACP5	261.00	313.00	248.00	274.00	257.00
ACP6	409.00	428.00	443.00	426.67	408.00
ACRBP	24.00	25.00	33.00	27.33	18.00
ACRC	199.00	205.00	219.00	207.67	290.00
ACRV1	21.00	16.00	13.00	16.67	14.00
ACSF2	406.00	396.00	387.00	396.33	304.00
ACSF3	669.00	672.00	676.00	672.33	714.00
ACSL1	487.00	523.00	512.00	507.33	478.00
ACSL3	3659.00	3392.00	3566.00	3539.00	3691.00

ACSL4	4029.00	3467.00	4422.00	3972.67	4039.00
ACSL5	678.00	627.00	679.00	661.33	458.00
ACSM3	5.00	4.00	13.00	7.33	8.00
ACSS2	1004.00	958.00	1051.00	1004.33	707.00
ACTA2	46.00	47.00	63.00	52.00	65.00
ACTA2-AS1	11.00	5.00	15.00	10.33	6.00
ACTB	44106.00	42682.00	45460.00	44082.67	60140.00
ACTG1	38194.00	36794.00	40707.00	38565.00	46347.00
ACTG1P17	33.00	13.00	24.00	23.33	36.00
ACTG1P20	60.00	67.00	62.00	63.00	44.00
ACTG1P4	25.00	21.00	24.00	23.33	18.00
ACTL10	26.00	34.00	22.00	27.33	28.00
ACTL6A	1647.00	1550.00	1753.00	1650.00	2190.00
ACTN1	7550.00	7167.00	7514.00	7410.33	6664.00
ACTN1-AS1	4.00	2.00	0.00	2.00	1.00
ACTN4	16481.00	15545.00	16400.00	16142.00	16248.00
ACTR10	969.00	1001.00	1086.00	1018.67	1267.00
ACTR1A	2586.00	2572.00	2640.00	2599.33	2726.00
ACTR1B	1255.00	1281.00	1247.00	1261.00	1383.00
ACTR2	5665.00	4986.00	5659.00	5436.67	6875.00
ACTR3	5797.00	5159.00	5781.00	5579.00	6436.00
ACTR3B	385.00	370.00	440.00	398.33	510.00
ACTR3C	4.00	9.00	6.00	6.33	7.00
ACTR5	439.00	443.00	454.00	445.33	429.00
ACTR6	487.00	463.00	509.00	486.33	600.00
ACTR8	570.00	576.00	612.00	586.00	601.00
ACTRT3	100.00	75.00	79.00	84.67	88.00
ACVR1	1420.00	1324.00	1501.00	1415.00	1524.00
ACVR1B	911.00	968.00	909.00	929.33	913.00
ACVR1C	25.00	18.00	30.00	24.33	17.00
ACVR2A	323.00	318.00	315.00	318.67	345.00
ACVR2B	506.00	499.00	593.00	532.67	585.00
ACVR2B-AS1	29.00	21.00	19.00	23.00	22.00
ACVRL1	6.00	4.00	0.00	3.33	5.00
ACY1	55.00	46.00	51.00	50.67	89.00
ACYP1	186.00	258.00	247.00	230.33	341.00
ACYP2	44.00	44.00	40.00	42.67	59.00
ADA	142.00	123.00	143.00	136.00	191.00
ADAL	391.00	427.00	408.00	408.67	369.00
ADAM10	2859.00	2648.00	2990.00	2832.33	2977.00
ADAM11	60.00	70.00	61.00	63.67	66.00
ADAM12	5016.00	4677.00	4792.00	4828.33	3050.00
ADAM15	5388.00	5480.00	5665.00	5511.00	3554.00
ADAM17	1535.00	1465.00	1605.00	1535.00	1411.00
ADAM19	860.00	922.00	916.00	899.33	662.00
ADAM1A	307.00	302.00	311.00	306.67	358.00
ADAM20	7.00	3.00	12.00	7.33	0.00
ADAM20P1	37.00	29.00	55.00	40.33	39.00

ADAM21	18.00	16.00	13.00	15.67	16.00
ADAM22	361.00	404.00	417.00	394.00	423.00
ADAM23	841.00	835.00	890.00	855.33	918.00
ADAM28	109.00	90.00	108.00	102.33	73.00
ADAM32	17.00	6.00	4.00	9.00	6.00
ADAM8	53.00	69.00	67.00	63.00	59.00
ADAM9	5697.00	5365.00	5938.00	5666.67	4709.00
ADAMTS1	18464.00	18480.00	21072.00	19338.67	14234.00
ADAMTS12	1145.00	997.00	1126.00	1089.33	729.00
ADAMTS13	262.00	252.00	269.00	261.00	269.00
ADAMTS15	21.00	19.00	18.00	19.33	5.00
ADAMTS16	411.00	341.00	394.00	382.00	338.00
ADAMTS17	96.00	96.00	88.00	93.33	67.00
ADAMTS19	16.00	15.00	25.00	18.67	32.00
ADAMTS2	3082.00	2917.00	3185.00	3061.33	2751.00
ADAMTS20	4.00	1.00	5.00	3.33	1.00
ADAMTS3	209.00	213.00	257.00	226.33	185.00
ADAMTS4	211.00	189.00	171.00	190.33	141.00
ADAMTS5	185.00	145.00	151.00	160.33	185.00
ADAMTS6	2583.00	2548.00	2887.00	2672.67	2928.00
ADAMTS9	68.00	46.00	39.00	51.00	53.00
ADAMTS9-AS2	10.00	12.00	10.00	10.67	7.00
ADAMTSL1	9595.00	9557.00	9833.00	9661.67	8692.00
ADAMTSL3	38.00	31.00	31.00	33.33	24.00
ADAMTSL4	134.00	137.00	139.00	136.67	88.00
ADAMTSL4-AS1	6.00	9.00	4.00	6.33	10.00
ADAMTSL5	224.00	239.00	276.00	246.33	223.00
ADAP1	14.00	12.00	7.00	11.00	9.00
ADAP2	13.00	5.00	11.00	9.67	9.00
ADAR	5809.00	5227.00	5815.00	5617.00	5271.00
ADARB1	1918.00	1879.00	1989.00	1928.67	1647.00
ADAT1	596.00	579.00	564.00	579.67	416.00
ADAT2	993.00	1004.00	1025.00	1007.33	1170.00
ADAT3	77.00	68.00	71.00	72.00	62.00
ADCK1	237.00	196.00	285.00	239.33	292.00
ADCK2	860.00	848.00	893.00	867.00	793.00
ADCK3	813.00	821.00	894.00	842.67	982.00
ADCK4	263.00	299.00	357.00	306.33	297.00
ADCK5	309.00	366.00	331.00	335.33	347.00
ADCY1	156.00	134.00	148.00	146.00	145.00
ADCY10P1	608.00	597.00	718.00	641.00	496.00
ADCY3	1937.00	2041.00	2107.00	2028.33	2051.00
ADCY6	1831.00	1768.00	1901.00	1833.33	1498.00
ADCY7	1566.00	1478.00	1541.00	1528.33	1666.00
ADCY8	4.00	3.00	4.00	3.67	6.00
ADCY9	554.00	602.00	583.00	579.67	532.00
ADD1	7705.00	7631.00	8008.00	7781.33	7392.00
ADD3	615.00	532.00	619.00	588.67	549.00

ADGRA2	488.00	523.00	509.00	506.67	349.00
ADGRA3	935.00	906.00	1038.00	959.67	899.00
ADGRB2	231.00	171.00	202.00	201.33	176.00
ADGRB3	134.00	141.00	117.00	130.67	128.00
ADGRE1	6.00	3.00	3.00	4.00	2.00
ADGRE5	1282.00	1340.00	1290.00	1304.00	1062.00
ADGRF3	18.00	15.00	16.00	16.33	9.00
ADGRF4	142.00	152.00	131.00	141.67	44.00
ADGRF5	33.00	35.00	40.00	36.00	10.00
ADGRG1	98.00	68.00	82.00	82.67	48.00
ADGRG4	24.00	28.00	25.00	25.67	8.00
ADGRG6	116.00	79.00	115.00	103.33	75.00
ADGRL1	184.00	188.00	210.00	194.00	234.00
ADGRL2	1478.00	1229.00	1473.00	1393.33	1365.00
ADGRL3	4.00	0.00	0.00	1.33	2.00
ADGRL4	4327.00	4042.00	4142.00	4170.33	3464.00
ADGRV1	113.00	92.00	131.00	112.00	99.00
ADH5	1311.00	1190.00	1346.00	1282.33	1483.00
ADHFE1	84.00	104.00	103.00	97.00	74.00
ADI1	352.00	324.00	358.00	344.67	384.00
ADIPOR1	1686.00	1575.00	1634.00	1631.67	1985.00
ADIPOR2	2796.00	2596.00	2758.00	2716.67	2730.00
ADK	1840.00	1595.00	1919.00	1784.67	1785.00
ADM	14.00	9.00	8.00	10.33	10.00
ADM2	389.00	357.00	416.00	387.33	391.00
ADM5	212.00	178.00	251.00	213.67	168.00
ADNP	4332.00	4210.00	4905.00	4482.33	4417.00
ADNP2	800.00	732.00	800.00	777.33	870.00
ADNP-AS1	32.00	31.00	49.00	37.33	35.00
ADO	1263.00	1164.00	1382.00	1269.67	1511.00
ADORA2A	9.00	17.00	15.00	13.67	7.00
ADORA2B	3049.00	2873.00	3070.00	2997.33	2045.00
ADPGK	1991.00	1824.00	2010.00	1941.67	2019.00
ADPRH	24.00	29.00	36.00	29.67	32.00
ADPRHL1	36.00	21.00	25.00	27.33	32.00
ADPRHL2	560.00	572.00	547.00	559.67	620.00
ADPRM	177.00	186.00	229.00	197.33	236.00
ADRB1	25.00	35.00	24.00	28.00	39.00
ADRB2	2312.00	2193.00	2174.00	2226.33	2751.00
ADRBK1	2329.00	2203.00	2341.00	2291.00	2515.00
ADRBK2	65.00	48.00	52.00	55.00	83.00
ADRM1	2800.00	2899.00	2920.00	2873.00	3880.00
ADSL	1703.00	1753.00	1763.00	1739.67	2271.00
ADSS	1430.00	1317.00	1495.00	1414.00	1618.00
ADSSL1	17.00	11.00	15.00	14.33	8.00
ADTRP	3864.00	3744.00	3808.00	3805.33	3779.00
AEBP1	6993.00	7310.00	7334.00	7212.33	5025.00
AEBP2	860.00	790.00	892.00	847.33	1045.00

AEN	1202.00	1210.00	1283.00	1231.67	1286.00
AES	3509.00	3344.00	3567.00	3473.33	4161.00
AFAP1	4590.00	4200.00	4681.00	4490.33	4009.00
AFAP1L1	962.00	959.00	1026.00	982.33	958.00
AFAP1L2	56.00	62.00	68.00	62.00	33.00
AFF1	707.00	628.00	732.00	689.00	482.00
AFF2	15.00	15.00	17.00	15.67	7.00
AFF3	1462.00	1239.00	1464.00	1388.33	1420.00
AFF4	3267.00	2706.00	3493.00	3155.33	2775.00
AFG3L1P	1518.00	1619.00	1638.00	1591.67	1631.00
AFG3L2	2499.00	2377.00	2641.00	2505.67	3153.00
AFMID	623.00	521.00	560.00	568.00	678.00
AFP	6.00	0.00	1.00	2.33	1.00
AFTPH	620.00	593.00	743.00	652.00	702.00
AGA	785.00	798.00	804.00	795.67	827.00
AGAP1	850.00	787.00	877.00	838.00	833.00
AGAP11	52.00	33.00	42.00	42.33	57.00
AGAP1-IT1	7.00	0.00	3.00	3.33	2.00
AGAP2	21.00	13.00	16.00	16.67	20.00
AGAP2-AS1	353.00	304.00	383.00	346.67	303.00
AGAP3	2852.00	2958.00	3152.00	2987.33	3134.00
AGAP4	250.00	214.00	295.00	253.00	231.00
AGAP5	163.00	185.00	214.00	187.33	176.00
AGAP6	848.00	896.00	949.00	897.67	918.00
AGAP7P	97.00	87.00	107.00	97.00	106.00
AGAP9	488.00	521.00	473.00	494.00	462.00
AGBL2	56.00	55.00	77.00	62.67	57.00
AGBL3	86.00	74.00	77.00	79.00	99.00
AGBL5	839.00	739.00	767.00	781.67	793.00
AGFG1	1429.00	1329.00	1574.00	1444.00	1571.00
AGFG2	279.00	248.00	286.00	271.00	191.00
AGGF1	732.00	684.00	727.00	714.33	766.00
AGK	331.00	331.00	337.00	333.00	339.00
AGL	719.00	591.00	788.00	699.33	707.00
AGMAT	91.00	116.00	111.00	106.00	117.00
AGO1	659.00	619.00	655.00	644.33	568.00
AGO2	577.00	501.00	588.00	555.33	488.00
AGO3	240.00	225.00	207.00	224.00	252.00
AGO4	800.00	830.00	957.00	862.33	727.00
AGPAT1	40.00	48.00	50.00	46.00	54.00
AGPAT2	662.00	764.00	749.00	725.00	878.00
AGPAT3	2672.00	2631.00	2717.00	2673.33	2622.00
AGPAT4	3030.00	3118.00	3488.00	3212.00	2461.00
AGPAT4-IT1	121.00	130.00	145.00	132.00	69.00
AGPAT5	2363.00	2225.00	2297.00	2295.00	2736.00
AGPS	1420.00	1224.00	1551.00	1398.33	1379.00
AGRN	7531.00	7315.00	7654.00	7500.00	4760.00
AGTPBP1	522.00	453.00	513.00	496.00	691.00

AGTR1	64.00	60.00	60.00	61.33	58.00
AGTRAP	758.00	825.00	753.00	778.67	909.00
AHCTF1	2414.00	2132.00	2565.00	2370.33	2468.00
AHCTF1P1	215.00	198.00	243.00	218.67	187.00
AHCY	5401.00	5048.00	5756.00	5401.67	7373.00
AHCYL1	2545.00	2351.00	2610.00	2502.00	2384.00
AHCYL2	871.00	794.00	924.00	863.00	764.00
AHDC1	436.00	446.00	465.00	449.00	284.00
AHI1	963.00	1004.00	1095.00	1020.67	1155.00
AHNAK	44265.00	41138.00	45687.00	43696.67	30121.00
AHNAK2	1746.00	1739.00	1960.00	1815.00	2125.00
AHR	339.00	273.00	429.00	347.00	283.00
AHRR	1459.00	1347.00	1720.00	1508.67	1411.00
AHSA1	2601.00	2722.00	2800.00	2707.67	3432.00
AHSA2	3294.00	3431.00	3635.00	3453.33	3325.00
AIDA	352.00	307.00	393.00	350.67	389.00
AIF1L	4.00	1.00	5.00	3.33	5.00
AIFM1	816.00	791.00	840.00	815.67	956.00
AIFM2	573.00	548.00	566.00	562.33	601.00
AIG1	1291.00	1240.00	1203.00	1244.67	1153.00
AIM1	114.00	127.00	103.00	114.67	59.00
AIM1L	13.00	18.00	4.00	11.67	9.00
AIM2	103.00	78.00	83.00	88.00	63.00
AIMP1	1493.00	1427.00	1560.00	1493.33	1820.00
AIMP2	1169.00	1104.00	1267.00	1180.00	1424.00
AIP	868.00	857.00	814.00	846.33	1039.00
AJUBA	4000.00	3746.00	4086.00	3944.00	2686.00
AK1	540.00	516.00	514.00	523.33	565.00
AK2	4912.00	4837.00	5218.00	4989.00	5591.00
AK3	1091.00	1107.00	1269.00	1155.67	1352.00
AK4	361.00	338.00	433.00	377.33	429.00
AK5	1043.00	901.00	1039.00	994.33	998.00
AK6	8.00	1.00	0.00	3.00	0.00
AK9	129.00	131.00	125.00	128.33	125.00
AKAP1	2122.00	2188.00	2225.00	2178.33	2097.00
AKAP10	782.00	771.00	919.00	824.00	926.00
AKAP11	1890.00	1641.00	1971.00	1834.00	1616.00
AKAP12	20215.00	18166.00	20185.00	19522.00	14538.00
AKAP13	2322.00	2191.00	2501.00	2338.00	2031.00
AKAP2	97.00	104.00	92.00	97.67	70.00
AKAP3	9.00	4.00	7.00	6.67	9.00
AKAP5	4.00	11.00	10.00	8.33	8.00
AKAP6	56.00	60.00	70.00	62.00	49.00
AKAP7	72.00	68.00	78.00	72.67	125.00
AKAP8	1039.00	1060.00	1108.00	1069.00	1056.00
AKAP8L	1427.00	1496.00	1513.00	1478.67	1431.00
AKAP9	1723.00	1594.00	1795.00	1704.00	1481.00
AKIP1	477.00	495.00	543.00	505.00	575.00

AKIRIN1	1465.00	1415.00	1515.00	1465.00	1762.00
AKIRIN2	1128.00	1093.00	1168.00	1129.67	1330.00
AKNA	422.00	360.00	397.00	393.00	381.00
AKNAD1	12.00	12.00	6.00	10.00	17.00
AKR1A1	1467.00	1531.00	1627.00	1541.67	2047.00
AKR1B1	2030.00	1972.00	2160.00	2054.00	2136.00
AKR1C1	32.00	37.00	28.00	32.33	30.00
AKR1C2	7.00	8.00	5.00	6.67	8.00
AKR1C3	80.00	75.00	90.00	81.67	104.00
AKR1C8P	5.00	2.00	1.00	2.67	2.00
AKR1E2	54.00	46.00	53.00	51.00	60.00
AKR7A2	763.00	833.00	855.00	817.00	1039.00
AKR7A3	18.00	12.00	11.00	13.67	9.00
AKR7L	12.00	16.00	25.00	17.67	17.00
AKT1	2920.00	2830.00	2990.00	2913.33	3137.00
AKT1S1	1217.00	1177.00	1304.00	1232.67	1437.00
AKT2	2596.00	2680.00	2853.00	2709.67	2749.00
AKTIP	371.00	327.00	401.00	366.33	438.00
ALAD	484.00	538.00	531.00	517.67	516.00
ALAS1	1038.00	896.00	1034.00	989.33	1148.00
ALB	7.00	4.00	2.00	4.33	3.00
ALCAM	6060.00	5412.00	5944.00	5805.33	6119.00
ALDH16A1	826.00	852.00	815.00	831.00	980.00
ALDH18A1	4632.00	4685.00	4527.00	4614.67	5220.00
ALDH1A3	12506.00	11741.00	13785.00	12677.33	22207.00
ALDH1B1	549.00	519.00	555.00	541.00	619.00
ALDH1L1	14.00	22.00	16.00	17.33	21.00
ALDH1L2	1321.00	1142.00	1197.00	1220.00	1119.00
ALDH2	4.00	3.00	4.00	3.67	4.00
ALDH3A2	374.00	312.00	372.00	352.67	303.00
ALDH3B1	211.00	221.00	198.00	210.00	88.00
ALDH4A1	479.00	484.00	476.00	479.67	424.00
ALDH5A1	161.00	194.00	177.00	177.33	228.00
ALDH6A1	354.00	351.00	382.00	362.33	324.00
ALDH7A1	1145.00	1159.00	1146.00	1150.00	1448.00
ALDH8A1	13.00	9.00	21.00	14.33	13.00
ALDH9A1	1297.00	1264.00	1321.00	1294.00	1557.00
ALDOA	11428.00	11193.00	11490.00	11370.33	14280.00
ALDOC	80.00	69.00	95.00	81.33	133.00
ALG1	1210.00	1250.00	1299.00	1253.00	1326.00
ALG10	237.00	238.00	243.00	239.33	232.00
ALG10B	1333.00	1122.00	1227.00	1227.33	1212.00
ALG11	177.00	194.00	178.00	183.00	112.00
ALG12	502.00	508.00	500.00	503.33	538.00
ALG13	2429.00	2606.00	2928.00	2654.33	2424.00
ALG14	139.00	161.00	163.00	154.33	276.00
ALG1L9P	10.00	13.00	13.00	12.00	7.00
ALG2	1053.00	1008.00	1090.00	1050.33	1089.00

ALG3	1771.00	1667.00	1675.00	1704.33	1976.00
ALG5	626.00	588.00	530.00	581.33	791.00
ALG6	542.00	607.00	635.00	594.67	749.00
ALG8	1206.00	1191.00	1166.00	1187.67	1235.00
ALG9	922.00	843.00	941.00	902.00	719.00
ALKBH1	243.00	199.00	245.00	229.00	267.00
ALKBH2	484.00	507.00	502.00	497.67	682.00
ALKBH3	506.00	495.00	575.00	525.33	661.00
ALKBH4	200.00	199.00	237.00	212.00	259.00
ALKBH5	1627.00	1455.00	1624.00	1568.67	1778.00
ALKBH6	401.00	429.00	373.00	401.00	436.00
ALKBH7	301.00	346.00	389.00	345.33	442.00
ALKBH8	317.00	311.00	326.00	318.00	354.00
ALMS1	556.00	494.00	571.00	540.33	512.00
ALMS1-IT1	5.00	10.00	15.00	10.00	20.00
ALOX12-AS1	73.00	81.00	56.00	70.00	70.00
ALOX15P1	4.00	4.00	5.00	4.33	7.00
ALOXE3	35.00	25.00	22.00	27.33	11.00
ALPK1	532.00	469.00	508.00	503.00	477.00
ALPK2	16.00	10.00	11.00	12.33	6.00
ALPK3	15.00	10.00	17.00	14.00	9.00
ALS2	1237.00	1170.00	1329.00	1245.33	1320.00
ALS2CR11	30.00	15.00	15.00	20.00	33.00
ALS2CR12	26.00	28.00	36.00	30.00	20.00
ALX1	160.00	185.00	174.00	173.00	215.00
ALYREF	879.00	954.00	1050.00	961.00	1280.00
AMACR	76.00	43.00	76.00	65.00	87.00
AMBRA1	939.00	939.00	936.00	938.00	713.00
AMD1	1520.00	1369.00	1568.00	1485.67	1566.00
AMDHD2	1219.00	1223.00	1191.00	1211.00	1327.00
AMER1	421.00	462.00	552.00	478.33	396.00
AMFR	2156.00	2125.00	2241.00	2174.00	2383.00
AMH	458.00	486.00	577.00	507.00	453.00
AMIGO1	95.00	96.00	85.00	92.00	80.00
AMIGO2	9225.00	8467.00	9703.00	9131.67	7662.00
AMIGO3	164.00	159.00	156.00	159.67	156.00
AMMECR1	396.00	377.00	457.00	410.00	443.00
AMMECR1L	1551.00	1509.00	1589.00	1549.67	1448.00
AMN	5.00	5.00	2.00	4.00	2.00
AMN1	93.00	91.00	101.00	95.00	136.00
AMOTL1	2328.00	2063.00	2360.00	2250.33	1760.00
AMOTL2	8806.00	8382.00	9225.00	8804.33	7877.00
AMPD2	3201.00	3352.00	3504.00	3352.33	3514.00
AMPD3	807.00	790.00	829.00	808.67	693.00
AMT	508.00	600.00	574.00	560.67	572.00
AMY2B	194.00	195.00	213.00	200.67	213.00
AMZ2	1586.00	1505.00	1698.00	1596.33	1841.00
AMZ2P1	259.00	252.00	283.00	264.67	311.00

ANAPC1	1587.00	1452.00	1674.00	1571.00	1432.00
ANAPC10	119.00	121.00	141.00	127.00	168.00
ANAPC11	1180.00	1187.00	1272.00	1213.00	1614.00
ANAPC13	1956.00	1775.00	2056.00	1929.00	2403.00
ANAPC15	291.00	275.00	325.00	297.00	404.00
ANAPC16	1387.00	1296.00	1486.00	1389.67	1669.00
ANAPC2	1114.00	1138.00	1152.00	1134.67	935.00
ANAPC4	705.00	710.00	694.00	703.00	882.00
ANAPC5	2505.00	2438.00	2628.00	2523.67	2794.00
ANAPC7	1879.00	1848.00	1834.00	1853.67	2233.00
ANG	13.00	22.00	19.00	18.00	17.00
ANGEL1	1240.00	1108.00	1254.00	1200.67	1007.00
ANGEL2	942.00	873.00	1026.00	947.00	1138.00
ANGPT1	55.00	46.00	46.00	49.00	93.00
ANGPT2	462.00	431.00	437.00	443.33	845.00
ANGPTL2	37.00	56.00	58.00	50.33	63.00
ANGPTL4	62.00	60.00	63.00	61.67	229.00
ANGPTL6	7.00	3.00	0.00	3.33	4.00
ANK2	1005.00	900.00	1048.00	984.33	980.00
ANK3	1187.00	970.00	1236.00	1131.00	1136.00
ANKAR	88.00	82.00	108.00	92.67	87.00
ANKDD1A	21.00	24.00	27.00	24.00	21.00
ANKDD1B	7.00	8.00	6.00	7.00	3.00
ANKEF1	109.00	110.00	112.00	110.33	127.00
ANKFN1	109.00	80.00	110.00	99.67	96.00
ANKFY1	2685.00	2414.00	2645.00	2581.33	2538.00
ANKH	1067.00	1092.00	1135.00	1098.00	1039.00
ANKHD1	306.00	259.00	308.00	291.00	338.00
ANKHD1-EIF4EBP3	14.00	26.00	41.00	27.00	27.00
ANKIB1	1371.00	1296.00	1442.00	1369.67	1446.00
ANKK1	22.00	13.00	11.00	15.33	12.00
ANKLE1	263.00	295.00	314.00	290.67	313.00
ANKLE2	3045.00	2942.00	3035.00	3007.33	2784.00
ANKMY1	596.00	607.00	635.00	612.67	406.00
ANKMY2	493.00	468.00	473.00	478.00	543.00
ANKRA2	295.00	312.00	349.00	318.67	327.00
ANKRD1	12519.00	12097.00	12925.00	12513.67	12452.00
ANKRD10	1498.00	1588.00	1724.00	1603.33	1629.00
ANKRD11	12039.00	11491.00	12443.00	11991.00	8932.00
ANKRD12	1286.00	1120.00	1373.00	1259.67	1200.00
ANKRD13A	1545.00	1459.00	1657.00	1553.67	1825.00
ANKRD13B	212.00	205.00	225.00	214.00	267.00
ANKRD13C	623.00	617.00	663.00	634.33	612.00
ANKRD13D	938.00	1020.00	1107.00	1021.67	1115.00
ANKRD16	293.00	322.00	316.00	310.33	346.00
ANKRD17	2407.00	2235.00	2686.00	2442.67	2157.00
ANKRD18A	245.00	246.00	263.00	251.33	262.00
ANKRD18B	191.00	178.00	260.00	209.67	249.00

ANKRD19P	295.00	342.00	343.00	326.67	337.00
ANKRD20A5P	28.00	36.00	24.00	29.33	13.00
ANKRD23	116.00	114.00	135.00	121.67	78.00
ANKRD24	76.00	93.00	113.00	94.00	66.00
ANKRD26	903.00	868.00	976.00	915.67	970.00
ANKRD27	872.00	759.00	890.00	840.33	967.00
ANKRD28	3197.00	2995.00	3486.00	3226.00	3664.00
ANKRD29	265.00	330.00	346.00	313.67	241.00
ANKRD31	28.00	28.00	27.00	27.67	34.00
ANKRD33B	1472.00	1346.00	1626.00	1481.33	676.00
ANKRD34A	162.00	140.00	125.00	142.33	135.00
ANKRD36	89.00	109.00	103.00	100.33	92.00
ANKRD36B	69.00	53.00	59.00	60.33	70.00
ANKRD36BP2	61.00	59.00	60.00	60.00	68.00
ANKRD36C	109.00	89.00	110.00	102.67	79.00
ANKRD37	35.00	40.00	45.00	40.00	45.00
ANKRD39	118.00	108.00	96.00	107.33	127.00
ANKRD40	1688.00	1527.00	1758.00	1657.67	1694.00
ANKRD42	372.00	341.00	334.00	349.00	266.00
ANKRD44	332.00	325.00	405.00	354.00	382.00
ANKRD45	4.00	0.00	1.00	1.67	5.00
ANKRD46	345.00	292.00	359.00	332.00	352.00
ANKRD49	360.00	292.00	389.00	347.00	313.00
ANKRD50	1081.00	956.00	1197.00	1078.00	841.00
ANKRD52	2945.00	2839.00	3242.00	3008.67	2355.00
ANKRD53	9.00	10.00	6.00	8.33	4.00
ANKRD54	314.00	367.00	375.00	352.00	440.00
ANKRD6	8.00	9.00	9.00	8.67	11.00
ANKRD61	6.00	11.00	9.00	8.67	14.00
ANKRD7	42.00	26.00	41.00	36.33	43.00
ANKRD9	188.00	200.00	204.00	197.33	224.00
ANKS1A	1058.00	984.00	1125.00	1055.67	891.00
ANKS3	518.00	519.00	642.00	559.67	582.00
ANKS6	1305.00	1305.00	1445.00	1351.67	1323.00
ANKZF1	1677.00	1655.00	1718.00	1683.33	1650.00
ANLN	3007.00	2759.00	2946.00	2904.00	3719.00
ANO1	4.00	2.00	6.00	4.00	1.00
ANO10	2098.00	2050.00	2187.00	2111.67	1612.00
ANO5	225.00	252.00	280.00	252.33	257.00
ANO6	3038.00	2786.00	3050.00	2958.00	2364.00
ANO7	32.00	18.00	24.00	24.67	21.00
ANO8	293.00	324.00	339.00	318.67	273.00
ANOS1	302.00	313.00	372.00	329.00	272.00
ANP32A	2816.00	2662.00	2978.00	2818.67	3776.00
ANP32A-IT1	31.00	24.00	27.00	27.33	9.00
ANP32B	3507.00	3454.00	3547.00	3502.67	4894.00
ANP32E	1161.00	915.00	1229.00	1101.67	1446.00
ANPEP	15491.00	15235.00	14621.00	15115.67	15374.00

ANTXR1	2352.00	2152.00	2359.00	2287.67	1508.00
ANTXR2	2432.00	2156.00	2276.00	2288.00	1712.00
ANXA1	4739.00	4548.00	4758.00	4681.67	5921.00
ANXA10	30.00	18.00	44.00	30.67	14.00
ANXA11	1529.00	1547.00	1514.00	1530.00	1694.00
ANXA2	25353.00	24446.00	26360.00	25386.33	31926.00
ANXA2P2	1274.00	1310.00	1385.00	1323.00	1750.00
ANXA2R	61.00	58.00	57.00	58.67	70.00
ANXA3	4.00	3.00	1.00	2.67	0.00
ANXA4	488.00	471.00	577.00	512.00	626.00
ANXA5	10112.00	9664.00	10946.00	10240.67	8855.00
ANXA6	2314.00	2220.00	2449.00	2327.67	2464.00
ANXA7	2755.00	2431.00	2795.00	2660.33	2340.00
ANXA9	5.00	0.00	1.00	2.00	2.00
AOC2	93.00	84.00	120.00	99.00	84.00
AOC3	34.00	22.00	32.00	29.33	32.00
AOX1	6999.00	6353.00	7835.00	7062.33	6765.00
AP1AR	788.00	675.00	940.00	801.00	916.00
AP1B1	2162.00	2100.00	2193.00	2151.67	2290.00
AP1G1	2069.00	1898.00	2148.00	2038.33	2125.00
AP1G2	391.00	359.00	384.00	378.00	292.00
AP1M1	1319.00	1272.00	1263.00	1284.67	1661.00
AP1S1	1483.00	1383.00	1473.00	1446.33	1796.00
AP1S2	1388.00	1203.00	1469.00	1353.33	1243.00
AP1S3	58.00	50.00	62.00	56.67	91.00
AP2A1	1723.00	1742.00	1833.00	1766.00	1857.00
AP2A2	217.00	191.00	221.00	209.67	238.00
AP2B1	8329.00	7437.00	8922.00	8229.33	7518.00
AP2M1	6501.00	6235.00	6809.00	6515.00	6318.00
AP2S1	1193.00	1263.00	1311.00	1255.67	1667.00
AP3B1	1244.00	1128.00	1350.00	1240.67	1367.00
AP3D1	5241.00	5123.00	5322.00	5228.67	5215.00
AP3M1	1993.00	1803.00	2126.00	1974.00	1949.00
AP3M2	1600.00	1413.00	1569.00	1527.33	1672.00
AP3S1	692.00	626.00	761.00	693.00	849.00
AP3S2	47.00	64.00	43.00	51.33	47.00
AP4B1	1254.00	1158.00	1211.00	1207.67	1397.00
AP4E1	527.00	479.00	581.00	529.00	573.00
AP4M1	505.00	443.00	465.00	471.00	795.00
AP4S1	236.00	210.00	223.00	223.00	254.00
AP5B1	1206.00	1139.00	1292.00	1212.33	1111.00
AP5M1	415.00	378.00	436.00	409.67	375.00
AP5S1	295.00	323.00	322.00	313.33	363.00
AP5Z1	1972.00	2017.00	2184.00	2057.67	2199.00
APAF1	421.00	386.00	417.00	408.00	373.00
APBA1	10.00	3.00	9.00	7.33	1.00
APBA2	61.00	85.00	105.00	83.67	84.00
APBA3	739.00	740.00	783.00	754.00	697.00

APBB1	713.00	666.00	727.00	702.00	685.00
APBB1IP	27.00	18.00	30.00	25.00	31.00
APBB2	4274.00	3890.00	4674.00	4279.33	4058.00
APBB3	1185.00	1314.00	1468.00	1322.33	1102.00
APC	916.00	797.00	935.00	882.67	751.00
APC2	8.00	9.00	11.00	9.33	12.00
APCDD1L	4.00	1.00	0.00	1.67	0.00
APEH	1646.00	1566.00	1700.00	1637.33	2036.00
APEX1	4576.00	4426.00	4916.00	4639.33	5569.00
APEX2	674.00	591.00	622.00	629.00	783.00
APH1A	2202.00	2254.00	2173.00	2209.67	2339.00
APH1B	1421.00	1224.00	1217.00	1287.33	1299.00
API5	2300.00	2072.00	2333.00	2235.00	2716.00
APIP	403.00	366.00	457.00	408.67	642.00
APITD1	50.00	39.00	30.00	39.67	57.00
APLF	160.00	166.00	163.00	163.00	168.00
APLN	222.00	194.00	189.00	201.67	494.00
APLP1	16.00	18.00	10.00	14.67	17.00
APLP2	6914.00	6797.00	7067.00	6926.00	6829.00
APMAP	5924.00	5960.00	5832.00	5905.33	5365.00
APOA1BP	876.00	905.00	901.00	894.00	1247.00
APOBEC3B	109.00	140.00	102.00	117.00	225.00
APOBEC3C	151.00	150.00	159.00	153.33	182.00
APOBEC3D	16.00	12.00	23.00	17.00	30.00
APOBEC3F	27.00	20.00	20.00	22.33	32.00
APOBR	5.00	3.00	3.00	3.67	1.00
APOC1	7.00	3.00	5.00	5.00	7.00
APOE	27.00	18.00	16.00	20.33	19.00
APOL1	29.00	30.00	46.00	35.00	19.00
APOL2	210.00	242.00	231.00	227.67	277.00
APOL3	34.00	58.00	46.00	46.00	28.00
APOL6	575.00	493.00	556.00	541.33	562.00
APOLD1	334.00	387.00	375.00	365.33	373.00
APOO	335.00	331.00	344.00	336.67	460.00
APOOL	522.00	477.00	701.00	566.67	610.00
APOPT1	419.00	395.00	499.00	437.67	554.00
APP	33512.00	32910.00	35084.00	33835.33	31640.00
APPBP2	1345.00	1261.00	1525.00	1377.00	1513.00
APPL1	921.00	829.00	1077.00	942.33	1083.00
APPL2	1001.00	963.00	1089.00	1017.67	1090.00
APRT	2389.00	2271.00	2374.00	2344.67	3421.00
APTR	178.00	196.00	196.00	190.00	229.00
APTX	1029.00	1118.00	1155.00	1100.67	1340.00
AQP11	54.00	71.00	68.00	64.33	69.00
AQP3	5.00	7.00	8.00	6.67	8.00
AQP7P1	95.00	97.00	120.00	104.00	116.00
AQP7P3	104.00	96.00	117.00	105.67	110.00
AQR	1292.00	1244.00	1419.00	1318.33	1330.00

AR	593.00	516.00	696.00	601.67	278.00
ARAF	1022.00	1049.00	1047.00	1039.33	1148.00
ARAP1	2760.00	2684.00	2882.00	2775.33	2673.00
ARAP2	123.00	103.00	124.00	116.67	95.00
ARAP3	1687.00	1699.00	1903.00	1763.00	1651.00
ARC	22.00	13.00	15.00	16.67	10.00
ARCN1	4114.00	3780.00	4427.00	4107.00	3838.00
AREG	73.00	73.00	66.00	70.67	100.00
AREL1	1499.00	1463.00	1518.00	1493.33	1526.00
ARF1	5987.00	5702.00	6483.00	6057.33	7270.00
ARF3	2951.00	2768.00	3024.00	2914.33	2937.00
ARF4	4031.00	3626.00	4166.00	3941.00	4618.00
ARF5	935.00	1028.00	1020.00	994.33	1295.00
ARF6	1894.00	1924.00	2156.00	1991.33	2328.00
ARFGAP1	3767.00	3845.00	4165.00	3925.67	3607.00
ARFGAP2	1382.00	1323.00	1416.00	1373.67	1636.00
ARFGAP3	925.00	820.00	916.00	887.00	842.00
ARFGEF1	2204.00	2012.00	2125.00	2113.67	2018.00
ARFGEF2	2739.00	2348.00	2814.00	2633.67	2460.00
ARFGEF3	418.00	392.00	451.00	420.33	364.00
ARFIP1	399.00	347.00	429.00	391.67	375.00
ARFIP2	1417.00	1292.00	1453.00	1387.33	1472.00
ARFRP1	1116.00	1081.00	1118.00	1105.00	1251.00
ARG2	326.00	278.00	332.00	312.00	460.00
ARGLU1	4100.00	4232.00	4476.00	4269.33	4284.00
ARHGAP1	1147.00	1183.00	1170.00	1166.67	1389.00
ARHGAP10	235.00	247.00	262.00	248.00	261.00
ARHGAP11A	24.00	20.00	22.00	22.00	22.00
ARHGAP11B	6.00	2.00	1.00	3.00	6.00
ARHGAP12	1799.00	1707.00	2012.00	1839.33	1723.00
ARHGAP17	1137.00	1076.00	1167.00	1126.67	959.00
ARHGAP18	3098.00	2899.00	3313.00	3103.33	3152.00
ARHGAP19	358.00	367.00	403.00	376.00	556.00
ARHGAP20	32.00	27.00	35.00	31.33	12.00
ARHGAP21	2041.00	1938.00	2238.00	2072.33	1811.00
ARHGAP22	2156.00	2200.00	2326.00	2227.33	2179.00
ARHGAP23	169.00	162.00	200.00	177.00	137.00
ARHGAP24	1032.00	1097.00	1190.00	1106.33	1478.00
ARHGAP25	7.00	8.00	8.00	7.67	11.00
ARHGAP26	268.00	238.00	273.00	259.67	133.00
ARHGAP27	24.00	20.00	12.00	18.67	18.00
ARHGAP29	12036.00	10828.00	12321.00	11728.33	11730.00
ARHGAP30	249.00	283.00	256.00	262.67	216.00
ARHGAP31	558.00	536.00	536.00	543.33	573.00
ARHGAP31-AS1	10.00	6.00	10.00	8.67	4.00
ARHGAP32	725.00	732.00	850.00	769.00	671.00
ARHGAP33	208.00	259.00	190.00	219.00	254.00
ARHGAP35	2641.00	2386.00	2806.00	2611.00	1908.00

ARHGAP39	749.00	730.00	782.00	753.67	660.00
ARHGAP4	18.00	21.00	17.00	18.67	19.00
ARHGAP40	1826.00	1747.00	1806.00	1793.00	2065.00
ARHGAP42	898.00	711.00	965.00	858.00	770.00
ARHGAP5	1322.00	1131.00	1467.00	1306.67	1116.00
ARHGAP5-AS1	30.00	50.00	46.00	42.00	61.00
ARHGAP6	177.00	182.00	183.00	180.67	165.00
ARHGAP9	35.00	29.00	17.00	27.00	20.00
ARHGDIA	8733.00	8933.00	9363.00	9009.67	7668.00
ARHGDIB	2306.00	2228.00	2382.00	2305.33	3110.00
ARHGEF1	2994.00	2979.00	2965.00	2979.33	2966.00
ARHGEF10L	120.00	114.00	146.00	126.67	111.00
ARHGEF11	2015.00	1973.00	2116.00	2034.67	1906.00
ARHGEF12	2087.00	1946.00	2350.00	2127.67	1870.00
ARHGEF17	1905.00	1884.00	2046.00	1945.00	1108.00
ARHGEF18	1299.00	1234.00	1441.00	1324.67	1015.00
ARHGEF19	296.00	301.00	333.00	310.00	278.00
ARHGEF2	9306.00	8858.00	9361.00	9175.00	7586.00
ARHGEF25	999.00	960.00	1059.00	1006.00	1201.00
ARHGEF26	18.00	21.00	27.00	22.00	14.00
ARHGEF26-AS1	23.00	20.00	17.00	20.00	17.00
ARHGEF28	3012.00	2964.00	3315.00	3097.00	2680.00
ARHGEF3	121.00	84.00	109.00	104.67	113.00
ARHGEF37	5.00	5.00	6.00	5.33	7.00
ARHGEF39	218.00	203.00	210.00	210.33	257.00
ARHGEF40	557.00	614.00	651.00	607.33	525.00
ARHGEF6	331.00	301.00	344.00	325.33	244.00
ARHGEF7	1032.00	924.00	1045.00	1000.33	1037.00
ARHGEF9	1144.00	1115.00	1256.00	1171.67	1089.00
ARID1A	2987.00	2847.00	3013.00	2949.00	1150.00
ARID1B	1971.00	1815.00	2131.00	1972.33	1810.00
ARID2	629.00	602.00	734.00	655.00	669.00
ARID3A	102.00	83.00	93.00	92.67	119.00
ARID3B	474.00	466.00	576.00	505.33	380.00
ARID4A	412.00	326.00	403.00	380.33	366.00
ARID4B	786.00	756.00	819.00	787.00	751.00
ARID5A	130.00	129.00	119.00	126.00	73.00
ARID5B	714.00	670.00	742.00	708.67	394.00
ARIH1	1382.00	1376.00	1501.00	1419.67	1408.00
ARIH2	2685.00	2711.00	2816.00	2737.33	3283.00
ARIH2OS	5.00	17.00	11.00	11.00	14.00
ARL1	1254.00	1088.00	1333.00	1225.00	1484.00
ARL10	98.00	94.00	122.00	104.67	41.00
ARL13B	378.00	317.00	338.00	344.33	395.00
ARL14EP	633.00	518.00	566.00	572.33	727.00
ARL14EPL	7.00	1.00	4.00	4.00	1.00
ARL15	210.00	140.00	189.00	179.67	203.00
ARL16	658.00	615.00	570.00	614.33	642.00

ARL17A	38.00	44.00	38.00	40.00	29.00
ARL2	1625.00	1603.00	1772.00	1666.67	2190.00
ARL2BP	1009.00	930.00	1015.00	984.67	1095.00
ARL3	364.00	382.00	422.00	389.33	467.00
ARL4A	386.00	374.00	449.00	403.00	488.00
ARL4C	708.00	672.00	784.00	721.33	658.00
ARL4D	132.00	120.00	115.00	122.33	107.00
ARL5A	553.00	451.00	591.00	531.67	672.00
ARL5B	294.00	240.00	326.00	286.67	276.00
ARL6	174.00	170.00	189.00	177.67	186.00
ARL6IP1	3346.00	3092.00	3103.00	3180.33	3655.00
ARL6IP4	1874.00	1896.00	1932.00	1900.67	2347.00
ARL6IP5	1391.00	1305.00	1335.00	1343.67	1668.00
ARL6IP6	527.00	538.00	558.00	541.00	570.00
ARL8A	395.00	408.00	423.00	408.67	432.00
ARL8B	1492.00	1489.00	1644.00	1541.67	1911.00
ARL9	7.00	4.00	8.00	6.33	7.00
ARMC1	827.00	804.00	908.00	846.33	994.00
ARMC10	234.00	167.00	206.00	202.33	219.00
ARMC12	22.00	21.00	15.00	19.33	22.00
ARMC2	59.00	46.00	39.00	48.00	51.00
ARMC4	626.00	560.00	685.00	623.67	1265.00
ARMC5	386.00	385.00	442.00	404.33	396.00
ARMC6	1179.00	1233.00	1299.00	1237.00	1494.00
ARMC7	169.00	185.00	185.00	179.67	237.00
ARMC8	850.00	816.00	885.00	850.33	881.00
ARMC9	722.00	723.00	803.00	749.33	665.00
ARMCX1	1019.00	1089.00	1000.00	1036.00	1094.00
ARMCX2	35.00	39.00	49.00	41.00	66.00
ARMCX3	810.00	810.00	834.00	818.00	786.00
ARMCX4	943.00	927.00	1020.00	963.33	849.00
ARMCX5	496.00	467.00	440.00	467.67	469.00
ARMCX5-GPRASP2	45.00	38.00	52.00	45.00	32.00
ARMCX6	247.00	310.00	269.00	275.33	261.00
ARMT1	493.00	480.00	533.00	502.00	658.00
ARNT	660.00	621.00	723.00	668.00	615.00
ARNT2	175.00	165.00	172.00	170.67	185.00
ARNTL	406.00	348.00	368.00	374.00	334.00
ARNTL2	2260.00	2174.00	2485.00	2306.33	4664.00
ARPC1A	2815.00	2708.00	3037.00	2853.33	4355.00
ARPC1B	3166.00	3190.00	3440.00	3265.33	4387.00
ARPC2	4158.00	4168.00	4446.00	4257.33	5416.00
ARPC3	2497.00	2662.00	2738.00	2632.33	3596.00
ARPC4	1438.00	1364.00	1558.00	1453.33	1881.00
ARPC5	2192.00	2007.00	2160.00	2119.67	3288.00
ARPC5L	761.00	792.00	788.00	780.33	1056.00
ARPIN	450.00	439.00	435.00	441.33	459.00
ARPP19	2097.00	1966.00	2423.00	2162.00	2343.00

ARPP21	6.00	13.00	12.00	10.33	20.00
ARRB1	80.00	50.00	51.00	60.33	73.00
ARRB2	406.00	421.00	440.00	422.33	637.00
ARRDC1	560.00	496.00	548.00	534.67	615.00
ARRDC1-AS1	544.00	595.00	549.00	562.67	597.00
ARRDC2	387.00	393.00	381.00	387.00	441.00
ARRDC3	671.00	627.00	809.00	702.33	644.00
ARRDC3-AS1	88.00	70.00	63.00	73.67	84.00
ARRDC4	37.00	43.00	43.00	41.00	42.00
ARSA	687.00	782.00	809.00	759.33	623.00
ARSB	464.00	482.00	488.00	478.00	449.00
ARSD	230.00	277.00	238.00	248.33	196.00
ARSE	28.00	31.00	39.00	32.67	39.00
ARSG	178.00	178.00	209.00	188.33	134.00
ARSJ	2033.00	1857.00	2177.00	2022.33	1487.00
ARSK	466.00	449.00	481.00	465.33	521.00
ARTN	58.00	82.00	92.00	77.33	87.00
ARV1	514.00	459.00	480.00	484.33	608.00
ARVCF	63.00	81.00	78.00	74.00	75.00
ASAH1	1634.00	1638.00	1671.00	1647.67	834.00
ASAH2	5.00	7.00	12.00	8.00	8.00
ASAH2B	28.00	20.00	28.00	25.33	30.00
ASAP1	8593.00	8045.00	9418.00	8685.33	6835.00
ASAP1-IT2	8.00	9.00	14.00	10.33	17.00
ASAP2	2632.00	2433.00	2803.00	2622.67	3160.00
ASAP3	55.00	80.00	82.00	72.33	31.00
ASB1	17369.00	16991.00	18783.00	17714.33	15037.00
ASB13	576.00	634.00	648.00	619.33	719.00
ASB16-AS1	190.00	193.00	202.00	195.00	181.00
ASB3	43.00	29.00	35.00	35.67	39.00
ASB6	923.00	931.00	1015.00	956.33	1029.00
ASB7	469.00	459.00	514.00	480.67	480.00
ASB8	335.00	302.00	340.00	325.67	362.00
ASB9	60.00	46.00	42.00	49.33	149.00
ASCC1	622.00	472.00	617.00	570.33	647.00
ASCC2	1287.00	1245.00	1405.00	1312.33	1395.00
ASCC3	1883.00	1697.00	2126.00	1902.00	1832.00
ASF1A	449.00	468.00	465.00	460.67	582.00
ASF1B	437.00	410.00	346.00	397.67	461.00
ASGR1	78.00	83.00	77.00	79.33	66.00
ASH1L	2028.00	1903.00	2127.00	2019.33	1600.00
ASH1L-AS1	61.00	59.00	64.00	61.33	46.00
ASH2L	814.00	792.00	850.00	818.67	1038.00
ASIC1	1008.00	1001.00	1073.00	1027.33	1015.00
ASIC2	7.00	2.00	1.00	3.33	2.00
ASIC3	93.00	100.00	139.00	110.67	69.00
ASL	804.00	896.00	857.00	852.33	859.00
ASMTL	5.00	2.00	0.00	2.33	1.00

ASNA1	1127.00	1158.00	1169.00	1151.33	1421.00
ASNS	10340.00	9684.00	10549.00	10191.00	12124.00
ASNSD1	1076.00	1004.00	1149.00	1076.33	1359.00
ASPH	10807.00	10239.00	11211.00	10752.33	10800.00
ASPHD1	199.00	160.00	184.00	181.00	243.00
ASPHD2	64.00	64.00	63.00	63.67	65.00
ASPM	2553.00	2360.00	2459.00	2457.33	2348.00
ASPRV1	15.00	12.00	5.00	10.67	7.00
ASPSCR1	1069.00	1147.00	1121.00	1112.33	1256.00
ASRGL1	18.00	17.00	13.00	16.00	29.00
ASS1	8.00	7.00	5.00	6.67	20.00
ASTE1	164.00	118.00	183.00	155.00	140.00
ASTN2	84.00	60.00	84.00	76.00	103.00
ASUN	1708.00	1582.00	2014.00	1768.00	2277.00
ASXL1	4310.00	4269.00	4542.00	4373.67	4062.00
ASXL2	809.00	631.00	798.00	746.00	520.00
ASXL3	12.00	8.00	4.00	8.00	3.00
ATAD1	2170.00	2051.00	2295.00	2172.00	2465.00
ATAD2	1285.00	1225.00	1261.00	1257.00	1876.00
ATAD2B	343.00	358.00	366.00	355.67	356.00
ATAD3A	890.00	851.00	870.00	870.33	1082.00
ATAD3B	812.00	822.00	819.00	817.67	1011.00
ATAD5	397.00	374.00	425.00	398.67	432.00
ATE1	1050.00	767.00	973.00	930.00	998.00
ATE1-AS1	43.00	33.00	36.00	37.33	32.00
ATF1	499.00	423.00	541.00	487.67	585.00
ATF2	934.00	916.00	999.00	949.67	956.00
ATF3	153.00	157.00	167.00	159.00	177.00
ATF4	9670.00	9296.00	10399.00	9788.33	12126.00
ATF5	213.00	196.00	253.00	220.67	258.00
ATF6	3179.00	2934.00	3215.00	3109.33	2871.00
ATF6B	94.00	111.00	102.00	102.33	134.00
ATF7	1134.00	1098.00	1203.00	1145.00	881.00
ATF7IP	1787.00	1639.00	1784.00	1736.67	1671.00
ATF7IP2	5.00	12.00	23.00	13.33	19.00
ATG10	90.00	94.00	120.00	101.33	134.00
ATG101	910.00	880.00	949.00	913.00	1103.00
ATG12	1097.00	1090.00	1191.00	1126.00	1313.00
ATG13	1457.00	1333.00	1477.00	1422.33	1213.00
ATG14	610.00	574.00	716.00	633.33	638.00
ATG16L1	724.00	741.00	798.00	754.33	757.00
ATG16L2	1120.00	1245.00	1255.00	1206.67	1478.00
ATG2A	1925.00	1883.00	1956.00	1921.33	1596.00
ATG2B	1376.00	1174.00	1432.00	1327.33	1208.00
ATG3	1138.00	1226.00	1293.00	1219.00	1537.00
ATG4A	274.00	308.00	293.00	291.67	237.00
ATG4B	1218.00	1235.00	1239.00	1230.67	1236.00
ATG4C	423.00	339.00	403.00	388.33	454.00

ATG4D	325.00	298.00	361.00	328.00	414.00
ATG5	687.00	640.00	721.00	682.67	561.00
ATG7	1031.00	980.00	1080.00	1030.33	1240.00
ATG9A	2276.00	2144.00	2209.00	2209.67	2096.00
ATG9B	11.00	13.00	9.00	11.00	9.00
ATHL1	1734.00	1862.00	2034.00	1876.67	1874.00
ATIC	4341.00	4143.00	4696.00	4393.33	5206.00
ATL1	5.00	5.00	0.00	3.33	3.00
ATL2	1790.00	1670.00	1840.00	1766.67	2005.00
ATL3	2983.00	2911.00	3197.00	3030.33	3315.00
ATM	2832.00	2594.00	3159.00	2861.67	2422.00
ATMIN	1430.00	1317.00	1556.00	1434.33	1383.00
ATN1	3626.00	3483.00	4064.00	3724.33	3172.00
ATOH8	101.00	97.00	107.00	101.67	49.00
ATOX1	865.00	831.00	903.00	866.33	1258.00
ATP10A	43.00	36.00	49.00	42.67	42.00
ATP10D	809.00	817.00	864.00	830.00	617.00
ATP11A	1250.00	1179.00	1261.00	1230.00	943.00
ATP11B	1755.00	1544.00	1686.00	1661.67	1884.00
ATP11C	995.00	847.00	1078.00	973.33	898.00
ATP13A1	3042.00	3128.00	3197.00	3122.33	2983.00
ATP13A2	1065.00	1015.00	1020.00	1033.33	848.00
ATP13A3	5022.00	4452.00	5290.00	4921.33	4622.00
ATP13A4	5.00	6.00	8.00	6.33	10.00
ATP1A1	19183.00	19613.00	19898.00	19564.67	17522.00
ATP1A1-AS1	36.00	40.00	46.00	40.67	46.00
ATP1A2	196.00	168.00	217.00	193.67	51.00
ATP1B1	1353.00	1399.00	1437.00	1396.33	1263.00
ATP1B3	5319.00	5133.00	5061.00	5171.00	5919.00
ATP2A1	49.00	51.00	72.00	57.33	55.00
ATP2A1-AS1	71.00	80.00	92.00	81.00	114.00
ATP2A2	11257.00	10661.00	11292.00	11070.00	10307.00
ATP2B1	2895.00	2695.00	3083.00	2891.00	2102.00
ATP2B2	1771.00	1690.00	1864.00	1775.00	1499.00
ATP2B4	1393.00	1413.00	1429.00	1411.67	985.00
ATP2C1	4138.00	3992.00	4120.00	4083.33	4418.00
ATP5A1	5392.00	5112.00	5899.00	5467.67	6405.00
ATP5B	13786.00	13186.00	14701.00	13891.00	17368.00
ATP5C1	2371.00	2363.00	2555.00	2429.67	2838.00
ATP5D	1130.00	1129.00	1178.00	1145.67	1496.00
ATP5E	857.00	1063.00	1023.00	981.00	1550.00
ATP5EP2	12.00	6.00	9.00	9.00	19.00
ATP5F1	2691.00	2614.00	2906.00	2737.00	3783.00
ATP5G1	1169.00	1201.00	1201.00	1190.33	1545.00
ATP5G2	3101.00	3019.00	3277.00	3132.33	3913.00
ATP5G3	2167.00	2212.00	2253.00	2210.67	3224.00
ATP5H	1370.00	1359.00	1318.00	1349.00	1824.00
ATP5I	556.00	552.00	523.00	543.67	765.00

ATP5J	1092.00	1120.00	1136.00	1116.00	1551.00
ATP5J2	691.00	757.00	788.00	745.33	1021.00
ATP5L	1626.00	1582.00	1608.00	1605.33	2240.00
ATP5O	1779.00	1794.00	1982.00	1851.67	2484.00
ATP5S	173.00	145.00	155.00	157.67	191.00
ATP5SL	467.00	468.00	495.00	476.67	546.00
ATP6AP1	2374.00	2556.00	2326.00	2418.67	2506.00
ATP6AP1L	203.00	253.00	281.00	245.67	251.00
ATP6AP2	2664.00	2702.00	2571.00	2645.67	2528.00
ATP6V0A1	2459.00	2489.00	2651.00	2533.00	2178.00
ATP6V0A2	1334.00	1286.00	1410.00	1343.33	1207.00
ATP6V0B	2408.00	2344.00	2208.00	2320.00	2599.00
ATP6V0C	1558.00	1472.00	1371.00	1467.00	1717.00
ATP6V0D1	1041.00	984.00	1019.00	1014.67	1250.00
ATP6V0E1	1747.00	1653.00	1603.00	1667.67	1880.00
ATP6V0E2	789.00	752.00	712.00	751.00	849.00
ATP6V0E2-AS1	205.00	207.00	228.00	213.33	191.00
ATP6V1A	976.00	877.00	1028.00	960.33	1065.00
ATP6V1B1	118.00	108.00	110.00	112.00	83.00
ATP6V1B1-AS1	90.00	88.00	84.00	87.33	98.00
ATP6V1B2	1159.00	1062.00	1213.00	1144.67	1186.00
ATP6V1C1	2297.00	2174.00	2490.00	2320.33	2763.00
ATP6V1C2	5.00	0.00	1.00	2.00	5.00
ATP6V1D	694.00	682.00	775.00	717.00	1001.00
ATP6V1E1	1430.00	1331.00	1474.00	1411.67	1676.00
ATP6V1E2	74.00	73.00	80.00	75.67	109.00
ATP6V1F	1332.00	1247.00	1328.00	1302.33	1820.00
ATP6V1G1	916.00	928.00	959.00	934.33	1321.00
ATP6V1H	1296.00	1261.00	1215.00	1257.33	1445.00
ATP7A	435.00	388.00	464.00	429.00	348.00
ATP7B	574.00	555.00	527.00	552.00	439.00
ATP8A1	35.00	37.00	34.00	35.33	40.00
ATP8A2	37.00	33.00	38.00	36.00	31.00
ATP8B1	82.00	77.00	113.00	90.67	41.00
ATP8B2	2119.00	2014.00	2003.00	2045.33	1743.00
ATP8B3	72.00	63.00	58.00	64.33	93.00
ATP8B5P	10.00	14.00	15.00	13.00	8.00
ATP9A	698.00	673.00	721.00	697.33	664.00
ATP9B	286.00	290.00	276.00	284.00	274.00
ATPAF1	670.00	632.00	633.00	645.00	853.00
ATPAF2	200.00	207.00	195.00	200.67	258.00
ATPIF1	982.00	947.00	985.00	971.33	1233.00
ATR	1940.00	1955.00	2200.00	2031.67	1978.00
ATRAID	2782.00	2829.00	2769.00	2793.33	3145.00
ATRIP	381.00	374.00	387.00	380.67	368.00
ATRN	2893.00	2622.00	3179.00	2898.00	2476.00
ATRNL1	43.00	35.00	37.00	38.33	36.00
ATRX	2404.00	2130.00	2744.00	2426.00	1961.00

ATXN1	790.00	702.00	868.00	786.67	516.00
ATXN10	1572.00	1460.00	1650.00	1560.67	1837.00
ATXN1L	1004.00	961.00	1036.00	1000.33	830.00
ATXN2	1472.00	1262.00	1456.00	1396.67	1353.00
ATXN2L	8450.00	8335.00	8636.00	8473.67	7851.00
ATXN3	334.00	341.00	392.00	355.67	381.00
ATXN7	717.00	722.00	795.00	744.67	734.00
ATXN7L1	533.00	460.00	585.00	526.00	467.00
ATXN7L2	404.00	408.00	451.00	421.00	414.00
ATXN7L3	1501.00	1497.00	1499.00	1499.00	1414.00
ATXN7L3B	2502.00	2395.00	2580.00	2492.33	2830.00
AUH	115.00	124.00	130.00	123.00	119.00
AUNIP	120.00	120.00	126.00	122.00	143.00
AUP1	4936.00	5142.00	5208.00	5095.33	5806.00
AURKA	1519.00	1411.00	1512.00	1480.67	2231.00
AURKAIP1	1159.00	1217.00	1250.00	1208.67	1583.00
AURKAPS1	35.00	44.00	30.00	36.33	28.00
AURKB	1170.00	1186.00	1122.00	1159.33	1737.00
AURKC	7.00	19.00	26.00	17.33	18.00
AUTS2	43.00	26.00	36.00	35.00	39.00
AVEN	388.00	362.00	388.00	379.33	400.00
AVIL	77.00	105.00	120.00	100.67	86.00
AVL9	2825.00	2468.00	2891.00	2728.00	2792.00
AVPI1	105.00	116.00	113.00	111.33	111.00
AWAT2	22.00	29.00	37.00	29.33	5.00
AXDND1	6.00	1.00	5.00	4.00	5.00
AXIN1	1048.00	1040.00	1061.00	1049.67	1159.00
AXIN2	19.00	11.00	12.00	14.00	22.00
AXL	27362.00	26150.00	27807.00	27106.33	17066.00
AZGP1	4.00	3.00	6.00	4.33	3.00
AZI2	876.00	797.00	979.00	884.00	1091.00
AZIN1	4309.00	3725.00	4522.00	4185.33	4984.00
AZIN1-AS1	20.00	16.00	16.00	17.33	29.00
AZIN2	83.00	95.00	123.00	100.33	101.00
B2M	1269.00	1269.00	1151.00	1229.67	1668.00
B3GALNT1	214.00	251.00	224.00	229.67	267.00
B3GALNT2	1163.00	1129.00	1195.00	1162.33	1428.00
B3GALT1	58.00	44.00	49.00	50.33	51.00
B3GALT5	68.00	60.00	68.00	65.33	43.00
B3GALT5-AS1	23.00	19.00	16.00	19.33	22.00
B3GALT6	1052.00	1026.00	1097.00	1058.33	1136.00
B3GAT2	6.00	4.00	1.00	3.67	5.00
B3GAT3	1593.00	1641.00	1574.00	1602.67	1733.00
B3GLCT	333.00	354.00	316.00	334.33	360.00
B3GNT2	501.00	487.00	495.00	494.33	463.00
B3GNT5	464.00	415.00	464.00	447.67	504.00
B3GNT8	4.00	1.00	0.00	1.67	1.00
B3GNT9	64.00	63.00	65.00	64.00	45.00

B3GNTL1	140.00	140.00	161.00	147.00	215.00
B4GALNT1	51.00	48.00	40.00	46.33	51.00
B4GALNT2	16.00	15.00	13.00	14.67	8.00
B4GALT1	1910.00	1776.00	1759.00	1815.00	1758.00
B4GALT1-AS1	5.00	8.00	13.00	8.67	10.00
B4GALT2	1308.00	1295.00	1303.00	1302.00	1527.00
B4GALT3	1541.00	1559.00	1615.00	1571.67	1533.00
B4GALT4	436.00	415.00	419.00	423.33	469.00
B4GALT5	2024.00	1898.00	1932.00	1951.33	2016.00
B4GALT6	577.00	492.00	649.00	572.67	707.00
B4GALT7	917.00	871.00	895.00	894.33	872.00
B4GAT1	2203.00	2248.00	2226.00	2225.67	1954.00
B9D1	319.00	318.00	310.00	315.67	393.00
B9D2	49.00	52.00	43.00	48.00	76.00
BAALC-AS1	5.00	8.00	3.00	5.33	3.00
BAAT	4.00	3.00	1.00	2.67	2.00
BABAM1	1491.00	1465.00	1562.00	1506.00	1753.00
BACE1	1458.00	1489.00	1490.00	1479.00	1127.00
BACE1-AS	95.00	93.00	116.00	101.33	99.00
BACE2	3023.00	3068.00	3004.00	3031.67	3592.00
BACH1	2225.00	1932.00	2504.00	2220.33	1990.00
BACH1-IT2	5.00	5.00	10.00	6.67	3.00
BACH2	118.00	122.00	102.00	114.00	95.00
BAD	565.00	593.00	624.00	594.00	748.00
BAG1	397.00	382.00	435.00	404.67	471.00
BAG2	662.00	586.00	681.00	643.00	865.00
BAG3	1316.00	1307.00	1349.00	1324.00	1239.00
BAG4	356.00	322.00	352.00	343.33	385.00
BAG5	982.00	907.00	1061.00	983.33	1042.00
BAG6	7.00	2.00	6.00	5.00	4.00
BAHCC1	165.00	166.00	216.00	182.33	165.00
BAHD1	861.00	869.00	833.00	854.33	953.00
BAIAP2	765.00	672.00	739.00	725.33	766.00
BAIAP2-AS1	245.00	269.00	310.00	274.67	267.00
BAIAP2L1	113.00	110.00	150.00	124.33	150.00
BAIAP2L2	107.00	131.00	154.00	130.67	217.00
BAIAP3	5.00	5.00	13.00	7.67	1.00
BAK1	394.00	413.00	428.00	411.67	504.00
BAMBI	84.00	87.00	82.00	84.33	77.00
BANF1	2600.00	2753.00	2732.00	2695.00	3585.00
BANP	540.00	557.00	622.00	573.00	625.00
BAP1	1781.00	1729.00	1804.00	1771.33	1724.00
BARD1	218.00	224.00	214.00	218.67	262.00
BARHL2	18.00	18.00	21.00	19.00	26.00
BASP1	4932.00	4834.00	5454.00	5073.33	5475.00
BATF	43.00	31.00	29.00	34.33	17.00
BATF2	77.00	51.00	68.00	65.33	85.00
BATF3	16.00	21.00	27.00	21.33	22.00

BAX	1112.00	1147.00	1137.00	1132.00	1356.00
BAZ1A	1970.00	1819.00	2022.00	1937.00	2022.00
BAZ1B	3399.00	3169.00	3794.00	3454.00	3336.00
BAZ2A	2704.00	2783.00	3110.00	2865.67	2224.00
BAZ2B	688.00	618.00	719.00	675.00	514.00
BBC3	129.00	140.00	141.00	136.67	127.00
BBIP1	401.00	344.00	398.00	381.00	503.00
BBS1	977.00	926.00	968.00	957.00	902.00
BBS10	917.00	750.00	898.00	855.00	815.00
BBS12	97.00	75.00	112.00	94.67	89.00
BBS2	1381.00	1387.00	1533.00	1433.67	1285.00
BBS4	324.00	312.00	339.00	325.00	375.00
BBS5	348.00	332.00	344.00	341.33	383.00
BBS7	487.00	478.00	540.00	501.67	464.00
BBS9	274.00	266.00	297.00	279.00	286.00
BBX	2476.00	2130.00	2529.00	2378.33	2213.00
BCAM	1378.00	1517.00	1657.00	1517.33	1379.00
BCAP29	1758.00	1546.00	1575.00	1626.33	1800.00
BCAP31	7119.00	7060.00	6762.00	6980.33	7225.00
BCAR1	3621.00	3742.00	3904.00	3755.67	5150.00
BCAR3	3986.00	3803.00	4199.00	3996.00	4253.00
BCAS1	6.00	7.00	9.00	7.33	7.00
BCAS2	656.00	635.00	653.00	648.00	857.00
BCAS3	427.00	364.00	461.00	417.33	393.00
BCAS4	280.00	352.00	294.00	308.67	326.00
BCAT1	6674.00	5879.00	7008.00	6520.33	6781.00
BCAT2	681.00	711.00	737.00	709.67	850.00
BCCIP	2180.00	2201.00	2295.00	2225.33	2599.00
BCDIN3D	174.00	208.00	167.00	183.00	206.00
BCDIN3D-AS1	31.00	22.00	23.00	25.33	19.00
BCHE	2000.00	1795.00	1956.00	1917.00	2251.00
BCKDHA	291.00	304.00	346.00	313.67	345.00
BCKDHB	358.00	380.00	376.00	371.33	395.00
BCKDK	766.00	773.00	781.00	773.33	803.00
BCL10	658.00	594.00	694.00	648.67	763.00
BCL11A	6.00	11.00	5.00	7.33	8.00
BCL2	278.00	281.00	368.00	309.00	453.00
BCL2A1	959.00	860.00	860.00	893.00	1863.00
BCL2L1	1452.00	1365.00	1472.00	1429.67	1430.00
BCL2L11	121.00	132.00	143.00	132.00	175.00
BCL2L12	502.00	475.00	513.00	496.67	731.00
BCL2L13	1376.00	1288.00	1317.00	1327.00	1488.00
BCL2L15	11.00	11.00	9.00	10.33	11.00
BCL2L2	1422.00	1417.00	1416.00	1418.33	1515.00
BCL2L2-PABPN1	7.00	1.00	1.00	3.00	7.00
BCL3	141.00	119.00	163.00	141.00	152.00
BCL6	1084.00	1037.00	1250.00	1123.67	1165.00
BCL7A	842.00	794.00	871.00	835.67	1369.00

BCL7B	801.00	713.00	765.00	759.67	858.00
BCL7C	539.00	524.00	528.00	530.33	734.00
BCL9	536.00	472.00	575.00	527.67	520.00
BCL9L	2539.00	2507.00	2549.00	2531.67	1765.00
BCLAF1	3759.00	3422.00	3917.00	3699.33	4078.00
BCO2	28.00	36.00	30.00	31.33	14.00
BCOR	519.00	543.00	611.00	557.67	446.00
BCORL1	316.00	239.00	317.00	290.67	224.00
BCR	775.00	760.00	751.00	762.00	772.00
BCRP2	8.00	5.00	4.00	5.67	4.00
BCS1L	1306.00	1414.00	1464.00	1394.67	1611.00
BCYRN1	12.00	28.00	15.00	18.33	72.00
BDH1	29.00	16.00	22.00	22.33	14.00
BDH2	229.00	224.00	239.00	230.67	260.00
BDKRB1	12.00	13.00	18.00	14.33	12.00
BDKRB2	4.00	12.00	7.00	7.67	3.00
BDNF	1180.00	1159.00	1280.00	1206.33	1874.00
BDNF-AS	40.00	33.00	42.00	38.33	25.00
BEAN1	24.00	20.00	18.00	20.67	22.00
BECN1	1136.00	1242.00	1329.00	1235.67	1485.00
BEND3	308.00	306.00	324.00	312.67	375.00
BEND4	786.00	683.00	813.00	760.67	679.00
BEND6	75.00	74.00	111.00	86.67	95.00
BEND7	304.00	303.00	279.00	295.33	223.00
BEST1	215.00	170.00	182.00	189.00	154.00
BEST3	71.00	57.00	97.00	75.00	119.00
BEST4	10.00	7.00	9.00	8.67	8.00
BET1	668.00	623.00	705.00	665.33	790.00
BET1L	1442.00	1366.00	1461.00	1423.00	1388.00
BEX1	43.00	59.00	47.00	49.67	44.00
BEX2	80.00	69.00	86.00	78.33	116.00
BEX4	619.00	655.00	678.00	650.67	707.00
BEX5	7.00	5.00	10.00	7.33	3.00
BFSP1	30.00	45.00	38.00	37.67	35.00
BGLAP	10.00	9.00	11.00	10.00	3.00
BGN	2429.00	2460.00	2409.00	2432.67	1703.00
BHLHA15	8.00	7.00	7.00	7.33	7.00
BHLHB9	76.00	73.00	92.00	80.33	66.00
BHLHE40	384.00	397.00	401.00	394.00	438.00
BHLHE40-AS1	9.00	7.00	4.00	6.67	8.00
BHLHE41	595.00	573.00	651.00	606.33	543.00
BICC1	177.00	150.00	195.00	174.00	274.00
BICD1	368.00	388.00	417.00	391.00	375.00
BICD2	1127.00	989.00	1149.00	1088.33	1034.00
BID	403.00	408.00	465.00	425.33	578.00
BIN1	240.00	240.00	276.00	252.00	244.00
BIN3	365.00	342.00	367.00	358.00	382.00
BIN3-IT1	4.00	3.00	3.00	3.33	1.00

BIRC2	8106.00	7843.00	8300.00	8083.00	10662.00
BIRC3	639.00	568.00	686.00	631.00	2234.00
BIRC5	770.00	775.00	752.00	765.67	1153.00
BIRC6	2368.00	2130.00	2464.00	2320.67	2056.00
BISPR	13.00	5.00	18.00	12.00	17.00
BIVM	888.00	889.00	916.00	897.67	1175.00
BLCAP	1307.00	1270.00	1206.00	1261.00	1453.00
BLID	4.00	10.00	10.00	8.00	7.00
BLK	1752.00	1869.00	2002.00	1874.33	2129.00
BLM	373.00	372.00	356.00	367.00	465.00
BLMH	656.00	611.00	653.00	640.00	668.00
BLOC1S1	218.00	210.00	223.00	217.00	231.00
BLOC1S2	554.00	560.00	569.00	561.00	730.00
BLOC1S3	295.00	240.00	299.00	278.00	355.00
BLOC1S4	196.00	201.00	252.00	216.33	216.00
BLOC1S6	1197.00	1100.00	1250.00	1182.33	1443.00
BLVRA	355.00	367.00	403.00	375.00	532.00
BLVRB	322.00	368.00	380.00	356.67	388.00
BLZF1	539.00	495.00	559.00	531.00	582.00
BMF	46.00	60.00	64.00	56.67	31.00
BMI1	77.00	59.00	88.00	74.67	80.00
BMP1	675.00	724.00	712.00	703.67	517.00
BMP2	485.00	475.00	527.00	495.67	534.00
BMP2K	238.00	224.00	252.00	238.00	200.00
BMP3	9.00	8.00	14.00	10.33	12.00
BMP4	84.00	90.00	73.00	82.33	78.00
BMP6	264.00	218.00	255.00	245.67	631.00
BMPER	356.00	376.00	420.00	384.00	304.00
BMPR1A	564.00	471.00	587.00	540.67	558.00
BMPR1B	104.00	115.00	116.00	111.67	75.00
BMPR2	1417.00	1346.00	1609.00	1457.33	1285.00
BMS1	2836.00	2795.00	2933.00	2854.67	2817.00
BMS1P20	457.00	437.00	426.00	440.00	432.00
BMS1P21	19.00	18.00	20.00	19.00	9.00
BMS1P4	304.00	284.00	281.00	289.67	191.00
BMS1P5	212.00	235.00	216.00	221.00	162.00
BMS1P6	99.00	82.00	112.00	97.67	68.00
BNC1	3219.00	2969.00	3425.00	3204.33	3505.00
BNC2	537.00	526.00	532.00	531.67	308.00
BNIP1	344.00	323.00	326.00	331.00	444.00
BNIP2	1305.00	1109.00	1294.00	1236.00	1538.00
BNIP3	1710.00	1631.00	1901.00	1747.33	2398.00
BNIP3L	950.00	905.00	970.00	941.67	863.00
BNIPL	40.00	39.00	44.00	41.00	34.00
BOC	369.00	399.00	398.00	388.67	295.00
BOD1	1497.00	1468.00	1602.00	1522.33	1932.00
BOD1L1	1394.00	1316.00	1495.00	1401.67	1205.00
BOK	114.00	98.00	118.00	110.00	96.00

BOLA1	234.00	250.00	250.00	244.67	291.00
BOLA3	348.00	311.00	377.00	345.33	468.00
BOLA3-AS1	77.00	94.00	68.00	79.67	76.00
BOP1	3303.00	3382.00	3383.00	3356.00	4125.00
BORA	282.00	289.00	325.00	298.67	376.00
BPGM	186.00	161.00	205.00	184.00	246.00
BPHL	317.00	320.00	343.00	326.67	368.00
BPNT1	472.00	467.00	488.00	475.67	595.00
BPTF	2444.00	2091.00	2501.00	2345.33	1944.00
BRAF	239.00	239.00	257.00	245.00	238.00
BRAP	541.00	496.00	530.00	522.33	583.00
BRAT1	1871.00	1891.00	1941.00	1901.00	2036.00
BRCA1	965.00	909.00	925.00	933.00	898.00
BRCA2	381.00	364.00	423.00	389.33	421.00
BRCAT54	9.00	14.00	21.00	14.67	14.00
BRCC3	442.00	484.00	459.00	461.67	576.00
BRD1	966.00	943.00	1056.00	988.33	961.00
BRD2	20.00	13.00	15.00	16.00	12.00
BRD3	969.00	938.00	995.00	967.33	832.00
BRD4	2057.00	1948.00	2066.00	2023.67	1994.00
BRD7	1219.00	1249.00	1296.00	1254.67	1559.00
BRD8	2103.00	2200.00	2307.00	2203.33	2319.00
BRD9	1166.00	1326.00	1440.00	1310.67	1354.00
BRDTP1	13.00	19.00	23.00	18.33	13.00
BRE	1967.00	1796.00	2069.00	1944.00	1851.00
BRE-AS1	24.00	33.00	29.00	28.67	18.00
BRF1	719.00	775.00	751.00	748.33	837.00
BRF2	310.00	345.00	343.00	332.67	411.00
BRI3	1273.00	1166.00	1207.00	1215.33	1212.00
BRI3BP	58.00	52.00	57.00	55.67	85.00
BRICD5	180.00	214.00	205.00	199.67	211.00
BRIP1	248.00	247.00	275.00	256.67	344.00
BRIX1	1162.00	1132.00	1250.00	1181.33	1722.00
BRK1	1480.00	1439.00	1593.00	1504.00	1674.00
BRMS1	1169.00	1221.00	1296.00	1228.67	1472.00
BRMS1L	226.00	250.00	268.00	248.00	288.00
BROX	1382.00	1237.00	1472.00	1363.67	1365.00
BRPF1	845.00	873.00	811.00	843.00	835.00
BRPF3	1919.00	1783.00	1971.00	1891.00	1634.00
BRSK1	193.00	184.00	193.00	190.00	160.00
BRSK2	21.00	39.00	34.00	31.33	32.00
BRWD1	1350.00	1291.00	1542.00	1394.33	1224.00
BRWD1-IT2	5.00	5.00	8.00	6.00	6.00
BRWD3	832.00	824.00	1009.00	888.33	815.00
BSCL2	163.00	192.00	167.00	174.00	147.00
BSDC1	1756.00	1775.00	1897.00	1809.33	1440.00
BSG	24092.00	25714.00	23756.00	24520.67	24681.00
BSN	27.00	27.00	38.00	30.67	18.00

BSN-AS2	5.00	3.00	5.00	4.33	5.00
BST1	372.00	359.00	388.00	373.00	332.00
BTAF1	3487.00	3258.00	3608.00	3451.00	2659.00
BTBD1	1431.00	1300.00	1636.00	1455.67	1689.00
BTBD10	828.00	677.00	828.00	777.67	901.00
BTBD11	351.00	357.00	416.00	374.67	255.00
BTBD19	509.00	566.00	563.00	546.00	387.00
BTBD2	1972.00	2000.00	2122.00	2031.33	2309.00
BTBD3	955.00	840.00	1114.00	969.67	1167.00
BTBD6	554.00	511.00	545.00	536.67	721.00
BTBD8	40.00	35.00	28.00	34.33	26.00
BTBD9	362.00	355.00	374.00	363.67	322.00
BTC	10.00	12.00	12.00	11.33	11.00
BTD	627.00	577.00	598.00	600.67	492.00
BTF3	8785.00	8843.00	9573.00	9067.00	12142.00
BTF3L4	1224.00	1105.00	1325.00	1218.00	1653.00
BTG1	1681.00	1504.00	1775.00	1653.33	2171.00
BTG3	428.00	426.00	451.00	435.00	638.00
BTN2A1	1302.00	1216.00	1288.00	1268.67	1260.00
BTN2A2	462.00	436.00	457.00	451.67	452.00
BTN2A3P	245.00	234.00	255.00	244.67	214.00
BTN3A1	637.00	612.00	649.00	632.67	690.00
BTN3A2	564.00	606.00	604.00	591.33	581.00
BTN3A3	308.00	289.00	309.00	302.00	255.00
BTNL8	11.00	2.00	0.00	4.33	27.00
BTRC	921.00	844.00	915.00	893.33	973.00
BUB1	1304.00	1210.00	1322.00	1278.67	1627.00
BUB1B	1129.00	1136.00	1144.00	1136.33	1360.00
BUB3	5649.00	5391.00	5958.00	5666.00	6069.00
BUD13	360.00	340.00	374.00	358.00	397.00
BUD31	1268.00	1277.00	1341.00	1295.33	1693.00
BVES	220.00	198.00	193.00	203.67	240.00
BVES-AS1	4.00	4.00	2.00	3.33	2.00
BYSL	961.00	1003.00	1052.00	1005.33	1159.00
BZRAP1	142.00	168.00	209.00	173.00	170.00
BZRAP1-AS1	69.00	56.00	48.00	57.67	79.00
BZW1	3669.00	3382.00	3806.00	3619.00	4880.00
BZW2	2162.00	2129.00	2401.00	2230.67	3130.00
C10orf10	11.00	8.00	6.00	8.33	18.00
C10orf11	73.00	75.00	72.00	73.33	92.00
C10orf111	42.00	49.00	46.00	45.67	32.00
C10orf12	112.00	88.00	112.00	104.00	67.00
C10orf2	1173.00	1278.00	1477.00	1309.33	1446.00
C10orf25	70.00	69.00	87.00	75.33	93.00
C10orf32	247.00	242.00	262.00	250.33	284.00
C10orf54	4.00	3.00	1.00	2.67	2.00
C10orf55	31.00	21.00	26.00	26.00	33.00
C10orf67	8.00	5.00	5.00	6.00	9.00

C10orf76	732.00	723.00	754.00	736.33	685.00
C10orf88	560.00	590.00	590.00	580.00	655.00
C10orf90	45.00	44.00	46.00	45.00	62.00
C11orf1	200.00	201.00	245.00	215.33	261.00
C11orf24	788.00	822.00	776.00	795.33	888.00
C11orf30	760.00	698.00	747.00	735.00	681.00
C11orf31	1062.00	981.00	1133.00	1058.67	1281.00
C11orf45	74.00	66.00	70.00	70.00	77.00
C11orf49	396.00	421.00	456.00	424.33	494.00
C11orf54	514.00	398.00	573.00	495.00	490.00
C11orf57	727.00	636.00	749.00	704.00	789.00
C11orf58	2380.00	2232.00	2578.00	2396.67	3015.00
C11orf63	4.00	1.00	3.00	2.67	1.00
C11orf65	6.00	7.00	5.00	6.00	6.00
C11orf68	1809.00	1754.00	1922.00	1828.33	2217.00
C11orf70	362.00	329.00	375.00	355.33	514.00
C11orf71	83.00	90.00	96.00	89.67	105.00
C11orf73	477.00	479.00	501.00	485.67	570.00
C11orf74	251.00	234.00	253.00	246.00	327.00
C11orf80	492.00	511.00	531.00	511.33	637.00
C11orf84	1275.00	1340.00	1368.00	1327.67	1442.00
C11orf91	118.00	104.00	149.00	123.67	149.00
C11orf94	6.00	8.00	9.00	7.67	5.00
C11orf95	1490.00	1465.00	1820.00	1591.67	1433.00
C11orf98	50.00	67.00	49.00	55.33	62.00
C12orf10	1183.00	1172.00	1186.00	1180.33	1612.00
C12orf29	581.00	543.00	652.00	592.00	619.00
C12orf4	574.00	537.00	592.00	567.67	699.00
C12orf43	273.00	284.00	236.00	264.33	306.00
C12orf45	151.00	171.00	177.00	166.33	213.00
C12orf49	852.00	863.00	879.00	864.67	916.00
C12orf56	72.00	58.00	46.00	58.67	74.00
C12orf57	1083.00	1054.00	1121.00	1086.00	1161.00
C12orf60	62.00	46.00	51.00	53.00	59.00
C12orf65	305.00	318.00	343.00	322.00	366.00
C12orf66	275.00	216.00	278.00	256.33	236.00
C12orf73	243.00	227.00	245.00	238.33	252.00
C12orf75	710.00	584.00	729.00	674.33	896.00
C12orf76	185.00	209.00	227.00	207.00	272.00
C14orf1	440.00	400.00	427.00	422.33	445.00
C14orf119	918.00	987.00	1064.00	989.67	1168.00
C14orf132	86.00	61.00	74.00	73.67	66.00
C14orf159	465.00	492.00	546.00	501.00	542.00
C14orf166	1954.00	1888.00	2218.00	2020.00	2640.00
C14orf169	418.00	398.00	433.00	416.33	480.00
C14orf2	822.00	843.00	882.00	849.00	1200.00
C14orf28	38.00	49.00	52.00	46.33	51.00
C14orf37	15.00	15.00	15.00	15.00	14.00

C14orf39	4.00	3.00	3.00	3.33	1.00
C14orf79	426.00	407.00	421.00	418.00	423.00
C14orf80	303.00	270.00	234.00	269.00	354.00
C14orf93	292.00	311.00	357.00	320.00	352.00
C15orf26	24.00	12.00	14.00	16.67	14.00
C15orf39	367.00	291.00	301.00	319.67	320.00
C15orf40	316.00	296.00	280.00	297.33	359.00
C15orf41	432.00	416.00	448.00	432.00	510.00
C15orf48	6.00	9.00	9.00	8.00	6.00
C15orf52	5598.00	5859.00	5863.00	5773.33	5241.00
C15orf54	4.00	1.00	2.00	2.33	8.00
C15orf56	5.00	4.00	9.00	6.00	3.00
C15orf57	206.00	195.00	231.00	210.67	194.00
C15orf61	128.00	108.00	128.00	121.33	201.00
C15orf65	8.00	13.00	7.00	9.33	19.00
C16orf13	772.00	783.00	912.00	822.33	1090.00
C16orf46	91.00	82.00	68.00	80.33	88.00
C16orf52	1782.00	1576.00	1924.00	1760.67	1960.00
C16orf58	3383.00	3318.00	3428.00	3376.33	3183.00
C16orf59	342.00	357.00	307.00	335.33	438.00
C16orf62	1151.00	1041.00	1257.00	1149.67	970.00
C16orf70	461.00	495.00	499.00	485.00	510.00
C16orf72	1020.00	814.00	1035.00	956.33	1097.00
C16orf74	409.00	420.00	418.00	415.67	295.00
C16orf86	20.00	41.00	21.00	27.33	27.00
C16orf87	71.00	71.00	63.00	68.33	117.00
C16orf91	283.00	262.00	275.00	273.33	329.00
C16orf93	86.00	74.00	62.00	74.00	83.00
C16orf95	95.00	103.00	98.00	98.67	114.00
C17orf100	101.00	83.00	94.00	92.67	95.00
C17orf104	5.00	11.00	7.00	7.67	14.00
C17orf107	18.00	25.00	36.00	26.33	23.00
C17orf51	2929.00	2672.00	3169.00	2923.33	2190.00
C17orf53	335.00	303.00	324.00	320.67	351.00
C17orf58	295.00	300.00	302.00	299.00	455.00
C17orf59	191.00	188.00	210.00	196.33	183.00
C17orf62	1510.00	1432.00	1642.00	1528.00	1736.00
C17orf67	51.00	56.00	67.00	58.00	59.00
C17orf75	518.00	505.00	518.00	513.67	676.00
C17orf80	839.00	795.00	900.00	844.67	892.00
C17orf85	2152.00	2076.00	2328.00	2185.33	2147.00
C17orf89	432.00	399.00	418.00	416.33	570.00
C17orf97	23.00	28.00	22.00	24.33	27.00
C18orf21	221.00	241.00	237.00	233.00	313.00
C18orf25	520.00	501.00	530.00	517.00	672.00
C18orf32	22.00	23.00	24.00	23.00	10.00
C18orf54	421.00	390.00	463.00	424.67	510.00
C18orf65	38.00	35.00	30.00	34.33	43.00

C18orf8	612.00	524.00	601.00	579.00	599.00
C19orf12	455.00	444.00	480.00	459.67	599.00
C19orf24	469.00	459.00	483.00	470.33	637.00
C19orf25	382.00	332.00	426.00	380.00	446.00
C19orf38	7.00	6.00	6.00	6.33	10.00
C19orf43	1665.00	1583.00	1711.00	1653.00	1993.00
C19orf44	388.00	359.00	367.00	371.33	376.00
C19orf47	146.00	144.00	156.00	148.67	182.00
C19orf48	2048.00	2050.00	2234.00	2110.67	2682.00
C19orf52	221.00	251.00	243.00	238.33	279.00
C19orf53	871.00	851.00	912.00	878.00	1188.00
C19orf54	680.00	687.00	723.00	696.67	656.00
C19orf57	40.00	42.00	46.00	42.67	34.00
C19orf60	410.00	473.00	466.00	449.67	585.00
C19orf66	517.00	535.00	541.00	531.00	506.00
C19orf68	324.00	421.00	363.00	369.33	386.00
C19orf70	463.00	430.00	537.00	476.67	566.00
C19orf71	26.00	26.00	18.00	23.33	18.00
C19orf73	19.00	21.00	10.00	16.67	16.00
C1D	225.00	236.00	241.00	234.00	322.00
C1GALT1	1692.00	1673.00	1921.00	1762.00	1913.00
C1GALT1C1	1089.00	1095.00	1009.00	1064.33	1199.00
C1GALT1C1L	26.00	25.00	25.00	25.33	30.00
C1orf101	27.00	26.00	22.00	25.00	21.00
C1orf109	714.00	671.00	754.00	713.00	821.00
C1orf110	29.00	27.00	23.00	26.33	72.00
C1orf112	426.00	425.00	391.00	414.00	581.00
C1orf122	390.00	400.00	463.00	417.67	472.00
C1orf123	792.00	805.00	931.00	842.67	921.00
C1orf131	169.00	152.00	200.00	173.67	228.00
C1orf159	442.00	426.00	445.00	437.67	523.00
C1orf162	42.00	39.00	51.00	44.00	44.00
C1orf174	599.00	525.00	623.00	582.33	742.00
C1orf189	4.00	4.00	0.00	2.67	0.00
C1orf198	365.00	326.00	372.00	354.33	378.00
C1orf204	169.00	174.00	183.00	175.33	167.00
C1orf21	473.00	433.00	454.00	453.33	544.00
C1orf216	792.00	798.00	916.00	835.33	992.00
C1orf220	54.00	62.00	65.00	60.33	56.00
C1orf226	70.00	78.00	74.00	74.00	70.00
C1orf228	11.00	11.00	16.00	12.67	21.00
C1orf27	1104.00	1075.00	1173.00	1117.33	1200.00
C1orf35	392.00	422.00	428.00	414.00	455.00
C1orf43	6231.00	6033.00	6295.00	6186.33	8006.00
C1orf50	119.00	164.00	160.00	147.67	190.00
C1orf52	516.00	507.00	555.00	526.00	631.00
C1orf53	38.00	53.00	37.00	42.67	66.00
C1orf54	11.00	16.00	11.00	12.67	19.00

C1orf56	158.00	174.00	176.00	169.33	202.00
C1orf74	205.00	198.00	209.00	204.00	247.00
C1QBP	4781.00	4774.00	4987.00	4847.33	6950.00
C1QL4	59.00	54.00	37.00	50.00	66.00
C1QTNF1	75.00	70.00	74.00	73.00	29.00
C1QTNF3	32.00	34.00	30.00	32.00	38.00
C1QTNF6	373.00	374.00	404.00	383.67	384.00
C1QTNF9B-AS1	21.00	19.00	28.00	22.67	16.00
C1R	46.00	32.00	42.00	40.00	42.00
C1RL	274.00	288.00	277.00	279.67	244.00
C1RL-AS1	640.00	621.00	677.00	646.00	377.00
C1S	49.00	32.00	32.00	37.67	29.00
C20orf194	1077.00	953.00	1018.00	1016.00	920.00
C20orf196	36.00	42.00	37.00	38.33	46.00
C20orf197	4.00	5.00	5.00	4.67	14.00
C20orf24	238.00	225.00	206.00	223.00	318.00
C20orf27	1214.00	1288.00	1252.00	1251.33	1606.00
C20orf96	438.00	446.00	468.00	450.67	460.00
C21orf2	229.00	211.00	252.00	230.67	231.00
C21orf33	70.00	64.00	95.00	76.33	110.00
C21orf58	631.00	509.00	488.00	542.67	566.00
C21orf59	1068.00	1050.00	1026.00	1048.00	1277.00
C21orf91	189.00	161.00	230.00	193.33	200.00
C22orf23	6.00	7.00	7.00	6.67	7.00
C22orf29	1077.00	1024.00	1157.00	1086.00	988.00
C22orf34	8.00	12.00	11.00	10.33	15.00
C22orf39	275.00	292.00	283.00	283.33	394.00
C22orf46	917.00	884.00	945.00	915.33	705.00
C2CD2	1609.00	1562.00	1708.00	1626.33	1286.00
C2CD2L	1018.00	1053.00	1042.00	1037.67	985.00
C2CD3	564.00	534.00	603.00	567.00	559.00
C2CD5	1402.00	1340.00	1492.00	1411.33	1573.00
C2orf16	11.00	8.00	9.00	9.33	12.00
C2orf27A	7.00	4.00	7.00	6.00	7.00
C2orf42	271.00	250.00	342.00	287.67	343.00
C2orf44	469.00	462.00	479.00	470.00	484.00
C2orf47	335.00	350.00	306.00	330.33	421.00
C2orf48	18.00	24.00	20.00	20.67	40.00
C2orf49	628.00	594.00	625.00	615.67	562.00
C2orf61	4.00	11.00	7.00	7.33	5.00
C2orf66	18.00	15.00	25.00	19.33	19.00
C2orf68	846.00	807.00	890.00	847.67	904.00
C2orf69	433.00	384.00	458.00	425.00	491.00
C2orf72	6.00	0.00	5.00	3.67	5.00
C2orf74	237.00	237.00	261.00	245.00	368.00
C2orf76	85.00	71.00	94.00	83.33	109.00
C2orf81	72.00	84.00	89.00	81.67	97.00
C2orf82	17.00	21.00	19.00	19.00	11.00

C2orf88	7.00	11.00	6.00	8.00	13.00
C3AR1	13.00	8.00	7.00	9.33	25.00
C3orf14	221.00	267.00	228.00	238.67	371.00
C3orf17	1529.00	1416.00	1532.00	1492.33	1572.00
C3orf18	52.00	54.00	57.00	54.33	69.00
C3orf20	15.00	9.00	9.00	11.00	5.00
C3orf33	158.00	139.00	158.00	151.67	191.00
C3orf35	46.00	46.00	47.00	46.33	43.00
C3orf38	360.00	326.00	382.00	356.00	381.00
C3orf52	327.00	275.00	287.00	296.33	201.00
C3orf58	495.00	491.00	583.00	523.00	532.00
C3orf62	122.00	137.00	153.00	137.33	151.00
C3orf67	163.00	155.00	141.00	153.00	197.00
C3orf70	13.00	8.00	17.00	12.67	16.00
C3orf80	14.00	19.00	13.00	15.33	23.00
C4orf19	138.00	114.00	124.00	125.33	71.00
C4orf22	7.00	12.00	2.00	7.00	11.00
C4orf27	262.00	260.00	223.00	248.33	333.00
C4orf29	131.00	112.00	128.00	123.67	139.00
C4orf3	496.00	442.00	517.00	485.00	549.00
C4orf32	219.00	227.00	255.00	233.67	310.00
C4orf33	107.00	121.00	106.00	111.33	132.00
C4orf36	12.00	18.00	21.00	17.00	16.00
C4orf46	279.00	265.00	286.00	276.67	342.00
C4orf47	26.00	22.00	34.00	27.33	19.00
C4orf48	223.00	257.00	287.00	255.67	336.00
C5	135.00	132.00	153.00	140.00	231.00
C5AR1	15.00	8.00	17.00	13.33	8.00
C5orf15	1268.00	1227.00	1212.00	1235.67	1481.00
C5orf22	645.00	538.00	662.00	615.00	662.00
C5orf24	1730.00	1514.00	1850.00	1698.00	1431.00
C5orf28	710.00	649.00	763.00	707.33	811.00
C5orf30	1025.00	852.00	1069.00	982.00	1477.00
C5orf34	544.00	468.00	449.00	487.00	590.00
C5orf42	720.00	647.00	710.00	692.33	519.00
C5orf45	731.00	756.00	850.00	779.00	702.00
C5orf51	1484.00	1483.00	1657.00	1541.33	1546.00
C5orf56	40.00	22.00	33.00	31.67	25.00
C5orf63	49.00	41.00	45.00	45.00	40.00
C5orf66	186.00	194.00	185.00	188.33	212.00
C6orf1	819.00	810.00	775.00	801.33	943.00
C6orf106	1371.00	1285.00	1489.00	1381.67	1484.00
C6orf120	2322.00	2140.00	2263.00	2241.67	2694.00
C6orf141	397.00	453.00	439.00	429.67	335.00
C6orf163	50.00	59.00	60.00	56.33	54.00
C6orf165	7.00	9.00	9.00	8.33	5.00
C6orf203	384.00	366.00	370.00	373.33	389.00
C6orf226	41.00	26.00	37.00	34.67	47.00

C6orf48	8.00	9.00	6.00	7.67	13.00
C6orf52	21.00	23.00	21.00	21.67	24.00
C6orf58	10.00	1.00	9.00	6.67	35.00
C6orf62	3883.00	3727.00	4293.00	3967.67	4894.00
C6orf89	3096.00	2888.00	3023.00	3002.33	2856.00
C7orf13	318.00	253.00	326.00	299.00	303.00
C7orf25	403.00	377.00	406.00	395.33	531.00
C7orf26	485.00	509.00	568.00	520.67	561.00
C7orf31	99.00	84.00	78.00	87.00	92.00
C7orf43	569.00	561.00	555.00	561.67	583.00
C7orf49	953.00	920.00	1004.00	959.00	1075.00
C7orf50	1895.00	1881.00	2080.00	1952.00	2473.00
C7orf55	118.00	144.00	116.00	126.00	131.00
C7orf57	21.00	19.00	22.00	20.67	17.00
C7orf60	187.00	154.00	180.00	173.67	175.00
C7orf61	28.00	27.00	20.00	25.00	17.00
C7orf73	730.00	673.00	749.00	717.33	1012.00
C8G	8.00	24.00	17.00	16.33	22.00
C8orf33	1466.00	1363.00	1432.00	1420.33	1682.00
C8orf37	166.00	155.00	192.00	171.00	162.00
C8orf4	9.00	6.00	16.00	10.33	7.00
C8orf44	152.00	143.00	194.00	163.00	152.00
C8orf46	45.00	36.00	49.00	43.33	50.00
C8orf48	68.00	82.00	72.00	74.00	75.00
C8orf58	364.00	395.00	368.00	375.67	526.00
C8orf59	713.00	708.00	742.00	721.00	946.00
C8orf76	114.00	141.00	126.00	127.00	127.00
C8orf82	391.00	381.00	456.00	409.33	490.00
C8orf88	311.00	254.00	345.00	303.33	400.00
C9orf114	1636.00	1735.00	1727.00	1699.33	1890.00
C9orf116	19.00	23.00	31.00	24.33	18.00
C9orf142	989.00	1017.00	1013.00	1006.33	1291.00
C9orf156	147.00	158.00	175.00	160.00	206.00
C9orf16	410.00	478.00	489.00	459.00	635.00
C9orf163	27.00	32.00	30.00	29.67	20.00
C9orf172	34.00	30.00	43.00	35.67	25.00
C9orf173-AS1	23.00	31.00	20.00	24.67	29.00
C9orf24	6.00	11.00	8.00	8.33	10.00
C9orf3	144.00	134.00	163.00	147.00	124.00
C9orf40	382.00	374.00	418.00	391.33	501.00
C9orf43	16.00	22.00	23.00	20.33	21.00
C9orf47	13.00	11.00	17.00	13.67	17.00
C9orf69	1965.00	2150.00	2067.00	2060.67	2245.00
C9orf72	528.00	537.00	655.00	573.33	527.00
C9orf78	935.00	943.00	1013.00	963.67	1199.00
C9orf85	147.00	139.00	182.00	156.00	209.00
C9orf89	296.00	280.00	275.00	283.67	301.00
C9orf9	115.00	116.00	140.00	123.67	120.00

C9orf91	855.00	841.00	856.00	850.67	1053.00
CA11	18.00	19.00	19.00	18.67	23.00
CA12	108.00	88.00	94.00	96.67	89.00
CA13	379.00	373.00	384.00	378.67	310.00
CA2	9.00	12.00	14.00	11.67	12.00
CA3	69.00	56.00	98.00	74.33	61.00
CA5B	197.00	163.00	188.00	182.67	194.00
CA5BP1	328.00	342.00	281.00	317.00	328.00
CA8	17.00	13.00	13.00	14.33	21.00
CAAP1	455.00	395.00	426.00	425.33	496.00
CAB39	1521.00	1260.00	1561.00	1447.33	1494.00
CAB39L	187.00	160.00	189.00	178.67	186.00
CABIN1	1415.00	1432.00	1501.00	1449.33	1354.00
CABLES1	299.00	311.00	342.00	317.33	245.00
CABLES2	158.00	147.00	196.00	167.00	164.00
CABYR	5.00	3.00	14.00	7.33	4.00
CACFD1	26.00	30.00	37.00	31.00	28.00
CACHD1	503.00	446.00	513.00	487.33	503.00
CACNA2D1	777.00	686.00	829.00	764.00	719.00
CACNA2D4	4696.00	4830.00	5203.00	4909.67	4360.00
CACNB1	346.00	368.00	363.00	359.00	346.00
CACNB3	527.00	462.00	531.00	506.67	510.00
CACNB4	89.00	64.00	89.00	80.67	102.00
CACNG6	114.00	106.00	145.00	121.67	104.00
CACNG7	204.00	163.00	182.00	183.00	126.00
CACNG8	1389.00	1381.00	1566.00	1445.33	955.00
CACTIN	901.00	956.00	999.00	952.00	903.00
CACTIN-AS1	22.00	13.00	25.00	20.00	23.00
CACUL1	1205.00	1179.00	1303.00	1229.00	1473.00
CACYBP	1660.00	1591.00	1698.00	1649.67	2542.00
CAD	6060.00	5929.00	6429.00	6139.33	5534.00
CADM4	11.00	11.00	9.00	10.33	5.00
CADPS	20.00	18.00	27.00	21.67	22.00
CADPS2	356.00	292.00	395.00	347.67	415.00
CAHM	43.00	43.00	33.00	39.67	35.00
CALCOCO1	1570.00	1577.00	1748.00	1631.67	1189.00
CALCOCO2	1734.00	1517.00	1736.00	1662.33	1930.00
CALCRL	66.00	35.00	51.00	50.67	56.00
CALD1	3406.00	3086.00	3593.00	3361.67	4108.00
CALHM2	23.00	20.00	25.00	22.67	19.00
CALM1	2136.00	1987.00	2178.00	2100.33	2743.00
CALM2	14641.00	13583.00	15636.00	14620.00	14343.00
CALM3	2098.00	2012.00	2205.00	2105.00	2642.00
CALML4	327.00	332.00	355.00	338.00	296.00
CALML6	15.00	20.00	14.00	16.33	13.00
CALR	18604.00	19029.00	17844.00	18492.33	18400.00
CALU	15901.00	15237.00	15344.00	15494.00	15514.00
CAMK1	409.00	416.00	410.00	411.67	492.00

CAMK1D	22.00	19.00	16.00	19.00	18.00
CAMK1G	644.00	631.00	685.00	653.33	842.00
CAMK2B	79.00	108.00	120.00	102.33	95.00
CAMK2D	402.00	345.00	432.00	393.00	404.00
CAMK2G	1256.00	1385.00	1293.00	1311.33	1449.00
CAMK2N1	503.00	485.00	596.00	528.00	615.00
CAMK2N2	22.00	26.00	19.00	22.33	35.00
CAMK4	85.00	95.00	85.00	88.33	106.00
CAMKK1	318.00	321.00	369.00	336.00	280.00
CAMKK2	1200.00	1082.00	1245.00	1175.67	1252.00
CAMKMT	122.00	108.00	148.00	126.00	164.00
CAMLG	1249.00	1240.00	1317.00	1268.67	1384.00
CAMSAP1	1657.00	1460.00	1753.00	1623.33	1485.00
CAMSAP2	1718.00	1498.00	1794.00	1670.00	1626.00
CAMTA1	356.00	294.00	366.00	338.67	463.00
CAMTA2	5684.00	5647.00	6283.00	5871.33	5341.00
CAND1	2625.00	2484.00	2756.00	2621.67	2579.00
CANT1	3120.00	3196.00	3048.00	3121.33	3098.00
CANX	30446.00	28914.00	31528.00	30296.00	26840.00
CAP1	7062.00	6624.00	7374.00	7020.00	7961.00
CAP2	825.00	670.00	846.00	780.33	741.00
CAPG	7.00	8.00	15.00	10.00	18.00
CAPN1	2917.00	2936.00	3014.00	2955.67	2999.00
CAPN10	720.00	719.00	748.00	729.00	695.00
CAPN10-AS1	216.00	214.00	195.00	208.33	154.00
CAPN11	19.00	15.00	14.00	16.00	12.00
CAPN15	2816.00	2854.00	3079.00	2916.33	2892.00
CAPN2	12275.00	11974.00	13401.00	12550.00	11920.00
CAPN3	116.00	118.00	124.00	119.33	109.00
CAPN5	382.00	394.00	409.00	395.00	285.00
CAPN7	708.00	715.00	801.00	741.33	657.00
CAPNS1	3485.00	3310.00	3565.00	3453.33	4208.00
CAPNS2	4.00	1.00	0.00	1.67	0.00
CAPRIN1	6160.00	5614.00	6505.00	6093.00	6869.00
CAPRIN2	4503.00	4485.00	5163.00	4717.00	5108.00
CAPS	208.00	239.00	232.00	226.33	199.00
CAPS2	62.00	80.00	64.00	68.67	75.00
CAPZA1	4012.00	3606.00	4231.00	3949.67	5496.00
CAPZA2	1108.00	1106.00	1276.00	1163.33	1589.00
CAPZB	2250.00	2203.00	2268.00	2240.33	2398.00
CARD10	1566.00	1636.00	1581.00	1594.33	1615.00
CARD6	172.00	150.00	185.00	169.00	152.00
CARD8	2281.00	2101.00	2328.00	2236.67	3017.00
CARD8-AS1	128.00	114.00	132.00	124.67	130.00
CARD9	46.00	56.00	38.00	46.67	25.00
CARF	317.00	299.00	308.00	308.00	276.00
CARHSP1	193.00	222.00	229.00	214.67	366.00
CARKD	1105.00	1135.00	1021.00	1087.00	1117.00

CARM1	1818.00	1644.00	1934.00	1798.67	1962.00
CARNMT1	311.00	245.00	366.00	307.33	386.00
CARS	276.00	260.00	282.00	272.67	277.00
CARS2	1117.00	1108.00	1284.00	1169.67	1275.00
CASC10	60.00	55.00	70.00	61.67	64.00
CASC11	6.00	3.00	5.00	4.67	2.00
CASC15	5.00	6.00	4.00	5.00	1.00
CASC2	44.00	26.00	37.00	35.67	48.00
CASC3	2805.00	2688.00	3027.00	2840.00	2892.00
CASC4	2050.00	1856.00	2135.00	2013.67	2020.00
CASC5	325.00	298.00	293.00	305.33	307.00
CASC9	25.00	21.00	25.00	23.67	28.00
CASD1	531.00	497.00	646.00	558.00	548.00
CASK	386.00	349.00	402.00	379.00	398.00
CASKIN2	513.00	471.00	545.00	509.67	518.00
CASP1	59.00	63.00	56.00	59.33	69.00
CASP10	13.00	10.00	17.00	13.33	12.00
CASP2	948.00	862.00	904.00	904.67	977.00
CASP3	469.00	426.00	512.00	469.00	711.00
CASP4	1997.00	2012.00	2185.00	2064.67	2224.00
CASP5	12.00	5.00	0.00	5.67	0.00
CASP6	97.00	66.00	104.00	89.00	96.00
CASP7	372.00	331.00	394.00	365.67	346.00
CASP8	837.00	785.00	825.00	815.67	862.00
CASP8AP2	746.00	696.00	811.00	751.00	712.00
CASP9	238.00	233.00	245.00	238.67	250.00
CASS4	68.00	58.00	78.00	68.00	125.00
CAST	3277.00	2949.00	3425.00	3217.00	3527.00
CAT	362.00	343.00	374.00	359.67	474.00
CATSPER1	165.00	179.00	191.00	178.33	232.00
CATSPER2	129.00	125.00	130.00	128.00	138.00
CATSPER2P1	27.00	23.00	24.00	24.67	22.00
CATSPER3	33.00	30.00	32.00	31.67	31.00
CATSPERG	6.00	3.00	2.00	3.67	2.00
CAV1	11457.00	9991.00	11245.00	10897.67	14862.00
CAV2	944.00	779.00	978.00	900.33	1135.00
CBARP	313.00	313.00	337.00	321.00	268.00
CBFA2T2	1106.00	1035.00	1129.00	1090.00	941.00
CBFB	1525.00	1408.00	1687.00	1540.00	2296.00
CBL	1804.00	1631.00	1939.00	1791.33	1442.00
CBLB	802.00	681.00	887.00	790.00	867.00
CBLL1	618.00	623.00	712.00	651.00	732.00
CBLN3	74.00	62.00	68.00	68.00	45.00
CBR1	935.00	954.00	1034.00	974.33	1274.00
CBR3	47.00	58.00	47.00	50.67	82.00
CBR3-AS1	11.00	22.00	22.00	18.33	16.00
CBR4	485.00	409.00	489.00	461.00	551.00
CBS	171.00	206.00	191.00	189.33	189.00

CBWD1	311.00	283.00	334.00	309.33	406.00
CBWD2	238.00	297.00	292.00	275.67	260.00
CBWD3	55.00	64.00	69.00	62.67	40.00
CBWD5	297.00	307.00	362.00	322.00	304.00
CBWD6	146.00	192.00	181.00	173.00	171.00
CBX1	1309.00	1281.00	1415.00	1335.00	1225.00
CBX2	182.00	190.00	139.00	170.33	216.00
CBX3	4392.00	4179.00	4552.00	4374.33	5975.00
CBX3P2	31.00	30.00	32.00	31.00	18.00
CBX4	541.00	596.00	627.00	588.00	624.00
CBX5	2245.00	2007.00	2407.00	2219.67	2502.00
CBX6	2186.00	2009.00	2359.00	2184.67	2485.00
CBX7	301.00	351.00	365.00	339.00	324.00
CBX8	119.00	132.00	115.00	122.00	145.00
CBY1	369.00	377.00	392.00	379.33	562.00
CC2D1A	1486.00	1458.00	1513.00	1485.67	1445.00
CC2D1B	1516.00	1557.00	1582.00	1551.67	1450.00
CC2D2A	537.00	466.00	543.00	515.33	556.00
CCAR1	3962.00	3722.00	4016.00	3900.00	3991.00
CCAR2	1966.00	1993.00	2044.00	2001.00	2176.00
CCBE1	5055.00	4624.00	4785.00	4821.33	3860.00
CCBL1	619.00	571.00	688.00	626.00	730.00
CCBL2	865.00	771.00	885.00	840.33	1090.00
CCDC101	209.00	210.00	205.00	208.00	257.00
CCDC102A	296.00	284.00	283.00	287.67	338.00
CCDC102B	25.00	32.00	32.00	29.67	35.00
CCDC103	16.00	15.00	13.00	14.67	28.00
CCDC106	261.00	269.00	274.00	268.00	292.00
CCDC107	194.00	225.00	194.00	204.33	244.00
CCDC109B	170.00	132.00	157.00	153.00	258.00
CCDC110	7.00	7.00	15.00	9.67	12.00
CCDC112	256.00	221.00	241.00	239.33	320.00
CCDC113	219.00	199.00	230.00	216.00	215.00
CCDC115	387.00	399.00	420.00	402.00	440.00
CCDC116	6.00	9.00	13.00	9.33	11.00
CCDC117	309.00	310.00	316.00	311.67	398.00
CCDC12	351.00	376.00	354.00	360.33	473.00
CCDC120	152.00	174.00	183.00	169.67	183.00
CCDC121	14.00	13.00	9.00	12.00	17.00
CCDC122	96.00	115.00	112.00	107.67	128.00
CCDC124	975.00	966.00	995.00	978.67	1280.00
CCDC125	6.00	22.00	21.00	16.33	29.00
CCDC126	159.00	166.00	162.00	162.33	189.00
CCDC127	348.00	357.00	332.00	345.67	317.00
CCDC130	1049.00	1115.00	1163.00	1109.00	1026.00
CCDC134	73.00	76.00	81.00	76.67	82.00
CCDC136	759.00	750.00	750.00	753.00	556.00
CCDC137	1359.00	1324.00	1358.00	1347.00	1577.00

CCDC138	140.00	139.00	159.00	146.00	208.00
CCDC14	4876.00	4762.00	5088.00	4908.67	5056.00
CCDC142	502.00	454.00	531.00	495.67	381.00
CCDC144A	25.00	31.00	28.00	28.00	20.00
CCDC144B	60.00	41.00	39.00	46.67	28.00
CCDC146	187.00	151.00	194.00	177.33	157.00
CCDC148	5.00	11.00	10.00	8.67	16.00
CCDC149	228.00	231.00	265.00	241.33	166.00
CCDC15	148.00	131.00	138.00	139.00	205.00
CCDC150	433.00	455.00	379.00	422.33	443.00
CCDC151	10.00	10.00	11.00	10.33	10.00
CCDC152	119.00	103.00	107.00	109.67	141.00
CCDC153	4.00	4.00	2.00	3.33	0.00
CCDC154	19.00	16.00	20.00	18.33	19.00
CCDC157	35.00	40.00	27.00	34.00	47.00
CCDC159	133.00	158.00	220.00	170.33	146.00
CCDC163P	214.00	240.00	229.00	227.67	197.00
CCDC167	177.00	145.00	134.00	152.00	195.00
CCDC168	5.00	8.00	4.00	5.67	4.00
CCDC169	33.00	40.00	53.00	42.00	46.00
CCDC17	139.00	165.00	219.00	174.33	128.00
CCDC170	9.00	6.00	4.00	6.33	3.00
CCDC171	85.00	74.00	82.00	80.33	122.00
CCDC173	6.00	4.00	6.00	5.33	9.00
CCDC174	493.00	493.00	561.00	515.67	521.00
CCDC176	72.00	69.00	89.00	76.67	79.00
CCDC18	708.00	623.00	672.00	667.67	700.00
CCDC181	99.00	66.00	90.00	85.00	111.00
CCDC183	17.00	36.00	24.00	25.67	13.00
CCDC183-AS1	66.00	51.00	56.00	57.67	50.00
CCDC186	300.00	279.00	327.00	302.00	278.00
CCDC22	569.00	589.00	571.00	576.33	661.00
CCDC24	90.00	89.00	82.00	87.00	122.00
CCDC25	520.00	508.00	561.00	529.67	608.00
CCDC28A	328.00	322.00	338.00	329.33	364.00
CCDC28B	58.00	67.00	62.00	62.33	104.00
CCDC30	22.00	12.00	20.00	18.00	10.00
CCDC34	243.00	257.00	306.00	268.67	361.00
CCDC36	153.00	110.00	101.00	121.33	109.00
CCDC38	4.00	0.00	0.00	1.33	2.00
CCDC39	4.00	0.00	0.00	1.33	4.00
CCDC40	42.00	54.00	64.00	53.33	36.00
CCDC43	456.00	458.00	488.00	467.33	570.00
CCDC47	5280.00	5194.00	5469.00	5314.33	5806.00
CCDC50	2275.00	2169.00	2438.00	2294.00	2007.00
CCDC51	354.00	365.00	357.00	358.67	415.00
CCDC53	338.00	352.00	366.00	352.00	447.00
CCDC57	1286.00	1274.00	1302.00	1287.33	1102.00

CCDC58	461.00	431.00	478.00	456.67	659.00
CCDC59	598.00	609.00	588.00	598.33	852.00
CCDC6	1582.00	1376.00	1811.00	1589.67	1884.00
CCDC61	239.00	246.00	268.00	251.00	337.00
CCDC62	23.00	13.00	29.00	21.67	22.00
CCDC66	641.00	702.00	748.00	697.00	680.00
CCDC69	119.00	101.00	83.00	101.00	90.00
CCDC7	142.00	153.00	164.00	153.00	175.00
CCDC70	6.00	16.00	15.00	12.33	9.00
CCDC71	413.00	465.00	465.00	447.67	566.00
CCDC71L	550.00	465.00	629.00	548.00	752.00
CCDC74A	17.00	19.00	30.00	22.00	50.00
CCDC74B	8.00	1.00	5.00	4.67	13.00
CCDC77	528.00	573.00	594.00	565.00	722.00
CCDC78	561.00	588.00	628.00	592.33	694.00
CCDC8	56.00	77.00	70.00	67.67	63.00
CCDC80	5540.00	5470.00	5702.00	5570.67	8368.00
CCDC82	715.00	647.00	676.00	679.33	769.00
CCDC84	254.00	303.00	295.00	284.00	303.00
CCDC85A	12.00	8.00	6.00	8.67	6.00
CCDC85B	915.00	900.00	967.00	927.33	1840.00
CCDC85C	433.00	423.00	463.00	439.67	450.00
CCDC86	1956.00	1951.00	2036.00	1981.00	2182.00
CCDC87	7.00	5.00	9.00	7.00	13.00
CCDC88A	1583.00	1498.00	1687.00	1589.33	1467.00
CCDC88B	49.00	52.00	49.00	50.00	80.00
CCDC88C	437.00	504.00	547.00	496.00	408.00
CCDC9	440.00	453.00	492.00	461.67	539.00
CCDC90B	616.00	593.00	708.00	639.00	808.00
CCDC91	539.00	541.00	557.00	545.67	598.00
CCDC92	25.00	22.00	22.00	23.00	25.00
CCDC93	2002.00	1962.00	2142.00	2035.33	1788.00
CCDC94	258.00	230.00	229.00	239.00	317.00
CCDC96	13.00	16.00	19.00	16.00	9.00
CCDC97	514.00	498.00	490.00	500.67	520.00
CCHCR1	45.00	44.00	38.00	42.33	36.00
CCK	5.00	6.00	6.00	5.67	0.00
CCL2	5.00	7.00	5.00	5.67	7.00
CCL20	60.00	52.00	70.00	60.67	114.00
CCL28	178.00	171.00	200.00	183.00	168.00
CCM2	687.00	819.00	782.00	762.67	875.00
CCNA1	26.00	35.00	35.00	32.00	51.00
CCNA2	555.00	498.00	572.00	541.67	713.00
CCNB1	2506.00	2294.00	2453.00	2417.67	3193.00
CCNB1IP1	1669.00	1707.00	1963.00	1779.67	1987.00
CCNB2	942.00	841.00	912.00	898.33	1099.00
CCNB3	8.00	14.00	7.00	9.67	7.00
CCNC	1033.00	898.00	1073.00	1001.33	1351.00

CCND1	12974.00	11982.00	13295.00	12750.33	13447.00
CCND3	409.00	355.00	361.00	375.00	571.00
CCNDBP1	1142.00	1102.00	1152.00	1132.00	1200.00
CCNE1	115.00	122.00	104.00	113.67	157.00
CCNE2	137.00	131.00	144.00	137.33	240.00
CCNF	478.00	443.00	435.00	452.00	577.00
CCNG1	1448.00	1269.00	1561.00	1426.00	1643.00
CCNG2	257.00	262.00	313.00	277.33	278.00
CCNH	1040.00	1052.00	1080.00	1057.33	1417.00
CCNI	2482.00	2206.00	2692.00	2460.00	2894.00
CCNJ	476.00	488.00	491.00	485.00	568.00
CCNJL	571.00	489.00	527.00	529.00	542.00
CCNK	1014.00	891.00	1029.00	978.00	1047.00
CCNL1	3733.00	3541.00	3944.00	3739.33	4061.00
CCNL2	8144.00	8096.00	8730.00	8323.33	7788.00
CCNO	344.00	365.00	423.00	377.33	394.00
CCNT1	1598.00	1401.00	1659.00	1552.67	1530.00
CCNT2	1734.00	1726.00	1932.00	1797.33	1807.00
CCNT2-AS1	17.00	14.00	21.00	17.33	8.00
CCNY	2009.00	1972.00	2294.00	2091.67	2501.00
CCNYL1	476.00	455.00	512.00	481.00	618.00
CCNYL2	198.00	237.00	219.00	218.00	195.00
CCP110	423.00	422.00	413.00	419.33	505.00
CCPG1	458.00	490.00	579.00	509.00	428.00
CCR1	29.00	13.00	20.00	20.67	31.00
CCR10	17.00	13.00	26.00	18.67	23.00
CCRL2	38.00	33.00	42.00	37.67	22.00
CCS	892.00	980.00	955.00	942.33	948.00
CCSAP	163.00	122.00	140.00	141.67	181.00
CCSER1	15.00	23.00	19.00	19.00	16.00
CCSER2	787.00	686.00	831.00	768.00	722.00
CCT2	6300.00	6099.00	6476.00	6291.67	7365.00
CCT3	12469.00	12089.00	12750.00	12436.00	15530.00
CCT4	6958.00	6752.00	7882.00	7197.33	9910.00
CCT5	10281.00	9764.00	10709.00	10251.33	13907.00
CCT6A	8906.00	8551.00	9852.00	9103.00	11110.00
CCT6B	18.00	27.00	18.00	21.00	43.00
CCT6P1	188.00	189.00	192.00	189.67	242.00
CCT6P3	57.00	48.00	83.00	62.67	52.00
CCT7	8514.00	8107.00	9108.00	8576.33	10739.00
CCT8	6098.00	5823.00	6655.00	6192.00	8842.00
CCZ1	478.00	423.00	470.00	457.00	595.00
CCZ1B	176.00	134.00	197.00	169.00	228.00
CD101	27.00	20.00	16.00	21.00	16.00
CD109	2390.00	2087.00	2557.00	2344.67	2039.00
CD14	7.00	10.00	13.00	10.00	5.00
CD151	8586.00	8749.00	8440.00	8591.67	8742.00
CD163L1	2780.00	2762.00	2972.00	2838.00	3249.00

CD164	7886.00	7076.00	7851.00	7604.33	8855.00
CD177	110.00	125.00	138.00	124.33	108.00
CD200	81.00	78.00	84.00	81.00	74.00
CD22	144.00	126.00	151.00	140.33	123.00
CD226	14.00	18.00	12.00	14.67	20.00
CD24	4.00	1.00	7.00	4.00	11.00
CD27	4.00	6.00	3.00	4.33	1.00
CD274	2777.00	2518.00	2666.00	2653.67	2195.00
CD276	1170.00	1104.00	1169.00	1147.67	1052.00
CD27-AS1	1366.00	1474.00	1533.00	1457.67	1654.00
CD2AP	801.00	709.00	879.00	796.33	869.00
CD2BP2	1753.00	1777.00	1881.00	1803.67	2054.00
CD302	27.00	25.00	18.00	23.33	28.00
CD320	1854.00	1939.00	1801.00	1864.67	2934.00
CD33	8.00	10.00	14.00	10.67	15.00
CD38	22.00	11.00	6.00	13.00	13.00
CD3EAP	746.00	738.00	801.00	761.67	801.00
CD44	24800.00	24398.00	24999.00	24732.33	29006.00
CD46	3869.00	3647.00	3792.00	3769.33	3529.00
CD47	2952.00	2749.00	2865.00	2855.33	3362.00
CD55	2419.00	2351.00	2435.00	2401.67	2393.00
CD58	394.00	350.00	298.00	347.33	305.00
CD59	21459.00	21158.00	20117.00	20911.33	26405.00
CD63	12546.00	13395.00	12117.00	12686.00	10008.00
CD68	1385.00	1435.00	1333.00	1384.33	1564.00
CD70	61.00	45.00	54.00	53.33	58.00
CD74	44.00	49.00	38.00	43.67	135.00
CD81	5442.00	5469.00	5026.00	5312.33	6162.00
CD81-AS1	5.00	5.00	3.00	4.33	3.00
CD82	128.00	132.00	123.00	127.67	83.00
CD83	178.00	205.00	198.00	193.67	423.00
CD9	1222.00	1250.00	1214.00	1228.67	1240.00
CD93	38.00	33.00	35.00	35.33	34.00
CD96	20.00	15.00	12.00	15.67	19.00
CD99	23.00	25.00	37.00	28.33	34.00
CD99L2	1493.00	1430.00	1347.00	1423.33	1430.00
CDA	240.00	261.00	293.00	264.67	231.00
CDADC1	154.00	128.00	155.00	145.67	159.00
CDAN1	811.00	787.00	835.00	811.00	673.00
CDC123	2945.00	2552.00	2825.00	2774.00	3628.00
CDC14A	188.00	172.00	189.00	183.00	160.00
CDC14B	385.00	391.00	405.00	393.67	416.00
CDC14C	4.00	2.00	2.00	2.67	1.00
CDC16	1607.00	1591.00	1729.00	1642.33	1828.00
CDC20	2333.00	2280.00	2214.00	2275.67	3130.00
CDC20B	13.00	9.00	9.00	10.33	18.00
CDC23	944.00	892.00	957.00	931.00	1025.00
CDC25A	292.00	291.00	279.00	287.33	454.00

CDC25B	1664.00	1627.00	1694.00	1661.67	1920.00
CDC25C	174.00	166.00	167.00	169.00	244.00
CDC26	164.00	149.00	196.00	169.67	190.00
CDC27	2914.00	2520.00	3060.00	2831.33	3071.00
CDC34	1177.00	1240.00	1280.00	1232.33	1474.00
CDC37	5333.00	5715.00	5842.00	5630.00	5770.00
CDC37L1	289.00	282.00	353.00	308.00	354.00
CDC37L1-AS1	15.00	13.00	8.00	12.00	17.00
CDC40	541.00	546.00	575.00	554.00	667.00
CDC42	2369.00	2172.00	2346.00	2295.67	2664.00
CDC42BPA	2203.00	2063.00	2375.00	2213.67	2136.00
CDC42BPB	3904.00	3686.00	4266.00	3952.00	3328.00
CDC42EP1	4500.00	4388.00	4392.00	4426.67	4541.00
CDC42EP2	1091.00	1062.00	1046.00	1066.33	907.00
CDC42EP3	6735.00	6034.00	6877.00	6548.67	6675.00
CDC42EP4	484.00	498.00	498.00	493.33	483.00
CDC42SE1	2082.00	2007.00	2194.00	2094.33	2158.00
CDC42SE2	587.00	571.00	646.00	601.33	698.00
CDC45	301.00	381.00	324.00	335.33	530.00
CDC5L	1700.00	1647.00	1875.00	1740.67	1914.00
CDC6	444.00	442.00	439.00	441.67	618.00
CDC7	770.00	808.00	802.00	793.33	1110.00
CDC73	974.00	791.00	1032.00	932.33	1030.00
CDCA2	315.00	272.00	255.00	280.67	346.00
CDCA3	456.00	431.00	400.00	429.00	618.00
CDCA4	732.00	750.00	725.00	735.67	1180.00
CDCA5	1193.00	1187.00	1354.00	1244.67	1798.00
CDCA7	1197.00	1223.00	1202.00	1207.33	1683.00
CDCA7L	1313.00	1297.00	1487.00	1365.67	1647.00
CDCA8	867.00	819.00	778.00	821.33	1126.00
CDCP1	55.00	60.00	59.00	58.00	42.00
CDH10	5.00	0.00	0.00	1.67	1.00
CDH12	269.00	236.00	309.00	271.33	230.00
CDH13	4931.00	4949.00	5242.00	5040.67	2502.00
CDH15	12.00	5.00	15.00	10.67	6.00
CDH18	78.00	58.00	108.00	81.33	68.00
CDH2	3934.00	3686.00	3989.00	3869.67	3318.00
CDH24	2124.00	2091.00	2267.00	2160.67	1014.00
CDH26	4.00	7.00	10.00	7.00	3.00
CDH5	3303.00	3329.00	3186.00	3272.67	1307.00
CDH6	47.00	44.00	48.00	46.33	30.00
CDHR3	172.00	157.00	235.00	188.00	112.00
CDIPT	1182.00	1119.00	1219.00	1173.33	1185.00
CDK1	1573.00	1405.00	1519.00	1499.00	1978.00
CDK10	2315.00	2463.00	2523.00	2433.67	2585.00
CDK11A	142.00	138.00	154.00	144.67	137.00
CDK11B	654.00	623.00	635.00	637.33	677.00
CDK12	2449.00	2322.00	2702.00	2491.00	2173.00

CDK13	1977.00	1836.00	2141.00	1984.67	1778.00
CDK14	395.00	339.00	438.00	390.67	457.00
CDK15	19.00	20.00	25.00	21.33	7.00
CDK16	2816.00	2861.00	2929.00	2868.67	3074.00
CDK17	2023.00	1805.00	2108.00	1978.67	2254.00
CDK18	47.00	38.00	37.00	40.67	37.00
CDK19	646.00	579.00	730.00	651.67	682.00
CDK2	782.00	732.00	774.00	762.67	1081.00
CDK20	88.00	93.00	94.00	91.67	98.00
CDK2AP1	761.00	737.00	781.00	759.67	922.00
CDK2AP2	520.00	415.00	465.00	466.67	504.00
CDK4	3752.00	3557.00	3785.00	3698.00	4647.00
CDK5	423.00	358.00	400.00	393.67	508.00
CDK5R1	174.00	179.00	171.00	174.67	202.00
CDK5R2	10.00	6.00	12.00	9.33	5.00
CDK5RAP1	1229.00	1183.00	1325.00	1245.67	1309.00
CDK5RAP2	1394.00	1239.00	1396.00	1343.00	1427.00
CDK5RAP3	5775.00	6305.00	6651.00	6243.67	6798.00
CDK6	3673.00	2995.00	3684.00	3450.67	3737.00
CDK7	7.00	11.00	9.00	9.00	2.00
CDK8	393.00	367.00	419.00	393.00	489.00
CDK9	889.00	819.00	853.00	853.67	1014.00
CDKAL1	367.00	370.00	382.00	373.00	378.00
CDKL1	60.00	66.00	54.00	60.00	85.00
CDKL2	10.00	8.00	19.00	12.33	14.00
CDKL3	93.00	85.00	90.00	89.33	91.00
CDKL4	4.00	4.00	6.00	4.67	9.00
CDKL5	17.00	21.00	26.00	21.33	8.00
CDKN1A	134.00	136.00	134.00	134.67	218.00
CDKN1B	632.00	632.00	769.00	677.67	550.00
CDKN2A	3818.00	3799.00	4266.00	3961.00	4614.00
CDKN2AIP	273.00	227.00	273.00	257.67	318.00
CDKN2AIPNL	462.00	449.00	532.00	481.00	702.00
CDKN2B	527.00	451.00	624.00	534.00	382.00
CDKN2B-AS1	132.00	112.00	123.00	122.33	76.00
CDKN2C	68.00	68.00	66.00	67.33	110.00
CDKN2D	70.00	80.00	68.00	72.67	92.00
CDKN3	308.00	260.00	334.00	300.67	543.00
CDNF	53.00	46.00	61.00	53.33	72.00
CDO1	155.00	118.00	149.00	140.67	187.00
CDON	113.00	113.00	118.00	114.67	140.00
CDPF1	350.00	366.00	397.00	371.00	473.00
CDR2	678.00	674.00	762.00	704.67	658.00
CDR2L	436.00	454.00	424.00	438.00	423.00
CDRT1	6.00	4.00	5.00	5.00	4.00
CDS1	13.00	14.00	7.00	11.33	6.00
CDS2	1601.00	1581.00	1535.00	1572.33	1337.00
CDT1	481.00	514.00	533.00	509.33	711.00

CDV3	5425.00	5176.00	5903.00	5501.33	6191.00
CDYL	861.00	853.00	950.00	888.00	1004.00
CDYL2	11.00	17.00	18.00	15.33	17.00
CEACAM19	532.00	586.00	652.00	590.00	471.00
CEBPB	1831.00	1656.00	1868.00	1785.00	2570.00
CEBPB-AS1	49.00	34.00	53.00	45.33	57.00
CEBPD	49.00	70.00	67.00	62.00	55.00
CEBPG	1694.00	1524.00	1853.00	1690.33	1939.00
CEBPZ	1911.00	1865.00	2088.00	1954.67	2256.00
CEBPZOS	914.00	833.00	973.00	906.67	1134.00
CECR5	757.00	856.00	880.00	831.00	1017.00
CECR5-AS1	5.00	10.00	8.00	7.67	8.00
CECR6	22.00	20.00	19.00	20.33	20.00
CECR7	249.00	227.00	286.00	254.00	240.00
CEL	22.00	34.00	24.00	26.67	19.00
CELF1	4222.00	4048.00	4543.00	4271.00	4292.00
CELF6	75.00	56.00	66.00	65.67	55.00
CELSR2	142.00	125.00	181.00	149.33	79.00
CELSR3	841.00	862.00	961.00	888.00	527.00
CELSR3-AS1	5.00	5.00	9.00	6.33	3.00
CEMIP	70.00	78.00	102.00	83.33	55.00
CENPA	269.00	297.00	312.00	292.67	338.00
CENPB	2714.00	2753.00	2875.00	2780.67	3050.00
CENPBD1	532.00	555.00	592.00	559.67	550.00
CENPBD1P1	441.00	401.00	428.00	423.33	441.00
CENPC	326.00	294.00	327.00	315.67	359.00
CENPE	915.00	881.00	932.00	909.33	1014.00
CENPF	3938.00	3882.00	3945.00	3921.67	4424.00
CENPH	349.00	365.00	376.00	363.33	471.00
CENPI	116.00	102.00	105.00	107.67	158.00
CENPJ	771.00	826.00	822.00	806.33	739.00
CENPK	453.00	345.00	406.00	401.33	538.00
CENPL	277.00	282.00	332.00	297.00	373.00
CENPM	112.00	92.00	96.00	100.00	161.00
CENPN	762.00	753.00	761.00	758.67	1038.00
CENPO	683.00	573.00	565.00	607.00	744.00
CENPP	114.00	93.00	80.00	95.67	138.00
CENPQ	248.00	262.00	282.00	264.00	356.00
CENPT	1098.00	1230.00	1148.00	1158.67	1297.00
CENPU	418.00	388.00	413.00	406.33	728.00
CENPV	285.00	267.00	316.00	289.33	347.00
CENPW	318.00	390.00	320.00	342.67	520.00
CEP104	1002.00	923.00	1056.00	993.67	943.00
CEP112	70.00	88.00	80.00	79.33	111.00
CEP120	974.00	905.00	1061.00	980.00	1108.00
CEP126	870.00	869.00	845.00	861.33	937.00
CEP128	255.00	249.00	257.00	253.67	263.00
CEP131	727.00	785.00	745.00	752.33	763.00

CEP135	505.00	502.00	592.00	533.00	588.00
CEP152	1640.00	1628.00	1682.00	1650.00	1381.00
CEP162	317.00	308.00	309.00	311.33	415.00
CEP164	2485.00	2487.00	2628.00	2533.33	2671.00
CEP170	15.00	14.00	13.00	14.00	17.00
CEP170B	2087.00	1998.00	2237.00	2107.33	1839.00
CEP170P1	26.00	16.00	24.00	22.00	23.00
CEP19	55.00	49.00	62.00	55.33	60.00
CEP192	924.00	902.00	1013.00	946.33	934.00
CEP250	3599.00	3810.00	4105.00	3838.00	2861.00
CEP290	1411.00	1369.00	1454.00	1411.33	1440.00
CEP295	655.00	622.00	696.00	657.67	652.00
CEP350	1239.00	1073.00	1255.00	1189.00	1078.00
CEP41	1129.00	1135.00	1076.00	1113.33	1207.00
CEP44	449.00	454.00	508.00	470.33	486.00
CEP55	922.00	854.00	904.00	893.33	1334.00
CEP57	3329.00	3139.00	3725.00	3397.67	3937.00
CEP57L1	288.00	244.00	255.00	262.33	302.00
CEP63	549.00	552.00	613.00	571.33	664.00
CEP68	477.00	421.00	410.00	436.00	430.00
CEP70	401.00	396.00	398.00	398.33	443.00
CEP72	149.00	131.00	169.00	149.67	175.00
CEP76	197.00	169.00	189.00	185.00	215.00
CEP78	415.00	442.00	374.00	410.33	459.00
CEP83	529.00	515.00	572.00	538.67	618.00
CEP83-AS1	24.00	28.00	33.00	28.33	25.00
CEP85	849.00	842.00	908.00	866.33	710.00
CEP85L	390.00	350.00	434.00	391.33	321.00
CEP89	263.00	237.00	259.00	253.00	212.00
CEP95	986.00	966.00	1052.00	1001.33	1011.00
CEP97	463.00	413.00	520.00	465.33	469.00
CEPT1	1209.00	1190.00	1271.00	1223.33	1253.00
CERCAM	3921.00	4025.00	3927.00	3957.67	4352.00
CERK	2100.00	1882.00	2150.00	2044.00	1902.00
CERS1	28.00	19.00	17.00	21.33	31.00
CERS2	5465.00	5265.00	5321.00	5350.33	5792.00
CERS5	1396.00	1534.00	1511.00	1480.33	1629.00
CERS6	2244.00	2121.00	2209.00	2191.33	2582.00
CES2	712.00	702.00	767.00	727.00	756.00
CETN2	532.00	455.00	482.00	489.67	497.00
CETN3	610.00	588.00	682.00	626.67	836.00
CETN4P	8.00	2.00	11.00	7.00	6.00
CFAP20	660.00	633.00	714.00	669.00	935.00
CFAP36	736.00	751.00	760.00	749.00	809.00
CFAP43	24.00	24.00	30.00	26.00	26.00
CFAP44	966.00	1005.00	1008.00	993.00	914.00
CFAP45	307.00	316.00	281.00	301.33	250.00
CFAP53	19.00	22.00	24.00	21.67	15.00

CFAP54	1108.00	1092.00	1189.00	1129.67	891.00
CFAP57	58.00	68.00	59.00	61.67	84.00
CFAP58	65.00	58.00	68.00	63.67	43.00
CFAP69	253.00	253.00	225.00	243.67	213.00
CFAP70	98.00	100.00	82.00	93.33	75.00
CFAP74	4.00	5.00	2.00	3.67	6.00
CFAP97	1237.00	1050.00	1413.00	1233.33	1294.00
CFDP1	891.00	917.00	992.00	933.33	1198.00
CFH	557.00	501.00	598.00	552.00	582.00
CFL1	16357.00	16038.00	16554.00	16316.33	22692.00
CFL1P1	22.00	31.00	36.00	29.67	22.00
CFL2	1434.00	1209.00	1437.00	1360.00	1293.00
CFLAR	1375.00	1406.00	1545.00	1442.00	1422.00
CFLAR-AS1	8.00	2.00	4.00	4.67	5.00
CFP	11.00	8.00	12.00	10.33	16.00
CGB	5.00	5.00	5.00	5.00	4.00
CGGBP1	2449.00	2346.00	2643.00	2479.33	2666.00
CGN	86.00	101.00	84.00	90.33	88.00
CGNL1	19.00	20.00	22.00	20.33	27.00
CGREF1	139.00	154.00	139.00	144.00	155.00
CGRRF1	193.00	176.00	211.00	193.33	226.00
CH17-340M24.3	19.00	26.00	18.00	21.00	26.00
CH25H	9.00	3.00	0.00	4.00	4.00
CHAC1	1180.00	1157.00	1248.00	1195.00	1399.00
CHAC2	104.00	122.00	112.00	112.67	132.00
CHAF1A	651.00	783.00	726.00	720.00	844.00
CHAF1B	178.00	221.00	212.00	203.67	336.00
CHAMP1	746.00	665.00	717.00	709.33	707.00
CHCHD1	585.00	669.00	737.00	663.67	894.00
CHCHD2	4042.00	3914.00	4236.00	4064.00	5380.00
CHCHD3	1718.00	1576.00	1794.00	1696.00	2253.00
CHCHD4	421.00	389.00	427.00	412.33	574.00
CHCHD5	179.00	202.00	218.00	199.67	308.00
CHCHD6	241.00	246.00	299.00	262.00	334.00
CHCHD7	603.00	675.00	657.00	645.00	737.00
CHD1	1922.00	1895.00	2209.00	2008.67	1873.00
CHD1L	2052.00	1940.00	2164.00	2052.00	2361.00
CHD2	3394.00	3336.00	3702.00	3477.33	2762.00
CHD3	5676.00	5638.00	6117.00	5810.33	6115.00
CHD4	7802.00	7204.00	8068.00	7691.33	6491.00
CHD5	5.00	6.00	14.00	8.33	7.00
CHD6	1866.00	1760.00	1959.00	1861.67	1398.00
CHD7	1825.00	1799.00	1964.00	1862.67	1774.00
CHD8	2616.00	2509.00	2585.00	2570.00	2158.00
CHD9	1718.00	1553.00	1840.00	1703.67	1528.00
CHEK1	329.00	306.00	341.00	325.33	486.00
CHEK2	495.00	484.00	512.00	497.00	747.00
CHERP	1888.00	1810.00	1967.00	1888.33	1863.00

CHFR	485.00	393.00	532.00	470.00	634.00
CHGB	18.00	15.00	25.00	19.33	44.00
CHIC1	522.00	436.00	525.00	494.33	499.00
CHIC2	289.00	257.00	351.00	299.00	346.00
CHID1	1732.00	1899.00	1802.00	1811.00	1948.00
CHKA	711.00	679.00	727.00	705.67	716.00
CHKB	23.00	14.00	15.00	17.33	20.00
CHKB-AS1	32.00	27.00	25.00	28.00	21.00
CHKB-CPT1B	21.00	21.00	29.00	23.67	22.00
CHL1	7.00	4.00	5.00	5.33	5.00
CHM	1019.00	902.00	1109.00	1010.00	1070.00
CHML	903.00	850.00	1006.00	919.67	832.00
CHMP1A	1727.00	1574.00	1698.00	1666.33	1936.00
CHMP1B	1214.00	1137.00	1286.00	1212.33	1391.00
CHMP1B2P	333.00	301.00	355.00	329.67	321.00
CHMP2A	994.00	889.00	967.00	950.00	1170.00
CHMP2B	672.00	648.00	749.00	689.67	808.00
CHMP3	75.00	77.00	97.00	83.00	112.00
CHMP4A	645.00	652.00	641.00	646.00	783.00
CHMP4B	1848.00	1841.00	1986.00	1891.67	1968.00
CHMP5	1309.00	1318.00	1435.00	1354.00	1869.00
CHMP6	262.00	273.00	234.00	256.33	240.00
CHMP7	798.00	730.00	820.00	782.67	998.00
CHN1	337.00	316.00	356.00	336.33	260.00
CHN2	37.00	29.00	34.00	33.33	28.00
CHORDC1	2924.00	2823.00	3393.00	3046.67	3567.00
CHP1	1019.00	1008.00	1143.00	1056.67	968.00
CHPF	4802.00	4975.00	4860.00	4879.00	3864.00
CHPF2	3910.00	4008.00	4123.00	4013.67	2988.00
CHPT1	843.00	847.00	852.00	847.33	1078.00
CHRAC1	1192.00	1098.00	1251.00	1180.33	1379.00
CHRDL1	2161.00	2088.00	2229.00	2159.33	2274.00
CHRFAM7A	3.00	5.00	4.00	4.00	3.00
CHRM3	30.00	15.00	33.00	26.00	21.00
CHRM3-AS2	76.00	73.00	104.00	84.33	53.00
CHRNA1	1261.00	1077.00	1150.00	1162.67	1076.00
CHRNA10	53.00	64.00	64.00	60.33	57.00
CHRNA5	157.00	129.00	156.00	147.33	202.00
CHRNA9	4.00	6.00	6.00	5.33	2.00
CHRNB1	40.00	47.00	45.00	44.00	48.00
CHRNE	26.00	28.00	30.00	28.00	28.00
CHST10	849.00	889.00	902.00	880.00	1025.00
CHST11	2273.00	2090.00	2149.00	2170.67	2080.00
CHST12	342.00	364.00	349.00	351.67	266.00
CHST14	1086.00	1111.00	1060.00	1085.67	968.00
CHST2	673.00	754.00	664.00	697.00	603.00
CHST3	34.00	35.00	20.00	29.67	26.00
CHST6	5.00	3.00	7.00	5.00	9.00

CHST7	174.00	179.00	174.00	175.67	198.00
CHSY1	889.00	959.00	1012.00	953.33	996.00
CHSY3	511.00	509.00	508.00	509.33	514.00
CHTF18	1371.00	1414.00	1402.00	1395.67	1528.00
CHTF8	2050.00	1993.00	2262.00	2101.67	2238.00
CHTOP	2363.00	2321.00	2547.00	2410.33	2855.00
CHUK	900.00	814.00	936.00	883.33	813.00
CHURC1	716.00	616.00	697.00	676.33	764.00
CIAO1	2554.00	2504.00	2754.00	2604.00	2963.00
CIAPIN1	1194.00	1140.00	1175.00	1169.67	1429.00
CIART	220.00	244.00	234.00	232.67	415.00
CIB1	1192.00	1203.00	1309.00	1234.67	1343.00
CIC	1455.00	1435.00	1530.00	1473.33	1194.00
CIDEA	3.00	5.00	3.00	3.67	11.00
CIDECP	137.00	117.00	111.00	121.67	155.00
CIITA	39.00	25.00	50.00	38.00	69.00
CILP	4.00	0.00	0.00	1.33	0.00
CILP2	3.00	12.00	8.00	7.67	11.00
CINP	285.00	243.00	281.00	269.67	334.00
CIPC	737.00	651.00	819.00	735.67	921.00
CIR1	497.00	505.00	612.00	538.00	601.00
CIRBP	4546.00	4799.00	4865.00	4736.67	5445.00
CIRBP-AS1	18.00	19.00	24.00	20.33	38.00
CIRH1A	469.00	473.00	517.00	486.33	465.00
CISD1	703.00	596.00	707.00	668.67	815.00
CISD2	400.00	390.00	376.00	388.67	475.00
CISH	9.00	5.00	2.00	5.33	9.00
CIT	1582.00	1513.00	1634.00	1576.33	1647.00
CITED1	7.00	8.00	8.00	7.67	10.00
CITED2	1323.00	1173.00	1353.00	1283.00	1707.00
CITED4	23.00	31.00	23.00	25.67	50.00
CIZ1	3448.00	3498.00	3709.00	3551.67	3396.00
CKAP2	1108.00	999.00	1125.00	1077.33	1363.00
CKAP2L	1123.00	1064.00	1084.00	1090.33	1259.00
CKAP4	10190.00	10066.00	10052.00	10102.67	8798.00
CKAP5	5080.00	4527.00	5139.00	4915.33	4666.00
CKB	12.00	16.00	24.00	17.33	5.00
CKLF	248.00	257.00	208.00	237.67	311.00
CKMT2-AS1	282.00	315.00	315.00	304.00	331.00
CKS1B	650.00	590.00	649.00	629.67	987.00
CKS2	672.00	663.00	735.00	690.00	1247.00
CLASP1	1743.00	1534.00	1773.00	1683.33	1542.00
CLASP2	1163.00	1024.00	1165.00	1117.33	1097.00
CLASRP	1186.00	1335.00	1279.00	1266.67	1257.00
CLCA3P	8.00	6.00	5.00	6.33	20.00
CLCC1	1570.00	1447.00	1524.00	1513.67	1818.00
CLCF1	331.00	324.00	332.00	329.00	173.00
CLCN2	305.00	346.00	297.00	316.00	289.00

CLCN3	2026.00	1898.00	2185.00	2036.33	1889.00
CLCN4	468.00	472.00	496.00	478.67	331.00
CLCN5	591.00	533.00	610.00	578.00	571.00
CLCN6	1278.00	1356.00	1406.00	1346.67	1041.00
CLCN7	4665.00	4777.00	4963.00	4801.67	4587.00
CLDN1	418.00	389.00	375.00	394.00	1020.00
CLDN11	688.00	630.00	633.00	650.33	787.00
CLDN12	3584.00	3269.00	3749.00	3534.00	3990.00
CLDN14	94.00	79.00	88.00	87.00	122.00
CLDN15	1132.00	1206.00	1242.00	1193.33	945.00
CLDN16	6.00	1.00	1.00	2.67	10.00
CLDN23	7.00	7.00	16.00	10.00	3.00
CLDN4	15.00	12.00	8.00	11.67	16.00
CLDN7	10.00	13.00	5.00	9.33	10.00
CLDN9	4.00	5.00	6.00	5.00	3.00
CLDND1	2422.00	2302.00	2441.00	2388.33	2546.00
CLDND2	7.00	6.00	10.00	7.67	10.00
CLEC11A	2704.00	3164.00	3060.00	2976.00	3763.00
CLEC12B	16.00	4.00	12.00	10.67	12.00
CLEC16A	741.00	756.00	819.00	772.00	702.00
CLEC18A	5.00	11.00	7.00	7.67	0.00
CLEC18B	3.00	6.00	3.00	4.00	2.00
CLEC2B	274.00	272.00	280.00	275.33	324.00
CLEC2D	902.00	909.00	992.00	934.33	805.00
CLEC4A	14.00	11.00	8.00	11.00	23.00
CLEC4GP1	14.00	15.00	9.00	12.67	39.00
CLEC4M	3.00	5.00	0.00	2.67	4.00
CLEC5A	5.00	2.00	2.00	3.00	1.00
CLGN	630.00	607.00	694.00	643.67	825.00
CLHC1	434.00	436.00	424.00	431.33	446.00
CLIC1	5.00	8.00	2.00	5.00	2.00
CLIC2	28.00	20.00	21.00	23.00	29.00
CLIC4	6085.00	5068.00	6657.00	5936.67	7056.00
CLINT1	2228.00	2031.00	2335.00	2198.00	2228.00
CLIP1	2442.00	2181.00	2622.00	2415.00	2107.00
CLIP2	1367.00	1285.00	1513.00	1388.33	971.00
CLIP3	84.00	65.00	74.00	74.33	38.00
CLIP4	2494.00	2292.00	2842.00	2542.67	2539.00
CLK1	2195.00	2144.00	2558.00	2299.00	2462.00
CLK2	4.00	1.00	3.00	2.67	0.00
CLK2P1	14.00	10.00	14.00	12.67	17.00
CLK3	1183.00	1193.00	1226.00	1200.67	1211.00
CLK4	478.00	436.00	499.00	471.00	506.00
CLMN	1674.00	1600.00	1831.00	1701.67	1227.00
CLMP	2458.00	2228.00	2457.00	2381.00	2187.00
CLN3	1112.00	1151.00	1099.00	1120.67	1100.00
CLN5	922.00	921.00	882.00	908.33	1000.00
CLN6	906.00	881.00	911.00	899.33	1001.00

CLNS1A	2143.00	2025.00	2105.00	2091.00	2692.00
CLOCK	637.00	598.00	698.00	644.33	603.00
CLP1	342.00	335.00	299.00	325.33	420.00
CLPB	475.00	485.00	490.00	483.33	472.00
CLPP	664.00	647.00	710.00	673.67	897.00
CLPTM1	2522.00	2557.00	2399.00	2492.67	2410.00
CLPTM1L	864.00	934.00	855.00	884.33	1056.00
CLPX	748.00	737.00	816.00	767.00	858.00
CLSPN	294.00	298.00	272.00	288.00	423.00
CLSTN1	6424.00	6291.00	6532.00	6415.67	5405.00
CLSTN2	3.00	4.00	22.00	9.67	8.00
CLSTN3	1176.00	1241.00	1311.00	1242.67	1058.00
CLTA	2670.00	2663.00	2753.00	2695.33	3430.00
CLTB	618.00	579.00	652.00	616.33	777.00
CLTC	13452.00	11914.00	14068.00	13144.67	13305.00
CLTCL1	387.00	379.00	399.00	388.33	227.00
CLU	118.00	100.00	103.00	107.00	85.00
CLUAP1	519.00	508.00	555.00	527.33	652.00
CLUH	4891.00	4863.00	5176.00	4976.67	4952.00
CLUHP3	11.00	8.00	10.00	9.67	7.00
CLVS2	39.00	40.00	42.00	40.33	37.00
CLYBL	57.00	59.00	57.00	57.67	58.00
CMAHP	22.00	22.00	20.00	21.33	23.00
CMAS	1083.00	1019.00	1169.00	1090.33	1317.00
CMBL	70.00	83.00	62.00	71.67	113.00
CMC1	488.00	541.00	513.00	514.00	702.00
CMC2	448.00	481.00	433.00	454.00	655.00
CMC4	95.00	90.00	103.00	96.00	133.00
CMIP	2091.00	1967.00	2249.00	2102.33	2520.00
CMPK1	1842.00	1609.00	1940.00	1797.00	2209.00
CMSS1	1577.00	1548.00	1626.00	1583.67	2135.00
CMTM1	43.00	34.00	43.00	40.00	50.00
CMTM3	1050.00	1074.00	1067.00	1063.67	1010.00
CMTM4	955.00	1012.00	1116.00	1027.67	870.00
CMTM6	2550.00	2304.00	2308.00	2387.33	2566.00
CMTM7	1500.00	1550.00	1446.00	1498.67	1659.00
CMTM8	38.00	37.00	45.00	40.00	43.00
CMTR1	2241.00	2134.00	2253.00	2209.33	2016.00
CMTR2	1389.00	1136.00	1347.00	1290.67	1254.00
CMYA5	17.00	21.00	21.00	19.67	11.00
CNBP	7713.00	7585.00	8434.00	7910.67	10057.00
CNDP2	1904.00	1897.00	2021.00	1940.67	2122.00
CNEP1R1	370.00	336.00	399.00	368.33	444.00
CNGA1	45.00	39.00	47.00	43.67	23.00
CNGA4	25.00	22.00	19.00	22.00	18.00
CNIH1	2148.00	2029.00	2039.00	2072.00	2686.00
CNIH2	13.00	7.00	17.00	12.33	10.00
CNIH3	272.00	287.00	333.00	297.33	200.00

CNIH4	1006.00	986.00	987.00	993.00	1332.00
CNKSR1	62.00	82.00	62.00	68.67	51.00
CNKSR2	60.00	51.00	75.00	62.00	71.00
CNKSR3	215.00	208.00	209.00	210.67	219.00
CNN1	5.00	5.00	3.00	4.33	1.00
CNN2	828.00	802.00	890.00	840.00	851.00
CNN3	3916.00	3818.00	4078.00	3937.33	5816.00
CNNM1	164.00	197.00	172.00	177.67	158.00
CNNM2	327.00	350.00	369.00	348.67	322.00
CNNM3	746.00	718.00	817.00	760.33	709.00
CNNM4	564.00	543.00	574.00	560.33	510.00
CNOT1	5330.00	4640.00	5584.00	5184.67	4724.00
CNOT10	779.00	688.00	771.00	746.00	930.00
CNOT11	1582.00	1541.00	1602.00	1575.00	1754.00
CNOT2	1427.00	1266.00	1432.00	1375.00	1529.00
CNOT3	69.00	73.00	95.00	79.00	56.00
CNOT4	649.00	607.00	730.00	662.00	670.00
CNOT6	1136.00	979.00	1252.00	1122.33	1260.00
CNOT6L	405.00	390.00	421.00	405.33	421.00
CNOT7	1087.00	1003.00	1216.00	1102.00	1303.00
CNOT8	630.00	694.00	772.00	698.67	778.00
CNP	1010.00	949.00	979.00	979.33	1137.00
CNPPD1	1229.00	1142.00	1164.00	1178.33	1470.00
CNPY2	2588.00	2702.00	2633.00	2641.00	3059.00
CNPY3	1307.00	1346.00	1309.00	1320.67	1518.00
CNPY4	250.00	261.00	245.00	252.00	232.00
CNR1	14.00	22.00	23.00	19.67	5.00
CNRIP1	1063.00	1040.00	1008.00	1037.00	1404.00
CNST	1391.00	1287.00	1399.00	1359.00	1733.00
CNTF	4.00	0.00	3.00	2.33	2.00
CNTLN	445.00	393.00	450.00	429.33	439.00
CNTN1	28.00	24.00	29.00	27.00	34.00
CNTN5	122.00	101.00	108.00	110.33	67.00
CNTNAP1	1596.00	1656.00	1694.00	1648.67	1707.00
CNTNAP3	124.00	145.00	157.00	142.00	99.00
CNTNAP3B	190.00	184.00	213.00	195.67	171.00
CNTNAP3P2	81.00	66.00	82.00	76.33	49.00
CNTRL	592.00	502.00	593.00	562.33	744.00
CNTROB	1733.00	1803.00	1855.00	1797.00	1891.00
COA1	2435.00	2354.00	2449.00	2412.67	2620.00
COA3	218.00	224.00	211.00	217.67	367.00
COA4	832.00	795.00	807.00	811.33	1069.00
COA5	613.00	572.00	700.00	628.33	745.00
COA6	717.00	745.00	761.00	741.00	1024.00
COA7	593.00	583.00	697.00	624.33	677.00
COASY	2639.00	2497.00	2547.00	2561.00	2645.00
COBLL1	150.00	155.00	149.00	151.33	158.00
COCH	26.00	22.00	25.00	24.33	22.00

COG1	830.00	860.00	899.00	863.00	673.00
COG2	647.00	605.00	734.00	662.00	723.00
COG3	941.00	913.00	1048.00	967.33	923.00
COG4	1634.00	1548.00	1659.00	1613.67	1627.00
COG5	1527.00	1347.00	1608.00	1494.00	1663.00
COG6	577.00	452.00	594.00	541.00	587.00
COG7	483.00	500.00	580.00	521.00	576.00
COG8	429.00	366.00	427.00	407.33	350.00
COIL	619.00	579.00	707.00	635.00	735.00
COL10A1	13.00	10.00	9.00	10.67	21.00
COL11A1	13.00	8.00	9.00	10.00	3.00
COL12A1	7061.00	6429.00	7238.00	6909.33	2674.00
COL13A1	7480.00	7946.00	7291.00	7572.33	4528.00
COL16A1	1025.00	1083.00	1083.00	1063.67	1200.00
COL17A1	193.00	213.00	222.00	209.33	160.00
COL18A1	3226.00	3247.00	3254.00	3242.33	3163.00
COL19A1	26.00	34.00	34.00	31.33	25.00
COL1A1	22824.00	22238.00	21503.00	22188.33	12853.00
COL1A2	7.00	0.00	1.00	2.67	1.00
COL21A1	13.00	17.00	22.00	17.33	3.00
COL24A1	4.00	10.00	4.00	6.00	3.00
COL25A1	55.00	58.00	67.00	60.00	72.00
COL27A1	4131.00	4065.00	4231.00	4142.33	4191.00
COL28A1	5.00	6.00	5.00	5.33	1.00
COL4A1	2921.00	2918.00	2936.00	2925.00	1968.00
COL4A2	5624.00	5954.00	5831.00	5803.00	4412.00
COL4A3	3.00	0.00	2.00	1.67	0.00
COL4A3BP	515.00	525.00	593.00	544.33	501.00
COL4A4	13.00	6.00	10.00	9.67	8.00
COL4A5	2474.00	2417.00	2729.00	2540.00	1967.00
COL4A6	1605.00	1525.00	1702.00	1610.67	1077.00
COL5A1	3715.00	3847.00	4017.00	3859.67	1592.00
COL5A2	1921.00	1930.00	1920.00	1923.67	2107.00
COL6A1	16283.00	17183.00	17365.00	16943.67	18208.00
COL6A2	32979.00	35177.00	34474.00	34210.00	34640.00
COL6A3	15337.00	15031.00	16065.00	15477.67	9061.00
COL7A1	291.00	361.00	391.00	347.67	219.00
COL8A1	24.00	28.00	23.00	25.00	8.00
COL9A2	39.00	46.00	45.00	43.33	49.00
COL9A3	122.00	125.00	147.00	131.33	157.00
COLEC12	13442.00	13205.00	13785.00	13477.33	14791.00
COLGALT1	2672.00	2676.00	2599.00	2649.00	2649.00
COLGALT2	350.00	319.00	394.00	354.33	383.00
COLQ	44.00	30.00	46.00	40.00	27.00
COMMD1	184.00	198.00	201.00	194.33	213.00
COMMD10	319.00	337.00	380.00	345.33	404.00
COMMD2	616.00	495.00	640.00	583.67	669.00
COMMD3	490.00	487.00	550.00	509.00	439.00

COMMD4	1177.00	1182.00	1198.00	1185.67	1583.00
COMMD5	446.00	485.00	483.00	471.33	509.00
COMMD6	933.00	985.00	1078.00	998.67	1325.00
COMMD7	597.00	597.00	678.00	624.00	611.00
COMMD8	306.00	239.00	317.00	287.33	389.00
COMMD9	392.00	368.00	370.00	376.67	495.00
COMT	3953.00	3989.00	3971.00	3971.00	4747.00
COMTD1	168.00	169.00	183.00	173.33	199.00
COPA	7984.00	7374.00	8247.00	7868.33	7137.00
COPB1	5079.00	4763.00	5353.00	5065.00	5493.00
COPB2	4489.00	4151.00	4768.00	4469.33	4550.00
COPE	1835.00	1819.00	1915.00	1856.33	2221.00
COPG1	4123.00	3841.00	4350.00	4104.67	4148.00
COPG2	1613.00	1476.00	1625.00	1571.33	1782.00
COPRS	616.00	676.00	747.00	679.67	1046.00
COPS2	1353.00	1225.00	1474.00	1350.67	1642.00
COPS3	1338.00	1321.00	1346.00	1335.00	1867.00
COPS4	543.00	496.00	534.00	524.33	591.00
COPS5	1486.00	1515.00	1599.00	1533.33	1972.00
COPS6	2357.00	2137.00	2432.00	2308.67	3092.00
COPS7A	1949.00	1819.00	2018.00	1928.67	2126.00
COPS7B	1025.00	1025.00	1096.00	1048.67	1110.00
COPS8	1388.00	1178.00	1329.00	1298.33	1514.00
COPZ1	1968.00	1838.00	2130.00	1978.67	2370.00
COPZ2	323.00	322.00	330.00	325.00	334.00
COQ10A	85.00	96.00	121.00	100.67	123.00
COQ10B	573.00	503.00	642.00	572.67	767.00
COQ2	301.00	297.00	345.00	314.33	427.00
COQ3	142.00	132.00	139.00	137.67	236.00
COQ4	959.00	960.00	1001.00	973.33	1109.00
COQ5	513.00	565.00	574.00	550.67	717.00
COQ6	449.00	449.00	383.00	427.00	477.00
COQ7	271.00	255.00	282.00	269.33	329.00
COQ9	1038.00	998.00	1100.00	1045.33	1169.00
CORIN	15.00	8.00	6.00	9.67	11.00
CORO1A	28.00	27.00	21.00	25.33	25.00
CORO1B	2129.00	2282.00	2276.00	2229.00	2516.00
CORO1C	4941.00	4657.00	5269.00	4955.67	5536.00
CORO2A	34.00	27.00	38.00	33.00	46.00
CORO2B	537.00	557.00	530.00	541.33	242.00
CORO6	20.00	23.00	25.00	22.67	18.00
COTL1	7010.00	6708.00	7017.00	6911.67	10063.00
COX10	266.00	271.00	296.00	277.67	314.00
COX10-AS1	292.00	258.00	273.00	274.33	232.00
COX11	701.00	567.00	809.00	692.33	738.00
COX14	166.00	210.00	185.00	187.00	254.00
COX15	1097.00	1071.00	1114.00	1094.00	1276.00
COX16	168.00	162.00	157.00	162.33	239.00

COX17	303.00	340.00	357.00	333.33	494.00
COX18	233.00	244.00	276.00	251.00	282.00
COX19	594.00	664.00	656.00	638.00	705.00
COX20	416.00	402.00	439.00	419.00	480.00
COX4I1	5300.00	5278.00	5573.00	5383.67	7050.00
COX5A	1344.00	1358.00	1357.00	1353.00	1866.00
COX5B	1744.00	1828.00	1804.00	1792.00	2415.00
COX6A1	2601.00	2599.00	2569.00	2589.67	1511.00
COX6B1	1024.00	1029.00	1128.00	1060.33	1429.00
COX6B2	3.00	1.00	0.00	1.33	1.00
COX6C	1823.00	1830.00	1763.00	1805.33	2499.00
COX7A2	1365.00	1402.00	1459.00	1408.67	2031.00
COX7A2L	1796.00	1761.00	2004.00	1853.67	2370.00
COX7B	1312.00	1319.00	1444.00	1358.33	1991.00
COX7C	3485.00	3582.00	3664.00	3577.00	4840.00
COX8A	3262.00	3172.00	3198.00	3210.67	4285.00
CPA4	25657.00	25375.00	27258.00	26096.67	10917.00
CPA5	11.00	10.00	16.00	12.33	3.00
CPA6	4.00	7.00	3.00	4.67	4.00
CPB2-AS1	10.00	9.00	8.00	9.00	7.00
CPD	3147.00	2759.00	3233.00	3046.33	2518.00
CPE	37.00	37.00	30.00	34.67	47.00
CPEB1	11.00	15.00	24.00	16.67	11.00
CPEB1-AS1	11.00	3.00	6.00	6.67	6.00
CPEB2	319.00	258.00	382.00	319.67	296.00
CPEB2-AS1	4.00	4.00	1.00	3.00	0.00
CPEB3	27.00	31.00	31.00	29.67	36.00
CPEB4	318.00	261.00	338.00	305.67	224.00
CPED1	1012.00	925.00	1079.00	1005.33	1296.00
CPLX3	5.00	1.00	3.00	3.00	5.00
CPM	290.00	283.00	337.00	303.33	218.00
CPNE1	4916.00	4676.00	5101.00	4897.67	5527.00
CPNE2	302.00	315.00	307.00	308.00	407.00
CPNE3	2330.00	1948.00	2398.00	2225.33	2076.00
CPNE4	50.00	35.00	41.00	42.00	67.00
CPNE5	27.00	23.00	33.00	27.67	19.00
CPNE7	65.00	64.00	90.00	73.00	35.00
CPNE8	745.00	692.00	801.00	746.00	977.00
CPOX	940.00	1015.00	1080.00	1011.67	1116.00
CPQ	393.00	382.00	415.00	396.67	332.00
CPS1	728.00	687.00	713.00	709.33	695.00
CPSF1	3224.00	3389.00	3605.00	3406.00	3530.00
CPSF2	2231.00	1992.00	2374.00	2199.00	2501.00
CPSF3	1115.00	1143.00	1291.00	1183.00	1510.00
CPSF3L	2267.00	2374.00	2421.00	2354.00	2486.00
CPSF4	985.00	1015.00	990.00	996.67	1248.00
CPSF4L	3.00	1.00	3.00	2.33	1.00
CPSF6	3148.00	2981.00	3490.00	3206.33	3460.00

CPSF7	2677.00	2671.00	2746.00	2698.00	2603.00
CPT1A	43.00	36.00	36.00	38.33	61.00
CPT1B	75.00	69.00	69.00	71.00	53.00
CPT1C	42.00	25.00	30.00	32.33	36.00
CPT2	453.00	443.00	461.00	452.33	596.00
CPTP	441.00	427.00	438.00	435.33	616.00
CPVL	694.00	652.00	661.00	669.00	584.00
CR2	16.00	12.00	14.00	14.00	12.00
CRABP2	5.00	7.00	5.00	5.67	22.00
CRACR2A	503.00	396.00	507.00	468.67	282.00
CRADD	275.00	270.00	301.00	282.00	375.00
CRAMP1L	1540.00	1562.00	1696.00	1599.33	1493.00
CRAT	636.00	588.00	662.00	628.67	562.00
CRBN	571.00	586.00	648.00	601.67	651.00
CRCP	891.00	846.00	938.00	891.67	928.00
CREB1	1358.00	1251.00	1471.00	1360.00	1500.00
CREB3	597.00	648.00	641.00	628.67	619.00
CREB3L1	841.00	823.00	801.00	821.67	375.00
CREB3L2	8963.00	8046.00	8816.00	8608.33	7975.00
CREB3L4	412.00	431.00	401.00	414.67	476.00
CREB5	1079.00	1040.00	1157.00	1092.00	844.00
CREBBP	2158.00	1840.00	2210.00	2069.33	1700.00
CREBL2	814.00	719.00	781.00	771.33	894.00
CREBRF	577.00	506.00	759.00	614.00	491.00
CREBZF	2439.00	2377.00	2676.00	2497.33	2265.00
CREG1	317.00	311.00	299.00	309.00	394.00
CREG2	7.00	5.00	6.00	6.00	21.00
CRELD1	729.00	779.00	750.00	752.67	677.00
CRELD2	666.00	734.00	678.00	692.67	734.00
CREM	699.00	687.00	762.00	716.00	867.00
CRHBP	5.00	1.00	5.00	3.67	2.00
CRHR1-IT1	64.00	37.00	91.00	64.00	41.00
CRIM1	8369.00	7542.00	8554.00	8155.00	5781.00
CRIP1	5.00	0.00	1.00	2.00	0.00
CRIP2	37.00	53.00	48.00	46.00	54.00
CRIPAK	386.00	381.00	386.00	384.33	338.00
CRIPT	261.00	239.00	307.00	269.00	306.00
CRISPLD1	313.00	300.00	299.00	304.00	284.00
CRISPLD2	190.00	178.00	235.00	201.00	60.00
CRK	112.00	66.00	95.00	91.00	72.00
CRKL	1595.00	1427.00	1613.00	1545.00	1697.00
CRLF3	440.00	351.00	433.00	408.00	541.00
CRLS1	833.00	849.00	902.00	861.33	1103.00
CRMP1	3.00	1.00	1.00	1.67	2.00
CRNDE	482.00	488.00	513.00	494.33	470.00
CRNKL1	826.00	844.00	954.00	874.67	1133.00
CROCC	1156.00	1282.00	1322.00	1253.33	1075.00
CROCCP2	790.00	712.00	802.00	768.00	832.00

CROCCP3	317.00	353.00	345.00	338.33	306.00
CROT	340.00	329.00	333.00	334.00	295.00
CRSP8P	54.00	55.00	59.00	56.00	47.00
CRTAP	8962.00	8902.00	8929.00	8931.00	8979.00
CRTC1	476.00	494.00	507.00	492.33	419.00
CRTC2	1462.00	1492.00	1575.00	1509.67	1435.00
CRTC3	818.00	707.00	890.00	805.00	758.00
CRTC3-AS1	6.00	2.00	6.00	4.67	5.00
CRY1	835.00	795.00	838.00	822.67	879.00
CRY2	312.00	283.00	328.00	307.67	305.00
CRYBB2P1	267.00	272.00	258.00	265.67	282.00
CRYBB3	9.00	8.00	3.00	6.67	4.00
CRYBG3	1058.00	965.00	1086.00	1036.33	966.00
CRYGS	86.00	134.00	99.00	106.33	109.00
CRYL1	471.00	379.00	421.00	423.67	597.00
CRYM	7.00	5.00	10.00	7.33	6.00
CRYM-AS1	36.00	16.00	30.00	27.33	21.00
CRYZ	625.00	604.00	715.00	648.00	753.00
CRYZL1	406.00	354.00	409.00	389.67	393.00
CS	3883.00	3748.00	3949.00	3860.00	4259.00
CSAD	402.00	430.00	473.00	435.00	597.00
CSAG1	22.00	37.00	41.00	33.33	43.00
CSAG3	7.00	11.00	7.00	8.33	17.00
CSDE1	11189.00	9979.00	12109.00	11092.33	11342.00
CSE1L	5335.00	4973.00	5570.00	5292.67	6462.00
CSF1	314.00	299.00	282.00	298.33	165.00
CSF1R	3.00	2.00	4.00	3.00	2.00
CSF2	5.00	7.00	1.00	4.33	1.00
CSF3	210.00	246.00	230.00	228.67	312.00
CSGALNACT1	3.00	3.00	0.00	2.00	1.00
CSGALNACT2	1399.00	1284.00	1450.00	1377.67	1271.00
CSK	1785.00	1838.00	1857.00	1826.67	2227.00
CSMD2	17.00	11.00	12.00	13.33	13.00
CSMD3	26.00	26.00	32.00	28.00	74.00
CSNK1A1	3923.00	3433.00	3986.00	3780.67	4790.00
CSNK1D	5208.00	5232.00	5512.00	5317.33	4930.00
CSNK1E	143.00	122.00	144.00	136.33	115.00
CSNK1G1	837.00	752.00	799.00	796.00	549.00
CSNK1G2	1826.00	1778.00	1904.00	1836.00	1936.00
CSNK1G3	1121.00	967.00	1230.00	1106.00	1357.00
CSNK2A1	3509.00	3321.00	3734.00	3521.33	3967.00
CSNK2A2	986.00	875.00	1008.00	956.33	1004.00
CSNK2A3	453.00	391.00	472.00	438.67	469.00
CSPG4	639.00	647.00	635.00	640.33	218.00
CSPG5	7.00	3.00	14.00	8.00	3.00
CSPP1	817.00	750.00	878.00	815.00	856.00
CSRNP1	401.00	387.00	464.00	417.33	284.00
CSRNP2	634.00	602.00	787.00	674.33	814.00

CSRP1	7043.00	6707.00	6972.00	6907.33	9970.00
CSRP2	73.00	65.00	74.00	70.67	117.00
CSRP2BP	569.00	537.00	639.00	581.67	644.00
CST3	2053.00	2115.00	2088.00	2085.33	2418.00
CSTA	15.00	16.00	8.00	13.00	15.00
CSTB	1753.00	1648.00	1721.00	1707.33	2124.00
CSTF1	942.00	885.00	1058.00	961.67	1126.00
CSTF2	465.00	488.00	510.00	487.67	630.00
CSTF2T	1257.00	1177.00	1285.00	1239.67	1461.00
CSTF3	1924.00	1768.00	1884.00	1858.67	1739.00
CSTF3-AS1	10.00	11.00	11.00	10.67	16.00
CTAGE1	6.00	11.00	10.00	9.00	7.00
CTAGE11P	5.00	2.00	6.00	4.33	10.00
CTAGE5	1164.00	1145.00	1159.00	1156.00	1251.00
CTAGE6	4.00	0.00	0.00	1.33	2.00
CTB-12O2.1	3.00	5.00	1.00	3.00	3.00
CTB-178M22.2	7.00	4.00	5.00	5.33	9.00
CTBP1	1874.00	1962.00	2084.00	1973.33	2283.00
CTBP1-AS2	165.00	192.00	178.00	178.33	188.00
CTBP2	1490.00	1425.00	1629.00	1514.67	1640.00
CTBS	823.00	752.00	875.00	816.67	750.00
CTC1	700.00	729.00	812.00	747.00	772.00
CTC-338M12.4	435.00	445.00	428.00	436.00	517.00
CTC-436P18.1	7.00	2.00	7.00	5.33	3.00
CTCF	1161.00	1027.00	1222.00	1136.67	1216.00
CTDNEP1	2791.00	2757.00	2930.00	2826.00	3281.00
CTDP1	206.00	190.00	199.00	198.33	195.00
CTDSP1	857.00	847.00	910.00	871.33	1018.00
CTDSP2	3420.00	3178.00	3721.00	3439.67	3163.00
CTDSPL	319.00	299.00	312.00	310.00	424.00
CTDSPL2	423.00	389.00	485.00	432.33	507.00
CTF1	4.00	7.00	3.00	4.67	7.00
CTGF	47683.00	45238.00	45448.00	46123.00	74244.00
CTGLF12P	151.00	140.00	188.00	159.67	135.00
CTH	889.00	882.00	902.00	891.00	976.00
CTHRC1	1271.00	1228.00	1212.00	1237.00	1471.00
CTIF	1071.00	1027.00	1196.00	1098.00	1260.00
CTNNA1	5690.00	5285.00	5818.00	5597.67	4802.00
CTNNAL1	2046.00	1859.00	2119.00	2008.00	2526.00
CTNNB1	5898.00	5764.00	6580.00	6080.67	6418.00
CTNNBIP1	319.00	292.00	280.00	297.00	385.00
CTNNBL1	1070.00	1109.00	1161.00	1113.33	1329.00
CTNND1	80.00	55.00	69.00	68.00	49.00
CTNND2	3.00	14.00	15.00	10.67	9.00
CTNS	1159.00	1161.00	1129.00	1149.67	1040.00
CTPS1	2600.00	2465.00	2567.00	2544.00	2499.00
CTPS2	1874.00	1717.00	2025.00	1872.00	1999.00
CTR9	1708.00	1578.00	1702.00	1662.67	1580.00

CTRL	39.00	62.00	49.00	50.00	39.00
CTSA	7406.00	7462.00	7013.00	7293.67	7439.00
CTSB	31922.00	32320.00	32152.00	32131.33	32900.00
CTSC	2964.00	2816.00	2973.00	2917.67	4609.00
CTSD	9359.00	9788.00	8909.00	9352.00	8850.00
CTSF	910.00	975.00	909.00	931.33	764.00
CTSH	705.00	722.00	657.00	694.67	905.00
CTSK	666.00	701.00	694.00	687.00	930.00
CTSL	4562.00	4708.00	4332.00	4534.00	4759.00
CTSS	80.00	79.00	85.00	81.33	110.00
CTSV	16.00	29.00	31.00	25.33	36.00
CTSW	7.00	7.00	6.00	6.67	1.00
CTSZ	2208.00	2168.00	2099.00	2158.33	2005.00
CTTN	39428.00	39815.00	42179.00	40474.00	40044.00
CTTNBP2	196.00	190.00	204.00	196.67	189.00
CTTNBP2NL	882.00	809.00	977.00	889.33	946.00
CTU1	239.00	248.00	303.00	263.33	334.00
CTU2	985.00	883.00	951.00	939.67	1079.00
CTXN1	68.00	58.00	69.00	65.00	116.00
CUBN	239.00	214.00	285.00	246.00	288.00
CUEDC1	1469.00	1528.00	1609.00	1535.33	1563.00
CUEDC2	1335.00	1437.00	1517.00	1429.67	1740.00
CUL1	2772.00	2621.00	3123.00	2838.67	3127.00
CUL2	2464.00	2466.00	2773.00	2567.67	2996.00
CUL3	1771.00	1730.00	1946.00	1815.67	2152.00
CUL4A	2033.00	1811.00	2102.00	1982.00	2135.00
CUL4B	2266.00	2266.00	2551.00	2361.00	2419.00
CUL5	597.00	496.00	641.00	578.00	637.00
CUL7	2309.00	2206.00	2229.00	2248.00	2095.00
CUL9	2237.00	2243.00	2333.00	2271.00	1897.00
CUTA	4.00	2.00	5.00	3.67	3.00
CUTC	468.00	455.00	509.00	477.33	562.00
CUX1	1696.00	1620.00	1850.00	1722.00	1493.00
CWC15	447.00	445.00	452.00	448.00	624.00
CWC22	1001.00	898.00	1133.00	1010.67	1088.00
CWC27	626.00	618.00	738.00	660.67	873.00
CWF19L1	733.00	600.00	678.00	670.33	799.00
CWF19L2	209.00	201.00	199.00	203.00	235.00
CXCL1	991.00	874.00	820.00	895.00	1973.00
CXCL2	653.00	659.00	670.00	660.67	1142.00
CXCL3	788.00	752.00	825.00	788.33	1251.00
CXCL5	26210.00	23267.00	23874.00	24450.33	45113.00
CXCL8	12741.00	11589.00	10367.00	11565.67	25570.00
CXorf23	634.00	608.00	657.00	633.00	572.00
CXorf38	410.00	360.00	405.00	391.67	388.00
CXorf40A	175.00	180.00	152.00	169.00	212.00
CXorf40B	338.00	328.00	333.00	333.00	368.00
CXorf56	257.00	190.00	239.00	228.67	305.00

CXorf57	252.00	255.00	286.00	264.33	243.00
CXXC1	1138.00	1116.00	1223.00	1159.00	1342.00
CXXC4	27.00	24.00	33.00	28.00	23.00
CXXC5	6.00	9.00	10.00	8.33	6.00
CYB561	627.00	616.00	615.00	619.33	660.00
CYB561A3	1297.00	1295.00	1306.00	1299.33	1088.00
CYB561D1	654.00	584.00	674.00	637.33	477.00
CYB561D2	306.00	265.00	267.00	279.33	323.00
CYB5A	114.00	123.00	126.00	121.00	191.00
CYB5B	1092.00	1018.00	1008.00	1039.33	1248.00
CYB5D1	1300.00	1233.00	1343.00	1292.00	1255.00
CYB5D2	667.00	648.00	724.00	679.67	717.00
CYB5R1	745.00	705.00	673.00	707.67	803.00
CYB5R2	4.00	2.00	3.00	3.00	5.00
CYB5R3	3058.00	2881.00	2797.00	2912.00	2728.00
CYB5R4	317.00	283.00	344.00	314.67	384.00
CYB5RL	297.00	288.00	301.00	295.33	217.00
CYBA	2581.00	2697.00	2338.00	2538.67	2635.00
CYBRD1	2633.00	2571.00	2603.00	2602.33	2858.00
CYC1	2824.00	2763.00	2912.00	2833.00	3522.00
CYCS	2997.00	2719.00	3236.00	2984.00	4342.00
CYFIP1	1180.00	1082.00	1159.00	1140.33	1639.00
CYFIP2	330.00	322.00	433.00	361.67	337.00
CYHR1	1497.00	1490.00	1581.00	1522.67	1427.00
CYLD	1076.00	1037.00	1158.00	1090.33	876.00
CYP11A1	22.00	31.00	26.00	26.33	19.00
CYP1A1	7.00	11.00	10.00	9.33	4.00
CYP1B1	15.00	9.00	9.00	11.00	5.00
CYP20A1	953.00	910.00	938.00	933.67	1083.00
CYP24A1	9.00	14.00	14.00	12.33	23.00
CYP27A1	298.00	331.00	307.00	312.00	303.00
CYP27B1	267.00	293.00	279.00	279.67	280.00
CYP2E1	3.00	3.00	7.00	4.33	7.00
CYP2J2	189.00	205.00	198.00	197.33	284.00
CYP2R1	518.00	534.00	581.00	544.33	587.00
CYP2S1	12.00	11.00	12.00	11.67	3.00
CYP2U1	514.00	500.00	523.00	512.33	473.00
CYP39A1	28.00	26.00	25.00	26.33	23.00
CYP4F29P	62.00	51.00	59.00	57.33	112.00
CYP4V2	508.00	501.00	517.00	508.67	581.00
CYP51A1	1333.00	1285.00	1275.00	1297.67	1550.00
CYR61	53773.00	55920.00	54736.00	54809.67	59644.00
CYSRT1	16.00	17.00	16.00	16.33	22.00
CYSTM1	365.00	317.00	372.00	351.33	437.00
CYTH1	411.00	431.00	430.00	424.00	427.00
CYTH2	2895.00	3117.00	3285.00	3099.00	2518.00
CYTH3	939.00	857.00	938.00	911.33	802.00
CYTH4	7.00	10.00	6.00	7.67	12.00

D2HGDH	742.00	814.00	808.00	788.00	875.00
DAAM1	197.00	174.00	211.00	194.00	147.00
DAAM2	3.00	5.00	5.00	4.33	1.00
DAB2	1578.00	1439.00	1580.00	1532.33	855.00
DAB2IP	542.00	517.00	565.00	541.33	536.00
DACH1	36.00	24.00	44.00	34.67	41.00
DACH2	54.00	44.00	73.00	57.00	69.00
DACT1	16.00	43.00	21.00	26.67	10.00
DACT3	3.00	2.00	3.00	2.67	3.00
DAD1	2601.00	2576.00	2357.00	2511.33	3109.00
DAG1	1697.00	1625.00	1791.00	1704.33	1555.00
DAGLA	630.00	683.00	675.00	662.67	489.00
DAGLB	1268.00	1337.00	1403.00	1336.00	1120.00
DALRD3	585.00	532.00	610.00	575.67	627.00
DANCR	973.00	995.00	1033.00	1000.33	1129.00
DAP	3745.00	3473.00	3738.00	3652.00	3884.00
DAP3	2850.00	2727.00	2858.00	2811.67	3653.00
DAPK2	13.00	8.00	7.00	9.33	13.00
DAPK3	1308.00	1402.00	1413.00	1374.33	1395.00
DARS	2319.00	2102.00	2529.00	2316.67	2689.00
DARS2	2231.00	2012.00	2279.00	2174.00	2849.00
DARS-AS1	37.00	40.00	38.00	38.33	36.00
DAW1	765.00	670.00	704.00	713.00	1641.00
DAXX	23.00	27.00	16.00	22.00	32.00
DAZAP1	4533.00	4403.00	4713.00	4549.67	5794.00
DAZAP2	1751.00	1536.00	1917.00	1734.67	2070.00
DBF4	627.00	610.00	730.00	655.67	947.00
DBF4B	801.00	870.00	860.00	843.67	889.00
DBH-AS1	63.00	64.00	53.00	60.00	18.00
DBI	1012.00	925.00	956.00	964.33	1433.00
DBIL5P	9.00	12.00	6.00	9.00	11.00
DBN1	6600.00	6480.00	6555.00	6545.00	5510.00
DBNDD1	110.00	119.00	128.00	119.00	155.00
DBNDD2	74.00	50.00	55.00	59.67	64.00
DBNL	1021.00	1002.00	954.00	992.33	1276.00
DBP	17.00	17.00	28.00	20.67	34.00
DBR1	338.00	353.00	332.00	341.00	410.00
DBT	577.00	546.00	652.00	591.67	593.00
DCAF10	1037.00	1076.00	1251.00	1121.33	1089.00
DCAF11	1044.00	995.00	1120.00	1053.00	1131.00
DCAF12	658.00	622.00	674.00	651.33	787.00
DCAF13	2435.00	2179.00	2414.00	2342.67	3062.00
DCAF13P3	6.00	2.00	3.00	3.67	3.00
DCAF15	903.00	950.00	944.00	932.33	1109.00
DCAF16	906.00	936.00	1041.00	961.00	953.00
DCAF17	1056.00	888.00	1177.00	1040.33	933.00
DCAF4	559.00	549.00	635.00	581.00	655.00
DCAF4L1	120.00	123.00	124.00	122.33	119.00

DCAF5	1595.00	1543.00	1614.00	1584.00	1475.00
DCAF6	925.00	923.00	1005.00	951.00	1213.00
DCAF7	3358.00	3015.00	3408.00	3260.33	3163.00
DCAF8	4879.00	4882.00	5166.00	4975.67	4636.00
DCAF8L2	15.00	12.00	7.00	11.33	21.00
DCAKD	694.00	613.00	694.00	667.00	704.00
DCBLD1	864.00	866.00	847.00	859.00	789.00
DCBLD2	24445.00	22359.00	25005.00	23936.33	25903.00
DCC	183.00	181.00	214.00	192.67	154.00
DCDC2	10.00	12.00	12.00	11.33	12.00
DCHS1	906.00	855.00	796.00	852.33	684.00
DCHS2	12.00	15.00	19.00	15.33	24.00
DCK	303.00	287.00	334.00	308.00	409.00
DCLK1	4.00	2.00	4.00	3.33	5.00
DCLK2	169.00	168.00	180.00	172.33	233.00
DCLRE1A	606.00	584.00	626.00	605.33	796.00
DCLRE1B	354.00	358.00	368.00	360.00	431.00
DCLRE1C	645.00	559.00	650.00	618.00	715.00
DCP1A	927.00	796.00	886.00	869.67	839.00
DCP1B	337.00	330.00	383.00	350.00	336.00
DCP2	741.00	633.00	809.00	727.67	744.00
DCPS	109.00	112.00	99.00	106.67	143.00
DCST2	15.00	14.00	17.00	15.33	19.00
DCTD	1848.00	1692.00	2054.00	1864.67	2201.00
DCTN1	4958.00	4809.00	5075.00	4947.33	4866.00
DCTN2	2567.00	2411.00	2656.00	2544.67	2775.00
DCTN3	484.00	517.00	483.00	494.67	675.00
DCTN4	1625.00	1343.00	1729.00	1565.67	1759.00
DCTN5	1276.00	1189.00	1295.00	1253.33	1164.00
DCTN6	257.00	259.00	282.00	266.00	339.00
DCTPP1	381.00	397.00	398.00	392.00	434.00
DCUN1D1	651.00	636.00	834.00	707.00	837.00
DCUN1D2	355.00	335.00	386.00	358.67	352.00
DCUN1D3	226.00	224.00	230.00	226.67	232.00
DCUN1D4	1020.00	866.00	1050.00	978.67	1034.00
DCUN1D5	3410.00	3192.00	3493.00	3365.00	4553.00
DCXR	768.00	819.00	978.00	855.00	1025.00
DDA1	887.00	823.00	861.00	857.00	810.00
DDAH1	3152.00	2910.00	3579.00	3213.67	4492.00
DDB1	5251.00	5016.00	5512.00	5259.67	4970.00
DDB2	243.00	235.00	236.00	238.00	330.00
DDHD1	1519.00	1431.00	1630.00	1526.67	1345.00
DDHD2	1154.00	1152.00	1342.00	1216.00	1297.00
DDI2	74.00	58.00	62.00	64.67	51.00
DDIAS	267.00	234.00	230.00	243.67	297.00
DDIT3	1292.00	1333.00	1333.00	1319.33	1428.00
DDIT4	1597.00	1612.00	1590.00	1599.67	1889.00
DDN	18.00	16.00	22.00	18.67	43.00

DDO	10.00	19.00	15.00	14.67	16.00
DDOST	11752.00	11703.00	11430.00	11628.33	11922.00
DDR1	50.00	39.00	58.00	49.00	36.00
DDR2	3311.00	2819.00	3153.00	3094.33	2523.00
DDRGK1	1048.00	1105.00	993.00	1048.67	1275.00
DDX1	3294.00	3090.00	3217.00	3200.33	4020.00
DDX10	1426.00	1373.00	1565.00	1454.67	1130.00
DDX11	1845.00	1899.00	1873.00	1872.33	2154.00
DDX11-AS1	58.00	71.00	65.00	64.67	81.00
DDX11L2	74.00	84.00	90.00	82.67	96.00
DDX11L9	3.00	1.00	0.00	1.33	0.00
DDX12P	969.00	1004.00	990.00	987.67	999.00
DDX17	13164.00	12372.00	13337.00	12957.67	13328.00
DDX18	2984.00	2681.00	3043.00	2902.67	3515.00
DDX19A	775.00	803.00	872.00	816.67	794.00
DDX19B	359.00	380.00	436.00	391.67	573.00
DDX20	853.00	805.00	912.00	856.67	969.00
DDX21	8965.00	7633.00	9155.00	8584.33	9233.00
DDX23	2009.00	1978.00	2084.00	2023.67	2124.00
DDX24	3.00	1.00	1.00	1.67	3.00
DDX26B	277.00	261.00	306.00	281.33	271.00
DDX27	2298.00	2268.00	2334.00	2300.00	2486.00
DDX28	299.00	292.00	339.00	310.00	378.00
DDX31	1491.00	1443.00	1602.00	1512.00	1522.00
DDX39A	1790.00	1749.00	1847.00	1795.33	2377.00
DDX39B	3.00	4.00	3.00	3.33	2.00
DDX3X	9379.00	8532.00	9659.00	9190.00	9485.00
DDX4	4.00	1.00	4.00	3.00	4.00
DDX41	1602.00	1656.00	1705.00	1654.33	1713.00
DDX42	4938.00	4841.00	5122.00	4967.00	5316.00
DDX46	2390.00	2140.00	2553.00	2361.00	2619.00
DDX47	1361.00	1303.00	1420.00	1361.33	1554.00
DDX49	725.00	683.00	742.00	716.67	998.00
DDX5	27591.00	26984.00	28461.00	27678.67	30919.00
DDX50	1401.00	1320.00	1570.00	1430.33	1885.00
DDX51	902.00	952.00	1006.00	953.33	938.00
DDX52	112.00	102.00	126.00	113.33	119.00
DDX53	20.00	20.00	9.00	16.33	19.00
DDX54	2256.00	2257.00	2389.00	2300.67	2306.00
DDX55	1247.00	1295.00	1235.00	1259.00	1503.00
DDX56	3005.00	3143.00	3195.00	3114.33	3413.00
DDX58	552.00	577.00	592.00	573.67	696.00
DDX59	449.00	394.00	497.00	446.67	514.00
DDX6	2383.00	2217.00	2637.00	2412.33	2398.00
DDX60	114.00	91.00	117.00	107.33	128.00
DDX60L	169.00	160.00	190.00	173.00	151.00
DEAF1	477.00	475.00	460.00	470.67	499.00
DECR1	1215.00	1166.00	1231.00	1204.00	1566.00

DEDD	848.00	769.00	868.00	828.33	928.00
DEDD2	307.00	258.00	301.00	288.67	310.00
DEF8	2529.00	2605.00	2839.00	2657.67	2672.00
DEGS1	2675.00	2551.00	2619.00	2615.00	2635.00
DEK	2760.00	2394.00	2893.00	2682.33	3540.00
DENND1A	445.00	369.00	490.00	434.67	514.00
DENND1B	147.00	138.00	166.00	150.33	155.00
DENND1C	3.00	0.00	1.00	1.33	1.00
DENND2A	716.00	670.00	654.00	680.00	523.00
DENND2C	49.00	31.00	47.00	42.33	39.00
DENND3	1222.00	1148.00	1250.00	1206.67	1374.00
DENND4A	643.00	614.00	799.00	685.33	665.00
DENND4B	2963.00	2710.00	2956.00	2876.33	2461.00
DENND4C	810.00	726.00	825.00	787.00	704.00
DENND5A	1822.00	1835.00	2014.00	1890.33	1619.00
DENND5B	721.00	667.00	808.00	732.00	607.00
DENND6A	498.00	465.00	585.00	516.00	533.00
DENND6B	19.00	22.00	24.00	21.67	22.00
DENR	2697.00	2456.00	2792.00	2648.33	3362.00
DEPDC1	1465.00	1256.00	1367.00	1362.67	1706.00
DEPDC1-AS1	5.00	2.00	3.00	3.33	6.00
DEPDC1B	272.00	214.00	282.00	256.00	335.00
DEPDC4	16.00	22.00	17.00	18.33	29.00
DEPDC5	317.00	343.00	355.00	338.33	368.00
DEPDC7	177.00	147.00	139.00	154.33	206.00
DEPTOR	19.00	38.00	38.00	31.67	43.00
DERA	530.00	482.00	535.00	515.67	737.00
DERL1	2745.00	2574.00	2658.00	2659.00	2574.00
DERL2	1628.00	1533.00	1609.00	1590.00	1736.00
DESI1	1845.00	1776.00	1957.00	1859.33	1819.00
DESI2	1444.00	1277.00	1562.00	1427.67	1848.00
DET1	237.00	211.00	257.00	235.00	244.00
DEXI	543.00	486.00	519.00	516.00	630.00
DFFA	972.00	874.00	924.00	923.33	1035.00
DFFB	364.00	361.00	386.00	370.33	397.00
DFNA5	2010.00	1752.00	2145.00	1969.00	1900.00
DFNB31	77.00	98.00	94.00	89.67	82.00
DFNB59	147.00	155.00	164.00	155.33	118.00
DGAT1	856.00	904.00	954.00	904.67	910.00
DGAT2	2445.00	2564.00	2441.00	2483.33	2794.00
DGCR11	17.00	31.00	23.00	23.67	25.00
DGCR14	390.00	389.00	446.00	408.33	391.00
DGCR2	1960.00	1946.00	1956.00	1954.00	1896.00
DGCR6L	361.00	365.00	373.00	366.33	470.00
DGCR8	1354.00	1348.00	1527.00	1409.67	1463.00
DGKA	419.00	414.00	509.00	447.33	409.00
DGKD	1490.00	1480.00	1460.00	1476.67	1115.00
DGKE	151.00	134.00	153.00	146.00	131.00

DGKG	105.00	128.00	104.00	112.33	71.00
DGKH	916.00	835.00	997.00	916.00	684.00
DGKI	59.00	71.00	46.00	58.67	58.00
DGKQ	602.00	595.00	622.00	606.33	563.00
DGKZ	1046.00	1026.00	1085.00	1052.33	1160.00
DGUOK	1091.00	1042.00	1092.00	1075.00	1494.00
DHCR24	3201.00	2924.00	2889.00	3004.67	2460.00
DHCR7	16037.00	15728.00	15874.00	15879.67	15222.00
DHDDS	689.00	682.00	747.00	706.00	678.00
DHDH	3.00	1.00	8.00	4.00	3.00
DHFR	248.00	251.00	243.00	247.33	312.00
DHFRL1	287.00	252.00	260.00	266.33	342.00
DHODH	408.00	408.00	422.00	412.67	537.00
DHPS	1370.00	1290.00	1417.00	1359.00	1493.00
DHRS1	360.00	329.00	349.00	346.00	375.00
DHRS12	54.00	56.00	62.00	57.33	95.00
DHRS13	77.00	94.00	90.00	87.00	145.00
DHRS2	3.00	2.00	2.00	2.33	1.00
DHRS3	185.00	207.00	211.00	201.00	199.00
DHRS4	155.00	142.00	132.00	143.00	186.00
DHRS4-AS1	248.00	224.00	265.00	245.67	277.00
DHRS4L2	68.00	72.00	85.00	75.00	112.00
DHRS7	1490.00	1587.00	1584.00	1553.67	2049.00
DHRS7B	326.00	358.00	312.00	332.00	427.00
DHTKD1	634.00	545.00	676.00	618.33	553.00
DHX15	3272.00	3209.00	3423.00	3301.33	3776.00
DHX29	1095.00	1082.00	1277.00	1151.33	1118.00
DHX30	2618.00	2694.00	2733.00	2681.67	2886.00
DHX32	1167.00	1004.00	1195.00	1122.00	1296.00
DHX33	2729.00	2572.00	2909.00	2736.67	2795.00
DHX34	1543.00	1516.00	1548.00	1535.67	1365.00
DHX35	643.00	679.00	732.00	684.67	695.00
DHX36	1174.00	1078.00	1328.00	1193.33	1291.00
DHX37	1207.00	1301.00	1295.00	1267.67	1276.00
DHX38	2209.00	2391.00	2247.00	2282.33	2207.00
DHX40	1289.00	1198.00	1375.00	1287.33	1426.00
DHX57	1023.00	964.00	1034.00	1007.00	1110.00
DHX58	42.00	47.00	43.00	44.00	45.00
DHX8	991.00	997.00	993.00	993.67	879.00
DHX9	5110.00	4918.00	5388.00	5138.67	5998.00
DIABLO	707.00	696.00	701.00	701.33	840.00
DIAPH1	3367.00	3109.00	3344.00	3273.33	3302.00
DIAPH2	413.00	333.00	360.00	368.67	434.00
DIAPH2-AS1	6.00	4.00	4.00	4.67	0.00
DIAPH3	371.00	312.00	339.00	340.67	374.00
DICER1	1991.00	1734.00	2039.00	1921.33	1825.00
DICER1-AS1	175.00	183.00	157.00	171.67	158.00
DIDO1	4247.00	4114.00	4439.00	4266.67	3696.00

DIEXF	1288.00	1200.00	1339.00	1275.67	1334.00
DIMT1	506.00	464.00	521.00	497.00	641.00
DIO2	17.00	17.00	15.00	16.33	12.00
DIP2A	1779.00	1826.00	2003.00	1869.33	1496.00
DIP2A-IT1	10.00	4.00	1.00	5.00	1.00
DIP2B	1143.00	1005.00	1168.00	1105.33	961.00
DIP2C	499.00	584.00	578.00	553.67	518.00
DIRAS3	7.00	10.00	17.00	11.33	7.00
DIRC2	742.00	726.00	664.00	710.67	792.00
DIRC3	3.00	0.00	0.00	1.00	1.00
DIS3	1598.00	1445.00	1737.00	1593.33	1770.00
DIS3L	1191.00	1144.00	1243.00	1192.67	1241.00
DIS3L2	953.00	888.00	977.00	939.33	865.00
DISC1	4.00	7.00	5.00	5.33	0.00
DISC1FP1	8.00	7.00	13.00	9.33	7.00
DISP1	294.00	264.00	250.00	269.33	191.00
DISP2	256.00	309.00	304.00	289.67	248.00
DIXDC1	271.00	223.00	303.00	265.67	321.00
DKC1	3217.00	3121.00	3439.00	3259.00	4342.00
DKFZP434A062	41.00	30.00	37.00	36.00	29.00
DKFZP434I0714	151.00	172.00	203.00	175.33	162.00
DKFZP586I1420	66.00	69.00	71.00	68.67	76.00
DKK1	8141.00	7899.00	8556.00	8198.67	9122.00
DKK2	182.00	137.00	180.00	166.33	173.00
DKK3	20483.00	20219.00	20554.00	20418.67	13594.00
DLAT	1035.00	860.00	1059.00	984.67	1159.00
DLC1	4957.00	4472.00	5307.00	4912.00	4950.00
DLD	1448.00	1422.00	1602.00	1490.67	1751.00
DLEU1	258.00	232.00	306.00	265.33	330.00
DLEU2	44.00	56.00	56.00	52.00	55.00
DLEU2L	11.00	11.00	15.00	12.33	14.00
DLG1	3010.00	2733.00	3147.00	2963.33	3168.00
DLG2	15.00	9.00	19.00	14.33	23.00
DLG3	654.00	613.00	685.00	650.67	627.00
DLG4	1388.00	1344.00	1568.00	1433.33	1389.00
DLG5	5.00	2.00	6.00	4.33	1.00
DLGAP1-AS1	370.00	374.00	387.00	377.00	404.00
DLGAP1-AS2	375.00	345.00	335.00	351.67	350.00
DLGAP3	3.00	0.00	1.00	1.33	0.00
DLGAP4	2706.00	2582.00	2904.00	2730.67	2251.00
DLGAP4-AS1	3.00	2.00	0.00	1.67	7.00
DLGAP5	579.00	502.00	544.00	541.67	775.00
DLK2	3.00	5.00	3.00	3.67	2.00
DLL3	87.00	88.00	92.00	89.00	139.00
DLL4	7.00	10.00	7.00	8.00	9.00
DLST	2431.00	2358.00	2415.00	2401.33	2619.00
DLSTP1	4.00	4.00	3.00	3.67	1.00
DLX1	786.00	707.00	787.00	760.00	961.00

DLX2	61.00	66.00	75.00	67.33	100.00
DLX2-AS1	4.00	3.00	7.00	4.67	12.00
DLX5	3.00	1.00	7.00	3.67	11.00
DLX6	21.00	16.00	19.00	18.67	35.00
DMAP1	642.00	643.00	672.00	652.33	709.00
DMD	46.00	63.00	78.00	62.33	49.00
DMGDH	34.00	32.00	24.00	30.00	18.00
DMPK	1459.00	1544.00	1719.00	1574.00	1464.00
DMRTA1	72.00	59.00	77.00	69.33	69.00
DMTF1	3294.00	3115.00	3589.00	3332.67	3301.00
DMTN	4.00	2.00	2.00	2.67	6.00
DMWD	1020.00	1024.00	1154.00	1066.00	873.00
DMXL1	743.00	726.00	860.00	776.33	668.00
DMXL2	1214.00	1033.00	1210.00	1152.33	1049.00
DNA2	441.00	450.00	452.00	447.67	577.00
DNAAF2	375.00	417.00	429.00	407.00	441.00
DNAAF3	4.00	14.00	14.00	10.67	12.00
DNAAF5	1791.00	1591.00	1708.00	1696.67	1794.00
DNAH1	217.00	221.00	249.00	229.00	150.00
DNAH11	684.00	671.00	757.00	704.00	508.00
DNAH12	26.00	19.00	34.00	26.33	15.00
DNAH14	661.00	666.00	664.00	663.67	727.00
DNAH2	219.00	223.00	246.00	229.33	191.00
DNAH5	388.00	358.00	445.00	397.00	301.00
DNAH6	41.00	23.00	30.00	31.33	25.00
DNAH7	17.00	12.00	9.00	12.67	12.00
DNAJA1	3537.00	3371.00	3720.00	3542.67	4141.00
DNAJA2	1874.00	1778.00	2058.00	1903.33	2333.00
DNAJA3	5.00	3.00	5.00	4.33	5.00
DNAJB1	1547.00	1472.00	1587.00	1535.33	1623.00
DNAJB11	2594.00	2652.00	2546.00	2597.33	2795.00
DNAJB12	1806.00	1793.00	1774.00	1791.00	2302.00
DNAJB13	10.00	12.00	11.00	11.00	8.00
DNAJB14	739.00	615.00	801.00	718.33	635.00
DNAJB2	594.00	673.00	759.00	675.33	698.00
DNAJB4	376.00	324.00	357.00	352.33	305.00
DNAJB5	141.00	148.00	131.00	140.00	159.00
DNAJB6	5689.00	5396.00	5868.00	5651.00	7369.00
DNAJB9	655.00	561.00	627.00	614.33	553.00
DNAJC1	1411.00	1433.00	1469.00	1437.67	1385.00
DNAJC10	6140.00	5565.00	6307.00	6004.00	5819.00
DNAJC11	2228.00	2056.00	2246.00	2176.67	2442.00
DNAJC12	3.00	6.00	4.00	4.33	9.00
DNAJC13	2789.00	2582.00	2875.00	2748.67	2729.00
DNAJC14	1322.00	1341.00	1415.00	1359.33	1342.00
DNAJC15	305.00	307.00	326.00	312.67	411.00
DNAJC16	1114.00	1064.00	1195.00	1124.33	995.00
DNAJC17	252.00	241.00	263.00	252.00	334.00

DNAJC18	905.00	749.00	858.00	837.33	832.00
DNAJC19	719.00	699.00	705.00	707.67	939.00
DNAJC2	1224.00	1219.00	1356.00	1266.33	1462.00
DNAJC21	877.00	851.00	956.00	894.67	1123.00
DNAJC22	308.00	300.00	296.00	301.33	333.00
DNAJC24	461.00	437.00	443.00	447.00	572.00
DNAJC25	236.00	249.00	242.00	242.33	278.00
DNAJC27	194.00	158.00	185.00	179.00	166.00
DNAJC3	1224.00	1155.00	1163.00	1180.67	1127.00
DNAJC30	175.00	164.00	155.00	164.67	207.00
DNAJC3-AS1	64.00	68.00	86.00	72.67	55.00
DNAJC4	925.00	903.00	935.00	921.00	958.00
DNAJC5	2474.00	2318.00	2500.00	2430.67	2564.00
DNAJC5B	3.00	5.00	0.00	2.67	7.00
DNAJC6	216.00	171.00	192.00	193.00	205.00
DNAJC7	1716.00	1619.00	1880.00	1738.33	2117.00
DNAJC8	1762.00	1739.00	1850.00	1783.67	2411.00
DNAJC9	1271.00	1194.00	1344.00	1269.67	1513.00
DNAJC9-AS1	4.00	2.00	5.00	3.67	3.00
DNAL1	284.00	305.00	331.00	306.67	294.00
DNAL4	262.00	248.00	261.00	257.00	318.00
DNASE1	164.00	222.00	224.00	203.33	158.00
DNASE1L1	355.00	396.00	383.00	378.00	464.00
DNASE1L2	92.00	91.00	91.00	91.33	81.00
DNASE2	1203.00	1110.00	1118.00	1143.67	1081.00
DND1	96.00	84.00	83.00	87.67	111.00
DNER	1341.00	1399.00	1415.00	1385.00	1430.00
DNHD1	2292.00	2175.00	2407.00	2291.33	1757.00
DNLZ	184.00	206.00	237.00	209.00	221.00
DNM1	1425.00	1429.00	1512.00	1455.33	1274.00
DNM1L	2329.00	2173.00	2528.00	2343.33	2759.00
DNM1P46	19.00	21.00	18.00	19.33	19.00
DNM2	3072.00	2967.00	3170.00	3069.67	3113.00
DNM3	19.00	22.00	19.00	20.00	24.00
DNMBP	8211.00	7643.00	7921.00	7925.00	7331.00
DNMBP-AS1	12.00	8.00	10.00	10.00	1.00
DNMT1	4093.00	3969.00	4141.00	4067.67	4809.00
DNMT3A	484.00	498.00	549.00	510.33	422.00
DNMT3B	250.00	231.00	271.00	250.67	202.00
DNPEP	913.00	972.00	1099.00	994.67	1047.00
DNPH1	721.00	743.00	804.00	756.00	1139.00
DNTTIP1	860.00	837.00	916.00	871.00	1131.00
DNTTIP2	1888.00	1768.00	1867.00	1841.00	2039.00
DOC2A	6.00	5.00	6.00	5.67	5.00
DOCK1	2357.00	2162.00	2422.00	2313.67	2057.00
DOCK10	2026.00	1895.00	2030.00	1983.67	1928.00
DOCK11	439.00	394.00	472.00	435.00	360.00
DOCK2	270.00	216.00	260.00	248.67	139.00

DOCK3	2029.00	2102.00	2288.00	2139.67	1484.00
DOCK4	3008.00	2486.00	2868.00	2787.33	2664.00
DOCK5	1520.00	1442.00	1510.00	1490.67	1320.00
DOCK6	954.00	944.00	1134.00	1010.67	849.00
DOCK7	1661.00	1491.00	1742.00	1631.33	1608.00
DOCK9	1215.00	1149.00	1321.00	1228.33	792.00
DOCK9-AS2	7.00	6.00	6.00	6.33	7.00
DOHH	381.00	398.00	393.00	390.67	439.00
DOK1	100.00	142.00	117.00	119.67	149.00
DOK2	7.00	1.00	3.00	3.67	5.00
DOK3	232.00	216.00	236.00	228.00	203.00
DOK4	45.00	34.00	22.00	33.67	37.00
DOK6	17.00	8.00	22.00	15.67	8.00
DOK7	8.00	7.00	14.00	9.67	3.00
DOLK	688.00	695.00	754.00	712.33	691.00
DOLPP1	666.00	661.00	585.00	637.33	672.00
DONSON	417.00	446.00	402.00	421.67	470.00
DOPEY1	539.00	490.00	527.00	518.67	456.00
DOPEY2	552.00	491.00	587.00	543.33	500.00
DOT1L	1648.00	1707.00	1870.00	1741.67	1709.00
DPAGT1	2687.00	2662.00	2813.00	2720.67	2591.00
DPCD	268.00	267.00	289.00	274.67	366.00
DPF1	60.00	85.00	76.00	73.67	62.00
DPF2	2206.00	2165.00	2393.00	2254.67	2381.00
DPF3	434.00	383.00	477.00	431.33	399.00
DPH1	613.00	713.00	723.00	683.00	740.00
DPH2	1378.00	1315.00	1329.00	1340.67	1459.00
DPH3	635.00	598.00	718.00	650.33	824.00
DPH5	1222.00	1295.00	1426.00	1314.33	1633.00
DPH6	258.00	270.00	237.00	255.00	291.00
DPH6-AS1	10.00	15.00	13.00	12.67	18.00
DPH7	1311.00	1339.00	1486.00	1378.67	1401.00
DPM1	1197.00	1060.00	1187.00	1148.00	1515.00
DPM2	965.00	949.00	865.00	926.33	1153.00
DPM3	276.00	280.00	241.00	265.67	315.00
DPP3	1451.00	1509.00	1594.00	1518.00	1652.00
DPP7	2836.00	2970.00	3113.00	2973.00	3143.00
DPP8	635.00	513.00	642.00	596.67	489.00
DPP9	6745.00	6719.00	7205.00	6889.67	6697.00
DPPA4	17.00	8.00	12.00	12.33	8.00
DPRXP4	5.00	9.00	10.00	8.00	8.00
DPY19L1	4222.00	3824.00	4219.00	4088.33	3347.00
DPY19L1P1	111.00	108.00	128.00	115.67	119.00
DPY19L2	734.00	688.00	697.00	706.33	689.00
DPY19L2P1	123.00	133.00	134.00	130.00	189.00
DPY19L2P2	185.00	188.00	199.00	190.67	205.00
DPY19L2P3	5.00	6.00	5.00	5.33	8.00
DPY19L2P4	48.00	43.00	36.00	42.33	41.00

DPY19L3	1448.00	1463.00	1577.00	1496.00	1311.00
DPY19L4	1349.00	1241.00	1424.00	1338.00	1295.00
DPY30	554.00	525.00	608.00	562.33	747.00
DPYD	385.00	312.00	352.00	349.67	429.00
DPYS	8.00	2.00	0.00	3.33	3.00
DPYSL2	822.00	825.00	774.00	807.00	853.00
DPYSL3	1741.00	1566.00	1742.00	1683.00	1325.00
DQX1	15.00	12.00	18.00	15.00	15.00
DR1	1243.00	1154.00	1267.00	1221.33	1425.00
DRAIC	3.00	3.00	3.00	3.00	0.00
DRAM1	1011.00	1079.00	1106.00	1065.33	1163.00
DRAM2	1219.00	1151.00	1164.00	1178.00	1391.00
DRAP1	11689.00	12784.00	12804.00	12425.67	10649.00
DRD1	20.00	23.00	32.00	25.00	15.00
DRD2	3.00	11.00	7.00	7.00	4.00
DRD4	4.00	2.00	6.00	4.00	3.00
DRG1	968.00	958.00	1100.00	1008.67	1259.00
DRG2	934.00	946.00	969.00	949.67	1091.00
DROSHA	2220.00	2107.00	2261.00	2196.00	2036.00
DRP2	92.00	75.00	78.00	81.67	60.00
DSC2	48.00	48.00	50.00	48.67	40.00
DSCAS	6.00	1.00	0.00	2.33	4.00
DSCC1	174.00	177.00	168.00	173.00	300.00
DSCR3	1134.00	1022.00	1199.00	1118.33	1164.00
DSCR4	5.00	2.00	7.00	4.67	7.00
DSCR8	31.00	53.00	42.00	42.00	38.00
DSE	987.00	897.00	1099.00	994.33	946.00
DSG2	322.00	307.00	350.00	326.33	304.00
DSN1	738.00	720.00	803.00	753.67	811.00
DST	13194.00	12474.00	13486.00	13051.33	10791.00
DSTN	2144.00	2182.00	2434.00	2253.33	2879.00
DSTNP2	88.00	62.00	67.00	72.33	87.00
DSTYK	650.00	615.00	679.00	648.00	519.00
DTD1	1010.00	1049.00	1200.00	1086.33	1399.00
DTD2	335.00	269.00	350.00	318.00	381.00
DTL	610.00	585.00	550.00	581.67	799.00
DTNA	710.00	643.00	672.00	675.00	690.00
DTNB	194.00	182.00	210.00	195.33	269.00
DTNBP1	378.00	342.00	351.00	357.00	498.00
DTWD1	581.00	496.00	600.00	559.00	646.00
DTWD2	57.00	48.00	60.00	55.00	56.00
DTX2	173.00	148.00	160.00	160.33	154.00
2P1-UPK3BP1-PMS2	30.00	44.00	38.00	37.33	32.00
DTX3	1830.00	1919.00	2060.00	1936.33	1819.00
DTX3L	732.00	595.00	799.00	708.67	582.00
DTYMK	1077.00	1066.00	1078.00	1073.67	1415.00
DUS1L	3432.00	3628.00	3571.00	3543.67	3766.00
DUS2	477.00	503.00	444.00	474.67	564.00

DUS3L	1064.00	1003.00	1061.00	1042.67	1281.00
DUS4L	525.00	478.00	488.00	497.00	618.00
DUSP1	3564.00	3421.00	3862.00	3615.67	4872.00
DUSP10	274.00	248.00	263.00	261.67	182.00
DUSP11	359.00	320.00	344.00	341.00	397.00
DUSP12	786.00	777.00	840.00	801.00	950.00
DUSP14	9.00	11.00	10.00	10.00	11.00
DUSP16	475.00	494.00	577.00	515.33	420.00
DUSP18	72.00	63.00	58.00	64.33	86.00
DUSP19	41.00	37.00	58.00	45.33	65.00
DUSP2	18.00	25.00	22.00	21.67	28.00
DUSP22	503.00	500.00	529.00	510.67	558.00
DUSP23	116.00	109.00	110.00	111.67	122.00
DUSP28	53.00	67.00	79.00	66.33	74.00
DUSP3	2580.00	2465.00	2712.00	2585.67	2006.00
DUSP4	1537.00	1516.00	1660.00	1571.00	617.00
DUSP5	4551.00	4425.00	4726.00	4567.33	5664.00
DUSP5P1	5.00	1.00	4.00	3.33	1.00
DUSP6	4915.00	4560.00	5012.00	4829.00	4153.00
DUSP7	941.00	887.00	1069.00	965.67	1133.00
DUT	1572.00	1428.00	1530.00	1510.00	1909.00
DUXAP10	3.00	4.00	7.00	4.67	2.00
DUXAP8	183.00	156.00	167.00	168.67	143.00
DVL1	2808.00	2814.00	3037.00	2886.33	3100.00
DVL2	1703.00	1710.00	1725.00	1712.67	1939.00
DVL3	2595.00	2664.00	2949.00	2736.00	2387.00
DXO	41.00	37.00	55.00	44.33	55.00
DYM	551.00	508.00	655.00	571.33	645.00
DYNAP	9.00	8.00	3.00	6.67	11.00
DYNC1H1	29813.00	26977.00	30524.00	29104.67	26552.00
DYNC1I1	38.00	37.00	45.00	40.00	54.00
DYNC1I2	1886.00	1720.00	1885.00	1830.33	2052.00
DYNC1LI1	984.00	921.00	1000.00	968.33	1316.00
DYNC1LI2	2922.00	2724.00	2981.00	2875.67	2753.00
DYNC2H1	3988.00	3622.00	4191.00	3933.67	3308.00
DYNC2LI1	446.00	400.00	516.00	454.00	508.00
DYNLL1	2442.00	2416.00	2614.00	2490.67	3138.00
DYNLL2	922.00	916.00	929.00	922.33	862.00
DYNLRB1	1218.00	1129.00	1356.00	1234.33	1609.00
DYNLT1	630.00	716.00	712.00	686.00	808.00
DYNLT3	293.00	252.00	330.00	291.67	337.00
DYRK1A	1071.00	985.00	1187.00	1081.00	1097.00
DYRK1B	134.00	133.00	130.00	132.33	119.00
DYRK2	807.00	690.00	929.00	808.67	652.00
DYRK3	558.00	602.00	621.00	593.67	523.00
DYRK4	361.00	387.00	401.00	383.00	417.00
DYSF	4.00	0.00	0.00	1.33	1.00
DYX1C1	39.00	40.00	31.00	36.67	52.00

DZANK1	180.00	197.00	196.00	191.00	174.00
DZIP1	965.00	884.00	1026.00	958.33	1049.00
DZIP1L	326.00	286.00	307.00	306.33	314.00
DZIP3	862.00	851.00	879.00	864.00	1138.00
E2F1	510.00	522.00	483.00	505.00	860.00
E2F2	15.00	10.00	11.00	12.00	43.00
E2F3	1883.00	1711.00	1895.00	1829.67	2168.00
E2F4	2364.00	2361.00	2511.00	2412.00	2767.00
E2F5	755.00	813.00	859.00	809.00	1003.00
E2F6	457.00	446.00	447.00	450.00	567.00
E2F7	5157.00	4948.00	5345.00	5150.00	3100.00
E2F8	50.00	40.00	45.00	45.00	121.00
E4F1	931.00	1042.00	1066.00	1013.00	1037.00
EAF1	745.00	593.00	757.00	698.33	725.00
EAF2	31.00	22.00	20.00	24.33	47.00
EAPP	413.00	415.00	438.00	422.00	484.00
EARS2	830.00	803.00	916.00	849.67	810.00
EBAG9	500.00	478.00	480.00	486.00	557.00
EBF1	33.00	17.00	25.00	25.00	14.00
EBF2	20.00	13.00	9.00	14.00	15.00
EBF4	17.00	16.00	19.00	17.33	10.00
EBI3	3.00	0.00	2.00	1.67	1.00
EBLN1	3.00	0.00	0.00	1.00	1.00
EBLN2	94.00	95.00	100.00	96.33	83.00
EBLN3	2097.00	1968.00	2373.00	2146.00	2155.00
EBNA1BP2	3037.00	3023.00	3073.00	3044.33	4725.00
EBP	559.00	576.00	507.00	547.33	614.00
EBPL	878.00	842.00	829.00	849.67	1106.00
ECD	1189.00	1124.00	1282.00	1198.33	1292.00
ECE1	3117.00	3030.00	2883.00	3010.00	2565.00
ECE2	104.00	98.00	130.00	110.67	141.00
ECH1	302.00	350.00	389.00	347.00	510.00
ECHDC1	1125.00	1095.00	1170.00	1130.00	1517.00
ECHDC2	647.00	728.00	693.00	689.33	723.00
ECHDC3	107.00	96.00	103.00	102.00	127.00
ECHS1	1714.00	1683.00	1756.00	1717.67	1645.00
ECI1	513.00	518.00	509.00	513.33	659.00
ECI2	7689.00	7525.00	8281.00	7831.67	9476.00
ECM1	264.00	253.00	243.00	253.33	456.00
ECSCR	511.00	500.00	519.00	510.00	855.00
ECSIT	531.00	479.00	539.00	516.33	642.00
ECT2	1542.00	1316.00	1592.00	1483.33	2058.00
ECT2L	3.00	3.00	1.00	2.33	0.00
EDA	13.00	10.00	10.00	11.00	13.00
EDA2R	402.00	375.00	493.00	423.33	349.00
EDC3	1159.00	1168.00	1259.00	1195.33	1190.00
EDC4	2071.00	2122.00	2206.00	2133.00	2072.00
EDEM1	3027.00	2794.00	3101.00	2974.00	3157.00

EDEM2	1594.00	1541.00	1681.00	1605.33	1616.00
EDEM3	2354.00	2072.00	2464.00	2296.67	2281.00
EDF1	2685.00	2639.00	2618.00	2647.33	3351.00
EDIL3	101.00	97.00	116.00	104.67	13.00
EDN1	1816.00	1942.00	1981.00	1913.00	3268.00
EDNRA	47.00	32.00	35.00	38.00	63.00
EDNRB	122.00	135.00	123.00	126.67	141.00
EDNRB-AS1	4.00	1.00	3.00	2.67	2.00
EDRF1	1296.00	1264.00	1289.00	1283.00	1228.00
EEA1	1190.00	986.00	1236.00	1137.33	1002.00
EED	334.00	333.00	318.00	328.33	401.00
EEF1A1	101709.00	93779.00	113343.00	102943.67	134787.00
EEF1A2	11.00	11.00	13.00	11.67	11.00
EEF1B2	6348.00	6228.00	6855.00	6477.00	9076.00
EEF1D	28.00	10.00	22.00	20.00	29.00
EEF1DP3	3.00	2.00	6.00	3.67	1.00
EEF1E1	396.00	382.00	453.00	410.33	593.00
EEF1G	22992.00	22243.00	24838.00	23357.67	30013.00
EEF2	65845.00	62362.00	71160.00	66455.67	68269.00
EEF2K	831.00	843.00	1043.00	905.67	1094.00
EEF2KMT	362.00	370.00	317.00	349.67	438.00
EEFSEC	343.00	397.00	406.00	382.00	407.00
EEPD1	44.00	44.00	67.00	51.67	102.00
EFCAB11	143.00	167.00	162.00	157.33	159.00
EFCAB12	3.00	2.00	3.00	2.67	1.00
EFCAB13	246.00	222.00	257.00	241.67	224.00
EFCAB14	1712.00	1629.00	1768.00	1703.00	1655.00
EFCAB2	120.00	112.00	116.00	116.00	143.00
EFCAB5	8.00	7.00	6.00	7.00	8.00
EFCAB7	191.00	192.00	211.00	198.00	231.00
EFEMP1	1209.00	1134.00	1237.00	1193.33	1810.00
EFEMP2	524.00	572.00	573.00	556.33	577.00
EFHC1	1405.00	1431.00	1455.00	1430.33	1427.00
EFHC2	11.00	17.00	5.00	11.00	13.00
EFHD2	2337.00	2296.00	2326.00	2319.67	3155.00
EFNA2	5.00	0.00	4.00	3.00	4.00
EFNA3	27.00	28.00	30.00	28.33	35.00
EFNA4	110.00	112.00	92.00	104.67	98.00
EFNA5	357.00	358.00	404.00	373.00	417.00
EFNB1	1033.00	1106.00	1123.00	1087.33	755.00
EFNB2	320.00	328.00	300.00	316.00	264.00
EFNB3	9.00	6.00	15.00	10.00	8.00
EFR3A	1731.00	1570.00	1933.00	1744.67	1788.00
EFTUD1	981.00	956.00	1110.00	1015.67	1089.00
EFTUD1P1	7.00	3.00	4.00	4.67	2.00
EFTUD2	3499.00	3294.00	3670.00	3487.67	3900.00
EGF	17.00	19.00	23.00	19.67	4.00
EGFEM1P	7.00	3.00	3.00	4.33	5.00

EGFL7	30.00	22.00	33.00	28.33	34.00
EGFR	10026.00	8684.00	10292.00	9667.33	5998.00
EGLN1	1200.00	1016.00	1220.00	1145.33	1413.00
EGLN2	19.00	13.00	16.00	16.00	31.00
EGR1	1389.00	1233.00	1691.00	1437.67	1888.00
EGR2	17.00	19.00	27.00	21.00	17.00
EHBP1	1742.00	1641.00	1772.00	1718.33	1867.00
EHBP1L1	2519.00	2721.00	2606.00	2615.33	2674.00
EHD1	2309.00	2151.00	2292.00	2250.67	2715.00
EHD2	1151.00	1079.00	1239.00	1156.33	858.00
EHD3	8.00	21.00	16.00	15.00	22.00
EHD4	2277.00	1974.00	2080.00	2110.33	3075.00
EHF	3.00	2.00	1.00	2.00	1.00
EHHADH	73.00	72.00	70.00	71.67	86.00
EHMT1	1462.00	1401.00	1563.00	1475.33	1401.00
EI24	1048.00	1028.00	1066.00	1047.33	1300.00
EID1	2270.00	2143.00	2435.00	2282.67	2601.00
EID2	204.00	197.00	210.00	203.67	225.00
EID2B	69.00	69.00	75.00	71.00	87.00
EID3	23.00	30.00	32.00	28.33	29.00
EIF1	13289.00	13226.00	14966.00	13827.00	17460.00
EIF1AD	902.00	900.00	921.00	907.67	771.00
EIF1AX	1949.00	1690.00	2078.00	1905.67	2403.00
EIF1B	1106.00	1088.00	1216.00	1136.67	1456.00
EIF1B-AS1	9.00	11.00	10.00	10.00	14.00
EIF2A	2913.00	2882.00	3331.00	3042.00	3828.00
EIF2AK1	2367.00	2259.00	2504.00	2376.67	2452.00
EIF2AK2	518.00	463.00	555.00	512.00	460.00
EIF2AK3	533.00	500.00	520.00	517.67	476.00
EIF2AK4	2681.00	2418.00	2802.00	2633.67	2554.00
EIF2B1	1176.00	1065.00	1155.00	1132.00	1365.00
EIF2B2	1018.00	956.00	1055.00	1009.67	1059.00
EIF2B3	698.00	678.00	683.00	686.33	909.00
EIF2B4	891.00	892.00	928.00	903.67	905.00
EIF2B5	1728.00	1628.00	1765.00	1707.00	1680.00
EIF2D	1914.00	1906.00	2040.00	1953.33	2057.00
EIF2S1	2108.00	1949.00	2219.00	2092.00	3006.00
EIF2S2	5109.00	4717.00	5404.00	5076.67	4374.00
EIF2S3	10383.00	9676.00	10740.00	10266.33	11354.00
EIF3A	13971.00	12996.00	14911.00	13959.33	13257.00
EIF3B	6563.00	6580.00	7015.00	6719.33	7941.00
EIF3D	6324.00	6110.00	6978.00	6470.67	8135.00
EIF3E	10866.00	10419.00	11640.00	10975.00	14071.00
EIF3F	1908.00	1905.00	2124.00	1979.00	2568.00
EIF3G	3124.00	3036.00	3196.00	3118.67	3833.00
EIF3H	4872.00	4561.00	5277.00	4903.33	6657.00
EIF3I	3863.00	3621.00	4216.00	3900.00	4929.00
EIF3J	1964.00	1913.00	2015.00	1964.00	2455.00

EIF3J-AS1	277.00	302.00	316.00	298.33	345.00
EIF3K	1873.00	1883.00	1962.00	1906.00	2615.00
EIF3L	5768.00	5268.00	6118.00	5718.00	6762.00
EIF3M	4181.00	4060.00	4592.00	4277.67	5851.00
EIF4A2	14151.00	14038.00	15565.00	14584.67	16342.00
EIF4A3	2219.00	2121.00	2287.00	2209.00	2678.00
EIF4B	8228.00	7489.00	9249.00	8322.00	9014.00
EIF4E	436.00	376.00	495.00	435.67	563.00
EIF4E2	818.00	834.00	916.00	856.00	1015.00
EIF4E3	29.00	28.00	15.00	24.00	27.00
EIF4EBP1	3338.00	3194.00	3434.00	3322.00	4454.00
EIF4EBP2	1434.00	1316.00	1536.00	1428.67	1570.00
EIF4EBP3	40.00	43.00	47.00	43.33	85.00
EIF4ENIF1	569.00	548.00	566.00	561.00	552.00
EIF4G1	18903.00	18701.00	19210.00	18938.00	18856.00
EIF4G2	18153.00	16485.00	19704.00	18114.00	19658.00
EIF4G3	3933.00	3759.00	4092.00	3928.00	3699.00
EIF4H	4720.00	4717.00	5005.00	4814.00	5747.00
EIF5	5314.00	5141.00	5469.00	5308.00	5681.00
EIF5A	10337.00	10063.00	10826.00	10408.67	14207.00
EIF5A2	256.00	263.00	330.00	283.00	298.00
EIF5AL1	332.00	278.00	331.00	313.67	466.00
EIF5B	5373.00	5194.00	5702.00	5423.00	7015.00
EIF6	4339.00	4247.00	4626.00	4404.00	5512.00
ELAC1	186.00	184.00	141.00	170.33	226.00
ELAC2	1603.00	1720.00	1638.00	1653.67	1838.00
ELAVL1	1853.00	1737.00	1875.00	1821.67	2300.00
ELAVL2	54.00	64.00	55.00	57.67	75.00
ELF1	669.00	566.00	642.00	625.67	560.00
ELF2	312.00	291.00	309.00	304.00	343.00
ELF4	363.00	334.00	337.00	344.67	296.00
ELFN1	9.00	11.00	3.00	7.67	8.00
ELFN1-AS1	38.00	58.00	45.00	47.00	67.00
ELFN2	3022.00	3013.00	3154.00	3063.00	2015.00
ELK1	753.00	730.00	786.00	756.33	897.00
ELK3	5447.00	4784.00	5510.00	5247.00	6694.00
ELK4	2022.00	1920.00	2204.00	2048.67	1851.00
ELL	724.00	663.00	713.00	700.00	758.00
ELL2	3273.00	2746.00	3606.00	3208.33	3827.00
ELL3	14.00	14.00	12.00	13.33	16.00
ELMO2	882.00	803.00	916.00	867.00	849.00
ELMO3	128.00	144.00	139.00	137.00	138.00
ELMOD1	130.00	144.00	136.00	136.67	168.00
ELMOD2	785.00	664.00	765.00	738.00	727.00
ELMOD3	645.00	672.00	660.00	659.00	687.00
ELMSAN1	949.00	775.00	923.00	882.33	770.00
ELOF1	625.00	650.00	728.00	667.67	751.00
ELOVL1	3626.00	3744.00	3700.00	3690.00	4441.00

ELOVL2	191.00	198.00	195.00	194.67	327.00
ELOVL3	8.00	11.00	15.00	11.33	21.00
ELOVL4	508.00	501.00	543.00	517.33	581.00
ELOVL5	4136.00	3942.00	4431.00	4169.67	4968.00
ELOVL6	1492.00	1405.00	1350.00	1415.67	1541.00
ELOVL7	61.00	62.00	58.00	60.33	62.00
ELP2	1272.00	1256.00	1387.00	1305.00	1421.00
ELP3	552.00	507.00	597.00	552.00	662.00
ELP4	230.00	236.00	280.00	248.67	294.00
ELP5	1284.00	1217.00	1324.00	1275.00	1660.00
ELP6	593.00	641.00	679.00	637.67	811.00
EMBP1	4.00	6.00	7.00	5.67	4.00
EMC1	4530.00	4501.00	4752.00	4594.33	4005.00
EMC10	1227.00	1300.00	1199.00	1242.00	1149.00
EMC2	756.00	690.00	806.00	750.67	996.00
EMC3	1135.00	1141.00	1036.00	1104.00	1185.00
EMC3-AS1	384.00	483.00	441.00	436.00	484.00
EMC4	1685.00	1731.00	1619.00	1678.33	2353.00
EMC6	592.00	582.00	591.00	588.33	735.00
EMC7	1698.00	1769.00	1585.00	1684.00	2086.00
EMC8	727.00	684.00	800.00	737.00	747.00
EMC9	219.00	232.00	207.00	219.33	246.00
EMD	1162.00	1177.00	1192.00	1177.00	1543.00
EME1	762.00	735.00	761.00	752.67	820.00
EME2	236.00	244.00	255.00	245.00	198.00
EMG1	896.00	858.00	962.00	905.33	1140.00
EMILIN1	271.00	268.00	237.00	258.67	184.00
EMILIN2	689.00	677.00	715.00	693.67	791.00
EML1	99.00	95.00	89.00	94.33	78.00
EML2	972.00	1047.00	1005.00	1008.00	980.00
EML2-AS1	202.00	192.00	208.00	200.67	127.00
EML3	1881.00	1914.00	2131.00	1975.33	1890.00
EML4	1394.00	1421.00	1574.00	1463.00	1508.00
EML5	76.00	51.00	72.00	66.33	69.00
EML6	219.00	200.00	202.00	207.00	154.00
EMP1	24554.00	23959.00	22949.00	23820.67	36952.00
EMP2	191.00	200.00	169.00	186.67	276.00
EMP3	1675.00	1591.00	1539.00	1601.67	2128.00
ENAH	1687.00	1482.00	1935.00	1701.33	1676.00
ENC1	5278.00	4880.00	5178.00	5112.00	5775.00
ENDOD1	818.00	830.00	826.00	824.67	634.00
ENDOG	185.00	125.00	171.00	160.33	206.00
ENDOU	3.00	9.00	2.00	4.67	11.00
ENDOV	371.00	401.00	459.00	410.33	347.00
ENG	16228.00	16187.00	15077.00	15830.67	12864.00
ENGASE	1002.00	1101.00	1116.00	1073.00	996.00
ENHO	4.00	4.00	2.00	3.33	10.00
ENKD1	471.00	542.00	505.00	506.00	613.00

ENKUR	9.00	11.00	9.00	9.67	13.00
ENO1	33552.00	31589.00	35324.00	33488.33	38214.00
ENO1-AS1	7.00	2.00	5.00	4.67	4.00
ENO2	1263.00	1433.00	1560.00	1418.67	1560.00
ENO3	636.00	629.00	700.00	655.00	812.00
ENOPH1	687.00	744.00	833.00	754.67	967.00
ENOSF1	3546.00	3491.00	3760.00	3599.00	4091.00
ENOX1	40.00	54.00	58.00	50.67	96.00
ENOX2	236.00	214.00	252.00	234.00	264.00
ENPEP	118.00	118.00	122.00	119.33	94.00
ENPP1	1880.00	1722.00	1739.00	1780.33	1787.00
ENPP2	51.00	42.00	50.00	47.67	98.00
ENPP3	5.00	6.00	1.00	4.00	3.00
ENPP4	349.00	348.00	363.00	353.33	304.00
ENSA	1640.00	1556.00	1683.00	1626.33	2033.00
ENTHD2	1151.00	1159.00	1199.00	1169.67	1167.00
ENTPD1-AS1	76.00	77.00	90.00	81.00	85.00
ENTPD3	498.00	463.00	518.00	493.00	125.00
ENTPD3-AS1	23.00	24.00	24.00	23.67	41.00
ENTPD4	3551.00	3436.00	3635.00	3540.67	3566.00
ENTPD5	263.00	265.00	235.00	254.33	216.00
ENTPD6	3182.00	3296.00	3220.00	3232.67	3144.00
ENTPD7	878.00	826.00	1009.00	904.33	996.00
ENY2	1004.00	957.00	1045.00	1002.00	1268.00
EOGT	1234.00	1208.00	1290.00	1244.00	1429.00
EP300	2240.00	1956.00	2388.00	2194.67	1594.00
EP300-AS1	3.00	2.00	5.00	3.33	3.00
EP400	2210.00	2147.00	2366.00	2241.00	1780.00
EP400NL	271.00	267.00	264.00	267.33	203.00
EPAS1	380.00	346.00	375.00	367.00	286.00
EPB41	890.00	901.00	1073.00	954.67	812.00
EPB41L1	567.00	519.00	555.00	547.00	562.00
EPB41L2	3597.00	3408.00	3495.00	3500.00	4042.00
EPB41L3	1605.00	1361.00	1688.00	1551.33	1945.00
EPB41L4A	10.00	14.00	27.00	17.00	20.00
EPB41L4A-AS1	683.00	676.00	734.00	697.67	845.00
EPB41L4B	109.00	90.00	118.00	105.67	145.00
EPB41L5	488.00	453.00	492.00	477.67	455.00
EPC1	790.00	714.00	887.00	797.00	752.00
EPC2	499.00	441.00	594.00	511.33	486.00
EPCAM	4.00	0.00	0.00	1.33	6.00
EPDR1	11.00	13.00	9.00	11.00	8.00
EPG5	6143.00	5720.00	6573.00	6145.33	6716.00
EPGN	4.00	8.00	8.00	6.67	6.00
EPHA1	3.00	1.00	3.00	2.33	0.00
EPHA2	16661.00	16783.00	16280.00	16574.67	13645.00
EPHA4	2244.00	2049.00	2231.00	2174.67	3299.00
EPHA5	117.00	92.00	135.00	114.67	132.00

EPHA5-AS1	6.00	5.00	6.00	5.67	25.00
EPHA7	17.00	30.00	34.00	27.00	31.00
EPHB1	121.00	121.00	121.00	121.00	162.00
EPHB2	3199.00	3180.00	3105.00	3161.33	1927.00
EPHB4	1955.00	1922.00	2013.00	1963.33	2023.00
EPHB6	24.00	13.00	14.00	17.00	7.00
EPHX1	565.00	575.00	514.00	551.33	481.00
EPHX3	4.00	3.00	3.00	3.33	6.00
EPHX4	206.00	247.00	213.00	222.00	286.00
EPM2A	125.00	144.00	140.00	136.33	112.00
EPM2AIP1	1374.00	1297.00	1443.00	1371.33	1139.00
EPN1	1863.00	1878.00	1976.00	1905.67	2113.00
EPN2	1333.00	1205.00	1320.00	1286.00	1087.00
EPOR	304.00	334.00	321.00	319.67	298.00
EPPK1	7.00	4.00	0.00	3.67	0.00
EPRS	9899.00	9035.00	10334.00	9756.00	9640.00
EPS15	1258.00	1123.00	1355.00	1245.33	1441.00
EPS15L1	881.00	800.00	834.00	838.33	795.00
EPS8	445.00	502.00	550.00	499.00	500.00
EPS8L2	15.00	15.00	14.00	14.67	20.00
EPT1	2336.00	2231.00	2420.00	2329.00	2191.00
ERAL1	1185.00	1084.00	1063.00	1110.67	1345.00
ERAP1	2425.00	2369.00	2597.00	2463.67	2403.00
ERAP2	272.00	296.00	318.00	295.33	281.00
ERBB2	48.00	59.00	35.00	47.33	76.00
ERBB2IP	2132.00	1851.00	2181.00	2054.67	2056.00
ERBB3	6.00	4.00	6.00	5.33	3.00
ERBB4	21.00	29.00	18.00	22.67	20.00
ERC1	1622.00	1448.00	1728.00	1599.33	1273.00
ERCC1	2412.00	2309.00	2521.00	2414.00	2442.00
ERCC2	2120.00	2003.00	2226.00	2116.33	1600.00
ERCC3	1445.00	1412.00	1613.00	1490.00	1644.00
ERCC4	494.00	439.00	557.00	496.67	425.00
ERCC5	58.00	59.00	66.00	61.00	49.00
ERCC6	280.00	291.00	326.00	299.00	285.00
ERCC6L	306.00	288.00	300.00	298.00	435.00
ERCC6L2	547.00	555.00	631.00	577.67	539.00
ERCC8	244.00	238.00	261.00	247.67	280.00
EREG	2051.00	1728.00	1983.00	1920.67	2508.00
ERF	1344.00	1378.00	1432.00	1384.67	1566.00
ERGIC1	7317.00	7140.00	7710.00	7389.00	8106.00
ERGIC2	1812.00	1673.00	1771.00	1752.00	2045.00
ERGIC3	5739.00	5589.00	5473.00	5600.33	6212.00
ERH	1639.00	1530.00	1592.00	1587.00	2593.00
ERI1	570.00	494.00	606.00	556.67	652.00
ERI2	487.00	439.00	502.00	476.00	591.00
ERI3	1235.00	1109.00	1246.00	1196.67	1374.00
ERI3-IT1	58.00	50.00	52.00	53.33	42.00

ERICH1	225.00	245.00	228.00	232.67	259.00
ERICH2	168.00	163.00	193.00	174.67	236.00
ERICH3	5.00	1.00	1.00	2.33	1.00
ERICH6	3.00	5.00	6.00	4.67	0.00
ERICH6-AS1	55.00	49.00	45.00	49.67	47.00
ERLEC1	1522.00	1419.00	1484.00	1475.00	1581.00
ERLIN1	2735.00	2676.00	2663.00	2691.33	3384.00
ERLIN2	4527.00	4040.00	4159.00	4242.00	3730.00
ERMAP	411.00	447.00	461.00	439.67	506.00
ERMP1	1073.00	1082.00	1177.00	1110.67	1074.00
ERN1	1096.00	870.00	1052.00	1006.00	570.00
ERO1A	3331.00	3397.00	3503.00	3410.33	4048.00
ERO1B	325.00	273.00	316.00	304.67	266.00
ERP29	5643.00	6090.00	5486.00	5739.67	7218.00
ERP44	1747.00	1728.00	1757.00	1744.00	1964.00
ERRFI1	17530.00	16098.00	18762.00	17463.33	17507.00
ERV3-1	28.00	21.00	28.00	25.67	22.00
ERVK13-1	244.00	220.00	239.00	234.33	216.00
ERVMER34-1	10.00	13.00	14.00	12.33	15.00
ESAM	194.00	196.00	213.00	201.00	118.00
ESCO1	364.00	346.00	366.00	358.67	374.00
ESCO2	79.00	68.00	81.00	76.00	105.00
ESD	1533.00	1506.00	1583.00	1540.67	1985.00
ESF1	1381.00	1375.00	1563.00	1439.67	1573.00
ESM1	18740.00	17505.00	17922.00	18055.67	27557.00
ESPL1	1588.00	1503.00	1535.00	1542.00	1854.00
ESPNL	7.00	5.00	3.00	5.00	10.00
ESR1	16.00	16.00	16.00	16.00	23.00
ESR2	21.00	28.00	18.00	22.33	22.00
ESRP1	14.00	11.00	14.00	13.00	7.00
ESRP2	11.00	13.00	14.00	12.67	25.00
ESRRA	2157.00	2125.00	2405.00	2229.00	2333.00
ESRRG	10.00	9.00	10.00	9.67	17.00
ESYT1	4239.00	4108.00	4045.00	4130.67	3628.00
ESYT2	4426.00	4005.00	4393.00	4274.67	4368.00
ESYT3	3.00	6.00	5.00	4.67	1.00
ETAA1	504.00	513.00	594.00	537.00	561.00
ETF1	3774.00	3461.00	3919.00	3718.00	4741.00
ETFA	1665.00	1507.00	1733.00	1635.00	1853.00
ETFB	607.00	688.00	697.00	664.00	740.00
ETFDH	383.00	372.00	395.00	383.33	427.00
ETHE1	476.00	490.00	548.00	504.67	588.00
ETNK1	2411.00	2173.00	2761.00	2448.33	2413.00
ETS1	5210.00	4538.00	5300.00	5016.00	5139.00
ETS2	1204.00	1164.00	1355.00	1241.00	1323.00
ETV1	1008.00	883.00	1008.00	966.33	1053.00
ETV2	9.00	4.00	5.00	6.00	9.00
ETV3	195.00	144.00	185.00	174.67	123.00

ETV4	770.00	710.00	837.00	772.33	947.00
ETV5	2784.00	2548.00	2484.00	2605.33	3113.00
ETV6	698.00	655.00	733.00	695.33	663.00
EVA1A	1465.00	1385.00	1405.00	1418.33	2025.00
EVA1B	319.00	288.00	389.00	332.00	355.00
EVA1C	65.00	65.00	74.00	68.00	72.00
EVC	5866.00	5443.00	6306.00	5871.67	3473.00
EVI2A	763.00	716.00	681.00	720.00	815.00
EVI2B	236.00	228.00	216.00	226.67	261.00
EVI5	753.00	692.00	748.00	731.00	760.00
EVI5L	573.00	542.00	542.00	552.33	633.00
EVL	458.00	445.00	462.00	455.00	545.00
EWSR1	7941.00	7877.00	8059.00	7959.00	9371.00
EXD1	8.00	4.00	3.00	5.00	8.00
EXD2	801.00	846.00	866.00	837.67	741.00
EXD3	434.00	493.00	511.00	479.33	401.00
EXO1	261.00	207.00	273.00	247.00	349.00
EXO5	81.00	96.00	85.00	87.33	89.00
EXOC1	976.00	824.00	1005.00	935.00	1014.00
EXOC2	1377.00	1414.00	1445.00	1412.00	1357.00
EXOC3	1158.00	1164.00	1246.00	1189.33	1096.00
EXOC3-AS1	86.00	76.00	57.00	73.00	95.00
EXOC4	1870.00	1826.00	1975.00	1890.33	1822.00
EXOC5	947.00	854.00	1007.00	936.00	988.00
EXOC6	277.00	257.00	320.00	284.67	299.00
EXOC6B	201.00	203.00	239.00	214.33	143.00
EXOC7	2446.00	2433.00	2489.00	2456.00	2113.00
EXOC8	593.00	574.00	622.00	596.33	539.00
EXOG	612.00	609.00	665.00	628.67	627.00
EXOSC1	701.00	691.00	768.00	720.00	963.00
EXOSC10	2294.00	2264.00	2462.00	2340.00	2365.00
EXOSC2	1133.00	1087.00	1147.00	1122.33	1265.00
EXOSC3	799.00	684.00	837.00	773.33	1042.00
EXOSC4	446.00	479.00	490.00	471.67	579.00
EXOSC5	572.00	562.00	563.00	565.67	670.00
EXOSC6	347.00	326.00	340.00	337.67	383.00
EXOSC7	834.00	918.00	902.00	884.67	1195.00
EXOSC8	1116.00	1079.00	1123.00	1106.00	1535.00
EXOSC9	616.00	593.00	587.00	598.67	692.00
EXPH5	142.00	117.00	145.00	134.67	116.00
EXT1	5693.00	5678.00	5852.00	5741.00	5119.00
EXT2	3520.00	3607.00	3706.00	3611.00	3266.00
EXTL2	2726.00	2570.00	2544.00	2613.33	3001.00
EXTL3	2033.00	2024.00	2044.00	2033.67	1680.00
EXTL3-AS1	13.00	7.00	3.00	7.67	4.00
EYA1	12.00	7.00	2.00	7.00	7.00
EYA3	769.00	689.00	741.00	733.00	713.00
EYA4	901.00	852.00	988.00	913.67	876.00

EYS	8.00	3.00	8.00	6.33	16.00
EZH1	2354.00	2535.00	2728.00	2539.00	2465.00
EZH2	856.00	830.00	815.00	833.67	1280.00
EZR	2696.00	2750.00	2739.00	2728.33	3294.00
F11-AS1	5.00	23.00	20.00	16.00	17.00
F11R	107.00	122.00	108.00	112.33	149.00
F12	69.00	60.00	86.00	71.67	80.00
F2R	4246.00	3891.00	4365.00	4167.33	2485.00
F2RL1	1089.00	997.00	1117.00	1067.67	475.00
F2RL2	151.00	155.00	185.00	163.67	176.00
F3	759.00	758.00	797.00	771.33	561.00
F8	100.00	71.00	89.00	86.67	107.00
F8A1	30.00	40.00	58.00	42.67	58.00
FAAH	15.00	9.00	19.00	14.33	10.00
FAAHP1	3.00	0.00	1.00	1.33	0.00
FAAP100	957.00	887.00	900.00	914.67	1079.00
FAAP20	1202.00	1229.00	1320.00	1250.33	1306.00
FAAP24	57.00	62.00	69.00	62.67	67.00
FABP5	437.00	466.00	431.00	444.67	1323.00
FABP6	10.00	12.00	20.00	14.00	58.00
FADD	3327.00	3311.00	3464.00	3367.33	4281.00
FADS1	3668.00	3460.00	3573.00	3567.00	3566.00
FADS2	1083.00	1067.00	1029.00	1059.67	1022.00
FADS3	713.00	845.00	819.00	792.33	550.00
FAF1	1520.00	1399.00	1530.00	1483.00	2058.00
FAF2	2771.00	2437.00	2547.00	2585.00	2592.00
FAH	1153.00	1046.00	1134.00	1111.00	1273.00
FAHD1	393.00	423.00	470.00	428.67	466.00
FAHD2A	738.00	768.00	710.00	738.67	756.00
FAHD2B	289.00	301.00	310.00	300.00	365.00
FAHD2CP	57.00	94.00	94.00	81.67	98.00
FAIM	226.00	219.00	239.00	228.00	255.00
FALEC	8.00	20.00	8.00	12.00	6.00
FAM101B	580.00	555.00	625.00	586.67	784.00
FAM102A	916.00	855.00	936.00	902.33	795.00
FAM102B	223.00	204.00	226.00	217.67	271.00
FAM103A1	157.00	134.00	200.00	163.67	185.00
FAM104A	661.00	701.00	751.00	704.33	745.00
FAM104B	213.00	171.00	175.00	186.33	330.00
FAM105A	60.00	59.00	55.00	58.00	33.00
FAM106A	3.00	5.00	4.00	4.00	3.00
FAM106B	5.00	5.00	5.00	5.00	5.00
FAM106CP	45.00	47.00	58.00	50.00	26.00
FAM107A	9.00	6.00	4.00	6.33	6.00
FAM107B	1099.00	901.00	1162.00	1054.00	824.00
FAM109A	293.00	292.00	286.00	290.33	215.00
FAM109B	98.00	136.00	88.00	107.33	93.00
FAM110A	147.00	149.00	115.00	137.00	204.00

FAM111A	577.00	510.00	566.00	551.00	778.00
FAM111B	82.00	75.00	69.00	75.33	152.00
FAM114A1	1404.00	1348.00	1453.00	1401.67	1331.00
FAM114A2	550.00	519.00	558.00	542.33	685.00
FAM117A	7.00	8.00	7.00	7.33	8.00
FAM117B	179.00	175.00	214.00	189.33	221.00
FAM118A	408.00	347.00	404.00	386.33	359.00
FAM118B	259.00	243.00	247.00	249.67	283.00
FAM120A	4526.00	4261.00	4935.00	4574.00	4603.00
FAM120AOS	312.00	336.00	344.00	330.67	434.00
FAM120C	409.00	362.00	425.00	398.67	411.00
FAM122A	278.00	294.00	364.00	312.00	276.00
FAM122B	987.00	1029.00	1031.00	1015.67	1135.00
FAM122C	24.00	30.00	24.00	26.00	37.00
FAM124A	23.00	25.00	33.00	27.00	40.00
FAM126A	1120.00	1000.00	1153.00	1091.00	1351.00
FAM126B	366.00	364.00	392.00	374.00	350.00
FAM127A	584.00	625.00	584.00	597.67	622.00
FAM127B	504.00	502.00	523.00	509.67	532.00
FAM127C	169.00	163.00	173.00	168.33	162.00
FAM129A	1420.00	1256.00	1396.00	1357.33	1616.00
FAM129B	12296.00	12109.00	12357.00	12254.00	11590.00
FAM129C	4.00	3.00	6.00	4.33	7.00
FAM131A	771.00	837.00	788.00	798.67	863.00
FAM131B	28.00	28.00	34.00	30.00	31.00
FAM131C	14.00	15.00	11.00	13.33	9.00
FAM132A	75.00	81.00	89.00	81.67	77.00
FAM132B	93.00	103.00	60.00	85.33	73.00
FAM133A	48.00	61.00	73.00	60.67	114.00
FAM133B	324.00	253.00	300.00	292.33	355.00
FAM133CP	12.00	12.00	13.00	12.33	24.00
FAM133DP	9.00	18.00	16.00	14.33	9.00
FAM134A	2468.00	2431.00	2453.00	2450.67	2563.00
FAM134B	37.00	39.00	41.00	39.00	43.00
FAM134C	1198.00	1131.00	1213.00	1180.67	1156.00
FAM135A	429.00	374.00	493.00	432.00	418.00
FAM136A	1213.00	1192.00	1293.00	1232.67	1868.00
FAM138B	14.00	12.00	5.00	10.33	5.00
FAM138E	3.00	6.00	0.00	3.00	6.00
FAM13A	409.00	369.00	462.00	413.33	410.00
FAM13A-AS1	27.00	40.00	49.00	38.67	30.00
FAM13B	859.00	793.00	839.00	830.33	797.00
FAM149A	19.00	14.00	20.00	17.67	29.00
FAM149B1	507.00	449.00	486.00	480.67	558.00
FAM151B	25.00	25.00	21.00	23.67	23.00
FAM155A	269.00	258.00	305.00	277.33	276.00
FAM156A	21.00	12.00	26.00	19.67	28.00
FAM160A1	36.00	32.00	26.00	31.33	20.00

FAM160A2	926.00	913.00	1067.00	968.67	864.00
FAM160B1	605.00	564.00	747.00	638.67	607.00
FAM160B2	1164.00	1144.00	1185.00	1164.33	1132.00
FAM161A	323.00	345.00	352.00	340.00	316.00
FAM161B	126.00	92.00	117.00	111.67	109.00
FAM162A	619.00	676.00	714.00	669.67	1070.00
FAM166B	19.00	13.00	21.00	17.67	11.00
FAM167A	536.00	508.00	630.00	558.00	585.00
FAM167B	19.00	17.00	27.00	21.00	10.00
FAM168A	1291.00	1270.00	1282.00	1281.00	1276.00
FAM168B	3988.00	3727.00	4303.00	4006.00	4236.00
FAM169A	109.00	99.00	125.00	111.00	126.00
FAM171A1	6236.00	6098.00	6370.00	6234.67	5681.00
FAM171A2	154.00	127.00	146.00	142.33	124.00
FAM171B	179.00	161.00	197.00	179.00	156.00
FAM172A	619.00	604.00	691.00	638.00	705.00
FAM172BP	11.00	9.00	11.00	10.33	6.00
FAM173A	315.00	304.00	312.00	310.33	307.00
FAM173B	281.00	296.00	288.00	288.33	413.00
FAM174A	240.00	212.00	250.00	234.00	273.00
FAM175A	525.00	529.00	578.00	544.00	552.00
FAM175B	608.00	580.00	628.00	605.33	694.00
FAM177A1	370.00	363.00	423.00	385.33	459.00
FAM179A	13.00	3.00	13.00	9.67	9.00
FAM179B	463.00	408.00	479.00	450.00	345.00
FAM180A	25.00	20.00	21.00	22.00	27.00
FAM182B	26.00	14.00	34.00	24.67	17.00
FAM184A	169.00	126.00	159.00	151.33	179.00
FAM185A	123.00	122.00	144.00	129.67	114.00
FAM186B	9.00	16.00	21.00	15.33	15.00
FAM188A	591.00	592.00	620.00	601.00	776.00
FAM188B	8.00	6.00	8.00	7.33	7.00
FAM189B	3.00	8.00	7.00	6.00	1.00
FAM192A	1914.00	1963.00	2310.00	2062.33	2155.00
FAM193A	601.00	544.00	614.00	586.33	588.00
FAM193B	1378.00	1456.00	1430.00	1421.33	1406.00
FAM195A	412.00	468.00	526.00	468.67	612.00
FAM195B	1630.00	1630.00	1632.00	1630.67	2004.00
FAM196B	1009.00	871.00	1071.00	983.67	696.00
FAM199X	1817.00	1700.00	2088.00	1868.33	1757.00
FAM19A1	3.00	1.00	8.00	4.00	3.00
FAM19A2	12.00	17.00	21.00	16.67	28.00
FAM200A	215.00	206.00	217.00	212.67	233.00
FAM200B	597.00	550.00	596.00	581.00	507.00
FAM201A	248.00	245.00	245.00	246.00	265.00
FAM204A	631.00	599.00	707.00	645.67	855.00
FAM206A	483.00	455.00	500.00	479.33	598.00
FAM207A	521.00	485.00	546.00	517.33	676.00

FAM208A	2769.00	2487.00	2962.00	2739.33	2811.00
FAM208B	4625.00	4172.00	4861.00	4552.67	4110.00
FAM20B	1520.00	1399.00	1531.00	1483.33	1572.00
FAM20C	2348.00	2591.00	2651.00	2530.00	1641.00
FAM210A	463.00	462.00	530.00	485.00	591.00
FAM210B	3041.00	2965.00	3235.00	3080.33	3803.00
FAM212A	3.00	0.00	1.00	1.33	3.00
FAM212B	30.00	36.00	39.00	35.00	17.00
FAM212B-AS1	4.00	3.00	4.00	3.67	0.00
FAM213A	1715.00	1770.00	1705.00	1730.00	1782.00
FAM214A	348.00	363.00	368.00	359.67	273.00
FAM214B	670.00	653.00	707.00	676.67	615.00
FAM216A	735.00	780.00	852.00	789.00	1054.00
FAM217B	408.00	380.00	460.00	416.00	478.00
FAM218A	21.00	34.00	31.00	28.67	29.00
FAM219A	796.00	722.00	817.00	778.33	812.00
FAM219B	2173.00	2132.00	2345.00	2216.67	2011.00
FAM21A	2026.00	2051.00	2202.00	2093.00	1908.00
FAM21C	1686.00	1677.00	1795.00	1719.33	1543.00
FAM21EP	9.00	13.00	6.00	9.33	5.00
FAM220A	449.00	372.00	492.00	437.67	528.00
FAM221A	107.00	78.00	94.00	93.00	110.00
FAM222A	10.00	4.00	5.00	6.33	6.00
FAM222B	344.00	307.00	355.00	335.33	393.00
FAM225A	13.00	12.00	22.00	15.67	24.00
FAM225B	3.00	12.00	2.00	5.67	4.00
FAM227A	311.00	335.00	323.00	323.00	286.00
FAM227B	46.00	47.00	47.00	46.67	43.00
FAM228B	133.00	173.00	171.00	159.00	166.00
FAM229A	82.00	92.00	96.00	90.00	79.00
FAM229B	182.00	154.00	182.00	172.67	216.00
FAM24B	311.00	246.00	295.00	284.00	345.00
FAM26E	83.00	101.00	75.00	86.33	126.00
FAM26F	46.00	56.00	61.00	54.33	98.00
FAM27B	14.00	19.00	10.00	14.33	17.00
FAM27E3	34.00	29.00	41.00	34.67	50.00
FAM32A	883.00	943.00	923.00	916.33	1105.00
FAM35A	667.00	598.00	731.00	665.33	735.00
FAM35BP	16.00	15.00	22.00	17.67	22.00
FAM35DP	5.00	17.00	11.00	11.00	18.00
FAM3A	844.00	837.00	797.00	826.00	836.00
FAM3C	1209.00	1229.00	1310.00	1249.33	1614.00
FAM43A	483.00	467.00	555.00	501.67	458.00
FAM45A	319.00	304.00	385.00	336.00	416.00
FAM45B	67.00	63.00	49.00	59.67	66.00
FAM46A	223.00	205.00	276.00	234.67	195.00
FAM46B	37.00	30.00	29.00	32.00	28.00
FAM46D	5.00	3.00	8.00	5.33	5.00

FAM47E	8.00	2.00	1.00	3.67	1.00
FAM49A	12.00	21.00	13.00	15.33	6.00
FAM49B	1306.00	1255.00	1317.00	1292.67	1665.00
FAM50A	2128.00	2276.00	2331.00	2245.00	2397.00
FAM50B	175.00	169.00	168.00	170.67	267.00
FAM53A	121.00	129.00	167.00	139.00	193.00
FAM53B	529.00	580.00	635.00	581.33	632.00
FAM53C	1293.00	1145.00	1312.00	1250.00	1150.00
FAM57A	918.00	952.00	893.00	921.00	988.00
FAM58A	206.00	214.00	272.00	230.67	349.00
FAM60A	8.00	2.00	3.00	4.33	10.00
FAM63A	236.00	238.00	230.00	234.67	226.00
FAM63B	140.00	110.00	170.00	140.00	108.00
FAM64A	243.00	212.00	184.00	213.00	292.00
FAM65A	2630.00	2586.00	2744.00	2653.33	2242.00
FAM65C	5.00	6.00	4.00	5.00	7.00
FAM66C	84.00	107.00	92.00	94.33	99.00
FAM66D	42.00	39.00	32.00	37.67	33.00
FAM66E	3.00	0.00	1.00	1.33	1.00
FAM69A	264.00	240.00	249.00	251.00	214.00
FAM71E2	5.00	1.00	2.00	2.67	0.00
FAM71F2	286.00	298.00	342.00	308.67	304.00
FAM72A	135.00	115.00	116.00	122.00	171.00
FAM72B	171.00	191.00	217.00	193.00	299.00
FAM72C	23.00	35.00	35.00	31.00	47.00
FAM72D	63.00	38.00	44.00	48.33	62.00
FAM73A	738.00	610.00	682.00	676.67	676.00
FAM73B	1480.00	1472.00	1442.00	1464.67	1402.00
FAM76A	157.00	138.00	172.00	155.67	160.00
FAM76B	695.00	675.00	739.00	703.00	762.00
FAM78A	54.00	73.00	99.00	75.33	83.00
FAM78B	4.00	4.00	3.00	3.67	4.00
FAM81A	139.00	134.00	175.00	149.33	210.00
FAM83A	4.00	3.00	3.00	3.33	1.00
FAM83B	9.00	11.00	11.00	10.33	13.00
FAM83D	604.00	521.00	598.00	574.33	771.00
FAM83E	4.00	0.00	4.00	2.67	5.00
FAM83G	662.00	653.00	759.00	691.33	573.00
FAM84A	50.00	38.00	51.00	46.33	107.00
FAM84B	345.00	286.00	359.00	330.00	291.00
FAM86B1	5.00	3.00	6.00	4.67	1.00
FAM86B2	5.00	5.00	2.00	4.00	3.00
FAM86B3P	448.00	416.00	449.00	437.67	352.00
FAM86C1	512.00	498.00	508.00	506.00	554.00
FAM86C2P	88.00	78.00	79.00	81.67	111.00
FAM86DP	404.00	410.00	419.00	411.00	331.00
FAM86EP	245.00	205.00	218.00	222.67	238.00
FAM86FP	47.00	58.00	51.00	52.00	46.00

FAM86HP	165.00	197.00	160.00	174.00	149.00
FAM86JP	90.00	82.00	90.00	87.33	96.00
FAM89A	230.00	231.00	261.00	240.67	281.00
FAM89B	636.00	729.00	673.00	679.33	752.00
FAM8A1	577.00	558.00	602.00	579.00	677.00
FAM91A1	1733.00	1485.00	1836.00	1684.67	1764.00
FAM92A1	683.00	693.00	729.00	701.67	823.00
FAM95B1	12.00	8.00	10.00	10.00	8.00
FAM95C	496.00	493.00	589.00	526.00	532.00
FAM96A	785.00	793.00	819.00	799.00	1051.00
FAM96B	656.00	701.00	724.00	693.67	949.00
FAM98A	1597.00	1442.00	1576.00	1538.33	1737.00
FAM98B	870.00	825.00	878.00	857.67	1122.00
FAM98C	234.00	216.00	241.00	230.33	280.00
FAN1	426.00	424.00	424.00	424.67	455.00
FANCA	1565.00	1579.00	1461.00	1535.00	1704.00
FANCB	91.00	64.00	70.00	75.00	72.00
FANCC	359.00	309.00	356.00	341.33	404.00
FANCD2	898.00	882.00	813.00	864.33	1020.00
FANCE	283.00	307.00	319.00	303.00	367.00
FANCF	432.00	405.00	460.00	432.33	567.00
FANCG	976.00	1040.00	913.00	976.33	1286.00
FANCI	1287.00	1201.00	1159.00	1215.67	1721.00
FANCL	595.00	642.00	642.00	626.33	827.00
FANCM	300.00	280.00	309.00	296.33	306.00
FANK1	18.00	9.00	12.00	13.00	5.00
FAP	209.00	176.00	200.00	195.00	191.00
FAR1	1752.00	1579.00	1928.00	1753.00	1982.00
FAR2	1268.00	1254.00	1363.00	1295.00	1161.00
FAR2P2	3.00	0.00	1.00	1.33	1.00
FARP1	680.00	722.00	693.00	698.33	789.00
FARP2	203.00	191.00	203.00	199.00	159.00
FARS2	196.00	185.00	212.00	197.67	288.00
FARSA	1728.00	1743.00	1795.00	1755.33	2016.00
FARSB	2623.00	2409.00	2834.00	2622.00	2902.00
FAS	134.00	127.00	101.00	120.67	141.00
FASLG	25.00	30.00	19.00	24.67	20.00
FASN	10535.00	10177.00	10780.00	10497.33	9370.00
FASTK	2398.00	2488.00	2432.00	2439.33	2793.00
FASTKD1	1188.00	1180.00	1301.00	1223.00	1250.00
FASTKD2	2120.00	1926.00	2222.00	2089.33	2016.00
FASTKD3	433.00	446.00	459.00	446.00	479.00
FASTKD5	557.00	581.00	540.00	559.33	616.00
FAT1	2217.00	2052.00	2237.00	2168.67	1744.00
FAT3	3.00	1.00	3.00	2.33	1.00
FAT4	665.00	626.00	712.00	667.67	1217.00
FAU	8415.00	8625.00	8929.00	8656.33	10774.00
FAXC	54.00	47.00	59.00	53.33	64.00

FAXDC2	14.00	22.00	19.00	18.33	13.00
FBF1	667.00	664.00	738.00	689.67	743.00
FBL	2342.00	2432.00	2491.00	2421.67	3646.00
FBLIM1	1556.00	1498.00	1642.00	1565.33	1130.00
FBLN2	25.00	29.00	38.00	30.67	33.00
FBLN7	35.00	30.00	38.00	34.33	20.00
FBN1	2982.00	2666.00	3321.00	2989.67	1609.00
FBN2	10489.00	9487.00	11144.00	10373.33	5081.00
FBRS	2306.00	2223.00	2438.00	2322.33	1933.00
FBRSL1	1129.00	1057.00	1163.00	1116.33	1259.00
FBXL12	461.00	439.00	457.00	452.33	508.00
FBXL13	15.00	12.00	29.00	18.67	19.00
FBXL14	369.00	342.00	406.00	372.33	393.00
FBXL15	404.00	398.00	431.00	411.00	426.00
FBXL17	596.00	475.00	599.00	556.67	538.00
FBXL18	827.00	724.00	887.00	812.67	735.00
FBXL19	1196.00	1314.00	1401.00	1303.67	1204.00
FBXL19-AS1	784.00	679.00	809.00	757.33	509.00
FBXL2	375.00	400.00	369.00	381.33	431.00
FBXL20	487.00	468.00	537.00	497.33	465.00
FBXL21	12.00	11.00	20.00	14.33	9.00
FBXL22	8.00	6.00	3.00	5.67	9.00
FBXL3	674.00	609.00	783.00	688.67	725.00
FBXL4	682.00	607.00	666.00	651.67	800.00
FBXL5	596.00	559.00	663.00	606.00	542.00
FBXL6	1043.00	1095.00	1155.00	1097.67	1187.00
FBXL7	203.00	192.00	237.00	210.67	245.00
FBXL8	316.00	324.00	363.00	334.33	275.00
FBXO10	562.00	481.00	502.00	515.00	490.00
FBXO11	1464.00	1412.00	1654.00	1510.00	1787.00
FBXO15	22.00	16.00	18.00	18.67	21.00
FBXO16	37.00	42.00	43.00	40.67	44.00
FBXO17	813.00	844.00	927.00	861.33	964.00
FBXO18	2841.00	2712.00	2930.00	2827.67	2834.00
FBXO21	580.00	472.00	534.00	528.67	472.00
FBXO22	717.00	657.00	785.00	719.67	867.00
FBXO24	14.00	12.00	11.00	12.33	21.00
FBXO25	93.00	68.00	122.00	94.33	129.00
FBXO28	976.00	877.00	1088.00	980.33	1060.00
FBXO3	350.00	304.00	355.00	336.33	349.00
FBXO30	1030.00	857.00	1080.00	989.00	962.00
FBXO31	1557.00	1588.00	1639.00	1594.67	1663.00
FBXO32	561.00	531.00	579.00	557.00	724.00
FBXO33	222.00	254.00	281.00	252.33	291.00
FBXO34	597.00	530.00	641.00	589.33	686.00
FBXO36	72.00	89.00	105.00	88.67	79.00
FBXO38	1063.00	1020.00	1158.00	1080.33	1065.00
FBXO4	333.00	307.00	358.00	332.67	482.00

FBXO41	905.00	976.00	974.00	951.67	917.00
FBXO42	574.00	546.00	599.00	573.00	625.00
FBXO43	203.00	203.00	184.00	196.67	254.00
FBXO44	341.00	411.00	411.00	387.67	420.00
FBXO45	617.00	536.00	646.00	599.67	682.00
FBXO46	474.00	490.00	580.00	514.67	602.00
FBXO48	66.00	58.00	76.00	66.67	45.00
FBXO5	381.00	349.00	391.00	373.67	510.00
FBXO6	8.00	11.00	17.00	12.00	32.00
FBXO7	1175.00	1168.00	1270.00	1204.33	1295.00
FBXO8	149.00	177.00	221.00	182.33	203.00
FBXO9	1410.00	1240.00	1469.00	1373.00	1391.00
FBXW11	1714.00	1496.00	1920.00	1710.00	1476.00
FBXW2	1832.00	1755.00	1991.00	1859.33	1746.00
FBXW4	759.00	755.00	790.00	768.00	859.00
FBXW4P1	5.00	5.00	8.00	6.00	2.00
FBXW5	1840.00	1827.00	2008.00	1891.67	2188.00
FBXW7	287.00	306.00	324.00	305.67	334.00
FBXW8	773.00	723.00	796.00	764.00	630.00
FBXW9	283.00	244.00	284.00	270.33	272.00
FCER1G	29.00	23.00	13.00	21.67	18.00
FCF1	877.00	806.00	923.00	868.67	1046.00
FCGBP	15.00	7.00	13.00	11.67	12.00
FCGRT	496.00	544.00	520.00	520.00	548.00
FCHO1	9.00	18.00	20.00	15.67	5.00
FCHO2	667.00	615.00	746.00	676.00	740.00
FCHSD1	1065.00	1144.00	1173.00	1127.33	934.00
FCHSD2	694.00	617.00	714.00	675.00	656.00
FCMR	20.00	19.00	18.00	19.00	8.00
FCRLA	9.00	13.00	14.00	12.00	3.00
FCRLB	105.00	82.00	96.00	94.33	103.00
FDFT1	1179.00	1096.00	1175.00	1150.00	1171.00
FDPS	2313.00	2344.00	2365.00	2340.67	2944.00
FDX1	581.00	531.00	585.00	565.67	654.00
FDX1L	367.00	388.00	371.00	375.33	402.00
FDXACB1	182.00	182.00	205.00	189.67	264.00
FDXR	182.00	191.00	204.00	192.33	165.00
FECH	288.00	295.00	292.00	291.67	317.00
FEM1A	728.00	720.00	755.00	734.33	848.00
FEM1B	1048.00	1010.00	1286.00	1114.67	1145.00
FEM1C	670.00	594.00	700.00	654.67	571.00
FEN1	826.00	783.00	818.00	809.00	1083.00
FENDRR	3.00	3.00	5.00	3.67	7.00
FER	690.00	604.00	757.00	683.67	672.00
FER1L4	85.00	91.00	90.00	88.67	42.00
FER1L6	15.00	21.00	17.00	17.67	1.00
FERMT1	1139.00	1085.00	1276.00	1166.67	1343.00
FERMT2	1738.00	1599.00	1916.00	1751.00	1605.00

FERMT3	577.00	554.00	557.00	562.67	373.00
FES	8.00	6.00	5.00	6.33	4.00
FEZ1	6.00	11.00	5.00	7.33	11.00
FEZ2	2461.00	2147.00	2632.00	2413.33	3937.00
FGD1	705.00	618.00	707.00	676.67	713.00
FGD4	11.00	12.00	12.00	11.67	13.00
FGD5-AS1	2055.00	1934.00	2190.00	2059.67	2406.00
FGD6	348.00	328.00	344.00	340.00	474.00
FGF1	108.00	78.00	84.00	90.00	213.00
FGF11	25.00	20.00	14.00	19.67	10.00
FGF12	65.00	75.00	68.00	69.33	99.00
FGF13	98.00	76.00	118.00	97.33	129.00
FGF18	16.00	6.00	10.00	10.67	4.00
FGF2	881.00	696.00	926.00	834.33	659.00
FGF20	3.00	1.00	4.00	2.67	4.00
FGF4	37.00	39.00	51.00	42.33	36.00
FGF5	2425.00	2167.00	2581.00	2391.00	2854.00
FGFBP3	126.00	117.00	112.00	118.33	140.00
FGFR1	4821.00	4946.00	5066.00	4944.33	3323.00
FGFR1OP	929.00	909.00	953.00	930.33	1060.00
FGFR1OP2	974.00	859.00	1022.00	951.67	1165.00
FGFR4	13.00	6.00	8.00	9.00	11.00
FGFRL1	478.00	534.00	573.00	528.33	465.00
FGGY	280.00	289.00	306.00	291.67	380.00
FH	1443.00	1455.00	1482.00	1460.00	1899.00
FHAD1	38.00	30.00	29.00	32.33	32.00
FHDC1	61.00	46.00	55.00	54.00	43.00
FHIT	98.00	77.00	98.00	91.00	81.00
FHL1	1220.00	1085.00	1236.00	1180.33	1053.00
FHL2	3630.00	3492.00	3995.00	3705.67	3467.00
FHL3	245.00	246.00	277.00	256.00	280.00
FHOD1	1488.00	1555.00	1589.00	1544.00	1692.00
FHOD3	608.00	592.00	703.00	634.33	832.00
FIBCD1	8.00	5.00	11.00	8.00	3.00
FIBP	1921.00	1788.00	1994.00	1901.00	2292.00
FICD	135.00	121.00	138.00	131.33	113.00
FIG4	704.00	655.00	733.00	697.33	645.00
FIGN	481.00	460.00	520.00	487.00	401.00
FIGNL1	1056.00	938.00	984.00	992.67	1026.00
FIGNL2	12.00	8.00	13.00	11.00	4.00
FIP1L1	1041.00	1039.00	1193.00	1091.00	1296.00
FIRRE	232.00	224.00	271.00	242.33	170.00
FIS1	664.00	694.00	668.00	675.33	736.00
FITM1	4.00	3.00	5.00	4.00	3.00
FITM2	82.00	88.00	97.00	89.00	74.00
FIZ1	223.00	216.00	256.00	231.67	299.00
FJX1	3734.00	3695.00	3842.00	3757.00	4373.00
FKBP10	14626.00	14924.00	14739.00	14763.00	12942.00

FKBP11	2091.00	2088.00	1963.00	2047.33	2334.00
FKBP14	1326.00	1211.00	1341.00	1292.67	1133.00
FKBP15	1699.00	1655.00	1805.00	1719.67	1659.00
FKBP1A	7545.00	7037.00	7201.00	7261.00	7881.00
FKBP1AP1	37.00	41.00	39.00	39.00	63.00
FKBP1B	10.00	13.00	5.00	9.33	16.00
FKBP2	1483.00	1680.00	1514.00	1559.00	1840.00
FKBP3	1421.00	1410.00	1529.00	1453.33	1832.00
FKBP4	3526.00	3337.00	3732.00	3531.67	4309.00
FKBP5	436.00	380.00	456.00	424.00	729.00
FKBP7	545.00	492.00	505.00	514.00	566.00
FKBP8	3591.00	3528.00	4180.00	3766.33	4122.00
FKBP9	1909.00	1983.00	1936.00	1942.67	1751.00
FKBP9P1	42.00	40.00	32.00	38.00	33.00
FKRP	959.00	835.00	910.00	901.33	762.00
FKTN	1822.00	1593.00	1806.00	1740.33	1629.00
FLAD1	1181.00	1154.00	1232.00	1189.00	1279.00
FLCN	1215.00	1220.00	1289.00	1241.33	1161.00
FLG-AS1	378.00	366.00	332.00	358.67	370.00
FLI1	832.00	852.00	948.00	877.33	932.00
FLII	4070.00	4053.00	4351.00	4158.00	3654.00
FLJ10038	298.00	285.00	312.00	298.33	322.00
FLJ20021	28.00	37.00	41.00	35.33	33.00
FLJ21408	32.00	25.00	27.00	28.00	17.00
FLJ22447	123.00	80.00	96.00	99.67	46.00
FLJ23867	1071.00	1133.00	1063.00	1089.00	709.00
FLJ27354	31.00	20.00	28.00	26.33	17.00
FLJ30679	3.00	3.00	2.00	2.67	1.00
FLJ31104	28.00	23.00	35.00	28.67	33.00
FLJ31356	11.00	13.00	16.00	13.33	13.00
FLJ32255	36.00	24.00	38.00	32.67	31.00
FLJ37035	35.00	27.00	35.00	32.33	34.00
FLJ37201	70.00	67.00	68.00	68.33	78.00
FLJ37453	74.00	87.00	97.00	86.00	68.00
FLJ41200	87.00	108.00	97.00	97.33	80.00
FLJ42351	105.00	80.00	95.00	93.33	103.00
FLJ42627	123.00	120.00	126.00	123.00	93.00
FLJ45513	14.00	30.00	34.00	26.00	35.00
FLJ46906	282.00	308.00	325.00	305.00	376.00
FLNA	40919.00	39191.00	43079.00	41063.00	29995.00
FLNB	17827.00	16972.00	17902.00	17567.00	14733.00
FLNC	15968.00	15739.00	15940.00	15882.33	5783.00
FLOT1	10.00	6.00	17.00	11.00	14.00
FLOT2	849.00	844.00	856.00	849.67	907.00
FLRT2	690.00	685.00	749.00	708.00	728.00
FLRT3	13.00	16.00	24.00	17.67	6.00
FLT3LG	66.00	67.00	60.00	64.33	71.00
FLVCR1	963.00	914.00	969.00	948.67	951.00

FLVCR1-AS1	14.00	16.00	21.00	17.00	21.00
FLVCR2	7.00	19.00	14.00	13.33	12.00
FLYWCH1	2623.00	2645.00	2608.00	2625.33	2682.00
FLYWCH2	387.00	367.00	404.00	386.00	446.00
FMN1	491.00	422.00	498.00	470.33	324.00
FMN2	211.00	159.00	218.00	196.00	278.00
FMNL1	1708.00	1727.00	1722.00	1719.00	1008.00
FMNL2	2855.00	2697.00	2978.00	2843.33	3222.00
FMNL3	2404.00	2259.00	2540.00	2401.00	1879.00
FMO4	60.00	70.00	58.00	62.67	84.00
FMR1	722.00	661.00	709.00	697.33	734.00
FN1	65121.00	59217.00	69496.00	64611.33	76876.00
FN3K	68.00	59.00	66.00	64.33	69.00
FN3KRP	593.00	539.00	606.00	579.33	640.00
FNBP1	1122.00	946.00	1116.00	1061.33	1061.00
FNBP1L	736.00	695.00	786.00	739.00	831.00
FNBP4	3482.00	3448.00	3796.00	3575.33	3229.00
FNDC3A	1564.00	1401.00	1729.00	1564.67	1683.00
FNDC3B	1606.00	1340.00	1600.00	1515.33	1310.00
FNDC4	75.00	95.00	111.00	93.67	72.00
FNDC7	5.00	2.00	0.00	2.33	3.00
FNIP1	1174.00	1001.00	1216.00	1130.33	1043.00
FNIP2	156.00	172.00	180.00	169.33	149.00
FNTA	1272.00	1249.00	1431.00	1317.33	1519.00
FNTB	23.00	24.00	21.00	22.67	32.00
FOCAD	1858.00	1725.00	1774.00	1785.67	1723.00
FOLH1	4.00	4.00	3.00	3.67	3.00
FOPNL	48.00	51.00	48.00	49.00	68.00
FOS	29.00	45.00	38.00	37.33	32.00
FOSB	118.00	113.00	118.00	116.33	107.00
FOSL1	14136.00	13800.00	14620.00	14185.33	14209.00
FOSL2	2910.00	2939.00	3237.00	3028.67	2526.00
FOXA1	24.00	29.00	24.00	25.67	28.00
FOXA2	874.00	927.00	1019.00	940.00	735.00
FOXC1	320.00	307.00	351.00	326.00	330.00
FOXC2	118.00	105.00	123.00	115.33	164.00
FOXC2-AS1	22.00	21.00	21.00	21.33	39.00
FOXD1	2874.00	2938.00	3002.00	2938.00	4973.00
FOXD2	9.00	13.00	11.00	11.00	19.00
FOXD2-AS1	82.00	93.00	84.00	86.33	82.00
FOXD3	36.00	41.00	31.00	36.00	30.00
FOXD3-AS1	63.00	68.00	53.00	61.33	64.00
FOXD4	21.00	20.00	29.00	23.33	14.00
FOXD4L1	9.00	14.00	7.00	10.00	11.00
FOXF1	232.00	233.00	267.00	244.00	540.00
FOXF2	523.00	562.00	586.00	557.00	718.00
FOXG1	110.00	114.00	111.00	111.67	129.00
FOXH1	16.00	24.00	23.00	21.00	18.00

FOXJ2	852.00	772.00	900.00	841.33	663.00
FOXJ3	2658.00	2500.00	2811.00	2656.33	2497.00
FOXK1	3164.00	2926.00	2476.00	2855.33	2285.00
FOXK2	2599.00	2511.00	2705.00	2605.00	2865.00
FOXL1	1337.00	1277.00	1423.00	1345.67	1458.00
FOXM1	2326.00	2205.00	2113.00	2214.67	2661.00
FOXN2	630.00	530.00	635.00	598.33	756.00
FOXN3	697.00	622.00	771.00	696.67	592.00
FOXN3-AS1	35.00	25.00	21.00	27.00	43.00
FOXO1	169.00	180.00	200.00	183.00	184.00
FOXO3	767.00	720.00	803.00	763.33	645.00
FOXO3B	309.00	281.00	317.00	302.33	313.00
FOXO4	229.00	260.00	285.00	258.00	205.00
FOXO6	3.00	4.00	11.00	6.00	0.00
FOXP1	1620.00	1526.00	1818.00	1654.67	1731.00
FOXP2	137.00	138.00	177.00	150.67	117.00
FOXP3	3.00	0.00	1.00	1.33	0.00
FOXP4	852.00	909.00	1007.00	922.67	921.00
FOXP4-AS1	26.00	21.00	19.00	22.00	36.00
FOXQ1	4.00	2.00	1.00	2.33	4.00
FOXRED1	726.00	726.00	798.00	750.00	748.00
FOXRED2	1019.00	1010.00	1021.00	1016.67	980.00
FPGS	1146.00	1207.00	1194.00	1182.33	1174.00
FPGT	248.00	223.00	254.00	241.67	281.00
FRA10AC1	978.00	975.00	1066.00	1006.33	1164.00
FRAS1	817.00	852.00	942.00	870.33	445.00
FRAT1	83.00	85.00	97.00	88.33	115.00
FRAT2	242.00	218.00	257.00	239.00	303.00
FREM1	3.00	0.00	2.00	1.67	3.00
FREM2	48.00	49.00	50.00	49.00	43.00
FRG1	3.00	2.00	6.00	3.67	1.00
FRG1BP	248.00	230.00	245.00	241.00	250.00
FRG1CP	368.00	390.00	403.00	387.00	317.00
FRG1DP	70.00	58.00	66.00	64.67	66.00
FRG1HP	340.00	320.00	387.00	349.00	364.00
FRG1JP	67.00	74.00	90.00	77.00	82.00
FRK	9.00	4.00	6.00	6.33	11.00
FRMD3	30.00	20.00	30.00	26.67	28.00
FRMD4A	3693.00	3529.00	3845.00	3689.00	2786.00
FRMD4B	233.00	166.00	188.00	195.67	256.00
FRMD5	1047.00	945.00	1070.00	1020.67	866.00
FRMD6	3757.00	3513.00	4289.00	3853.00	4695.00
FRMD6-AS1	17.00	14.00	16.00	15.67	16.00
FRMD8	1204.00	1236.00	1134.00	1191.33	1278.00
FRMPD2	3.00	1.00	2.00	2.00	0.00
FRMPD4	8.00	7.00	10.00	8.33	9.00
FRRS1	120.00	122.00	173.00	138.33	151.00
FRS2	408.00	423.00	464.00	431.67	433.00

FRS3	188.00	152.00	235.00	191.67	154.00
FRY	118.00	104.00	134.00	118.67	131.00
FRYL	1674.00	1504.00	1630.00	1602.67	1570.00
FRZB	16.00	16.00	15.00	15.67	12.00
FSCN1	1503.00	1547.00	1680.00	1576.67	2192.00
FSD1	768.00	770.00	773.00	770.33	905.00
FSD1L	211.00	184.00	219.00	204.67	198.00
FSIP1	12.00	20.00	19.00	17.00	21.00
FSIP2	81.00	75.00	80.00	78.67	32.00
FST	2564.00	2325.00	2583.00	2490.67	3640.00
FSTL1	5931.00	5787.00	5681.00	5799.67	5694.00
FSTL3	453.00	473.00	512.00	479.33	332.00
FTCD	21.00	12.00	17.00	16.67	10.00
FTCDNL1	30.00	38.00	24.00	30.67	43.00
FTH1	6401.00	6379.00	6836.00	6538.67	7264.00
FTH1P3	79.00	63.00	66.00	69.33	72.00
FTL	14798.00	15157.00	16649.00	15534.67	13052.00
FTO	749.00	717.00	765.00	743.67	743.00
FTO-IT1	6.00	7.00	7.00	6.67	3.00
FTSJ1	1162.00	1191.00	1152.00	1168.33	1236.00
FTSJ2	639.00	638.00	708.00	661.67	894.00
FTSJ3	2464.00	2453.00	2480.00	2465.67	2519.00
FTX	42.00	38.00	42.00	40.67	39.00
FUBP1	3838.00	3551.00	4034.00	3807.67	3627.00
FUBP3	1732.00	1642.00	1928.00	1767.33	1739.00
FUCA1	145.00	118.00	124.00	129.00	165.00
FUCA2	5411.00	5416.00	5089.00	5305.33	5487.00
FUK	594.00	589.00	658.00	613.67	574.00
FUNDC1	250.00	207.00	254.00	237.00	316.00
FUNDC2	327.00	377.00	393.00	365.67	448.00
FUOM	42.00	35.00	38.00	38.33	52.00
FURIN	2217.00	2212.00	2289.00	2239.33	1733.00
FUS	9417.00	9965.00	9999.00	9793.67	12138.00
FUT1	8.00	3.00	4.00	5.00	8.00
FUT10	145.00	121.00	153.00	139.67	160.00
FUT11	221.00	181.00	232.00	211.33	160.00
FUT4	407.00	442.00	386.00	411.67	414.00
FUT8	3891.00	3934.00	4082.00	3969.00	4756.00
FUT8-AS1	181.00	176.00	162.00	173.00	214.00
FUZ	401.00	379.00	419.00	399.67	540.00
FXN	241.00	237.00	246.00	241.33	315.00
FXR1	3519.00	3388.00	3793.00	3566.67	4524.00
FXR2	1377.00	1444.00	1646.00	1489.00	1403.00
FXYD5	2795.00	2765.00	2685.00	2748.33	3463.00
FYB	4.00	0.00	0.00	1.33	1.00
FYCO1	1422.00	1197.00	1396.00	1338.33	1080.00
FYN	2337.00	2183.00	2182.00	2234.00	2537.00
FYTTD1	1057.00	1027.00	1123.00	1069.00	1137.00

FZD1	444.00	417.00	396.00	419.00	394.00
FZD2	1145.00	1173.00	1194.00	1170.67	1217.00
FZD3	379.00	345.00	420.00	381.33	327.00
FZD4	191.00	211.00	218.00	206.67	151.00
FZD5	184.00	172.00	202.00	186.00	180.00
FZD6	2857.00	2626.00	2965.00	2816.00	2831.00
FZD7	349.00	342.00	379.00	356.67	304.00
FZD8	881.00	887.00	932.00	900.00	854.00
FZD9	9.00	4.00	7.00	6.67	4.00
FZR1	900.00	842.00	892.00	878.00	854.00
G0S2	6820.00	7039.00	6563.00	6807.33	8080.00
G2E3	804.00	743.00	853.00	800.00	806.00
G3BP1	4115.00	3584.00	4049.00	3916.00	4611.00
G3BP2	1918.00	1740.00	2076.00	1911.33	1574.00
G6PC3	2145.00	2219.00	1990.00	2118.00	2413.00
G6PD	1095.00	1075.00	1083.00	1084.33	1076.00
GAA	4659.00	4682.00	4678.00	4673.00	4472.00
GAB1	236.00	217.00	251.00	234.67	179.00
GAB2	409.00	373.00	436.00	406.00	298.00
GAB3	7.00	6.00	7.00	6.67	13.00
GABARAP	2287.00	2270.00	2374.00	2310.33	3014.00
GABARAPL1	568.00	557.00	644.00	589.67	663.00
GABARAPL2	809.00	785.00	887.00	827.00	1028.00
GABBR2	26.00	10.00	25.00	20.33	25.00
GABPA	770.00	727.00	883.00	793.33	842.00
GABPB1	478.00	425.00	524.00	475.67	699.00
GABPB1-AS1	1295.00	1159.00	1169.00	1207.67	671.00
GABPB2	163.00	145.00	168.00	158.67	132.00
GABRA2	27.00	37.00	38.00	34.00	33.00
GABRA3	75.00	66.00	72.00	71.00	78.00
GABRB2	8.00	6.00	2.00	5.33	16.00
GABRE	761.00	749.00	824.00	778.00	326.00
GABRG1	48.00	31.00	49.00	42.67	76.00
GABRG2	27.00	18.00	37.00	27.33	33.00
GABRQ	11.00	14.00	8.00	11.00	7.00
GABRR2	4.00	3.00	10.00	5.67	6.00
GACAT2	46.00	48.00	73.00	55.67	105.00
GAD1	72.00	80.00	69.00	73.67	97.00
GADD45A	1846.00	1741.00	1915.00	1834.00	2346.00
GADD45B	975.00	1024.00	1072.00	1023.67	1169.00
GADD45GIP1	821.00	815.00	894.00	843.33	1064.00
GAK	1686.00	1686.00	1791.00	1721.00	1458.00
GAL3ST4	39.00	57.00	35.00	43.67	42.00
GALC	321.00	317.00	334.00	324.00	229.00
GALE	1832.00	1765.00	1897.00	1831.33	1945.00
GALK1	263.00	321.00	319.00	301.00	459.00
GALK2	535.00	503.00	533.00	523.67	530.00
GALM	10.00	10.00	13.00	11.00	9.00

GALNS	1317.00	1298.00	1184.00	1266.33	1374.00
GALNT1	1173.00	989.00	1100.00	1087.33	1285.00
GALNT10	2569.00	2296.00	2726.00	2530.33	2011.00
GALNT11	1115.00	1050.00	1043.00	1069.33	1271.00
GALNT12	150.00	125.00	135.00	136.67	181.00
GALNT13	4.00	1.00	9.00	4.67	2.00
GALNT15	20.00	23.00	30.00	24.33	12.00
GALNT16	150.00	169.00	191.00	170.00	296.00
GALNT18	190.00	202.00	228.00	206.67	346.00
GALNT2	5099.00	4953.00	4797.00	4949.67	3886.00
GALNT3	3.00	8.00	6.00	5.67	7.00
GALNT4	17.00	14.00	15.00	15.33	3.00
GALNT5	8.00	6.00	7.00	7.00	1.00
GALNT6	3432.00	3334.00	3412.00	3392.67	3153.00
GALNT7	1223.00	1098.00	1208.00	1176.33	813.00
GALNTL6	6.00	7.00	17.00	10.00	11.00
GALR3	4.00	9.00	8.00	7.00	8.00
GALT	359.00	386.00	342.00	362.33	390.00
GAMT	444.00	392.00	390.00	408.67	559.00
GAN	47.00	40.00	64.00	50.33	70.00
GANAB	25839.00	25179.00	26236.00	25751.33	21023.00
GANC	345.00	249.00	359.00	317.67	296.00
GAP43	5.00	2.00	4.00	3.67	9.00
GAPDH	62649.00	60444.00	69670.00	64254.33	82176.00
GAPLINC	86.00	93.00	71.00	83.33	189.00
GAPVD1	1328.00	1262.00	1345.00	1311.67	1254.00
GAR1	717.00	663.00	715.00	698.33	946.00
GAREM	91.00	82.00	101.00	91.33	82.00
GAREML	16.00	17.00	15.00	16.00	24.00
GARNL3	169.00	128.00	172.00	156.33	122.00
GARS	14840.00	13949.00	15101.00	14630.00	14810.00
GART	3178.00	2901.00	3309.00	3129.33	3507.00
GAS2	12.00	9.00	14.00	11.67	23.00
GAS2L1	304.00	337.00	324.00	321.67	347.00
GAS2L3	535.00	475.00	595.00	535.00	633.00
GAS5	10950.00	11507.00	12257.00	11571.33	15524.00
GAS6	2259.00	2399.00	2500.00	2386.00	2336.00
GAS6-AS1	20.00	18.00	17.00	18.33	6.00
GAS6-AS2	83.00	64.00	77.00	74.67	61.00
GAS8	726.00	762.00	782.00	756.67	861.00
GATA2	216.00	207.00	200.00	207.67	243.00
GATA2-AS1	54.00	60.00	58.00	57.33	30.00
GATA6	425.00	442.00	504.00	457.00	312.00
GATA6-AS1	53.00	53.00	52.00	52.67	36.00
GATAD1	1929.00	1907.00	2242.00	2026.00	2143.00
GATAD2A	2684.00	2643.00	2784.00	2703.67	3028.00
GATAD2B	17.00	14.00	8.00	13.00	6.00
GATB	545.00	496.00	569.00	536.67	642.00

GATC	853.00	842.00	903.00	866.00	995.00
GATM	13.00	9.00	3.00	8.33	13.00
GATS	113.00	95.00	94.00	100.67	76.00
GATSL2	97.00	90.00	86.00	91.00	114.00
GATSL3	223.00	229.00	237.00	229.67	232.00
GBA	16.00	13.00	19.00	16.00	18.00
GBA2	2328.00	2248.00	2510.00	2362.00	2039.00
GBAP1	45.00	70.00	75.00	63.33	62.00
GBAS	919.00	847.00	999.00	921.67	1151.00
GBAT2	7.00	6.00	3.00	5.33	8.00
GBE1	1638.00	1550.00	1785.00	1657.67	1917.00
GBF1	4241.00	3957.00	4387.00	4195.00	3668.00
GBP1	121.00	114.00	154.00	129.67	187.00
GBP2	10.00	8.00	20.00	12.67	25.00
GBP3	95.00	100.00	102.00	99.00	137.00
GBP5	6.00	8.00	9.00	7.67	8.00
GBX2	21.00	15.00	20.00	18.67	38.00
GCA	224.00	197.00	199.00	206.67	264.00
GCAT	445.00	435.00	499.00	459.67	647.00
GCC1	668.00	685.00	788.00	713.67	690.00
GCC2	1183.00	1024.00	1198.00	1135.00	1027.00
GCDH	419.00	441.00	405.00	421.67	470.00
GCFC2	609.00	622.00	739.00	656.67	858.00
GCH1	39.00	51.00	63.00	51.00	70.00
GCLC	794.00	805.00	908.00	835.67	859.00
GCLM	713.00	638.00	746.00	699.00	1009.00
GCN1	7009.00	6711.00	7168.00	6962.67	5830.00
GCNT1	15.00	18.00	25.00	19.33	20.00
GCNT2	806.00	738.00	701.00	748.33	760.00
GCNT3	4.00	6.00	4.00	4.67	11.00
GCSH	244.00	243.00	257.00	248.00	342.00
GCSHP3	107.00	94.00	92.00	97.67	128.00
GDAP1	375.00	369.00	366.00	370.00	431.00
GDAP1L1	3.00	1.00	7.00	3.67	7.00
GDAP2	439.00	392.00	468.00	433.00	460.00
GDE1	1118.00	1114.00	1121.00	1117.67	1368.00
GDF11	348.00	359.00	337.00	348.00	288.00
GDF15	1063.00	1068.00	1015.00	1048.67	510.00
GDF9	39.00	33.00	27.00	33.00	31.00
GDI1	2447.00	2534.00	2675.00	2552.00	2706.00
GDI2	6354.00	5879.00	6981.00	6404.67	7983.00
GDNF	481.00	518.00	589.00	529.33	945.00
GDNF-AS1	724.00	752.00	784.00	753.33	750.00
GDPD1	314.00	337.00	343.00	331.33	309.00
GDPD3	63.00	71.00	62.00	65.33	51.00
GDPD5	206.00	205.00	213.00	208.00	161.00
GDPGP1	27.00	25.00	37.00	29.67	30.00
GEM	870.00	850.00	976.00	898.67	857.00

GEMIN2	200.00	220.00	233.00	217.67	229.00
GEMIN4	1234.00	1219.00	1204.00	1219.00	1265.00
GEMIN5	1291.00	1217.00	1279.00	1262.33	1339.00
GEMIN6	281.00	251.00	278.00	270.00	333.00
GEMIN7	228.00	233.00	264.00	241.67	337.00
GEMIN8	538.00	578.00	658.00	591.33	669.00
GEMIN8P4	14.00	8.00	16.00	12.67	25.00
GEN1	887.00	840.00	802.00	843.00	1010.00
GET4	1325.00	1358.00	1465.00	1382.67	1550.00
GFER	482.00	503.00	515.00	500.00	574.00
GFI1	40.00	34.00	30.00	34.67	38.00
GFM1	1526.00	1345.00	1592.00	1487.67	1771.00
GFM2	1185.00	1161.00	1163.00	1169.67	1290.00
GFOD1	96.00	106.00	115.00	105.67	99.00
GFOD2	555.00	537.00	560.00	550.67	568.00
GFPT1	2577.00	2296.00	2792.00	2555.00	2534.00
GFPT2	1326.00	1330.00	1378.00	1344.67	1164.00
GGA1	3215.00	3230.00	3469.00	3304.67	3003.00
GGA2	1215.00	1255.00	1398.00	1289.33	1304.00
GGA3	1527.00	1531.00	1643.00	1567.00	1394.00
GGACT	54.00	35.00	49.00	46.00	58.00
GGCT	759.00	708.00	787.00	751.33	1097.00
GGCX	1427.00	1449.00	1365.00	1413.67	1257.00
GGH	2354.00	2318.00	2113.00	2261.67	2836.00
GGN	14.00	13.00	15.00	14.00	14.00
GGNBP1	4.00	2.00	1.00	2.33	5.00
GGNBP2	5.00	5.00	10.00	6.67	7.00
GGPS1	603.00	496.00	637.00	578.67	636.00
GGT1	673.00	650.00	628.00	650.33	632.00
GGT5	2198.00	2259.00	2189.00	2215.33	1245.00
GGT7	2823.00	2965.00	3028.00	2938.67	2493.00
GHDC	1168.00	1139.00	1267.00	1191.33	1090.00
GHET1	7.00	4.00	10.00	7.00	9.00
GHITM	4177.00	3646.00	4307.00	4043.33	4897.00
GHR	97.00	69.00	82.00	82.67	94.00
GHRL	7.00	3.00	2.00	4.00	3.00
GHRLOS	85.00	98.00	82.00	88.33	105.00
GID4	512.00	461.00	606.00	526.33	487.00
GID8	1157.00	1066.00	1161.00	1128.00	1087.00
GIGYF1	4740.00	4922.00	5257.00	4973.00	5266.00
GIGYF2	1297.00	1239.00	1353.00	1296.33	1207.00
GIMAP2	13.00	11.00	17.00	13.67	24.00
GIN1	170.00	150.00	185.00	168.33	149.00
GINM1	684.00	649.00	660.00	664.33	796.00
GINS1	415.00	392.00	407.00	404.67	521.00
GINS2	184.00	203.00	179.00	188.67	299.00
GINS3	201.00	235.00	209.00	215.00	353.00
GINS4	499.00	517.00	465.00	493.67	624.00

GIPC1	1460.00	1381.00	1532.00	1457.67	1428.00
GIPR	5.00	5.00	8.00	6.00	1.00
GIT1	2095.00	2065.00	2309.00	2156.33	2214.00
GIT2	1250.00	1250.00	1325.00	1275.00	1286.00
GJA3	129.00	121.00	147.00	132.33	165.00
GJB2	27.00	19.00	11.00	19.00	37.00
GJC1	1095.00	1080.00	1188.00	1121.00	1018.00
GJC2	14.00	12.00	13.00	13.00	16.00
GJC3	3.00	1.00	4.00	2.67	0.00
GJD4	4.00	3.00	1.00	2.67	7.00
GK	223.00	235.00	249.00	235.67	231.00
GK5	906.00	821.00	931.00	886.00	872.00
GKAP1	99.00	95.00	100.00	98.00	121.00
GLA	388.00	401.00	371.00	386.67	422.00
GLB1	3321.00	3380.00	3449.00	3383.33	3050.00
GLB1L	159.00	207.00	184.00	183.33	185.00
GLCCI1	316.00	260.00	333.00	303.00	276.00
GLCE	1382.00	1257.00	1431.00	1356.67	884.00
GLDC	14.00	19.00	21.00	18.00	47.00
GLE1	1505.00	1330.00	1566.00	1467.00	1668.00
GLG1	7417.00	6894.00	7740.00	7350.33	5657.00
GLI1	7.00	5.00	8.00	6.67	6.00
GLI2	1275.00	1272.00	1487.00	1344.67	1062.00
GLI3	1163.00	1093.00	1177.00	1144.33	1053.00
GLI4	630.00	620.00	770.00	673.33	650.00
GLIDR	138.00	109.00	119.00	122.00	119.00
GLIPR1	1445.00	1339.00	1399.00	1394.33	1209.00
GLIPR1L2	6.00	13.00	6.00	8.33	7.00
GLIPR2	186.00	221.00	219.00	208.67	144.00
GLIS1	69.00	82.00	96.00	82.33	48.00
GLIS2	18.00	7.00	33.00	19.33	23.00
GLIS3	1189.00	1071.00	1266.00	1175.33	948.00
GLMN	506.00	468.00	509.00	494.33	610.00
GLMP	3039.00	2987.00	2896.00	2974.00	2937.00
GLO1	2801.00	2504.00	3033.00	2779.33	4520.00
GLOD4	901.00	950.00	1011.00	954.00	1189.00
GLRB	464.00	413.00	467.00	448.00	503.00
GLRX	962.00	845.00	913.00	906.67	1450.00
GLRX2	373.00	357.00	381.00	370.33	510.00
GLRX3	1740.00	1644.00	1908.00	1764.00	2325.00
GLRX5	511.00	540.00	531.00	527.33	669.00
GLS	3497.00	3146.00	3838.00	3493.67	4321.00
GLT8D1	1549.00	1506.00	1475.00	1510.00	1666.00
GLT8D2	338.00	322.00	364.00	341.33	291.00
GLTP	830.00	810.00	854.00	831.33	1200.00
GLTSCR1	421.00	387.00	402.00	403.33	379.00
GLTSCR1L	593.00	620.00	607.00	606.67	522.00
GLTSCR2	3180.00	3272.00	3439.00	3297.00	4013.00

GLUD1	2437.00	2350.00	2635.00	2474.00	3008.00
GLUD1P3	343.00	361.00	346.00	350.00	246.00
GLUD1P7	13.00	5.00	11.00	9.67	11.00
GLUD2	30.00	39.00	47.00	38.67	47.00
GLUL	726.00	772.00	793.00	763.67	982.00
GLYATL1	6.00	3.00	5.00	4.67	7.00
GLYATL2	5.00	4.00	4.00	4.33	4.00
GLYCTK	534.00	498.00	502.00	511.33	705.00
GLYR1	2433.00	2357.00	2623.00	2471.00	2567.00
GM2A	822.00	725.00	749.00	765.33	919.00
GMCL1	358.00	314.00	368.00	346.67	382.00
GMDS	339.00	344.00	348.00	343.67	398.00
GMDS-AS1	30.00	40.00	32.00	34.00	32.00
GMEB1	273.00	292.00	251.00	272.00	287.00
GMEB2	878.00	928.00	903.00	903.00	1164.00
GMFB	1229.00	1136.00	1422.00	1262.33	1582.00
GMFG	10.00	9.00	7.00	8.67	0.00
GMIP	527.00	509.00	455.00	497.00	412.00
GMNN	333.00	307.00	386.00	342.00	528.00
GMPPA	1005.00	978.00	1133.00	1038.67	996.00
GMPPB	323.00	307.00	340.00	323.33	332.00
GMPR	54.00	39.00	54.00	49.00	58.00
GMPR2	1586.00	1560.00	1815.00	1653.67	1621.00
GMPS	1970.00	1819.00	2089.00	1959.33	2416.00
GNA11	1543.00	1440.00	1598.00	1527.00	1570.00
GNA12	1967.00	1950.00	2158.00	2025.00	1984.00
GNA13	1169.00	1049.00	1372.00	1196.67	1294.00
GNA14	3.00	2.00	0.00	1.67	2.00
GNAI1	273.00	211.00	245.00	243.00	287.00
GNAI2	3358.00	3248.00	3395.00	3333.67	3575.00
GNAI3	1403.00	1149.00	1309.00	1287.00	1731.00
GNAQ	816.00	667.00	907.00	796.67	908.00
GNAS	8791.00	8587.00	8864.00	8747.33	10450.00
GNAS-AS1	24.00	18.00	25.00	22.33	16.00
GNAT2	4.00	7.00	6.00	5.67	2.00
GNAZ	7.00	8.00	11.00	8.67	10.00
GNB1	5557.00	5235.00	5670.00	5487.33	6672.00
GNB1L	197.00	208.00	180.00	195.00	204.00
GNB2	2351.00	2306.00	2481.00	2379.33	2618.00
GNB2L1	33460.00	32101.00	37496.00	34352.33	39910.00
GNB3	970.00	1001.00	1112.00	1027.67	906.00
GNB4	833.00	626.00	827.00	762.00	846.00
GNB5	356.00	346.00	365.00	355.67	352.00
GNE	1009.00	924.00	1003.00	978.67	804.00
GNG10	37.00	46.00	56.00	46.33	75.00
GNG11	738.00	744.00	818.00	766.67	989.00
GNG12	2767.00	2308.00	2982.00	2685.67	2441.00
GNG12-AS1	6.00	17.00	10.00	11.00	8.00

GNG2	41.00	36.00	52.00	43.00	56.00
GNG4	124.00	129.00	170.00	141.00	222.00
GNG5	1545.00	1528.00	1696.00	1589.67	2098.00
GNGT2	21.00	13.00	5.00	13.00	18.00
GNL2	2041.00	1898.00	2216.00	2051.67	2772.00
GNL3	3366.00	3354.00	3610.00	3443.33	3857.00
GNL3L	1148.00	1014.00	1087.00	1083.00	1237.00
GNMT	10.00	5.00	3.00	6.00	5.00
GNPAT	1205.00	1116.00	1229.00	1183.33	1448.00
GNPDA1	2736.00	2628.00	2938.00	2767.33	3087.00
GNPDA2	740.00	687.00	772.00	733.00	819.00
GNPNAT1	2658.00	2330.00	2942.00	2643.33	2859.00
GNPTAB	1009.00	983.00	1144.00	1045.33	952.00
GNPTG	1050.00	1180.00	1164.00	1131.33	1156.00
GNRH1	44.00	59.00	56.00	53.00	59.00
GNRH2	8.00	7.00	6.00	7.00	2.00
GNRHR2	9.00	12.00	22.00	14.33	19.00
GNS	6513.00	5977.00	6165.00	6218.33	3400.00
GOLGA1	806.00	789.00	796.00	797.00	770.00
GOLGA2	2936.00	2780.00	2972.00	2896.00	2869.00
GOLGA2P5	47.00	42.00	39.00	42.67	32.00
GOLGA2P7	102.00	105.00	93.00	100.00	98.00
GOLGA3	3332.00	3268.00	3329.00	3309.67	2925.00
GOLGA4	2586.00	2379.00	2804.00	2589.67	2381.00
GOLGA5	975.00	862.00	1067.00	968.00	1131.00
GOLGA6L10	13.00	18.00	13.00	14.67	13.00
GOLGA6L4	3.00	4.00	3.00	3.33	1.00
GOLGA6L5P	4.00	5.00	7.00	5.33	9.00
GOLGA6L9	43.00	49.00	57.00	49.67	40.00
GOLGA7	701.00	631.00	726.00	686.00	873.00
GOLGA7B	4.00	5.00	2.00	3.67	5.00
GOLGA8A	4380.00	4550.00	4858.00	4596.00	4617.00
GOLGA8B	2993.00	3117.00	3406.00	3172.00	3278.00
GOLGA8J	8.00	2.00	7.00	5.67	1.00
GOLGA8O	5.00	6.00	4.00	5.00	5.00
GOLGA8R	6.00	6.00	5.00	5.67	4.00
GOLGB1	3498.00	3169.00	3484.00	3383.67	2517.00
GOLIM4	1751.00	1619.00	1800.00	1723.33	1696.00
GOLM1	3581.00	3501.00	3512.00	3531.33	3517.00
GOLPH3	989.00	877.00	1104.00	990.00	1191.00
GOLPH3L	566.00	499.00	596.00	553.67	675.00
GOLT1B	2669.00	2415.00	2939.00	2674.33	3073.00
GON4L	1561.00	1419.00	1559.00	1513.00	1158.00
GOPC	1624.00	1552.00	1687.00	1621.00	1647.00
GORAB	580.00	593.00	611.00	594.67	723.00
GORASP1	1300.00	1301.00	1486.00	1362.33	1319.00
GORASP2	1826.00	1642.00	1884.00	1784.00	2022.00
GOSR1	826.00	799.00	927.00	850.67	933.00

GOSR2	1147.00	1145.00	1202.00	1164.67	1119.00
GOT1	1842.00	1724.00	1787.00	1784.33	2179.00
GOT2	2589.00	2433.00	2590.00	2537.33	3069.00
GPAA1	3483.00	3430.00	3214.00	3375.67	3457.00
GPALPP1	320.00	302.00	318.00	313.33	335.00
GPAM	1689.00	1508.00	1700.00	1632.33	1159.00
GPAT3	248.00	263.00	254.00	255.00	228.00
GPAT4	3610.00	3630.00	3679.00	3639.67	3475.00
GPATCH1	213.00	227.00	227.00	222.33	240.00
GPATCH11	435.00	352.00	428.00	405.00	397.00
GPATCH2	493.00	432.00	445.00	456.67	479.00
GPATCH2L	1594.00	1484.00	1694.00	1590.67	1319.00
GPATCH3	264.00	303.00	315.00	294.00	323.00
GPATCH4	1113.00	1171.00	1209.00	1164.33	1493.00
GPATCH8	2151.00	2044.00	2266.00	2153.67	1752.00
GPBAR1	4.00	3.00	1.00	2.67	6.00
GPBP1	1612.00	1640.00	1803.00	1685.00	1774.00
GPBP1L1	2974.00	3013.00	3230.00	3072.33	3196.00
GPC1	98.00	103.00	90.00	97.00	107.00
GPC2	237.00	245.00	246.00	242.67	294.00
GPC5	8.00	7.00	8.00	7.67	9.00
GPC6	158.00	132.00	154.00	148.00	83.00
GPCPD1	1394.00	1448.00	1591.00	1477.67	1458.00
GPD1	12.00	15.00	9.00	12.00	20.00
GPD1L	531.00	464.00	598.00	531.00	578.00
GPD2	799.00	708.00	857.00	788.00	1012.00
GPER1	6.00	5.00	5.00	5.33	10.00
GPHN	560.00	560.00	582.00	567.33	545.00
GPI	2342.00	2208.00	2377.00	2309.00	2949.00
GPKOW	549.00	567.00	576.00	564.00	664.00
GPLD1	33.00	27.00	24.00	28.00	41.00
GPM6A	13.00	13.00	11.00	12.33	15.00
GPM6B	88.00	94.00	98.00	93.33	142.00
GPN1	771.00	706.00	849.00	775.33	834.00
GPN2	512.00	438.00	541.00	497.00	467.00
GPN3	546.00	478.00	530.00	518.00	678.00
GPNMB	1703.00	1692.00	1627.00	1674.00	892.00
GPR1	5.00	5.00	10.00	6.67	4.00
GPR107	3105.00	2992.00	3187.00	3094.67	2944.00
GPR108	1742.00	1764.00	1750.00	1752.00	1754.00
GPR135	306.00	311.00	374.00	330.33	215.00
GPR137	1622.00	1605.00	1653.00	1626.67	1655.00
GPR137B	544.00	519.00	499.00	520.67	497.00
GPR137C	62.00	66.00	53.00	60.33	52.00
GPR143	1271.00	1257.00	1295.00	1274.33	1562.00
GPR146	24.00	14.00	24.00	20.67	13.00
GPR153	151.00	181.00	177.00	169.67	116.00
GPR155	169.00	149.00	165.00	161.00	118.00

GPR156	30.00	35.00	32.00	32.33	27.00
GPR157	68.00	52.00	70.00	63.33	45.00
GPR158	4.00	0.00	6.00	3.33	9.00
GPR160	23.00	16.00	23.00	20.67	27.00
GPR161	866.00	878.00	963.00	902.33	773.00
GPR162	126.00	128.00	110.00	121.33	117.00
GPR173	605.00	672.00	661.00	646.00	588.00
GPR176	882.00	822.00	955.00	886.33	905.00
GPR180	870.00	845.00	941.00	885.33	967.00
GPR19	46.00	67.00	46.00	53.00	48.00
GPR3	306.00	289.00	341.00	312.00	293.00
GPR35	15.00	10.00	14.00	13.00	16.00
GPR37	181.00	193.00	177.00	183.67	211.00
GPR39	1624.00	1628.00	1749.00	1667.00	1635.00
GPR4	243.00	194.00	212.00	216.33	103.00
GPR50	3.00	0.00	1.00	1.33	0.00
GPR63	453.00	496.00	519.00	489.33	451.00
GPR65	3.00	3.00	1.00	2.33	7.00
GPR68	58.00	62.00	61.00	60.33	39.00
GPR75	155.00	175.00	168.00	166.00	125.00
GPR83	5.00	4.00	5.00	4.67	5.00
GPR85	74.00	57.00	84.00	71.67	72.00
GPR88	4.00	3.00	6.00	4.33	3.00
GPR89A	157.00	128.00	136.00	140.33	144.00
GPR89B	102.00	102.00	121.00	108.33	96.00
GPRASP1	469.00	436.00	486.00	463.67	414.00
GPRASP2	27.00	18.00	23.00	22.67	15.00
GPRC5A	786.00	821.00	914.00	840.33	598.00
GPRIN1	162.00	162.00	188.00	170.67	163.00
GPRIN3	3.00	3.00	2.00	2.67	1.00
GPS1	2308.00	2184.00	2317.00	2269.67	2506.00
GPS2	778.00	849.00	790.00	805.67	882.00
GPSM1	2164.00	2222.00	2403.00	2263.00	1930.00
GPSM2	342.00	352.00	429.00	374.33	472.00
GPT	12.00	27.00	30.00	23.00	18.00
GPT2	2304.00	2373.00	2399.00	2358.67	2422.00
GPX1	2222.00	2196.00	2204.00	2207.33	2788.00
GPX4	1782.00	1741.00	1830.00	1784.33	2412.00
GPX8	2381.00	2452.00	2296.00	2376.33	3135.00
GRAMD1A	1237.00	1224.00	1226.00	1229.00	1179.00
GRAMD1C	132.00	114.00	113.00	119.67	117.00
GRAMD3	112.00	119.00	108.00	113.00	117.00
GRAMD4	388.00	338.00	359.00	361.67	412.00
GRB10	3107.00	3012.00	3144.00	3087.67	2949.00
GRB14	85.00	71.00	90.00	82.00	101.00
GRB2	1890.00	1739.00	2001.00	1876.67	2068.00
GREB1	51.00	54.00	71.00	58.67	56.00
GREB1L	67.00	83.00	87.00	79.00	69.00

GRHL1	38.00	23.00	29.00	30.00	31.00
GRHPR	1525.00	1566.00	1723.00	1604.67	2030.00
GRIA1	4.00	4.00	4.00	4.00	7.00
GRID2	8.00	8.00	13.00	9.67	5.00
GRID2IP	3.00	0.00	1.00	1.33	0.00
GRIK1-AS1	3.00	4.00	2.00	3.00	1.00
GRIK1-AS2	28.00	30.00	46.00	34.67	18.00
GRIK2	25.00	28.00	31.00	28.00	16.00
GRIK5	14.00	20.00	26.00	20.00	13.00
GRIN2D	109.00	100.00	85.00	98.00	73.00
GRINA	4891.00	4885.00	4873.00	4883.00	4680.00
GRIP1	23.00	31.00	28.00	27.33	24.00
GRIPAP1	1641.00	1675.00	1667.00	1661.00	1557.00
GRK4	213.00	165.00	189.00	189.00	183.00
GRK5	277.00	262.00	259.00	266.00	245.00
GRK6	797.00	761.00	779.00	779.00	1166.00
GRK7	5.00	2.00	3.00	3.33	3.00
GRM1	12.00	10.00	7.00	9.67	5.00
GRM5	3.00	2.00	0.00	1.67	9.00
GRM8	3.00	3.00	14.00	6.67	10.00
GRN	12724.00	13356.00	12765.00	12948.33	10451.00
GRPEL1	1045.00	998.00	995.00	1012.67	1379.00
GRPEL2	986.00	927.00	1023.00	978.67	1353.00
GRPR	459.00	436.00	441.00	445.33	438.00
GRSF1	2191.00	1978.00	2275.00	2148.00	2627.00
GRWD1	844.00	757.00	831.00	810.67	867.00
GS1-124K5.11	392.00	391.00	428.00	403.67	388.00
GS1-259H13.2	11.00	14.00	10.00	11.67	9.00
GSAP	586.00	622.00	684.00	630.67	882.00
GSDMB	500.00	580.00	663.00	581.00	532.00
GSE1	1826.00	1705.00	2006.00	1845.67	1292.00
GSG1	12.00	22.00	14.00	16.00	21.00
GSG2	271.00	232.00	254.00	252.33	273.00
GSK3A	1023.00	950.00	1054.00	1009.00	1130.00
GSK3B	1734.00	1596.00	1940.00	1756.67	1664.00
GSKIP	241.00	214.00	281.00	245.33	340.00
GSN	282.00	298.00	277.00	285.67	378.00
GSPT1	5498.00	4993.00	5611.00	5367.33	5695.00
GSPT2	374.00	430.00	450.00	418.00	418.00
GSR	672.00	598.00	677.00	649.00	846.00
GSS	2922.00	2954.00	3084.00	2986.67	3130.00
GSTA4	150.00	152.00	151.00	151.00	191.00
GSTCD	383.00	308.00	375.00	355.33	401.00
GSTK1	2315.00	2352.00	2547.00	2404.67	2689.00
GSTM2	61.00	56.00	60.00	59.00	78.00
GSTM3	678.00	700.00	727.00	701.67	777.00
GSTM4	186.00	140.00	214.00	180.00	209.00
GSTO1	3849.00	3599.00	4118.00	3855.33	4852.00

GSTO2	62.00	56.00	84.00	67.33	94.00
GSTP1	3490.00	3379.00	3416.00	3428.33	4543.00
GSTZ1	186.00	257.00	310.00	251.00	276.00
GTDC1	414.00	378.00	453.00	415.00	606.00
GTF2A1	985.00	937.00	1156.00	1026.00	1228.00
GTF2A2	529.00	521.00	584.00	544.67	657.00
GTF2B	518.00	487.00	563.00	522.67	652.00
GTF2E1	575.00	513.00	566.00	551.33	659.00
GTF2E2	519.00	479.00	548.00	515.33	639.00
GTF2F1	1751.00	1730.00	1807.00	1762.67	1669.00
GTF2F2	1053.00	1082.00	1184.00	1106.33	1510.00
GTF2H1	1105.00	1036.00	1206.00	1115.67	1436.00
GTF2H2B	11.00	10.00	13.00	11.33	15.00
GTF2H2C	10.00	5.00	5.00	6.67	3.00
GTF2H3	1418.00	1302.00	1531.00	1417.00	1692.00
GTF2H4	141.00	106.00	143.00	130.00	114.00
GTF2H5	455.00	398.00	554.00	469.00	581.00
GTF2I	1037.00	982.00	1077.00	1032.00	852.00
GTF2IP1	96.00	99.00	98.00	97.67	117.00
GTF2IP20	524.00	490.00	531.00	515.00	485.00
GTF2IRD1	541.00	535.00	596.00	557.33	589.00
GTF2IRD2	34.00	34.00	21.00	29.67	16.00
GTF2IRD2B	54.00	50.00	59.00	54.33	43.00
GTF3A	1608.00	1556.00	1826.00	1663.33	2135.00
GTF3C1	3826.00	3717.00	3862.00	3801.67	3316.00
GTF3C2	1800.00	1725.00	1915.00	1813.33	2027.00
GTF3C3	2039.00	1823.00	2051.00	1971.00	1975.00
GTF3C4	689.00	561.00	637.00	629.00	699.00
GTF3C5	1292.00	1294.00	1302.00	1296.00	1478.00
GTF3C6	747.00	649.00	725.00	707.00	990.00
GTPBP1	1193.00	1058.00	1204.00	1151.67	872.00
GTPBP10	746.00	686.00	774.00	735.33	766.00
GTPBP2	6801.00	6809.00	6866.00	6825.33	6464.00
GTPBP3	922.00	961.00	964.00	949.00	961.00
GTPBP4	3846.00	3470.00	3874.00	3730.00	4288.00
GTPBP6	6.00	3.00	2.00	3.67	5.00
GTPBP8	351.00	322.00	319.00	330.67	447.00
GTSE1	568.00	512.00	501.00	527.00	545.00
GTSE1-AS1	5.00	7.00	6.00	6.00	13.00
GUCA1B	55.00	48.00	69.00	57.33	52.00
GUCD1	909.00	872.00	860.00	880.33	927.00
GUCY1A2	8.00	6.00	11.00	8.33	7.00
GUCY1A3	23.00	13.00	24.00	20.00	28.00
GUCY1B3	139.00	112.00	133.00	128.00	165.00
GUF1	1940.00	1857.00	2090.00	1962.33	2068.00
GUK1	1587.00	1680.00	1730.00	1665.67	1919.00
GULP1	198.00	183.00	218.00	199.67	230.00
GUSB	2601.00	2724.00	2568.00	2631.00	2928.00

GUSBP1	212.00	202.00	192.00	202.00	230.00
GUSBP11	527.00	550.00	592.00	556.33	430.00
GUSBP2	20.00	15.00	17.00	17.33	23.00
GUSBP4	299.00	343.00	398.00	346.67	399.00
GVINP1	7.00	16.00	14.00	12.33	8.00
GXYLT1	1562.00	1379.00	1566.00	1502.33	1415.00
GXYLT2	79.00	74.00	87.00	80.00	95.00
GYG1	985.00	974.00	1135.00	1031.33	1382.00
GYPC	392.00	434.00	484.00	436.67	460.00
GYPE	35.00	29.00	32.00	32.00	38.00
GYS1	1126.00	1057.00	1053.00	1078.67	1119.00
GZF1	861.00	794.00	961.00	872.00	873.00
GZMA	83.00	93.00	102.00	92.67	77.00
H1F0	1415.00	1326.00	1420.00	1387.00	1620.00
H1FX	1143.00	1216.00	1244.00	1201.00	1252.00
H1FX-AS1	129.00	106.00	130.00	121.67	131.00
H2AFJ	388.00	444.00	416.00	416.00	526.00
H2AFV	2124.00	2102.00	2131.00	2119.00	2659.00
H2AFX	977.00	1010.00	900.00	962.33	1188.00
H2AFY	2604.00	2708.00	2642.00	2651.33	2806.00
H2AFZ	4042.00	3765.00	3938.00	3915.00	6020.00
H3F3AP4	78.00	57.00	59.00	64.67	132.00
H3F3B	8154.00	7702.00	8966.00	8274.00	11633.00
H3F3C	53.00	45.00	47.00	48.33	54.00
H6PD	3609.00	3559.00	3747.00	3638.33	2599.00
HABP4	425.00	425.00	428.00	426.00	453.00
HACD1	433.00	469.00	517.00	473.00	567.00
HACD2	514.00	362.00	409.00	428.33	343.00
HACD3	3651.00	3560.00	3683.00	3631.33	4185.00
HACD4	168.00	167.00	138.00	157.67	212.00
HACE1	479.00	379.00	443.00	433.67	475.00
HACL1	583.00	533.00	590.00	568.67	721.00
HADH	456.00	427.00	464.00	449.00	594.00
HADHA	3956.00	3822.00	4004.00	3927.33	4707.00
HADHB	1898.00	1778.00	2004.00	1893.33	2369.00
HAGH	524.00	523.00	539.00	528.67	628.00
HAGHL	528.00	548.00	658.00	578.00	574.00
HAGLR	208.00	214.00	219.00	213.67	156.00
HAL	4.00	4.00	3.00	3.67	7.00
HAND2	64.00	46.00	50.00	53.33	79.00
HAND2-AS1	4.00	3.00	6.00	4.33	6.00
HAPLN3	46.00	39.00	38.00	41.00	56.00
HARBI1	82.00	74.00	74.00	76.67	100.00
HARS	2152.00	2116.00	2144.00	2137.33	2340.00
HARS2	1176.00	1245.00	1239.00	1220.00	1338.00
HAS2	55.00	54.00	74.00	61.00	89.00
HAS2-AS1	4.00	7.00	6.00	5.67	14.00
HAS3	103.00	116.00	77.00	98.67	100.00

HAT1	872.00	842.00	886.00	866.67	1305.00
HAUS1	251.00	227.00	279.00	252.33	383.00
HAUS2	404.00	359.00	428.00	397.00	504.00
HAUS3	427.00	431.00	419.00	425.67	462.00
HAUS4	698.00	653.00	737.00	696.00	845.00
HAUS5	731.00	781.00	800.00	770.67	787.00
HAUS6	1641.00	1471.00	1745.00	1619.00	1831.00
HAUS7	516.00	546.00	598.00	553.33	662.00
HAUS8	138.00	166.00	174.00	159.33	247.00
HAVCR2	4.00	3.00	6.00	4.33	6.00
HAX1	2654.00	2571.00	2712.00	2645.67	3642.00
HBBP1	111.00	96.00	130.00	112.33	241.00
HBD	3.00	1.00	3.00	2.33	3.00
HBE1	16.00	17.00	18.00	17.00	23.00
HBEGF	7187.00	6902.00	6884.00	6991.00	7283.00
HBP1	647.00	643.00	730.00	673.33	698.00
HBQ1	3.00	5.00	7.00	5.00	3.00
HBS1L	1202.00	1115.00	1271.00	1196.00	1611.00
HCAR1	96.00	105.00	107.00	102.67	99.00
HCCS	452.00	414.00	457.00	441.00	518.00
HCFC1	2815.00	2549.00	2905.00	2756.33	2502.00
HCFC1R1	447.00	418.00	443.00	436.00	707.00
HCFC2	227.00	214.00	236.00	225.67	250.00
HCG11	18.00	14.00	10.00	14.00	15.00
HCG2040054	3.00	3.00	5.00	3.67	2.00
HCG8	7.00	16.00	20.00	14.33	18.00
HCLS1	3684.00	3715.00	3989.00	3796.00	4385.00
HCN2	127.00	138.00	136.00	133.67	64.00
HCN4	3.00	0.00	4.00	2.33	0.00
HDAC1	2504.00	2297.00	2589.00	2463.33	2652.00
HDAC10	1245.00	1276.00	1413.00	1311.33	1174.00
HDAC11	61.00	50.00	52.00	54.33	71.00
HDAC2	3323.00	3112.00	3514.00	3316.33	4260.00
HDAC3	1199.00	1212.00	1311.00	1240.67	1367.00
HDAC4	459.00	425.00	497.00	460.33	454.00
HDAC5	463.00	507.00	538.00	502.67	392.00
HDAC6	1292.00	1346.00	1389.00	1342.33	1164.00
HDAC7	3756.00	3696.00	3765.00	3739.00	3804.00
HDAC8	371.00	396.00	424.00	397.00	440.00
HDAC9	664.00	613.00	741.00	672.67	961.00
HDDC2	964.00	863.00	1052.00	959.67	1345.00
HDDC3	397.00	371.00	414.00	394.00	473.00
HDGF	7301.00	6978.00	7293.00	7190.67	8383.00
HDGFRP2	1264.00	1402.00	1387.00	1351.00	1546.00
HDGFRP3	801.00	676.00	885.00	787.33	1106.00
HDHD1	511.00	430.00	461.00	467.33	556.00
HDHD2	456.00	534.00	612.00	534.00	565.00
HDHD3	264.00	280.00	282.00	275.33	314.00

HDLBP	12703.00	11932.00	13877.00	12837.33	12900.00
HDX	118.00	127.00	126.00	123.67	118.00
HEATR1	4326.00	3917.00	4641.00	4294.67	4172.00
HEATR3	627.00	586.00	630.00	614.33	605.00
HEATR5A	618.00	580.00	709.00	635.67	481.00
HEATR5B	1275.00	1284.00	1437.00	1332.00	1222.00
HEATR6	2284.00	2210.00	2497.00	2330.33	2169.00
HEBP1	1053.00	979.00	1078.00	1036.67	1333.00
HEBP2	470.00	446.00	496.00	470.67	514.00
HECA	487.00	482.00	514.00	494.33	528.00
HECTD1	4192.00	3868.00	4516.00	4192.00	3653.00
HECTD2	422.00	358.00	435.00	405.00	398.00
HECTD3	1138.00	1130.00	1052.00	1106.67	971.00
HECTD4	1830.00	1795.00	1997.00	1874.00	1259.00
HECW2	842.00	744.00	811.00	799.00	997.00
HEG1	2425.00	2184.00	2646.00	2418.33	1466.00
HEIH	400.00	395.00	396.00	397.00	483.00
HELB	67.00	72.00	69.00	69.33	73.00
HELLS	247.00	272.00	261.00	260.00	449.00
HELQ	239.00	225.00	238.00	234.00	278.00
HELZ	968.00	862.00	981.00	937.00	846.00
HELZ2	1047.00	965.00	1159.00	1057.00	1126.00
HEMK1	1826.00	1844.00	1840.00	1836.67	1461.00
HEPACAM2	4.00	2.00	7.00	4.33	2.00
HERC1	1259.00	1232.00	1405.00	1298.67	1099.00
HERC2	1869.00	1900.00	1960.00	1909.67	1407.00
HERC2P2	577.00	599.00	574.00	583.33	526.00
HERC2P3	266.00	242.00	298.00	268.67	165.00
HERC2P4	51.00	48.00	60.00	53.00	51.00
HERC2P7	19.00	19.00	25.00	21.00	23.00
HERC3	177.00	151.00	177.00	168.33	132.00
HERC4	3173.00	3070.00	3437.00	3226.67	3180.00
HERC5	52.00	46.00	73.00	57.00	87.00
HERC6	137.00	99.00	116.00	117.33	130.00
HERPUD1	2225.00	1990.00	2163.00	2126.00	2348.00
HERPUD2	931.00	835.00	962.00	909.33	857.00
HES1	158.00	194.00	211.00	187.67	123.00
HES2	3.00	1.00	0.00	1.33	0.00
HES4	67.00	68.00	92.00	75.67	96.00
HES6	72.00	86.00	80.00	79.33	149.00
HES7	29.00	16.00	32.00	25.67	26.00
HESX1	11.00	22.00	26.00	19.67	39.00
HEXA	5530.00	5563.00	5466.00	5519.67	5359.00
HEXA-AS1	14.00	12.00	11.00	12.33	8.00
HEXB	4749.00	4920.00	4583.00	4750.67	5327.00
HEXDC	498.00	484.00	515.00	499.00	513.00
HEXIM1	587.00	621.00	665.00	624.33	731.00
HEXIM2	75.00	109.00	89.00	91.00	108.00

HEY1	496.00	505.00	527.00	509.33	567.00
HEY2	68.00	62.00	67.00	65.67	73.00
HFE	322.00	376.00	338.00	345.33	393.00
HFM1	38.00	40.00	48.00	42.00	60.00
HGD	4.00	2.00	0.00	2.00	1.00
HGFAC	8.00	12.00	19.00	13.00	6.00
HGH1	710.00	658.00	706.00	691.33	757.00
HGS	9123.00	8879.00	9482.00	9161.33	8775.00
HGSNAT	1586.00	1562.00	1615.00	1587.67	1548.00
HHAT	262.00	300.00	283.00	281.67	212.00
HHEX	222.00	208.00	228.00	219.33	358.00
HHIP	456.00	429.00	467.00	450.67	496.00
HHIP-AS1	103.00	102.00	137.00	114.00	115.00
HHIPL1	19.00	23.00	25.00	22.33	16.00
HHIPL2	10.00	15.00	21.00	15.33	19.00
HHLA3	207.00	210.00	211.00	209.33	290.00
HIAT1	1249.00	1199.00	1263.00	1237.00	1572.00
HIATL1	1384.00	1422.00	1404.00	1403.33	1595.00
HIATL2	97.00	92.00	74.00	87.67	71.00
HIBADH	1331.00	1218.00	1484.00	1344.33	1846.00
HIBCH	425.00	398.00	408.00	410.33	487.00
HIC1	147.00	174.00	193.00	171.33	133.00
HIC2	165.00	172.00	174.00	170.33	175.00
HID1	9.00	15.00	8.00	10.67	22.00
HIF1A	8733.00	7513.00	9423.00	8556.33	6408.00
HIF1A-AS1	8.00	3.00	13.00	8.00	13.00
HIF1AN	1982.00	1995.00	2178.00	2051.67	1915.00
HIGD1A	1086.00	1067.00	1122.00	1091.67	1514.00
HIGD2A	574.00	597.00	631.00	600.67	822.00
HILPDA	349.00	333.00	327.00	336.33	433.00
HINFP	478.00	504.00	511.00	497.67	555.00
HINT1	4453.00	4387.00	4720.00	4520.00	5937.00
HINT2	473.00	527.00	487.00	495.67	612.00
HINT3	538.00	522.00	552.00	537.33	637.00
HIP1	1636.00	1568.00	1698.00	1634.00	1179.00
HIP1R	781.00	834.00	826.00	813.67	673.00
HIPK1	1123.00	989.00	1298.00	1136.67	932.00
HIPK1-AS1	18.00	13.00	13.00	14.67	9.00
HIPK2	5935.00	5161.00	6070.00	5722.00	5217.00
HIPK3	862.00	773.00	883.00	839.33	779.00
HIRA	1121.00	1127.00	1130.00	1126.00	1262.00
HIRIP3	427.00	446.00	408.00	427.00	566.00
HIST1H1C	24.00	20.00	20.00	21.33	23.00
HIST1H2AC	13.00	9.00	15.00	12.33	12.00
HIST1H2AE	3.00	1.00	6.00	3.33	2.00
HIST1H2AK	6.00	5.00	4.00	5.00	8.00
HIST1H2AM	3.00	0.00	0.00	1.00	3.00
HIST1H2BC	3.00	1.00	4.00	2.67	1.00

HIST1H2BD	23.00	16.00	15.00	18.00	23.00
HIST1H2BH	7.00	2.00	5.00	4.67	2.00
HIST1H2BK	217.00	214.00	247.00	226.00	244.00
HIST1H2BN	7.00	17.00	12.00	12.00	16.00
HIST1H3B	3.00	1.00	1.00	1.67	0.00
HIST1H3D	4.00	3.00	1.00	2.67	1.00
HIST1H3E	17.00	4.00	12.00	11.00	12.00
HIST2H2BA	10.00	21.00	21.00	17.33	9.00
HIST2H2BC	8.00	8.00	2.00	6.00	3.00
HIST2H2BE	9.00	8.00	18.00	11.67	22.00
HIST2H2BF	4.00	1.00	1.00	2.00	2.00
HIST3H2A	41.00	45.00	50.00	45.33	44.00
HIST4H4	6.00	9.00	10.00	8.33	4.00
HIVEP1	1076.00	875.00	1155.00	1035.33	645.00
HIVEP2	1408.00	1199.00	1449.00	1352.00	1074.00
HIVEP3	473.00	466.00	453.00	464.00	489.00
HJURP	1378.00	1465.00	1468.00	1437.00	1879.00
HK1	4946.00	4884.00	5177.00	5002.33	4923.00
HK2	4882.00	4636.00	5048.00	4855.33	5632.00
HKDC1	203.00	185.00	208.00	198.67	132.00
HKR1	513.00	575.00	508.00	532.00	587.00
HLA-A	2475.00	2996.00	2587.00	2686.00	2678.00
HLA-B	381.00	376.00	329.00	362.00	365.00
HLA-C	883.00	804.00	831.00	839.33	782.00
HLA-DMA	10.00	8.00	5.00	7.67	11.00
HLA-DRB1	74.00	94.00	85.00	84.33	133.00
HLA-DRB5	5.00	6.00	6.00	5.67	7.00
HLA-H	4.00	0.00	1.00	1.67	2.00
HLCS	1280.00	1146.00	1242.00	1222.67	1237.00
HLF	30.00	16.00	18.00	21.33	32.00
HLTF	2526.00	2376.00	2642.00	2514.67	2854.00
HLX	246.00	284.00	290.00	273.33	260.00
HLX-AS1	11.00	15.00	14.00	13.33	9.00
HM13	5835.00	6027.00	6018.00	5960.00	5487.00
HMBOX1	111.00	87.00	102.00	100.00	53.00
HMBS	692.00	658.00	731.00	693.67	861.00
HMCES	563.00	561.00	567.00	563.67	815.00
HMCN1	78.00	48.00	77.00	67.67	61.00
HMCN2	7.00	5.00	0.00	4.00	8.00
HMG20A	961.00	823.00	931.00	905.00	917.00
HMG20B	1467.00	1608.00	1699.00	1591.33	1974.00
HMGA1	41750.00	40059.00	43831.00	41880.00	35150.00
HMGA2	5156.00	5100.00	5891.00	5382.33	5253.00
HMGB1	3412.00	3475.00	3497.00	3461.33	4932.00
HMGB2	2454.00	2342.00	2513.00	2436.33	3811.00
HMGB3	500.00	470.00	514.00	494.67	697.00
HMGCL	674.00	651.00	719.00	681.33	720.00
HMGCR	1289.00	1216.00	1276.00	1260.33	1336.00

HMGCS1	629.00	573.00	673.00	625.00	688.00
HMGN1	2659.00	2689.00	2807.00	2718.33	3645.00
HMGN2	2329.00	2272.00	2424.00	2341.67	3068.00
HMGN3	912.00	852.00	935.00	899.67	939.00
HMGN3-AS1	32.00	19.00	17.00	22.67	17.00
HMGN4	1974.00	1798.00	2174.00	1982.00	2162.00
HMGN5	295.00	287.00	357.00	313.00	483.00
HMGXB3	1874.00	1780.00	1971.00	1875.00	1845.00
HMGXB4	900.00	824.00	946.00	890.00	913.00
HMHA1	81.00	94.00	109.00	94.67	63.00
HMMR	930.00	798.00	890.00	872.67	1022.00
HMOX1	305.00	369.00	354.00	342.67	622.00
HN1	787.00	706.00	843.00	778.67	1056.00
HN1L	1346.00	1307.00	1480.00	1377.67	1416.00
HNF4G	69.00	67.00	80.00	72.00	121.00
HNMT	3.00	3.00	0.00	2.00	2.00
HNRNPA0	3181.00	3094.00	3243.00	3172.67	4017.00
HNRNPA1	9531.00	9447.00	10328.00	9768.67	12823.00
HNRNPA1L2	252.00	212.00	223.00	229.00	222.00
HNRNPA1P10	1833.00	1858.00	1954.00	1881.67	2808.00
HNRNPA2B1	23910.00	23772.00	24633.00	24105.00	30997.00
HNRNPA3	9827.00	9893.00	10344.00	10021.33	11659.00
HNRNPAB	3987.00	3910.00	4257.00	4051.33	5608.00
HNRNPC	12066.00	11414.00	12741.00	12073.67	15797.00
HNRNPD	4735.00	4526.00	4669.00	4643.33	6272.00
HNRNPDL	6041.00	6065.00	6258.00	6121.33	6983.00
HNRNPF	6650.00	6482.00	7164.00	6765.33	8291.00
HNRNPH1	14022.00	13790.00	14890.00	14234.00	15269.00
HNRNPH2	31.00	23.00	34.00	29.33	40.00
HNRNPH3	5234.00	5229.00	5558.00	5340.33	6680.00
HNRNPK	13327.00	12734.00	14074.00	13378.33	15680.00
HNRNPL	4309.00	4224.00	4464.00	4332.33	5409.00
HNRNPLL	628.00	601.00	685.00	638.00	696.00
HNRNPM	6154.00	6037.00	6322.00	6171.00	7446.00
HNRNPR	3903.00	3852.00	3992.00	3915.67	4672.00
HNRNPU	13045.00	12455.00	13402.00	12967.33	16826.00
HNRNPU-AS1	2390.00	2494.00	2872.00	2585.33	2306.00
HNRNPUL1	4264.00	4023.00	4423.00	4236.67	4638.00
HNRNPUL2	1260.00	1222.00	1349.00	1277.00	1440.00
HOGA1	40.00	33.00	36.00	36.33	42.00
HOMER1	281.00	237.00	298.00	272.00	287.00
HOMER2	57.00	32.00	51.00	46.67	32.00
HOMER3	1087.00	1143.00	1137.00	1122.33	1348.00
HOMEZ	312.00	273.00	275.00	286.67	255.00
HOOK2	749.00	782.00	806.00	779.00	676.00
HOOK3	809.00	697.00	839.00	781.67	798.00
HOPX	1257.00	1193.00	1330.00	1260.00	1459.00
HOTAIR	21.00	26.00	32.00	26.33	18.00

HOTAIRM1	812.00	796.00	874.00	827.33	815.00
HOXA1	128.00	111.00	136.00	125.00	182.00
HOXA10	201.00	206.00	229.00	212.00	306.00
HOXA11	59.00	65.00	61.00	61.67	99.00
HOXA11-AS	168.00	123.00	134.00	141.67	165.00
HOXA13	13.00	2.00	12.00	9.00	11.00
HOXA2	26.00	36.00	22.00	28.00	20.00
HOXA3	157.00	142.00	137.00	145.33	171.00
HOXA4	116.00	150.00	142.00	136.00	172.00
HOXA5	83.00	93.00	79.00	85.00	88.00
HOXA6	15.00	22.00	19.00	18.67	19.00
HOXA7	120.00	152.00	104.00	125.33	154.00
HOXA9	5.00	4.00	0.00	3.00	12.00
HOXA-AS2	49.00	62.00	47.00	52.67	56.00
HOXB2	1053.00	1041.00	1080.00	1058.00	1651.00
HOXB3	1117.00	1088.00	1166.00	1123.67	1170.00
HOXB4	454.00	514.00	479.00	482.33	577.00
HOXB5	385.00	334.00	403.00	374.00	398.00
HOXB6	828.00	788.00	867.00	827.67	785.00
HOXB7	467.00	448.00	484.00	466.33	613.00
HOXB8	235.00	219.00	205.00	219.67	246.00
HOXB9	2348.00	2242.00	2452.00	2347.33	2516.00
HOXB-AS1	523.00	641.00	656.00	606.67	626.00
HOXC10	22.00	28.00	21.00	23.67	37.00
HOXC11	36.00	41.00	37.00	38.00	56.00
HOXC13	48.00	39.00	40.00	42.33	55.00
HOXC4	236.00	242.00	246.00	241.33	266.00
HOXC5	75.00	58.00	64.00	65.67	66.00
HOXC6	426.00	417.00	418.00	420.33	398.00
HOXC8	433.00	458.00	518.00	469.67	418.00
HOXC9	152.00	143.00	167.00	154.00	205.00
HOXC-AS1	22.00	29.00	47.00	32.67	35.00
HOXD1	10.00	9.00	13.00	10.67	11.00
HOXD13	79.00	61.00	78.00	72.67	93.00
HOXD3	232.00	265.00	281.00	259.33	250.00
HOXD4	187.00	181.00	196.00	188.00	205.00
HOXD8	274.00	276.00	315.00	288.33	330.00
HOXD9	221.00	281.00	253.00	251.67	303.00
HOXD-AS2	90.00	99.00	95.00	94.67	137.00
HP09053	4.00	12.00	5.00	7.00	6.00
HP1BP3	6932.00	6635.00	7408.00	6991.67	6334.00
HPCAL1	1498.00	1449.00	1529.00	1492.00	1478.00
HPDL	320.00	310.00	325.00	318.33	510.00
HPRT1	514.00	451.00	555.00	506.67	729.00
HPS1	1100.00	1110.00	1213.00	1141.00	1233.00
HPS3	1474.00	1374.00	1494.00	1447.33	1422.00
HPS4	1451.00	1400.00	1551.00	1467.33	1316.00
HPS5	690.00	603.00	670.00	654.33	784.00

HPS6	592.00	579.00	632.00	601.00	635.00
HPSE	264.00	245.00	295.00	268.00	308.00
HPX	8.00	9.00	7.00	8.00	14.00
HRASLS	96.00	93.00	99.00	96.00	123.00
HRASLS2	3.00	0.00	0.00	1.00	0.00
HRAT5	4.00	3.00	2.00	3.00	8.00
HRAT92	225.00	266.00	259.00	250.00	131.00
HRH1	770.00	741.00	752.00	754.33	565.00
HRSP12	348.00	326.00	419.00	364.33	527.00
HS1BP3	481.00	513.00	541.00	511.67	530.00
HS1BP3-IT1	3.00	1.00	5.00	3.00	3.00
HS2ST1	1486.00	1375.00	1610.00	1490.33	1687.00
HS3ST1	29.00	47.00	18.00	31.33	26.00
HS3ST3A1	9.00	3.00	4.00	5.33	6.00
HS3ST5	20.00	20.00	29.00	23.00	24.00
HS6ST1	266.00	298.00	295.00	286.33	337.00
HS6ST2	5.00	1.00	9.00	5.00	3.00
HS6ST3	57.00	57.00	56.00	56.67	27.00
HSBP1	1940.00	1836.00	1947.00	1907.67	2703.00
HSBP1L1	53.00	38.00	41.00	44.00	34.00
HSCB	207.00	203.00	185.00	198.33	271.00
HSD11B1	17.00	16.00	13.00	15.33	7.00
HSD11B1L	200.00	190.00	186.00	192.00	238.00
HSD17B1	30.00	32.00	44.00	35.33	36.00
HSD17B10	676.00	662.00	683.00	673.67	906.00
HSD17B11	968.00	930.00	1035.00	977.67	1268.00
HSD17B12	1964.00	1966.00	1991.00	1973.67	2382.00
HSD17B13	3.00	5.00	5.00	4.33	3.00
HSD17B14	55.00	71.00	63.00	63.00	46.00
HSD17B3	4.00	2.00	4.00	3.33	7.00
HSD17B4	1546.00	1571.00	1801.00	1639.33	1792.00
HSD17B6	19.00	20.00	29.00	22.67	20.00
HSD17B7	84.00	72.00	76.00	77.33	92.00
HSD17B7P2	56.00	68.00	61.00	61.67	40.00
HSD3B7	112.00	106.00	153.00	123.67	149.00
HSD52	5.00	6.00	6.00	5.67	7.00
HSDL1	1043.00	941.00	1231.00	1071.67	1074.00
HSDL2	484.00	475.00	565.00	508.00	583.00
HSF1	2685.00	2597.00	2805.00	2695.67	2679.00
HSF2	654.00	607.00	777.00	679.33	744.00
HSF2BP	49.00	31.00	48.00	42.67	36.00
HSF4	1315.00	1278.00	1427.00	1340.00	1148.00
HSH2D	6.00	10.00	11.00	9.00	6.00
HSP90AA1	21616.00	19963.00	21903.00	21160.67	27257.00
HSP90AB1	48643.00	45977.00	50481.00	48367.00	55257.00
HSP90B1	38685.00	39220.00	39708.00	39204.33	38475.00
HSP90B2P	186.00	161.00	175.00	174.00	157.00
HSPA12A	49.00	51.00	45.00	48.33	38.00

HSPA13	3762.00	3278.00	3567.00	3535.67	3454.00
HSPA14	1384.00	1270.00	1381.00	1345.00	1617.00
HSPA1A	22.00	29.00	33.00	28.00	17.00
HSPA1B	7.00	11.00	11.00	9.67	11.00
HSPA2	105.00	104.00	84.00	97.67	108.00
HSPA4	4258.00	3919.00	4540.00	4239.00	5537.00
HSPA4L	243.00	245.00	286.00	258.00	297.00
HSPA5	30237.00	30313.00	30545.00	30365.00	25526.00
HSPA8	26188.00	26586.00	30814.00	27862.67	29937.00
HSPA9	15888.00	14728.00	16612.00	15742.67	18843.00
HSPB1	2109.00	1992.00	2195.00	2098.67	3300.00
HSPB11	224.00	233.00	257.00	238.00	330.00
HSPBAP1	417.00	429.00	531.00	459.00	612.00
HSPBP1	833.00	848.00	875.00	852.00	972.00
HSPD1	14166.00	13240.00	15067.00	14157.67	16246.00
HSPE1	1100.00	1095.00	1090.00	1095.00	1546.00
HSPG2	2354.00	2208.00	2244.00	2268.67	1548.00
HSPH1	3167.00	3089.00	3418.00	3224.67	3939.00
HTATIP2	899.00	914.00	990.00	934.33	1037.00
HTATSF1	1257.00	1292.00	1335.00	1294.67	1519.00
HTATSF1P2	281.00	299.00	269.00	283.00	279.00
HTR1B	7.00	3.00	0.00	3.33	2.00
HTR4	3.00	4.00	2.00	3.00	0.00
HTR7	624.00	582.00	643.00	616.33	618.00
HTR7P1	253.00	249.00	256.00	252.67	264.00
HTRA1	261.00	280.00	300.00	280.33	339.00
HTRA2	621.00	682.00	722.00	675.00	767.00
HTRA4	3.00	0.00	4.00	2.33	4.00
HTT	4048.00	3893.00	4190.00	4043.67	3150.00
HULC	4.00	7.00	8.00	6.33	4.00
HUS1	355.00	385.00	432.00	390.67	395.00
HUWE1	9571.00	8615.00	9698.00	9294.67	7128.00
HVCN1	4.00	5.00	4.00	4.33	4.00
HYAL1	10.00	13.00	14.00	12.33	6.00
HYAL2	1171.00	1260.00	1131.00	1187.33	1213.00
HYAL3	216.00	218.00	259.00	231.00	176.00
HYI	2118.00	2077.00	2278.00	2157.67	1868.00
HYKK	54.00	56.00	85.00	65.00	72.00
HYLS1	187.00	189.00	188.00	188.00	262.00
HYOU1	7543.00	7577.00	8007.00	7709.00	6495.00
IAH1	492.00	459.00	527.00	492.67	602.00
IARS	9773.00	9102.00	9944.00	9606.33	9604.00
IARS2	3465.00	3202.00	3549.00	3405.33	3941.00
IBA57	249.00	212.00	202.00	221.00	198.00
IBA57-AS1	4.00	4.00	1.00	3.00	2.00
IBTK	2113.00	1822.00	2180.00	2038.33	1798.00
ICA1	507.00	445.00	496.00	482.67	586.00
ICA1L	111.00	120.00	126.00	119.00	142.00

ICAM1	5162.00	5203.00	4895.00	5086.67	5768.00
ICAM3	137.00	150.00	131.00	139.33	198.00
ICAM4	7.00	5.00	5.00	5.67	10.00
ICAM5	1482.00	1601.00	1774.00	1619.00	1487.00
ICE1	2504.00	2320.00	2690.00	2504.67	2296.00
ICE2	1389.00	1364.00	1521.00	1424.67	1408.00
ICK	299.00	275.00	354.00	309.33	284.00
ICMT	1937.00	1758.00	1889.00	1861.33	1943.00
ICT1	419.00	379.00	430.00	409.33	531.00
ID1	234.00	290.00	240.00	254.67	344.00
ID2	5.00	10.00	4.00	6.33	6.00
ID3	442.00	468.00	479.00	463.00	518.00
IDE	1316.00	1163.00	1422.00	1300.33	1281.00
IDH1	1553.00	1533.00	1626.00	1570.67	1816.00
IDH1-AS1	4.00	6.00	4.00	4.67	8.00
IDH2	345.00	344.00	312.00	333.67	484.00
IDH3A	942.00	912.00	1017.00	957.00	1047.00
IDH3B	1572.00	1614.00	1657.00	1614.33	1763.00
IDH3G	661.00	671.00	721.00	684.33	763.00
IDI1	427.00	467.00	506.00	466.67	545.00
IDI2-AS1	50.00	46.00	43.00	46.33	55.00
IDNK	71.00	66.00	53.00	63.33	66.00
IDS	2531.00	2267.00	2726.00	2508.00	1684.00
IDUA	370.00	430.00	447.00	415.67	357.00
IER2	2360.00	2295.00	2367.00	2340.67	3024.00
IER3IP1	732.00	695.00	772.00	733.00	1072.00
IER5	1166.00	1179.00	1223.00	1189.33	1161.00
IER5L	62.00	56.00	84.00	67.33	65.00
IFFO1	1198.00	1213.00	1327.00	1246.00	1193.00
IFFO2	549.00	482.00	632.00	554.33	581.00
IFI16	6518.00	6128.00	6709.00	6451.67	5927.00
IFI27L1	14.00	7.00	14.00	11.67	17.00
IFI30	40.00	42.00	43.00	41.67	23.00
IFI35	136.00	122.00	111.00	123.00	177.00
IFI44	579.00	599.00	560.00	579.33	555.00
IFI44L	90.00	101.00	109.00	100.00	50.00
IFI6	105.00	98.00	82.00	95.00	150.00
IFIH1	80.00	85.00	96.00	87.00	118.00
IFIT1	17.00	7.00	13.00	12.33	9.00
IFIT2	59.00	54.00	67.00	60.00	58.00
IFIT3	157.00	173.00	192.00	174.00	196.00
IFIT5	681.00	636.00	772.00	696.33	808.00
IFITM1	53.00	64.00	66.00	61.00	67.00
IFITM2	724.00	718.00	802.00	748.00	990.00
IFITM3	870.00	796.00	869.00	845.00	1071.00
IFNAR1	1061.00	902.00	886.00	949.67	1064.00
IFNAR2	310.00	309.00	343.00	320.67	436.00
IFNE	382.00	416.00	473.00	423.67	314.00

IFNGR1	1166.00	1138.00	1212.00	1172.00	1223.00
IFNGR2	33.00	44.00	57.00	44.67	40.00
IFRD1	2182.00	1949.00	2202.00	2111.00	2547.00
IFRD2	2234.00	2288.00	2281.00	2267.67	2797.00
IFT122	338.00	400.00	393.00	377.00	424.00
IFT140	900.00	931.00	971.00	934.00	912.00
IFT172	1397.00	1381.00	1421.00	1399.67	1266.00
IFT20	420.00	437.00	439.00	432.00	404.00
IFT22	162.00	152.00	169.00	161.00	214.00
IFT27	366.00	370.00	377.00	371.00	402.00
IFT43	294.00	298.00	271.00	287.67	290.00
IFT46	464.00	490.00	542.00	498.67	559.00
IFT52	337.00	360.00	373.00	356.67	421.00
IFT57	773.00	673.00	814.00	753.33	985.00
IFT74	440.00	418.00	450.00	436.00	545.00
IFT80	1444.00	1417.00	1511.00	1457.33	1324.00
IFT81	571.00	616.00	621.00	602.67	663.00
IFT88	358.00	351.00	335.00	348.00	429.00
IGBP1	1367.00	1386.00	1487.00	1413.33	1594.00
IGDCC4	3.00	8.00	3.00	4.67	3.00
IGF1R	2273.00	2342.00	2432.00	2349.00	2093.00
IGF2BP1	2516.00	2359.00	2662.00	2512.33	2357.00
IGF2BP2	6153.00	5968.00	6301.00	6140.67	6383.00
IGF2BP2-AS1	55.00	74.00	52.00	60.33	77.00
IGF2BP3	3026.00	2671.00	3072.00	2923.00	3185.00
IGF2R	12203.00	11518.00	12384.00	12035.00	8516.00
IGFBP1	6.00	14.00	3.00	7.67	16.00
IGFBP3	126.00	104.00	134.00	121.33	785.00
IGFBP4	41.00	36.00	45.00	40.67	38.00
IGFBP6	392.00	503.00	444.00	446.33	576.00
IGFBP7	471.00	459.00	464.00	464.67	276.00
IGFLR1	22.00	32.00	22.00	25.33	28.00
IGFN1	235.00	239.00	246.00	240.00	124.00
IGHMBP2	543.00	552.00	610.00	568.33	619.00
IGIP	186.00	172.00	248.00	202.00	174.00
IGSF1	5.00	5.00	4.00	4.67	5.00
IGSF10	1061.00	1006.00	1106.00	1057.67	738.00
IGSF23	6.00	4.00	8.00	6.00	1.00
IGSF3	94.00	91.00	109.00	98.00	86.00
IGSF5	8.00	3.00	7.00	6.00	3.00
IGSF8	1392.00	1444.00	1265.00	1367.00	1188.00
IK	2230.00	2222.00	2409.00	2287.00	2567.00
IKBIP	1264.00	1234.00	1130.00	1209.33	1516.00
IKBKAP	1931.00	1759.00	2055.00	1915.00	1890.00
IKBKB	3242.00	3180.00	3283.00	3235.00	2921.00
IKBKE	199.00	206.00	236.00	213.67	170.00
IKBKG	346.00	308.00	337.00	330.33	362.00
IKZF2	82.00	73.00	72.00	75.67	44.00

IKZF4	277.00	289.00	307.00	291.00	248.00
IKZF5	628.00	526.00	666.00	606.67	616.00
IL10RB	427.00	428.00	431.00	428.67	542.00
IL11	678.00	625.00	659.00	654.00	98.00
IL11RA	102.00	102.00	127.00	110.33	77.00
IL12A	388.00	379.00	409.00	392.00	803.00
IL12A-AS1	10.00	11.00	12.00	11.00	10.00
IL12RB1	3.00	6.00	4.00	4.33	0.00
IL12RB2	239.00	232.00	212.00	227.67	191.00
IL13RA1	1605.00	1557.00	1619.00	1593.67	1537.00
IL13RA2	176.00	203.00	159.00	179.33	148.00
IL15	8.00	8.00	14.00	10.00	13.00
IL15RA	24.00	18.00	26.00	22.67	9.00
IL16	14.00	13.00	21.00	16.00	19.00
IL17D	68.00	74.00	81.00	74.33	83.00
IL17RA	662.00	692.00	668.00	674.00	676.00
IL17RC	462.00	455.00	488.00	468.33	539.00
IL17RD	484.00	442.00	486.00	470.67	462.00
IL17RE	15.00	10.00	18.00	14.33	11.00
IL18BP	907.00	768.00	965.00	880.00	494.00
IL18R1	35.00	35.00	49.00	39.67	42.00
IL1A	101.00	94.00	108.00	101.00	148.00
IL1B	6601.00	6221.00	6349.00	6390.33	12035.00
IL1R1	42.00	52.00	48.00	47.33	48.00
IL1RAP	1050.00	1011.00	1159.00	1073.33	1013.00
IL1RAPL1	114.00	101.00	123.00	112.67	77.00
IL1RL1	36.00	36.00	47.00	39.67	26.00
IL20RB	104.00	136.00	113.00	117.67	103.00
IL21R	52.00	35.00	44.00	43.67	89.00
IL23A	9.00	13.00	7.00	9.67	15.00
IL24	75.00	74.00	129.00	92.67	59.00
IL27RA	616.00	585.00	613.00	604.67	727.00
IL2RB	3.00	4.00	3.00	3.33	4.00
IL2RG	6.00	0.00	0.00	2.00	5.00
IL31RA	1167.00	952.00	1130.00	1083.00	754.00
IL32	19.00	17.00	31.00	22.33	66.00
IL37	17.00	11.00	14.00	14.00	17.00
IL4I1	25.00	24.00	43.00	30.67	33.00
IL4R	985.00	960.00	905.00	950.00	852.00
IL6	35.00	33.00	36.00	34.67	69.00
IL6R	33.00	32.00	33.00	32.67	9.00
IL6ST	5644.00	5052.00	5436.00	5377.33	5132.00
IL7	28.00	38.00	44.00	36.67	52.00
IL7R	562.00	452.00	443.00	485.67	126.00
ILF2	4002.00	3819.00	4026.00	3949.00	5112.00
ILF3	11707.00	11906.00	12463.00	12025.33	12564.00
ILF3-AS1	318.00	359.00	371.00	349.33	347.00
ILK	2796.00	2759.00	2958.00	2837.67	3730.00

ILKAP	677.00	735.00	772.00	728.00	847.00
ILVBL	1334.00	1317.00	1453.00	1368.00	1499.00
IMMP1L	64.00	57.00	76.00	65.67	88.00
IMMP2L	330.00	299.00	350.00	326.33	371.00
IMMT	2212.00	2122.00	2282.00	2205.33	2733.00
IMP3	1121.00	1068.00	1141.00	1110.00	1447.00
IMP4	1260.00	1254.00	1251.00	1255.00	1430.00
IMPA1	750.00	695.00	821.00	755.33	885.00
IMPA2	63.00	59.00	77.00	66.33	98.00
IMPACT	894.00	794.00	976.00	888.00	1165.00
IMPAD1	3689.00	3249.00	3654.00	3530.67	3520.00
IMPDH1	1830.00	1740.00	1886.00	1818.67	2177.00
IMPDH2	8930.00	8677.00	9989.00	9198.67	9965.00
IMPG2	50.00	32.00	42.00	41.33	28.00
INA	66.00	72.00	66.00	68.00	35.00
INAFM1	66.00	66.00	72.00	68.00	69.00
INAFM2	620.00	631.00	630.00	627.00	686.00
INCA1	15.00	16.00	17.00	16.00	13.00
INCENP	935.00	839.00	918.00	897.33	980.00
INE1	134.00	120.00	133.00	129.00	91.00
INF2	2554.00	2660.00	2865.00	2693.00	2445.00
ING1	207.00	207.00	217.00	210.33	245.00
ING2	85.00	81.00	90.00	85.33	116.00
ING3	316.00	312.00	340.00	322.67	424.00
ING4	598.00	601.00	633.00	610.67	587.00
ING5	985.00	1022.00	1156.00	1054.33	1135.00
INHA	30.00	27.00	36.00	31.00	30.00
INHBA	2539.00	2471.00	2762.00	2590.67	2529.00
INHBA-AS1	13.00	7.00	14.00	11.33	11.00
INHBC	9.00	4.00	4.00	5.67	4.00
INHBE	491.00	461.00	481.00	477.67	264.00
INIP	178.00	127.00	209.00	171.33	160.00
INO80	1486.00	1363.00	1559.00	1469.33	1374.00
INO80B	68.00	57.00	73.00	66.00	36.00
INO80C	130.00	117.00	135.00	127.33	211.00
INO80D	659.00	570.00	738.00	655.67	676.00
INO80E	1224.00	1218.00	1250.00	1230.67	1482.00
INPP1	798.00	733.00	810.00	780.33	804.00
INPP4A	1109.00	1054.00	1186.00	1116.33	1134.00
INPP4B	457.00	372.00	444.00	424.33	226.00
INPP5A	478.00	448.00	491.00	472.33	506.00
INPP5B	721.00	649.00	668.00	679.33	751.00
INPP5E	1135.00	1167.00	1282.00	1194.67	1105.00
INPP5F	744.00	747.00	828.00	773.00	675.00
INPP5J	20.00	33.00	34.00	29.00	24.00
INPP5K	462.00	475.00	521.00	486.00	468.00
INPPL1	5877.00	5778.00	6415.00	6023.33	5490.00
INSC	24.00	22.00	29.00	25.00	66.00

INSIG1	879.00	836.00	894.00	869.67	1235.00
INSIG2	432.00	407.00	392.00	410.33	362.00
INSR	3.00	2.00	6.00	3.67	8.00
INTS1	4176.00	3917.00	4027.00	4040.00	3668.00
INTS10	839.00	789.00	848.00	825.33	958.00
INTS12	437.00	405.00	440.00	427.33	474.00
INTS2	1145.00	1057.00	1250.00	1150.67	1156.00
INTS3	7.00	6.00	7.00	6.67	13.00
INTS4	495.00	434.00	531.00	486.67	479.00
INTS4P2	9.00	9.00	13.00	10.33	7.00
INTS5	646.00	607.00	600.00	617.67	556.00
INTS6	1026.00	959.00	1097.00	1027.33	1144.00
INTS6-AS1	41.00	59.00	41.00	47.00	52.00
INTS7	821.00	699.00	764.00	761.33	890.00
INTS8	1619.00	1603.00	1794.00	1672.00	1740.00
INTS9	416.00	430.00	394.00	413.33	482.00
INTU	154.00	153.00	163.00	156.67	221.00
INVS	488.00	436.00	440.00	454.67	472.00
IP6K1	974.00	890.00	1006.00	956.67	994.00
IP6K2	1960.00	1938.00	1998.00	1965.33	2197.00
IPCEF1	5.00	4.00	12.00	7.00	4.00
IPMK	295.00	244.00	295.00	278.00	248.00
IPO11	268.00	236.00	269.00	257.67	252.00
IPO13	1244.00	1292.00	1271.00	1269.00	1160.00
IPO4	2165.00	2189.00	2055.00	2136.33	2458.00
IPO5	5851.00	5233.00	6116.00	5733.33	6678.00
IPO5P1	8.00	15.00	11.00	11.33	12.00
IPO7	8688.00	7729.00	9405.00	8607.33	9079.00
IPO8	1979.00	1772.00	1955.00	1902.00	1909.00
IPO9	2656.00	2629.00	2785.00	2690.00	2363.00
IPO9-AS1	6.00	5.00	0.00	3.67	9.00
IPP	316.00	277.00	282.00	291.67	310.00
IPPK	473.00	452.00	485.00	470.00	598.00
IPW	814.00	827.00	983.00	874.67	852.00
IQCB1	1261.00	1216.00	1330.00	1269.00	1491.00
IQCC	142.00	155.00	168.00	155.00	203.00
IQCD	24.00	25.00	22.00	23.67	17.00
IQCE	382.00	402.00	494.00	426.00	388.00
IQCG	465.00	413.00	455.00	444.33	629.00
IQCH	114.00	128.00	121.00	121.00	118.00
IQCH-AS1	245.00	253.00	254.00	250.67	191.00
IQCJ	16.00	22.00	18.00	18.67	24.00
IQCK	69.00	65.00	67.00	67.00	81.00
IQGAP1	7749.00	7020.00	8309.00	7692.67	6382.00
IQGAP2	17.00	18.00	13.00	16.00	6.00
IQGAP3	3222.00	3057.00	3077.00	3118.67	2840.00
IQSEC1	687.00	669.00	810.00	722.00	574.00
IQSEC2	73.00	77.00	63.00	71.00	69.00

IQUB	9.00	8.00	10.00	9.00	12.00
IRAIN	18.00	9.00	10.00	12.33	17.00
IRAK1	5417.00	5680.00	5783.00	5626.67	6788.00
IRAK1BP1	174.00	159.00	198.00	177.00	181.00
IRAK2	259.00	261.00	249.00	256.33	230.00
IRAK3	12.00	9.00	8.00	9.67	8.00
IRAK4	605.00	631.00	651.00	629.00	857.00
IREB2	1257.00	1184.00	1427.00	1289.33	1010.00
IRF1	270.00	259.00	320.00	283.00	294.00
IRF2	309.00	282.00	345.00	312.00	348.00
IRF2BP1	595.00	616.00	720.00	643.67	821.00
IRF2BP2	2625.00	2486.00	2990.00	2700.33	2927.00
IRF2BPL	1253.00	1176.00	1476.00	1301.67	1051.00
IRF3	1598.00	1603.00	1655.00	1618.67	1732.00
IRF7	6.00	6.00	2.00	4.67	4.00
IRF9	331.00	303.00	330.00	321.33	340.00
IRG1	5.00	7.00	6.00	6.00	8.00
IRGQ	919.00	868.00	996.00	927.67	716.00
IRS1	3445.00	3351.00	3869.00	3555.00	4152.00
IRS2	572.00	523.00	649.00	581.33	672.00
IRX3	148.00	128.00	175.00	150.33	129.00
IRX5	34.00	36.00	44.00	38.00	32.00
ISCA1	299.00	332.00	366.00	332.33	421.00
ISCA2	208.00	167.00	170.00	181.67	207.00
ISCU	830.00	924.00	932.00	895.33	941.00
ISG15	55.00	59.00	69.00	61.00	48.00
ISG20	415.00	462.00	530.00	469.00	615.00
ISG20L2	886.00	873.00	971.00	910.00	798.00
ISL1	43.00	49.00	37.00	43.00	44.00
ISL2	102.00	79.00	93.00	91.33	108.00
ISM1	111.00	110.00	128.00	116.33	136.00
ISOC1	375.00	325.00	373.00	357.67	542.00
ISOC2	395.00	421.00	486.00	434.00	547.00
ISPD	49.00	50.00	61.00	53.33	57.00
IST1	2287.00	2277.00	2425.00	2329.67	2465.00
ISY1	346.00	334.00	397.00	359.00	406.00
ISYNA1	212.00	186.00	210.00	202.67	175.00
ITCH	1799.00	1585.00	1886.00	1756.67	1904.00
ITFG1	1649.00	1582.00	1668.00	1633.00	1682.00
ITFG2	464.00	471.00	489.00	474.67	488.00
ITFG3	2146.00	2153.00	1995.00	2098.00	2085.00
ITGA1	517.00	447.00	497.00	487.00	272.00
ITGA10	900.00	918.00	948.00	922.00	1574.00
ITGA11	21.00	21.00	24.00	22.00	5.00
ITGA2	4388.00	4102.00	4628.00	4372.67	2355.00
ITGA2B	3.00	6.00	4.00	4.33	2.00
ITGA3	42824.00	42922.00	44349.00	43365.00	22400.00
ITGA4	2256.00	2093.00	2323.00	2224.00	2284.00

ITGA5	7517.00	7724.00	7633.00	7624.67	5894.00
ITGA6	17752.00	16096.00	17542.00	17130.00	20076.00
ITGA7	62.00	47.00	71.00	60.00	61.00
ITGA9-AS1	7.00	4.00	6.00	5.67	13.00
ITGAE	389.00	351.00	391.00	377.00	456.00
ITGAL	3.00	1.00	0.00	1.33	1.00
ITGAV	2369.00	2164.00	2511.00	2348.00	1979.00
ITGAX	115.00	122.00	105.00	114.00	74.00
ITGB1	34834.00	33200.00	34581.00	34205.00	32367.00
ITGB1BP1	595.00	549.00	661.00	601.67	794.00
ITGB1BP2	30.00	27.00	40.00	32.33	29.00
ITGB2	3707.00	3743.00	4030.00	3826.67	3070.00
ITGB2-AS1	686.00	635.00	731.00	684.00	727.00
ITGB3	802.00	847.00	837.00	828.67	184.00
ITGB3BP	346.00	324.00	370.00	346.67	465.00
ITGB4	15.00	15.00	16.00	15.33	7.00
ITGB5	4942.00	5177.00	5090.00	5069.67	2762.00
ITGB7	6.00	2.00	6.00	4.67	2.00
ITGB8	155.00	150.00	200.00	168.33	369.00
ITGBL1	5427.00	5054.00	5389.00	5290.00	4955.00
ITIH4	27.00	30.00	31.00	29.33	22.00
ITM2B	2451.00	2461.00	2402.00	2438.00	2642.00
ITM2C	3773.00	3791.00	3651.00	3738.33	3444.00
ITPA	1282.00	1356.00	1431.00	1356.33	1862.00
ITPKA	168.00	163.00	193.00	174.67	177.00
ITPKB	334.00	349.00	368.00	350.33	448.00
ITPKB-IT1	3.00	0.00	1.00	1.33	1.00
ITPKC	281.00	295.00	338.00	304.67	310.00
ITPR1	410.00	389.00	477.00	425.33	465.00
ITPR1-AS1	17.00	13.00	13.00	14.33	16.00
ITPR2	1526.00	1374.00	1548.00	1482.67	1294.00
ITPR3	3270.00	3157.00	3605.00	3344.00	2209.00
ITPRIP	5632.00	5648.00	5660.00	5646.67	4752.00
ITPRIPL1	10.00	17.00	17.00	14.67	13.00
ITPRIPL2	4132.00	3790.00	4101.00	4007.67	3343.00
ITSN1	924.00	883.00	912.00	906.33	1160.00
ITSN2	1132.00	1012.00	1204.00	1116.00	970.00
IVD	1800.00	1590.00	1782.00	1724.00	1728.00
IVNS1ABP	2589.00	2659.00	2977.00	2741.67	3001.00
IWS1	1350.00	1254.00	1364.00	1322.67	1553.00
IZUMO4	49.00	40.00	72.00	53.67	48.00
JADE1	661.00	596.00	746.00	667.67	786.00
JADE2	851.00	735.00	938.00	841.33	774.00
JADE3	495.00	420.00	481.00	465.33	461.00
JAG1	2544.00	2460.00	2744.00	2582.67	1059.00
JAGN1	614.00	600.00	620.00	611.33	599.00
JAK1	4067.00	3788.00	4181.00	4012.00	3860.00
JAK2	540.00	470.00	542.00	517.33	445.00

JAK3	8.00	4.00	5.00	5.67	3.00
JAM2	185.00	191.00	200.00	192.00	190.00
JAM3	1112.00	1115.00	1161.00	1129.33	1144.00
JARID2	691.00	699.00	799.00	729.67	618.00
JAZF1	166.00	150.00	179.00	165.00	177.00
JAZF1-AS1	6.00	0.00	3.00	3.00	0.00
JDP2	3.00	0.00	1.00	1.33	4.00
JHDM1D-AS1	65.00	90.00	82.00	79.00	87.00
JKAMP	1358.00	1346.00	1344.00	1349.33	1529.00
JMJD1C	4074.00	3439.00	3921.00	3811.33	3663.00
JMJD4	394.00	354.00	309.00	352.33	450.00
JMJD6	678.00	652.00	683.00	671.00	705.00
JMJD7	104.00	90.00	105.00	99.67	100.00
JMJD8	822.00	872.00	856.00	850.00	1005.00
JMY	847.00	623.00	809.00	759.67	576.00
JOSD1	1590.00	1568.00	1751.00	1636.33	1863.00
JOSD2	291.00	290.00	312.00	297.67	336.00
JPH1	219.00	194.00	243.00	218.67	270.00
JPX	165.00	138.00	174.00	159.00	217.00
JRK	2422.00	2285.00	2547.00	2418.00	2044.00
JRKL	875.00	885.00	957.00	905.67	965.00
JTB	1650.00	1617.00	1759.00	1675.33	2118.00
JUN	7846.00	7757.00	7953.00	7852.00	9217.00
JUNB	493.00	542.00	515.00	516.67	741.00
JUND	326.00	274.00	375.00	325.00	268.00
KALRN	17.00	17.00	17.00	17.00	7.00
KANK1	1187.00	1163.00	1288.00	1212.67	909.00
KANK2	3559.00	3603.00	3715.00	3625.67	1478.00
KANSL1	289.00	312.00	309.00	303.33	298.00
KANSL1L	248.00	212.00	301.00	253.67	242.00
KANSL2	543.00	522.00	600.00	555.00	641.00
KANSL3	2122.00	2103.00	2416.00	2213.67	2337.00
KANTR	7.00	9.00	6.00	7.33	19.00
KARS	3131.00	2906.00	3271.00	3102.67	3708.00
KAT2A	4664.00	4928.00	4993.00	4861.67	4615.00
KAT2B	236.00	202.00	236.00	224.67	312.00
KAT5	1314.00	1212.00	1461.00	1329.00	1421.00
KAT6A	1476.00	1195.00	1522.00	1397.67	833.00
KAT6B	1052.00	983.00	1182.00	1072.33	881.00
KAT7	959.00	912.00	960.00	943.67	884.00
KAT8	689.00	712.00	815.00	738.67	701.00
KATNA1	570.00	496.00	495.00	520.33	685.00
KATNAL1	237.00	207.00	258.00	234.00	219.00
KATNAL2	111.00	137.00	145.00	131.00	130.00
KATNB1	1056.00	1008.00	1063.00	1042.33	1195.00
KATNBL1	521.00	492.00	543.00	518.67	631.00
KATNBL1P6	17.00	24.00	43.00	28.00	27.00
KAZALD1	103.00	92.00	75.00	90.00	99.00

KBTBD2	1855.00	1931.00	2044.00	1943.33	2307.00
KBTBD3	42.00	31.00	50.00	41.00	47.00
KBTBD4	244.00	247.00	246.00	245.67	302.00
KBTBD6	364.00	340.00	388.00	364.00	375.00
KBTBD7	81.00	48.00	81.00	70.00	98.00
KBTBD8	238.00	254.00	290.00	260.67	279.00
KC6	287.00	322.00	366.00	325.00	269.00
KCMF1	1106.00	1091.00	1284.00	1160.33	1540.00
KCNA6	3.00	0.00	1.00	1.33	2.00
KCNAB1	10.00	11.00	14.00	11.67	23.00
KCNAB2	3508.00	3577.00	3708.00	3597.67	3118.00
KCNAB3	163.00	149.00	154.00	155.33	136.00
KCNC4	111.00	87.00	117.00	105.00	95.00
KCND1	164.00	183.00	184.00	177.00	104.00
KCNE2	4.00	3.00	2.00	3.00	1.00
KCNE4	136.00	123.00	125.00	128.00	139.00
KCNE5	37.00	32.00	30.00	33.00	35.00
KCNG1	3443.00	3522.00	3639.00	3534.67	4022.00
KCNG3	12.00	14.00	9.00	11.67	19.00
KCNH1	157.00	158.00	201.00	172.00	79.00
KCNH3	16.00	17.00	23.00	18.67	26.00
KCNH5	3.00	1.00	6.00	3.33	2.00
KCNH8	12.00	9.00	4.00	8.33	8.00
KCNIP2	235.00	266.00	274.00	258.33	183.00
KCNIP2-AS1	20.00	21.00	13.00	18.00	20.00
KCNIP3	630.00	620.00	664.00	638.00	584.00
KCNJ14	248.00	251.00	311.00	270.00	248.00
KCNJ15	9.00	14.00	11.00	11.33	8.00
KCNJ2	277.00	260.00	283.00	273.33	351.00
KCNJ2-AS1	11.00	21.00	19.00	17.00	29.00
KCNJ3	4.00	3.00	8.00	5.00	7.00
KCNJ4	5.00	3.00	4.00	4.00	4.00
KCNJ8	76.00	76.00	73.00	75.00	154.00
KCNK1	114.00	109.00	131.00	118.00	121.00
KCNK2	157.00	153.00	181.00	163.67	460.00
KCNK4	6.00	4.00	12.00	7.33	5.00
KCNK6	155.00	157.00	190.00	167.33	103.00
KCNK7	4.00	2.00	3.00	3.00	0.00
KCNMA1	4500.00	4121.00	4763.00	4461.33	4037.00
KCNMB2-AS1	22.00	16.00	18.00	18.67	19.00
KCNMB3	148.00	147.00	161.00	152.00	175.00
KCNMB4	24.00	21.00	23.00	22.67	26.00
KCNN2	83.00	78.00	94.00	85.00	85.00
KCNN4	445.00	495.00	498.00	479.33	525.00
KCNQ1OT1	306.00	305.00	313.00	308.00	193.00
KCNQ3	11.00	15.00	11.00	12.33	7.00
KCNQ5	1736.00	1497.00	1678.00	1637.00	1398.00
KCNQ5-IT1	4.00	2.00	6.00	4.00	6.00

KCNRG	8.00	17.00	12.00	12.33	6.00
KCNS3	59.00	60.00	62.00	60.33	48.00
KCNT2	261.00	187.00	231.00	226.33	206.00
KCNU1	133.00	123.00	144.00	133.33	111.00
KCP	22.00	17.00	22.00	20.33	9.00
KCTD1	407.00	375.00	440.00	407.33	403.00
KCTD10	1394.00	1379.00	1543.00	1438.67	1330.00
KCTD11	461.00	442.00	489.00	464.00	454.00
KCTD12	900.00	827.00	1010.00	912.33	1134.00
KCTD13	577.00	569.00	605.00	583.67	657.00
KCTD15	207.00	223.00	266.00	232.00	214.00
KCTD16	19.00	13.00	11.00	14.33	13.00
KCTD17	284.00	291.00	303.00	292.67	284.00
KCTD18	659.00	675.00	751.00	695.00	755.00
KCTD19	13.00	9.00	13.00	11.67	16.00
KCTD2	582.00	568.00	622.00	590.67	649.00
KCTD20	1250.00	1184.00	1394.00	1276.00	1355.00
KCTD21	149.00	185.00	168.00	167.33	164.00
KCTD21-AS1	13.00	6.00	14.00	11.00	9.00
KCTD3	908.00	799.00	958.00	888.33	938.00
KCTD4	218.00	253.00	251.00	240.67	227.00
KCTD5	670.00	578.00	701.00	649.67	686.00
KCTD6	205.00	194.00	213.00	204.00	250.00
KCTD7	508.00	573.00	645.00	575.33	489.00
KCTD8	3.00	9.00	7.00	6.33	9.00
KCTD9	414.00	403.00	503.00	440.00	478.00
KDELC1	329.00	328.00	323.00	326.67	426.00
KDELC2	1513.00	1372.00	1489.00	1458.00	1588.00
KDELR1	4050.00	4054.00	3777.00	3960.33	4071.00
KDELR2	5614.00	5220.00	5248.00	5360.67	5626.00
KDELR3	527.00	483.00	465.00	491.67	501.00
KDM1A	2275.00	2197.00	2326.00	2266.00	2642.00
KDM1B	749.00	612.00	764.00	708.33	951.00
KDM2A	2640.00	2484.00	2722.00	2615.33	2589.00
KDM2B	698.00	656.00	774.00	709.33	743.00
KDM3A	1421.00	1314.00	1513.00	1416.00	1466.00
KDM3B	2310.00	2139.00	2450.00	2299.67	2192.00
KDM4A	2267.00	2228.00	2295.00	2263.33	2549.00
KDM4A-AS1	48.00	38.00	53.00	46.33	70.00
KDM4B	870.00	795.00	801.00	822.00	750.00
KDM4C	950.00	945.00	1034.00	976.33	993.00
KDM4D	48.00	50.00	64.00	54.00	62.00
KDM5A	1356.00	1331.00	1373.00	1353.33	1288.00
KDM5B	1887.00	1694.00	1921.00	1834.00	1863.00
KDM5C	6262.00	6344.00	6509.00	6371.67	5644.00
KDM6A	671.00	695.00	785.00	717.00	706.00
KDM6B	1381.00	1271.00	1612.00	1421.33	1058.00
KDM7A	1176.00	1038.00	1323.00	1179.00	1067.00

KDM8	78.00	78.00	96.00	84.00	77.00
KDR	64.00	58.00	69.00	63.67	61.00
KDSR	2027.00	1924.00	2006.00	1985.67	2049.00
KEAP1	809.00	862.00	872.00	847.67	976.00
KGFLP1	6.00	6.00	2.00	4.67	5.00
KGFLP2	8.00	14.00	12.00	11.33	22.00
KHDC1	131.00	151.00	147.00	143.00	135.00
KHDRBS1	3523.00	3396.00	3544.00	3487.67	4558.00
KHDRBS3	125.00	123.00	125.00	124.33	170.00
KHK	14.00	21.00	22.00	19.00	30.00
KHNYN	2509.00	2562.00	2853.00	2641.33	2199.00
KHSRP	4495.00	4213.00	4459.00	4389.00	4644.00
KIAA0020	2423.00	2245.00	2523.00	2397.00	2874.00
KIAA0100	5845.00	5576.00	6109.00	5843.33	4642.00
KIAA0101	243.00	204.00	265.00	237.33	429.00
KIAA0141	2186.00	2131.00	2374.00	2230.33	2191.00
KIAA0195	2386.00	2394.00	2451.00	2410.33	1961.00
KIAA0196	1624.00	1576.00	1815.00	1671.67	1769.00
KIAA0226	907.00	794.00	975.00	892.00	998.00
KIAA0226L	24.00	20.00	19.00	21.00	5.00
KIAA0232	847.00	800.00	1014.00	887.00	834.00
KIAA0319L	2607.00	2406.00	2315.00	2442.67	2363.00
KIAA0355	200.00	180.00	216.00	198.67	136.00
KIAA0368	3967.00	3728.00	4171.00	3955.33	3742.00
KIAA0391	659.00	567.00	635.00	620.33	699.00
KIAA0513	232.00	237.00	247.00	238.67	248.00
KIAA0556	969.00	848.00	1000.00	939.00	689.00
KIAA0586	737.00	609.00	691.00	679.00	670.00
KIAA0753	1246.00	1164.00	1194.00	1201.33	1165.00
KIAA0754	2059.00	1511.00	1940.00	1836.67	886.00
KIAA0825	20.00	24.00	16.00	20.00	18.00
KIAA0895	168.00	183.00	207.00	186.00	202.00
KIAA0895L	741.00	788.00	742.00	757.00	717.00
KIAA0907	4459.00	4606.00	5037.00	4700.67	4480.00
KIAA0922	737.00	720.00	750.00	735.67	737.00
KIAA0930	478.00	505.00	560.00	514.33	557.00
KIAA1024	121.00	158.00	146.00	141.67	113.00
KIAA1033	1746.00	1679.00	1859.00	1761.33	1567.00
KIAA1107	274.00	242.00	288.00	268.00	254.00
KIAA1109	2405.00	2179.00	2470.00	2351.33	1890.00
KIAA1143	420.00	346.00	484.00	416.67	429.00
KIAA1161	21.00	26.00	13.00	20.00	21.00
KIAA1191	1701.00	1622.00	1828.00	1717.00	1945.00
KIAA1211	242.00	242.00	235.00	239.67	196.00
KIAA1217	471.00	473.00	522.00	488.67	402.00
KIAA1279	867.00	865.00	962.00	898.00	1061.00
KIAA1324	4.00	10.00	6.00	6.67	1.00
KIAA1324L	75.00	86.00	77.00	79.33	54.00

KIAA1328	127.00	102.00	122.00	117.00	90.00
KIAA1407	410.00	347.00	387.00	381.33	461.00
KIAA1429	2663.00	2505.00	2871.00	2679.67	2597.00
KIAA1462	1006.00	949.00	1167.00	1040.67	505.00
KIAA1467	955.00	866.00	905.00	908.67	930.00
KIAA1468	759.00	744.00	849.00	784.00	644.00
KIAA1522	48.00	39.00	50.00	45.67	42.00
KIAA1524	844.00	746.00	799.00	796.33	1051.00
KIAA1549	1374.00	1329.00	1445.00	1382.67	1203.00
KIAA1549L	4757.00	4485.00	4776.00	4672.67	4113.00
KIAA1551	853.00	825.00	948.00	875.33	855.00
KIAA1586	216.00	190.00	216.00	207.33	248.00
KIAA1656	144.00	171.00	145.00	153.33	264.00
KIAA1671	1240.00	1101.00	1249.00	1196.67	972.00
KIAA1683	5.00	4.00	2.00	3.67	0.00
KIAA1715	919.00	838.00	963.00	906.67	976.00
KIAA1755	128.00	107.00	155.00	130.00	166.00
KIAA1804	407.00	407.00	455.00	423.00	469.00
KIAA1841	138.00	124.00	137.00	133.00	158.00
KIAA1875	89.00	100.00	110.00	99.67	71.00
KIAA1919	206.00	198.00	185.00	196.33	126.00
KIAA1958	155.00	175.00	210.00	180.00	146.00
KIAA2013	1732.00	1694.00	1735.00	1720.33	1748.00
KIAA2018	376.00	276.00	359.00	337.00	315.00
KIAA2026	595.00	603.00	695.00	631.00	535.00
KIDINS220	2451.00	2140.00	2409.00	2333.33	2034.00
KIF11	1254.00	1040.00	1167.00	1153.67	1518.00
KIF12	3.00	0.00	0.00	1.00	0.00
KIF13A	1223.00	1141.00	1365.00	1243.00	1216.00
KIF13B	447.00	416.00	487.00	450.00	378.00
KIF14	1057.00	979.00	1072.00	1036.00	1219.00
KIF15	453.00	399.00	399.00	417.00	596.00
KIF16B	515.00	501.00	535.00	517.00	450.00
KIF17	46.00	43.00	36.00	41.67	28.00
KIF18A	855.00	873.00	859.00	862.33	1170.00
KIF18B	1009.00	985.00	949.00	981.00	1337.00
KIF1B	1438.00	1294.00	1596.00	1442.67	1207.00
KIF1C	4882.00	4955.00	4924.00	4920.33	5158.00
KIF20A	559.00	558.00	555.00	557.33	695.00
KIF20B	1447.00	1323.00	1462.00	1410.67	1403.00
KIF21A	1228.00	1175.00	1338.00	1247.00	1221.00
KIF21B	1084.00	905.00	1119.00	1036.00	612.00
KIF22	1307.00	1273.00	1296.00	1292.00	1676.00
KIF23	1292.00	1224.00	1233.00	1249.67	1508.00
KIF24	286.00	241.00	273.00	266.67	344.00
KIF27	196.00	173.00	143.00	170.67	145.00
KIF2A	912.00	874.00	928.00	904.67	1062.00
KIF2C	1365.00	1351.00	1407.00	1374.33	1791.00

KIF3A	716.00	691.00	675.00	694.00	635.00
KIF3B	1343.00	1142.00	1345.00	1276.67	1300.00
KIF3C	582.00	478.00	564.00	541.33	598.00
KIF4A	1228.00	1101.00	1121.00	1150.00	1425.00
KIF4B	17.00	10.00	18.00	15.00	18.00
KIF5B	4622.00	3961.00	4949.00	4510.67	4078.00
KIF5C	246.00	241.00	268.00	251.67	175.00
KIF6	60.00	41.00	63.00	54.67	52.00
KIF7	940.00	915.00	914.00	923.00	754.00
KIF9	120.00	157.00	166.00	147.67	191.00
KIF9-AS1	49.00	61.00	46.00	52.00	29.00
KIFAP3	615.00	563.00	665.00	614.33	658.00
KIFC1	8.00	5.00	9.00	7.33	4.00
KIFC2	2638.00	2940.00	3087.00	2888.33	2685.00
KIFC3	2000.00	2091.00	2101.00	2064.00	2223.00
KIN	598.00	608.00	557.00	587.67	639.00
KIRREL	5736.00	5305.00	5783.00	5608.00	4839.00
KISS1	40.00	49.00	40.00	43.00	21.00
KIT	64.00	67.00	53.00	61.33	10.00
KITLG	207.00	187.00	190.00	194.67	192.00
KIZ	453.00	434.00	520.00	469.00	480.00
KLC1	1841.00	1834.00	1896.00	1857.00	2067.00
KLC2	2586.00	2561.00	2809.00	2652.00	2702.00
KLC4	742.00	767.00	855.00	788.00	672.00
KLF10	719.00	692.00	797.00	736.00	799.00
KLF11	60.00	67.00	96.00	74.33	95.00
KLF12	349.00	326.00	373.00	349.33	348.00
KLF13	31.00	31.00	41.00	34.33	43.00
KLF14	14.00	14.00	8.00	12.00	23.00
KLF15	147.00	124.00	154.00	141.67	151.00
KLF16	1138.00	1259.00	1268.00	1221.67	1344.00
KLF2	345.00	328.00	298.00	323.67	625.00
KLF3	1574.00	1365.00	1734.00	1557.67	1627.00
KLF3-AS1	55.00	56.00	37.00	49.33	68.00
KLF4	414.00	390.00	407.00	403.67	372.00
KLF5	46.00	29.00	38.00	37.67	45.00
KLF6	2295.00	2242.00	2511.00	2349.33	2404.00
KLF7	1115.00	987.00	1243.00	1115.00	1055.00
KLF9	230.00	227.00	276.00	244.33	269.00
KLHDC1	38.00	36.00	43.00	39.00	41.00
KLHDC10	1366.00	1245.00	1493.00	1368.00	1352.00
KLHDC2	618.00	592.00	589.00	599.67	605.00
KLHDC3	1091.00	1117.00	1181.00	1129.67	1404.00
KLHDC4	874.00	917.00	903.00	898.00	891.00
KLHDC7B	28.00	15.00	33.00	25.33	10.00
KLHDC8B	92.00	135.00	98.00	108.33	142.00
KLHL11	292.00	286.00	373.00	317.00	346.00
KLHL12	1385.00	1326.00	1463.00	1391.33	1620.00

KLHL13	17.00	24.00	18.00	19.67	22.00
KLHL14	15.00	16.00	10.00	13.67	34.00
KLHL15	209.00	181.00	261.00	217.00	247.00
KLHL17	1090.00	1212.00	1198.00	1166.67	1172.00
KLHL18	703.00	697.00	709.00	703.00	794.00
KLHL2	328.00	327.00	374.00	343.00	340.00
KLHL20	635.00	541.00	591.00	589.00	589.00
KLHL21	2070.00	1993.00	2127.00	2063.33	1751.00
KLHL22	442.00	441.00	513.00	465.33	502.00
KLHL23	16.00	9.00	10.00	11.67	18.00
KLHL24	477.00	412.00	501.00	463.33	365.00
KLHL25	142.00	128.00	131.00	133.67	144.00
KLHL26	169.00	143.00	197.00	169.67	189.00
KLHL28	205.00	171.00	215.00	197.00	180.00
KLHL29	1316.00	1281.00	1369.00	1322.00	1067.00
KLHL3	868.00	796.00	919.00	861.00	754.00
KLHL31	61.00	59.00	61.00	60.33	65.00
KLHL35	49.00	28.00	45.00	40.67	50.00
KLHL36	1721.00	1694.00	1930.00	1781.67	1542.00
KLHL4	320.00	315.00	424.00	353.00	304.00
KLHL41	22.00	14.00	12.00	16.00	17.00
KLHL42	2396.00	2331.00	2699.00	2475.33	2519.00
KLHL5	2935.00	2613.00	3184.00	2910.67	3013.00
KLHL7	816.00	749.00	774.00	779.67	847.00
KLHL7-AS1	37.00	46.00	48.00	43.67	51.00
KLHL8	372.00	314.00	401.00	362.33	417.00
KLHL9	2182.00	1968.00	2370.00	2173.33	2399.00
KLK14	52.00	41.00	65.00	52.67	37.00
KLKB1	7.00	12.00	10.00	9.67	15.00
KLLN	4.00	10.00	8.00	7.33	12.00
KLRAP1	71.00	71.00	89.00	77.00	91.00
KLRC2	42.00	46.00	45.00	44.33	38.00
KLRC3	10.00	8.00	22.00	13.33	4.00
KLRC4	4.00	11.00	5.00	6.67	1.00
KLRG1	43.00	45.00	48.00	45.33	83.00
KMT2A	3497.00	3251.00	3795.00	3514.33	2651.00
KMT2B	2202.00	2215.00	2407.00	2274.67	1858.00
KMT2C	1477.00	1282.00	1626.00	1461.67	1307.00
KMT2D	5618.00	4994.00	6102.00	5571.33	3455.00
KMT2E	1319.00	1176.00	1401.00	1298.67	968.00
KMT2E-AS1	90.00	84.00	84.00	86.00	90.00
KNOP1	373.00	365.00	370.00	369.33	450.00
KNSTRN	605.00	535.00	590.00	576.67	810.00
KNTC1	1318.00	1282.00	1274.00	1291.33	1493.00
KPNA1	2288.00	2168.00	2507.00	2321.00	2495.00
KPNA2	3254.00	2992.00	3234.00	3160.00	4373.00
KPNA3	1113.00	1001.00	1119.00	1077.67	1406.00
KPNA4	3031.00	2695.00	3228.00	2984.67	3721.00

KPNA5	160.00	140.00	178.00	159.33	141.00
KPNA6	2303.00	2138.00	2430.00	2290.33	2196.00
KPNB1	9061.00	8356.00	9279.00	8898.67	9731.00
KPRP	4.00	4.00	6.00	4.67	6.00
KPTN	275.00	270.00	294.00	279.67	288.00
KRAS	918.00	779.00	998.00	898.33	913.00
KRBA1	335.00	343.00	369.00	349.00	348.00
KRBA2	81.00	64.00	78.00	74.33	42.00
KRBOX4	696.00	739.00	729.00	721.33	890.00
KRCC1	634.00	638.00	715.00	662.33	832.00
KREMEN1	342.00	333.00	337.00	337.33	253.00
KREMEN2	6.00	5.00	3.00	4.67	3.00
KRI1	1955.00	1996.00	1936.00	1962.33	2257.00
KRIT1	1347.00	1307.00	1533.00	1395.67	1328.00
KRR1	1019.00	869.00	1053.00	980.33	1101.00
KRT10	452.00	424.00	448.00	441.33	511.00
KRT15	117.00	115.00	104.00	112.00	294.00
KRT18	3996.00	3906.00	4071.00	3991.00	3701.00
KRT7	5781.00	5804.00	6249.00	5944.67	3828.00
KRT79	331.00	321.00	372.00	341.33	298.00
KRT8	1022.00	953.00	1120.00	1031.67	971.00
KRT80	739.00	747.00	810.00	765.33	648.00
KRT81	17011.00	16825.00	17276.00	17037.33	10223.00
KRT83	20.00	32.00	21.00	24.33	15.00
KRT85	7.00	5.00	6.00	6.00	0.00
KRT86	173.00	201.00	164.00	179.33	108.00
KRTAP2-3	28.00	41.00	21.00	30.00	312.00
KRTAP2-4	38.00	58.00	25.00	40.33	98.00
KRTAP4-12	11.00	21.00	11.00	14.33	14.00
KRTAP4-7	34.00	34.00	29.00	32.33	106.00
KRTAP4-9	5.00	4.00	14.00	7.67	22.00
KRTAP5-11	7.00	5.00	6.00	6.00	2.00
KRTAP5-9	10.00	8.00	4.00	7.33	0.00
KRTCAP2	1305.00	1311.00	1324.00	1313.33	1706.00
KRTCAP3	38.00	33.00	47.00	39.33	47.00
KSR1	400.00	361.00	441.00	400.67	405.00
KTN1	5729.00	5125.00	5747.00	5533.67	5628.00
KTN1-AS1	59.00	67.00	54.00	60.00	64.00
KXD1	1674.00	1583.00	1805.00	1687.33	1990.00
KYNU	4.00	2.00	5.00	3.67	7.00
L1CAM	5.00	3.00	1.00	3.00	1.00
L2HGDH	271.00	252.00	285.00	269.33	290.00
L3HYPDH	870.00	862.00	952.00	894.67	741.00
L3MBTL1	422.00	389.00	382.00	397.67	256.00
L3MBTL2	619.00	602.00	608.00	609.67	721.00
L3MBTL3	684.00	699.00	734.00	705.67	802.00
LACC1	413.00	414.00	463.00	430.00	438.00
LACE1	113.00	80.00	93.00	95.33	103.00

LACTB	1018.00	960.00	1052.00	1010.00	1032.00
LACTB2	501.00	449.00	537.00	495.67	636.00
LAG3	7.00	7.00	8.00	7.33	3.00
LAGE3	166.00	186.00	211.00	187.67	287.00
LAMA1	136.00	130.00	158.00	141.33	147.00
LAMA2	15.00	16.00	14.00	15.00	36.00
LAMA3	887.00	823.00	985.00	898.33	385.00
LAMA4	5358.00	5011.00	5671.00	5346.67	5162.00
LAMA5	3667.00	3525.00	3760.00	3650.67	2757.00
LAMB1	27462.00	26647.00	29298.00	27802.33	20644.00
LAMB2	6895.00	6936.00	7434.00	7088.33	5391.00
LAMB2P1	5.00	3.00	5.00	4.33	2.00
LAMB3	28972.00	29501.00	28361.00	28944.67	27079.00
LAMB4	7.00	3.00	4.00	4.67	7.00
LAMC1	21091.00	19798.00	21223.00	20704.00	20158.00
LAMC2	1058.00	1115.00	1116.00	1096.33	1303.00
LAMP1	8565.00	8691.00	8527.00	8594.33	5928.00
LAMP2	3408.00	3120.00	3403.00	3310.33	2904.00
LAMTOR1	1714.00	1727.00	1703.00	1714.67	2039.00
LAMTOR2	507.00	484.00	522.00	504.33	653.00
LAMTOR3	318.00	377.00	382.00	359.00	490.00
LAMTOR4	669.00	754.00	781.00	734.67	901.00
LAMTOR5	930.00	1022.00	1093.00	1015.00	1251.00
LAMTOR5-AS1	4.00	11.00	4.00	6.33	12.00
LANCL1	1617.00	1459.00	1683.00	1586.33	1714.00
LANCL2	404.00	380.00	438.00	407.33	495.00
LAP3	696.00	692.00	759.00	715.67	856.00
LAPTM4A	3381.00	3296.00	3329.00	3335.33	3346.00
LAPTM4B	5424.00	5297.00	5084.00	5268.33	6173.00
LARP1	10728.00	10401.00	11104.00	10744.33	10981.00
LARP1B	351.00	286.00	357.00	331.33	327.00
LARP4	1938.00	1759.00	2180.00	1959.00	2087.00
LARP4B	2543.00	2376.00	2540.00	2486.33	2831.00
LARP6	1542.00	1587.00	1729.00	1619.33	1700.00
LARP7	672.00	709.00	786.00	722.33	879.00
LARS	10977.00	10308.00	11909.00	11064.67	11099.00
LARS2	1436.00	1383.00	1402.00	1407.00	1425.00
LAS1L	2776.00	2728.00	2972.00	2825.33	3031.00
LASP1	4497.00	4092.00	4217.00	4268.67	3541.00
LAT	626.00	616.00	747.00	663.00	455.00
LAT2	30.00	37.00	45.00	37.33	211.00
LATS1	791.00	690.00	882.00	787.67	770.00
LATS2	511.00	452.00	484.00	482.33	521.00
LAYN	628.00	589.00	667.00	628.00	842.00
LBHD1	567.00	553.00	599.00	573.00	809.00
LBR	2435.00	2095.00	2221.00	2250.33	2311.00
LBX2	78.00	74.00	87.00	79.67	87.00
LBX2-AS1	153.00	139.00	135.00	142.33	143.00

LCA5	142.00	121.00	148.00	137.00	146.00
LCA5L	30.00	33.00	32.00	31.67	31.00
LCAT	204.00	163.00	181.00	182.67	204.00
LCLAT1	585.00	503.00	615.00	567.67	695.00
LCMT1	362.00	358.00	358.00	359.33	424.00
LCMT1-AS1	5.00	11.00	11.00	9.00	9.00
LCMT2	264.00	279.00	340.00	294.33	308.00
LCOR	537.00	521.00	650.00	569.33	505.00
LCORL	144.00	123.00	139.00	135.33	125.00
LCP1	65.00	78.00	80.00	74.33	43.00
LCT	3.00	1.00	1.00	1.67	0.00
LCTL	69.00	65.00	60.00	64.67	107.00
LDAH	581.00	566.00	626.00	591.00	642.00
LDB1	1794.00	1702.00	1996.00	1830.67	1573.00
LDHA	8179.00	7646.00	8420.00	8081.67	13030.00
LDHB	16543.00	15348.00	17751.00	16547.33	23576.00
LDHC	72.00	59.00	73.00	68.00	113.00
LDHD	15.00	11.00	13.00	13.00	20.00
LDLR	2008.00	1988.00	1991.00	1995.67	1257.00
LDLRAD3	1079.00	940.00	981.00	1000.00	1273.00
LDLRAP1	277.00	291.00	308.00	292.00	335.00
LDOC1L	647.00	607.00	687.00	647.00	496.00
LEAP2	56.00	60.00	47.00	54.33	66.00
LEF1	238.00	214.00	274.00	242.00	352.00
LEF1-AS1	13.00	4.00	10.00	9.00	12.00
LEMD2	2337.00	2283.00	2353.00	2324.33	2259.00
LEMD3	856.00	776.00	890.00	840.67	723.00
LENG8	187.00	176.00	121.00	161.33	80.00
LENG8-AS1	6.00	9.00	11.00	8.67	13.00
LEO1	671.00	634.00	645.00	650.00	795.00
LEPR	362.00	323.00	384.00	356.33	397.00
LEPROT	2236.00	2137.00	2189.00	2187.33	2374.00
LEPROTL1	809.00	721.00	738.00	756.00	893.00
LETM1	2751.00	2757.00	2955.00	2821.00	2996.00
LETM2	565.00	512.00	575.00	550.67	692.00
LETMD1	2593.00	2404.00	2652.00	2549.67	2592.00
LFNG	42.00	41.00	39.00	40.67	35.00
LGALS1	6169.00	6047.00	6559.00	6258.33	7989.00
LGALS3	835.00	851.00	941.00	875.67	953.00
LGALS3BP	17787.00	18539.00	18567.00	18297.67	17045.00
LGALS8	1148.00	1117.00	1186.00	1150.33	1052.00
LGALS9	5.00	3.00	0.00	2.67	2.00
LGALSL	68.00	83.00	103.00	84.67	121.00
LGI2	3.00	5.00	0.00	2.67	1.00
LGMN	1381.00	1422.00	1298.00	1367.00	1581.00
LGR4	152.00	158.00	185.00	165.00	218.00
LGR5	702.00	657.00	741.00	700.00	458.00
LGR6	4.00	1.00	2.00	2.33	0.00

LHB	26.00	23.00	16.00	21.67	24.00
LHFP	495.00	472.00	460.00	475.67	404.00
LHFPL2	1374.00	1278.00	1492.00	1381.33	1433.00
LHPP	114.00	103.00	118.00	111.67	128.00
LHX4	3.00	1.00	4.00	2.67	1.00
LHX4-AS1	736.00	791.00	832.00	786.33	799.00
LHX9	4.00	0.00	7.00	3.67	2.00
LIAS	953.00	1014.00	1108.00	1025.00	1071.00
LIF	8994.00	9013.00	9582.00	9196.33	8807.00
LIFR	836.00	711.00	937.00	828.00	570.00
LIFR-AS1	17.00	16.00	22.00	18.33	24.00
LIG1	685.00	822.00	788.00	765.00	929.00
LIG3	868.00	826.00	823.00	839.00	768.00
LIG4	584.00	504.00	596.00	561.33	545.00
LIMA1	1228.00	1183.00	1359.00	1256.67	1211.00
LIMCH1	4.00	1.00	1.00	2.00	1.00
LIMD1	2068.00	1999.00	2228.00	2098.33	2077.00
LIMD1-AS1	3.00	7.00	7.00	5.67	6.00
LIMD2	658.00	698.00	657.00	671.00	716.00
LIME1	994.00	1127.00	1191.00	1104.00	1148.00
LIMK1	1923.00	1961.00	1988.00	1957.33	1513.00
LIMK2	332.00	276.00	303.00	303.67	352.00
LIMS1	832.00	755.00	904.00	830.33	1050.00
LIN28B	74.00	57.00	65.00	65.33	71.00
LIN37	211.00	187.00	197.00	198.33	266.00
LIN52	197.00	179.00	193.00	189.67	210.00
LIN54	235.00	197.00	248.00	226.67	266.00
LIN7B	63.00	74.00	83.00	73.33	65.00
LIN7C	356.00	365.00	430.00	383.67	534.00
LIN9	233.00	219.00	233.00	228.33	298.00
LINC00094	661.00	639.00	696.00	665.33	713.00
LINC00115	24.00	28.00	36.00	29.33	20.00
LINC00116	223.00	241.00	220.00	228.00	332.00
LINC00152	298.00	275.00	306.00	293.00	303.00
LINC00173	41.00	50.00	39.00	43.33	67.00
LINC00174	789.00	755.00	861.00	801.67	762.00
LINC00176	99.00	96.00	96.00	97.00	80.00
LINC00184	11.00	7.00	11.00	9.67	15.00
LINC00189	40.00	45.00	71.00	52.00	56.00
LINC00202-1	3.00	23.00	22.00	16.00	14.00
LINC00211	20.00	26.00	16.00	20.67	30.00
LINC00240	9.00	13.00	14.00	12.00	20.00
LINC00260	6.00	4.00	5.00	5.00	4.00
LINC00261	561.00	567.00	567.00	565.00	370.00
LINC00265	239.00	218.00	235.00	230.67	221.00
LINC00271	3.00	2.00	4.00	3.00	1.00
LINC00282	8.00	5.00	2.00	5.00	4.00
LINC00294	375.00	369.00	423.00	389.00	418.00

LINC00304	3.00	2.00	2.00	2.33	2.00
LINC00310	11.00	8.00	14.00	11.00	28.00
LINC00311	5.00	5.00	2.00	4.00	5.00
LINC00312	3.00	2.00	4.00	3.00	4.00
LINC00317	3.00	3.00	7.00	4.33	6.00
LINC00324	10.00	10.00	9.00	9.67	6.00
LINC00326	33.00	33.00	38.00	34.67	64.00
LINC00327	9.00	11.00	7.00	9.00	8.00
LINC00339	287.00	324.00	312.00	307.67	402.00
LINC00341	171.00	170.00	209.00	183.33	185.00
LINC00342	707.00	697.00	710.00	704.67	401.00
LINC00346	290.00	263.00	265.00	272.67	221.00
LINC00412	17.00	8.00	6.00	10.33	17.00
LINC00431	6.00	7.00	10.00	7.67	6.00
LINC00441	4.00	3.00	0.00	2.33	2.00
LINC00452	6.00	4.00	3.00	4.33	4.00
LINC00460	3.00	3.00	1.00	2.33	1.00
LINC00461	29.00	27.00	26.00	27.33	27.00
LINC00467	157.00	138.00	151.00	148.67	212.00
LINC00470	127.00	134.00	130.00	130.33	132.00
LINC00471	45.00	58.00	55.00	52.67	51.00
LINC00472	447.00	442.00	513.00	467.33	512.00
LINC00475	3.00	1.00	0.00	1.33	1.00
LINC00476	96.00	78.00	94.00	89.33	95.00
LINC00486	3.00	1.00	0.00	1.33	0.00
LINC00493	579.00	659.00	608.00	615.33	906.00
LINC00515	3.00	0.00	1.00	1.33	3.00
LINC00518	11.00	9.00	13.00	11.00	18.00
LINC00520	5.00	9.00	1.00	5.00	1.00
LINC00526	82.00	93.00	82.00	85.67	99.00
LINC00536	3.00	2.00	0.00	1.67	0.00
LINC00562	5.00	6.00	8.00	6.33	8.00
LINC00565	773.00	766.00	785.00	774.67	734.00
LINC00571	5.00	1.00	1.00	2.33	2.00
LINC00592	15.00	16.00	14.00	15.00	24.00
LINC00598	12.00	3.00	5.00	6.67	0.00
LINC00601	36.00	53.00	44.00	44.33	43.00
LINC00615	3.00	1.00	3.00	2.33	0.00
LINC00622	58.00	62.00	51.00	57.00	44.00
LINC00623	151.00	157.00	141.00	149.67	158.00
LINC00629	77.00	75.00	61.00	71.00	66.00
LINC00630	86.00	80.00	89.00	85.00	71.00
LINC00632	80.00	88.00	102.00	90.00	57.00
LINC00634	3.00	2.00	4.00	3.00	4.00
LINC00638	145.00	127.00	168.00	146.67	113.00
LINC00640	11.00	14.00	9.00	11.33	8.00
LINC00641	582.00	554.00	659.00	598.33	369.00
LINC00643	77.00	66.00	64.00	69.00	101.00

LINC00645	5.00	2.00	4.00	3.67	4.00
LINC00648	129.00	126.00	150.00	135.00	152.00
LINC00654	19.00	11.00	10.00	13.33	12.00
LINC00657	9769.00	8800.00	10775.00	9781.33	9603.00
LINC00659	3.00	0.00	0.00	1.00	7.00
LINC00662	100.00	88.00	94.00	94.00	113.00
LINC00663	36.00	20.00	38.00	31.33	36.00
LINC00665	35.00	34.00	42.00	37.00	41.00
LINC00667	1447.00	1411.00	1594.00	1484.00	1547.00
LINC00669	14.00	15.00	8.00	12.33	18.00
LINC00672	16.00	11.00	19.00	15.33	14.00
LINC00673	60.00	56.00	61.00	59.00	44.00
LINC00674	813.00	802.00	941.00	852.00	729.00
LINC00682	3.00	0.00	0.00	1.00	0.00
LINC00685	8.00	18.00	9.00	11.67	11.00
LINC00696	3.00	0.00	1.00	1.33	0.00
LINC00702	43.00	46.00	48.00	45.67	33.00
LINC00704	3258.00	3161.00	3383.00	3267.33	2197.00
LINC00706	8.00	8.00	14.00	10.00	13.00
LINC00707	948.00	823.00	899.00	890.00	2106.00
LINC00839	848.00	912.00	954.00	904.67	1060.00
LINC00842	25.00	21.00	25.00	23.67	15.00
LINC00847	239.00	225.00	287.00	250.33	243.00
LINC00852	35.00	51.00	51.00	45.67	38.00
LINC00857	15.00	19.00	12.00	15.33	14.00
LINC00858	39.00	35.00	40.00	38.00	36.00
LINC00862	6.00	5.00	14.00	8.33	10.00
LINC00869	48.00	54.00	49.00	50.33	43.00
LINC00880	6.00	4.00	11.00	7.00	9.00
LINC00882	18.00	17.00	11.00	15.33	19.00
LINC00883	226.00	200.00	218.00	214.67	240.00
LINC00886	43.00	20.00	23.00	28.67	25.00
LINC00888	145.00	145.00	166.00	152.00	147.00
LINC00889	4.00	9.00	7.00	6.67	4.00
LINC00893	20.00	15.00	22.00	19.00	13.00
LINC00894	96.00	92.00	108.00	98.67	93.00
LINC00896	8.00	11.00	12.00	10.33	8.00
LINC00899	181.00	167.00	177.00	175.00	193.00
LINC00909	234.00	215.00	251.00	233.33	318.00
LINC00910	68.00	83.00	56.00	69.00	72.00
LINC00917	39.00	36.00	42.00	39.00	69.00
LINC00920	42.00	31.00	33.00	35.33	55.00
LINC00921	45.00	51.00	46.00	47.33	56.00
LINC00922	54.00	44.00	44.00	47.33	52.00
LINC00926	35.00	40.00	28.00	34.33	44.00
LINC00936	26.00	26.00	25.00	25.67	11.00
LINC00938	231.00	218.00	229.00	226.00	244.00
LINC00941	343.00	386.00	359.00	362.67	450.00

LINC00942	78.00	93.00	89.00	86.67	113.00
LINC00950	12.00	6.00	13.00	10.33	3.00
LINC00957	10.00	2.00	13.00	8.33	10.00
LINC00959	48.00	28.00	44.00	40.00	57.00
LINC00960	24.00	32.00	33.00	29.67	29.00
LINC00963	324.00	304.00	322.00	316.67	292.00
LINC00987	3.00	1.00	2.00	2.00	1.00
LINC00997	131.00	147.00	166.00	148.00	183.00
LINC00998	857.00	835.00	870.00	854.00	1078.00
LINC00999	210.00	195.00	226.00	210.33	156.00
LINC01000	3545.00	3234.00	3644.00	3474.33	2737.00
LINC01003	67.00	57.00	55.00	59.67	73.00
LINC01004	577.00	649.00	725.00	650.33	494.00
LINC01011	56.00	46.00	48.00	50.00	75.00
LINC01012	4.00	5.00	6.00	5.00	5.00
LINC01021	113.00	98.00	125.00	112.00	87.00
LINC01023	14.00	21.00	14.00	16.33	31.00
LINC01024	56.00	70.00	75.00	67.00	81.00
LINC01029	24.00	31.00	19.00	24.67	27.00
LINC01032	3.00	6.00	9.00	6.00	7.00
LINC01057	3.00	5.00	3.00	3.67	3.00
LINC01061	292.00	302.00	330.00	308.00	243.00
LINC01085	7.00	3.00	6.00	5.33	3.00
LINC01088	7.00	6.00	7.00	6.67	5.00
LINC01089	626.00	607.00	662.00	631.67	605.00
LINC01094	35.00	28.00	53.00	38.67	29.00
LINC01096	3.00	7.00	5.00	5.00	4.00
LINC01098	7.00	3.00	5.00	5.00	5.00
LINC01111	47.00	51.00	70.00	56.00	79.00
LINC01116	385.00	328.00	388.00	367.00	478.00
LINC01117	48.00	23.00	45.00	38.67	48.00
LINC01118	6.00	10.00	8.00	8.00	9.00
LINC01119	37.00	43.00	46.00	42.00	45.00
LINC01124	3.00	3.00	3.00	3.00	0.00
LINC01125	34.00	28.00	40.00	34.00	37.00
LINC01126	134.00	154.00	122.00	136.67	74.00
LINC01128	300.00	286.00	283.00	289.67	248.00
LINC01134	6.00	9.00	12.00	9.00	2.00
LINC01135	9.00	7.00	7.00	7.67	9.00
LINC01137	41.00	28.00	48.00	39.00	60.00
LINC01138	196.00	167.00	185.00	182.67	141.00
LINC01139	225.00	214.00	285.00	241.33	298.00
LINC01140	18.00	22.00	27.00	22.33	31.00
LINC01144	45.00	53.00	45.00	47.67	45.00
LINC01151	5.00	0.00	1.00	2.00	1.00
LINC01152	14.00	7.00	17.00	12.67	28.00
LINC01160	6.00	4.00	7.00	5.67	6.00
LINC01176	13.00	16.00	22.00	17.00	9.00

LINC01184	374.00	317.00	368.00	353.00	437.00
LINC01202	3.00	2.00	7.00	4.00	0.00
LINC01204	356.00	361.00	378.00	365.00	370.00
LINC01212	4.00	1.00	0.00	1.67	2.00
LINC01232	15.00	9.00	19.00	14.33	14.00
LINC01234	111.00	139.00	141.00	130.33	204.00
LINC01239	93.00	98.00	93.00	94.67	83.00
LINC01252	62.00	54.00	69.00	61.67	79.00
LINC01270	105.00	83.00	102.00	96.67	186.00
LINC01277	6.00	10.00	5.00	7.00	3.00
LINC01278	1084.00	960.00	1051.00	1031.67	1017.00
LINC01279	379.00	348.00	348.00	358.33	556.00
LINC01291	22.00	23.00	33.00	26.00	37.00
LINC01296	146.00	144.00	194.00	161.33	136.00
LINC01301	11.00	15.00	15.00	13.67	15.00
LINC01311	97.00	107.00	109.00	104.33	103.00
LINC01317	18.00	15.00	14.00	15.67	15.00
LINC01322	152.00	141.00	130.00	141.00	161.00
LINC01336	3.00	0.00	0.00	1.00	0.00
LINC01340	6.00	2.00	3.00	3.67	2.00
LINC01341	14.00	14.00	14.00	14.00	18.00
LINC01347	3.00	5.00	6.00	4.67	3.00
LINC01348	3.00	5.00	1.00	3.00	2.00
LINC01355	655.00	620.00	736.00	670.33	643.00
LINC01356	10.00	16.00	20.00	15.33	13.00
LINC01358	58.00	61.00	93.00	70.67	145.00
LINC01359	7.00	7.00	11.00	8.33	4.00
LINC01372	26.00	22.00	16.00	21.33	16.00
LINC01389	4.00	4.00	1.00	3.00	2.00
LINC01397	4.00	3.00	2.00	3.00	2.00
LINC01399	76.00	86.00	92.00	84.67	58.00
LINC01410	512.00	470.00	501.00	494.33	408.00
LINC01420	630.00	563.00	637.00	610.00	747.00
LINC01424	19.00	34.00	19.00	24.00	27.00
LINC01425	22.00	16.00	29.00	22.33	25.00
LINC01426	300.00	328.00	311.00	313.00	346.00
LINC01431	6.00	9.00	7.00	7.33	8.00
LINC01433	11.00	5.00	8.00	8.00	5.00
LINC01435	6.00	4.00	8.00	6.00	15.00
LINC01436	419.00	412.00	385.00	405.33	472.00
LINC01461	11.00	12.00	15.00	12.67	9.00
LINC01465	5.00	14.00	10.00	9.67	11.00
LINC01468	42.00	37.00	40.00	39.67	38.00
LINC01473	14.00	7.00	14.00	11.67	12.00
LINC01481	71.00	88.00	77.00	78.67	66.00
LINC01503	3.00	0.00	0.00	1.00	0.00
LINC01508	4.00	1.00	0.00	1.67	0.00
LINC01512	14.00	20.00	18.00	17.33	8.00

LINC01518	62.00	45.00	69.00	58.67	38.00
LINC01521	79.00	88.00	93.00	86.67	109.00
LINC01530	4.00	5.00	8.00	5.67	3.00
LINC01534	14.00	7.00	16.00	12.33	5.00
LINC01535	38.00	30.00	32.00	33.33	30.00
LINC01543	3.00	3.00	1.00	2.33	2.00
LINC01545	6.00	1.00	5.00	4.00	6.00
LINC01547	14.00	8.00	13.00	11.67	6.00
LINC01551	48.00	77.00	66.00	63.67	76.00
LINC01554	4.00	5.00	5.00	4.67	5.00
LINC01560	57.00	54.00	49.00	53.33	68.00
LINC01561	5.00	3.00	4.00	4.00	3.00
LINC01569	97.00	136.00	132.00	121.67	136.00
LINC01572	57.00	64.00	58.00	59.67	58.00
LINC01573	60.00	56.00	59.00	58.33	86.00
LINC01578	468.00	432.00	489.00	463.00	666.00
LINC01583	1519.00	1509.00	1676.00	1568.00	1223.00
LINC01588	36.00	33.00	57.00	42.00	42.00
LINC01590	57.00	63.00	65.00	61.67	57.00
LINC01599	7.00	9.00	14.00	10.00	3.00
LINC01600	7.00	3.00	13.00	7.67	7.00
LINC01602	3.00	2.00	2.00	2.33	4.00
LINC01604	245.00	246.00	223.00	238.00	227.00
LINC01605	905.00	776.00	842.00	841.00	833.00
LINC01611	5.00	12.00	17.00	11.33	11.00
LINC-PINT	1176.00	1164.00	1270.00	1203.33	1047.00
LINCR-0001	6.00	6.00	14.00	8.67	4.00
LINGO2	45.00	40.00	79.00	54.67	39.00
LINS	280.00	243.00	234.00	252.33	253.00
LIPA	3840.00	3586.00	3744.00	3723.33	3639.00
LIPE	16.00	16.00	23.00	18.33	24.00
LIPE-AS1	17.00	16.00	12.00	15.00	22.00
LIPT1	292.00	246.00	304.00	280.67	290.00
LIPT2	17.00	6.00	9.00	10.67	33.00
LITAF	45.00	38.00	57.00	46.67	79.00
LIX1L	1073.00	966.00	1063.00	1034.00	1193.00
LLGL1	1449.00	1434.00	1487.00	1456.67	1347.00
LLGL2	19.00	40.00	29.00	29.33	30.00
LLPH	519.00	483.00	515.00	505.67	622.00
LLPH-AS1	12.00	10.00	14.00	12.00	14.00
LMAN1	6717.00	6004.00	6781.00	6500.67	5975.00
LMAN2	6008.00	6196.00	5767.00	5990.33	4661.00
LMAN2L	800.00	889.00	900.00	863.00	809.00
LMBR1	1180.00	1110.00	1227.00	1172.33	1189.00
LMBR1L	1232.00	1236.00	1317.00	1261.67	1113.00
LMBRD1	661.00	653.00	721.00	678.33	705.00
LMBRD2	341.00	280.00	316.00	312.33	200.00
LMCD1	54.00	70.00	72.00	65.33	59.00

LMF2	1959.00	2047.00	1997.00	2001.00	1973.00
LMLN	869.00	845.00	848.00	854.00	816.00
LMLN-AS1	15.00	20.00	15.00	16.67	20.00
LMNA	3470.00	3437.00	3433.00	3446.67	3962.00
LMNB1	874.00	768.00	878.00	840.00	1287.00
LMNB2	7016.00	7012.00	7178.00	7068.67	6347.00
LMNTD2	3.00	2.00	3.00	2.67	1.00
LMO4	1623.00	1542.00	1836.00	1667.00	2278.00
LMO7	1033.00	1000.00	1053.00	1028.67	947.00
LMOD3	4.00	2.00	1.00	2.33	1.00
LMTK2	1177.00	1203.00	1168.00	1182.67	860.00
LNP1	110.00	94.00	131.00	111.67	153.00
LNPEP	391.00	309.00	344.00	348.00	206.00
LNX1	42.00	37.00	26.00	35.00	43.00
LNX2	197.00	155.00	190.00	180.67	159.00
LOC100049716	3.00	0.00	0.00	1.00	2.00
LOC100126784	169.00	196.00	210.00	191.67	202.00
LOC100128076	3.00	3.00	7.00	4.33	13.00
LOC100128164	5.00	11.00	3.00	6.33	8.00
LOC100128233	17.00	11.00	9.00	12.33	3.00
LOC100128288	74.00	52.00	80.00	68.67	68.00
LOC100128361	44.00	38.00	34.00	38.67	44.00
LOC100128398	9.00	11.00	3.00	7.67	5.00
LOC100128494	82.00	73.00	69.00	74.67	57.00
LOC100128568	19.00	13.00	12.00	14.67	10.00
LOC100128573	3.00	1.00	1.00	1.67	2.00
LOC100128770	10.00	10.00	11.00	10.33	2.00
LOC100129027	16.00	25.00	27.00	22.67	12.00
LOC100129034	1813.00	1818.00	1840.00	1823.67	1329.00
LOC100129203	23.00	26.00	30.00	26.33	15.00
LOC100129434	5.00	1.00	4.00	3.33	1.00
LOC100129461	90.00	69.00	92.00	83.67	59.00
LOC100129518	5.00	1.00	1.00	2.33	7.00
LOC100129534	108.00	89.00	86.00	94.33	77.00
LOC100129550	386.00	348.00	383.00	372.33	307.00
LOC100129617	27.00	28.00	26.00	27.00	31.00
LOC100129917	92.00	85.00	94.00	90.33	33.00
LOC100129924	6.00	6.00	3.00	5.00	2.00
LOC100130357	119.00	121.00	134.00	124.67	92.00
LOC100130451	5.00	11.00	0.00	5.33	2.00
LOC100130691	204.00	188.00	207.00	199.67	201.00
LOC100130705	31.00	31.00	44.00	35.33	24.00
LOC100130744	225.00	228.00	265.00	239.33	213.00
LOC100130950	62.00	81.00	58.00	67.00	61.00
LOC100130987	28.00	35.00	25.00	29.33	38.00
LOC100130992	25.00	22.00	31.00	26.00	22.00
LOC100131564	1945.00	1940.00	2128.00	2004.33	1788.00
LOC100131655	17.00	10.00	7.00	11.33	13.00

LOC100132057	11.00	13.00	16.00	13.33	9.00
LOC100132356	22.00	23.00	18.00	21.00	26.00
LOC100133050	8.00	3.00	2.00	4.33	3.00
LOC100133077	3.00	0.00	0.00	1.00	0.00
LOC100133315	67.00	96.00	90.00	84.33	82.00
LOC100133331	85.00	72.00	96.00	84.33	79.00
LOC100133985	9.00	4.00	17.00	10.00	4.00
LOC100134368	18.00	11.00	13.00	14.00	9.00
LOC100134868	35.00	42.00	45.00	40.67	40.00
LOC100147773	18.00	15.00	10.00	14.33	14.00
LOC100190986	37.00	41.00	37.00	38.33	13.00
LOC100233156	232.00	225.00	280.00	245.67	225.00
LOC100268168	215.00	229.00	203.00	215.67	268.00
LOC100270746	25.00	17.00	27.00	23.00	28.00
LOC100270804	130.00	125.00	114.00	123.00	117.00
LOC100272217	72.00	61.00	75.00	69.33	48.00
LOC100287015	71.00	61.00	53.00	61.67	83.00
LOC100287036	94.00	91.00	92.00	92.33	36.00
LOC100287042	103.00	99.00	77.00	93.00	100.00
LOC100287072	6.00	6.00	10.00	7.33	4.00
LOC100287225	5.00	7.00	5.00	5.67	5.00
LOC100288069	54.00	50.00	63.00	55.67	30.00
LOC100288152	387.00	427.00	474.00	429.33	378.00
LOC100288181	18.00	13.00	21.00	17.33	30.00
LOC100288637	67.00	76.00	71.00	71.33	92.00
LOC100288778	9.00	22.00	23.00	18.00	17.00
LOC100288798	31.00	31.00	38.00	33.33	30.00
LOC100288842	30.00	23.00	25.00	26.00	34.00
LOC100288846	8.00	10.00	5.00	7.67	6.00
LOC100289230	73.00	111.00	99.00	94.33	116.00
LOC100289361	17.00	9.00	13.00	13.00	17.00
LOC100289473	5.00	2.00	3.00	3.33	1.00
LOC100289495	6.00	17.00	25.00	16.00	10.00
LOC100289561	27.00	28.00	23.00	26.00	46.00
LOC100289650	40.00	37.00	36.00	37.67	29.00
LOC100289673	6.00	1.00	5.00	4.00	1.00
LOC100294362	50.00	37.00	46.00	44.33	39.00
LOC100335030	11.00	8.00	13.00	10.67	26.00
LOC100379224	5.00	11.00	15.00	10.33	17.00
LOC100499484	58.00	58.00	48.00	54.67	44.00
LOC100499489	68.00	92.00	71.00	77.00	109.00
LOC100505549	70.00	51.00	60.00	60.33	78.00
LOC100505622	54.00	53.00	50.00	52.33	54.00
LOC100505635	7.00	4.00	3.00	4.67	3.00
LOC100505666	44.00	50.00	60.00	51.33	67.00
LOC100505715	38.00	36.00	44.00	39.33	35.00
LOC100505912	19.00	15.00	18.00	17.33	6.00
LOC100505938	33.00	37.00	27.00	32.33	22.00

LOC100505942	6.00	8.00	2.00	5.33	2.00
LOC100505984	4.00	9.00	12.00	8.33	9.00
LOC100506022	10.00	6.00	19.00	11.67	16.00
LOC100506023	20.00	24.00	23.00	22.33	18.00
LOC100506076	4.00	3.00	4.00	3.67	1.00
LOC100506082	5.00	4.00	3.00	4.00	5.00
LOC100506083	19.00	39.00	23.00	27.00	29.00
LOC100506100	24.00	25.00	19.00	22.67	38.00
LOC100506124	43.00	46.00	48.00	45.67	48.00
LOC100506125	3.00	2.00	0.00	1.67	0.00
LOC100506127	369.00	313.00	390.00	357.33	277.00
LOC100506136	24.00	22.00	26.00	24.00	31.00
LOC100506178	44.00	60.00	56.00	53.33	103.00
LOC100506188	5.00	3.00	10.00	6.00	0.00
LOC100506207	19.00	28.00	21.00	22.67	31.00
LOC100506271	5.00	8.00	10.00	7.67	2.00
LOC100506302	207.00	197.00	243.00	215.67	222.00
LOC100506368	6.00	4.00	2.00	4.00	10.00
LOC100506472	38.00	40.00	42.00	40.00	52.00
LOC100506476	48.00	52.00	57.00	52.33	50.00
LOC100506548	2782.00	2772.00	3175.00	2909.67	2928.00
LOC100506603	256.00	281.00	234.00	257.00	255.00
LOC100506606	49.00	41.00	44.00	44.67	34.00
LOC100506639	196.00	171.00	210.00	192.33	156.00
LOC100506730	61.00	68.00	79.00	69.33	64.00
LOC100506746	52.00	49.00	40.00	47.00	42.00
LOC100506801	5.00	8.00	13.00	8.67	12.00
LOC100506804	21.00	19.00	20.00	20.00	16.00
LOC100506844	133.00	156.00	137.00	142.00	170.00
LOC100506860	72.00	58.00	54.00	61.33	33.00
LOC100506990	34.00	37.00	36.00	35.67	33.00
LOC100507002	8.00	12.00	10.00	10.00	3.00
LOC100507053	86.00	108.00	82.00	92.00	117.00
LOC100507065	1818.00	1658.00	1979.00	1818.33	1350.00
LOC100507144	3.00	6.00	3.00	4.00	7.00
LOC100507156	30.00	22.00	27.00	26.33	20.00
LOC100507250	5.00	16.00	6.00	9.00	16.00
LOC100507283	33.00	39.00	34.00	35.33	32.00
LOC100507291	33.00	39.00	47.00	39.67	23.00
LOC100507373	193.00	223.00	235.00	217.00	247.00
LOC100507412	20.00	58.00	13.00	30.33	10.00
LOC100507424	84.00	85.00	101.00	90.00	111.00
LOC100507437	39.00	28.00	32.00	33.00	24.00
LOC100507462	71.00	55.00	71.00	65.67	91.00
LOC100507487	93.00	85.00	124.00	100.67	111.00
LOC100507506	45.00	37.00	42.00	41.33	45.00
LOC100507557	14.00	10.00	20.00	14.67	7.00
LOC100507564	28.00	23.00	23.00	24.67	24.00

LOC100507577	286.00	286.00	298.00	290.00	308.00
LOC100507600	6.00	4.00	10.00	6.67	19.00
LOC100507634	21.00	12.00	33.00	22.00	18.00
LOC100630923	10.00	8.00	15.00	11.00	16.00
LOC100652758	32.00	30.00	41.00	34.33	47.00
LOC100652768	33.00	17.00	27.00	25.67	15.00
LOC100996351	4.00	1.00	2.00	2.33	2.00
LOC100996437	79.00	78.00	86.00	81.00	95.00
LOC100996634	4.00	0.00	1.00	1.67	0.00
LOC101059948	11.00	23.00	14.00	16.00	21.00
LOC101060091	9.00	7.00	8.00	8.00	12.00
LOC101241902	6.00	9.00	17.00	10.67	5.00
LOC101409256	33.00	18.00	35.00	28.67	22.00
LOC101448202	2689.00	2502.00	2942.00	2711.00	2474.00
LOC101559451	11.00	11.00	8.00	10.00	19.00
LOC101926911	4.00	2.00	4.00	3.33	8.00
LOC101926913	5.00	4.00	6.00	5.00	10.00
LOC101926935	32.00	24.00	24.00	26.67	24.00
LOC101926940	10.00	12.00	10.00	10.67	10.00
LOC101926941	47.00	47.00	43.00	45.67	37.00
LOC101926963	102.00	91.00	98.00	97.00	101.00
LOC101926975	6.00	4.00	13.00	7.67	5.00
LOC101927021	170.00	185.00	170.00	175.00	257.00
LOC101927027	649.00	618.00	611.00	626.00	488.00
LOC101927040	310.00	304.00	341.00	318.33	219.00
LOC101927043	5.00	1.00	1.00	2.33	0.00
LOC101927045	132.00	134.00	124.00	130.00	117.00
LOC101927051	4.00	6.00	6.00	5.33	6.00
LOC101927056	25.00	24.00	33.00	27.33	18.00
LOC101927087	9.00	10.00	15.00	11.33	0.00
LOC101927124	3.00	1.00	0.00	1.33	5.00
LOC101927132	3.00	8.00	5.00	5.33	3.00
LOC101927151	128.00	99.00	130.00	119.00	108.00
LOC101927178	11.00	7.00	7.00	8.33	6.00
LOC101927181	69.00	51.00	69.00	63.00	49.00
LOC101927196	5.00	6.00	2.00	4.33	2.00
LOC101927204	24.00	15.00	33.00	24.00	14.00
LOC101927230	67.00	64.00	75.00	68.67	41.00
LOC101927237	6.00	7.00	2.00	5.00	5.00
LOC101927282	3.00	1.00	3.00	2.33	0.00
LOC101927322	20.00	11.00	14.00	15.00	15.00
LOC101927354	7.00	8.00	12.00	9.00	5.00
LOC101927359	7.00	2.00	7.00	5.33	4.00
LOC101927365	53.00	50.00	54.00	52.33	67.00
LOC101927391	22.00	24.00	22.00	22.67	18.00
LOC101927415	37.00	34.00	36.00	35.67	19.00
LOC101927481	8.00	3.00	12.00	7.67	10.00
LOC101927482	23.00	8.00	18.00	16.33	17.00

LOC101927497	155.00	141.00	124.00	140.00	347.00
LOC101927549	7.00	6.00	5.00	6.00	8.00
LOC101927550	58.00	50.00	55.00	54.33	50.00
LOC101927571	3.00	1.00	5.00	3.00	3.00
LOC101927572	25.00	30.00	25.00	26.67	42.00
LOC101927588	3.00	2.00	3.00	2.67	7.00
LOC101927604	5.00	3.00	3.00	3.67	2.00
LOC101927620	10.00	2.00	4.00	5.33	1.00
LOC101927701	10.00	6.00	11.00	9.00	23.00
LOC101927746	91.00	93.00	88.00	90.67	135.00
LOC101927755	9.00	5.00	9.00	7.67	9.00
LOC101927762	3.00	2.00	5.00	3.33	2.00
LOC101927765	37.00	40.00	55.00	44.00	44.00
LOC101927768	8.00	6.00	11.00	8.33	5.00
LOC101927780	17.00	23.00	32.00	24.00	13.00
LOC101927787	15.00	8.00	13.00	12.00	11.00
LOC101927811	15.00	13.00	15.00	14.33	29.00
LOC101927843	47.00	28.00	37.00	37.33	36.00
LOC101927865	61.00	54.00	59.00	58.00	79.00
LOC101927911	4.00	5.00	2.00	3.67	8.00
LOC101927919	24.00	17.00	19.00	20.00	23.00
LOC101927932	5.00	3.00	15.00	7.67	12.00
LOC101928020	43.00	54.00	47.00	48.00	38.00
LOC101928053	14.00	14.00	20.00	16.00	18.00
LOC101928068	116.00	126.00	120.00	120.67	115.00
LOC101928069	163.00	150.00	128.00	147.00	152.00
LOC101928103	12.00	6.00	8.00	8.67	12.00
LOC101928161	3.00	3.00	9.00	5.00	8.00
LOC101928201	22.00	33.00	20.00	25.00	15.00
LOC101928222	16.00	15.00	16.00	15.67	16.00
LOC101928269	24.00	34.00	28.00	28.67	38.00
LOC101928307	5.00	11.00	6.00	7.33	14.00
LOC101928323	14.00	7.00	6.00	9.00	2.00
LOC101928324	4.00	12.00	6.00	7.33	7.00
LOC101928402	4.00	8.00	8.00	6.67	3.00
LOC101928414	7.00	4.00	7.00	6.00	8.00
LOC101928416	3.00	1.00	0.00	1.33	0.00
LOC101928438	6.00	8.00	1.00	5.00	6.00
LOC101928445	8.00	10.00	9.00	9.00	13.00
LOC101928446	3.00	3.00	3.00	3.00	2.00
LOC101928453	141.00	156.00	165.00	154.00	174.00
LOC101928461	3.00	1.00	2.00	2.00	5.00
LOC101928489	16.00	12.00	20.00	16.00	11.00
LOC101928509	8.00	9.00	25.00	14.00	9.00
LOC101928530	5.00	4.00	2.00	3.67	2.00
LOC101928673	34.00	47.00	42.00	41.00	60.00
LOC101928674	5.00	4.00	10.00	6.33	7.00
LOC101928710	21.00	17.00	23.00	20.33	16.00

LOC101928766	3.00	0.00	1.00	1.33	0.00
LOC101928767	6.00	2.00	2.00	3.33	7.00
LOC101928794	5.00	9.00	6.00	6.67	6.00
LOC101928796	30.00	32.00	38.00	33.33	20.00
LOC101928837	6.00	5.00	8.00	6.33	4.00
LOC101928841	4.00	1.00	4.00	3.00	1.00
LOC101928865	35.00	30.00	26.00	30.33	17.00
LOC101928979	9.00	2.00	8.00	6.33	1.00
LOC101928994	18.00	17.00	19.00	18.00	10.00
LOC101929057	3.00	5.00	12.00	6.67	2.00
LOC101929066	17.00	27.00	25.00	23.00	38.00
LOC101929125	22.00	19.00	27.00	22.67	19.00
LOC101929140	14.00	19.00	13.00	15.33	27.00
LOC101929147	614.00	700.00	716.00	676.67	783.00
LOC101929217	3.00	0.00	0.00	1.00	0.00
LOC101929224	5.00	10.00	8.00	7.67	8.00
LOC101929234	30.00	34.00	47.00	37.00	32.00
LOC101929268	37.00	37.00	37.00	37.00	31.00
LOC101929295	8.00	12.00	8.00	9.33	5.00
LOC101929378	45.00	28.00	33.00	35.33	32.00
LOC101929380	41.00	30.00	30.00	33.67	84.00
LOC101929406	4.00	0.00	1.00	1.67	1.00
LOC101929464	5.00	2.00	2.00	3.00	3.00
LOC101929555	3.00	1.00	3.00	2.33	8.00
LOC101929574	4.00	8.00	3.00	5.00	10.00
LOC101929577	3.00	5.00	7.00	5.00	5.00
LOC101929679	12.00	11.00	7.00	10.00	6.00
LOC101929705	66.00	48.00	77.00	63.67	20.00
LOC101929709	220.00	239.00	250.00	236.33	226.00
LOC101929710	7.00	4.00	13.00	8.00	5.00
LOC101929715	51.00	61.00	45.00	52.33	61.00
LOC101929719	23.00	26.00	16.00	21.67	25.00
LOC101929741	7.00	10.00	8.00	8.33	1.00
LOC101929762	6.00	12.00	5.00	7.67	9.00
LOC101929767	90.00	74.00	107.00	90.33	72.00
LOC101930071	10.00	10.00	11.00	10.33	9.00
LOC101930452	17.00	26.00	19.00	20.67	21.00
LOC102477328	26.00	20.00	18.00	21.33	27.00
LOC102546294	14.00	12.00	15.00	13.67	24.00
LOC102606465	360.00	383.00	416.00	386.33	464.00
LOC102723354	41.00	36.00	25.00	34.00	23.00
LOC102723505	3.00	1.00	5.00	3.00	5.00
LOC102723582	8.00	4.00	11.00	7.67	7.00
LOC102723701	5.00	5.00	4.00	4.67	4.00
LOC102723703	20.00	21.00	31.00	24.00	17.00
LOC102723780	8.00	6.00	7.00	7.00	5.00
LOC102723968	4.00	5.00	6.00	5.00	0.00
LOC102724297	3.00	1.00	0.00	1.33	3.00

LOC102724301	7.00	3.00	3.00	4.33	8.00
LOC102724434	145.00	134.00	150.00	143.00	210.00
LOC102724450	53.00	43.00	56.00	50.67	71.00
LOC102724467	29.00	27.00	24.00	26.67	27.00
LOC102724552	7.00	5.00	3.00	5.00	4.00
LOC102724596	18.00	20.00	20.00	19.33	16.00
LOC102724623	4.00	0.00	5.00	3.00	11.00
LOC102724699	226.00	227.00	224.00	225.67	237.00
LOC102724814	675.00	758.00	759.00	730.67	483.00
LOC102724919	4.00	4.00	2.00	3.33	2.00
LOC103091866	32.00	24.00	49.00	35.00	23.00
LOC103344931	414.00	456.00	434.00	434.67	372.00
LOC103611081	282.00	287.00	277.00	282.00	232.00
LOC103908605	60.00	82.00	90.00	77.33	99.00
LOC104968399	5.00	3.00	0.00	2.67	3.00
LOC105274304	19.00	7.00	9.00	11.67	18.00
LOC105373300	14.00	9.00	12.00	11.67	9.00
LOC105373383	3.00	4.00	5.00	4.00	5.00
LOC105377348	13.00	17.00	26.00	18.67	20.00
LOC105378732	13.00	9.00	11.00	11.00	12.00
LOC105447645	9.00	7.00	14.00	10.00	8.00
LOC105747689	395.00	414.00	422.00	410.33	343.00
LOC105748977	7.00	8.00	3.00	6.00	8.00
LOC106099130	9.00	7.00	2.00	6.00	3.00
LOC113230	8.00	9.00	12.00	9.67	8.00
LOC145783	43.00	53.00	46.00	47.33	57.00
LOC146880	2000.00	2046.00	2255.00	2100.33	2130.00
LOC148413	617.00	658.00	687.00	654.00	698.00
LOC150776	945.00	982.00	1032.00	986.33	936.00
LOC152225	170.00	176.00	169.00	171.67	174.00
LOC153684	95.00	92.00	113.00	100.00	75.00
LOC154761	4.00	2.00	5.00	3.67	2.00
LOC155060	1028.00	1122.00	1197.00	1115.67	783.00
LOC171391	211.00	228.00	203.00	214.00	223.00
LOC202181	73.00	89.00	85.00	82.33	61.00
LOC220729	530.00	527.00	548.00	535.00	467.00
LOC254896	57.00	66.00	83.00	68.67	84.00
LOC255187	14.00	14.00	13.00	13.67	40.00
LOC257396	231.00	214.00	195.00	213.33	253.00
LOC283140	3.00	3.00	5.00	3.67	4.00
LOC283335	12.00	17.00	16.00	15.00	6.00
LOC283352	13.00	15.00	15.00	14.33	13.00
LOC283440	4.00	2.00	2.00	2.67	0.00
LOC283575	9.00	6.00	8.00	7.67	3.00
LOC283683	3.00	11.00	5.00	6.33	7.00
LOC283788	484.00	394.00	474.00	450.67	442.00
LOC283922	153.00	142.00	162.00	152.33	128.00
LOC284009	25.00	32.00	26.00	27.67	33.00

LOC284023	119.00	133.00	150.00	134.00	208.00
LOC284080	192.00	176.00	189.00	185.67	126.00
LOC284454	701.00	760.00	816.00	759.00	780.00
LOC284581	487.00	504.00	559.00	516.67	440.00
LOC284930	22.00	9.00	12.00	14.33	15.00
LOC284950	3.00	0.00	3.00	2.00	0.00
LOC285074	178.00	179.00	231.00	196.00	157.00
LOC285696	15.00	12.00	19.00	15.33	16.00
LOC285768	29.00	12.00	32.00	24.33	23.00
LOC286437	224.00	185.00	257.00	222.00	203.00
LOC339803	280.00	221.00	254.00	251.67	320.00
LOC339874	9.00	10.00	9.00	9.33	13.00
LOC344887	78.00	123.00	110.00	103.67	98.00
LOC374443	296.00	286.00	364.00	315.33	341.00
LOC388849	244.00	308.00	286.00	279.33	258.00
LOC389199	3.00	1.00	3.00	2.33	2.00
LOC389247	10.00	11.00	8.00	9.67	5.00
LOC389602	67.00	52.00	74.00	64.33	71.00
LOC389641	32.00	60.00	47.00	46.33	40.00
LOC389765	50.00	50.00	61.00	53.67	52.00
LOC389831	134.00	124.00	124.00	127.33	168.00
LOC389834	421.00	396.00	462.00	426.33	368.00
LOC389906	136.00	119.00	132.00	129.00	158.00
LOC391322	5.00	11.00	12.00	9.33	8.00
LOC399715	26.00	23.00	40.00	29.67	32.00
LOC399815	318.00	315.00	300.00	311.00	418.00
LOC400655	595.00	568.00	707.00	623.33	518.00
LOC400684	48.00	60.00	40.00	49.33	71.00
LOC400867	3.00	0.00	0.00	1.00	0.00
LOC400927	12.00	13.00	13.00	12.67	5.00
LOC401010	7.00	5.00	14.00	8.67	9.00
LOC401127	15.00	9.00	11.00	11.67	7.00
LOC401320	353.00	329.00	344.00	342.00	316.00
LOC403323	8.00	3.00	6.00	5.67	4.00
LOC407835	9.00	9.00	6.00	8.00	13.00
LOC439994	39.00	43.00	74.00	52.00	42.00
LOC440028	11.00	6.00	22.00	13.00	10.00
LOC440311	22.00	23.00	23.00	22.67	21.00
LOC440434	22.00	27.00	22.00	23.67	17.00
LOC440600	7.00	9.00	8.00	8.00	9.00
LOC440896	11.00	6.00	16.00	11.00	17.00
LOC440982	3.00	2.00	3.00	2.67	8.00
LOC441155	25.00	33.00	32.00	30.00	47.00
LOC441242	127.00	147.00	146.00	140.00	144.00
LOC441666	53.00	46.00	46.00	48.33	36.00
LOC541472	8.00	6.00	3.00	5.67	12.00
LOC550113	4.00	0.00	1.00	1.67	2.00
LOC553103	5.00	5.00	15.00	8.33	6.00

LOC554206	32.00	30.00	30.00	30.67	33.00
LOC613037	16.00	10.00	24.00	16.67	83.00
LOC642361	30.00	43.00	39.00	37.33	51.00
LOC642366	31.00	38.00	34.00	34.33	22.00
LOC642423	18.00	21.00	16.00	18.33	19.00
LOC642846	719.00	752.00	724.00	731.67	750.00
LOC642852	2390.00	2318.00	2617.00	2441.67	1967.00
LOC643072	16.00	15.00	17.00	16.00	25.00
LOC643201	12.00	2.00	7.00	7.00	3.00
LOC643923	3.00	0.00	4.00	2.33	4.00
LOC644285	31.00	40.00	38.00	36.33	18.00
LOC644656	91.00	68.00	65.00	74.67	106.00
LOC645166	72.00	88.00	84.00	81.33	79.00
LOC645513	105.00	105.00	105.00	105.00	97.00
LOC646471	6.00	2.00	1.00	3.00	0.00
LOC646626	24.00	30.00	31.00	28.33	52.00
LOC646762	414.00	366.00	416.00	398.67	417.00
LOC646903	45.00	64.00	50.00	53.00	80.00
LOC648987	255.00	237.00	259.00	250.33	250.00
LOC652276	119.00	88.00	105.00	104.00	77.00
LOC653160	97.00	105.00	95.00	99.00	121.00
LOC653513	3.00	12.00	17.00	10.67	25.00
LOC653602	72.00	65.00	54.00	63.67	90.00
LOC653653	63.00	34.00	64.00	53.67	66.00
LOC653712	20.00	23.00	24.00	22.33	19.00
LOC654342	140.00	144.00	144.00	142.67	151.00
LOC654841	35.00	31.00	33.00	33.00	27.00
LOC727751	3.00	1.00	4.00	2.67	2.00
LOC727896	111.00	92.00	104.00	102.33	145.00
LOC728024	11.00	13.00	15.00	13.00	18.00
LOC728175	4.00	3.00	8.00	5.00	1.00
LOC728323	32.00	29.00	33.00	31.33	28.00
LOC728392	466.00	450.00	464.00	460.00	445.00
LOC728673	30.00	29.00	33.00	30.67	21.00
LOC728730	124.00	125.00	121.00	123.33	118.00
LOC728743	91.00	93.00	121.00	101.67	119.00
LOC728752	19.00	21.00	31.00	23.67	28.00
LOC729218	937.00	810.00	936.00	894.33	537.00
LOC729506	7.00	6.00	8.00	7.00	9.00
LOC729603	157.00	134.00	173.00	154.67	132.00
LOC729683	33.00	24.00	23.00	26.67	18.00
LOC729732	3.00	1.00	1.00	1.67	2.00
LOC729970	116.00	98.00	100.00	104.67	103.00
LOC729987	126.00	122.00	143.00	130.33	173.00
LOC730101	250.00	284.00	288.00	274.00	292.00
LOC730102	476.00	440.00	436.00	450.67	573.00
LOC730183	18.00	21.00	8.00	15.67	22.00
LOC730202	23.00	37.00	27.00	29.00	40.00

LOC730668	7.00	8.00	8.00	7.67	5.00
LOC731157	4.00	4.00	0.00	2.67	2.00
LOC79160	18.00	26.00	18.00	20.67	20.00
LOC81691	212.00	218.00	204.00	211.33	302.00
LOC90246	24.00	22.00	36.00	27.33	34.00
LOC90768	215.00	225.00	204.00	214.67	269.00
LOC90784	289.00	296.00	363.00	316.00	328.00
LOC93622	270.00	318.00	336.00	308.00	388.00
LOH12CR1	121.00	95.00	132.00	116.00	122.00
LOH12CR2	5.00	7.00	4.00	5.33	13.00
LONP1	5030.00	5227.00	5198.00	5151.67	5453.00
LONP2	1645.00	1554.00	1779.00	1659.33	1548.00
LONRF1	490.00	538.00	616.00	548.00	625.00
LONRF2	815.00	775.00	862.00	817.33	639.00
LOX	11571.00	10686.00	10701.00	10986.00	28674.00
LOXL1	3185.00	3187.00	3111.00	3161.00	2923.00
LOXL1-AS1	608.00	562.00	619.00	596.33	715.00
LOXL2	14020.00	13988.00	13937.00	13981.67	11800.00
LOXL3	534.00	531.00	544.00	536.33	341.00
LPAL2	224.00	225.00	276.00	241.67	196.00
LPAR1	1201.00	1145.00	1193.00	1179.67	1038.00
LPAR2	153.00	130.00	168.00	150.33	164.00
LPAR3	209.00	159.00	183.00	183.67	194.00
LPAR6	68.00	74.00	92.00	78.00	58.00
LPCAT1	64.00	35.00	46.00	48.33	40.00
LPCAT2	422.00	361.00	426.00	403.00	361.00
LPCAT3	460.00	463.00	457.00	460.00	367.00
LPCAT4	1408.00	1490.00	1425.00	1441.00	1686.00
LPGAT1	2110.00	1889.00	2107.00	2035.33	2451.00
LPIN1	1393.00	1309.00	1515.00	1405.67	1566.00
LPIN2	4369.00	4110.00	4418.00	4299.00	3475.00
LPIN3	8.00	10.00	14.00	10.67	13.00
LPP	2744.00	2353.00	2928.00	2675.00	2552.00
LPP-AS2	37.00	35.00	41.00	37.67	55.00
LPPR2	635.00	617.00	652.00	634.67	625.00
LPPR4	6.00	4.00	6.00	5.33	1.00
LPPR5	4.00	2.00	5.00	3.67	12.00
LPXN	2701.00	2670.00	2954.00	2775.00	1399.00
LRBA	1139.00	1030.00	1183.00	1117.33	973.00
LRCH1	708.00	635.00	735.00	692.67	782.00
LRCH2	73.00	67.00	69.00	69.67	76.00
LRCH3	442.00	426.00	487.00	451.67	360.00
LRCH4	1277.00	1385.00	1496.00	1386.00	1056.00
LRFN1	97.00	113.00	84.00	98.00	80.00
LRFN3	234.00	251.00	259.00	248.00	273.00
LRFN4	2089.00	2098.00	2153.00	2113.33	2545.00
LRFN5	214.00	234.00	237.00	228.33	364.00
LRGUK	13.00	19.00	23.00	18.33	14.00

LRIF1	662.00	699.00	769.00	710.00	879.00
LRIG1	823.00	746.00	825.00	798.00	646.00
LRIG2	667.00	695.00	730.00	697.33	616.00
LRIG3	246.00	201.00	270.00	239.00	282.00
LRIT3	12.00	15.00	11.00	12.67	17.00
LRP1	3723.00	3533.00	3796.00	3684.00	2310.00
LRP10	5377.00	5236.00	5320.00	5311.00	4265.00
LRP11	959.00	917.00	1025.00	967.00	1175.00
LRP12	1502.00	1362.00	1434.00	1432.67	1420.00
LRP1-AS	4.00	3.00	1.00	2.67	0.00
LRP1B	20.00	19.00	17.00	18.67	12.00
LRP2BP	13.00	24.00	20.00	19.00	25.00
LRP4	480.00	484.00	523.00	495.67	390.00
LRP4-AS1	7.00	5.00	6.00	6.00	8.00
LRP5	370.00	352.00	372.00	364.67	368.00
LRP5L	129.00	148.00	164.00	147.00	134.00
LRP6	1466.00	1373.00	1416.00	1418.33	1277.00
LRP8	2217.00	2206.00	2240.00	2221.00	2138.00
LRPAP1	6619.00	6892.00	6511.00	6674.00	6681.00
LRPPRC	4891.00	4399.00	5327.00	4872.33	5150.00
LRR1	359.00	333.00	350.00	347.33	430.00
LRRC1	146.00	152.00	175.00	157.67	163.00
LRRC14	1800.00	1857.00	1903.00	1853.33	1862.00
LRRC16A	158.00	173.00	174.00	168.33	204.00
LRRC16B	10.00	7.00	10.00	9.00	15.00
LRRC17	1576.00	1519.00	1693.00	1596.00	1485.00
LRRC2	176.00	118.00	166.00	153.33	228.00
LRRC20	1045.00	1034.00	1114.00	1064.33	1230.00
LRRC23	82.00	103.00	96.00	93.67	87.00
LRRC24	109.00	128.00	136.00	124.33	142.00
LRRC27	306.00	280.00	303.00	296.33	307.00
LRRC28	118.00	105.00	130.00	117.67	123.00
LRRC29	43.00	49.00	44.00	45.33	43.00
LRRC2-AS1	22.00	19.00	15.00	18.67	17.00
LRRC34	148.00	175.00	202.00	175.00	204.00
LRRC36	4.00	6.00	2.00	4.00	1.00
LRRC37A3	114.00	119.00	166.00	133.00	155.00
LRRC37A4P	30.00	24.00	18.00	24.00	25.00
LRRC37B	99.00	86.00	107.00	97.33	125.00
LRRC37BP1	294.00	272.00	324.00	296.67	279.00
LRRC39	25.00	30.00	23.00	26.00	25.00
LRRC40	530.00	498.00	611.00	546.33	667.00
LRRC41	2878.00	2820.00	3092.00	2930.00	2724.00
LRRC42	671.00	628.00	698.00	665.67	787.00
LRRC45	470.00	445.00	449.00	454.67	474.00
LRRC46	24.00	15.00	10.00	16.33	27.00
LRRC47	1238.00	1215.00	1223.00	1225.33	1385.00
LRRC48	30.00	13.00	18.00	20.33	18.00

LRRC49	197.00	168.00	224.00	196.33	207.00
LRRC56	56.00	58.00	66.00	60.00	46.00
LRRC57	423.00	391.00	344.00	386.00	412.00
LRRC58	1604.00	1330.00	1792.00	1575.33	1768.00
LRRC59	4777.00	4454.00	4815.00	4682.00	5408.00
LRRC6	71.00	65.00	67.00	67.67	57.00
LRRC63	24.00	22.00	26.00	24.00	22.00
LRRC66	12.00	10.00	14.00	12.00	15.00
LRRC69	81.00	79.00	64.00	74.67	44.00
LRRC71	256.00	260.00	297.00	271.00	238.00
LRRC73	3.00	3.00	4.00	3.33	10.00
LRRC75A-AS1	15065.00	15008.00	16966.00	15679.67	19048.00
LRRC75B	68.00	92.00	64.00	74.67	92.00
LRRC8A	3329.00	3434.00	3434.00	3399.00	2522.00
LRRC8B	369.00	309.00	411.00	363.00	402.00
LRRC8C	1072.00	918.00	984.00	991.33	896.00
LRRC8D	2335.00	2139.00	2357.00	2277.00	2340.00
LRRC8E	624.00	633.00	629.00	628.67	579.00
LRRCC1	339.00	310.00	348.00	332.33	493.00
LRRD1	3.00	2.00	1.00	2.00	4.00
LRRFIP1	2943.00	2613.00	3122.00	2892.67	2934.00
LRRFIP2	1438.00	1351.00	1482.00	1423.67	2080.00
LRRIQ1	3.00	7.00	8.00	6.00	5.00
LRRIQ3	24.00	18.00	12.00	18.00	33.00
LRRIQ4	7.00	4.00	3.00	4.67	11.00
LRRK1	1281.00	1281.00	1450.00	1337.33	1064.00
LRRK2	117.00	89.00	115.00	107.00	125.00
LRRTM2	7.00	11.00	7.00	8.33	10.00
LRRTM3	5.00	0.00	0.00	1.67	0.00
LRSAM1	949.00	960.00	1018.00	975.67	941.00
LRTM2	5.00	6.00	4.00	5.00	3.00
LRTOMT	113.00	94.00	88.00	98.33	143.00
LRWD1	736.00	676.00	731.00	714.33	826.00
LSAMP	4.00	4.00	7.00	5.00	4.00
LSG1	2233.00	2207.00	2380.00	2273.33	2233.00
LSM1	515.00	485.00	502.00	500.67	715.00
LSM10	436.00	477.00	490.00	467.67	620.00
LSM11	387.00	385.00	411.00	394.33	292.00
LSM12	252.00	201.00	210.00	221.00	297.00
LSM14A	27.00	33.00	25.00	28.33	27.00
LSM14B	1661.00	1580.00	1657.00	1632.67	1753.00
LSM3	374.00	365.00	379.00	372.67	514.00
LSM4	1884.00	1975.00	2089.00	1982.67	2530.00
LSM5	969.00	983.00	1134.00	1028.67	1535.00
LSM6	251.00	209.00	243.00	234.33	309.00
LSM7	925.00	962.00	1024.00	970.33	1228.00
LSM8	1289.00	1402.00	1537.00	1409.33	1555.00
LSMEM1	133.00	148.00	158.00	146.33	128.00

LSR	18.00	25.00	21.00	21.33	31.00
LSS	143.00	127.00	138.00	136.00	101.00
LTA4H	2125.00	1871.00	2241.00	2079.00	2286.00
LTB4R	2761.00	2910.00	3122.00	2931.00	2702.00
LTB4R2	677.00	649.00	837.00	721.00	441.00
LTBP1	305.00	284.00	339.00	309.33	210.00
LTBP2	7456.00	7175.00	7967.00	7532.67	6756.00
LTBP3	6706.00	6742.00	7139.00	6862.33	4015.00
LTBP4	4465.00	4493.00	4732.00	4563.33	3682.00
LTBR	3201.00	3513.00	3449.00	3387.67	3279.00
LTK	7.00	16.00	15.00	12.67	8.00
LTN1	1575.00	1530.00	1737.00	1614.00	1526.00
LTV1	1473.00	1470.00	1602.00	1515.00	1826.00
LUC7L	832.00	972.00	909.00	904.33	991.00
LUC7L2	197.00	182.00	229.00	202.67	178.00
LUC7L3	10974.00	11235.00	11942.00	11383.67	11489.00
LUCAT1	34.00	35.00	40.00	36.33	17.00
LURAP1	28.00	32.00	37.00	32.33	33.00
LURAP1L	315.00	339.00	366.00	340.00	465.00
LURAP1L-AS1	442.00	466.00	477.00	461.67	352.00
LUZP1	2109.00	1870.00	2221.00	2066.67	1391.00
LUZP2	6.00	3.00	3.00	4.00	3.00
LVCAT1	34.00	46.00	37.00	39.00	53.00
LVCAT5	6.00	7.00	9.00	7.33	6.00
LVRN	8.00	3.00	22.00	11.00	7.00
LXN	310.00	320.00	318.00	316.00	502.00
LY6K	4189.00	4333.00	4018.00	4180.00	4031.00
LY96	664.00	674.00	577.00	638.33	857.00
LYAR	692.00	680.00	710.00	694.00	919.00
LYG1	26.00	43.00	47.00	38.67	41.00
LYL1	26.00	22.00	17.00	21.67	36.00
LYN	104.00	106.00	105.00	105.00	117.00
LYPD1	86.00	67.00	70.00	74.33	37.00
LYPD6	67.00	68.00	72.00	69.00	105.00
LYPLA1	916.00	714.00	1000.00	876.67	1150.00
LYPLA2	968.00	878.00	964.00	936.67	1114.00
LYPLAL1	535.00	561.00	619.00	571.67	633.00
LYPLAL1-AS1	355.00	341.00	400.00	365.33	212.00
LYRM1	428.00	382.00	456.00	422.00	562.00
LYRM2	812.00	749.00	857.00	806.00	998.00
LYRM4	515.00	492.00	559.00	522.00	647.00
LYRM5	233.00	212.00	251.00	232.00	263.00
LYRM7	937.00	822.00	999.00	919.33	962.00
LYRM9	14.00	25.00	14.00	17.67	26.00
LYSMD1	283.00	286.00	379.00	316.00	314.00
LYSMD2	245.00	180.00	237.00	220.67	285.00
LYSMD3	767.00	645.00	747.00	719.67	662.00
LYSMD4	264.00	282.00	300.00	282.00	261.00

LYST	610.00	616.00	782.00	669.33	490.00
LYZ	5.00	6.00	4.00	5.00	7.00
LZIC	321.00	321.00	370.00	337.33	455.00
LZTFL1	458.00	412.00	479.00	449.67	545.00
LZTR1	2358.00	2381.00	2525.00	2421.33	2322.00
LZTS2	1824.00	1903.00	2035.00	1920.67	1592.00
LZTS3	255.00	259.00	285.00	266.33	269.00
M6PR	4275.00	4325.00	4337.00	4312.33	4145.00
MAATS1	3.00	3.00	1.00	2.33	6.00
MAB21L1	7.00	4.00	16.00	9.00	14.00
MAB21L3	5.00	10.00	2.00	5.67	3.00
MACF1	20133.00	18545.00	20739.00	19805.67	19095.00
MACROD1	1177.00	1201.00	1374.00	1250.67	1376.00
MACROD2	22.00	16.00	20.00	19.33	16.00
MAD1L1	1025.00	1033.00	1083.00	1047.00	1289.00
MAD2L1	478.00	478.00	495.00	483.67	809.00
MAD2L1BP	584.00	500.00	538.00	540.67	648.00
MAD2L2	521.00	531.00	554.00	535.33	649.00
MADD	1565.00	1502.00	1621.00	1562.67	1231.00
MAEA	700.00	717.00	740.00	719.00	849.00
MAF1	2602.00	2845.00	2893.00	2780.00	3236.00
MAFB	39.00	35.00	39.00	37.67	19.00
MAFF	1635.00	1561.00	1585.00	1593.67	2072.00
MAFG	3752.00	3692.00	3981.00	3808.33	3468.00
MAFG-AS1	122.00	97.00	87.00	102.00	136.00
MAFIP	351.00	382.00	458.00	397.00	386.00
MAFK	1340.00	1377.00	1562.00	1426.33	1468.00
MAGEA1	7.00	7.00	8.00	7.33	13.00
MAGEA10	42.00	36.00	44.00	40.67	51.00
MAGEA11	46.00	36.00	65.00	49.00	56.00
MAGEA12	109.00	104.00	104.00	105.67	128.00
MAGEA3	359.00	388.00	395.00	380.67	435.00
MAGEA4	4.00	7.00	6.00	5.67	1.00
MAGEA6	69.00	78.00	85.00	77.33	112.00
MAGEB17	18.00	24.00	19.00	20.33	26.00
MAGEB2	27.00	24.00	38.00	29.67	21.00
MAGEB3	3.00	7.00	10.00	6.67	5.00
MAGEC1	167.00	167.00	171.00	168.33	176.00
MAGEC2	25.00	17.00	12.00	18.00	26.00
MAGEC3	9.00	13.00	14.00	12.00	6.00
MAGED1	2555.00	2352.00	2652.00	2519.67	2593.00
MAGED2	3867.00	3828.00	4063.00	3919.33	3839.00
MAGEE1	38.00	47.00	53.00	46.00	60.00
MAGEF1	867.00	859.00	844.00	856.67	1095.00
MAGEH1	59.00	89.00	67.00	71.67	83.00
MAGI1	1761.00	1703.00	1988.00	1817.33	1651.00
MAGI2	162.00	183.00	188.00	177.67	182.00
MAGI2-AS3	1539.00	1432.00	1634.00	1535.00	1292.00

MAGI3	384.00	346.00	404.00	378.00	424.00
MAGIX	17.00	8.00	12.00	12.33	4.00
MAGOH	747.00	790.00	773.00	770.00	1141.00
MAGOHB	516.00	504.00	575.00	531.67	920.00
MAGT1	2846.00	2664.00	2817.00	2775.67	2474.00
MAK	3.00	0.00	1.00	1.33	10.00
MAK16	763.00	665.00	785.00	737.67	885.00
MALAT1	5984.00	6138.00	6286.00	6136.00	4351.00
MALRD1	6.00	6.00	5.00	5.67	3.00
MALSU1	422.00	444.00	504.00	456.67	501.00
MALT1	734.00	672.00	804.00	736.67	564.00
MAMDC4	435.00	457.00	490.00	460.67	523.00
MAML1	1237.00	1236.00	1238.00	1237.00	1179.00
MAML2	1480.00	1365.00	1572.00	1472.33	1289.00
MAML3	504.00	398.00	490.00	464.00	339.00
MAMLD1	217.00	203.00	197.00	205.67	148.00
MAMSTR	30.00	16.00	22.00	22.67	27.00
MAN1A1	768.00	718.00	797.00	761.00	719.00
MAN1A2	940.00	836.00	969.00	915.00	717.00
MAN1B1	3947.00	3986.00	3905.00	3946.00	3623.00
MAN1B1-AS1	39.00	26.00	36.00	33.67	45.00
MAN1C1	4.00	0.00	1.00	1.67	0.00
MAN2A1	4940.00	4412.00	5225.00	4859.00	3751.00
MAN2A2	1465.00	1518.00	1499.00	1494.00	1182.00
MAN2B1	1793.00	1848.00	1928.00	1856.33	1705.00
MAN2B2	700.00	703.00	646.00	683.00	603.00
MAN2C1	3105.00	3064.00	3372.00	3180.33	2761.00
MANBA	640.00	724.00	705.00	689.67	684.00
MANBAL	1114.00	1042.00	1214.00	1123.33	1287.00
MANEA	882.00	793.00	840.00	838.33	924.00
MANEA-AS1	31.00	49.00	62.00	47.33	45.00
MANEAL	69.00	77.00	84.00	76.67	72.00
MANF	1669.00	1722.00	1580.00	1657.00	1848.00
MANSC1	43.00	45.00	48.00	45.33	45.00
MANSC4	5.00	1.00	2.00	2.67	9.00
MAP1A	52.00	57.00	66.00	58.33	66.00
MAP1B	3469.00	2869.00	3539.00	3292.33	2176.00
MAP1LC3A	23.00	29.00	28.00	26.67	55.00
MAP1LC3B	2099.00	2046.00	2096.00	2080.33	2735.00
MAP1LC3B2	61.00	47.00	70.00	59.33	91.00
MAP1S	939.00	993.00	945.00	959.00	883.00
MAP2	180.00	150.00	203.00	177.67	131.00
MAP2K1	1453.00	1514.00	1609.00	1525.33	1704.00
MAP2K2	2407.00	2472.00	2681.00	2520.00	2949.00
MAP2K3	1308.00	1237.00	1315.00	1286.67	1427.00
MAP2K4	775.00	779.00	870.00	808.00	734.00
MAP2K5	453.00	453.00	531.00	479.00	490.00
MAP2K7	1145.00	1218.00	1239.00	1200.67	1312.00

MAP3K1	259.00	254.00	284.00	265.67	159.00
MAP3K10	545.00	554.00	595.00	564.67	595.00
MAP3K11	1842.00	1806.00	1854.00	1834.00	1736.00
MAP3K12	947.00	1003.00	1003.00	984.33	951.00
MAP3K13	469.00	443.00	484.00	465.33	563.00
MAP3K14	1477.00	1475.00	1517.00	1489.67	1594.00
MAP3K2	1137.00	1105.00	1306.00	1182.67	931.00
MAP3K3	1253.00	1271.00	1306.00	1276.67	1154.00
MAP3K4	862.00	802.00	969.00	877.67	759.00
MAP3K5	177.00	151.00	177.00	168.33	217.00
MAP3K6	389.00	458.00	434.00	427.00	590.00
MAP3K7	1648.00	1659.00	1758.00	1688.33	1774.00
MAP3K7CL	294.00	267.00	309.00	290.00	524.00
MAP3K8	15.00	19.00	31.00	21.67	21.00
MAP3K9	165.00	165.00	172.00	167.33	114.00
MAP4	8760.00	8432.00	8996.00	8729.33	6919.00
MAP4K1	18.00	7.00	19.00	14.67	22.00
MAP4K2	1078.00	1028.00	1091.00	1065.67	1211.00
MAP4K3	1376.00	1195.00	1576.00	1382.33	1309.00
MAP4K4	9164.00	8650.00	9459.00	9091.00	9760.00
MAP4K5	1817.00	1764.00	2060.00	1880.33	1991.00
MAP6	6.00	2.00	3.00	3.67	0.00
MAP6D1	80.00	73.00	98.00	83.67	106.00
MAP7	4.00	8.00	13.00	8.33	12.00
MAP7D1	2003.00	2026.00	1993.00	2007.33	1809.00
MAP7D2	3.00	4.00	4.00	3.67	9.00
MAP7D3	2145.00	1923.00	2132.00	2066.67	1730.00
MAP9	529.00	503.00	589.00	540.33	541.00
MAPK1	2358.00	2216.00	2517.00	2363.67	2597.00
MAPK10	7.00	6.00	9.00	7.33	4.00
MAPK11	309.00	337.00	357.00	334.33	362.00
MAPK12	1028.00	1051.00	1076.00	1051.67	987.00
MAPK13	1214.00	1279.00	1477.00	1323.33	1267.00
MAPK14	1671.00	1437.00	1624.00	1577.33	1692.00
MAPK1IP1L	1940.00	1824.00	2094.00	1952.67	2286.00
MAPK3	678.00	664.00	753.00	698.33	812.00
MAPK6	1232.00	1107.00	1455.00	1264.67	1467.00
MAPK7	313.00	336.00	302.00	317.00	330.00
MAPK8	1151.00	1102.00	1226.00	1159.67	1266.00
MAPK8IP1	227.00	197.00	224.00	216.00	224.00
MAPK8IP2	17.00	15.00	11.00	14.33	20.00
MAPK8IP3	3863.00	4152.00	4404.00	4139.67	3651.00
MAPK9	775.00	796.00	819.00	796.67	922.00
MAPKAP1	2489.00	2262.00	2566.00	2439.00	3298.00
MAPKAPK2	2105.00	2066.00	2331.00	2167.33	2376.00
MAPKAPK3	1228.00	1216.00	1285.00	1243.00	906.00
MAPKAPK5	692.00	677.00	740.00	703.00	803.00
MAPKAPK5-AS1	449.00	444.00	503.00	465.33	501.00

MAPKBP1	1485.00	1309.00	1492.00	1428.67	1312.00
MAPRE1	2655.00	2306.00	2636.00	2532.33	3401.00
MAPRE2	415.00	381.00	434.00	410.00	413.00
MAPRE3	179.00	147.00	172.00	166.00	192.00
MAPT	16.00	15.00	21.00	17.33	14.00
MAR11.	11.00	4.00	2.00	5.67	11.00
MAR3.	55.00	39.00	50.00	48.00	74.00
MAR4.	2731.00	2793.00	2907.00	2810.33	4283.00
MAR5.	949.00	913.00	1096.00	986.00	1129.00
MAR6.	5533.00	5314.00	6055.00	5634.00	5471.00
MAR7.	2184.00	2036.00	2465.00	2228.33	2179.00
MAR8.	59.00	64.00	69.00	64.00	45.00
MAR9.	608.00	547.00	637.00	597.33	595.00
MARCKS	2504.00	2466.00	2577.00	2515.67	2620.00
MARCKSL1	1227.00	1126.00	1219.00	1190.67	1897.00
MARK1	293.00	306.00	354.00	317.67	362.00
MARK2	1377.00	1366.00	1487.00	1410.00	1525.00
MARK3	1993.00	1989.00	2105.00	2029.00	1954.00
MARK4	579.00	598.00	675.00	617.33	548.00
MARS	11869.00	11211.00	11931.00	11670.33	12901.00
MARS2	845.00	815.00	916.00	858.67	860.00
MARVELD1	2377.00	2358.00	2461.00	2398.67	2154.00
MASP1	23.00	23.00	30.00	25.33	9.00
MASP2	5.00	3.00	5.00	4.33	10.00
MAST2	3797.00	3716.00	4058.00	3857.00	3072.00
MAST3	260.00	239.00	268.00	255.67	219.00
MAST4	601.00	508.00	614.00	574.33	628.00
MASTL	533.00	526.00	549.00	536.00	648.00
MAT2A	13774.00	13131.00	14376.00	13760.33	12771.00
MAT2B	1240.00	1198.00	1297.00	1245.00	1545.00
MATN1	5.00	3.00	8.00	5.33	4.00
MATN1-AS1	15.00	11.00	15.00	13.67	11.00
MATN2	153.00	190.00	177.00	173.33	80.00
MATN3	50.00	48.00	57.00	51.67	71.00
MATN4	4.00	7.00	4.00	5.00	3.00
MATR3	7154.00	6377.00	7530.00	7020.33	8106.00
MAU2	1523.00	1457.00	1540.00	1506.67	1458.00
MAVS	4094.00	3783.00	4147.00	4008.00	2874.00
MAX	1273.00	1178.00	1254.00	1235.00	1304.00
MAZ	1955.00	2090.00	2340.00	2128.33	2664.00
MB21D2	388.00	315.00	321.00	341.33	314.00
MBD1	2260.00	2261.00	2328.00	2283.00	2131.00
MBD2	675.00	771.00	810.00	752.00	885.00
MBD3	2866.00	2945.00	3198.00	3003.00	3459.00
MBD4	819.00	771.00	816.00	802.00	852.00
MBD5	251.00	214.00	294.00	253.00	241.00
MBD6	1219.00	1210.00	1395.00	1274.67	1241.00
MBIP	247.00	241.00	275.00	254.33	309.00

MBL1P	182.00	167.00	198.00	182.33	174.00
MBLAC1	11.00	26.00	24.00	20.33	28.00
MBLAC2	435.00	406.00	467.00	436.00	472.00
MBNL1	3846.00	3478.00	4305.00	3876.33	3707.00
MBNL1-AS1	49.00	36.00	46.00	43.67	48.00
MBNL2	515.00	393.00	482.00	463.33	364.00
MBNL3	210.00	187.00	204.00	200.33	254.00
MBOAT1	238.00	257.00	225.00	240.00	256.00
MBOAT2	426.00	365.00	399.00	396.67	412.00
MBOAT7	39.00	43.00	33.00	38.33	24.00
MBP	908.00	865.00	905.00	892.67	650.00
MBTD1	1053.00	975.00	1120.00	1049.33	1067.00
MBTPS1	7117.00	6833.00	6950.00	6966.67	6408.00
MBTPS2	895.00	810.00	836.00	847.00	917.00
MC1R	1530.00	1647.00	1725.00	1634.00	1802.00
MC4R	3.00	0.00	0.00	1.00	4.00
MCAM	97.00	99.00	105.00	100.33	33.00
MCAT	217.00	212.00	237.00	222.00	296.00
MCC	672.00	554.00	644.00	623.33	698.00
MCCC1	771.00	772.00	781.00	774.67	797.00
MCCC2	1344.00	1209.00	1354.00	1302.33	1467.00
MCEE	121.00	119.00	112.00	117.33	125.00
MCF2L	13.00	10.00	11.00	11.33	4.00
MCF2L2	28.00	36.00	40.00	34.67	45.00
MCFD2	16094.00	14551.00	15316.00	15320.33	15302.00
MCIDAS	175.00	161.00	229.00	188.33	221.00
MCL1	14729.00	14120.00	15201.00	14683.33	17909.00
MCM10	324.00	277.00	324.00	308.33	433.00
MCM2	1736.00	1800.00	1770.00	1768.67	2059.00
MCM3	2730.00	2643.00	2790.00	2721.00	3355.00
MCM3AP	3567.00	3535.00	3614.00	3572.00	3331.00
MCM3AP-AS1	207.00	192.00	213.00	204.00	173.00
MCM4	2342.00	2166.00	2326.00	2278.00	2227.00
MCM5	1286.00	1289.00	1189.00	1254.67	1192.00
MCM6	1408.00	1296.00	1414.00	1372.67	1671.00
MCM7	4153.00	4153.00	4112.00	4139.33	6020.00
MCM8	1437.00	1471.00	1511.00	1473.00	1783.00
MCM9	278.00	324.00	333.00	311.67	351.00
MCMBP	1582.00	1395.00	1638.00	1538.33	1922.00
MCMDC2	22.00	27.00	25.00	24.67	25.00
MCOLN1	783.00	680.00	675.00	712.67	649.00
MCOLN2	776.00	770.00	827.00	791.00	926.00
MCOLN3	245.00	254.00	262.00	253.67	214.00
MCPH1	345.00	334.00	360.00	346.33	357.00
MCPH1-AS1	26.00	12.00	24.00	20.67	32.00
MCRS1	1248.00	1280.00	1344.00	1290.67	1571.00
MCTP1	664.00	643.00	730.00	679.00	828.00
MCTS1	1148.00	1069.00	1191.00	1136.00	1281.00

MCTS2P	214.00	240.00	229.00	227.67	189.00
MCU	686.00	611.00	734.00	677.00	702.00
MCUR1	745.00	676.00	801.00	740.67	847.00
MDC1	3.00	4.00	1.00	2.67	1.00
MDFIC	700.00	621.00	832.00	717.67	809.00
MDGA1	72.00	60.00	83.00	71.67	30.00
MDGA2	116.00	99.00	133.00	116.00	102.00
MDH1	2624.00	2432.00	2683.00	2579.67	3565.00
MDH1B	20.00	26.00	27.00	24.33	39.00
MDH2	3421.00	3300.00	3570.00	3430.33	4650.00
MDK	1491.00	1703.00	1543.00	1579.00	1905.00
MDM1	332.00	323.00	358.00	337.67	390.00
MDM2	1127.00	993.00	1133.00	1084.33	1239.00
MDM4	2031.00	2058.00	2196.00	2095.00	2319.00
MDN1	4019.00	3650.00	4433.00	4034.00	3503.00
MDP1	52.00	42.00	42.00	45.33	39.00
ME1	731.00	682.00	760.00	724.33	935.00
ME2	1199.00	1109.00	1240.00	1182.67	1482.00
ME3	67.00	56.00	98.00	73.67	47.00
MEA1	939.00	997.00	1003.00	979.67	1314.00
MEAF6	1212.00	1130.00	1349.00	1230.33	1706.00
MECOM	1079.00	838.00	1098.00	1005.00	1020.00
MECP2	1408.00	1350.00	1530.00	1429.33	1153.00
MECR	451.00	441.00	492.00	461.33	498.00
MED1	2623.00	2333.00	2864.00	2606.67	2186.00
MED10	570.00	560.00	620.00	583.33	769.00
MED11	229.00	209.00	204.00	214.00	282.00
MED12	2019.00	1868.00	2011.00	1966.00	1599.00
MED12L	94.00	109.00	104.00	102.33	74.00
MED13	2217.00	1938.00	2492.00	2215.67	1953.00
MED13L	3008.00	2636.00	3293.00	2979.00	2553.00
MED14	1477.00	1412.00	1588.00	1492.33	1505.00
MED14OS	6.00	0.00	2.00	2.67	0.00
MED15	1916.00	1866.00	2018.00	1933.33	2015.00
MED16	651.00	661.00	649.00	653.67	631.00
MED17	875.00	866.00	905.00	882.00	968.00
MED18	310.00	276.00	323.00	303.00	381.00
MED19	336.00	326.00	354.00	338.67	409.00
MED20	616.00	591.00	658.00	621.67	725.00
MED21	603.00	524.00	577.00	568.00	645.00
MED22	1430.00	1367.00	1442.00	1413.00	1592.00
MED23	1473.00	1411.00	1516.00	1466.67	1353.00
MED24	2293.00	2235.00	2310.00	2279.33	2245.00
MED25	609.00	568.00	600.00	592.33	588.00
MED26	254.00	220.00	221.00	231.67	239.00
MED27	477.00	459.00	497.00	477.67	607.00
MED28	480.00	458.00	530.00	489.33	540.00
MED29	530.00	554.00	633.00	572.33	596.00

MED30	269.00	250.00	277.00	265.33	400.00
MED31	148.00	159.00	150.00	152.33	181.00
MED4	435.00	401.00	400.00	412.00	541.00
MED6	468.00	400.00	453.00	440.33	528.00
MED7	264.00	237.00	309.00	270.00	286.00
MED8	1433.00	1387.00	1521.00	1447.00	1919.00
MED9	222.00	251.00	210.00	227.67	223.00
MEF2A	1251.00	1037.00	1286.00	1191.33	1207.00
MEF2B	6.00	3.00	5.00	4.67	1.00
MEF2BNB	95.00	68.00	78.00	80.33	113.00
MEF2BNB-MEF2B	10.00	7.00	5.00	7.33	9.00
MEF2C	248.00	209.00	261.00	239.33	246.00
MEF2C-AS1	12.00	5.00	11.00	9.33	13.00
MEF2D	1671.00	1644.00	1683.00	1666.00	1442.00
MEGF6	11.00	12.00	7.00	10.00	2.00
MEGF8	2164.00	2029.00	2173.00	2122.00	1392.00
MEGF9	271.00	280.00	319.00	290.00	210.00
MEI1	15.00	12.00	11.00	12.67	15.00
MEIG1	8.00	5.00	8.00	7.00	6.00
MEIS1	124.00	129.00	147.00	133.33	159.00
MEIS1-AS3	38.00	38.00	37.00	37.67	35.00
MEIS2	402.00	397.00	456.00	418.33	445.00
MEIS3	5.00	4.00	8.00	5.67	6.00
MEIS3P1	145.00	139.00	157.00	147.00	151.00
MELK	1100.00	1056.00	997.00	1051.00	1571.00
MEMO1	416.00	420.00	494.00	443.33	652.00
MEN1	3763.00	3527.00	3836.00	3708.67	3627.00
MEPCE	1982.00	1926.00	2009.00	1972.33	1955.00
MERTK	438.00	391.00	471.00	433.33	346.00
MESDC1	750.00	757.00	790.00	765.67	1057.00
MESDC2	1950.00	1963.00	1856.00	1923.00	2024.00
MESP1	12.00	16.00	17.00	15.00	32.00
MESP2	9.00	7.00	8.00	8.00	11.00
MEST	16.00	17.00	26.00	19.67	18.00
MET	7276.00	6975.00	7652.00	7301.00	5277.00
METAP1	1183.00	1151.00	1302.00	1212.00	1546.00
METAP1D	616.00	613.00	630.00	619.67	543.00
METAP2	2173.00	2072.00	2375.00	2206.67	2881.00
METRN	576.00	666.00	691.00	644.33	700.00
METRNL	166.00	176.00	182.00	174.67	183.00
METTL1	389.00	402.00	425.00	405.33	505.00
METTL10	491.00	444.00	515.00	483.33	642.00
METTL12	43.00	46.00	34.00	41.00	49.00
METTL13	1626.00	1512.00	1627.00	1588.33	1688.00
METTL14	374.00	417.00	409.00	400.00	453.00
METTL15	477.00	464.00	495.00	478.67	556.00
METTL16	1108.00	1082.00	1145.00	1111.67	1169.00
METTL17	1418.00	1438.00	1421.00	1425.67	1565.00

METTL18	206.00	204.00	189.00	199.67	282.00
METTL20	55.00	42.00	34.00	43.67	42.00
METTL21A	205.00	182.00	208.00	198.33	312.00
METTL21B	308.00	285.00	333.00	308.67	320.00
METTL21EP	3.00	7.00	3.00	4.33	4.00
METTL22	433.00	419.00	434.00	428.67	416.00
METTL23	598.00	588.00	661.00	615.67	731.00
METTL25	65.00	61.00	62.00	62.67	67.00
METTL2A	479.00	457.00	513.00	483.00	595.00
METTL2B	662.00	623.00	665.00	650.00	714.00
METTL3	2699.00	2844.00	2854.00	2799.00	3041.00
METTL4	965.00	1020.00	1095.00	1026.67	1111.00
METTL5	818.00	758.00	844.00	806.67	1098.00
METTL6	404.00	367.00	406.00	392.33	421.00
METTL7A	4.00	2.00	1.00	2.33	0.00
METTL7B	18.00	12.00	9.00	13.00	9.00
METTL8	674.00	634.00	728.00	678.67	808.00
METTL9	3675.00	3560.00	3851.00	3695.33	5476.00
MEX3A	732.00	765.00	961.00	819.33	889.00
MEX3B	144.00	124.00	166.00	144.67	136.00
MEX3C	981.00	901.00	1075.00	985.67	1259.00
MEX3D	271.00	286.00	281.00	279.33	322.00
MFAP1	709.00	668.00	687.00	688.00	817.00
MFAP2	80.00	111.00	88.00	93.00	90.00
MFAP3	1146.00	981.00	1182.00	1103.00	924.00
MFAP3L	196.00	180.00	207.00	194.33	195.00
MFF	1451.00	1369.00	1579.00	1466.33	1909.00
MFGE8	1987.00	2085.00	2095.00	2055.67	1472.00
MFHAS1	609.00	542.00	650.00	600.33	468.00
MFI2	2272.00	2487.00	2595.00	2451.33	2039.00
MFI2-AS1	93.00	79.00	97.00	89.67	138.00
MFN1	993.00	953.00	1047.00	997.67	1055.00
MFN2	2686.00	2699.00	2954.00	2779.67	3026.00
MFNG	50.00	58.00	66.00	58.00	54.00
MFSD1	2606.00	2519.00	2385.00	2503.33	2574.00
MFSD10	6739.00	6725.00	6858.00	6774.00	7374.00
MFSD11	530.00	510.00	535.00	525.00	659.00
MFSD12	1794.00	1929.00	2049.00	1924.00	1453.00
MFSD2A	363.00	327.00	323.00	337.67	323.00
MFSD3	1002.00	965.00	1050.00	1005.67	915.00
MFSD5	751.00	761.00	787.00	766.33	747.00
MFSD6	122.00	138.00	131.00	130.33	83.00
MFSD8	531.00	562.00	608.00	567.00	589.00
MFSD9	175.00	191.00	187.00	184.33	151.00
MGA	1345.00	1180.00	1427.00	1317.33	1259.00
MGAT1	2685.00	2674.00	2653.00	2670.67	2460.00
MGAT2	1358.00	1169.00	1394.00	1307.00	1434.00
MGAT4A	12.00	4.00	6.00	7.33	14.00

MGAT4B	3949.00	4041.00	4070.00	4020.00	4314.00
MGAT5	2186.00	1930.00	2065.00	2060.33	1924.00
MGAT5B	789.00	774.00	818.00	793.67	445.00
MGC16142	28.00	17.00	17.00	20.67	15.00
MGC16275	3.00	4.00	1.00	2.67	7.00
MGC27345	784.00	750.00	801.00	778.33	769.00
MGC2889	21.00	19.00	21.00	20.33	17.00
MGC57346	103.00	96.00	127.00	108.67	106.00
MGC72080	118.00	141.00	142.00	133.67	187.00
MGEA5	7614.00	7290.00	8237.00	7713.67	7569.00
MGLL	5747.00	5426.00	5711.00	5628.00	6431.00
MGME1	769.00	786.00	860.00	805.00	1022.00
MGRN1	1035.00	983.00	1055.00	1024.33	904.00
MGST1	1765.00	1732.00	1680.00	1725.67	2020.00
MGST2	140.00	168.00	128.00	145.33	174.00
MGST3	1139.00	1139.00	1080.00	1119.33	1154.00
MIA3	3552.00	3299.00	3488.00	3446.33	2946.00
MIAT	15.00	11.00	10.00	12.00	16.00
MIATNB	41.00	39.00	37.00	39.00	44.00
MIB1	1376.00	1260.00	1667.00	1434.33	1300.00
MIB2	1122.00	1218.00	1300.00	1213.33	1010.00
MICA	216.00	233.00	273.00	240.67	291.00
MICAL1	1881.00	1914.00	1993.00	1929.33	1767.00
MICAL2	10066.00	9207.00	10964.00	10079.00	8300.00
MICAL3	3770.00	3682.00	3880.00	3777.33	2904.00
MICALCL	93.00	81.00	81.00	85.00	111.00
MICALL1	1357.00	1248.00	1273.00	1292.67	1019.00
MICALL2	456.00	442.00	504.00	467.33	349.00
MICU1	875.00	925.00	876.00	892.00	1031.00
MICU2	694.00	694.00	748.00	712.00	796.00
MICU3	182.00	151.00	187.00	173.33	154.00
MID1	618.00	572.00	680.00	623.33	515.00
MID1IP1	558.00	583.00	639.00	593.33	684.00
MID1IP1-AS1	23.00	22.00	23.00	22.67	14.00
MIDN	1421.00	1390.00	1505.00	1438.67	916.00
MIEF1	1547.00	1263.00	1548.00	1452.67	1432.00
MIEF2	225.00	211.00	204.00	213.33	158.00
MIEN1	425.00	393.00	457.00	425.00	537.00
MIER1	602.00	545.00	669.00	605.33	699.00
MIER2	749.00	775.00	812.00	778.67	842.00
MIER3	542.00	553.00	630.00	575.00	597.00
MIF	7.00	4.00	3.00	4.67	8.00
MIF4GD	364.00	328.00	375.00	355.67	422.00
MIIP	746.00	820.00	809.00	791.67	978.00
MILR1	359.00	348.00	325.00	344.00	304.00
MINA	1975.00	1861.00	2104.00	1980.00	2266.00
MINK1	4053.00	3971.00	4458.00	4160.67	4064.00
MINOS1	708.00	709.00	701.00	706.00	939.00

MINPP1	1236.00	1208.00	1214.00	1219.33	1272.00
MIOS	1130.00	1065.00	1236.00	1143.67	1381.00
MIOX	10.00	9.00	24.00	14.33	2.00
MIPEP	341.00	355.00	378.00	358.00	461.00
MIPEPP3	51.00	54.00	71.00	58.67	60.00
MIPOL1	446.00	391.00	494.00	443.67	534.00
MIR100HG	5058.00	4985.00	6285.00	5442.67	4338.00
MIR103A2	6.00	5.00	3.00	4.67	8.00
MIR106B	8.00	12.00	7.00	9.00	4.00
MIR10A	7.00	6.00	3.00	5.33	5.00
MIR10B	4.00	3.00	4.00	3.67	5.00
MIR1204	4.00	16.00	9.00	9.67	5.00
MIR1254-1	63.00	74.00	72.00	69.67	53.00
MIR125B1	14.00	10.00	15.00	13.00	0.00
MIR1268A	3.00	14.00	4.00	7.00	8.00
MIR1285-1	8.00	6.00	6.00	6.67	5.00
MIR1299	3.00	0.00	0.00	1.00	0.00
MIR1304	101.00	107.00	122.00	110.00	110.00
MIR137HG	8.00	11.00	11.00	10.00	8.00
MIR138-1	57.00	52.00	48.00	52.33	19.00
MIR142	73.00	60.00	86.00	73.00	96.00
MIR146A	7.00	8.00	5.00	6.67	7.00
MIR155HG	214.00	218.00	243.00	225.00	218.00
MIR17HG	609.00	566.00	752.00	642.33	566.00
MIR186	57.00	38.00	52.00	49.00	19.00
MIR1908	3.00	3.00	5.00	3.67	3.00
MIR1910	4.00	1.00	3.00	2.67	1.00
MIR1914	67.00	61.00	80.00	69.33	87.00
MIR199A1	11.00	18.00	18.00	15.67	16.00
MIR21	28.00	28.00	45.00	33.67	25.00
MIR221	786.00	850.00	880.00	838.67	739.00
MIR222	78.00	66.00	82.00	75.33	58.00
MIR224	11.00	32.00	27.00	23.33	9.00
MIR23A	4.00	2.00	8.00	4.67	2.00
MIR24-1	3.00	2.00	2.00	2.33	2.00
MIR25	49.00	35.00	46.00	43.33	70.00
MIR26A1	3.00	0.00	0.00	1.00	0.00
MIR26A2	3.00	2.00	1.00	2.00	3.00
MIR27A	5.00	3.00	2.00	3.33	2.00
MIR27B	4.00	2.00	6.00	4.00	2.00
MIR298	8.00	1.00	12.00	7.00	4.00
MIR29A	60.00	43.00	42.00	48.33	45.00
MIR29B1	3.00	3.00	13.00	6.33	6.00
MIR29B2	7.00	10.00	24.00	13.67	12.00
MIR29C	23.00	28.00	33.00	28.00	29.00
MIR3064	259.00	225.00	262.00	248.67	303.00
MIR30C2	25.00	18.00	29.00	24.00	26.00
MIR31	3.00	6.00	6.00	5.00	1.00

MIR3136	3.00	3.00	3.00	3.00	1.00
MIR3149	8.00	1.00	7.00	5.33	1.00
MIR3153	6.00	11.00	9.00	8.67	3.00
MIR3164	43.00	26.00	43.00	37.33	36.00
MIR3174	3.00	2.00	0.00	1.67	0.00
MIR3176	41.00	42.00	48.00	43.67	22.00
MIR3191	12.00	10.00	4.00	8.67	2.00
MIR3192	5.00	4.00	5.00	4.67	5.00
MIR31HG	443.00	465.00	523.00	477.00	395.00
MIR339	4.00	1.00	1.00	2.00	1.00
MIR34A	3.00	5.00	6.00	4.67	1.00
MIR3605	7.00	12.00	6.00	8.33	11.00
MIR3607	6.00	6.00	7.00	6.33	4.00
MIR3611	3.00	0.00	0.00	1.00	0.00
MIR3654	1784.00	1689.00	1806.00	1759.67	2210.00
MIR3657	5.00	3.00	1.00	3.00	3.00
MIR3662	4.00	2.00	7.00	4.33	0.00
MIR3671	8.00	5.00	9.00	7.33	2.00
MIR3677	6.00	1.00	6.00	4.33	7.00
MIR3682	37.00	23.00	44.00	34.67	51.00
MIR3685	5.00	10.00	4.00	6.33	11.00
MIR3689B	6.00	5.00	2.00	4.33	0.00
MIR3689C	6.00	4.00	6.00	5.33	0.00
MIR3689F	4.00	4.00	3.00	3.67	1.00
MIR374B	7.00	4.00	6.00	5.67	3.00
MIR378H	3.00	2.00	5.00	3.33	6.00
MIR3911	7.00	5.00	4.00	5.33	9.00
MIR3916	121.00	145.00	134.00	133.33	115.00
MIR3942	5.00	3.00	5.00	4.33	3.00
MIR421	10.00	13.00	4.00	9.00	2.00
MIR423	3.00	1.00	4.00	2.67	4.00
MIR4258	24.00	26.00	22.00	24.00	20.00
MIR4263	4.00	7.00	2.00	4.33	12.00
MIR4271	16.00	18.00	13.00	15.67	10.00
MIR4292	30.00	20.00	27.00	25.67	18.00
MIR4326	4.00	8.00	3.00	5.00	4.00
MIR4426	2697.00	2694.00	3015.00	2802.00	3547.00
MIR4435-2HG	185.00	160.00	177.00	174.00	151.00
MIR4442	33.00	17.00	42.00	30.67	29.00
MIR4458HG	244.00	274.00	297.00	271.67	308.00
MIR4477B	7.00	14.00	11.00	10.67	16.00
MIR4479	3.00	1.00	2.00	2.00	2.00
MIR4482	5.00	1.00	0.00	2.00	1.00
MIR4489	4.00	0.00	1.00	1.67	2.00
MIR4500HG	5.00	9.00	10.00	8.00	22.00
MIR4512	5.00	3.00	1.00	3.00	1.00
MIR4517	51.00	23.00	32.00	35.33	26.00
MIR4653	12.00	9.00	10.00	10.33	7.00

MIR4668	3.00	4.00	1.00	2.67	1.00
MIR4691	27.00	31.00	19.00	25.67	16.00
MIR4697HG	6.00	5.00	7.00	6.00	5.00
MIR4701	9.00	4.00	6.00	6.33	1.00
MIR4712	215.00	183.00	199.00	199.00	99.00
MIR4720	7.00	10.00	8.00	8.33	11.00
MIR4730	8.00	8.00	3.00	6.33	3.00
MIR4746	5.00	10.00	6.00	7.00	3.00
MIR4766	3.00	1.00	2.00	2.00	1.00
MIR4782	3.00	5.00	0.00	2.67	2.00
MIR4784	8.00	11.00	8.00	9.00	2.00
MIR4786	8.00	10.00	4.00	7.33	10.00
MIR4787	3.00	1.00	0.00	1.33	2.00
MIR491	6.00	9.00	6.00	7.00	4.00
MIR499B	8.00	5.00	1.00	4.67	4.00
MIR5006	4.00	3.00	2.00	3.00	4.00
MIR5010	6.00	20.00	19.00	15.00	9.00
MIR503	92.00	118.00	117.00	109.00	73.00
MIR503HG	1054.00	1068.00	1203.00	1108.33	1082.00
MIR5047	1840.00	1895.00	1870.00	1868.33	2251.00
MIR542	3.00	2.00	0.00	1.67	1.00
MIR548A3	6.00	2.00	1.00	3.00	0.00
MIR548AM	4.00	3.00	1.00	2.67	0.00
MIR548AN	4.00	2.00	3.00	3.00	1.00
MIR548AR	16.00	5.00	3.00	8.00	5.00
MIR548D1	3.00	2.00	4.00	3.00	5.00
MIR553	5.00	2.00	4.00	3.67	3.00
MIR5581	14.00	12.00	4.00	10.00	13.00
MIR5587	14.00	15.00	15.00	14.67	4.00
MIR563	3.00	3.00	2.00	2.67	4.00
MIR573	12.00	10.00	18.00	13.33	9.00
MIR574	3.00	1.00	2.00	2.00	0.00
MIR589	6.00	9.00	1.00	5.33	3.00
MIR590	48.00	33.00	47.00	42.67	30.00
MIR600HG	191.00	231.00	224.00	215.33	191.00
MIR614	42.00	45.00	52.00	46.33	37.00
MIR616	19.00	18.00	18.00	18.33	12.00
MIR622	27.00	26.00	20.00	24.33	9.00
MIR631	17.00	12.00	25.00	18.00	22.00
MIR635	19.00	15.00	10.00	14.67	4.00
MIR637	21.00	23.00	25.00	23.00	24.00
MIR641	30.00	24.00	23.00	25.67	7.00
MIR646HG	25.00	17.00	21.00	21.00	10.00
MIR647	53.00	70.00	73.00	65.33	69.00
MIR6505	5.00	3.00	2.00	3.33	4.00
MIR6516	3.00	1.00	0.00	1.33	0.00
MIR661	4.00	3.00	3.00	3.33	3.00
MIR6728	5.00	11.00	3.00	6.33	1.00

MIR6733	4.00	0.00	4.00	2.67	2.00
MIR6734	3.00	2.00	0.00	1.67	1.00
MIR6739	3.00	6.00	5.00	4.67	3.00
MIR6753	3.00	8.00	9.00	6.67	4.00
MIR6754	3.00	1.00	2.00	2.00	0.00
MIR6758	26.00	22.00	28.00	25.33	19.00
MIR6763	5.00	3.00	4.00	4.00	0.00
MIR6772	5.00	7.00	4.00	5.33	4.00
MIR6775	6.00	4.00	7.00	5.67	6.00
MIR6797	5.00	4.00	3.00	4.00	11.00
MIR6835	98.00	101.00	127.00	108.67	52.00
MIR6840	13.00	13.00	9.00	11.67	7.00
MIR6845	12.00	8.00	6.00	8.67	2.00
MIR6847	11.00	3.00	7.00	7.00	1.00
MIR6855	4.00	0.00	0.00	1.33	0.00
MIR6884	6.00	5.00	6.00	5.67	3.00
MIR6895	5.00	3.00	3.00	3.67	4.00
MIR7-1	38.00	48.00	44.00	43.33	58.00
MIR7106	4.00	1.00	2.00	2.33	5.00
MIR7111	12.00	6.00	14.00	10.67	18.00
MIR7161	9.00	5.00	7.00	7.00	7.00
MIR762HG	26.00	58.00	52.00	45.33	39.00
MIR7641-2	10.00	3.00	4.00	5.67	2.00
MIR765	3.00	0.00	2.00	1.67	0.00
MIR7851	5.00	1.00	2.00	2.67	2.00
MIR7856	4.00	8.00	4.00	5.33	7.00
MIR8058	6.00	6.00	6.00	6.00	4.00
MIR93	11.00	10.00	14.00	11.67	11.00
MIR940	5.00	8.00	17.00	10.00	14.00
MIR99AHG	133.00	123.00	163.00	139.67	103.00
MIRLET7BHG	346.00	334.00	459.00	379.67	272.00
MIRLET7D	52.00	76.00	58.00	62.00	56.00
MIRLET7F1	14.00	19.00	11.00	14.67	3.00
MIRLET7G	3.00	3.00	4.00	3.33	1.00
MIRLET7I	8.00	18.00	11.00	12.33	11.00
MIS12	1493.00	1474.00	1599.00	1522.00	1608.00
MIS18A	364.00	368.00	364.00	365.33	505.00
MIS18BP1	991.00	893.00	979.00	954.33	954.00
MITD1	565.00	531.00	575.00	557.00	688.00
MITF	179.00	207.00	190.00	192.00	153.00
MIXL1	4.00	0.00	1.00	1.67	3.00
MKI67	5085.00	4895.00	5014.00	4998.00	5001.00
MKKS	1156.00	1119.00	1189.00	1154.67	1430.00
MKL1	517.00	517.00	555.00	529.67	501.00
MKL2	655.00	569.00	618.00	614.00	548.00
MKLN1	2192.00	2003.00	2351.00	2182.00	2227.00
MKLN1-AS	88.00	85.00	101.00	91.33	86.00
MKNK1	687.00	613.00	686.00	662.00	661.00

MKNK2	1792.00	1788.00	2100.00	1893.33	1487.00
MKRN1	1896.00	1875.00	2064.00	1945.00	2087.00
MKRN2	993.00	942.00	1121.00	1018.67	1101.00
MKS1	897.00	852.00	888.00	879.00	875.00
MKX	126.00	101.00	126.00	117.67	126.00
MLEC	5927.00	5576.00	5878.00	5793.67	4562.00
MLF1	332.00	328.00	397.00	352.33	463.00
MLF2	2714.00	2640.00	2836.00	2730.00	3306.00
MLH1	879.00	778.00	835.00	830.67	1000.00
MLH3	792.00	692.00	846.00	776.67	792.00
MLKL	582.00	619.00	599.00	600.00	572.00
MLLT1	2408.00	2285.00	2477.00	2390.00	2604.00
MLLT10	1939.00	1765.00	2016.00	1906.67	2097.00
MLLT11	148.00	131.00	129.00	136.00	174.00
MLLT3	134.00	124.00	144.00	134.00	149.00
MLLT4	3700.00	3422.00	3907.00	3676.33	3361.00
MLLT4-AS1	21.00	22.00	23.00	22.00	31.00
MLLT6	6.00	4.00	9.00	6.33	2.00
MLPH	12379.00	12737.00	13886.00	13000.67	9807.00
MLST8	834.00	840.00	858.00	844.00	1191.00
MLX	1109.00	1120.00	1058.00	1095.67	1460.00
MLXIP	2391.00	2426.00	2717.00	2511.33	2049.00
MLYCD	255.00	205.00	239.00	233.00	244.00
MMAA	43.00	42.00	52.00	45.67	70.00
MMAB	265.00	249.00	258.00	257.33	317.00
MMACHC	263.00	228.00	279.00	256.67	254.00
MMADHC	1960.00	1798.00	2181.00	1979.67	2646.00
MMD	282.00	280.00	307.00	289.67	290.00
MME	7559.00	7011.00	7765.00	7445.00	10683.00
MMGT1	833.00	699.00	785.00	772.33	939.00
MMP1	930124.00	919814.00	938341.00	929426.33	940851.00
MMP10	12.00	17.00	5.00	11.33	15.00
MMP12	4.00	5.00	8.00	5.67	5.00
MMP13	4.00	5.00	5.00	4.67	5.00
MMP14	17728.00	18442.00	18289.00	18153.00	15792.00
MMP15	15.00	9.00	11.00	11.67	13.00
MMP16	416.00	393.00	459.00	422.67	329.00
MMP17	349.00	338.00	369.00	352.00	284.00
MMP19	207.00	184.00	228.00	206.33	172.00
MMP2	3870.00	3859.00	3737.00	3822.00	2108.00
MMP24-AS1	802.00	835.00	830.00	822.33	810.00
MMP25	31.00	27.00	39.00	32.33	22.00
MMP25-AS1	105.00	101.00	116.00	107.33	70.00
MMP3	4626.00	4479.00	4484.00	4529.67	2578.00
MMS19	3286.00	3089.00	3410.00	3261.67	3442.00
MMS22L	590.00	535.00	603.00	576.00	725.00
MN1	3.00	2.00	1.00	2.00	1.00
MNAT1	739.00	668.00	690.00	699.00	985.00

MND1	115.00	127.00	105.00	115.67	180.00
MNS1	96.00	95.00	68.00	86.33	122.00
MNT	1066.00	1040.00	1167.00	1091.00	1117.00
MOB1A	678.00	606.00	757.00	680.33	747.00
MOB1B	580.00	581.00	578.00	579.67	686.00
MOB2	351.00	385.00	382.00	372.67	441.00
MOB3A	1006.00	1018.00	1069.00	1031.00	858.00
MOB3B	9.00	10.00	10.00	9.67	17.00
MOB3C	216.00	274.00	274.00	254.67	250.00
MOB4	30.00	30.00	48.00	36.00	45.00
MOCOS	1733.00	1600.00	1705.00	1679.33	1178.00
MOCS1	568.00	565.00	554.00	562.33	577.00
MOCS2	1040.00	895.00	1014.00	983.00	1311.00
MOCS3	400.00	380.00	433.00	404.33	465.00
MOGAT1	3.00	0.00	1.00	1.33	0.00
MOGS	4099.00	4259.00	4265.00	4207.67	3607.00
MOK	20547.00	20739.00	22141.00	21142.33	30034.00
MON1A	488.00	418.00	499.00	468.33	464.00
MON1B	1085.00	1058.00	1019.00	1054.00	905.00
MON2	1147.00	1031.00	1117.00	1098.33	1083.00
MORC2	2226.00	2096.00	2328.00	2216.67	2183.00
MORC3	632.00	551.00	632.00	605.00	627.00
MORC4	562.00	525.00	581.00	556.00	725.00
MORF4L1	2288.00	2064.00	2274.00	2208.67	2706.00
MORF4L2	8697.00	8287.00	8805.00	8596.33	9501.00
MORF4L2-AS1	144.00	134.00	156.00	144.67	156.00
MORN1	166.00	166.00	136.00	156.00	140.00
MORN2	149.00	167.00	166.00	160.67	222.00
MORN3	5.00	5.00	3.00	4.33	3.00
MORN4	539.00	506.00	532.00	525.67	469.00
MOSPD1	333.00	301.00	341.00	325.00	381.00
MOSPD2	376.00	345.00	411.00	377.33	337.00
MOSPD3	263.00	316.00	270.00	283.00	327.00
MOV10	1430.00	1408.00	1480.00	1439.33	1300.00
MOXD1	1297.00	1287.00	1245.00	1276.33	1720.00
MOXD2P	12.00	2.00	11.00	8.33	0.00
MPC1	590.00	522.00	575.00	562.33	629.00
MPC2	409.00	383.00	424.00	405.33	435.00
MPDU1	1370.00	1415.00	1227.00	1337.33	1599.00
MPDZ	4080.00	3809.00	4483.00	4124.00	3343.00
MPG	1212.00	1203.00	1277.00	1230.67	1120.00
MPHOSPH10	933.00	927.00	1014.00	958.00	1053.00
MPHOSPH6	845.00	886.00	1004.00	911.67	1118.00
MPHOSPH8	1117.00	1036.00	1188.00	1113.67	1003.00
MPHOSPH9	518.00	498.00	630.00	548.67	499.00
MPI	571.00	560.00	568.00	566.33	605.00
MPL	5.00	2.00	3.00	3.33	9.00
MPLKIP	775.00	793.00	848.00	805.33	1031.00

MPND	115.00	119.00	106.00	113.33	129.00
MPP1	98.00	119.00	79.00	98.67	182.00
MPP2	51.00	27.00	45.00	41.00	46.00
MPP3	917.00	1036.00	1071.00	1008.00	871.00
MPP4	1328.00	1253.00	1312.00	1297.67	1327.00
MPP5	834.00	796.00	907.00	845.67	978.00
MPP6	957.00	886.00	1100.00	981.00	1210.00
MPP7	199.00	166.00	211.00	192.00	156.00
MPPE1	412.00	432.00	464.00	436.00	499.00
MPRIP	8648.00	8026.00	8720.00	8464.67	6645.00
MPST	568.00	594.00	622.00	594.67	645.00
MPV17	1489.00	1476.00	1510.00	1491.67	1308.00
MPV17L2	336.00	345.00	355.00	345.33	395.00
MPZ	51.00	35.00	32.00	39.33	14.00
MPZL1	2961.00	2877.00	2775.00	2871.00	2699.00
MR1	351.00	320.00	321.00	330.67	284.00
MRAP2	6.00	7.00	4.00	5.67	13.00
MRAS	60.00	85.00	80.00	75.00	79.00
MRC1	5.00	6.00	2.00	4.33	4.00
MRC2	684.00	663.00	609.00	652.00	444.00
MRE11A	1272.00	1133.00	1279.00	1228.00	1372.00
MREG	120.00	126.00	123.00	123.00	127.00
MRFAP1	2432.00	2401.00	2588.00	2473.67	3312.00
MRFAP1L1	753.00	703.00	755.00	737.00	900.00
MRGBP	940.00	1032.00	1041.00	1004.33	1268.00
MRI1	1289.00	1379.00	1524.00	1397.33	1392.00
MRM1	18.00	13.00	18.00	16.33	13.00
MROH1	1758.00	1762.00	1984.00	1834.67	1255.00
MROH8	5.00	5.00	9.00	6.33	11.00
MRPL1	398.00	384.00	430.00	404.00	516.00
MRPL10	1070.00	954.00	1075.00	1033.00	1264.00
MRPL11	1482.00	1527.00	1589.00	1532.67	1983.00
MRPL12	1636.00	1459.00	1707.00	1600.67	2324.00
MRPL13	996.00	907.00	990.00	964.33	1299.00
MRPL14	570.00	629.00	667.00	622.00	760.00
MRPL15	1384.00	1325.00	1417.00	1375.33	1793.00
MRPL16	683.00	639.00	697.00	673.00	882.00
MRPL17	1033.00	1009.00	1109.00	1050.33	1461.00
MRPL18	794.00	779.00	814.00	795.67	1024.00
MRPL19	1102.00	933.00	1130.00	1055.00	1358.00
MRPL2	713.00	687.00	767.00	722.33	955.00
MRPL20	755.00	757.00	823.00	778.33	1072.00
MRPL21	660.00	688.00	726.00	691.33	979.00
MRPL22	431.00	432.00	417.00	426.67	552.00
MRPL23	79.00	73.00	77.00	76.33	108.00
MRPL24	1949.00	2037.00	2080.00	2022.00	2461.00
MRPL27	629.00	636.00	644.00	636.33	768.00
MRPL28	806.00	875.00	886.00	855.67	1002.00

MRPL3	2907.00	2841.00	3108.00	2952.00	3907.00
MRPL30	1319.00	1183.00	1438.00	1313.33	1587.00
MRPL32	795.00	818.00	939.00	850.67	999.00
MRPL33	1179.00	1252.00	1171.00	1200.67	1598.00
MRPL34	566.00	585.00	711.00	620.67	900.00
MRPL35	845.00	748.00	855.00	816.00	972.00
MRPL36	514.00	514.00	581.00	536.33	689.00
MRPL37	1679.00	1587.00	1836.00	1700.67	2048.00
MRPL38	1006.00	1050.00	1108.00	1054.67	1273.00
MRPL39	547.00	439.00	519.00	501.67	658.00
MRPL4	1613.00	1508.00	1637.00	1586.00	1941.00
MRPL40	561.00	579.00	566.00	568.67	707.00
MRPL41	474.00	474.00	536.00	494.67	636.00
MRPL42	1074.00	955.00	1170.00	1066.33	1149.00
MRPL43	927.00	937.00	971.00	945.00	1071.00
MRPL44	591.00	531.00	613.00	578.33	717.00
MRPL45	120.00	89.00	121.00	110.00	154.00
MRPL45P2	63.00	56.00	59.00	59.33	64.00
MRPL46	436.00	429.00	422.00	429.00	516.00
MRPL47	927.00	827.00	904.00	886.00	1251.00
MRPL48	282.00	287.00	318.00	295.67	493.00
MRPL49	1244.00	1188.00	1336.00	1256.00	1320.00
MRPL50	419.00	350.00	401.00	390.00	557.00
MRPL51	1722.00	1795.00	1883.00	1800.00	2373.00
MRPL52	1115.00	1101.00	1175.00	1130.33	1534.00
MRPL53	358.00	388.00	360.00	368.67	550.00
MRPL54	312.00	312.00	315.00	313.00	482.00
MRPL55	345.00	321.00	351.00	339.00	393.00
MRPL57	540.00	593.00	602.00	578.33	710.00
MRPL9	1349.00	1271.00	1422.00	1347.33	1758.00
MRPS10	1443.00	1284.00	1517.00	1414.67	1661.00
MRPS11	506.00	466.00	551.00	507.67	490.00
MRPS12	555.00	632.00	706.00	631.00	736.00
MRPS14	502.00	453.00	500.00	485.00	500.00
MRPS15	779.00	750.00	864.00	797.67	1018.00
MRPS16	2776.00	2539.00	2888.00	2734.33	3211.00
MRPS17	345.00	335.00	366.00	348.67	459.00
MRPS18A	480.00	395.00	468.00	447.67	609.00
MRPS18C	228.00	225.00	196.00	216.33	296.00
MRPS2	1381.00	1408.00	1595.00	1461.33	1713.00
MRPS21	1408.00	1476.00	1588.00	1490.67	1914.00
MRPS22	1013.00	1023.00	1106.00	1047.33	1234.00
MRPS23	974.00	925.00	1086.00	995.00	1285.00
MRPS24	63.00	81.00	86.00	76.67	123.00
MRPS25	2786.00	2782.00	2886.00	2818.00	2738.00
MRPS26	797.00	835.00	843.00	825.00	1063.00
MRPS27	1809.00	1821.00	2045.00	1891.67	2018.00
MRPS28	475.00	473.00	512.00	486.67	653.00

MRPS30	797.00	725.00	808.00	776.67	1007.00
MRPS31	454.00	453.00	479.00	462.00	447.00
MRPS31P5	239.00	234.00	270.00	247.67	231.00
MRPS33	911.00	855.00	846.00	870.67	1103.00
MRPS34	1416.00	1396.00	1537.00	1449.67	1980.00
MRPS35	1253.00	1121.00	1437.00	1270.33	1665.00
MRPS36	117.00	105.00	89.00	103.67	138.00
MRPS5	1855.00	1765.00	1958.00	1859.33	2245.00
MRPS6	734.00	656.00	733.00	707.67	1047.00
MRPS7	722.00	721.00	816.00	753.00	1016.00
MRPS9	883.00	830.00	858.00	857.00	1223.00
MRRF	785.00	768.00	866.00	806.33	927.00
MRS2	886.00	852.00	972.00	903.33	1063.00
MRTO4	1303.00	1311.00	1390.00	1334.67	1772.00
MSANTD1	10.00	9.00	9.00	9.33	13.00
MSANTD2	840.00	801.00	930.00	857.00	801.00
MSANTD3	1105.00	1095.00	1234.00	1144.67	1372.00
MSANTD4	430.00	454.00	573.00	485.67	478.00
MSC	11.00	15.00	10.00	12.00	10.00
MSC-AS1	88.00	79.00	109.00	92.00	79.00
MSH2	1317.00	1230.00	1324.00	1290.33	1491.00
MSH3	451.00	403.00	471.00	441.67	416.00
MSH4	20.00	15.00	20.00	18.33	22.00
MSH6	1785.00	1700.00	1853.00	1779.33	1833.00
MSI2	372.00	344.00	387.00	367.67	323.00
MSL1	2025.00	1979.00	2151.00	2051.67	2116.00
MSL2	758.00	719.00	1012.00	829.67	972.00
MSL3	1360.00	1357.00	1457.00	1391.33	1296.00
MSL3P1	357.00	324.00	409.00	363.33	364.00
MSMO1	696.00	632.00	676.00	668.00	706.00
MSMP	4.00	2.00	2.00	2.67	2.00
MSN	24667.00	23073.00	24789.00	24176.33	22150.00
MSR1	31.00	27.00	27.00	28.33	36.00
MSRA	127.00	146.00	157.00	143.33	217.00
MSRB1	160.00	185.00	179.00	174.67	201.00
MSRB2	364.00	424.00	377.00	388.33	442.00
MSRB3	816.00	778.00	947.00	847.00	906.00
MSS51	326.00	349.00	364.00	346.33	383.00
MST1	173.00	162.00	168.00	167.67	168.00
MST1L	17.00	14.00	18.00	16.33	10.00
MST1P2	94.00	100.00	101.00	98.33	101.00
MST1R	4.00	1.00	1.00	2.00	3.00
MSTN	5.00	1.00	1.00	2.33	1.00
MSTO1	84.00	96.00	98.00	92.67	93.00
MSTO2P	101.00	121.00	118.00	113.33	132.00
MSX1	124.00	105.00	108.00	112.33	116.00
MT1E	2273.00	2165.00	2219.00	2219.00	3104.00
MT1F	3.00	0.00	1.00	1.33	0.00

MT1X	180.00	195.00	214.00	196.33	294.00
MT2A	6498.00	6391.00	6833.00	6574.00	5365.00
MTA1	2591.00	2613.00	2862.00	2688.67	2937.00
MTA2	2687.00	2765.00	2733.00	2728.33	3084.00
MTA3	682.00	607.00	715.00	668.00	799.00
MTAP	2997.00	2694.00	3092.00	2927.67	3504.00
MTBP	544.00	509.00	526.00	526.33	667.00
MTCH1	4997.00	5124.00	5316.00	5145.67	5443.00
MTCH2	1639.00	1544.00	1619.00	1600.67	2193.00
MTCL1	4150.00	4189.00	4608.00	4315.67	4185.00
MTCP1	138.00	146.00	140.00	141.33	167.00
MTDH	7320.00	6797.00	7434.00	7183.67	7636.00
MTERF1	349.00	365.00	363.00	359.00	406.00
MTERF2	226.00	262.00	247.00	245.00	293.00
MTERF3	884.00	910.00	975.00	923.00	1117.00
MTERF4	1093.00	1092.00	1204.00	1129.67	1117.00
MTF1	587.00	547.00	620.00	584.67	547.00
MTF2	654.00	557.00	618.00	609.67	821.00
MTFMT	238.00	287.00	332.00	285.67	337.00
MTFP1	396.00	377.00	357.00	376.67	525.00
MTFR1	676.00	639.00	699.00	671.33	907.00
MTFR1L	803.00	701.00	836.00	780.00	858.00
MTFR2	137.00	150.00	151.00	146.00	206.00
MTG1	1539.00	1554.00	1560.00	1551.00	1673.00
MTG2	1224.00	1364.00	1296.00	1294.67	1223.00
MTHFD1	2961.00	2760.00	2911.00	2877.33	3564.00
MTHFD1L	3109.00	2958.00	3319.00	3128.67	3608.00
MTHFD2	5442.00	4835.00	5582.00	5286.33	6915.00
MTHFD2L	129.00	107.00	118.00	118.00	169.00
MTHFR	219.00	210.00	249.00	226.00	218.00
MTHFS	27.00	32.00	38.00	32.33	56.00
MTHFSD	782.00	750.00	767.00	766.33	785.00
MTIF2	1813.00	1743.00	1859.00	1805.00	2298.00
MTIF3	375.00	405.00	440.00	406.67	513.00
MTL5	104.00	106.00	81.00	97.00	108.00
MTM1	179.00	182.00	204.00	188.33	171.00
MTMR1	1599.00	1459.00	1688.00	1582.00	1886.00
MTMR10	30.00	21.00	37.00	29.33	23.00
MTMR11	325.00	311.00	303.00	313.00	356.00
MTMR12	843.00	707.00	930.00	826.67	978.00
MTMR14	725.00	707.00	709.00	713.67	823.00
MTMR2	1839.00	1722.00	2067.00	1876.00	2171.00
MTMR3	1077.00	1055.00	1174.00	1102.00	987.00
MTMR4	1733.00	1603.00	1898.00	1744.67	1881.00
MTMR6	722.00	516.00	688.00	642.00	736.00
MTMR7	8.00	5.00	8.00	7.00	3.00
MTMR9	518.00	489.00	488.00	498.33	484.00
MTMR9LP	163.00	169.00	187.00	173.00	76.00

MTO1	933.00	960.00	1048.00	980.33	1125.00
MTOR	2533.00	2316.00	2489.00	2446.00	2263.00
MTPAP	841.00	855.00	929.00	875.00	1035.00
MTR	1232.00	1112.00	1220.00	1188.00	1228.00
MTRF1	554.00	574.00	609.00	579.00	622.00
MTRF1L	397.00	292.00	376.00	355.00	349.00
MTRNR2L2	12.00	10.00	17.00	13.00	26.00
MTRR	1387.00	1271.00	1490.00	1382.67	1254.00
MTSS1	128.00	131.00	131.00	130.00	126.00
MTSS1L	1212.00	1288.00	1418.00	1306.00	942.00
MTURN	665.00	595.00	702.00	654.00	609.00
MTUS1	30.00	20.00	29.00	26.33	11.00
MTX1	272.00	313.00	294.00	293.00	351.00
MTX2	946.00	942.00	999.00	962.33	1267.00
MTX3	1147.00	1086.00	1303.00	1178.67	1193.00
MUC1	91.00	57.00	81.00	76.33	65.00
MUL1	814.00	823.00	744.00	793.67	712.00
MUM1	1486.00	1407.00	1418.00	1437.00	1506.00
MUM1L1	84.00	70.00	104.00	86.00	104.00
MURC	50.00	59.00	76.00	61.67	81.00
MUS81	2049.00	2120.00	2069.00	2079.33	2454.00
MUSK	43.00	35.00	50.00	42.67	39.00
MUSTN1	29.00	35.00	46.00	36.67	31.00
MUT	1111.00	1037.00	1196.00	1114.67	1219.00
MUTYH	830.00	994.00	965.00	929.67	1142.00
MVB12A	644.00	625.00	640.00	636.33	745.00
MVB12B	330.00	287.00	321.00	312.67	314.00
MVD	756.00	743.00	797.00	765.33	831.00
MVK	156.00	158.00	151.00	155.00	160.00
MVP	1958.00	1939.00	1988.00	1961.67	1950.00
MX1	15.00	13.00	25.00	17.67	31.00
MXD1	563.00	564.00	630.00	585.67	648.00
MXD3	218.00	184.00	230.00	210.67	220.00
MXD4	436.00	441.00	482.00	453.00	398.00
MXI1	753.00	723.00	890.00	788.67	958.00
MXRA7	1905.00	1782.00	1905.00	1864.00	2040.00
MXRA8	6.00	1.00	0.00	2.33	2.00
MYADM	690.00	611.00	569.00	623.33	597.00
MYB	18.00	8.00	6.00	10.67	32.00
MYBBP1A	4758.00	4855.00	4901.00	4838.00	4560.00
MYBL1	917.00	826.00	919.00	887.33	1130.00
MYBL2	375.00	393.00	300.00	356.00	625.00
MYC	6156.00	6092.00	6415.00	6221.00	7550.00
MYCBP	7.00	5.00	7.00	6.33	10.00
MYCBP2	2921.00	2753.00	3187.00	2953.67	2433.00
MYCBPAP	7.00	5.00	10.00	7.33	7.00
MYCT1	238.00	231.00	193.00	220.67	241.00
MYD88	368.00	354.00	377.00	366.33	383.00

MYDGF	2620.00	2701.00	2325.00	2548.67	2893.00
MYEF2	1564.00	1452.00	1665.00	1560.33	1804.00
MYEOV	3460.00	3335.00	3610.00	3468.33	2255.00
MYEOV2	380.00	356.00	374.00	370.00	496.00
MYH10	1296.00	1153.00	1235.00	1228.00	1163.00
MYH15	56.00	53.00	55.00	54.67	118.00
MYH16	4.00	3.00	6.00	4.33	6.00
MYH3	133.00	163.00	152.00	149.33	132.00
MYH7B	10.00	6.00	5.00	7.00	9.00
MYH9	22354.00	20953.00	22210.00	21839.00	13558.00
MYL12A	5513.00	5386.00	6077.00	5658.67	7669.00
MYL12B	5146.00	4899.00	5268.00	5104.33	6939.00
MYL5	59.00	69.00	73.00	67.00	71.00
MYL6	3569.00	3604.00	3598.00	3590.33	4463.00
MYL6B	1361.00	1359.00	1463.00	1394.33	1513.00
MYLIP	137.00	121.00	157.00	138.33	115.00
MYLK	4120.00	3894.00	4144.00	4052.67	3190.00
MYLK2	7.00	8.00	5.00	6.67	9.00
MYLK4	60.00	48.00	62.00	56.67	41.00
MYLK-AS1	33.00	43.00	36.00	37.33	42.00
MYNN	916.00	866.00	928.00	903.33	1041.00
MYO10	8150.00	7747.00	9185.00	8360.67	7683.00
MYO15A	6.00	2.00	6.00	4.67	6.00
MYO15B	22.00	34.00	38.00	31.33	25.00
MYO18A	712.00	671.00	768.00	717.00	580.00
MYO19	4.00	14.00	23.00	13.67	10.00
MYO1A	7.00	1.00	2.00	3.33	0.00
MYO1B	3019.00	2861.00	3305.00	3061.67	3091.00
MYO1C	2508.00	2445.00	2726.00	2559.67	2430.00
MYO1D	221.00	222.00	210.00	217.67	180.00
MYO1E	1048.00	964.00	1115.00	1042.33	878.00
MYO1F	6.00	3.00	3.00	4.00	4.00
MYO5A	2133.00	1992.00	2362.00	2162.33	1521.00
MYO5B	35.00	20.00	27.00	27.33	19.00
MYO5C	4.00	5.00	1.00	3.33	5.00
MYO6	1546.00	1378.00	1654.00	1526.00	1398.00
MYO7A	31.00	28.00	25.00	28.00	21.00
MYO9A	466.00	396.00	490.00	450.67	299.00
MYO9B	3773.00	3870.00	3978.00	3873.67	3649.00
MYOF	39067.00	34732.00	41595.00	38464.67	39619.00
MYOM1	28.00	45.00	45.00	39.33	23.00
MYOZ3	8.00	1.00	9.00	6.00	3.00
MYPN	643.00	653.00	782.00	692.67	414.00
MYPOP	151.00	162.00	175.00	162.67	194.00
MYRF	207.00	221.00	205.00	211.00	50.00
MYRFL	7.00	11.00	15.00	11.00	21.00
MYRIP	81.00	82.00	101.00	88.00	92.00
MYSM1	3009.00	2853.00	3299.00	3053.67	3066.00

MZB1	4.00	1.00	4.00	3.00	0.00
MZF1	853.00	835.00	991.00	893.00	808.00
MZF1-AS1	123.00	117.00	105.00	115.00	119.00
MZT1	477.00	444.00	520.00	480.33	672.00
MZT2A	461.00	463.00	452.00	458.67	596.00
MZT2B	1164.00	1130.00	1127.00	1140.33	1446.00
N4BP1	872.00	806.00	906.00	861.33	982.00
N4BP2	725.00	653.00	758.00	712.00	627.00
N4BP2L1	30.00	18.00	31.00	26.33	24.00
N4BP2L2	1848.00	1655.00	1999.00	1834.00	1959.00
N4BP2L2-IT2	267.00	291.00	224.00	260.67	220.00
N6AMT1	306.00	302.00	295.00	301.00	278.00
N6AMT2	106.00	112.00	149.00	122.33	142.00
NAA10	1494.00	1569.00	1680.00	1581.00	2160.00
NAA15	1539.00	1332.00	1507.00	1459.33	1616.00
NAA16	484.00	456.00	560.00	500.00	463.00
NAA20	1681.00	1719.00	1722.00	1707.33	2171.00
NAA25	1881.00	1713.00	1927.00	1840.33	1833.00
NAA30	502.00	421.00	567.00	496.67	547.00
NAA35	637.00	524.00	597.00	586.00	620.00
NAA38	768.00	828.00	800.00	798.67	1119.00
NAA40	1337.00	1273.00	1297.00	1302.33	1361.00
NAA50	3588.00	3191.00	3890.00	3556.33	3857.00
NAA60	571.00	584.00	579.00	578.00	621.00
NAAA	179.00	191.00	159.00	176.33	160.00
NAALAD2	4.00	2.00	7.00	4.33	0.00
NAALADL2	34.00	27.00	32.00	31.00	37.00
NAB1	1230.00	1179.00	1316.00	1241.67	1517.00
NAB2	680.00	655.00	738.00	691.00	1240.00
NABP1	2638.00	2524.00	2853.00	2671.67	2139.00
NABP2	678.00	633.00	736.00	682.33	798.00
NACA	10932.00	10813.00	11957.00	11234.00	14993.00
NACC1	3043.00	2870.00	3130.00	3014.33	2889.00
NACC2	1065.00	1021.00	1069.00	1051.67	934.00
NADK	1119.00	1105.00	1257.00	1160.33	1162.00
NADK2	766.00	754.00	938.00	819.33	1007.00
NADSYN1	8120.00	8594.00	8946.00	8553.33	7591.00
NAE1	1777.00	1601.00	1867.00	1748.33	2195.00
NAF1	304.00	229.00	278.00	270.33	296.00
NAGA	882.00	848.00	926.00	885.33	786.00
NAGK	525.00	532.00	543.00	533.33	513.00
NAGLU	1345.00	1427.00	1383.00	1385.00	1481.00
NAGPA	1009.00	1008.00	981.00	999.33	1001.00
NAGPA-AS1	8.00	2.00	10.00	6.67	1.00
NAGS	89.00	78.00	72.00	79.67	69.00
NAIF1	289.00	288.00	337.00	304.67	286.00
NAIP	9.00	6.00	8.00	7.67	13.00
NALCN	586.00	562.00	695.00	614.33	466.00

NAMPT	4547.00	4128.00	4804.00	4493.00	4679.00
NANOS1	4.00	3.00	0.00	2.33	0.00
NANP	374.00	360.00	436.00	390.00	387.00
NANS	649.00	600.00	698.00	649.00	763.00
NAP1L1	15044.00	14114.00	16531.00	15229.67	18121.00
NAP1L2	33.00	27.00	22.00	27.33	41.00
NAP1L4	68.00	54.00	70.00	64.00	75.00
NAP1L5	192.00	196.00	214.00	200.67	257.00
NAP1L6	9.00	3.00	11.00	7.67	8.00
NAPA	898.00	844.00	995.00	912.33	984.00
NAPA-AS1	10.00	8.00	13.00	10.33	21.00
NAPB	547.00	511.00	582.00	546.67	516.00
NAPEPLD	14.00	4.00	10.00	9.33	6.00
NAPG	1311.00	1219.00	1377.00	1302.33	1410.00
NAPSA	12.00	15.00	9.00	12.00	11.00
NARF	739.00	714.00	803.00	752.00	808.00
NARFL	575.00	531.00	633.00	579.67	670.00
NARS	7349.00	6802.00	7423.00	7191.33	8000.00
NARS2	540.00	442.00	528.00	503.33	522.00
NASP	2867.00	2761.00	2935.00	2854.33	3694.00
NAT1	42.00	38.00	49.00	43.00	56.00
NAT10	2467.00	2437.00	2609.00	2504.33	2437.00
NAT14	556.00	671.00	578.00	601.67	634.00
NAT16	4.00	1.00	0.00	1.67	0.00
NAT6	204.00	180.00	230.00	204.67	221.00
NAT8L	1592.00	1667.00	1732.00	1663.67	1665.00
NAT9	1974.00	1983.00	2006.00	1987.67	2126.00
NATD1	18.00	19.00	19.00	18.67	20.00
NAV1	4550.00	4184.00	4600.00	4444.67	2676.00
NAV2	4538.00	4361.00	4901.00	4600.00	2973.00
NAV3	3579.00	3170.00	3717.00	3488.67	3818.00
NBAS	1506.00	1494.00	1627.00	1542.33	1523.00
NBEA	465.00	394.00	465.00	441.33	354.00
NBEAL1	89.00	76.00	96.00	87.00	84.00
NBEAL2	2958.00	2993.00	3461.00	3137.33	2860.00
NBL1	17.00	11.00	13.00	13.67	11.00
NBN	1671.00	1589.00	1785.00	1681.67	2056.00
NBPF1	441.00	337.00	462.00	413.33	304.00
NBPF10	98.00	56.00	75.00	76.33	60.00
NBPF11	375.00	353.00	380.00	369.33	338.00
NBPF12	495.00	479.00	531.00	501.67	433.00
NBPF14	435.00	385.00	416.00	412.00	350.00
NBPF15	2029.00	2057.00	2206.00	2097.33	2204.00
NBPF20	206.00	184.00	218.00	202.67	236.00
NBPF25P	32.00	29.00	37.00	32.67	18.00
NBPF3	306.00	314.00	318.00	312.67	287.00
NBPF6	3.00	2.00	1.00	2.00	0.00
NBPF7	3.00	1.00	7.00	3.67	0.00

NBPF8	1358.00	1372.00	1459.00	1396.33	1561.00
NBPF9	1065.00	964.00	1090.00	1039.67	1197.00
NBR1	3554.00	3140.00	3639.00	3444.33	3111.00
NBR2	280.00	273.00	325.00	292.67	238.00
NCAM1	69.00	46.00	54.00	56.33	71.00
NCAM2	275.00	267.00	353.00	298.33	445.00
NCAPD2	5116.00	4704.00	4931.00	4917.00	5115.00
NCAPD3	1049.00	975.00	1067.00	1030.33	942.00
NCAPG	782.00	687.00	763.00	744.00	1097.00
NCAPG2	1831.00	1708.00	1721.00	1753.33	2160.00
NCAPH	616.00	551.00	587.00	584.67	781.00
NCAPH2	671.00	719.00	796.00	728.67	914.00
NCBP1	1728.00	1596.00	1873.00	1732.33	1800.00
NCBP2	2959.00	2720.00	3126.00	2935.00	3770.00
NCBP2-AS2	332.00	319.00	364.00	338.33	424.00
NCDN	1807.00	1778.00	1857.00	1814.00	1720.00
NCEH1	2164.00	2038.00	2217.00	2139.67	3477.00
NCF2	10.00	26.00	9.00	15.00	11.00
NCK1	642.00	628.00	756.00	675.33	711.00
NCK1-AS1	13.00	20.00	21.00	18.00	32.00
NCK2	1022.00	992.00	1108.00	1040.67	931.00
NCKAP1	4904.00	4204.00	5387.00	4831.67	5081.00
NCKAP1L	5.00	2.00	6.00	4.33	6.00
NCKAP5	77.00	69.00	86.00	77.33	76.00
NCKAP5L	1604.00	1603.00	1577.00	1594.67	1305.00
NCKIPSD	760.00	720.00	782.00	754.00	666.00
NCL	21252.00	20768.00	22910.00	21643.33	25812.00
NCLN	3986.00	4076.00	4001.00	4021.00	4424.00
NCOA1	450.00	389.00	471.00	436.67	369.00
NCOA2	538.00	428.00	647.00	537.67	400.00
NCOA3	1422.00	1285.00	1457.00	1388.00	1117.00
NCOA4	3457.00	3061.00	3716.00	3411.33	3487.00
NCOA5	1485.00	1485.00	1638.00	1536.00	1581.00
NCOA6	1681.00	1569.00	1867.00	1705.67	1372.00
NCOA7	828.00	694.00	885.00	802.33	667.00
NCOR1	2463.00	2173.00	2457.00	2364.33	2066.00
NCOR1P1	4.00	6.00	4.00	4.67	4.00
NCOR2	8887.00	8612.00	9297.00	8932.00	7215.00
NCR3LG1	710.00	671.00	757.00	712.67	485.00
NCRNA00250	3.00	5.00	0.00	2.67	4.00
NCS1	2266.00	2230.00	2284.00	2260.00	2178.00
NCSTN	6451.00	6216.00	6367.00	6344.67	5504.00
NDC1	2790.00	2560.00	2637.00	2662.33	3286.00
NDC80	812.00	847.00	860.00	839.67	1226.00
NDEL1	1302.00	1327.00	1382.00	1337.00	1320.00
NDFIP1	1891.00	1664.00	1794.00	1783.00	2204.00
NDFIP2	1113.00	901.00	1115.00	1043.00	1031.00
NDNF	87.00	92.00	82.00	87.00	63.00

NDOR1	2932.00	2924.00	3065.00	2973.67	2710.00
NDP	8.00	6.00	6.00	6.67	2.00
NDRG1	464.00	469.00	443.00	458.67	403.00
NDRG3	932.00	1016.00	1069.00	1005.67	1057.00
NDRG4	18.00	15.00	8.00	13.67	15.00
NDST1	4886.00	4539.00	4719.00	4714.67	3136.00
NDST2	1036.00	1043.00	1145.00	1074.67	864.00
NDST3	5.00	11.00	4.00	6.67	0.00
NDUFA1	537.00	600.00	563.00	566.67	734.00
NDUFA10	1379.00	1418.00	1502.00	1433.00	1635.00
NDUFA11	1009.00	1040.00	1083.00	1044.00	1234.00
NDUFA12	557.00	509.00	500.00	522.00	735.00
NDUFA13	1356.00	1445.00	1533.00	1444.67	1918.00
NDUFA2	380.00	365.00	461.00	402.00	506.00
NDUFA4	1846.00	1738.00	1763.00	1782.33	2473.00
NDUFA4L2	61.00	57.00	61.00	59.67	58.00
NDUFA5	739.00	699.00	764.00	734.00	918.00
NDUFA7	615.00	556.00	645.00	605.33	978.00
NDUFA8	569.00	581.00	610.00	586.67	812.00
NDUFA9	1521.00	1418.00	1546.00	1495.00	1894.00
NDUFAB1	1053.00	1000.00	1079.00	1044.00	1512.00
NDUFAF1	298.00	311.00	280.00	296.33	344.00
NDUFAF2	542.00	521.00	532.00	531.67	745.00
NDUFAF3	339.00	358.00	368.00	355.00	466.00
NDUFAF4	656.00	629.00	696.00	660.33	821.00
NDUFAF5	243.00	213.00	232.00	229.33	320.00
NDUFAF6	359.00	356.00	399.00	371.33	406.00
NDUFAF7	627.00	670.00	642.00	646.33	767.00
NDUFB1	323.00	343.00	304.00	323.33	488.00
NDUFB10	1129.00	1063.00	1113.00	1101.67	1340.00
NDUFB11	849.00	804.00	795.00	816.00	1086.00
NDUFB2	1107.00	1071.00	1119.00	1099.00	1605.00
NDUFB2-AS1	33.00	46.00	33.00	37.33	40.00
NDUFB3	593.00	608.00	611.00	604.00	789.00
NDUFB4	1234.00	1209.00	1289.00	1244.00	1647.00
NDUFB5	932.00	853.00	943.00	909.33	1308.00
NDUFB6	747.00	743.00	720.00	736.67	1077.00
NDUFB7	902.00	965.00	955.00	940.67	1240.00
NDUFB8	1744.00	1699.00	1777.00	1740.00	2367.00
NDUFB9	3596.00	3826.00	3723.00	3715.00	4417.00
NDUFC1	299.00	304.00	300.00	301.00	475.00
NDUFC2	277.00	271.00	280.00	276.00	331.00
NDUFS1	1581.00	1433.00	1635.00	1549.67	1891.00
NDUFS2	3235.00	3122.00	3303.00	3220.00	3992.00
NDUFS3	820.00	844.00	829.00	831.00	1118.00
NDUFS4	694.00	672.00	747.00	704.33	947.00
NDUFS5	1429.00	1321.00	1479.00	1409.67	2107.00
NDUFS6	824.00	777.00	873.00	824.67	1199.00

NDUFS7	721.00	847.00	875.00	814.33	1107.00
NDUFS8	824.00	874.00	910.00	869.33	1041.00
NDUFV1	2785.00	2825.00	2859.00	2823.00	3296.00
NDUFV2	1798.00	1751.00	1908.00	1819.00	2724.00
NDUFV2-AS1	12.00	18.00	18.00	16.00	9.00
NDUFV3	591.00	622.00	649.00	620.67	659.00
NEAT1	19490.00	19969.00	19585.00	19681.33	18060.00
NEB	10.00	12.00	12.00	11.33	14.00
NEBL	4.00	4.00	5.00	4.33	4.00
NECAB2	38.00	19.00	22.00	26.33	44.00
NECAB3	556.00	581.00	604.00	580.33	642.00
NECAP1	811.00	860.00	876.00	849.00	1013.00
NECAP2	1033.00	945.00	1037.00	1005.00	1147.00
NEDD1	661.00	613.00	725.00	666.33	804.00
NEDD4	2646.00	2342.00	2664.00	2550.67	2785.00
NEDD4L	2070.00	1889.00	2137.00	2032.00	1793.00
NEDD8	942.00	949.00	968.00	953.00	1284.00
NEDD9	3.00	1.00	3.00	2.33	3.00
NEFL	257.00	230.00	297.00	261.33	327.00
NEGR1	227.00	196.00	257.00	226.67	190.00
NEIL1	46.00	61.00	59.00	55.33	69.00
NEIL2	424.00	419.00	394.00	412.33	552.00
NEIL3	107.00	82.00	106.00	98.33	149.00
NEK1	407.00	371.00	434.00	404.00	355.00
NEK10	171.00	171.00	196.00	179.33	146.00
NEK11	101.00	107.00	90.00	99.33	103.00
NEK2	505.00	436.00	478.00	473.00	593.00
NEK3	388.00	363.00	393.00	381.33	554.00
NEK4	479.00	525.00	583.00	529.00	538.00
NEK5	17.00	14.00	16.00	15.67	20.00
NEK6	1748.00	1634.00	1758.00	1713.33	1375.00
NEK7	1014.00	875.00	1122.00	1003.67	1182.00
NEK8	118.00	126.00	133.00	125.67	85.00
NEK9	4558.00	4188.00	4710.00	4485.33	3880.00
NELFA	1433.00	1509.00	1546.00	1496.00	1684.00
NELFB	1520.00	1590.00	1713.00	1607.67	1816.00
NELFCD	2521.00	2489.00	2615.00	2541.67	2796.00
NELL2	101.00	134.00	131.00	122.00	113.00
NEMF	1682.00	1670.00	1854.00	1735.33	1835.00
NEMP1	2174.00	2093.00	2188.00	2151.67	2425.00
NEMP2	372.00	364.00	316.00	350.67	572.00
NENF	564.00	647.00	625.00	612.00	869.00
NEO1	624.00	648.00	709.00	660.33	495.00
NES	93.00	97.00	103.00	97.67	110.00
NET1	1790.00	1827.00	1864.00	1827.00	2509.00
NETO2	2195.00	2010.00	2399.00	2201.33	1974.00
NEU3	380.00	347.00	412.00	379.67	296.00
NEURL2	244.00	223.00	231.00	232.67	199.00

NEURL4	2409.00	2309.00	2446.00	2388.00	2331.00
NEUROG2	15.00	8.00	7.00	10.00	3.00
NEXN	983.00	995.00	1062.00	1013.33	1028.00
NEXN-AS1	73.00	72.00	71.00	72.00	97.00
NF1	2324.00	2022.00	2586.00	2310.67	2074.00
NF2	3023.00	2883.00	3125.00	3010.33	3918.00
NFAT5	2879.00	2692.00	3153.00	2908.00	2376.00
NFATC2	28.00	18.00	29.00	25.00	20.00
NFATC2IP	2276.00	2399.00	2411.00	2362.00	2404.00
NFATC3	635.00	560.00	682.00	625.67	492.00
NFATC4	844.00	921.00	927.00	897.33	918.00
NFE2L1	11525.00	11532.00	11692.00	11583.00	10206.00
NFE2L2	2521.00	2362.00	2749.00	2544.00	2765.00
NFE2L3	1804.00	1868.00	1863.00	1845.00	3350.00
NFIA	215.00	224.00	269.00	236.00	328.00
NFIB	1807.00	1664.00	2021.00	1830.67	1893.00
NFIC	1455.00	1476.00	1650.00	1527.00	1311.00
NFIL3	475.00	464.00	502.00	480.33	477.00
NFIX	503.00	455.00	515.00	491.00	486.00
NFKB1	1057.00	1055.00	1141.00	1084.33	1242.00
NFKB2	2004.00	1934.00	1997.00	1978.33	2857.00
NFKBIA	570.00	483.00	594.00	549.00	776.00
NFKBIB	458.00	494.00	507.00	486.33	688.00
NFKBID	111.00	131.00	121.00	121.00	111.00
NFKBIE	214.00	245.00	294.00	251.00	281.00
NFKBIL1	28.00	35.00	30.00	31.00	25.00
NFKBIZ	1442.00	1347.00	1527.00	1438.67	1305.00
NFRKB	785.00	741.00	757.00	761.00	660.00
NFS1	887.00	807.00	897.00	863.67	943.00
NFU1	363.00	333.00	438.00	378.00	530.00
NFX1	1782.00	1743.00	1878.00	1801.00	1813.00
NFXL1	704.00	644.00	700.00	682.67	673.00
NFYA	2164.00	2156.00	2468.00	2262.67	2034.00
NFYB	398.00	364.00	444.00	402.00	515.00
NFYC	1011.00	931.00	1029.00	990.33	1179.00
NFYC-AS1	327.00	306.00	315.00	316.00	300.00
NGDN	641.00	653.00	666.00	653.33	831.00
NGF	182.00	204.00	189.00	191.67	88.00
NGFR	590.00	581.00	584.00	585.00	816.00
NGFRAP1	3424.00	3396.00	3830.00	3550.00	4831.00
NGLY1	861.00	875.00	1044.00	926.67	971.00
NGRN	1653.00	1548.00	1748.00	1649.67	2105.00
NHEJ1	290.00	295.00	331.00	305.33	330.00
NHLH1	5.00	5.00	6.00	5.33	5.00
NHLH2	3.00	2.00	12.00	5.67	5.00
NHLRC1	11.00	13.00	10.00	11.33	22.00
NHLRC2	413.00	365.00	411.00	396.33	367.00
NHLRC3	745.00	733.00	784.00	754.00	766.00

NHLRC4	8.00	11.00	16.00	11.67	14.00
NHP2	1554.00	1574.00	1788.00	1638.67	2086.00
NHS	176.00	182.00	234.00	197.33	78.00
NHSL1	439.00	403.00	467.00	436.33	493.00
NHSL2	11.00	9.00	9.00	9.67	4.00
NICN1	773.00	821.00	849.00	814.33	662.00
NID1	197.00	195.00	205.00	199.00	233.00
NID2	7.00	4.00	12.00	7.67	7.00
NIF3L1	905.00	919.00	1080.00	968.00	1097.00
NIFK	1584.00	1410.00	1689.00	1561.00	1994.00
NIFK-AS1	82.00	65.00	65.00	70.67	100.00
NIM1K	23.00	20.00	34.00	25.67	18.00
NIN	1536.00	1460.00	1711.00	1569.00	1595.00
NINJ1	377.00	368.00	378.00	374.33	415.00
NINL	904.00	977.00	1127.00	1002.67	926.00
NIP7	1227.00	1219.00	1253.00	1233.00	1637.00
NIPA1	1096.00	1010.00	1158.00	1088.00	1255.00
NIPA2	1912.00	1759.00	2021.00	1897.33	2293.00
NIPAL1	248.00	218.00	275.00	247.00	164.00
NIPAL2	148.00	131.00	118.00	132.33	120.00
NIPAL3	697.00	613.00	670.00	660.00	550.00
NIPAL4	128.00	103.00	107.00	112.67	79.00
NIPBL	2208.00	2137.00	2338.00	2227.67	2024.00
NIPBL-AS1	460.00	428.00	469.00	452.33	398.00
NIPSNAP1	1215.00	1179.00	1246.00	1213.33	1749.00
NIPSNAP3A	238.00	192.00	244.00	224.67	226.00
NIPSNAP3B	44.00	64.00	64.00	57.33	53.00
NISCH	3388.00	3433.00	3616.00	3479.00	2896.00
NIT1	782.00	709.00	768.00	753.00	846.00
NIT2	1093.00	1054.00	1158.00	1101.67	1436.00
NKAP	376.00	340.00	363.00	359.67	420.00
NKILA	14.00	13.00	12.00	13.00	7.00
NKIRAS1	410.00	394.00	423.00	409.00	500.00
NKIRAS2	703.00	680.00	744.00	709.00	668.00
NKRF	466.00	432.00	457.00	451.67	507.00
NKTR	4611.00	4457.00	4884.00	4650.67	4158.00
NKX2-1	16.00	28.00	30.00	24.67	18.00
NKX2-4	93.00	76.00	67.00	78.67	108.00
NKX2-6	132.00	146.00	167.00	148.33	191.00
NKX2-8	12.00	21.00	21.00	18.00	20.00
NKX3-1	109.00	129.00	136.00	124.67	105.00
NKX3-2	20.00	19.00	28.00	22.33	29.00
NKX6-1	106.00	129.00	135.00	123.33	103.00
NLE1	888.00	778.00	851.00	839.00	994.00
NLGN1	80.00	57.00	67.00	68.00	80.00
NLGN2	3028.00	3236.00	3435.00	3233.00	2991.00
NLGN3	10.00	11.00	9.00	10.00	10.00
NLK	775.00	730.00	844.00	783.00	585.00

NLN	1222.00	1145.00	1315.00	1227.33	1428.00
NLRC5	420.00	405.00	478.00	434.33	344.00
NLRP1	2772.00	2816.00	3026.00	2871.33	2984.00
NLRP12	3.00	4.00	3.00	3.33	2.00
NLRP14	7.00	2.00	4.00	4.33	2.00
NLRP3	730.00	662.00	648.00	680.00	1037.00
NLRX1	414.00	407.00	400.00	407.00	452.00
NMB	197.00	183.00	196.00	192.00	257.00
NMBR	4.00	2.00	1.00	2.33	2.00
NMD3	2157.00	1860.00	2156.00	2057.67	2503.00
NME1	1600.00	1421.00	1610.00	1543.67	2270.00
NME2	511.00	522.00	569.00	534.00	675.00
NME3	571.00	630.00	580.00	593.67	709.00
NME4	1446.00	1629.00	1624.00	1566.33	2062.00
NME6	431.00	386.00	459.00	425.33	461.00
NME7	556.00	542.00	569.00	555.67	625.00
NMI	228.00	210.00	258.00	232.00	273.00
NMNAT1	363.00	328.00	351.00	347.33	409.00
NMNAT2	1703.00	1672.00	1720.00	1698.33	2584.00
NMRK1	477.00	461.00	484.00	474.00	549.00
NMT1	2748.00	2495.00	2844.00	2695.67	2836.00
NMT2	3871.00	3523.00	4115.00	3836.33	2825.00
NMU	7.00	11.00	3.00	7.00	24.00
NNMT	111.00	130.00	120.00	120.33	150.00
NNT	1660.00	1438.00	1645.00	1581.00	1404.00
NNT-AS1	443.00	399.00	570.00	470.67	394.00
NOA1	541.00	486.00	588.00	538.33	590.00
NOB1	1803.00	1755.00	1975.00	1844.33	2166.00
NOC2L	3882.00	3790.00	3936.00	3869.33	4281.00
NOC3L	2490.00	2274.00	2493.00	2419.00	2785.00
NOC4L	602.00	558.00	644.00	601.33	707.00
NOCT	118.00	80.00	137.00	111.67	110.00
NOD1	257.00	233.00	280.00	256.67	313.00
NOG	15.00	23.00	23.00	20.33	18.00
NOL10	998.00	941.00	1095.00	1011.33	1304.00
NOL11	1547.00	1506.00	1671.00	1574.67	1841.00
NOL12	731.00	763.00	758.00	750.67	814.00
NOL3	870.00	842.00	885.00	865.67	804.00
NOL4	7.00	12.00	20.00	13.00	4.00
NOL4L	1920.00	1903.00	2113.00	1978.67	1927.00
NOL6	3483.00	3488.00	3590.00	3520.33	3791.00
NOL7	1403.00	1272.00	1311.00	1328.67	1827.00
NOL8	1584.00	1535.00	1677.00	1598.67	1661.00
NOL9	845.00	762.00	668.00	758.33	955.00
NOLC1	5978.00	5488.00	6210.00	5892.00	6743.00
NOM1	1386.00	1265.00	1441.00	1364.00	1263.00
NOMO1	178.00	202.00	222.00	200.67	186.00
NOMO2	733.00	622.00	684.00	679.67	529.00

NONO	11133.00	10453.00	11239.00	10941.67	11917.00
NOP10	1254.00	1239.00	1320.00	1271.00	2058.00
NOP14	5605.00	5427.00	6029.00	5687.00	5957.00
NOP14-AS1	269.00	291.00	297.00	285.67	338.00
NOP16	1267.00	1221.00	1343.00	1277.00	1628.00
NOP2	3307.00	3171.00	3654.00	3377.33	3551.00
NOP56	7464.00	7561.00	7751.00	7592.00	9016.00
NOP58	3873.00	3722.00	3936.00	3843.67	4868.00
NOP9	2659.00	2326.00	2776.00	2587.00	1465.00
NOS1AP	20.00	15.00	14.00	16.33	11.00
NOS3	185.00	185.00	170.00	180.00	166.00
NOSIP	1229.00	1133.00	1184.00	1182.00	1565.00
NOTCH1	1836.00	1911.00	2065.00	1937.33	1445.00
NOTCH2	14010.00	12484.00	14420.00	13638.00	10099.00
NOTCH2NL	160.00	139.00	114.00	137.67	129.00
NOTCH3	6.00	1.00	4.00	3.67	7.00
NOV	324.00	295.00	296.00	305.00	195.00
NOVA1	3.00	6.00	2.00	3.67	3.00
NOX3	3.00	0.00	0.00	1.00	0.00
NOX4	132.00	136.00	152.00	140.00	172.00
NOX5	26.00	14.00	25.00	21.67	18.00
NOXA1	82.00	75.00	92.00	83.00	95.00
NOXO1	4.00	4.00	2.00	3.33	4.00
NOXRED1	20.00	22.00	18.00	20.00	25.00
NPAS1	10.00	16.00	21.00	15.67	10.00
NPAS2	6783.00	6582.00	6988.00	6784.33	8315.00
NPAS3	102.00	73.00	95.00	90.00	95.00
NPAT	373.00	342.00	428.00	381.00	348.00
NPC1	4834.00	4729.00	5185.00	4916.00	4188.00
NPC1L1	3.00	4.00	4.00	3.67	4.00
NPC2	734.00	745.00	650.00	709.67	602.00
NPDC1	3834.00	3865.00	4076.00	3925.00	4058.00
NPEPL1	28.00	36.00	42.00	35.33	42.00
NPEPPS	2060.00	1962.00	2149.00	2057.00	2223.00
NPFF	63.00	83.00	59.00	68.33	86.00
NPFFR2	12.00	18.00	19.00	16.33	48.00
NPHP1	174.00	172.00	162.00	169.33	157.00
NPHP3	394.00	356.00	439.00	396.33	371.00
NPHP3-ACAD11	210.00	172.00	261.00	214.33	248.00
NPHP4	704.00	682.00	631.00	672.33	549.00
NPIPA1	26.00	31.00	18.00	25.00	41.00
NPIPB11	88.00	89.00	107.00	94.67	75.00
NPIPB15	56.00	71.00	75.00	67.33	66.00
NPIPB3	96.00	99.00	116.00	103.67	88.00
NPIPB4	188.00	169.00	160.00	172.33	150.00
NPIPB5	371.00	445.00	460.00	425.33	475.00
NPIPB6	7.00	6.00	4.00	5.67	2.00
NPIPB8	14.00	21.00	17.00	17.33	19.00

NPIPB9	15.00	18.00	18.00	17.00	6.00
NPL	164.00	162.00	167.00	164.33	197.00
NPLOC4	3951.00	3860.00	4128.00	3979.67	3663.00
NPM1	22549.00	21879.00	24628.00	23018.67	31981.00
NPM3	1099.00	1080.00	1226.00	1135.00	1579.00
NPPA-AS1	83.00	67.00	89.00	79.67	109.00
NPR2	622.00	602.00	675.00	633.00	572.00
NPR3	302.00	260.00	332.00	298.00	285.00
NPRL2	625.00	577.00	674.00	625.33	688.00
NPRL3	1346.00	1255.00	1343.00	1314.67	1673.00
NPTN	1939.00	1718.00	1701.00	1786.00	1861.00
NPTN-IT1	164.00	146.00	145.00	151.67	91.00
NPTXR	16.00	17.00	23.00	18.67	5.00
NPW	78.00	87.00	89.00	84.67	99.00
NPY1R	94.00	101.00	107.00	100.67	113.00
NPY5R	4.00	4.00	7.00	5.00	10.00
NQO1	2622.00	2559.00	2492.00	2557.67	2363.00
NQO2	410.00	393.00	358.00	387.00	467.00
NR1D1	242.00	273.00	243.00	252.67	315.00
NR1D2	1244.00	1172.00	1527.00	1314.33	1550.00
NR1H2	971.00	921.00	1000.00	964.00	1124.00
NR1H3	197.00	144.00	188.00	176.33	218.00
NR1I3	3.00	0.00	0.00	1.00	0.00
NR2C1	818.00	828.00	977.00	874.33	724.00
NR2C2	1785.00	1610.00	1876.00	1757.00	1606.00
NR2C2AP	512.00	547.00	525.00	528.00	672.00
NR2E3	18.00	20.00	12.00	16.67	6.00
NR2F1	738.00	680.00	813.00	743.67	993.00
NR2F1-AS1	480.00	409.00	551.00	480.00	561.00
NR2F2	3226.00	3011.00	3605.00	3280.67	3424.00
NR2F2-AS1	215.00	199.00	227.00	213.67	124.00
NR2F6	828.00	845.00	972.00	881.67	901.00
NR3C1	1313.00	1175.00	1463.00	1317.00	1298.00
NR3C2	287.00	238.00	287.00	270.67	244.00
NR4A1	229.00	223.00	221.00	224.33	192.00
NR4A2	18.00	21.00	21.00	20.00	18.00
NR4A3	11.00	5.00	13.00	9.67	9.00
NR5A2	3.00	9.00	9.00	7.00	13.00
NR6A1	200.00	188.00	210.00	199.33	171.00
NRARP	33.00	33.00	55.00	40.33	53.00
NRAS	1827.00	1582.00	1876.00	1761.67	2336.00
NRAV	197.00	183.00	180.00	186.67	169.00
NRBF2	536.00	494.00	524.00	518.00	537.00
NRBP1	2177.00	2029.00	2316.00	2174.00	2316.00
NRBP2	1148.00	1219.00	1350.00	1239.00	1167.00
NRD1	5749.00	5627.00	6175.00	5850.33	6412.00
NRDE2	359.00	378.00	353.00	363.33	354.00
NREP	377.00	370.00	418.00	388.33	510.00

NRF1	474.00	481.00	495.00	483.33	476.00
NRG1	1974.00	1677.00	1867.00	1839.33	1662.00
NRG2	89.00	82.00	85.00	85.33	51.00
NRGN	177.00	136.00	149.00	154.00	185.00
NRIP1	2110.00	1895.00	2228.00	2077.67	1607.00
NRIP3	958.00	932.00	982.00	957.33	1587.00
NRK	1066.00	930.00	992.00	996.00	797.00
NRL	8.00	9.00	5.00	7.33	12.00
NRN1	4.00	2.00	2.00	2.67	1.00
NRN1L	9.00	12.00	4.00	8.33	5.00
NRP1	13376.00	12787.00	14264.00	13475.67	8341.00
NRP2	2533.00	2474.00	2588.00	2531.67	2785.00
NRROS	475.00	535.00	555.00	521.67	515.00
NRSN2	1236.00	1222.00	1180.00	1212.67	1216.00
NRSN2-AS1	149.00	124.00	135.00	136.00	105.00
NRXN1	6.00	2.00	1.00	3.00	4.00
NRXN3	142.00	117.00	153.00	137.33	86.00
NSA2	1284.00	1246.00	1286.00	1272.00	1775.00
NSD1	2181.00	2016.00	2292.00	2163.00	1726.00
NSDHL	510.00	504.00	535.00	516.33	657.00
NSF	29.00	33.00	28.00	30.00	40.00
NSFL1C	1381.00	1398.00	1465.00	1414.67	1580.00
NSFP1	5.00	5.00	8.00	6.00	4.00
NSL1	469.00	464.00	461.00	464.67	587.00
NSMAF	2436.00	2335.00	2610.00	2460.33	2725.00
NSMCE1	781.00	779.00	819.00	793.00	964.00
NSMCE2	468.00	469.00	511.00	482.67	542.00
NSMCE4A	838.00	870.00	910.00	872.67	1100.00
NSMF	2379.00	2399.00	2639.00	2472.33	2203.00
NSRP1	1046.00	1077.00	1152.00	1091.67	1395.00
NSUN2	3349.00	3234.00	3679.00	3420.67	3650.00
NSUN3	128.00	90.00	129.00	115.67	161.00
NSUN4	914.00	861.00	945.00	906.67	957.00
NSUN5	413.00	472.00	457.00	447.33	579.00
NSUN5P1	804.00	884.00	927.00	871.67	908.00
NSUN5P2	651.00	737.00	788.00	725.33	767.00
NSUN6	643.00	622.00	666.00	643.67	641.00
NSUN7	8.00	4.00	2.00	4.67	3.00
NT5C	726.00	758.00	777.00	753.67	991.00
NT5C2	2400.00	2385.00	2608.00	2464.33	2310.00
NT5C3A	813.00	841.00	825.00	826.33	1067.00
NT5C3B	838.00	863.00	877.00	859.33	1099.00
NT5DC1	560.00	516.00	617.00	564.33	639.00
NT5DC2	2685.00	2667.00	2680.00	2677.33	2586.00
NT5DC3	358.00	409.00	455.00	407.33	522.00
NT5E	27695.00	26190.00	28421.00	27435.33	25067.00
NT5M	97.00	106.00	105.00	102.67	135.00
NTAN1	12.00	12.00	30.00	18.00	11.00

NTHL1	455.00	502.00	469.00	475.33	582.00
NTM	3713.00	3679.00	3973.00	3788.33	3777.00
NTMT1	1086.00	1064.00	1043.00	1064.33	1229.00
NTN4	2148.00	1929.00	2075.00	2050.67	942.00
NTNG1	3064.00	2929.00	3103.00	3032.00	2389.00
NTNG2	29.00	37.00	36.00	34.00	8.00
NTPCR	253.00	236.00	260.00	249.67	329.00
NTSR1	6.00	6.00	9.00	7.00	9.00
NUAK1	207.00	179.00	202.00	196.00	200.00
NUAK2	268.00	236.00	267.00	257.00	223.00
NUB1	1439.00	1231.00	1454.00	1374.67	1216.00
NUBP1	299.00	326.00	341.00	322.00	351.00
NUBP2	724.00	759.00	854.00	779.00	915.00
NUBPL	417.00	400.00	458.00	425.00	502.00
NUCB1	3677.00	3894.00	3499.00	3690.00	3371.00
NUCB2	1382.00	1367.00	1311.00	1353.33	1867.00
NUCKS1	3979.00	3692.00	4107.00	3926.00	5001.00
NUDC	4681.00	4892.00	5114.00	4895.67	6394.00
NUDCD1	1293.00	1184.00	1355.00	1277.33	1670.00
NUDCD2	571.00	555.00	589.00	571.67	707.00
NUDCD3	2648.00	2595.00	2834.00	2692.33	2998.00
NUDT1	313.00	259.00	312.00	294.67	440.00
NUDT10	8.00	5.00	3.00	5.33	4.00
NUDT11	293.00	268.00	313.00	291.33	375.00
NUDT12	394.00	400.00	391.00	395.00	464.00
NUDT13	266.00	284.00	270.00	273.33	366.00
NUDT14	22.00	27.00	25.00	24.67	26.00
NUDT15	412.00	383.00	417.00	404.00	570.00
NUDT16	2001.00	1949.00	2044.00	1998.00	1823.00
NUDT16L1	323.00	299.00	358.00	326.67	358.00
NUDT17	135.00	150.00	117.00	134.00	142.00
NUDT18	35.00	44.00	44.00	41.00	68.00
NUDT19	494.00	403.00	492.00	463.00	613.00
NUDT2	573.00	608.00	585.00	588.67	807.00
NUDT21	2884.00	2357.00	2858.00	2699.67	3289.00
NUDT22	1405.00	1445.00	1553.00	1467.67	1636.00
NUDT3	62.00	102.00	94.00	86.00	121.00
NUDT4	536.00	529.00	606.00	557.00	612.00
NUDT5	3045.00	2895.00	3046.00	2995.33	3626.00
NUDT6	95.00	102.00	115.00	104.00	103.00
NUDT8	15.00	21.00	26.00	20.67	21.00
NUDT9	497.00	484.00	511.00	497.33	530.00
NUDT9P1	27.00	20.00	21.00	22.67	21.00
NUF2	706.00	768.00	725.00	733.00	1187.00
NUFIP1	273.00	293.00	276.00	280.67	376.00
NUFIP2	4108.00	3743.00	4754.00	4201.67	3671.00
NUMA1	12363.00	12206.00	13076.00	12548.33	10065.00
NUMB	2146.00	1997.00	2161.00	2101.33	2012.00

NUMBL	517.00	521.00	542.00	526.67	607.00
NUP107	2458.00	2436.00	2533.00	2475.67	2778.00
NUP133	1440.00	1260.00	1448.00	1382.67	1628.00
NUP153	2912.00	2648.00	3118.00	2892.67	2992.00
NUP155	1356.00	1157.00	1302.00	1271.67	1469.00
NUP160	1398.00	1181.00	1348.00	1309.00	1419.00
NUP188	3250.00	3001.00	3151.00	3134.00	3094.00
NUP205	4095.00	3784.00	4276.00	4051.67	4535.00
NUP214	2111.00	2033.00	2304.00	2149.33	1721.00
NUP35	497.00	555.00	555.00	535.67	689.00
NUP37	981.00	871.00	976.00	942.67	1140.00
NUP43	1001.00	890.00	984.00	958.33	1235.00
NUP50	1352.00	1329.00	1562.00	1414.33	1657.00
NUP50-AS1	221.00	219.00	264.00	234.67	323.00
NUP54	700.00	670.00	718.00	696.00	833.00
NUP62	1589.00	1623.00	1686.00	1632.67	1845.00
NUP62CL	13.00	8.00	13.00	11.33	21.00
NUP85	1086.00	1133.00	1069.00	1096.00	1249.00
NUP88	1733.00	1546.00	1794.00	1691.00	2052.00
NUP93	1953.00	1902.00	1980.00	1945.00	2396.00
NUP98	3752.00	3627.00	4029.00	3802.67	3625.00
NUPL1	1317.00	1160.00	1335.00	1270.67	1463.00
NUPL2	967.00	830.00	1007.00	934.67	980.00
NUPR1	450.00	491.00	444.00	461.67	317.00
NUS1	1081.00	1023.00	1099.00	1067.67	1288.00
NUSAP1	1062.00	976.00	1034.00	1024.00	1518.00
NUTF2	1429.00	1373.00	1529.00	1443.67	1963.00
NUTM2A	12.00	11.00	21.00	14.67	19.00
NUTM2A-AS1	349.00	300.00	337.00	328.67	384.00
NUTM2B	9.00	8.00	4.00	7.00	13.00
NUTM2B-AS1	358.00	399.00	467.00	408.00	454.00
NUTM2D	26.00	24.00	19.00	23.00	25.00
NVL	773.00	752.00	764.00	763.00	919.00
NXF1	2003.00	2078.00	2176.00	2085.67	2032.00
NXF3	27.00	20.00	13.00	20.00	16.00
NXN	792.00	770.00	878.00	813.33	932.00
NXNL2	12.00	15.00	15.00	14.00	13.00
NXPE3	911.00	786.00	955.00	884.00	678.00
NXPH2	37.00	27.00	19.00	27.67	42.00
NXPH4	58.00	68.00	58.00	61.33	75.00
NXT1	502.00	490.00	552.00	514.67	628.00
NXT2	123.00	94.00	131.00	116.00	130.00
NYAP1	34.00	59.00	29.00	40.67	32.00
OAF	2183.00	2197.00	2035.00	2138.33	1842.00
OARD1	579.00	599.00	592.00	590.00	734.00
OAS1	3.00	3.00	2.00	2.67	7.00
OAS2	6.00	1.00	2.00	3.00	8.00
OAS3	61.00	78.00	62.00	67.00	94.00

OAT	2113.00	1875.00	2109.00	2032.33	2073.00
OAZ1	8270.00	8255.00	8833.00	8452.67	10465.00
OAZ2	852.00	855.00	914.00	873.67	1219.00
OAZ3	22.00	13.00	14.00	16.33	32.00
OBFC1	484.00	578.00	575.00	545.67	588.00
OBSCN	219.00	224.00	223.00	222.00	132.00
OBSL1	1763.00	1945.00	1948.00	1885.33	2024.00
OCA2	3.00	2.00	2.00	2.33	2.00
OCEL1	93.00	67.00	63.00	74.33	91.00
OCIAD1	3966.00	3785.00	3855.00	3868.67	4581.00
OCIAD2	1816.00	1872.00	1891.00	1859.67	2637.00
OCLM	22.00	16.00	19.00	19.00	22.00
OCLN	18.00	5.00	20.00	14.33	25.00
OCRL	738.00	648.00	770.00	718.67	724.00
ODC1	8620.00	8005.00	9092.00	8572.33	9640.00
ODF2	3229.00	3368.00	3518.00	3371.67	3529.00
ODF2L	1674.00	1651.00	1784.00	1703.00	1833.00
ODF3B	8.00	5.00	5.00	6.00	3.00
OFD1	1841.00	1790.00	1998.00	1876.33	1755.00
OGDH	2421.00	2363.00	2458.00	2414.00	2528.00
OGDHL	55.00	52.00	44.00	50.33	82.00
OGFOD1	1521.00	1578.00	1745.00	1614.67	1782.00
OGFOD2	746.00	819.00	865.00	810.00	779.00
OGFOD3	1138.00	1198.00	1135.00	1157.00	1142.00
OGFR	1053.00	1201.00	1129.00	1127.67	1192.00
OGFRL1	531.00	451.00	524.00	502.00	466.00
OGFRP1	4.00	7.00	7.00	6.00	10.00
OGG1	352.00	340.00	351.00	347.67	355.00
OGT	29893.00	29625.00	32479.00	30665.67	30951.00
OIP5	143.00	122.00	132.00	132.33	166.00
OIP5-AS1	259.00	250.00	319.00	276.00	274.00
OLA1	2556.00	2445.00	2779.00	2593.33	3312.00
OLAH	23.00	29.00	31.00	27.67	25.00
OLFML2B	16.00	17.00	3.00	12.00	35.00
OLFML3	937.00	908.00	888.00	911.00	918.00
OLIG2	10.00	7.00	12.00	9.67	14.00
OLMALINC	208.00	234.00	278.00	240.00	268.00
OMA1	776.00	815.00	933.00	841.33	923.00
ONECUT2	125.00	112.00	125.00	120.67	143.00
OPA1	1981.00	1943.00	2205.00	2043.00	2095.00
OPA3	447.00	430.00	508.00	461.67	470.00
OPCML	5.00	5.00	5.00	5.00	26.00
OPHN1	323.00	285.00	317.00	308.33	264.00
OPLAH	3.00	3.00	5.00	3.67	9.00
OPN3	795.00	678.00	791.00	754.67	597.00
OPRL1	3.00	9.00	9.00	7.00	3.00
OPTN	751.00	725.00	798.00	758.00	916.00
OR1F1	9.00	2.00	2.00	4.33	2.00

OR2T33	3.00	0.00	1.00	1.33	2.00
OR2T8	17.00	10.00	4.00	10.33	6.00
OR2W3	40.00	33.00	36.00	36.33	35.00
OR51E2	29.00	31.00	29.00	29.67	37.00
OR51M1	37.00	35.00	23.00	31.67	14.00
OR6F1	19.00	17.00	24.00	20.00	20.00
ORAI1	714.00	634.00	651.00	666.33	745.00
ORAI2	1485.00	1442.00	1500.00	1475.67	1105.00
ORAI3	403.00	412.00	400.00	405.00	336.00
ORAOV1	4063.00	4102.00	4344.00	4169.67	4248.00
ORC1	158.00	131.00	114.00	134.33	217.00
ORC2	1055.00	1065.00	1188.00	1102.67	1230.00
ORC3	898.00	898.00	978.00	924.67	1183.00
ORC4	638.00	699.00	738.00	691.67	792.00
ORC5	844.00	840.00	829.00	837.67	1127.00
ORC6	944.00	1013.00	876.00	944.33	1285.00
ORMDL1	2230.00	2126.00	2321.00	2225.67	2277.00
ORMDL2	243.00	231.00	221.00	231.67	270.00
ORMDL3	863.00	909.00	875.00	882.33	887.00
OS9	7123.00	7201.00	7271.00	7198.33	6500.00
OSBP	1711.00	1628.00	1779.00	1706.00	1663.00
OSBP2	125.00	126.00	151.00	134.00	146.00
OSBPL10	383.00	382.00	377.00	380.67	418.00
OSBPL11	443.00	407.00	465.00	438.33	516.00
OSBPL1A	865.00	778.00	896.00	846.33	978.00
OSBPL2	902.00	926.00	926.00	918.00	902.00
OSBPL3	1334.00	1291.00	1452.00	1359.00	1370.00
OSBPL5	371.00	428.00	427.00	408.67	424.00
OSBPL6	818.00	774.00	902.00	831.33	1126.00
OSBPL7	666.00	634.00	758.00	686.00	503.00
OSBPL8	1170.00	1007.00	1360.00	1179.00	1077.00
OSBPL9	1708.00	1716.00	1841.00	1755.00	1780.00
OSCAR	76.00	80.00	94.00	83.33	47.00
OSCP1	56.00	69.00	82.00	69.00	80.00
OSER1	1072.00	1033.00	1170.00	1091.67	1175.00
OSER1-AS1	54.00	41.00	44.00	46.33	57.00
OSGEP	1094.00	1023.00	1083.00	1066.67	1054.00
OSGEPL1	470.00	434.00	468.00	457.33	504.00
OSGEPL1-AS1	15.00	8.00	14.00	12.33	18.00
OSGIN1	198.00	206.00	239.00	214.33	213.00
OSGIN2	668.00	594.00	721.00	661.00	754.00
OSMR	7943.00	7474.00	8317.00	7911.33	6130.00
OSMR-AS1	9.00	19.00	11.00	13.00	13.00
OSR2	31.00	33.00	55.00	39.67	48.00
OST4	1112.00	1088.00	1146.00	1115.33	1557.00
OSTC	1252.00	1170.00	1274.00	1232.00	1476.00
OSTF1	347.00	380.00	350.00	359.00	584.00
OSTM1	1150.00	1101.00	1238.00	1163.00	1323.00

OTOGL	11.00	17.00	22.00	16.67	7.00
OTOS	3.00	1.00	0.00	1.33	0.00
OTUB1	2618.00	2551.00	2546.00	2571.67	2936.00
OTUD1	161.00	132.00	198.00	163.67	177.00
OTUD3	858.00	829.00	937.00	874.67	788.00
OTUD4	1563.00	1523.00	1858.00	1648.00	1559.00
OTUD5	1216.00	1226.00	1326.00	1256.00	1324.00
OTUD6B	800.00	681.00	857.00	779.33	878.00
OTUD6B-AS1	663.00	542.00	669.00	624.67	735.00
OTUD7B	717.00	661.00	706.00	694.67	653.00
OTULIN	682.00	583.00	713.00	659.33	586.00
OTX1	52.00	62.00	54.00	56.00	64.00
OVAAL	342.00	362.00	367.00	357.00	378.00
OVCA2	60.00	63.00	56.00	59.67	69.00
OVGP1	44.00	81.00	66.00	63.67	40.00
OXA1L	2311.00	2282.00	2501.00	2364.67	2763.00
OXCT1	1659.00	1636.00	1814.00	1703.00	2190.00
OXCT1-AS1	11.00	3.00	5.00	6.33	10.00
OXCT2	7.00	4.00	0.00	3.67	3.00
OXGR1	7.00	5.00	2.00	4.67	3.00
OXLD1	1341.00	1327.00	1453.00	1373.67	1528.00
OXNAD1	361.00	370.00	435.00	388.67	422.00
OXR1	663.00	597.00	764.00	674.67	801.00
OXSM	560.00	529.00	596.00	561.67	580.00
OXSR1	1680.00	1605.00	1769.00	1684.67	1595.00
OXTR	459.00	479.00	468.00	468.67	424.00
P2RX4	270.00	298.00	299.00	289.00	230.00
P2RX5	500.00	501.00	463.00	488.00	543.00
P2RX5-TAX1BP3	28.00	41.00	37.00	35.33	35.00
P2RX6	13.00	5.00	15.00	11.00	14.00
P2RX7	4.00	7.00	7.00	6.00	7.00
P2RY1	161.00	128.00	133.00	140.67	97.00
P2RY11	119.00	141.00	145.00	135.00	119.00
P2RY2	8.00	4.00	9.00	7.00	5.00
P2RY4	6.00	7.00	15.00	9.33	2.00
P3H1	2837.00	2844.00	2739.00	2806.67	2563.00
P3H2	1558.00	1502.00	1496.00	1518.67	1501.00
P3H3	3942.00	4202.00	4041.00	4061.67	3884.00
P3H4	1138.00	1283.00	1213.00	1211.33	1276.00
P4HA1	2675.00	2559.00	2625.00	2619.67	2826.00
P4HA2	899.00	909.00	891.00	899.67	890.00
P4HA3	35.00	38.00	35.00	36.00	55.00
P4HB	23486.00	23451.00	22398.00	23111.67	21999.00
P4HTM	338.00	348.00	353.00	346.33	330.00
PA2G4	5483.00	5116.00	5581.00	5393.33	6988.00
PA2G4P4	155.00	125.00	129.00	136.33	162.00
PAAF1	474.00	493.00	489.00	485.33	543.00
PABPC1	37071.00	35224.00	40570.00	37621.67	43031.00

PABPC1L	4559.00	4578.00	4836.00	4657.67	4771.00
PABPC3	169.00	123.00	143.00	145.00	170.00
PABPC4	8543.00	8265.00	9046.00	8618.00	9453.00
PABPN1	829.00	928.00	1073.00	943.33	1035.00
PACERR	38.00	36.00	36.00	36.67	24.00
PACRG	4.00	3.00	2.00	3.00	5.00
PACRGL	291.00	268.00	328.00	295.67	316.00
PACS1	2281.00	2212.00	2376.00	2289.67	1804.00
PACS2	1463.00	1366.00	1610.00	1479.67	1321.00
PACSIN1	28.00	20.00	40.00	29.33	29.00
PACSIN2	1166.00	1053.00	1197.00	1138.67	1140.00
PACSIN3	530.00	627.00	571.00	576.00	610.00
PADI1	4.00	2.00	5.00	3.67	2.00
PAF1	827.00	828.00	894.00	849.67	894.00
PAFAH1B1	4246.00	3910.00	4504.00	4220.00	4460.00
PAFAH1B2	1211.00	1002.00	1135.00	1116.00	1395.00
PAFAH1B3	298.00	285.00	308.00	297.00	437.00
PAFAH2	398.00	373.00	425.00	398.67	400.00
PAG1	817.00	724.00	862.00	801.00	496.00
PAGE1	3.00	0.00	3.00	2.00	12.00
PAGR1	505.00	521.00	539.00	521.67	570.00
PAICS	3745.00	3493.00	3977.00	3738.33	4767.00
PAIP1	1563.00	1443.00	1743.00	1583.00	1959.00
PAIP2	930.00	910.00	1104.00	981.33	1269.00
PAIP2B	73.00	85.00	107.00	88.33	82.00
PAK1	1013.00	950.00	1057.00	1006.67	1178.00
PAK1IP1	1187.00	1156.00	1273.00	1205.33	1505.00
PAK2	2496.00	2053.00	2436.00	2328.33	2420.00
PAK3	145.00	158.00	156.00	153.00	127.00
PAK4	594.00	602.00	636.00	610.67	763.00
PAK6	3.00	2.00	2.00	2.33	2.00
PALB2	569.00	555.00	584.00	569.33	618.00
PALLD	269.00	300.00	328.00	299.00	420.00
PALM2	75.00	77.00	87.00	79.67	71.00
PALM2-AKAP2	14.00	5.00	5.00	8.00	4.00
PAM	4612.00	4606.00	4652.00	4623.33	3668.00
PAM16	3.00	2.00	1.00	2.00	0.00
PAMR1	2810.00	2798.00	2950.00	2852.67	1800.00
PAN2	3000.00	3022.00	3404.00	3142.00	2561.00
PAN3	1068.00	1033.00	1179.00	1093.33	1111.00
PAN3-AS1	68.00	66.00	79.00	71.00	64.00
PANK1	251.00	238.00	262.00	250.33	329.00
PANK2	595.00	572.00	652.00	606.33	717.00
PANK3	369.00	292.00	332.00	331.00	223.00
PANO1	17.00	27.00	21.00	21.67	17.00
PANX1	501.00	469.00	490.00	486.67	500.00
PANX2	191.00	209.00	195.00	198.33	151.00
PAOX	28.00	30.00	39.00	32.33	41.00

PAPD4	831.00	760.00	860.00	817.00	854.00
PAPD5	505.00	415.00	587.00	502.33	486.00
PAPD7	1372.00	1390.00	1474.00	1412.00	1330.00
PAPLN	277.00	255.00	293.00	275.00	366.00
PAPOLA	3587.00	3138.00	3797.00	3507.33	4030.00
PAPOLG	456.00	441.00	495.00	464.00	490.00
PAPPA	262.00	251.00	298.00	270.33	124.00
PAPPA2	2120.00	1754.00	2108.00	1994.00	1047.00
PAPSS1	721.00	675.00	820.00	738.67	802.00
PAPSS2	695.00	649.00	728.00	690.67	759.00
PAQR3	774.00	624.00	767.00	721.67	685.00
PAQR4	371.00	396.00	392.00	386.33	572.00
PAQR5	48.00	45.00	47.00	46.67	41.00
PAQR6	285.00	252.00	323.00	286.67	237.00
PAQR7	922.00	1008.00	928.00	952.67	1149.00
PAQR8	939.00	919.00	878.00	912.00	651.00
PARD3	2075.00	2067.00	2283.00	2141.67	2030.00
PARD3-AS1	20.00	11.00	22.00	17.67	16.00
PARD3B	104.00	116.00	120.00	113.33	102.00
PARD6A	25.00	31.00	20.00	25.33	25.00
PARD6B	76.00	56.00	64.00	65.33	91.00
PARD6G	6.00	2.00	3.00	3.67	5.00
PARD6G-AS1	5.00	9.00	5.00	6.33	6.00
PARG	658.00	672.00	683.00	671.00	730.00
PARGP1	176.00	146.00	183.00	168.33	95.00
PARK2	34.00	36.00	40.00	36.67	44.00
PARK7	2855.00	2821.00	2978.00	2884.67	3813.00
PARL	728.00	732.00	680.00	713.33	869.00
PARM1	4.00	4.00	4.00	4.00	1.00
PARN	6.00	6.00	7.00	6.33	1.00
PARP1	2814.00	2690.00	2929.00	2811.00	3476.00
PARP10	731.00	871.00	901.00	834.33	827.00
PARP11	227.00	210.00	214.00	217.00	224.00
PARP12	362.00	308.00	337.00	335.67	381.00
PARP14	834.00	847.00	1015.00	898.67	845.00
PARP16	219.00	181.00	201.00	200.33	258.00
PARP2	778.00	680.00	845.00	767.67	861.00
PARP3	1083.00	1150.00	1268.00	1167.00	1120.00
PARP4	2771.00	2492.00	2794.00	2685.67	2146.00
PARP6	1679.00	1737.00	1753.00	1723.00	1631.00
PARP8	563.00	553.00	614.00	576.67	531.00
PARP9	291.00	270.00	287.00	282.67	240.00
PARPBP	395.00	363.00	402.00	386.67	484.00
PARS2	187.00	200.00	207.00	198.00	198.00
PART1	4.00	3.00	4.00	3.67	5.00
PARTICL	61.00	55.00	47.00	54.33	67.00
PARVA	2222.00	2095.00	2504.00	2273.67	2011.00
PARVB	857.00	842.00	941.00	880.00	1138.00

PASK	415.00	379.00	459.00	417.67	467.00
PATE2	9.00	8.00	7.00	8.00	7.00
PATL1	1147.00	1170.00	1266.00	1194.33	1195.00
PATL2	21.00	31.00	33.00	28.33	38.00
PATZ1	1080.00	1106.00	1215.00	1133.67	1177.00
PAWR	393.00	393.00	476.00	420.67	349.00
PAX3	614.00	544.00	604.00	587.33	573.00
PAX8	24.00	28.00	24.00	25.33	21.00
PAX8-AS1	11.00	4.00	17.00	10.67	10.00
PAX9	134.00	136.00	131.00	133.67	150.00
PAXBP1	1734.00	1759.00	1926.00	1806.33	1819.00
PAXBP1-AS1	32.00	33.00	33.00	32.67	26.00
PAXIP1	734.00	773.00	774.00	760.33	818.00
PAXIP1-AS1	319.00	268.00	329.00	305.33	322.00
PAXIP1-AS2	124.00	132.00	134.00	130.00	117.00
PBDC1	485.00	497.00	569.00	517.00	615.00
PBK	335.00	322.00	329.00	328.67	545.00
PBLD	111.00	126.00	135.00	124.00	152.00
PBRM1	1557.00	1458.00	1734.00	1583.00	1385.00
PBX2	19.00	22.00	25.00	22.00	17.00
PBX3	273.00	289.00	360.00	307.33	587.00
PBXIP1	1193.00	1236.00	1242.00	1223.67	1011.00
PC	835.00	873.00	865.00	857.67	926.00
PCAT6	11.00	18.00	8.00	12.33	9.00
PCBD1	976.00	894.00	943.00	937.67	1427.00
PCBD2	295.00	277.00	280.00	284.00	321.00
PCBP1	4871.00	4687.00	4669.00	4742.33	6029.00
PCBP1-AS1	237.00	268.00	273.00	259.33	261.00
PCBP2	10352.00	9988.00	11012.00	10450.67	12642.00
PCBP2-OT1	19.00	6.00	8.00	11.00	8.00
PCBP3	12.00	11.00	16.00	13.00	6.00
PCBP4	637.00	666.00	705.00	669.33	697.00
PCCA	435.00	372.00	426.00	411.00	350.00
PCCB	1005.00	972.00	1050.00	1009.00	1135.00
PCDH1	3.00	2.00	0.00	1.67	2.00
PCDH10	50.00	48.00	47.00	48.33	37.00
PCDH15	257.00	208.00	259.00	241.33	268.00
PCDH18	12.00	17.00	12.00	13.67	18.00
PCDH20	5.00	6.00	6.00	5.67	8.00
PCDH7	36.00	28.00	34.00	32.67	14.00
PCDH9	60.00	70.00	54.00	61.33	43.00
PCDHA4	3.00	5.00	3.00	3.67	3.00
PCDHA6	11.00	5.00	6.00	7.33	1.00
PCDHB10	12.00	14.00	17.00	14.33	15.00
PCDHB11	3.00	5.00	9.00	5.67	7.00
PCDHB12	10.00	19.00	13.00	14.00	18.00
PCDHB13	30.00	41.00	18.00	29.67	35.00
PCDHB14	69.00	56.00	50.00	58.33	63.00

PCDHB15	60.00	80.00	69.00	69.67	58.00
PCDHB16	41.00	45.00	39.00	41.67	38.00
PCDHB18P	3.00	1.00	4.00	2.67	0.00
PCDHB19P	4.00	6.00	2.00	4.00	3.00
PCDHB2	55.00	55.00	48.00	52.67	49.00
PCDHB3	3.00	3.00	1.00	2.33	5.00
PCDHB4	3.00	8.00	8.00	6.33	8.00
PCDHB5	46.00	42.00	38.00	42.00	59.00
PCDHB6	34.00	34.00	31.00	33.00	49.00
PCDHB7	11.00	11.00	11.00	11.00	16.00
PCDHB8	12.00	13.00	11.00	12.00	13.00
PCDHB9	15.00	11.00	7.00	11.00	10.00
PCDHGA1	30.00	33.00	36.00	33.00	17.00
PCDHGA10	96.00	121.00	110.00	109.00	42.00
PCDHGA11	314.00	347.00	333.00	331.33	173.00
PCDHGA12	11.00	3.00	8.00	7.33	4.00
PCDHGA2	26.00	25.00	20.00	23.67	25.00
PCDHGA3	13.00	7.00	16.00	12.00	7.00
PCDHGA4	20.00	17.00	21.00	19.33	9.00
PCDHGA5	11.00	13.00	17.00	13.67	11.00
PCDHGA6	27.00	30.00	31.00	29.33	32.00
PCDHGA7	11.00	13.00	12.00	12.00	9.00
PCDHGA9	18.00	6.00	17.00	13.67	4.00
PCDHGB1	54.00	57.00	64.00	58.33	36.00
PCDHGB2	24.00	28.00	24.00	25.33	9.00
PCDHGB3	15.00	18.00	24.00	19.00	4.00
PCDHGB4	41.00	39.00	34.00	38.00	26.00
PCDHGB5	227.00	201.00	212.00	213.33	119.00
PCDHGB6	12.00	13.00	26.00	17.00	21.00
PCDHGB7	19.00	27.00	27.00	24.33	10.00
PCDHGC3	970.00	834.00	832.00	878.67	404.00
PCDHGC4	16.00	15.00	10.00	13.67	5.00
PCDHGC5	23.00	15.00	10.00	16.00	4.00
PCED1A	3414.00	3699.00	3815.00	3642.67	3894.00
PCED1B	433.00	406.00	433.00	424.00	348.00
PCF11	1995.00	1883.00	2172.00	2016.67	1815.00
PCGF1	415.00	455.00	452.00	440.67	561.00
PCGF3	694.00	681.00	751.00	708.67	755.00
PCGF5	827.00	790.00	958.00	858.33	812.00
PCGF6	328.00	314.00	315.00	319.00	435.00
PCID2	1402.00	1429.00	1604.00	1478.33	1670.00
PCIF1	612.00	521.00	518.00	550.33	595.00
PCK2	4655.00	4387.00	4287.00	4443.00	4869.00
PCLO	484.00	420.00	505.00	469.67	343.00
PCM1	1716.00	1597.00	1922.00	1745.00	1857.00
PCMT1	1505.00	1356.00	1342.00	1401.00	1835.00
PCMTD1	790.00	777.00	922.00	829.67	809.00
PCMTD2	658.00	583.00	771.00	670.67	761.00

PCNA	997.00	970.00	960.00	975.67	1535.00
PCNP	1901.00	1660.00	2071.00	1877.33	2286.00
PCNT	2470.00	2306.00	2457.00	2411.00	2164.00
PCNX	2475.00	2187.00	2612.00	2424.67	2021.00
PCNXL2	1248.00	1209.00	1232.00	1229.67	966.00
PCNXL3	4854.00	4668.00	4951.00	4824.33	3489.00
PCNXL4	2536.00	2270.00	2581.00	2462.33	2454.00
PCOLCE	6304.00	6439.00	5908.00	6217.00	5669.00
PCOLCE2	503.00	475.00	511.00	496.33	640.00
PCOLCE-AS1	55.00	53.00	62.00	56.67	51.00
PCSK1	9.00	8.00	6.00	7.67	4.00
PCSK4	131.00	146.00	186.00	154.33	124.00
PCSK7	1263.00	1214.00	1238.00	1238.33	1219.00
PCTP	295.00	270.00	312.00	292.33	375.00
PCYOX1	1763.00	1654.00	1948.00	1788.33	1403.00
PCYOX1L	507.00	471.00	464.00	480.67	484.00
PCYT1A	544.00	531.00	562.00	545.67	495.00
PCYT2	654.00	686.00	664.00	668.00	726.00
PDAP1	2559.00	2699.00	2537.00	2598.33	3385.00
PDC	4.00	1.00	4.00	3.00	0.00
PDCD10	732.00	630.00	718.00	693.33	910.00
PDCD11	6300.00	6047.00	6457.00	6268.00	5444.00
PDCD1LG2	1066.00	944.00	1097.00	1035.67	1210.00
PDCD2	1467.00	1357.00	1616.00	1480.00	1982.00
PDCD2L	214.00	230.00	227.00	223.67	356.00
PDCD4	2667.00	2551.00	2899.00	2705.67	2459.00
PDCD4-AS1	145.00	186.00	148.00	159.67	179.00
PDCD5	961.00	979.00	1032.00	990.67	1449.00
PDCD6	2941.00	2886.00	3048.00	2958.33	4000.00
PDCD6IP	4568.00	4231.00	4700.00	4499.67	5035.00
PDCD6IPP2	37.00	35.00	23.00	31.67	29.00
PDCD7	874.00	849.00	910.00	877.67	1041.00
PDCL	252.00	252.00	279.00	261.00	244.00
PDCL3	356.00	330.00	355.00	347.00	430.00
PDCL3P4	45.00	44.00	30.00	39.67	54.00
PDDC1	2800.00	2989.00	2993.00	2927.33	2653.00
PDE10A	12.00	17.00	26.00	18.33	15.00
PDE11A	19.00	10.00	14.00	14.33	9.00
PDE12	708.00	661.00	738.00	702.33	759.00
PDE1C	1150.00	1097.00	1202.00	1149.67	1263.00
PDE2A	152.00	154.00	166.00	157.33	142.00
PDE3A	20.00	20.00	21.00	20.33	9.00
PDE3B	462.00	436.00	533.00	477.00	608.00
PDE4A	563.00	482.00	553.00	532.67	496.00
PDE4B	224.00	183.00	203.00	203.33	232.00
PDE4C	10.00	12.00	19.00	13.67	10.00
PDE4D	792.00	784.00	934.00	836.67	824.00
PDE4DIP	2917.00	2948.00	2951.00	2938.67	2678.00

PDE5A	82.00	93.00	103.00	92.67	98.00
PDE6D	461.00	432.00	444.00	445.67	530.00
PDE6G	3.00	5.00	9.00	5.67	4.00
PDE7A	697.00	755.00	810.00	754.00	829.00
PDE7B	102.00	77.00	100.00	93.00	206.00
PDE8A	909.00	880.00	959.00	916.00	877.00
PDF	71.00	63.00	54.00	62.67	77.00
PDGFA	1349.00	1377.00	1454.00	1393.33	854.00
PDGFB	25.00	36.00	25.00	28.67	16.00
PDGFC	2641.00	2374.00	2591.00	2535.33	2435.00
PDGFD	125.00	96.00	113.00	111.33	123.00
PDGFRA	1397.00	1233.00	1371.00	1333.67	988.00
PDGFRB	58.00	63.00	68.00	63.00	29.00
PDGFRL	105.00	113.00	104.00	107.33	77.00
PDHA1	1176.00	1123.00	1286.00	1195.00	1361.00
PDHB	840.00	817.00	853.00	836.67	1123.00
PDHX	718.00	669.00	703.00	696.67	903.00
PDIA3	15725.00	15204.00	15473.00	15467.33	14544.00
PDIA3P1	1449.00	1358.00	1476.00	1427.67	1394.00
PDIA4	14463.00	14599.00	13884.00	14315.33	13957.00
PDIA5	1296.00	1275.00	1222.00	1264.33	1399.00
PDIA6	13719.00	13599.00	13451.00	13589.67	14895.00
PDIK1L	169.00	136.00	159.00	154.67	166.00
PDK1	502.00	519.00	614.00	545.00	755.00
PDK2	291.00	258.00	301.00	283.33	293.00
PDK3	205.00	170.00	210.00	195.00	200.00
PDK4	6.00	7.00	10.00	7.67	14.00
PDLIM1	887.00	920.00	1006.00	937.67	896.00
PDLIM2	449.00	410.00	397.00	418.67	532.00
PDLIM3	67.00	50.00	61.00	59.33	28.00
PDLIM4	635.00	628.00	699.00	654.00	723.00
PDLIM5	498.00	480.00	520.00	499.33	546.00
PDLIM7	1645.00	1700.00	1865.00	1736.67	2202.00
PDP1	4212.00	3924.00	4676.00	4270.67	3958.00
PDP2	737.00	692.00	888.00	772.33	626.00
PDPK1	785.00	725.00	815.00	775.00	575.00
PDPN	3.00	4.00	8.00	5.00	2.00
PDPR	2419.00	2202.00	2482.00	2367.67	1890.00
PDRG1	497.00	480.00	511.00	496.00	649.00
PDS5A	5309.00	4691.00	5784.00	5261.33	5756.00
PDS5B	624.00	603.00	682.00	636.33	647.00
PDSS1	342.00	313.00	343.00	332.67	478.00
PDSS2	430.00	371.00	421.00	407.33	472.00
PDXDC1	96.00	98.00	121.00	105.00	111.00
PDXDC2P	560.00	591.00	580.00	577.00	406.00
PDXK	2030.00	2049.00	2214.00	2097.67	1902.00
PDXP	486.00	441.00	437.00	454.67	588.00
PDZD11	998.00	875.00	1014.00	962.33	1190.00

PDZD2	26.00	23.00	22.00	23.67	7.00
PDZD7	5.00	3.00	6.00	4.67	4.00
PDZD8	1821.00	1682.00	1981.00	1828.00	1815.00
PDZK1	8.00	4.00	13.00	8.33	4.00
PDZRN3	20.00	27.00	24.00	23.67	23.00
PDZRN3-AS1	3.00	0.00	2.00	1.67	1.00
PEA15	6188.00	5932.00	6443.00	6187.67	5751.00
PEAK1	1334.00	1171.00	1281.00	1262.00	1050.00
PEAR1	7441.00	7554.00	7433.00	7476.00	6172.00
PEBP1	3314.00	3157.00	3529.00	3333.33	4116.00
PECAM1	3.00	4.00	6.00	4.33	5.00
PECR	136.00	109.00	150.00	131.67	179.00
PEF1	962.00	921.00	939.00	940.67	1057.00
PEG10	1201.00	1022.00	1242.00	1155.00	1389.00
PELI1	229.00	210.00	261.00	233.33	275.00
PELI2	29.00	27.00	37.00	31.00	40.00
PELI3	178.00	160.00	169.00	169.00	111.00
PELO	314.00	335.00	351.00	333.33	362.00
PELP1	2730.00	2806.00	2733.00	2756.33	2970.00
PEMT	717.00	716.00	745.00	726.00	839.00
PEPD	846.00	785.00	830.00	820.33	932.00
PER1	726.00	636.00	753.00	705.00	674.00
PER2	412.00	368.00	395.00	391.67	306.00
PER3	359.00	293.00	381.00	344.33	414.00
PERP	2276.00	2251.00	2189.00	2238.67	2563.00
PES1	2612.00	2555.00	2838.00	2668.33	3241.00
PET100	211.00	311.00	291.00	271.00	370.00
PET117	113.00	117.00	125.00	118.33	135.00
PEX1	897.00	947.00	983.00	942.33	847.00
PEX10	664.00	716.00	718.00	699.33	823.00
PEX11A	171.00	185.00	205.00	187.00	187.00
PEX11B	408.00	441.00	433.00	427.33	536.00
PEX11G	26.00	37.00	30.00	31.00	45.00
PEX12	289.00	273.00	276.00	279.33	312.00
PEX13	393.00	316.00	378.00	362.33	428.00
PEX14	505.00	497.00	499.00	500.33	572.00
PEX16	407.00	423.00	431.00	420.33	438.00
PEX19	1080.00	906.00	1046.00	1010.67	1083.00
PEX2	566.00	608.00	739.00	637.67	772.00
PEX26	591.00	521.00	587.00	566.33	550.00
PEX3	464.00	468.00	489.00	473.67	546.00
PEX5	1482.00	1390.00	1571.00	1481.00	1407.00
PEX5L	142.00	136.00	119.00	132.33	370.00
PEX6	3136.00	3096.00	3284.00	3172.00	2883.00
PEX7	186.00	194.00	205.00	195.00	277.00
PF4	8.00	8.00	13.00	9.67	8.00
PFAS	4929.00	4553.00	4899.00	4793.67	4693.00
PFDN1	2272.00	2141.00	2273.00	2228.67	3021.00

PFDN2	1855.00	1925.00	1902.00	1894.00	2690.00
PFDN4	551.00	538.00	608.00	565.67	749.00
PFDN5	3495.00	3643.00	3879.00	3672.33	4862.00
PFDN6	3.00	0.00	3.00	2.00	0.00
PFKFB2	438.00	389.00	467.00	431.33	417.00
PFKFB3	1411.00	1484.00	1645.00	1513.33	1809.00
PFKFB4	144.00	170.00	158.00	157.33	112.00
PFKL	2112.00	2103.00	2221.00	2145.33	2200.00
PFKM	3462.00	3326.00	3886.00	3558.00	3829.00
PFKP	11303.00	10832.00	11510.00	11215.00	10140.00
PFN1	18361.00	17762.00	18757.00	18293.33	25412.00
PFN2	4499.00	4404.00	4846.00	4583.00	6404.00
PFN4	29.00	19.00	22.00	23.33	21.00
PGAM1	1441.00	1304.00	1496.00	1413.67	1964.00
PGAM2	4.00	7.00	10.00	7.00	12.00
PGAM4	3.00	9.00	10.00	7.33	8.00
PGAM5	812.00	714.00	836.00	787.33	1032.00
PGAP1	560.00	485.00	611.00	552.00	450.00
PGAP2	621.00	649.00	651.00	640.33	843.00
PGAP3	258.00	189.00	237.00	228.00	153.00
PGBD1	284.00	278.00	333.00	298.33	336.00
PGBD2	243.00	284.00	245.00	257.33	292.00
PGBD4	261.00	250.00	263.00	258.00	238.00
PGD	3550.00	3313.00	3618.00	3493.67	3615.00
PGF	594.00	593.00	553.00	580.00	533.00
PGGT1B	180.00	175.00	199.00	184.67	185.00
PGK1	9451.00	8839.00	9724.00	9338.00	12212.00
PGLS	1651.00	1674.00	1840.00	1721.67	2026.00
PGM1	678.00	690.00	692.00	686.67	737.00
PGM2	1568.00	1406.00	1638.00	1537.33	1969.00
PGM2L1	170.00	159.00	160.00	163.00	141.00
PGM3	1833.00	1779.00	2020.00	1877.33	1882.00
PGM5	62.00	19.00	34.00	38.33	21.00
PGM5P2	1099.00	1081.00	1264.00	1148.00	553.00
PGM5P3-AS1	32.00	52.00	41.00	41.67	45.00
PGP	806.00	847.00	850.00	834.33	840.00
PGPEP1	623.00	676.00	717.00	672.00	503.00
PGR	112.00	87.00	129.00	109.33	88.00
PGRMC1	1085.00	1174.00	1118.00	1125.67	1523.00
PGRMC2	1052.00	1107.00	1106.00	1088.33	1147.00
PGS1	1584.00	1603.00	1710.00	1632.33	1494.00
PHACTR1	274.00	238.00	251.00	254.33	278.00
PHACTR2	555.00	492.00	536.00	527.67	594.00
PHACTR4	881.00	846.00	866.00	864.33	876.00
PHAX	653.00	621.00	720.00	664.67	780.00
PHB	2912.00	2887.00	3090.00	2963.00	3940.00
PHB2	4535.00	4829.00	4988.00	4784.00	6146.00
PHC1	689.00	655.00	812.00	718.67	551.00

PHC2	3457.00	3304.00	3502.00	3421.00	3569.00
PHC3	1315.00	1204.00	1573.00	1364.00	1152.00
PHEX	53.00	50.00	69.00	57.33	75.00
PHF1	79.00	76.00	101.00	85.33	84.00
PHF10	807.00	745.00	901.00	817.67	791.00
PHF11	142.00	126.00	138.00	135.33	147.00
PHF12	1207.00	1144.00	1138.00	1163.00	944.00
PHF13	559.00	563.00	570.00	564.00	619.00
PHF14	1366.00	1355.00	1400.00	1373.67	1726.00
PHF19	917.00	838.00	898.00	884.33	1125.00
PHF2	848.00	849.00	877.00	858.00	757.00
PHF20	1388.00	1329.00	1570.00	1429.00	1374.00
PHF20L1	2099.00	2043.00	2292.00	2144.67	2169.00
PHF21A	932.00	867.00	895.00	898.00	883.00
PHF23	1388.00	1417.00	1625.00	1476.67	1759.00
PHF3	1779.00	1675.00	2101.00	1851.67	1651.00
PHF5A	531.00	527.00	546.00	534.67	793.00
PHF6	926.00	783.00	947.00	885.33	1010.00
PHF7	124.00	148.00	133.00	135.00	127.00
PHF8	1423.00	1351.00	1448.00	1407.33	1074.00
PHGDH	12180.00	11948.00	12431.00	12186.33	14692.00
PHIP	1772.00	1723.00	1988.00	1827.67	1764.00
PHKA1	725.00	697.00	777.00	733.00	659.00
PHKA2	725.00	737.00	843.00	768.33	671.00
PHKB	1035.00	1108.00	1149.00	1097.33	1088.00
PHKG1	21.00	24.00	33.00	26.00	13.00
PHKG2	734.00	777.00	840.00	783.67	789.00
PHLDA1	8017.00	7583.00	8807.00	8135.67	7430.00
PHLDA3	217.00	247.00	239.00	234.33	218.00
PHLDB1	1899.00	1993.00	1941.00	1944.33	1857.00
PHLDB2	1019.00	865.00	1040.00	974.67	261.00
PHLDB3	127.00	107.00	114.00	116.00	129.00
PHLPP1	374.00	388.00	398.00	386.67	249.00
PHLPP2	694.00	607.00	650.00	650.33	605.00
PHOSPHO2	66.00	62.00	63.00	63.67	46.00
PHPT1	1072.00	1078.00	1091.00	1080.33	1272.00
PHRF1	31.00	30.00	31.00	30.67	50.00
PHTF1	512.00	503.00	482.00	499.00	537.00
PHTF2	757.00	688.00	730.00	725.00	901.00
PHYH	502.00	477.00	561.00	513.33	577.00
PHYHD1	137.00	125.00	112.00	124.67	141.00
PHYHIP	31.00	14.00	18.00	21.00	15.00
PHYKPL	787.00	681.00	745.00	737.67	696.00
PI15	5.00	1.00	6.00	4.00	2.00
PI4K2A	1036.00	1013.00	1111.00	1053.33	1021.00
PI4K2B	239.00	183.00	239.00	220.33	313.00
PI4KA	1700.00	1608.00	1783.00	1697.00	1419.00
PI4KAP1	60.00	82.00	109.00	83.67	74.00

PI4KAP2	157.00	140.00	166.00	154.33	160.00
PI4KB	1967.00	1769.00	1976.00	1904.00	2013.00
PIANP	8.00	12.00	8.00	9.33	3.00
PIAS1	332.00	316.00	368.00	338.67	326.00
PIAS2	589.00	578.00	599.00	588.67	619.00
PIAS3	879.00	854.00	905.00	879.33	1135.00
PIAS4	449.00	443.00	512.00	468.00	478.00
PIBF1	476.00	443.00	468.00	462.33	589.00
PICALM	2710.00	2439.00	2891.00	2680.00	2735.00
PICK1	224.00	276.00	204.00	234.67	243.00
PID1	30.00	19.00	25.00	24.67	36.00
PIDD1	608.00	578.00	635.00	607.00	567.00
PIEZO1	8079.00	7865.00	8297.00	8080.33	6811.00
PIF1	809.00	776.00	778.00	787.67	963.00
PIGA	198.00	192.00	225.00	205.00	233.00
PIGB	559.00	551.00	507.00	539.00	561.00
PIGBOS1	180.00	183.00	191.00	184.67	250.00
PIGC	694.00	699.00	625.00	672.67	836.00
PIGF	574.00	628.00	545.00	582.33	708.00
PIGG	1176.00	1146.00	1193.00	1171.67	1009.00
PIGH	375.00	392.00	366.00	377.67	491.00
PIGK	2716.00	2520.00	2585.00	2607.00	2650.00
PIGL	232.00	234.00	266.00	244.00	262.00
PIGM	684.00	638.00	683.00	668.33	646.00
PIGN	838.00	767.00	868.00	824.33	699.00
PIGO	2097.00	1912.00	1964.00	1991.00	1757.00
PIGP	220.00	205.00	227.00	217.33	300.00
PIGQ	991.00	975.00	1038.00	1001.33	1090.00
PIGS	1062.00	1057.00	1012.00	1043.67	1077.00
PIGT	4609.00	4483.00	4664.00	4585.33	4388.00
PIGU	1365.00	1331.00	1348.00	1348.00	1349.00
PIGV	784.00	740.00	769.00	764.33	841.00
PIGX	269.00	266.00	268.00	267.67	289.00
PIGZ	187.00	171.00	183.00	180.33	205.00
PIH1D1	812.00	730.00	795.00	779.00	918.00
PIH1D2	44.00	36.00	52.00	44.00	41.00
PIK3C2A	1358.00	1242.00	1476.00	1358.67	1106.00
PIK3C2B	21.00	16.00	8.00	15.00	16.00
PIK3C3	570.00	535.00	584.00	563.00	545.00
PIK3CA	625.00	486.00	575.00	562.00	538.00
PIK3CB	698.00	594.00	706.00	666.00	679.00
PIK3CD	1578.00	1435.00	1543.00	1518.67	1271.00
PIK3CD-AS2	19.00	18.00	16.00	17.67	37.00
PIK3CG	139.00	115.00	140.00	131.33	134.00
PIK3IP1	19.00	11.00	14.00	14.67	15.00
PIK3R1	523.00	499.00	581.00	534.33	500.00
PIK3R2	1574.00	1523.00	1606.00	1567.67	1416.00
PIK3R3	61.00	59.00	53.00	57.67	54.00

PIK3R4	1501.00	1575.00	1679.00	1585.00	1510.00
PIKFYVE	1912.00	1766.00	2010.00	1896.00	1696.00
PILRA	10.00	12.00	6.00	9.33	8.00
PILRB	21.00	28.00	20.00	23.00	35.00
PIM1	874.00	816.00	914.00	868.00	1090.00
PIM2	140.00	117.00	145.00	134.00	167.00
PIM3	1125.00	1059.00	1205.00	1129.67	1492.00
PIN1	829.00	736.00	860.00	808.33	933.00
PIN4	359.00	378.00	370.00	369.00	503.00
PIN4P1	13.00	19.00	17.00	16.33	8.00
PINK1	1248.00	1273.00	1287.00	1269.33	1039.00
PINK1-AS	339.00	296.00	359.00	331.33	301.00
PINLYP	65.00	65.00	87.00	72.33	35.00
PINX1	222.00	256.00	251.00	243.00	279.00
PIP4K2A	1689.00	1593.00	1866.00	1716.00	1484.00
PIP4K2C	783.00	786.00	865.00	811.33	834.00
PIP5K1A	2713.00	2378.00	2777.00	2622.67	2693.00
PIP5K1C	1209.00	1103.00	1268.00	1193.33	1059.00
PIP5KL1	31.00	40.00	40.00	37.00	30.00
PIPSL	40.00	21.00	37.00	32.67	23.00
PIR	35.00	31.00	47.00	37.67	50.00
PISD	1346.00	1282.00	1321.00	1316.33	1246.00
PITHD1	611.00	553.00	664.00	609.33	934.00
PITPNA	1678.00	1554.00	1599.00	1610.33	1624.00
PITPNA-AS1	164.00	185.00	180.00	176.33	210.00
PITPNB	1857.00	1750.00	1953.00	1853.33	2235.00
PITPNC1	295.00	294.00	350.00	313.00	349.00
PITPNM1	1118.00	1128.00	1083.00	1109.67	907.00
PITPNM2	1043.00	1077.00	1304.00	1141.33	920.00
PITPNM3	29.00	29.00	42.00	33.33	33.00
PITRM1	4258.00	4168.00	4751.00	4392.33	3857.00
PITRM1-AS1	13.00	10.00	14.00	12.33	13.00
PITX1	186.00	178.00	175.00	179.67	252.00
PITX2	47.00	46.00	40.00	44.33	50.00
PITX3	3.00	1.00	0.00	1.33	1.00
PIWIL4	58.00	38.00	52.00	49.33	45.00
PJA1	901.00	788.00	891.00	860.00	933.00
PJA2	1876.00	1546.00	1914.00	1778.67	1590.00
PKD1	4981.00	4889.00	5496.00	5122.00	3685.00
PKD1L1	3.00	7.00	6.00	5.33	8.00
PKD1L2	47.00	45.00	58.00	50.00	48.00
PKD1P1	153.00	155.00	149.00	152.33	116.00
PKD1P6	262.00	332.00	317.00	303.67	266.00
PKD2	1409.00	1368.00	1551.00	1442.67	1497.00
PKD2L2	5.00	2.00	5.00	4.00	5.00
PKDCC	34.00	45.00	58.00	45.67	66.00
PKI55	302.00	297.00	316.00	305.00	317.00
PKIA	805.00	800.00	886.00	830.33	1243.00

PKIB	20.00	21.00	35.00	25.33	28.00
PKIG	152.00	176.00	198.00	175.33	259.00
PKM	26262.00	24961.00	26148.00	25790.33	26940.00
PKMYT1	498.00	431.00	412.00	447.00	600.00
PKN1	3957.00	4027.00	4252.00	4078.67	4204.00
PKN2	1933.00	1631.00	1994.00	1852.67	1848.00
PKN2-AS1	8.00	5.00	5.00	6.00	14.00
PKN3	673.00	755.00	768.00	732.00	886.00
PKNOX1	619.00	629.00	686.00	644.67	589.00
PKP3	39.00	37.00	61.00	45.67	64.00
PKP4	1972.00	1845.00	2057.00	1958.00	2092.00
PLA2G12A	313.00	277.00	304.00	298.00	307.00
PLA2G12B	3.00	1.00	0.00	1.33	0.00
PLA2G15	675.00	640.00	681.00	665.33	601.00
PLA2G16	156.00	147.00	157.00	153.33	133.00
PLA2G4A	389.00	369.00	356.00	371.33	305.00
PLA2G4B	24.00	12.00	23.00	19.67	23.00
PLA2G4C	50.00	38.00	45.00	44.33	54.00
PLA2G6	316.00	339.00	347.00	334.00	285.00
PLA2G7	7.00	7.00	11.00	8.33	10.00
PLA2R1	4.00	6.00	5.00	5.00	3.00
PLAA	1038.00	1012.00	1078.00	1042.67	1360.00
PLAC1	63.00	64.00	64.00	63.67	82.00
PLAC8	5.00	5.00	3.00	4.33	10.00
PLAC8L1	53.00	51.00	51.00	51.67	72.00
PLAG1	711.00	659.00	799.00	723.00	631.00
PLAGL1	1188.00	1160.00	1263.00	1203.67	1010.00
PLAGL2	911.00	786.00	867.00	854.67	847.00
PLAT	42487.00	41434.00	39963.00	41294.67	48035.00
PLAU	23078.00	22743.00	21550.00	22457.00	16792.00
PLAUR	12406.00	12817.00	11642.00	12288.33	12789.00
PLB1	6.00	6.00	8.00	6.67	6.00
PLBD2	2127.00	2052.00	2034.00	2071.00	1286.00
PLCB1	766.00	603.00	791.00	720.00	601.00
PLCB2	67.00	113.00	96.00	92.00	123.00
PLCB3	2963.00	2918.00	3055.00	2978.67	2418.00
PLCB4	184.00	215.00	194.00	197.67	180.00
PLCD1	30.00	63.00	39.00	44.00	38.00
PLCD3	294.00	249.00	271.00	271.33	255.00
PLCD4	12.00	16.00	28.00	18.67	46.00
PLCE1	780.00	682.00	780.00	747.33	545.00
PLCG1	4279.00	4299.00	4836.00	4471.33	4054.00
PLCG1-AS1	20.00	7.00	14.00	13.67	9.00
PLCG2	7.00	3.00	2.00	4.00	4.00
PLCH1	79.00	67.00	64.00	70.00	71.00
PLCL2	191.00	193.00	208.00	197.33	185.00
PLCXD2	78.00	64.00	71.00	71.00	31.00
PLCZ1	47.00	50.00	55.00	50.67	48.00

PLD1	116.00	78.00	118.00	104.00	132.00
PLD2	1196.00	1275.00	1283.00	1251.33	1151.00
PLD3	4135.00	4400.00	4355.00	4296.67	3780.00
PLD6	465.00	405.00	403.00	424.33	536.00
PLEC	30576.00	30685.00	32591.00	31284.00	17659.00
PLEK2	33.00	41.00	47.00	40.33	34.00
PLEKHA1	450.00	473.00	525.00	482.67	472.00
PLEKHA2	1383.00	1161.00	1259.00	1267.67	1518.00
PLEKHA3	309.00	279.00	355.00	314.33	352.00
PLEKHA4	217.00	175.00	195.00	195.67	235.00
PLEKHA5	1266.00	1186.00	1310.00	1254.00	996.00
PLEKHA6	55.00	63.00	58.00	58.67	23.00
PLEKHA7	6.00	5.00	7.00	6.00	8.00
PLEKHA8	658.00	679.00	790.00	709.00	613.00
PLEKHA8P1	101.00	104.00	115.00	106.67	109.00
PLEKHB2	2716.00	2631.00	2975.00	2774.00	3162.00
PLEKHF1	146.00	141.00	130.00	139.00	157.00
PLEKHF2	334.00	310.00	354.00	332.67	316.00
PLEKHG2	1028.00	979.00	1060.00	1022.33	952.00
PLEKHG3	536.00	601.00	594.00	577.00	599.00
PLEKHG4	2842.00	2931.00	3064.00	2945.67	1954.00
PLEKHG4B	51.00	36.00	47.00	44.67	34.00
PLEKHG5	1527.00	1565.00	1653.00	1581.67	1568.00
PLEKHH1	502.00	429.00	473.00	468.00	473.00
PLEKHH2	129.00	155.00	164.00	149.33	134.00
PLEKHH3	438.00	482.00	498.00	472.67	535.00
PLEKHJ1	897.00	989.00	972.00	952.67	1093.00
PLEKHM1	149.00	142.00	152.00	147.67	147.00
PLEKHM1P	758.00	685.00	780.00	741.00	442.00
PLEKHM2	2412.00	2297.00	2522.00	2410.33	2151.00
PLEKHM3	343.00	382.00	391.00	372.00	329.00
PLEKHN1	178.00	179.00	204.00	187.00	199.00
PLEKHO1	517.00	522.00	565.00	534.67	570.00
PLEKHO2	743.00	782.00	777.00	767.33	898.00
PLGRKT	634.00	610.00	632.00	625.33	752.00
PLIN1	9.00	9.00	5.00	7.67	6.00
PLIN2	539.00	549.00	602.00	563.33	596.00
PLIN3	1517.00	1448.00	1507.00	1490.67	1856.00
PLK1	1238.00	1118.00	1208.00	1188.00	1547.00
PLK2	1868.00	1737.00	1839.00	1814.67	2418.00
PLK3	464.00	439.00	448.00	450.33	503.00
PLK4	422.00	398.00	440.00	420.00	462.00
PLK5	3.00	4.00	1.00	2.67	1.00
PLOD1	7099.00	7354.00	7159.00	7204.00	5803.00
PLOD2	18904.00	17494.00	19513.00	18637.00	18583.00
PLOD3	6203.00	6405.00	6334.00	6314.00	6204.00
PLP2	1778.00	1809.00	1673.00	1753.33	1990.00
PLRG1	1765.00	1627.00	1820.00	1737.33	2034.00

PLS1	177.00	133.00	192.00	167.33	206.00
PLS3	8023.00	7137.00	8391.00	7850.33	8869.00
PLS3-AS1	64.00	91.00	77.00	77.33	94.00
PLSCR1	666.00	608.00	719.00	664.33	786.00
PLSCR3	8.00	16.00	17.00	13.67	20.00
PLSCR4	156.00	124.00	140.00	140.00	113.00
PLTP	3450.00	3537.00	3495.00	3494.00	3833.00
PLXDC2	5.00	2.00	6.00	4.33	3.00
PLXNA1	5790.00	5476.00	5818.00	5694.67	3975.00
PLXNA2	4274.00	3997.00	3826.00	4032.33	5292.00
PLXNA3	732.00	748.00	904.00	794.67	694.00
PLXNA4	3475.00	3216.00	3493.00	3394.67	2005.00
PLXNB1	137.00	155.00	156.00	149.33	109.00
PLXNB2	2072.00	2100.00	2057.00	2076.33	1294.00
PLXNB3	985.00	1043.00	1071.00	1033.00	772.00
PLXNC1	140.00	121.00	154.00	138.33	131.00
PLXND1	3365.00	3357.00	3583.00	3435.00	2195.00
PM20D2	374.00	310.00	436.00	373.33	443.00
PMAIP1	1359.00	1154.00	1476.00	1329.67	1928.00
PMEL	4.00	6.00	10.00	6.67	7.00
PMEPA1	222.00	229.00	296.00	249.00	189.00
PMF1	222.00	180.00	202.00	201.33	265.00
PMF1-BGLAP	9.00	9.00	9.00	9.00	7.00
PMFBP1	20.00	26.00	25.00	23.67	18.00
PML	801.00	763.00	784.00	782.67	772.00
PMM1	420.00	437.00	400.00	419.00	541.00
PMM2	903.00	894.00	970.00	922.33	929.00
PMP22	487.00	439.00	486.00	470.67	464.00
PMPCA	2055.00	2008.00	2027.00	2030.00	2178.00
PMPCB	1318.00	1292.00	1380.00	1330.00	1457.00
PMS1	1259.00	1196.00	1315.00	1256.67	1466.00
PMS2	538.00	507.00	561.00	535.33	533.00
PMS2CL	130.00	150.00	164.00	148.00	165.00
PMS2P1	246.00	234.00	277.00	252.33	373.00
PMS2P2	3.00	2.00	2.00	2.33	5.00
PMS2P3	167.00	200.00	181.00	182.67	153.00
PMS2P4	19.00	24.00	20.00	21.00	39.00
PMS2P5	51.00	56.00	61.00	56.00	64.00
PMS2P7	11.00	15.00	9.00	11.67	11.00
PMS2P9	9.00	14.00	8.00	10.33	10.00
PMVK	496.00	503.00	540.00	513.00	729.00
PNCK	4.00	1.00	0.00	1.67	3.00
PNISR	6218.00	6223.00	6869.00	6436.67	5898.00
PNKD	462.00	504.00	513.00	493.00	507.00
PNKP	517.00	567.00	539.00	541.00	538.00
PNLDC1	3.00	6.00	3.00	4.00	2.00
PNMA1	1226.00	1178.00	1257.00	1220.33	1312.00
PNMA2	257.00	220.00	273.00	250.00	158.00

PNMA6A	14.00	9.00	6.00	9.67	9.00
PNMAL1	523.00	547.00	559.00	543.00	711.00
PNN	9432.00	9454.00	9810.00	9565.33	10340.00
PNO1	908.00	816.00	981.00	901.67	1048.00
PNP	2046.00	2022.00	2183.00	2083.67	3276.00
PNPLA2	1454.00	1390.00	1515.00	1453.00	1229.00
PNPLA3	286.00	256.00	354.00	298.67	183.00
PNPLA4	334.00	358.00	417.00	369.67	441.00
PNPLA6	3120.00	3216.00	3121.00	3152.33	2775.00
PNPLA7	27.00	37.00	31.00	31.67	38.00
PNPLA8	526.00	461.00	545.00	510.67	466.00
PNPO	750.00	633.00	703.00	695.33	776.00
PNPT1	1503.00	1444.00	1539.00	1495.33	1754.00
PNRC1	278.00	249.00	299.00	275.33	226.00
PNRC2	1456.00	1430.00	1503.00	1463.00	1833.00
POC1A	413.00	424.00	439.00	425.33	558.00
POC1B	661.00	541.00	678.00	626.67	622.00
POC5	370.00	352.00	383.00	368.33	454.00
PODNL1	3.00	9.00	7.00	6.33	5.00
PODXL	4382.00	4329.00	4514.00	4408.33	4206.00
PODXL2	1890.00	2111.00	2201.00	2067.33	2466.00
POFUT1	3909.00	3561.00	3858.00	3776.00	2811.00
POFUT2	1993.00	2142.00	1982.00	2039.00	1731.00
POGK	2700.00	2555.00	2890.00	2715.00	3086.00
POGLUT1	1295.00	1227.00	1290.00	1270.67	1013.00
POGZ	3539.00	3422.00	3787.00	3582.67	3584.00
POLA1	526.00	475.00	511.00	504.00	586.00
POLA2	985.00	898.00	905.00	929.33	1221.00
POLB	420.00	413.00	424.00	419.00	491.00
POLD1	1271.00	1217.00	1341.00	1276.33	1476.00
POLD2	2731.00	2645.00	2778.00	2718.00	3585.00
POLD3	442.00	412.00	449.00	434.33	585.00
POLD4	633.00	727.00	727.00	695.67	827.00
POLDIP2	2050.00	2079.00	2287.00	2138.67	2630.00
POLDIP3	1337.00	1268.00	1440.00	1348.33	1225.00
POLE	1764.00	1804.00	1801.00	1789.67	1869.00
POLE2	135.00	124.00	118.00	125.67	199.00
POLE3	1055.00	976.00	1052.00	1027.67	1450.00
POLE4	482.00	500.00	537.00	506.33	751.00
POLG	2020.00	1918.00	2201.00	2046.33	1944.00
POLG2	1040.00	1073.00	1117.00	1076.67	1028.00
POLH	314.00	264.00	320.00	299.33	297.00
POLI	917.00	878.00	1008.00	934.33	959.00
POLK	995.00	928.00	1016.00	979.67	1009.00
POLL	1022.00	1066.00	1024.00	1037.33	1107.00
POLM	1656.00	1705.00	1827.00	1729.33	1982.00
POLN	136.00	123.00	142.00	133.67	103.00
POLQ	488.00	456.00	454.00	466.00	590.00

POLR1A	2166.00	1910.00	2187.00	2087.67	1825.00
POLR1B	1983.00	1826.00	2124.00	1977.67	2194.00
POLR1C	1146.00	1165.00	1289.00	1200.00	1398.00
POLR1D	1709.00	1677.00	1847.00	1744.33	2244.00
POLR1E	652.00	690.00	749.00	697.00	951.00
POLR2A	6623.00	6244.00	6403.00	6423.33	5016.00
POLR2B	3317.00	3101.00	3630.00	3349.33	3764.00
POLR2C	1399.00	1247.00	1407.00	1351.00	1905.00
POLR2D	605.00	626.00	654.00	628.33	813.00
POLR2E	1829.00	1841.00	1969.00	1879.67	2282.00
POLR2F	440.00	467.00	504.00	470.33	571.00
POLR2G	906.00	965.00	1004.00	958.33	1280.00
POLR2H	1468.00	1379.00	1521.00	1456.00	1775.00
POLR2I	265.00	260.00	296.00	273.67	392.00
POLR2J	194.00	180.00	206.00	193.33	263.00
POLR2J3	44.00	30.00	27.00	33.67	23.00
POLR2J4	256.00	271.00	291.00	272.67	267.00
POLR2K	747.00	749.00	838.00	778.00	1094.00
POLR2L	909.00	899.00	889.00	899.00	1205.00
POLR2M	51.00	36.00	45.00	44.00	48.00
POLR3A	2615.00	2487.00	2851.00	2651.00	2543.00
POLR3B	642.00	580.00	633.00	618.33	644.00
POLR3C	985.00	954.00	909.00	949.33	1154.00
POLR3D	871.00	791.00	930.00	864.00	864.00
POLR3E	1466.00	1439.00	1655.00	1520.00	1638.00
POLR3F	492.00	432.00	536.00	486.67	566.00
POLR3G	861.00	730.00	1012.00	867.67	978.00
POLR3GL	315.00	264.00	278.00	285.67	364.00
POLR3H	1061.00	1132.00	1079.00	1090.67	1196.00
POLR3K	258.00	288.00	283.00	276.33	424.00
POLRMT	2452.00	2508.00	2688.00	2549.33	2357.00
POM121	1126.00	1098.00	1076.00	1100.00	834.00
POM121C	1546.00	1370.00	1435.00	1450.33	1262.00
POM121L10P	3.00	6.00	7.00	5.33	2.00
POM121L9P	105.00	109.00	107.00	107.00	68.00
POMGNT1	2798.00	2758.00	2808.00	2788.00	2618.00
POMGNT2	477.00	489.00	554.00	506.67	510.00
POMK	25.00	34.00	29.00	29.33	19.00
POMP	1098.00	1090.00	1057.00	1081.67	1492.00
POMT1	1382.00	1429.00	1427.00	1412.67	1307.00
POMT2	1650.00	1626.00	1658.00	1644.67	1219.00
PON2	2183.00	2206.00	2148.00	2179.00	2474.00
POP1	719.00	631.00	732.00	694.00	775.00
POP4	430.00	423.00	475.00	442.67	564.00
POP5	370.00	447.00	475.00	430.67	565.00
POP7	510.00	487.00	559.00	518.67	667.00
POPDC2	36.00	19.00	14.00	23.00	22.00
POPDC3	554.00	561.00	602.00	572.33	847.00

POR	2246.00	2506.00	2380.00	2377.33	2364.00
PORCN	437.00	520.00	509.00	488.67	406.00
POT1	848.00	808.00	957.00	871.00	1026.00
POT1-AS1	5.00	13.00	6.00	8.00	20.00
POU2F1	940.00	844.00	944.00	909.33	788.00
POU2F2	1922.00	1988.00	2086.00	1998.67	2243.00
POU3F2	29.00	29.00	28.00	28.67	40.00
POU4F1	1077.00	1052.00	1140.00	1089.67	1301.00
POU6F1	129.00	147.00	184.00	153.33	125.00
PP7080	152.00	166.00	174.00	164.00	180.00
PPA1	2768.00	2613.00	3025.00	2802.00	3636.00
PPA2	669.00	639.00	710.00	672.67	830.00
PPAN	228.00	227.00	259.00	238.00	201.00
PPAP2A	356.00	313.00	305.00	324.67	361.00
PPAP2B	2271.00	2123.00	2264.00	2219.33	1831.00
PPAP2C	1602.00	1627.00	1513.00	1580.67	1879.00
PPAPDC1A	45.00	51.00	33.00	43.00	36.00
PPAPDC1B	1075.00	1042.00	1103.00	1073.33	1130.00
PPAPDC2	617.00	566.00	588.00	590.33	549.00
PPAPDC3	62.00	55.00	72.00	63.00	61.00
PPARA	472.00	424.00	449.00	448.33	294.00
PPARD	3129.00	3064.00	3170.00	3121.00	3433.00
PPARG	522.00	505.00	549.00	525.33	434.00
PPARGC1A	6.00	13.00	21.00	13.33	17.00
PPARGC1B	347.00	369.00	396.00	370.67	347.00
PPAT	617.00	589.00	674.00	626.67	752.00
PPCDC	202.00	232.00	209.00	214.33	323.00
PPCS	692.00	617.00	733.00	680.67	922.00
PPDPF	1206.00	1106.00	1244.00	1185.33	1566.00
PPFIA1	8155.00	7894.00	8815.00	8288.00	7574.00
PPFIA2	187.00	189.00	150.00	175.33	167.00
PPFIA3	235.00	246.00	300.00	260.33	231.00
PPFIA4	10.00	7.00	10.00	9.00	8.00
PPFIBP1	4256.00	3915.00	4419.00	4196.67	3024.00
PPFIBP2	24.00	26.00	25.00	25.00	34.00
PPHLN1	1205.00	1167.00	1370.00	1247.33	1249.00
PPIA	8984.00	8812.00	9372.00	9056.00	12491.00
PPIAL4A	19.00	34.00	24.00	25.67	35.00
PPIAP30	3.00	0.00	1.00	1.33	0.00
PPIB	12832.00	13090.00	12226.00	12716.00	12334.00
PPIC	677.00	701.00	646.00	674.67	784.00
PPID	815.00	780.00	894.00	829.67	1151.00
PPIE	1011.00	1000.00	1041.00	1017.33	1316.00
PPIEL	162.00	177.00	176.00	171.67	140.00
PPIF	2556.00	2476.00	2671.00	2567.67	2894.00
PPIG	1039.00	991.00	1122.00	1050.67	1207.00
PPIH	703.00	671.00	670.00	681.33	964.00
PPIL1	984.00	854.00	1013.00	950.33	1340.00

PPIL2	881.00	791.00	844.00	838.67	924.00
PPIL3	497.00	451.00	524.00	490.67	644.00
PPIL4	784.00	741.00	786.00	770.33	956.00
PPIL6	50.00	33.00	61.00	48.00	58.00
PPIP5K1	640.00	601.00	638.00	626.33	616.00
PPIP5K2	771.00	675.00	922.00	789.33	892.00
PPM1A	1098.00	991.00	1177.00	1088.67	1250.00
PPM1B	650.00	595.00	689.00	644.67	714.00
PPM1D	245.00	245.00	248.00	246.00	325.00
PPM1E	431.00	361.00	435.00	409.00	450.00
PPM1F	2219.00	2195.00	2239.00	2217.67	2085.00
PPM1G	3238.00	3082.00	3343.00	3221.00	4054.00
PPM1H	149.00	130.00	169.00	149.33	146.00
PPM1J	31.00	22.00	21.00	24.67	31.00
PPM1K	464.00	356.00	484.00	434.67	493.00
PPM1L	78.00	70.00	101.00	83.00	36.00
PPM1M	653.00	576.00	645.00	624.67	636.00
PPM1N	17.00	35.00	22.00	24.67	23.00
PPME1	1053.00	1007.00	1075.00	1045.00	1029.00
PPOX	941.00	924.00	936.00	933.67	986.00
PPP1CA	1871.00	1770.00	1913.00	1851.33	2531.00
PPP1CB	1829.00	1562.00	2008.00	1799.67	2202.00
PPP1CC	4025.00	3721.00	4328.00	4024.67	5153.00
PPP1R11	85.00	86.00	85.00	85.33	83.00
PPP1R12A	1452.00	1316.00	1542.00	1436.67	1423.00
PPP1R12B	514.00	459.00	518.00	497.00	363.00
PPP1R12C	2112.00	2214.00	2299.00	2208.33	2236.00
PPP1R13B	344.00	340.00	352.00	345.33	319.00
PPP1R13L	323.00	329.00	305.00	319.00	295.00
PPP1R14B	4357.00	4366.00	4590.00	4437.67	5589.00
PPP1R15A	11212.00	11454.00	11626.00	11430.67	10516.00
PPP1R15B	2562.00	2338.00	2806.00	2568.67	2696.00
PPP1R16A	1476.00	1482.00	1520.00	1492.67	1674.00
PPP1R18	3.00	3.00	3.00	3.00	4.00
PPP1R1C	3.00	4.00	5.00	4.00	4.00
PPP1R2	702.00	632.00	721.00	685.00	778.00
PPP1R21	413.00	438.00	487.00	446.00	498.00
PPP1R26	1392.00	1420.00	1425.00	1412.33	1236.00
PPP1R26-AS1	59.00	59.00	64.00	60.67	54.00
PPP1R2P3	24.00	15.00	18.00	19.00	24.00
PPP1R32	17.00	27.00	20.00	21.33	25.00
PPP1R35	882.00	807.00	852.00	847.00	1154.00
PPP1R36	3.00	5.00	4.00	4.00	7.00
PPP1R37	722.00	762.00	677.00	720.33	738.00
PPP1R3B	551.00	484.00	640.00	558.33	865.00
PPP1R3D	135.00	135.00	158.00	142.67	147.00
PPP1R3E	689.00	734.00	792.00	738.33	660.00
PPP1R3F	228.00	255.00	284.00	255.67	306.00

PPP1R7	717.00	638.00	705.00	686.67	877.00
PPP1R8	980.00	855.00	1031.00	955.33	1140.00
PPP1R9A	357.00	318.00	367.00	347.33	376.00
PPP1R9B	2811.00	3048.00	3251.00	3036.67	3324.00
PPP2CA	3237.00	3166.00	3422.00	3275.00	3832.00
PPP2CB	864.00	886.00	954.00	901.33	1089.00
PPP2R1A	3169.00	3033.00	3366.00	3189.33	3801.00
PPP2R1B	523.00	548.00	585.00	552.00	523.00
PPP2R2A	1430.00	1452.00	1635.00	1505.67	1933.00
PPP2R2B	10.00	16.00	15.00	13.67	10.00
PPP2R2D	2273.00	2169.00	2412.00	2284.67	2191.00
PPP2R3A	332.00	291.00	400.00	341.00	294.00
PPP2R3B	121.00	146.00	152.00	139.67	140.00
PPP2R3C	555.00	522.00	605.00	560.67	733.00
PPP2R4	2138.00	2100.00	2262.00	2166.67	2333.00
PPP2R5A	599.00	547.00	641.00	595.67	673.00
PPP2R5B	710.00	715.00	842.00	755.67	809.00
PPP2R5C	1474.00	1380.00	1630.00	1494.67	2024.00
PPP2R5D	1088.00	1050.00	1094.00	1077.33	1234.00
PPP2R5E	864.00	797.00	899.00	853.33	1033.00
PPP3CA	712.00	687.00	754.00	717.67	610.00
PPP3CB	1380.00	1421.00	1485.00	1428.67	1792.00
PPP3CB-AS1	333.00	324.00	329.00	328.67	309.00
PPP3CC	109.00	130.00	107.00	115.33	126.00
PPP3R1	1140.00	1067.00	1166.00	1124.33	1478.00
PPP4C	1583.00	1580.00	1576.00	1579.67	1967.00
PPP4R1	2183.00	2148.00	2442.00	2257.67	2439.00
PPP4R1L	340.00	318.00	362.00	340.00	255.00
PPP4R2	675.00	529.00	651.00	618.33	725.00
PPP4R3A	1711.00	1665.00	1778.00	1718.00	1875.00
PPP4R3B	1673.00	1546.00	1743.00	1654.00	1793.00
PPP5C	1018.00	1106.00	1144.00	1089.33	1368.00
PPP6C	788.00	762.00	845.00	798.33	1039.00
PPP6R1	2857.00	2824.00	3015.00	2898.67	3025.00
PPP6R2	1481.00	1398.00	1468.00	1449.00	1568.00
PPP6R3	2164.00	2034.00	2302.00	2166.67	2295.00
PPRC1	3981.00	3879.00	4146.00	4002.00	3481.00
PPT1	4030.00	3980.00	3936.00	3982.00	5070.00
PPTC7	537.00	473.00	580.00	530.00	571.00
PPWD1	1073.00	1041.00	1160.00	1091.33	1313.00
PQBP1	968.00	999.00	1006.00	991.00	1173.00
PQLC1	552.00	540.00	572.00	554.67	641.00
PQLC2	675.00	694.00	693.00	687.33	736.00
PQLC3	154.00	149.00	132.00	145.00	171.00
PRADC1	124.00	130.00	134.00	129.33	193.00
PRAF2	715.00	788.00	694.00	732.33	891.00
PRAME	7.00	4.00	3.00	4.67	1.00
PRC1	1834.00	1721.00	1761.00	1772.00	2281.00

PRC1-AS1	20.00	19.00	27.00	22.00	17.00
PRCAT47	395.00	423.00	415.00	411.00	492.00
PRCC	1329.00	1254.00	1277.00	1286.67	1628.00
PRCD	236.00	232.00	242.00	236.67	140.00
PRCP	1906.00	1770.00	1894.00	1856.67	1574.00
PRDM1	39.00	36.00	40.00	38.33	107.00
PRDM10	276.00	273.00	299.00	282.67	314.00
PRDM11	27.00	21.00	18.00	22.00	16.00
PRDM12	13.00	12.00	10.00	11.67	8.00
PRDM13	45.00	45.00	61.00	50.33	74.00
PRDM15	573.00	563.00	544.00	560.00	614.00
PRDM2	811.00	779.00	835.00	808.33	656.00
PRDM4	1735.00	1685.00	1877.00	1765.67	1675.00
PRDM5	179.00	186.00	196.00	187.00	167.00
PRDM6	10.00	15.00	26.00	17.00	17.00
PRDM8	615.00	616.00	607.00	612.67	602.00
PRDX1	5395.00	5357.00	5426.00	5392.67	7643.00
PRDX2	1466.00	1448.00	1513.00	1475.67	2067.00
PRDX3	2729.00	2613.00	2796.00	2712.67	3476.00
PRDX4	4211.00	4307.00	4141.00	4219.67	5015.00
PRDX5	4591.00	4584.00	4691.00	4622.00	6188.00
PRDX6	4073.00	4195.00	4441.00	4236.33	5659.00
PREB	910.00	891.00	874.00	891.67	973.00
PRELID1	2005.00	2021.00	2201.00	2075.67	2984.00
PRELID2	130.00	125.00	127.00	127.33	140.00
PREP	785.00	809.00	902.00	832.00	908.00
PREPL	2708.00	2445.00	2856.00	2669.67	2645.00
PREX1	3181.00	3056.00	3112.00	3116.33	3131.00
PRG2	6.00	2.00	9.00	5.67	1.00
PRICKLE1	77.00	108.00	70.00	85.00	87.00
PRICKLE2	176.00	156.00	179.00	170.33	144.00
PRICKLE3	340.00	358.00	343.00	347.00	255.00
PRICKLE4	195.00	206.00	216.00	205.67	137.00
PRIM1	220.00	239.00	267.00	242.00	342.00
PRIM2	456.00	487.00	492.00	478.33	695.00
PRIMPOL	335.00	363.00	338.00	345.33	496.00
PRKAA1	2626.00	2153.00	2762.00	2513.67	2388.00
PRKAA2	583.00	532.00	646.00	587.00	530.00
PRKAB1	511.00	497.00	575.00	527.67	528.00
PRKAB2	1457.00	1279.00	1379.00	1371.67	1268.00
PRKACA	724.00	765.00	729.00	739.33	748.00
PRKACB	1154.00	1025.00	1202.00	1127.00	1166.00
PRKAG1	1574.00	1531.00	1672.00	1592.33	1738.00
PRKAG2	2505.00	2349.00	2645.00	2499.67	2805.00
PRKAR1A	3682.00	3211.00	3756.00	3549.67	2840.00
PRKAR1B	402.00	449.00	407.00	419.33	411.00
PRKAR2A	369.00	334.00	417.00	373.33	277.00
PRKAR2A-AS1	40.00	27.00	31.00	32.67	21.00

PRKAR2B	120.00	119.00	128.00	122.33	197.00
PRKCA	5762.00	5441.00	5852.00	5685.00	6099.00
PRKCD	786.00	739.00	765.00	763.33	882.00
PRKCDBP	178.00	157.00	165.00	166.67	344.00
PRKCE	685.00	609.00	660.00	651.33	760.00
PRKCG	5.00	6.00	4.00	5.00	0.00
PRKCH	254.00	269.00	295.00	272.67	210.00
PRKCI	1436.00	1348.00	1599.00	1461.00	1680.00
PRKCQ-AS1	79.00	74.00	82.00	78.33	97.00
PRKCSH	12016.00	12505.00	11779.00	12100.00	12437.00
PRKD1	236.00	211.00	233.00	226.67	215.00
PRKD2	472.00	460.00	484.00	472.00	377.00
PRKD3	1759.00	1581.00	1862.00	1734.00	1994.00
PRKDC	15109.00	13788.00	15573.00	14823.33	14128.00
PRKG1	151.00	133.00	193.00	159.00	112.00
PRKG1-AS1	42.00	30.00	41.00	37.67	33.00
PRKG2	34.00	25.00	34.00	31.00	35.00
PRKRA	389.00	422.00	454.00	421.67	520.00
PRKRIP1	45.00	53.00	48.00	48.67	45.00
PRKRIR	1390.00	1264.00	1459.00	1371.00	1458.00
PRKX	209.00	186.00	209.00	201.33	176.00
PRKXP1	438.00	443.00	484.00	455.00	380.00
PRLR	70.00	85.00	68.00	74.33	87.00
PRMT1	3099.00	3047.00	3292.00	3146.00	4214.00
PRMT2	1697.00	1689.00	1680.00	1688.67	1575.00
PRMT3	888.00	796.00	935.00	873.00	1066.00
PRMT5	2577.00	2325.00	2776.00	2559.33	2773.00
PRMT6	640.00	636.00	762.00	679.33	777.00
PRMT7	781.00	805.00	889.00	825.00	921.00
PRMT9	260.00	261.00	292.00	271.00	276.00
PRNP	12037.00	11811.00	11433.00	11760.33	16616.00
PROB1	80.00	100.00	114.00	98.00	45.00
PROCA1	84.00	88.00	73.00	81.67	99.00
PROCR	1960.00	1914.00	1943.00	1939.00	2443.00
PROM1	4.00	1.00	3.00	2.67	3.00
PROM2	43.00	39.00	44.00	42.00	41.00
PRORSD1P	47.00	64.00	40.00	50.33	64.00
PROS1	621.00	599.00	657.00	625.67	634.00
PROSC	826.00	810.00	929.00	855.00	1104.00
PROSER1	1459.00	1237.00	1577.00	1424.33	1469.00
PROSER2	68.00	65.00	75.00	69.33	61.00
PROSER3	362.00	364.00	382.00	369.33	344.00
PROX1	9.00	8.00	10.00	9.00	5.00
PRPF18	311.00	281.00	327.00	306.33	362.00
PRPF19	3481.00	3312.00	3520.00	3437.67	4053.00
PRPF3	1486.00	1522.00	1576.00	1528.00	1672.00
PRPF31	21.00	36.00	25.00	27.33	15.00
PRPF38A	1150.00	1149.00	1296.00	1198.33	1332.00

PRPF38B	3068.00	3242.00	3325.00	3211.67	3628.00
PRPF39	1740.00	1722.00	1909.00	1790.33	1904.00
PRPF4	805.00	761.00	816.00	794.00	868.00
PRPF40A	3399.00	3093.00	3623.00	3371.67	3749.00
PRPF40B	466.00	502.00	526.00	498.00	504.00
PRPF4B	3780.00	3650.00	4160.00	3863.33	3533.00
PRPF6	3076.00	2968.00	3243.00	3095.67	3339.00
PRPF8	14.00	21.00	16.00	17.00	12.00
PRPS1	809.00	780.00	924.00	837.67	1059.00
PRPS2	369.00	361.00	411.00	380.33	463.00
PRPSAP1	627.00	581.00	639.00	615.67	752.00
PRPSAP2	674.00	555.00	693.00	640.67	731.00
PRR11	2330.00	2070.00	2351.00	2250.33	2557.00
PRR12	922.00	928.00	966.00	938.67	875.00
PRR13	942.00	873.00	922.00	912.33	1268.00
PRR14	753.00	770.00	869.00	797.33	899.00
PRR14L	1521.00	1515.00	1686.00	1574.00	1477.00
PRR16	207.00	154.00	195.00	185.33	163.00
PRR19	11.00	14.00	24.00	16.33	18.00
PRR22	172.00	186.00	212.00	190.00	242.00
PRR29	4.00	9.00	6.00	6.33	11.00
PRR34	5.00	3.00	4.00	4.00	9.00
PRR34-AS1	63.00	63.00	57.00	61.00	83.00
PRR5L	41.00	32.00	34.00	35.67	57.00
PRR7	240.00	229.00	280.00	249.67	317.00
PRR7-AS1	132.00	121.00	103.00	118.67	122.00
PRRC1	1345.00	1245.00	1428.00	1339.33	1447.00
PRRC2A	24.00	16.00	17.00	19.00	19.00
PRRC2B	10053.00	9416.00	10540.00	10003.00	9027.00
PRRC2C	10074.00	8801.00	10441.00	9772.00	7480.00
PRRG1	245.00	194.00	247.00	228.67	232.00
PRRG2	9.00	9.00	7.00	8.33	9.00
PRRG4	9.00	1.00	6.00	5.33	11.00
PRRT2	11.00	15.00	17.00	14.33	13.00
PRRT3	75.00	83.00	54.00	70.67	51.00
PRRT3-AS1	61.00	66.00	54.00	60.33	64.00
PRRX1	1375.00	1239.00	1539.00	1384.33	1289.00
PRSS12	1220.00	1194.00	1281.00	1231.67	1261.00
PRSS23	6088.00	5836.00	6018.00	5980.67	4407.00
PRSS27	24.00	16.00	21.00	20.33	20.00
PRSS3	736.00	685.00	807.00	742.67	859.00
PRSS35	5.00	3.00	5.00	4.33	6.00
PRSS36	6.00	8.00	3.00	5.67	3.00
PRSS53	470.00	504.00	517.00	497.00	472.00
PRTFDC1	3.00	4.00	3.00	3.33	2.00
PRTG	704.00	703.00	801.00	736.00	684.00
PRUNE	235.00	210.00	249.00	231.33	218.00
PRUNE2	11.00	12.00	7.00	10.00	10.00

PRX	121.00	114.00	141.00	125.33	142.00
PSAP	34245.00	35506.00	34985.00	34912.00	29818.00
PSAT1	7383.00	6540.00	7421.00	7114.67	9158.00
PSCA	22.00	15.00	36.00	24.33	27.00
PSD	22.00	12.00	23.00	19.00	18.00
PSD3	824.00	755.00	793.00	790.67	455.00
PSD4	848.00	808.00	916.00	857.33	754.00
PSEN1	1536.00	1486.00	1624.00	1548.67	1593.00
PSEN2	1076.00	1087.00	1136.00	1099.67	1273.00
PSENEN	807.00	846.00	745.00	799.33	1033.00
PSG1	30.00	35.00	54.00	39.67	36.00
PSG11	3.00	9.00	5.00	5.67	29.00
PSG2	6.00	5.00	4.00	5.00	7.00
PSG4	2456.00	2363.00	2520.00	2446.33	2340.00
PSG5	102.00	99.00	105.00	102.00	116.00
PSG6	29.00	17.00	29.00	25.00	27.00
PSG7	7.00	13.00	8.00	9.33	32.00
PSG9	33.00	26.00	27.00	28.67	45.00
PSIP1	2041.00	1919.00	2264.00	2074.67	2820.00
PSKH1	549.00	500.00	534.00	527.67	660.00
PSMA1	3122.00	3067.00	3330.00	3173.00	4378.00
PSMA2	1949.00	1862.00	2053.00	1954.67	2783.00
PSMA3	1250.00	1285.00	1268.00	1267.67	1659.00
PSMA3-AS1	1436.00	1437.00	1482.00	1451.67	1648.00
PSMA4	2067.00	1889.00	2086.00	2014.00	2682.00
PSMA5	1997.00	1937.00	1993.00	1975.67	2575.00
PSMA6	1825.00	1730.00	1803.00	1786.00	2174.00
PSMA7	4480.00	4487.00	4670.00	4545.67	5805.00
PSMB1	2018.00	1945.00	2097.00	2020.00	2803.00
PSMB10	274.00	254.00	281.00	269.67	320.00
PSMB2	1751.00	1617.00	1802.00	1723.33	2258.00
PSMB4	3634.00	3548.00	3688.00	3623.33	5077.00
PSMB5	2257.00	2289.00	2331.00	2292.33	3019.00
PSMB6	2096.00	2115.00	2064.00	2091.67	2938.00
PSMB7	1871.00	1876.00	1981.00	1909.33	2520.00
PSMC1	1116.00	1085.00	1207.00	1136.00	1482.00
PSMC2	1550.00	1513.00	1591.00	1551.33	2115.00
PSMC3	1975.00	1884.00	1971.00	1943.33	2480.00
PSMC3IP	306.00	339.00	301.00	315.33	538.00
PSMC4	1608.00	1575.00	1768.00	1650.33	2147.00
PSMC5	3214.00	3127.00	3395.00	3245.33	4458.00
PSMC6	1461.00	1373.00	1449.00	1427.67	1681.00
PSMD1	2433.00	2319.00	2564.00	2438.67	2647.00
PSMD10	1246.00	1067.00	1121.00	1144.67	1412.00
PSMD11	1904.00	1828.00	1895.00	1875.67	2267.00
PSMD12	1525.00	1343.00	1485.00	1451.00	1704.00
PSMD13	1912.00	1947.00	1929.00	1929.33	2623.00
PSMD14	2459.00	2441.00	2633.00	2511.00	3457.00

PSMD2	13390.00	12925.00	13843.00	13386.00	12614.00
PSMD3	2449.00	2486.00	2573.00	2502.67	3231.00
PSMD4	2507.00	2342.00	2616.00	2488.33	3414.00
PSMD5	748.00	723.00	808.00	759.67	938.00
PSMD5-AS1	703.00	668.00	635.00	668.67	736.00
PSMD6	1415.00	1242.00	1455.00	1370.67	1802.00
PSMD6-AS2	129.00	102.00	138.00	123.00	90.00
PSMD7	1767.00	1797.00	1894.00	1819.33	2438.00
PSMD8	1773.00	1730.00	1938.00	1813.67	2369.00
PSMD9	871.00	807.00	923.00	867.00	1209.00
PSME1	1016.00	1020.00	1129.00	1055.00	1235.00
PSME2	1035.00	988.00	1045.00	1022.67	1325.00
PSME3	2443.00	2179.00	2488.00	2370.00	2572.00
PSME4	2415.00	2335.00	2826.00	2525.33	2417.00
PSMF1	1964.00	1901.00	1956.00	1940.33	2187.00
PSMG1	762.00	716.00	782.00	753.33	980.00
PSMG2	1075.00	1023.00	1197.00	1098.33	1367.00
PSMG3	1505.00	1455.00	1619.00	1526.33	1931.00
PSMG3-AS1	148.00	151.00	174.00	157.67	130.00
PSMG4	293.00	328.00	342.00	321.00	418.00
PSPC1	659.00	656.00	629.00	648.00	737.00
PSPH	1077.00	1044.00	1053.00	1058.00	1459.00
PSPN	17.00	19.00	15.00	17.00	13.00
PSRC1	628.00	669.00	664.00	653.67	826.00
PSTK	128.00	139.00	127.00	131.33	138.00
PSTPIP2	143.00	114.00	149.00	135.33	184.00
PTAFR	4.00	3.00	12.00	6.33	2.00
PTAR1	1532.00	1344.00	1641.00	1505.67	1234.00
PTBP1	7521.00	6933.00	7602.00	7352.00	8475.00
PTBP2	739.00	692.00	840.00	757.00	887.00
PTBP3	1251.00	1033.00	1367.00	1217.00	1240.00
PTCD1	19.00	14.00	19.00	17.33	9.00
PTCD2	361.00	309.00	361.00	343.67	311.00
PTCD3	2421.00	2325.00	2669.00	2471.67	2645.00
PTCH1	144.00	141.00	171.00	152.00	128.00
PTCH2	9.00	27.00	13.00	16.33	8.00
PTDSS1	3763.00	3834.00	3718.00	3771.67	3851.00
PTDSS2	188.00	162.00	214.00	188.00	175.00
PTEN	1199.00	1051.00	1336.00	1195.33	1450.00
PTENP1	3.00	8.00	10.00	7.00	11.00
PTENP1-AS	7.00	3.00	3.00	4.33	6.00
PTER	578.00	557.00	601.00	578.67	696.00
PTGER1	23.00	22.00	20.00	21.67	23.00
PTGER2	111.00	104.00	121.00	112.00	66.00
PTGER3	8.00	5.00	11.00	8.00	5.00
PTGER4	188.00	156.00	140.00	161.33	236.00
PTGES2	1375.00	1351.00	1514.00	1413.33	1544.00
PTGES2-AS1	8.00	20.00	9.00	12.33	9.00

PTGES3	6096.00	5810.00	6060.00	5988.67	8294.00
PTGFR	1266.00	1124.00	1266.00	1218.67	1032.00
PTGFRN	2468.00	2355.00	2407.00	2410.00	2196.00
PTGIR	19.00	15.00	14.00	16.00	15.00
PTGR1	848.00	919.00	950.00	905.67	1050.00
PTGR2	498.00	474.00	490.00	487.33	501.00
PTGS1	765.00	781.00	714.00	753.33	209.00
PTGS2	465.00	454.00	441.00	453.33	477.00
PTH2R	3.00	0.00	1.00	1.33	0.00
PTHLH	65.00	54.00	63.00	60.67	45.00
PTK2	3550.00	3407.00	3873.00	3610.00	3401.00
PTK2B	70.00	71.00	72.00	71.00	69.00
PTK6	8.00	8.00	9.00	8.33	7.00
PTK7	484.00	551.00	509.00	514.67	444.00
PTMA	9008.00	9123.00	9528.00	9219.67	14112.00
PTMS	1455.00	1543.00	1473.00	1490.33	1891.00
PTOV1	1209.00	1275.00	1363.00	1282.33	1391.00
PTOV1-AS1	7.00	9.00	11.00	9.00	7.00
PTOV1-AS2	265.00	319.00	298.00	294.00	303.00
PTP4A1	1938.00	1772.00	2087.00	1932.33	2565.00
PTP4A2	4515.00	4124.00	4854.00	4497.67	5941.00
PTP4A3	7.00	16.00	16.00	13.00	9.00
PTPDC1	1118.00	999.00	1095.00	1070.67	1062.00
PTPMT1	645.00	671.00	622.00	646.00	777.00
PTPN1	2339.00	2160.00	2346.00	2281.67	2310.00
PTPN11	4085.00	3683.00	4290.00	4019.33	4478.00
PTPN12	2627.00	2317.00	2781.00	2575.00	3216.00
PTPN13	898.00	777.00	938.00	871.00	833.00
PTPN14	4892.00	4296.00	5194.00	4794.00	3942.00
PTPN18	1411.00	1511.00	1536.00	1486.00	1519.00
PTPN2	1172.00	1041.00	1246.00	1153.00	1534.00
PTPN20	7.00	4.00	5.00	5.33	8.00
PTPN21	521.00	512.00	579.00	537.33	548.00
PTPN22	441.00	453.00	486.00	460.00	542.00
PTPN23	2158.00	2098.00	2223.00	2159.67	1694.00
PTPN3	308.00	331.00	359.00	332.67	311.00
PTPN4	295.00	291.00	341.00	309.00	383.00
PTPN7	17.00	8.00	15.00	13.33	32.00
PTPN9	1103.00	1024.00	1228.00	1118.33	1129.00
PTPRA	1944.00	1881.00	1981.00	1935.33	2247.00
PTPRB	523.00	523.00	610.00	552.00	525.00
PTPRCAP	68.00	59.00	76.00	67.67	61.00
PTPRD	164.00	140.00	172.00	158.67	122.00
PTPRE	670.00	673.00	652.00	665.00	575.00
PTPRF	17001.00	16229.00	17306.00	16845.33	15142.00
PTPRG	1607.00	1374.00	1616.00	1532.33	1361.00
PTPRG-AS1	50.00	40.00	60.00	50.00	67.00
PTPRH	103.00	99.00	132.00	111.33	145.00

PTPRJ	789.00	896.00	825.00	836.67	717.00
PTPRK	6.00	6.00	2.00	4.67	3.00
PTPRM	964.00	915.00	1012.00	963.67	736.00
PTPRN	3.00	1.00	0.00	1.33	0.00
PTPRN2	305.00	281.00	294.00	293.33	318.00
PTPRO	414.00	362.00	409.00	395.00	302.00
PTPRQ	4.00	2.00	3.00	3.00	3.00
PTPRR	18.00	18.00	20.00	18.67	16.00
PTPRS	2111.00	2051.00	2282.00	2148.00	1864.00
PTPRU	4.00	1.00	1.00	2.00	1.00
PTPRZ1	102.00	96.00	81.00	93.00	246.00
PTRF	3687.00	3516.00	3710.00	3637.67	4580.00
PTRH1	152.00	180.00	147.00	159.67	172.00
PTRH2	1135.00	1140.00	1079.00	1118.00	1383.00
PTRHD1	170.00	134.00	162.00	155.33	229.00
PTS	372.00	362.00	370.00	368.00	497.00
PTTG1	1129.00	1046.00	1036.00	1070.33	1575.00
PTTG1IP	13974.00	13442.00	13149.00	13521.67	16638.00
PTTG3P	7.00	4.00	4.00	5.00	9.00
PTX3	35851.00	36057.00	34630.00	35512.67	45384.00
PUF60	76.00	64.00	56.00	65.33	108.00
PUM1	2205.00	2093.00	2304.00	2200.67	2154.00
PUM2	2676.00	2454.00	2944.00	2691.33	3014.00
PURA	493.00	434.00	545.00	490.67	474.00
PURB	2734.00	2523.00	2960.00	2739.00	2626.00
PURG	20.00	18.00	31.00	23.00	35.00
PUS1	849.00	932.00	997.00	926.00	1117.00
PUS10	169.00	140.00	159.00	156.00	137.00
PUS3	144.00	126.00	167.00	145.67	212.00
PUS7	1050.00	1017.00	1117.00	1061.33	1176.00
PUS7L	424.00	402.00	448.00	424.67	542.00
PUSL1	142.00	117.00	172.00	143.67	174.00
PVR	9265.00	9129.00	9374.00	9256.00	5842.00
PVRIG	5.00	4.00	2.00	3.67	2.00
PVRL1	733.00	747.00	727.00	735.67	889.00
PVRL2	2477.00	2549.00	2343.00	2456.33	2305.00
PVRL3	977.00	855.00	974.00	935.33	1025.00
PVRL3-AS1	5.00	2.00	2.00	3.00	15.00
PVRL4	3.00	6.00	3.00	4.00	1.00
PVT1	1347.00	1226.00	1387.00	1320.00	1402.00
PWAR1	8.00	7.00	9.00	8.00	4.00
PWAR5	71.00	58.00	60.00	63.00	52.00
PWARSN	21.00	17.00	21.00	19.67	17.00
PWP1	1553.00	1409.00	1635.00	1532.33	1745.00
PWP2	318.00	274.00	325.00	305.67	377.00
PWWP2A	897.00	864.00	1022.00	927.67	1485.00
PWWP2B	309.00	336.00	396.00	347.00	340.00
PXDC1	1085.00	1095.00	1213.00	1131.00	1221.00

PXDN	7453.00	7252.00	7474.00	7393.00	6092.00
PXDNL	4.00	6.00	9.00	6.33	5.00
PXK	943.00	831.00	887.00	887.00	681.00
PXMP2	171.00	177.00	202.00	183.33	255.00
PXMP4	204.00	204.00	201.00	203.00	193.00
PXN	9075.00	8847.00	9442.00	9121.33	8735.00
PXN-AS1	28.00	23.00	26.00	25.67	41.00
PXYLP1	200.00	180.00	225.00	201.67	231.00
PYCARD-AS1	3.00	2.00	0.00	1.67	0.00
PYCR1	4941.00	4762.00	5231.00	4978.00	5166.00
PYCR2	2502.00	2577.00	2628.00	2569.00	3380.00
PYGB	4864.00	4783.00	5286.00	4977.67	4545.00
PYGL	95.00	98.00	109.00	100.67	200.00
PYGM	11.00	23.00	17.00	17.00	12.00
PYGO1	338.00	316.00	410.00	354.67	340.00
PYGO2	1184.00	1090.00	1219.00	1164.33	1317.00
PYROXD1	469.00	435.00	560.00	488.00	646.00
PYROXD2	454.00	487.00	470.00	470.33	441.00
PYY	18.00	16.00	21.00	18.33	30.00
PYY2	6.00	11.00	4.00	7.00	1.00
QARS	4002.00	4089.00	4274.00	4121.67	4358.00
QDPR	364.00	375.00	348.00	362.33	474.00
QKI	3013.00	2678.00	3281.00	2990.67	2783.00
QPCT	893.00	902.00	946.00	913.67	863.00
QPCTL	639.00	629.00	671.00	646.33	625.00
QPRT	174.00	142.00	171.00	162.33	224.00
QRICH1	1976.00	1945.00	2010.00	1977.00	2090.00
QRICH2	217.00	223.00	262.00	234.00	185.00
QRSL1	554.00	536.00	575.00	555.00	625.00
QSER1	1737.00	1570.00	1797.00	1701.33	1917.00
QSOX1	39887.00	39340.00	38776.00	39334.33	29417.00
QSOX2	3662.00	3820.00	3846.00	3776.00	3468.00
QTRT1	1497.00	1643.00	1651.00	1597.00	1528.00
QTRTD1	1384.00	1201.00	1339.00	1308.00	1540.00
R3HCC1	233.00	222.00	271.00	242.00	351.00
R3HCC1L	536.00	567.00	556.00	553.00	607.00
R3HDM1	1144.00	1121.00	1281.00	1182.00	1186.00
R3HDM2	2420.00	2420.00	2798.00	2546.00	2493.00
R3HDM4	1140.00	1233.00	1270.00	1214.33	1323.00
RAB10	2613.00	2325.00	2882.00	2606.67	2804.00
RAB11A	1143.00	1032.00	1167.00	1114.00	1445.00
RAB11B	928.00	962.00	978.00	956.00	1269.00
RAB11B-AS1	26.00	27.00	39.00	30.67	41.00
RAB11FIP1	277.00	256.00	271.00	268.00	293.00
RAB11FIP2	831.00	812.00	1020.00	887.67	895.00
RAB11FIP3	16.00	22.00	12.00	16.67	30.00
RAB11FIP4	4.00	7.00	10.00	7.00	7.00
RAB11FIP5	1637.00	1555.00	1792.00	1661.33	1441.00

RAB12	1149.00	1044.00	1257.00	1150.00	1361.00
RAB13	2948.00	2994.00	3302.00	3081.33	2991.00
RAB14	1605.00	1307.00	1665.00	1525.67	1802.00
RAB15	199.00	189.00	214.00	200.67	245.00
RAB18	1292.00	1122.00	1379.00	1264.33	1536.00
RAB1A	1866.00	1748.00	2080.00	1898.00	2393.00
RAB1B	3526.00	3428.00	3718.00	3557.33	4028.00
RAB20	18.00	19.00	27.00	21.33	25.00
RAB21	635.00	629.00	686.00	650.00	721.00
RAB22A	1363.00	1265.00	1379.00	1335.67	1314.00
RAB23	684.00	579.00	737.00	666.67	647.00
RAB24	1328.00	1300.00	1392.00	1340.00	1509.00
RAB26	32.00	37.00	46.00	38.33	34.00
RAB27A	176.00	127.00	161.00	154.67	172.00
RAB27B	149.00	136.00	156.00	147.00	207.00
RAB28	295.00	294.00	328.00	305.67	328.00
RAB29	1200.00	1204.00	1239.00	1214.33	1240.00
RAB2A	1827.00	1659.00	2021.00	1835.67	2233.00
RAB2B	440.00	420.00	495.00	451.67	509.00
RAB30	29.00	27.00	31.00	29.00	60.00
RAB30-AS1	58.00	63.00	76.00	65.67	77.00
RAB31	1296.00	1114.00	1411.00	1273.67	1349.00
RAB32	868.00	780.00	908.00	852.00	1071.00
RAB33B	127.00	118.00	168.00	137.67	161.00
RAB34	1642.00	1640.00	1821.00	1701.00	1935.00
RAB35	1349.00	1248.00	1303.00	1300.00	1526.00
RAB36	128.00	95.00	82.00	101.67	80.00
RAB38	228.00	202.00	248.00	226.00	237.00
RAB39A	18.00	20.00	20.00	19.33	22.00
RAB3A	16.00	23.00	20.00	19.67	35.00
RAB3B	6810.00	6231.00	6979.00	6673.33	7781.00
RAB3C	8.00	7.00	9.00	8.00	7.00
RAB3D	43.00	38.00	34.00	38.33	60.00
RAB3GAP1	1835.00	1771.00	1945.00	1850.33	1960.00
RAB3GAP2	1755.00	1576.00	1821.00	1717.33	1672.00
RAB3IL1	10.00	9.00	5.00	8.00	12.00
RAB3IP	594.00	541.00	683.00	606.00	775.00
RAB40A	8.00	5.00	14.00	9.00	7.00
RAB40AL	6.00	2.00	2.00	3.33	5.00
RAB40B	178.00	153.00	163.00	164.67	197.00
RAB40C	519.00	486.00	508.00	504.33	465.00
RAB43	35.00	43.00	61.00	46.33	20.00
RAB4A	340.00	280.00	392.00	337.33	514.00
RAB5A	1153.00	963.00	1158.00	1091.33	1268.00
RAB5B	1405.00	1257.00	1452.00	1371.33	1326.00
RAB5C	1845.00	1784.00	2044.00	1891.00	2091.00
RAB6A	1828.00	1644.00	2000.00	1824.00	1904.00
RAB6B	49.00	44.00	43.00	45.33	78.00

RAB7A	5507.00	5211.00	5701.00	5473.00	6588.00
RAB8A	815.00	780.00	809.00	801.33	978.00
RAB8B	858.00	718.00	957.00	844.33	641.00
RAB9A	565.00	600.00	607.00	590.67	625.00
RAB9B	14.00	20.00	7.00	13.67	36.00
RABAC1	764.00	779.00	731.00	758.00	745.00
RABEP1	2404.00	2213.00	2605.00	2407.33	2844.00
RABEP2	606.00	597.00	634.00	612.33	751.00
RABEPK	681.00	698.00	819.00	732.67	943.00
RABGAP1	1291.00	1183.00	1322.00	1265.33	1192.00
RABGAP1L	440.00	369.00	469.00	426.00	527.00
RABGEF1	1218.00	1110.00	1208.00	1178.67	1353.00
RABGGTA	482.00	493.00	522.00	499.00	531.00
RABGGTB	6672.00	6421.00	6845.00	6646.00	8030.00
RABIF	202.00	177.00	189.00	189.33	224.00
RABL2A	98.00	87.00	94.00	93.00	82.00
RABL2B	417.00	440.00	486.00	447.67	486.00
RABL3	522.00	471.00	608.00	533.67	565.00
RABL6	2331.00	2346.00	2512.00	2396.33	2818.00
RAC1	5122.00	4718.00	5557.00	5132.33	6602.00
RAC2	1681.00	1649.00	1721.00	1683.67	1798.00
RAC3	748.00	804.00	841.00	797.67	1048.00
RACGAP1	1213.00	1062.00	1205.00	1160.00	1502.00
RAD1	730.00	644.00	684.00	686.00	805.00
RAD17	60.00	65.00	70.00	65.00	69.00
RAD18	847.00	840.00	885.00	857.33	863.00
RAD21	5613.00	5242.00	6211.00	5688.67	6731.00
RAD21-AS1	9.00	5.00	10.00	8.00	8.00
RAD21L1	17.00	12.00	15.00	14.67	29.00
RAD23A	2481.00	2417.00	2477.00	2458.33	3107.00
RAD23B	2562.00	2341.00	2756.00	2553.00	3146.00
RAD50	2242.00	2332.00	2377.00	2317.00	2210.00
RAD51	115.00	102.00	92.00	103.00	189.00
RAD51AP1	401.00	384.00	357.00	380.67	392.00
RAD51-AS1	138.00	142.00	184.00	154.67	56.00
RAD51B	219.00	221.00	198.00	212.67	241.00
RAD51C	546.00	605.00	660.00	603.67	813.00
RAD51D	266.00	255.00	240.00	253.67	285.00
RAD52	1037.00	1054.00	1071.00	1054.00	1028.00
RAD54B	271.00	277.00	301.00	283.00	379.00
RAD54L	363.00	362.00	363.00	362.67	426.00
RAD54L2	547.00	542.00	650.00	579.67	328.00
RAD9A	280.00	336.00	282.00	299.33	305.00
RAD9B	12.00	14.00	16.00	14.00	21.00
RAE1	1332.00	1324.00	1471.00	1375.67	1731.00
RAET1E	31.00	22.00	17.00	23.33	18.00
RAET1G	11.00	3.00	9.00	7.67	9.00
RAET1K	27.00	28.00	26.00	27.00	37.00

RAF1	1911.00	1793.00	1927.00	1877.00	1953.00
RAG1	24.00	12.00	22.00	19.33	13.00
RAI1	1798.00	1690.00	1868.00	1785.33	1391.00
RAI14	8129.00	7168.00	8571.00	7956.00	8355.00
RALA	1236.00	1177.00	1368.00	1260.33	1472.00
RALB	990.00	862.00	972.00	941.33	1152.00
RALBP1	2220.00	2029.00	2429.00	2226.00	2373.00
RALGAPA1	359.00	365.00	413.00	379.00	343.00
RALGAPA1P	25.00	21.00	33.00	26.33	27.00
RALGAPA2	546.00	508.00	619.00	557.67	551.00
RALGAPB	2763.00	2650.00	2949.00	2787.33	2629.00
RALGDS	708.00	802.00	876.00	795.33	978.00
RALGPS1	53.00	64.00	74.00	63.67	62.00
RALGPS2	925.00	830.00	975.00	910.00	654.00
RALY	2778.00	2599.00	2883.00	2753.33	3413.00
RALY-AS1	12.00	10.00	9.00	10.33	7.00
RAMP2	5.00	5.00	7.00	5.67	2.00
RAMP2-AS1	11.00	4.00	10.00	8.33	7.00
RAN	5751.00	5533.00	5911.00	5731.67	7961.00
RANBP1	1372.00	1305.00	1354.00	1343.67	1802.00
RANBP10	746.00	734.00	698.00	726.00	725.00
RANBP17	369.00	334.00	379.00	360.67	346.00
RANBP2	4231.00	3838.00	4357.00	4142.00	4066.00
RANBP3	1055.00	1125.00	1097.00	1092.33	1236.00
RANBP6	1638.00	1529.00	1803.00	1656.67	1658.00
RANBP9	945.00	977.00	1003.00	975.00	1117.00
RANGAP1	5172.00	5199.00	5273.00	5214.67	5680.00
RANGRF	1025.00	1025.00	1133.00	1061.00	1185.00
RAP1A	610.00	533.00	636.00	593.00	783.00
RAP1B	1358.00	1200.00	1300.00	1286.00	1570.00
RAP1GAP2	4859.00	4644.00	5335.00	4946.00	5907.00
RAP1GDS1	960.00	892.00	1031.00	961.00	1078.00
RAP2A	445.00	390.00	482.00	439.00	516.00
RAP2B	1972.00	1818.00	2090.00	1960.00	3600.00
RAP2C	444.00	437.00	506.00	462.33	566.00
RAP2C-AS1	31.00	29.00	31.00	30.33	11.00
RAPGEF1	1305.00	1317.00	1436.00	1352.67	1490.00
RAPGEF2	844.00	783.00	892.00	839.67	545.00
RAPGEF3	389.00	389.00	412.00	396.67	387.00
RAPGEF4	12.00	3.00	10.00	8.33	7.00
RAPGEF5	147.00	165.00	170.00	160.67	223.00
RAPGEF6	705.00	611.00	716.00	677.33	576.00
RAPGEFL1	178.00	191.00	206.00	191.67	218.00
RAPH1	2517.00	2190.00	2485.00	2397.33	2890.00
RAPSN	8.00	15.00	17.00	13.33	3.00
RARA	843.00	845.00	924.00	870.67	676.00
RARA-AS1	19.00	29.00	26.00	24.67	46.00
RARB	124.00	90.00	116.00	110.00	86.00

RARG	291.00	288.00	311.00	296.67	249.00
RARRES1	52.00	47.00	39.00	46.00	92.00
RARRES3	6.00	6.00	6.00	6.00	10.00
RARS	2469.00	2299.00	2582.00	2450.00	2884.00
RARS2	680.00	665.00	693.00	679.33	795.00
RASA1	2605.00	2389.00	2871.00	2621.67	3058.00
RASA2	1291.00	1243.00	1426.00	1320.00	1676.00
RASA3	1610.00	1503.00	1757.00	1623.33	1387.00
RASA4B	4.00	5.00	4.00	4.33	1.00
RASA4CP	8.00	11.00	4.00	7.67	9.00
RASAL2	1723.00	1666.00	1785.00	1724.67	1732.00
RASAL2-AS1	47.00	53.00	42.00	47.33	59.00
RASAL3	11.00	9.00	5.00	8.33	5.00
RASD2	6.00	3.00	4.00	4.33	5.00
RASEF	7.00	3.00	3.00	4.33	5.00
RASGRF1	693.00	766.00	762.00	740.33	663.00
RASGRF2	321.00	321.00	389.00	343.67	211.00
RASGRP3	8.00	7.00	6.00	7.00	8.00
RASL10B	55.00	46.00	59.00	53.33	60.00
RASL11A	8.00	4.00	8.00	6.67	11.00
RASL11B	8.00	2.00	4.00	4.67	6.00
RASSF1	647.00	628.00	658.00	644.33	786.00
RASSF1-AS1	5.00	3.00	3.00	3.67	2.00
RASSF3	836.00	786.00	864.00	828.67	802.00
RASSF4	379.00	371.00	391.00	380.33	501.00
RASSF5	7.00	2.00	3.00	4.00	3.00
RASSF7	4.00	6.00	7.00	5.67	13.00
RASSF8	2122.00	1693.00	2186.00	2000.33	1678.00
RASSF8-AS1	135.00	101.00	130.00	122.00	131.00
RAVER1	1757.00	1624.00	1796.00	1725.67	1774.00
RAVER2	596.00	552.00	765.00	637.67	640.00
RB1	770.00	640.00	730.00	713.33	799.00
RB1CC1	2317.00	2142.00	2574.00	2344.33	2308.00
RBAK	874.00	794.00	923.00	863.67	804.00
RBBP4	2461.00	2449.00	2711.00	2540.33	3113.00
RBBP5	545.00	570.00	622.00	579.00	679.00
RBBP6	2624.00	2610.00	3023.00	2752.33	2659.00
RBBP7	3567.00	3413.00	3669.00	3549.67	4181.00
RBBP8	991.00	874.00	1066.00	977.00	1306.00
RBBP9	380.00	368.00	386.00	378.00	475.00
RBCK1	3242.00	3347.00	3503.00	3364.00	2594.00
RBFA	537.00	566.00	637.00	580.00	564.00
RBFADN	14.00	26.00	26.00	22.00	15.00
RBFOX1	4.00	3.00	4.00	3.67	2.00
RBFOX2	102.00	80.00	106.00	96.00	86.00
RBKS	68.00	58.00	67.00	64.33	97.00
RBL1	477.00	439.00	488.00	468.00	598.00
RBL2	3023.00	2760.00	3106.00	2963.00	2774.00

RBM10	1934.00	1753.00	1835.00	1840.67	2037.00
RBM12	2460.00	2286.00	2615.00	2453.67	2604.00
RBM12B	983.00	922.00	947.00	950.67	869.00
RBM14	1115.00	1022.00	1036.00	1057.67	1321.00
RBM15	492.00	535.00	575.00	534.00	592.00
RBM15B	1706.00	1665.00	1932.00	1767.67	1825.00
RBM17	2241.00	2317.00	2343.00	2300.33	2645.00
RBM18	492.00	457.00	509.00	486.00	569.00
RBM19	1750.00	1823.00	1924.00	1832.33	1801.00
RBM20	9.00	12.00	18.00	13.00	17.00
RBM22	1311.00	1302.00	1476.00	1363.00	1416.00
RBM23	927.00	892.00	947.00	922.00	884.00
RBM24	29.00	28.00	16.00	24.33	18.00
RBM25	4600.00	4455.00	4790.00	4615.00	4884.00
RBM26	1759.00	1649.00	1842.00	1750.00	1839.00
RBM26-AS1	97.00	110.00	106.00	104.33	114.00
RBM27	859.00	736.00	915.00	836.67	969.00
RBM28	1574.00	1504.00	1663.00	1580.33	1792.00
RBM3	2015.00	1993.00	2103.00	2037.00	2850.00
RBM33	3018.00	3115.00	3367.00	3166.67	2473.00
RBM34	796.00	728.00	742.00	755.33	913.00
RBM38	393.00	400.00	384.00	392.33	470.00
RBM39	11567.00	11997.00	12963.00	12175.67	12440.00
RBM4	1518.00	1578.00	1608.00	1568.00	1654.00
RBM41	844.00	712.00	742.00	766.00	797.00
RBM42	1108.00	1116.00	1125.00	1116.33	1354.00
RBM43	128.00	103.00	172.00	134.33	182.00
RBM44	35.00	39.00	33.00	35.67	25.00
RBM45	229.00	232.00	245.00	235.33	301.00
RBM47	10.00	2.00	13.00	8.33	4.00
RBM48	545.00	479.00	551.00	525.00	540.00
RBM4B	601.00	592.00	706.00	633.00	677.00
RBM5	2833.00	2977.00	3038.00	2949.33	2929.00
RBM6	2153.00	2298.00	2478.00	2309.67	2359.00
RBM7	506.00	373.00	471.00	450.00	484.00
RBM8A	1672.00	1669.00	1660.00	1667.00	2153.00
RBMS1	1586.00	1377.00	1757.00	1573.33	1803.00
RBMS2	752.00	750.00	805.00	769.00	637.00
RBMS3	412.00	389.00	502.00	434.33	431.00
RBMS3-AS3	29.00	14.00	27.00	23.33	25.00
RBMX	5567.00	5323.00	5703.00	5531.00	6667.00
RBMX2	345.00	321.00	333.00	333.00	389.00
RBMXL1	519.00	483.00	546.00	516.00	631.00
RBP4	5.00	2.00	2.00	3.00	5.00
RBPJ	1076.00	1041.00	1136.00	1084.33	935.00
RBPMS	114.00	92.00	129.00	111.67	122.00
RBSN	996.00	942.00	1084.00	1007.33	946.00
RBX1	438.00	384.00	393.00	405.00	566.00

RC3H1	727.00	624.00	695.00	682.00	690.00
RC3H2	317.00	301.00	353.00	323.67	286.00
RCAN1	1438.00	1280.00	1490.00	1402.67	2252.00
RCAN2	10.00	13.00	6.00	9.67	13.00
RCAN3	35.00	22.00	26.00	27.67	14.00
RCBTB1	862.00	765.00	844.00	823.67	881.00
RCBTB2	140.00	154.00	151.00	148.33	126.00
RCC1	2149.00	2029.00	2200.00	2126.00	2523.00
RCC2	2197.00	2222.00	2205.00	2208.00	2842.00
RCCD1	646.00	664.00	714.00	674.67	748.00
RCE1	898.00	1010.00	875.00	927.67	1037.00
RCHY1	275.00	216.00	306.00	265.67	298.00
RCL1	587.00	571.00	659.00	605.67	796.00
RCN1	9555.00	9400.00	9312.00	9422.33	10236.00
RCN2	2594.00	2462.00	2554.00	2536.67	2910.00
RCN3	6023.00	6379.00	5805.00	6069.00	5546.00
RCOR1	622.00	597.00	653.00	624.00	628.00
RCOR2	32.00	23.00	23.00	26.00	29.00
RCOR3	890.00	894.00	1017.00	933.67	898.00
RDH10	366.00	358.00	438.00	387.33	443.00
RDH11	4229.00	4027.00	3926.00	4060.67	4898.00
RDH12	5.00	5.00	6.00	5.33	8.00
RDH14	62.00	59.00	57.00	59.33	76.00
RDH16	5.00	10.00	20.00	11.67	18.00
RDH5	35.00	42.00	43.00	40.00	16.00
RDX	2843.00	2545.00	3077.00	2821.67	3207.00
REC8	54.00	80.00	72.00	68.67	87.00
RECK	267.00	251.00	268.00	262.00	242.00
RECQL	2676.00	2283.00	2827.00	2595.33	3173.00
RECQL4	1166.00	1109.00	1022.00	1099.00	1412.00
RECQL5	1354.00	1493.00	1444.00	1430.33	1428.00
REEP1	4.00	1.00	2.00	2.33	3.00
REEP2	70.00	65.00	80.00	71.67	87.00
REEP3	921.00	790.00	963.00	891.33	1056.00
REEP4	991.00	948.00	898.00	945.67	1134.00
REEP5	3034.00	3126.00	2896.00	3018.67	3281.00
REEP6	68.00	93.00	87.00	82.67	90.00
REL	99.00	82.00	105.00	95.33	67.00
RELA	2628.00	2410.00	2588.00	2542.00	2906.00
RELB	444.00	441.00	471.00	452.00	838.00
RELL1	235.00	292.00	225.00	250.67	320.00
RELL2	198.00	227.00	215.00	213.33	166.00
RELN	116.00	95.00	117.00	109.33	40.00
RELT	623.00	585.00	587.00	598.33	490.00
REM2	13.00	23.00	15.00	17.00	13.00
RENBP	52.00	62.00	61.00	58.33	43.00
REP15	40.00	47.00	47.00	44.67	31.00
REPIN1	3369.00	3414.00	3569.00	3450.67	3933.00

REPS1	1483.00	1445.00	1627.00	1518.33	1493.00
REPS2	29.00	34.00	34.00	32.33	18.00
RER1	3365.00	3451.00	3430.00	3415.33	3417.00
RERE	2423.00	2392.00	2704.00	2506.33	2251.00
RERGL	61.00	63.00	84.00	69.33	54.00
REST	984.00	898.00	1048.00	976.67	1087.00
RETSAT	1748.00	1815.00	1846.00	1803.00	1630.00
REV1	995.00	1029.00	1089.00	1037.67	1104.00
REV3L	649.00	591.00	698.00	646.00	507.00
REXO1	1371.00	1430.00	1485.00	1428.67	1525.00
REXO2	1717.00	1790.00	1959.00	1822.00	2030.00
REXO4	794.00	773.00	821.00	796.00	982.00
RFC1	2385.00	2129.00	2438.00	2317.33	2650.00
RFC2	449.00	396.00	414.00	419.67	684.00
RFC3	520.00	480.00	518.00	506.00	668.00
RFC4	952.00	912.00	978.00	947.33	1295.00
RFC5	644.00	655.00	735.00	678.00	925.00
RFESD	58.00	74.00	72.00	68.00	81.00
RFFL	113.00	111.00	124.00	116.00	101.00
RFK	453.00	356.00	398.00	402.33	507.00
RFNG	1736.00	1842.00	1801.00	1793.00	1794.00
RFPL3S	13.00	10.00	10.00	11.00	11.00
RFT1	1706.00	1656.00	1733.00	1698.33	1691.00
RFTN1	1223.00	1096.00	1329.00	1216.00	1369.00
RFTN2	3.00	2.00	2.00	2.33	8.00
RFWD2	770.00	799.00	872.00	813.67	1089.00
RFWD3	1513.00	1483.00	1771.00	1589.00	1939.00
RFX1	467.00	473.00	442.00	460.67	423.00
RFX2	7.00	8.00	11.00	8.67	16.00
RFX3	514.00	426.00	634.00	524.67	600.00
RFX3-AS1	41.00	40.00	38.00	39.67	44.00
RFX5	1208.00	1127.00	1207.00	1180.67	1554.00
RFX7	617.00	555.00	586.00	586.00	537.00
RFX8	2809.00	2696.00	3011.00	2838.67	3276.00
RFXANK	783.00	765.00	823.00	790.33	948.00
RFXAP	206.00	245.00	273.00	241.33	239.00
RGAG1	4.00	3.00	6.00	4.33	2.00
RGAG4	27.00	32.00	36.00	31.67	27.00
RGCC	22.00	25.00	34.00	27.00	51.00
RGL1	418.00	387.00	426.00	410.33	432.00
RGL2	9.00	14.00	22.00	15.00	16.00
RGL3	520.00	464.00	559.00	514.33	422.00
RGMB	6427.00	6003.00	6494.00	6308.00	7462.00
RGMB-AS1	6.00	5.00	3.00	4.67	3.00
RGP1	430.00	361.00	390.00	393.67	206.00
RGPD3	18.00	11.00	16.00	15.00	6.00
RGS10	1575.00	1627.00	1728.00	1643.33	1531.00
RGS12	1452.00	1529.00	1504.00	1495.00	1217.00

RGS14	238.00	199.00	254.00	230.33	218.00
RGS16	15.00	26.00	21.00	20.67	25.00
RGS17	91.00	79.00	96.00	88.67	85.00
RGS19	273.00	251.00	232.00	252.00	340.00
RGS2	94.00	110.00	133.00	112.33	129.00
RGS20	549.00	521.00	584.00	551.33	699.00
RGS22	5.00	9.00	2.00	5.33	4.00
RGS3	1853.00	1833.00	2048.00	1911.33	3129.00
RGS4	1124.00	1000.00	1162.00	1095.33	3246.00
RGS5	177.00	149.00	162.00	162.67	183.00
RGS9	9.00	4.00	3.00	5.33	2.00
RGS9BP	11.00	3.00	8.00	7.33	7.00
RHBDD1	1799.00	1762.00	1970.00	1843.67	1824.00
RHBDD2	518.00	453.00	468.00	479.67	560.00
RHBDD3	620.00	677.00	624.00	640.33	737.00
RHBDF1	1440.00	1446.00	1609.00	1498.33	975.00
RHBDF2	1231.00	1215.00	1351.00	1265.67	1091.00
RHBDL1	123.00	128.00	139.00	130.00	94.00
RHCE	8.00	8.00	6.00	7.33	4.00
RHEB	1750.00	1630.00	1850.00	1743.33	2327.00
RHEBL1	135.00	117.00	138.00	130.00	132.00
RHNO1	582.00	579.00	606.00	589.00	838.00
RHOA	5353.00	5034.00	5538.00	5308.33	6709.00
RHOB	193.00	156.00	180.00	176.33	93.00
RHOBTB1	493.00	504.00	527.00	508.00	490.00
RHOBTB2	262.00	276.00	267.00	268.33	317.00
RHOBTB3	3360.00	3148.00	3676.00	3394.67	3238.00
RHOC	3014.00	3066.00	3189.00	3089.67	4016.00
RHOF	81.00	95.00	103.00	93.00	135.00
RHOG	605.00	643.00	617.00	621.67	739.00
RHOH	8.00	5.00	5.00	6.00	2.00
RHOJ	224.00	209.00	272.00	235.00	319.00
RHOQ	960.00	999.00	1071.00	1010.00	1133.00
RHOT1	811.00	683.00	922.00	805.33	929.00
RHOT2	2641.00	2827.00	2886.00	2784.67	2942.00
RHPN1	111.00	85.00	116.00	104.00	134.00
RHPN1-AS1	9.00	12.00	5.00	8.67	12.00
RHPN2	101.00	73.00	93.00	89.00	95.00
RIBC1	9.00	12.00	10.00	10.33	3.00
RIBC2	9.00	2.00	8.00	6.33	16.00
RIC1	1121.00	1030.00	1169.00	1106.67	955.00
RIC8A	1883.00	1819.00	1954.00	1885.33	2085.00
RIC8B	412.00	428.00	469.00	436.33	466.00
RICTOR	1437.00	1347.00	1617.00	1467.00	1155.00
RIF1	1571.00	1475.00	1708.00	1584.67	1568.00
RILPL1	601.00	631.00	660.00	630.67	543.00
RILPL2	196.00	225.00	243.00	221.33	232.00
RIMKLA	5.00	1.00	1.00	2.33	3.00

RIMKLB	1115.00	1047.00	1151.00	1104.33	1109.00
RIMS1	26.00	25.00	33.00	28.00	14.00
RIMS2	79.00	51.00	98.00	76.00	83.00
RIMS3	3.00	0.00	3.00	2.00	4.00
RIN1	1390.00	1354.00	1396.00	1380.00	1203.00
RIN2	1764.00	1675.00	1937.00	1792.00	2519.00
RIN3	993.00	974.00	1003.00	990.00	751.00
RINL	67.00	77.00	62.00	68.67	58.00
RINT1	650.00	658.00	696.00	668.00	788.00
RIOK1	1006.00	939.00	1001.00	982.00	1280.00
RIOK2	1162.00	1060.00	1283.00	1168.33	1255.00
RIOK3	1580.00	1470.00	1780.00	1610.00	1629.00
RIPK1	1026.00	979.00	1024.00	1009.67	1112.00
RIPK2	1123.00	1051.00	1332.00	1168.67	1250.00
RIPPLY2	54.00	61.00	78.00	64.33	85.00
RIT1	580.00	486.00	567.00	544.33	642.00
RITA1	533.00	504.00	618.00	551.67	676.00
RLF	472.00	508.00	562.00	514.00	500.00
RLIM	1874.00	1663.00	2053.00	1863.33	1885.00
RLTPR	10.00	19.00	8.00	12.33	15.00
RMDN1	1117.00	1096.00	1194.00	1135.67	1405.00
RMDN2	103.00	79.00	97.00	93.00	107.00
RMDN3	1045.00	1043.00	1062.00	1050.00	1268.00
RMI1	213.00	168.00	167.00	182.67	293.00
RMI2	166.00	165.00	167.00	166.00	216.00
RMND1	634.00	682.00	790.00	702.00	733.00
RMND5A	646.00	568.00	675.00	629.67	807.00
RMND5B	308.00	296.00	327.00	310.33	416.00
RMRP	18.00	15.00	6.00	13.00	27.00
RN7SK	28.00	46.00	31.00	35.00	64.00
RN7SL1	440.00	427.00	114.00	327.00	301.00
RN7SL2	643.00	529.00	100.00	424.00	301.00
RNA28S5	3.00	2.00	4.00	3.00	6.00
RNA45S5	10.00	9.00	12.00	10.33	12.00
RNASE4	13.00	21.00	21.00	18.33	32.00
RNASEH1	646.00	692.00	645.00	661.00	678.00
RNASEH1-AS1	222.00	256.00	274.00	250.67	344.00
RNASEH2A	726.00	664.00	697.00	695.67	944.00
RNASEH2B	423.00	392.00	444.00	419.67	568.00
RNASEH2B-AS1	5.00	8.00	11.00	8.00	3.00
RNASEH2C	1756.00	1786.00	1917.00	1819.67	1815.00
RNASEK	86.00	124.00	112.00	107.33	130.00
RNASEL	43.00	50.00	58.00	50.33	65.00
RNASET2	502.00	516.00	541.00	519.67	587.00
RND2	4.00	0.00	0.00	1.33	0.00
RND3	5162.00	4932.00	5572.00	5222.00	7042.00
RNF10	2037.00	1878.00	2105.00	2006.67	1876.00
RNF103	775.00	695.00	769.00	746.33	710.00

RNF11	1117.00	947.00	1161.00	1075.00	911.00
RNF111	998.00	816.00	985.00	933.00	788.00
RNF112	6.00	2.00	4.00	4.00	2.00
RNF113A	235.00	244.00	239.00	239.33	276.00
RNF114	1776.00	1707.00	1923.00	1802.00	2339.00
RNF115	472.00	394.00	457.00	441.00	389.00
RNF121	1583.00	1723.00	1637.00	1647.67	1464.00
RNF122	19.00	9.00	9.00	12.33	14.00
RNF123	1466.00	1376.00	1540.00	1460.67	1424.00
RNF125	167.00	122.00	162.00	150.33	235.00
RNF126	931.00	892.00	993.00	938.67	1169.00
RNF128	98.00	103.00	100.00	100.33	85.00
RNF13	1089.00	947.00	1119.00	1051.67	1181.00
RNF130	652.00	668.00	647.00	655.67	692.00
RNF133	3.00	1.00	2.00	2.00	3.00
RNF135	592.00	594.00	680.00	622.00	788.00
RNF138	348.00	313.00	423.00	361.33	470.00
RNF139	1191.00	1191.00	1262.00	1214.67	1226.00
RNF139-AS1	95.00	82.00	81.00	86.00	113.00
RNF14	825.00	687.00	845.00	785.67	944.00
RNF141	443.00	424.00	473.00	446.67	546.00
RNF144B	105.00	92.00	135.00	110.67	123.00
RNF145	1778.00	1678.00	1711.00	1722.33	1754.00
RNF146	1326.00	1303.00	1495.00	1374.67	1421.00
RNF149	3097.00	3103.00	2989.00	3063.00	3137.00
RNF150	45.00	43.00	60.00	49.33	34.00
RNF152	13.00	19.00	27.00	19.67	10.00
RNF157	125.00	118.00	129.00	124.00	89.00
RNF166	913.00	905.00	1005.00	941.00	1050.00
RNF167	2103.00	2104.00	2183.00	2130.00	2172.00
RNF168	421.00	371.00	432.00	408.00	414.00
RNF169	732.00	729.00	806.00	755.67	732.00
RNF170	697.00	645.00	743.00	695.00	790.00
RNF180	14.00	7.00	8.00	9.67	2.00
RNF181	945.00	921.00	944.00	936.67	1098.00
RNF182	185.00	170.00	194.00	183.00	178.00
RNF185	697.00	699.00	756.00	717.33	753.00
RNF187	1600.00	1585.00	1667.00	1617.33	1667.00
RNF19A	856.00	770.00	950.00	858.67	898.00
RNF19B	529.00	512.00	588.00	543.00	605.00
RNF2	483.00	404.00	502.00	463.00	661.00
RNF20	1381.00	1308.00	1508.00	1399.00	1282.00
RNF207	710.00	717.00	775.00	734.00	766.00
RNF208	33.00	45.00	46.00	41.33	52.00
RNF213	6097.00	5336.00	6485.00	5972.67	4482.00
RNF214	511.00	443.00	530.00	494.67	459.00
RNF215	811.00	893.00	835.00	846.33	1070.00
RNF216	2910.00	2894.00	3199.00	3001.00	2858.00

RNF216P1	715.00	683.00	724.00	707.33	799.00
RNF217	304.00	268.00	300.00	290.67	289.00
RNF217-AS1	6.00	13.00	15.00	11.33	16.00
RNF219	806.00	786.00	829.00	807.00	929.00
RNF219-AS1	3.00	1.00	0.00	1.33	0.00
RNF220	1734.00	1722.00	1946.00	1800.67	1526.00
RNF24	550.00	462.00	515.00	509.00	367.00
RNF25	506.00	481.00	548.00	511.67	629.00
RNF26	1804.00	1813.00	1717.00	1778.00	1864.00
RNF31	1121.00	1091.00	1218.00	1143.33	1139.00
RNF32	110.00	108.00	119.00	112.33	144.00
RNF34	585.00	611.00	573.00	589.67	723.00
RNF38	467.00	424.00	467.00	452.67	459.00
RNF4	641.00	676.00	723.00	680.00	781.00
RNF40	2563.00	2512.00	2554.00	2543.00	2371.00
RNF41	992.00	851.00	970.00	937.67	967.00
RNF44	1617.00	1558.00	1852.00	1675.67	1695.00
RNF5P1	19.00	20.00	17.00	18.67	24.00
RNF6	951.00	928.00	1084.00	987.67	1094.00
RNF7	1266.00	1330.00	1366.00	1320.67	1655.00
RNF8	485.00	470.00	489.00	481.33	529.00
RNFT1	623.00	613.00	587.00	607.67	700.00
RNFT2	119.00	135.00	129.00	127.67	167.00
RNGTT	700.00	545.00	645.00	630.00	668.00
RNH1	5.00	6.00	10.00	7.00	13.00
RNLS	56.00	42.00	43.00	47.00	42.00
RNMT	2180.00	1963.00	2367.00	2170.00	1957.00
RNMTL1	372.00	364.00	334.00	356.67	431.00
RNPC3	670.00	641.00	740.00	683.67	636.00
RNPEP	1385.00	1454.00	1438.00	1425.67	1693.00
RNPEPL1	1028.00	1111.00	1155.00	1098.00	1116.00
RNPS1	2801.00	2600.00	2810.00	2737.00	3558.00
RNU11	4.00	1.00	3.00	2.67	2.00
RNU2-1	3.00	3.00	2.00	2.67	4.00
RNU6-26P	48.00	44.00	61.00	51.00	18.00
RNU86	73.00	77.00	67.00	72.33	77.00
ROBO1	1511.00	1365.00	1561.00	1479.00	1314.00
ROBO2	71.00	77.00	94.00	80.67	71.00
ROBO3	392.00	448.00	405.00	415.00	589.00
ROBO4	1806.00	1820.00	1796.00	1807.33	1840.00
ROCK1	1556.00	1428.00	1689.00	1557.67	1492.00
ROCK1P1	34.00	29.00	53.00	38.67	36.00
ROCK2	1218.00	1065.00	1290.00	1191.00	966.00
ROGDI	85.00	103.00	113.00	100.33	112.00
ROM1	75.00	88.00	97.00	86.67	68.00
ROMO1	697.00	656.00	607.00	653.33	839.00
ROR1	29.00	21.00	35.00	28.33	21.00
RORA	141.00	135.00	169.00	148.33	188.00

RORA-AS1	23.00	18.00	33.00	24.67	25.00
RORB	5.00	17.00	12.00	11.33	6.00
ROS1	184.00	216.00	183.00	194.33	88.00
RP1	14.00	15.00	9.00	12.67	10.00
RP1L1	55.00	50.00	49.00	51.33	40.00
RP2	254.00	218.00	256.00	242.67	270.00
RP9	284.00	277.00	324.00	295.00	405.00
RP9P	70.00	84.00	67.00	73.67	94.00
RPA1	1293.00	1331.00	1427.00	1350.33	1633.00
RPA2	643.00	666.00	701.00	670.00	870.00
RPA3	562.00	507.00	545.00	538.00	736.00
RPA4	7.00	8.00	4.00	6.33	11.00
RPAIN	1400.00	1256.00	1437.00	1364.33	1496.00
RPAP1	1646.00	1604.00	1738.00	1662.67	1484.00
RPAP2	490.00	411.00	481.00	460.67	511.00
RPAP3	883.00	748.00	875.00	835.33	1053.00
RPARP-AS1	233.00	231.00	255.00	239.67	311.00
RPE	1320.00	1219.00	1520.00	1353.00	1507.00
RPEL1	16.00	26.00	19.00	20.33	27.00
RPF1	1041.00	955.00	1065.00	1020.33	1325.00
RPF2	1364.00	1231.00	1468.00	1354.33	1748.00
RPGR	227.00	198.00	182.00	202.33	305.00
RPGRIP1	5.00	3.00	5.00	4.33	1.00
RPGRIP1L	514.00	498.00	522.00	511.33	488.00
RPIA	778.00	716.00	859.00	784.33	1061.00
RPL10	16704.00	16769.00	17960.00	17144.33	22462.00
RPL10A	11534.00	11336.00	12685.00	11851.67	16055.00
RPL11	16077.00	16285.00	17698.00	16686.67	21672.00
RPL12	17826.00	18042.00	20353.00	18740.33	25954.00
RPL13	22691.00	22775.00	25683.00	23716.33	29859.00
RPL13A	28221.00	27354.00	31178.00	28917.67	36255.00
RPL13AP20	75.00	79.00	65.00	73.00	81.00
RPL13AP5	2211.00	1987.00	2337.00	2178.33	3127.00
RPL13AP6	3.00	0.00	1.00	1.33	3.00
RPL13P5	35.00	40.00	44.00	39.67	40.00
RPL14	9584.00	9006.00	10142.00	9577.33	12468.00
RPL15	16311.00	15915.00	18021.00	16749.00	21376.00
RPL17	1078.00	1080.00	1076.00	1078.00	1347.00
RPL18	10901.00	11099.00	11844.00	11281.33	14495.00
RPL18A	6662.00	6679.00	7250.00	6863.67	8960.00
RPL19	21590.00	21513.00	23939.00	22347.33	28649.00
RPL19P12	27.00	31.00	30.00	29.33	34.00
RPL21	672.00	673.00	793.00	712.67	996.00
RPL21P44	11.00	7.00	9.00	9.00	6.00
RPL22	5198.00	5048.00	5664.00	5303.33	7107.00
RPL22L1	3808.00	3912.00	3940.00	3886.67	5181.00
RPL23	14274.00	14404.00	15522.00	14733.33	19851.00
RPL23A	7338.00	7488.00	8276.00	7700.67	9967.00

RPL23AP53	125.00	99.00	113.00	112.33	106.00
RPL23AP7	175.00	179.00	154.00	169.33	179.00
RPL23AP82	114.00	115.00	94.00	107.67	128.00
RPL23P8	63.00	68.00	69.00	66.67	94.00
RPL24	6349.00	6508.00	7181.00	6679.33	9087.00
RPL26	19431.00	19283.00	21782.00	20165.33	27991.00
RPL26L1	764.00	703.00	771.00	746.00	1136.00
RPL27	12275.00	12251.00	13370.00	12632.00	17463.00
RPL27A	13338.00	13336.00	14583.00	13752.33	17444.00
RPL28	7978.00	7963.00	9019.00	8320.00	9991.00
RPL29	10705.00	10697.00	12172.00	11191.33	14537.00
RPL3	38683.00	39014.00	43704.00	40467.00	50408.00
RPL30	14397.00	14235.00	15797.00	14809.67	19381.00
RPL31	9661.00	9537.00	10751.00	9983.00	14280.00
RPL32	10609.00	10376.00	11934.00	10973.00	15400.00
RPL32P3	405.00	329.00	361.00	365.00	351.00
RPL34	4670.00	4667.00	5056.00	4797.67	6235.00
RPL34-AS1	5.00	5.00	12.00	7.33	16.00
RPL35	6393.00	6487.00	6824.00	6568.00	9064.00
RPL35A	9039.00	8966.00	9936.00	9313.67	13096.00
RPL36	6935.00	7276.00	7706.00	7305.67	9986.00
RPL36A	435.00	428.00	416.00	426.33	431.00
RPL36AL	1491.00	1617.00	1664.00	1590.67	1917.00
RPL37	18044.00	19039.00	18742.00	18608.33	26566.00
RPL37A	17856.00	18718.00	19453.00	18675.67	25570.00
RPL38	4821.00	4866.00	5432.00	5039.67	7654.00
RPL39	3940.00	4070.00	4345.00	4118.33	5454.00
RPL39L	559.00	522.00	533.00	538.00	834.00
RPL4	30515.00	28968.00	33783.00	31088.67	39424.00
RPL41	1043.00	1055.00	1172.00	1090.00	1592.00
RPL5	26750.00	26422.00	29311.00	27494.33	37013.00
RPL6	15494.00	15034.00	17409.00	15979.00	21336.00
RPL7	10527.00	10273.00	11576.00	10792.00	15195.00
RPL7A	23684.00	23666.00	26252.00	24534.00	31753.00
RPL7L1	1292.00	1140.00	1382.00	1271.33	1410.00
RPL8	31110.00	31494.00	34722.00	32442.00	45259.00
RPL9	10687.00	10896.00	11622.00	11068.33	15019.00
RPLP0	32502.00	31320.00	35830.00	33217.33	41796.00
RPLP0P2	75.00	54.00	72.00	67.00	63.00
RPLP1	21062.00	22391.00	23372.00	22275.00	29408.00
RPLP2	10483.00	10381.00	10727.00	10530.33	14251.00
RPN1	9007.00	8772.00	9038.00	8939.00	9139.00
RPN2	14400.00	13707.00	14109.00	14072.00	13456.00
RPP14	633.00	574.00	708.00	638.33	669.00
RPP25	24.00	24.00	22.00	23.33	45.00
RPP25L	485.00	464.00	513.00	487.33	634.00
RPP30	1018.00	981.00	1103.00	1034.00	1264.00
RPP38	282.00	268.00	325.00	291.67	429.00

RPP40	294.00	289.00	335.00	306.00	373.00
RPPH1	16.00	21.00	4.00	13.67	20.00
RPRD1A	1249.00	1272.00	1353.00	1291.33	1521.00
RPRD1B	1351.00	1281.00	1307.00	1313.00	1366.00
RPRD2	1491.00	1354.00	1519.00	1454.67	1345.00
RPRM	4.00	6.00	6.00	5.33	3.00
RPS10	698.00	704.00	731.00	711.00	1007.00
RPS10P7	68.00	80.00	76.00	74.67	94.00
RPS11	11790.00	11638.00	13193.00	12207.00	16453.00
RPS12	17272.00	17050.00	18930.00	17750.67	23754.00
RPS13	8452.00	8501.00	9280.00	8744.33	11642.00
RPS14	14014.00	14027.00	15177.00	14406.00	19691.00
RPS14P3	46.00	56.00	68.00	56.67	56.00
RPS15	4136.00	4097.00	4616.00	4283.00	5531.00
RPS15A	10377.00	10414.00	11203.00	10664.67	14363.00
RPS15AP10	12.00	13.00	14.00	13.00	6.00
RPS16	10765.00	10501.00	11724.00	10996.67	13938.00
RPS17	14.00	19.00	16.00	16.33	15.00
RPS18	160.00	172.00	154.00	162.00	229.00
RPS18P9	83.00	104.00	88.00	91.67	128.00
RPS19	12577.00	13242.00	14280.00	13366.33	16404.00
RPS19BP1	1044.00	1115.00	1157.00	1105.33	1314.00
RPS2	24143.00	26175.00	28009.00	26109.00	39103.00
RPS20	13852.00	13961.00	15194.00	14335.67	19380.00
RPS21	8449.00	9010.00	9192.00	8883.67	11758.00
RPS23	12441.00	12567.00	13621.00	12876.33	18374.00
RPS24	12434.00	12369.00	13446.00	12749.67	18131.00
RPS25	8830.00	9135.00	9776.00	9247.00	12905.00
RPS26	204.00	207.00	225.00	212.00	303.00
RPS27	5458.00	5774.00	6235.00	5822.33	7529.00
RPS27A	10617.00	10699.00	11553.00	10956.33	14683.00
RPS27L	902.00	824.00	984.00	903.33	1191.00
RPS28	1966.00	1959.00	2204.00	2043.00	2585.00
RPS29	5633.00	5851.00	6100.00	5861.33	8411.00
RPS2P32	30.00	29.00	18.00	25.67	16.00
RPS3	35876.00	35899.00	38969.00	36914.67	49092.00
RPS3A	7996.00	7980.00	9115.00	8363.67	11071.00
RPS4X	34099.00	33747.00	38450.00	35432.00	47539.00
RPS5	10641.00	10448.00	11703.00	10930.67	13287.00
RPS6	28439.00	27842.00	31938.00	29406.33	37153.00
RPS6KA1	347.00	330.00	417.00	364.67	385.00
RPS6KA2	4220.00	3883.00	4370.00	4157.67	4630.00
RPS6KA2-IT1	15.00	17.00	15.00	15.67	18.00
RPS6KA3	1277.00	1169.00	1321.00	1255.67	1152.00
RPS6KA4	1930.00	1850.00	1932.00	1904.00	2202.00
RPS6KA5	76.00	66.00	80.00	74.00	59.00
RPS6KA6	134.00	134.00	163.00	143.67	127.00
RPS6KB1	906.00	862.00	978.00	915.33	888.00

RPS6KB2	591.00	583.00	605.00	593.00	715.00
RPS6KC1	664.00	633.00	669.00	655.33	598.00
RPS6KL1	142.00	112.00	141.00	131.67	116.00
RPS7	13795.00	14448.00	15504.00	14582.33	20205.00
RPS8	18653.00	18021.00	20375.00	19016.33	24859.00
RPS9	350.00	365.00	358.00	357.67	433.00
RPSA	12595.00	12413.00	14296.00	13101.33	16892.00
RPSAP52	67.00	55.00	53.00	58.33	42.00
RPSAP58	1847.00	1879.00	2104.00	1943.33	2623.00
RPSAP9	42.00	49.00	64.00	51.67	59.00
RPTOR	1761.00	1702.00	1800.00	1754.33	1629.00
RPUSD1	1015.00	1042.00	1057.00	1038.00	1142.00
RPUSD2	317.00	366.00	354.00	345.67	370.00
RPUSD3	715.00	679.00	689.00	694.33	818.00
RPUSD4	699.00	625.00	719.00	681.00	815.00
RQCD1	1608.00	1551.00	1655.00	1604.67	2206.00
RRAD	8.00	18.00	12.00	12.67	13.00
RRAGA	799.00	754.00	816.00	789.67	954.00
RRAGB	330.00	330.00	380.00	346.67	305.00
RRAGC	686.00	689.00	732.00	702.33	656.00
RRAGD	7.00	11.00	15.00	11.00	14.00
RRAS	313.00	333.00	365.00	337.00	409.00
RRAS2	1406.00	1140.00	1517.00	1354.33	1784.00
RRBP1	8205.00	8752.00	8480.00	8479.00	6543.00
RREB1	1112.00	1010.00	1179.00	1100.33	931.00
RRH	4.00	3.00	3.00	3.33	4.00
RRM1	1473.00	1414.00	1419.00	1435.33	2250.00
RRM2	1326.00	1269.00	1367.00	1320.67	2406.00
RRM2B	516.00	457.00	566.00	513.00	625.00
RRN3	11.00	6.00	10.00	9.00	13.00
RRN3P1	341.00	293.00	317.00	317.00	320.00
RRN3P2	23.00	18.00	28.00	23.00	12.00
RRN3P3	109.00	108.00	102.00	106.33	117.00
RRNAD1	1106.00	1083.00	1163.00	1117.33	1107.00
RRP1	1130.00	1231.00	1114.00	1158.33	1341.00
RRP12	3168.00	3048.00	3284.00	3166.67	3011.00
RRP15	979.00	911.00	1082.00	990.67	1139.00
RRP1B	2245.00	2115.00	2419.00	2259.67	2235.00
RRP36	767.00	709.00	868.00	781.33	936.00
RRP7A	700.00	694.00	695.00	696.33	814.00
RRP7BP	224.00	263.00	208.00	231.67	197.00
RRP8	383.00	422.00	398.00	401.00	442.00
RRP9	1163.00	1234.00	1318.00	1238.33	1377.00
RRS1	1775.00	1847.00	1767.00	1796.33	2206.00
RRS1-AS1	8.00	3.00	2.00	4.33	4.00
RSAD1	2839.00	2922.00	3224.00	2995.00	3253.00
RSBN1	430.00	364.00	448.00	414.00	380.00
RSBN1L	400.00	359.00	414.00	391.00	410.00

RSC1A1	76.00	65.00	80.00	73.67	40.00
RSF1	711.00	606.00	786.00	701.00	639.00
RSG1	107.00	81.00	108.00	98.67	107.00
RSL1D1	4055.00	4064.00	4645.00	4254.67	5121.00
RSL24D1	3061.00	2988.00	3402.00	3150.33	4390.00
RSPH3	106.00	98.00	113.00	105.67	76.00
RSPH4A	8.00	8.00	3.00	6.33	6.00
RSPO2	16.00	9.00	6.00	10.33	12.00
RSPRY1	1289.00	1141.00	1353.00	1261.00	1726.00
RSRC1	371.00	389.00	404.00	388.00	473.00
RSRC2	1557.00	1615.00	1631.00	1601.00	1961.00
RSRP1	1596.00	1813.00	1855.00	1754.67	1690.00
RSU1	1856.00	1682.00	1933.00	1823.67	2257.00
RTCA	1359.00	1262.00	1432.00	1351.00	1738.00
RTCA-AS1	12.00	14.00	20.00	15.33	21.00
RTCB	1685.00	1686.00	1788.00	1719.67	2073.00
RTEL1	151.00	157.00	143.00	150.33	144.00
RTEL1-TNFRSF6B	8.00	5.00	7.00	6.67	17.00
RTF1	976.00	888.00	1094.00	986.00	1059.00
RTFDC1	2161.00	1980.00	2250.00	2130.33	2728.00
RTKN	398.00	319.00	369.00	362.00	436.00
RTKN2	140.00	132.00	169.00	147.00	196.00
RTN1	388.00	392.00	415.00	398.33	652.00
RTN2	142.00	144.00	116.00	134.00	103.00
RTN3	6626.00	6273.00	6136.00	6345.00	7021.00
RTN4	24993.00	23999.00	23762.00	24251.33	28744.00
RTN4IP1	239.00	210.00	263.00	237.33	303.00
RTN4RL2	32.00	34.00	27.00	31.00	37.00
RTP4	6.00	3.00	4.00	4.33	3.00
RTTN	473.00	375.00	426.00	424.67	419.00
RUFY1	787.00	768.00	841.00	798.67	824.00
RUFY2	653.00	555.00	672.00	626.67	548.00
RUFY3	1221.00	1167.00	1314.00	1234.00	1035.00
RUNDC1	448.00	411.00	436.00	431.67	384.00
RUNDC3A-AS1	9.00	4.00	8.00	7.00	9.00
RUNDC3B	27.00	27.00	23.00	25.67	34.00
RUNX1	2161.00	1927.00	2270.00	2119.33	2123.00
RUNX1-IT1	3.00	4.00	1.00	2.67	5.00
RUNX1T1	234.00	193.00	228.00	218.33	323.00
RUNX2	1098.00	999.00	1196.00	1097.67	959.00
RUSC1	647.00	710.00	715.00	690.67	681.00
RUSC1-AS1	31.00	25.00	22.00	26.00	19.00
RUSC2	3799.00	3789.00	3870.00	3819.33	2813.00
RUVBL1	2706.00	2583.00	2781.00	2690.00	3263.00
RUVBL2	2265.00	2246.00	2401.00	2304.00	2944.00
RWDD1	786.00	788.00	794.00	789.33	987.00
RWDD2A	260.00	242.00	309.00	270.33	280.00
RWDD2B	522.00	464.00	513.00	499.67	711.00

RWDD3	273.00	326.00	266.00	288.33	339.00
RWDD4	227.00	203.00	217.00	215.67	264.00
RXFP2	136.00	149.00	167.00	150.67	172.00
RXRA	320.00	344.00	370.00	344.67	351.00
RXRB	11.00	1.00	11.00	7.67	6.00
RYBP	710.00	600.00	721.00	677.00	750.00
RYK	2399.00	2281.00	2370.00	2350.00	2822.00
RYR2	101.00	84.00	81.00	88.67	77.00
S100A10	373.00	344.00	409.00	375.33	394.00
S100A11	1792.00	1734.00	1912.00	1812.67	2269.00
S100A13	1074.00	1090.00	1148.00	1104.00	1454.00
S100A14	7.00	5.00	14.00	8.67	11.00
S100A16	3530.00	3334.00	3764.00	3542.67	4800.00
S100A2	63.00	71.00	75.00	69.67	65.00
S100A3	10.00	7.00	5.00	7.33	6.00
S100A4	6.00	3.00	2.00	3.67	6.00
S100A5	4.00	1.00	3.00	2.67	5.00
S100A6	5180.00	5068.00	5458.00	5235.33	4884.00
S100G	14.00	8.00	7.00	9.67	19.00
S100PBP	863.00	818.00	952.00	877.67	862.00
S1PR1	237.00	246.00	179.00	220.67	233.00
S1PR2	105.00	89.00	103.00	99.00	78.00
S1PR3	714.00	625.00	675.00	671.33	585.00
SAAL1	525.00	512.00	514.00	517.00	660.00
SAC3D1	238.00	275.00	286.00	266.33	377.00
SACM1L	1065.00	1099.00	1183.00	1115.67	1272.00
SACS	4761.00	4269.00	5236.00	4755.33	3888.00
SAE1	2175.00	2082.00	2291.00	2182.67	2693.00
SAFB	2967.00	2770.00	3010.00	2915.67	3190.00
SAFB2	1471.00	1481.00	1593.00	1515.00	1547.00
SALL2	92.00	66.00	111.00	89.67	76.00
SAMD1	397.00	407.00	446.00	416.67	474.00
SAMD10	238.00	247.00	191.00	225.33	283.00
SAMD12	296.00	254.00	358.00	302.67	238.00
SAMD13	41.00	41.00	36.00	39.33	42.00
SAMD15	22.00	18.00	14.00	18.00	25.00
SAMD3	514.00	516.00	575.00	535.00	556.00
SAMD4A	2884.00	2787.00	2992.00	2887.67	3271.00
SAMD4B	2255.00	2046.00	2398.00	2233.00	1880.00
SAMD5	138.00	128.00	162.00	142.67	153.00
SAMD8	1471.00	1425.00	1550.00	1482.00	1420.00
SAMD9	603.00	525.00	576.00	568.00	502.00
SAMD9L	367.00	372.00	362.00	367.00	305.00
SAMHD1	570.00	513.00	529.00	537.33	522.00
SAMM50	718.00	726.00	751.00	731.67	875.00
SAMSN1	9.00	6.00	10.00	8.33	11.00
SAP130	639.00	617.00	729.00	661.67	666.00
SAP18	1247.00	1154.00	1438.00	1279.67	1724.00

SAP25	113.00	120.00	143.00	125.33	107.00
SAP30	136.00	160.00	160.00	152.00	217.00
SAP30BP	933.00	1053.00	1051.00	1012.33	1186.00
SAP30L	550.00	501.00	630.00	560.33	504.00
SAP30L-AS1	20.00	15.00	15.00	16.67	11.00
SAPCD2	543.00	599.00	724.00	622.00	682.00
SAR1A	1990.00	1879.00	2038.00	1969.00	2414.00
SAR1B	975.00	853.00	963.00	930.33	1115.00
SARAF	1906.00	1861.00	1888.00	1885.00	1769.00
SARDH	80.00	62.00	90.00	77.33	60.00
SARM1	820.00	770.00	832.00	807.33	840.00
SARNP	613.00	618.00	606.00	612.33	773.00
SARS	9673.00	9144.00	10046.00	9621.00	10974.00
SARS2	401.00	405.00	427.00	411.00	495.00
SART1	3119.00	3283.00	3159.00	3187.00	3765.00
SART3	1385.00	1392.00	1524.00	1433.67	1495.00
SASH1	2207.00	2020.00	2298.00	2175.00	2715.00
SASS6	472.00	419.00	487.00	459.33	517.00
SAT1	401.00	399.00	515.00	438.33	371.00
SAT2	841.00	888.00	972.00	900.33	961.00
SATB1	629.00	547.00	595.00	590.33	560.00
SATB1-AS1	7.00	2.00	4.00	4.33	10.00
SATB2	985.00	1040.00	1108.00	1044.33	1033.00
SAV1	288.00	242.00	303.00	277.67	307.00
SAXO2	12.00	11.00	7.00	10.00	8.00
SAYSD1	341.00	282.00	355.00	326.00	373.00
SBDS	1819.00	1788.00	1839.00	1815.33	1942.00
SBDSP1	626.00	634.00	686.00	648.67	837.00
SBF1	2382.00	2432.00	2514.00	2442.67	2187.00
SBF2	1298.00	1169.00	1385.00	1284.00	1123.00
SBF2-AS1	77.00	84.00	75.00	78.67	77.00
SBK1	5.00	1.00	11.00	5.67	7.00
SBNO1	1461.00	1416.00	1702.00	1526.33	1647.00
SBNO2	51.00	56.00	68.00	58.33	37.00
SBSN	267.00	287.00	253.00	269.00	225.00
SC5D	658.00	637.00	609.00	634.67	749.00
SCAF1	1608.00	1526.00	1604.00	1579.33	1468.00
SCAF11	2532.00	2420.00	2687.00	2546.33	2344.00
SCAF4	1267.00	1247.00	1358.00	1290.67	1296.00
SCAF8	1863.00	1759.00	1861.00	1827.67	1997.00
SCAI	294.00	276.00	354.00	308.00	269.00
SCAMP1	871.00	717.00	844.00	810.67	861.00
SCAMP1-AS1	151.00	182.00	166.00	166.33	185.00
SCAMP2	1355.00	1373.00	1290.00	1339.33	1246.00
SCAMP4	1856.00	1928.00	1743.00	1842.33	1791.00
SCAMP5	38.00	48.00	60.00	48.67	84.00
SCAND1	543.00	546.00	538.00	542.33	718.00
SCAND2P	223.00	197.00	179.00	199.67	200.00

SCAP	3015.00	3094.00	2964.00	3024.33	2689.00
SCAPER	248.00	203.00	235.00	228.67	276.00
SCARA3	25.00	17.00	24.00	22.00	15.00
SCARB1	2517.00	2412.00	2313.00	2414.00	2475.00
SCARB2	4356.00	4169.00	4389.00	4304.67	4740.00
SCARF2	900.00	969.00	922.00	930.33	797.00
SCARNA1	3.00	2.00	0.00	1.67	0.00
SCARNA12	147.00	139.00	128.00	138.00	151.00
SCARNA15	41.00	30.00	26.00	32.33	34.00
SCARNA16	17.00	20.00	18.00	18.33	15.00
SCARNA17	9.00	2.00	2.00	4.33	6.00
SCARNA2	3.00	1.00	2.00	2.00	5.00
SCARNA22	15.00	19.00	21.00	18.33	18.00
SCARNA27	39.00	40.00	33.00	37.33	22.00
SCARNA4	4.00	5.00	1.00	3.33	5.00
SCARNA5	3.00	4.00	4.00	3.67	4.00
SCARNA6	4.00	3.00	1.00	2.67	5.00
SCARNA7	4.00	1.00	1.00	2.00	6.00
SCARNA9	7.00	6.00	4.00	5.67	7.00
SCART1	189.00	221.00	225.00	211.67	236.00
SCCPDH	1258.00	1159.00	1219.00	1212.00	1543.00
SCD	9500.00	8804.00	9105.00	9136.33	10727.00
SCD5	211.00	164.00	185.00	186.67	189.00
SCEL	25.00	22.00	13.00	20.00	21.00
SCFD1	978.00	946.00	902.00	942.00	1124.00
SCFD2	470.00	397.00	497.00	454.67	496.00
SCG2	46.00	39.00	55.00	46.67	53.00
SCHIP1	4.00	3.00	3.00	3.33	2.00
SCLT1	391.00	382.00	366.00	379.67	398.00
SCLY	22.00	12.00	23.00	19.00	21.00
SCMH1	1460.00	1361.00	1350.00	1390.33	1457.00
SCML1	284.00	271.00	290.00	281.67	389.00
SCML2	148.00	145.00	149.00	147.33	162.00
SCN11A	3.00	0.00	0.00	1.00	0.00
SCN1B	10.00	4.00	7.00	7.00	4.00
SCN2A	33.00	29.00	29.00	30.33	46.00
SCN3A	6.00	5.00	6.00	5.67	4.00
SCN5A	317.00	361.00	359.00	345.67	146.00
SCN7A	8.00	12.00	4.00	8.00	2.00
SCN8A	982.00	1003.00	1010.00	998.33	690.00
SCN9A	27.00	30.00	22.00	26.33	16.00
SCNM1	86.00	77.00	103.00	88.67	122.00
SCNN1A	16.00	12.00	19.00	15.67	16.00
SCNN1D	518.00	586.00	520.00	541.33	484.00
SCO1	852.00	765.00	853.00	823.33	940.00
SCO2	296.00	282.00	298.00	292.00	283.00
SCOC	859.00	737.00	903.00	833.00	1073.00
SCOC-AS1	6.00	14.00	7.00	9.00	23.00

SCP2	954.00	819.00	923.00	898.67	1081.00
SCPEP1	1330.00	1357.00	1361.00	1349.33	1472.00
SCRIB	6.00	15.00	3.00	8.00	16.00
SCRN1	8947.00	7939.00	9152.00	8679.33	8465.00
SCRN2	734.00	687.00	663.00	694.67	789.00
SCRN3	537.00	515.00	527.00	526.33	588.00
SCUBE2	7.00	7.00	5.00	6.33	10.00
SCUBE3	44.00	47.00	38.00	43.00	54.00
SCX	76.00	109.00	105.00	96.67	116.00
SCYL1	3083.00	3145.00	3216.00	3148.00	3371.00
SCYL2	761.00	742.00	821.00	774.67	843.00
SCYL3	380.00	319.00	401.00	366.67	374.00
SDAD1	1120.00	1033.00	1191.00	1114.67	1335.00
SDC1	5692.00	5998.00	5727.00	5805.67	8906.00
SDC2	84.00	105.00	87.00	92.00	63.00
SDC3	1234.00	1197.00	1232.00	1221.00	1058.00
SDC4	1007.00	966.00	900.00	957.67	1380.00
SDCBP	3092.00	2791.00	3290.00	3057.67	3264.00
SDCBP2-AS1	84.00	105.00	61.00	83.33	122.00
SDCCAG3	2670.00	2697.00	2822.00	2729.67	3271.00
SDCCAG8	7.00	28.00	12.00	15.67	22.00
SDE2	606.00	550.00	591.00	582.33	585.00
SDF2	751.00	686.00	733.00	723.33	758.00
SDF2L1	366.00	369.00	333.00	356.00	471.00
SDF4	4672.00	4885.00	4347.00	4634.67	4887.00
SDHA	2398.00	2443.00	2569.00	2470.00	2683.00
SDHAF1	149.00	157.00	174.00	160.00	173.00
SDHAF2	538.00	458.00	572.00	522.67	658.00
SDHAF3	143.00	137.00	148.00	142.67	198.00
SDHAF4	141.00	140.00	176.00	152.33	232.00
SDHAP1	416.00	402.00	414.00	410.67	324.00
SDHAP2	8.00	1.00	1.00	3.33	0.00
SDHAP3	25.00	16.00	19.00	20.00	13.00
SDHB	1139.00	1142.00	1198.00	1159.67	1526.00
SDHC	1283.00	1187.00	1321.00	1263.67	1305.00
SDHD	1249.00	1171.00	1361.00	1260.33	1073.00
SDK1	24.00	18.00	28.00	23.33	20.00
SDR39U1	605.00	699.00	699.00	667.67	806.00
SDSL	135.00	188.00	166.00	163.00	173.00
SEC11A	3087.00	3071.00	3049.00	3069.00	4007.00
SEC11C	431.00	465.00	442.00	446.00	588.00
SEC13	2129.00	2008.00	2290.00	2142.33	2385.00
SEC14L1	2680.00	2418.00	2757.00	2618.33	2325.00
SEC14L1P1	58.00	55.00	49.00	54.00	61.00
SEC14L2	245.00	294.00	284.00	274.33	298.00
SEC16A	5157.00	4647.00	5003.00	4935.67	3996.00
SEC16B	138.00	140.00	162.00	146.67	164.00
SEC22A	508.00	471.00	513.00	497.33	565.00

SEC22B	1666.00	1527.00	1693.00	1628.67	1390.00
SEC22C	1272.00	1299.00	1332.00	1301.00	1247.00
SEC23A	1636.00	1429.00	1792.00	1619.00	1619.00
SEC23B	1767.00	1578.00	1862.00	1735.67	1848.00
SEC23IP	1769.00	1578.00	1814.00	1720.33	1760.00
SEC24A	960.00	756.00	1026.00	914.00	650.00
SEC24B	886.00	765.00	954.00	868.33	894.00
SEC24B-AS1	26.00	27.00	13.00	22.00	13.00
SEC24C	4496.00	4296.00	4639.00	4477.00	4010.00
SEC24D	1263.00	1198.00	1283.00	1248.00	996.00
SEC31A	2574.00	2379.00	2765.00	2572.67	2295.00
SEC31B	923.00	1003.00	1036.00	987.33	762.00
SEC61A1	18381.00	17967.00	18341.00	18229.67	15624.00
SEC61A2	439.00	409.00	482.00	443.33	429.00
SEC61B	1033.00	954.00	907.00	964.67	1194.00
SEC61G	838.00	829.00	871.00	846.00	1129.00
SEC62	3241.00	3112.00	3287.00	3213.33	3434.00
SEC63	5058.00	4766.00	4814.00	4879.33	5457.00
SECISBP2	1435.00	1404.00	1477.00	1438.67	1665.00
SECISBP2L	871.00	726.00	937.00	844.67	749.00
SECTM1	4.00	9.00	11.00	8.00	9.00
SEH1L	1632.00	1647.00	1942.00	1740.33	2355.00
SEL1L	3938.00	3574.00	4067.00	3859.67	2821.00
SEL1L3	717.00	655.00	719.00	697.00	405.00
SELENBP1	24.00	22.00	20.00	22.00	42.00
SELK	449.00	500.00	486.00	478.33	600.00
SELM	16.00	23.00	19.00	19.33	19.00
SELO	562.00	592.00	643.00	599.00	660.00
SELPLG	33.00	18.00	22.00	24.33	19.00
SELT	1764.00	1590.00	1755.00	1703.00	2226.00
SEMA3A	2075.00	1811.00	2152.00	2012.67	1638.00
SEMA3B	140.00	159.00	141.00	146.67	78.00
SEMA3C	843.00	755.00	965.00	854.33	1493.00
SEMA3D	29.00	29.00	34.00	30.67	20.00
SEMA3F	48.00	59.00	49.00	52.00	24.00
SEMA4A	90.00	103.00	79.00	90.67	128.00
SEMA4B	1457.00	1470.00	1335.00	1420.67	880.00
SEMA4C	3004.00	3220.00	3248.00	3157.33	2181.00
SEMA4D	169.00	145.00	161.00	158.33	143.00
SEMA4F	812.00	775.00	800.00	795.67	801.00
SEMA4G	64.00	47.00	53.00	54.67	54.00
SEMA5A	836.00	783.00	877.00	832.00	1074.00
SEMA6A	35.00	13.00	21.00	23.00	20.00
SEMA6A-AS1	17.00	28.00	38.00	27.67	24.00
SEMA6B	97.00	110.00	107.00	104.67	47.00
SEMA6C	231.00	226.00	256.00	237.67	260.00
SEMA6D	23.00	19.00	17.00	19.67	9.00
SEMA7A	8845.00	8953.00	8553.00	8783.67	4378.00

SENCR	72.00	57.00	73.00	67.33	89.00
SENP1	579.00	516.00	627.00	574.00	593.00
SENP2	823.00	756.00	819.00	799.33	963.00
SENP3	382.00	337.00	363.00	360.67	450.00
SENP3-EIF4A1	28.00	26.00	29.00	27.67	18.00
SENP5	1371.00	1271.00	1440.00	1360.67	1349.00
SENP6	2341.00	2354.00	2611.00	2435.33	2785.00
SENP7	325.00	327.00	353.00	335.00	314.00
SENP8	62.00	56.00	52.00	56.67	58.00
SEP1.	16.00	15.00	18.00	16.33	18.00
SEP10.	1286.00	1165.00	1370.00	1273.67	1570.00
SEP11.	2363.00	2067.00	2416.00	2282.00	2759.00
SEP15.	3099.00	3111.00	2992.00	3067.33	4094.00
SEP2.	7394.00	6868.00	7878.00	7380.00	8219.00
SEP6.	55.00	59.00	55.00	56.33	51.00
SEP7.	3122.00	2861.00	3144.00	3042.33	3668.00
SEP8.	1845.00	1854.00	1801.00	1833.33	1838.00
SEP9.	2675.00	2554.00	2738.00	2655.67	2446.00
SEPHS1	1847.00	1743.00	1782.00	1790.67	2241.00
SEPHS2	1399.00	1316.00	1432.00	1382.33	1739.00
SEPN1	3063.00	2970.00	2992.00	3008.33	2894.00
SEPSECS	260.00	216.00	285.00	253.67	267.00
SEPSECS-AS1	3.00	6.00	10.00	6.33	8.00
SEPT7-AS1	25.00	26.00	21.00	24.00	23.00
SEPT7P2	425.00	449.00	482.00	452.00	372.00
SEPT7P9	6.00	10.00	8.00	8.00	5.00
SEPW1	351.00	306.00	360.00	339.00	452.00
SERAC1	139.00	139.00	144.00	140.67	126.00
SERBP1	7410.00	6989.00	7973.00	7457.33	9721.00
SERF2	3509.00	3331.00	3571.00	3470.33	4286.00
SERGEF	221.00	204.00	261.00	228.67	271.00
SERINC1	6220.00	5981.00	6391.00	6197.33	6231.00
SERINC2	5170.00	4787.00	4725.00	4894.00	967.00
SERINC3	5113.00	4642.00	4965.00	4906.67	4260.00
SERINC4	3.00	5.00	8.00	5.33	3.00
SERINC5	400.00	453.00	385.00	412.67	328.00
SERP1	2850.00	2664.00	3142.00	2885.33	3342.00
SERP2	3.00	6.00	9.00	6.00	10.00
SERPINA1	15.00	10.00	12.00	12.33	35.00
SERPINA3	38.00	34.00	38.00	36.67	110.00
SERPINA5	71.00	76.00	86.00	77.67	146.00
SERPINB1	724.00	659.00	859.00	747.33	863.00
SERPINB2	13807.00	11529.00	13951.00	13095.67	20160.00
SERPINB6	1885.00	1798.00	1991.00	1891.33	2276.00
SERPINB7	1193.00	1087.00	1091.00	1123.67	1367.00
SERPINB8	1629.00	1516.00	1610.00	1585.00	2294.00
SERPINB9P1	5.00	5.00	10.00	6.67	6.00
SERPIND1	166.00	145.00	145.00	152.00	171.00

SERPINE1	82134.00	78740.00	84922.00	81932.00	63676.00
SERPINE2	729.00	739.00	644.00	704.00	569.00
SERPINF1	582.00	588.00	667.00	612.33	838.00
SERPINH1	10152.00	10495.00	9441.00	10029.33	8992.00
SERPINI1	136.00	121.00	118.00	125.00	147.00
SERTAD1	225.00	245.00	282.00	250.67	312.00
SERTAD2	1236.00	1173.00	1448.00	1285.67	1490.00
SERTAD3	177.00	189.00	193.00	186.33	220.00
SERTAD4	10.00	10.00	8.00	9.33	5.00
SERTAD4-AS1	13.00	13.00	18.00	14.67	6.00
SESN1	145.00	121.00	169.00	145.00	158.00
SESN2	4167.00	3870.00	4141.00	4059.33	4361.00
SESN3	301.00	255.00	317.00	291.00	244.00
SESTD1	239.00	233.00	267.00	246.33	219.00
SET	8724.00	8318.00	9143.00	8728.33	11367.00
SETBP1	125.00	103.00	126.00	118.00	69.00
SETD1A	1234.00	1173.00	1338.00	1248.33	1063.00
SETD1B	1003.00	976.00	1015.00	998.00	808.00
SETD2	1972.00	1944.00	2175.00	2030.33	1653.00
SETD3	914.00	808.00	888.00	870.00	1029.00
SETD4	824.00	855.00	857.00	845.33	845.00
SETD5	4510.00	4518.00	4952.00	4660.00	4225.00
SETD6	746.00	780.00	774.00	766.67	803.00
SETD7	1387.00	1255.00	1475.00	1372.33	1351.00
SETD8	1482.00	1424.00	1489.00	1465.00	1511.00
SETD9	134.00	148.00	168.00	150.00	223.00
SETDB1	1643.00	1433.00	1615.00	1563.67	1508.00
SETDB2	342.00	330.00	378.00	350.00	355.00
SETMAR	188.00	132.00	147.00	155.67	178.00
SETSIP	33.00	26.00	30.00	29.67	44.00
SETX	1722.00	1614.00	1882.00	1739.33	1694.00
SEZ6L2	777.00	795.00	838.00	803.33	660.00
SF1	8138.00	8231.00	8738.00	8369.00	8693.00
SF3A1	2940.00	2687.00	2931.00	2852.67	2741.00
SF3A2	1542.00	1571.00	1737.00	1616.67	1659.00
SF3A3	2608.00	2541.00	2736.00	2628.33	3101.00
SF3B1	10804.00	10071.00	11521.00	10798.67	10977.00
SF3B2	7886.00	7461.00	8019.00	7788.67	8683.00
SF3B3	7363.00	6954.00	7763.00	7360.00	8079.00
SF3B4	1643.00	1513.00	1599.00	1585.00	1843.00
SF3B5	1105.00	1080.00	1086.00	1090.33	1396.00
SF3B6	1919.00	1837.00	1887.00	1881.00	3510.00
SFI1	808.00	862.00	801.00	823.67	919.00
SFMBT1	379.00	367.00	394.00	380.00	380.00
SFMBT2	601.00	553.00	645.00	599.67	580.00
SFPQ	6921.00	6421.00	6966.00	6769.33	8404.00
SFR1	164.00	135.00	170.00	156.33	205.00
SFRP1	12762.00	12019.00	12886.00	12555.67	13839.00

SFSWAP	1017.00	1031.00	1100.00	1049.33	994.00
SFT2D1	1816.00	1874.00	1891.00	1860.33	1723.00
SFT2D2	86.00	79.00	100.00	88.33	101.00
SFT2D3	176.00	192.00	186.00	184.67	276.00
SFTA1P	8.00	8.00	9.00	8.33	2.00
SFXN1	988.00	890.00	954.00	944.00	1122.00
SFXN2	255.00	224.00	227.00	235.33	235.00
SFXN3	602.00	589.00	675.00	622.00	977.00
SFXN4	1155.00	1089.00	1279.00	1174.33	1565.00
SFXN5	360.00	353.00	347.00	353.33	276.00
SGCB	687.00	675.00	621.00	661.00	718.00
SGCE	1004.00	992.00	996.00	997.33	976.00
SGCG	3.00	3.00	1.00	2.33	1.00
SGCZ	12.00	11.00	18.00	13.67	14.00
SGK1	3693.00	3748.00	4095.00	3845.33	6173.00
SGK2	8.00	7.00	7.00	7.33	9.00
SGK223	689.00	661.00	747.00	699.00	390.00
SGK3	3.00	0.00	3.00	2.00	6.00
SGK494	807.00	787.00	859.00	817.67	643.00
SGMS1	520.00	517.00	641.00	559.33	524.00
SGMS1-AS1	52.00	50.00	55.00	52.33	53.00
SGMS2	342.00	288.00	320.00	316.67	232.00
SGOL1	256.00	256.00	253.00	255.00	319.00
SGOL2	380.00	342.00	387.00	369.67	479.00
SGPL1	1909.00	1822.00	1850.00	1860.33	2380.00
SGPP1	1061.00	1033.00	1062.00	1052.00	824.00
SGSH	1108.00	1176.00	1084.00	1122.67	1026.00
SGSM2	3675.00	3904.00	4183.00	3920.67	3543.00
SGSM3	1503.00	1509.00	1622.00	1544.67	1569.00
SGTA	1644.00	1665.00	1732.00	1680.33	1930.00
SGTB	455.00	381.00	485.00	440.33	425.00
SH2B1	1930.00	1899.00	1982.00	1937.00	1782.00
SH2B2	68.00	74.00	72.00	71.33	57.00
SH2B3	4394.00	4020.00	4466.00	4293.33	3138.00
SH2D1B	3.00	5.00	4.00	4.00	1.00
SH2D2A	9.00	11.00	11.00	10.33	18.00
SH2D5	1265.00	1276.00	1369.00	1303.33	1310.00
SH2D6	5.00	5.00	3.00	4.33	1.00
SH3BGR	36.00	28.00	30.00	31.33	31.00
SH3BGRL	1131.00	931.00	1247.00	1103.00	1277.00
SH3BGRL2	267.00	246.00	254.00	255.67	241.00
SH3BGRL3	2798.00	2639.00	2868.00	2768.33	3195.00
SH3BP1	234.00	236.00	277.00	249.00	261.00
SH3BP2	9948.00	9773.00	10895.00	10205.33	7814.00
SH3BP4	4188.00	3862.00	4539.00	4196.33	2261.00
SH3BP5	1226.00	1223.00	1351.00	1266.67	1504.00
SH3BP5-AS1	327.00	393.00	420.00	380.00	326.00
SH3BP5L	3643.00	3522.00	3447.00	3537.33	3152.00

SH3D19	524.00	510.00	572.00	535.33	558.00
SH3D21	485.00	520.00	503.00	502.67	312.00
SH3GL1	1157.00	1108.00	1154.00	1139.67	1440.00
SH3GL1P1	27.00	29.00	21.00	25.67	13.00
SH3GL2	7.00	13.00	16.00	12.00	22.00
SH3GL3	26.00	28.00	36.00	30.00	53.00
SH3GLB1	1440.00	1355.00	1574.00	1456.33	1568.00
SH3GLB2	1283.00	1349.00	1441.00	1357.67	1575.00
SH3KBP1	5039.00	4853.00	4917.00	4936.33	4449.00
SH3PXD2A	5203.00	4899.00	5550.00	5217.33	4523.00
SH3PXD2B	1199.00	1096.00	1263.00	1186.00	782.00
SH3RF1	753.00	638.00	740.00	710.33	629.00
SH3RF2	134.00	126.00	111.00	123.67	122.00
SH3RF3	908.00	830.00	887.00	875.00	704.00
SH3RF3-AS1	7.00	11.00	9.00	9.00	11.00
SH3TC1	193.00	185.00	249.00	209.00	214.00
SH3TC2	1024.00	952.00	1048.00	1008.00	1688.00
SH3YL1	1095.00	1047.00	1173.00	1105.00	1033.00
SHANK1	981.00	914.00	1095.00	996.67	799.00
SHANK2	1366.00	1266.00	1439.00	1357.00	1112.00
SHARPIN	1031.00	1064.00	1133.00	1076.00	1070.00
SHB	773.00	694.00	786.00	751.00	818.00
SHBG	7.00	5.00	6.00	6.00	3.00
SHC1	6559.00	6365.00	6925.00	6616.33	6563.00
SHC2	67.00	60.00	73.00	66.67	40.00
SHC3	49.00	58.00	50.00	52.33	42.00
SHC4	35.00	30.00	41.00	35.33	16.00
SHCBP1	638.00	600.00	638.00	625.33	733.00
SHF	28.00	24.00	26.00	26.00	21.00
SHFM1	1042.00	1145.00	1149.00	1112.00	1471.00
SHISA2	124.00	134.00	178.00	145.33	279.00
SHISA3	169.00	173.00	174.00	172.00	201.00
SHISA4	102.00	103.00	112.00	105.67	142.00
SHISA5	3001.00	2997.00	2839.00	2945.67	3580.00
SHKBP1	1241.00	1206.00	1326.00	1257.67	1436.00
SHMT1	332.00	345.00	340.00	339.00	484.00
SHMT2	10573.00	10092.00	10698.00	10454.33	11866.00
SHOC2	655.00	603.00	774.00	677.33	742.00
SHOX2	549.00	599.00	582.00	576.67	636.00
SHPK	534.00	471.00	551.00	518.67	389.00
SHPRH	624.00	631.00	702.00	652.33	590.00
SHQ1	462.00	432.00	504.00	466.00	591.00
SHROOM1	407.00	373.00	408.00	396.00	397.00
SHROOM2	713.00	631.00	619.00	654.33	714.00
SHROOM3	16.00	32.00	23.00	23.67	45.00
SHROOM4	23.00	31.00	27.00	27.00	22.00
SHTN1	1478.00	1434.00	1605.00	1505.67	1614.00
SIAE	911.00	830.00	976.00	905.67	695.00

SIAH1	456.00	411.00	464.00	443.67	563.00
SIAH2	637.00	590.00	625.00	617.33	791.00
SIDT2	449.00	484.00	470.00	467.67	327.00
SIGIRR	320.00	303.00	284.00	302.33	505.00
SIGLEC15	414.00	428.00	446.00	429.33	216.00
SIGLEC9	9.00	10.00	7.00	8.67	3.00
SIGMAR1	4642.00	4584.00	4305.00	4510.33	5095.00
SIK1	81.00	64.00	64.00	69.67	70.00
SIK2	504.00	459.00	526.00	496.33	412.00
SIK3	669.00	629.00	745.00	681.00	626.00
SIKE1	1498.00	1393.00	1669.00	1520.00	1471.00
SIL1	1783.00	1681.00	1582.00	1682.00	1831.00
SIM1	12.00	7.00	16.00	11.67	11.00
SIM2	82.00	74.00	71.00	75.67	80.00
SIMC1	317.00	301.00	303.00	307.00	343.00
SIN3A	1462.00	1435.00	1439.00	1445.33	1361.00
SIN3B	2291.00	2309.00	2519.00	2373.00	2002.00
SIPA1	908.00	882.00	865.00	885.00	835.00
SIPA1L1	2235.00	1956.00	2249.00	2146.67	1516.00
SIPA1L2	53.00	65.00	67.00	61.67	47.00
SIPA1L3	2891.00	2894.00	3246.00	3010.33	2612.00
SIRPA	695.00	723.00	733.00	717.00	615.00
SIRPB1	228.00	253.00	253.00	244.67	123.00
SIRPD	3.00	2.00	0.00	1.67	2.00
SIRT1	581.00	613.00	751.00	648.33	765.00
SIRT2	664.00	625.00	642.00	643.67	695.00
SIRT3	400.00	426.00	448.00	424.67	416.00
SIRT4	8.00	5.00	9.00	7.33	9.00
SIRT5	685.00	586.00	668.00	646.33	670.00
SIRT6	819.00	638.00	713.00	723.33	759.00
SIRT7	805.00	879.00	958.00	880.67	957.00
SIVA1	608.00	595.00	560.00	587.67	687.00
SIX1	449.00	397.00	406.00	417.33	436.00
SIX4	716.00	560.00	832.00	702.67	631.00
SIX5	257.00	303.00	310.00	290.00	333.00
SKA2	513.00	523.00	541.00	525.67	825.00
SKA3	400.00	380.00	484.00	421.33	539.00
SKAP1	10.00	20.00	9.00	13.00	12.00
SKAP2	1277.00	1157.00	1354.00	1262.67	1008.00
SKI	1843.00	1799.00	1859.00	1833.67	1983.00
SKIDA1	64.00	71.00	58.00	64.33	59.00
SKIL	827.00	744.00	949.00	840.00	832.00
SKIV2L	5.00	2.00	4.00	3.67	6.00
SKIV2L2	2611.00	2303.00	2728.00	2547.33	2873.00
SKOR1	11.00	20.00	16.00	15.67	13.00
SKP1	2362.00	2235.00	2478.00	2358.33	2979.00
SKP1P2	22.00	25.00	21.00	22.67	20.00
SKP2	652.00	604.00	743.00	666.33	854.00

SLA	5.00	2.00	5.00	4.00	2.00
SLAIN1	463.00	391.00	489.00	447.67	522.00
SLAIN2	924.00	850.00	990.00	921.33	946.00
SLBP	4056.00	3774.00	4140.00	3990.00	5071.00
SLC10A3	523.00	573.00	566.00	554.00	539.00
SLC10A5	7.00	5.00	2.00	4.67	9.00
SLC10A7	260.00	219.00	287.00	255.33	252.00
SLC11A2	1761.00	1668.00	1844.00	1757.67	1621.00
SLC12A2	690.00	597.00	675.00	654.00	661.00
SLC12A4	1169.00	1186.00	1220.00	1191.67	1233.00
SLC12A6	2078.00	1912.00	2160.00	2050.00	1968.00
SLC12A8	1368.00	1306.00	1396.00	1356.67	1526.00
SLC12A9	1854.00	1887.00	1928.00	1889.67	2010.00
SLC13A3	921.00	792.00	898.00	870.33	708.00
SLC14A1	4.00	8.00	4.00	5.33	1.00
SLC15A2	27.00	30.00	34.00	30.33	35.00
SLC15A4	558.00	471.00	542.00	523.67	591.00
SLC16A1	4860.00	4554.00	5120.00	4844.67	4670.00
SLC16A10	42.00	56.00	71.00	56.33	86.00
SLC16A12	89.00	59.00	64.00	70.67	36.00
SLC16A13	65.00	67.00	76.00	69.33	53.00
SLC16A14	66.00	61.00	61.00	62.67	50.00
SLC16A1-AS1	206.00	193.00	201.00	200.00	184.00
SLC16A2	526.00	496.00	483.00	501.67	597.00
SLC16A3	535.00	535.00	581.00	550.33	527.00
SLC16A4	21.00	34.00	27.00	27.33	28.00
SLC16A5	108.00	95.00	124.00	109.00	88.00
SLC16A6	192.00	184.00	230.00	202.00	231.00
SLC16A7	594.00	458.00	601.00	551.00	784.00
SLC16A9	1955.00	1815.00	1945.00	1905.00	1604.00
SLC17A5	569.00	530.00	579.00	559.33	289.00
SLC17A7	3.00	6.00	3.00	4.00	8.00
SLC18B1	749.00	769.00	714.00	744.00	717.00
SLC19A1	1164.00	1231.00	1245.00	1213.33	1244.00
SLC19A2	919.00	842.00	1030.00	930.33	902.00
SLC19A3	260.00	222.00	280.00	254.00	352.00
SLC1A1	232.00	245.00	267.00	248.00	314.00
SLC1A2	60.00	61.00	56.00	59.00	28.00
SLC1A3	1247.00	1266.00	1448.00	1320.33	1569.00
SLC1A4	2649.00	2559.00	2682.00	2630.00	2627.00
SLC1A5	16906.00	17051.00	17914.00	17290.33	16333.00
SLC20A1	9019.00	8499.00	8821.00	8779.67	11988.00
SLC20A2	1074.00	1005.00	1106.00	1061.67	931.00
SLC22A1	32.00	27.00	37.00	32.00	23.00
SLC22A13	16.00	9.00	9.00	11.33	2.00
SLC22A14	5.00	9.00	3.00	5.67	1.00
SLC22A15	548.00	495.00	537.00	526.67	392.00
SLC22A17	3.00	2.00	2.00	2.33	3.00

SLC22A18	57.00	39.00	37.00	44.33	61.00
SLC22A18AS	5.00	4.00	6.00	5.00	0.00
SLC22A20	14.00	15.00	10.00	13.00	2.00
SLC22A3	698.00	630.00	749.00	692.33	512.00
SLC22A4	95.00	98.00	99.00	97.33	101.00
SLC22A5	220.00	251.00	237.00	236.00	160.00
SLC23A2	2635.00	2469.00	2713.00	2605.67	2265.00
SLC23A3	160.00	185.00	213.00	186.00	115.00
SLC24A1	727.00	666.00	675.00	689.33	568.00
SLC24A2	3.00	6.00	2.00	3.67	0.00
SLC25A1	402.00	401.00	458.00	420.33	606.00
SLC25A10	410.00	358.00	340.00	369.33	475.00
SLC25A11	1089.00	1053.00	1062.00	1068.00	1373.00
SLC25A12	950.00	881.00	991.00	940.67	1034.00
SLC25A13	857.00	751.00	886.00	831.33	1170.00
SLC25A14	164.00	218.00	209.00	197.00	199.00
SLC25A15	468.00	434.00	461.00	454.33	609.00
SLC25A16	451.00	455.00	490.00	465.33	554.00
SLC25A17	453.00	382.00	460.00	431.67	598.00
SLC25A19	327.00	330.00	338.00	331.67	395.00
SLC25A20	132.00	122.00	105.00	119.67	160.00
SLC25A21	61.00	55.00	62.00	59.33	112.00
SLC25A21-AS1	92.00	83.00	107.00	94.00	94.00
SLC25A22	815.00	868.00	1005.00	896.00	1032.00
SLC25A23	190.00	172.00	178.00	180.00	181.00
SLC25A24	1438.00	1253.00	1451.00	1380.67	1668.00
SLC25A25	458.00	503.00	444.00	468.33	510.00
SLC25A25-AS1	473.00	535.00	659.00	555.67	423.00
SLC25A26	278.00	252.00	295.00	275.00	344.00
SLC25A27	939.00	922.00	1076.00	979.00	771.00
SLC25A28	643.00	801.00	757.00	733.67	780.00
SLC25A29	1114.00	1126.00	1276.00	1172.00	1151.00
SLC25A3	8427.00	8420.00	9468.00	8771.67	11197.00
SLC25A30	322.00	290.00	366.00	326.00	308.00
SLC25A32	1326.00	1201.00	1402.00	1309.67	1617.00
SLC25A33	328.00	336.00	335.00	333.00	442.00
SLC25A34	56.00	66.00	55.00	59.00	55.00
SLC25A35	255.00	265.00	256.00	258.67	311.00
SLC25A36	2220.00	2096.00	2542.00	2286.00	2526.00
SLC25A37	1343.00	1340.00	1490.00	1391.00	1088.00
SLC25A38	1016.00	969.00	1121.00	1035.33	1141.00
SLC25A39	2481.00	2522.00	2651.00	2551.33	2721.00
SLC25A4	295.00	237.00	301.00	277.67	375.00
SLC25A40	274.00	236.00	269.00	259.67	374.00
SLC25A42	105.00	144.00	106.00	118.33	114.00
SLC25A43	328.00	303.00	318.00	316.33	361.00
SLC25A44	763.00	765.00	750.00	759.33	866.00
SLC25A45	73.00	79.00	87.00	79.67	229.00

SLC25A46	1227.00	1018.00	1260.00	1168.33	1265.00
SLC25A5	3739.00	3523.00	4018.00	3760.00	4856.00
SLC25A51	254.00	221.00	229.00	234.67	273.00
SLC25A53	110.00	116.00	110.00	112.00	108.00
SLC25A5-AS1	28.00	28.00	21.00	25.67	32.00
SLC25A6	18.00	19.00	15.00	17.33	30.00
SLC26A1	6.00	4.00	4.00	4.67	4.00
SLC26A10	178.00	165.00	170.00	171.00	162.00
SLC26A11	461.00	442.00	457.00	453.33	487.00
SLC26A2	1106.00	1026.00	1174.00	1102.00	1063.00
SLC26A4	14.00	11.00	7.00	10.67	9.00
SLC26A6	1062.00	1112.00	1197.00	1123.67	810.00
SLC26A7	16.00	16.00	25.00	19.00	16.00
SLC27A1	328.00	339.00	379.00	348.67	291.00
SLC27A4	1655.00	1699.00	1567.00	1640.33	1708.00
SLC27A5	55.00	63.00	60.00	59.33	69.00
SLC27A6	56.00	65.00	55.00	58.67	60.00
SLC29A1	2522.00	2628.00	2471.00	2540.33	3184.00
SLC29A2	68.00	59.00	99.00	75.33	93.00
SLC29A3	9.00	5.00	16.00	10.00	12.00
SLC29A4	23.00	21.00	26.00	23.33	26.00
SLC2A1	2621.00	2552.00	2619.00	2597.33	2369.00
SLC2A10	89.00	111.00	104.00	101.33	69.00
SLC2A11	5.00	4.00	0.00	3.00	2.00
SLC2A12	37.00	38.00	47.00	40.67	50.00
SLC2A13	807.00	743.00	898.00	816.00	623.00
SLC2A1-AS1	12.00	13.00	18.00	14.33	10.00
SLC2A3	803.00	881.00	881.00	855.00	873.00
SLC2A4	5.00	3.00	3.00	3.67	1.00
SLC2A4RG	1305.00	1306.00	1374.00	1328.33	946.00
SLC2A6	253.00	222.00	203.00	226.00	206.00
SLC2A8	409.00	457.00	414.00	426.67	366.00
SLC30A1	487.00	414.00	516.00	472.33	422.00
SLC30A4	71.00	44.00	70.00	61.67	40.00
SLC30A5	1865.00	1783.00	1851.00	1833.00	2023.00
SLC30A6	1171.00	1031.00	1195.00	1132.33	1160.00
SLC30A7	2355.00	2167.00	2428.00	2316.67	2200.00
SLC30A9	2399.00	2053.00	2457.00	2303.00	2697.00
SLC31A1	1552.00	1490.00	1424.00	1488.67	1319.00
SLC31A2	168.00	168.00	150.00	162.00	178.00
SLC33A1	1801.00	1641.00	1908.00	1783.33	1614.00
SLC35A1	834.00	835.00	764.00	811.00	895.00
SLC35A2	813.00	848.00	706.00	789.00	891.00
SLC35A3	822.00	760.00	781.00	787.67	817.00
SLC35A4	1198.00	1074.00	1128.00	1133.33	1171.00
SLC35A5	1524.00	1349.00	1383.00	1418.67	1586.00
SLC35B1	901.00	886.00	914.00	900.33	1029.00
SLC35B2	2576.00	2573.00	2443.00	2530.67	2669.00

SLC35B3	775.00	764.00	817.00	785.33	877.00
SLC35B4	1324.00	1244.00	1291.00	1286.33	1200.00
SLC35C1	727.00	829.00	703.00	753.00	726.00
SLC35C2	2609.00	2659.00	2658.00	2642.00	2576.00
SLC35D1	696.00	660.00	717.00	691.00	728.00
SLC35D2	714.00	676.00	746.00	712.00	871.00
SLC35D3	3.00	2.00	4.00	3.00	1.00
SLC35E1	1277.00	1258.00	1271.00	1268.67	1383.00
SLC35E2	559.00	540.00	580.00	559.67	593.00
SLC35E2B	1719.00	1656.00	1826.00	1733.67	1617.00
SLC35E3	272.00	263.00	254.00	263.00	265.00
SLC35E4	536.00	595.00	570.00	567.00	446.00
SLC35F1	7.00	6.00	2.00	5.00	11.00
SLC35F2	1656.00	1698.00	1621.00	1658.33	1759.00
SLC35F3	8.00	9.00	11.00	9.33	8.00
SLC35F5	1251.00	1085.00	1130.00	1155.33	1316.00
SLC35F6	1164.00	1112.00	1059.00	1111.67	1091.00
SLC35G1	229.00	251.00	253.00	244.33	288.00
SLC35G2	1111.00	1146.00	1141.00	1132.67	1419.00
SLC36A1	866.00	808.00	782.00	818.67	752.00
SLC36A4	358.00	348.00	397.00	367.67	389.00
SLC37A2	877.00	824.00	883.00	861.33	470.00
SLC37A3	1729.00	1494.00	1695.00	1639.33	1844.00
SLC37A4	679.00	724.00	686.00	696.33	803.00
SLC38A1	20450.00	18781.00	21173.00	20134.67	16952.00
SLC38A10	5066.00	5240.00	5455.00	5253.67	4797.00
SLC38A2	25457.00	23590.00	28223.00	25756.67	24361.00
SLC38A4	52.00	59.00	61.00	57.33	80.00
SLC38A5	12.00	26.00	20.00	19.33	10.00
SLC38A6	1237.00	1232.00	1258.00	1242.33	1373.00
SLC38A7	1394.00	1307.00	1376.00	1359.00	1178.00
SLC38A9	751.00	685.00	722.00	719.33	660.00
SLC39A1	3329.00	3423.00	3348.00	3366.67	3441.00
SLC39A10	1505.00	1301.00	1646.00	1484.00	1631.00
SLC39A11	544.00	539.00	531.00	538.00	589.00
SLC39A13	3302.00	3588.00	3630.00	3506.67	3159.00
SLC39A14	6077.00	5540.00	5890.00	5835.67	5026.00
SLC39A3	723.00	770.00	732.00	741.67	830.00
SLC39A4	212.00	205.00	166.00	194.33	303.00
SLC39A5	5.00	6.00	5.00	5.33	6.00
SLC39A6	2233.00	2111.00	2290.00	2211.33	2133.00
SLC39A8	410.00	389.00	413.00	404.00	427.00
SLC39A9	1622.00	1521.00	1643.00	1595.33	1644.00
SLC3A2	15204.00	16012.00	15747.00	15654.33	15408.00
SLC41A1	2162.00	2145.00	2183.00	2163.33	1627.00
SLC41A2	187.00	145.00	200.00	177.33	138.00
SLC41A3	1100.00	1089.00	1076.00	1088.33	1293.00
SLC43A1	1198.00	1241.00	1257.00	1232.00	1433.00

SLC43A2	3.00	1.00	1.00	1.67	0.00
SLC43A3	7706.00	7548.00	7716.00	7656.67	7408.00
SLC44A1	1027.00	926.00	1018.00	990.33	1048.00
SLC44A2	1108.00	1122.00	1145.00	1125.00	795.00
SLC44A3	15.00	7.00	6.00	9.33	11.00
SLC44A5	769.00	615.00	670.00	684.67	631.00
SLC45A1	78.00	99.00	100.00	92.33	109.00
SLC45A3	701.00	658.00	778.00	712.33	579.00
SLC45A4	785.00	802.00	827.00	804.67	931.00
SLC46A1	197.00	190.00	211.00	199.33	178.00
SLC46A3	206.00	178.00	163.00	182.33	119.00
SLC48A1	340.00	390.00	398.00	376.00	398.00
SLC4A11	26.00	24.00	25.00	25.00	60.00
SLC4A1AP	840.00	778.00	921.00	846.33	899.00
SLC4A2	7129.00	7234.00	7225.00	7196.00	7374.00
SLC4A3	158.00	157.00	161.00	158.67	133.00
SLC4A4	51.00	37.00	54.00	47.33	56.00
SLC4A5	7.00	11.00	11.00	9.67	3.00
SLC4A7	3432.00	2887.00	3959.00	3426.00	2777.00
SLC4A8	556.00	535.00	611.00	567.33	564.00
SLC4A9	14.00	26.00	24.00	21.33	20.00
SLC50A1	1123.00	1247.00	1120.00	1163.33	1216.00
SLC52A2	3506.00	3467.00	3520.00	3497.67	3669.00
SLC5A3	1303.00	1194.00	1273.00	1256.67	1229.00
SLC5A6	2686.00	2704.00	2898.00	2762.67	2624.00
SLC6A13	4.00	7.00	5.00	5.33	9.00
SLC6A15	4647.00	4411.00	5052.00	4703.33	4550.00
SLC6A17	265.00	287.00	292.00	281.33	212.00
SLC6A6	1231.00	1260.00	1180.00	1223.67	935.00
SLC6A8	1468.00	1538.00	1666.00	1557.33	1698.00
SLC6A9	544.00	587.00	568.00	566.33	511.00
SLC7A1	9818.00	9371.00	10103.00	9764.00	8702.00
SLC7A11	5136.00	4513.00	5022.00	4890.33	2991.00
SLC7A11-AS1	241.00	237.00	266.00	248.00	293.00
SLC7A2	1287.00	1183.00	1441.00	1303.67	883.00
SLC7A3	4.00	2.00	4.00	3.33	1.00
SLC7A5	25633.00	25253.00	24306.00	25064.00	24375.00
SLC7A5P1	45.00	39.00	34.00	39.33	33.00
SLC7A5P2	72.00	56.00	62.00	63.33	47.00
SLC7A6	2410.00	2458.00	2624.00	2497.33	2130.00
SLC7A6OS	318.00	318.00	396.00	344.00	338.00
SLC7A7	30.00	29.00	40.00	33.00	25.00
SLC7A8	60.00	67.00	75.00	67.33	32.00
SLC7A9	6.00	2.00	4.00	4.00	4.00
SLC8A1	1504.00	1396.00	1536.00	1478.67	1409.00
SLC8A1-AS1	6.00	8.00	14.00	9.33	3.00
SLC8A2	3.00	3.00	3.00	3.00	0.00
SLC8B1	344.00	361.00	330.00	345.00	264.00

SLC9A1	2044.00	2003.00	2049.00	2032.00	1607.00
SLC9A2	18.00	17.00	21.00	18.67	23.00
SLC9A3R1	375.00	371.00	422.00	389.33	413.00
SLC9A3R2	2098.00	2054.00	2107.00	2086.33	1115.00
SLC9A5	710.00	704.00	729.00	714.33	659.00
SLC9A6	714.00	671.00	680.00	688.33	588.00
SLC9A7	3828.00	3795.00	3956.00	3859.67	3854.00
SLC9A8	1324.00	1313.00	1329.00	1322.00	1113.00
SLC9A9	38.00	49.00	50.00	45.67	53.00
SLC9B2	467.00	517.00	536.00	506.67	523.00
SLCO2A1	3.00	4.00	2.00	3.00	2.00
SLCO3A1	59.00	82.00	66.00	69.00	55.00
SLCO4A1	1604.00	1773.00	1642.00	1673.00	1679.00
SLF1	259.00	249.00	255.00	254.33	375.00
SLF2	1631.00	1460.00	1654.00	1581.67	1622.00
SLFN11	828.00	789.00	892.00	836.33	1111.00
SLFN12	402.00	389.00	378.00	389.67	364.00
SLFN12L	8.00	3.00	10.00	7.00	5.00
SLFN13	6.00	13.00	6.00	8.33	7.00
SLFN5	3407.00	2991.00	3529.00	3309.00	2500.00
SLFNL1	8.00	8.00	9.00	8.33	12.00
SLFNL1-AS1	177.00	183.00	156.00	172.00	151.00
SLIRP	745.00	744.00	809.00	766.00	1041.00
SLIT2	889.00	844.00	914.00	882.33	1204.00
SLIT3	2102.00	2107.00	2156.00	2121.67	1506.00
SLITRK3	6.00	7.00	2.00	5.00	5.00
SLITRK5	676.00	690.00	746.00	704.00	824.00
SLK	1774.00	1519.00	1929.00	1740.67	1673.00
SLMAP	1077.00	975.00	1163.00	1071.67	1101.00
SLMO1	350.00	322.00	362.00	344.67	376.00
SLMO2	965.00	837.00	1084.00	962.00	1102.00
SLTM	3630.00	3515.00	3817.00	3654.00	3838.00
SLU7	1171.00	1094.00	1310.00	1191.67	1237.00
SLX4	641.00	636.00	636.00	637.67	627.00
SLX4IP	121.00	77.00	78.00	92.00	71.00
SMA4	27.00	15.00	17.00	19.67	14.00
SMAD1	217.00	198.00	233.00	216.00	255.00
SMAD2	980.00	870.00	1109.00	986.33	1228.00
SMAD3	7307.00	7016.00	7721.00	7348.00	4253.00
SMAD4	1111.00	1138.00	1336.00	1195.00	1169.00
SMAD5	1951.00	1757.00	2204.00	1970.67	1854.00
SMAD6	14.00	12.00	26.00	17.33	24.00
SMAD7	142.00	158.00	176.00	158.67	101.00
SMAD9	106.00	116.00	135.00	119.00	137.00
SMAGP	1105.00	1108.00	1130.00	1114.33	1361.00
SMAP1	690.00	639.00	758.00	695.67	851.00
SMAP2	1417.00	1293.00	1359.00	1356.33	1904.00
SMARCA1	845.00	809.00	905.00	853.00	1079.00

SMARCA2	965.00	916.00	885.00	922.00	957.00
SMARCA4	4221.00	4263.00	4320.00	4268.00	4407.00
SMARCA5	1989.00	1832.00	2129.00	1983.33	2219.00
SMARCAD1	1459.00	1478.00	1681.00	1539.33	1605.00
SMARCAL1	823.00	727.00	835.00	795.00	882.00
SMARCC1	3136.00	2785.00	3239.00	3053.33	2734.00
SMARCC2	2868.00	2789.00	2954.00	2870.33	2592.00
SMARCD1	3114.00	3041.00	3252.00	3135.67	3802.00
SMARCD2	2477.00	2627.00	2742.00	2615.33	2983.00
SMARCD3	169.00	207.00	198.00	191.33	151.00
SMARCE1	1934.00	1724.00	2010.00	1889.33	2396.00
SMC1A	4478.00	4193.00	4403.00	4358.00	4385.00
SMC2	1106.00	1040.00	1075.00	1073.67	1268.00
SMC3	2713.00	2475.00	2753.00	2647.00	3032.00
SMC4	4807.00	4413.00	4775.00	4665.00	6040.00
SMC5	1096.00	939.00	1018.00	1017.67	989.00
SMC5-AS1	13.00	7.00	6.00	8.67	11.00
SMC6	1735.00	1586.00	1849.00	1723.33	1832.00
SMCHD1	3010.00	2714.00	3122.00	2948.67	2878.00
SMCO2	58.00	50.00	36.00	48.00	71.00
SMCO4	81.00	72.00	67.00	73.33	135.00
SMCR8	1921.00	1671.00	2022.00	1871.33	1452.00
SMG1	4039.00	3575.00	4424.00	4012.67	3679.00
SMG1P1	87.00	57.00	91.00	78.33	44.00
SMG1P2	33.00	28.00	31.00	30.67	25.00
SMG1P3	74.00	66.00	56.00	65.33	28.00
SMG1P5	13.00	11.00	16.00	13.33	8.00
SMG1P7	176.00	146.00	186.00	169.33	222.00
SMG5	4068.00	4091.00	4472.00	4210.33	4025.00
SMG6	1829.00	1760.00	1873.00	1820.67	2322.00
SMG7	2590.00	2385.00	2680.00	2551.67	2428.00
SMG7-AS1	5.00	4.00	5.00	4.67	3.00
SMG8	1051.00	1041.00	1070.00	1054.00	1113.00
SMG9	1561.00	1526.00	1561.00	1549.33	1958.00
SMIM10	108.00	83.00	99.00	96.67	124.00
SMIM10L1	108.00	133.00	127.00	122.67	130.00
SMIM12	723.00	721.00	742.00	728.67	674.00
SMIM13	729.00	715.00	877.00	773.67	1127.00
SMIM14	144.00	141.00	149.00	144.67	159.00
SMIM15	1230.00	1116.00	1368.00	1238.00	1595.00
SMIM17	6.00	9.00	6.00	7.00	6.00
SMIM19	359.00	349.00	397.00	368.33	460.00
SMIM20	289.00	280.00	319.00	296.00	356.00
SMIM2-AS1	24.00	17.00	18.00	19.67	24.00
SMIM3	85.00	96.00	96.00	92.33	89.00
SMIM4	213.00	231.00	185.00	209.67	244.00
SMIM7	854.00	773.00	859.00	828.67	1119.00
SMIM8	130.00	116.00	144.00	130.00	161.00

SMKR1	45.00	36.00	49.00	43.33	52.00
SMNDC1	1165.00	1098.00	1230.00	1164.33	1312.00
SMO	173.00	204.00	211.00	196.00	197.00
SMOC1	509.00	496.00	501.00	502.00	596.00
SMOC2	47.00	52.00	37.00	45.33	39.00
SMOX	719.00	780.00	828.00	775.67	633.00
SMPD1	1288.00	1434.00	1297.00	1339.67	1327.00
SMPD2	189.00	175.00	174.00	179.33	235.00
SMPD3	5.00	6.00	8.00	6.33	2.00
SMPD4	1679.00	1591.00	1677.00	1649.00	1522.00
SMPDL3A	194.00	147.00	185.00	175.33	198.00
SMS	2725.00	2764.00	2956.00	2815.00	4297.00
SMTN	7797.00	7865.00	7912.00	7858.00	7814.00
SMU1	1500.00	1493.00	1593.00	1528.67	1784.00
SMUG1	557.00	558.00	589.00	568.00	620.00
SMURF1	1100.00	1025.00	1228.00	1117.67	1031.00
SMURF2	8200.00	7657.00	8872.00	8243.00	11001.00
SMYD2	611.00	565.00	595.00	590.33	689.00
SMYD3	450.00	405.00	537.00	464.00	590.00
SMYD4	499.00	492.00	444.00	478.33	448.00
SMYD5	1082.00	1055.00	1140.00	1092.33	1127.00
SNAI1	34.00	29.00	36.00	33.00	50.00
SNAI2	1173.00	1104.00	1277.00	1184.67	1101.00
SNAI3	3.00	0.00	3.00	2.00	0.00
SNAI3-AS1	87.00	83.00	69.00	79.67	77.00
SNAP23	1501.00	1426.00	1568.00	1498.33	1597.00
SNAP25	137.00	119.00	122.00	126.00	185.00
SNAP29	504.00	504.00	492.00	500.00	552.00
SNAP47	513.00	563.00	645.00	573.67	678.00
SNAP91	67.00	64.00	56.00	62.33	67.00
SNAPC1	1981.00	1818.00	2058.00	1952.33	1956.00
SNAPC2	675.00	687.00	683.00	681.67	782.00
SNAPC3	661.00	712.00	735.00	702.67	744.00
SNAPC4	1439.00	1496.00	1485.00	1473.33	1472.00
SNAPC5	173.00	175.00	156.00	168.00	212.00
SNAPIN	395.00	364.00	426.00	395.00	466.00
SNCA	306.00	293.00	319.00	306.00	234.00
SNCAIP	44.00	43.00	67.00	51.33	93.00
SNCG	39.00	41.00	38.00	39.33	30.00
SND1	7450.00	6983.00	7837.00	7423.33	7045.00
SNF8	813.00	742.00	813.00	789.33	975.00
SNHG1	5536.00	5507.00	5716.00	5586.33	6789.00
SNHG10	290.00	375.00	374.00	346.33	395.00
SNHG11	494.00	474.00	512.00	493.33	550.00
SNHG12	850.00	865.00	935.00	883.33	976.00
SNHG15	1115.00	1141.00	1190.00	1148.67	1571.00
SNHG16	2266.00	2129.00	2436.00	2277.00	2693.00
SNHG17	1592.00	1590.00	1708.00	1630.00	2247.00

SNHG18	270.00	229.00	234.00	244.33	267.00
SNHG19	493.00	554.00	519.00	522.00	552.00
SNHG20	510.00	493.00	543.00	515.33	517.00
SNHG21	220.00	229.00	231.00	226.67	237.00
SNHG22	8.00	10.00	9.00	9.00	13.00
SNHG3	5222.00	5257.00	5735.00	5404.67	5662.00
SNHG4	1615.00	1500.00	1659.00	1591.33	1411.00
SNHG5	1134.00	1177.00	1238.00	1183.00	1490.00
SNHG6	1699.00	1687.00	1670.00	1685.33	2431.00
SNHG7	2964.00	2929.00	3103.00	2998.67	3356.00
SNHG8	2044.00	1986.00	2199.00	2076.33	2520.00
SNHG9	49.00	54.00	48.00	50.33	62.00
SNIP1	459.00	365.00	428.00	417.33	455.00
SNN	165.00	117.00	141.00	141.00	162.00
SNORA1	45.00	36.00	47.00	42.67	48.00
SNORA10	24.00	30.00	25.00	26.33	46.00
SNORA11	4.00	7.00	5.00	5.33	2.00
SNORA12	7.00	3.00	2.00	4.00	6.00
SNORA13	9.00	13.00	9.00	10.33	18.00
SNORA14B	12.00	13.00	15.00	13.33	20.00
SNORA15	3.00	5.00	1.00	3.00	4.00
SNORA16A	54.00	57.00	52.00	54.33	49.00
SNORA16B	3.00	0.00	1.00	1.33	0.00
SNORA18	207.00	226.00	207.00	213.33	209.00
SNORA20	6.00	6.00	3.00	5.00	7.00
SNORA21	31.00	21.00	35.00	29.00	28.00
SNORA22	5.00	5.00	6.00	5.33	3.00
SNORA23	6.00	2.00	6.00	4.67	3.00
SNORA24	38.00	45.00	40.00	41.00	41.00
SNORA25	125.00	127.00	135.00	129.00	125.00
SNORA26	25.00	22.00	25.00	24.00	24.00
SNORA27	40.00	45.00	32.00	39.00	43.00
SNORA29	7.00	1.00	6.00	4.67	5.00
SNORA2A	6.00	3.00	10.00	6.33	6.00
SNORA31	11.00	15.00	22.00	16.00	17.00
SNORA32	128.00	166.00	137.00	143.67	159.00
SNORA33	114.00	112.00	108.00	111.33	111.00
SNORA4	44.00	28.00	49.00	40.33	65.00
SNORA40	224.00	265.00	251.00	246.67	283.00
SNORA41	21.00	24.00	27.00	24.00	22.00
SNORA44	60.00	45.00	54.00	53.00	52.00
SNORA45A	46.00	52.00	50.00	49.33	38.00
SNORA45B	58.00	67.00	52.00	59.00	67.00
SNORA47	11.00	14.00	12.00	12.33	12.00
SNORA48	66.00	59.00	68.00	64.33	34.00
SNORA51	30.00	34.00	35.00	33.00	29.00
SNORA52	66.00	63.00	41.00	56.67	58.00
SNORA53	7.00	8.00	12.00	9.00	11.00

SNORA55	41.00	25.00	33.00	33.00	33.00
SNORA57	18.00	24.00	25.00	22.33	25.00
SNORA58	3.00	2.00	2.00	2.33	0.00
SNORA5A	9.00	18.00	13.00	13.33	15.00
SNORA5B	19.00	31.00	26.00	25.33	18.00
SNORA5C	55.00	43.00	51.00	49.67	44.00
SNORA6	54.00	66.00	61.00	60.33	39.00
SNORA60	18.00	27.00	29.00	24.67	24.00
SNORA61	165.00	219.00	182.00	188.67	181.00
SNORA62	84.00	83.00	76.00	81.00	83.00
SNORA63	74.00	51.00	64.00	63.00	65.00
SNORA64	55.00	66.00	61.00	60.67	66.00
SNORA65	33.00	35.00	33.00	33.67	36.00
SNORA66	37.00	27.00	33.00	32.33	48.00
SNORA67	100.00	124.00	119.00	114.33	119.00
SNORA68	39.00	47.00	44.00	43.33	36.00
SNORA69	28.00	21.00	12.00	20.33	18.00
SNORA70	281.00	300.00	320.00	300.33	332.00
SNORA71A	46.00	39.00	35.00	40.00	37.00
SNORA71B	44.00	54.00	51.00	49.67	49.00
SNORA71C	51.00	54.00	70.00	58.33	61.00
SNORA71E	9.00	16.00	12.00	12.33	5.00
SNORA72	41.00	31.00	31.00	34.33	43.00
SNORA73A	243.00	259.00	257.00	253.00	262.00
SNORA73B	145.00	125.00	135.00	135.00	155.00
SNORA74A	6.00	6.00	10.00	7.33	7.00
SNORA75	43.00	19.00	31.00	31.00	36.00
SNORA76C	20.00	9.00	16.00	15.00	17.00
SNORA78	5.00	1.00	1.00	2.33	1.00
SNORA7B	10.00	9.00	10.00	9.67	5.00
SNORA8	196.00	191.00	212.00	199.67	242.00
SNORA80B	13.00	6.00	10.00	9.67	8.00
SNORA80E	10.00	4.00	4.00	6.00	2.00
SNORA81	87.00	84.00	74.00	81.67	58.00
SNORA9	52.00	67.00	86.00	68.33	57.00
SNORD10	81.00	89.00	85.00	85.00	92.00
SNORD100	45.00	44.00	27.00	38.67	56.00
SNORD101	63.00	49.00	57.00	56.33	71.00
SNORD102	42.00	41.00	42.00	41.67	46.00
SNORD104	83.00	116.00	90.00	96.33	111.00
SNORD11	9.00	10.00	10.00	9.67	9.00
SNORD110	113.00	109.00	126.00	116.00	101.00
SNORD116-13	5.00	4.00	1.00	3.33	0.00
SNORD116-2	4.00	1.00	1.00	2.00	2.00
SNORD116-20	4.00	4.00	11.00	6.33	3.00
SNORD116-21	14.00	7.00	4.00	8.33	7.00
SNORD116-22	4.00	14.00	11.00	9.67	6.00
SNORD116-23	5.00	4.00	2.00	3.67	2.00

SNORD116-24	9.00	5.00	4.00	6.00	5.00
SNORD116-28	12.00	8.00	13.00	11.00	8.00
SNORD116-4	41.00	27.00	49.00	39.00	16.00
SNORD119	5.00	7.00	6.00	6.00	13.00
SNORD11B	3.00	1.00	3.00	2.33	2.00
SNORD12	73.00	73.00	60.00	68.67	44.00
SNORD121A	3.00	3.00	2.00	2.67	2.00
SNORD123	7.00	2.00	4.00	4.33	8.00
SNORD124	7.00	8.00	12.00	9.00	10.00
SNORD12B	60.00	46.00	55.00	53.67	43.00
SNORD12C	9.00	12.00	11.00	10.67	5.00
SNORD14A	69.00	75.00	63.00	69.00	74.00
SNORD14B	91.00	102.00	111.00	101.33	112.00
SNORD14C	11.00	5.00	5.00	7.00	7.00
SNORD14D	16.00	18.00	24.00	19.33	30.00
SNORD14E	16.00	17.00	15.00	16.00	11.00
SNORD15A	21.00	21.00	22.00	21.33	17.00
SNORD15B	30.00	29.00	27.00	28.67	26.00
SNORD16	16.00	15.00	30.00	20.33	23.00
SNORD17	21.00	16.00	20.00	19.00	29.00
SNORD18B	11.00	12.00	15.00	12.67	9.00
SNORD18C	50.00	50.00	38.00	46.00	49.00
SNORD19	7.00	19.00	17.00	14.33	15.00
SNORD19B	25.00	38.00	36.00	33.00	38.00
SNORD1A	18.00	12.00	18.00	16.00	9.00
SNORD1B	8.00	5.00	10.00	7.67	5.00
SNORD1C	19.00	33.00	24.00	25.33	18.00
SNORD2	67.00	54.00	67.00	62.67	64.00
SNORD20	17.00	20.00	19.00	18.67	21.00
SNORD21	27.00	31.00	25.00	27.67	25.00
SNORD22	140.00	182.00	171.00	164.33	186.00
SNORD23	16.00	17.00	12.00	15.00	23.00
SNORD24	15.00	15.00	18.00	16.00	15.00
SNORD25	57.00	70.00	54.00	60.33	69.00
SNORD26	37.00	39.00	20.00	32.00	32.00
SNORD27	5.00	5.00	11.00	7.00	17.00
SNORD28	60.00	57.00	80.00	65.67	85.00
SNORD29	24.00	14.00	29.00	22.33	21.00
SNORD30	30.00	28.00	33.00	30.33	43.00
SNORD31	213.00	228.00	223.00	221.33	216.00
SNORD32A	26.00	21.00	22.00	23.00	20.00
SNORD35A	36.00	44.00	31.00	37.00	39.00
SNORD35B	29.00	37.00	44.00	36.67	34.00
SNORD36A	196.00	188.00	188.00	190.67	280.00
SNORD36C	37.00	27.00	30.00	31.33	29.00
SNORD37	5.00	4.00	3.00	4.00	6.00
SNORD38A	10.00	13.00	8.00	10.33	17.00
SNORD38B	14.00	29.00	31.00	24.67	39.00

SNORD3A	5.00	3.00	1.00	3.00	3.00
SNORD42A	9.00	20.00	11.00	13.33	18.00
SNORD42B	16.00	10.00	9.00	11.67	2.00
SNORD43	26.00	34.00	30.00	30.00	32.00
SNORD44	137.00	129.00	121.00	129.00	156.00
SNORD45A	137.00	124.00	166.00	142.33	113.00
SNORD45B	207.00	213.00	222.00	214.00	191.00
SNORD45C	109.00	120.00	126.00	118.33	86.00
SNORD46	18.00	21.00	22.00	20.33	28.00
SNORD47	171.00	196.00	184.00	183.67	156.00
SNORD49A	99.00	91.00	107.00	99.00	95.00
SNORD4A	34.00	30.00	35.00	33.00	35.00
SNORD4B	5.00	5.00	3.00	4.33	5.00
SNORD5	87.00	76.00	112.00	91.67	88.00
SNORD50A	3.00	3.00	4.00	3.33	1.00
SNORD50B	29.00	45.00	41.00	38.33	37.00
SNORD51	9.00	14.00	7.00	10.00	16.00
SNORD53	3.00	3.00	2.00	2.67	2.00
SNORD54	26.00	36.00	43.00	35.00	40.00
SNORD55	24.00	23.00	30.00	25.67	26.00
SNORD57	5.00	6.00	2.00	4.33	8.00
SNORD58A	50.00	46.00	57.00	51.00	52.00
SNORD58C	43.00	91.00	57.00	63.67	72.00
SNORD59A	3.00	3.00	2.00	2.67	3.00
SNORD59B	6.00	6.00	5.00	5.67	2.00
SNORD6	43.00	27.00	41.00	37.00	37.00
SNORD60	8.00	7.00	18.00	11.00	17.00
SNORD63	22.00	27.00	26.00	25.00	22.00
SNORD65	10.00	11.00	12.00	11.00	11.00
SNORD67	5.00	3.00	1.00	3.00	2.00
SNORD68	31.00	23.00	24.00	26.00	34.00
SNORD69	5.00	9.00	6.00	6.67	9.00
SNORD7	10.00	10.00	5.00	8.33	11.00
SNORD72	9.00	4.00	6.00	6.33	3.00
SNORD74	45.00	58.00	34.00	45.67	45.00
SNORD75	4.00	3.00	4.00	3.67	9.00
SNORD76	50.00	43.00	43.00	45.33	36.00
SNORD77	80.00	76.00	74.00	76.67	75.00
SNORD78	70.00	99.00	88.00	85.67	101.00
SNORD79	26.00	22.00	26.00	24.67	26.00
SNORD80	443.00	458.00	415.00	438.67	456.00
SNORD81	23.00	31.00	25.00	26.33	28.00
SNORD83A	97.00	97.00	82.00	92.00	79.00
SNORD83B	118.00	118.00	127.00	121.00	99.00
SNORD85	3.00	1.00	2.00	2.00	0.00
SNORD87	31.00	28.00	31.00	30.00	25.00
SNORD88B	14.00	26.00	13.00	17.67	28.00
SNORD88C	3.00	2.00	10.00	5.00	16.00

SNORD89	44.00	36.00	28.00	36.00	46.00
SNORD93	12.00	10.00	12.00	11.33	13.00
SNORD94	18.00	16.00	19.00	17.67	13.00
SNORD95	28.00	20.00	18.00	22.00	10.00
SNORD96A	289.00	307.00	280.00	292.00	301.00
SNORD97	18.00	16.00	25.00	19.67	21.00
SNORD98	4.00	8.00	1.00	4.33	1.00
SNORD99	16.00	21.00	22.00	19.67	20.00
SNPH	493.00	547.00	555.00	531.67	615.00
SNRK	626.00	560.00	623.00	603.00	588.00
SNRK-AS1	383.00	361.00	438.00	394.00	306.00
SNRNP200	8198.00	7855.00	8655.00	8236.00	7408.00
SNRNP25	249.00	210.00	174.00	211.00	310.00
SNRNP27	487.00	427.00	548.00	487.33	704.00
SNRNP35	259.00	231.00	275.00	255.00	336.00
SNRNP40	1169.00	1055.00	1153.00	1125.67	1385.00
SNRNP48	750.00	648.00	718.00	705.33	726.00
SNRNP70	3760.00	3920.00	4024.00	3901.33	4247.00
SNRPA	1921.00	1875.00	1920.00	1905.33	2497.00
SNRPA1	942.00	990.00	989.00	973.67	1235.00
SNRPB	5331.00	5178.00	5576.00	5361.67	7324.00
SNRPB2	1658.00	1588.00	1621.00	1622.33	2194.00
SNRPC	1870.00	1935.00	1904.00	1903.00	2823.00
SNRPD1	1227.00	1144.00	1195.00	1188.67	1882.00
SNRPD2	1912.00	1933.00	2018.00	1954.33	2744.00
SNRPD3	1647.00	1612.00	1754.00	1671.00	2198.00
SNRPE	1242.00	1183.00	1237.00	1220.67	1949.00
SNRPF	1043.00	1071.00	1077.00	1063.67	1577.00
SNRPG	967.00	934.00	1018.00	973.00	1459.00
SNTA1	107.00	122.00	130.00	119.67	148.00
SNTB1	100.00	122.00	103.00	108.33	108.00
SNTB2	930.00	837.00	944.00	903.67	1024.00
SNU13	1739.00	1639.00	1794.00	1724.00	2514.00
SNUPN	547.00	518.00	552.00	539.00	666.00
SNW1	1832.00	1778.00	1857.00	1822.33	2122.00
SNX1	2671.00	2525.00	2776.00	2657.33	2493.00
SNX10	56.00	47.00	62.00	55.00	56.00
SNX11	367.00	374.00	412.00	384.33	383.00
SNX12	1044.00	893.00	1094.00	1010.33	1303.00
SNX13	1130.00	1041.00	1134.00	1101.67	1166.00
SNX14	2726.00	2548.00	2551.00	2608.33	2791.00
SNX16	244.00	227.00	291.00	254.00	320.00
SNX17	1834.00	1816.00	1772.00	1807.33	2276.00
SNX18	356.00	320.00	316.00	330.67	407.00
SNX19	1889.00	1732.00	1889.00	1836.67	1534.00
SNX2	1444.00	1324.00	1606.00	1458.00	1918.00
SNX21	451.00	433.00	461.00	448.33	408.00
SNX22	3.00	1.00	0.00	1.33	1.00

SNX24	239.00	265.00	295.00	266.33	358.00
SNX25	417.00	433.00	469.00	439.67	576.00
SNX27	1308.00	1257.00	1379.00	1314.67	1265.00
SNX29	539.00	494.00	500.00	511.00	365.00
SNX29P1	12.00	9.00	17.00	12.67	6.00
SNX29P2	5.00	2.00	5.00	4.00	2.00
SNX3	1832.00	1735.00	1871.00	1812.67	2166.00
SNX30	511.00	395.00	544.00	483.33	433.00
SNX32	64.00	52.00	58.00	58.00	61.00
SNX33	1558.00	1557.00	1651.00	1588.67	1171.00
SNX4	296.00	325.00	298.00	306.33	427.00
SNX5	2676.00	2756.00	2751.00	2727.67	3371.00
SNX6	1708.00	1504.00	1735.00	1649.00	1839.00
SNX7	481.00	463.00	495.00	479.67	632.00
SNX8	1080.00	1049.00	1130.00	1086.33	1140.00
SNX9	1692.00	1691.00	1861.00	1748.00	1581.00
SOAT1	2707.00	2561.00	2752.00	2673.33	2842.00
SOBP	163.00	180.00	173.00	172.00	159.00
SOCS1	5.00	13.00	6.00	8.00	11.00
SOCS2	515.00	469.00	568.00	517.33	658.00
SOCS2-AS1	81.00	50.00	64.00	65.00	59.00
SOCS3	1080.00	1031.00	1196.00	1102.33	1099.00
SOCS4	1267.00	1245.00	1403.00	1305.00	1284.00
SOCS5	604.00	526.00	672.00	600.67	556.00
SOCS6	385.00	370.00	432.00	395.67	337.00
SOCS7	65.00	80.00	67.00	70.67	61.00
SOD1	1884.00	1872.00	1936.00	1897.33	2540.00
SOD2	493.00	508.00	617.00	539.33	689.00
SOGA1	955.00	919.00	1026.00	966.67	626.00
SOGA3	515.00	467.00	532.00	504.67	325.00
SOHLH2	5.00	2.00	9.00	5.33	3.00
SON	7891.00	7151.00	7894.00	7645.33	6748.00
SORBS1	8.00	5.00	6.00	6.33	4.00
SORBS2	107.00	111.00	130.00	116.00	102.00
SORBS3	794.00	776.00	811.00	793.67	895.00
SORCS1	12.00	8.00	13.00	11.00	6.00
SORCS2	424.00	426.00	446.00	432.00	276.00
SORCS3	759.00	754.00	780.00	764.33	572.00
SORD	946.00	921.00	921.00	929.33	1211.00
SORT1	852.00	803.00	777.00	810.67	779.00
SOS1	1391.00	1170.00	1382.00	1314.33	1225.00
SOS2	857.00	699.00	835.00	797.00	758.00
SOST	3.00	0.00	0.00	1.00	1.00
SOWAHC	264.00	295.00	313.00	290.67	370.00
SOX12	1481.00	1511.00	1549.00	1513.67	1847.00
SOX13	161.00	136.00	127.00	141.33	113.00
SOX15	16.00	9.00	17.00	14.00	14.00
SOX21-AS1	15.00	16.00	14.00	15.00	14.00

SOX2-OT	3.00	11.00	7.00	7.00	12.00
SOX30	3.00	1.00	7.00	3.67	3.00
SOX4	576.00	515.00	676.00	589.00	629.00
SOX5	13.00	8.00	29.00	16.67	12.00
SOX6	10.00	12.00	23.00	15.00	7.00
SOX7	44.00	53.00	65.00	54.00	42.00
SOX9	1559.00	1583.00	1583.00	1575.00	2439.00
SOX9-AS1	74.00	71.00	63.00	69.33	115.00
SP1	2264.00	2119.00	2306.00	2229.67	1965.00
SP100	2068.00	1986.00	2221.00	2091.67	2069.00
SP110	333.00	306.00	345.00	328.00	347.00
SP140	152.00	171.00	167.00	163.33	132.00
SP140L	780.00	783.00	835.00	799.33	818.00
SP2	481.00	437.00	468.00	462.00	495.00
SP2-AS1	27.00	32.00	38.00	32.33	39.00
SP3	1525.00	1315.00	1639.00	1493.00	1647.00
SP4	408.00	298.00	402.00	369.33	392.00
SP5	8.00	7.00	9.00	8.00	11.00
SP6	4.00	4.00	10.00	6.00	7.00
SP8	13.00	13.00	15.00	13.67	17.00
SPA17	126.00	116.00	134.00	125.33	190.00
SPACA6P	92.00	91.00	85.00	89.33	88.00
SPAG1	180.00	153.00	157.00	163.33	222.00
SPAG16	286.00	291.00	318.00	298.33	378.00
SPAG4	219.00	215.00	222.00	218.67	140.00
SPAG5	1332.00	1274.00	1302.00	1302.67	1450.00
SPAG5-AS1	3.00	4.00	5.00	4.00	7.00
SPAG7	2530.00	2705.00	2825.00	2686.67	2767.00
SPAG8	38.00	48.00	41.00	42.33	43.00
SPAG9	3976.00	3656.00	4231.00	3954.33	3545.00
SPANXA2-OT1	3.00	3.00	3.00	3.00	3.00
SPANXB1	277.00	239.00	245.00	253.67	335.00
SPANXC	30.00	23.00	24.00	25.67	34.00
SPARC	6149.00	6098.00	5637.00	5961.33	5932.00
SPAST	412.00	316.00	430.00	386.00	528.00
SPATA1	5.00	2.00	6.00	4.33	8.00
SPATA13	1162.00	1127.00	1144.00	1144.33	886.00
SPATA17	70.00	49.00	59.00	59.33	62.00
SPATA2	516.00	530.00	517.00	521.00	641.00
SPATA20	2113.00	2109.00	2310.00	2177.33	1985.00
SPATA24	77.00	87.00	60.00	74.67	85.00
SPATA25	214.00	179.00	174.00	189.00	191.00
SPATA2L	201.00	196.00	172.00	189.67	308.00
SPATA33	205.00	240.00	207.00	217.33	206.00
SPATA3-AS1	5.00	8.00	4.00	5.67	14.00
SPATA4	21.00	9.00	13.00	14.33	21.00
SPATA5	208.00	176.00	243.00	209.00	205.00
SPATA5L1	549.00	524.00	579.00	550.67	570.00

SPATA6	55.00	64.00	80.00	66.33	92.00
SPATA6L	55.00	64.00	52.00	57.00	34.00
SPATA7	178.00	182.00	185.00	181.67	189.00
SPATA9	9.00	6.00	11.00	8.67	11.00
SPATS2	4381.00	4124.00	4574.00	4359.67	6027.00
SPATS2L	2094.00	2057.00	2266.00	2139.00	2244.00
SPC24	95.00	97.00	109.00	100.33	127.00
SPC25	5.00	6.00	2.00	4.33	9.00
SPCS1	1882.00	1877.00	1779.00	1846.00	2465.00
SPCS2	581.00	550.00	483.00	538.00	698.00
SPCS3	3117.00	2856.00	3001.00	2991.33	3870.00
SPDL1	1227.00	1185.00	1318.00	1243.33	1367.00
SPDYA	8.00	4.00	10.00	7.33	6.00
SPDYC	3.00	4.00	5.00	4.00	3.00
SPECC1	82.00	69.00	91.00	80.67	109.00
SPECC1L	1077.00	1002.00	1076.00	1051.67	1087.00
SPEF1	5.00	19.00	14.00	12.67	7.00
SPEF2	169.00	130.00	191.00	163.33	165.00
SPEG	3021.00	3202.00	3443.00	3222.00	2410.00
SPEN	4031.00	3654.00	3996.00	3893.67	2374.00
SPESP1	144.00	158.00	121.00	141.00	157.00
SPG11	2134.00	1851.00	2068.00	2017.67	1789.00
SPG20	1730.00	1562.00	1844.00	1712.00	1910.00
SPG20-AS1	23.00	31.00	27.00	27.00	30.00
SPG21	1704.00	1668.00	1851.00	1741.00	2069.00
SPG7	3228.00	3400.00	3468.00	3365.33	3395.00
SPHK1	3568.00	3607.00	3765.00	3646.67	3216.00
SPHK2	607.00	604.00	659.00	623.33	694.00
SPICE1	453.00	420.00	448.00	440.33	409.00
SPIDR	937.00	964.00	1089.00	996.67	961.00
SPIN1	1962.00	1721.00	2019.00	1900.67	1889.00
SPIN2B	27.00	29.00	37.00	31.00	42.00
SPIN3	857.00	883.00	886.00	875.33	867.00
SPIN4	408.00	367.00	464.00	413.00	566.00
SPINK1	4.00	7.00	6.00	5.67	2.00
SPINK2	3.00	2.00	1.00	2.00	1.00
SPINT2	33.00	35.00	26.00	31.33	22.00
SPIRE1	2586.00	2432.00	2874.00	2630.67	2851.00
SPIRE2	365.00	392.00	390.00	382.33	365.00
SPNS1	1640.00	1697.00	1631.00	1656.00	1803.00
SPNS2	7.00	2.00	3.00	4.00	7.00
SPNS3	30.00	39.00	40.00	36.33	21.00
SPOCD1	2476.00	2571.00	2766.00	2604.33	2167.00
SPOCK1	361.00	328.00	438.00	375.67	139.00
SPOCK2	3.00	7.00	12.00	7.33	4.00
SPOCK3	7.00	7.00	10.00	8.00	6.00
SPOP	1116.00	1069.00	1100.00	1095.00	1339.00
SPOPL	580.00	513.00	636.00	576.33	702.00

SPP1	76.00	81.00	84.00	80.33	141.00
SPPL2A	873.00	787.00	801.00	820.33	1026.00
SPPL2B	1674.00	1753.00	1717.00	1714.67	1655.00
SPPL3	889.00	899.00	920.00	902.67	1100.00
SPR	414.00	373.00	440.00	409.00	657.00
SPRED1	2545.00	2293.00	2840.00	2559.33	1938.00
SPRED2	1782.00	1667.00	1768.00	1739.00	2150.00
SPRED3	299.00	280.00	303.00	294.00	307.00
SPRTN	225.00	228.00	252.00	235.00	305.00
SPRY1	75.00	64.00	86.00	75.00	114.00
SPRY2	4612.00	4263.00	4930.00	4601.67	5536.00
SPRY4	3603.00	3363.00	3934.00	3633.33	3466.00
SPRY4-IT1	123.00	85.00	105.00	104.33	50.00
SPRYD3	525.00	545.00	604.00	558.00	620.00
SPRYD4	255.00	244.00	244.00	247.67	206.00
SPRYD7	249.00	239.00	252.00	246.67	338.00
SPSB1	864.00	839.00	984.00	895.67	868.00
SPSB2	276.00	283.00	303.00	287.33	304.00
SPSB3	653.00	662.00	726.00	680.33	678.00
SPTAN1	5716.00	5339.00	5890.00	5648.33	4827.00
SPTB	4.00	3.00	3.00	3.33	2.00
SPTBN1	4415.00	4109.00	4438.00	4320.67	2938.00
SPTBN2	3631.00	3759.00	3929.00	3773.00	3689.00
SPTBN4	8.00	3.00	6.00	5.67	0.00
SPTBN5	193.00	212.00	204.00	203.00	190.00
SPTLC1	2168.00	2121.00	1984.00	2091.00	2186.00
SPTLC2	1268.00	1223.00	1252.00	1247.67	1082.00
SPTLC3	464.00	459.00	513.00	478.67	418.00
SPTSSA	359.00	315.00	417.00	363.67	467.00
SPTY2D1	567.00	546.00	598.00	570.33	550.00
SPTY2D1-AS1	41.00	31.00	38.00	36.67	44.00
SPX	703.00	575.00	764.00	680.67	644.00
SQLE	1670.00	1597.00	1686.00	1651.00	1844.00
SQRDL	318.00	281.00	300.00	299.67	300.00
SQSTM1	8120.00	8042.00	8386.00	8182.67	6556.00
SRA1	885.00	903.00	947.00	911.67	1112.00
SRBD1	397.00	356.00	428.00	393.67	433.00
SRC	2204.00	2194.00	2291.00	2229.67	2176.00
SRCAP	4390.00	4100.00	4737.00	4409.00	3116.00
SRD5A1	430.00	436.00	419.00	428.33	443.00
SRD5A3	187.00	148.00	163.00	166.00	281.00
SRD5A3-AS1	14.00	6.00	15.00	11.67	7.00
SREBF1	3682.00	3603.00	3799.00	3694.67	3800.00
SREBF2	2232.00	2310.00	2248.00	2263.33	2281.00
SREK1	2911.00	2908.00	3322.00	3047.00	3272.00
SREK1IP1	602.00	495.00	672.00	589.67	636.00
SRF	1579.00	1619.00	1700.00	1632.67	1897.00
SRFBP1	467.00	448.00	456.00	457.00	527.00

SRGAP1	2226.00	1983.00	2319.00	2176.00	2211.00
SRGAP2	1784.00	1744.00	1939.00	1822.33	1612.00
SRGAP2B	108.00	99.00	109.00	105.33	79.00
SRGAP2C	72.00	41.00	74.00	62.33	58.00
SRGAP2D	25.00	29.00	51.00	35.00	21.00
SRGAP3	11.00	8.00	17.00	12.00	6.00
SRGN	7099.00	6573.00	6338.00	6670.00	9712.00
SRI	1342.00	1308.00	1320.00	1323.33	1899.00
SRM	1645.00	1516.00	1635.00	1598.67	2104.00
SRP14	1926.00	1822.00	1984.00	1910.67	2330.00
SRP14-AS1	228.00	264.00	219.00	237.00	152.00
SRP19	701.00	689.00	651.00	680.33	740.00
SRP54	1801.00	1531.00	1596.00	1642.67	1755.00
SRP68	1764.00	1674.00	1794.00	1744.00	1990.00
SRP72	2145.00	1852.00	2195.00	2064.00	2328.00
SRP9	2795.00	2533.00	2814.00	2714.00	3697.00
SRPK1	3626.00	3372.00	3769.00	3589.00	4275.00
SRPK2	684.00	695.00	807.00	728.67	852.00
SRPR	3423.00	3208.00	3290.00	3307.00	3436.00
SRPRB	2419.00	2414.00	2320.00	2384.33	2710.00
SRPX	5974.00	6053.00	5754.00	5927.00	7331.00
SRPX2	1290.00	1328.00	1311.00	1309.67	1116.00
SRR	151.00	194.00	165.00	170.00	176.00
SRRD	322.00	328.00	353.00	334.33	409.00
SRRM1	3058.00	3074.00	3525.00	3219.00	2957.00
SRRM2	31849.00	30292.00	34391.00	32177.33	22692.00
SRRM2-AS1	9.00	9.00	7.00	8.33	6.00
SRRM5	18.00	21.00	22.00	20.33	25.00
SRRT	2958.00	3166.00	3104.00	3076.00	3427.00
SRSF1	8553.00	7998.00	9010.00	8520.33	10303.00
SRSF10	3095.00	2960.00	3266.00	3107.00	3662.00
SRSF11	6920.00	6713.00	7529.00	7054.00	8062.00
SRSF2	7266.00	7214.00	7820.00	7433.33	8586.00
SRSF3	5458.00	5320.00	5911.00	5563.00	7168.00
SRSF4	2570.00	2544.00	2791.00	2635.00	2777.00
SRSF5	5397.00	5487.00	5756.00	5546.67	5610.00
SRSF6	7999.00	8287.00	8946.00	8410.67	8852.00
SRSF7	3468.00	3464.00	3527.00	3486.33	4288.00
SRSF8	747.00	628.00	811.00	728.67	801.00
SRSF9	2573.00	2573.00	2668.00	2604.67	3209.00
SRXN1	1620.00	1458.00	1619.00	1565.67	2010.00
SS18	2376.00	2342.00	2521.00	2413.00	2230.00
SS18L1	1219.00	1237.00	1328.00	1261.33	1315.00
SS18L2	302.00	281.00	274.00	285.67	367.00
SSB	3062.00	2930.00	3260.00	3084.00	3986.00
SSBP1	601.00	528.00	585.00	571.33	781.00
SSBP2	114.00	95.00	109.00	106.00	148.00
SSBP3	1100.00	1122.00	1148.00	1123.33	1268.00

SSBP4	993.00	904.00	1049.00	982.00	1095.00
SSC4D	10.00	13.00	18.00	13.67	14.00
SSC5D	40.00	38.00	45.00	41.00	28.00
SSFA2	3347.00	2792.00	3620.00	3253.00	3320.00
SSH1	1916.00	1692.00	2017.00	1875.00	1513.00
SSH2	702.00	594.00	712.00	669.33	610.00
SSH3	211.00	202.00	213.00	208.67	216.00
SSNA1	663.00	687.00	710.00	686.67	937.00
SSPN	10.00	8.00	15.00	11.00	13.00
SSPO	7.00	2.00	6.00	5.00	1.00
SSR1	7603.00	7179.00	7338.00	7373.33	7616.00
SSR2	5530.00	5488.00	5207.00	5408.33	6936.00
SSR3	21072.00	19343.00	19538.00	19984.33	28925.00
SSR4	2263.00	2179.00	2030.00	2157.33	2542.00
SSR4P1	74.00	71.00	76.00	73.67	86.00
SSRP1	5247.00	4987.00	5656.00	5296.67	6486.00
SSSCA1	639.00	622.00	628.00	629.67	858.00
SSSCA1-AS1	59.00	43.00	44.00	48.67	43.00
SSU72	1501.00	1497.00	1633.00	1543.67	2072.00
SSUH2	14.00	14.00	14.00	14.00	9.00
SSX2IP	486.00	434.00	535.00	485.00	615.00
ST13	2815.00	2713.00	3009.00	2845.67	3566.00
ST13P4	41.00	31.00	43.00	38.33	77.00
ST20	365.00	377.00	383.00	375.00	413.00
ST20-AS1	40.00	58.00	58.00	52.00	72.00
ST20-MTHFS	7.00	6.00	6.00	6.33	10.00
ST3GAL1	2931.00	2791.00	3025.00	2915.67	1944.00
ST3GAL2	568.00	579.00	575.00	574.00	572.00
ST3GAL3	595.00	595.00	648.00	612.67	666.00
ST3GAL4	289.00	260.00	241.00	263.33	269.00
ST3GAL4-AS1	39.00	41.00	27.00	35.67	30.00
ST3GAL5	128.00	121.00	129.00	126.00	112.00
ST3GAL6	853.00	887.00	993.00	911.00	564.00
ST3GAL6-AS1	78.00	86.00	95.00	86.33	59.00
ST5	668.00	638.00	666.00	657.33	552.00
ST6GAL1	349.00	372.00	402.00	374.33	434.00
ST6GAL2	14.00	10.00	14.00	12.67	17.00
ST6GALNAC2	8.00	3.00	3.00	4.67	1.00
ST6GALNAC3	298.00	232.00	290.00	273.33	229.00
ST6GALNAC4	554.00	551.00	501.00	535.33	597.00
ST6GALNAC6	832.00	902.00	961.00	898.33	1147.00
ST7	968.00	929.00	1030.00	975.67	880.00
ST7-AS1	50.00	47.00	48.00	48.33	35.00
ST7L	1136.00	1115.00	1173.00	1141.33	1260.00
ST7-OT4	11.00	9.00	19.00	13.00	7.00
ST8SIA1	246.00	208.00	244.00	232.67	268.00
ST8SIA4	83.00	92.00	81.00	85.33	90.00
ST8SIA5	55.00	39.00	39.00	44.33	33.00

STAB1	5.00	4.00	5.00	4.67	2.00
STAC	154.00	134.00	167.00	151.67	118.00
STAC3	71.00	81.00	72.00	74.67	70.00
STAG1	1076.00	974.00	1141.00	1063.67	1011.00
STAG2	2315.00	1952.00	2466.00	2244.33	2254.00
STAG3	239.00	265.00	246.00	250.00	303.00
STAG3L1	39.00	22.00	24.00	28.33	20.00
STAG3L2	258.00	236.00	197.00	230.33	167.00
STAG3L3	77.00	98.00	107.00	94.00	75.00
STAG3L4	184.00	166.00	206.00	185.33	204.00
STAG3L5P	5.00	4.00	8.00	5.67	8.00
AG3L5P-PVRIG2P-PIL	820.00	850.00	882.00	850.67	782.00
STAM	906.00	883.00	1009.00	932.67	1055.00
STAM2	632.00	600.00	715.00	649.00	704.00
STAM-AS1	77.00	85.00	110.00	90.67	78.00
STAMBP	1027.00	877.00	1062.00	988.67	1147.00
STAMBPL1	974.00	846.00	1004.00	941.33	1054.00
STAP2	76.00	63.00	97.00	78.67	119.00
STARD10	179.00	194.00	238.00	203.67	261.00
STARD13	2744.00	2516.00	2905.00	2721.67	2582.00
STARD3	1601.00	1604.00	1585.00	1596.67	1619.00
STARD3NL	1332.00	1208.00	1348.00	1296.00	1286.00
STARD4	331.00	297.00	357.00	328.33	451.00
STARD5	58.00	50.00	55.00	54.33	45.00
STARD7	3226.00	3009.00	3365.00	3200.00	3767.00
STARD7-AS1	228.00	222.00	249.00	233.00	205.00
STARD8	483.00	441.00	511.00	478.33	460.00
STARD9	756.00	685.00	778.00	739.67	448.00
STAT1	1482.00	1387.00	1651.00	1506.67	1669.00
STAT2	1298.00	1299.00	1336.00	1311.00	1101.00
STAT3	1151.00	1075.00	1111.00	1112.33	1156.00
STAT4	30.00	35.00	40.00	35.00	22.00
STAT5A	146.00	148.00	170.00	154.67	158.00
STAT5B	1669.00	1589.00	1788.00	1682.00	1579.00
STAT6	4107.00	4100.00	4461.00	4222.67	3822.00
STAU1	2526.00	2361.00	2631.00	2506.00	2896.00
STAU2	657.00	653.00	647.00	652.33	702.00
STBD1	7.00	17.00	12.00	12.00	11.00
STC1	4321.00	4087.00	4611.00	4339.67	3521.00
STC2	15862.00	16470.00	16653.00	16328.33	13437.00
STEAP1	1293.00	1271.00	1181.00	1248.33	2130.00
STEAP1B	934.00	853.00	863.00	883.33	1551.00
STEAP2	813.00	798.00	955.00	855.33	1092.00
STEAP3	5071.00	4978.00	5338.00	5129.00	4132.00
STIL	575.00	593.00	607.00	591.67	782.00
STIM1	1303.00	1161.00	1222.00	1228.67	1030.00
STIM2	372.00	379.00	360.00	370.33	412.00
STIP1	9115.00	9098.00	10202.00	9471.67	10739.00

STK10	1153.00	1133.00	1125.00	1137.00	832.00
STK11	1261.00	1268.00	1392.00	1307.00	1483.00
STK11IP	672.00	699.00	741.00	704.00	869.00
STK16	661.00	635.00	687.00	661.00	763.00
STK17A	1533.00	1446.00	1650.00	1543.00	1850.00
STK17B	550.00	505.00	593.00	549.33	467.00
STK24	1877.00	1784.00	2023.00	1894.67	2119.00
STK25	2575.00	2471.00	2628.00	2558.00	3029.00
STK26	373.00	305.00	424.00	367.33	420.00
STK3	527.00	472.00	513.00	504.00	608.00
STK32A	37.00	54.00	70.00	53.67	48.00
STK32C	36.00	32.00	40.00	36.00	40.00
STK33	201.00	183.00	252.00	212.00	199.00
STK35	1530.00	1450.00	1584.00	1521.33	1448.00
STK36	1460.00	1435.00	1482.00	1459.00	1304.00
STK38	1169.00	1252.00	1255.00	1225.33	1241.00
STK38L	496.00	470.00	559.00	508.33	637.00
STK39	1225.00	1122.00	1225.00	1190.67	1277.00
STK4	1933.00	1668.00	1944.00	1848.33	1528.00
STK40	1429.00	1337.00	1351.00	1372.33	1139.00
STK4-AS1	7.00	8.00	12.00	9.00	20.00
STKLD1	11.00	14.00	8.00	11.00	15.00
STMN1	2286.00	2057.00	2218.00	2187.00	3811.00
STMN3	964.00	993.00	1041.00	999.33	1490.00
STOM	1295.00	1201.00	1299.00	1265.00	1611.00
STOML1	322.00	324.00	285.00	310.33	331.00
STOML2	3867.00	3597.00	4090.00	3851.33	5625.00
STON1	109.00	99.00	100.00	102.67	87.00
STOX1	3.00	4.00	4.00	3.67	5.00
STPG1	400.00	323.00	288.00	337.00	299.00
STPG2	3.00	4.00	1.00	2.67	4.00
STRA13	608.00	581.00	675.00	621.33	922.00
STRADA	927.00	956.00	1053.00	978.67	1123.00
STRADB	673.00	620.00	722.00	671.67	677.00
STRAP	4272.00	4058.00	4562.00	4297.33	4040.00
STRBP	529.00	553.00	592.00	558.00	813.00
STRIP1	1149.00	1095.00	1162.00	1135.33	988.00
STRIP2	2363.00	2335.00	2411.00	2369.67	1399.00
STRN	833.00	782.00	954.00	856.33	767.00
STRN3	567.00	564.00	614.00	581.67	642.00
STRN4	1859.00	1811.00	1999.00	1889.67	2106.00
STS	224.00	237.00	249.00	236.67	200.00
STT3A	5703.00	5653.00	6018.00	5791.33	5464.00
STT3B	7968.00	7584.00	8124.00	7892.00	7989.00
STUB1	1980.00	1977.00	2094.00	2017.00	2139.00
STX10	825.00	879.00	934.00	879.33	1173.00
STX11	38.00	35.00	24.00	32.33	26.00
STX12	682.00	644.00	762.00	696.00	825.00

STX16	4597.00	4451.00	4965.00	4671.00	4462.00
STX16-NPEPL1	48.00	32.00	42.00	40.67	39.00
STX17	539.00	447.00	514.00	500.00	396.00
STX18	441.00	435.00	417.00	431.00	498.00
STX18-AS1	25.00	34.00	35.00	31.33	20.00
STX19	4.00	1.00	1.00	2.00	2.00
STX1A	1512.00	1484.00	1470.00	1488.67	1017.00
STX1B	788.00	831.00	845.00	821.33	551.00
STX2	784.00	790.00	880.00	818.00	909.00
STX3	1241.00	1156.00	1459.00	1285.33	1206.00
STX4	921.00	928.00	897.00	915.33	960.00
STX5	529.00	525.00	577.00	543.67	537.00
STX6	990.00	862.00	902.00	918.00	880.00
STX7	848.00	748.00	939.00	845.00	1060.00
STX8	302.00	316.00	344.00	320.67	435.00
STXBP1	1541.00	1452.00	1567.00	1520.00	1511.00
STXBP2	5.00	7.00	4.00	5.33	2.00
STXBP3	763.00	695.00	863.00	773.67	917.00
STXBP4	196.00	194.00	186.00	192.00	233.00
STXBP5	755.00	700.00	889.00	781.33	671.00
STXBP5-AS1	273.00	287.00	333.00	297.67	310.00
STYK1	87.00	72.00	53.00	70.67	285.00
STYX	405.00	347.00	392.00	381.33	440.00
STYXL1	870.00	845.00	921.00	878.67	1071.00
SUB1	2012.00	1897.00	2219.00	2042.67	2597.00
SUCLA2	408.00	417.00	505.00	443.33	482.00
SUCLG1	999.00	955.00	952.00	968.67	1300.00
SUCLG2	2064.00	1757.00	2094.00	1971.67	1919.00
SUCLG2-AS1	156.00	141.00	189.00	162.00	117.00
SUCO	1963.00	1757.00	1916.00	1878.67	1663.00
SUDS3	1127.00	1054.00	1113.00	1098.00	1338.00
SUFU	654.00	591.00	669.00	638.00	516.00
SUGCT	92.00	90.00	115.00	99.00	136.00
SUGP1	725.00	778.00	736.00	746.33	816.00
SUGP2	4166.00	3936.00	4323.00	4141.67	3508.00
SUGT1	691.00	670.00	720.00	693.67	995.00
SUGT1P1	142.00	153.00	185.00	160.00	186.00
SUGT1P3	9.00	0.00	6.00	5.00	10.00
SULT1C2	159.00	185.00	208.00	184.00	62.00
SUMF1	771.00	728.00	699.00	732.67	715.00
SUMF2	6814.00	6890.00	6631.00	6778.33	7988.00
SUMO1	1357.00	1224.00	1462.00	1347.67	1971.00
SUMO1P3	22.00	23.00	24.00	23.00	33.00
SUMO2	1468.00	1363.00	1548.00	1459.67	2045.00
SUMO3	1682.00	1643.00	1792.00	1705.67	2340.00
SUN1	4347.00	4374.00	4641.00	4454.00	4054.00
SUN2	1864.00	2003.00	1851.00	1906.00	1762.00
SUOX	299.00	350.00	325.00	324.67	378.00

SUPT16H	4697.00	4517.00	4881.00	4698.33	5388.00
SUPT20H	1191.00	1147.00	1282.00	1206.67	1279.00
SUPT3H	610.00	570.00	685.00	621.67	729.00
SUPT4H1	1026.00	921.00	1012.00	986.33	1144.00
SUPT5H	3113.00	3197.00	3449.00	3253.00	2940.00
SUPT6H	4196.00	4152.00	4396.00	4248.00	3878.00
SUPT7L	2840.00	2825.00	2983.00	2882.67	2812.00
SUPV3L1	1228.00	1147.00	1278.00	1217.67	1387.00
SURF1	507.00	622.00	513.00	547.33	644.00
SURF2	285.00	314.00	340.00	313.00	412.00
SURF4	9243.00	9438.00	8871.00	9184.00	8481.00
SURF6	1205.00	1218.00	1288.00	1237.00	1550.00
SUSD1	144.00	114.00	112.00	123.33	130.00
SUSD2	7.00	5.00	10.00	7.33	7.00
SUSD5	106.00	87.00	105.00	99.33	82.00
SUSD6	532.00	539.00	528.00	533.00	506.00
SUV39H1	439.00	391.00	424.00	418.00	535.00
SUV39H2	438.00	387.00	404.00	409.67	516.00
SUV420H1	857.00	746.00	921.00	841.33	840.00
SUV420H2	239.00	266.00	273.00	259.33	260.00
SUZ12	1056.00	971.00	1130.00	1052.33	1046.00
SUZ12P1	203.00	216.00	220.00	213.00	240.00
SV2A	1184.00	1119.00	1165.00	1156.00	861.00
SVBP	74.00	79.00	79.00	77.33	124.00
SVEP1	94.00	89.00	90.00	91.00	103.00
SVIL	972.00	835.00	949.00	918.67	954.00
SVIL-AS1	624.00	551.00	611.00	595.33	608.00
SVILP1	79.00	91.00	103.00	91.00	53.00
SVIP	290.00	288.00	336.00	304.67	515.00
SWAP70	966.00	868.00	1018.00	950.67	1183.00
SWI5	725.00	664.00	732.00	707.00	915.00
SWSAP1	33.00	38.00	22.00	31.00	49.00
SWT1	174.00	160.00	179.00	171.00	169.00
SYAP1	1147.00	1081.00	1156.00	1128.00	1398.00
SYBU	24.00	39.00	40.00	34.33	51.00
SYCE1L	3.00	2.00	1.00	2.00	4.00
SYCP2	25.00	27.00	42.00	31.33	46.00
SYCP2L	57.00	34.00	38.00	43.00	51.00
SYCP3	4.00	5.00	4.00	4.33	2.00
SYDE1	912.00	918.00	837.00	889.00	932.00
SYDE2	101.00	92.00	120.00	104.33	129.00
SYF2	855.00	811.00	992.00	886.00	1141.00
SYMPK	2763.00	2845.00	2940.00	2849.33	3031.00
SYNC	329.00	318.00	319.00	322.00	433.00
SYNCRIP	7457.00	6707.00	7622.00	7262.00	8218.00
SYNE1	1408.00	1395.00	1587.00	1463.33	1347.00
SYNE2	373.00	355.00	354.00	360.67	415.00
SYNE3	208.00	200.00	212.00	206.67	111.00

SYNE4	39.00	41.00	46.00	42.00	57.00
SYNGAP1	176.00	106.00	136.00	139.33	66.00
SYNGR1	310.00	298.00	299.00	302.33	220.00
SYNGR2	1195.00	1207.00	1058.00	1153.33	1305.00
SYNGR3	11.00	11.00	4.00	8.67	10.00
SYNJ1	393.00	321.00	360.00	358.00	306.00
SYNJ2	1976.00	2029.00	2191.00	2065.33	1476.00
SYNJ2BP	714.00	672.00	742.00	709.33	718.00
SYNM	433.00	385.00	452.00	423.33	397.00
SYNPO	1610.00	1536.00	1634.00	1593.33	930.00
SYNPO2	12.00	22.00	16.00	16.67	7.00
SYNPR-AS1	6.00	3.00	3.00	4.00	1.00
SYNRG	5.00	4.00	4.00	4.33	7.00
SYP	38.00	45.00	47.00	43.33	54.00
SYPL1	2746.00	2801.00	2776.00	2774.33	3289.00
SYPL2	10.00	7.00	8.00	8.33	12.00
SYS1	711.00	703.00	642.00	685.33	759.00
SYT1	501.00	443.00	542.00	495.33	531.00
SYT11	866.00	832.00	910.00	869.33	883.00
SYT14	90.00	105.00	118.00	104.33	104.00
SYT16	52.00	41.00	51.00	48.00	61.00
SYT17	3.00	2.00	2.00	2.33	0.00
SYT6	4.00	7.00	6.00	5.67	10.00
SYT9	116.00	76.00	111.00	101.00	141.00
SYTL1	10.00	10.00	10.00	10.00	41.00
SYTL2	654.00	628.00	741.00	674.33	841.00
SYTL3	244.00	254.00	227.00	241.67	195.00
SYTL4	123.00	112.00	112.00	115.67	122.00
SYVN1	3217.00	3097.00	3100.00	3138.00	2595.00
SZRD1	3259.00	3266.00	3401.00	3308.67	3752.00
SZT2	1841.00	1824.00	2015.00	1893.33	1380.00
TAB1	584.00	619.00	613.00	605.33	592.00
TAB2	1632.00	1454.00	1743.00	1609.67	1686.00
TAB3	445.00	420.00	516.00	460.33	449.00
TACC1	3288.00	3142.00	3368.00	3266.00	1935.00
TACC2	1237.00	1189.00	1292.00	1239.33	1219.00
TACC3	8151.00	8261.00	7850.00	8087.33	9437.00
TACO1	580.00	501.00	619.00	566.67	697.00
TACSTD2	19.00	26.00	33.00	26.00	4.00
TADA1	254.00	266.00	300.00	273.33	317.00
TADA2A	10.00	7.00	22.00	13.00	14.00
TADA2B	530.00	485.00	578.00	531.00	531.00
TADA3	2324.00	2170.00	2352.00	2282.00	2827.00
TAF1	2265.00	1995.00	2299.00	2186.33	1724.00
TAF10	707.00	618.00	692.00	672.33	888.00
TAF11	633.00	608.00	640.00	627.00	720.00
TAF12	476.00	485.00	507.00	489.33	532.00
TAF13	186.00	158.00	207.00	183.67	236.00

TAF15	22.00	10.00	7.00	13.00	13.00
TAF1A	399.00	350.00	350.00	366.33	409.00
TAF1A-AS1	47.00	30.00	30.00	35.67	38.00
TAF1B	456.00	380.00	398.00	411.33	594.00
TAF1C	2321.00	2405.00	2414.00	2380.00	2377.00
TAF1D	1904.00	1951.00	2181.00	2012.00	2115.00
TAF1L	3.00	4.00	5.00	4.00	2.00
TAF2	1674.00	1493.00	1837.00	1668.00	1594.00
TAF3	296.00	245.00	270.00	270.33	272.00
TAF4	119.00	119.00	129.00	122.33	110.00
TAF4B	608.00	540.00	612.00	586.67	690.00
TAF5	219.00	177.00	219.00	205.00	253.00
TAF5L	699.00	673.00	710.00	694.00	760.00
TAF6	1650.00	1528.00	1707.00	1628.33	1675.00
TAF6L	408.00	414.00	431.00	417.67	476.00
TAF7	2024.00	1864.00	2077.00	1988.33	2171.00
TAF8	613.00	507.00	534.00	551.33	563.00
TAF9	11.00	5.00	0.00	5.33	0.00
TAF9B	676.00	562.00	712.00	650.00	846.00
TAGLN	61.00	68.00	94.00	74.33	61.00
TAGLN2	4455.00	4362.00	4348.00	4388.33	4844.00
TAGLN3	640.00	591.00	649.00	626.67	919.00
TAL2	5.00	7.00	3.00	5.00	7.00
TALDO1	2862.00	2634.00	2851.00	2782.33	3506.00
TAMM41	226.00	207.00	237.00	223.33	262.00
TANC1	851.00	821.00	955.00	875.67	674.00
TANC2	1571.00	1433.00	1752.00	1585.33	974.00
TANGO2	385.00	374.00	370.00	376.33	400.00
TANGO6	413.00	429.00	459.00	433.67	463.00
TANK	653.00	677.00	745.00	691.67	838.00
TAOK1	1730.00	1569.00	2017.00	1772.00	1611.00
TAOK2	1952.00	1946.00	2048.00	1982.00	1536.00
TAOK3	1580.00	1449.00	1551.00	1526.67	2146.00
TAP1	3.00	1.00	1.00	1.67	1.00
TAP2	14.00	12.00	5.00	10.33	9.00
TAPBP	30.00	22.00	29.00	27.00	23.00
TAPBPL	241.00	244.00	225.00	236.67	211.00
TAPT1	388.00	362.00	366.00	372.00	348.00
TAPT1-AS1	73.00	62.00	82.00	72.33	77.00
TARBP1	1787.00	1576.00	1956.00	1773.00	1626.00
TARBP2	723.00	719.00	872.00	771.33	829.00
TARDBP	4284.00	4292.00	4442.00	4339.33	4382.00
TARID	8.00	5.00	11.00	8.00	11.00
TARS	10354.00	9634.00	11297.00	10428.33	11752.00
TARS2	1252.00	1170.00	1306.00	1242.67	1236.00
TARSL2	230.00	236.00	258.00	241.33	217.00
TAS2R19	4.00	3.00	1.00	2.67	10.00
TAS2R4	10.00	22.00	18.00	16.67	12.00

TAS2R5	112.00	109.00	132.00	117.67	111.00
TASP1	216.00	180.00	205.00	200.33	253.00
TATDN1	973.00	880.00	998.00	950.33	1332.00
TATDN2	1058.00	1000.00	1080.00	1046.00	944.00
TATDN3	267.00	241.00	274.00	260.67	374.00
TAX1BP1	2735.00	2466.00	2759.00	2653.33	2937.00
TAX1BP3	151.00	170.00	153.00	158.00	199.00
TAZ	1255.00	1212.00	1312.00	1259.67	1281.00
TBC1D1	1452.00	1513.00	1517.00	1494.00	1451.00
TBC1D10A	250.00	282.00	214.00	248.67	302.00
TBC1D10B	1220.00	1291.00	1302.00	1271.00	1222.00
TBC1D12	305.00	275.00	290.00	290.00	293.00
TBC1D13	990.00	949.00	977.00	972.00	1012.00
TBC1D14	1649.00	1596.00	1796.00	1680.33	1762.00
TBC1D15	745.00	712.00	851.00	769.33	776.00
TBC1D16	2227.00	2395.00	2426.00	2349.33	1795.00
TBC1D17	513.00	477.00	557.00	515.67	593.00
TBC1D19	51.00	43.00	53.00	49.00	64.00
TBC1D2	206.00	229.00	211.00	215.33	234.00
TBC1D20	1591.00	1620.00	1751.00	1654.00	1319.00
TBC1D22A	423.00	425.00	476.00	441.33	465.00
TBC1D22B	316.00	334.00	380.00	343.33	459.00
TBC1D23	1906.00	1651.00	1873.00	1810.00	2902.00
TBC1D24	839.00	797.00	828.00	821.33	775.00
TBC1D25	374.00	399.00	401.00	391.33	452.00
TBC1D2B	1407.00	1417.00	1547.00	1457.00	966.00
TBC1D30	177.00	178.00	181.00	178.67	207.00
TBC1D31	466.00	398.00	469.00	444.33	518.00
TBC1D32	287.00	289.00	349.00	308.33	303.00
TBC1D3P1-DHX40P1	3.00	3.00	2.00	2.67	1.00
TBC1D4	878.00	816.00	949.00	881.00	1013.00
TBC1D5	2464.00	2231.00	2644.00	2446.33	2219.00
TBC1D7	236.00	209.00	232.00	225.67	214.00
TBC1D8	663.00	618.00	722.00	667.67	515.00
TBC1D8B	370.00	322.00	419.00	370.33	344.00
TBC1D9	818.00	716.00	856.00	796.67	763.00
TBC1D9B	4399.00	4371.00	4793.00	4521.00	4148.00
TBCA	1416.00	1362.00	1428.00	1402.00	2109.00
TBCB	992.00	949.00	992.00	977.67	1268.00
TBCC	532.00	503.00	547.00	527.33	665.00
TBCCD1	250.00	243.00	233.00	242.00	273.00
TBCD	985.00	930.00	997.00	970.67	850.00
TBCE	688.00	671.00	695.00	684.67	888.00
TBCEL	600.00	636.00	670.00	635.33	621.00
TBCK	551.00	569.00	650.00	590.00	498.00
TBK1	883.00	882.00	1002.00	922.33	1123.00
TBKBP1	123.00	146.00	160.00	143.00	131.00
TBL1X	815.00	749.00	875.00	813.00	780.00

TBL1XR1	2199.00	2071.00	2557.00	2275.67	2686.00
TBL2	1371.00	1373.00	1256.00	1333.33	1439.00
TBL3	1100.00	1100.00	1033.00	1077.67	1182.00
TBP	390.00	363.00	403.00	385.33	496.00
TBPL1	564.00	527.00	573.00	554.67	752.00
TBRG1	1139.00	1093.00	1203.00	1145.00	1116.00
TBRG4	3438.00	3331.00	3642.00	3470.33	3762.00
TBX18	848.00	840.00	935.00	874.33	883.00
TBX19	82.00	85.00	95.00	87.33	80.00
TBX2	348.00	385.00	362.00	365.00	371.00
TBX2-AS1	74.00	114.00	104.00	97.33	97.00
TBX3	2829.00	2856.00	3208.00	2964.33	2264.00
TBX6	34.00	51.00	52.00	45.67	54.00
TBXA2R	24.00	24.00	27.00	25.00	22.00
TBXAS1	223.00	214.00	232.00	223.00	218.00
TCAF1	1299.00	1322.00	1508.00	1376.33	1084.00
TCAF2	47.00	57.00	43.00	49.00	77.00
TCAIM	585.00	513.00	605.00	567.67	562.00
TCAM1P	36.00	41.00	39.00	38.67	51.00
TCAP	14.00	18.00	13.00	15.00	3.00
TCEA1	3286.00	2997.00	3717.00	3333.33	4183.00
TCEA2	449.00	440.00	517.00	468.67	412.00
TCEA3	4.00	6.00	10.00	6.67	12.00
TCEAL1	448.00	459.00	461.00	456.00	391.00
TCEAL3	385.00	404.00	414.00	401.00	398.00
TCEAL4	2084.00	2140.00	2240.00	2154.67	2609.00
TCEAL8	1239.00	1206.00	1374.00	1273.00	1644.00
TCEANC	59.00	80.00	98.00	79.00	87.00
TCEANC2	504.00	438.00	444.00	462.00	400.00
TCEB1	1255.00	1174.00	1307.00	1245.33	1683.00
TCEB2	1295.00	1260.00	1344.00	1299.67	1641.00
TCEB3	1958.00	1873.00	2086.00	1972.33	1997.00
TCEB3-AS1	78.00	101.00	90.00	89.67	96.00
TCERG1	3118.00	3083.00	3222.00	3141.00	3455.00
TCF12	1910.00	1577.00	2015.00	1834.00	1886.00
TCF24	10.00	12.00	13.00	11.67	11.00
TCF25	2591.00	2545.00	2728.00	2621.33	2558.00
TCF3	2310.00	2229.00	2437.00	2325.33	2734.00
TCF4	378.00	313.00	359.00	350.00	319.00
TCF7	29.00	31.00	31.00	30.33	39.00
TCF7L1	5.00	7.00	14.00	8.67	12.00
TCF7L2	513.00	491.00	559.00	521.00	519.00
TCFL5	889.00	942.00	1030.00	953.67	1157.00
TCHP	885.00	928.00	934.00	915.67	1035.00
TCIRG1	1082.00	1136.00	1106.00	1108.00	1014.00
TCN1	3.00	2.00	2.00	2.33	15.00
TCN2	43.00	72.00	55.00	56.67	138.00
TCOF1	4253.00	4180.00	4323.00	4252.00	4229.00

TCONS_00029157	4.00	6.00	6.00	5.33	3.00
TCP1	5651.00	5363.00	5868.00	5627.33	7730.00
TCP11	8.00	5.00	6.00	6.33	11.00
TCP11L1	328.00	276.00	268.00	290.67	273.00
TCP11L2	127.00	95.00	91.00	104.33	74.00
TCTA	436.00	392.00	434.00	420.67	382.00
TCTEX1D2	192.00	179.00	207.00	192.67	237.00
TCTN1	1074.00	930.00	998.00	1000.67	983.00
TCTN2	211.00	214.00	206.00	210.33	193.00
TCTN3	1823.00	1653.00	1731.00	1735.67	1746.00
TDG	496.00	534.00	581.00	537.00	544.00
TDP1	921.00	880.00	967.00	922.67	842.00
TDP2	1017.00	974.00	1113.00	1034.67	1278.00
TDRD3	272.00	316.00	361.00	316.33	354.00
TDRD6	31.00	50.00	39.00	40.00	29.00
TDRD7	115.00	122.00	120.00	119.00	127.00
TDRKH	217.00	224.00	240.00	227.00	318.00
TDRP	135.00	124.00	140.00	133.00	127.00
TEAD1	2812.00	2553.00	3058.00	2807.67	2071.00
TEAD2	78.00	85.00	86.00	83.00	96.00
TEAD3	359.00	341.00	345.00	348.33	321.00
TEAD4	733.00	812.00	919.00	821.33	916.00
TEC	125.00	108.00	120.00	117.67	93.00
TECPR1	1008.00	998.00	1124.00	1043.33	1047.00
TECPR2	884.00	839.00	914.00	879.00	680.00
TECR	2197.00	2355.00	1978.00	2176.67	2631.00
TECTA	10.00	21.00	27.00	19.33	23.00
TECTB	4.00	5.00	4.00	4.33	2.00
TEF	110.00	97.00	121.00	109.33	125.00
TEFM	208.00	223.00	214.00	215.00	222.00
TEKT4P2	60.00	45.00	51.00	52.00	46.00
TELO2	1047.00	1078.00	1066.00	1063.67	1163.00
TEN1-CDK3	51.00	71.00	61.00	61.00	68.00
TENM1	375.00	354.00	438.00	389.00	260.00
TENM2	1081.00	893.00	1087.00	1020.33	665.00
TENM3	1223.00	1183.00	1272.00	1226.00	1153.00
TENM4	261.00	199.00	284.00	248.00	170.00
TEP1	1493.00	1369.00	1477.00	1446.33	1200.00
TERF1	574.00	550.00	616.00	580.00	676.00
TERF2	671.00	642.00	736.00	683.00	784.00
TERF2IP	1064.00	1006.00	1113.00	1061.00	1350.00
TERT	220.00	238.00	235.00	231.00	281.00
TES	345.00	358.00	341.00	348.00	317.00
TESK1	461.00	515.00	531.00	502.33	481.00
TESK2	86.00	83.00	108.00	92.33	60.00
TET1	65.00	63.00	55.00	61.00	58.00
TET2	316.00	268.00	321.00	301.67	293.00
TET3	1277.00	1143.00	1396.00	1272.00	1062.00

TEX10	1423.00	1251.00	1417.00	1363.67	1641.00
TEX12	5.00	4.00	6.00	5.00	5.00
TEX14	7.00	4.00	7.00	6.00	8.00
TEX15	63.00	66.00	98.00	75.67	128.00
TEX19	33.00	31.00	40.00	34.67	18.00
TEX2	1365.00	1283.00	1315.00	1321.00	1251.00
TEX21P	24.00	14.00	16.00	18.00	14.00
TEX22	33.00	32.00	31.00	32.00	44.00
TEX261	2837.00	2799.00	2856.00	2830.67	2680.00
TEX264	1294.00	1296.00	1232.00	1274.00	1353.00
TEX30	344.00	344.00	374.00	354.00	541.00
TEX41	16.00	13.00	16.00	15.00	7.00
TEX9	70.00	50.00	79.00	66.33	101.00
TF	3.00	3.00	4.00	3.33	1.00
TFAM	1773.00	1627.00	1712.00	1704.00	2203.00
TFAP2A	338.00	320.00	385.00	347.67	386.00
TFAP2A-AS1	19.00	20.00	23.00	20.67	15.00
TFAP2C	287.00	313.00	308.00	302.67	315.00
TFAP2E	7.00	10.00	9.00	8.67	3.00
TFAP4	653.00	686.00	771.00	703.33	815.00
TFB1M	526.00	518.00	550.00	531.33	638.00
TFB2M	660.00	619.00	738.00	672.33	825.00
TFCP2	900.00	913.00	958.00	923.67	1086.00
TFDP1	1745.00	1660.00	1859.00	1754.67	2295.00
TFDP2	787.00	724.00	809.00	773.33	878.00
TFE3	2476.00	2442.00	2426.00	2448.00	2817.00
TFEB	12.00	13.00	11.00	12.00	7.00
TFG	2832.00	2696.00	2940.00	2822.67	3064.00
TFIP11	910.00	931.00	966.00	935.67	908.00
TFPI	17535.00	17024.00	18755.00	17771.33	14801.00
TFPI2	5412.00	5301.00	5137.00	5283.33	7130.00
TFPT	10.00	7.00	11.00	9.33	5.00
TFR2	44.00	65.00	54.00	54.33	51.00
TFRC	6775.00	6402.00	6537.00	6571.33	5802.00
TG	4.00	4.00	12.00	6.67	6.00
TGDS	308.00	289.00	328.00	308.33	401.00
TGFB1	1687.00	1764.00	1675.00	1708.67	1501.00
TGFB1I1	1096.00	1065.00	1083.00	1081.33	1116.00
TGFB2	544.00	457.00	496.00	499.00	361.00
TGFB2-AS1	18.00	7.00	15.00	13.33	18.00
TGFB3	13.00	23.00	8.00	14.67	16.00
TGFBI	2113.00	1991.00	2307.00	2137.00	1176.00
TGFBR1	652.00	570.00	632.00	618.00	620.00
TGFBR2	7410.00	6669.00	7245.00	7108.00	6374.00
TGFBR3	298.00	322.00	346.00	322.00	268.00
TGFBR3L	17.00	16.00	36.00	23.00	26.00
TGFBRAP1	1050.00	981.00	1090.00	1040.33	1129.00
TGIF1	1100.00	1069.00	1256.00	1141.67	1058.00

TGIF2	462.00	497.00	445.00	468.00	767.00
TGM1	31.00	39.00	39.00	36.33	23.00
TGM2	18265.00	17660.00	19049.00	18324.67	18381.00
TGM4	5.00	5.00	8.00	6.00	1.00
TGOLN2	9540.00	8718.00	9347.00	9201.67	6681.00
TGS1	806.00	777.00	834.00	805.67	834.00
THADA	1656.00	1665.00	1781.00	1700.67	1567.00
THAP1	202.00	228.00	251.00	227.00	264.00
THAP10	147.00	129.00	132.00	136.00	142.00
THAP11	630.00	574.00	630.00	611.33	707.00
THAP2	148.00	126.00	144.00	139.33	150.00
THAP3	363.00	338.00	392.00	364.33	404.00
THAP4	1227.00	1246.00	1284.00	1252.33	1475.00
THAP5	369.00	427.00	439.00	411.67	516.00
THAP6	221.00	223.00	272.00	238.67	315.00
THAP7	251.00	272.00	277.00	266.67	316.00
THAP7-AS1	40.00	34.00	37.00	37.00	36.00
THAP8	71.00	48.00	51.00	56.67	69.00
THAP9	95.00	83.00	100.00	92.67	75.00
THAP9-AS1	1211.00	1210.00	1394.00	1271.67	1626.00
THBS1	1680.00	1556.00	1805.00	1680.33	1641.00
THBS2	5.00	7.00	16.00	9.33	8.00
THBS3	861.00	841.00	935.00	879.00	991.00
THBS4	24.00	22.00	25.00	23.67	18.00
THCAT158	7.00	3.00	8.00	6.00	8.00
THEGL	22.00	16.00	19.00	19.00	4.00
THEM4	509.00	526.00	580.00	538.33	576.00
THEM6	976.00	1123.00	1176.00	1091.67	1170.00
THEMIS2	16.00	28.00	30.00	24.67	8.00
THG1L	306.00	377.00	395.00	359.33	457.00
THNSL1	712.00	667.00	744.00	707.67	730.00
THNSL2	83.00	90.00	76.00	83.00	74.00
THOC1	2521.00	2626.00	2761.00	2636.00	3040.00
THOC2	1671.00	1489.00	1694.00	1618.00	1548.00
THOC3	255.00	282.00	252.00	263.00	316.00
THOC5	1239.00	1157.00	1325.00	1240.33	1413.00
THOC6	774.00	814.00	794.00	794.00	1033.00
THOC7	900.00	873.00	908.00	893.67	1154.00
THOP1	2029.00	2029.00	2115.00	2057.67	2313.00
THRA	270.00	275.00	327.00	290.67	294.00
THRAP3	4020.00	3883.00	4189.00	4030.67	4161.00
THRSP	3.00	1.00	2.00	2.00	1.00
THSD1	96.00	73.00	110.00	93.00	124.00
THSD4	1933.00	1823.00	2144.00	1966.67	1110.00
THSD7A	3.00	4.00	5.00	4.00	3.00
THTPA	354.00	441.00	444.00	413.00	304.00
THUMPD1	978.00	918.00	1064.00	986.67	1147.00
THUMPD2	449.00	452.00	571.00	490.67	541.00

THUMPD3	968.00	901.00	1047.00	972.00	1059.00
THUMPD3-AS1	575.00	541.00	610.00	575.33	525.00
THY1	825.00	767.00	684.00	758.67	736.00
THYN1	388.00	408.00	405.00	400.33	472.00
TIA1	2450.00	2562.00	2794.00	2602.00	2467.00
TIAF1	18.00	13.00	19.00	16.67	5.00
TIAL1	3884.00	3938.00	4266.00	4029.33	4727.00
TIAM1	404.00	321.00	432.00	385.67	331.00
TIAM2	24.00	20.00	34.00	26.00	15.00
TICAM1	389.00	400.00	449.00	412.67	458.00
TICAM2	3.00	2.00	2.00	2.33	1.00
TICRR	578.00	524.00	513.00	538.33	612.00
TIE1	14927.00	15295.00	14959.00	15060.33	13062.00
TIFA	192.00	171.00	174.00	179.00	243.00
TIGAR	368.00	332.00	372.00	357.33	448.00
TIGD1	272.00	263.00	322.00	285.67	288.00
TIGD2	182.00	151.00	203.00	178.67	190.00
TIGD3	6.00	6.00	10.00	7.33	10.00
TIGD4	18.00	25.00	27.00	23.33	24.00
TIGD6	156.00	160.00	161.00	159.00	166.00
TIGD7	21.00	26.00	20.00	22.33	28.00
TIMELESS	1264.00	1233.00	1250.00	1249.00	1518.00
TIMM10	407.00	383.00	437.00	409.00	548.00
TIMM10B	639.00	658.00	680.00	659.00	673.00
TIMM13	686.00	693.00	788.00	722.33	1019.00
TIMM17A	919.00	913.00	886.00	906.00	1323.00
TIMM17B	717.00	804.00	722.00	747.67	833.00
TIMM21	516.00	532.00	539.00	529.00	701.00
TIMM23	1310.00	1273.00	1349.00	1310.67	1809.00
TIMM23B	391.00	333.00	422.00	382.00	358.00
TIMM44	1569.00	1512.00	1637.00	1572.67	1768.00
TIMM50	967.00	875.00	945.00	929.00	1091.00
TIMM8A	215.00	182.00	231.00	209.33	245.00
TIMM8B	304.00	319.00	311.00	311.33	428.00
TIMM9	575.00	630.00	642.00	615.67	843.00
TIMMDC1	2100.00	1972.00	1981.00	2017.67	2272.00
TIMP1	8176.00	8339.00	7602.00	8039.00	9081.00
TIMP2	6350.00	5973.00	6010.00	6111.00	5336.00
TIMP3	4655.00	4360.00	4095.00	4370.00	3938.00
TINAGL1	20.00	23.00	31.00	24.67	9.00
TINCR	13.00	33.00	20.00	22.00	22.00
TINF2	593.00	599.00	636.00	609.33	666.00
TIPARP	756.00	685.00	782.00	741.00	801.00
TIPARP-AS1	45.00	32.00	38.00	38.33	46.00
TIPIN	237.00	245.00	242.00	241.33	320.00
TIPRL	1217.00	1162.00	1244.00	1207.67	1626.00
TIRAP	70.00	109.00	86.00	88.33	68.00
TJAP1	1716.00	1629.00	1732.00	1692.33	1736.00

TJP1	85.00	83.00	79.00	82.33	70.00
TJP2	1593.00	1532.00	1711.00	1612.00	2031.00
TK1	285.00	287.00	237.00	269.67	516.00
TK2	1011.00	997.00	1110.00	1039.33	1047.00
TKFC	1154.00	1214.00	1257.00	1208.33	1337.00
TKT	7574.00	7314.00	7840.00	7576.00	6870.00
TLCD1	115.00	125.00	98.00	112.67	145.00
TLDC1	2539.00	2430.00	2623.00	2530.67	2408.00
TLE1	2156.00	2114.00	2195.00	2155.00	2315.00
TLE3	793.00	739.00	814.00	782.00	693.00
TLE4	188.00	221.00	189.00	199.33	248.00
TLK1	898.00	761.00	1026.00	895.00	1025.00
TLK2	1060.00	1051.00	1168.00	1093.00	1130.00
TLL1	7.00	2.00	5.00	4.67	4.00
TLL2	3.00	1.00	5.00	3.00	3.00
TLN1	19645.00	18730.00	20019.00	19464.67	12875.00
TLN2	285.00	262.00	303.00	283.33	388.00
TLR1	16.00	17.00	24.00	19.00	22.00
TLR3	15.00	26.00	18.00	19.67	24.00
TLR4	48.00	57.00	55.00	53.33	9.00
TLR6	445.00	428.00	485.00	452.67	552.00
TLR9	5.00	1.00	2.00	2.67	1.00
TM2D1	390.00	366.00	391.00	382.33	457.00
TM2D2	945.00	929.00	908.00	927.33	1018.00
TM2D3	541.00	494.00	532.00	522.33	656.00
TM4SF1	11375.00	10737.00	9965.00	10692.33	25975.00
TM4SF18	364.00	314.00	319.00	332.33	480.00
TM4SF19	58.00	64.00	72.00	64.67	32.00
TM4SF19-AS1	24.00	24.00	23.00	23.67	40.00
TM4SF1-AS1	5.00	3.00	3.00	3.67	10.00
TM6SF1	9.00	10.00	14.00	11.00	3.00
TM7SF2	215.00	233.00	231.00	226.33	224.00
TM7SF3	2903.00	2792.00	2952.00	2882.33	3571.00
TM9SF1	2645.00	2601.00	2644.00	2630.00	2630.00
TM9SF2	4427.00	4217.00	4520.00	4388.00	4263.00
TM9SF3	9624.00	8955.00	9711.00	9430.00	10531.00
TM9SF4	4727.00	4657.00	4800.00	4728.00	4180.00
TMA16	401.00	399.00	425.00	408.33	597.00
TMA7	983.00	1020.00	1103.00	1035.33	1548.00
TMBIM1	2820.00	2610.00	2700.00	2710.00	2921.00
TMBIM4	1208.00	1155.00	1172.00	1178.33	1453.00
TMBIM6	28741.00	27001.00	27334.00	27692.00	25434.00
TMC3	3.00	6.00	5.00	4.67	1.00
TMC3-AS1	25.00	31.00	22.00	26.00	23.00
TMC6	42.00	35.00	36.00	37.67	32.00
TMC7	245.00	262.00	270.00	259.00	364.00
TMC8	6.00	5.00	1.00	4.00	0.00
TMCC1	407.00	379.00	468.00	418.00	466.00

TMCC1-AS1	668.00	675.00	706.00	683.00	769.00
TMCC2	73.00	62.00	62.00	65.67	86.00
TMCC3	334.00	277.00	346.00	319.00	290.00
TMCO1	2405.00	2300.00	2270.00	2325.00	2795.00
TMCO3	1002.00	976.00	1010.00	996.00	814.00
TMCO4	263.00	268.00	323.00	284.67	329.00
TMCO6	669.00	636.00	676.00	660.33	618.00
TMED1	814.00	852.00	764.00	810.00	832.00
TMED10	6545.00	6094.00	6225.00	6288.00	6632.00
TMED2	4304.00	4072.00	4161.00	4179.00	4677.00
TMED3	1727.00	1707.00	1655.00	1696.33	2243.00
TMED4	3330.00	3399.00	3285.00	3338.00	3569.00
TMED5	2505.00	2344.00	2725.00	2524.67	2603.00
TMED6	17.00	17.00	6.00	13.33	8.00
TMED7	1278.00	1188.00	1300.00	1255.33	1384.00
TMED7-TICAM2	11.00	16.00	17.00	14.67	9.00
TMED8	66.00	45.00	57.00	56.00	49.00
TMED9	5304.00	5613.00	5099.00	5338.67	5554.00
TMEFF2	40.00	20.00	23.00	27.67	25.00
TMEM101	1283.00	1264.00	1315.00	1287.33	1544.00
TMEM102	65.00	50.00	48.00	54.33	43.00
TMEM104	846.00	910.00	852.00	869.33	712.00
TMEM106A	167.00	181.00	162.00	170.00	178.00
TMEM106B	2168.00	1844.00	2135.00	2049.00	1844.00
TMEM106C	1181.00	1106.00	1196.00	1161.00	1430.00
TMEM107	498.00	494.00	428.00	473.33	558.00
TMEM108	197.00	175.00	200.00	190.67	183.00
TMEM108-AS1	380.00	404.00	342.00	375.33	316.00
TMEM109	3000.00	3002.00	2779.00	2927.00	3539.00
TMEM11	338.00	329.00	374.00	347.00	406.00
TMEM110	617.00	621.00	662.00	633.33	530.00
TMEM115	1891.00	1993.00	1919.00	1934.33	1860.00
TMEM116	218.00	203.00	209.00	210.00	294.00
TMEM117	61.00	88.00	91.00	80.00	63.00
TMEM120A	421.00	416.00	436.00	424.33	522.00
TMEM120B	2049.00	2178.00	2163.00	2130.00	1795.00
TMEM121	11.00	12.00	26.00	16.33	30.00
TMEM123	14927.00	13162.00	14887.00	14325.33	13421.00
TMEM126A	250.00	238.00	259.00	249.00	302.00
TMEM126B	576.00	514.00	629.00	573.00	737.00
TMEM127	1702.00	1676.00	1644.00	1674.00	1492.00
TMEM128	280.00	304.00	303.00	295.67	366.00
TMEM129	6501.00	6620.00	6398.00	6506.33	7167.00
TMEM131	2968.00	2841.00	3076.00	2961.67	2354.00
TMEM132A	2418.00	2431.00	2412.00	2420.33	2532.00
TMEM132B	4.00	8.00	1.00	4.33	4.00
TMEM133	488.00	473.00	525.00	495.33	523.00
TMEM134	203.00	199.00	239.00	213.67	260.00

TMEM135	284.00	219.00	310.00	271.00	289.00
TMEM136	470.00	431.00	503.00	468.00	528.00
TMEM138	861.00	864.00	900.00	875.00	1000.00
TMEM140	30.00	29.00	41.00	33.33	32.00
TMEM141	323.00	320.00	351.00	331.33	443.00
TMEM143	118.00	122.00	139.00	126.33	134.00
TMEM144	149.00	118.00	143.00	136.67	230.00
TMEM145	24.00	26.00	27.00	25.67	29.00
TMEM147	1430.00	1419.00	1487.00	1445.33	1694.00
TMEM147-AS1	950.00	1095.00	1067.00	1037.33	1209.00
TMEM14A	250.00	234.00	213.00	232.33	407.00
TMEM14B	1663.00	1558.00	1491.00	1570.67	2162.00
TMEM14C	2168.00	2164.00	2160.00	2164.00	2734.00
TMEM150A	419.00	433.00	444.00	432.00	434.00
TMEM150C	16.00	22.00	16.00	18.00	33.00
TMEM154	595.00	478.00	504.00	525.67	466.00
TMEM156	3708.00	3438.00	3604.00	3583.33	3463.00
TMEM158	1934.00	2034.00	2193.00	2053.67	3125.00
TMEM159	385.00	330.00	351.00	355.33	330.00
TMEM160	169.00	129.00	181.00	159.67	240.00
TMEM161A	1271.00	1375.00	1337.00	1327.67	1234.00
TMEM161B	1249.00	1227.00	1368.00	1281.33	1200.00
TMEM161B-AS1	257.00	289.00	282.00	276.00	335.00
TMEM164	1643.00	1529.00	1689.00	1620.33	1352.00
TMEM165	1696.00	1545.00	1674.00	1638.33	1789.00
TMEM167A	1823.00	1685.00	2015.00	1841.00	2172.00
TMEM167B	842.00	834.00	984.00	886.67	1120.00
TMEM168	645.00	580.00	605.00	610.00	654.00
TMEM169	7.00	2.00	4.00	4.33	2.00
TMEM17	237.00	255.00	231.00	241.00	312.00
TMEM170A	813.00	710.00	833.00	785.33	883.00
TMEM170B	187.00	159.00	224.00	190.00	274.00
TMEM171	72.00	75.00	82.00	76.33	128.00
TMEM173	456.00	381.00	377.00	404.67	526.00
TMEM175	440.00	460.00	438.00	446.00	535.00
TMEM177	362.00	364.00	345.00	357.00	438.00
TMEM178A	44.00	32.00	47.00	41.00	35.00
TMEM179B	623.00	615.00	562.00	600.00	703.00
TMEM18	682.00	728.00	749.00	719.67	919.00
TMEM180	320.00	357.00	366.00	347.67	278.00
TMEM181	3478.00	3577.00	3739.00	3598.00	3718.00
TMEM182	115.00	98.00	121.00	111.33	169.00
TMEM183A	487.00	496.00	584.00	522.33	558.00
TMEM183B	43.00	20.00	54.00	39.00	43.00
TMEM184A	20.00	20.00	28.00	22.67	24.00
TMEM184B	1864.00	1844.00	1743.00	1817.00	1743.00
TMEM184C	800.00	745.00	807.00	784.00	739.00
TMEM185A	212.00	195.00	189.00	198.67	199.00

TMEM185B	3737.00	3444.00	3583.00	3588.00	3777.00
TMEM186	368.00	373.00	388.00	376.33	409.00
TMEM187	287.00	294.00	268.00	283.00	322.00
TMEM189	646.00	686.00	584.00	638.67	817.00
TMEM19	601.00	541.00	558.00	566.67	647.00
TMEM191A	3.00	6.00	2.00	3.67	5.00
TMEM191C	4.00	3.00	2.00	3.00	0.00
TMEM192	805.00	742.00	824.00	790.33	777.00
TMEM198	162.00	157.00	166.00	161.67	111.00
TMEM198B	1426.00	1407.00	1580.00	1471.00	1128.00
TMEM199	537.00	522.00	540.00	533.00	612.00
TMEM2	404.00	448.00	409.00	420.33	424.00
TMEM200A	4366.00	4136.00	4377.00	4293.00	3837.00
TMEM200B	44.00	61.00	52.00	52.33	76.00
TMEM201	965.00	941.00	928.00	944.67	1422.00
TMEM203	798.00	789.00	849.00	812.00	949.00
TMEM204	462.00	511.00	497.00	490.00	483.00
TMEM205	743.00	770.00	745.00	752.67	835.00
TMEM206	993.00	976.00	945.00	971.33	1054.00
TMEM208	632.00	663.00	540.00	611.67	718.00
TMEM209	2544.00	2371.00	2660.00	2525.00	2764.00
TMEM214	2669.00	2502.00	2408.00	2526.33	1671.00
TMEM216	213.00	210.00	198.00	207.00	158.00
TMEM217	33.00	23.00	15.00	23.67	26.00
TMEM218	265.00	292.00	280.00	279.00	287.00
TMEM219	1085.00	1120.00	1191.00	1132.00	1359.00
TMEM220	15.00	13.00	21.00	16.33	15.00
TMEM222	713.00	767.00	791.00	757.00	815.00
TMEM223	232.00	265.00	230.00	242.33	303.00
TMEM230	2351.00	2141.00	2456.00	2316.00	2921.00
TMEM231	446.00	434.00	461.00	447.00	450.00
TMEM232	44.00	47.00	37.00	42.67	31.00
TMEM234	139.00	132.00	142.00	137.67	154.00
TMEM236	86.00	74.00	96.00	85.33	62.00
TMEM237	1098.00	1053.00	1106.00	1085.67	1204.00
TMEM238	4.00	1.00	2.00	2.33	4.00
TMEM240	18.00	19.00	15.00	17.33	11.00
TMEM241	208.00	201.00	253.00	220.67	248.00
TMEM242	443.00	371.00	422.00	412.00	594.00
TMEM243	581.00	554.00	571.00	568.67	613.00
TMEM244	5.00	5.00	14.00	8.00	34.00
TMEM245	2259.00	2095.00	2389.00	2247.67	2269.00
TMEM246	1124.00	1075.00	1090.00	1096.33	1088.00
TMEM248	2570.00	2515.00	2582.00	2555.67	2355.00
TMEM249	102.00	131.00	128.00	120.33	89.00
TMEM25	6.00	0.00	5.00	3.67	1.00
TMEM254	677.00	575.00	629.00	627.00	667.00
TMEM254-AS1	53.00	60.00	81.00	64.67	50.00

TMEM255B	710.00	687.00	638.00	678.33	343.00
TMEM256	374.00	356.00	437.00	389.00	474.00
TMEM258	816.00	745.00	851.00	804.00	1085.00
TMEM259	6710.00	6776.00	6887.00	6791.00	6897.00
TMEM26	21.00	19.00	10.00	16.67	17.00
TMEM260	531.00	557.00	568.00	552.00	626.00
TMEM261	1191.00	1135.00	1205.00	1177.00	1509.00
TMEM263	1177.00	1129.00	1361.00	1222.33	1424.00
TMEM265	314.00	335.00	340.00	329.67	320.00
TMEM27	17.00	17.00	15.00	16.33	11.00
TMEM30A	6835.00	6201.00	6660.00	6565.33	5138.00
TMEM33	6521.00	6202.00	6809.00	6510.67	7243.00
TMEM38A	21.00	18.00	18.00	19.00	20.00
TMEM38B	838.00	756.00	791.00	795.00	928.00
TMEM39A	2029.00	1981.00	2185.00	2065.00	1882.00
TMEM39B	1212.00	1127.00	1120.00	1153.00	1252.00
TMEM41A	1047.00	950.00	935.00	977.33	1015.00
TMEM41B	1128.00	1067.00	1075.00	1090.00	1192.00
TMEM42	532.00	548.00	579.00	553.00	582.00
TMEM43	5693.00	5705.00	5517.00	5638.33	4571.00
TMEM44	1220.00	1265.00	1289.00	1258.00	1019.00
TMEM44-AS1	120.00	87.00	114.00	107.00	124.00
TMEM45A	175.00	197.00	200.00	190.67	181.00
TMEM47	5.00	4.00	4.00	4.33	6.00
TMEM5	863.00	882.00	917.00	887.33	1126.00
TMEM50A	1888.00	1858.00	1788.00	1844.67	2367.00
TMEM50B	6.00	1.00	13.00	6.67	2.00
TMEM51	213.00	210.00	205.00	209.33	191.00
TMEM52	3.00	0.00	3.00	2.00	3.00
TMEM53	78.00	64.00	93.00	78.33	83.00
TMEM54	458.00	462.00	459.00	459.67	541.00
TMEM55A	398.00	327.00	390.00	371.67	510.00
TMEM55B	581.00	553.00	633.00	589.00	596.00
TMEM56	31.00	29.00	29.00	29.67	48.00
TMEM56-RWDD3	50.00	29.00	44.00	41.00	22.00
TMEM57	806.00	824.00	753.00	794.33	814.00
TMEM59	2637.00	2532.00	2568.00	2579.00	2714.00
TMEM59L	73.00	78.00	70.00	73.67	36.00
TMEM60	296.00	292.00	301.00	296.33	350.00
TMEM62	359.00	271.00	297.00	309.00	440.00
TMEM63A	1136.00	1125.00	1185.00	1148.67	952.00
TMEM63B	1492.00	1451.00	1337.00	1426.67	1358.00
TMEM64	547.00	500.00	574.00	540.33	686.00
TMEM65	341.00	299.00	378.00	339.33	309.00
TMEM67	721.00	692.00	727.00	713.33	735.00
TMEM68	1058.00	980.00	1097.00	1045.00	1385.00
TMEM69	878.00	831.00	840.00	849.67	1145.00
TMEM70	663.00	663.00	763.00	696.33	851.00

TMEM71	167.00	184.00	202.00	184.33	209.00
TMEM74	24.00	30.00	25.00	26.33	27.00
TMEM79	298.00	264.00	306.00	289.33	322.00
TMEM80	684.00	622.00	637.00	647.67	710.00
TMEM81	65.00	87.00	91.00	81.00	90.00
TMEM86A	13.00	5.00	16.00	11.33	7.00
TMEM86B	141.00	141.00	179.00	153.67	140.00
TMEM87A	1596.00	1606.00	1758.00	1653.33	1656.00
TMEM87B	1616.00	1459.00	1655.00	1576.67	1331.00
TMEM88	18.00	23.00	25.00	22.00	15.00
TMEM8A	1550.00	1717.00	1779.00	1682.00	1727.00
TMEM8B	398.00	373.00	382.00	384.33	310.00
TMEM9	1779.00	1852.00	1682.00	1771.00	1866.00
TMEM91	188.00	194.00	236.00	206.00	182.00
TMEM92-AS1	29.00	32.00	23.00	28.00	34.00
TMEM97	620.00	560.00	554.00	578.00	844.00
TMEM98	485.00	471.00	479.00	478.33	618.00
TMEM99	338.00	304.00	345.00	329.00	399.00
TMEM9B	765.00	736.00	686.00	729.00	833.00
TMEM9B-AS1	260.00	229.00	292.00	260.33	140.00
TMF1	1140.00	1055.00	1181.00	1125.33	1117.00
TMLHE	146.00	128.00	152.00	142.00	135.00
TMOD2	338.00	317.00	416.00	357.00	319.00
TMOD3	825.00	686.00	828.00	779.67	859.00
TMOD4	20.00	21.00	26.00	22.33	19.00
TMPO	1791.00	1590.00	1933.00	1771.33	2470.00
TMPO-AS1	32.00	42.00	40.00	38.00	67.00
TMPPE	52.00	50.00	44.00	48.67	22.00
TMPRSS15	896.00	790.00	1072.00	919.33	796.00
TMPRSS5	11.00	14.00	12.00	12.33	10.00
TMPRSS7	5.00	3.00	5.00	4.33	3.00
TMSB10	11371.00	11523.00	11851.00	11581.67	18708.00
TMSB15A	7.00	7.00	5.00	6.33	12.00
TMSB4X	2875.00	3052.00	3173.00	3033.33	5257.00
TMTC1	5.00	6.00	6.00	5.67	3.00
TMTC2	302.00	300.00	337.00	313.00	283.00
TMTC3	657.00	598.00	720.00	658.33	620.00
TMTC4	536.00	535.00	538.00	536.33	674.00
TMUB1	1065.00	1060.00	1045.00	1056.67	1306.00
TMUB2	1200.00	1286.00	1281.00	1255.67	1196.00
TMX1	1822.00	1600.00	1617.00	1679.67	2064.00
TMX2	2513.00	2447.00	2387.00	2449.00	2905.00
TMX3	3491.00	3405.00	3519.00	3471.67	3593.00
TMX4	1062.00	919.00	930.00	970.33	1020.00
TNC	10415.00	9684.00	10955.00	10351.33	6783.00
TNFAIP1	670.00	582.00	698.00	650.00	657.00
TNFAIP2	1428.00	1307.00	1352.00	1362.33	1060.00
TNFAIP3	760.00	816.00	835.00	803.67	703.00

TNFAIP6	3.00	1.00	7.00	3.67	0.00
TNFAIP8	105.00	64.00	101.00	90.00	116.00
TNFAIP8L1	244.00	220.00	269.00	244.33	376.00
TNFRSF10A	465.00	473.00	494.00	477.33	401.00
TNFRSF10B	5984.00	5867.00	5841.00	5897.33	4943.00
TNFRSF10C	8.00	3.00	5.00	5.33	1.00
TNFRSF10D	5816.00	5574.00	5277.00	5555.67	4324.00
TNFRSF11A	170.00	142.00	147.00	153.00	145.00
TNFRSF11B	14.00	9.00	13.00	12.00	11.00
TNFRSF12A	7975.00	8905.00	8461.00	8447.00	10347.00
TNFRSF19	717.00	657.00	693.00	689.00	858.00
TNFRSF1A	2843.00	2864.00	2643.00	2783.33	2517.00
TNFRSF21	913.00	914.00	927.00	918.00	760.00
TNFRSF25	594.00	706.00	743.00	681.00	801.00
TNFRSF6B	283.00	320.00	257.00	286.67	653.00
TNFRSF9	40.00	32.00	28.00	33.33	25.00
TNFSF12	168.00	164.00	175.00	169.00	215.00
TNFSF13	7.00	20.00	11.00	12.67	8.00
TNFSF13B	43.00	47.00	54.00	48.00	43.00
TNFSF15	25.00	18.00	30.00	24.33	21.00
TNFSF18	7.00	0.00	2.00	3.00	11.00
TNFSF4	59.00	82.00	78.00	73.00	96.00
TNFSF9	5.00	7.00	8.00	6.67	5.00
TNIK	435.00	419.00	424.00	426.00	342.00
TNIP1	2324.00	2291.00	2395.00	2336.67	1929.00
TNIP2	543.00	564.00	608.00	571.67	683.00
TNIP3	4.00	2.00	3.00	3.00	4.00
TNK2	1864.00	1920.00	2046.00	1943.33	1878.00
TNKS	1267.00	1116.00	1365.00	1249.33	1239.00
TNKS1BP1	3898.00	3981.00	4080.00	3986.33	3687.00
TNKS2	2407.00	2171.00	2559.00	2379.00	2402.00
TNKS2-AS1	14.00	10.00	15.00	13.00	16.00
TNNC1	5.00	3.00	6.00	4.67	2.00
TNNT1	51.00	65.00	76.00	64.00	54.00
TNPO1	5390.00	4614.00	6209.00	5404.33	5182.00
TNPO2	4144.00	4194.00	4360.00	4232.67	4411.00
TNPO3	2686.00	2575.00	2804.00	2688.33	2482.00
TNRC18	5872.00	5685.00	5995.00	5850.67	5335.00
TNRC6A	3006.00	2869.00	3118.00	2997.67	2735.00
TNRC6B	721.00	718.00	787.00	742.00	618.00
TNRC6C	402.00	400.00	439.00	413.67	351.00
TNRC6C-AS1	7.00	3.00	3.00	4.33	4.00
TNS2	753.00	733.00	767.00	751.00	402.00
TNS3	2445.00	2229.00	2374.00	2349.33	1990.00
TOB1	176.00	194.00	207.00	192.33	133.00
TOB1-AS1	6.00	13.00	14.00	11.00	10.00
TOB2	1212.00	1211.00	1249.00	1224.00	1191.00
TOB2P1	38.00	32.00	26.00	32.00	45.00

TOE1	655.00	606.00	682.00	647.67	814.00
TOLLIP	885.00	831.00	823.00	846.33	839.00
TOLLIP-AS1	11.00	12.00	11.00	11.33	12.00
TOM1	536.00	527.00	577.00	546.67	609.00
TOM1L1	831.00	781.00	805.00	805.67	1097.00
TOM1L2	1027.00	1011.00	1066.00	1034.67	885.00
TOMM20	3874.00	3812.00	4182.00	3956.00	5047.00
TOMM22	960.00	968.00	962.00	963.33	1394.00
TOMM34	6789.00	6238.00	7114.00	6713.67	7563.00
TOMM40	1464.00	1476.00	1503.00	1481.00	1973.00
TOMM40L	484.00	432.00	457.00	457.67	526.00
TOMM5	1120.00	1187.00	1242.00	1183.00	1794.00
TOMM6	1263.00	1205.00	1413.00	1293.67	1159.00
TOMM7	1801.00	1851.00	2042.00	1898.00	2593.00
TOMM70A	3129.00	2720.00	3144.00	2997.67	3423.00
TONSL	1357.00	1555.00	1529.00	1480.33	1680.00
TONSL-AS1	21.00	26.00	32.00	26.33	21.00
TOP1	5010.00	4719.00	5138.00	4955.67	4653.00
TOP1MT	1358.00	1412.00	1658.00	1476.00	1670.00
TOP1P1	49.00	51.00	60.00	53.33	45.00
TOP1P2	3.00	0.00	2.00	1.67	2.00
TOP2A	3991.00	3497.00	3679.00	3722.33	4298.00
TOP2B	3312.00	3029.00	3669.00	3336.67	3632.00
TOP3A	815.00	805.00	838.00	819.33	807.00
TOP3B	796.00	805.00	876.00	825.67	762.00
TOPBP1	1567.00	1443.00	1687.00	1565.67	1637.00
TOPORS	791.00	728.00	838.00	785.67	874.00
TOPORS-AS1	29.00	52.00	38.00	39.67	33.00
TOR1A	1747.00	1682.00	1542.00	1657.00	1877.00
TOR1AIP1	1275.00	1230.00	1213.00	1239.33	1412.00
TOR1AIP2	1730.00	1670.00	1832.00	1744.00	1983.00
TOR1B	1152.00	1211.00	1116.00	1159.67	1132.00
TOR2A	506.00	510.00	486.00	500.67	527.00
TOR3A	784.00	779.00	741.00	768.00	915.00
TOR4A	1519.00	1636.00	1789.00	1648.00	1866.00
TOX	56.00	63.00	66.00	61.67	84.00
TOX2	1830.00	1734.00	1886.00	1816.67	1547.00
TOX4	1190.00	1168.00	1215.00	1191.00	1142.00
TP53	1264.00	1234.00	1360.00	1286.00	1937.00
TP53AIP1	3.00	2.00	2.00	2.33	0.00
TP53BP1	2384.00	2185.00	2385.00	2318.00	2093.00
TP53BP2	1499.00	1406.00	1534.00	1479.67	1424.00
TP53I13	763.00	759.00	700.00	740.67	786.00
TP53I3	247.00	232.00	280.00	253.00	287.00
TP53INP1	168.00	169.00	192.00	176.33	124.00
TP53INP2	267.00	272.00	331.00	290.00	166.00
TP53RK	525.00	496.00	552.00	524.33	654.00
TP53TG1	40.00	30.00	57.00	42.33	63.00

TP53TG3D	30.00	24.00	23.00	25.67	20.00
TP63	707.00	614.00	649.00	656.67	871.00
TPBG	4716.00	4549.00	4891.00	4718.67	2179.00
TPCN1	5013.00	4932.00	5099.00	5014.67	3364.00
TPCN2	3667.00	3403.00	3506.00	3525.33	3302.00
TPD52	693.00	632.00	780.00	701.67	942.00
TPD52L1	21.00	23.00	23.00	22.33	33.00
TPD52L2	4384.00	4299.00	4448.00	4377.00	5153.00
TPGS1	149.00	145.00	136.00	143.33	168.00
TPGS2	881.00	906.00	907.00	898.00	949.00
TPH1	26.00	16.00	17.00	19.67	31.00
TPI1	9473.00	9209.00	9896.00	9526.00	12547.00
TPI1P2	46.00	51.00	39.00	45.33	58.00
TPK1	82.00	85.00	123.00	96.67	132.00
TPM1	5774.00	5763.00	6318.00	5951.67	7601.00
TPM2	1367.00	1483.00	1661.00	1503.67	1678.00
TPM3	6335.00	5829.00	6321.00	6161.67	8346.00
TPM3P9	191.00	201.00	221.00	204.33	202.00
TPM4	10368.00	9984.00	11165.00	10505.67	13278.00
TPMT	381.00	395.00	462.00	412.67	500.00
TPP1	19207.00	19267.00	19813.00	19429.00	14348.00
TPP2	2040.00	1920.00	2218.00	2059.33	2162.00
TPPP	5.00	5.00	3.00	4.33	1.00
TPPP3	5.00	11.00	5.00	7.00	9.00
TPR	5775.00	5501.00	6063.00	5779.67	4949.00
TPRA1	1599.00	1660.00	1755.00	1671.33	1536.00
TPRG1	8.00	1.00	1.00	3.33	3.00
TPRG1-AS1	4.00	6.00	3.00	4.33	2.00
TPRG1L	719.00	744.00	807.00	756.67	724.00
TPRKB	421.00	445.00	468.00	444.67	570.00
TPRN	687.00	732.00	809.00	742.67	731.00
TPST1	846.00	879.00	844.00	856.33	936.00
TPST2	503.00	417.00	496.00	472.00	420.00
TPT1	25252.00	24733.00	27115.00	25700.00	31328.00
TPT1-AS1	158.00	138.00	170.00	155.33	178.00
TPTE2	4.00	2.00	6.00	4.00	3.00
TPTE2P1	7.00	8.00	15.00	10.00	9.00
TPTE2P5	19.00	14.00	22.00	18.33	23.00
TPTE2P6	19.00	17.00	21.00	19.00	7.00
TPX2	4366.00	3995.00	4109.00	4156.67	4358.00
TRA2A	1876.00	1857.00	2108.00	1947.00	2510.00
TRA2B	3549.00	3412.00	3803.00	3588.00	4612.00
TRABD	1456.00	1475.00	1620.00	1517.00	1765.00
TRABD2A	705.00	669.00	704.00	692.67	684.00
TRADD	249.00	289.00	282.00	273.33	278.00
TRAF1	48.00	91.00	75.00	71.33	121.00
TRAF2	740.00	844.00	874.00	819.33	974.00
TRAF3	1455.00	1393.00	1498.00	1448.67	1407.00

TRAF3IP1	392.00	412.00	487.00	430.33	400.00
TRAF3IP2	344.00	281.00	313.00	312.67	293.00
TRAF3IP2-AS1	205.00	177.00	234.00	205.33	199.00
TRAF4	705.00	716.00	737.00	719.33	751.00
TRAF5	778.00	672.00	662.00	704.00	867.00
TRAF6	405.00	389.00	417.00	403.67	342.00
TRAF7	2687.00	2715.00	2956.00	2786.00	2108.00
TRAFD1	513.00	445.00	475.00	477.67	487.00
TRAIP	313.00	384.00	287.00	328.00	424.00
TRAK1	1077.00	1105.00	1137.00	1106.33	1172.00
TRAK2	1151.00	1033.00	1198.00	1127.33	909.00
TRAM1	3805.00	3425.00	4015.00	3748.33	3320.00
TRAM1L1	77.00	62.00	91.00	76.67	92.00
TRAM2	6544.00	6013.00	6496.00	6351.00	4253.00
TRAM2-AS1	207.00	156.00	206.00	189.67	248.00
TRANK1	47.00	56.00	36.00	46.33	40.00
TRAP1	3415.00	3381.00	3618.00	3471.33	4065.00
TRAPPC1	1225.00	1260.00	1212.00	1232.33	1725.00
TRAPPC10	857.00	877.00	882.00	872.00	756.00
TRAPPC11	763.00	716.00	758.00	745.67	673.00
TRAPPC12	331.00	346.00	392.00	356.33	280.00
TRAPPC13	654.00	550.00	626.00	610.00	789.00
TRAPPC2	404.00	406.00	428.00	412.67	547.00
TRAPPC2B	61.00	57.00	80.00	66.00	69.00
TRAPPC2L	539.00	536.00	593.00	556.00	747.00
TRAPPC3	1090.00	1170.00	1223.00	1161.00	1260.00
TRAPPC3L	3.00	0.00	0.00	1.00	0.00
TRAPPC4	669.00	605.00	660.00	644.67	761.00
TRAPPC5	396.00	380.00	390.00	388.67	495.00
TRAPPC6A	75.00	65.00	98.00	79.33	76.00
TRAPPC6B	694.00	548.00	646.00	629.33	682.00
TRAPPC8	1007.00	917.00	1042.00	988.67	999.00
TRAPPC9	385.00	373.00	429.00	395.67	379.00
TRDMT1	266.00	288.00	292.00	282.00	290.00
TRERF1	1393.00	1223.00	1414.00	1343.33	1154.00
TREX1	243.00	265.00	252.00	253.33	268.00
TREX2	33.00	31.00	32.00	32.00	33.00
TRG-AS1	3.00	6.00	13.00	7.33	7.00
TRHDE	1532.00	1337.00	1606.00	1491.67	1157.00
TRHDE-AS1	798.00	796.00	841.00	811.67	655.00
TRIAP1	308.00	325.00	355.00	329.33	450.00
TRIB1	524.00	518.00	548.00	530.00	658.00
TRIB2	728.00	684.00	755.00	722.33	582.00
TRIB3	6311.00	5836.00	6224.00	6123.67	5130.00
TRIL	3.00	7.00	20.00	10.00	19.00
TRIM11	923.00	915.00	992.00	943.33	1116.00
TRIM13	592.00	581.00	674.00	615.67	660.00
TRIM14	791.00	684.00	777.00	750.67	808.00

TRIM16	575.00	605.00	651.00	610.33	692.00
TRIM16L	229.00	199.00	231.00	219.67	257.00
TRIM2	16.00	22.00	18.00	18.67	26.00
TRIM21	260.00	238.00	238.00	245.33	302.00
TRIM23	296.00	241.00	289.00	275.33	302.00
TRIM24	400.00	361.00	405.00	388.67	461.00
TRIM25	4058.00	3858.00	4339.00	4085.00	3459.00
TRIM28	6988.00	6860.00	6837.00	6895.00	7718.00
TRIM3	521.00	512.00	515.00	516.00	505.00
TRIM32	650.00	657.00	698.00	668.33	610.00
TRIM33	1336.00	1199.00	1385.00	1306.67	1174.00
TRIM34	13.00	10.00	13.00	12.00	8.00
TRIM35	299.00	271.00	341.00	303.67	349.00
TRIM36	144.00	148.00	165.00	152.33	190.00
TRIM37	1110.00	984.00	1107.00	1067.00	1171.00
TRIM38	1296.00	1215.00	1305.00	1272.00	1288.00
TRIM4	309.00	264.00	294.00	289.00	283.00
TRIM41	2112.00	2089.00	2170.00	2123.67	2321.00
TRIM44	5124.00	4625.00	5418.00	5055.67	4574.00
TRIM45	561.00	548.00	491.00	533.33	567.00
TRIM46	303.00	315.00	345.00	321.00	316.00
TRIM47	30.00	39.00	37.00	35.33	54.00
TRIM5	908.00	821.00	940.00	889.67	1030.00
TRIM52	443.00	449.00	477.00	456.33	352.00
TRIM52-AS1	222.00	199.00	253.00	224.67	237.00
TRIM55	2184.00	2004.00	2235.00	2141.00	2917.00
TRIM56	772.00	736.00	874.00	794.00	437.00
TRIM58	884.00	872.00	921.00	892.33	799.00
TRIM59	393.00	332.00	390.00	371.67	438.00
TRIM6	139.00	147.00	151.00	145.67	167.00
TRIM62	192.00	191.00	198.00	193.67	186.00
TRIM65	1470.00	1508.00	1752.00	1576.67	2098.00
TRIM66	1234.00	1129.00	1231.00	1198.00	909.00
TRIM68	374.00	389.00	413.00	392.00	385.00
TRIM7	87.00	76.00	83.00	82.00	95.00
TRIM8	3747.00	3675.00	4096.00	3839.33	3989.00
TRIM9	138.00	104.00	123.00	121.67	197.00
TRIO	13442.00	12723.00	14462.00	13542.33	9261.00
TRIOBP	2575.00	2488.00	2529.00	2530.67	2289.00
TRIP10	1552.00	1537.00	1639.00	1576.00	1752.00
TRIP11	1739.00	1672.00	1777.00	1729.33	1593.00
TRIP12	5677.00	5176.00	5923.00	5592.00	5362.00
TRIP13	934.00	826.00	910.00	890.00	1265.00
TRIP4	462.00	462.00	553.00	492.33	567.00
TRIP6	2737.00	2818.00	3037.00	2864.00	3362.00
TRIQK	696.00	609.00	845.00	716.67	854.00
TRIT1	865.00	863.00	1040.00	922.67	1009.00
TRMT1	3526.00	3736.00	3799.00	3687.00	4060.00

TRMT10A	165.00	157.00	167.00	163.00	182.00
TRMT10B	303.00	279.00	317.00	299.67	335.00
TRMT10C	736.00	699.00	758.00	731.00	879.00
TRMT11	654.00	595.00	674.00	641.00	689.00
TRMT112	3345.00	3305.00	3463.00	3371.00	4349.00
TRMT12	378.00	378.00	390.00	382.00	377.00
TRMT13	1022.00	1042.00	1204.00	1089.33	1054.00
TRMT1L	757.00	778.00	832.00	789.00	813.00
TRMT2A	1021.00	1089.00	1020.00	1043.33	1161.00
TRMT2B	351.00	348.00	404.00	367.67	483.00
TRMT44	356.00	334.00	327.00	339.00	322.00
TRMT5	1006.00	875.00	969.00	950.00	1062.00
TRMT6	1095.00	896.00	1027.00	1006.00	1296.00
TRMT61A	999.00	990.00	1027.00	1005.33	1237.00
TRMT61B	507.00	436.00	535.00	492.67	503.00
TRMU	704.00	716.00	803.00	741.00	763.00
TRNAU1AP	215.00	244.00	258.00	239.00	278.00
TRNP1	1660.00	1463.00	1627.00	1583.33	1097.00
TRNT1	827.00	751.00	817.00	798.33	823.00
TRO	3.00	2.00	2.00	2.33	0.00
TROAP	1102.00	1124.00	968.00	1064.67	1314.00
TROVE2	402.00	365.00	433.00	400.00	407.00
TRPA1	148.00	123.00	161.00	144.00	147.00
TRPC1	837.00	863.00	919.00	873.00	726.00
TRPC3	17.00	18.00	39.00	24.67	24.00
TRPC4	45.00	40.00	40.00	41.67	58.00
TRPC4AP	3211.00	3214.00	3530.00	3318.33	3291.00
TRPC6	98.00	120.00	142.00	120.00	119.00
TRPM2	679.00	729.00	725.00	711.00	851.00
TRPM2-AS	3.00	1.00	3.00	2.33	1.00
TRPM4	935.00	916.00	981.00	944.00	905.00
TRPM6	3.00	6.00	2.00	3.67	1.00
TRPM7	2946.00	2683.00	3232.00	2953.67	3038.00
TRPS1	496.00	463.00	578.00	512.33	428.00
TRPT1	865.00	916.00	899.00	893.33	1070.00
TRPV1	545.00	601.00	662.00	602.67	528.00
TRPV2	1966.00	1988.00	1766.00	1906.67	1627.00
TRRAP	2878.00	2543.00	2887.00	2769.33	2272.00
TRUB1	848.00	699.00	875.00	807.33	881.00
TRUB2	711.00	690.00	784.00	728.33	731.00
TSACC	15.00	8.00	14.00	12.33	18.00
TSC1	1815.00	1851.00	1886.00	1850.67	1715.00
TSC2	2539.00	2356.00	2554.00	2483.00	2041.00
TSC22D1	1651.00	1611.00	1973.00	1745.00	1222.00
TSC22D1-AS1	36.00	25.00	40.00	33.67	35.00
TSC22D2	976.00	986.00	1164.00	1042.00	1064.00
TSC22D3	530.00	480.00	546.00	518.67	611.00
TSC22D4	1180.00	1218.00	1293.00	1230.33	1048.00

TSEN15	1372.00	1364.00	1454.00	1396.67	1817.00
TSEN2	673.00	673.00	692.00	679.33	583.00
TSEN54	515.00	613.00	628.00	585.33	630.00
TSFM	529.00	491.00	537.00	519.00	664.00
TSG101	1344.00	1214.00	1331.00	1296.33	1465.00
TSGA10	63.00	78.00	79.00	73.33	73.00
TSGA10IP	14.00	13.00	10.00	12.33	13.00
TSHZ1	249.00	233.00	271.00	251.00	228.00
TSHZ3	507.00	459.00	470.00	478.67	531.00
TSIX	9.00	9.00	8.00	8.67	0.00
TSKU	1008.00	936.00	1045.00	996.33	958.00
TSLP	597.00	458.00	630.00	561.67	349.00
TSN	1812.00	1649.00	1848.00	1769.67	2255.00
TSNARE1	229.00	231.00	253.00	237.67	253.00
TSNAX	573.00	487.00	594.00	551.33	701.00
TSNAXIP1	30.00	32.00	44.00	35.33	28.00
TSPAN1	6.00	2.00	3.00	3.67	5.00
TSPAN10	70.00	65.00	91.00	75.33	119.00
TSPAN12	134.00	98.00	110.00	114.00	143.00
TSPAN13	105.00	99.00	94.00	99.33	132.00
TSPAN14	4059.00	4064.00	3987.00	4036.67	2459.00
TSPAN15	141.00	133.00	129.00	134.33	110.00
TSPAN17	1118.00	1102.00	1222.00	1147.33	1178.00
TSPAN2	7.00	5.00	1.00	4.33	12.00
TSPAN3	3647.00	3870.00	3571.00	3696.00	4155.00
TSPAN31	489.00	515.00	561.00	521.67	538.00
TSPAN33	20.00	28.00	16.00	21.33	50.00
TSPAN4	4052.00	4106.00	4159.00	4105.67	4719.00
TSPAN5	2157.00	2081.00	2150.00	2129.33	1787.00
TSPAN6	880.00	891.00	908.00	893.00	1030.00
TSPAN7	437.00	390.00	379.00	402.00	348.00
TSPAN8	3.00	6.00	2.00	3.67	4.00
TSPAN9	785.00	781.00	778.00	781.33	614.00
TSPO	2128.00	2106.00	2080.00	2104.67	2792.00
TSPYL1	1123.00	1038.00	1171.00	1110.67	1269.00
TSPYL2	1255.00	1377.00	1418.00	1350.00	1097.00
TSPYL4	1096.00	1031.00	1191.00	1106.00	1104.00
TSR1	4497.00	4310.00	4632.00	4479.67	5295.00
TSR2	546.00	521.00	530.00	532.33	675.00
TSR3	755.00	775.00	827.00	785.67	979.00
TSSC1	499.00	549.00	550.00	532.67	682.00
TSSC2	153.00	148.00	213.00	171.33	115.00
TSSC4	769.00	811.00	838.00	806.00	906.00
TSSK3	4.00	3.00	1.00	2.67	5.00
TSSK6	60.00	83.00	73.00	72.00	75.00
TST	194.00	183.00	168.00	181.67	240.00
TSTA3	20.00	35.00	39.00	31.33	30.00
TSTD2	719.00	722.00	726.00	722.33	672.00

TSTD3	104.00	80.00	88.00	90.67	85.00
TTBK2	277.00	238.00	326.00	280.33	197.00
TTC1	1284.00	1251.00	1249.00	1261.33	1605.00
TTC12	416.00	471.00	445.00	444.00	426.00
TTC13	785.00	805.00	871.00	820.33	918.00
TTC14	2168.00	2094.00	2369.00	2210.33	2214.00
TTC17	3554.00	3513.00	3650.00	3572.33	3110.00
TTC19	1654.00	1640.00	1631.00	1641.67	1608.00
TTC21A	131.00	132.00	134.00	132.33	84.00
TTC21B	726.00	683.00	797.00	735.33	772.00
TTC23	361.00	376.00	391.00	376.00	405.00
TTC23L	4.00	1.00	0.00	1.67	0.00
TTC25	21.00	30.00	21.00	24.00	26.00
TTC26	526.00	514.00	576.00	538.67	577.00
TTC27	515.00	511.00	525.00	517.00	564.00
TTC28	734.00	640.00	782.00	718.67	701.00
TTC28-AS1	199.00	184.00	261.00	214.67	183.00
TTC29	3.00	4.00	2.00	3.00	3.00
TTC3	3704.00	3569.00	3946.00	3739.67	3616.00
TTC30A	136.00	123.00	132.00	130.33	129.00
TTC30B	84.00	64.00	74.00	74.00	82.00
TTC31	1099.00	1100.00	1092.00	1097.00	1069.00
TTC32	143.00	121.00	143.00	135.67	143.00
TTC33	391.00	338.00	432.00	387.00	489.00
TTC37	4663.00	4363.00	5163.00	4729.67	4447.00
TTC38	456.00	486.00	556.00	499.33	448.00
TTC39A	3.00	0.00	0.00	1.00	0.00
TTC39B	104.00	97.00	117.00	106.00	147.00
TTC39C	215.00	199.00	222.00	212.00	272.00
TTC3P1	40.00	36.00	33.00	36.33	33.00
TTC4	88.00	69.00	79.00	78.67	79.00
TTC41P	32.00	15.00	16.00	21.00	21.00
TTC5	313.00	291.00	338.00	314.00	320.00
TTC6	8.00	1.00	7.00	5.33	3.00
TTC7A	440.00	469.00	472.00	460.33	455.00
TTC7B	924.00	811.00	920.00	885.00	1162.00
TTC8	565.00	528.00	645.00	579.33	623.00
TTC9B	3.00	3.00	2.00	2.67	2.00
TTC9C	440.00	466.00	490.00	465.33	492.00
TTF1	418.00	378.00	457.00	417.67	478.00
TTF2	1390.00	1326.00	1335.00	1350.33	1267.00
TTI1	1077.00	1043.00	1136.00	1085.33	1032.00
TTI2	246.00	221.00	207.00	224.67	255.00
TTK	779.00	793.00	853.00	808.33	1003.00
TTL	2519.00	2389.00	2605.00	2504.33	3004.00
TTLL1	78.00	74.00	93.00	81.67	91.00
TTLL11	415.00	385.00	413.00	404.33	268.00
TTLL12	1361.00	1278.00	1510.00	1383.00	1577.00

TTLL13P	12.00	1.00	14.00	9.00	8.00
TTLL3	163.00	155.00	178.00	165.33	83.00
TTLL4	1451.00	1392.00	1547.00	1463.33	1242.00
TTLL5	910.00	876.00	999.00	928.33	845.00
TTLL7	236.00	198.00	235.00	223.00	148.00
TTN	5.00	4.00	4.00	4.33	2.00
TTN-AS1	249.00	218.00	229.00	232.00	178.00
TTPA	19.00	32.00	29.00	26.67	28.00
TTPAL	1260.00	1067.00	1230.00	1185.67	1147.00
TTYH2	321.00	308.00	329.00	319.33	213.00
TTYH3	3278.00	3375.00	3539.00	3397.33	2415.00
TUBA1A	483.00	482.00	660.00	541.67	461.00
TUBA1B	5097.00	4766.00	5705.00	5189.33	6869.00
TUBA1C	6459.00	6198.00	6757.00	6471.33	7712.00
TUBA3FP	3.00	3.00	6.00	4.00	2.00
TUBA4A	3.00	1.00	1.00	1.67	1.00
TUBA8	23.00	17.00	15.00	18.33	20.00
TUBB	13.00	10.00	12.00	11.67	7.00
TUBB1	3.00	4.00	3.00	3.33	0.00
TUBB2A	60.00	64.00	66.00	63.33	71.00
TUBB3	701.00	758.00	747.00	735.33	833.00
TUBB4B	4458.00	4400.00	4135.00	4331.00	5211.00
TUBB6	6759.00	6536.00	6963.00	6752.67	11677.00
TUBD1	119.00	135.00	138.00	130.67	148.00
TUBE1	1427.00	1227.00	1446.00	1366.67	1705.00
TUBG1	739.00	775.00	627.00	713.67	923.00
TUBG2	783.00	804.00	797.00	794.67	725.00
TUBGCP2	1729.00	1653.00	1784.00	1722.00	1907.00
TUBGCP3	373.00	362.00	407.00	380.67	356.00
TUBGCP4	451.00	409.00	530.00	463.33	428.00
TUBGCP5	31.00	48.00	48.00	42.33	41.00
TUBGCP6	1920.00	1912.00	1957.00	1929.67	1784.00
TUFM	6201.00	6117.00	6167.00	6161.67	7287.00
TUFT1	157.00	128.00	169.00	151.33	276.00
TUG1	6710.00	6381.00	6996.00	6695.67	6108.00
TULP3	801.00	835.00	808.00	814.67	781.00
TULP4	793.00	693.00	768.00	751.33	759.00
TUSC2	563.00	504.00	595.00	554.00	638.00
TUSC3	1615.00	1633.00	1742.00	1663.33	1954.00
TUT1	923.00	837.00	944.00	901.33	897.00
TVP23B	669.00	695.00	680.00	681.33	869.00
TVP23C	251.00	287.00	260.00	266.00	236.00
TWF1	1296.00	1115.00	1475.00	1295.33	1748.00
TWF2	1421.00	1503.00	1351.00	1425.00	1531.00
TWIST1	266.00	254.00	221.00	247.00	330.00
TWIST2	318.00	304.00	313.00	311.67	351.00
TWISTNB	1009.00	845.00	1149.00	1001.00	1119.00
TWSG1	1233.00	1161.00	1311.00	1235.00	1155.00

TXK	76.00	54.00	72.00	67.33	55.00
TXLNA	4013.00	3936.00	4266.00	4071.67	3928.00
TXLNB	19.00	10.00	15.00	14.67	20.00
TXLNG	1937.00	1804.00	2137.00	1959.33	1952.00
TXN	2653.00	2668.00	2802.00	2707.67	4234.00
TXN2	862.00	878.00	985.00	908.33	1161.00
TXNDC11	871.00	863.00	916.00	883.33	831.00
TXNDC12	1991.00	1864.00	1890.00	1915.00	2499.00
TXNDC15	1321.00	1325.00	1289.00	1311.67	1443.00
TXNDC16	140.00	120.00	131.00	130.33	131.00
TXNDC17	770.00	814.00	809.00	797.67	1159.00
TXNDC5	278.00	230.00	259.00	255.67	209.00
TXNDC9	624.00	597.00	707.00	642.67	798.00
TXNIP	230.00	167.00	143.00	180.00	277.00
TXNL1	907.00	855.00	1042.00	934.67	1263.00
TXNL4A	650.00	593.00	727.00	656.67	962.00
TXNL4B	512.00	441.00	527.00	493.33	568.00
TXNRD1	14967.00	13796.00	14736.00	14499.67	18305.00
TXNRD2	1546.00	1591.00	1769.00	1635.33	1913.00
TXNRD3	210.00	182.00	204.00	198.67	167.00
TYK2	2397.00	2431.00	2543.00	2457.00	2642.00
TYMP	20.00	20.00	19.00	19.67	25.00
TYMS	995.00	971.00	924.00	963.33	2332.00
TYMSOS	15.00	13.00	7.00	11.67	8.00
TYRO3	1231.00	1271.00	1216.00	1239.33	1165.00
TYSND1	1134.00	1139.00	1268.00	1180.33	1305.00
TYW1	768.00	738.00	822.00	776.00	808.00
TYW1B	43.00	56.00	58.00	52.33	70.00
TYW3	1201.00	1050.00	1305.00	1185.33	1423.00
TYW5	475.00	450.00	512.00	479.00	429.00
U2AF1	47.00	30.00	32.00	36.33	34.00
U2AF1L4	170.00	186.00	159.00	171.67	188.00
U2AF2	5247.00	5274.00	5510.00	5343.67	6143.00
U2SURP	4088.00	3850.00	4449.00	4129.00	5010.00
UACA	8665.00	8465.00	9125.00	8751.67	8831.00
UAP1	8030.00	7225.00	8008.00	7754.33	16758.00
UAP1L1	417.00	445.00	492.00	451.33	423.00
UBA1	10545.00	10162.00	10665.00	10457.33	11019.00
UBA2	2451.00	2265.00	2722.00	2479.33	2525.00
UBA3	1197.00	1036.00	1197.00	1143.33	1289.00
UBA5	1070.00	1035.00	1107.00	1070.67	1306.00
UBA52	5357.00	5227.00	5850.00	5478.00	7184.00
UBA6	1201.00	1136.00	1123.00	1153.33	1179.00
UBA6-AS1	229.00	220.00	187.00	212.00	241.00
UBA7	57.00	68.00	89.00	71.33	62.00
UBAC1	738.00	683.00	835.00	752.00	868.00
UBAC2	1429.00	1542.00	1423.00	1464.67	1643.00
UBAC2-AS1	43.00	47.00	43.00	44.33	47.00

UBALD1	568.00	584.00	627.00	593.00	666.00
UBALD2	629.00	562.00	590.00	593.67	576.00
UBAP1	703.00	698.00	784.00	728.33	694.00
UBAP1L	63.00	64.00	54.00	60.33	50.00
UBAP2	1420.00	1324.00	1375.00	1373.00	1269.00
UBAP2L	4287.00	4206.00	4554.00	4349.00	3856.00
UBASH3B	1247.00	1077.00	1274.00	1199.33	1570.00
UBB	9839.00	9531.00	10010.00	9793.33	12588.00
UBC	7627.00	7490.00	8277.00	7798.00	8359.00
UBE2A	630.00	602.00	641.00	624.33	763.00
UBE2B	654.00	589.00	741.00	661.33	771.00
UBE2C	1135.00	1132.00	1193.00	1153.33	1706.00
UBE2D1	449.00	347.00	420.00	405.33	525.00
UBE2D2	1324.00	1306.00	1432.00	1354.00	1727.00
UBE2D3	4271.00	3976.00	4488.00	4245.00	4894.00
UBE2D4	500.00	425.00	482.00	469.00	641.00
UBE2E1	2019.00	1958.00	2185.00	2054.00	2743.00
UBE2E2	453.00	454.00	542.00	483.00	721.00
UBE2E3	828.00	820.00	895.00	847.67	1124.00
UBE2E4P	40.00	33.00	30.00	34.33	38.00
UBE2F	423.00	374.00	387.00	394.67	507.00
UBE2G1	1186.00	1134.00	1213.00	1177.67	1530.00
UBE2G2	3690.00	3523.00	3822.00	3678.33	3885.00
UBE2H	2714.00	2459.00	2805.00	2659.33	2220.00
UBE2I	1947.00	2032.00	2058.00	2012.33	2656.00
UBE2J1	1302.00	1168.00	1318.00	1262.67	1582.00
UBE2J2	1124.00	1144.00	1188.00	1152.00	1266.00
UBE2K	2823.00	2729.00	3067.00	2873.00	3346.00
UBE2L3	1275.00	1174.00	1261.00	1236.67	1527.00
UBE2L6	78.00	95.00	108.00	93.67	102.00
UBE2M	1278.00	1276.00	1412.00	1322.00	1804.00
UBE2MP1	54.00	42.00	52.00	49.33	60.00
UBE2N	1559.00	1541.00	1605.00	1568.33	2034.00
UBE2O	1618.00	1783.00	1750.00	1717.00	1465.00
UBE2Q1	1725.00	1704.00	1721.00	1716.67	1965.00
UBE2Q2	666.00	616.00	747.00	676.33	810.00
UBE2Q2P1	56.00	35.00	46.00	45.67	34.00
UBE2R2	1874.00	1679.00	1910.00	1821.00	2019.00
UBE2S	1406.00	1344.00	1469.00	1406.33	1955.00
UBE2T	383.00	374.00	370.00	375.67	619.00
UBE2V1	89.00	82.00	93.00	88.00	78.00
UBE2V2	839.00	781.00	914.00	844.67	1249.00
UBE2W	945.00	863.00	1138.00	982.00	1185.00
UBE2Z	2915.00	2800.00	3051.00	2922.00	3373.00
UBE3A	2199.00	2046.00	2399.00	2214.67	2339.00
UBE3B	1561.00	1507.00	1574.00	1547.33	1067.00
UBE3C	3731.00	3448.00	3780.00	3653.00	2924.00
UBE3D	191.00	174.00	195.00	186.67	281.00

UBE4A	2240.00	2013.00	2231.00	2161.33	2112.00
UBE4B	1421.00	1343.00	1432.00	1398.67	1282.00
UBFD1	1748.00	1509.00	1738.00	1665.00	1649.00
UBIAD1	763.00	779.00	744.00	762.00	797.00
UBL3	709.00	586.00	758.00	684.33	639.00
UBL4A	1038.00	1002.00	1023.00	1021.00	1207.00
UBL5	856.00	938.00	955.00	916.33	1195.00
UBL7	934.00	933.00	1028.00	965.00	1255.00
UBL7-AS1	86.00	88.00	113.00	95.67	101.00
UBLCP1	956.00	818.00	1015.00	929.67	1112.00
UBN1	1702.00	1661.00	1861.00	1741.33	1362.00
UBN2	1162.00	1159.00	1288.00	1203.00	958.00
UBOX5	297.00	246.00	261.00	268.00	295.00
UBP1	2677.00	2618.00	2902.00	2732.33	3252.00
UBQLN1	2483.00	2329.00	2731.00	2514.33	3078.00
UBQLN2	433.00	446.00	497.00	458.67	512.00
UBQLN4	1401.00	1374.00	1469.00	1414.67	1337.00
UBQLNL	7.00	10.00	20.00	12.33	19.00
UBR1	1125.00	1013.00	1189.00	1109.00	1167.00
UBR2	1544.00	1356.00	1602.00	1500.67	1308.00
UBR3	917.00	773.00	901.00	863.67	777.00
UBR4	7322.00	6739.00	7576.00	7212.33	5143.00
UBR5	5061.00	4524.00	5380.00	4988.33	4115.00
UBR5-AS1	35.00	31.00	32.00	32.67	30.00
UBTD1	460.00	404.00	402.00	422.00	450.00
UBTD2	426.00	418.00	484.00	442.67	554.00
UBTF	2182.00	2234.00	2426.00	2280.67	2419.00
UBXN1	1840.00	1914.00	1978.00	1910.67	2042.00
UBXN11	342.00	419.00	340.00	367.00	334.00
UBXN2A	189.00	173.00	191.00	184.33	213.00
UBXN2B	735.00	659.00	823.00	739.00	775.00
UBXN4	3333.00	3254.00	3507.00	3364.67	3459.00
UBXN6	1535.00	1496.00	1634.00	1555.00	1748.00
UBXN7	511.00	415.00	480.00	468.67	332.00
UBXN8	515.00	513.00	511.00	513.00	593.00
UCHL1	12287.00	12251.00	12616.00	12384.67	15036.00
UCHL3	849.00	838.00	873.00	853.33	1074.00
UCHL5	1129.00	1069.00	1177.00	1125.00	1483.00
UCK1	480.00	487.00	450.00	472.33	492.00
UCK2	1980.00	1863.00	2131.00	1991.33	2624.00
UCKL1	1218.00	1255.00	1388.00	1287.00	1286.00
UCKL1-AS1	41.00	44.00	68.00	51.00	49.00
UCN	43.00	48.00	49.00	46.67	29.00
UCN2	17.00	16.00	17.00	16.67	6.00
UCP2	3.00	5.00	7.00	5.00	9.00
UCP3	30.00	20.00	23.00	24.33	18.00
UEVLD	293.00	228.00	281.00	267.33	258.00
UFC1	1209.00	1204.00	1375.00	1262.67	1650.00

UFD1L	517.00	507.00	592.00	538.67	718.00
UFL1	1525.00	1274.00	1454.00	1417.67	1460.00
UFM1	1689.00	1587.00	1892.00	1722.67	2147.00
UFSP1	34.00	42.00	44.00	40.00	49.00
UFSP2	363.00	312.00	376.00	350.33	448.00
UGCG	5696.00	5371.00	5598.00	5555.00	5717.00
UGDH	2061.00	1969.00	2141.00	2057.00	1660.00
UGDH-AS1	127.00	87.00	131.00	115.00	96.00
UGGT1	4352.00	3964.00	4200.00	4172.00	3568.00
UGGT2	1155.00	1068.00	1252.00	1158.33	1135.00
UGP2	1349.00	1243.00	1328.00	1306.67	1241.00
UGT8	266.00	230.00	258.00	251.33	237.00
UHMK1	3307.00	2775.00	3475.00	3185.67	2569.00
UHRF1	1216.00	1156.00	1120.00	1164.00	1530.00
UHRF1BP1	1921.00	1798.00	1963.00	1894.00	1768.00
UHRF1BP1L	368.00	314.00	388.00	356.67	387.00
UHRF2	1813.00	1796.00	1894.00	1834.33	1815.00
UIMC1	1030.00	1009.00	1074.00	1037.67	1137.00
ULBP1	35.00	21.00	32.00	29.33	17.00
ULBP2	235.00	195.00	186.00	205.33	209.00
ULBP3	87.00	86.00	77.00	83.33	64.00
ULK1	1025.00	1066.00	1146.00	1079.00	983.00
ULK2	397.00	417.00	405.00	406.33	372.00
ULK3	1250.00	1263.00	1256.00	1256.33	1541.00
ULK4	102.00	94.00	122.00	106.00	129.00
UMAD1	268.00	191.00	266.00	241.67	256.00
UMPS	955.00	872.00	916.00	914.33	992.00
UNC119	434.00	446.00	489.00	456.33	578.00
UNC119B	1559.00	1442.00	1531.00	1510.67	1542.00
UNC13A	8.00	1.00	2.00	3.67	3.00
UNC13B	983.00	930.00	1095.00	1002.67	821.00
UNC13D	67.00	68.00	67.00	67.33	65.00
UNC45A	1832.00	1857.00	2049.00	1912.67	1925.00
UNC50	1242.00	1203.00	1259.00	1234.67	1334.00
UNC5C	5.00	3.00	7.00	5.00	3.00
UNC5CL	44.00	41.00	45.00	43.33	20.00
UNC93B1	499.00	505.00	518.00	507.33	552.00
UNG	285.00	302.00	328.00	305.00	525.00
UNK	967.00	960.00	1023.00	983.33	1184.00
UNKL	681.00	756.00	712.00	716.33	896.00
UOX	3.00	3.00	2.00	2.67	5.00
UPF1	3285.00	3128.00	3347.00	3253.33	3259.00
UPF2	1565.00	1479.00	1584.00	1542.67	1699.00
UPF3A	851.00	890.00	948.00	896.33	930.00
UPF3B	461.00	425.00	420.00	435.33	538.00
UPK3B	3.00	0.00	3.00	2.00	1.00
UPK3BL	21.00	31.00	17.00	23.00	40.00
UPP1	2979.00	2956.00	2994.00	2976.33	3242.00

UPRT	179.00	170.00	188.00	179.00	191.00
UQCC1	1966.00	1989.00	2148.00	2034.33	2073.00
UQCC2	690.00	734.00	760.00	728.00	781.00
UQCC3	302.00	346.00	365.00	337.67	394.00
UQCR10	689.00	676.00	698.00	687.67	1022.00
UQCR11	1203.00	1177.00	1242.00	1207.33	1674.00
UQCRB	2833.00	2821.00	2857.00	2837.00	3837.00
UQCRC1	2468.00	2382.00	2504.00	2451.33	2619.00
UQCRC2	2586.00	2343.00	2782.00	2570.33	2955.00
UQCRFS1	861.00	819.00	1018.00	899.33	1267.00
UQCRH	2563.00	2715.00	2979.00	2752.33	4114.00
UQCRHL	98.00	113.00	138.00	116.33	139.00
UQCRQ	1121.00	1238.00	1219.00	1192.67	1588.00
URB1	3775.00	3479.00	3952.00	3735.33	3193.00
URB1-AS1	58.00	55.00	41.00	51.33	53.00
URB2	848.00	807.00	872.00	842.33	809.00
URGCP	1598.00	1625.00	1873.00	1698.67	1915.00
URI1	1436.00	1355.00	1586.00	1459.00	1811.00
URM1	1513.00	1398.00	1563.00	1491.33	1865.00
UROD	2623.00	2745.00	2746.00	2704.67	3227.00
UROS	710.00	673.00	729.00	704.00	806.00
USB1	975.00	917.00	1026.00	972.67	1029.00
USE1	408.00	381.00	414.00	401.00	497.00
USF1	839.00	876.00	994.00	903.00	1011.00
USF2	1012.00	1041.00	1130.00	1061.00	1233.00
USH2A	7.00	4.00	5.00	5.33	7.00
USMG5	1275.00	1263.00	1297.00	1278.33	1822.00
USO1	2164.00	2032.00	2204.00	2133.33	2211.00
USP1	1237.00	1134.00	1199.00	1190.00	1585.00
USP10	3311.00	3030.00	3414.00	3251.67	3505.00
USP11	1471.00	1501.00	1608.00	1526.67	1607.00
USP12	955.00	846.00	1008.00	936.33	824.00
USP12-AS2	3.00	8.00	6.00	5.67	3.00
USP13	1206.00	1167.00	1341.00	1238.00	1450.00
USP14	4473.00	4033.00	4557.00	4354.33	5243.00
USP15	1375.00	1233.00	1424.00	1344.00	1438.00
USP16	1010.00	1031.00	1120.00	1053.67	1216.00
USP18	17.00	11.00	23.00	17.00	17.00
USP19	2374.00	2287.00	2559.00	2406.67	2232.00
USP20	1465.00	1521.00	1497.00	1494.33	1540.00
USP21	1007.00	1046.00	1093.00	1048.67	1146.00
USP22	5126.00	4829.00	5368.00	5107.67	5242.00
USP24	2991.00	2684.00	3031.00	2902.00	2699.00
USP25	836.00	832.00	1023.00	897.00	1109.00
USP27X	90.00	104.00	119.00	104.33	129.00
USP27X-AS1	19.00	21.00	23.00	21.00	17.00
USP28	918.00	955.00	960.00	944.33	878.00
USP2-AS1	17.00	25.00	23.00	21.67	16.00

USP3	1357.00	1316.00	1389.00	1354.00	1491.00
USP30	319.00	348.00	344.00	337.00	336.00
USP31	725.00	671.00	877.00	757.67	804.00
USP32	1791.00	1684.00	1925.00	1800.00	1752.00
USP32P1	2157.00	2170.00	2280.00	2202.33	1930.00
USP32P2	14.00	19.00	30.00	21.00	20.00
USP33	1738.00	1625.00	1815.00	1726.00	1959.00
USP34	3186.00	2992.00	3428.00	3202.00	3199.00
USP35	292.00	279.00	303.00	291.33	242.00
USP36	2800.00	2586.00	2882.00	2756.00	2115.00
USP37	286.00	285.00	333.00	301.33	290.00
USP38	395.00	325.00	407.00	375.67	330.00
USP39	1113.00	1091.00	1168.00	1124.00	1343.00
USP3-AS1	35.00	27.00	27.00	29.67	24.00
USP4	1623.00	1532.00	1680.00	1611.67	1671.00
USP40	2321.00	2153.00	2392.00	2288.67	2283.00
USP42	610.00	572.00	640.00	607.33	619.00
USP45	837.00	716.00	870.00	807.67	640.00
USP46	542.00	501.00	561.00	534.67	547.00
USP46-AS1	25.00	27.00	17.00	23.00	26.00
USP47	2221.00	1970.00	2324.00	2171.67	2378.00
USP48	1934.00	1923.00	2100.00	1985.67	2036.00
USP49	484.00	550.00	521.00	518.33	452.00
USP5	2608.00	2372.00	2561.00	2513.67	2659.00
USP51	33.00	34.00	40.00	35.67	47.00
USP53	1386.00	1286.00	1473.00	1381.67	1314.00
USP54	1045.00	992.00	1065.00	1034.00	923.00
USP6	43.00	47.00	33.00	41.00	61.00
USP6NL	569.00	550.00	626.00	581.67	666.00
USP7	3194.00	3083.00	3438.00	3238.33	3370.00
USP8	1295.00	1119.00	1391.00	1268.33	1344.00
USP9X	3799.00	3556.00	4199.00	3851.33	3432.00
USPL1	800.00	803.00	873.00	825.33	774.00
UST	951.00	932.00	914.00	932.33	927.00
UTP11L	921.00	794.00	914.00	876.33	1181.00
UTP14A	1369.00	1345.00	1647.00	1453.67	1705.00
UTP14C	863.00	788.00	856.00	835.67	744.00
UTP15	658.00	630.00	708.00	665.33	762.00
UTP18	1902.00	1793.00	2086.00	1927.00	2277.00
UTP20	2042.00	1977.00	2075.00	2031.33	1871.00
UTP23	598.00	546.00	663.00	602.33	763.00
UTP3	889.00	953.00	912.00	918.00	1126.00
UTP6	1535.00	1502.00	1620.00	1552.33	1885.00
UTRN	2008.00	1616.00	1926.00	1850.00	1026.00
UVRAG	802.00	710.00	778.00	763.33	851.00
UVSSA	516.00	437.00	538.00	497.00	445.00
UXS1	864.00	831.00	902.00	865.67	1024.00
UXT	574.00	508.00	550.00	544.00	643.00

UXT-AS1	8.00	11.00	12.00	10.33	7.00
VAC14	1240.00	1220.00	1343.00	1267.67	1217.00
VAMP1	482.00	498.00	521.00	500.33	433.00
VAMP2	1593.00	1537.00	1751.00	1627.00	1962.00
VAMP3	1356.00	1249.00	1483.00	1362.67	1637.00
VAMP4	752.00	705.00	813.00	756.67	770.00
VAMP5	23.00	16.00	21.00	20.00	26.00
VAMP8	5.00	5.00	2.00	4.00	1.00
VANGL1	1029.00	938.00	986.00	984.33	947.00
VAPA	3846.00	3457.00	4207.00	3836.67	4861.00
VAPB	1598.00	1454.00	1618.00	1556.67	1808.00
VARS	10.00	7.00	2.00	6.33	5.00
VARS2	181.00	162.00	162.00	168.33	138.00
VASH1	166.00	163.00	189.00	172.67	172.00
VASH2	7.00	3.00	5.00	5.00	6.00
VASP	1810.00	1748.00	1775.00	1777.67	1756.00
VAT1	5429.00	5398.00	5665.00	5497.33	4464.00
VAV2	1012.00	1001.00	1161.00	1058.00	982.00
VAV3	4.00	5.00	6.00	5.00	3.00
VAX2	45.00	64.00	47.00	52.00	74.00
VBP1	826.00	720.00	859.00	801.67	1158.00
VCAM1	1807.00	1757.00	1768.00	1777.33	2633.00
VCAN	483.00	421.00	495.00	466.33	416.00
VCL	11048.00	10190.00	11219.00	10819.00	9974.00
VCP	7581.00	7431.00	7625.00	7545.67	8400.00
VCPIP1	690.00	615.00	729.00	678.00	671.00
VCPKMT	273.00	278.00	310.00	287.00	315.00
VDAC1	4663.00	4345.00	4830.00	4612.67	6886.00
VDAC2	2344.00	2390.00	2514.00	2416.00	3189.00
VDAC3	1390.00	1364.00	1424.00	1392.67	1841.00
VDR	192.00	164.00	184.00	180.00	99.00
VEGFA	8362.00	8343.00	8572.00	8425.67	6382.00
VEGFB	1332.00	1421.00	1475.00	1409.33	1425.00
VEGFC	1760.00	1673.00	1660.00	1697.67	1457.00
VEPH1	1461.00	1243.00	1532.00	1412.00	1815.00
VEZF1	1679.00	1567.00	1877.00	1707.67	1794.00
VEZT	1326.00	1295.00	1348.00	1323.00	1474.00
VGF	214.00	263.00	241.00	239.33	190.00
VGLL2	49.00	44.00	42.00	45.00	62.00
VGLL3	627.00	521.00	745.00	631.00	402.00
VGLL4	1039.00	1033.00	1209.00	1093.67	1105.00
VHL	1216.00	1230.00	1262.00	1236.00	1425.00
VILL	55.00	53.00	54.00	54.00	59.00
VIM	101688.00	98800.00	108880.00	103122.67	138701.00
VIM-AS1	75.00	103.00	93.00	90.33	137.00
VIMP	1199.00	1141.00	1113.00	1151.00	1255.00
VIP	17.00	13.00	18.00	16.00	10.00
VIPAS39	576.00	609.00	664.00	616.33	646.00

VIPR1	157.00	170.00	162.00	163.00	62.00
VKORC1	2292.00	2251.00	2055.00	2199.33	2385.00
VKORC1L1	1281.00	1244.00	1347.00	1290.67	1391.00
VLDLR	1518.00	1480.00	1430.00	1476.00	1327.00
VLDLR-AS1	122.00	102.00	108.00	110.67	155.00
VMA21	1027.00	960.00	1118.00	1035.00	1526.00
VMAC	101.00	89.00	110.00	100.00	67.00
VMO1	7.00	4.00	14.00	8.33	9.00
VMP1	3925.00	3917.00	4067.00	3969.67	4298.00
VN1R1	6.00	6.00	7.00	6.33	2.00
VNN1	345.00	344.00	384.00	357.67	486.00
VOPP1	807.00	827.00	817.00	817.00	907.00
VPRBP	1340.00	1289.00	1483.00	1370.67	1328.00
VPS11	950.00	859.00	1060.00	956.33	995.00
VPS13A	1457.00	1410.00	1551.00	1472.67	1246.00
VPS13B	1530.00	1406.00	1699.00	1545.00	1266.00
VPS13C	1782.00	1684.00	1977.00	1814.33	1661.00
VPS13D	1245.00	1137.00	1239.00	1207.00	929.00
VPS16	1100.00	1182.00	1211.00	1164.33	1289.00
VPS18	739.00	764.00	820.00	774.33	689.00
VPS25	1354.00	1277.00	1408.00	1346.33	1594.00
VPS26A	1759.00	1596.00	1883.00	1746.00	2107.00
VPS26B	825.00	749.00	865.00	813.00	844.00
VPS28	1964.00	1922.00	2038.00	1974.67	2530.00
VPS29	927.00	941.00	1036.00	968.00	1282.00
VPS33A	780.00	753.00	801.00	778.00	835.00
VPS33B	374.00	390.00	353.00	372.33	351.00
VPS35	2624.00	2388.00	2722.00	2578.00	3017.00
VPS36	1115.00	1132.00	1305.00	1184.00	1365.00
VPS37A	607.00	549.00	674.00	610.00	675.00
VPS37B	1196.00	1227.00	1345.00	1256.00	1340.00
VPS37C	518.00	469.00	538.00	508.33	637.00
VPS37D	28.00	26.00	34.00	29.33	49.00
VPS39	2033.00	1830.00	1970.00	1944.33	1718.00
VPS41	1378.00	1227.00	1415.00	1340.00	1382.00
VPS45	747.00	815.00	802.00	788.00	932.00
VPS4A	1874.00	1834.00	2002.00	1903.33	2088.00
VPS4B	893.00	749.00	903.00	848.33	855.00
VPS50	586.00	547.00	703.00	612.00	637.00
VPS51	2130.00	2257.00	2447.00	2278.00	2454.00
VPS52	53.00	41.00	38.00	44.00	53.00
VPS53	704.00	671.00	647.00	674.00	722.00
VPS54	1176.00	1035.00	1224.00	1145.00	1240.00
VPS72	1058.00	1022.00	1078.00	1052.67	1331.00
VPS8	1219.00	1242.00	1277.00	1246.00	1130.00
VPS9D1	485.00	506.00	494.00	495.00	522.00
VPS9D1-AS1	719.00	677.00	756.00	717.33	640.00
VRK1	653.00	617.00	604.00	624.67	884.00

VRK2	499.00	473.00	578.00	516.67	588.00
VRK3	615.00	641.00	594.00	616.67	599.00
VSIG10	572.00	580.00	607.00	586.33	615.00
VSIG10L	19.00	23.00	14.00	18.67	20.00
VSIG2	5.00	5.00	8.00	6.00	5.00
VSIG8	8.00	7.00	3.00	6.00	5.00
VTA1	799.00	664.00	769.00	744.00	1126.00
VTI1A	628.00	629.00	740.00	665.67	599.00
VTI1B	898.00	891.00	1030.00	939.67	1026.00
VTN	45.00	39.00	34.00	39.33	34.00
VWA5A	5.00	17.00	11.00	11.00	18.00
VWA8	843.00	794.00	868.00	835.00	794.00
VWA9	1291.00	1220.00	1308.00	1273.00	1494.00
VWCE	5.00	9.00	5.00	6.33	12.00
VWDE	5.00	7.00	18.00	10.00	5.00
VWF	4.00	0.00	2.00	2.00	3.00
WAC	4314.00	4153.00	4697.00	4388.00	4781.00
WAC-AS1	600.00	583.00	599.00	594.00	673.00
WAPL	1898.00	1740.00	2050.00	1896.00	1952.00
WARS	5469.00	5021.00	5429.00	5306.33	5751.00
WARS2	295.00	277.00	298.00	290.00	353.00
WAS	6.00	5.00	1.00	4.00	0.00
WASF1	824.00	674.00	798.00	765.33	725.00
WASF2	2429.00	2306.00	2675.00	2470.00	1871.00
WASF3	556.00	561.00	649.00	588.67	619.00
WASH1	226.00	234.00	233.00	231.00	222.00
WASH2P	149.00	200.00	208.00	185.67	189.00
WASH3P	220.00	236.00	171.00	209.00	198.00
WASH5P	12.00	23.00	13.00	16.00	12.00
WASH7P	117.00	119.00	132.00	122.67	125.00
WASL	962.00	790.00	1081.00	944.33	933.00
WBP1	160.00	140.00	203.00	167.67	152.00
WBP11	2179.00	2003.00	2197.00	2126.33	2448.00
WBP1L	826.00	814.00	805.00	815.00	806.00
WBP2	1825.00	1780.00	1784.00	1796.33	1942.00
WBP4	343.00	346.00	360.00	349.67	412.00
WBP5	1396.00	1515.00	1541.00	1484.00	1756.00
WBSCR16	917.00	877.00	897.00	897.00	1055.00
WBSCR22	2062.00	1996.00	1990.00	2016.00	2452.00
WBSCR27	621.00	562.00	666.00	616.33	641.00
WDFY1	1736.00	1495.00	1914.00	1715.00	1487.00
WDFY2	1383.00	1243.00	1267.00	1297.67	1585.00
WDFY3	626.00	622.00	622.00	623.33	489.00
WDFY3-AS2	40.00	30.00	34.00	34.67	18.00
WDHD1	626.00	601.00	628.00	618.33	755.00
WDPCP	95.00	105.00	89.00	96.33	91.00
WDR1	5271.00	5078.00	5454.00	5267.67	4886.00
WDR11	2076.00	1823.00	2115.00	2004.67	2018.00

WDR12	1732.00	1670.00	1830.00	1744.00	2219.00
WDR13	912.00	882.00	914.00	902.67	1047.00
WDR17	463.00	414.00	516.00	464.33	559.00
WDR18	1042.00	920.00	1066.00	1009.33	1310.00
WDR19	2252.00	2081.00	2277.00	2203.33	1828.00
WDR20	475.00	426.00	456.00	452.33	467.00
WDR24	441.00	544.00	531.00	505.33	520.00
WDR25	189.00	161.00	171.00	173.67	161.00
WDR26	3362.00	3225.00	3598.00	3395.00	3428.00
WDR27	2874.00	2885.00	2781.00	2846.67	2302.00
WDR3	3166.00	3003.00	3357.00	3175.33	3431.00
WDR31	51.00	59.00	73.00	61.00	55.00
WDR33	2414.00	2444.00	2507.00	2455.00	2379.00
WDR34	700.00	732.00	745.00	725.67	1121.00
WDR35	2259.00	2050.00	2434.00	2247.67	2267.00
WDR36	2537.00	2238.00	2613.00	2462.67	2313.00
WDR37	988.00	939.00	1022.00	983.00	955.00
WDR4	525.00	481.00	502.00	502.67	422.00
WDR41	516.00	419.00	501.00	478.67	548.00
WDR43	2761.00	2519.00	3114.00	2798.00	3072.00
WDR44	637.00	522.00	585.00	581.33	472.00
WDR45	1057.00	1064.00	1174.00	1098.33	1112.00
WDR45B	2432.00	2397.00	2441.00	2423.33	3039.00
WDR46	118.00	153.00	115.00	128.67	146.00
WDR47	362.00	332.00	401.00	365.00	373.00
WDR48	852.00	819.00	985.00	885.33	963.00
WDR5	1900.00	1960.00	1957.00	1939.00	2318.00
WDR53	344.00	326.00	318.00	329.33	378.00
WDR54	750.00	772.00	755.00	759.00	1057.00
WDR55	707.00	766.00	775.00	749.33	674.00
WDR59	1854.00	1970.00	2109.00	1977.67	2190.00
WDR5B	601.00	667.00	695.00	654.33	591.00
WDR6	6399.00	6451.00	6881.00	6577.00	6087.00
WDR60	597.00	543.00	668.00	602.67	546.00
WDR61	708.00	660.00	731.00	699.67	853.00
WDR62	643.00	658.00	658.00	653.00	782.00
WDR63	11.00	7.00	13.00	10.33	10.00
WDR64	3.00	3.00	0.00	2.00	0.00
WDR66	374.00	322.00	434.00	376.67	1083.00
WDR7	334.00	327.00	413.00	358.00	319.00
WDR70	838.00	820.00	886.00	848.00	989.00
WDR72	35.00	17.00	28.00	26.67	31.00
WDR73	1286.00	1346.00	1256.00	1296.00	1319.00
WDR74	1528.00	1578.00	1672.00	1592.67	1692.00
WDR75	2791.00	2683.00	3043.00	2839.00	3176.00
WDR76	140.00	140.00	124.00	134.67	224.00
WDR77	1893.00	1768.00	1831.00	1830.67	2236.00
WDR78	63.00	79.00	89.00	77.00	98.00

WDR81	311.00	255.00	271.00	279.00	318.00
WDR82	1706.00	1603.00	1827.00	1712.00	1749.00
WDR83	195.00	198.00	205.00	199.33	269.00
WDR83OS	1482.00	1462.00	1456.00	1466.67	1846.00
WDR89	492.00	450.00	489.00	477.00	505.00
WDR90	1366.00	1398.00	1360.00	1374.67	1486.00
WDR91	626.00	636.00	680.00	647.33	555.00
WDR92	434.00	365.00	434.00	411.00	466.00
WDSUB1	329.00	304.00	356.00	329.67	396.00
WDTC1	921.00	994.00	999.00	971.33	1000.00
WDYHV1	327.00	278.00	336.00	313.67	451.00
WEE1	439.00	439.00	477.00	451.67	590.00
WFDC3	7.00	6.00	2.00	5.00	6.00
WFIKKN1	20.00	29.00	18.00	22.33	24.00
WFS1	1010.00	1043.00	1012.00	1021.67	886.00
WHAMM	241.00	211.00	243.00	231.67	216.00
WHAMMP1	85.00	118.00	114.00	105.67	87.00
WHAMMP2	5.00	2.00	2.00	3.00	5.00
WHAMMP3	83.00	108.00	112.00	101.00	124.00
WHSC1	8622.00	8251.00	8732.00	8535.00	10057.00
WHSC1L1	1291.00	1106.00	1289.00	1228.67	1116.00
WIBG	275.00	267.00	259.00	267.00	293.00
WIPF1	703.00	644.00	810.00	719.00	683.00
WIPF2	905.00	856.00	923.00	894.67	784.00
WIPI1	556.00	544.00	565.00	555.00	580.00
WIPI2	1995.00	1828.00	1975.00	1932.67	2061.00
WISP1	25.00	17.00	29.00	23.67	39.00
WIZ	1157.00	1086.00	1202.00	1148.33	1219.00
WLS	4596.00	4494.00	4728.00	4606.00	4523.00
WNK1	6964.00	6214.00	7463.00	6880.33	5614.00
WNK3	8.00	6.00	8.00	7.33	8.00
WNK4	2505.00	2514.00	2776.00	2598.33	1281.00
WNT10A	3.00	5.00	4.00	4.00	1.00
WNT10B	7.00	5.00	10.00	7.33	20.00
WNT2B	93.00	80.00	92.00	88.33	76.00
WNT5A	11.00	5.00	12.00	9.33	11.00
WNT5B	438.00	479.00	487.00	468.00	1043.00
WNT7B	4.00	2.00	9.00	5.00	0.00
WRAP53	484.00	462.00	449.00	465.00	523.00
WRAP73	642.00	656.00	657.00	651.67	703.00
WRB	427.00	390.00	462.00	426.33	586.00
WRN	870.00	783.00	941.00	864.67	934.00
WRNIP1	2171.00	2106.00	2277.00	2184.67	2545.00
WSB1	1880.00	1711.00	1808.00	1799.67	2061.00
WSB2	812.00	702.00	823.00	779.00	1012.00
WT1	33.00	30.00	23.00	28.67	43.00
WT1-AS	13.00	21.00	22.00	18.67	18.00
WTAP	1193.00	1175.00	1348.00	1238.67	1463.00

WTAPP1	92.00	92.00	94.00	92.67	162.00
WTH3DI	18.00	14.00	24.00	18.67	18.00
WTIP	11.00	18.00	7.00	12.00	16.00
WWC1	2609.00	2475.00	2651.00	2578.33	3350.00
WWC2	1039.00	942.00	1106.00	1029.00	1011.00
WWC2-AS2	5.00	6.00	9.00	6.67	6.00
WWC3	1053.00	1045.00	1078.00	1058.67	819.00
WWP1	781.00	658.00	850.00	763.00	869.00
WWP2	814.00	896.00	887.00	865.67	837.00
WWTR1	607.00	550.00	631.00	596.00	482.00
WWTR1-AS1	9.00	7.00	12.00	9.33	14.00
XAB2	1693.00	1654.00	1629.00	1658.67	1606.00
XAF1	49.00	40.00	59.00	49.33	71.00
XBP1	6581.00	6282.00	6159.00	6340.67	7182.00
XDH	189.00	136.00	163.00	162.67	618.00
XIAP	1159.00	1005.00	1300.00	1154.67	1060.00
XIRP2	329.00	271.00	318.00	306.00	302.00
XIST	22549.00	22088.00	24496.00	23044.33	17212.00
XK	15.00	10.00	8.00	11.00	22.00
XKR9	6.00	1.00	1.00	2.67	1.00
XPA	162.00	154.00	159.00	158.33	200.00
XPC	1510.00	1483.00	1665.00	1552.67	1274.00
XPNPEP1	1317.00	1240.00	1348.00	1301.67	1451.00
XPNPEP3	651.00	664.00	722.00	679.00	766.00
XPO1	10271.00	10192.00	11526.00	10663.00	10837.00
XPO4	1234.00	1023.00	1300.00	1185.67	1249.00
XPO5	2884.00	2726.00	3161.00	2923.67	2971.00
XPO6	3302.00	3372.00	3547.00	3407.00	3560.00
XPO7	1460.00	1330.00	1573.00	1454.33	1387.00
XPOT	10375.00	9381.00	11076.00	10277.33	11245.00
XPR1	1321.00	1181.00	1318.00	1273.33	1231.00
XRCC1	844.00	832.00	852.00	842.67	861.00
XRCC2	469.00	450.00	439.00	452.67	492.00
XRCC3	790.00	842.00	841.00	824.33	948.00
XRCC4	260.00	239.00	240.00	246.33	332.00
XRCC5	7584.00	6958.00	7975.00	7505.67	8841.00
XRCC6	6915.00	6443.00	7071.00	6809.67	7905.00
XRN1	1243.00	1022.00	1259.00	1174.67	968.00
XRN2	4443.00	3939.00	4537.00	4306.33	4645.00
XRRA1	632.00	639.00	702.00	657.67	532.00
XXYLT1	704.00	758.00	757.00	739.67	724.00
XYLB	231.00	231.00	229.00	230.33	219.00
XYLT2	2046.00	2042.00	2156.00	2081.33	1823.00
YAE1D1	287.00	349.00	371.00	335.67	428.00
YAF2	263.00	237.00	280.00	260.00	333.00
YAP1	14182.00	13148.00	15479.00	14269.67	14930.00
YARS	6774.00	6518.00	6926.00	6739.33	7668.00
YARS2	823.00	823.00	868.00	838.00	920.00

YBEY	267.00	207.00	238.00	237.33	249.00
YBX1	10758.00	10092.00	11133.00	10661.00	15796.00
YBX3	4450.00	4369.00	4716.00	4511.67	6045.00
YBX3P1	28.00	21.00	25.00	24.67	34.00
YDJC	610.00	627.00	635.00	624.00	757.00
YEATS2	2225.00	2031.00	2433.00	2229.67	2245.00
YEATS4	457.00	438.00	461.00	452.00	626.00
YES1	2651.00	2389.00	2882.00	2640.67	3906.00
YIF1A	2399.00	2342.00	2132.00	2291.00	2730.00
YIF1B	1372.00	1294.00	1210.00	1292.00	1120.00
YIPF1	528.00	493.00	501.00	507.33	582.00
YIPF2	838.00	847.00	860.00	848.33	895.00
YIPF3	4056.00	4000.00	3733.00	3929.67	3968.00
YIPF4	914.00	803.00	854.00	857.00	876.00
YIPF5	1501.00	1482.00	1538.00	1507.00	1583.00
YIPF6	2228.00	2148.00	2256.00	2210.67	1979.00
YJEFN3	358.00	421.00	431.00	403.33	308.00
YKT6	2592.00	2513.00	2890.00	2665.00	2946.00
YLPM1	2327.00	2134.00	2413.00	2291.33	2031.00
YME1L1	4361.00	3954.00	4629.00	4314.67	5037.00
YOD1	578.00	541.00	724.00	614.33	459.00
YPEL1	7.00	9.00	11.00	9.00	14.00
YPEL2	54.00	55.00	49.00	52.67	64.00
YPEL3	225.00	191.00	230.00	215.33	193.00
YPEL4	7.00	5.00	2.00	4.67	3.00
YPEL5	626.00	556.00	680.00	620.67	677.00
YRDC	797.00	700.00	830.00	775.67	1049.00
YTHDC1	4.00	1.00	2.00	2.33	1.00
YTHDC2	1496.00	1407.00	1551.00	1484.67	1427.00
YTHDF1	1447.00	1418.00	1465.00	1443.33	1648.00
YTHDF2	2018.00	2041.00	2073.00	2044.00	2396.00
YTHDF3	2102.00	1856.00	2346.00	2101.33	2272.00
YTHDF3-AS1	6.00	2.00	9.00	5.67	19.00
YWHAB	5555.00	5439.00	5865.00	5619.67	7114.00
YWHAE	31.00	36.00	38.00	35.00	51.00
YWHAEP1	9.00	7.00	8.00	8.00	9.00
YWHAG	3695.00	3342.00	3782.00	3606.33	4014.00
YWHAH	915.00	857.00	926.00	899.33	1187.00
YWHAQ	4483.00	4179.00	4678.00	4446.67	5430.00
YWHAZ	10152.00	9189.00	11040.00	10127.00	12545.00
YY1	1390.00	1234.00	1520.00	1381.33	1538.00
YY1AP1	1104.00	1018.00	1082.00	1068.00	1038.00
YY2	95.00	89.00	114.00	99.33	83.00
ZADH2	787.00	720.00	916.00	807.67	877.00
ZAK	1148.00	967.00	1188.00	1101.00	1087.00
ZAP70	4.00	3.00	0.00	2.33	0.00
ZAR1	3.00	0.00	0.00	1.00	1.00
ZBBX	8.00	13.00	5.00	8.67	9.00

ZBED3	118.00	107.00	109.00	111.33	69.00
ZBED3-AS1	30.00	57.00	49.00	45.33	57.00
ZBED4	1411.00	1317.00	1513.00	1413.67	1362.00
ZBED5	1279.00	1281.00	1365.00	1308.33	1298.00
ZBED5-AS1	61.00	68.00	67.00	65.33	87.00
ZBED6	288.00	190.00	209.00	229.00	129.00
ZBED8	264.00	206.00	238.00	236.00	262.00
ZBTB1	1126.00	1149.00	1190.00	1155.00	1200.00
ZBTB10	498.00	395.00	563.00	485.33	490.00
ZBTB11	1201.00	1086.00	1237.00	1174.67	1169.00
ZBTB11-AS1	80.00	92.00	76.00	82.67	84.00
ZBTB12	20.00	7.00	8.00	11.67	13.00
ZBTB14	582.00	600.00	604.00	595.33	658.00
ZBTB17	841.00	896.00	884.00	873.67	959.00
ZBTB18	1075.00	1080.00	1224.00	1126.33	1159.00
ZBTB2	505.00	529.00	559.00	531.00	509.00
ZBTB20	499.00	477.00	445.00	473.67	323.00
ZBTB21	956.00	904.00	1101.00	987.00	896.00
ZBTB22	13.00	5.00	3.00	7.00	10.00
ZBTB24	508.00	433.00	499.00	480.00	545.00
ZBTB25	223.00	235.00	249.00	235.67	256.00
ZBTB26	286.00	293.00	349.00	309.33	236.00
ZBTB3	106.00	97.00	91.00	98.00	74.00
ZBTB33	518.00	523.00	617.00	552.67	559.00
ZBTB34	348.00	333.00	420.00	367.00	339.00
ZBTB37	50.00	32.00	51.00	44.33	43.00
ZBTB38	4342.00	3663.00	4484.00	4163.00	4016.00
ZBTB39	521.00	491.00	564.00	525.33	493.00
ZBTB4	2005.00	1896.00	2264.00	2055.00	1754.00
ZBTB40	3427.00	3335.00	3621.00	3461.00	2907.00
ZBTB41	374.00	298.00	376.00	349.33	308.00
ZBTB42	25.00	25.00	31.00	27.00	17.00
ZBTB43	497.00	549.00	608.00	551.33	601.00
ZBTB44	549.00	616.00	718.00	627.67	636.00
ZBTB45	210.00	226.00	258.00	231.33	219.00
ZBTB46	14.00	6.00	13.00	11.00	5.00
ZBTB47	584.00	574.00	629.00	595.67	500.00
ZBTB48	602.00	660.00	710.00	657.33	692.00
ZBTB49	111.00	101.00	115.00	109.00	138.00
ZBTB5	478.00	514.00	593.00	528.33	458.00
ZBTB6	296.00	257.00	323.00	292.00	285.00
ZBTB7A	960.00	972.00	1101.00	1011.00	969.00
ZBTB7B	681.00	602.00	630.00	637.67	640.00
ZBTB8A	109.00	108.00	121.00	112.67	109.00
ZBTB8OS	318.00	335.00	311.00	321.33	441.00
ZBTB9	25.00	33.00	23.00	27.00	17.00
ZC2HC1A	339.00	244.00	359.00	314.00	299.00
ZC2HC1C	15.00	15.00	26.00	18.67	20.00

ZC3H10	143.00	147.00	161.00	150.33	143.00
ZC3H11A	4290.00	4194.00	4916.00	4466.67	4904.00
ZC3H12A	241.00	217.00	203.00	220.33	199.00
ZC3H12C	1062.00	976.00	1214.00	1084.00	866.00
ZC3H12D	5.00	1.00	4.00	3.33	5.00
ZC3H13	1787.00	1726.00	1898.00	1803.67	1509.00
ZC3H14	2023.00	2021.00	2136.00	2060.00	2268.00
ZC3H15	2640.00	2464.00	2830.00	2644.67	3481.00
ZC3H18	1987.00	1920.00	1963.00	1956.67	1826.00
ZC3H3	430.00	383.00	385.00	399.33	374.00
ZC3H4	1220.00	1129.00	1082.00	1143.67	1130.00
ZC3H6	382.00	351.00	402.00	378.33	307.00
ZC3H7A	1545.00	1420.00	1581.00	1515.33	1745.00
ZC3H7B	3558.00	3485.00	3665.00	3569.33	4116.00
ZC3H8	499.00	498.00	549.00	515.33	625.00
ZC3HAV1	1133.00	1140.00	1172.00	1148.33	1123.00
ZC3HAV1L	287.00	264.00	266.00	272.33	241.00
ZC3HC1	764.00	805.00	798.00	789.00	1007.00
ZCCHC10	153.00	139.00	173.00	155.00	188.00
ZCCHC11	1674.00	1619.00	1748.00	1680.33	1536.00
ZCCHC14	1558.00	1489.00	1728.00	1591.67	1493.00
ZCCHC17	649.00	599.00	638.00	628.67	721.00
ZCCHC18	226.00	232.00	213.00	223.67	272.00
ZCCHC2	323.00	314.00	385.00	340.67	350.00
ZCCHC24	142.00	171.00	191.00	168.00	90.00
ZCCHC3	836.00	791.00	886.00	837.67	1100.00
ZCCHC4	301.00	307.00	325.00	311.00	447.00
ZCCHC6	587.00	566.00	668.00	607.00	539.00
ZCCHC7	890.00	916.00	1078.00	961.33	1137.00
ZCCHC8	1368.00	1269.00	1323.00	1320.00	1519.00
ZCCHC9	1092.00	1074.00	1307.00	1157.67	1602.00
ZCRB1	788.00	727.00	810.00	775.00	920.00
ZCWPW1	224.00	209.00	244.00	225.67	221.00
ZCWPW2	18.00	20.00	15.00	17.67	21.00
ZDBF2	992.00	916.00	998.00	968.67	745.00
ZDHHC1	261.00	302.00	283.00	282.00	295.00
ZDHHC11	71.00	76.00	95.00	80.67	50.00
ZDHHC12	332.00	348.00	273.00	317.67	447.00
ZDHHC13	419.00	440.00	444.00	434.33	515.00
ZDHHC14	539.00	471.00	533.00	514.33	482.00
ZDHHC16	1033.00	1016.00	995.00	1014.67	1083.00
ZDHHC17	1090.00	1013.00	1041.00	1048.00	901.00
ZDHHC18	748.00	784.00	771.00	767.67	818.00
ZDHHC2	386.00	414.00	518.00	439.33	414.00
ZDHHC20	879.00	814.00	943.00	878.67	901.00
ZDHHC21	1194.00	1133.00	1299.00	1208.67	1343.00
ZDHHC23	408.00	420.00	547.00	458.33	613.00
ZDHHC24	724.00	710.00	666.00	700.00	846.00

ZDHHC3	1184.00	1154.00	1180.00	1172.67	1046.00
ZDHHC4	923.00	955.00	1001.00	959.67	1127.00
ZDHHC5	3917.00	3729.00	3756.00	3800.67	3490.00
ZDHHC6	2070.00	2125.00	2148.00	2114.33	2303.00
ZDHHC7	3280.00	3141.00	3292.00	3237.67	3188.00
ZDHHC8	2118.00	2036.00	2121.00	2091.67	2117.00
ZDHHC9	1356.00	1342.00	1400.00	1366.00	1421.00
ZEB1	2302.00	2120.00	2586.00	2336.00	2462.00
ZEB1-AS1	347.00	322.00	354.00	341.00	369.00
ZEB2	1768.00	1573.00	1843.00	1728.00	1678.00
ZER1	1073.00	1047.00	1051.00	1057.00	867.00
ZFAND1	1447.00	1391.00	1599.00	1479.00	1929.00
ZFAND2A	385.00	347.00	343.00	358.33	449.00
ZFAND2B	428.00	440.00	402.00	423.33	467.00
ZFAND3	2182.00	2129.00	2232.00	2181.00	2366.00
ZFAND4	129.00	116.00	155.00	133.33	186.00
ZFAND5	1962.00	1923.00	2185.00	2023.33	2028.00
ZFAND6	862.00	751.00	925.00	846.00	1055.00
ZFAS1	4093.00	3995.00	4487.00	4191.67	5537.00
ZFAT	315.00	264.00	381.00	320.00	301.00
ZFC3H1	3641.00	3492.00	3875.00	3669.33	3195.00
ZFHX2	32.00	26.00	37.00	31.67	28.00
ZFHX3	193.00	221.00	221.00	211.67	137.00
ZFHX4	1349.00	1105.00	1410.00	1288.00	1008.00
ZFHX4-AS1	19.00	10.00	31.00	20.00	16.00
ZFP1	259.00	268.00	262.00	263.00	290.00
ZFP14	16.00	18.00	22.00	18.67	21.00
ZFP2	4.00	4.00	5.00	4.33	5.00
ZFP28	52.00	36.00	57.00	48.33	38.00
ZFP30	108.00	83.00	88.00	93.00	102.00
ZFP36	135.00	118.00	141.00	131.33	136.00
ZFP36L1	3778.00	3575.00	4071.00	3808.00	2637.00
ZFP36L2	677.00	583.00	676.00	645.33	801.00
ZFP37	137.00	91.00	144.00	124.00	109.00
ZFP41	963.00	784.00	925.00	890.67	643.00
ZFP62	1416.00	1407.00	1633.00	1485.33	1567.00
ZFP64	343.00	339.00	360.00	347.33	412.00
ZFP69	136.00	136.00	142.00	138.00	172.00
ZFP69B	211.00	199.00	232.00	214.00	230.00
ZFP82	25.00	19.00	19.00	21.00	24.00
ZFP90	549.00	530.00	554.00	544.33	539.00
ZFP91	1613.00	1444.00	1867.00	1641.33	1769.00
ZFPL1	1173.00	1196.00	1261.00	1210.00	1417.00
ZFPM1	164.00	156.00	169.00	163.00	203.00
ZFPM2	113.00	106.00	107.00	108.67	221.00
ZFPM2-AS1	294.00	297.00	273.00	288.00	197.00
ZFR	2862.00	2558.00	2982.00	2800.67	2873.00
ZFX	1065.00	1002.00	1184.00	1083.67	1146.00

ZFYVE1	442.00	420.00	450.00	437.33	348.00
ZFYVE16	873.00	837.00	935.00	881.67	814.00
ZFYVE19	531.00	573.00	542.00	548.67	619.00
ZFYVE21	527.00	560.00	567.00	551.33	522.00
ZFYVE26	1187.00	1173.00	1255.00	1205.00	1025.00
ZFYVE27	1945.00	1955.00	1969.00	1956.33	1844.00
ZFYVE28	78.00	79.00	67.00	74.67	46.00
ZFYVE9	1111.00	1072.00	1149.00	1110.67	1033.00
ZGLP1	203.00	227.00	211.00	213.67	187.00
ZGPAT	1326.00	1486.00	1538.00	1450.00	1507.00
ZGRF1	390.00	352.00	358.00	366.67	334.00
ZHX1	706.00	631.00	766.00	701.00	616.00
ZHX1-C8orf76	30.00	30.00	37.00	32.33	26.00
ZHX2	203.00	220.00	227.00	216.67	211.00
ZHX3	2330.00	2273.00	2391.00	2331.33	2621.00
ZIC1	201.00	235.00	291.00	242.33	274.00
ZIC2	204.00	173.00	200.00	192.33	226.00
ZIC4	7.00	11.00	9.00	9.00	10.00
ZIC5	127.00	72.00	131.00	110.00	121.00
ZIK1	15.00	19.00	17.00	17.00	14.00
ZKSCAN1	3735.00	3506.00	4085.00	3775.33	3396.00
ZKSCAN2	350.00	287.00	356.00	331.00	303.00
ZKSCAN3	142.00	151.00	166.00	153.00	169.00
ZKSCAN4	183.00	212.00	210.00	201.67	208.00
ZKSCAN5	565.00	542.00	579.00	562.00	588.00
ZKSCAN8	2328.00	2221.00	2603.00	2384.00	1597.00
ZMAT1	366.00	383.00	392.00	380.33	444.00
ZMAT2	838.00	777.00	840.00	818.33	980.00
ZMAT3	411.00	386.00	476.00	424.33	443.00
ZMAT5	320.00	299.00	348.00	322.33	349.00
ZMIZ1	1663.00	1611.00	1716.00	1663.33	1166.00
ZMIZ1-AS1	33.00	36.00	34.00	34.33	29.00
ZMIZ2	3146.00	3032.00	3210.00	3129.33	3011.00
ZMPSTE24	2190.00	1974.00	2173.00	2112.33	2144.00
ZMYM1	930.00	896.00	1081.00	969.00	1164.00
ZMYM2	1913.00	1921.00	2209.00	2014.33	2073.00
ZMYM3	1388.00	1293.00	1411.00	1364.00	1171.00
ZMYM4	2019.00	1787.00	2067.00	1957.67	1988.00
ZMYM5	397.00	371.00	440.00	402.67	350.00
ZMYM6	812.00	728.00	779.00	773.00	743.00
ZMYM6NB	786.00	804.00	809.00	799.67	961.00
ZMYND10	9.00	12.00	12.00	11.00	10.00
ZMYND11	2164.00	1945.00	2386.00	2165.00	2322.00
ZMYND12	3.00	11.00	15.00	9.67	7.00
ZMYND19	628.00	685.00	645.00	652.67	773.00
ZMYND8	4413.00	4286.00	4573.00	4424.00	4800.00
ZNF10	352.00	377.00	389.00	372.67	372.00
ZNF101	186.00	156.00	194.00	178.67	226.00

ZNF106	2315.00	2064.00	2536.00	2305.00	2149.00
ZNF107	630.00	554.00	657.00	613.67	707.00
ZNF112	298.00	275.00	285.00	286.00	270.00
ZNF117	43.00	41.00	58.00	47.33	63.00
ZNF12	1522.00	1501.00	1722.00	1581.67	1511.00
ZNF121	325.00	258.00	313.00	298.67	208.00
ZNF124	192.00	175.00	200.00	189.00	253.00
ZNF131	1295.00	1255.00	1433.00	1327.67	1497.00
ZNF132	31.00	41.00	25.00	32.33	20.00
ZNF133	891.00	929.00	986.00	935.33	923.00
ZNF134	274.00	211.00	269.00	251.33	249.00
ZNF136	74.00	86.00	103.00	87.67	123.00
ZNF137P	97.00	82.00	108.00	95.67	110.00
ZNF138	138.00	138.00	143.00	139.67	192.00
ZNF14	88.00	86.00	80.00	84.67	91.00
ZNF140	144.00	146.00	142.00	144.00	171.00
ZNF141	20.00	25.00	17.00	20.67	13.00
ZNF142	807.00	753.00	814.00	791.33	745.00
ZNF143	353.00	327.00	337.00	339.00	363.00
ZNF146	2778.00	2799.00	3077.00	2884.67	3327.00
ZNF148	994.00	849.00	1104.00	982.33	1181.00
ZNF155	160.00	175.00	206.00	180.33	188.00
ZNF157	6.00	5.00	2.00	4.33	2.00
ZNF16	252.00	291.00	273.00	272.00	305.00
ZNF160	310.00	333.00	378.00	340.33	383.00
ZNF165	15.00	18.00	15.00	16.00	19.00
ZNF169	167.00	173.00	182.00	174.00	117.00
ZNF17	97.00	80.00	103.00	93.33	54.00
ZNF174	292.00	292.00	306.00	296.67	333.00
ZNF175	359.00	440.00	428.00	409.00	317.00
ZNF18	354.00	460.00	415.00	409.67	550.00
ZNF180	195.00	162.00	208.00	188.33	209.00
ZNF181	257.00	224.00	242.00	241.00	176.00
ZNF182	378.00	343.00	384.00	368.33	334.00
ZNF184	186.00	158.00	204.00	182.67	219.00
ZNF185	4049.00	3935.00	4151.00	4045.00	3135.00
ZNF189	631.00	574.00	618.00	607.67	636.00
ZNF19	96.00	88.00	83.00	89.00	99.00
ZNF192P1	122.00	112.00	97.00	110.33	90.00
ZNF195	902.00	860.00	863.00	875.00	1050.00
ZNF197	460.00	503.00	508.00	490.33	488.00
ZNF2	132.00	110.00	127.00	123.00	124.00
ZNF200	297.00	257.00	309.00	287.67	347.00
ZNF202	1335.00	1296.00	1429.00	1353.33	1368.00
ZNF204P	11.00	15.00	13.00	13.00	15.00
ZNF205	378.00	382.00	443.00	401.00	450.00
ZNF205-AS1	8.00	5.00	9.00	7.33	5.00
ZNF207	3449.00	3263.00	3792.00	3501.33	3521.00

ZNF211	278.00	288.00	291.00	285.67	274.00
ZNF212	690.00	726.00	752.00	722.67	823.00
ZNF213	393.00	343.00	322.00	352.67	338.00
ZNF213-AS1	231.00	222.00	221.00	224.67	210.00
ZNF214	34.00	31.00	36.00	33.67	38.00
ZNF215	407.00	381.00	360.00	382.67	405.00
ZNF217	1448.00	1295.00	1555.00	1432.67	1412.00
ZNF219	76.00	75.00	79.00	76.67	124.00
ZNF22	1318.00	1172.00	1349.00	1279.67	1227.00
ZNF221	32.00	23.00	24.00	26.33	14.00
ZNF222	69.00	78.00	82.00	76.33	116.00
ZNF223	37.00	48.00	43.00	42.67	48.00
ZNF224	474.00	502.00	513.00	496.33	462.00
ZNF225	125.00	131.00	147.00	134.33	136.00
ZNF226	422.00	444.00	439.00	435.00	467.00
ZNF227	252.00	240.00	298.00	263.33	259.00
ZNF229	6.00	3.00	5.00	4.67	0.00
ZNF23	895.00	880.00	974.00	916.33	1095.00
ZNF230	103.00	101.00	124.00	109.33	111.00
ZNF232	314.00	345.00	271.00	310.00	358.00
ZNF234	394.00	354.00	416.00	388.00	358.00
ZNF235	59.00	51.00	71.00	60.33	88.00
ZNF236	711.00	662.00	729.00	700.67	566.00
ZNF239	703.00	730.00	725.00	719.33	1070.00
ZNF24	1685.00	1533.00	1813.00	1677.00	1735.00
ZNF248	1260.00	1212.00	1336.00	1269.33	1209.00
ZNF25	355.00	355.00	456.00	388.67	560.00
ZNF250	285.00	232.00	234.00	250.33	217.00
ZNF252P	696.00	639.00	708.00	681.00	773.00
ZNF252P-AS1	26.00	30.00	22.00	26.00	27.00
ZNF253	19.00	26.00	33.00	26.00	28.00
ZNF254	96.00	94.00	132.00	107.33	105.00
ZNF256	23.00	17.00	15.00	18.33	16.00
ZNF26	745.00	754.00	768.00	755.67	957.00
ZNF260	276.00	267.00	300.00	281.00	315.00
ZNF263	1089.00	1091.00	1143.00	1107.67	1191.00
ZNF264	335.00	338.00	359.00	344.00	344.00
ZNF266	1071.00	1217.00	1255.00	1181.00	1226.00
ZNF267	401.00	420.00	518.00	446.33	458.00
ZNF268	331.00	297.00	342.00	323.33	355.00
ZNF271P	491.00	516.00	571.00	526.00	705.00
ZNF273	63.00	50.00	64.00	59.00	62.00
ZNF274	559.00	585.00	566.00	570.00	728.00
ZNF275	571.00	549.00	600.00	573.33	508.00
ZNF276	2467.00	2630.00	2911.00	2669.33	2162.00
ZNF277	379.00	293.00	388.00	353.33	475.00
ZNF28	137.00	114.00	165.00	138.67	182.00
ZNF280C	225.00	196.00	253.00	224.67	240.00

ZNF280D	792.00	740.00	892.00	808.00	971.00
ZNF281	1521.00	1474.00	1613.00	1536.00	1511.00
ZNF282	1161.00	1141.00	1294.00	1198.67	1216.00
ZNF283	125.00	119.00	146.00	130.00	115.00
ZNF284	108.00	117.00	125.00	116.67	119.00
ZNF285	124.00	94.00	127.00	115.00	123.00
ZNF286A	545.00	524.00	520.00	529.67	548.00
ZNF286B	153.00	164.00	178.00	165.00	188.00
ZNF287	102.00	100.00	128.00	110.00	135.00
ZNF292	1007.00	909.00	1067.00	994.33	1044.00
ZNF296	17.00	16.00	20.00	17.67	10.00
ZNF3	1966.00	2050.00	2068.00	2028.00	2223.00
ZNF30	162.00	146.00	175.00	161.00	134.00
ZNF300	1002.00	996.00	1022.00	1006.67	1313.00
ZNF302	291.00	323.00	338.00	317.33	295.00
ZNF304	241.00	275.00	255.00	257.00	258.00
ZNF311	13.00	17.00	17.00	15.67	17.00
ZNF316	1525.00	1527.00	1624.00	1558.67	1490.00
ZNF317	1026.00	1000.00	1091.00	1039.00	1020.00
ZNF318	1132.00	1004.00	1196.00	1110.67	988.00
ZNF319	472.00	496.00	474.00	480.67	497.00
ZNF32	326.00	398.00	377.00	367.00	489.00
ZNF320	19.00	20.00	30.00	23.00	15.00
ZNF322	242.00	223.00	285.00	250.00	264.00
ZNF324	216.00	246.00	244.00	235.33	179.00
ZNF324B	104.00	93.00	95.00	97.33	93.00
ZNF326	816.00	830.00	801.00	815.67	1052.00
ZNF329	190.00	177.00	187.00	184.67	225.00
ZNF32-AS2	4.00	4.00	5.00	4.33	3.00
ZNF330	656.00	699.00	673.00	676.00	937.00
ZNF331	246.00	273.00	288.00	269.00	300.00
ZNF333	598.00	603.00	648.00	616.33	630.00
ZNF335	1220.00	1254.00	1371.00	1281.67	1148.00
ZNF337	2624.00	2698.00	2966.00	2762.67	2267.00
ZNF337-AS1	317.00	306.00	323.00	315.33	338.00
ZNF33A	1113.00	1043.00	1260.00	1138.67	971.00
ZNF33B	757.00	768.00	824.00	783.00	771.00
ZNF34	217.00	166.00	185.00	189.33	194.00
ZNF341	178.00	148.00	158.00	161.33	196.00
ZNF341-AS1	4.00	3.00	4.00	3.67	5.00
ZNF343	692.00	681.00	741.00	704.67	688.00
ZNF345	3.00	0.00	0.00	1.00	0.00
ZNF346	472.00	414.00	387.00	424.33	423.00
ZNF35	69.00	71.00	66.00	68.67	80.00
ZNF350	71.00	66.00	93.00	76.67	80.00
ZNF354A	589.00	594.00	657.00	613.33	678.00
ZNF354B	438.00	424.00	513.00	458.33	385.00
ZNF354C	4.00	1.00	6.00	3.67	2.00

ZNF358	705.00	606.00	791.00	700.67	800.00
ZNF362	169.00	158.00	182.00	169.67	203.00
ZNF365	99.00	95.00	122.00	105.33	115.00
ZNF367	102.00	119.00	112.00	111.00	167.00
ZNF37A	1637.00	1445.00	1713.00	1598.33	1360.00
ZNF37BP	1516.00	1647.00	1689.00	1617.33	1629.00
ZNF383	51.00	34.00	32.00	39.00	41.00
ZNF384	1855.00	1805.00	1956.00	1872.00	1883.00
ZNF385A	67.00	57.00	61.00	61.67	67.00
ZNF385D	190.00	170.00	226.00	195.33	128.00
ZNF391	74.00	61.00	51.00	62.00	52.00
ZNF394	325.00	325.00	312.00	320.67	415.00
ZNF395	813.00	756.00	803.00	790.67	727.00
ZNF396	7.00	15.00	16.00	12.67	12.00
ZNF397	199.00	189.00	211.00	199.67	270.00
ZNF398	392.00	359.00	451.00	400.67	334.00
ZNF404	76.00	62.00	82.00	73.33	68.00
ZNF407	453.00	417.00	500.00	456.67	420.00
ZNF408	328.00	322.00	364.00	338.00	370.00
ZNF41	381.00	381.00	440.00	400.67	326.00
ZNF410	858.00	800.00	858.00	838.67	1050.00
ZNF414	164.00	125.00	174.00	154.33	190.00
ZNF416	138.00	108.00	119.00	121.67	120.00
ZNF417	191.00	157.00	180.00	176.00	121.00
ZNF418	34.00	32.00	44.00	36.67	33.00
ZNF419	384.00	410.00	405.00	399.67	432.00
ZNF420	45.00	38.00	56.00	46.33	46.00
ZNF425	82.00	67.00	71.00	73.33	89.00
ZNF426	182.00	171.00	197.00	183.33	164.00
ZNF428	470.00	519.00	462.00	483.67	640.00
ZNF429	5.00	0.00	5.00	3.33	1.00
ZNF43	4.00	1.00	3.00	2.67	1.00
ZNF430	130.00	136.00	173.00	146.33	132.00
ZNF431	285.00	241.00	278.00	268.00	259.00
ZNF432	181.00	162.00	152.00	165.00	154.00
ZNF433	34.00	32.00	36.00	34.00	29.00
ZNF436	260.00	264.00	308.00	277.33	217.00
ZNF436-AS1	189.00	186.00	208.00	194.33	145.00
ZNF438	398.00	374.00	418.00	396.67	541.00
ZNF439	7.00	4.00	2.00	4.33	12.00
ZNF44	153.00	185.00	176.00	171.33	194.00
ZNF440	361.00	328.00	383.00	357.33	320.00
ZNF441	425.00	380.00	386.00	397.00	284.00
ZNF442	6.00	4.00	3.00	4.33	4.00
ZNF443	12.00	14.00	14.00	13.33	29.00
ZNF444	556.00	602.00	652.00	603.33	675.00
ZNF445	1064.00	961.00	1095.00	1040.00	843.00
ZNF446	383.00	366.00	351.00	366.67	376.00

ZNF449	248.00	267.00	275.00	263.33	272.00
ZNF45	281.00	301.00	281.00	287.67	319.00
ZNF451	1184.00	1170.00	1354.00	1236.00	1215.00
ZNF460	22.00	20.00	23.00	21.67	16.00
ZNF461	34.00	31.00	41.00	35.33	35.00
ZNF462	1003.00	951.00	1149.00	1034.33	775.00
ZNF468	54.00	40.00	45.00	46.33	41.00
ZNF469	27.00	18.00	31.00	25.33	13.00
ZNF470	102.00	85.00	119.00	102.00	113.00
ZNF473	500.00	509.00	588.00	532.33	491.00
ZNF474	7.00	16.00	11.00	11.33	6.00
ZNF48	296.00	286.00	333.00	305.00	306.00
ZNF480	337.00	334.00	346.00	339.00	334.00
ZNF484	100.00	86.00	116.00	100.67	106.00
ZNF485	453.00	452.00	488.00	464.33	521.00
ZNF486	43.00	38.00	42.00	41.00	52.00
ZNF487	25.00	15.00	25.00	21.67	35.00
ZNF488	34.00	30.00	40.00	34.67	34.00
ZNF490	105.00	97.00	91.00	97.67	120.00
ZNF491	17.00	18.00	14.00	16.33	24.00
ZNF493	105.00	113.00	103.00	107.00	86.00
ZNF496	1020.00	953.00	1036.00	1003.00	855.00
ZNF497	91.00	78.00	104.00	91.00	94.00
ZNF500	335.00	356.00	313.00	334.67	305.00
ZNF501	47.00	32.00	47.00	42.00	39.00
ZNF502	51.00	39.00	50.00	46.67	76.00
ZNF503	292.00	306.00	308.00	302.00	334.00
ZNF503-AS2	39.00	28.00	44.00	37.00	28.00
ZNF506	63.00	51.00	60.00	58.00	64.00
ZNF507	1166.00	1020.00	1304.00	1163.33	984.00
ZNF510	317.00	273.00	339.00	309.67	254.00
ZNF511	414.00	471.00	469.00	451.33	631.00
ZNF512	1849.00	1772.00	1956.00	1859.00	1987.00
ZNF512B	2466.00	2467.00	2563.00	2498.67	2229.00
ZNF513	487.00	505.00	516.00	502.67	503.00
ZNF514	1331.00	1334.00	1401.00	1355.33	1338.00
ZNF516	1325.00	1333.00	1458.00	1372.00	1122.00
ZNF517	69.00	77.00	73.00	73.00	84.00
ZNF518A	1314.00	1273.00	1493.00	1360.00	1202.00
ZNF518B	151.00	180.00	223.00	184.67	155.00
ZNF519	306.00	322.00	361.00	329.67	402.00
ZNF521	298.00	270.00	319.00	295.67	291.00
ZNF524	133.00	165.00	135.00	144.33	168.00
ZNF525	50.00	48.00	51.00	49.67	51.00
ZNF526	282.00	228.00	296.00	268.67	245.00
ZNF527	121.00	113.00	115.00	116.33	136.00
ZNF528	19.00	39.00	35.00	31.00	39.00
ZNF528-AS1	10.00	8.00	9.00	9.00	15.00

ZNF529	279.00	268.00	260.00	269.00	276.00
ZNF529-AS1	42.00	23.00	46.00	37.00	33.00
ZNF530	122.00	120.00	90.00	110.67	144.00
ZNF532	7.00	1.00	6.00	4.67	5.00
ZNF540	6.00	5.00	8.00	6.33	8.00
ZNF542P	12.00	9.00	7.00	9.33	13.00
ZNF543	76.00	75.00	78.00	76.33	66.00
ZNF544	579.00	546.00	535.00	553.33	645.00
ZNF546	70.00	59.00	70.00	66.33	58.00
ZNF547	63.00	80.00	74.00	72.33	83.00
ZNF548	455.00	452.00	494.00	467.00	448.00
ZNF549	215.00	227.00	241.00	227.67	232.00
ZNF550	633.00	664.00	740.00	679.00	622.00
ZNF551	147.00	158.00	154.00	153.00	159.00
ZNF552	101.00	94.00	94.00	96.33	82.00
ZNF554	85.00	96.00	92.00	91.00	80.00
ZNF555	122.00	156.00	168.00	148.67	159.00
ZNF557	138.00	114.00	142.00	131.33	122.00
ZNF559	407.00	423.00	414.00	414.67	528.00
ZNF561	610.00	616.00	679.00	635.00	627.00
ZNF561-AS1	39.00	45.00	36.00	40.00	40.00
ZNF562	565.00	534.00	641.00	580.00	545.00
ZNF563	6.00	8.00	6.00	6.67	8.00
ZNF564	162.00	152.00	196.00	170.00	178.00
ZNF565	71.00	87.00	97.00	85.00	87.00
ZNF566	153.00	154.00	145.00	150.67	175.00
ZNF567	101.00	104.00	115.00	106.67	110.00
ZNF569	79.00	70.00	78.00	75.67	76.00
ZNF57	107.00	116.00	111.00	111.33	108.00
ZNF570	14.00	17.00	24.00	18.33	10.00
ZNF571	29.00	26.00	32.00	29.00	48.00
ZNF572	80.00	97.00	98.00	91.67	80.00
ZNF573	14.00	20.00	23.00	19.00	9.00
ZNF574	399.00	381.00	437.00	405.67	424.00
ZNF575	16.00	14.00	20.00	16.67	25.00
ZNF576	103.00	125.00	119.00	115.67	117.00
ZNF577	323.00	301.00	362.00	328.67	310.00
ZNF579	328.00	288.00	340.00	318.67	368.00
ZNF580	589.00	546.00	613.00	582.67	633.00
ZNF581	445.00	508.00	564.00	505.67	556.00
ZNF583	80.00	42.00	37.00	53.00	59.00
ZNF584	202.00	225.00	233.00	220.00	275.00
ZNF585A	106.00	87.00	94.00	95.67	104.00
ZNF585B	38.00	36.00	29.00	34.33	42.00
ZNF586	112.00	125.00	137.00	124.67	122.00
ZNF587	1045.00	1014.00	1156.00	1071.67	726.00
ZNF587B	282.00	215.00	274.00	257.00	352.00
ZNF589	927.00	942.00	977.00	948.67	1003.00

ZNF592	1022.00	941.00	1116.00	1026.33	863.00
ZNF593	435.00	456.00	493.00	461.33	587.00
ZNF594	431.00	427.00	428.00	428.67	363.00
ZNF595	138.00	162.00	133.00	144.33	171.00
ZNF596	83.00	78.00	101.00	87.33	93.00
ZNF597	90.00	94.00	93.00	92.33	102.00
ZNF598	3457.00	3491.00	3641.00	3529.67	2828.00
ZNF599	65.00	61.00	78.00	68.00	65.00
ZNF600	75.00	76.00	80.00	77.00	101.00
ZNF605	434.00	410.00	493.00	445.67	418.00
ZNF606	17.00	19.00	21.00	19.00	16.00
ZNF607	78.00	65.00	85.00	76.00	67.00
ZNF608	94.00	59.00	98.00	83.67	39.00
ZNF609	2072.00	1845.00	1990.00	1969.00	1421.00
ZNF610	8.00	7.00	9.00	8.00	9.00
ZNF611	152.00	135.00	141.00	142.67	91.00
ZNF613	60.00	60.00	54.00	58.00	67.00
ZNF614	241.00	208.00	234.00	227.67	256.00
ZNF615	137.00	138.00	134.00	136.33	143.00
ZNF616	154.00	199.00	205.00	186.00	188.00
ZNF618	432.00	440.00	466.00	446.00	353.00
ZNF619	197.00	184.00	213.00	198.00	192.00
ZNF620	154.00	127.00	124.00	135.00	118.00
ZNF621	2379.00	2173.00	2502.00	2351.33	2073.00
ZNF622	1002.00	1051.00	1052.00	1035.00	1137.00
ZNF623	42.00	38.00	45.00	41.67	31.00
ZNF624	129.00	117.00	128.00	124.67	133.00
ZNF625	25.00	22.00	15.00	20.67	16.00
ZNF625-ZNF20	11.00	8.00	7.00	8.67	8.00
ZNF626	16.00	15.00	25.00	18.67	21.00
ZNF627	352.00	333.00	385.00	356.67	365.00
ZNF628	149.00	115.00	106.00	123.33	141.00
ZNF629	821.00	833.00	806.00	820.00	877.00
ZNF638	4019.00	3917.00	4444.00	4126.67	4068.00
ZNF639	847.00	867.00	956.00	890.00	1123.00
ZNF641	112.00	111.00	116.00	113.00	116.00
ZNF644	1253.00	1073.00	1302.00	1209.33	1328.00
ZNF646	1047.00	1119.00	1088.00	1084.67	879.00
ZNF649	115.00	98.00	151.00	121.33	124.00
ZNF652	631.00	659.00	695.00	661.67	576.00
ZNF653	84.00	77.00	85.00	82.00	83.00
ZNF654	222.00	193.00	274.00	229.67	202.00
ZNF655	3259.00	3223.00	3498.00	3326.67	2978.00
ZNF658	60.00	57.00	81.00	66.00	67.00
ZNF658B	29.00	20.00	21.00	23.33	23.00
ZNF660	14.00	16.00	20.00	16.67	17.00
ZNF664	3028.00	2926.00	3408.00	3120.67	3284.00
ZNF668	330.00	352.00	383.00	355.00	323.00

ZNF669	102.00	93.00	98.00	97.67	88.00
ZNF670	134.00	140.00	156.00	143.33	156.00
ZNF671	94.00	102.00	93.00	96.33	119.00
ZNF672	815.00	832.00	839.00	828.67	729.00
ZNF674	148.00	150.00	169.00	155.67	230.00
ZNF674-AS1	377.00	392.00	367.00	378.67	398.00
ZNF675	74.00	64.00	89.00	75.67	81.00
ZNF678	297.00	295.00	341.00	311.00	332.00
ZNF680	100.00	93.00	126.00	106.33	127.00
ZNF681	31.00	31.00	26.00	29.33	20.00
ZNF682	5.00	7.00	10.00	7.33	10.00
ZNF684	71.00	77.00	88.00	78.67	105.00
ZNF687	1061.00	865.00	901.00	942.33	927.00
ZNF688	239.00	245.00	245.00	243.00	251.00
ZNF689	289.00	231.00	249.00	256.33	268.00
ZNF691	441.00	453.00	497.00	463.67	491.00
ZNF692	1882.00	1997.00	2028.00	1969.00	2145.00
ZNF695	54.00	45.00	49.00	49.33	63.00
ZNF696	660.00	626.00	691.00	659.00	649.00
ZNF697	1493.00	1284.00	1531.00	1436.00	1370.00
ZNF699	54.00	49.00	59.00	54.00	35.00
ZNF7	1143.00	1122.00	1190.00	1151.67	1172.00
ZNF70	140.00	146.00	134.00	140.00	100.00
ZNF700	675.00	740.00	765.00	726.67	874.00
ZNF701	220.00	204.00	181.00	201.67	139.00
ZNF703	952.00	904.00	1044.00	966.67	1096.00
ZNF704	175.00	197.00	207.00	193.00	160.00
ZNF705E	12.00	9.00	8.00	9.67	8.00
ZNF706	1114.00	1093.00	1218.00	1141.67	1456.00
ZNF708	116.00	98.00	125.00	113.00	134.00
ZNF709	11.00	8.00	10.00	9.67	12.00
ZNF71	112.00	110.00	115.00	112.33	132.00
ZNF710	914.00	938.00	1031.00	961.00	957.00
ZNF711	32.00	31.00	30.00	31.00	33.00
ZNF713	14.00	23.00	25.00	20.67	24.00
ZNF714	222.00	190.00	185.00	199.00	210.00
ZNF717	269.00	235.00	263.00	255.67	245.00
ZNF718	52.00	30.00	37.00	39.67	36.00
ZNF720	125.00	141.00	131.00	132.33	189.00
ZNF721	570.00	546.00	636.00	584.00	526.00
ZNF724P	3.00	0.00	2.00	1.67	2.00
ZNF726	4.00	1.00	5.00	3.33	6.00
ZNF736	174.00	142.00	180.00	165.33	178.00
ZNF737	5.00	3.00	3.00	3.67	16.00
ZNF738	85.00	109.00	94.00	96.00	95.00
ZNF74	467.00	495.00	463.00	475.00	541.00
ZNF740	1205.00	1175.00	1207.00	1195.67	1252.00
ZNF746	754.00	794.00	847.00	798.33	895.00

ZNF747	231.00	207.00	225.00	221.00	196.00
ZNF749	146.00	123.00	156.00	141.67	115.00
ZNF75A	203.00	197.00	186.00	195.33	185.00
ZNF75D	174.00	179.00	225.00	192.67	205.00
ZNF76	961.00	1007.00	1069.00	1012.33	1040.00
ZNF761	512.00	483.00	543.00	512.67	496.00
ZNF763	14.00	13.00	14.00	13.67	6.00
ZNF764	202.00	230.00	237.00	223.00	241.00
ZNF765	95.00	74.00	98.00	89.00	68.00
ZNF766	244.00	244.00	260.00	249.33	265.00
ZNF767P	1091.00	1050.00	1097.00	1079.33	998.00
ZNF768	1688.00	1745.00	1847.00	1760.00	2412.00
ZNF77	130.00	132.00	141.00	134.33	120.00
ZNF770	1785.00	1693.00	2118.00	1865.33	1821.00
ZNF771	111.00	126.00	139.00	125.33	146.00
ZNF772	48.00	39.00	43.00	43.33	36.00
ZNF773	149.00	147.00	147.00	147.67	156.00
ZNF774	24.00	33.00	47.00	34.67	41.00
ZNF775	193.00	168.00	187.00	182.67	211.00
ZNF776	359.00	362.00	379.00	366.67	332.00
ZNF777	659.00	615.00	661.00	645.00	623.00
ZNF778	305.00	301.00	306.00	304.00	262.00
ZNF780A	321.00	296.00	347.00	321.33	284.00
ZNF780B	581.00	500.00	559.00	546.67	558.00
ZNF781	5.00	3.00	10.00	6.00	7.00
ZNF782	156.00	161.00	150.00	155.67	166.00
ZNF783	2720.00	2742.00	3105.00	2855.67	2575.00
ZNF784	48.00	53.00	41.00	47.33	46.00
ZNF785	414.00	438.00	432.00	428.00	317.00
ZNF786	405.00	444.00	439.00	429.33	389.00
ZNF787	698.00	725.00	706.00	709.67	871.00
ZNF788	5.00	13.00	8.00	8.67	15.00
ZNF789	406.00	395.00	396.00	399.00	351.00
ZNF79	141.00	143.00	120.00	134.67	171.00
ZNF791	407.00	464.00	422.00	431.00	331.00
ZNF792	157.00	160.00	160.00	159.00	87.00
ZNF799	50.00	32.00	27.00	36.33	61.00
ZNF8	156.00	161.00	168.00	161.67	165.00
ZNF800	535.00	458.00	597.00	530.00	546.00
ZNF804A	487.00	427.00	577.00	497.00	506.00
ZNF805	200.00	203.00	227.00	210.00	190.00
ZNF808	107.00	100.00	90.00	99.00	68.00
ZNF81	227.00	232.00	193.00	217.33	187.00
ZNF812	6.00	5.00	1.00	4.00	2.00
ZNF814	122.00	124.00	130.00	125.33	103.00
ZNF815P	31.00	28.00	20.00	26.33	36.00
ZNF816	14.00	12.00	12.00	12.67	17.00
ZNF821	76.00	73.00	61.00	70.00	73.00

ZNF823	100.00	96.00	111.00	102.33	144.00
ZNF827	925.00	814.00	995.00	911.33	840.00
ZNF83	1829.00	1855.00	2046.00	1910.00	1662.00
ZNF830	393.00	405.00	443.00	413.67	497.00
ZNF833P	16.00	11.00	21.00	16.00	27.00
ZNF836	34.00	37.00	34.00	35.00	38.00
ZNF837	39.00	32.00	35.00	35.33	52.00
ZNF839	979.00	1027.00	1011.00	1005.67	1193.00
ZNF84	1222.00	1250.00	1336.00	1269.33	1380.00
ZNF841	447.00	447.00	534.00	476.00	418.00
ZNF844	4.00	7.00	1.00	4.00	1.00
ZNF845	179.00	134.00	172.00	161.67	189.00
ZNF846	65.00	70.00	58.00	64.33	48.00
ZNF850	6.00	6.00	13.00	8.33	11.00
ZNF852	119.00	84.00	96.00	99.67	59.00
ZNF860	7.00	10.00	7.00	8.00	7.00
ZNF862	1240.00	1313.00	1401.00	1318.00	1066.00
ZNF865	439.00	424.00	509.00	457.33	431.00
ZNF879	10.00	12.00	3.00	8.33	7.00
ZNF880	70.00	62.00	56.00	62.67	73.00
ZNF883	187.00	174.00	193.00	184.67	206.00
ZNF891	70.00	80.00	75.00	75.00	71.00
ZNF90	34.00	27.00	32.00	31.00	44.00
ZNF91	123.00	118.00	131.00	124.00	100.00
ZNF92	295.00	231.00	278.00	268.00	332.00
ZNF93	35.00	36.00	42.00	37.67	38.00
ZNFX1	2350.00	2206.00	2456.00	2337.33	2024.00
ZNHIT1	612.00	605.00	603.00	606.67	782.00
ZNHIT2	385.00	382.00	430.00	399.00	540.00
ZNHIT6	1943.00	1800.00	2039.00	1927.33	2399.00
ZNRD1	57.00	49.00	52.00	52.67	72.00
ZNRD1-AS1	7.00	15.00	4.00	8.67	5.00
ZNRF1	296.00	292.00	323.00	303.67	282.00
ZNRF2	76.00	81.00	105.00	87.33	81.00
ZNRF2P1	17.00	15.00	19.00	17.00	16.00
ZNRF2P2	9.00	4.00	7.00	6.67	6.00
ZNRF3	554.00	568.00	598.00	573.33	524.00
ZP1	49.00	39.00	40.00	42.67	12.00
ZP3	51.00	39.00	44.00	44.67	81.00
ZPLD1	16.00	14.00	19.00	16.33	6.00
ZPR1	1118.00	1117.00	1241.00	1158.67	1284.00
ZRANB1	1336.00	1258.00	1385.00	1326.33	1319.00
ZRANB2	3926.00	3781.00	4343.00	4016.67	3935.00
ZRANB2-AS2	4.00	4.00	3.00	3.67	3.00
ZRANB3	311.00	328.00	307.00	315.33	365.00
ZRSR2	437.00	508.00	542.00	495.67	539.00
ZSCAN12	522.00	433.00	484.00	479.67	489.00
ZSCAN12P1	75.00	51.00	79.00	68.33	76.00

ZSCAN16	84.00	104.00	111.00	99.67	115.00
ZSCAN16-AS1	103.00	99.00	106.00	102.67	103.00
ZSCAN2	170.00	185.00	208.00	187.67	189.00
ZSCAN20	248.00	261.00	285.00	264.67	246.00
ZSCAN21	216.00	246.00	269.00	243.67	224.00
ZSCAN22	113.00	130.00	146.00	129.67	138.00
ZSCAN23	78.00	66.00	70.00	71.33	46.00
ZSCAN25	404.00	419.00	422.00	415.00	375.00
ZSCAN26	489.00	470.00	582.00	513.67	606.00
ZSCAN29	1252.00	1206.00	1353.00	1270.33	1062.00
ZSCAN30	1427.00	1391.00	1586.00	1468.00	1263.00
ZSCAN31	28.00	27.00	30.00	28.33	31.00
ZSCAN32	556.00	551.00	528.00	545.00	585.00
ZSCAN5A	16.00	15.00	22.00	17.67	16.00
ZSCAN9	559.00	547.00	539.00	548.33	512.00
ZSWIM1	189.00	195.00	203.00	195.67	157.00
ZSWIM3	156.00	150.00	162.00	156.00	174.00
ZSWIM4	419.00	426.00	486.00	443.67	574.00
ZSWIM5	211.00	173.00	240.00	208.00	168.00
ZSWIM6	2043.00	1877.00	2254.00	2058.00	1480.00
ZSWIM7	165.00	166.00	174.00	168.33	310.00
ZSWIM8	2501.00	2538.00	2524.00	2521.00	2024.00
ZSWIM8-AS1	249.00	255.00	269.00	257.67	220.00
ZUFSP	387.00	345.00	399.00	377.00	430.00
ZW10	726.00	582.00	688.00	665.33	773.00
ZWILCH	948.00	858.00	958.00	921.33	1148.00
ZWINT	992.00	1085.00	1003.00	1026.67	1579.00
ZXDA	81.00	74.00	103.00	86.00	76.00
ZXDB	288.00	275.00	361.00	308.00	291.00
ZXDC	1064.00	915.00	1116.00	1031.67	887.00
ZYG11A	3.00	0.00	0.00	1.00	0.00
ZYG11B	642.00	629.00	796.00	689.00	690.00
ZYX	5671.00	5478.00	5814.00	5654.33	5629.00
ZZEF1	2047.00	2001.00	2087.00	2045.00	1660.00
ZZZ3	2467.00	2251.00	2781.00	2499.67	2799.00

ES2 siARID1A			Fold change (siARID1A/scramble, tagwise dispersions)	P-value (tagwise dispersions)
Expression value 2	Expression value 3	Average (Expression value)		
79.00	64.00	70.67	-1.20	0.09
131.00	139.00	134.67	-1.19	0.03
1.00	1.00	1.33	-3.79	0.01
13.00	13.00	14.00	1.22	0.43
1596.00	1794.00	1752.00	1.07	0.10
430.00	455.00	455.33	-1.29	0.00
64.00	89.00	81.67	-1.06	0.59
0.00	5.00	2.33	-1.57	0.50
365.00	433.00	421.00	1.14	0.04
149.00	180.00	157.33	1.41	0.00
1440.00	1522.00	1528.00	1.30	0.00
1479.00	1584.00	1527.33	-1.17	0.00
43.00	63.00	61.67	1.14	0.33
2984.00	3192.00	3222.33	1.03	0.38
4.00	2.00	3.67	-1.55	0.35
822.00	800.00	833.33	-1.04	0.42
32.00	21.00	24.33	1.57	0.03
9724.00	10225.00	10069.33	-1.07	0.03
1243.00	1389.00	1330.00	-1.05	0.19
18.00	8.00	15.33	1.29	0.31
474.00	488.00	455.33	1.04	0.61
837.00	951.00	892.67	1.19	0.00
1694.00	2003.00	1872.00	-1.10	0.02
4.00	5.00	3.33	-1.11	1.00
4.00	3.00	3.00	-2.42	0.03
552.00	544.00	537.67	-1.67	0.00
13.00	8.00	14.33	1.62	0.07
144.00	152.00	158.00	1.16	0.09
3.00	1.00	4.00	-1.51	0.37
1738.00	1626.00	1755.33	-1.10	0.04
8.00	10.00	11.67	-1.33	0.25
3.00	3.00	3.33	-1.31	0.68
265.00	299.00	276.00	1.04	0.55
236.00	205.00	232.67	-1.41	0.00
1.00	0.00	1.33	-1.94	0.40
140.00	201.00	175.33	1.02	0.83
469.00	552.00	505.00	1.15	0.01
123.00	150.00	133.67	-1.37	0.00
607.00	677.00	643.00	-1.08	0.12
735.00	742.00	744.67	1.02	0.68
1575.00	1595.00	1674.00	-1.06	0.15
238.00	214.00	232.00	-1.49	0.00
332.00	291.00	320.67	-1.14	0.03

970.00	929.00	968.00	-1.18	0.00
1.00	6.00	3.67	-1.90	0.12
3.00	2.00	2.33	1.14	1.00
790.00	891.00	847.33	1.01	0.81
209.00	173.00	182.67	-4.42	0.00
1831.00	1827.00	1840.33	-1.01	0.81
2527.00	2579.00	2594.67	-1.21	0.00
482.00	474.00	468.33	1.12	0.06
194.00	170.00	185.00	-1.07	0.38
981.00	1166.00	1081.33	1.09	0.06
974.00	1080.00	1082.67	-1.24	0.00
3112.00	3512.00	3355.00	1.11	0.00
4.00	2.00	2.00	-1.49	0.61
3578.00	3704.00	3734.00	1.02	0.62
1133.00	1116.00	1169.67	-1.02	0.75
231.00	266.00	244.33	-1.07	0.44
781.00	924.00	863.67	1.08	0.13
315.00	320.00	347.67	1.28	0.00
1153.00	1138.00	1147.33	-1.07	0.14
413.00	455.00	435.67	1.20	0.00
191.00	184.00	189.33	1.01	0.89
3081.00	3099.00	3242.33	1.05	0.19
222.00	256.00	243.00	-1.01	0.91
534.00	511.00	528.67	1.13	0.07
187.00	201.00	196.33	1.03	0.70
237.00	289.00	274.33	1.06	0.36
3623.00	3195.00	3277.33	-1.32	0.00
275.00	261.00	269.00	1.26	0.00
645.00	586.00	635.33	-1.46	0.00
876.00	974.00	955.33	-1.19	0.00
287.00	273.00	297.67	1.18	0.02
174.00	137.00	147.67	1.04	0.68
1404.00	1605.00	1519.00	1.10	0.03
2339.00	2663.00	2514.00	1.05	0.25
75.00	48.00	64.33	-1.20	0.14
274.00	306.00	291.00	-1.46	0.00
5802.00	6082.00	6036.33	1.24	0.00
6599.00	6653.00	6549.00	1.03	0.42
3.00	7.00	10.00	-1.53	0.16
3.00	6.00	6.67	1.50	0.31
1366.00	1309.00	1357.00	-1.71	0.00
941.00	1101.00	1067.67	1.12	0.01
855.00	874.00	891.33	1.19	0.00
901.00	1018.00	1018.33	-1.09	0.08
117.00	124.00	114.33	-1.48	0.00
761.00	844.00	836.00	1.04	0.37
220.00	270.00	268.00	1.53	0.00
36.00	35.00	29.00	1.34	0.14

35.00	36.00	33.67	1.19	0.30
1149.00	1169.00	1188.00	-1.11	0.01
224.00	203.00	207.00	-1.10	0.31
384.00	433.00	423.00	-1.24	0.00
755.00	891.00	850.33	-1.01	0.77
15.00	12.00	12.67	1.38	0.23
953.00	1111.00	1061.00	1.26	0.00
92.00	100.00	105.33	-1.08	0.42
550.00	606.00	581.00	-1.07	0.18
6258.00	6329.00	6629.33	-1.06	0.12
11.00	2.00	8.00	1.18	0.67
1648.00	1995.00	1754.67	1.27	0.00
694.00	614.00	687.67	-1.29	0.00
1321.00	1443.00	1469.00	1.24	0.00
297.00	384.00	359.67	1.22	0.00
911.00	1145.00	999.33	1.06	0.29
145.00	176.00	182.00	-1.20	0.02
796.00	850.00	851.67	1.05	0.26
737.00	741.00	773.67	-1.03	0.57
45.00	48.00	52.00	1.20	0.17
341.00	364.00	379.33	1.00	0.97
535.00	588.00	607.67	1.05	0.41
43.00	36.00	39.00	1.99	0.00
764.00	828.00	805.33	-1.03	0.58
5474.00	5707.00	5777.33	-1.17	0.00
6575.00	6436.00	6517.67	1.00	0.97
2378.00	2449.00	2409.67	-1.09	0.04
2450.00	2398.00	2463.67	1.10	0.03
13.00	12.00	14.33	1.76	0.03
596.00	606.00	660.67	1.32	0.00
136.00	164.00	153.67	1.10	0.23
1328.00	1357.00	1443.33	1.32	0.00
409.00	381.00	412.67	1.01	0.87
583.00	637.00	626.33	1.07	0.17
541.00	549.00	546.67	-1.03	0.61
75.00	83.00	80.00	2.51	0.00
277.00	290.00	285.67	-1.05	0.42
1659.00	1859.00	1868.33	1.15	0.01
623.00	639.00	663.00	-1.15	0.01
202.00	165.00	208.00	-1.34	0.00
355.00	362.00	375.00	-1.15	0.01
15.00	32.00	21.67	-1.28	0.20
175.00	194.00	219.67	1.04	0.72
16.00	13.00	14.33	-1.18	0.55
307.00	296.00	302.33	-1.33	0.00
558.00	590.00	620.67	-1.10	0.07
471.00	496.00	481.67	-1.07	0.23
3841.00	3955.00	3829.00	1.07	0.12

4158.00	4652.00	4283.00	1.07	0.21
425.00	458.00	447.00	-1.50	0.00
8.00	8.00	8.00	1.08	0.89
623.00	699.00	676.33	-1.51	0.00
66.00	53.00	61.33	1.16	0.22
9.00	11.00	8.67	-1.20	0.61
50644.00	52800.00	54528.00	1.22	0.00
40345.00	39528.00	42073.33	1.08	0.04
19.00	30.00	28.33	1.20	0.37
43.00	27.00	38.00	-1.68	0.00
24.00	14.00	18.67	-1.26	0.27
28.00	36.00	30.67	1.11	0.62
1720.00	1931.00	1947.00	1.16	0.00
6497.00	6517.00	6559.33	-1.14	0.00
1.00	2.00	1.33	-1.48	0.76
16902.00	16469.00	16539.67	1.01	0.75
1038.00	1180.00	1161.67	1.12	0.01
2570.00	2667.00	2654.33	1.01	0.84
1058.00	1157.00	1199.33	-1.07	0.18
6536.00	7284.00	6898.33	1.25	0.00
5976.00	6527.00	6313.00	1.12	0.00
407.00	440.00	452.33	1.12	0.05
7.00	6.00	6.67	1.04	1.00
445.00	407.00	427.00	-1.06	0.34
501.00	567.00	556.00	1.13	0.02
550.00	565.00	572.00	-1.04	0.42
94.00	81.00	87.67	1.02	0.85
1470.00	1413.00	1469.00	1.03	0.54
905.00	828.00	882.00	-1.07	0.21
16.00	21.00	18.00	-1.37	0.13
301.00	352.00	332.67	1.03	0.64
504.00	567.00	552.00	1.02	0.66
15.00	20.00	19.00	-1.23	0.27
2.00	5.00	4.00	1.18	0.84
66.00	57.00	70.67	1.37	0.01
276.00	301.00	306.00	1.31	0.00
36.00	42.00	45.67	1.05	0.78
135.00	153.00	159.67	1.16	0.09
286.00	361.00	338.67	-1.23	0.00
2858.00	3030.00	2955.00	1.03	0.41
73.00	74.00	71.00	1.10	0.42
3202.00	3143.00	3131.67	-1.56	0.00
3484.00	3366.00	3468.00	-1.61	0.00
1426.00	1464.00	1433.67	-1.08	0.05
655.00	618.00	645.00	-1.41	0.00
268.00	326.00	317.33	1.02	0.79
2.00	9.00	3.67	-1.98	0.14
34.00	26.00	33.00	-1.24	0.19

17.00	14.00	15.67	-1.01	1.00
422.00	466.00	437.00	1.10	0.13
951.00	912.00	927.00	1.07	0.14
45.00	63.00	60.33	-1.72	0.00
2.00	5.00	4.33	-2.08	0.07
23.00	30.00	37.33	-1.72	0.00
4955.00	4965.00	4876.33	-1.17	0.00
13601.00	14414.00	14083.00	-1.39	0.00
865.00	793.00	795.67	-1.38	0.00
204.00	194.00	222.33	-1.19	0.02
10.00	8.00	7.67	-2.53	0.00
368.00	424.00	376.67	-1.03	0.74
59.00	55.00	60.33	-1.57	0.00
17.00	29.00	26.00	1.37	0.10
2399.00	2469.00	2539.67	-1.22	0.00
9.00	0.00	3.33	1.01	1.00
189.00	206.00	193.33	-1.18	0.02
150.00	161.00	150.67	-1.28	0.00
236.00	203.00	208.00	1.28	0.01
2512.00	2710.00	2716.67	1.00	0.94
41.00	48.00	47.33	-1.09	0.56
5.00	7.00	6.33	-1.69	0.10
8419.00	8139.00	8416.67	-1.16	0.00
22.00	25.00	23.67	-1.43	0.03
80.00	56.00	74.67	-1.85	0.00
7.00	5.00	7.33	1.14	0.76
190.00	204.00	205.67	-1.21	0.00
5.00	4.00	6.00	-1.84	0.06
6.00	4.00	6.33	-1.54	0.21
5486.00	5279.00	5345.33	-1.06	0.15
1564.00	1593.00	1601.33	-1.22	0.00
538.00	530.00	494.67	-1.18	0.03
964.00	1132.00	1088.67	1.06	0.16
70.00	61.00	64.33	-1.13	0.28
233.00	251.00	258.67	1.07	0.40
785.00	766.00	781.33	-1.12	0.01
856.00	928.00	922.00	1.08	0.07
310.00	308.00	305.00	-1.02	0.85
326.00	319.00	330.67	-1.03	0.67
156.00	145.00	148.67	1.01	0.95
477.00	506.00	493.00	-1.32	0.00
2118.00	2094.00	2087.67	1.02	0.70
1500.00	1502.00	1500.00	-1.24	0.00
1557.00	1484.00	1569.00	1.01	0.76
2.00	6.00	4.67	1.25	0.69
561.00	498.00	530.33	-1.11	0.09
7006.00	7102.00	7166.67	-1.10	0.00
526.00	625.00	566.67	-1.05	0.38

379.00	341.00	356.33	-1.44	0.00
885.00	871.00	885.00	-1.10	0.04
152.00	174.00	167.33	-1.22	0.01
139.00	142.00	136.33	1.03	0.76
7.00	1.00	3.33	-1.21	0.84
1025.00	1073.00	1053.33	-1.26	0.00
8.00	13.00	10.00	-1.65	0.05
69.00	52.00	55.00	-2.60	0.00
24.00	31.00	21.67	-1.68	0.00
49.00	34.00	43.67	-1.91	0.00
8.00	12.00	9.33	-2.76	0.00
75.00	82.00	77.33	-1.35	0.00
248.00	236.00	239.33	1.22	0.00
1323.00	1450.00	1379.33	-1.02	0.66
0.00	5.00	2.33	1.66	0.58
3444.00	3631.00	3513.00	-1.20	0.00
89.00	97.00	95.00	-1.19	0.06
1261.00	1417.00	1387.00	1.07	0.11
61.00	67.00	67.33	-1.46	0.00
305.00	362.00	350.33	1.00	0.99
1733.00	1777.00	1831.67	1.11	0.01
2653.00	2619.00	2667.33	-1.03	0.42
1523.00	1644.00	1650.67	-1.10	0.03
12.00	6.00	9.33	-1.12	0.80
353.00	298.00	347.33	-1.13	0.07
164.00	160.00	164.00	-1.32	0.00
3930.00	4370.00	4239.00	-1.07	0.06
776.00	816.00	820.67	1.04	0.35
28.00	40.00	34.33	-1.10	0.57
1234.00	1415.00	1386.67	1.08	0.09
10.00	6.00	7.67	-1.79	0.04
1685.00	1794.00	1841.33	-1.65	0.00
1796.00	1772.00	1862.33	-1.06	0.16
25.00	27.00	28.00	-1.07	0.72
29.00	22.00	27.67	-1.00	1.00
532.00	661.00	604.33	1.06	0.28
175.00	176.00	195.67	-1.02	0.79
40.00	37.00	38.67	1.36	0.05
2573.00	2591.00	2638.33	1.17	0.00
2259.00	2113.00	2295.67	-1.01	0.78
71.00	86.00	80.00	1.43	0.00
3279.00	3635.00	3598.00	1.23	0.00
1953.00	2047.00	2090.33	1.18	0.00
1488.00	1615.00	1573.67	1.10	0.02
8.00	8.00	8.00	-1.80	0.03
3189.00	3573.00	3513.67	-1.10	0.01
4824.00	4991.00	4946.67	-1.48	0.00
903.00	1146.00	1031.33	1.20	0.00

1167.00	1163.00	1205.33	-1.04	0.39
3542.00	3572.00	3758.33	1.07	0.09
4158.00	3978.00	4048.33	-1.12	0.01
952.00	1049.00	986.33	-1.01	0.86
29.00	32.00	31.33	-2.00	0.00
546.00	518.00	515.33	-1.35	0.00
16.00	10.00	11.00	-1.44	0.16
1558.00	1369.00	1449.00	1.03	0.55
2907.00	2993.00	2891.67	-1.10	0.07
1402.00	1662.00	1565.00	-1.03	0.47
2672.00	2999.00	2941.33	1.16	0.00
569.00	590.00	612.33	1.06	0.28
2.00	0.00	1.00	-2.21	0.38
651.00	645.00	666.00	1.01	0.86
862.00	818.00	835.67	1.04	0.46
797.00	806.00	812.00	-1.05	0.31
43.00	53.00	51.00	1.19	0.21
0.00	5.00	2.33	-1.43	0.66
12.00	15.00	15.67	-1.08	0.77
298.00	291.00	297.33	-1.18	0.01
2923.00	3040.00	3032.33	1.00	0.96
200.00	224.00	218.33	-1.17	0.03
158.00	126.00	153.33	-1.24	0.02
787.00	881.00	862.00	-1.06	0.22
94.00	111.00	103.67	1.05	0.56
402.00	496.00	453.33	-1.11	0.10
60.00	81.00	66.00	1.04	0.77
67.00	101.00	89.00	1.11	0.37
768.00	690.00	750.33	-1.06	0.32
1517.00	1536.00	1541.33	1.05	0.19
179.00	213.00	194.33	-1.41	0.00
681.00	797.00	748.00	1.03	0.50
286.00	282.00	302.33	-1.12	0.06
719.00	766.00	730.67	1.03	0.57
139.00	101.00	119.00	1.11	0.33
601.00	587.00	585.33	-1.11	0.04
544.00	482.00	504.67	-1.11	0.09
246.00	221.00	239.67	1.06	0.47
711.00	767.00	735.00	-1.19	0.00
51.00	55.00	53.33	1.14	0.30
760.00	755.00	797.67	1.08	0.13
2636.00	2535.00	2597.67	-1.04	0.31
2284.00	2404.00	2383.00	-1.36	0.00
79.00	88.00	78.67	-1.70	0.00
2571.00	2753.00	2686.67	1.15	0.00
1214.00	1492.00	1361.67	-1.04	0.46
4638.00	4606.00	4668.00	-1.63	0.00
596.00	731.00	672.67	1.34	0.00

64.00	74.00	65.33	1.05	0.68
851.00	762.00	840.67	1.06	0.28
2256.00	2613.00	2445.67	1.02	0.66
226.00	193.00	202.00	-1.09	0.28
6432.00	6697.00	6834.00	1.25	0.00
2334.00	2361.00	2359.67	-1.07	0.06
741.00	710.00	738.33	-1.18	0.00
337.00	305.00	308.67	-1.47	0.00
1056.00	1159.00	1123.33	1.09	0.05
31868.00	32656.00	31548.33	-1.40	0.00
2173.00	1983.00	2093.67	1.14	0.00
280.00	268.00	277.00	-1.26	0.01
1241.00	1214.00	1288.67	-1.18	0.00
2887.00	3146.00	3155.00	1.15	0.00
2882.00	3210.00	3139.00	-1.12	0.00
361.00	417.00	389.00	1.10	0.13
4.00	0.00	3.00	-1.12	1.00
800.00	909.00	888.33	1.07	0.11
467.00	514.00	527.33	-1.08	0.13
1103.00	1206.00	1154.00	-1.09	0.05
52.00	58.00	56.33	-2.06	0.00
5.00	4.00	6.00	-1.96	0.04
40.00	64.00	55.67	-1.60	0.00
1471.00	1721.00	1670.67	1.10	0.02
1090.00	1238.00	1250.67	1.04	0.37
894.00	947.00	960.00	1.12	0.02
2482.00	2736.00	2634.67	-1.52	0.00
457.00	526.00	516.00	-1.03	0.58
4915.00	5432.00	5312.67	1.05	0.14
1093.00	1325.00	1256.67	1.07	0.14
427.00	346.00	400.67	1.05	0.50
878.00	1010.00	962.00	-1.05	0.32
1.00	0.00	0.33	-6.90	0.04
80.00	125.00	110.00	-1.19	0.08
1922.00	1934.00	1984.33	-1.11	0.00
791.00	818.00	845.00	1.01	0.80
1821.00	1930.00	1789.00	-1.03	0.56
15439.00	15613.00	15196.67	-1.30	0.00
2323.00	2199.00	2184.33	-1.08	0.14
89.00	44.00	67.67	-1.46	0.01
3.00	14.00	8.67	1.28	0.48
7.00	20.00	11.67	1.38	0.30
48.00	46.00	47.67	-1.32	0.02
111.00	121.00	119.00	1.61	0.00
854.00	1030.00	980.00	-1.11	0.03
1269.00	1304.00	1334.67	-1.12	0.00
1455.00	1505.00	1480.33	-1.17	0.00
435.00	533.00	514.33	1.00	0.97

1500.00	1643.00	1635.00	1.10	0.01
1077.00	1177.00	1194.67	1.04	0.34
364.00	361.00	368.67	-1.08	0.18
12.00	19.00	16.00	1.57	0.06
1504.00	1720.00	1757.00	1.12	0.02
1764.00	1826.00	1908.67	-1.09	0.03
18.00	23.00	23.67	-1.38	0.05
9.00	1.00	6.00	-1.12	0.88
86.00	94.00	94.67	1.14	0.16
2.00	2.00	2.00	-1.33	0.79
54.00	49.00	54.33	1.05	0.71
864.00	943.00	948.67	1.14	0.00
6.00	8.00	7.67	-1.79	0.04
9.00	18.00	14.67	-1.22	0.39
2726.00	2818.00	2893.67	-1.02	0.55
1286.00	1308.00	1343.67	1.08	0.06
2381.00	2493.00	2541.00	-1.08	0.03
378.00	403.00	406.33	1.09	0.10
445.00	494.00	485.00	-1.08	0.13
1031.00	1011.00	1063.33	1.06	0.20
5.00	6.00	4.67	1.06	1.00
6091.00	6912.00	6374.00	1.08	0.07
933.00	925.00	946.00	1.12	0.01
4798.00	4800.00	4939.33	1.05	0.17
20274.00	20876.00	21119.00	1.65	0.00
599.00	625.00	614.33	1.12	0.02
27.00	11.00	19.67	1.12	0.61
1186.00	1248.00	1184.33	-1.04	0.44
6.00	2.00	4.00	1.07	1.00
291.00	310.00	301.33	-1.19	0.01
97.00	87.00	90.67	-2.35	0.00
387.00	378.00	396.33	-1.23	0.00
259.00	183.00	223.33	1.24	0.03
315.00	312.00	317.00	-1.16	0.01
1240.00	1437.00	1375.00	1.18	0.00
11.00	7.00	10.33	-1.40	0.22
1499.00	1553.00	1536.33	1.17	0.00
12346.00	12821.00	13149.00	1.14	0.00
88.00	73.00	98.00	1.19	0.19
1233.00	1241.00	1266.67	-1.00	0.95
212.00	316.00	253.33	1.04	0.65
1224.00	1505.00	1313.67	1.06	0.37
168.00	151.00	143.67	-1.29	0.02
469.00	461.00	489.33	-1.04	0.42
2107.00	2336.00	2289.00	-1.18	0.00
190.00	197.00	221.00	1.41	0.00
11.00	23.00	13.67	1.12	0.68
1011.00	1067.00	1055.67	-1.01	0.85

1651.00	1826.00	1817.67	1.05	0.25
695.00	760.00	748.67	1.27	0.00
658.00	737.00	714.67	1.19	0.00
1167.00	1155.00	1185.67	-1.02	0.72
681.00	752.00	717.33	-1.27	0.00
242.00	237.00	248.67	1.07	0.30
563.00	615.00	620.00	1.23	0.00
509.00	576.00	582.00	1.09	0.11
212.00	245.00	238.67	1.11	0.12
1558.00	1588.00	1641.33	1.03	0.44
368.00	380.00	394.67	-1.03	0.61
396.00	401.00	413.00	1.18	0.01
346.00	385.00	361.67	1.12	0.05
560.00	512.00	528.00	-1.03	0.57
9.00	8.00	12.33	1.21	0.57
52.00	60.00	60.67	-1.17	0.19
3.00	2.00	4.00	-1.10	1.00
19.00	16.00	15.33	-1.80	0.00
438.00	505.00	473.33	-1.08	0.17
5.00	1.00	4.00	-3.06	0.00
7.00	10.00	8.67	-1.63	0.08
1128.00	1302.00	1250.00	-1.01	0.81
16.00	28.00	25.67	1.26	0.27
23.00	41.00	28.00	-1.08	0.74
165.00	181.00	187.00	1.06	0.44
1141.00	1255.00	1225.33	1.26	0.00
68.00	60.00	71.67	1.09	0.53
782.00	738.00	744.33	-1.28	0.00
1523.00	1699.00	1596.00	1.06	0.18
1170.00	1091.00	1196.00	-1.03	0.57
448.00	427.00	423.67	-1.14	0.07
2234.00	2200.00	2272.33	1.03	0.40
412.00	391.00	418.67	-1.23	0.00
105.00	90.00	91.67	-1.02	0.92
7380.00	8035.00	7692.33	-1.20	0.00
152.00	125.00	144.33	-1.12	0.17
387.00	438.00	422.67	1.02	0.76
1389.00	1549.00	1462.00	-1.07	0.09
4.00	2.00	2.67	-1.50	0.51
117.00	129.00	127.33	1.32	0.00
1867.00	1896.00	1841.00	-1.24	0.00
7804.00	7976.00	7885.67	-1.13	0.00
3201.00	3133.00	3282.67	-1.04	0.37
612.00	560.00	621.67	-1.32	0.00
400.00	465.00	479.00	-1.19	0.01
178.00	216.00	202.33	-1.01	0.94
1499.00	1665.00	1668.33	1.03	0.47
263.00	302.00	292.00	1.09	0.16

1457.00	1551.00	1480.00	-1.07	0.11
166.00	203.00	179.00	1.39	0.00
1276.00	1399.00	1429.67	1.16	0.00
1913.00	2110.00	2142.00	1.09	0.04
318.00	342.00	354.67	1.18	0.01
1422.00	1548.00	1546.33	1.10	0.02
875.00	833.00	881.00	-1.31	0.00
678.00	841.00	800.33	1.12	0.04
2384.00	2636.00	2604.67	1.02	0.63
1749.00	2039.00	2007.00	1.06	0.17
15.00	24.00	18.67	1.02	0.93
1028.00	1063.00	1032.67	-1.18	0.00
990.00	1069.00	1065.67	1.11	0.01
96.00	97.00	95.33	1.92	0.00
756.00	790.00	797.00	1.77	0.00
66.00	84.00	71.00	1.39	0.01
209.00	214.00	217.33	3.47	0.00
1.00	1.00	2.00	-1.65	0.48
1080.00	988.00	1016.00	1.02	0.68
1345.00	1370.00	1283.67	1.13	0.07
92.00	85.00	88.00	-1.07	0.54
16.00	21.00	19.33	-1.26	0.23
0.00	1.00	1.33	-4.95	0.00
114.00	136.00	125.67	1.12	0.18
76.00	88.00	86.67	-1.17	0.13
2444.00	2491.00	2491.00	-1.05	0.21
960.00	996.00	998.33	-1.11	0.01
276.00	295.00	303.00	1.03	0.69
22.00	30.00	26.33	-1.04	0.90
1286.00	1427.00	1386.33	-1.00	0.98
11.00	15.00	12.67	-1.23	0.47
261.00	241.00	271.67	-1.09	0.25
2539.00	2735.00	2686.00	-1.14	0.00
480.00	467.00	451.00	-1.37	0.00
398.00	548.00	496.33	1.02	0.77
279.00	353.00	319.67	-1.01	0.88
10298.00	11585.00	11445.00	-1.11	0.00
1266.00	1433.00	1442.67	-1.13	0.01
10089.00	9378.00	9466.33	-1.28	0.00
1346.00	1365.00	1303.67	1.03	0.65
1532.00	1725.00	1694.00	1.08	0.07
233.00	249.00	249.67	1.15	0.03
524.00	662.00	599.33	-1.07	0.19
922.00	979.00	1005.33	-1.03	0.52
304.00	334.00	328.00	1.04	0.50
2306.00	2209.00	2224.00	-1.11	0.03
210.00	237.00	236.33	-1.08	0.23
254.00	248.00	250.33	1.18	0.04

319.00	266.00	307.33	-1.08	0.31
14.00	20.00	15.67	-1.89	0.00
75.00	64.00	72.33	-1.70	0.00
53.00	54.00	57.67	-1.65	0.00
875.00	1057.00	967.33	1.04	0.38
894.00	944.00	935.00	1.10	0.04
3122.00	3647.00	3477.67	1.06	0.12
186.00	216.00	214.33	-1.48	0.00
45.00	58.00	45.67	1.63	0.00
722.00	775.00	724.33	-2.07	0.00
157.00	149.00	147.00	1.02	0.86
96.00	105.00	97.67	-1.04	0.70
65.00	78.00	71.00	1.16	0.18
58.00	61.00	62.33	1.02	0.85
70.00	66.00	71.67	-1.45	0.00
43.00	40.00	42.67	1.05	0.76
92.00	109.00	109.33	1.00	1.00
1603.00	1673.00	1656.67	-1.01	0.75
263.00	293.00	274.00	-1.29	0.00
309.00	400.00	363.67	1.01	0.85
1.00	2.00	2.67	1.54	0.59
345.00	406.00	367.67	1.09	0.17
249.00	325.00	295.67	-1.19	0.02
891.00	882.00	871.33	-1.25	0.00
2394.00	2488.00	2412.33	-1.26	0.00
5.00	7.00	5.33	-1.57	0.22
339.00	371.00	383.33	1.07	0.29
10.00	15.00	12.00	1.36	0.26
6.00	6.00	8.67	-1.01	1.00
44.00	38.00	41.67	1.13	0.40
236.00	206.00	222.00	1.11	0.15
890.00	892.00	891.00	-1.20	0.00
502.00	520.00	534.67	-1.06	0.29
1306.00	1234.00	1287.67	-1.06	0.16
1295.00	1481.00	1475.33	-1.16	0.00
3848.00	4142.00	3903.00	1.33	0.00
3.00	8.00	4.00	-1.01	1.00
1496.00	1582.00	1563.33	-1.37	0.00
241.00	285.00	261.00	1.02	0.76
2362.00	2361.00	2362.33	-1.27	0.00
18.00	17.00	18.67	-1.34	0.16
231.00	237.00	247.00	-1.31	0.00
297.00	294.00	287.67	-1.16	0.03
3009.00	3588.00	3457.67	1.21	0.00
18.00	11.00	12.67	-2.17	0.00
4101.00	4708.00	4567.67	1.28	0.00
1401.00	1852.00	1566.33	1.41	0.00
14141.00	15223.00	14912.67	-1.03	0.47

1688.00	1533.00	1576.33	-1.47	0.00
1622.00	1808.00	1714.00	-1.35	0.00
4753.00	5598.00	5424.00	1.14	0.00
14.00	19.00	15.67	-1.97	0.00
1653.00	1651.00	1666.00	1.07	0.09
26582.00	30667.00	29725.00	1.15	0.00
1312.00	1685.00	1582.33	1.18	0.00
58.00	47.00	58.33	-1.02	0.92
1.00	2.00	1.00	-2.51	0.23
509.00	600.00	578.33	1.11	0.05
7447.00	8198.00	8166.67	-1.27	0.00
2228.00	2440.00	2377.33	1.01	0.83
1948.00	2209.00	2165.67	-1.25	0.00
1.00	7.00	3.33	1.61	0.49
54.00	79.00	72.33	-1.39	0.00
24.00	13.00	23.00	-1.29	0.17
6477.00	6695.00	6645.67	-1.07	0.07
720.00	981.00	872.33	1.08	0.30
2219.00	2291.00	2266.67	1.04	0.30
1961.00	2081.00	2055.67	-1.00	0.91
232.00	240.00	254.67	-1.51	0.00
1403.00	1441.00	1501.67	1.15	0.00
1508.00	1550.00	1618.00	1.10	0.02
1039.00	1302.00	1194.67	-1.15	0.01
82.00	103.00	92.00	1.60	0.00
1731.00	1641.00	1743.00	-1.03	0.52
226.00	217.00	227.00	1.07	0.33
7402.00	7380.00	7433.33	-1.12	0.00
6242.00	6221.00	6260.33	-1.05	0.14
1300.00	1363.00	1443.33	1.13	0.01
1272.00	1365.00	1334.67	1.06	0.15
4906.00	4931.00	5017.33	-1.06	0.11
1858.00	1984.00	1930.33	-1.03	0.40
1551.00	1686.00	1636.33	1.06	0.17
700.00	838.00	795.67	1.13	0.01
49.00	49.00	48.33	-1.08	0.58
1166.00	1386.00	1316.33	1.07	0.11
522.00	564.00	553.00	1.03	0.53
704.00	729.00	742.67	1.55	0.00
220.00	239.00	237.67	1.05	0.46
1192.00	1106.00	1136.33	-1.08	0.12
360.00	404.00	379.67	-1.09	0.11
332.00	302.00	332.33	1.05	0.48
2046.00	1974.00	2073.00	-1.01	0.89
376.00	400.00	383.00	-1.08	0.18
13.00	3.00	5.67	-1.28	0.65
90.00	78.00	84.00	-1.01	1.00
688.00	721.00	702.00	-1.09	0.07

631.00	690.00	668.67	-1.06	0.17
39.00	31.00	33.67	1.33	0.08
3755.00	3971.00	3928.00	-1.10	0.01
943.00	802.00	949.00	-1.41	0.00
828.00	933.00	837.33	-1.07	0.32
9.00	19.00	13.33	1.40	0.20
0.00	0.00	0.00	-14.45	0.07
1825.00	1861.00	1907.33	1.15	0.00
4384.00	4851.00	4934.67	1.05	0.25
644.00	750.00	725.67	1.14	0.01
2095.00	2152.00	2195.33	-1.02	0.60
1275.00	1189.00	1254.33	-1.04	0.47
2609.00	2795.00	2706.67	1.20	0.00
485.00	575.00	567.33	1.37	0.00
36.00	39.00	44.00	1.09	0.59
141.00	182.00	163.67	-1.01	0.92
408.00	428.00	443.33	2.16	0.00
27.00	18.00	20.67	1.39	0.15
6224.00	6776.00	6609.67	-1.06	0.07
5020.00	5017.00	5134.00	-1.17	0.00
1029.00	975.00	1083.67	1.19	0.00
166.00	215.00	202.00	1.69	0.00
245.00	203.00	210.00	1.36	0.00
32.00	42.00	34.67	2.01	0.00
27.00	18.00	25.67	1.13	0.53
1.00	2.00	1.33	-2.63	0.12
8.00	2.00	5.67	1.12	0.86
13.00	14.00	15.33	-1.34	0.20
23.00	29.00	23.67	-1.49	0.02
224.00	273.00	258.00	1.12	0.12
30.00	36.00	31.33	-1.49	0.01
512.00	526.00	533.33	-1.03	0.59
368.00	416.00	385.67	1.04	0.53
365.00	399.00	408.00	1.19	0.00
589.00	699.00	632.67	1.11	0.18
465.00	497.00	505.33	1.14	0.02
29550.00	32233.00	31141.00	-1.10	0.00
1351.00	1505.00	1456.33	1.04	0.30
1051.00	1158.00	1097.33	1.15	0.01
985.00	1021.00	1032.00	1.00	0.99
2683.00	2873.00	2992.33	1.26	0.00
194.00	227.00	216.67	1.12	0.09
1067.00	1203.00	1203.33	1.08	0.12
57.00	49.00	58.33	-1.12	0.38
8.00	5.00	7.00	1.04	1.00
103.00	83.00	100.67	-1.05	0.69
84.00	84.00	92.67	-1.16	0.12
1272.00	1427.00	1343.00	1.01	0.88

285.00	327.00	296.67	-2.05	0.00
980.00	968.00	1032.00	-1.02	0.63
2652.00	2582.00	2635.67	-1.07	0.09
111.00	124.00	110.00	-1.07	0.47
1564.00	1553.00	1589.33	-1.12	0.00
7.00	16.00	11.00	-1.53	0.09
3789.00	3796.00	3807.67	-1.09	0.02
71.00	78.00	83.00	1.16	0.19
1542.00	1477.00	1515.00	1.00	0.96
6231.00	6323.00	6608.00	1.08	0.04
2735.00	2862.00	2844.67	-1.04	0.29
3630.00	4251.00	4166.33	1.04	0.34
1050.00	1065.00	1136.67	1.13	0.02
2186.00	2331.00	2281.67	1.13	0.00
3128.00	3231.00	3322.00	-1.20	0.00
1366.00	1526.00	1509.33	1.08	0.05
802.00	787.00	810.33	-1.11	0.02
2113.00	2026.00	2052.33	-1.04	0.37
2552.00	2605.00	2539.00	-1.05	0.32
379.00	410.00	384.33	-1.11	0.09
377.00	437.00	396.33	1.00	1.00
1160.00	1316.00	1316.00	-1.07	0.13
1112.00	1127.00	1163.33	1.04	0.37
308.00	333.00	367.00	1.16	0.07
3645.00	4273.00	4067.33	-1.07	0.10
1257.00	1311.00	1319.00	1.11	0.01
213.00	227.00	233.67	-1.08	0.26
18.00	19.00	19.67	-1.13	0.54
7.00	10.00	7.67	2.46	0.02
1653.00	1795.00	1723.67	-1.08	0.06
866.00	967.00	930.67	-1.23	0.00
2766.00	3201.00	3039.67	-1.03	0.37
493.00	537.00	528.67	1.39	0.00
21.00	32.00	21.67	-1.46	0.05
1778.00	1785.00	1791.33	-1.17	0.00
1981.00	1976.00	2045.33	-1.10	0.01
151.00	102.00	130.00	-1.38	0.00
1269.00	1421.00	1389.33	1.24	0.00
1.00	6.00	6.00	-1.29	0.55
134.00	148.00	138.33	-1.90	0.00
10.00	16.00	14.67	-1.29	0.22
11003.00	12695.00	11809.33	-1.01	0.89
176.00	183.00	191.67	-1.39	0.00
569.00	490.00	544.00	-1.01	0.85
12.00	6.00	7.33	-1.19	0.68
644.00	651.00	655.33	-1.19	0.00
246.00	242.00	247.33	1.11	0.24
2246.00	2020.00	2058.00	-1.28	0.00

657.00	631.00	649.33	-1.18	0.00
9.00	6.00	11.33	-1.66	0.03
1682.00	1672.00	1806.33	-1.01	0.86
906.00	910.00	862.00	-1.00	0.98
1182.00	1303.00	1200.33	-1.10	0.11
38.00	45.00	48.00	1.12	0.47
144.00	164.00	157.67	-1.16	0.04
27.00	19.00	22.00	-1.24	0.28
7035.00	7084.00	7262.33	-1.26	0.00
2452.00	2787.00	2783.00	1.19	0.00
2625.00	2755.00	2782.00	-1.09	0.02
111.00	95.00	105.67	-1.21	0.03
1777.00	1723.00	1802.00	-1.14	0.00
1912.00	1920.00	1900.67	-1.13	0.01
1058.00	976.00	1047.33	-1.88	0.00
1019.00	1043.00	1025.67	-1.31	0.00
227.00	244.00	249.67	-1.26	0.00
7454.00	7777.00	7605.67	-1.22	0.00
906.00	973.00	1026.67	1.00	0.94
7.00	23.00	14.67	-1.52	0.05
8.00	6.00	10.33	-1.95	0.00
2452.00	2693.00	2608.33	-1.20	0.00
103.00	93.00	103.00	-1.03	0.79
6.00	7.00	6.67	1.23	0.62
259.00	270.00	262.00	1.23	0.00
503.00	504.00	510.67	-1.20	0.00
209.00	206.00	219.67	-1.50	0.00
922.00	983.00	980.67	-1.03	0.43
1101.00	1123.00	1104.33	-1.07	0.11
1128.00	1137.00	1138.33	-2.62	0.00
1922.00	1776.00	1836.00	-1.09	0.08
695.00	711.00	691.67	1.04	0.41
102.00	119.00	113.33	1.21	0.04
319.00	334.00	344.33	-1.49	0.00
357.00	406.00	376.33	-1.02	0.75
720.00	873.00	781.33	-1.02	0.71
77.00	88.00	79.33	-1.61	0.00
328.00	319.00	347.00	-2.07	0.00
1305.00	1435.00	1382.67	-1.04	0.32
2752.00	2866.00	2967.00	1.07	0.09
4.00	14.00	10.67	-1.05	0.91
1275.00	1501.00	1420.00	1.14	0.00
75.00	65.00	60.33	-1.75	0.00
384.00	417.00	398.67	1.14	0.03
593.00	649.00	656.33	1.13	0.03
1.00	5.00	2.33	-1.70	0.38
202.00	228.00	211.00	1.16	0.09
607.00	726.00	658.33	1.06	0.39

34.00	46.00	36.33	-1.12	0.47
1770.00	1631.00	1863.67	1.10	0.07
868.00	892.00	951.67	-1.05	0.31
388.00	409.00	421.33	1.07	0.24
433.00	467.00	462.67	1.13	0.02
660.00	658.00	658.67	-1.11	0.03
95.00	101.00	101.00	-1.23	0.02
623.00	733.00	676.00	1.26	0.00
293.00	347.00	305.33	1.05	0.51
159.00	195.00	180.00	-1.00	1.00
3418.00	3373.00	3482.00	1.08	0.06
1837.00	1974.00	2052.67	1.06	0.18
1454.00	1629.00	1583.67	1.16	0.00
577.00	686.00	611.00	1.12	0.07
396.00	391.00	406.33	-1.02	0.72
1837.00	1847.00	1865.00	1.19	0.00
5.00	6.00	6.00	-1.07	1.00
884.00	1000.00	959.33	1.12	0.01
255.00	285.00	253.00	1.24	0.02
20.00	13.00	18.33	-1.07	0.79
61.00	66.00	59.33	1.22	0.14
1134.00	1200.00	1199.67	1.90	0.00
340.00	323.00	353.00	-1.16	0.01
1237.00	1296.00	1342.33	1.07	0.12
194.00	206.00	212.33	1.16	0.03
785.00	873.00	846.33	-1.02	0.67
653.00	724.00	680.67	-1.11	0.03
1039.00	1060.00	1064.33	1.01	0.80
44.00	50.00	53.33	1.28	0.07
735.00	825.00	782.00	-1.06	0.20
799.00	933.00	860.33	-1.13	0.01
451.00	446.00	455.33	-1.04	0.48
38.00	31.00	33.67	-1.35	0.03
226.00	239.00	242.00	-1.16	0.05
560.00	611.00	609.67	1.20	0.00
592.00	610.00	605.67	-1.12	0.02
177.00	194.00	185.33	1.07	0.34
302.00	291.00	309.00	-1.23	0.00
4117.00	4631.00	4470.67	1.91	0.00
3562.00	3890.00	3935.67	1.36	0.00
3575.00	3738.00	3900.00	1.18	0.00
4555.00	4832.00	4934.33	1.14	0.00
2843.00	3122.00	3187.00	1.19	0.00
1617.00	1673.00	1723.67	1.17	0.00
2691.00	2901.00	2960.00	1.38	0.00
880.00	930.00	955.33	1.21	0.00
517.00	467.00	481.00	1.08	0.24
2146.00	2098.00	2195.67	1.00	0.93

12.00	20.00	17.33	1.65	0.04
60.00	89.00	74.00	1.21	0.17
525.00	576.00	579.33	1.35	0.00
483.00	526.00	541.33	-1.00	0.97
483.00	541.00	540.33	-1.06	0.31
372.00	400.00	404.33	1.03	0.62
502.00	625.00	590.33	-1.21	0.00
63.00	71.00	72.67	-1.03	0.81
31.00	41.00	38.00	-1.09	0.54
616.00	607.00	615.33	-1.25	0.00
512.00	447.00	469.33	-1.03	0.66
181.00	196.00	191.00	-1.32	0.00
53.00	37.00	43.00	1.30	0.07
146.00	143.00	141.00	-1.35	0.00
1469.00	1455.00	1470.33	-1.39	0.00
466.00	569.00	518.67	1.10	0.08
59.00	58.00	68.00	-1.15	0.27
495.00	520.00	541.00	1.10	0.08
55.00	69.00	66.33	-1.13	0.28
792.00	867.00	831.00	-2.01	0.00
12.00	9.00	9.67	1.19	0.59
32.00	39.00	33.67	1.31	0.10
6771.00	6986.00	6864.00	-1.28	0.00
5.00	8.00	10.00	-1.05	0.90
2591.00	2929.00	2893.33	1.09	0.03
33.00	38.00	34.00	-2.15	0.00
13876.00	15260.00	14724.33	-1.22	0.00
598.00	559.00	625.33	-1.01	0.93
170.00	183.00	178.00	-1.11	0.13
28.00	35.00	34.00	-1.06	0.70
894.00	877.00	933.33	-1.04	0.40
455.00	475.00	470.00	-1.04	0.49
291.00	335.00	329.33	-1.00	0.97
109.00	149.00	135.67	2.70	0.00
570.00	593.00	603.33	1.04	0.47
1187.00	1244.00	1275.33	-1.04	0.30
1918.00	2008.00	1919.33	-1.00	1.00
532.00	533.00	549.00	1.18	0.00
387.00	429.00	425.67	1.05	0.48
50.00	48.00	54.67	-1.47	0.00
1703.00	1668.00	1657.00	-1.23	0.00
56.00	71.00	57.67	-1.08	0.58
798.00	919.00	918.33	1.10	0.04
956.00	920.00	963.67	-1.08	0.07
2.00	4.00	2.67	-1.26	0.82
67.00	58.00	64.67	-1.73	0.00
761.00	836.00	818.67	-1.06	0.26
2.00	1.00	1.33	-1.71	0.56

1061.00	1231.00	1237.67	1.06	0.27
9910.00	11134.00	11056.00	1.07	0.07
1142.00	1265.00	1255.33	1.15	0.00
10069.00	10843.00	10570.67	-1.03	0.35
193.00	202.00	212.67	1.16	0.05
55.00	65.00	61.67	-1.05	0.70
2206.00	2573.00	2375.67	-1.05	0.29
5.00	10.00	7.33	-1.47	0.24
1054.00	1185.00	1165.00	1.03	0.50
22.00	24.00	25.00	1.54	0.03
10.00	9.00	13.00	1.90	0.02
142.00	147.00	143.00	-1.10	0.34
77.00	72.00	84.00	1.09	0.45
1786.00	2103.00	2055.33	1.15	0.01
3678.00	3894.00	3878.00	-1.14	0.00
737.00	648.00	635.00	-1.18	0.07
9.00	4.00	5.33	-1.51	0.29
2215.00	2307.00	2329.00	1.06	0.11
1693.00	1990.00	1853.00	1.45	0.00
318.00	364.00	346.00	-1.04	0.47
1052.00	1006.00	1046.67	1.19	0.00
798.00	857.00	888.67	1.07	0.18
464.00	480.00	458.67	1.14	0.03
922.00	1032.00	984.00	1.05	0.47
20.00	32.00	28.00	-1.35	0.05
494.00	586.00	555.00	1.12	0.04
876.00	1042.00	958.00	-1.00	0.93
169.00	157.00	167.67	1.04	0.60
9799.00	10579.00	10834.67	1.09	0.02
234.00	249.00	247.00	1.11	0.13
2700.00	2927.00	2832.67	-1.11	0.00
72.00	107.00	104.33	1.00	1.00
893.00	876.00	883.33	-1.31	0.00
1671.00	1697.00	1679.67	-1.05	0.27
9.00	13.00	13.67	1.01	1.00
108.00	127.00	123.00	1.20	0.04
937.00	925.00	988.33	1.07	0.15
1128.00	1299.00	1246.67	1.09	0.03
1197.00	1125.00	1178.33	-1.22	0.00
605.00	597.00	613.33	-1.04	0.41
687.00	755.00	733.00	-1.04	0.35
1310.00	1312.00	1366.67	1.12	0.01
1558.00	1474.00	1542.67	-1.26	0.00
1159.00	1199.00	1188.67	-1.13	0.01
1278.00	1550.00	1455.00	1.18	0.00
219.00	230.00	228.67	-1.29	0.00
1111.00	1097.00	1148.00	-1.09	0.05
380.00	458.00	430.67	1.09	0.15

342.00	366.00	374.00	1.12	0.04
491.00	562.00	538.00	-1.29	0.00
1169.00	1222.00	1210.33	1.16	0.00
2027.00	1939.00	2020.67	-1.11	0.01
4.00	7.00	6.67	-1.66	0.11
1620.00	1620.00	1704.67	-1.12	0.01
4644.00	5059.00	4969.67	1.12	0.00
4.00	1.00	2.67	-1.26	0.82
1817.00	1984.00	1935.33	1.08	0.04
2888.00	3289.00	3164.00	1.03	0.42
2159.00	2559.00	2380.00	-1.22	0.00
1319.00	1328.00	1343.33	-1.08	0.06
3333.00	3043.00	3182.67	-1.18	0.00
44.00	33.00	42.00	-2.45	0.00
841.00	966.00	1021.67	1.16	0.02
44.00	25.00	37.00	-1.17	0.30
669.00	672.00	652.67	-1.29	0.00
1090.00	949.00	994.00	-1.25	0.00
1881.00	1914.00	1893.00	1.13	0.01
849.00	1019.00	922.00	-1.07	0.25
2747.00	2575.00	2768.33	-1.14	0.00
865.00	746.00	819.67	-1.28	0.00
5163.00	5075.00	4953.33	-1.00	0.96
3.00	6.00	6.33	-1.01	1.00
16743.00	17581.00	17282.00	-1.15	0.00
37.00	33.00	38.67	-1.07	0.67
48.00	68.00	55.67	-3.52	0.00
1148.00	1185.00	1198.67	-1.18	0.00
5241.00	5402.00	5520.67	1.05	0.19
32.00	45.00	44.00	-1.32	0.04
125.00	99.00	112.67	1.37	0.00
10749.00	10483.00	10513.00	-1.07	0.12
2138.00	2224.00	2154.67	-1.36	0.00
1460.00	1352.00	1437.00	-1.25	0.00
984.00	1053.00	1007.33	-1.42	0.00
4121.00	4147.00	4228.67	1.02	0.54
5406.00	5809.00	5873.33	1.06	0.11
14771.00	15728.00	15955.67	1.13	0.00
2325.00	2600.00	2587.67	1.05	0.21
1328.00	1313.00	1379.00	1.19	0.00
1078.00	1164.00	1264.00	1.26	0.00
10.00	16.00	15.00	1.63	0.07
3029.00	3256.00	3356.00	1.21	0.00
1257.00	1285.00	1362.33	1.13	0.01
3258.00	3381.00	3517.33	1.11	0.01
2405.00	2783.00	2804.00	1.25	0.00
1487.00	1661.00	1657.33	1.21	0.00
484.00	638.00	629.00	1.13	0.10

1267.00	1279.00	1365.67	1.20	0.00
834.00	938.00	931.00	1.23	0.00
1736.00	1935.00	1970.33	1.21	0.00
2072.00	2252.00	2269.33	1.21	0.00
170.00	189.00	183.33	1.15	0.07
434.00	474.00	484.67	1.00	0.99
2169.00	2288.00	2321.00	-1.06	0.21
204.00	248.00	234.33	-1.06	0.45
2236.00	2555.00	2439.67	-1.10	0.02
2030.00	1979.00	2062.33	-1.24	0.00
1231.00	1356.00	1264.67	-1.07	0.12
2477.00	2348.00	2474.67	1.05	0.28
1519.00	1498.00	1578.00	1.06	0.25
994.00	1087.00	1110.33	1.08	0.11
1667.00	1769.00	1772.00	1.05	0.29
702.00	860.00	803.67	1.05	0.35
199.00	203.00	197.67	-1.09	0.20
1026.00	1111.00	1067.33	1.10	0.04
67.00	68.00	72.67	-1.56	0.00
88.00	111.00	99.00	1.12	0.26
1171.00	1182.00	1179.67	1.02	0.67
2508.00	2707.00	2659.33	1.13	0.00
3.00	2.00	3.33	1.61	0.47
758.00	844.00	867.67	1.19	0.00
1406.00	1460.00	1514.00	1.06	0.17
105.00	103.00	105.67	1.38	0.00
1328.00	1437.00	1528.33	1.15	0.01
974.00	1136.00	1143.67	1.20	0.00
1276.00	1339.00	1353.33	1.06	0.18
326.00	382.00	352.00	-1.23	0.00
413.00	395.00	415.67	-1.35	0.00
19.00	27.00	28.67	-1.25	0.16
47.00	48.00	42.00	1.15	0.35
53.00	67.00	53.67	-1.70	0.00
1716.00	1682.00	1713.67	-1.21	0.00
82.00	85.00	86.67	1.33	0.01
11.00	11.00	10.00	-1.31	0.35
606.00	592.00	620.67	-1.14	0.00
248.00	260.00	260.67	-1.10	0.11
692.00	747.00	764.00	1.17	0.00
228.00	220.00	235.33	1.16	0.04
953.00	1080.00	1088.67	1.10	0.04
1801.00	2029.00	1936.00	-1.06	0.13
2877.00	2886.00	2969.33	1.05	0.23
349.00	372.00	363.00	-1.06	0.26
2554.00	2569.00	2533.00	-1.16	0.00
51.00	37.00	41.33	1.07	0.68
2142.00	2385.00	2162.67	-1.13	0.04

507.00	479.00	500.67	-1.59	0.00
1557.00	1713.00	1702.33	1.08	0.06
869.00	858.00	852.33	-1.19	0.00
1396.00	1405.00	1384.67	-1.02	0.68
7569.00	7219.00	7546.33	-1.14	0.00
351.00	404.00	378.67	1.05	0.39
742.00	760.00	745.33	-1.01	0.83
463.00	479.00	469.67	-1.13	0.03
379.00	334.00	375.67	-1.14	0.03
1326.00	1277.00	1339.00	-1.14	0.00
2408.00	2519.00	2585.67	1.02	0.55
136.00	126.00	127.00	1.02	0.83
132.00	153.00	142.67	1.15	0.08
5055.00	5190.00	5350.33	1.03	0.35
1745.00	2033.00	2003.00	1.33	0.00
1325.00	1337.00	1415.00	1.15	0.00
38.00	33.00	33.00	-1.12	0.53
1364.00	1490.00	1530.33	1.30	0.00
19.00	10.00	15.67	-1.12	0.74
45.00	40.00	41.33	1.17	0.33
348.00	352.00	366.67	-1.05	0.38
69.00	71.00	75.33	-1.35	0.01
2779.00	2797.00	2789.33	1.01	0.79
113.00	107.00	110.33	-1.02	0.84
2.00	1.00	2.67	-10.69	0.00
3.00	1.00	3.00	-1.34	0.67
1056.00	1152.00	1122.33	1.05	0.20
10.00	16.00	16.00	1.12	0.69
18027.00	16822.00	17305.00	-1.58	0.00
1.00	3.00	2.33	-1.84	0.27
866.00	943.00	966.67	1.08	0.13
4776.00	5160.00	4973.33	1.17	0.00
17.00	20.00	22.00	1.25	0.29
97.00	103.00	100.33	-1.01	0.95
1529.00	1573.00	1590.00	1.27	0.00
264.00	284.00	271.67	1.17	0.03
1310.00	1344.00	1360.67	1.15	0.00
58.00	61.00	56.67	1.11	0.42
60.00	56.00	53.00	-1.25	0.07
12.00	14.00	16.00	-1.22	0.35
1106.00	1173.00	1138.33	1.06	0.15
5.00	7.00	5.67	1.51	0.35
1527.00	1444.00	1568.00	-1.04	0.43
382.00	423.00	388.33	1.15	0.05
502.00	533.00	499.33	-1.00	0.99
493.00	505.00	500.67	1.10	0.05
0.00	0.00	0.33	-3.96	0.23
43.00	30.00	39.33	-1.65	0.00

208.00	208.00	210.33	1.41	0.00
49.00	41.00	47.00	1.00	1.00
4.00	6.00	6.00	-2.45	0.00
1802.00	1594.00	1718.00	-1.07	0.19
1.00	7.00	6.00	-1.46	0.32
1281.00	1310.00	1372.67	1.04	0.39
1489.00	1346.00	1456.00	-1.09	0.05
361.00	405.00	411.67	-1.04	0.45
2200.00	1974.00	2063.33	1.05	0.38
746.00	904.00	785.67	1.36	0.00
820.00	771.00	821.00	-1.10	0.03
1723.00	1884.00	1853.67	-1.22	0.00
290.00	328.00	337.00	1.05	0.47
72.00	69.00	72.33	1.49	0.00
7.00	7.00	5.67	1.05	1.00
1.00	2.00	1.67	-1.58	0.59
1648.00	1619.00	1673.33	1.10	0.02
1135.00	1179.00	1147.00	-1.31	0.00
78.00	71.00	82.67	-1.24	0.03
3438.00	3376.00	3468.67	1.13	0.00
1957.00	2284.00	2077.00	-1.08	0.16
2.00	3.00	2.67	-2.46	0.04
89.00	102.00	95.33	-1.21	0.04
570.00	563.00	627.00	1.04	0.54
458.00	455.00	461.33	1.13	0.02
730.00	868.00	821.00	1.26	0.00
1127.00	1159.00	1175.00	-1.14	0.00
371.00	429.00	395.00	1.14	0.03
1021.00	1070.00	1044.33	1.05	0.27
6.00	3.00	4.33	-1.16	0.85
164.00	125.00	151.33	-1.22	0.03
810.00	918.00	893.67	1.03	0.54
706.00	645.00	705.67	-1.04	0.44
240.00	214.00	240.33	-1.16	0.04
143.00	108.00	133.67	1.06	0.55
175.00	186.00	192.67	1.45	0.00
6.00	8.00	5.00	-1.54	0.28
445.00	419.00	456.00	1.09	0.12
48.00	50.00	58.33	-1.47	0.00
2877.00	3019.00	3160.33	1.15	0.00
573.00	571.00	589.67	1.02	0.75
1590.00	1530.00	1614.67	-1.11	0.01
241.00	278.00	260.33	1.17	0.02
37.00	19.00	27.33	1.42	0.06
4998.00	5107.00	5193.33	1.01	0.75
21.00	22.00	20.00	-1.74	0.00
66.00	79.00	76.67	1.16	0.20
21.00	17.00	20.00	-1.08	0.80

1088.00	1123.00	1189.00	1.03	0.49
1766.00	1994.00	1927.33	-1.02	0.62
3402.00	3300.00	3346.00	-1.04	0.33
2470.00	2218.00	2304.00	-1.26	0.00
612.00	541.00	555.67	-1.23	0.00
130.00	124.00	127.00	-1.09	0.30
361.00	446.00	436.67	1.13	0.07
849.00	840.00	863.67	-1.12	0.01
741.00	825.00	793.67	-1.09	0.09
77.00	109.00	91.67	-1.05	0.71
1178.00	1258.00	1240.33	-1.17	0.00
329.00	378.00	360.67	1.09	0.11
345.00	408.00	378.67	1.09	0.12
465.00	555.00	494.67	-1.03	0.68
272.00	339.00	299.00	1.06	0.41
2376.00	2308.00	2299.00	-1.04	0.37
1268.00	1196.00	1281.00	-1.20	0.00
1697.00	1955.00	1817.33	1.10	0.05
6686.00	6644.00	6851.67	-1.03	0.41
4557.00	4767.00	4824.67	1.27	0.00
3969.00	4111.00	4111.00	1.02	0.64
2.00	4.00	4.33	-1.70	0.18
673.00	730.00	753.33	1.14	0.01
337.00	342.00	357.33	-1.18	0.00
311.00	325.00	320.67	1.02	0.77
6394.00	7049.00	6741.33	1.02	0.59
698.00	689.00	745.67	1.04	0.51
2077.00	2328.00	2334.67	1.03	0.43
179.00	136.00	173.67	-1.07	0.52
30.00	22.00	23.67	-1.08	0.70
2057.00	2291.00	2199.67	1.13	0.00
298.00	280.00	307.67	-1.03	0.62
480.00	434.00	436.33	1.16	0.04
673.00	750.00	742.00	-1.06	0.22
712.00	788.00	754.33	1.15	0.00
14.00	21.00	14.33	1.91	0.02
439.00	496.00	462.67	1.48	0.00
1402.00	1767.00	1677.33	1.85	0.00
1229.00	1258.00	1305.67	-1.11	0.01
157.00	169.00	167.00	1.25	0.00
572.00	552.00	618.33	1.23	0.00
1359.00	1387.00	1411.33	1.05	0.25
16.00	17.00	14.67	1.40	0.18
1274.00	1447.00	1412.00	-1.02	0.64
1.00	13.00	7.00	2.23	0.13
123.00	134.00	136.33	-1.05	0.58
1016.00	1086.00	1089.00	-1.05	0.31
1267.00	1289.00	1308.33	1.55	0.00

681.00	723.00	754.00	-1.02	0.66
654.00	684.00	690.67	1.28	0.00
538.00	511.00	523.00	-1.02	0.74
1943.00	1809.00	1839.00	-1.39	0.00
3699.00	4376.00	4051.00	1.08	0.06
22.00	20.00	18.67	-1.70	0.00
449.00	457.00	450.67	-1.25	0.00
237.00	226.00	229.00	-1.28	0.00
690.00	734.00	732.00	-1.06	0.23
7.00	3.00	4.67	-1.22	0.73
1338.00	1327.00	1425.33	1.01	0.89
26.00	19.00	39.00	2.05	0.03
26.00	8.00	16.00	-1.40	0.18
204.00	230.00	231.33	-1.01	0.88
11.00	8.00	10.33	-1.40	0.21
4.00	4.00	3.67	-2.08	0.07
1706.00	1664.00	1748.00	1.43	0.00
27.00	25.00	25.67	-1.51	0.01
32.00	12.00	22.00	1.05	0.82
1233.00	1480.00	1399.33	1.12	0.02
280.00	329.00	328.00	1.03	0.62
653.00	747.00	693.00	-1.11	0.04
57.00	66.00	72.67	-1.21	0.13
227.00	219.00	223.00	-1.34	0.00
133.00	151.00	146.00	-1.31	0.00
118.00	125.00	120.67	1.59	0.00
11.00	11.00	10.00	1.14	0.70
695.00	795.00	760.00	1.13	0.01
1294.00	1249.00	1310.33	-1.10	0.02
41.00	44.00	43.00	-1.17	0.25
82.00	85.00	94.33	1.19	0.10
667.00	718.00	697.33	1.06	0.24
6.00	11.00	6.67	-1.11	0.88
30.00	56.00	40.33	1.05	0.79
8.00	5.00	5.33	-1.88	0.06
1563.00	1532.00	1599.33	-1.54	0.00
10.00	11.00	9.33	1.25	0.49
65.00	59.00	63.33	-1.28	0.02
401.00	409.00	416.00	1.04	0.45
3.00	1.00	4.00	-1.67	0.23
547.00	545.00	545.00	-1.13	0.02
224.00	208.00	235.33	1.33	0.00
415.00	383.00	391.00	-1.01	0.87
996.00	1011.00	1013.67	-1.09	0.06
448.00	493.00	506.33	1.17	0.00
253.00	264.00	253.67	-1.01	0.97
336.00	332.00	350.00	-1.04	0.52
0.00	5.00	2.00	-1.65	0.46

8805.00	10488.00	9985.00	1.22	0.00
1975.00	2276.00	2161.67	3.38	0.00
954.00	1015.00	1040.67	1.34	0.00
2186.00	2127.00	2123.00	-1.10	0.03
20.00	10.00	15.67	1.29	0.32
930.00	1204.00	1103.00	1.21	0.00
1336.00	1277.00	1355.33	1.06	0.23
5.00	7.00	6.33	-1.28	0.55
1944.00	1981.00	2018.00	1.06	0.13
379.00	491.00	445.00	1.19	0.01
507.00	628.00	601.00	-1.08	0.15
179.00	205.00	205.00	-1.07	0.29
593.00	665.00	662.67	1.16	0.00
313.00	275.00	314.33	1.12	0.13
188.00	209.00	204.33	-1.07	0.34
1209.00	1487.00	1379.67	1.15	0.00
419.00	503.00	484.67	1.27	0.00
336.00	312.00	345.33	-1.05	0.48
518.00	522.00	540.67	1.00	0.93
23.00	27.00	27.00	-2.12	0.00
73.00	66.00	73.00	-1.04	0.77
534.00	521.00	524.00	-1.36	0.00
518.00	525.00	525.67	1.05	0.35
203.00	211.00	204.67	-1.18	0.01
10.00	7.00	9.67	-1.08	0.90
65.00	88.00	77.00	-1.09	0.45
593.00	596.00	606.67	2.44	0.00
303.00	304.00	303.67	-1.28	0.00
643.00	640.00	613.67	1.12	0.08
86.00	93.00	84.67	-1.34	0.00
1240.00	1329.00	1284.67	-1.15	0.00
2615.00	2927.00	2786.33	-1.04	0.31
431.00	410.00	424.33	-1.05	0.39
19.00	3.00	10.33	-1.85	0.02
233.00	236.00	220.00	-1.33	0.00
180.00	156.00	166.00	-1.35	0.00
85.00	95.00	82.67	-1.19	0.11
3253.00	3488.00	3415.33	1.05	0.15
305.00	319.00	310.67	-1.73	0.00
394.00	425.00	421.00	1.25	0.00
1336.00	1533.00	1469.00	1.17	0.00
1882.00	2169.00	2149.67	1.21	0.00
859.00	1009.00	910.33	-1.05	0.38
24.00	41.00	33.00	-1.26	0.12
265.00	251.00	270.33	-1.46	0.00
1560.00	1776.00	1756.00	1.14	0.00
1266.00	1303.00	1258.00	-1.13	0.01
79.00	95.00	90.00	-1.24	0.02

251.00	239.00	260.33	1.05	0.48
353.00	405.00	408.67	1.17	0.01
90.00	72.00	79.33	-1.02	0.91
3708.00	3967.00	3933.33	1.16	0.00
379.00	404.00	386.33	1.28	0.00
187.00	244.00	225.67	1.21	0.01
260.00	289.00	305.67	-1.09	0.23
521.00	576.00	564.00	1.17	0.00
2126.00	1934.00	2001.33	-1.18	0.00
278.00	279.00	265.00	1.07	0.36
541.00	591.00	571.67	1.08	0.12
1778.00	1836.00	1883.33	-1.02	0.53
982.00	1046.00	975.33	1.03	0.56
378.00	473.00	424.00	1.07	0.23
14.00	21.00	16.33	1.10	0.69
475.00	553.00	534.67	1.14	0.02
917.00	913.00	930.33	-1.08	0.08
10.00	13.00	11.67	-1.39	0.20
862.00	898.00	864.00	-1.13	0.01
1880.00	1880.00	1918.00	-1.07	0.08
1415.00	1502.00	1492.00	1.17	0.00
1944.00	2326.00	2196.33	-1.02	0.67
1227.00	1274.00	1285.00	-1.03	0.49
20.00	19.00	17.33	-1.07	0.85
1551.00	1675.00	1692.33	-1.16	0.00
20.00	21.00	19.67	-1.47	0.03
758.00	865.00	820.00	1.08	0.12
392.00	368.00	390.33	1.16	0.01
1075.00	1108.00	1131.67	-1.09	0.04
88.00	96.00	89.67	1.59	0.00
140.00	178.00	176.33	-1.15	0.10
339.00	409.00	364.00	1.40	0.00
1339.00	1542.00	1534.33	1.28	0.00
1335.00	1480.00	1496.33	-1.02	0.64
1246.00	1344.00	1354.00	1.09	0.05
294.00	329.00	303.67	1.21	0.01
1196.00	1431.00	1330.67	-1.04	0.42
669.00	810.00	771.33	-1.11	0.05
1697.00	1574.00	1635.00	-1.17	0.00
145.00	158.00	154.33	-1.25	0.00
37.00	28.00	32.33	1.02	0.95
1070.00	1258.00	1184.00	-1.19	0.00
5.00	11.00	7.33	1.20	0.64
754.00	879.00	816.00	-1.10	0.07
154.00	123.00	141.33	-1.25	0.01
1511.00	1363.00	1438.00	-1.27	0.00
22016.00	21932.00	22876.33	-1.09	0.05
40.00	28.00	28.67	-1.08	0.76

3.00	3.00	3.67	-1.19	0.84
283.00	279.00	298.00	-1.27	0.00
2344.00	2746.00	2583.00	-1.35	0.00
1577.00	1819.00	1695.00	1.15	0.00
773.00	920.00	864.67	1.10	0.08
219.00	194.00	222.67	-1.70	0.00
426.00	409.00	407.33	-1.36	0.00
2030.00	2112.00	2150.33	1.04	0.24
1077.00	1102.00	1115.33	1.14	0.01
631.00	664.00	672.00	1.23	0.00
24.00	22.00	24.00	-1.45	0.02
335.00	327.00	328.00	-1.12	0.05
13.00	6.00	10.00	-1.15	0.72
477.00	550.00	506.33	-1.20	0.00
9746.00	10872.00	10920.00	1.19	0.00
1332.00	1531.00	1505.33	1.22	0.00
1771.00	1910.00	1950.67	1.16	0.00
542.00	640.00	606.67	1.37	0.00
1183.00	1242.00	1228.33	-1.05	0.26
454.00	473.00	459.67	1.00	0.94
174.00	196.00	194.67	-1.27	0.00
658.00	612.00	653.33	1.02	0.70
590.00	573.00	581.33	-1.03	0.58
229.00	273.00	252.33	-1.21	0.00
14.00	14.00	18.33	4.06	0.00
845.00	870.00	896.00	-1.01	0.80
1554.00	1611.00	1597.33	1.23	0.00
1332.00	1458.00	1383.33	1.20	0.00
5191.00	6009.00	5756.33	1.00	0.96
336.00	370.00	367.67	1.01	0.83
1289.00	1537.00	1506.33	1.14	0.00
237.00	258.00	245.00	1.19	0.02
3.00	6.00	3.67	1.08	1.00
982.00	1017.00	1052.67	1.03	0.47
159.00	125.00	151.33	-1.16	0.15
73.00	62.00	71.33	1.22	0.10
4481.00	4706.00	4689.00	1.28	0.00
2607.00	2860.00	2865.67	1.27	0.00
15.00	14.00	15.67	1.84	0.02
77.00	67.00	78.67	1.06	0.64
19.00	34.00	28.33	-1.63	0.00
94.00	88.00	83.00	-1.27	0.03
1302.00	1341.00	1363.00	1.03	0.56
95.00	77.00	88.33	1.16	0.16
225.00	297.00	268.67	1.06	0.42
1.00	0.00	1.00	-2.51	0.23
19.00	21.00	24.33	-1.08	0.70
3.00	6.00	6.00	-1.01	1.00

616.00	597.00	632.67	-1.18	0.00
530.00	643.00	609.33	1.03	0.53
36.00	43.00	47.00	1.03	0.91
237.00	221.00	239.67	1.10	0.17
764.00	811.00	821.00	1.02	0.74
654.00	668.00	667.67	-1.12	0.02
1066.00	1174.00	1173.67	1.09	0.04
57.00	46.00	60.00	-1.18	0.16
393.00	428.00	438.33	1.02	0.76
461.00	456.00	469.00	-1.07	0.35
760.00	762.00	770.33	1.08	0.10
2608.00	3030.00	2884.33	1.19	0.00
4.00	6.00	3.67	1.34	0.65
6.00	9.00	7.00	1.15	0.75
1883.00	1998.00	2032.67	1.10	0.01
431.00	497.00	480.67	1.33	0.00
83.00	118.00	102.00	1.12	0.26
444.00	513.00	509.00	1.03	0.57
278.00	282.00	295.67	1.19	0.01
435.00	538.00	536.67	1.03	0.65
1356.00	1337.00	1378.33	1.02	0.57
109.00	130.00	129.33	1.03	0.75
3.00	5.00	4.33	-1.77	0.14
1357.00	1301.00	1363.67	-1.18	0.00
44.00	54.00	53.33	-1.05	0.66
1386.00	1437.00	1478.33	1.23	0.00
538.00	706.00	621.00	1.04	0.57
536.00	701.00	645.33	1.12	0.05
255.00	247.00	269.33	1.00	1.00
198.00	214.00	208.33	1.24	0.00
863.00	944.00	907.67	1.04	0.43
67.00	76.00	72.33	1.21	0.12
987.00	1062.00	1070.00	-1.03	0.47
47.00	44.00	50.00	-1.07	0.60
291.00	350.00	335.67	1.03	0.67
222.00	252.00	236.67	-1.10	0.20
230.00	262.00	248.00	1.03	0.68
785.00	885.00	855.33	1.25	0.00
181.00	215.00	222.67	1.06	0.52
401.00	387.00	411.00	-1.04	0.46
971.00	1104.00	1081.00	1.08	0.11
55.00	60.00	60.33	-1.24	0.06
453.00	450.00	481.67	-1.05	0.34
2046.00	2432.00	2372.67	1.16	0.00
428.00	405.00	437.67	1.04	0.51
920.00	998.00	1039.33	1.20	0.00
50.00	60.00	53.67	1.14	0.31
11.00	21.00	15.33	1.01	1.00

4.00	4.00	3.00	-1.12	1.00
387.00	414.00	408.00	-1.04	0.47
330.00	314.00	332.67	1.22	0.01
256.00	314.00	307.33	-1.06	0.42
10.00	13.00	12.33	-1.37	0.22
283.00	267.00	290.00	-1.12	0.13
294.00	293.00	315.33	1.04	0.53
457.00	521.00	496.00	1.13	0.01
10.00	4.00	6.67	-1.21	0.66
4913.00	4995.00	5049.67	-1.16	0.00
3.00	6.00	5.67	2.32	0.07
5.00	1.00	3.00	-1.98	0.13
224.00	212.00	210.00	-1.01	0.88
158.00	182.00	180.33	1.47	0.00
6.00	14.00	13.00	1.37	0.30
888.00	925.00	967.67	1.16	0.00
87.00	92.00	89.00	1.09	0.42
1741.00	2149.00	1950.00	1.09	0.07
3232.00	2914.00	3109.67	-1.10	0.03
365.00	390.00	397.67	1.17	0.02
920.00	905.00	931.67	-1.25	0.00
449.00	482.00	480.33	-1.02	0.64
1000.00	1090.00	1062.33	1.10	0.07
260.00	242.00	265.67	-1.59	0.00
26.00	34.00	29.00	1.04	0.90
102.00	104.00	107.67	1.55	0.00
271.00	251.00	283.67	1.02	0.75
60.00	68.00	70.33	-1.07	0.57
107.00	75.00	98.67	-1.01	0.92
71.00	87.00	84.33	-1.11	0.28
12.00	24.00	16.67	2.13	0.00
22.00	17.00	20.67	-1.29	0.21
2117.00	2208.00	2171.67	-1.36	0.00
283.00	296.00	310.00	-1.05	0.43
335.00	408.00	399.33	1.31	0.00
197.00	178.00	186.00	-1.07	0.38
1601.00	1555.00	1630.67	1.05	0.19
83.00	71.00	71.00	1.21	0.12
576.00	608.00	620.00	1.19	0.00
776.00	824.00	830.67	-1.03	0.48
2086.00	2189.00	2140.67	-1.03	0.39
463.00	516.00	516.33	1.22	0.00
25.00	31.00	27.67	1.12	0.59
230.00	244.00	262.33	1.11	0.17
569.00	574.00	605.00	1.15	0.00
11.00	7.00	9.33	-2.48	0.00
406.00	517.00	477.67	1.11	0.08
30.00	34.00	35.67	1.02	0.95

559.00	569.00	575.67	-1.02	0.72
485.00	537.00	540.33	1.16	0.00
553.00	508.00	566.00	1.19	0.00
360.00	420.00	408.67	1.06	0.33
8.00	11.00	9.67	1.50	0.20
1808.00	1833.00	1878.00	1.12	0.00
317.00	356.00	349.67	-1.08	0.19
192.00	164.00	179.33	1.19	0.03
2305.00	2319.00	2435.33	1.14	0.00
255.00	259.00	264.33	1.09	0.17
918.00	926.00	1010.67	1.13	0.02
597.00	634.00	629.00	-1.12	0.01
52.00	30.00	38.67	-1.12	0.51
575.00	576.00	578.67	1.27	0.00
421.00	434.00	453.67	-1.19	0.00
382.00	407.00	391.67	1.05	0.56
485.00	475.00	508.67	1.05	0.36
23.00	19.00	20.00	-1.18	0.41
10.00	9.00	11.67	-1.45	0.13
274.00	327.00	307.67	1.30	0.00
1833.00	2070.00	1938.67	1.09	0.05
1059.00	1202.00	1153.33	1.07	0.18
26.00	22.00	26.00	1.01	1.00
11.00	12.00	14.67	-1.72	0.00
735.00	768.00	774.67	1.07	0.11
58.00	66.00	65.33	2.44	0.00
463.00	538.00	527.33	1.25	0.00
428.00	413.00	437.67	1.03	0.55
734.00	854.00	836.33	-1.02	0.66
194.00	181.00	201.00	1.14	0.08
442.00	426.00	463.67	1.04	0.45
36.00	47.00	42.33	-1.05	0.73
598.00	643.00	661.00	1.12	0.03
4.00	1.00	1.67	-1.58	0.59
400.00	382.00	386.67	1.08	0.20
124.00	137.00	142.67	-1.25	0.00
498.00	468.00	503.33	1.10	0.09
887.00	979.00	952.67	1.13	0.01
49.00	41.00	48.67	-1.26	0.07
68.00	65.00	67.67	-1.11	0.35
20.00	16.00	19.00	1.48	0.07
1013.00	1124.00	1112.33	-1.02	0.65
441.00	406.00	434.00	1.03	0.56
7202.00	7216.00	7474.67	1.19	0.00
124.00	140.00	151.33	1.01	0.97
511.00	586.00	576.00	1.08	0.13
49.00	43.00	52.67	1.21	0.17
8.00	8.00	11.67	-1.10	0.74

154.00	162.00	172.67	1.00	0.98
201.00	247.00	231.67	1.12	0.10
5728.00	6183.00	6287.00	1.28	0.00
76.00	79.00	73.67	1.45	0.00
34.00	34.00	32.33	-2.28	0.00
19.00	31.00	29.33	-1.11	0.54
318.00	357.00	353.00	-1.10	0.07
17.00	18.00	17.00	-1.35	0.16
30.00	58.00	43.33	1.07	0.71
245.00	252.00	247.00	-1.15	0.03
481.00	415.00	424.33	-1.54	0.00
29.00	29.00	29.00	-1.32	0.08
909.00	966.00	931.67	-1.10	0.04
35.00	45.00	42.00	1.08	0.63
8.00	13.00	11.67	2.43	0.00
330.00	280.00	309.33	1.37	0.00
1382.00	1412.00	1466.67	1.15	0.00
348.00	350.00	386.00	-1.19	0.01
184.00	193.00	202.67	-1.15	0.04
85.00	108.00	101.00	1.31	0.01
547.00	542.00	551.67	1.00	0.98
1082.00	1257.00	1205.33	1.13	0.01
208.00	223.00	210.33	1.08	0.36
5.00	7.00	6.33	-1.07	1.00
991.00	958.00	979.00	-1.12	0.01
4.00	16.00	11.67	1.11	0.81
298.00	381.00	357.67	1.24	0.00
683.00	695.00	694.33	-1.34	0.00
1320.00	1292.00	1299.33	-1.27	0.00
1030.00	939.00	984.67	-1.07	0.20
551.00	514.00	541.33	-1.06	0.25
1572.00	1725.00	1623.33	1.14	0.00
8.00	12.00	10.67	1.13	0.71
7.00	5.00	6.33	1.04	1.00
294.00	359.00	332.00	1.14	0.07
420.00	474.00	459.33	-1.04	0.46
289.00	350.00	353.33	1.05	0.55
27.00	30.00	32.33	1.54	0.02
474.00	594.00	543.33	-1.15	0.01
2.00	4.00	3.67	-1.99	0.09
14.00	27.00	20.00	1.02	1.00
753.00	831.00	829.33	-1.04	0.41
452.00	496.00	479.67	1.11	0.04
5.00	0.00	3.33	-1.11	1.00
261.00	283.00	304.00	1.22	0.01
85.00	118.00	104.00	1.23	0.04
83.00	88.00	89.33	1.08	0.44
23.00	8.00	14.00	-1.37	0.20

7.00	6.00	8.67	1.07	0.89
26.00	14.00	21.67	2.27	0.00
290.00	365.00	342.00	1.41	0.00
1420.00	1576.00	1522.67	1.01	0.87
64.00	65.00	66.00	1.20	0.11
1.00	7.00	4.33	-2.53	0.01
155.00	163.00	169.67	1.10	0.20
57.00	44.00	48.00	1.02	0.87
345.00	364.00	363.33	1.01	0.90
204.00	203.00	202.67	-1.48	0.00
547.00	614.00	564.33	1.07	0.27
107.00	122.00	126.67	-1.10	0.28
164.00	172.00	177.67	1.14	0.09
27.00	20.00	21.00	1.63	0.02
22.00	19.00	21.33	1.37	0.12
56.00	62.00	63.00	-2.02	0.00
7.00	7.00	8.33	1.17	0.67
271.00	323.00	309.00	1.22	0.01
120.00	113.00	124.00	-1.01	0.92
489.00	523.00	520.33	1.06	0.25
258.00	330.00	299.33	1.26	0.00
88.00	124.00	114.67	1.01	0.95
13.00	14.00	14.33	-1.20	0.49
319.00	343.00	334.67	1.19	0.00
30.00	29.00	26.00	-1.06	0.83
327.00	284.00	315.67	1.22	0.01
190.00	229.00	216.67	1.53	0.00
2.00	5.00	5.00	-2.66	0.00
1337.00	1462.00	1426.67	1.14	0.00
587.00	655.00	634.67	1.02	0.72
1352.00	1624.00	1469.00	-1.17	0.00
757.00	810.00	792.67	1.11	0.03
1233.00	1420.00	1376.67	1.38	0.00
517.00	574.00	560.33	1.13	0.04
574.00	562.00	551.67	-1.27	0.00
665.00	661.00	676.00	-1.17	0.00
1492.00	1519.00	1519.00	-1.03	0.50
15.00	20.00	20.00	-1.60	0.01
29.00	36.00	35.00	-1.30	0.06
189.00	232.00	211.00	1.10	0.18
798.00	763.00	834.67	1.03	0.65
1352.00	1381.00	1405.67	1.00	0.91
2783.00	2722.00	2733.00	1.20	0.00
270.00	298.00	301.00	-1.45	0.00
61.00	58.00	57.67	1.01	0.96
4.00	4.00	4.33	-1.92	0.08
340.00	392.00	373.67	-1.01	0.82
24.00	32.00	34.33	-1.02	0.95

49.00	25.00	29.00	3.74	0.00
22.00	33.00	26.33	1.20	0.34
29.00	28.00	30.67	4.47	0.00
3826.00	4528.00	4416.00	1.10	0.03
2879.00	2581.00	2772.00	-1.10	0.04
277.00	294.00	291.33	-1.04	0.58
403.00	498.00	477.33	1.19	0.00
512.00	488.00	520.33	-1.01	0.81
53.00	78.00	74.33	-1.19	0.13
501.00	547.00	543.67	-1.05	0.34
946.00	990.00	1003.67	1.03	0.44
2077.00	2304.00	2284.67	1.15	0.00
111.00	112.00	118.00	-1.08	0.38
13.00	13.00	14.33	-1.46	0.09
167.00	195.00	179.00	1.02	0.84
14.00	23.00	18.00	-1.41	0.08
886.00	992.00	963.33	1.32	0.00
7.00	15.00	14.67	-1.14	0.68
1501.00	1576.00	1586.33	1.10	0.01
138.00	172.00	157.33	-1.10	0.22
9.00	6.00	7.33	-1.42	0.29
142.00	149.00	147.67	-1.12	0.18
47.00	47.00	48.00	1.09	0.53
58.00	70.00	67.67	-1.11	0.34
418.00	457.00	467.00	1.22	0.00
629.00	845.00	806.67	1.10	0.16
121.00	134.00	127.33	-1.01	0.90
463.00	416.00	456.33	1.10	0.10
309.00	348.00	352.33	1.15	0.05
1564.00	1652.00	1702.00	-1.01	0.74
23.00	29.00	23.33	-1.06	0.88
1095.00	1033.00	1139.67	1.12	0.03
142.00	186.00	178.00	1.10	0.29
502.00	601.00	579.33	1.24	0.00
19.00	24.00	21.00	-1.43	0.04
38.00	34.00	32.33	-1.12	0.56
22.00	20.00	23.67	-1.06	0.75
2.00	3.00	5.00	-1.68	0.17
135.00	162.00	140.33	-1.06	0.54
441.00	523.00	488.33	1.23	0.00
12.00	11.00	14.67	-1.40	0.11
11.00	8.00	12.00	-1.15	0.66
1997.00	2080.00	2107.33	1.01	0.86
540.00	542.00	536.33	-1.08	0.18
1032.00	1139.00	1123.33	1.15	0.00
157.00	185.00	183.67	1.16	0.06
252.00	235.00	262.67	-1.10	0.18
116.00	105.00	113.67	-1.10	0.28

940.00	912.00	968.33	1.12	0.01
22.00	9.00	18.00	-1.05	0.93
79.00	85.00	84.33	-1.16	0.12
293.00	354.00	319.00	-1.20	0.00
21.00	15.00	16.00	1.35	0.21
63.00	61.00	61.67	-1.22	0.12
139.00	160.00	164.33	-1.13	0.13
300.00	315.00	314.33	-1.02	0.74
16.00	10.00	15.67	1.08	0.76
399.00	567.00	487.33	1.13	0.10
1420.00	1626.00	1513.33	1.03	0.51
181.00	175.00	180.67	-1.00	1.00
1388.00	1376.00	1372.67	-1.07	0.12
202.00	220.00	222.33	-1.45	0.00
131.00	148.00	147.67	-1.15	0.09
7.00	6.00	5.67	-1.30	0.55
36.00	34.00	32.67	1.04	0.79
507.00	566.00	525.33	1.07	0.26
831.00	922.00	824.00	1.07	0.33
3514.00	3677.00	3850.33	-1.29	0.00
291.00	270.00	302.33	-1.20	0.00
406.00	452.00	456.00	-1.13	0.03
124.00	107.00	111.00	1.36	0.00
94.00	122.00	106.67	-1.15	0.14
115.00	116.00	119.00	-1.56	0.00
1020.00	1055.00	1010.00	-1.45	0.00
893.00	912.00	902.67	-1.07	0.15
16.00	15.00	18.00	-1.13	0.59
1285.00	1473.00	1410.33	1.13	0.00
1927.00	2311.00	2260.00	1.35	0.00
5544.00	5443.00	5507.00	-1.13	0.00
8.00	6.00	6.33	-1.64	0.13
12.00	24.00	19.33	-1.14	0.55
401.00	374.00	396.67	1.13	0.07
38.00	36.00	36.33	-1.11	0.50
1143.00	1155.00	1162.33	-1.42	0.00
1584.00	1824.00	1779.33	1.06	0.22
67.00	77.00	66.67	1.30	0.07
3957.00	4340.00	4135.00	1.22	0.00
21.00	19.00	19.67	-1.17	0.49
2417.00	2643.00	2601.00	1.22	0.00
11620.00	13996.00	13319.67	-1.11	0.01
2330.00	2460.00	2477.33	1.16	0.00
274.00	262.00	277.33	-1.24	0.00
7.00	15.00	11.67	-1.42	0.17
16778.00	17174.00	17450.67	-1.08	0.07
14789.00	14885.00	15062.67	-1.04	0.25
410.00	444.00	448.67	1.07	0.19

20.00	12.00	16.67	-1.15	0.58
747.00	787.00	792.00	1.20	0.00
97.00	94.00	95.33	-1.09	0.43
356.00	346.00	368.67	-1.08	0.20
1279.00	1338.00	1355.33	1.02	0.71
484.00	545.00	548.00	1.02	0.68
24.00	32.00	30.33	1.34	0.11
104.00	97.00	102.33	1.14	0.17
304.00	298.00	294.00	-1.16	0.02
1202.00	1257.00	1237.00	1.04	0.36
99.00	124.00	129.00	1.01	0.96
1274.00	1199.00	1285.67	-1.00	1.00
1597.00	1529.00	1537.00	-1.07	0.19
1616.00	1719.00	1653.67	-1.02	0.65
274.00	374.00	370.33	1.07	0.44
4724.00	4652.00	4905.67	-1.21	0.00
2612.00	2885.00	2692.00	1.01	0.73
2925.00	2888.00	2970.33	-1.07	0.12
25400.00	27588.00	26609.33	-1.15	0.00
6999.00	7284.00	7414.67	1.04	0.21
609.00	668.00	672.67	-1.18	0.00
18.00	21.00	19.00	1.86	0.01
2718.00	2796.00	2837.67	-1.06	0.12
672.00	602.00	656.33	-1.13	0.02
172.00	168.00	164.67	-1.28	0.00
8.00	10.00	10.00	-1.61	0.06
2772.00	2557.00	2740.33	-1.08	0.07
11032.00	11414.00	11455.33	-1.11	0.00
108.00	101.00	106.00	-1.14	0.14
286.00	240.00	270.33	-1.48	0.00
630.00	664.00	650.33	-1.15	0.00
3643.00	3793.00	3881.33	1.11	0.00
3.00	1.00	1.33	-1.24	1.00
6587.00	6804.00	6753.33	1.10	0.01
4540.00	4548.00	4732.00	-1.01	0.80
131.00	146.00	158.67	-1.45	0.00
51.00	70.00	65.33	-1.07	0.58
4291.00	4823.00	4870.00	1.21	0.00
1249.00	1478.00	1438.67	1.22	0.00
2078.00	2046.00	2174.00	-1.05	0.27
1510.00	1437.00	1520.67	-1.06	0.17
112.00	128.00	130.67	-1.31	0.00
2650.00	3256.00	2974.33	1.31	0.00
117.00	132.00	126.33	-1.00	1.00
45.00	22.00	30.67	-1.54	0.02
277.00	330.00	294.33	-1.06	0.39
289.00	258.00	304.33	1.40	0.00
960.00	994.00	1023.67	-1.08	0.13

1905.00	1899.00	1922.00	1.06	0.18
370.00	377.00	377.67	1.22	0.01
254.00	265.00	265.33	-1.04	0.50
1146.00	1186.00	1202.33	1.01	0.72
93.00	84.00	80.33	1.29	0.03
5.00	4.00	3.67	-1.28	0.69
4.00	3.00	2.67	-1.86	0.22
26.00	34.00	36.00	-1.01	1.00
2751.00	2833.00	2825.33	-1.02	0.63
2060.00	2114.00	2064.67	1.01	0.75
352.00	382.00	347.00	1.12	0.13
34.00	35.00	32.33	1.35	0.07
478.00	566.00	530.67	-1.06	0.29
384.00	401.00	394.33	1.03	0.62
566.00	519.00	534.33	1.04	0.53
53.00	69.00	63.67	1.06	0.67
8.00	8.00	9.33	-1.44	0.19
935.00	995.00	969.00	1.06	0.22
570.00	650.00	643.67	1.35	0.00
1939.00	2002.00	2055.00	-1.02	0.62
13.00	11.00	8.00	1.40	0.52
104.00	87.00	95.67	1.06	0.57
319.00	345.00	336.67	-1.10	0.09
736.00	917.00	838.33	1.01	0.81
700.00	775.00	729.00	-1.04	0.40
232.00	215.00	232.33	-1.04	0.55
122.00	146.00	131.00	1.90	0.00
3264.00	3558.00	3449.67	1.06	0.12
357.00	366.00	399.00	1.09	0.17
192.00	170.00	198.00	1.09	0.26
109.00	143.00	130.00	1.00	1.00
26.00	16.00	21.33	-1.17	0.46
28.00	35.00	31.33	-1.02	0.89
7.00	5.00	4.67	1.25	0.70
13972.00	14950.00	14594.67	1.32	0.00
1065.00	1226.00	1142.00	1.25	0.00
269.00	240.00	259.00	-1.26	0.00
926.00	939.00	935.33	-1.18	0.00
1872.00	2190.00	2119.33	1.36	0.00
1650.00	1630.00	1574.00	-1.15	0.01
830.00	857.00	851.33	1.07	0.22
662.00	768.00	720.67	1.09	0.07
67.00	68.00	60.00	-1.15	0.31
984.00	1074.00	1110.67	1.12	0.02
76.00	92.00	83.33	1.62	0.00
22.00	21.00	19.67	1.06	0.79
495.00	652.00	566.00	1.21	0.00
207.00	200.00	198.67	1.04	0.66

251.00	347.00	334.67	1.06	0.47
244.00	297.00	267.00	-1.05	0.55
48.00	84.00	57.33	-1.11	0.56
312.00	333.00	316.33	-1.03	0.66
154.00	179.00	168.00	-1.04	0.60
1161.00	1162.00	1182.67	-1.14	0.00
209.00	201.00	208.67	1.21	0.04
4869.00	5509.00	5451.00	1.23	0.00
39.00	35.00	30.67	-1.02	0.95
565.00	616.00	601.67	1.01	0.86
2563.00	2693.00	2586.00	1.15	0.00
2376.00	2332.00	2397.67	1.08	0.04
304.00	298.00	308.67	-1.11	0.09
141.00	149.00	145.00	1.17	0.06
448.00	439.00	483.00	1.25	0.00
1340.00	1362.00	1382.33	-1.09	0.03
1248.00	1295.00	1331.00	-1.18	0.00
569.00	563.00	562.67	1.08	0.15
3557.00	4149.00	3899.00	-1.01	0.71
1914.00	2037.00	2042.33	1.01	0.88
3935.00	4072.00	3955.67	-1.23	0.00
561.00	594.00	628.33	-1.01	0.85
840.00	939.00	956.33	1.12	0.02
203.00	208.00	222.67	1.05	0.46
290.00	308.00	312.00	1.07	0.26
34.00	40.00	36.33	1.21	0.25
22.00	19.00	23.00	1.54	0.04
224.00	263.00	259.67	-1.05	0.48
203.00	225.00	224.00	1.08	0.31
208.00	266.00	244.00	1.57	0.00
15.00	17.00	14.67	1.49	0.11
296.00	369.00	328.33	1.35	0.00
198.00	202.00	205.00	-1.07	0.33
301.00	377.00	372.67	-1.10	0.17
6.00	4.00	7.00	-1.35	0.41
310.00	371.00	359.67	1.14	0.03
381.00	462.00	438.67	1.20	0.00
147.00	211.00	180.33	1.05	0.61
18.00	20.00	18.33	1.50	0.07
119.00	132.00	126.33	1.16	0.10
996.00	1080.00	1118.67	1.13	0.01
17.00	17.00	21.00	1.26	0.34
160.00	191.00	180.00	1.09	0.23
331.00	361.00	336.33	-1.04	0.54
851.00	994.00	957.00	-1.18	0.00
84.00	59.00	75.00	-1.04	0.81
559.00	513.00	542.67	-1.41	0.00
1424.00	1524.00	1508.33	1.10	0.01

182.00	161.00	183.67	1.24	0.01
4089.00	5084.00	4743.00	-1.05	0.24
376.00	427.00	394.67	-1.27	0.00
19.00	15.00	18.00	-1.57	0.01
26.00	35.00	29.67	-1.59	0.00
110.00	135.00	134.00	-1.34	0.00
15.00	20.00	17.00	1.92	0.01
200.00	163.00	176.33	-1.38	0.00
183.00	187.00	191.67	1.36	0.00
360.00	407.00	403.33	-1.07	0.35
6.00	5.00	7.00	-1.49	0.22
145.00	148.00	144.67	1.30	0.00
0.00	0.00	0.00	-27.86	0.00
14.00	10.00	14.33	-1.29	0.28
43.00	36.00	42.00	1.22	0.20
151.00	127.00	141.33	-1.22	0.08
167.00	158.00	174.00	-1.33	0.00
144.00	168.00	169.00	1.09	0.34
4.00	4.00	4.00	-1.42	0.47
45.00	51.00	47.33	1.11	0.47
133.00	131.00	130.67	-1.35	0.00
5.00	8.00	5.33	-1.20	0.74
95.00	81.00	99.33	1.22	0.07
8.00	4.00	7.00	1.29	0.52
468.00	468.00	485.67	-1.08	0.15
67.00	84.00	76.67	-1.01	0.93
600.00	695.00	665.00	-1.02	0.72
94.00	119.00	108.00	1.25	0.03
10.00	6.00	9.67	-2.68	0.00
33.00	34.00	39.00	-1.50	0.00
310.00	310.00	299.33	-1.02	0.79
545.00	530.00	578.67	-1.01	0.84
67.00	98.00	95.67	1.08	0.54
565.00	568.00	580.33	1.08	0.10
290.00	340.00	331.33	-1.01	0.89
56.00	65.00	75.00	1.18	0.22
18.00	15.00	14.33	-1.27	0.33
345.00	348.00	351.33	1.29	0.00
132.00	160.00	133.67	1.09	0.51
0.00	0.00	0.67	-1.88	0.71
2.00	5.00	3.67	2.57	0.13
33.00	37.00	35.33	-1.53	0.00
489.00	555.00	538.00	1.14	0.01
5163.00	5584.00	5517.67	1.02	0.47
1821.00	1935.00	1921.00	-1.21	0.00
338.00	397.00	383.33	1.05	0.38
347.00	430.00	408.00	1.14	0.03
1111.00	1047.00	1086.67	-1.20	0.00

542.00	588.00	596.33	1.29	0.00
612.00	788.00	750.67	1.23	0.00
1735.00	1898.00	1839.00	1.14	0.01
274.00	311.00	307.33	1.21	0.00
20.00	26.00	22.67	1.03	0.93
584.00	667.00	643.67	-1.10	0.06
74.00	67.00	77.00	-1.33	0.01
158.00	201.00	178.00	1.15	0.08
3.00	9.00	7.00	-1.77	0.06
388.00	433.00	462.33	1.01	0.86
680.00	766.00	732.67	1.32	0.00
40.00	56.00	48.67	2.18	0.00
7.00	6.00	8.67	1.81	0.09
617.00	716.00	685.00	1.20	0.00
582.00	611.00	629.00	1.05	0.36
63.00	72.00	66.00	-1.04	0.79
8015.00	8619.00	8334.00	1.48	0.00
723.00	830.00	774.00	1.12	0.02
227.00	255.00	261.67	-1.10	0.17
9.00	11.00	8.67	-1.01	1.00
1659.00	1618.00	1705.67	1.81	0.00
453.00	477.00	460.00	1.03	0.54
1934.00	2014.00	2043.33	1.02	0.65
15.00	7.00	11.67	1.63	0.09
1471.00	1571.00	1503.00	-1.07	0.11
75.00	77.00	77.33	1.52	0.00
451.00	409.00	422.67	-1.19	0.01
533.00	566.00	546.00	1.17	0.00
645.00	717.00	723.33	1.12	0.03
577.00	645.00	606.67	1.10	0.07
21.00	22.00	22.67	-1.03	0.93
1721.00	1773.00	1760.67	-1.17	0.00
260.00	282.00	286.33	1.18	0.01
10.00	11.00	10.00	-1.61	0.06
449.00	484.00	484.33	-1.05	0.35
32.00	32.00	33.33	-1.29	0.08
1.00	1.00	0.67	-7.41	0.00
10.00	5.00	7.33	1.27	0.53
87.00	120.00	107.00	1.74	0.00
158.00	177.00	167.67	-1.11	0.17
803.00	880.00	852.67	1.10	0.07
34.00	40.00	41.67	1.28	0.10
642.00	749.00	701.33	1.28	0.00
2681.00	3092.00	2988.67	1.22	0.00
1523.00	1700.00	1736.67	-1.04	0.41
983.00	1136.00	1072.67	1.18	0.00
11.00	18.00	12.00	1.22	0.49
1058.00	1311.00	1240.00	1.22	0.00

13754.00	12964.00	13388.33	1.04	0.35
412.00	457.00	480.00	1.26	0.00
994.00	1138.00	1110.67	-1.03	0.42
122.00	126.00	135.00	1.17	0.09
192.00	260.00	230.67	1.66	0.00
498.00	578.00	551.00	1.20	0.00
1333.00	1477.00	1484.33	1.03	0.57
276.00	286.00	280.00	-1.00	1.00
1226.00	1348.00	1330.33	1.24	0.00
2610.00	2565.00	2689.67	1.08	0.07
446.00	531.00	515.00	1.05	0.42
505.00	455.00	500.67	-1.07	0.25
997.00	934.00	992.67	1.00	0.96
3273.00	4003.00	3779.00	-1.00	0.92
6472.00	6970.00	7076.67	-1.19	0.00
395.00	394.00	394.33	1.03	0.59
1529.00	1606.00	1555.00	-1.01	0.83
1549.00	1968.00	1774.67	-1.03	0.58
14.00	9.00	10.33	-1.69	0.03
2181.00	2423.00	2368.33	1.12	0.00
577.00	601.00	598.67	1.23	0.00
207.00	274.00	225.33	1.02	0.85
469.00	545.00	506.33	1.19	0.00
395.00	428.00	417.00	-1.24	0.00
33.00	35.00	33.00	1.57	0.01
19.00	28.00	23.33	1.23	0.31
21.00	25.00	22.67	-1.68	0.00
791.00	939.00	892.67	-1.07	0.16
155.00	201.00	179.00	1.25	0.01
15.00	19.00	16.67	-1.15	0.58
685.00	746.00	717.67	-1.08	0.10
6370.00	7267.00	7000.67	1.10	0.01
13168.00	14383.00	14360.33	1.14	0.00
8232.00	8808.00	8983.33	1.23	0.00
11731.00	13390.00	13009.33	1.25	0.00
9400.00	10817.00	10442.33	1.13	0.00
24.00	24.00	30.33	1.42	0.07
180.00	286.00	236.00	1.22	0.03
41.00	71.00	54.67	-1.16	0.35
9149.00	10250.00	10046.00	1.16	0.00
7336.00	8180.00	8119.33	1.29	0.00
496.00	570.00	553.67	1.19	0.00
188.00	230.00	215.33	1.26	0.01
23.00	22.00	20.33	-1.05	0.87
2264.00	2375.00	2226.00	-1.06	0.27
7.00	5.00	5.67	-1.77	0.09
7581.00	7476.00	7933.00	-1.10	0.02
2844.00	3217.00	3103.33	1.08	0.03

8384.00	9279.00	8839.33	1.15	0.00
78.00	76.00	87.33	-1.44	0.00
90.00	87.00	83.67	1.02	0.84
129.00	95.00	115.67	-1.23	0.03
10.00	16.00	15.33	1.03	1.00
3.00	10.00	8.00	1.94	0.08
1.00	1.00	1.00	-4.01	0.02
1966.00	2037.00	2066.00	-1.30	0.00
933.00	950.00	978.33	-1.19	0.00
1406.00	1439.00	1499.67	1.01	0.76
828.00	983.00	893.33	1.11	0.06
1784.00	1929.00	1922.33	1.05	0.18
35.00	30.00	31.00	1.31	0.12
2673.00	2516.00	2707.67	1.43	0.00
9.00	3.00	9.00	-1.20	0.62
6.00	16.00	11.67	-1.13	0.78
760.00	773.00	778.00	1.01	0.85
27293.00	28346.00	28215.00	1.13	0.00
3128.00	3544.00	3400.33	-1.12	0.00
3326.00	3456.00	3381.33	1.17	0.00
2174.00	2206.00	2257.67	-1.08	0.04
278.00	338.00	307.00	-1.15	0.09
24973.00	24538.00	25305.33	1.19	0.00
9036.00	8837.00	9293.67	-1.39	0.00
1617.00	1506.00	1562.33	1.11	0.03
37.00	54.00	49.67	-1.09	0.51
107.00	117.00	119.67	2.70	0.00
5541.00	5620.00	5774.33	1.07	0.12
0.00	1.00	1.33	-3.10	0.05
74.00	96.00	84.33	-1.53	0.00
381.00	347.00	383.67	1.95	0.00
986.00	1075.00	1100.33	-1.13	0.01
32.00	28.00	31.33	-1.14	0.38
21.00	26.00	22.00	1.38	0.10
38.00	32.00	34.67	1.21	0.25
1460.00	1262.00	1384.00	-1.04	0.46
188.00	183.00	200.67	-1.34	0.00
146.00	152.00	152.33	1.03	0.68
694.00	681.00	682.67	-1.20	0.00
3096.00	3224.00	3316.00	1.18	0.00
184.00	236.00	193.33	1.04	0.66
398.00	372.00	395.33	-1.01	0.88
1.00	2.00	1.33	-1.94	0.39
1475.00	1783.00	1695.33	1.02	0.70
2772.00	2998.00	2966.67	1.28	0.00
14.00	20.00	17.33	1.65	0.03
880.00	1073.00	992.67	1.05	0.29
356.00	418.00	409.33	1.40	0.00

1646.00	1688.00	1751.33	1.04	0.34
181.00	257.00	227.33	1.32	0.00
145.00	161.00	165.33	-1.04	0.63
2871.00	3150.00	3030.67	1.06	0.18
1251.00	1258.00	1327.67	1.06	0.17
4816.00	5167.00	5251.00	-1.09	0.03
269.00	341.00	321.33	1.03	0.69
7.00	5.00	9.67	-1.26	0.49
628.00	685.00	660.00	1.18	0.00
2285.00	2570.00	2506.33	1.08	0.05
2079.00	2222.00	2145.67	-1.04	0.28
3221.00	3269.00	3272.67	-1.22	0.00
3969.00	3956.00	4155.33	-1.08	0.04
794.00	777.00	826.00	-1.31	0.00
5959.00	6620.00	6418.00	-1.03	0.35
475.00	416.00	458.00	-1.09	0.13
1909.00	1957.00	2008.00	-1.06	0.12
656.00	706.00	686.67	1.13	0.01
433.00	474.00	479.00	1.40	0.00
1746.00	1979.00	1879.67	1.07	0.11
527.00	590.00	578.33	1.29	0.00
1016.00	1101.00	1075.67	1.34	0.00
1109.00	1165.00	1101.33	1.17	0.01
325.00	335.00	335.33	1.18	0.02
508.00	533.00	553.00	1.27	0.00
907.00	1072.00	1053.00	1.41	0.00
1587.00	1748.00	1711.00	1.36	0.00
1295.00	1588.00	1522.00	1.24	0.00
1460.00	1488.00	1531.67	1.11	0.01
944.00	990.00	1020.00	1.22	0.00
41.00	52.00	45.00	-1.31	0.03
2.00	0.00	1.00	-1.61	0.75
264.00	233.00	242.33	-1.13	0.12
2557.00	2557.00	2538.67	-2.01	0.00
8.00	12.00	8.67	-1.24	0.53
86.00	85.00	79.67	-1.03	0.87
3146.00	3524.00	3329.33	-1.18	0.00
961.00	854.00	943.00	-2.32	0.00
6.00	1.00	3.33	-2.09	0.08
1346.00	1293.00	1315.33	-2.52	0.00
28.00	21.00	26.33	-1.78	0.00
132.00	148.00	130.67	-1.45	0.00
1087.00	1009.00	1093.67	-1.09	0.06
1713.00	2109.00	1933.33	1.27	0.00
2054.00	2195.00	2278.00	-1.09	0.06
129.00	113.00	126.33	-1.16	0.07
596.00	635.00	636.00	-1.02	0.73
2437.00	2459.00	2356.33	-1.07	0.21

1700.00	1912.00	1796.67	-1.12	0.01
435.00	447.00	446.33	1.13	0.03
12.00	13.00	10.67	-2.01	0.00
2673.00	2655.00	2800.67	-1.04	0.32
2009.00	2452.00	2238.33	1.12	0.02
44.00	39.00	40.00	-1.03	0.86
657.00	685.00	674.67	1.02	0.65
850.00	1030.00	987.00	1.27	0.00
134.00	91.00	107.67	1.16	0.20
842.00	857.00	873.67	1.13	0.00
439.00	415.00	452.67	-1.05	0.49
4101.00	4064.00	4270.67	1.14	0.00
364.00	421.00	431.00	1.08	0.27
196.00	183.00	193.67	1.09	0.22
9.00	5.00	6.33	-1.48	0.26
1049.00	1225.00	1194.33	-1.06	0.19
1317.00	1389.00	1377.67	1.01	0.76
5301.00	5889.00	5996.00	-1.06	0.21
3693.00	4069.00	3833.00	1.10	0.06
7.00	4.00	4.33	-2.07	0.04
400.00	510.00	466.33	1.17	0.01
851.00	875.00	913.33	1.05	0.26
415.00	415.00	402.67	1.07	0.29
71.00	85.00	80.33	1.32	0.01
21.00	19.00	18.00	1.44	0.11
77.00	78.00	82.00	-1.10	0.31
2.00	6.00	5.67	1.19	0.73
22.00	8.00	12.67	-1.69	0.03
156.00	187.00	187.00	1.37	0.00
529.00	638.00	572.33	-1.20	0.00
3900.00	3989.00	4167.67	1.04	0.33
258.00	290.00	288.67	1.10	0.12
522.00	554.00	592.67	1.21	0.00
315.00	331.00	342.67	-1.58	0.00
101.00	100.00	92.33	-1.34	0.00
80.00	94.00	94.67	1.38	0.00
79.00	82.00	84.33	1.14	0.21
349.00	430.00	440.67	1.44	0.00
59.00	72.00	67.67	1.25	0.06
135.00	137.00	153.00	1.07	0.47
101.00	130.00	123.67	1.06	0.52
343.00	390.00	402.00	1.07	0.32
591.00	691.00	646.67	-1.10	0.04
388.00	379.00	396.67	-1.12	0.05
7.00	5.00	5.33	1.05	1.00
6.00	11.00	7.67	-1.49	0.20
1398.00	1278.00	1337.67	-1.19	0.00
628.00	653.00	664.00	1.29	0.00

5556.00	5918.00	5888.33	1.06	0.09
838.00	914.00	918.67	1.02	0.65
8.00	12.00	12.33	-1.26	0.34
353.00	383.00	402.33	-1.49	0.00
2422.00	2301.00	2431.00	1.34	0.00
38.00	55.00	50.00	1.09	0.56
55.00	59.00	56.33	-1.12	0.39
1721.00	1833.00	1831.00	1.07	0.10
1914.00	2125.00	2098.33	1.06	0.13
1011.00	1085.00	1076.67	1.17	0.00
899.00	943.00	953.00	1.13	0.01
7.00	12.00	9.00	1.16	0.68
15.00	14.00	16.33	-1.26	0.31
225.00	250.00	238.33	-1.08	0.26
19.00	23.00	20.33	-1.33	0.13
3937.00	4189.00	4139.33	-1.05	0.18
49.00	50.00	51.33	-1.30	0.03
113.00	91.00	94.33	-1.59	0.00
531.00	487.00	515.00	-1.74	0.00
6.00	1.00	3.33	-1.89	0.15
60.00	60.00	58.33	-1.44	0.00
324.00	362.00	341.33	1.15	0.02
2576.00	2625.00	2750.33	-1.03	0.51
510.00	496.00	518.67	-1.09	0.07
452.00	395.00	429.33	1.00	0.98
321.00	409.00	363.00	1.13	0.05
937.00	1003.00	984.67	1.07	0.11
3917.00	4329.00	4223.33	1.06	0.09
414.00	523.00	469.33	1.27	0.00
116.00	157.00	143.67	1.31	0.00
662.00	786.00	729.00	-1.12	0.02
431.00	598.00	522.33	1.28	0.00
329.00	374.00	358.67	1.19	0.00
126.00	132.00	139.67	1.38	0.00
892.00	1034.00	988.00	1.28	0.00
654.00	671.00	689.67	1.12	0.06
117.00	117.00	124.00	1.28	0.01
289.00	325.00	323.33	1.21	0.00
1077.00	1067.00	1147.00	-1.03	0.61
592.00	730.00	683.33	1.66	0.00
319.00	354.00	340.00	1.16	0.01
362.00	445.00	442.33	1.27	0.00
909.00	957.00	936.33	-1.07	0.10
109.00	105.00	108.33	1.35	0.00
995.00	1124.00	1075.67	1.08	0.06
768.00	881.00	862.00	-1.01	0.75
246.00	268.00	259.00	1.01	0.92
759.00	792.00	771.33	1.01	0.83

587.00	685.00	620.00	1.15	0.02
1350.00	1501.00	1410.67	-1.18	0.00
326.00	341.00	360.67	1.14	0.03
2526.00	2717.00	2638.00	1.03	0.45
15.00	11.00	14.33	1.01	1.00
1812.00	1856.00	1835.67	-1.16	0.00
16.00	17.00	18.67	-1.19	0.38
64.00	54.00	59.33	1.06	0.65
1083.00	1094.00	1037.00	1.09	0.16
2899.00	2911.00	2890.33	-1.34	0.00
1247.00	1463.00	1383.33	-1.03	0.41
553.00	602.00	602.33	-1.11	0.03
1112.00	1168.00	1119.33	-1.07	0.14
1160.00	1306.00	1224.33	1.08	0.10
438.00	516.00	480.00	1.01	0.90
1119.00	1430.00	1294.33	1.43	0.00
3357.00	3520.00	3604.67	1.05	0.23
247.00	320.00	289.67	1.09	0.24
583.00	639.00	628.67	1.09	0.08
415.00	493.00	446.00	1.01	0.91
349.00	411.00	401.00	-1.01	0.88
130.00	141.00	148.67	-1.02	0.83
229.00	217.00	220.33	1.18	0.02
427.00	450.00	445.33	1.07	0.30
539.00	635.00	597.33	1.09	0.07
29.00	37.00	30.33	1.06	0.72
658.00	755.00	707.67	-1.24	0.00
328.00	408.00	352.33	-1.12	0.11
198.00	245.00	218.33	-1.17	0.02
777.00	967.00	918.33	-1.11	0.04
459.00	542.00	490.00	1.04	0.52
1079.00	1213.00	1181.67	-1.05	0.22
4030.00	3817.00	4066.33	1.01	0.76
1749.00	1880.00	1843.67	-1.12	0.00
13.00	28.00	24.00	1.11	0.70
5408.00	5381.00	5527.00	1.02	0.61
1408.00	1432.00	1489.67	-1.01	0.85
2597.00	2608.00	2595.67	1.17	0.00
651.00	726.00	711.00	-1.04	0.42
376.00	438.00	437.00	-1.14	0.03
710.00	816.00	787.33	1.24	0.00
2.00	7.00	5.00	-1.41	0.42
834.00	843.00	870.67	1.28	0.00
637.00	814.00	753.33	-1.01	0.85
27.00	36.00	29.67	1.13	0.51
811.00	880.00	868.33	-1.16	0.00
202.00	249.00	233.67	-1.31	0.00
13.00	10.00	12.67	-1.73	0.01

809.00	903.00	867.67	-1.32	0.00
71.00	74.00	76.33	1.22	0.07
49.00	54.00	48.67	-1.32	0.02
175.00	205.00	197.67	-1.25	0.00
62.00	57.00	64.67	-1.46	0.00
4.00	3.00	4.33	1.16	0.84
1397.00	1544.00	1411.67	1.13	0.04
1035.00	1174.00	1135.67	1.20	0.00
484.00	531.00	532.33	-1.05	0.33
17943.00	19702.00	20112.33	1.21	0.00
27.00	34.00	27.67	-1.09	0.72
1119.00	1289.00	1233.67	-1.12	0.02
1260.00	1338.00	1340.00	-1.09	0.03
2.00	3.00	3.33	-1.40	0.55
8.00	11.00	11.67	1.11	0.72
3.00	7.00	4.67	-1.08	1.00
2261.00	2647.00	2524.67	1.00	0.92
94.00	95.00	92.33	1.01	0.97
24.00	23.00	24.67	1.20	0.36
133.00	169.00	152.33	1.04	0.64
203.00	193.00	207.33	1.06	0.42
17.00	29.00	24.00	1.13	0.63
8.00	5.00	5.67	1.39	0.49
1102.00	1087.00	1196.00	-1.01	0.77
97.00	131.00	120.00	1.05	0.64
755.00	830.00	809.67	1.11	0.06
250.00	326.00	304.00	1.47	0.00
720.00	715.00	714.00	-1.01	0.92
641.00	723.00	752.67	1.12	0.10
4125.00	4744.00	4749.67	1.15	0.00
1921.00	2165.00	2113.00	1.23	0.00
484.00	455.00	504.33	1.21	0.00
187.00	235.00	243.33	1.20	0.06
266.00	314.00	304.67	1.15	0.04
656.00	705.00	699.33	1.07	0.18
1887.00	2070.00	1943.33	-1.05	0.34
2043.00	2178.00	2194.00	1.05	0.14
2719.00	2774.00	2751.67	-1.28	0.00
5689.00	5728.00	5844.00	-1.01	0.83
6544.00	6713.00	6582.67	-1.18	0.00
7.00	8.00	7.33	-1.15	0.78
1426.00	1462.00	1428.67	-1.32	0.00
1880.00	1957.00	1870.33	-1.01	0.88
2170.00	2349.00	2225.67	-1.17	0.00
1460.00	1664.00	1550.67	-1.11	0.02
412.00	516.00	471.33	1.43	0.00
623.00	637.00	669.00	1.33	0.00
1869.00	1820.00	1850.67	-1.03	0.43

455.00	492.00	527.00	1.11	0.17
41.00	41.00	42.00	2.14	0.00
428.00	534.00	487.00	-1.03	0.64
260.00	303.00	303.00	-1.00	1.00
1747.00	1768.00	1821.00	-1.01	0.83
697.00	653.00	688.67	-1.04	0.44
14.00	11.00	15.00	-1.17	0.56
16.00	35.00	24.00	-1.18	0.35
28.00	17.00	22.33	-1.07	0.81
7.00	13.00	8.33	1.53	0.22
1099.00	1278.00	1149.00	1.13	0.04
767.00	939.00	846.00	-1.10	0.07
1674.00	1636.00	1748.67	1.03	0.43
1135.00	1155.00	1227.00	-1.00	0.96
325.00	350.00	332.00	-1.01	0.96
982.00	1100.00	1084.00	1.12	0.01
644.00	812.00	754.67	1.08	0.15
97.00	99.00	102.67	1.22	0.04
649.00	709.00	713.67	1.09	0.08
1751.00	1823.00	1847.33	-1.04	0.31
1498.00	1712.00	1693.00	1.23	0.00
244.00	221.00	235.00	-1.11	0.18
827.00	893.00	906.00	1.14	0.00
256.00	254.00	256.67	-1.33	0.00
28.00	44.00	33.33	-1.01	0.95
2975.00	3817.00	3453.00	1.12	0.03
939.00	906.00	937.67	-1.14	0.00
3707.00	3530.00	3700.33	-1.34	0.00
2979.00	2982.00	2983.00	-1.36	0.00
902.00	956.00	978.67	1.14	0.00
1253.00	1441.00	1357.67	1.14	0.00
2036.00	2282.00	2197.33	1.00	0.92
3.00	4.00	3.33	-1.21	0.83
26.00	25.00	24.00	-1.10	0.71
40.00	62.00	51.67	-1.65	0.00
967.00	1055.00	1032.67	-1.14	0.00
40.00	41.00	46.00	-1.33	0.02
207.00	228.00	212.33	1.42	0.00
4.00	1.00	2.33	-2.25	0.10
38.00	35.00	40.33	-1.11	0.48
16.00	14.00	19.33	-1.47	0.04
886.00	1013.00	974.67	1.09	0.05
2115.00	2112.00	2102.33	-1.05	0.31
251.00	244.00	253.67	-1.41	0.00
931.00	938.00	945.67	-1.16	0.00
573.00	602.00	592.67	-1.19	0.00
41.00	38.00	35.00	1.16	0.40
3.00	10.00	7.33	1.44	0.34

227.00	217.00	214.00	1.20	0.02
883.00	1085.00	988.00	1.02	0.67
526.00	520.00	520.00	1.01	0.89
1384.00	1376.00	1429.33	1.01	0.82
1905.00	1934.00	2025.67	-1.05	0.20
2404.00	2750.00	2669.67	1.09	0.02
745.00	803.00	787.00	-1.14	0.00
683.00	730.00	725.67	1.06	0.23
2499.00	2644.00	2702.00	1.02	0.54
1133.00	1253.00	1271.67	1.07	0.13
291.00	344.00	350.00	1.48	0.00
1174.00	1191.00	1236.00	-1.01	0.76
1216.00	1109.00	1173.00	-1.27	0.00
2.00	5.00	6.00	1.59	0.28
110.00	147.00	137.33	1.11	0.27
84.00	59.00	70.67	1.84	0.00
0.00	0.00	0.00	-11.76	0.14
6.00	5.00	7.33	-1.06	1.00
315.00	346.00	331.67	1.21	0.00
786.00	817.00	841.33	1.13	0.01
527.00	569.00	565.67	1.04	0.48
4479.00	4749.00	4891.00	1.02	0.68
31.00	16.00	28.33	1.37	0.10
466.00	456.00	462.33	-1.07	0.23
727.00	765.00	769.00	1.14	0.01
492.00	526.00	497.67	1.26	0.00
4.00	6.00	6.33	1.17	0.74
1652.00	1792.00	1697.00	1.06	0.15
3.00	7.00	6.67	-1.16	0.77
1702.00	1620.00	1676.33	1.29	0.00
51.00	38.00	46.33	1.78	0.00
3358.00	3163.00	3305.67	-1.09	0.03
1292.00	1543.00	1399.33	1.28	0.00
1175.00	1227.00	1220.33	1.10	0.02
8242.00	8688.00	8576.00	-1.19	0.00
4696.00	4982.00	4781.33	-1.04	0.35
8.00	13.00	8.67	-2.01	0.01
260.00	288.00	286.33	1.19	0.02
315.00	321.00	322.33	1.05	0.46
788.00	884.00	886.33	1.39	0.00
847.00	950.00	1014.67	1.45	0.00
1567.00	1558.00	1555.67	-1.09	0.04
1138.00	1223.00	1152.67	1.02	0.69
1159.00	1153.00	1189.67	-1.08	0.10
14.00	17.00	17.00	2.62	0.00
1582.00	1740.00	1713.33	1.12	0.01
148.00	137.00	152.67	-2.18	0.00
259.00	246.00	264.67	-1.21	0.01

1854.00	1909.00	1884.00	-1.09	0.02
323.00	336.00	330.00	-1.47	0.00
554.00	551.00	558.67	-1.05	0.35
1124.00	987.00	1050.67	-1.30	0.00
4276.00	3872.00	4245.00	-1.15	0.00
813.00	924.00	919.00	2.30	0.00
712.00	767.00	755.33	1.15	0.01
3629.00	4331.00	3983.33	1.11	0.01
105.00	73.00	100.00	1.13	0.29
837.00	865.00	882.33	-1.37	0.00
5.00	6.00	7.00	2.52	0.03
10.00	7.00	6.67	-1.51	0.23
13.00	25.00	18.00	1.52	0.07
8.00	10.00	9.33	-1.01	1.00
1.00	3.00	2.33	-2.11	0.14
2414.00	2576.00	2512.00	1.04	0.31
6.00	5.00	7.00	-1.11	0.88
3333.00	3394.00	3496.67	1.16	0.00
13.00	20.00	15.00	1.38	0.20
693.00	604.00	666.33	-1.17	0.00
3.00	5.00	2.67	-2.83	0.01
3.00	0.00	1.67	-2.33	0.15
280.00	264.00	289.33	1.04	0.58
640.00	805.00	750.00	-1.26	0.00
6.00	17.00	15.33	1.36	0.26
27.00	29.00	31.67	2.45	0.00
1.00	0.00	1.67	-1.58	0.59
3.00	4.00	2.67	-1.13	1.00
726.00	752.00	767.67	1.18	0.00
330.00	445.00	407.00	-1.08	0.26
5.00	3.00	3.33	-1.50	0.44
24.00	19.00	24.00	1.03	0.94
6981.00	7263.00	7100.00	1.18	0.00
2052.00	2271.00	2183.67	-1.02	0.62
2295.00	2392.00	2264.67	-1.08	0.15
1019.00	986.00	992.00	-1.41	0.00
52.00	36.00	42.00	-1.79	0.00
2506.00	2838.00	2627.67	1.02	0.64
2036.00	2369.00	2289.00	-1.02	0.68
0.00	2.00	0.67	-3.57	0.12
12.00	9.00	12.67	-1.01	1.00
1010.00	1229.00	1150.00	-1.06	0.20
368.00	457.00	443.67	-1.08	0.21
1104.00	1203.00	1178.00	-1.46	0.00
2528.00	2372.00	2362.33	-1.02	0.75
1005.00	951.00	1018.67	-1.12	0.02
926.00	994.00	973.33	1.06	0.23
1015.00	971.00	995.67	1.09	0.05

2051.00	2359.00	2367.33	1.11	0.02
610.00	640.00	617.67	-1.06	0.29
297.00	324.00	347.00	1.05	0.57
506.00	493.00	490.33	1.00	0.97
780.00	731.00	802.67	1.18	0.00
2296.00	2312.00	2339.33	-1.08	0.07
861.00	927.00	948.00	1.05	0.31
732.00	850.00	813.33	1.05	0.33
411.00	407.00	413.67	1.42	0.00
5225.00	5615.00	5415.00	-1.20	0.00
7.00	4.00	6.33	-1.51	0.38
810.00	942.00	936.67	-1.35	0.00
2762.00	2986.00	3059.33	1.12	0.01
618.00	622.00	672.33	1.08	0.18
13028.00	13686.00	13339.67	1.00	0.93
217.00	226.00	223.33	-1.76	0.00
66.00	83.00	78.00	-1.39	0.00
559.00	627.00	612.67	1.15	0.00
4657.00	4570.00	4726.33	-1.07	0.06
11.00	8.00	8.67	-1.13	0.79
34.00	36.00	35.67	-1.15	0.34
65.00	61.00	61.33	1.05	0.70
22.00	19.00	21.33	-1.01	1.00
1110.00	1375.00	1267.33	1.15	0.00
93.00	91.00	99.00	1.36	0.00
552.00	609.00	621.00	1.19	0.00
534.00	576.00	588.33	1.28	0.00
76.00	108.00	105.67	1.08	0.49
2320.00	2465.00	2435.00	1.14	0.00
1844.00	2054.00	2035.67	1.12	0.01
1738.00	2038.00	1970.33	1.23	0.00
54.00	25.00	43.00	1.06	0.77
958.00	861.00	943.00	-1.14	0.01
947.00	875.00	897.33	-1.16	0.01
2558.00	2596.00	2573.33	1.06	0.17
1339.00	1364.00	1454.00	-1.05	0.35
37.00	42.00	40.67	1.00	1.00
1873.00	1927.00	1938.67	-1.16	0.00
1147.00	1389.00	1263.33	-1.03	0.53
12.00	17.00	13.33	-1.49	0.08
8076.00	8802.00	8978.33	1.12	0.00
1896.00	1966.00	1994.67	1.01	0.71
347.00	436.00	409.00	1.09	0.14
18.00	38.00	26.33	-1.68	0.00
22.00	12.00	17.33	-1.28	0.22
2283.00	2660.00	2543.00	1.21	0.00
10.00	12.00	10.67	-1.17	0.64
183.00	222.00	201.67	-1.49	0.00

1085.00	1158.00	1191.67	1.18	0.00
60.00	41.00	50.67	-1.37	0.02
75.00	71.00	72.33	1.15	0.21
202.00	273.00	231.33	1.08	0.32
0.00	4.00	1.67	-2.52	0.10
799.00	775.00	808.33	-1.05	0.25
4908.00	5809.00	5511.00	1.38	0.00
136.00	179.00	157.67	-1.14	0.12
354.00	283.00	319.67	-1.10	0.18
646.00	672.00	675.67	-1.14	0.00
551.00	501.00	520.67	-1.09	0.13
5020.00	4800.00	4848.00	-1.08	0.10
791.00	826.00	849.00	1.12	0.01
1580.00	1774.00	1702.67	1.07	0.10
1333.00	1461.00	1441.00	1.03	0.42
61.00	61.00	59.33	-1.35	0.01
571.00	589.00	610.00	-1.10	0.06
1177.00	1280.00	1239.00	1.09	0.07
398.00	461.00	426.67	1.04	0.49
1070.00	1306.00	1226.33	1.10	0.05
685.00	750.00	737.67	1.04	0.42
957.00	992.00	1028.67	1.03	0.45
1207.00	1339.00	1338.67	1.12	0.01
2618.00	2784.00	2820.33	1.05	0.21
1395.00	1390.00	1434.33	1.07	0.11
230.00	227.00	229.67	-1.11	0.11
16.00	9.00	10.00	-1.98	0.01
1196.00	1306.00	1302.00	1.24	0.00
1681.00	1693.00	1702.33	1.24	0.00
1.00	6.00	3.00	1.26	0.81
372.00	461.00	424.00	-1.03	0.66
38.00	51.00	41.00	1.50	0.01
68.00	73.00	69.33	-1.61	0.00
1570.00	1649.00	1642.00	-1.02	0.65
101.00	114.00	104.67	-1.37	0.00
133.00	156.00	153.33	-1.29	0.00
48.00	62.00	53.00	-1.46	0.00
606.00	752.00	700.67	1.23	0.00
1702.00	1770.00	1787.67	-1.02	0.62
2173.00	2454.00	2415.67	-1.01	0.72
243.00	262.00	290.67	1.31	0.00
814.00	850.00	911.00	1.10	0.07
527.00	621.00	631.00	-1.01	0.86
750.00	871.00	881.67	1.17	0.00
659.00	635.00	657.00	1.04	0.44
2190.00	2265.00	2366.67	-1.10	0.02
175.00	169.00	167.33	1.09	0.27
25.00	25.00	24.00	-1.03	1.00

652.00	703.00	676.00	-1.29	0.00
624.00	683.00	676.67	1.01	0.86
771.00	874.00	856.00	-1.15	0.00
1509.00	1650.00	1595.33	-1.03	0.52
1520.00	1615.00	1599.33	1.06	0.17
502.00	637.00	575.33	1.05	0.46
487.00	539.00	534.00	1.01	0.84
390.00	366.00	368.67	-1.12	0.09
680.00	705.00	706.67	1.10	0.05
16.00	21.00	19.33	1.78	0.01
6.00	11.00	6.67	-1.51	0.22
3157.00	3156.00	2995.67	-2.33	0.00
4153.00	4586.00	4422.33	-1.74	0.00
978.00	992.00	1056.67	-1.02	0.64
174.00	168.00	167.33	-1.27	0.00
3085.00	3171.00	3139.67	-1.05	0.24
28.00	27.00	26.67	-1.19	0.31
11878.00	12840.00	12523.67	-1.80	0.00
2.00	2.00	1.67	-1.58	0.61
3.00	5.00	3.67	-4.66	0.00
5.00	4.00	4.00	-1.51	0.37
67.00	68.00	69.00	1.13	0.26
3749.00	3811.00	3917.00	-1.07	0.04
3.00	0.00	1.33	-3.79	0.01
2032.00	2147.00	2049.00	-1.45	0.00
4759.00	4762.00	4644.33	-1.26	0.00
0.00	1.00	0.33	-3.95	0.23
597.00	585.00	561.00	1.02	0.75
4.00	9.00	7.00	-1.39	0.34
1783.00	1997.00	1915.67	-1.34	0.00
1120.00	1070.00	1089.00	-1.50	0.00
1752.00	1709.00	1684.33	-2.32	0.00
2010.00	2205.00	2107.33	1.08	0.06
17556.00	17873.00	17879.00	1.04	0.26
32751.00	33591.00	33660.67	-1.03	0.42
8976.00	9206.00	9081.00	-1.73	0.00
206.00	219.00	214.67	-1.64	0.00
13.00	19.00	13.33	-1.89	0.00
40.00	44.00	44.33	1.01	1.00
147.00	142.00	148.67	1.12	0.16
13515.00	13854.00	14053.33	1.03	0.37
2589.00	2429.00	2555.67	-1.05	0.26
415.00	403.00	400.33	1.12	0.07
17.00	28.00	24.00	-1.69	0.00
185.00	218.00	205.33	1.04	0.58
316.00	349.00	356.33	1.02	0.80
546.00	617.00	610.67	1.03	0.59
355.00	348.00	380.67	-1.36	0.00

1131.00	1322.00	1345.33	1.11	0.05
415.00	498.00	474.00	-1.01	0.87
966.00	1182.00	1157.67	1.14	0.02
588.00	600.00	599.67	-1.05	0.28
311.00	385.00	361.67	1.24	0.00
340.00	381.00	405.33	1.06	0.45
4224.00	4139.00	4370.00	1.08	0.04
212.00	184.00	198.33	1.13	0.10
7107.00	6834.00	7026.00	-1.13	0.00
4907.00	5527.00	5309.00	1.03	0.33
4382.00	4668.00	4533.33	1.00	0.95
1818.00	1900.00	1979.67	1.05	0.23
3664.00	4031.00	3947.67	-1.05	0.13
1626.00	1766.00	1724.67	1.08	0.04
856.00	866.00	922.67	1.34	0.00
1472.00	1656.00	1590.00	1.16	0.00
1434.00	1596.00	1632.33	1.20	0.00
498.00	580.00	556.33	1.05	0.38
1501.00	1807.00	1760.00	1.13	0.01
2606.00	2745.00	2814.33	1.20	0.00
1799.00	1773.00	1899.33	-1.03	0.49
997.00	1103.00	1070.00	1.01	0.88
1202.00	1355.00	1357.00	1.03	0.55
1970.00	2079.00	2139.67	1.07	0.11
284.00	298.00	305.33	-1.08	0.18
104.00	111.00	112.67	1.10	0.29
567.00	694.00	676.00	1.16	0.01
387.00	357.00	390.33	1.23	0.00
159.00	184.00	193.00	1.38	0.00
905.00	896.00	970.00	-1.02	0.70
548.00	657.00	640.67	1.15	0.02
374.00	420.00	423.67	-1.03	0.71
269.00	266.00	288.00	1.05	0.41
993.00	1129.00	1097.00	1.03	0.41
4.00	10.00	8.33	-1.18	0.70
28.00	16.00	23.00	-1.12	0.59
2235.00	2253.00	2334.67	1.03	0.43
5288.00	5717.00	5513.67	1.10	0.01
36.00	38.00	40.00	1.20	0.22
240.00	274.00	252.00	-2.18	0.00
27.00	22.00	22.33	-1.03	1.00
8321.00	8801.00	9061.67	1.29	0.00
325.00	280.00	306.33	1.09	0.19
280.00	249.00	253.67	-1.09	0.25
636.00	770.00	714.67	1.02	0.76
197.00	180.00	210.33	1.11	0.28
1079.00	1222.00	1192.33	1.07	0.09
152.00	216.00	202.33	1.22	0.03

347.00	430.00	423.67	1.25	0.00
257.00	266.00	268.33	1.06	0.39
628.00	637.00	656.67	1.01	0.78
418.00	465.00	454.33	1.07	0.19
5866.00	6390.00	6435.33	1.18	0.00
1548.00	1697.00	1703.67	1.24	0.00
1885.00	2123.00	2141.00	1.18	0.00
1141.00	1532.00	1394.67	-1.89	0.00
1034.00	1132.00	1198.33	1.11	0.05
0.00	0.00	0.33	-3.22	0.38
2004.00	2174.00	2225.67	1.21	0.00
1421.00	1559.00	1670.33	1.16	0.01
1869.00	1961.00	2066.67	1.10	0.04
1446.00	1669.00	1702.00	1.23	0.00
3560.00	4008.00	4136.00	1.14	0.01
3331.00	3701.00	3772.33	1.16	0.00
10087.00	10483.00	10495.67	-2.52	0.00
4.00	4.00	3.67	-3.33	0.00
2.00	3.00	3.00	-1.55	0.41
15.00	9.00	10.33	1.13	0.70
2601.00	2681.00	2600.00	-1.18	0.00
32.00	28.00	35.67	1.01	1.00
14.00	13.00	12.67	-1.33	0.26
7.00	2.00	5.00	-1.34	0.52
279.00	352.00	309.00	-1.05	0.61
1.00	1.00	0.67	-4.00	0.07
39.00	47.00	40.67	1.35	0.05
265.00	280.00	256.33	-1.20	0.02
1511.00	1452.00	1419.67	1.40	0.00
1.00	4.00	3.33	1.09	1.00
257.00	236.00	237.00	-1.29	0.00
4679.00	4740.00	4982.00	1.00	0.95
383.00	445.00	411.67	1.32	0.00
2154.00	2360.00	2196.67	-1.02	0.66
66.00	59.00	64.00	1.50	0.00
17.00	26.00	20.67	-1.35	0.09
32.00	32.00	33.00	-2.24	0.00
797.00	982.00	918.67	1.21	0.00
1070.00	1174.00	1120.00	1.09	0.05
355.00	352.00	346.33	-1.16	0.01
682.00	706.00	694.33	-1.04	0.47
3274.00	3226.00	3343.33	-1.03	0.40
2378.00	2500.00	2459.67	1.11	0.01
1245.00	1413.00	1389.33	1.16	0.00
2324.00	2161.00	2323.67	-1.03	0.54
1068.00	1090.00	1135.33	1.12	0.01
0.00	1.00	0.67	-3.14	0.19
3049.00	3877.00	3462.00	1.07	0.19

2356.00	2462.00	2473.67	-1.11	0.00
55.00	64.00	60.00	1.54	0.00
55.00	68.00	58.67	-1.23	0.08
31.00	43.00	36.67	1.12	0.53
521.00	553.00	556.67	1.21	0.00
492.00	462.00	523.33	1.18	0.01
515.00	500.00	533.00	-1.27	0.00
9.00	14.00	11.67	-1.21	0.51
1.00	6.00	9.67	1.63	0.31
433.00	362.00	359.00	-1.31	0.01
363.00	366.00	368.00	1.29	0.00
1515.00	1493.00	1500.33	-1.08	0.07
549.00	535.00	548.67	-1.16	0.00
536.00	609.00	598.67	-1.02	0.70
985.00	943.00	952.00	1.06	0.26
1423.00	1652.00	1525.00	1.11	0.03
592.00	555.00	588.67	-1.08	0.13
379.00	349.00	367.67	-2.26	0.00
8769.00	7668.00	8137.33	-1.07	0.19
420.00	389.00	428.33	1.02	0.79
878.00	894.00	872.00	-1.27	0.00
1900.00	1754.00	1784.67	-1.17	0.00
762.00	925.00	860.33	1.10	0.06
596.00	561.00	549.33	-1.12	0.17
2134.00	2757.00	2385.33	-1.06	0.29
355.00	397.00	382.00	1.22	0.00
14.00	11.00	15.33	2.49	0.00
608.00	607.00	630.67	-1.21	0.00
644.00	650.00	676.00	-1.04	0.45
667.00	834.00	789.33	1.09	0.12
7.00	1.00	3.33	-1.11	1.00
58.00	48.00	49.00	-1.31	0.14
5989.00	6419.00	6063.00	-1.36	0.00
6.00	1.00	2.33	1.16	1.00
40.00	75.00	56.33	1.21	0.23
278.00	318.00	311.33	-1.25	0.00
234.00	308.00	282.67	1.04	0.63
300.00	398.00	327.33	1.06	0.48
37.00	37.00	44.67	-4.55	0.00
83.00	91.00	82.00	-1.12	0.38
1539.00	1550.00	1595.33	1.02	0.64
464.00	470.00	491.67	1.19	0.00
902.00	1022.00	1009.00	1.15	0.00
2.00	0.00	1.33	-1.24	1.00
425.00	526.00	473.67	-1.06	0.32
1004.00	1083.00	1073.33	1.21	0.00
1016.00	1013.00	1034.67	-1.23	0.00
641.00	706.00	726.33	-1.07	0.17

287.00	292.00	295.00	-1.16	0.01
308.00	307.00	303.33	-1.12	0.07
35.00	54.00	45.33	-1.25	0.08
8439.00	8369.00	8595.67	-1.05	0.15
358.00	334.00	370.33	-1.35	0.00
1247.00	1250.00	1310.67	-1.17	0.00
727.00	697.00	727.33	-1.12	0.03
6.00	5.00	5.33	1.12	0.86
789.00	937.00	868.33	1.04	0.40
279.00	287.00	290.33	-1.07	0.23
255.00	267.00	268.00	-1.01	0.94
3.00	12.00	6.33	-1.07	1.00
941.00	1037.00	981.33	-1.07	0.14
68.00	76.00	84.33	-1.29	0.06
498.00	513.00	536.00	1.25	0.00
3.00	9.00	6.00	-1.23	0.64
30.00	20.00	23.67	-1.17	0.48
698.00	826.00	759.00	1.16	0.01
369.00	401.00	387.67	-1.02	0.76
3898.00	4115.00	4090.67	1.05	0.18
494.00	555.00	548.67	1.24	0.00
32.00	35.00	36.67	1.08	0.66
12.00	7.00	12.00	1.41	0.22
11589.00	12169.00	11700.00	1.04	0.32
5675.00	6452.00	6196.33	1.15	0.00
129.00	156.00	150.00	-2.02	0.00
1.00	2.00	1.67	-1.77	0.43
0.00	3.00	1.33	-3.10	0.06
281.00	308.00	300.33	1.29	0.00
1.00	1.00	1.00	-1.91	0.51
1284.00	1353.00	1302.67	-1.07	0.12
2054.00	2084.00	2121.67	1.15	0.00
9.00	11.00	11.00	-1.23	0.49
64.00	71.00	69.67	2.45	0.00
4842.00	5095.00	4909.00	1.28	0.00
4631.00	4731.00	4764.00	-1.13	0.00
157.00	110.00	127.33	-1.08	0.49
596.00	561.00	568.67	-1.42	0.00
1809.00	1744.00	1829.67	-1.02	0.67
1228.00	1359.00	1314.67	1.17	0.00
3715.00	3925.00	3869.00	1.09	0.02
999.00	955.00	986.00	1.02	0.69
479.00	434.00	460.67	1.04	0.52
235.00	183.00	212.00	-3.06	0.00
5.00	6.00	4.67	-1.71	0.16
789.00	850.00	831.67	1.01	0.87
253.00	252.00	263.00	-1.61	0.00
720.00	758.00	764.00	1.12	0.04

8490.00	8880.00	9113.33	1.30	0.00
81.00	110.00	102.67	1.43	0.00
609.00	593.00	615.33	1.04	0.37
2721.00	2271.00	2470.00	1.17	0.01
9.00	13.00	12.33	-1.07	0.91
1688.00	1787.00	1866.33	1.08	0.10
1048.00	1043.00	1072.33	1.10	0.03
498.00	607.00	578.33	1.17	0.00
1306.00	1404.00	1390.33	1.11	0.01
1684.00	1790.00	1737.67	-1.08	0.05
19.00	17.00	17.33	1.60	0.04
6.00	8.00	7.00	-1.30	0.48
5.00	8.00	7.67	1.72	0.14
1130.00	1147.00	1176.00	1.00	0.95
0.00	2.00	1.33	-1.01	1.00
7.00	1.00	3.67	1.20	0.83
9.00	5.00	7.67	1.41	0.35
1970.00	1952.00	2068.33	1.03	0.43
189.00	165.00	180.67	-1.00	1.00
1451.00	1566.00	1552.33	1.01	0.77
819.00	880.00	816.33	-1.01	0.86
597.00	647.00	672.00	-1.13	0.02
469.00	472.00	486.00	1.10	0.08
11.00	3.00	5.67	1.05	1.00
1171.00	1144.00	1177.00	1.02	0.60
2767.00	3024.00	3024.00	1.05	0.14
213.00	205.00	204.33	1.02	0.82
915.00	908.00	947.00	1.07	0.10
3267.00	3062.00	3164.00	-1.10	0.03
327.00	375.00	375.33	1.19	0.00
533.00	526.00	522.00	1.19	0.00
6.00	6.00	6.33	1.33	0.49
65131.00	70618.00	69997.67	1.49	0.00
117.00	130.00	127.33	-1.27	0.00
917.00	962.00	951.67	1.05	0.22
1339.00	1437.00	1415.67	1.13	0.00
1164.00	1087.00	1170.33	1.05	0.26
4621.00	4852.00	4758.33	-1.19	0.00
2160.00	2431.00	2372.33	1.17	0.00
5965.00	6420.00	6267.67	1.02	0.60
322.00	343.00	350.00	1.16	0.02
1165.00	1242.00	1245.33	1.10	0.02
51.00	42.00	47.33	-1.45	0.00
13.00	13.00	11.67	1.08	0.82
975.00	986.00	1000.33	-1.17	0.00
2221.00	2414.00	2378.00	-1.09	0.02
1690.00	1905.00	1864.67	-1.02	0.67
1559.00	1736.00	1625.00	-1.04	0.43

26.00	35.00	33.33	-1.52	0.00
6646.00	6793.00	6959.33	-1.06	0.13
31330.00	30445.00	31558.33	-1.03	0.39
3913.00	4327.00	4283.00	1.45	0.00
8152.00	8045.00	8349.00	-1.14	0.00
805.00	794.00	787.67	-1.20	0.00
835.00	843.00	861.00	1.22	0.00
817.00	807.00	851.33	1.22	0.00
4040.00	4118.00	4305.67	-1.07	0.13
92.00	96.00	99.33	1.20	0.05
17.00	35.00	29.33	1.14	0.51
1.00	0.00	0.67	-8.69	0.00
1904.00	1937.00	1948.67	-1.12	0.01
35989.00	36646.00	37559.67	-1.09	0.01
169.00	167.00	175.00	-1.14	0.06
982.00	938.00	955.33	1.06	0.22
282.00	294.00	303.33	1.14	0.05
1009.00	981.00	1023.00	1.07	0.11
102.00	96.00	104.67	1.59	0.00
294.00	297.00	293.00	1.18	0.02
1398.00	1395.00	1452.00	-1.07	0.08
1430.00	1470.00	1546.67	1.07	0.16
2818.00	2983.00	2976.00	1.04	0.35
2490.00	2880.00	2788.67	1.07	0.08
1836.00	2097.00	2028.33	1.10	0.01
2062.00	2094.00	2097.00	1.05	0.26
2356.00	2481.00	2418.67	1.01	0.75
597.00	703.00	645.67	1.10	0.09
1912.00	2005.00	2004.00	-1.14	0.00
1807.00	1695.00	1799.67	-1.28	0.00
2.00	1.00	2.00	-1.81	0.34
455.00	548.00	521.67	1.08	0.16
1458.00	1327.00	1426.00	-1.22	0.00
440.00	498.00	520.67	1.14	0.04
933.00	1019.00	1013.33	-1.01	0.84
698.00	839.00	803.33	1.20	0.00
735.00	768.00	767.33	1.13	0.02
191.00	255.00	227.00	1.10	0.20
1648.00	2113.00	1911.33	2.10	0.00
968.00	1116.00	1075.33	1.60	0.00
939.00	1175.00	1121.67	1.40	0.00
45019.00	46665.00	45599.00	1.84	0.00
23052.00	23538.00	24053.33	2.05	0.00
490.00	664.00	575.33	-1.12	0.07
332.00	386.00	368.67	-1.08	0.19
157.00	167.00	178.67	1.04	0.67
305.00	315.00	329.33	-1.03	0.66
241.00	321.00	289.00	1.25	0.01

246.00	270.00	253.00	-1.06	0.40
1037.00	1183.00	1187.33	1.01	0.86
20.00	23.00	22.00	-1.29	0.16
5.00	9.00	6.67	-1.26	0.56
592.00	587.00	613.00	-1.02	0.62
1033.00	1023.00	1048.00	-1.26	0.00
495.00	510.00	494.00	-1.31	0.00
276.00	289.00	296.00	1.04	0.51
137.00	162.00	163.33	1.33	0.00
1100.00	1208.00	1185.33	1.12	0.01
1135.00	1200.00	1196.67	-1.09	0.02
592.00	621.00	643.33	-1.07	0.15
773.00	714.00	763.33	1.06	0.26
3.00	3.00	3.67	1.20	0.83
2570.00	2396.00	2564.67	-1.15	0.00
368.00	366.00	372.67	1.17	0.01
276.00	198.00	230.33	-1.29	0.00
2228.00	2210.00	2357.67	-1.10	0.09
2846.00	2678.00	2794.00	1.06	0.16
3120.00	3202.00	3281.33	1.14	0.00
3576.00	4405.00	4107.67	1.36	0.00
1531.00	1541.00	1570.33	1.36	0.00
341.00	332.00	336.67	-1.09	0.25
1273.00	1304.00	1334.67	-1.16	0.00
884.00	911.00	890.33	-1.24	0.00
16.00	20.00	18.33	-1.45	0.05
4.00	9.00	5.67	-1.65	0.14
8.00	5.00	6.00	-1.84	0.06
1063.00	1066.00	1070.67	1.13	0.00
22.00	26.00	23.67	1.88	0.00
287.00	274.00	288.00	-1.10	0.14
218.00	223.00	240.33	-1.18	0.02
1.00	2.00	3.33	-1.31	0.69
238.00	272.00	264.67	1.32	0.00
478.00	491.00	518.67	-1.06	0.23
8.00	1.00	4.00	-2.89	0.00
525.00	434.00	477.33	-1.09	0.20
22.00	23.00	22.67	-1.18	0.38
47.00	59.00	72.67	1.24	0.22
513.00	523.00	539.00	1.04	0.37
1546.00	1438.00	1511.33	1.15	0.00
51875.00	56185.00	55901.33	1.00	0.91
22.00	20.00	21.33	1.29	0.20
305.00	345.00	362.33	1.02	0.85
428.00	423.00	426.00	-1.01	0.89
2247.00	2269.00	2344.67	-1.34	0.00
759.00	754.00	771.67	-1.20	0.00
16.00	11.00	13.00	1.66	0.06

722.00	725.00	774.00	-1.03	0.52
134.00	151.00	144.00	-1.36	0.00
3.00	8.00	4.00	-1.09	1.00
839.00	765.00	819.67	-1.89	0.00
493.00	494.00	507.67	-1.08	0.11
29.00	43.00	37.67	1.07	0.67
49.00	73.00	63.67	1.10	0.45
21.00	12.00	14.33	-1.88	0.03
8.00	5.00	5.33	1.93	0.16
2655.00	2520.00	2761.33	1.08	0.14
1549.00	1529.00	1544.33	-1.12	0.01
484.00	494.00	489.00	-1.37	0.00
952.00	1073.00	1048.33	-1.29	0.00
544.00	547.00	572.67	-1.02	0.71
847.00	947.00	974.33	-1.04	0.40
3299.00	3409.00	3530.67	-1.05	0.19
2975.00	3293.00	3307.00	1.16	0.00
8.00	4.00	8.33	-1.13	0.79
1186.00	1220.00	1267.00	-1.10	0.03
2371.00	2704.00	2588.00	1.10	0.02
2785.00	2815.00	2816.33	1.28	0.00
35.00	28.00	33.00	-1.18	0.27
1339.00	1456.00	1478.67	2.04	0.00
23.00	20.00	25.00	1.12	0.64
5063.00	5493.00	5450.00	1.18	0.00
1822.00	1925.00	1939.00	1.10	0.02
789.00	838.00	858.00	1.29	0.00
831.00	893.00	871.00	1.02	0.70
28.00	31.00	25.67	-2.36	0.00
1056.00	1254.00	1247.67	1.27	0.00
8.00	5.00	8.00	-1.14	0.79
5302.00	5285.00	5365.67	-1.24	0.00
139.00	115.00	136.33	1.13	0.17
75.00	57.00	65.33	1.08	0.55
1133.00	1155.00	1188.00	1.18	0.00
17.00	19.00	23.33	1.11	0.63
362.00	390.00	387.33	1.12	0.05
568.00	565.00	575.33	-1.04	0.43
1015.00	1041.00	1048.33	-1.08	0.08
907.00	995.00	1011.00	-1.06	0.21
684.00	743.00	738.00	1.12	0.01
2533.00	2888.00	2827.67	1.19	0.00
6.00	5.00	4.67	1.25	0.70
970.00	1040.00	1039.67	1.10	0.03
817.00	935.00	901.67	-1.08	0.08
920.00	1016.00	956.33	-1.10	0.09
550.00	574.00	593.00	1.01	0.90
117.00	98.00	111.33	-1.11	0.23

1267.00	1373.00	1371.67	-1.17	0.00
1026.00	1070.00	1103.00	1.14	0.00
3242.00	3218.00	3207.67	-1.03	0.51
4086.00	4570.00	4430.67	-1.14	0.00
9.00	9.00	13.00	1.13	0.74
608.00	557.00	623.00	-1.09	0.14
792.00	789.00	790.00	-1.10	0.04
26872.00	26397.00	26390.67	1.09	0.03
216.00	156.00	175.33	-1.11	0.34
20.00	6.00	12.67	1.10	0.74
698.00	666.00	682.67	-1.27	0.00
18.00	17.00	19.67	1.26	0.30
374.00	450.00	411.00	1.32	0.00
1.00	0.00	2.00	-1.65	0.46
233.00	226.00	230.67	1.32	0.00
680.00	792.00	756.00	1.23	0.00
401.00	410.00	414.00	1.13	0.02
633.00	750.00	699.33	1.12	0.03
783.00	898.00	840.00	-1.05	0.34
281.00	330.00	315.67	-1.12	0.04
727.00	746.00	739.00	1.00	0.93
126.00	132.00	133.67	1.24	0.01
10.00	14.00	14.33	-1.08	0.76
1833.00	2064.00	2032.67	1.08	0.09
4437.00	4613.00	4638.67	-1.08	0.02
2344.00	2614.00	2577.67	-1.00	0.97
526.00	518.00	573.00	1.14	0.04
1557.00	1616.00	1644.00	1.04	0.44
1063.00	1058.00	1095.00	-1.16	0.00
299.00	322.00	320.00	1.19	0.00
384.00	432.00	416.67	1.05	0.39
827.00	922.00	862.00	1.21	0.00
251.00	309.00	304.00	-1.20	0.01
224.00	226.00	227.33	-1.01	0.89
887.00	1082.00	1001.00	1.01	0.86
3685.00	4233.00	4157.00	1.22	0.00
820.00	819.00	888.00	1.02	0.69
725.00	758.00	764.33	-1.14	0.00
3880.00	4211.00	4194.33	1.29	0.00
5063.00	4907.00	4980.00	-1.07	0.09
283.00	313.00	308.67	1.28	0.00
1167.00	1406.00	1306.00	-1.18	0.00
1202.00	1316.00	1271.67	1.03	0.45
44.00	60.00	51.67	-1.27	0.05
267.00	320.00	294.67	1.19	0.01
996.00	1169.00	1197.67	-1.12	0.04
1577.00	1767.00	1744.33	1.07	0.09
28.00	36.00	35.67	1.88	0.00

14.00	17.00	15.67	1.05	0.84
10988.00	10678.00	11196.00	-1.05	0.18
36.00	26.00	32.67	-1.52	0.00
3277.00	2774.00	2858.00	-1.09	0.22
1176.00	1187.00	1212.67	1.14	0.01
3269.00	3875.00	3721.33	1.14	0.00
992.00	1083.00	1068.33	-1.38	0.00
2012.00	2109.00	2091.67	1.10	0.01
62.00	61.00	68.00	1.04	0.79
62.00	74.00	77.33	-1.08	0.45
0.00	0.00	0.00	-11.75	0.13
947.00	972.00	972.67	-1.03	0.51
12094.00	12767.00	12729.67	-1.03	0.32
3175.00	3408.00	3366.00	1.14	0.00
821.00	839.00	818.00	-1.01	0.85
524.00	515.00	537.33	1.35	0.00
844.00	988.00	933.67	1.08	0.11
9595.00	10076.00	9634.67	1.11	0.03
1939.00	2076.00	2046.33	-1.00	0.95
1.00	1.00	1.67	-1.01	1.00
204.00	268.00	247.67	-1.15	0.04
2334.00	2672.00	2497.33	1.07	0.09
298.00	349.00	341.67	1.09	0.17
1390.00	1455.00	1455.67	-1.05	0.18
1948.00	2330.00	2218.33	1.22	0.00
1.00	4.00	2.33	-1.43	0.63
9060.00	9627.00	9390.67	1.01	0.79
2.00	5.00	3.67	1.20	0.83
1522.00	1654.00	1629.67	-1.03	0.45
4721.00	5158.00	5065.00	1.01	0.87
2455.00	2811.00	2628.33	1.10	0.03
1440.00	1503.00	1499.00	1.09	0.03
827.00	879.00	901.33	1.24	0.00
27146.00	31617.00	29894.00	1.06	0.08
1579.00	1732.00	1732.00	1.19	0.00
808.00	848.00	864.67	-1.12	0.01
112.00	99.00	110.00	-1.04	0.66
12.00	13.00	14.67	-1.13	0.63
2268.00	2156.00	2243.33	-1.04	0.35
1208.00	1374.00	1361.67	1.06	0.19
3034.00	3329.00	3258.67	1.03	0.40
674.00	745.00	705.00	1.21	0.00
442.00	482.00	479.33	1.06	0.29
2246.00	2317.00	2320.33	-1.05	0.19
135.00	142.00	135.00	1.24	0.01
109.00	155.00	138.33	-1.27	0.00
477.00	495.00	490.33	1.03	0.61
1229.00	1335.00	1376.67	1.13	0.01

801.00	872.00	867.00	1.03	0.47
278.00	228.00	272.00	-1.08	0.35
2235.00	2309.00	2405.33	-1.12	0.00
2427.00	2403.00	2488.33	-1.07	0.10
3441.00	3769.00	3583.33	1.32	0.00
463.00	422.00	466.33	1.06	0.37
162.00	204.00	173.67	1.14	0.13
0.00	0.00	0.33	-3.22	0.39
466.00	449.00	479.33	-1.44	0.00
42.00	51.00	44.00	1.03	0.91
1069.00	1203.00	1215.33	-1.01	0.85
605.00	608.00	626.00	-1.11	0.07
2329.00	2199.00	2329.67	-1.25	0.00
722.00	714.00	713.33	-1.12	0.02
1661.00	1637.00	1639.00	-1.17	0.00
675.00	655.00	645.67	-1.15	0.02
512.00	576.00	540.33	1.04	0.54
21.00	13.00	18.67	-1.18	0.49
2779.00	3254.00	3131.67	1.17	0.00
1573.00	1877.00	1718.67	1.24	0.00
4.00	3.00	4.33	1.27	0.68
297.00	399.00	343.67	1.33	0.00
20.00	14.00	21.00	1.13	0.60
338.00	311.00	339.00	-1.01	0.86
168.00	192.00	188.67	1.20	0.02
36.00	38.00	39.00	1.21	0.24
574.00	651.00	654.00	1.25	0.00
2650.00	2449.00	2557.67	-1.05	0.25
1503.00	1631.00	1623.33	1.01	0.88
1590.00	1682.00	1697.00	-1.11	0.00
1530.00	1687.00	1688.33	1.17	0.00
211.00	255.00	236.67	-1.01	0.93
481.00	504.00	538.33	1.03	0.66
1205.00	1136.00	1125.33	1.21	0.00
368.00	368.00	377.67	1.01	0.92
1809.00	1873.00	1860.67	-1.07	0.11
73.00	72.00	75.67	-1.20	0.07
125.00	174.00	139.00	-1.13	0.22
806.00	808.00	841.33	-1.09	0.05
2651.00	2499.00	2648.00	1.05	0.26
24.00	34.00	27.67	1.15	0.50
332.00	367.00	363.33	-1.14	0.02
1737.00	1843.00	1825.33	-1.09	0.03
426.00	386.00	427.33	1.15	0.02
1285.00	1443.00	1397.00	-1.02	0.58
365.00	409.00	394.33	-1.15	0.02
1166.00	1093.00	1124.67	-1.33	0.00
123.00	164.00	139.33	-1.06	0.51

46.00	79.00	65.33	-1.75	0.00
786.00	866.00	778.67	-1.19	0.01
43.00	45.00	48.67	-1.23	0.13
473.00	424.00	486.67	-1.26	0.00
961.00	1032.00	1051.00	-1.02	0.71
1127.00	1293.00	1304.67	1.19	0.00
2441.00	2349.00	2416.67	-1.26	0.00
14113.00	14714.00	14683.00	-1.10	0.01
581.00	609.00	622.67	-1.15	0.00
2.00	3.00	2.67	-1.50	0.52
238.00	331.00	293.67	1.17	0.04
330.00	307.00	326.33	1.21	0.00
461.00	499.00	499.00	1.19	0.00
1336.00	1397.00	1408.67	1.02	0.56
290.00	308.00	324.33	-1.08	0.20
74.00	58.00	75.67	1.30	0.03
115.00	136.00	132.00	1.49	0.00
3.00	2.00	2.00	-1.17	1.00
132.00	166.00	165.67	-1.23	0.01
162.00	168.00	172.00	1.19	0.03
249.00	243.00	256.33	1.03	0.66
92.00	116.00	106.67	1.40	0.00
1846.00	1848.00	1914.33	1.21	0.00
328.00	396.00	383.67	1.14	0.07
594.00	565.00	570.67	-1.09	0.12
3388.00	3945.00	3703.00	1.11	0.01
1048.00	1142.00	1102.67	-1.06	0.21
2589.00	2661.00	2712.00	-1.00	0.94
1068.00	1135.00	1166.33	1.03	0.60
2642.00	2630.00	2689.00	-1.03	0.41
1224.00	1335.00	1308.00	-1.19	0.00
630.00	643.00	656.00	-1.06	0.23
1236.00	1249.00	1258.67	1.04	0.34
1201.00	1136.00	1204.33	-1.07	0.16
2132.00	2225.00	2188.00	-1.06	0.20
1194.00	1361.00	1327.00	1.02	0.70
991.00	1179.00	1093.33	1.07	0.13
36.00	39.00	40.00	-1.12	0.44
950.00	979.00	936.00	-1.07	0.17
5452.00	6031.00	5827.00	1.12	0.00
707.00	819.00	788.67	1.11	0.03
3246.00	3388.00	3312.00	-1.00	0.99
381.00	419.00	411.33	1.10	0.12
7.00	9.00	5.33	1.13	0.86
436.00	482.00	430.67	1.25	0.00
1772.00	2009.00	1868.67	-1.04	0.40
130.00	156.00	148.00	-1.18	0.04
3787.00	3772.00	3751.67	-1.15	0.00

1239.00	1296.00	1289.67	-1.00	0.97
516.00	596.00	584.33	1.16	0.00
8.00	19.00	13.00	-1.27	0.36
1375.00	1447.00	1439.33	-1.32	0.00
6.00	2.00	3.00	-1.66	0.37
1036.00	1066.00	1021.00	-1.09	0.08
468.00	481.00	489.00	-1.15	0.01
14.00	12.00	11.00	-1.04	1.00
676.00	698.00	722.00	1.00	1.00
1.00	0.00	0.67	-1.44	1.00
1628.00	1759.00	1719.00	1.07	0.12
1141.00	1288.00	1223.33	1.01	0.77
853.00	770.00	829.33	-1.15	0.00
7.00	2.00	3.00	-1.77	0.24
5.00	8.00	6.67	-1.41	0.32
192.00	170.00	184.33	-1.48	0.00
243.00	263.00	251.33	-1.17	0.03
315.00	339.00	325.00	1.21	0.00
3569.00	3924.00	3945.00	1.19	0.00
36.00	27.00	30.67	-1.19	0.29
149.00	157.00	156.00	-1.14	0.10
52.00	80.00	69.33	-1.00	1.00
7837.00	8621.00	8526.67	1.03	0.44
118.00	166.00	152.33	-1.11	0.27
12970.00	12717.00	13093.67	-1.58	0.00
1027.00	1189.00	1125.00	1.13	0.01
4914.00	5021.00	4961.67	-1.00	0.99
1577.00	1777.00	1701.67	1.13	0.00
247.00	272.00	283.00	1.05	0.49
44.00	42.00	47.00	-1.12	0.39
15.00	18.00	15.67	1.25	0.34
2929.00	3220.00	3105.67	1.03	0.36
19.00	30.00	24.00	1.65	0.02
603.00	609.00	613.00	-1.08	0.12
1303.00	1228.00	1306.67	-1.11	0.02
3.00	1.00	1.67	-2.52	0.10
395.00	380.00	393.00	1.03	0.61
290.00	343.00	327.67	-1.09	0.17
2.00	2.00	1.33	-1.01	1.00
2055.00	1991.00	2099.00	-1.32	0.00
3.00	8.00	6.00	3.37	0.01
739.00	844.00	786.00	1.43	0.00
1.00	2.00	1.67	-2.14	0.22
126.00	109.00	124.67	1.38	0.00
5.00	2.00	5.33	-1.51	0.28
2328.00	2464.00	2470.33	1.01	0.70
3.00	2.00	2.00	-1.81	0.34
778.00	850.00	863.00	1.12	0.02

81.00	116.00	99.00	1.45	0.00
11.00	7.00	10.00	2.09	0.03
2.00	5.00	6.00	1.59	0.29
29.00	22.00	28.67	1.51	0.02
626.00	633.00	656.00	-1.01	0.86
50.00	54.00	51.00	-1.24	0.10
13.00	13.00	14.67	-2.07	0.00
1304.00	1234.00	1334.00	-1.20	0.00
50.00	63.00	60.67	-1.16	0.20
2807.00	3294.00	3134.00	-1.08	0.05
5.00	2.00	4.33	1.58	0.39
999.00	812.00	894.67	-1.20	0.00
693.00	721.00	694.00	-1.13	0.02
1087.00	1262.00	1132.67	-1.03	0.63
498.00	586.00	553.67	1.22	0.00
413.00	434.00	429.33	1.04	0.47
12.00	6.00	10.00	-1.08	0.91
1602.00	1732.00	1709.33	-1.01	0.88
150.00	117.00	139.00	-1.67	0.00
534.00	427.00	489.67	-1.45	0.00
20.00	24.00	19.67	-1.35	0.13
595.00	709.00	677.00	1.00	0.94
223.00	193.00	202.33	-1.15	0.07
348.00	338.00	329.00	-1.22	0.00
40.00	46.00	37.00	1.17	0.40
10.00	18.00	13.33	1.04	0.91
3624.00	4347.00	4037.33	1.12	0.00
1855.00	2110.00	2099.33	1.09	0.05
3.00	1.00	3.00	-1.45	0.53
1361.00	1291.00	1425.00	-1.09	0.06
2506.00	2652.00	2651.00	1.01	0.90
2067.00	1974.00	2114.33	1.16	0.00
6.00	8.00	7.33	-1.51	0.19
622.00	772.00	676.33	-1.07	0.29
655.00	658.00	670.33	-1.02	0.73
253.00	293.00	283.67	-1.26	0.00
181.00	147.00	162.33	1.14	0.13
6571.00	6786.00	6908.67	1.21	0.00
598.00	644.00	598.33	-1.04	0.54
1268.00	1352.00	1335.00	-1.09	0.02
6048.00	6327.00	6064.67	-1.00	0.99
2261.00	2386.00	2363.00	1.07	0.06
7.00	7.00	7.67	1.72	0.14
2707.00	2813.00	2749.67	-1.01	0.77
1266.00	1243.00	1283.67	-1.07	0.08
304.00	396.00	370.33	1.17	0.02
951.00	963.00	969.67	-1.17	0.00
237.00	266.00	279.00	1.09	0.24

834.00	805.00	823.67	-1.03	0.59
674.00	842.00	818.33	1.14	0.03
1255.00	1473.00	1396.67	1.09	0.05
934.00	1086.00	1047.67	1.15	0.00
340.00	297.00	323.33	1.06	0.38
441.00	540.00	517.67	1.14	0.02
257.00	281.00	272.00	1.11	0.11
133.00	169.00	156.00	-1.16	0.05
1180.00	1129.00	1145.33	-1.04	0.38
161.00	176.00	181.33	1.08	0.31
62.00	80.00	65.67	-1.12	0.36
838.00	833.00	876.33	-1.07	0.15
2346.00	2421.00	2443.67	-1.01	0.82
3.00	10.00	6.67	2.40	0.04
216.00	166.00	195.67	1.00	1.00
1744.00	1933.00	1931.33	1.10	0.02
1796.00	2054.00	2087.00	1.15	0.00
1120.00	1272.00	1301.67	1.01	0.86
3.00	8.00	4.67	1.25	0.70
246.00	282.00	274.00	-1.13	0.04
237.00	228.00	261.00	-1.00	1.00
181.00	174.00	171.00	-1.20	0.05
377.00	378.00	406.33	1.06	0.37
73.00	74.00	76.00	-1.22	0.04
1006.00	973.00	1020.00	-1.14	0.01
88.00	116.00	105.00	1.18	0.09
1358.00	1316.00	1368.00	-1.03	0.54
1663.00	1622.00	1680.67	-1.38	0.00
227.00	231.00	226.33	1.07	0.35
1250.00	1296.00	1273.33	-1.16	0.00
2414.00	2700.00	2624.33	1.11	0.01
12.00	18.00	16.33	-1.20	0.40
2829.00	2792.00	2911.33	-1.07	0.06
26.00	27.00	25.67	1.26	0.19
6934.00	7010.00	7091.67	-1.13	0.00
3.00	3.00	2.33	-4.17	0.00
4525.00	4643.00	4659.00	1.13	0.00
394.00	436.00	417.33	-1.24	0.00
202.00	214.00	206.00	-1.23	0.00
924.00	930.00	967.00	-1.04	0.38
844.00	941.00	974.67	1.27	0.00
869.00	1020.00	1006.67	1.14	0.01
1778.00	2004.00	1940.33	1.04	0.33
10.00	4.00	6.33	1.10	0.87
2233.00	2209.00	2166.33	-1.08	0.11
2012.00	2145.00	2028.33	1.01	0.82
331.00	336.00	342.33	-1.29	0.00
184.00	158.00	160.33	-1.57	0.00

1389.00	1411.00	1428.00	-1.52	0.00
2889.00	3081.00	2878.00	1.02	0.69
1396.00	1489.00	1401.67	-1.08	0.13
826.00	827.00	834.00	-1.23	0.00
1873.00	1750.00	1743.67	1.06	0.30
781.00	734.00	769.00	-1.62	0.00
5.00	11.00	7.67	1.19	0.65
388.00	436.00	421.00	1.06	0.27
114.00	128.00	130.33	1.07	0.51
3.00	1.00	3.00	-1.23	0.83
158.00	167.00	176.00	-1.31	0.00
27.00	35.00	33.00	-1.04	0.85
14.00	23.00	15.00	-1.05	0.93
4.00	3.00	3.33	-2.87	0.00
609.00	696.00	665.33	-1.09	0.08
659.00	620.00	650.33	1.01	0.93
378.00	446.00	431.33	1.01	0.94
393.00	422.00	423.67	-1.24	0.00
554.00	491.00	515.00	-1.07	0.30
1599.00	1579.00	1629.00	-1.08	0.05
2268.00	2434.00	2431.00	-1.14	0.00
308.00	340.00	338.00	1.21	0.00
66.00	73.00	67.00	-1.11	0.36
2170.00	2194.00	2248.33	-1.02	0.66
372.00	392.00	387.67	-1.13	0.03
733.00	650.00	707.67	1.02	0.70
1263.00	1267.00	1329.67	-1.02	0.59
658.00	719.00	733.67	1.11	0.04
1309.00	1411.00	1451.00	1.09	0.07
238.00	275.00	268.00	1.03	0.64
6.00	23.00	15.67	1.21	0.49
1183.00	1208.00	1264.00	-1.11	0.02
1174.00	1458.00	1382.33	1.19	0.00
911.00	996.00	1020.00	1.08	0.15
310.00	303.00	309.33	1.15	0.05
1534.00	1539.00	1575.00	1.02	0.55
2726.00	2643.00	2837.33	-1.06	0.14
519.00	565.00	524.33	-1.15	0.03
6344.00	6308.00	6449.67	-1.08	0.02
12.00	11.00	10.33	-1.21	0.56
12.00	4.00	8.00	-1.01	1.00
3422.00	3695.00	3488.00	-1.19	0.00
81.00	85.00	95.00	-1.23	0.03
579.00	813.00	693.67	-1.03	0.62
159.00	203.00	183.67	1.39	0.00
173.00	200.00	192.67	-1.00	0.96
7.00	1.00	5.33	-1.01	1.00
31.00	64.00	45.33	1.05	0.78

1245.00	1393.00	1316.33	-1.15	0.00
1182.00	1400.00	1292.33	-1.05	0.29
637.00	733.00	705.67	1.24	0.00
397.00	400.00	408.67	1.15	0.02
0.00	2.00	1.67	-1.97	0.37
745.00	751.00	783.00	-1.05	0.36
1317.00	1357.00	1333.00	-1.28	0.00
15.00	13.00	14.33	-1.06	0.92
1295.00	1440.00	1386.67	1.12	0.00
3.00	4.00	2.33	-1.29	0.81
1182.00	1066.00	1137.00	1.05	0.28
1300.00	1304.00	1331.67	1.11	0.01
8783.00	9543.00	9658.33	-1.31	0.00
16.00	11.00	14.00	-1.80	0.00
2.00	7.00	4.33	-1.62	0.25
0.00	2.00	1.67	-2.33	0.15
1061.00	1048.00	1122.67	1.10	0.05
899.00	908.00	966.00	1.00	0.98
1983.00	2084.00	2034.33	-1.09	0.02
70.00	56.00	62.00	-1.33	0.01
55.00	48.00	47.67	-1.03	0.87
0.00	0.00	1.33	-1.73	0.60
265.00	320.00	295.00	1.68	0.00
1188.00	1080.00	1144.00	1.01	0.81
3.00	6.00	5.33	1.12	0.86
36.00	42.00	38.67	-1.10	0.53
977.00	940.00	954.33	-1.05	0.30
352.00	329.00	328.33	-1.00	0.97
735.00	849.00	798.33	1.05	0.34
10604.00	11499.00	10964.67	-1.20	0.00
2297.00	2763.00	2646.33	1.16	0.00
80.00	80.00	82.33	1.12	0.29
514.00	522.00	518.33	-1.27	0.00
1105.00	1223.00	1242.33	1.13	0.01
313.00	362.00	352.00	1.09	0.17
718.00	798.00	771.67	1.31	0.00
639.00	699.00	676.00	-1.01	0.81
212.00	234.00	238.33	1.20	0.01
382.00	434.00	438.00	1.21	0.00
463.00	558.00	555.67	-1.02	0.74
67.00	73.00	65.33	1.17	0.18
141.00	119.00	138.00	-1.18	0.05
35.00	37.00	34.67	-1.09	0.57
1345.00	1467.00	1543.67	-1.28	0.00
549.00	536.00	555.67	-1.29	0.00
1142.00	1287.00	1281.33	1.18	0.00
3077.00	3287.00	3376.67	-1.07	0.12
455.00	498.00	505.67	1.05	0.45

1074.00	1073.00	1142.67	1.08	0.09
489.00	594.00	567.00	1.12	0.04
4328.00	4716.00	4638.67	1.27	0.00
172.00	179.00	177.67	-1.49	0.00
396.00	434.00	409.00	1.18	0.00
785.00	917.00	884.00	1.09	0.06
9.00	7.00	9.00	-1.12	0.80
409.00	429.00	419.33	-1.24	0.00
88.00	81.00	85.00	1.30	0.01
46.00	54.00	55.00	1.20	0.17
21.00	24.00	24.33	1.11	0.62
415.00	498.00	490.33	-1.06	0.32
117.00	123.00	120.67	1.07	0.45
57.00	57.00	62.67	-1.07	0.56
1928.00	1968.00	1967.33	-1.33	0.00
640.00	625.00	627.33	-2.53	0.00
4933.00	5005.00	5200.67	1.12	0.00
1.00	2.00	1.33	-2.40	0.19
3559.00	4319.00	4010.33	-1.22	0.00
988.00	1030.00	1050.33	1.07	0.11
1551.00	1809.00	1756.33	1.15	0.00
8.00	7.00	5.67	1.19	0.73
147.00	137.00	142.33	-1.20	0.02
2829.00	2653.00	2860.67	-1.02	0.59
1649.00	1716.00	1768.00	1.02	0.68
2224.00	2146.00	2252.33	-1.23	0.00
54.00	52.00	53.67	1.19	0.16
605.00	588.00	612.67	1.06	0.29
8.00	2.00	7.00	1.03	1.00
28265.00	27908.00	27575.00	-1.07	0.14
39.00	41.00	44.67	1.10	0.52
1860.00	2150.00	2020.67	1.09	0.04
982.00	1118.00	1138.67	1.16	0.00
2612.00	2946.00	2770.33	-1.05	0.21
3463.00	3750.00	3507.00	-1.13	0.01
442.00	554.00	501.33	1.09	0.17
2341.00	2699.00	2726.00	1.08	0.11
721.00	864.00	815.67	-1.15	0.00
1128.00	1273.00	1336.67	1.07	0.28
639.00	754.00	733.67	1.05	0.35
236.00	376.00	316.33	1.07	0.47
994.00	1094.00	1061.67	-1.03	0.49
129.00	114.00	120.67	-1.11	0.22
655.00	719.00	675.33	-1.21	0.00
558.00	543.00	541.33	-1.11	0.06
360.00	444.00	407.00	1.05	0.44
2.00	0.00	1.00	-1.31	1.00
46.00	48.00	48.67	1.31	0.05

145.00	177.00	165.33	-1.17	0.03
944.00	1083.00	1025.33	1.06	0.22
286.00	341.00	313.67	1.01	0.89
984.00	1148.00	1090.00	1.24	0.00
692.00	723.00	758.33	1.48	0.00
36.00	37.00	38.67	3.16	0.00
1899.00	2097.00	2054.67	1.11	0.01
2291.00	2462.00	2506.67	1.02	0.54
879.00	944.00	942.00	1.15	0.00
454.00	491.00	504.00	1.10	0.07
2980.00	3261.00	3113.67	-1.68	0.00
120.00	165.00	135.33	2.96	0.00
868.00	914.00	939.67	-1.09	0.06
676.00	712.00	704.33	-1.00	0.96
28.00	62.00	45.67	1.84	0.00
475.00	471.00	476.67	1.11	0.03
766.00	762.00	779.33	-1.10	0.03
562.00	599.00	572.67	1.16	0.00
17.00	16.00	15.67	-1.61	0.02
18.00	20.00	17.67	1.24	0.33
12.00	10.00	10.67	-1.64	0.04
4.00	2.00	2.33	1.36	0.78
0.00	0.00	0.33	-2.49	0.64
71.00	106.00	86.67	-1.13	0.25
1834.00	2272.00	2087.00	-1.04	0.39
3915.00	4463.00	4367.67	1.41	0.00
536.00	564.00	571.33	1.03	0.65
946.00	1031.00	1027.67	1.19	0.00
1265.00	1268.00	1275.00	1.05	0.23
2561.00	2490.00	2538.67	-1.20	0.00
146.00	124.00	137.00	1.22	0.02
430.00	508.00	482.67	1.37	0.00
1244.00	1438.00	1399.67	1.22	0.00
603.00	680.00	668.67	-1.05	0.38
153.00	110.00	130.00	1.26	0.02
1289.00	1446.00	1460.00	-1.20	0.00
564.00	599.00	607.33	1.17	0.00
7771.00	8822.00	8689.67	1.09	0.01
364.00	357.00	392.33	1.52	0.00
699.00	776.00	776.67	1.50	0.00
577.00	523.00	580.67	1.11	0.06
1969.00	2262.00	2096.33	1.40	0.00
1.00	2.00	1.00	-2.21	0.35
10.00	11.00	11.33	1.02	1.00
306.00	315.00	323.33	-1.33	0.00
1059.00	1109.00	1119.33	-1.08	0.04
1944.00	1924.00	1980.00	-1.09	0.02
3052.00	2993.00	3067.33	1.02	0.62

1563.00	1644.00	1607.67	-1.01	0.78
2333.00	2524.00	2379.33	1.02	0.60
2711.00	3005.00	3022.33	1.12	0.01
17.00	24.00	18.00	-5.86	0.00
2951.00	3094.00	3104.33	1.60	0.00
52.00	49.00	54.67	1.42	0.01
135.00	143.00	139.67	1.09	0.32
3.00	1.00	2.00	-1.33	0.79
1090.00	1179.00	1165.67	-1.12	0.01
1157.00	1264.00	1141.00	-1.01	0.94
345.00	384.00	376.67	1.13	0.03
113106.00	120887.00	122926.67	1.18	0.00
6.00	5.00	7.33	-1.60	0.12
6796.00	7655.00	7842.33	1.19	0.00
15.00	13.00	19.00	-1.07	0.82
10.00	7.00	6.00	1.60	0.29
514.00	608.00	571.67	1.37	0.00
24995.00	26908.00	27305.33	1.15	0.00
66894.00	64842.00	66668.33	-1.01	0.82
1064.00	1059.00	1072.33	1.17	0.00
339.00	403.00	393.33	1.11	0.15
322.00	375.00	368.00	-1.05	0.40
74.00	73.00	83.00	1.58	0.00
173.00	144.00	158.67	-1.00	1.00
2.00	1.00	1.33	-1.94	0.39
185.00	195.00	201.33	-1.22	0.00
1712.00	1720.00	1695.67	-1.02	0.71
119.00	131.00	131.00	1.11	0.20
8.00	14.00	10.00	1.40	0.27
195.00	272.00	232.67	1.16	0.06
1487.00	1716.00	1671.00	1.38	0.00
494.00	488.00	519.67	-1.09	0.12
1226.00	1374.00	1342.33	-1.08	0.05
11.00	15.00	13.00	1.16	0.66
2757.00	2858.00	2923.33	1.24	0.00
1.00	1.00	2.00	-1.49	0.62
28.00	30.00	31.00	1.08	0.72
108.00	96.00	100.67	-1.05	0.59
305.00	397.00	373.00	-1.02	0.82
762.00	795.00	770.67	-1.43	0.00
268.00	265.00	265.67	-1.21	0.00
11.00	5.00	8.00	-1.26	0.51
1841.00	1933.00	1854.00	1.05	0.29
1058.00	1097.00	1081.33	1.05	0.24
3.00	3.00	2.67	-1.74	0.29
3528.00	3830.00	3752.67	1.06	0.08
13.00	11.00	9.33	-2.12	0.00
3.00	8.00	5.33	1.21	0.72

27.00	28.00	29.67	1.03	0.88
8363.00	6619.00	6993.33	-1.39	0.00
1236.00	1345.00	1331.33	1.15	0.00
24.00	20.00	25.00	1.54	0.03
1669.00	1577.00	1711.33	1.18	0.00
23.00	22.00	20.67	-1.03	1.00
1787.00	1832.00	1828.67	1.05	0.19
2493.00	2670.00	2612.33	-1.02	0.71
2561.00	2571.00	2615.67	1.15	0.00
909.00	781.00	849.33	-1.38	0.00
12.00	18.00	17.33	1.14	0.63
2980.00	2866.00	2973.67	1.39	0.00
0.00	3.00	1.33	-1.48	0.76
72.00	106.00	88.00	1.21	0.08
1375.00	1442.00	1406.00	-1.06	0.14
1234.00	1205.00	1246.33	1.17	0.00
2061.00	2466.00	2376.00	1.03	0.56
166.00	203.00	198.00	-1.04	0.54
77.00	83.00	82.33	1.14	0.19
27.00	47.00	34.33	1.19	0.33
13704.00	15438.00	15534.00	1.11	0.01
771.00	708.00	750.00	-1.23	0.00
2121.00	2483.00	2335.67	1.21	0.00
1137.00	1280.00	1291.00	1.12	0.01
11.00	14.00	13.00	1.28	0.35
3009.00	3383.00	3406.67	1.10	0.02
2327.00	2374.00	2384.33	-1.01	0.80
566.00	534.00	520.00	1.01	0.92
487.00	451.00	471.33	-1.11	0.05
2495.00	2531.00	2526.67	-1.05	0.18
1207.00	1239.00	1270.33	1.11	0.01
1070.00	1050.00	1059.67	1.04	0.41
754.00	902.00	855.00	1.23	0.00
750.00	829.00	828.00	-1.11	0.02
1557.00	1567.00	1601.33	-1.08	0.04
1879.00	1946.00	1960.67	-1.01	0.79
2462.00	2821.00	2763.00	1.30	0.00
3717.00	4199.00	4096.67	-1.26	0.00
9848.00	10641.00	10614.33	1.02	0.55
14152.00	14662.00	14023.67	-1.01	0.90
7152.00	7480.00	7524.33	1.10	0.00
6985.00	7588.00	7569.33	1.15	0.00
11636.00	12808.00	12838.33	1.15	0.00
2075.00	2288.00	2310.33	1.15	0.00
3265.00	3557.00	3551.67	1.12	0.00
5269.00	5910.00	5945.33	1.19	0.00
3881.00	4222.00	4344.00	1.10	0.03
1951.00	2291.00	2232.33	1.12	0.01

281.00	287.00	304.33	1.01	0.95
2104.00	2288.00	2335.67	1.21	0.00
5900.00	6225.00	6295.67	1.09	0.02
4822.00	5357.00	5343.33	1.23	0.00
13374.00	15657.00	15124.33	1.02	0.56
2341.00	2447.00	2488.67	1.11	0.00
8764.00	8896.00	8891.33	1.06	0.17
499.00	509.00	523.67	1.19	0.00
922.00	904.00	947.00	1.09	0.05
25.00	25.00	25.67	1.05	0.88
3694.00	3929.00	4025.67	1.19	0.00
1587.00	1581.00	1579.33	1.09	0.04
49.00	52.00	62.00	1.41	0.01
539.00	543.00	544.67	-1.04	0.39
18393.00	18596.00	18615.00	-1.03	0.39
17907.00	19251.00	18938.67	1.03	0.36
3461.00	3560.00	3573.33	-1.11	0.00
5054.00	5052.00	5284.33	1.08	0.03
5156.00	5397.00	5411.33	1.01	0.86
11719.00	12243.00	12723.00	1.20	0.00
296.00	280.00	291.33	1.02	0.80
346.00	357.00	389.67	1.22	0.00
5832.00	6757.00	6534.67	1.19	0.00
4441.00	4620.00	4857.67	1.09	0.04
157.00	192.00	191.67	1.10	0.32
1561.00	1696.00	1698.33	1.01	0.81
2139.00	2045.00	2161.33	1.17	0.00
59.00	76.00	70.00	1.20	0.12
527.00	564.00	550.33	-1.15	0.01
264.00	367.00	324.67	1.05	0.49
292.00	287.00	291.67	-1.20	0.00
2.00	12.00	7.33	-1.06	1.00
63.00	74.00	68.00	1.43	0.00
2041.00	1980.00	2012.00	-1.54	0.00
780.00	766.00	814.33	1.06	0.19
6283.00	6796.00	6591.00	1.24	0.00
1681.00	1862.00	1798.00	-1.15	0.00
726.00	708.00	730.67	1.03	0.52
3470.00	4134.00	3810.33	1.17	0.00
12.00	15.00	14.33	1.06	0.83
843.00	905.00	865.67	-1.01	0.78
100.00	136.00	124.67	-1.12	0.20
160.00	132.00	153.33	1.11	0.24
777.00	961.00	821.67	1.10	0.17
528.00	617.00	610.67	-1.10	0.08
802.00	813.00	795.00	-1.12	0.04
581.00	599.00	643.67	-1.05	0.36
3610.00	3960.00	4003.67	1.07	0.11

290.00	319.00	312.00	1.58	0.00
19.00	26.00	22.00	1.91	0.00
535.00	538.00	551.33	1.05	0.29
4638.00	4939.00	4848.33	1.15	0.00
1471.00	1623.00	1545.00	1.08	0.12
60.00	74.00	65.33	1.07	0.57
1321.00	1396.00	1379.33	1.04	0.27
633.00	654.00	649.67	1.16	0.00
243.00	285.00	274.00	1.09	0.21
1334.00	1433.00	1475.67	1.14	0.00
606.00	740.00	719.00	1.11	0.07
4.00	5.00	4.33	-1.32	0.59
3887.00	4368.00	4086.67	-1.14	0.00
1184.00	1134.00	1155.67	-1.09	0.09
781.00	923.00	900.00	1.18	0.00
931.00	943.00	1019.67	-1.10	0.09
348.00	421.00	417.67	-1.06	0.42
1939.00	1979.00	2090.33	1.23	0.00
582.00	689.00	668.67	1.12	0.03
1980.00	1912.00	1992.67	1.17	0.00
639.00	673.00	686.33	-1.09	0.07
187.00	175.00	202.67	-1.10	0.25
1148.00	1266.00	1319.00	1.10	0.06
691.00	836.00	782.33	1.02	0.63
202.00	187.00	195.67	-1.27	0.00
1036.00	1086.00	1087.33	1.19	0.00
155.00	156.00	165.00	-1.59	0.00
761.00	760.00	770.67	1.10	0.04
82.00	84.00	81.33	-1.18	0.11
843.00	824.00	882.33	-1.16	0.00
118.00	102.00	115.67	-1.76	0.00
1714.00	1779.00	1794.33	-1.12	0.00
1427.00	1486.00	1473.67	-1.01	0.90
73.00	64.00	68.67	1.02	0.86
155.00	136.00	148.33	-1.41	0.00
32631.00	35618.00	35067.00	1.45	0.00
213.00	258.00	249.00	1.31	0.00
1846.00	1874.00	1949.33	1.20	0.00
1687.00	1873.00	1745.33	1.02	0.77
4988.00	5524.00	5429.00	1.05	0.20
718.00	710.00	687.33	-1.21	0.00
164.00	161.00	177.00	1.09	0.36
3.00	7.00	7.00	1.46	0.34
311.00	295.00	317.67	-1.31	0.00
12014.00	11995.00	12291.00	-1.31	0.00
908.00	890.00	931.33	-1.17	0.00
6.00	10.00	8.67	2.51	0.01
495.00	579.00	562.33	1.09	0.13

14.00	17.00	14.67	1.49	0.11
33084.00	34386.00	35228.00	1.04	0.25
3.00	7.00	4.67	-1.01	1.00
1212.00	1354.00	1375.33	-1.05	0.38
596.00	649.00	685.67	1.03	0.61
805.00	938.00	903.33	1.18	0.00
3528.00	3900.00	3839.67	1.05	0.14
83.00	88.00	89.00	1.73	0.00
209.00	209.00	227.33	-1.04	0.54
89.00	124.00	102.33	-1.18	0.08
1741.00	1911.00	1813.00	1.00	0.92
89.00	103.00	96.67	2.00	0.00
3.00	8.00	4.67	1.15	0.85
331.00	406.00	347.00	-1.03	0.71
1728.00	1892.00	1884.33	1.14	0.00
1024.00	997.00	1062.67	-1.12	0.01
81.00	87.00	84.33	1.03	0.80
158.00	153.00	145.33	-3.43	0.00
26.00	32.00	33.00	1.37	0.07
3474.00	3517.00	3519.00	-1.02	0.60
264.00	261.00	247.00	-1.04	0.63
2881.00	2901.00	2975.33	-1.10	0.01
1106.00	908.00	1003.33	1.10	0.13
912.00	1154.00	1111.33	1.09	0.11
1271.00	1448.00	1382.67	1.10	0.02
1739.00	1634.00	1655.67	-1.34	0.00
3.00	5.00	3.67	1.08	1.00
1822.00	1782.00	1794.67	-1.26	0.00
187.00	211.00	200.33	-1.35	0.00
298.00	260.00	281.33	-1.32	0.00
868.00	793.00	824.33	-1.17	0.00
536.00	491.00	529.67	-1.05	0.40
3749.00	4315.00	4035.33	1.14	0.00
1911.00	2007.00	1954.33	1.25	0.00
11.00	13.00	14.67	-1.17	0.51
726.00	741.00	770.67	1.09	0.06
97.00	118.00	120.00	1.12	0.25
441.00	468.00	454.67	-1.06	0.23
713.00	754.00	739.67	-1.09	0.08
507.00	521.00	504.67	-1.02	0.74
1.00	0.00	2.33	1.65	0.60
20.00	21.00	16.33	1.46	0.13
6539.00	6642.00	6632.33	1.07	0.07
3.00	6.00	5.00	-1.34	0.51
1.00	2.00	1.00	-2.21	0.35
13229.00	13380.00	13418.00	-1.25	0.00
3178.00	3342.00	3273.00	1.49	0.00
168.00	184.00	161.33	1.40	0.00

14.00	21.00	20.00	3.43	0.00
30.00	41.00	34.00	1.24	0.20
169.00	141.00	157.33	1.28	0.00
1927.00	1797.00	1883.67	-1.70	0.00
1931.00	1943.00	1965.67	-1.01	0.75
6.00	10.00	7.67	-2.23	0.00
407.00	412.00	433.33	-1.29	0.00
0.00	5.00	3.67	1.08	1.00
269.00	264.00	273.00	1.21	0.01
116.00	119.00	115.67	-1.19	0.04
1063.00	1220.00	1140.67	-1.22	0.00
1897.00	1989.00	1999.67	1.03	0.35
1024.00	998.00	1036.33	-1.26	0.00
295.00	252.00	281.67	-1.15	0.04
1.00	1.00	0.67	-4.86	0.03
9113.00	9900.00	9551.00	-1.03	0.35
1280.00	1414.00	1378.33	1.09	0.04
798.00	888.00	827.00	-1.03	0.61
450.00	481.00	477.00	-1.06	0.30
16.00	10.00	15.33	1.03	0.92
2259.00	2403.00	2284.33	-1.03	0.48
1119.00	1205.00	1223.00	1.08	0.09
2479.00	2653.00	2511.67	1.01	0.86
254.00	290.00	275.00	-1.09	0.18
48.00	61.00	61.67	1.28	0.09
1876.00	2023.00	1985.00	-1.05	0.25
1.00	2.00	2.00	-2.60	0.06
21.00	34.00	25.00	1.09	0.71
1458.00	1409.00	1380.00	-1.17	0.00
2047.00	2131.00	2206.67	-1.11	0.01
1672.00	1546.00	1606.00	-1.33	0.00
1504.00	1512.00	1553.33	1.03	0.46
451.00	500.00	458.67	-1.09	0.17
73.00	75.00	65.67	1.06	0.61
306.00	349.00	313.33	1.04	0.62
428.00	462.00	441.67	1.46	0.00
551.00	551.00	547.00	-1.07	0.21
235.00	277.00	264.00	1.05	0.42
2540.00	2965.00	2671.00	1.37	0.00
1491.00	1378.00	1478.33	1.05	0.23
7954.00	7393.00	7817.67	1.05	0.25
1907.00	1996.00	1982.67	1.12	0.00
5717.00	5761.00	5896.67	1.04	0.33
1798.00	2147.00	2179.33	1.35	0.00
594.00	710.00	652.00	1.16	0.01
483.00	593.00	555.67	1.15	0.01
1160.00	1198.00	1244.00	1.03	0.57
26.00	22.00	30.00	-1.80	0.00

229.00	274.00	254.00	1.08	0.29
187.00	227.00	216.67	1.22	0.00
1.00	0.00	0.67	-3.14	0.19
2.00	5.00	2.33	-1.97	0.20
37.00	73.00	52.33	1.04	0.83
1518.00	1524.00	1541.00	1.03	0.45
3184.00	3145.00	3237.67	1.19	0.00
3671.00	3834.00	3745.00	-1.15	0.00
493.00	465.00	488.00	1.10	0.10
1023.00	1061.00	1052.67	-1.07	0.11
890.00	665.00	708.33	-1.43	0.00
3948.00	4034.00	4010.00	1.16	0.00
288.00	253.00	269.00	-1.15	0.05
6126.00	6627.00	6657.00	1.14	0.00
1680.00	1922.00	1855.33	1.05	0.24
15064.00	16102.00	16224.33	-1.09	0.01
13.00	27.00	20.67	-1.26	0.23
185.00	190.00	197.00	-1.21	0.00
28.00	9.00	17.33	1.39	0.21
77.00	73.00	89.33	-2.28	0.00
359.00	408.00	380.33	1.05	0.43
92.00	110.00	102.33	1.33	0.00
1464.00	1786.00	1745.00	1.11	0.03
1559.00	1767.00	1633.00	1.12	0.01
24404.00	26826.00	26262.33	1.43	0.00
1792.00	1873.00	1839.67	1.18	0.00
8.00	7.00	8.33	1.63	0.16
20.00	26.00	23.00	1.41	0.09
26.00	17.00	21.67	-1.04	0.87
11.00	2.00	6.67	-1.96	0.02
16.00	24.00	21.67	1.68	0.02
1902.00	2144.00	2126.33	-1.06	0.12
16.00	11.00	14.67	1.49	0.11
3449.00	3671.00	3582.67	-1.17	0.00
4692.00	4495.00	4518.33	1.05	0.32
3.00	5.00	3.00	-1.55	0.41
420.00	498.00	493.00	-1.11	0.08
4015.00	4684.00	4480.00	1.19	0.00
1666.00	1830.00	1783.00	1.08	0.06
629.00	628.00	665.67	-1.01	0.82
331.00	409.00	389.00	-1.00	1.00
346.00	433.00	455.67	-1.13	0.16
2140.00	2946.00	2499.67	1.01	0.89
5116.00	5115.00	5123.33	1.01	0.81
1267.00	1313.00	1301.00	1.04	0.39
1125.00	1203.00	1127.00	1.15	0.01
3.00	4.00	5.33	-1.14	0.87
149.00	173.00	148.33	-1.19	0.09

983.00	925.00	951.67	1.22	0.00
2805.00	3001.00	2973.00	1.12	0.01
553.00	589.00	601.67	-1.17	0.00
1842.00	1926.00	1931.00	1.34	0.00
317.00	277.00	316.33	-1.06	0.43
68.00	71.00	70.33	1.02	0.85
4595.00	3442.00	3836.67	-1.54	0.00
606.00	656.00	692.33	-1.06	0.35
176.00	210.00	215.67	-1.07	0.41
689.00	750.00	733.00	-1.01	0.82
542.00	600.00	591.67	1.06	0.28
466.00	454.00	488.33	1.06	0.30
7899.00	8822.00	8697.33	1.08	0.04
2.00	4.00	4.67	-1.09	1.00
711.00	699.00	717.00	-1.18	0.00
373.00	342.00	372.00	-1.31	0.00
316.00	385.00	350.00	1.40	0.00
93.00	95.00	92.33	1.04	0.70
908.00	1051.00	991.00	1.05	0.35
1251.00	1364.00	1324.00	-1.08	0.05
1064.00	1007.00	1055.67	-1.14	0.00
75.00	67.00	79.00	1.06	0.64
1877.00	1773.00	1824.00	-1.05	0.27
1003.00	1213.00	1068.00	1.13	0.04
302.00	344.00	315.00	1.09	0.18
194.00	164.00	167.00	-1.29	0.01
1801.00	1992.00	1968.67	-1.27	0.00
507.00	541.00	529.00	-1.14	0.00
580.00	652.00	619.67	-1.03	0.57
688.00	719.00	790.00	1.08	0.22
2038.00	2187.00	2196.67	-1.08	0.03
1165.00	1259.00	1229.67	1.08	0.05
799.00	900.00	913.67	1.16	0.00
446.00	462.00	495.67	1.03	0.58
507.00	666.00	614.33	1.07	0.27
380.00	400.00	387.67	1.13	0.03
905.00	1017.00	1039.00	1.16	0.01
1213.00	1399.00	1382.33	1.23	0.00
598.00	761.00	683.67	1.13	0.04
109.00	112.00	112.33	-1.21	0.02
4739.00	4741.00	4866.33	-1.20	0.00
2941.00	3085.00	3097.33	-1.18	0.00
2628.00	2872.00	2833.67	1.07	0.10
1718.00	1830.00	1742.67	-1.18	0.00
9.00	9.00	7.33	-1.06	1.00
5.00	7.00	6.33	-1.12	0.88
664.00	711.00	696.00	-1.07	0.16
742.00	855.00	824.33	-1.12	0.01

6.00	14.00	12.00	1.85	0.04
2146.00	2282.00	2297.67	-1.12	0.00
1008.00	1187.00	1158.33	1.37	0.00
3025.00	3549.00	3289.33	1.19	0.00
14.00	13.00	14.67	-1.11	0.79
145.00	154.00	149.33	1.31	0.00
82.00	70.00	77.33	1.07	0.56
2388.00	2547.00	2473.33	-1.71	0.00
483.00	510.00	489.33	-2.21	0.00
163.00	172.00	170.33	1.03	0.72
523.00	546.00	543.33	-1.44	0.00
88.00	93.00	96.00	1.09	0.37
43.00	41.00	47.33	1.09	0.54
8.00	10.00	9.33	-1.55	0.11
0.00	0.00	0.00	-11.74	0.13
913.00	960.00	984.00	1.06	0.21
1192.00	1179.00	1225.67	-1.03	0.42
73.00	91.00	77.00	1.21	0.09
916.00	1026.00	1088.33	2.40	0.00
39.00	42.00	46.33	3.24	0.00
3728.00	3820.00	3943.00	1.15	0.00
3556.00	3349.00	3490.33	-1.03	0.41
977.00	1011.00	1003.33	-1.07	0.13
462.00	466.00	492.67	-1.63	0.00
1717.00	1882.00	1885.67	1.25	0.00
2565.00	2413.00	2523.33	-1.04	0.42
1002.00	1088.00	1121.00	-1.01	0.89
418.00	468.00	450.67	1.04	0.51
599.00	606.00	653.67	-1.15	0.02
337.00	335.00	345.67	1.14	0.02
72.00	89.00	86.33	1.04	0.77
219.00	232.00	235.33	1.02	0.80
13.00	7.00	8.67	-1.40	0.32
692.00	731.00	735.67	1.24	0.00
752.00	657.00	734.67	-1.24	0.00
239.00	254.00	254.67	1.15	0.02
173.00	183.00	180.33	1.09	0.30
646.00	690.00	693.67	-1.03	0.53
182.00	229.00	247.00	1.30	0.02
29.00	33.00	31.67	-1.85	0.00
1.00	0.00	1.33	-2.87	0.08
1.00	0.00	2.00	-2.44	0.08
28.00	31.00	28.33	-1.78	0.00
2.00	5.00	4.33	-1.47	0.39
705.00	817.00	782.00	-1.36	0.00
217.00	203.00	211.67	-1.39	0.00
99.00	95.00	95.67	-1.14	0.27
168.00	162.00	178.00	1.28	0.01

622.00	755.00	718.33	1.28	0.00
115.00	140.00	135.67	1.77	0.00
1260.00	1199.00	1263.33	-1.12	0.00
611.00	617.00	637.67	1.16	0.00
7.00	6.00	7.00	-1.06	1.00
221.00	213.00	218.33	1.14	0.06
373.00	396.00	376.00	-1.04	0.54
243.00	243.00	256.33	1.01	0.87
4530.00	4804.00	4645.67	1.00	0.91
296.00	404.00	378.00	1.12	0.12
350.00	389.00	383.33	-1.05	0.34
287.00	270.00	277.67	-1.14	0.07
931.00	1219.00	1095.00	1.06	0.27
32.00	37.00	35.33	1.34	0.08
36.00	21.00	32.33	1.18	0.34
1278.00	1493.00	1374.00	1.24	0.00
306.00	413.00	356.33	-1.06	0.36
457.00	536.00	538.33	-1.13	0.04
448.00	484.00	488.00	-1.06	0.24
145.00	144.00	150.33	-1.13	0.09
1821.00	1679.00	1705.33	1.24	0.00
10634.00	10806.00	11010.00	-1.13	0.00
7.00	3.00	5.67	1.28	0.59
740.00	786.00	796.33	-1.02	0.71
29.00	23.00	27.67	-1.10	0.61
10.00	11.00	10.00	-1.35	0.29
69.00	71.00	72.33	-1.14	0.20
70.00	93.00	78.67	-1.10	0.49
111.00	100.00	108.33	1.76	0.00
269.00	336.00	320.00	1.08	0.30
13.00	18.00	18.33	1.46	0.10
22.00	14.00	15.00	1.04	0.92
2470.00	2373.00	2468.67	-1.01	0.88
43.00	46.00	44.00	1.11	0.47
1101.00	1073.00	1110.00	-1.08	0.07
414.00	449.00	427.00	-1.02	0.73
1475.00	1709.00	1684.00	1.35	0.00
6.00	6.00	5.67	-1.83	0.07
5.00	6.00	5.67	1.83	0.18
397.00	451.00	419.33	1.00	0.97
27.00	24.00	27.00	-1.45	0.02
671.00	747.00	738.33	-1.14	0.00
22.00	15.00	22.00	1.23	0.34
462.00	468.00	496.00	1.02	0.77
21.00	31.00	25.00	1.04	0.94
259.00	287.00	274.00	-1.03	0.71
22.00	22.00	24.00	1.20	0.36
29.00	26.00	25.00	-1.27	0.17

815.00	820.00	833.00	-1.18	0.00
608.00	699.00	638.00	-1.01	0.87
928.00	931.00	997.00	-1.19	0.00
284.00	337.00	312.33	-1.10	0.10
90.00	108.00	102.33	-1.11	0.28
767.00	876.00	904.33	1.33	0.00
10.00	29.00	16.67	-1.07	0.87
626.00	577.00	596.00	1.06	0.34
6.00	18.00	11.33	-1.87	0.01
1192.00	1346.00	1271.33	-1.02	0.62
3896.00	3928.00	4020.00	-1.01	0.80
120.00	129.00	125.00	1.11	0.23
5625.00	5450.00	5585.33	-1.13	0.00
151.00	117.00	130.67	-1.10	0.33
205.00	172.00	177.67	-1.02	0.89
556.00	644.00	635.00	-1.02	0.71
7.00	5.00	6.00	-1.73	0.09
315.00	282.00	301.33	-1.04	0.52
325.00	368.00	368.67	1.26	0.00
297.00	324.00	298.00	1.26	0.00
484.00	602.00	546.00	-1.01	0.86
565.00	673.00	644.00	1.05	0.34
430.00	488.00	459.00	1.18	0.00
4.00	5.00	6.00	-1.62	0.17
368.00	395.00	369.33	-1.23	0.00
45.00	22.00	31.33	1.41	0.08
16.00	22.00	18.33	-1.36	0.15
148.00	144.00	157.00	1.02	0.80
114.00	137.00	121.67	-1.08	0.40
12.00	24.00	17.00	1.09	0.71
626.00	844.00	748.67	1.23	0.00
9.00	6.00	7.33	-1.01	1.00
11.00	2.00	4.67	-1.28	0.66
1835.00	2119.00	2036.33	-1.03	0.56
534.00	566.00	562.67	-1.06	0.25
1265.00	1384.00	1351.67	-1.07	0.12
465.00	475.00	517.33	1.09	0.22
1664.00	1743.00	1803.67	1.09	0.04
848.00	800.00	781.33	-1.27	0.00
1753.00	1882.00	1797.33	-1.05	0.29
4.00	2.00	3.00	-1.33	0.67
18.00	19.00	21.67	1.28	0.24
223.00	218.00	224.67	1.04	0.54
388.00	493.00	462.67	-1.27	0.00
205.00	238.00	236.00	-1.06	0.39
704.00	703.00	754.00	1.15	0.01
500.00	499.00	532.33	1.09	0.08
531.00	581.00	596.00	1.14	0.02

2750.00	3094.00	2885.00	1.04	0.36
4023.00	4237.00	4123.33	-1.12	0.00
1567.00	1524.00	1554.33	1.04	0.41
1630.00	1727.00	1666.00	-1.54	0.00
537.00	531.00	553.00	1.13	0.02
3532.00	3678.00	3671.00	1.18	0.00
2.00	1.00	2.00	1.44	0.76
14.00	14.00	15.00	-2.35	0.00
0.00	1.00	0.33	-8.37	0.01
1642.00	1678.00	1700.67	-1.03	0.45
277.00	319.00	289.67	-1.26	0.00
538.00	559.00	570.67	-1.20	0.00
827.00	953.00	944.67	1.18	0.00
484.00	466.00	476.00	1.13	0.03
24.00	43.00	32.00	1.10	0.65
739.00	728.00	759.67	-1.04	0.42
1818.00	1940.00	1923.00	-1.17	0.00
1817.00	1806.00	1843.67	-1.15	0.00
1379.00	1434.00	1452.00	-1.20	0.00
4.00	8.00	5.67	-1.66	0.15
397.00	469.00	464.67	1.05	0.49
55.00	84.00	83.00	-1.14	0.30
5.00	7.00	6.00	-1.07	1.00
350.00	328.00	357.00	1.05	0.41
22.00	20.00	22.00	1.38	0.10
14.00	10.00	9.33	1.61	0.25
263.00	303.00	284.00	-1.15	0.02
44.00	57.00	48.00	1.01	0.96
159.00	166.00	163.67	1.02	0.86
80.00	95.00	84.67	-1.08	0.46
162.00	155.00	177.67	1.01	0.89
306.00	342.00	331.00	1.15	0.03
113.00	118.00	119.00	1.36	0.00
68.00	95.00	87.00	1.58	0.00
15.00	12.00	14.67	1.01	1.00
44.00	45.00	46.33	1.32	0.04
905.00	1013.00	1007.67	1.08	0.09
673.00	772.00	726.67	1.08	0.13
13.00	19.00	18.00	1.01	1.00
20.00	8.00	15.33	1.37	0.25
777.00	713.00	775.33	-1.08	0.14
1541.00	1761.00	1638.67	1.29	0.00
480.00	397.00	445.00	-1.14	0.04
330.00	384.00	376.67	1.11	0.11
62.00	63.00	63.67	1.05	0.71
194.00	230.00	206.33	-1.15	0.08
24.00	17.00	23.00	-1.41	0.04
4.00	6.00	5.00	-1.08	1.00

2.00	2.00	1.67	-2.14	0.23
18.00	11.00	11.67	-1.33	0.32
1500.00	1690.00	1618.33	1.23	0.00
1985.00	2123.00	2168.33	-1.05	0.23
233.00	245.00	248.33	1.43	0.00
192.00	162.00	182.33	1.30	0.00
527.00	516.00	558.33	-1.06	0.34
1068.00	1127.00	1115.00	-1.14	0.00
1067.00	1011.00	1022.00	1.10	0.09
267.00	263.00	293.00	1.25	0.00
5.00	2.00	5.67	1.28	0.60
241.00	222.00	229.67	-1.04	0.63
145.00	116.00	123.00	-1.15	0.24
273.00	269.00	278.00	1.29	0.00
2092.00	1955.00	2096.33	-1.28	0.00
11.00	10.00	9.33	1.82	0.07
88.00	98.00	95.00	-1.01	0.97
31.00	38.00	34.00	-1.12	0.44
1.00	0.00	0.67	-1.86	0.69
232.00	196.00	214.00	-1.19	0.02
0.00	0.00	0.00	-22.48	0.01
208.00	273.00	261.67	-1.20	0.01
133.00	141.00	148.33	1.20	0.04
226.00	248.00	257.67	1.32	0.00
41.00	67.00	51.67	1.64	0.00
68.00	58.00	62.67	1.28	0.06
767.00	744.00	729.00	1.07	0.31
1123.00	1223.00	1249.33	-1.19	0.00
115.00	157.00	144.00	-1.10	0.28
565.00	837.00	721.33	1.01	0.89
80.00	89.00	84.00	1.10	0.44
7.00	3.00	4.67	1.25	0.69
202.00	207.00	206.33	1.36	0.00
0.00	4.00	1.67	-1.96	0.31
14.00	5.00	10.67	1.02	1.00
680.00	745.00	732.00	1.26	0.00
1.00	2.00	2.67	-1.01	1.00
601.00	567.00	580.33	-1.20	0.00
108.00	124.00	113.00	2.40	0.00
274.00	265.00	276.67	-1.21	0.00
2.00	5.00	2.67	-1.74	0.29
1.00	0.00	1.33	-2.87	0.08
309.00	339.00	333.33	-1.33	0.00
495.00	497.00	515.33	1.00	0.95
88.00	99.00	99.33	1.20	0.07
312.00	261.00	301.33	-1.38	0.00
198.00	258.00	231.33	1.02	0.77
41.00	46.00	44.33	-1.19	0.19

147.00	147.00	147.67	-1.20	0.04
84.00	77.00	85.67	-1.03	0.75
239.00	243.00	254.33	1.04	0.52
664.00	624.00	680.00	-1.01	0.81
705.00	712.00	698.00	1.19	0.00
1636.00	1955.00	1785.00	1.05	0.36
671.00	799.00	764.33	1.07	0.14
12.00	12.00	10.67	1.05	0.90
474.00	521.00	509.00	-1.05	0.39
778.00	993.00	940.67	1.16	0.01
763.00	779.00	830.33	1.18	0.00
1580.00	1791.00	1702.67	1.09	0.03
1014.00	1118.00	1084.67	1.25	0.00
192.00	252.00	241.33	1.03	0.70
321.00	408.00	394.67	-1.09	0.16
1529.00	1665.00	1632.67	1.05	0.31
64.00	79.00	71.67	-1.06	0.62
390.00	374.00	389.33	1.13	0.04
945.00	1004.00	989.67	1.13	0.01
326.00	355.00	349.33	1.14	0.03
456.00	450.00	491.00	1.12	0.05
1118.00	1186.00	1196.67	1.21	0.00
1575.00	1763.00	1686.33	1.37	0.00
618.00	835.00	760.00	1.19	0.00
273.00	348.00	309.00	1.03	0.67
7.00	12.00	8.00	-1.64	0.09
166.00	205.00	187.33	-1.06	0.49
1898.00	2357.00	2079.00	1.17	0.00
1122.00	1105.00	1129.33	-1.16	0.00
0.00	1.00	0.67	-1.86	0.69
732.00	667.00	729.33	1.03	0.59
148.00	157.00	154.67	-1.30	0.00
236.00	244.00	256.00	1.28	0.00
1740.00	1832.00	1862.67	1.05	0.24
2512.00	2806.00	2740.00	1.03	0.42
97.00	150.00	129.33	1.05	0.66
18.00	17.00	18.33	-1.36	0.11
10027.00	9426.00	9607.67	-1.10	0.02
2292.00	2333.00	2472.67	-1.00	0.95
1045.00	1133.00	1142.67	-1.09	0.05
1909.00	2180.00	2035.00	-1.04	0.37
428.00	462.00	456.33	1.01	0.87
551.00	564.00	577.00	1.02	0.77
1804.00	1914.00	1820.67	-1.21	0.00
1.00	6.00	2.67	1.12	1.00
1280.00	1425.00	1307.33	1.94	0.00
8774.00	9242.00	9596.67	1.09	0.02
52.00	57.00	57.67	1.07	0.61

11.00	16.00	13.33	-1.39	0.17
691.00	680.00	704.67	1.01	0.86
2672.00	3084.00	3134.00	1.27	0.00
987.00	1022.00	1046.33	-1.52	0.00
29.00	22.00	28.00	-1.11	0.57
17.00	25.00	20.67	-1.68	0.00
1669.00	1736.00	1671.33	-1.81	0.00
5371.00	5639.00	5363.67	-1.95	0.00
1928.00	1657.00	1839.33	-1.28	0.00
1158.00	1147.00	1188.00	1.05	0.23
429.00	452.00	463.00	1.01	0.88
12.00	15.00	15.33	-1.23	0.36
422.00	402.00	405.67	1.08	0.21
336.00	324.00	362.00	-1.15	0.02
543.00	556.00	545.67	-1.03	0.61
749.00	766.00	750.00	-1.10	0.08
1053.00	1051.00	1102.67	-1.20	0.00
519.00	503.00	510.33	-1.50	0.00
369.00	430.00	410.00	1.06	0.34
424.00	423.00	437.33	-1.15	0.01
10.00	5.00	8.00	-1.80	0.03
7.00	4.00	6.67	1.16	0.75
647.00	782.00	718.00	1.03	0.60
735.00	749.00	761.33	1.15	0.00
447.00	544.00	511.00	-1.20	0.00
1041.00	1039.00	1089.00	-1.02	0.61
216.00	235.00	232.00	1.09	0.21
242.00	216.00	244.33	-1.39	0.00
515.00	454.00	486.33	-1.07	0.28
1534.00	1730.00	1683.67	1.10	0.02
23.00	21.00	21.67	1.14	0.48
36.00	32.00	37.33	-1.10	0.50
831.00	818.00	871.00	-1.00	0.96
2477.00	2647.00	2652.67	-1.08	0.02
549.00	534.00	518.33	-1.03	0.68
720.00	838.00	808.33	1.11	0.03
17.00	10.00	16.00	1.28	0.30
115.00	98.00	114.00	1.20	0.12
1021.00	1057.00	1046.00	1.05	0.25
346.00	350.00	348.33	1.02	0.70
912.00	980.00	951.33	-1.05	0.32
1397.00	1514.00	1524.67	-1.06	0.13
655.00	665.00	681.33	1.21	0.00
279.00	298.00	289.33	1.13	0.06
601.00	576.00	621.00	1.04	0.46
79.00	81.00	79.67	-1.13	0.26
964.00	1140.00	1056.33	-1.04	0.44
429.00	513.00	474.67	1.41	0.00

832.00	863.00	870.67	-1.11	0.02
629.00	629.00	627.67	1.08	0.11
228.00	276.00	252.67	1.27	0.00
374.00	442.00	412.00	1.05	0.47
664.00	702.00	682.67	1.12	0.02
487.00	462.00	517.00	-1.01	0.89
48.00	71.00	54.67	-1.23	0.10
426.00	551.00	495.67	1.31	0.00
18.00	21.00	23.67	1.93	0.00
1217.00	1200.00	1237.33	1.01	0.73
150.00	196.00	183.00	-1.01	0.92
1195.00	1342.00	1309.33	-1.06	0.15
1379.00	1495.00	1450.00	-1.19	0.00
1633.00	1771.00	1716.67	-1.10	0.02
664.00	739.00	754.00	-1.03	0.49
3.00	3.00	2.67	-2.22	0.08
1787.00	1750.00	1908.33	-1.01	0.90
333.00	347.00	338.00	1.09	0.14
656.00	642.00	642.67	-1.20	0.00
221.00	217.00	236.67	-1.16	0.03
15.00	14.00	15.67	-1.40	0.12
932.00	974.00	984.00	1.12	0.01
5.00	12.00	9.67	-1.22	0.55
450.00	392.00	463.33	-1.14	0.06
12.00	18.00	11.67	-1.35	0.28
691.00	836.00	755.67	1.10	0.07
886.00	949.00	923.00	-1.24	0.00
663.00	743.00	687.33	1.01	0.91
7.00	5.00	6.67	-2.85	0.00
2.00	2.00	2.33	-5.00	0.00
108.00	78.00	96.33	1.01	0.97
983.00	1078.00	1077.33	-1.08	0.05
2485.00	2730.00	2719.67	1.14	0.00
541.00	714.00	636.33	1.11	0.08
359.00	378.00	379.67	-1.00	0.97
201.00	247.00	237.33	1.23	0.00
156.00	170.00	163.67	-1.19	0.01
266.00	254.00	279.00	-1.06	0.36
730.00	830.00	802.67	1.08	0.09
1105.00	1212.00	1154.00	1.02	0.64
643.00	687.00	633.67	-1.04	0.50
958.00	1057.00	1032.67	1.26	0.00
10.00	6.00	7.67	2.03	0.06
634.00	630.00	645.33	-1.07	0.18
44.00	26.00	37.33	-2.40	0.00
5.00	10.00	5.33	-3.30	0.00
1343.00	1289.00	1325.00	1.12	0.01
1472.00	1728.00	1601.67	-1.11	0.02

319.00	368.00	353.33	-1.62	0.00
5.00	3.00	4.00	-1.59	0.29
6.00	7.00	8.00	1.07	0.89
3274.00	3744.00	3651.67	1.49	0.00
610.00	652.00	658.33	-1.04	0.39
8.00	6.00	9.00	-1.31	0.39
2112.00	2284.00	2267.33	1.09	0.02
425.00	485.00	461.33	1.34	0.00
176.00	224.00	204.33	2.24	0.00
10.00	7.00	9.00	-2.20	0.00
139.00	113.00	117.00	1.67	0.00
100.00	112.00	113.67	1.15	0.15
4.00	9.00	5.67	-1.89	0.05
698.00	818.00	725.00	-1.16	0.03
5.00	1.00	3.33	1.22	0.82
36.00	29.00	33.67	-1.27	0.09
2827.00	3177.00	2952.67	1.22	0.00
116.00	114.00	123.33	1.03	0.80
3211.00	3268.00	3267.33	-1.53	0.00
808.00	1080.00	982.67	1.04	0.49
1042.00	1193.00	1133.33	1.18	0.00
18.00	11.00	13.33	1.46	0.16
446.00	427.00	446.00	-1.20	0.00
350.00	378.00	369.33	1.25	0.00
1537.00	1672.00	1702.67	1.15	0.00
35.00	22.00	29.67	-1.10	0.59
37.00	45.00	41.67	-1.31	0.04
62.00	68.00	70.33	-1.31	0.01
910.00	938.00	967.00	-1.24	0.00
2976.00	3086.00	3176.33	-1.18	0.00
244.00	236.00	253.33	-1.02	0.72
1565.00	1545.00	1600.67	1.02	0.58
730.00	742.00	768.00	1.20	0.00
1.00	1.00	1.67	-4.59	0.00
1909.00	2017.00	2072.67	1.07	0.07
104.00	115.00	110.67	-1.20	0.03
555.00	600.00	600.00	-1.18	0.00
364.00	412.00	392.33	-1.26	0.00
912.00	1084.00	1007.33	1.00	1.00
7.00	10.00	7.00	-1.58	0.14
1109.00	1236.00	1213.67	1.10	0.03
173.00	188.00	177.00	-1.39	0.00
647.00	717.00	700.00	1.02	0.67
4.00	1.00	2.67	-1.50	0.51
83.00	74.00	77.00	-1.17	0.14
295.00	266.00	286.67	1.22	0.00
4366.00	4303.00	4347.33	1.14	0.00
12009.00	12549.00	12500.00	-1.20	0.00

2084.00	2192.00	2203.33	1.06	0.18
1212.00	1123.00	1156.00	-1.13	0.01
1587.00	1695.00	1647.00	-1.06	0.15
6398.00	7089.00	7122.67	-1.04	0.38
41.00	45.00	49.67	1.25	0.11
8.00	7.00	10.33	1.09	0.81
1656.00	1679.00	1725.00	1.09	0.08
1391.00	1595.00	1606.00	1.09	0.07
3799.00	3870.00	3992.67	1.12	0.00
579.00	641.00	649.67	1.51	0.00
441.00	543.00	516.67	-1.01	0.86
3875.00	4014.00	4003.67	1.05	0.20
1838.00	1836.00	1808.33	-1.09	0.07
51.00	51.00	45.00	1.17	0.30
664.00	700.00	708.67	-1.29	0.00
1669.00	1688.00	1662.00	-1.06	0.20
1036.00	1146.00	1153.67	-1.05	0.29
1072.00	1015.00	1082.67	-1.16	0.00
282.00	342.00	331.33	-1.10	0.16
876.00	939.00	915.67	1.03	0.49
3584.00	3438.00	3558.67	-1.18	0.00
269.00	333.00	308.00	1.02	0.79
22.00	27.00	27.33	-1.31	0.09
23.00	21.00	20.33	-1.39	0.07
38.00	48.00	44.00	-2.29	0.00
679.00	714.00	700.67	-1.58	0.00
31.00	22.00	23.33	-1.14	0.55
2.00	4.00	2.33	-1.15	1.00
28.00	28.00	29.67	1.02	0.94
4.00	4.00	7.00	-1.92	0.03
34.00	42.00	35.67	1.08	0.65
15.00	31.00	26.67	-1.23	0.24
51.00	69.00	66.00	-1.05	0.69
58.00	56.00	60.67	-1.44	0.00
54.00	77.00	70.33	-1.40	0.00
102.00	108.00	104.33	1.10	0.31
100.00	91.00	94.67	-1.32	0.00
20.00	27.00	27.33	1.04	0.89
307.00	355.00	346.00	1.12	0.06
34287.00	30735.00	31672.33	-1.31	0.00
15634.00	14628.00	14998.33	-1.18	0.00
6674.00	6102.00	6186.33	-2.59	0.00
9.00	18.00	13.67	1.22	0.44
906.00	817.00	876.67	1.02	0.71
651.00	757.00	712.00	-1.01	0.87
8.00	12.00	8.67	-2.05	0.00
52.00	47.00	56.67	-1.15	0.24
901.00	1044.00	965.33	1.00	0.93

21.00	20.00	20.67	1.20	0.36
8.00	4.00	8.00	-1.68	0.08
2447.00	2442.00	2523.67	-1.06	0.17
371.00	370.00	395.67	1.01	0.86
434.00	413.00	390.33	-1.21	0.02
285.00	266.00	276.33	1.39	0.00
898.00	930.00	945.33	-1.84	0.00
3128.00	3527.00	3292.33	1.14	0.00
1898.00	1780.00	1852.33	-1.31	0.00
49.00	61.00	64.67	1.01	0.97
713.00	745.00	730.67	1.03	0.47
82899.00	81936.00	80570.33	1.24	0.00
63.00	65.00	65.67	1.01	0.96
595.00	659.00	631.33	1.08	0.13
932.00	966.00	986.33	-1.09	0.06
783.00	905.00	839.67	1.12	0.02
2967.00	3405.00	3200.33	-1.13	0.00
1657.00	1800.00	1713.33	1.08	0.08
1429.00	1514.00	1417.67	-1.08	0.17
64.00	84.00	73.33	-1.29	0.02
2.00	3.00	2.67	1.12	1.00
1026.00	1060.00	1043.00	-1.10	0.05
167.00	163.00	159.67	-1.07	0.39
1250.00	1337.00	1368.67	1.02	0.57
22.00	22.00	25.33	1.10	0.69
1624.00	1669.00	1672.00	-1.08	0.05
3.00	2.00	2.67	-1.38	0.65
63.00	66.00	65.67	1.32	0.02
29.00	34.00	31.67	-1.19	0.25
95.00	81.00	94.33	-1.25	0.02
12410.00	12555.00	13058.00	-1.10	0.00
3102.00	2815.00	2814.33	-1.09	0.18
23.00	20.00	23.67	-1.10	0.58
644.00	631.00	670.00	-1.42	0.00
328.00	359.00	339.00	1.03	0.65
122.00	124.00	136.67	1.17	0.08
22.00	19.00	26.67	1.23	0.28
4304.00	4480.00	4585.67	1.54	0.00
12.00	17.00	16.00	1.43	0.13
67.00	102.00	83.67	-1.05	0.69
32.00	19.00	27.00	-1.35	0.07
65.00	83.00	70.67	1.14	0.30
18.00	16.00	16.00	-1.47	0.06
5.00	8.00	8.00	-1.26	0.51
529.00	508.00	525.67	2.13	0.00
695.00	691.00	701.33	1.24	0.00
105.00	136.00	123.33	1.09	0.34
12.00	23.00	17.67	-1.20	0.37

700.00	733.00	698.67	-1.22	0.00
2310.00	2658.00	2488.33	-1.08	0.05
2279.00	2379.00	2314.33	-1.25	0.00
2542.00	2506.00	2637.67	-1.00	0.97
1360.00	1333.00	1383.67	1.02	0.71
2394.00	2483.00	2512.67	1.12	0.01
655.00	787.00	732.67	1.21	0.00
548.00	535.00	558.33	-1.26	0.00
30.00	32.00	35.00	1.28	0.15
141.00	174.00	166.33	-1.12	0.14
644.00	669.00	652.67	-1.18	0.00
286.00	308.00	302.33	-1.01	0.83
198.00	176.00	193.00	-1.35	0.00
6.00	6.00	4.00	-1.50	0.40
1589.00	1552.00	1624.00	-1.03	0.46
118.00	124.00	119.67	-1.27	0.00
1.00	2.00	1.00	-1.31	1.00
797.00	815.00	844.33	-1.11	0.03
36.00	33.00	35.00	1.57	0.01
4.00	3.00	3.67	1.52	0.49
630.00	630.00	669.33	-1.14	0.01
937.00	954.00	957.00	-1.08	0.08
1129.00	1132.00	1145.00	-1.05	0.29
251.00	329.00	287.00	1.17	0.03
1023.00	1297.00	1161.33	1.14	0.01
505.00	512.00	487.33	-1.80	0.00
84.00	93.00	97.33	1.09	0.41
300.00	295.00	299.33	1.24	0.00
2.00	2.00	2.33	1.35	0.78
49.00	45.00	45.67	-1.09	0.54
0.00	1.00	0.67	-4.85	0.02
229.00	228.00	235.67	-1.04	0.58
342.00	424.00	361.00	-1.08	0.29
41.00	60.00	55.67	-1.18	0.17
381.00	393.00	379.33	1.07	0.23
81.00	79.00	80.67	1.03	0.77
8.00	9.00	9.33	1.45	0.25
17.00	22.00	22.33	-1.21	0.31
2477.00	2596.00	2619.67	-1.43	0.00
205.00	245.00	235.33	1.18	0.04
827.00	832.00	841.67	-1.23	0.00
4501.00	4790.00	4662.00	1.20	0.00
12.00	18.00	15.33	-1.04	1.00
1167.00	1271.00	1238.67	1.02	0.62
0.00	0.00	0.00	-17.11	0.03
6.00	10.00	8.33	-1.01	1.00
137.00	152.00	146.67	1.05	0.60
423.00	469.00	441.67	1.01	0.86

185.00	168.00	169.00	-1.14	0.18
122.00	121.00	124.67	1.04	0.69
1561.00	1896.00	1675.67	1.03	0.56
17.00	11.00	13.33	-1.19	0.53
2098.00	2012.00	2100.67	1.32	0.00
796.00	791.00	830.67	1.06	0.19
188.00	227.00	204.33	-1.01	0.87
8.00	19.00	16.00	-1.08	0.78
35.00	30.00	32.33	-2.46	0.00
3461.00	3553.00	3551.33	1.41	0.00
5489.00	5629.00	5604.00	-1.05	0.22
296.00	266.00	298.00	-1.63	0.00
6.00	14.00	10.00	-1.68	0.04
36.00	37.00	38.67	1.24	0.18
5954.00	6317.00	6511.67	-1.02	0.60
67.00	55.00	64.67	-1.09	0.50
11503.00	11676.00	12077.00	-1.30	0.00
699.00	666.00	702.67	-1.07	0.13
2.00	3.00	2.67	-2.47	0.04
1105.00	1216.00	1185.67	1.00	1.00
756.00	792.00	814.00	1.21	0.00
2137.00	2416.00	2357.33	-1.06	0.12
34.00	37.00	36.67	-1.12	0.42
3451.00	3809.00	3629.00	-1.06	0.11
1797.00	1948.00	1828.00	1.02	0.62
138.00	141.00	148.00	1.13	0.15
4900.00	5286.00	5224.33	-1.03	0.45
569.00	542.00	561.67	-1.11	0.04
255.00	329.00	300.00	1.25	0.00
347.00	403.00	399.33	1.08	0.26
58.00	69.00	59.67	1.53	0.00
1764.00	1676.00	1724.33	-1.31	0.00
10094.00	11795.00	11342.33	1.14	0.00
1.00	8.00	5.67	1.11	0.87
146.00	125.00	143.67	1.02	0.87
236.00	169.00	188.33	-1.13	0.29
409.00	436.00	419.67	1.00	0.95
4253.00	4289.00	4432.67	1.10	0.01
180.00	186.00	193.33	1.10	0.20
496.00	518.00	518.00	1.28	0.00
270.00	272.00	285.67	1.17	0.01
3893.00	4187.00	4201.33	1.16	0.00
1236.00	1373.00	1337.33	-1.13	0.01
3167.00	3279.00	3303.00	1.18	0.00
2.00	2.00	1.67	1.21	1.00
1292.00	1130.00	1167.33	-1.16	0.02
2287.00	2503.00	2442.33	1.08	0.07
996.00	1072.00	1068.33	-1.01	0.72

319.00	394.00	369.00	-1.15	0.02
1034.00	1115.00	1122.00	-1.06	0.17
370.00	397.00	364.67	-1.06	0.44
194.00	196.00	180.33	-1.16	0.10
187.00	189.00	185.33	-1.02	0.86
2728.00	2874.00	2811.00	-1.01	0.71
287.00	290.00	293.67	-1.23	0.00
790.00	834.00	826.00	-1.10	0.02
7.00	6.00	5.67	-1.19	0.75
800.00	798.00	817.33	-1.09	0.05
6965.00	7338.00	7461.00	1.08	0.07
805.00	978.00	863.00	1.07	0.27
4546.00	5107.00	4754.67	1.20	0.00
1538.00	1653.00	1588.33	-1.22	0.00
2133.00	2121.00	2222.33	1.03	0.51
880.00	909.00	955.00	-1.15	0.00
4455.00	4158.00	4361.67	-1.09	0.05
174.00	195.00	182.67	-1.30	0.00
253.00	242.00	264.33	-1.56	0.00
4.00	6.00	7.67	1.13	0.77
2316.00	2342.00	2557.33	1.09	0.09
483.00	561.00	569.00	-1.05	0.38
865.00	925.00	939.33	1.12	0.01
20.00	25.00	23.33	1.13	0.57
821.00	1113.00	925.33	1.15	0.05
483.00	592.00	591.33	1.22	0.00
853.00	810.00	778.00	-1.57	0.00
126.00	153.00	137.00	-1.17	0.05
24.00	20.00	25.67	-1.34	0.07
90.00	71.00	79.67	1.11	0.34
10.00	17.00	14.33	2.61	0.00
232.00	304.00	287.33	-2.75	0.00
72.00	88.00	78.67	1.82	0.00
31.00	35.00	33.00	1.19	0.30
11.00	5.00	7.67	-1.45	0.24
3.00	3.00	4.00	-1.42	0.47
93.00	90.00	96.00	1.70	0.00
81.00	75.00	84.33	1.13	0.27
1823.00	2059.00	2076.00	1.11	0.01
977.00	971.00	1039.00	1.00	1.00
989.00	1009.00	1020.67	1.19	0.00
1442.00	1378.00	1426.00	-1.22	0.00
39.00	19.00	33.33	-1.33	0.14
218.00	263.00	236.67	-1.39	0.00
1530.00	1614.00	1696.33	-1.10	0.04
395.00	383.00	412.33	1.35	0.00
450.00	480.00	486.67	-1.09	0.08
11.00	5.00	8.33	-1.33	0.37

1285.00	1247.00	1302.00	1.01	0.80
1369.00	1245.00	1299.67	1.18	0.00
1962.00	1920.00	1964.33	-1.30	0.00
1107.00	1254.00	1210.67	1.12	0.01
141.00	147.00	156.33	1.13	0.15
3.00	5.00	3.33	-1.40	0.56
22.00	8.00	14.00	-1.75	0.01
184.00	235.00	238.33	1.38	0.00
332.00	336.00	338.00	1.61	0.00
4007.00	3842.00	3911.67	-1.28	0.00
11.00	5.00	7.67	1.33	0.44
9.00	5.00	5.67	-2.70	0.00
6.00	3.00	3.33	-2.09	0.08
3193.00	3075.00	3140.33	-1.09	0.03
800.00	888.00	833.67	-1.43	0.00
11.00	15.00	12.33	1.22	0.48
4.00	5.00	5.67	-1.25	0.63
340.00	296.00	342.00	-1.08	0.32
433.00	433.00	475.00	1.14	0.05
52.00	74.00	65.33	1.28	0.05
20471.00	21497.00	20997.00	-1.24	0.00
323.00	318.00	312.33	-1.03	0.77
5.00	6.00	6.67	1.77	0.16
67710.00	70448.00	73444.67	1.13	0.00
156.00	144.00	163.00	1.92	0.00
1192.00	1292.00	1246.00	-1.07	0.11
683.00	806.00	811.67	1.14	0.02
78.00	97.00	85.67	-1.08	0.46
27.00	26.00	25.67	1.58	0.02
122.00	116.00	120.00	-1.32	0.00
13618.00	14592.00	14340.00	-1.03	0.29
3583.00	3507.00	3532.33	1.12	0.01
20.00	19.00	20.67	1.74	0.01
312.00	313.00	324.00	-1.01	0.92
603.00	696.00	644.00	1.19	0.00
11277.00	12692.00	13164.33	1.12	0.03
2034.00	2132.00	2167.33	-1.12	0.00
13.00	4.00	7.67	-2.40	0.00
55.00	66.00	60.67	-1.25	0.05
743.00	676.00	760.00	-1.01	0.86
179.00	217.00	213.00	1.01	0.93
56.00	43.00	43.00	-1.35	0.04
318.00	291.00	307.00	-1.51	0.00
32.00	28.00	32.00	-1.67	0.00
1836.00	2128.00	2035.67	-1.01	0.84
2825.00	2804.00	2885.67	1.05	0.15
23.00	2.00	10.33	-1.25	0.63
600.00	614.00	618.67	1.14	0.01

823.00	851.00	889.67	1.01	0.79
3.00	8.00	8.00	-1.06	1.00
76.00	65.00	72.33	-1.41	0.00
119.00	99.00	110.67	1.20	0.06
199.00	188.00	206.33	-1.13	0.08
14.00	9.00	13.67	-1.18	0.54
1910.00	1819.00	1922.67	-1.24	0.00
34.00	49.00	48.33	-1.33	0.05
1099.00	1113.00	1121.00	1.20	0.00
9.00	7.00	8.00	1.47	0.28
1736.00	1865.00	1839.33	1.10	0.02
3727.00	3691.00	3695.33	-1.15	0.00
164.00	191.00	180.67	1.38	0.00
17.00	25.00	22.33	1.73	0.01
117.00	103.00	119.00	1.19	0.07
4.00	9.00	7.00	-1.11	0.88
16.00	19.00	24.33	1.28	0.24
221.00	239.00	241.33	1.15	0.04
464.00	515.00	542.00	1.16	0.02
687.00	686.00	687.67	-1.05	0.33
1062.00	1078.00	1055.67	-1.09	0.08
413.00	476.00	453.00	1.06	0.33
785.00	819.00	820.67	1.23	0.00
59.00	77.00	68.67	1.33	0.02
795.00	830.00	828.00	-1.02	0.62
928.00	1099.00	1012.00	1.43	0.00
6017.00	5690.00	5845.67	-1.20	0.00
9.00	15.00	14.67	-1.33	0.18
677.00	692.00	709.67	-1.07	0.20
3.00	12.00	8.67	1.81	0.09
344.00	349.00	345.00	1.37	0.00
95.00	143.00	122.00	1.23	0.04
377.00	453.00	420.33	1.12	0.05
5.00	1.00	4.33	1.16	0.84
436.00	429.00	441.67	1.01	0.89
1177.00	1258.00	1267.67	1.12	0.01
293.00	286.00	289.00	-1.22	0.00
524.00	438.00	490.67	-2.17	0.00
28.00	12.00	23.67	-1.41	0.06
2484.00	2629.00	2606.33	1.01	0.84
6585.00	7382.00	7316.67	1.13	0.00
911.00	935.00	930.33	1.74	0.00
625.00	670.00	681.67	-1.12	0.01
371.00	365.00	348.33	1.04	0.59
57.00	48.00	52.00	-1.27	0.05
133.00	133.00	142.33	-1.48	0.00
36.00	27.00	31.00	1.03	0.83
719.00	763.00	779.67	-1.17	0.00

267.00	244.00	246.67	1.12	0.13
1262.00	1217.00	1248.00	1.01	0.82
1260.00	1343.00	1314.00	1.03	0.51
256.00	297.00	295.33	1.08	0.25
267.00	265.00	289.67	1.18	0.01
479.00	606.00	584.67	-1.03	0.68
16.00	9.00	16.67	1.29	0.32
775.00	947.00	910.67	1.06	0.29
1295.00	1325.00	1390.00	-1.01	0.83
557.00	480.00	537.00	1.06	0.32
33.00	52.00	41.00	1.17	0.34
1638.00	1823.00	1744.00	1.16	0.00
1078.00	1133.00	1167.00	-1.02	0.69
100.00	88.00	95.67	-1.12	0.23
501.00	507.00	525.33	-1.06	0.21
2493.00	2711.00	2579.33	-1.00	0.98
1067.00	1167.00	1132.67	-1.20	0.00
2730.00	2721.00	2818.00	-1.19	0.00
1205.00	1240.00	1249.67	-1.05	0.29
1348.00	1242.00	1328.00	-1.19	0.00
42.00	43.00	47.67	1.02	0.91
890.00	958.00	981.67	1.29	0.00
1240.00	1281.00	1259.33	-1.14	0.01
2424.00	2789.00	2683.00	1.17	0.00
17.00	10.00	13.67	-1.04	1.00
1.00	0.00	2.00	-1.17	1.00
6.00	11.00	8.00	1.18	0.66
553.00	613.00	600.67	1.02	0.65
597.00	568.00	599.00	-1.10	0.07
1208.00	1152.00	1201.67	-1.87	0.00
2213.00	2220.00	2308.67	-1.29	0.00
901.00	984.00	991.67	-1.22	0.00
4.00	1.00	4.67	-1.51	0.33
4077.00	4477.00	4483.67	1.09	0.03
100.00	120.00	104.67	1.25	0.03
3.00	9.00	5.00	1.23	0.71
82.00	99.00	95.33	1.06	0.58
460.00	462.00	469.67	-1.13	0.03
966.00	1080.00	1044.33	-1.10	0.03
4668.00	4787.00	4907.00	-1.03	0.44
1102.00	1223.00	1177.33	-1.12	0.01
21.00	30.00	25.00	1.80	0.00
142.00	151.00	147.33	-1.16	0.06
714.00	706.00	738.67	1.10	0.05
410.00	604.00	511.67	1.24	0.00
253.00	304.00	285.33	1.49	0.00
270.00	311.00	311.33	1.42	0.00
660.00	653.00	645.67	1.29	0.00

1397.00	1391.00	1405.33	-1.05	0.22
3.00	1.00	1.67	-3.46	0.01
2020.00	2060.00	2098.00	-1.04	0.27
1208.00	1234.00	1242.67	-1.04	0.33
149.00	152.00	155.33	1.16	0.06
23.00	30.00	30.00	1.55	0.02
1043.00	1079.00	1046.67	-1.08	0.07
9.00	6.00	10.33	-1.27	0.42
0.00	0.00	0.00	-22.46	0.01
1.00	4.00	4.00	1.46	0.51
220.00	209.00	220.00	-1.09	0.21
799.00	953.00	874.67	-1.03	0.60
86.00	108.00	105.00	1.06	0.59
350.00	386.00	386.00	-1.02	0.77
2705.00	2931.00	2895.33	-1.19	0.00
172.00	213.00	190.00	1.02	0.82
256.00	303.00	278.33	-1.10	0.16
890.00	925.00	899.67	-1.53	0.00
39.00	24.00	36.67	2.00	0.00
1435.00	1630.00	1577.67	1.06	0.16
5367.00	5727.00	5583.67	-1.33	0.00
5.00	3.00	4.67	-1.44	0.40
1015.00	1024.00	1033.67	-1.32	0.00
1153.00	1021.00	1075.67	-1.08	0.16
607.00	585.00	614.00	-1.11	0.05
92.00	111.00	107.33	-1.15	0.12
1183.00	1175.00	1189.00	-1.19	0.00
4.00	15.00	8.67	1.02	1.00
113.00	125.00	127.33	-1.66	0.00
39.00	42.00	43.00	-1.94	0.00
25.00	21.00	23.00	1.18	0.50
986.00	939.00	957.67	-1.24	0.00
472.00	560.00	547.33	1.09	0.11
2646.00	2708.00	2763.67	-1.09	0.03
3930.00	4310.00	4253.33	1.51	0.00
951.00	1116.00	1085.33	1.12	0.01
473.00	526.00	500.67	1.10	0.06
1162.00	1340.00	1317.33	1.43	0.00
372.00	460.00	447.33	1.19	0.01
1869.00	2086.00	2093.33	1.17	0.00
556.00	582.00	602.33	1.13	0.02
4181.00	4611.00	4371.00	1.24	0.00
1437.00	1617.00	1573.33	1.03	0.55
238.00	250.00	259.67	-1.33	0.00
961.00	944.00	1035.00	1.23	0.00
352.00	312.00	347.67	-1.18	0.01
558.00	595.00	558.33	-1.10	0.10
3504.00	3594.00	3703.67	1.11	0.00

2753.00	2920.00	2893.67	1.15	0.00
270.00	274.00	263.33	-1.35	0.00
8.00	15.00	11.33	1.15	0.63
35.00	45.00	42.33	1.08	0.63
849.00	903.00	911.33	1.18	0.00
3.00	6.00	5.33	1.12	0.86
1.00	1.00	2.00	-2.13	0.17
561.00	643.00	636.33	1.23	0.00
2178.00	2418.00	2387.67	-1.05	0.18
785.00	761.00	821.67	1.06	0.29
354.00	397.00	377.67	1.08	0.20
374.00	354.00	375.33	1.08	0.19
20.00	27.00	26.33	-1.31	0.09
271.00	294.00	284.00	1.03	0.70
1054.00	999.00	1072.33	1.17	0.00
1454.00	1773.00	1603.00	1.26	0.00
2.00	4.00	2.00	-4.19	0.00
428.00	374.00	404.67	-1.25	0.00
360.00	472.00	453.33	1.31	0.00
872.00	893.00	920.33	-1.14	0.00
253.00	294.00	293.00	-1.12	0.06
47.00	42.00	49.00	-1.01	0.96
1297.00	1515.00	1477.67	-1.14	0.00
2262.00	2362.00	2346.67	1.18	0.00
1586.00	1576.00	1577.33	1.02	0.62
2063.00	1969.00	2005.33	-1.02	0.63
1279.00	1328.00	1300.33	1.08	0.15
3.00	1.00	2.00	1.17	1.00
281.00	291.00	286.33	1.16	0.03
3375.00	3281.00	3410.33	1.01	0.80
1662.00	1783.00	1725.33	1.32	0.00
867.00	933.00	902.67	1.12	0.04
8565.00	9062.00	9359.00	1.05	0.17
20.00	24.00	20.00	-1.13	0.61
5.00	8.00	5.00	-1.14	0.86
17.00	15.00	14.00	1.59	0.08
6015.00	6168.00	6285.00	1.13	0.00
192.00	199.00	198.33	1.00	1.00
2241.00	2363.00	2407.33	-1.00	0.94
33001.00	35083.00	35998.00	1.03	0.38
787.00	782.00	825.00	-1.26	0.00
885.00	918.00	883.00	1.15	0.03
331.00	341.00	341.33	-1.06	0.32
807.00	889.00	833.33	-1.19	0.00
40.00	45.00	53.33	1.13	0.44
758.00	849.00	865.33	1.11	0.04
2476.00	2546.00	2487.67	-1.09	0.08
5.00	7.00	6.67	-1.66	0.11

48.00	46.00	50.00	1.15	0.32
204.00	206.00	210.67	1.48	0.00
1702.00	1969.00	1923.00	1.19	0.00
10.00	13.00	13.67	1.03	1.00
2318.00	2590.00	2560.00	1.23	0.00
3225.00	3745.00	3609.00	1.03	0.39
1086.00	1174.00	1165.67	1.06	0.17
1.00	5.00	3.67	-1.64	0.28
1167.00	1252.00	1289.00	1.07	0.11
2743.00	2628.00	2819.33	1.01	0.90
794.00	812.00	808.33	1.09	0.06
2704.00	2980.00	2847.67	1.07	0.16
965.00	1066.00	994.33	-1.06	0.22
1104.00	1048.00	1102.67	-1.04	0.42
32.00	47.00	46.00	-1.17	0.25
8.00	3.00	4.33	-1.62	0.24
8.00	16.00	14.33	-1.01	1.00
3756.00	3662.00	3606.00	-1.74	0.00
693.00	663.00	708.67	-1.14	0.01
2712.00	2854.00	2811.67	-1.04	0.23
42.00	39.00	37.67	-1.15	0.37
90.00	87.00	91.67	-1.11	0.30
2676.00	3012.00	2871.00	-1.17	0.00
2442.00	2500.00	2441.00	-1.07	0.11
937.00	1037.00	1035.00	1.06	0.26
8.00	6.00	9.00	-1.64	0.07
1.00	5.00	2.33	-1.43	0.64
6.00	4.00	6.33	1.17	0.74
31.00	34.00	35.00	-1.44	0.01
729.00	830.00	810.67	1.17	0.00
3.00	0.00	2.67	-1.38	0.65
3423.00	4234.00	4091.33	-1.14	0.01
2359.00	2869.00	2835.33	-1.14	0.01
5.00	0.00	2.00	-2.75	0.04
1.00	4.00	3.33	-1.50	0.43
0.00	5.00	3.00	-1.88	0.18
2378.00	2316.00	2403.67	-1.43	0.00
1665.00	1751.00	1704.00	-1.02	0.56
3529.00	3615.00	3553.67	-1.01	0.87
1055.00	1097.00	1114.33	1.11	0.02
608.00	638.00	640.33	1.14	0.01
3126.00	3318.00	3172.33	1.17	0.00
1287.00	1247.00	1230.67	-1.24	0.00
1519.00	1564.00	1576.67	-1.04	0.27
581.00	644.00	649.33	1.08	0.14
1234.00	1294.00	1282.33	-1.08	0.07
1877.00	1846.00	1915.00	1.06	0.14
805.00	910.00	882.67	1.02	0.60

979.00	1031.00	1043.00	-1.13	0.00
1927.00	2065.00	2057.00	1.14	0.00
2713.00	2794.00	2858.67	1.11	0.00
3036.00	3042.00	3178.33	-1.08	0.09
324.00	354.00	337.67	1.06	0.29
1217.00	1326.00	1234.00	-1.34	0.00
189.00	238.00	218.33	-1.19	0.01
3255.00	3405.00	3378.33	-1.09	0.01
207.00	234.00	227.00	1.01	0.93
365.00	472.00	411.33	1.00	0.97
441.00	473.00	464.33	1.00	0.97
1372.00	1387.00	1359.33	-1.18	0.00
261.00	254.00	279.33	-1.07	0.35
1167.00	1388.00	1349.33	1.14	0.01
1751.00	1633.00	1712.00	-1.27	0.00
0.00	1.00	2.33	-1.15	1.00
1555.00	1729.00	1686.00	-1.01	0.74
2777.00	3157.00	3043.33	-1.02	0.52
116.00	116.00	113.00	1.15	0.14
258.00	236.00	262.67	1.07	0.34
4.00	4.00	5.67	-1.36	0.44
128.00	119.00	110.00	-1.36	0.01
1238.00	1420.00	1372.00	-1.09	0.04
8.00	6.00	11.33	-1.08	0.83
533.00	582.00	564.33	1.05	0.37
900.00	992.00	968.00	1.21	0.00
3.00	7.00	6.67	1.23	0.63
583.00	594.00	574.00	-1.00	1.00
2443.00	2656.00	2682.67	1.14	0.00
532.00	633.00	609.67	1.06	0.23
31.00	40.00	37.33	1.31	0.09
13.00	17.00	15.00	1.20	0.45
127.00	112.00	127.00	1.34	0.00
689.00	902.00	808.33	1.03	0.62
506.00	414.00	462.33	-1.09	0.23
567.00	649.00	631.33	1.20	0.00
907.00	849.00	882.67	-1.92	0.00
3.00	5.00	4.00	-1.67	0.22
2912.00	2956.00	2937.33	-1.07	0.08
1398.00	1571.00	1574.33	-1.13	0.00
226.00	198.00	213.00	-1.57	0.00
1419.00	1433.00	1502.33	-1.10	0.02
469.00	473.00	479.67	-1.10	0.07
41.00	69.00	54.00	-1.13	0.34
1150.00	1290.00	1334.00	1.03	0.58
12.00	10.00	11.67	-1.79	0.01
107.00	118.00	113.67	-1.51	0.00
96.00	119.00	111.00	-1.47	0.00

22.00	20.00	23.00	-1.42	0.03
42.00	23.00	36.67	-1.75	0.00
10.00	3.00	7.33	2.13	0.06
23.00	18.00	22.67	1.08	0.74
810.00	746.00	776.33	-1.18	0.00
103.00	102.00	107.33	-1.15	0.12
477.00	448.00	504.33	-1.30	0.00
883.00	834.00	874.00	-1.03	0.58
1005.00	1031.00	1001.00	1.12	0.02
37.00	66.00	50.33	-1.07	0.67
297.00	288.00	292.67	-1.08	0.22
10.00	8.00	11.33	-1.16	0.65
200.00	198.00	203.00	1.09	0.23
1439.00	1467.00	1513.67	-1.12	0.00
104.00	101.00	102.67	-2.13	0.00
0.00	0.00	0.00	-11.74	0.13
461.00	539.00	483.67	-1.02	0.71
4.00	3.00	4.67	1.92	0.20
44.00	43.00	42.00	-1.45	0.00
125.00	133.00	127.67	-1.32	0.00
6.00	6.00	5.67	1.19	0.72
71.00	58.00	67.00	-1.08	0.52
4.00	3.00	3.33	-1.31	0.68
138.00	130.00	137.33	-1.04	0.68
104.00	92.00	97.33	-1.13	0.20
393.00	418.00	408.33	-1.15	0.01
18.00	8.00	13.67	-1.67	0.02
725.00	612.00	645.00	-1.31	0.00
166.00	153.00	160.67	-1.08	0.34
5.00	2.00	2.67	-1.01	1.00
2188.00	2279.00	2324.33	1.01	0.80
711.00	799.00	797.33	-1.03	0.61
1851.00	1897.00	1892.67	-1.21	0.00
444.00	533.00	483.00	1.27	0.00
20.00	19.00	19.00	-1.22	0.37
2174.00	2128.00	2241.33	-1.07	0.10
2370.00	2410.00	2522.67	1.13	0.00
2041.00	2118.00	2190.33	1.21	0.00
2667.00	2776.00	2859.33	1.18	0.00
1134.00	1115.00	1142.67	-1.09	0.04
89.00	104.00	103.33	-1.17	0.08
111.00	133.00	120.33	1.05	0.60
395.00	351.00	386.00	1.05	0.41
2778.00	2875.00	2867.33	-1.09	0.01
65.00	95.00	87.00	1.05	0.71
1931.00	1989.00	1996.00	1.05	0.19
41.00	35.00	44.00	-1.35	0.02
92.00	86.00	82.33	1.03	0.79

27.00	30.00	29.33	-1.04	0.83
1736.00	1790.00	1852.00	1.14	0.00
7.00	3.00	5.67	1.39	0.47
4.00	10.00	6.33	-1.54	0.21
0.00	0.00	0.00	-11.74	0.13
2.00	0.00	1.00	-2.81	0.15
14.00	19.00	17.00	-2.06	0.00
11.00	20.00	15.67	-1.81	0.00
9.00	15.00	12.33	-1.64	0.03
71.00	82.00	75.33	-1.32	0.01
4356.00	4424.00	4486.67	-1.10	0.01
19.00	18.00	20.33	-1.36	0.08
1365.00	1480.00	1467.33	-1.15	0.00
165.00	157.00	168.33	-1.14	0.10
213.00	249.00	235.67	-1.14	0.04
976.00	1054.00	1065.33	1.35	0.00
0.00	1.00	1.33	-2.40	0.19
3.00	1.00	3.00	-3.18	0.00
5.00	2.00	5.33	3.00	0.03
11.00	7.00	9.33	1.38	0.35
9534.00	9883.00	9956.00	-1.32	0.00
1132.00	1163.00	1224.67	1.19	0.00
1087.00	1239.00	1226.33	1.23	0.00
465.00	429.00	444.00	-1.02	0.80
2166.00	2442.00	2411.67	1.11	0.01
881.00	868.00	872.00	1.06	0.20
372.00	407.00	389.00	-1.05	0.36
4.00	8.00	7.00	-1.68	0.09
721.00	834.00	812.33	1.27	0.00
444.00	492.00	489.33	-1.20	0.00
1346.00	1302.00	1313.33	-1.42	0.00
15.00	14.00	16.67	1.03	0.92
268.00	259.00	266.67	1.04	0.52
1025.00	1113.00	1089.33	1.07	0.12
1653.00	1622.00	1646.33	-1.08	0.09
310.00	267.00	305.67	1.23	0.01
323.00	331.00	344.00	1.19	0.01
5094.00	5620.00	5469.67	1.01	0.87
414.00	348.00	393.33	-1.08	0.29
791.00	765.00	800.67	1.22	0.00
2610.00	2980.00	2906.67	-1.04	0.26
162.00	211.00	188.00	1.23	0.01
378.00	441.00	406.67	1.13	0.05
2207.00	2298.00	2398.00	-1.02	0.67
50.00	55.00	61.00	1.02	0.92
621.00	611.00	669.67	-1.06	0.25
197.00	187.00	197.67	1.09	0.33
3811.00	3983.00	4215.33	1.08	0.09

77.00	65.00	78.67	1.15	0.23
3762.00	4148.00	4151.00	1.19	0.00
215.00	200.00	230.33	-1.10	0.36
536.00	619.00	587.00	1.40	0.00
1235.00	1442.00	1301.67	1.26	0.00
576.00	648.00	627.00	1.14	0.01
512.00	603.00	589.00	1.11	0.05
601.00	597.00	619.00	1.11	0.04
566.00	631.00	612.00	1.17	0.00
1667.00	1749.00	1695.00	-1.05	0.21
1095.00	1357.00	1320.67	1.17	0.00
1272.00	1327.00	1345.00	1.19	0.00
13.00	4.00	10.67	-1.08	0.91
6.00	12.00	7.00	1.04	1.00
1550.00	1778.00	1673.33	1.17	0.00
97.00	102.00	104.33	-1.26	0.01
468.00	523.00	524.00	1.10	0.13
1085.00	912.00	949.67	-1.10	0.18
92.00	86.00	98.33	-1.01	0.94
452.00	472.00	469.67	-1.11	0.03
494.00	498.00	527.00	-1.07	0.17
31.00	12.00	19.67	-1.52	0.06
43.00	38.00	41.33	-1.33	0.03
1753.00	2001.00	1963.00	1.16	0.00
3336.00	3446.00	3366.00	-1.14	0.00
1735.00	1770.00	1844.00	1.00	0.95
1768.00	2095.00	1946.00	-1.03	0.55
840.00	690.00	743.00	1.17	0.03
1262.00	1323.00	1354.33	1.03	0.49
683.00	862.00	845.00	1.18	0.01
1018.00	930.00	940.00	-1.24	0.00
670.00	811.00	749.00	1.00	0.93
5411.00	5734.00	5869.67	-1.18	0.00
878.00	935.00	924.67	-1.04	0.35
4040.00	4459.00	4262.33	1.13	0.00
9.00	1.00	5.00	1.34	0.57
392.00	381.00	406.67	1.21	0.00
512.00	527.00	528.00	-1.01	0.83
8.00	12.00	11.00	1.79	0.05
64.00	44.00	53.33	-1.09	0.54
877.00	841.00	881.67	-1.01	0.80
5.00	14.00	8.67	1.03	1.00
27.00	18.00	24.33	1.20	0.33
144.00	146.00	151.67	1.17	0.05
1969.00	2094.00	2043.67	1.03	0.45
1570.00	1673.00	1720.67	1.02	0.69
236.00	309.00	258.33	1.28	0.00
2562.00	2643.00	2711.00	1.01	0.74

167.00	228.00	208.33	1.01	0.89
418.00	400.00	416.00	-1.35	0.00
17.00	20.00	20.00	1.14	0.58
325.00	380.00	368.00	1.05	0.50
11.00	23.00	14.00	1.12	0.70
1477.00	1550.00	1480.67	-1.03	0.59
69.00	91.00	85.00	1.05	0.66
1232.00	1187.00	1267.00	1.21	0.00
445.00	395.00	433.33	-1.02	0.76
29.00	37.00	34.67	1.07	0.74
1144.00	1189.00	1150.67	1.05	0.28
798.00	825.00	832.00	-1.06	0.19
66.00	104.00	82.33	-1.14	0.23
1398.00	1414.00	1477.33	1.05	0.23
1169.00	1091.00	1170.67	-1.04	0.40
85.00	107.00	107.67	-1.15	0.14
433.00	488.00	482.33	1.14	0.02
2205.00	2543.00	2469.00	1.15	0.00
1105.00	1147.00	1146.67	1.17	0.00
2388.00	2704.00	2632.67	-1.02	0.57
4612.00	5340.00	5324.00	1.34	0.00
109.00	104.00	115.00	1.75	0.00
9108.00	10056.00	10265.67	1.22	0.00
67.00	60.00	60.33	1.23	0.10
2900.00	2590.00	2696.33	-1.36	0.00
479.00	473.00	468.33	1.09	0.15
447.00	593.00	535.67	1.12	0.09
476.00	430.00	416.33	-1.04	0.74
3870.00	4045.00	4033.33	1.10	0.01
229.00	207.00	216.00	1.35	0.00
429.00	516.00	473.33	1.08	0.25
664.00	728.00	704.33	1.22	0.00
571.00	568.00	577.67	1.27	0.00
4020.00	4435.00	4387.33	1.10	0.01
2010.00	2196.00	2191.67	1.14	0.00
556.00	568.00	584.00	1.09	0.07
506.00	480.00	520.00	-1.13	0.04
107.00	132.00	131.67	-1.65	0.00
4.00	10.00	7.00	1.85	0.12
79.00	63.00	73.67	1.36	0.01
7.00	8.00	7.00	1.58	0.24
44.00	43.00	47.67	1.15	0.33
80.00	91.00	90.33	1.16	0.14
1990.00	2364.00	2231.33	1.03	0.51
1038.00	1324.00	1233.33	-1.01	0.91
62.00	81.00	77.33	1.25	0.05
12.00	9.00	11.67	2.01	0.02
102.00	83.00	95.00	-1.05	0.66

1059.00	1189.00	1184.33	1.35	0.00
277.00	306.00	322.00	1.26	0.00
488.00	474.00	488.67	1.22	0.00
395.00	491.00	449.33	1.04	0.51
751.00	772.00	789.33	1.12	0.01
737.00	733.00	752.33	-1.04	0.42
1684.00	1842.00	1785.67	1.09	0.03
524.00	577.00	587.67	1.05	0.40
179.00	185.00	203.67	1.26	0.01
3.00	1.00	3.33	-1.31	0.68
2920.00	3130.00	3230.67	1.20	0.00
183.00	208.00	210.67	1.85	0.00
3.00	0.00	2.00	-1.17	1.00
25.00	21.00	23.00	1.33	0.13
6512.00	6209.00	6668.00	-1.06	0.14
652.00	649.00	666.33	-1.02	0.63
5.00	1.00	3.00	-1.66	0.32
1450.00	1581.00	1547.33	1.28	0.00
99.00	92.00	96.67	-1.08	0.44
402.00	460.00	460.00	1.03	0.64
2645.00	2518.00	2555.00	-1.09	0.05
576.00	619.00	634.00	1.43	0.00
229.00	259.00	246.00	1.08	0.25
17.00	5.00	12.33	-1.15	0.68
1.00	4.00	2.33	-1.56	0.49
7.00	22.00	15.67	1.08	0.86
3691.00	4068.00	4048.00	1.05	0.16
65.00	63.00	64.00	-2.11	0.00
1.00	0.00	0.33	-5.41	0.08
2381.00	2545.00	2526.00	1.01	0.74
979.00	995.00	1049.33	-1.27	0.00
38.00	53.00	54.00	-1.02	0.89
3618.00	3974.00	3950.67	1.17	0.00
1142.00	1244.00	1251.00	-1.01	0.88
435.00	438.00	442.33	-1.05	0.32
407.00	370.00	389.67	-1.31	0.00
1027.00	958.00	1049.67	-1.30	0.00
3345.00	3305.00	3484.67	-1.09	0.03
374.00	459.00	424.33	1.05	0.37
833.00	899.00	897.67	1.32	0.00
1111.00	1180.00	1212.00	1.25	0.00
356.00	432.00	420.33	1.05	0.41
7661.00	7914.00	7986.00	1.10	0.01
1387.00	1584.00	1505.67	1.10	0.04
941.00	1065.00	1037.33	1.30	0.00
489.00	508.00	517.67	1.09	0.11
454.00	529.00	516.00	-1.05	0.46
266.00	281.00	287.00	1.03	0.68

12092.00	12173.00	12388.33	-1.05	0.17
123.00	139.00	126.67	1.01	0.90
3914.00	4080.00	4055.33	-1.07	0.06
603.00	666.00	624.67	1.00	0.94
606.00	537.00	541.33	-1.18	0.02
1165.00	1256.00	1214.33	-1.11	0.01
1973.00	2182.00	2108.00	-1.12	0.00
1091.00	1213.00	1212.33	1.15	0.00
407.00	435.00	452.00	-1.06	0.31
460.00	510.00	499.33	-1.00	0.95
3725.00	3795.00	3724.33	-1.14	0.00
394.00	421.00	404.33	-1.01	0.83
864.00	861.00	898.67	-1.25	0.00
1448.00	1283.00	1330.00	-1.42	0.00
1003.00	1016.00	1005.33	1.24	0.00
1759.00	1604.00	1609.67	-1.51	0.00
430.00	463.00	458.67	1.14	0.01
64.00	85.00	74.00	1.05	0.66
359.00	438.00	415.33	1.57	0.00
205.00	216.00	233.00	-1.02	0.79
887.00	891.00	874.67	-1.08	0.10
1184.00	1070.00	1126.67	1.05	0.29
1426.00	1450.00	1445.67	-1.29	0.00
1.00	3.00	2.00	-2.12	0.17
1151.00	1224.00	1158.00	-1.13	0.01
1501.00	1509.00	1472.33	-1.31	0.00
392.00	475.00	464.33	-1.28	0.00
183.00	143.00	163.67	-1.66	0.00
44.00	50.00	48.33	-1.11	0.42
25.00	20.00	22.67	1.07	0.74
176.00	168.00	158.67	-1.07	0.48
2883.00	3147.00	3070.00	-1.06	0.07
90.00	101.00	92.67	1.61	0.00
108.00	119.00	119.00	1.00	1.00
2057.00	1965.00	2123.33	-1.02	0.73
861.00	891.00	869.67	-1.06	0.23
115.00	122.00	120.00	-1.58	0.00
0.00	0.00	0.00	-11.75	0.13
94.00	99.00	96.33	1.26	0.02
109.00	143.00	133.67	1.66	0.00
16.00	22.00	21.33	-1.22	0.31
34.00	33.00	35.33	1.77	0.00
4992.00	4875.00	5075.33	-1.10	0.01
6.00	11.00	8.33	-1.49	0.17
4979.00	5048.00	5118.00	1.06	0.15
446.00	472.00	477.00	-1.06	0.23
535.00	656.00	640.67	1.01	0.88
100.00	88.00	98.67	1.07	0.55

446.00	486.00	499.67	-1.03	0.52
54.00	66.00	64.33	-1.04	0.78
417.00	409.00	406.33	1.16	0.03
47.00	59.00	55.33	1.30	0.04
3.00	1.00	1.67	-1.20	1.00
11.00	2.00	6.33	-2.05	0.02
734.00	748.00	746.33	1.07	0.16
7712.00	7620.00	8035.67	-1.16	0.00
1427.00	1422.00	1465.67	-1.10	0.02
204.00	191.00	202.33	-1.41	0.00
275.00	335.00	322.67	1.45	0.00
449.00	544.00	496.33	1.09	0.13
95.00	108.00	106.00	-1.09	0.37
9.00	10.00	11.67	-1.93	0.00
12.00	13.00	14.67	-1.06	0.92
217.00	257.00	254.67	1.20	0.01
1355.00	1529.00	1485.33	1.18	0.00
1367.00	1457.00	1473.00	1.03	0.42
82.00	73.00	75.33	-1.18	0.14
1433.00	1623.00	1634.00	1.20	0.00
416.00	525.00	476.00	1.14	0.02
113.00	129.00	125.00	-1.39	0.00
152.00	154.00	160.33	-1.08	0.32
10.00	17.00	16.33	1.50	0.11
7084.00	7425.00	6972.33	-1.24	0.00
14.00	20.00	15.67	1.92	0.01
1830.00	1809.00	1851.33	-1.12	0.00
1186.00	1423.00	1374.33	1.24	0.00
598.00	597.00	672.33	1.10	0.15
396.00	425.00	418.00	1.23	0.00
454.00	501.00	503.33	-1.00	0.95
4699.00	5171.00	5269.00	1.15	0.00
488.00	569.00	556.33	1.10	0.09
617.00	616.00	623.33	1.14	0.00
1272.00	1293.00	1248.00	-1.32	0.00
634.00	662.00	656.33	-1.26	0.00
925.00	1058.00	971.67	-1.18	0.00
13.00	24.00	15.33	1.03	0.92
6139.00	5619.00	5658.33	-1.02	0.73
772.00	896.00	815.67	-1.04	0.45
1021.00	1100.00	1127.67	-1.01	0.75
458.00	549.00	524.33	1.21	0.00
13.00	24.00	20.00	-1.08	0.73
15.00	16.00	14.33	1.15	0.59
4.00	0.00	2.00	-1.65	0.47
3.00	2.00	4.33	-1.17	0.85
1.00	3.00	2.33	2.16	0.35
3.00	4.00	2.67	-1.01	1.00

16.00	11.00	16.67	-1.09	0.71
3.00	2.00	2.33	-1.97	0.20
182.00	208.00	211.33	-1.08	0.24
6.00	13.00	11.67	-1.04	0.91
0.00	1.00	0.33	-3.95	0.22
2.00	4.00	2.33	-1.15	1.00
9.00	7.00	9.33	-1.19	0.63
15.00	14.00	12.67	-1.38	0.20
1.00	3.00	2.33	-2.53	0.05
11.00	19.00	17.33	1.46	0.12
3.00	3.00	2.67	1.30	0.79
50.00	44.00	46.00	1.00	1.00
4.00	8.00	5.33	-1.57	0.22
715.00	639.00	666.33	-1.57	0.00
1220.00	1093.00	1129.00	-1.21	0.00
462.00	424.00	458.33	-1.03	0.65
1556.00	1737.00	1724.00	1.18	0.00
4661.00	4647.00	4743.67	-1.07	0.05
5513.00	5759.00	5634.67	1.15	0.00
153.00	139.00	141.33	-1.42	0.00
439.00	504.00	510.00	-1.06	0.36
2567.00	2451.00	2565.33	-1.06	0.28
344.00	365.00	358.00	-1.03	0.69
691.00	700.00	724.33	-1.18	0.00
12.00	23.00	15.33	1.96	0.01
137.00	121.00	130.33	1.52	0.00
2.00	9.00	6.00	1.04	1.00
1.00	2.00	1.67	-1.01	1.00
1195.00	1158.00	1196.67	-1.03	0.44
46.00	33.00	37.00	1.71	0.00
2533.00	3020.00	2802.33	1.10	0.02
219.00	267.00	248.67	-1.11	0.11
9.00	8.00	8.67	-1.55	0.12
4908.00	5016.00	5137.00	-1.18	0.00
64.00	66.00	61.00	-1.66	0.00
677.00	834.00	790.67	1.12	0.02
630.00	664.00	703.00	1.23	0.00
65.00	61.00	62.33	-1.10	0.47
3.00	4.00	5.00	1.22	0.71
929.00	968.00	938.00	1.02	0.63
1683.00	1750.00	1802.33	1.12	0.01
31721.00	31469.00	32780.00	-1.29	0.00
4948.00	5469.00	5223.33	-1.04	0.28
3999.00	4986.00	4639.00	1.32	0.00
2937.00	3560.00	3436.00	1.39	0.00
548.00	598.00	614.33	1.22	0.00
642.00	616.00	659.33	-1.05	0.34
1238.00	1349.00	1307.67	1.02	0.57

565.00	660.00	637.67	1.01	0.91
3196.00	3497.00	3446.00	1.25	0.00
2416.00	2761.00	2748.33	1.16	0.00
754.00	946.00	879.67	-1.04	0.45
18.00	32.00	22.33	-1.03	0.94
1852.00	2090.00	2034.67	1.01	0.75
414.00	517.00	471.33	1.49	0.00
1644.00	1717.00	1735.33	-1.10	0.01
827.00	888.00	876.00	-1.03	0.50
59.00	64.00	62.00	-1.55	0.00
923.00	1063.00	1002.67	1.13	0.01
498.00	577.00	565.67	1.63	0.00
876.00	948.00	960.00	1.22	0.00
1213.00	1310.00	1313.00	-1.06	0.12
83.00	111.00	105.00	1.44	0.00
4.00	0.00	2.00	-1.01	1.00
3619.00	3717.00	3784.33	1.18	0.00
10339.00	11704.00	11622.00	1.17	0.00
209.00	227.00	219.33	-1.06	0.42
2293.00	2876.00	2659.00	1.39	0.00
25453.00	31085.00	29178.33	1.19	0.00
9446.00	11207.00	10770.67	1.06	0.15
4697.00	5199.00	5168.00	1.26	0.00
12817.00	14545.00	14386.33	1.17	0.00
5015.00	5863.00	5716.67	1.21	0.00
5888.00	6935.00	6602.00	1.06	0.11
7179.00	7629.00	7699.67	1.12	0.00
13032.00	15928.00	14743.00	1.02	0.61
34.00	29.00	34.33	1.15	0.37
5390.00	6450.00	6173.33	1.14	0.00
13541.00	15091.00	14770.67	1.09	0.01
4602.00	4903.00	4971.33	1.13	0.00
716.00	686.00	699.33	1.08	0.11
6806.00	7641.00	7297.67	1.17	0.00
4279.00	4943.00	4631.33	1.17	0.00
15129.00	16462.00	16139.00	1.23	0.00
2069.00	2613.00	2329.33	-1.12	0.03
4732.00	4670.00	4680.00	1.09	0.02
1195.00	1384.00	1339.67	1.03	0.42
36.00	24.00	34.00	-1.08	0.61
347.00	390.00	341.33	1.24	0.01
51.00	57.00	46.67	-1.01	1.00
1198.00	1218.00	1254.67	1.10	0.02
216.00	240.00	237.00	-1.23	0.00
664.00	644.00	661.33	-1.19	0.00
723.00	843.00	788.00	-1.00	0.94
1127.00	1231.00	1272.33	-1.01	0.90
19.00	22.00	19.67	-1.35	0.12

680.00	703.00	732.67	-1.15	0.00
129.00	127.00	146.00	1.15	0.13
291.00	302.00	299.67	1.39	0.00
92.00	88.00	93.00	1.49	0.00
153.00	139.00	152.33	1.06	0.52
6.00	3.00	6.67	-1.36	0.44
25.00	27.00	24.00	-1.18	0.38
143.00	164.00	159.33	1.08	0.34
138.00	160.00	156.67	1.14	0.14
70.00	91.00	83.00	-1.04	0.72
17.00	11.00	15.67	-1.21	0.45
133.00	173.00	153.33	1.20	0.09
4.00	4.00	6.67	2.14	0.08
48.00	33.00	45.67	-1.17	0.28
1273.00	1504.00	1476.00	1.37	0.00
965.00	1068.00	1067.67	-1.07	0.11
475.00	548.00	533.33	1.09	0.14
357.00	355.00	370.00	-1.02	0.69
776.00	739.00	766.67	-1.09	0.05
496.00	543.00	550.67	1.16	0.00
209.00	216.00	223.67	1.00	0.98
2361.00	2309.00	2395.33	1.01	0.84
558.00	568.00	584.00	-1.05	0.39
24.00	29.00	30.00	1.25	0.23
38.00	51.00	48.33	1.25	0.11
44.00	47.00	48.67	1.13	0.36
243.00	270.00	259.67	1.06	0.35
63.00	88.00	72.33	1.09	0.48
337.00	385.00	373.33	-1.14	0.01
358.00	421.00	399.00	-1.19	0.00
154.00	180.00	179.67	1.15	0.07
31.00	27.00	31.00	-1.07	0.75
6.00	6.00	7.67	-1.40	0.29
96.00	94.00	94.33	1.28	0.01
241.00	259.00	250.00	-1.05	0.46
192.00	199.00	198.67	1.04	0.54
272.00	286.00	296.00	1.01	0.86
264.00	229.00	265.33	1.04	0.66
127.00	97.00	120.33	1.25	0.02
4.00	12.00	7.33	1.03	1.00
5779.00	6590.00	6234.33	-1.14	0.00
1346.00	1257.00	1360.33	-1.11	0.01
447.00	458.00	471.67	1.46	0.00
639.00	773.00	713.67	1.39	0.00
1105.00	1078.00	1138.67	-1.02	0.72
1318.00	1530.00	1423.33	-1.03	0.49
1178.00	1189.00	1227.67	-1.21	0.00
721.00	730.00	745.00	1.12	0.01

574.00	577.00	595.33	-1.02	0.63
209.00	231.00	249.33	-1.09	0.26
8.00	10.00	10.67	1.31	0.36
118.00	141.00	127.33	1.31	0.00
1.00	0.00	0.33	-2.48	0.64
3.00	2.00	4.33	1.41	0.53
111.00	134.00	125.33	-2.02	0.00
530.00	529.00	541.33	-1.41	0.00
377.00	496.00	466.67	1.26	0.00
529.00	481.00	513.33	-1.01	0.88
0.00	1.00	1.33	-2.17	0.28
1646.00	1843.00	1725.33	1.14	0.00
39.00	36.00	33.67	1.06	0.85
15.00	3.00	8.00	1.48	0.35
19.00	21.00	21.33	-1.09	0.68
308.00	330.00	325.00	1.12	0.06
12.00	3.00	6.00	1.20	0.78
42.00	43.00	37.33	-1.54	0.00
2164.00	2370.00	2412.33	1.25	0.00
34.00	20.00	29.33	-1.52	0.01
164.00	250.00	228.33	1.13	0.23
6.00	8.00	7.00	-2.20	0.00
197.00	177.00	204.00	1.05	0.57
30.00	25.00	30.33	-1.18	0.32
706.00	809.00	807.00	1.18	0.00
1061.00	1146.00	1158.33	1.17	0.00
2151.00	2311.00	2281.33	1.14	0.00
9.00	3.00	5.00	1.14	0.85
60.00	43.00	49.67	-1.28	0.05
1.00	11.00	6.33	1.84	0.16
1668.00	1846.00	1768.67	1.07	0.13
22.00	20.00	20.67	-1.11	0.67
85.00	79.00	85.33	1.09	0.43
47.00	55.00	47.33	-1.32	0.03
105.00	127.00	127.00	1.01	0.91
1.00	4.00	4.00	-1.42	0.47
932.00	968.00	991.33	-1.09	0.08
518.00	600.00	567.00	1.10	0.06
2429.00	2348.00	2485.33	-1.10	0.01
631.00	740.00	705.00	1.02	0.65
47.00	36.00	39.67	-1.09	0.65
1050.00	1047.00	1081.67	-1.26	0.00
3.00	12.00	7.00	-1.30	0.49
24744.00	28105.00	26702.00	1.25	0.00
50340.00	54586.00	53394.33	1.09	0.01
33538.00	38419.00	36810.67	-1.08	0.03
140.00	142.00	146.33	-1.21	0.01
39.00	43.00	40.00	-1.22	0.13

3353.00	3714.00	3507.00	-1.02	0.64
1370.00	1478.00	1488.33	1.09	0.03
29.00	20.00	22.00	-1.29	0.20
5.00	7.00	7.67	-1.27	0.50
100.00	109.00	105.67	1.07	0.53
4845.00	5420.00	5267.33	1.23	0.00
232.00	305.00	278.00	1.06	0.40
22752.00	25401.00	24559.67	-1.25	0.00
27127.00	29243.00	28769.00	1.02	0.59
16733.00	18226.00	17934.00	1.12	0.00
2657.00	2780.00	2912.33	1.37	0.00
235.00	301.00	288.67	1.19	0.01
444.00	529.00	528.33	1.13	0.06
880.00	801.00	884.33	1.02	0.64
15018.00	16594.00	15952.67	1.11	0.00
1078.00	1260.00	1294.67	1.16	0.01
1833.00	1471.00	1617.33	-1.42	0.00
3516.00	3964.00	3806.33	1.16	0.00
971.00	1024.00	1010.67	1.07	0.12
1327.00	1520.00	1455.33	1.11	0.01
312.00	316.00	302.33	1.05	0.47
4.00	2.00	2.67	-1.26	0.82
1.00	1.00	0.67	-4.00	0.07
563.00	548.00	576.33	-1.08	0.09
240.00	307.00	270.33	1.06	0.45
310.00	313.00	320.67	1.13	0.04
578.00	681.00	675.33	-1.02	0.79
3.00	7.00	4.67	1.92	0.20
3391.00	3222.00	3254.33	-1.26	0.00
0.00	5.00	3.00	-2.09	0.09
358.00	334.00	362.33	-1.09	0.15
8022.00	7202.00	7450.67	-1.26	0.00
4.00	4.00	4.00	-1.10	1.00
12.00	8.00	8.67	-1.44	0.22
1162.00	1146.00	1173.67	-1.03	0.60
212.00	183.00	190.33	-1.23	0.01
1731.00	1673.00	1757.33	-1.24	0.00
61.00	81.00	71.33	1.08	0.54
196.00	204.00	220.67	1.16	0.05
6418.00	6642.00	6518.33	-1.20	0.00
457.00	509.00	522.67	1.05	0.44
8893.00	9421.00	9306.00	-1.05	0.16
3899.00	4018.00	3952.67	1.15	0.00
185.00	184.00	189.00	-1.19	0.03
8.00	7.00	5.67	1.83	0.18
1916.00	2003.00	1905.67	-1.08	0.13
533.00	555.00	558.00	1.14	0.01
154.00	142.00	146.00	1.21	0.02

5240.00	5464.00	5490.67	1.06	0.13
166.00	164.00	176.00	1.24	0.01
2.00	3.00	5.00	-1.15	0.86
1406.00	1445.00	1446.00	-1.13	0.00
2244.00	2410.00	2316.67	-1.09	0.03
1272.00	1394.00	1358.00	-1.06	0.12
289.00	265.00	279.33	-1.12	0.10
1874.00	1971.00	1929.33	1.02	0.57
409.00	442.00	460.67	1.11	0.08
268.00	288.00	300.00	1.16	0.06
10.00	5.00	7.00	1.09	0.88
378.00	431.00	442.33	-1.06	0.31
1225.00	1361.00	1289.00	-1.02	0.67
1574.00	1771.00	1720.33	1.08	0.04
8.00	7.00	7.67	1.60	0.20
430.00	444.00	452.67	1.34	0.00
962.00	1102.00	1037.00	1.07	0.12
1392.00	1481.00	1545.33	-1.06	0.18
625.00	695.00	694.33	1.00	1.00
477.00	535.00	519.00	1.10	0.08
37.00	52.00	48.00	1.02	0.91
59.00	94.00	73.00	1.13	0.37
1868.00	1848.00	1800.00	-1.41	0.00
301.00	299.00	319.00	-1.32	0.00
2496.00	2554.00	2691.33	1.13	0.00
881.00	997.00	983.33	1.32	0.00
1073.00	1099.00	1111.00	-1.09	0.04
61.00	48.00	58.00	-1.17	0.20
1012.00	1123.00	1109.33	-1.14	0.00
560.00	576.00	572.33	1.02	0.71
5144.00	5726.00	5599.00	-1.17	0.00
1.00	18.00	12.00	1.01	1.00
20.00	17.00	20.00	-2.10	0.00
156.00	131.00	154.67	1.24	0.02
490.00	595.00	546.67	-1.08	0.20
35.00	51.00	45.33	-2.23	0.00
116.00	109.00	125.00	1.29	0.01
98.00	105.00	107.00	1.21	0.04
7.00	29.00	15.00	1.20	0.61
64.00	56.00	59.33	-1.02	0.88
171.00	179.00	182.00	1.03	0.68
660.00	704.00	724.00	1.03	0.62
77.00	62.00	68.67	1.11	0.37
731.00	826.00	849.00	1.12	0.04
910.00	846.00	942.33	1.10	0.07
1128.00	1133.00	1108.33	1.15	0.02
346.00	376.00	386.00	1.19	0.00
269.00	346.00	309.67	-1.39	0.00

1021.00	1103.00	1115.67	-1.07	0.12
62.00	38.00	46.67	1.04	0.82
2240.00	2464.00	2417.00	1.13	0.00
2438.00	2596.00	2610.33	1.13	0.00
384.00	387.00	398.33	1.04	0.50
843.00	902.00	885.67	-1.07	0.12
1170.00	1199.00	1211.67	-1.17	0.00
377.00	366.00	382.33	-1.15	0.01
166.00	167.00	182.33	1.12	0.16
359.00	345.00	368.67	-1.02	0.73
294.00	287.00	290.33	-1.00	0.96
510.00	525.00	531.33	1.05	0.32
350.00	381.00	384.00	1.06	0.29
770.00	893.00	882.67	1.15	0.01
411.00	520.00	492.00	1.11	0.07
1063.00	1350.00	1245.67	-1.19	0.00
534.00	689.00	628.67	1.03	0.65
348.00	446.00	407.67	1.15	0.03
1237.00	1425.00	1418.67	-1.01	0.79
6.00	1.00	3.33	-1.40	0.55
2037.00	2096.00	2075.33	-1.15	0.00
2333.00	2317.00	2335.67	-1.09	0.03
5886.00	5991.00	6086.67	-1.02	0.49
65.00	88.00	76.67	1.25	0.07
3028.00	3437.00	3216.67	1.09	0.05
9536.00	9209.00	9087.00	-1.34	0.00
14.00	11.00	13.67	1.74	0.04
682.00	676.00	714.33	5.80	0.00
16.00	44.00	32.67	-1.26	0.17
469.00	536.00	527.00	1.16	0.03
310.00	327.00	304.33	-1.55	0.00
27.00	32.00	29.00	1.13	0.55
112.00	66.00	100.67	-2.42	0.00
571.00	589.00	593.00	1.03	0.54
160.00	208.00	180.67	-1.13	0.18
4.00	4.00	4.33	-1.09	1.00
737.00	751.00	742.00	-1.44	0.00
0.00	2.00	1.00	-5.51	0.00
96.00	86.00	89.33	-1.11	0.31
1.00	5.00	3.00	-1.99	0.13
1024.00	1138.00	1116.67	-1.24	0.00
2103.00	2484.00	2384.67	1.03	0.51
1230.00	1518.00	1421.33	1.16	0.01
1914.00	2109.00	1971.00	1.02	0.70
2716.00	2685.00	2774.00	-1.18	0.00
140.00	136.00	148.67	-1.46	0.00
306.00	331.00	333.00	-1.01	0.93
59.00	62.00	55.00	-1.39	0.00

207.00	214.00	223.00	-1.32	0.00
596.00	747.00	653.00	1.06	0.35
538.00	510.00	530.00	1.22	0.00
106.00	107.00	103.67	-6.39	0.00
65.00	63.00	68.33	-1.63	0.00
681.00	747.00	743.67	1.87	0.00
5.00	11.00	8.67	-1.28	0.45
3.00	4.00	2.33	-1.84	0.27
214.00	225.00	210.00	-1.10	0.24
1617.00	1473.00	1542.33	-1.05	0.34
125.00	157.00	143.33	-1.27	0.01
8.00	10.00	10.33	1.02	1.00
17.00	10.00	12.00	-1.90	0.00
7.00	12.00	12.67	-1.28	0.30
64.00	59.00	68.67	-1.10	0.41
632.00	667.00	658.33	-1.04	0.44
548.00	453.00	513.33	1.08	0.20
486.00	422.00	456.67	-1.04	0.49
8.00	13.00	10.67	-1.36	0.26
575.00	511.00	526.67	-1.69	0.00
31.00	44.00	39.00	-1.03	0.90
101.00	123.00	124.00	1.21	0.04
9994.00	11007.00	11012.00	1.70	0.00
48.00	36.00	44.00	-1.09	0.54
985.00	1106.00	1034.67	-1.05	0.29
78.00	65.00	73.33	-1.56	0.00
12.00	6.00	14.67	-2.73	0.00
84.00	92.00	93.00	-1.28	0.01
73.00	64.00	75.33	1.70	0.00
7.00	10.00	10.67	1.09	0.80
47.00	52.00	52.67	-1.78	0.00
703.00	749.00	726.33	1.19	0.00
5.00	0.00	3.00	-1.12	1.00
3.00	2.00	3.33	1.60	0.48
837.00	761.00	784.00	-1.40	0.00
30.00	48.00	48.00	2.11	0.00
13.00	14.00	14.67	1.03	0.92
26.00	21.00	26.67	-1.16	0.41
763.00	785.00	800.00	-1.21	0.00
63.00	88.00	73.33	2.08	0.00
11.00	9.00	9.67	-3.39	0.00
5500.00	5526.00	5386.00	-1.01	0.84
44.00	78.00	58.00	1.56	0.00
126.00	144.00	132.00	-3.73	0.00
4449.00	4881.00	4814.00	1.20	0.00
11927.00	12129.00	12206.67	1.00	0.95
363.00	361.00	357.00	1.01	0.89
3144.00	3294.00	3389.33	1.18	0.00

715.00	771.00	777.67	1.05	0.28
1251.00	1220.00	1323.33	-1.05	0.30
77.00	71.00	78.67	1.18	0.13
291.00	387.00	349.67	1.06	0.42
2344.00	2572.00	2549.67	1.14	0.00
1247.00	1326.00	1340.00	1.19	0.00
1276.00	1296.00	1334.00	1.05	0.26
759.00	932.00	858.67	1.12	0.02
90.00	71.00	86.33	1.28	0.02
1041.00	1206.00	1137.33	1.26	0.00
3460.00	3812.00	3597.33	1.01	0.88
1896.00	1880.00	1984.33	1.08	0.06
8704.00	9308.00	9325.67	1.00	0.99
39.00	32.00	33.00	-1.27	0.13
45.00	54.00	44.67	-1.54	0.00
62.00	83.00	71.33	1.03	0.79
598.00	610.00	631.33	-1.01	0.88
12.00	23.00	16.00	-1.01	1.00
958.00	971.00	969.67	1.07	0.15
103.00	102.00	98.67	-1.32	0.00
2339.00	2288.00	2357.33	-1.16	0.00
217.00	236.00	232.67	1.09	0.18
94.00	107.00	105.67	1.22	0.04
296.00	392.00	370.67	1.13	0.08
490.00	542.00	539.67	-1.15	0.00
1003.00	953.00	1030.33	-1.04	0.45
40.00	30.00	33.33	1.06	0.69
2892.00	2682.00	2701.00	1.03	0.53
14.00	7.00	10.67	-1.08	0.91
5.00	2.00	3.67	-1.55	0.36
262.00	250.00	258.67	-1.87	0.00
190.00	184.00	178.00	1.03	0.77
1372.00	1309.00	1351.67	-1.10	0.03
38.00	36.00	36.67	-1.82	0.00
182.00	217.00	203.33	1.57	0.00
561.00	677.00	638.00	-1.04	0.48
1396.00	1313.00	1397.00	1.12	0.01
633.00	792.00	743.00	-1.07	0.21
1052.00	1031.00	1072.33	-1.05	0.20
251.00	247.00	241.33	-1.78	0.00
442.00	429.00	459.00	-1.04	0.42
747.00	762.00	753.33	1.09	0.07
1014.00	1043.00	1054.00	-1.15	0.00
699.00	665.00	679.67	-1.15	0.00
20.00	11.00	18.33	-1.60	0.02
420.00	477.00	455.00	-1.08	0.13
5113.00	5073.00	5225.33	-1.17	0.00
77.00	91.00	78.00	3.07	0.00

1164.00	1227.00	1208.67	1.37	0.00
356.00	359.00	359.00	-1.16	0.01
3.00	9.00	6.67	1.77	0.16
3675.00	3610.00	3651.00	-1.12	0.00
827.00	892.00	892.33	1.07	0.13
398.00	438.00	436.67	1.01	0.90
1159.00	1243.00	1186.00	1.02	0.68
7.00	8.00	9.33	1.37	0.32
429.00	450.00	452.67	-1.09	0.11
7.00	7.00	7.00	-1.49	0.22
511.00	526.00	531.00	-1.18	0.00
1086.00	1156.00	1128.67	1.08	0.05
31.00	67.00	50.00	1.04	0.84
783.00	875.00	849.33	1.10	0.05
1465.00	1679.00	1628.00	-1.04	0.31
407.00	413.00	434.00	1.03	0.59
154.00	191.00	188.67	1.19	0.03
402.00	427.00	433.67	-1.06	0.27
861.00	884.00	913.00	-1.06	0.16
1786.00	1985.00	1989.33	-1.00	0.93
1.00	1.00	2.00	-3.39	0.01
252.00	320.00	273.33	-1.03	0.74
250.00	279.00	260.33	-1.00	1.00
1106.00	1228.00	1164.67	-1.10	0.03
2152.00	2215.00	2275.00	1.05	0.28
6070.00	6478.00	6408.67	1.10	0.01
5.00	9.00	8.67	-1.32	0.38
8596.00	9387.00	9020.67	1.04	0.37
1891.00	2001.00	1933.67	1.00	0.92
2428.00	2448.00	2413.00	-1.13	0.00
8.00	10.00	9.00	2.37	0.02
286.00	338.00	311.33	1.05	0.44
453.00	489.00	513.33	1.08	0.21
707.00	890.00	816.33	-1.09	0.13
1208.00	1379.00	1359.33	1.06	0.20
148.00	178.00	176.33	1.12	0.16
9.00	27.00	17.67	-1.36	0.12
419.00	357.00	388.00	-1.11	0.15
559.00	568.00	585.33	1.30	0.00
127.00	129.00	124.67	1.02	0.87
198.00	243.00	210.67	-1.21	0.01
23.00	34.00	27.00	1.42	0.06
75.00	75.00	77.00	1.13	0.24
6683.00	6876.00	6647.00	-1.17	0.00
8.00	8.00	7.33	-2.19	0.00
2621.00	2697.00	2719.33	-1.16	0.00
651.00	606.00	610.33	-1.19	0.00
70.00	74.00	71.00	-1.01	0.93

7.00	4.00	7.67	-1.19	0.68
16.00	10.00	14.33	1.15	0.59
6034.00	5734.00	6185.33	1.08	0.05
189.00	204.00	191.33	1.07	0.38
227.00	265.00	240.67	-1.08	0.27
12.00	6.00	8.67	-1.13	0.79
762.00	827.00	815.33	1.28	0.00
970.00	1148.00	1042.67	-1.25	0.00
237.00	237.00	256.00	-1.12	0.09
303.00	274.00	308.33	-1.03	0.72
699.00	699.00	739.67	1.13	0.02
2682.00	2799.00	2802.67	1.03	0.51
1054.00	1049.00	1051.33	-1.25	0.00
1539.00	1619.00	1630.00	-1.01	0.85
7.00	5.00	5.33	1.12	0.86
256.00	288.00	294.67	-1.11	0.10
5.00	8.00	7.00	1.15	0.75
675.00	693.00	694.67	-1.35	0.00
4192.00	4199.00	4181.00	1.16	0.00
728.00	693.00	697.67	1.19	0.00
132.00	118.00	126.33	-1.20	0.03
30.00	47.00	36.33	-1.06	0.71
327.00	404.00	384.00	1.14	0.05
165.00	216.00	196.00	1.06	0.48
752.00	847.00	846.67	-1.07	0.15
44.00	38.00	43.33	-1.43	0.00
461.00	499.00	525.00	1.10	0.14
733.00	738.00	756.33	-1.22	0.00
30.00	46.00	40.00	-1.09	0.56
82.00	104.00	98.00	1.06	0.61
124.00	134.00	131.33	1.11	0.20
473.00	482.00	499.00	1.38	0.00
404.00	407.00	452.67	1.03	0.71
59.00	78.00	64.67	1.20	0.14
2333.00	2379.00	2392.33	1.01	0.71
348.00	366.00	373.33	1.03	0.65
133.00	124.00	144.00	-1.43	0.00
1715.00	1792.00	1803.67	1.01	0.73
1602.00	1640.00	1641.33	-1.01	0.84
471.00	458.00	472.33	-1.02	0.73
1881.00	1742.00	1902.67	-1.12	0.02
310.00	294.00	292.00	-1.69	0.00
1318.00	1395.00	1429.00	1.53	0.00
6.00	2.00	4.33	-5.03	0.00
2477.00	2691.00	2507.67	-1.76	0.00
5.00	3.00	3.33	-1.31	0.68
21453.00	21658.00	21837.00	-2.01	0.00
2254.00	2520.00	2352.67	1.05	0.30

5732.00	5880.00	5835.33	-1.32	0.00
20843.00	21591.00	20836.67	1.20	0.00
30.00	55.00	48.67	-1.25	0.11
8.00	6.00	9.00	1.55	0.18
335.00	423.00	404.67	1.06	0.39
0.00	0.00	0.33	-3.22	0.38
2155.00	2465.00	2199.67	-1.08	0.19
40.00	58.00	57.33	-2.02	0.00
31760.00	33234.00	32453.67	-1.07	0.07
622.00	751.00	722.33	1.18	0.00
28.00	26.00	27.67	-1.18	0.32
2806.00	2932.00	2936.00	-1.32	0.00
564.00	673.00	654.67	-1.06	0.25
188.00	193.00	188.33	-4.46	0.00
356.00	418.00	413.00	1.17	0.01
14.00	23.00	14.67	-1.06	0.92
2549.00	2517.00	2609.33	-1.97	0.00
3.00	1.00	2.00	-2.28	0.12
371.00	486.00	408.67	2.40	0.00
4407.00	4700.00	4687.33	-1.14	0.00
12.00	19.00	17.67	-1.68	0.00
2577.00	2614.00	2611.00	1.06	0.18
3189.00	3089.00	3240.67	-1.17	0.00
1490.00	1620.00	1657.33	1.20	0.00
137.00	157.00	157.00	-1.13	0.11
463.00	432.00	447.67	1.26	0.00
3.00	0.00	1.33	-1.01	1.00
264.00	255.00	276.33	-1.12	0.08
510.00	483.00	486.00	1.13	0.05
15.00	13.00	14.67	1.01	1.00
1310.00	1293.00	1299.00	-1.15	0.00
2322.00	1914.00	2148.33	-1.57	0.00
4595.00	4493.00	4613.33	-1.24	0.00
17.00	25.00	18.33	1.23	0.34
3497.00	3373.00	3404.33	-1.19	0.00
1164.00	1198.00	1174.00	1.28	0.00
1070.00	1004.00	1014.67	-1.11	0.05
1624.00	1607.00	1653.00	-1.06	0.18
2634.00	3047.00	2894.00	1.04	0.31
1360.00	1536.00	1483.00	1.11	0.01
26.00	36.00	36.67	-1.48	0.01
714.00	859.00	786.33	1.16	0.01
865.00	842.00	827.00	-1.03	0.67
513.00	486.00	486.67	1.03	0.59
1041.00	1175.00	1091.67	-2.39	0.00
568.00	551.00	572.67	-1.08	0.10
3538.00	3839.00	3745.67	-1.09	0.02
415.00	573.00	477.67	-1.10	0.21

4.00	6.00	4.33	-1.32	0.59
193.00	171.00	184.67	-1.05	0.49
1169.00	1149.00	1154.00	1.01	0.83
636.00	643.00	632.33	-1.17	0.00
169.00	213.00	186.33	1.11	0.17
2.00	3.00	1.67	-1.76	0.44
0.00	0.00	1.33	-1.02	1.00
67.00	83.00	79.00	-1.01	0.91
1419.00	1443.00	1463.67	1.07	0.10
3528.00	3703.00	3631.33	-1.06	0.15
396.00	362.00	402.67	1.12	0.13
606.00	609.00	640.00	-1.06	0.19
82.00	93.00	91.67	-1.10	0.30
851.00	860.00	905.33	1.05	0.32
562.00	652.00	596.67	-1.29	0.00
1737.00	1670.00	1756.67	1.06	0.15
279.00	298.00	304.33	1.01	0.91
231.00	236.00	245.67	1.11	0.12
165.00	155.00	179.00	1.11	0.23
2136.00	2089.00	2089.67	-1.17	0.00
880.00	1109.00	984.67	1.07	0.19
1921.00	2006.00	2015.00	1.19	0.00
9136.00	9912.00	9421.67	1.18	0.00
613.00	681.00	678.33	1.29	0.00
276.00	234.00	259.33	-1.27	0.00
7.00	12.00	8.67	-1.97	0.01
994.00	913.00	938.67	-1.31	0.00
1478.00	1498.00	1484.67	-2.47	0.00
295.00	260.00	284.33	-1.08	0.24
231.00	255.00	242.67	-1.06	0.48
575.00	585.00	600.33	1.07	0.17
1836.00	2083.00	2085.33	-1.08	0.09
21.00	7.00	15.67	2.09	0.01
3322.00	3593.00	3541.00	1.13	0.00
3995.00	4220.00	4276.67	-1.15	0.00
276.00	326.00	304.67	1.34	0.00
1260.00	1277.00	1319.33	-1.02	0.64
987.00	914.00	911.33	-1.55	0.00
968.00	937.00	928.67	-1.17	0.00
885.00	835.00	868.00	-1.10	0.04
644.00	660.00	668.33	-1.12	0.02
583.00	641.00	636.33	1.20	0.00
218.00	227.00	221.33	-1.07	0.33
131.00	127.00	129.33	-1.03	0.81
983.00	1061.00	1079.67	1.02	0.65
524.00	658.00	604.33	1.15	0.01
32.00	29.00	29.33	1.04	0.85
92.00	96.00	95.67	1.05	0.67

1876.00	2140.00	2107.67	1.07	0.11
25.00	45.00	39.00	-1.07	0.74
285.00	290.00	292.33	1.17	0.01
375.00	459.00	403.00	1.09	0.18
107.00	72.00	92.33	1.31	0.05
236.00	301.00	272.00	1.03	0.69
238.00	271.00	259.33	-1.27	0.00
1264.00	1383.00	1395.67	1.19	0.00
0.00	1.00	1.00	-1.31	1.00
13.00	15.00	17.00	1.43	0.14
2947.00	2801.00	2955.33	-1.23	0.00
142.00	141.00	139.67	-1.13	0.13
101.00	85.00	93.67	-1.13	0.21
82.00	86.00	90.67	-1.98	0.00
3.00	1.00	1.67	-1.77	0.43
126.00	125.00	130.00	1.00	1.00
15.00	20.00	23.33	-1.43	0.04
3831.00	3683.00	3845.33	1.07	0.06
19.00	21.00	19.67	1.66	0.02
122.00	93.00	98.00	-1.77	0.00
25.00	20.00	23.67	1.25	0.23
5.00	1.00	2.67	-1.25	0.82
5.00	4.00	5.67	-1.48	0.30
151.00	155.00	163.00	-1.61	0.00
12.00	18.00	16.67	-1.10	0.64
490.00	499.00	524.33	-1.23	0.00
221.00	222.00	230.33	-1.19	0.01
10.00	6.00	8.00	-1.43	0.25
326.00	328.00	335.00	1.21	0.00
23.00	31.00	27.67	1.60	0.01
5.00	7.00	6.33	1.24	0.61
2.00	8.00	4.67	1.15	0.85
149.00	146.00	149.67	1.97	0.00
109.00	105.00	111.67	-1.07	0.43
394.00	418.00	424.00	2.55	0.00
2.00	0.00	2.33	-3.07	0.01
101.00	111.00	105.00	-1.61	0.00
1.00	5.00	2.00	-1.49	0.62
4054.00	4063.00	4051.33	-1.11	0.01
16.00	11.00	15.33	-1.23	0.39
153.00	179.00	169.00	1.10	0.22
26.00	21.00	24.33	1.06	0.75
74.00	108.00	89.00	1.03	0.77
418.00	444.00	462.33	-1.05	0.37
220.00	236.00	216.33	-1.44	0.00
3.00	3.00	4.33	-2.83	0.00
1438.00	1468.00	1434.67	-1.15	0.00
3.00	5.00	4.67	1.15	0.85

5.00	13.00	8.00	-1.55	0.14
50.00	46.00	48.00	-1.27	0.05
211.00	257.00	224.67	-1.02	0.84
92.00	101.00	101.33	-1.33	0.00
9.00	7.00	8.33	-2.45	0.00
438.00	426.00	422.33	1.03	0.67
1231.00	1314.00	1291.67	-1.13	0.00
378.00	422.00	418.00	-1.13	0.02
1113.00	1268.00	1171.67	1.27	0.00
546.00	560.00	587.67	-1.01	0.88
209.00	199.00	207.33	-1.13	0.09
9.00	10.00	10.67	-1.36	0.26
263.00	268.00	271.67	-1.09	0.14
669.00	732.00	718.67	1.02	0.66
11.00	10.00	12.33	1.04	0.91
527.00	561.00	579.00	-1.03	0.49
1258.00	1284.00	1299.00	1.01	0.89
142.00	135.00	147.00	-1.15	0.08
7.00	13.00	9.67	-1.15	0.71
798.00	911.00	882.33	-1.02	0.67
209.00	259.00	231.67	-1.05	0.48
596.00	620.00	634.00	-1.04	0.46
194.00	216.00	220.00	1.06	0.39
528.00	494.00	503.67	-1.15	0.02
6.00	12.00	9.00	1.39	0.32
409.00	508.00	465.00	1.04	0.49
347.00	355.00	376.00	1.13	0.04
1613.00	1595.00	1598.67	1.08	0.06
3514.00	3729.00	3771.33	-1.07	0.13
5450.00	5486.00	5520.67	1.02	0.69
459.00	513.00	491.00	-1.02	0.78
2451.00	2546.00	2546.33	1.11	0.00
959.00	975.00	961.67	1.34	0.00
2652.00	2552.00	2597.67	-1.02	0.66
771.00	732.00	748.67	1.04	0.39
1398.00	1530.00	1464.67	1.02	0.60
2210.00	2028.00	2143.33	-1.09	0.06
2347.00	2401.00	2432.33	1.06	0.11
40.00	49.00	53.00	1.13	0.42
684.00	697.00	710.33	-1.17	0.00
933.00	996.00	974.00	-1.02	0.72
61.00	68.00	63.67	1.16	0.20
1347.00	1481.00	1372.00	1.00	0.98
1762.00	1784.00	1803.00	-1.03	0.45
5563.00	5228.00	5478.33	-1.18	0.00
686.00	713.00	701.67	-1.03	0.49
1116.00	949.00	1041.00	-1.38	0.00
1102.00	1225.00	1131.33	-1.05	0.36

76.00	71.00	74.67	-1.14	0.23
79.00	62.00	67.33	1.05	0.72
2082.00	1931.00	2020.67	1.01	0.91
831.00	817.00	874.67	1.02	0.74
4.00	1.00	3.33	-1.40	0.55
4.00	5.00	10.33	-1.13	0.78
118.00	170.00	141.00	-1.03	0.78
3725.00	4466.00	4249.67	1.20	0.00
137.00	153.00	153.33	1.22	0.02
29.00	29.00	29.33	1.52	0.02
2230.00	2117.00	2182.00	-1.22	0.00
4634.00	4475.00	4584.33	1.03	0.42
2586.00	2954.00	2804.67	1.15	0.00
5136.00	4751.00	4843.00	-1.22	0.00
292.00	400.00	373.67	1.55	0.00
1952.00	1960.00	2034.33	-1.11	0.00
1829.00	1835.00	1875.00	-1.30	0.00
1660.00	1669.00	1699.33	1.00	0.92
879.00	911.00	929.33	1.03	0.54
4.00	6.00	5.00	-4.18	0.00
860.00	833.00	842.33	-1.06	0.25
2319.00	2238.00	2306.67	-1.07	0.14
178.00	156.00	156.67	-1.28	0.01
3558.00	3834.00	3711.33	-1.08	0.03
698.00	723.00	706.67	1.13	0.02
232.00	201.00	227.00	-1.07	0.36
715.00	697.00	700.33	-1.36	0.00
740.00	660.00	690.00	1.01	0.93
1039.00	1255.00	1153.00	-1.06	0.24
2012.00	1325.00	1407.67	-1.30	0.11
20.00	28.00	22.00	1.08	0.74
187.00	194.00	194.33	1.03	0.67
584.00	651.00	650.67	-1.18	0.00
3968.00	4298.00	4248.67	-1.12	0.00
682.00	716.00	711.67	-1.05	0.29
537.00	505.00	533.00	1.02	0.67
96.00	105.00	104.67	-1.37	0.00
1573.00	1769.00	1636.33	-1.09	0.06
199.00	231.00	228.00	-1.19	0.01
1766.00	1963.00	1873.00	-1.27	0.00
510.00	544.00	494.33	1.18	0.05
12.00	22.00	18.33	-1.11	0.61
1474.00	1643.00	1687.33	-1.03	0.48
260.00	236.00	230.67	-1.05	0.59
423.00	391.00	405.33	-1.22	0.00
908.00	1042.00	1003.67	1.10	0.02
2.00	4.00	2.33	-2.80	0.02
68.00	71.00	64.33	-1.25	0.05

97.00	78.00	88.33	-1.34	0.00
351.00	408.00	406.67	1.05	0.44
2545.00	2683.00	2608.33	-1.04	0.32
661.00	511.00	559.00	-1.87	0.00
901.00	997.00	942.67	1.02	0.63
580.00	684.00	636.00	-1.25	0.00
44.00	52.00	46.00	-1.01	1.00
934.00	1102.00	1029.00	1.27	0.00
1174.00	1155.00	1177.33	-1.19	0.00
4414.00	4285.00	4270.67	-1.11	0.02
758.00	809.00	807.33	-1.10	0.03
241.00	258.00	249.00	1.19	0.01
201.00	242.00	235.67	1.51	0.00
1223.00	1031.00	1075.33	-1.12	0.09
1.00	1.00	0.67	-4.85	0.02
1048.00	1073.00	1032.33	1.13	0.02
133.00	145.00	148.00	1.12	0.18
405.00	475.00	449.67	1.05	0.38
155.00	171.00	161.33	1.20	0.02
73.00	55.00	66.33	-1.52	0.00
199.00	148.00	157.67	-1.26	0.05
129.00	167.00	147.33	-1.24	0.01
1495.00	1602.00	1615.00	-1.08	0.04
373.00	326.00	338.00	-1.01	0.96
565.00	615.00	571.67	-1.12	0.06
2070.00	2033.00	2045.67	-1.15	0.00
1376.00	1590.00	1494.67	1.28	0.00
0.00	0.00	0.00	-9.06	0.27
1223.00	1217.00	1218.67	-1.03	0.50
407.00	369.00	384.67	-1.18	0.00
1278.00	1359.00	1285.33	1.23	0.00
533.00	625.00	584.67	1.38	0.00
475.00	532.00	485.67	-1.08	0.21
23.00	16.00	22.33	-1.89	0.00
953.00	1168.00	1097.00	1.25	0.00
1170.00	1217.00	1241.33	1.25	0.00
1336.00	1414.00	1319.00	-1.10	0.08
4712.00	4857.00	4909.00	-1.02	0.64
622.00	686.00	667.67	1.18	0.00
1370.00	1519.00	1430.67	1.00	0.97
1262.00	1406.00	1296.33	1.03	0.57
659.00	601.00	624.00	-1.68	0.00
1472.00	1620.00	1589.33	1.21	0.00
1321.00	1636.00	1488.33	1.17	0.00
267.00	305.00	305.33	1.13	0.07
152.00	149.00	148.67	-1.16	0.11
1073.00	1094.00	1076.33	1.18	0.00
1564.00	1747.00	1700.67	1.22	0.00

641.00	814.00	696.67	-1.01	0.89
1281.00	1347.00	1309.33	1.01	0.77
667.00	587.00	617.33	1.13	0.06
1296.00	1552.00	1424.33	1.22	0.00
29.00	32.00	26.33	1.73	0.01
4436.00	4698.00	4404.00	-1.03	0.54
178.00	196.00	183.00	-1.39	0.00
46.00	49.00	49.00	-1.13	0.35
733.00	786.00	757.67	-1.23	0.00
149.00	162.00	167.33	1.12	0.22
46.00	34.00	36.33	-1.45	0.01
582.00	665.00	635.00	1.02	0.69
8.00	11.00	7.67	1.03	1.00
2357.00	2328.00	2456.67	-1.19	0.00
1895.00	1994.00	2037.33	-1.03	0.48
509.00	628.00	592.00	-1.01	0.87
5221.00	4900.00	4986.67	-1.14	0.00
20.00	34.00	25.00	-1.74	0.00
9.00	18.00	12.33	-5.01	0.00
187.00	222.00	200.33	1.02	0.85
381.00	424.00	428.33	-1.11	0.06
1865.00	1943.00	1958.33	1.04	0.29
2456.00	2562.00	2573.33	-1.04	0.21
653.00	653.00	659.33	-1.21	0.00
686.00	772.00	752.33	1.01	0.86
77.00	74.00	82.00	1.09	0.47
312.00	305.00	321.67	-1.10	0.09
25.00	25.00	31.00	-1.12	0.51
8.00	16.00	15.67	1.28	0.35
95.00	153.00	133.00	-1.08	0.43
1132.00	1205.00	1227.00	-1.01	0.81
606.00	540.00	590.33	1.80	0.00
1559.00	1691.00	1625.67	1.03	0.50
42.00	69.00	59.67	1.19	0.26
348.00	373.00	364.33	-1.12	0.03
30.00	49.00	41.33	1.08	0.64
2198.00	2372.00	2324.67	-1.02	0.52
1012.00	1029.00	1032.00	-1.09	0.06
265.00	258.00	264.00	1.07	0.32
33.00	45.00	39.67	1.00	1.00
1247.00	1292.00	1297.00	-1.07	0.11
557.00	673.00	611.67	1.01	0.93
1170.00	1225.00	1266.33	1.10	0.02
817.00	916.00	874.67	-1.04	0.38
18.00	19.00	15.67	-1.63	0.02
107.00	126.00	125.00	1.13	0.27
290.00	344.00	326.67	1.02	0.80
1424.00	1533.00	1525.67	1.08	0.04

9.00	33.00	21.33	1.06	0.89
28.00	17.00	26.33	1.89	0.00
220.00	250.00	239.00	1.09	0.26
1047.00	1027.00	1082.00	-1.09	0.06
789.00	695.00	759.33	1.07	0.22
354.00	391.00	361.67	1.04	0.51
571.00	583.00	581.00	-1.03	0.62
1853.00	1729.00	1777.67	-1.17	0.00
362.00	466.00	443.33	-1.07	0.32
18.00	16.00	17.33	1.46	0.10
416.00	440.00	407.00	-1.15	0.04
117.00	124.00	128.33	-1.06	0.51
148.00	136.00	157.67	-1.09	0.34
189.00	212.00	193.67	-1.03	0.74
996.00	1001.00	1021.33	-1.31	0.00
685.00	755.00	731.33	-1.19	0.00
49.00	64.00	59.33	-1.03	0.81
60.00	47.00	52.33	1.27	0.09
1513.00	1523.00	1526.00	-1.18	0.00
324.00	348.00	325.33	-1.10	0.24
12.00	16.00	15.00	-1.08	0.84
2228.00	2550.00	2432.33	-1.03	0.44
2899.00	3154.00	3022.00	1.03	0.53
771.00	859.00	825.67	1.04	0.35
46.00	41.00	46.00	1.04	0.82
385.00	421.00	407.67	1.11	0.07
2235.00	2408.00	2347.33	1.07	0.10
47.00	32.00	38.67	-1.38	0.03
11.00	16.00	14.00	1.42	0.17
3.00	8.00	7.67	1.03	1.00
73.00	92.00	85.33	1.09	0.39
34.00	17.00	29.67	-1.51	0.01
8.00	11.00	7.67	-1.74	0.07
2.00	1.00	1.33	-4.72	0.00
52.00	58.00	64.33	1.40	0.01
2954.00	2816.00	2807.00	-1.26	0.00
1936.00	1816.00	1870.00	-1.23	0.00
1307.00	1436.00	1350.00	-1.09	0.08
4718.00	3596.00	3923.00	-1.43	0.00
1055.00	1027.00	1016.67	-1.29	0.00
88.00	63.00	80.33	-1.08	0.45
471.00	462.00	461.00	1.23	0.00
609.00	723.00	714.00	1.22	0.00
1394.00	1532.00	1473.00	1.12	0.01
2165.00	2404.00	2354.67	1.00	0.98
3868.00	4211.00	4150.67	1.30	0.00
1232.00	1465.00	1367.67	1.25	0.00
3186.00	3579.00	3495.33	1.16	0.00

174.00	180.00	165.00	1.03	0.78
2150.00	2104.00	2150.00	-1.08	0.05
9437.00	9785.00	9651.00	1.07	0.05
2.00	7.00	5.00	1.06	1.00
266.00	334.00	296.00	1.04	0.51
865.00	997.00	925.00	1.02	0.74
313.00	325.00	328.67	-1.08	0.19
52.00	48.00	47.33	-1.59	0.00
635.00	731.00	752.00	1.02	0.70
674.00	854.00	786.67	1.17	0.00
250.00	230.00	244.33	-1.40	0.00
0.00	4.00	2.33	-1.98	0.20
1910.00	2012.00	2059.67	1.03	0.44
1209.00	1372.00	1303.00	-1.08	0.06
985.00	1146.00	1077.33	1.09	0.09
435.00	466.00	470.67	1.05	0.33
229.00	281.00	268.00	2.36	0.00
2951.00	3281.00	3311.00	-1.22	0.00
3205.00	3389.00	3474.00	-1.74	0.00
220.00	249.00	255.67	-1.35	0.00
788.00	838.00	865.67	-1.21	0.00
571.00	565.00	594.67	-1.30	0.00
8529.00	8811.00	9187.67	-1.88	0.00
12.00	14.00	13.67	-1.80	0.00
0.00	3.00	1.00	-5.51	0.00
85.00	71.00	88.00	-2.07	0.00
206.00	148.00	222.00	7.20	0.00
191.00	93.00	127.33	3.12	0.00
11.00	23.00	16.00	1.10	0.78
86.00	103.00	98.33	2.99	0.00
27.00	23.00	24.00	3.06	0.00
5.00	1.00	2.67	-2.22	0.08
2.00	2.00	1.33	-5.18	0.00
1338.00	1424.00	1489.33	1.12	0.02
37.00	39.00	41.00	1.03	0.86
406.00	375.00	395.33	-1.03	0.69
5890.00	5951.00	5823.00	1.04	0.35
44.00	59.00	55.67	-1.09	0.47
1617.00	1667.00	1758.00	1.03	0.54
5.00	0.00	4.00	1.07	1.00
3.00	1.00	1.67	-1.77	0.43
304.00	338.00	310.67	1.14	0.05
696.00	779.00	738.67	-1.23	0.00
267.00	255.00	259.33	-1.55	0.00
648.00	696.00	688.33	1.11	0.02
647.00	738.00	729.00	1.02	0.72
399.00	427.00	421.33	-1.03	0.52
129.00	99.00	110.33	1.15	0.23

974.00	1064.00	1023.33	1.00	1.00
539.00	624.00	599.67	1.19	0.00
10.00	9.00	7.33	-1.01	1.00
208.00	239.00	244.67	1.28	0.00
136.00	111.00	131.33	-1.09	0.33
18.00	14.00	22.67	1.48	0.08
445.00	418.00	416.00	-2.18	0.00
4922.00	5392.00	5158.67	-1.05	0.19
2764.00	2616.00	2712.33	-1.36	0.00
18755.00	20188.00	19862.33	-1.42	0.00
4847.00	5041.00	5093.00	-1.41	0.00
0.00	6.00	2.67	-1.62	0.39
24771.00	26014.00	25954.67	-1.13	0.00
6.00	6.00	6.33	1.33	0.49
19532.00	20479.00	20056.33	-1.05	0.20
1192.00	1215.00	1236.67	1.11	0.01
5596.00	6028.00	5850.67	-1.49	0.00
2911.00	3062.00	2959.00	-1.13	0.00
1584.00	1651.00	1758.00	1.01	0.87
543.00	645.00	613.67	1.20	0.00
386.00	452.00	442.67	1.21	0.00
698.00	776.00	791.67	1.06	0.28
919.00	1083.00	1084.33	1.05	0.37
14.00	12.00	12.67	1.95	0.02
1715.00	1775.00	1734.67	1.08	0.07
451.00	503.00	483.00	1.17	0.00
747.00	797.00	800.00	1.10	0.02
2986.00	3084.00	3138.67	-1.08	0.04
5666.00	5809.00	5882.67	1.10	0.02
10797.00	10641.00	10806.33	-1.01	0.85
327.00	366.00	340.00	1.01	0.83
1970.00	2281.00	2112.67	1.07	0.17
2461.00	2667.00	2653.00	1.05	0.16
1484.00	1558.00	1580.67	-1.04	0.33
699.00	837.00	805.00	1.10	0.07
10595.00	11497.00	11063.67	-1.01	0.74
1316.00	1409.00	1383.33	-1.03	0.44
2905.00	3142.00	3026.00	1.06	0.13
3282.00	3343.00	3388.67	-1.28	0.00
452.00	410.00	439.00	-1.53	0.00
180.00	192.00	194.33	5.12	0.00
725.00	756.00	750.33	-1.06	0.24
479.00	463.00	487.67	-1.00	0.97
849.00	854.00	848.33	1.33	0.00
713.00	713.00	745.00	1.28	0.00
2269.00	2467.00	2349.00	1.03	0.52
79.00	88.00	84.67	1.05	0.65
119.00	140.00	134.00	-1.08	0.37

121.00	132.00	133.00	-1.04	0.62
37.00	39.00	35.67	1.11	0.55
180.00	191.00	191.67	1.03	0.64
617.00	658.00	656.67	1.14	0.01
369.00	355.00	382.67	1.05	0.41
5.00	15.00	9.67	1.06	0.90
284.00	287.00	293.00	-1.02	0.82
534.00	561.00	533.33	-1.08	0.22
114.00	137.00	125.33	-1.09	0.29
59.00	46.00	49.33	-1.52	0.00
2.00	1.00	1.00	-1.61	0.73
117.00	100.00	108.00	1.65	0.00
578.00	617.00	612.33	1.02	0.63
1419.00	1365.00	1452.33	-1.28	0.00
10915.00	12490.00	12145.00	1.48	0.00
19473.00	21396.00	21481.67	1.28	0.00
95.00	96.00	101.33	1.47	0.00
15.00	13.00	16.00	1.21	0.41
1315.00	1281.00	1284.33	-1.57	0.00
1443.00	1239.00	1318.33	1.30	0.00
269.00	276.00	293.33	-1.01	0.88
577.00	564.00	545.67	-1.20	0.00
47.00	56.00	56.33	1.02	0.92
311.00	297.00	320.00	1.31	0.00
7.00	10.00	9.67	1.06	0.90
2086.00	2061.00	2135.33	-1.10	0.01
813.00	883.00	806.33	-1.05	0.40
112.00	122.00	104.67	-1.56	0.00
12.00	14.00	13.00	1.47	0.15
695.00	825.00	771.67	1.17	0.00
353.00	409.00	386.33	1.07	0.23
2188.00	2344.00	2302.00	1.04	0.32
827.00	861.00	860.33	1.12	0.02
2576.00	2728.00	2766.67	-1.03	0.34
560.00	616.00	622.67	1.11	0.03
2186.00	2463.00	2413.67	-1.07	0.06
25.00	29.00	29.67	-1.39	0.03
5774.00	6785.00	6849.33	1.08	0.13
749.00	782.00	828.00	-1.07	0.16
15193.00	16346.00	16194.67	-1.15	0.00
927.00	1014.00	997.67	-1.17	0.00
1.00	7.00	3.33	1.22	0.82
91.00	103.00	105.00	1.22	0.05
1.00	0.00	0.67	-3.58	0.12
1417.00	1467.00	1488.33	1.07	0.14
221.00	252.00	230.33	1.38	0.00
458.00	471.00	462.33	-1.53	0.00
0.00	2.00	0.67	-3.14	0.19

23.00	21.00	22.67	1.03	0.93
337.00	354.00	365.00	-1.32	0.00
1306.00	1339.00	1359.33	-1.03	0.48
115.00	114.00	119.00	1.05	0.58
6.00	3.00	3.33	1.23	0.82
679.00	659.00	712.33	-1.12	0.03
2.00	3.00	2.33	-1.56	0.49
965.00	1048.00	1028.00	-1.01	0.82
7802.00	8056.00	8221.67	-1.13	0.00
590.00	590.00	583.33	-1.43	0.00
9.00	12.00	15.00	-1.24	0.34
888.00	955.00	924.00	1.19	0.00
840.00	730.00	779.33	-1.09	0.14
465.00	592.00	534.00	-1.07	0.28
1095.00	1084.00	1130.00	-1.13	0.00
5.00	0.00	2.00	-1.01	1.00
2072.00	1967.00	2038.67	-1.04	0.32
10.00	6.00	7.33	1.27	0.53
568.00	609.00	631.00	-1.08	0.15
992.00	1116.00	1085.33	-1.03	0.52
1477.00	1407.00	1465.67	-1.35	0.00
316.00	315.00	327.67	1.06	0.32
1009.00	1111.00	1056.67	1.26	0.00
86.00	72.00	76.33	1.15	0.20
195.00	213.00	224.67	1.12	0.15
196.00	228.00	211.33	1.10	0.18
259.00	276.00	267.00	1.16	0.02
48.00	76.00	63.00	-1.18	0.16
440.00	548.00	507.33	1.30	0.00
261.00	318.00	292.33	1.26	0.00
627.00	629.00	656.33	-1.03	0.56
23.00	31.00	24.67	-1.20	0.34
222.00	248.00	267.33	1.15	0.10
240.00	248.00	263.67	-1.13	0.06
61.00	54.00	60.67	1.38	0.01
636.00	713.00	703.67	-1.16	0.00
78.00	73.00	77.00	-1.28	0.01
8.00	13.00	12.00	1.22	0.47
44.00	58.00	52.67	1.00	1.00
20.00	18.00	17.33	1.07	0.84
16.00	19.00	21.67	1.03	1.00
15.00	18.00	17.67	1.45	0.10
3.00	2.00	3.00	-1.66	0.31
337.00	362.00	356.33	-1.61	0.00
241.00	254.00	238.67	1.02	0.76
1.00	3.00	1.67	-1.77	0.43
5.00	5.00	4.67	-1.08	1.00
331.00	352.00	367.00	-1.07	0.21

2.00	2.00	2.00	-1.17	1.00
16.00	22.00	22.00	1.96	0.00
5.00	5.00	5.00	1.23	0.71
1.00	3.00	2.67	-1.13	1.00
7.00	5.00	6.00	1.36	0.48
8.00	3.00	5.67	-1.71	0.11
52.00	67.00	61.00	1.73	0.00
8.00	3.00	6.33	-1.43	0.31
307.00	330.00	346.33	1.11	0.13
114.00	142.00	147.00	-1.27	0.01
360.00	381.00	380.67	-1.88	0.00
187.00	207.00	205.00	-1.35	0.00
7.00	14.00	12.67	1.20	0.57
4.00	6.00	5.33	-1.45	0.35
5.00	3.00	3.33	1.39	0.64
7.00	2.00	4.33	-1.01	1.00
6.00	3.00	3.33	1.39	0.64
36.00	44.00	35.67	1.29	0.12
171.00	169.00	184.00	1.22	0.01
109.00	133.00	124.67	-1.06	0.48
54.00	48.00	51.00	-1.05	0.75
486.00	578.00	525.33	1.11	0.07
1.00	2.00	1.33	-1.01	1.00
86.00	94.00	91.67	1.01	0.91
0.00	0.00	0.00	-11.75	0.13
633.00	730.00	756.33	1.21	0.01
0.00	5.00	2.67	1.89	0.40
16.00	9.00	14.33	1.28	0.32
2.00	4.00	2.33	-2.12	0.15
83.00	108.00	96.67	1.11	0.29
0.00	0.00	0.00	-14.44	0.07
7.00	2.00	5.67	-1.13	0.87
576.00	629.00	646.33	-1.22	0.00
3.00	1.00	2.00	-1.17	1.00
7.00	10.00	13.67	-1.12	0.69
8.00	8.00	5.33	-1.25	0.69
39.00	54.00	45.33	1.01	1.00
2.00	1.00	1.00	-2.21	0.35
45.00	49.00	46.00	-1.26	0.07
114.00	120.00	130.67	-1.16	0.08
73.00	83.00	74.00	1.03	0.84
60.00	66.00	65.67	-1.31	0.01
69.00	54.00	60.00	-1.52	0.00
2.00	1.00	2.33	-1.29	0.81
110.00	133.00	118.67	-1.25	0.01
8.00	10.00	8.67	-1.32	0.38
443.00	486.00	432.67	-1.40	0.00
79.00	96.00	92.00	1.31	0.01

3.00	4.00	3.67	-1.01	1.00
95.00	97.00	114.67	-1.20	0.08
19.00	18.00	16.33	1.21	0.42
10003.00	10458.00	10021.33	1.01	0.75
1.00	1.00	3.00	2.72	0.18
87.00	99.00	99.67	1.05	0.68
23.00	18.00	25.67	-1.24	0.26
47.00	38.00	42.00	1.12	0.46
1336.00	1421.00	1434.67	-1.05	0.22
11.00	21.00	16.67	1.33	0.26
7.00	10.00	10.33	-1.50	0.12
31.00	32.00	35.67	-1.68	0.00
617.00	644.00	663.33	-1.30	0.00
0.00	0.00	0.00	-9.06	0.27
12.00	15.00	12.67	1.07	0.82
2.00	0.00	0.67	-1.86	0.69
32.00	29.00	31.33	-1.48	0.01
1843.00	1976.00	2005.33	-1.65	0.00
13.00	16.00	14.00	1.38	0.21
1746.00	1918.00	1923.33	2.13	0.00
927.00	979.00	988.67	1.08	0.09
11.00	9.00	11.67	-2.05	0.00
197.00	227.00	222.33	-1.14	0.06
36.00	33.00	35.67	-1.30	0.07
6.00	22.00	14.00	-1.11	0.70
28.00	18.00	27.33	-1.41	0.03
10.00	10.00	10.00	1.18	0.60
36.00	43.00	40.67	-1.25	0.09
4.00	7.00	6.67	-1.06	1.00
9.00	14.00	14.00	-1.11	0.68
195.00	231.00	222.00	1.02	0.80
28.00	35.00	29.33	1.01	1.00
118.00	104.00	123.00	-1.25	0.01
6.00	8.00	6.00	-1.12	0.87
17.00	13.00	14.33	-1.34	0.21
75.00	72.00	80.00	-1.25	0.02
1.00	8.00	5.67	-1.83	0.07
158.00	151.00	167.33	-1.06	0.45
221.00	303.00	280.67	1.18	0.03
70.00	54.00	65.33	-1.07	0.61
32.00	65.00	55.33	1.39	0.04
51.00	53.00	53.00	1.48	0.00
36.00	56.00	49.33	1.03	0.91
41.00	41.00	44.67	-1.07	0.61
41.00	20.00	35.00	1.00	1.00
17.00	19.00	15.67	-1.65	0.01
203.00	243.00	230.00	1.00	0.98
372.00	414.00	412.00	1.12	0.06

125.00	112.00	116.67	1.33	0.00
6.00	9.00	6.00	-1.73	0.10
14.00	7.00	10.33	1.22	0.53
55.00	47.00	53.00	1.31	0.05
29.00	37.00	31.67	1.05	0.83
263.00	273.00	276.00	-1.16	0.01
0.00	1.00	0.67	-2.72	0.29
127.00	169.00	159.67	1.06	0.50
886.00	1043.00	1002.33	1.16	0.00
138.00	153.00	149.00	-1.43	0.00
2861.00	2704.00	2767.33	-1.27	0.00
57.00	82.00	70.67	1.17	0.19
463.00	568.00	508.33	-1.30	0.00
56.00	74.00	68.33	1.35	0.01
11.00	2.00	6.00	1.18	0.74
93.00	112.00	97.33	-1.16	0.11
14.00	21.00	22.00	1.32	0.23
76.00	69.00	75.33	1.11	0.36
18.00	16.00	20.33	-1.23	0.26
6.00	8.00	7.00	1.15	0.75
0.00	2.00	1.67	-2.15	0.22
245.00	227.00	238.33	-1.31	0.00
2.00	0.00	1.67	-3.09	0.03
9.00	6.00	6.67	-1.01	1.00
522.00	575.00	567.33	-1.13	0.01
16.00	23.00	22.67	-1.72	0.00
3.00	6.00	4.33	-1.17	0.85
2.00	1.00	2.67	-1.86	0.22
64.00	61.00	68.00	1.20	0.14
402.00	471.00	450.33	1.21	0.00
37.00	38.00	41.00	1.05	0.78
7.00	8.00	8.00	-1.01	1.00
32.00	41.00	39.33	-1.08	0.59
1.00	5.00	2.00	-1.49	0.61
24.00	25.00	28.67	-1.20	0.27
115.00	77.00	88.67	-1.56	0.00
218.00	237.00	234.33	-1.25	0.00
6.00	6.00	4.67	-1.93	0.07
6.00	10.00	8.33	1.07	0.89
39.00	29.00	42.67	1.08	0.65
107.00	142.00	130.00	-1.42	0.00
212.00	252.00	254.00	1.04	0.66
11.00	21.00	21.00	-1.08	0.75
36.00	50.00	43.67	-1.11	0.47
0.00	3.00	1.33	-1.48	0.77
15.00	14.00	19.00	1.47	0.10
2.00	4.00	4.00	-1.42	0.47
15.00	21.00	15.00	-1.15	0.63

349.00	442.00	409.33	1.14	0.03
0.00	2.00	0.67	-5.27	0.01
318.00	385.00	357.67	-1.03	0.55
2.00	0.00	1.33	-1.24	1.00
16.00	8.00	12.67	-1.14	0.67
127.00	164.00	165.00	1.24	0.03
47.00	51.00	60.33	-1.59	0.00
54.00	61.00	64.67	1.03	0.82
208.00	197.00	197.00	2.01	0.00
3.00	4.00	3.33	-2.09	0.08
925.00	869.00	937.00	-1.12	0.02
547.00	583.00	562.00	1.55	0.00
34.00	24.00	31.67	1.20	0.29
122.00	124.00	127.33	-1.28	0.00
12.00	19.00	15.33	1.11	0.68
97.00	110.00	103.33	-1.02	0.83
12.00	18.00	15.00	-1.06	0.92
130.00	120.00	137.00	-1.04	0.62
0.00	1.00	0.33	-2.48	0.64
2.00	3.00	2.33	-1.56	0.49
12.00	18.00	16.00	1.13	0.61
2.00	4.00	3.00	-1.55	0.41
6.00	0.00	2.67	-1.13	1.00
532.00	729.00	634.67	-1.07	0.28
8.00	6.00	9.00	-1.72	0.04
131.00	138.00	138.00	1.93	0.00
7.00	2.00	4.33	-1.92	0.08
17.00	12.00	15.00	-1.44	0.08
1.00	3.00	2.00	-1.49	0.61
0.00	3.00	1.67	-1.77	0.43
50.00	52.00	53.33	-1.61	0.00
323.00	362.00	364.33	-1.38	0.00
598.00	647.00	664.00	1.07	0.17
17.00	24.00	22.67	-1.08	0.70
17.00	27.00	23.00	1.02	1.00
296.00	317.00	319.67	1.01	0.94
9.00	9.00	8.67	1.16	0.67
2.00	1.00	2.67	-2.95	0.01
7.00	16.00	12.67	2.06	0.01
355.00	414.00	413.67	1.00	0.98
4.00	9.00	7.33	-1.74	0.06
8.00	11.00	10.00	1.02	1.00
41.00	53.00	44.00	1.09	0.56
5.00	7.00	8.00	-1.47	0.21
58.00	64.00	62.67	-1.27	0.02
5.00	0.00	1.67	1.59	0.75
0.00	0.00	0.00	-14.45	0.07
17.00	14.00	13.00	-1.35	0.23

32.00	22.00	30.67	-1.93	0.00
115.00	136.00	120.00	1.37	0.00
3.00	5.00	3.67	-1.55	0.35
10.00	7.00	7.33	-1.69	0.08
37.00	28.00	31.67	-1.07	0.74
2.00	1.00	1.67	-1.39	0.78
6.00	4.00	5.33	1.31	0.58
8.00	8.00	7.33	-1.60	0.12
70.00	58.00	68.00	1.05	0.68
4.00	1.00	3.33	-1.40	0.55
57.00	55.00	60.00	1.11	0.39
1.00	3.00	2.33	-1.70	0.37
112.00	127.00	125.00	1.01	0.91
58.00	46.00	54.00	-1.12	0.37
61.00	85.00	77.33	1.31	0.02
505.00	588.00	586.33	1.25	0.00
1122.00	1202.00	1182.33	-1.34	0.00
38.00	24.00	34.67	-1.22	0.22
47.00	83.00	62.33	-1.00	1.00
3.00	0.00	2.00	-4.82	0.00
10.00	5.00	7.33	-1.06	1.00
1.00	2.00	2.33	-1.01	1.00
205.00	179.00	203.67	-1.19	0.02
655.00	734.00	740.67	-1.15	0.01
10.00	10.00	10.33	-1.11	0.81
957.00	1054.00	1019.33	-1.20	0.00
5.00	2.00	3.67	-2.35	0.02
30.00	42.00	37.00	-1.49	0.01
218.00	263.00	244.67	-1.05	0.53
3339.00	3524.00	3500.67	-1.08	0.03
18.00	19.00	20.33	1.09	0.72
14.00	15.00	17.00	1.12	0.69
245.00	275.00	270.00	-1.05	0.42
19.00	24.00	25.33	2.32	0.00
50.00	54.00	61.00	1.29	0.05
1115.00	1095.00	1134.33	1.08	0.06
1206.00	1186.00	1246.33	-1.19	0.00
23.00	27.00	26.67	-1.12	0.54
534.00	625.00	593.67	1.16	0.00
5.00	12.00	10.33	-1.18	0.64
6175.00	6519.00	6223.00	-1.06	0.21
4370.00	4244.00	4425.00	-1.37	0.00
767.00	776.00	784.00	-1.12	0.02
1257.00	1303.00	1249.67	1.05	0.26
995.00	970.00	1026.00	-1.25	0.00
586.00	685.00	658.67	-1.04	0.36
311.00	338.00	283.00	-1.11	0.38
50.00	66.00	58.33	-1.14	0.31

1761.00	1794.00	1842.67	-1.10	0.02
731.00	795.00	780.67	-1.11	0.02
23.00	17.00	20.00	1.18	0.41
3624.00	3908.00	3831.33	1.10	0.01
1249.00	1403.00	1313.00	1.54	0.00
6471.00	6017.00	6278.33	-1.14	0.00
1.00	1.00	1.00	-2.51	0.23
1875.00	1989.00	2047.33	1.21	0.00
901.00	1008.00	952.00	-1.09	0.04
3.00	2.00	2.00	-1.17	1.00
1013.00	1047.00	973.33	-1.23	0.00
142.00	165.00	153.33	1.36	0.00
286.00	259.00	250.33	-1.40	0.00
26.00	53.00	40.67	1.14	0.46
132.00	213.00	168.00	-1.09	0.42
3.00	2.00	2.33	2.16	0.35
195.00	179.00	192.00	-1.01	0.91
7.00	7.00	9.00	2.02	0.04
6.00	8.00	7.33	1.14	0.76
5.00	10.00	6.00	-2.06	0.02
44.00	58.00	56.67	-1.23	0.09
36.00	35.00	38.33	-1.02	0.90
2.00	8.00	5.00	-1.54	0.28
37.00	47.00	47.00	-1.61	0.00
8.00	12.00	10.00	-1.48	0.14
0.00	3.00	1.67	-1.01	1.00
6.00	3.00	3.67	-2.79	0.00
16.00	22.00	16.67	-1.37	0.14
1389.00	1281.00	1333.00	-1.38	0.00
14.00	17.00	15.33	-1.73	0.01
1.00	2.00	1.33	-2.40	0.19
72.00	56.00	62.33	-1.36	0.01
1.00	2.00	3.33	1.38	0.64
73.00	75.00	75.00	-1.27	0.02
326.00	302.00	311.67	-1.21	0.00
34.00	32.00	32.33	1.18	0.35
78.00	48.00	53.00	-1.71	0.00
3.00	6.00	3.67	-1.37	0.56
87.00	89.00	89.33	-1.41	0.00
3.00	2.00	2.33	-2.27	0.15
167.00	199.00	189.00	-1.07	0.32
25.00	27.00	25.33	-1.41	0.03
244.00	235.00	230.67	-1.05	0.53
68.00	74.00	67.67	-1.00	1.00
22.00	31.00	30.33	1.02	1.00
26.00	28.00	25.33	-1.04	0.94
1475.00	1578.00	1613.67	-1.26	0.00
14.00	8.00	11.67	1.02	1.00

17.00	10.00	12.00	-1.12	0.74
21.00	13.00	20.00	-1.06	0.73
1.00	1.00	1.67	-2.52	0.11
0.00	0.00	0.00	-9.06	0.27
76.00	82.00	80.00	-1.07	0.54
62.00	83.00	74.67	-1.14	0.21
2.00	5.00	3.67	-2.70	0.01
7.00	5.00	7.00	-2.01	0.01
37.00	32.00	36.33	-1.13	0.39
18.00	13.00	15.00	1.03	0.92
27.00	30.00	23.33	-1.66	0.00
150.00	195.00	190.00	-1.31	0.00
193.00	212.00	224.33	1.02	0.80
22.00	24.00	24.67	1.06	0.81
100.00	125.00	114.00	-1.09	0.30
59.00	76.00	61.00	-1.15	0.27
61.00	51.00	65.00	1.04	0.83
32.00	53.00	40.33	-2.32	0.00
73.00	56.00	76.33	-1.24	0.10
4.00	6.00	4.67	-1.58	0.25
5.00	15.00	8.33	1.44	0.31
39.00	37.00	35.33	-1.59	0.00
302.00	310.00	330.00	-1.32	0.00
32.00	31.00	31.00	1.76	0.00
86.00	92.00	90.00	1.24	0.03
15.00	23.00	18.33	1.01	1.00
21.00	34.00	28.33	-1.19	0.30
30.00	42.00	35.33	1.34	0.08
5.00	19.00	10.00	1.28	0.48
75.00	95.00	95.33	-1.01	0.98
6.00	14.00	12.33	-1.07	0.83
3.00	1.00	1.67	-1.95	0.31
17.00	14.00	13.67	-1.18	0.60
33.00	33.00	37.33	1.41	0.03
26.00	32.00	29.00	-1.32	0.07
0.00	2.00	1.00	-3.71	0.04
21.00	37.00	32.33	-1.39	0.03
8.00	11.00	15.00	1.38	0.26
14.00	17.00	16.00	1.52	0.08
52.00	53.00	49.67	-1.12	0.41
70.00	84.00	87.67	1.12	0.37
80.00	79.00	79.00	1.29	0.02
56.00	32.00	47.33	-1.12	0.43
2.00	4.00	3.00	-1.55	0.41
64.00	73.00	68.00	1.31	0.02
37.00	35.00	35.67	-1.12	0.45
5.00	11.00	7.33	-2.37	0.00
36.00	26.00	28.00	-1.17	0.38

3.00	6.00	3.67	-1.46	0.45
6.00	4.00	6.33	-1.33	0.47
13.00	22.00	17.00	1.43	0.13
35.00	25.00	26.00	1.15	0.45
3.00	1.00	1.67	-2.14	0.22
3.00	4.00	4.00	-1.01	1.00
20.00	27.00	25.33	-1.08	0.68
30.00	28.00	32.00	1.39	0.06
35.00	39.00	40.67	-1.14	0.35
8.00	1.00	3.00	1.74	0.50
309.00	342.00	309.33	-1.17	0.04
30.00	27.00	29.33	1.21	0.29
90.00	121.00	104.67	1.93	0.00
0.00	2.00	0.67	-7.82	0.00
29.00	32.00	30.67	1.33	0.11
1.00	0.00	1.00	-7.01	0.00
169.00	195.00	195.33	-1.12	0.12
3.00	3.00	5.33	1.30	0.58
31.00	40.00	41.00	1.01	1.00
47.00	47.00	48.00	-1.10	0.45
2515.00	2887.00	2776.67	-1.06	0.12
234.00	212.00	233.67	-1.12	0.15
29.00	64.00	42.33	-1.07	0.72
190.00	149.00	165.00	-1.18	0.06
79.00	74.00	72.33	1.03	0.80
37.00	29.00	36.00	-1.32	0.05
13.00	20.00	15.00	1.70	0.04
20.00	25.00	20.33	1.00	1.00
135.00	160.00	155.00	1.08	0.39
24.00	31.00	29.33	-2.12	0.00
30.00	25.00	29.33	-1.23	0.17
7.00	5.00	5.00	-2.00	0.04
81.00	87.00	95.00	1.01	0.95
1244.00	1237.00	1277.00	-1.44	0.00
5.00	6.00	6.00	1.46	0.37
13.00	21.00	18.00	-1.48	0.04
9.00	7.00	10.67	1.16	0.64
28.00	21.00	27.00	-1.33	0.07
27.00	45.00	31.67	-1.27	0.14
193.00	172.00	204.00	-1.08	0.36
14.00	18.00	14.00	-2.21	0.02
79.00	87.00	92.33	1.01	0.94
44.00	20.00	29.33	-1.13	0.53
51.00	65.00	69.00	1.03	0.84
76.00	97.00	94.67	-1.08	0.48
50.00	46.00	47.00	1.12	0.42
10.00	15.00	10.67	-1.39	0.23
9.00	14.00	15.67	-1.59	0.02

278.00	275.00	287.00	-1.02	0.69
10.00	5.00	11.33	1.66	0.10
25.00	28.00	23.67	1.07	0.77
5.00	7.00	9.33	-1.19	0.63
34.00	47.00	42.67	1.23	0.15
25.00	22.00	20.67	-1.25	0.28
2.00	5.00	3.00	1.26	0.81
131.00	111.00	112.33	1.37	0.00
3.00	0.00	1.00	-1.61	0.74
14.00	10.00	15.00	-1.08	0.85
12.00	5.00	9.67	1.19	0.59
3.00	5.00	4.33	-2.45	0.01
27.00	21.00	23.33	-1.24	0.27
2464.00	2164.00	2367.33	-1.16	0.00
8.00	14.00	13.67	1.34	0.30
3.00	5.00	5.33	1.56	0.34
2.00	8.00	6.67	1.31	0.51
26.00	21.00	23.67	-1.14	0.49
4.00	7.00	7.00	-1.54	0.18
43.00	45.00	41.67	-1.11	0.45
86.00	75.00	87.33	-1.13	0.23
4.00	6.00	5.00	-1.54	0.27
197.00	183.00	212.33	1.19	0.04
463.00	579.00	510.00	-1.24	0.00
208.00	258.00	228.33	-1.41	0.00
1.00	2.00	1.00	-2.21	0.36
95.00	113.00	108.33	-1.22	0.02
6.00	8.00	6.67	1.23	0.62
11.00	26.00	18.33	-1.51	0.03
1.00	5.00	2.00	-5.46	0.00
1.00	1.00	2.33	1.66	0.56
4.00	5.00	4.00	-1.34	0.58
116.00	135.00	119.67	-1.01	0.98
9.00	5.00	6.67	-1.26	0.56
59.00	43.00	50.33	-1.27	0.06
2.00	1.00	1.67	-2.52	0.10
23.00	33.00	23.33	-1.04	0.95
16.00	41.00	32.67	-2.13	0.00
7.00	6.00	6.00	1.18	0.73
0.00	1.00	0.33	-5.42	0.07
10.00	7.00	10.67	-1.42	0.19
7.00	13.00	8.33	-1.09	0.89
0.00	4.00	2.67	-1.98	0.16
61.00	66.00	64.67	1.22	0.09
14.00	17.00	16.33	-1.40	0.11
17.00	42.00	26.00	-1.39	0.08
1.00	5.00	5.33	-1.45	0.37
24.00	27.00	22.67	1.37	0.13

287.00	298.00	310.67	2.18	0.00
3.00	4.00	5.00	-1.21	0.73
48.00	47.00	48.33	-1.14	0.31
2.00	1.00	2.00	-1.49	0.61
42.00	27.00	37.00	1.37	0.06
2.00	1.00	3.33	1.22	0.82
0.00	1.00	1.00	-3.41	0.06
1.00	6.00	2.67	-1.98	0.18
10.00	14.00	15.67	1.71	0.04
99.00	123.00	119.00	1.29	0.01
2.00	13.00	8.00	1.03	1.00
5.00	1.00	2.67	-1.25	0.82
32.00	41.00	39.00	-1.14	0.38
12.00	15.00	10.67	1.26	0.45
16.00	8.00	12.33	-1.96	0.00
21.00	13.00	15.00	1.23	0.40
29.00	24.00	27.33	1.87	0.00
29.00	18.00	27.67	-1.37	0.06
68.00	90.00	79.00	1.34	0.01
1.00	2.00	3.67	-1.02	1.00
24.00	18.00	21.67	1.07	0.73
3.00	3.00	6.00	-1.29	0.62
45.00	61.00	48.00	-1.01	0.96
20.00	11.00	16.33	1.01	1.00
99.00	114.00	109.33	-1.12	0.20
155.00	137.00	148.00	-1.01	0.94
10.00	15.00	12.33	1.40	0.22
4.00	8.00	6.67	1.31	0.51
13.00	27.00	18.33	-1.38	0.12
13.00	12.00	13.67	-1.16	0.60
35.00	43.00	38.67	1.33	0.07
9.00	10.00	11.00	1.47	0.19
3.00	8.00	4.33	-2.07	0.05
4.00	8.00	6.33	-1.17	0.76
4.00	3.00	3.33	-1.99	0.10
1.00	6.00	5.00	-1.21	0.73
1.00	0.00	0.33	-3.22	0.38
4.00	1.00	3.67	-1.37	0.57
9.00	6.00	9.33	1.02	1.00
4.00	2.00	2.67	-1.13	1.00
126.00	139.00	146.33	-1.07	0.43
1.00	3.00	3.00	1.45	0.61
24.00	26.00	20.33	1.26	0.30
15.00	7.00	10.33	-1.36	0.34
3.00	2.00	2.33	-1.56	0.49
44.00	60.00	54.67	1.31	0.04
5.00	16.00	9.33	1.45	0.27
10.00	8.00	11.33	-1.81	0.01

0.00	1.00	0.33	-3.22	0.39
1.00	4.00	4.00	1.18	0.84
9.00	8.00	7.67	1.13	0.77
33.00	31.00	28.00	-1.20	0.30
7.00	1.00	4.00	-1.59	0.29
3.00	1.00	1.67	-1.76	0.43
28.00	22.00	22.33	-1.37	0.07
4.00	8.00	4.33	-1.46	0.40
12.00	8.00	10.00	-1.81	0.01
4.00	6.00	4.00	-1.66	0.23
24.00	32.00	31.33	1.34	0.11
18.00	16.00	17.67	-1.30	0.22
29.00	17.00	24.33	1.56	0.02
682.00	746.00	737.00	1.07	0.16
0.00	2.00	0.67	-1.44	1.00
4.00	9.00	7.00	-1.11	0.88
31.00	20.00	27.67	-1.35	0.06
34.00	27.00	30.67	-1.22	0.18
7.00	14.00	8.67	-1.09	0.90
30.00	32.00	31.33	-1.14	0.39
82.00	62.00	76.00	2.22	0.00
0.00	0.00	0.33	-3.96	0.23
0.00	5.00	2.67	-1.14	1.00
1.00	1.00	3.33	1.38	0.65
10.00	5.00	8.33	1.63	0.17
2.00	5.00	4.00	-1.26	0.71
11.00	6.00	7.67	-1.32	0.42
21.00	19.00	20.00	-3.21	0.00
195.00	208.00	209.67	-1.14	0.04
11.00	2.00	6.00	-1.34	0.48
41.00	59.00	53.67	1.01	1.00
20.00	23.00	22.67	1.03	1.00
4.00	8.00	4.33	-1.92	0.08
10.00	6.00	8.33	1.07	0.89
68.00	75.00	71.67	-1.28	0.02
6.00	3.00	6.00	-1.73	0.09
22.00	26.00	23.00	1.10	0.68
29.00	19.00	25.00	1.16	0.47
20.00	23.00	22.33	1.61	0.02
371.00	455.00	430.00	1.10	0.12
25.00	16.00	21.33	-1.61	0.01
0.00	7.00	4.00	1.30	0.68
3.00	12.00	7.33	-1.06	1.00
9.00	9.00	7.33	1.54	0.25
19.00	10.00	15.33	-1.58	0.03
6.00	8.00	6.33	-1.12	0.88
2.00	5.00	2.33	-2.11	0.14
1.00	0.00	1.33	-1.01	1.00

9.00	12.00	9.67	2.17	0.02
182.00	227.00	206.33	1.42	0.00
44.00	48.00	54.33	1.06	0.73
19.00	29.00	25.00	-1.08	0.65
2.00	4.00	3.33	-1.50	0.43
24.00	19.00	19.67	1.00	1.00
7.00	8.00	8.67	2.78	0.01
216.00	258.00	237.00	1.04	0.62
428.00	433.00	448.00	-1.65	0.00
1.00	2.00	1.67	-1.96	0.31
26.00	25.00	24.67	-1.43	0.04
334.00	375.00	360.33	-1.22	0.00
186.00	189.00	202.33	-1.41	0.00
74.00	93.00	88.67	1.13	0.26
1.00	2.00	2.00	-1.33	0.79
9.00	17.00	14.67	1.24	0.46
1.00	9.00	6.33	-1.85	0.05
3.00	1.00	3.00	-1.34	0.67
16.00	16.00	17.33	-1.09	0.78
14.00	17.00	14.33	1.28	0.32
7.00	7.00	7.33	-1.38	0.34
304.00	300.00	315.67	-1.32	0.00
3.00	3.00	4.67	-1.30	0.61
7.00	4.00	4.67	-1.30	0.61
7.00	10.00	8.33	-1.17	0.69
61.00	44.00	54.00	1.13	0.38
1691.00	1900.00	1907.00	-1.12	0.01
565.00	643.00	635.33	-1.04	0.38
850.00	875.00	887.00	-1.13	0.00
151.00	190.00	171.67	-1.01	0.86
84.00	69.00	76.00	-1.33	0.00
6.00	6.00	4.67	1.25	0.70
811.00	759.00	784.33	-1.44	0.00
171.00	183.00	192.33	-1.13	0.10
71.00	56.00	62.67	-1.33	0.01
434.00	490.00	463.67	-1.17	0.00
44.00	57.00	61.67	-1.13	0.35
26.00	47.00	37.67	2.70	0.00
209.00	227.00	229.67	1.06	0.44
3.00	12.00	6.33	1.68	0.22
10.00	7.00	7.67	-1.97	0.01
12.00	16.00	13.67	-1.06	0.92
3.00	2.00	1.67	-1.58	0.59
2.00	4.00	3.00	-2.53	0.02
8.00	9.00	8.00	1.24	0.55
391.00	391.00	408.00	-1.12	0.05
146.00	133.00	135.67	-1.14	0.12
30.00	43.00	35.33	1.26	0.17

181.00	169.00	186.00	1.37	0.00
88.00	100.00	104.67	-1.80	0.00
656.00	725.00	720.33	-1.07	0.17
378.00	461.00	426.33	-1.23	0.00
10.00	7.00	10.67	-1.36	0.28
0.00	0.00	0.00	-17.09	0.03
162.00	196.00	171.67	-1.15	0.10
15.00	18.00	16.33	1.05	0.84
25.00	19.00	22.33	-1.10	0.71
217.00	198.00	206.00	-1.09	0.30
307.00	286.00	304.33	1.19	0.01
6.00	15.00	11.33	1.19	0.54
97.00	110.00	101.67	-1.03	0.79
280.00	279.00	300.00	-1.06	0.35
229.00	230.00	239.00	-1.19	0.02
1.00	4.00	2.33	-1.01	1.00
6.00	9.00	6.67	-1.46	0.26
77.00	87.00	78.33	1.20	0.10
41.00	34.00	38.33	-1.23	0.19
48.00	45.00	48.33	-1.12	0.36
95.00	121.00	128.00	-1.01	0.90
374.00	395.00	379.00	-1.14	0.02
136.00	150.00	148.00	1.13	0.11
11.00	8.00	9.00	-1.05	1.00
31.00	18.00	27.00	-1.11	0.58
383.00	478.00	426.33	1.35	0.00
514.00	503.00	511.67	-1.23	0.00
53.00	52.00	58.67	1.17	0.27
0.00	0.00	0.00	-9.06	0.27
6.00	4.00	5.00	-2.53	0.00
11.00	14.00	11.33	1.29	0.38
6.00	5.00	6.00	-1.95	0.03
305.00	326.00	315.67	-1.10	0.11
8.00	3.00	5.00	-1.14	0.86
11.00	5.00	9.67	1.19	0.59
43.00	62.00	49.00	-1.07	0.71
6.00	9.00	8.33	-1.57	0.14
34.00	35.00	30.00	1.31	0.12
24.00	21.00	20.67	-1.16	0.46
9.00	9.00	9.00	1.11	0.78
11.00	17.00	15.00	1.34	0.23
3.00	4.00	5.00	1.81	0.22
34.00	29.00	36.67	1.20	0.27
137.00	150.00	143.67	1.01	0.91
47.00	58.00	47.00	-1.04	0.79
6.00	6.00	8.00	1.38	0.36
0.00	0.00	0.67	-2.29	0.47
4.00	6.00	5.33	-1.57	0.23

18.00	22.00	24.33	-1.28	0.14
5.00	32.00	40.00	2.31	0.02
38.00	50.00	46.33	1.22	0.16
31.00	38.00	30.33	-1.15	0.43
15.00	30.00	21.33	1.15	0.55
637.00	729.00	705.33	-1.05	0.29
1968.00	1965.00	1966.67	-1.26	0.00
17.00	14.00	18.67	1.15	0.57
7.00	2.00	4.00	-1.75	0.19
1.00	3.00	2.67	1.12	1.00
10.00	18.00	15.33	-2.39	0.00
63.00	85.00	84.67	1.11	0.41
57.00	50.00	62.00	-1.33	0.01
104.00	80.00	93.67	-1.14	0.19
1.00	1.00	0.67	-4.00	0.07
34.00	24.00	36.67	1.27	0.18
340.00	350.00	369.00	-1.10	0.11
55.00	74.00	69.67	1.29	0.04
224.00	256.00	243.33	-1.04	0.50
86.00	90.00	84.33	-1.25	0.03
86.00	73.00	93.33	-1.08	0.50
20.00	25.00	23.33	2.15	0.00
72.00	73.00	78.33	1.21	0.09
62.00	42.00	56.67	1.04	0.78
16.00	17.00	17.33	-1.30	0.21
118.00	106.00	125.00	-1.16	0.09
33.00	25.00	28.33	-1.18	0.33
0.00	3.00	1.67	-1.58	0.59
103.00	140.00	129.33	1.25	0.02
16.00	14.00	16.00	1.21	0.40
4.00	3.00	2.67	-1.86	0.22
17.00	31.00	25.33	-1.25	0.17
362.00	393.00	400.00	-1.17	0.00
12.00	19.00	17.33	-1.79	0.00
108.00	98.00	108.00	-1.16	0.09
104.00	94.00	105.67	1.03	0.78
26.00	33.00	29.00	1.21	0.29
619.00	600.00	585.33	-1.54	0.00
8.00	1.00	6.00	-1.18	0.76
103.00	94.00	109.67	-1.43	0.00
22.00	23.00	21.00	-1.29	0.18
0.00	0.00	0.67	-2.29	0.46
101.00	103.00	102.33	-1.04	0.72
132.00	177.00	160.67	1.22	0.02
271.00	296.00	286.33	1.03	0.63
484.00	581.00	546.00	1.19	0.00
10.00	16.00	16.00	1.00	1.00
23.00	21.00	28.00	-1.05	0.79

1.00	3.00	3.00	-2.53	0.02
4.00	3.00	3.00	1.10	1.00
16.00	25.00	20.33	-1.03	0.86
260.00	289.00	283.67	1.32	0.00
22.00	11.00	22.33	-1.24	0.31
201.00	250.00	240.00	1.10	0.21
257.00	311.00	298.67	-1.07	0.30
315.00	331.00	344.67	1.10	0.14
101.00	114.00	112.33	-1.05	0.64
7.00	13.00	11.00	2.01	0.02
5052.00	5213.00	5239.33	1.00	0.94
1540.00	1562.00	1550.00	-1.08	0.05
540.00	574.00	579.67	1.04	0.43
659.00	649.00	649.00	-1.27	0.00
24623.00	27771.00	27022.67	2.42	0.00
2926.00	2863.00	2904.00	-1.10	0.02
600.00	686.00	667.00	1.10	0.04
11210.00	11607.00	11539.00	-1.23	0.00
347.00	376.00	354.67	-1.53	0.00
174.00	182.00	184.00	-1.33	0.00
1036.00	1052.00	1042.00	-1.15	0.00
136.00	125.00	141.67	-1.08	0.39
196.00	200.00	196.67	1.06	0.48
73.00	73.00	68.00	-1.16	0.20
50.00	44.00	44.67	-1.09	0.55
364.00	351.00	358.67	-1.14	0.03
365.00	355.00	362.33	-1.29	0.00
1599.00	1553.00	1612.67	1.10	0.03
2371.00	2423.00	2415.00	1.17	0.00
1555.00	1589.00	1570.00	1.10	0.02
3435.00	3489.00	3466.33	-1.26	0.00
5.00	12.00	10.00	-1.08	0.90
2644.00	2749.00	2648.33	-1.02	0.69
39.00	52.00	48.67	1.27	0.08
637.00	556.00	606.00	-1.06	0.29
5.00	9.00	5.00	-1.08	1.00
14.00	8.00	11.33	2.98	0.00
1259.00	1238.00	1298.67	-2.17	0.00
1054.00	1069.00	1032.00	-1.09	0.08
719.00	687.00	729.33	1.04	0.42
68.00	97.00	80.33	1.14	0.25
445.00	365.00	390.00	-1.17	0.04
984.00	910.00	983.33	-1.43	0.00
103.00	101.00	94.67	-1.05	0.69
232.00	256.00	253.67	1.01	0.92
2317.00	2192.00	2351.33	1.10	0.02
319.00	320.00	334.33	1.44	0.00
14.00	17.00	15.00	-1.24	0.39

728.00	823.00	810.00	1.12	0.01
626.00	636.00	636.00	-1.27	0.00
524.00	585.00	575.00	-1.23	0.00
257.00	255.00	264.67	1.09	0.19
9.00	14.00	13.33	1.04	0.91
2718.00	2430.00	2486.00	-1.50	0.00
4208.00	4016.00	4163.00	-1.29	0.00
1123.00	1232.00	1176.67	1.20	0.00
1314.00	1403.00	1379.00	-1.05	0.21
0.00	0.00	0.00	-22.49	0.01
14.00	20.00	15.33	-1.23	0.39
19.00	24.00	22.67	1.18	0.44
396.00	395.00	393.67	-1.27	0.00
4.00	7.00	6.33	1.04	1.00
430.00	401.00	399.67	1.08	0.23
129.00	127.00	130.00	-1.15	0.10
1296.00	1405.00	1326.00	-1.08	0.09
2178.00	2284.00	2200.00	-1.02	0.61
6269.00	6239.00	6396.33	-1.06	0.16
4949.00	5293.00	5130.67	1.04	0.31
369.00	416.00	405.00	1.15	0.01
158.00	176.00	165.67	1.04	0.64
1586.00	1563.00	1670.33	-1.13	0.01
189.00	190.00	194.33	1.14	0.07
5.00	11.00	10.33	1.13	0.70
1357.00	1550.00	1464.00	-1.10	0.01
183.00	200.00	203.67	1.31	0.00
1110.00	1116.00	1152.00	1.07	0.10
82.00	88.00	85.67	-1.11	0.30
119.00	98.00	119.67	-1.05	0.60
265.00	308.00	293.33	-1.02	0.70
98.00	120.00	113.67	-1.05	0.59
37.00	48.00	42.67	-1.08	0.60
26.00	33.00	25.33	1.34	0.15
184.00	202.00	196.67	1.11	0.18
1.00	5.00	2.33	-1.70	0.37
110.00	119.00	128.00	-1.05	0.63
26.00	19.00	23.33	-1.04	0.87
89.00	101.00	105.00	1.06	0.52
270.00	254.00	267.67	-1.12	0.06
24.00	22.00	23.67	-1.11	0.58
609.00	644.00	640.00	1.16	0.00
2529.00	2607.00	2620.00	-1.13	0.00
717.00	767.00	757.00	1.12	0.01
451.00	465.00	463.33	1.01	0.93
14.00	36.00	25.67	1.54	0.08
1215.00	1386.00	1328.67	1.07	0.12
24.00	18.00	20.00	-1.03	1.00

190.00	236.00	211.00	1.06	0.44
39.00	46.00	43.67	-1.39	0.01
348.00	380.00	380.00	-1.03	0.64
1696.00	1949.00	1804.33	1.13	0.02
4783.00	5250.00	5147.00	1.08	0.02
48.00	61.00	55.33	-1.24	0.07
9.00	18.00	16.33	-1.49	0.04
14.00	8.00	12.33	1.01	1.00
49.00	74.00	55.67	-1.36	0.02
185.00	203.00	208.67	-1.32	0.00
5.00	2.00	5.67	1.65	0.26
14660.00	15719.00	16475.67	1.03	0.45
70.00	69.00	77.00	1.01	0.94
2646.00	2504.00	2557.33	-1.35	0.00
446.00	428.00	425.33	1.16	0.03
925.00	939.00	920.00	-1.09	0.09
2195.00	2232.00	2255.67	-1.02	0.55
487.00	514.00	526.67	-1.21	0.00
431.00	498.00	474.00	1.41	0.00
1.00	3.00	2.67	1.30	0.79
2947.00	3178.00	3019.67	1.03	0.47
1791.00	2092.00	1987.67	1.38	0.00
6.00	10.00	7.00	1.15	0.76
24.00	13.00	23.33	1.27	0.33
5.00	5.00	7.00	1.47	0.32
1104.00	1070.00	1079.33	-1.25	0.00
98.00	128.00	117.00	1.08	0.44
9.00	6.00	8.33	-1.01	1.00
0.00	2.00	0.67	-2.29	0.49
811.00	851.00	867.67	-1.14	0.00
2.00	1.00	2.00	-2.44	0.08
128.00	153.00	141.33	1.42	0.00
762.00	757.00	781.67	1.08	0.09
0.00	1.00	1.67	-2.90	0.05
2063.00	2215.00	2170.33	-1.06	0.10
530.00	581.00	608.67	1.20	0.00
459.00	470.00	516.33	1.09	0.21
287.00	331.00	303.33	-1.32	0.00
242.00	289.00	276.00	1.23	0.01
30.00	35.00	30.67	1.07	0.78
1535.00	1571.00	1619.67	-1.02	0.57
417.00	470.00	467.00	1.23	0.00
2041.00	2240.00	2270.33	1.13	0.00
1122.00	1387.00	1348.00	1.29	0.00
287.00	295.00	297.00	1.25	0.00
948.00	970.00	1048.67	1.06	0.24
1391.00	1576.00	1507.33	1.06	0.23
99.00	133.00	120.00	-1.24	0.01

20.00	21.00	24.00	1.11	0.68
93.00	115.00	103.00	-1.34	0.00
2115.00	2251.00	2217.33	1.05	0.19
2316.00	2361.00	2459.67	-1.21	0.00
578.00	459.00	492.67	-1.47	0.00
242.00	228.00	226.67	-1.38	0.00
6632.00	6615.00	6667.67	-1.14	0.00
3928.00	3859.00	3934.00	-1.77	0.00
3338.00	3424.00	3481.33	-1.33	0.00
3236.00	3073.00	3196.00	-1.07	0.11
10.00	7.00	8.33	-1.53	0.14
1548.00	1707.00	1593.67	-1.02	0.61
1475.00	1814.00	1705.00	1.11	0.02
845.00	921.00	919.00	1.00	1.00
212.00	224.00	204.67	-1.00	1.00
9239.00	11594.00	10774.00	-1.07	0.11
12.00	15.00	14.67	-2.50	0.00
18.00	20.00	23.67	-1.38	0.06
377.00	446.00	429.33	1.24	0.00
282.00	302.00	312.00	-1.50	0.00
1623.00	1533.00	1515.67	-1.38	0.00
0.00	2.00	1.67	-2.33	0.15
44.00	51.00	49.33	1.25	0.11
10.00	9.00	8.33	1.12	0.78
11.00	14.00	10.67	-1.03	1.00
447.00	537.00	495.33	1.55	0.00
3486.00	3688.00	3735.00	-1.14	0.00
676.00	670.00	734.33	1.13	0.07
716.00	835.00	823.33	1.17	0.00
34.00	38.00	37.67	-1.04	0.81
14.00	15.00	21.67	-1.02	0.94
90.00	131.00	112.67	1.06	0.58
30.00	27.00	31.33	-2.40	0.00
103.00	122.00	110.00	1.57	0.00
1150.00	1426.00	1242.00	1.40	0.00
885.00	888.00	962.33	1.01	0.82
502.00	666.00	600.33	1.03	0.58
166.00	190.00	189.33	-1.96	0.00
471.00	527.00	520.00	1.22	0.00
855.00	955.00	936.00	1.15	0.00
488.00	564.00	566.33	1.07	0.23
217.00	273.00	251.00	1.07	0.34
893.00	1076.00	977.00	1.05	0.35
24.00	30.00	26.67	1.48	0.05
283.00	281.00	292.67	-1.09	0.22
232.00	281.00	266.00	1.19	0.02
593.00	716.00	657.00	-1.11	0.06
229.00	277.00	255.67	-1.12	0.08

462.00	514.00	488.67	-1.39	0.00
6.00	7.00	6.67	1.31	0.51
379.00	388.00	407.33	1.19	0.00
428.00	483.00	485.33	1.06	0.26
2132.00	2104.00	2186.00	-1.12	0.00
1536.00	1585.00	1571.00	-1.24	0.00
262.00	245.00	258.67	-1.04	0.51
4102.00	3935.00	4060.67	-1.08	0.07
3.00	4.00	4.33	1.79	0.27
13.00	10.00	12.33	1.35	0.28
3.00	0.00	2.00	-2.77	0.04
18899.00	19725.00	19239.67	-1.04	0.26
1180.00	1163.00	1239.67	-1.02	0.63
22.00	19.00	19.00	-1.03	1.00
1081.00	1136.00	1168.67	1.10	0.02
704.00	760.00	757.67	1.54	0.00
481.00	527.00	552.00	1.00	0.96
520.00	598.00	589.00	1.08	0.11
1188.00	1138.00	1185.67	-1.33	0.00
733.00	866.00	816.00	1.12	0.02
2758.00	2984.00	2992.67	1.06	0.15
21.00	15.00	18.33	-2.07	0.00
1922.00	1944.00	1979.33	1.22	0.00
3375.00	3407.00	3416.67	-1.13	0.00
119.00	141.00	132.00	1.27	0.01
298.00	342.00	342.00	-1.18	0.02
1125.00	1235.00	1276.00	-1.13	0.01
9.00	9.00	10.33	1.38	0.28
38.00	39.00	42.67	1.03	0.86
52.00	49.00	52.33	1.06	0.68
118.00	121.00	122.33	1.14	0.13
353.00	436.00	408.00	1.06	0.37
4.00	4.00	3.00	-1.88	0.18
112.00	109.00	111.00	1.42	0.00
29.00	26.00	27.00	1.31	0.15
21.00	19.00	20.33	-1.47	0.04
1.00	2.00	2.67	-2.47	0.04
209.00	187.00	190.67	1.12	0.15
13.00	13.00	17.33	-1.06	0.79
9.00	19.00	11.33	-1.07	0.91
2610.00	2430.00	2544.33	-1.00	0.97
3388.00	3446.00	3557.67	-1.12	0.00
52.00	28.00	46.67	1.00	1.00
864.00	996.00	985.00	1.13	0.01
104.00	77.00	88.00	1.21	0.15
1616.00	1709.00	1658.67	-1.11	0.01
156.00	155.00	164.33	-1.10	0.23
1102.00	1444.00	1279.33	-1.22	0.00

391.00	440.00	418.33	1.09	0.11
5.00	2.00	3.67	-3.33	0.00
781.00	921.00	947.67	1.21	0.00
606.00	839.00	788.33	1.46	0.00
2495.00	2791.00	2586.67	-1.09	0.07
2.00	7.00	6.33	4.36	0.00
805.00	910.00	866.67	1.16	0.00
3852.00	4679.00	4294.00	-1.45	0.00
5.00	5.00	4.33	-1.32	0.59
374.00	436.00	437.00	-1.06	0.33
592.00	623.00	593.00	-1.26	0.00
435.00	421.00	459.67	-1.02	0.78
1179.00	1072.00	1143.33	-1.10	0.05
1256.00	1380.00	1308.33	-1.14	0.00
402.00	403.00	381.33	-1.23	0.01
127.00	91.00	122.00	-1.71	0.00
12.00	12.00	17.00	-1.35	0.17
746.00	812.00	759.00	-1.01	0.79
871.00	890.00	826.00	-1.12	0.10
3376.00	3340.00	3446.33	-1.16	0.00
31.00	52.00	42.67	1.25	0.13
0.00	1.00	0.33	-3.95	0.23
3831.00	4332.00	3971.33	-1.24	0.00
1139.00	1114.00	1145.00	-1.32	0.00
1728.00	1756.00	1729.67	-1.09	0.05
627.00	556.00	595.33	-1.16	0.01
2589.00	2376.00	2575.33	-1.25	0.00
587.00	608.00	626.33	-1.12	0.03
1031.00	1075.00	1131.00	-1.01	0.87
876.00	1039.00	946.33	1.11	0.04
41.00	67.00	51.00	1.06	0.71
86.00	86.00	81.33	1.05	0.66
1588.00	1668.00	1701.33	1.01	0.83
43.00	40.00	42.67	-1.08	0.60
1.00	0.00	3.33	1.21	0.85
86.00	70.00	74.00	1.25	0.06
2357.00	2327.00	2286.67	-1.45	0.00
34.00	32.00	40.33	1.49	0.02
2109.00	2369.00	2404.33	1.14	0.00
73.00	60.00	74.67	1.24	0.08
874.00	895.00	884.00	-1.10	0.05
135.00	134.00	133.33	-1.35	0.00
1525.00	1516.00	1581.67	1.02	0.58
2601.00	2723.00	2757.67	1.08	0.03
1188.00	1164.00	1259.67	-1.04	0.43
704.00	701.00	713.00	-1.15	0.00
486.00	517.00	497.67	1.03	0.63
1162.00	1182.00	1218.67	1.00	1.00

186.00	186.00	177.00	-1.52	0.00
481.00	514.00	530.00	-1.08	0.12
1718.00	1732.00	1728.67	-1.07	0.07
842.00	898.00	897.00	-1.11	0.01
443.00	466.00	490.67	1.04	0.50
1541.00	1615.00	1583.33	1.05	0.23
922.00	1091.00	981.33	-1.22	0.00
961.00	1085.00	1066.67	-1.21	0.00
696.00	770.00	741.67	-1.20	0.00
233.00	219.00	223.00	1.31	0.00
445.00	480.00	505.00	1.16	0.02
1589.00	1836.00	1733.00	1.01	0.76
416.00	487.00	475.67	1.62	0.00
26.00	23.00	23.33	1.06	0.75
127.00	117.00	119.33	-1.42	0.00
6995.00	6804.00	6906.00	-1.28	0.00
8.00	14.00	14.67	-1.01	1.00
1137.00	1087.00	1145.00	1.06	0.17
1261.00	1526.00	1365.33	-1.02	0.70
9559.00	9572.00	9630.33	1.05	0.19
1828.00	2060.00	1959.67	1.03	0.48
2.00	2.00	1.33	-2.63	0.12
107.00	90.00	101.00	1.19	0.08
15.00	11.00	12.67	1.49	0.14
1704.00	1746.00	1753.00	-1.16	0.00
5.00	5.00	6.33	1.68	0.21
1546.00	1757.00	1677.67	-1.25	0.00
542.00	601.00	561.33	1.03	0.62
2398.00	2602.00	2532.33	1.06	0.12
1.00	3.00	2.67	-2.71	0.02
291.00	306.00	319.67	-1.06	0.33
861.00	958.00	935.33	-1.14	0.00
1048.00	1038.00	1117.67	-1.20	0.00
1533.00	1758.00	1661.00	1.04	0.39
1889.00	2049.00	2074.67	1.05	0.24
736.00	681.00	743.00	1.05	0.33
1421.00	1444.00	1444.00	1.13	0.01
304.00	314.00	316.00	-1.02	0.78
1019.00	1175.00	1153.33	-1.02	0.64
209.00	222.00	218.33	-1.00	0.98
11.00	5.00	12.00	-1.21	0.53
2902.00	3189.00	3247.33	-1.29	0.00
884.00	890.00	898.67	1.11	0.01
3063.00	3145.00	3168.67	1.28	0.00
2120.00	2079.00	2191.67	-1.00	0.96
932.00	903.00	913.67	-1.38	0.00
759.00	733.00	765.00	1.07	0.11
361.00	432.00	431.33	-1.10	0.13

1274.00	1269.00	1285.00	-1.13	0.01
2876.00	3139.00	3138.67	1.22	0.00
411.00	379.00	401.00	-1.04	0.55
153.00	176.00	173.67	1.03	0.71
25.00	19.00	19.33	1.10	0.65
10.00	8.00	9.67	1.67	0.12
93.00	72.00	79.67	1.64	0.00
4065.00	4167.00	4171.67	1.47	0.00
954.00	1030.00	1037.67	1.04	0.40
4878.00	5445.00	5264.67	-1.08	0.02
2025.00	2540.00	2248.00	-1.00	0.96
66.00	46.00	52.33	-1.24	0.11
529.00	499.00	541.00	-1.12	0.03
2409.00	2635.00	2554.67	1.00	0.97
1570.00	1524.00	1663.67	1.38	0.00
291.00	415.00	356.00	1.11	0.19
1433.00	1469.00	1475.67	1.03	0.40
1656.00	1837.00	1815.67	-1.13	0.00
530.00	535.00	537.67	-1.16	0.00
11613.00	12331.00	12281.67	1.04	0.24
769.00	823.00	817.33	-1.06	0.14
2189.00	2194.00	2179.00	-1.11	0.01
15.00	11.00	11.67	-2.19	0.00
5.00	3.00	6.00	1.36	0.48
2815.00	2972.00	2953.00	-1.32	0.00
219.00	247.00	228.33	-1.13	0.06
658.00	676.00	654.00	1.13	0.03
492.00	644.00	594.67	1.09	0.13
12570.00	13712.00	13017.67	-1.07	0.07
1337.00	1524.00	1468.67	1.16	0.00
12.00	3.00	6.33	1.17	0.75
16.00	8.00	11.67	-1.18	0.58
101.00	114.00	98.33	-1.78	0.00
91.00	60.00	74.00	1.42	0.01
4.00	3.00	3.33	-1.50	0.43
7685.00	8896.00	8229.00	1.16	0.00
1254.00	1240.00	1317.33	-1.16	0.00
2994.00	2931.00	2933.00	-1.38	0.00
1061.00	1119.00	1161.33	-1.08	0.08
2221.00	2387.00	2424.00	1.12	0.01
256.00	305.00	291.67	-1.19	0.02
2002.00	2042.00	2058.33	-1.12	0.00
710.00	805.00	800.00	1.05	0.38
2805.00	3021.00	3095.00	1.02	0.71
723.00	781.00	785.33	-1.04	0.42
235.00	186.00	220.67	-1.16	0.08
1131.00	1083.00	1151.67	-1.12	0.01
301.00	343.00	317.67	1.23	0.00

157.00	164.00	165.00	-1.12	0.12
29.00	29.00	28.67	1.39	0.06
432.00	498.00	467.33	1.06	0.29
3631.00	4015.00	3784.33	-1.04	0.44
43.00	33.00	41.33	-1.07	0.64
329.00	329.00	340.67	-1.38	0.00
256.00	284.00	264.67	1.30	0.00
226.00	222.00	234.67	-1.04	0.60
427.00	402.00	413.67	1.03	0.62
21.00	16.00	20.33	-1.91	0.00
608.00	625.00	627.67	-1.44	0.00
976.00	1075.00	1039.33	-1.02	0.60
6054.00	5840.00	6100.67	-1.16	0.00
1008.00	952.00	959.00	1.12	0.04
1624.00	1666.00	1697.33	1.02	0.55
0.00	6.00	3.33	3.03	0.11
49.00	32.00	38.00	-2.67	0.00
261.00	275.00	277.33	1.23	0.00
650.00	601.00	649.67	1.03	0.61
768.00	820.00	795.00	1.01	0.79
1363.00	1444.00	1424.67	1.08	0.05
94.00	102.00	107.00	-1.11	0.23
6.00	6.00	5.33	-2.13	0.02
25.00	24.00	31.33	-1.12	0.49
16618.00	15236.00	15718.67	1.01	0.76
180.00	195.00	198.67	1.04	0.62
15684.00	17220.00	16937.67	1.14	0.00
422.00	522.00	459.00	1.47	0.00
1988.00	2093.00	2046.67	1.14	0.00
3049.00	3259.00	3221.00	1.17	0.00
3272.00	3281.00	3294.67	-1.10	0.01
178.00	179.00	176.67	-1.17	0.03
2360.00	2550.00	2379.00	1.03	0.51
1132.00	1166.00	1163.33	-1.09	0.06
1732.00	1842.00	1748.33	1.26	0.00
5310.00	5853.00	5727.67	1.36	0.00
1522.00	1908.00	1737.67	1.16	0.00
318.00	351.00	340.00	1.08	0.24
1608.00	1782.00	1770.67	1.14	0.00
19.00	26.00	23.33	-1.07	0.69
598.00	611.00	619.33	-1.17	0.00
855.00	886.00	889.00	1.11	0.01
214.00	236.00	221.33	-1.16	0.02
336.00	377.00	356.67	1.02	0.78
23.00	18.00	24.33	1.16	0.48
1330.00	1384.00	1428.33	1.09	0.03
867.00	837.00	844.00	1.23	0.00
1085.00	1239.00	1201.67	1.04	0.31

180.00	162.00	177.00	-1.30	0.00
637.00	657.00	665.33	-1.03	0.53
767.00	871.00	828.33	1.10	0.03
2.00	0.00	1.00	-2.51	0.23
793.00	941.00	847.67	1.17	0.01
35.00	25.00	30.00	-2.41	0.00
80.00	106.00	96.00	-1.22	0.03
2884.00	3179.00	3209.33	1.23	0.00
36.00	28.00	34.33	1.39	0.04
4194.00	4283.00	4375.67	1.26	0.00
1790.00	1714.00	1803.00	1.13	0.02
325.00	404.00	373.00	1.09	0.15
1067.00	1208.00	1171.33	1.07	0.14
1935.00	2153.00	2135.67	1.00	0.91
3555.00	3662.00	3573.33	-1.14	0.00
37.00	38.00	38.00	-1.21	0.17
805.00	850.00	863.33	1.18	0.00
1301.00	1537.00	1440.00	1.20	0.00
29.00	36.00	37.33	-1.99	0.00
1020.00	1192.00	1175.33	1.18	0.00
1318.00	1557.00	1527.00	1.22	0.00
1062.00	1264.00	1115.33	1.10	0.16
1163.00	1114.00	1143.33	-1.26	0.00
468.00	458.00	474.67	1.02	0.76
2434.00	2276.00	2298.67	-1.14	0.01
663.00	766.00	732.67	1.24	0.00
233.00	223.00	246.00	1.13	0.09
1660.00	1523.00	1594.00	-1.25	0.00
98.00	100.00	90.67	-1.14	0.22
2051.00	2228.00	2077.33	-1.08	0.17
2778.00	2854.00	2728.33	-1.10	0.06
1433.00	1484.00	1474.00	-1.03	0.53
1.00	2.00	1.00	-2.51	0.25
1833.00	1941.00	1929.67	-1.02	0.67
661.00	647.00	646.33	-1.02	0.66
868.00	954.00	930.00	1.04	0.35
296.00	284.00	320.33	1.04	0.57
257.00	361.00	342.33	-1.01	0.93
677.00	704.00	702.00	1.11	0.02
493.00	616.00	584.67	1.01	0.83
1585.00	1531.00	1569.33	1.10	0.03
1259.00	1405.00	1339.00	-1.11	0.01
2114.00	2027.00	2128.67	-1.09	0.04
532.00	545.00	555.00	-1.08	0.10
203.00	232.00	224.67	-1.05	0.51
502.00	549.00	552.67	1.14	0.01
441.00	493.00	491.33	-1.01	0.86
584.00	548.00	576.00	-1.01	0.93

318.00	324.00	347.33	1.29	0.00
141.00	170.00	164.00	1.06	0.46
474.00	469.00	494.67	1.18	0.00
489.00	526.00	514.33	1.15	0.01
248.00	266.00	266.67	-1.03	0.73
1518.00	1700.00	1712.33	1.17	0.00
194.00	209.00	208.67	-1.11	0.17
1147.00	1257.00	1203.67	-1.00	0.99
0.00	3.00	1.33	-3.33	0.03
103.00	96.00	104.00	1.28	0.02
4.00	8.00	7.00	-1.06	1.00
198.00	220.00	221.33	-1.10	0.18
9.00	12.00	11.33	1.20	0.54
1377.00	1379.00	1399.33	-1.21	0.00
6.00	3.00	3.67	-2.71	0.01
1519.00	1550.00	1487.00	-1.44	0.00
202.00	189.00	200.33	-1.47	0.00
8.00	18.00	13.67	1.06	0.83
13.00	12.00	10.33	1.45	0.23
131.00	166.00	152.00	1.12	0.15
21.00	26.00	27.33	-1.40	0.03
392.00	432.00	423.00	-1.00	0.98
1.00	0.00	2.33	-2.39	0.07
121.00	144.00	138.67	-1.07	0.36
1277.00	1432.00	1426.67	1.34	0.00
549.00	572.00	591.00	1.32	0.00
3210.00	3228.00	3355.00	-1.12	0.00
1813.00	1811.00	1859.67	-1.08	0.05
313.00	357.00	338.67	-1.30	0.00
945.00	1022.00	1008.00	1.30	0.00
1948.00	1883.00	1951.67	1.00	0.99
26.00	34.00	30.67	2.01	0.00
20.00	17.00	16.00	1.96	0.01
17.00	19.00	18.00	-1.11	0.72
5109.00	5322.00	5236.00	-1.41	0.00
1313.00	1517.00	1458.67	1.19	0.00
473.00	559.00	525.00	-1.20	0.00
2451.00	2752.00	2694.67	1.20	0.00
618.00	589.00	635.67	-1.03	0.63
159.00	161.00	167.67	-1.06	0.46
382.00	399.00	428.67	1.04	0.52
524.00	624.00	596.67	1.22	0.00
24.00	25.00	32.67	-1.28	0.15
1431.00	1462.00	1527.00	-1.06	0.19
396.00	457.00	435.33	1.07	0.22
472.00	548.00	525.33	1.08	0.12
1140.00	1261.00	1190.00	1.06	0.21
1362.00	1439.00	1455.33	1.01	0.90

202.00	248.00	244.00	1.20	0.02
47.00	43.00	44.00	-1.01	1.00
259.00	299.00	290.00	1.44	0.00
260.00	299.00	293.00	-1.07	0.27
2.00	2.00	2.67	-1.62	0.39
355.00	358.00	376.33	-1.16	0.01
596.00	683.00	670.00	1.07	0.15
48.00	53.00	56.00	-1.13	0.28
603.00	606.00	601.33	1.23	0.00
733.00	779.00	742.00	1.13	0.02
2635.00	2864.00	2846.67	1.00	0.96
1022.00	1145.00	1092.67	1.05	0.27
809.00	981.00	962.67	1.17	0.00
452.00	481.00	451.33	1.14	0.03
0.00	1.00	0.33	-5.43	0.07
6.00	6.00	7.00	-1.87	0.03
703.00	802.00	771.00	1.12	0.01
4874.00	4930.00	5093.33	1.36	0.00
859.00	861.00	869.67	1.05	0.38
117.00	154.00	135.67	-1.08	0.39
1094.00	1149.00	1167.33	1.17	0.00
324.00	336.00	327.33	1.16	0.02
699.00	820.00	778.67	1.12	0.02
71.00	87.00	82.67	-1.14	0.23
893.00	866.00	894.33	-1.25	0.00
219.00	217.00	210.33	1.07	0.35
1560.00	1753.00	1740.67	1.17	0.00
1376.00	1364.00	1404.00	-1.48	0.00
569.00	490.00	509.00	-1.19	0.01
1984.00	1907.00	1976.67	-1.26	0.00
119.00	110.00	122.33	1.35	0.00
1002.00	1148.00	1068.33	1.06	0.22
2623.00	2763.00	2804.00	-1.01	0.89
35.00	50.00	46.33	-1.27	0.06
2489.00	2519.00	2527.33	-1.00	0.92
6113.00	6104.00	6530.33	-1.05	0.20
512.00	636.00	602.33	1.13	0.03
1472.00	1474.00	1466.33	-1.33	0.00
258.00	276.00	285.67	-1.20	0.00
870.00	838.00	874.33	-1.17	0.00
656.00	622.00	675.00	-1.15	0.00
90.00	82.00	85.00	-1.55	0.00
465.00	519.00	524.33	-1.10	0.08
163.00	153.00	155.67	-1.20	0.02
1166.00	1252.00	1225.67	-1.09	0.05
2287.00	2367.00	2371.33	-1.14	0.00
1280.00	1373.00	1362.33	1.03	0.50
7.00	13.00	11.33	1.51	0.16

3853.00	3751.00	3972.67	-1.03	0.50
1975.00	1948.00	1949.00	-1.07	0.14
432.00	443.00	440.00	-1.83	0.00
15.00	16.00	15.33	-1.36	0.17
0.00	0.00	2.33	-1.17	1.00
622.00	665.00	685.33	-1.15	0.00
13.00	21.00	17.00	-1.21	0.41
114.00	89.00	103.00	-1.07	0.53
135.00	176.00	166.00	1.22	0.02
7041.00	7693.00	7434.33	-1.05	0.15
5799.00	5712.00	5980.67	1.05	0.19
719.00	900.00	880.33	1.08	0.22
926.00	862.00	897.33	-1.16	0.00
1615.00	1878.00	1837.67	1.05	0.32
180.00	202.00	185.33	1.26	0.01
950.00	994.00	1032.67	-1.10	0.04
2681.00	2900.00	2842.33	-1.23	0.00
5.00	15.00	12.00	-1.02	1.00
43.00	33.00	40.00	1.01	1.00
1475.00	1584.00	1453.00	1.01	0.93
955.00	898.00	954.33	-1.29	0.00
214.00	245.00	250.00	1.02	0.78
1557.00	1633.00	1652.33	-1.18	0.00
8457.00	8089.00	8282.00	-1.23	0.00
2940.00	2761.00	2868.33	-1.33	0.00
116.00	97.00	108.00	1.25	0.02
1044.00	1088.00	1050.33	-1.25	0.00
381.00	339.00	356.33	-1.33	0.00
883.00	899.00	937.67	1.04	0.48
699.00	769.00	754.67	1.05	0.31
123.00	204.00	160.33	-1.10	0.40
422.00	480.00	472.33	-1.34	0.00
529.00	586.00	599.67	-1.00	0.94
11.00	14.00	13.00	-1.76	0.01
800.00	801.00	839.00	-1.74	0.00
1403.00	1363.00	1399.33	-1.05	0.32
127.00	148.00	144.33	-1.50	0.00
386.00	457.00	460.00	1.07	0.31
643.00	720.00	687.33	1.12	0.02
727.00	751.00	773.33	-1.02	0.64
516.00	642.00	585.00	1.00	0.95
5.00	13.00	8.67	1.81	0.09
358.00	366.00	382.00	1.06	0.31
767.00	738.00	827.67	1.03	0.65
345.00	364.00	337.67	-1.03	0.68
1903.00	2134.00	2101.00	1.05	0.24
3922.00	3635.00	3873.67	-1.09	0.04
703.00	724.00	788.67	1.10	0.11

1258.00	1239.00	1256.33	1.02	0.70
1172.00	1357.00	1303.33	1.12	0.01
11.00	8.00	7.00	-2.03	0.03
434.00	426.00	440.33	1.21	0.00
48.00	49.00	52.33	-1.14	0.32
429.00	544.00	502.33	1.12	0.07
3462.00	4337.00	4045.67	-1.36	0.00
1.00	11.00	6.67	1.40	0.41
13.00	8.00	8.33	-1.09	0.89
3.00	1.00	3.00	-1.77	0.24
1.00	5.00	3.67	-1.01	1.00
14.00	16.00	11.67	1.19	0.61
45.00	72.00	56.67	-1.25	0.08
0.00	5.00	1.67	-7.42	0.00
7.00	5.00	6.67	-1.07	1.00
12.00	7.00	8.00	1.18	0.66
0.00	0.00	0.00	-9.06	0.27
86.00	111.00	102.33	-1.09	0.34
14.00	24.00	15.33	1.51	0.12
36.00	34.00	29.67	-1.78	0.00
54.00	68.00	72.67	-1.02	0.88
9.00	4.00	6.67	-1.01	1.00
172.00	262.00	217.33	-1.05	0.57
572.00	701.00	613.00	-1.06	0.43
37.00	56.00	37.33	-1.32	0.18
7.00	1.00	3.67	-1.01	1.00
0.00	1.00	0.67	-3.57	0.11
53.00	61.00	67.00	-1.05	0.70
24.00	17.00	19.00	1.20	0.40
13.00	15.00	17.67	-1.92	0.00
606.00	667.00	670.67	-1.27	0.00
41.00	55.00	51.33	-1.49	0.00
4.00	3.00	5.33	-4.37	0.00
0.00	1.00	1.00	-4.31	0.01
2.00	1.00	1.67	-1.39	0.78
41.00	55.00	55.33	1.26	0.10
0.00	0.00	0.00	-9.06	0.27
0.00	0.00	1.00	-1.92	0.52
1.00	0.00	1.00	-3.11	0.10
2.00	2.00	2.00	-1.97	0.24
3.00	4.00	3.67	-1.90	0.13
35.00	35.00	38.33	-1.28	0.08
4.00	2.00	4.00	-1.58	0.32
5.00	4.00	7.00	-1.96	0.05
19.00	19.00	22.33	-1.27	0.19
228.00	267.00	266.00	1.05	0.45
42.00	34.00	34.00	1.40	0.05
3.00	4.00	2.67	-1.86	0.22

1.00	2.00	1.33	-2.17	0.27
3.00	4.00	2.67	-1.98	0.16
6.00	3.00	4.00	-2.16	0.04
25.00	27.00	29.33	-1.29	0.12
0.00	3.00	1.00	-1.61	0.73
12.00	35.00	23.00	-1.92	0.00
3.00	4.00	3.00	-2.85	0.01
5.00	5.00	5.00	1.06	1.00
287.00	361.00	347.67	-1.39	0.00
0.00	0.00	0.33	-4.69	0.13
4.00	3.00	2.67	-1.74	0.29
12.00	11.00	11.33	1.34	0.31
4.00	5.00	4.33	-1.47	0.38
0.00	0.00	0.00	-9.06	0.27
1888.00	1809.00	1969.00	1.10	0.03
2.00	0.00	1.67	-1.77	0.43
4.00	1.00	1.67	-2.52	0.10
4.00	3.00	3.00	-2.42	0.03
0.00	6.00	4.33	-1.01	1.00
30.00	42.00	41.00	1.17	0.32
6.00	3.00	6.67	1.04	1.00
5.00	0.00	1.67	-2.52	0.11
1.00	1.00	0.67	-6.98	0.00
1.00	0.00	0.67	-4.85	0.02
3.00	6.00	4.00	-1.42	0.47
4.00	1.00	3.67	1.08	1.00
7.00	10.00	8.67	1.59	0.17
121.00	121.00	119.00	-1.14	0.15
4.00	1.00	2.67	-1.62	0.39
4.00	7.00	4.33	-2.08	0.05
5.00	8.00	5.67	2.05	0.11
23.00	25.00	22.67	-1.07	0.75
5.00	12.00	9.67	2.16	0.02
10.00	7.00	9.00	-1.75	0.03
26.00	19.00	21.00	-1.24	0.30
3.00	3.00	3.33	-1.50	0.43
3005.00	3055.00	3202.33	1.13	0.00
147.00	139.00	145.67	-1.21	0.01
27.00	32.00	29.33	-1.06	0.79
227.00	243.00	259.33	-1.06	0.40
19.00	13.00	16.00	1.47	0.11
1.00	5.00	2.67	1.30	0.79
2.00	0.00	1.00	-1.91	0.52
3.00	1.00	2.00	1.17	1.00
11.00	14.00	15.67	1.92	0.01
2.00	5.00	2.67	-1.13	1.00
25.00	20.00	23.67	-1.51	0.03
8.00	9.00	8.00	-1.30	0.43

3.00	2.00	2.00	-1.33	0.79
25.00	17.00	19.33	-1.34	0.13
15.00	8.00	9.33	1.53	0.20
4.00	1.00	2.00	-3.08	0.02
136.00	158.00	131.00	-1.53	0.00
10.00	7.00	9.33	1.10	0.79
8.00	1.00	4.00	-1.59	0.30
9.00	1.00	4.33	-1.62	0.25
1.00	0.00	0.67	-2.72	0.29
0.00	0.00	0.67	-3.58	0.12
8.00	9.00	6.33	-1.43	0.32
13.00	6.00	9.67	1.30	0.42
1.00	1.00	1.33	-1.01	1.00
3.00	4.00	3.67	-1.90	0.12
1.00	7.00	4.00	-1.18	0.85
1.00	1.00	2.00	-1.49	0.61
9.00	21.00	13.00	-1.17	0.67
55.00	60.00	62.67	-1.76	0.00
829.00	986.00	965.67	-1.17	0.00
1808.00	2174.00	2077.67	1.09	0.05
1.00	0.00	0.67	-2.29	0.46
0.00	0.00	0.00	-25.19	0.00
0.00	1.00	0.33	-6.16	0.04
5.00	0.00	2.00	-1.49	0.62
5.00	4.00	4.67	-1.72	0.20
3.00	6.00	4.67	1.51	0.41
4.00	2.00	3.00	-1.23	0.83
19.00	15.00	15.67	1.54	0.08
17.00	10.00	10.33	-1.43	0.19
6.00	6.00	5.33	1.93	0.16
10.00	5.00	8.00	-1.68	0.07
3.00	2.00	1.67	-1.20	1.00
2.00	0.00	1.67	-3.10	0.03
27.00	30.00	29.00	-1.49	0.01
188.00	185.00	188.00	-1.16	0.04
38.00	28.00	34.33	-1.37	0.03
5.00	18.00	11.67	-1.59	0.04
25.00	13.00	15.67	-1.57	0.04
19.00	15.00	18.67	1.02	0.93
10.00	5.00	6.33	-2.32	0.00
19.00	24.00	22.33	-1.04	0.87
10.00	9.00	8.67	-2.97	0.00
9.00	10.00	9.67	-2.19	0.00
56.00	46.00	57.00	-1.16	0.22
2.00	3.00	3.00	-1.12	1.00
0.00	0.00	0.00	-11.75	0.13
1.00	1.00	1.67	-1.96	0.31
2.00	0.00	1.00	-5.82	0.00

10.00	3.00	5.00	1.83	0.25
2.00	1.00	1.33	-1.24	1.00
3.00	3.00	3.00	-1.55	0.41
7.00	4.00	5.00	-1.34	0.51
2.00	0.00	0.67	-2.71	0.30
20.00	20.00	19.67	-1.30	0.18
2.00	1.00	1.00	-3.71	0.04
6.00	2.00	4.00	-1.34	0.58
4.00	2.00	4.00	-1.42	0.47
3.00	7.00	7.00	1.70	0.17
57.00	54.00	54.33	-2.02	0.00
7.00	14.00	9.33	-1.26	0.47
6.00	4.00	4.00	-2.16	0.04
2.00	5.00	2.67	-2.58	0.03
0.00	0.00	0.00	-11.76	0.14
5.00	5.00	4.33	-1.32	0.59
3.00	0.00	2.33	-1.56	0.49
25.00	45.00	42.67	-1.03	0.84
2.00	4.00	3.67	1.52	0.49
10.00	10.00	12.67	1.17	0.57
5.00	5.00	5.67	-1.25	0.63
56.00	50.00	48.33	1.06	0.78
6.00	3.00	3.67	-1.55	0.36
1.00	0.00	0.33	-3.95	0.23
1.00	0.00	1.00	-2.51	0.24
1.00	2.00	3.33	-1.60	0.34
1.00	2.00	2.33	-2.53	0.05
8.00	11.00	10.00	-1.18	0.63
9.00	4.00	9.00	-1.12	0.81
116.00	127.00	115.33	-1.22	0.03
210.00	233.00	238.33	-1.61	0.00
37.00	55.00	49.33	-1.28	0.06
3.00	6.00	4.00	-3.64	0.00
1.00	1.00	1.00	-3.11	0.10
18.00	2.00	10.33	-1.20	0.66
1315.00	1550.00	1491.00	-1.04	0.40
371.00	454.00	443.33	1.19	0.00
896.00	1101.00	983.67	1.02	0.75
535.00	639.00	620.67	1.10	0.08
140.00	150.00	147.67	-1.32	0.00
2.00	2.00	2.33	1.35	0.78
5094.00	5082.00	5059.00	-1.00	1.00
1147.00	1332.00	1303.00	1.11	0.02
502.00	501.00	501.33	-1.07	0.18
594.00	539.00	560.33	-1.11	0.08
2090.00	2282.00	2199.67	-1.00	0.92
102.00	88.00	92.00	-1.00	1.00
532.00	587.00	593.33	-1.13	0.01

1349.00	1313.00	1383.00	-1.39	0.00
1776.00	1899.00	1920.67	-1.03	0.48
941.00	967.00	1003.00	-1.03	0.52
794.00	809.00	826.00	-1.08	0.08
141.00	144.00	137.00	1.15	0.11
4973.00	4631.00	4722.00	-1.24	0.00
411.00	434.00	436.00	1.22	0.00
2792.00	2942.00	3013.33	1.09	0.02
877.00	962.00	946.33	1.12	0.01
654.00	759.00	735.00	-1.07	0.17
512.00	521.00	535.00	-1.14	0.01
2485.00	2544.00	2544.33	1.05	0.17
1839.00	2056.00	1997.33	1.03	0.39
132.00	139.00	148.33	1.07	0.41
148.00	181.00	159.33	1.17	0.05
3235.00	3627.00	3407.67	-1.09	0.03
26.00	21.00	26.00	1.16	0.42
4.00	7.00	4.33	-1.47	0.39
8719.00	8893.00	9139.67	-1.44	0.00
987.00	986.00	1054.67	1.23	0.00
1212.00	1246.00	1306.00	1.17	0.00
1895.00	1868.00	1937.33	-1.31	0.00
235.00	212.00	230.33	-1.02	0.75
67.00	68.00	68.33	1.48	0.00
265.00	280.00	287.33	1.10	0.12
262.00	281.00	265.67	1.02	0.74
2141.00	2405.00	2397.33	1.19	0.00
275.00	304.00	289.67	-1.01	0.85
10640.00	10682.00	10668.33	1.42	0.00
993.00	965.00	965.67	1.24	0.00
842639.00	936150.00	906546.67	-1.04	0.23
15.00	11.00	13.67	1.19	0.59
5.00	10.00	6.67	1.16	0.75
3.00	1.00	3.00	-1.55	0.41
14950.00	15961.00	15567.67	-1.18	0.00
10.00	8.00	10.33	-1.14	0.72
395.00	411.00	378.33	-1.13	0.10
276.00	309.00	289.67	-1.23	0.00
178.00	187.00	179.00	-1.17	0.04
1998.00	1897.00	2001.00	-1.94	0.00
653.00	723.00	728.67	-1.15	0.00
17.00	18.00	19.00	-1.72	0.00
82.00	101.00	84.33	-1.29	0.01
2406.00	2686.00	2556.67	-1.80	0.00
2997.00	3256.00	3231.67	-1.02	0.50
650.00	770.00	715.00	1.22	0.00
2.00	1.00	1.33	-1.48	0.76
791.00	928.00	901.33	1.27	0.00

138.00	215.00	177.67	1.51	0.00
132.00	124.00	126.00	1.44	0.00
1072.00	1119.00	1102.67	-1.00	0.98
760.00	747.00	751.33	1.09	0.09
691.00	784.00	720.33	1.23	0.00
331.00	358.00	376.67	-1.01	0.93
810.00	813.00	827.00	-1.26	0.00
16.00	6.00	13.00	1.32	0.30
233.00	217.00	233.33	-1.11	0.19
30.00	42.00	39.00	1.07	0.67
1205.00	1217.00	1200.00	-1.42	0.00
492.00	504.00	524.33	-1.09	0.10
1034.00	1175.00	1173.33	1.18	0.00
385.00	392.00	414.00	1.01	0.87
0.00	0.00	0.00	-11.74	0.13
3570.00	3569.00	3582.00	-1.19	0.00
23872.00	26037.00	26647.67	1.24	0.00
418.00	411.00	431.00	-1.10	0.08
877.00	880.00	887.33	-1.20	0.00
1028.00	1135.00	1082.00	-1.03	0.53
1987.00	2041.00	2070.33	-1.08	0.02
641.00	706.00	658.00	1.07	0.19
567.00	683.00	658.33	1.17	0.00
2300.00	2515.00	2507.00	1.12	0.00
7589.00	8902.00	8664.00	-1.01	0.84
117.00	137.00	136.67	-1.07	0.38
125.00	133.00	132.67	-1.19	0.04
189.00	216.00	209.00	1.28	0.00
1.00	2.00	2.00	-2.13	0.17
469.00	436.00	458.00	-1.16	0.00
291.00	330.00	334.00	1.01	0.86
338.00	344.00	339.67	-1.12	0.04
281.00	275.00	294.33	1.02	0.76
1219.00	1231.00	1250.00	-1.17	0.00
1641.00	1789.00	1716.67	1.33	0.00
5.00	5.00	3.33	-2.46	0.03
471.00	575.00	558.33	-1.02	0.69
371.00	430.00	412.00	1.00	0.98
1296.00	1301.00	1398.67	1.03	0.65
3379.00	3530.00	3417.33	-1.22	0.00
1084.00	1070.00	1091.33	-1.14	0.00
859.00	1098.00	1003.33	1.03	0.53
861.00	1100.00	1026.33	1.11	0.06
933.00	1065.00	1000.33	-1.13	0.01
583.00	593.00	558.33	1.01	0.90
516.00	552.00	557.67	-1.03	0.54
5.00	5.00	6.33	1.84	0.14
913.00	949.00	964.33	1.18	0.00

121.00	131.00	127.00	1.10	0.26
167.00	122.00	157.00	1.56	0.00
60.00	39.00	48.33	1.17	0.32
855.00	847.00	857.67	-1.19	0.00
1252.00	1448.00	1342.33	1.02	0.65
1028.00	1122.00	1042.67	1.22	0.00
1167.00	1168.00	1181.67	1.19	0.00
180.00	214.00	183.33	-1.06	0.55
430.00	469.00	466.00	1.05	0.31
6859.00	6784.00	6762.67	-1.27	0.00
593.00	593.00	610.33	1.01	0.80
1079.00	1107.00	1164.67	-1.30	0.00
351.00	349.00	365.00	1.04	0.46
20.00	13.00	15.67	-2.53	0.00
2739.00	2535.00	2657.67	-1.09	0.05
235.00	237.00	252.00	-1.33	0.00
6.00	15.00	11.33	1.95	0.03
84.00	63.00	75.33	-1.01	0.97
8.00	6.00	6.00	1.36	0.48
465.00	450.00	453.00	-1.46	0.00
1189.00	1500.00	1353.67	1.09	0.09
89.00	129.00	115.00	-1.09	0.35
2752.00	2898.00	2987.33	1.19	0.00
718.00	778.00	798.67	1.07	0.18
1123.00	1173.00	1188.00	1.17	0.00
1218.00	1437.00	1349.00	-1.05	0.29
18.00	7.00	12.67	-1.30	0.31
1310.00	1009.00	1191.33	-1.56	0.00
5.00	7.00	7.67	1.19	0.65
406.00	504.00	475.33	1.16	0.01
1016.00	1076.00	1118.67	1.07	0.17
1747.00	1899.00	1876.33	1.21	0.00
1847.00	2061.00	2077.33	1.28	0.00
972.00	1189.00	1153.33	1.18	0.00
602.00	676.00	679.33	1.08	0.17
1435.00	1572.00	1600.00	1.15	0.00
691.00	775.00	782.67	1.15	0.01
1254.00	1372.00	1362.33	1.28	0.00
917.00	920.00	953.67	1.18	0.00
1171.00	1282.00	1270.33	1.19	0.00
801.00	840.00	865.33	1.18	0.00
813.00	860.00	915.00	1.16	0.01
812.00	888.00	893.00	1.27	0.00
482.00	497.00	510.33	1.18	0.00
63.00	73.00	81.33	1.05	0.71
2059.00	2318.00	2279.33	1.11	0.01
599.00	682.00	683.00	1.06	0.29
808.00	961.00	923.67	1.06	0.22

3062.00	3549.00	3506.00	1.17	0.00
1410.00	1421.00	1472.67	1.11	0.02
812.00	1012.00	941.00	1.09	0.09
1171.00	1303.00	1357.33	1.11	0.06
633.00	696.00	743.00	1.18	0.02
868.00	943.00	927.67	1.12	0.01
555.00	624.00	622.67	1.14	0.01
1786.00	1892.00	1908.67	1.11	0.01
1070.00	1189.00	1177.33	1.10	0.02
515.00	566.00	579.67	1.14	0.03
1595.00	1731.00	1755.67	1.09	0.03
608.00	659.00	658.00	1.14	0.01
508.00	562.00	568.67	1.13	0.02
985.00	1203.00	1112.33	1.03	0.56
879.00	947.00	965.67	1.01	0.89
649.00	736.00	700.67	1.20	0.00
112.00	136.00	134.00	1.20	0.05
57.00	68.00	63.00	1.05	0.70
397.00	509.00	474.00	1.09	0.18
1035.00	1130.00	1138.67	1.27	0.00
369.00	422.00	428.00	1.43	0.00
1237.00	1223.00	1260.00	-1.01	0.82
465.00	487.00	503.00	1.27	0.00
1875.00	2038.00	2095.33	1.15	0.00
1294.00	1390.00	1406.00	1.23	0.00
358.00	455.00	454.33	1.21	0.02
363.00	354.00	399.67	1.26	0.00
333.00	342.00	356.00	1.04	0.54
627.00	631.00	656.00	1.12	0.03
1465.00	1558.00	1593.67	1.17	0.00
1512.00	1533.00	1568.67	1.09	0.03
431.00	466.00	462.33	-1.11	0.03
619.00	635.00	663.33	1.04	0.53
421.00	440.00	453.67	-1.08	0.12
821.00	933.00	924.00	1.14	0.00
2954.00	2854.00	3006.33	1.09	0.03
329.00	380.00	389.33	1.10	0.15
557.00	570.00	578.67	1.28	0.00
207.00	242.00	248.33	1.13	0.15
1360.00	1554.00	1542.33	1.04	0.38
1407.00	1603.00	1641.33	1.08	0.12
1050.00	1224.00	1169.33	1.10	0.02
1149.00	1205.00	1213.00	1.20	0.00
86.00	76.00	95.00	1.22	0.10
2400.00	2574.00	2570.67	-1.11	0.00
962.00	1031.00	1018.67	1.22	0.00
1771.00	1843.00	1877.33	-1.02	0.59
470.00	569.00	564.00	1.14	0.03

892.00	962.00	953.67	1.21	0.00
390.00	451.00	429.33	-1.09	0.09
218.00	234.00	227.67	-1.10	0.13
829.00	1012.00	981.33	1.11	0.06
1661.00	1793.00	1811.33	1.23	0.00
1328.00	1460.00	1484.33	1.15	0.00
91.00	89.00	106.00	1.00	1.00
1751.00	1946.00	1980.67	1.05	0.27
870.00	1039.00	985.33	1.37	0.00
805.00	967.00	929.33	1.22	0.00
1036.00	1192.00	1150.33	1.32	0.00
706.00	868.00	833.67	1.02	0.73
942.00	918.00	974.33	1.06	0.16
1374.00	1578.00	1574.67	1.16	0.00
3.00	8.00	8.00	-1.18	0.69
562.00	652.00	671.67	-1.30	0.00
1150.00	1301.00	1274.33	1.10	0.03
448.00	506.00	477.33	-1.03	0.67
26.00	20.00	18.67	1.53	0.08
65.00	81.00	75.00	-1.24	0.04
1206.00	1574.00	1423.67	1.09	0.10
449.00	415.00	426.67	-1.05	0.45
12.00	13.00	15.67	-1.19	0.44
1734.00	1858.00	1808.33	1.00	0.93
359.00	372.00	351.33	-1.06	0.39
1839.00	2171.00	2042.00	-1.02	0.64
909.00	997.00	959.33	1.14	0.03
1136.00	1321.00	1251.00	-1.13	0.00
322.00	361.00	349.00	-1.05	0.38
638.00	714.00	686.00	1.01	0.79
4.00	3.00	3.00	1.11	1.00
21783.00	22160.00	22031.00	-1.11	0.00
29.00	31.00	32.00	1.11	0.57
152.00	156.00	175.00	1.20	0.05
194.00	179.00	191.33	1.08	0.31
362.00	373.00	392.33	-1.01	0.93
948.00	872.00	908.67	1.06	0.25
370.00	371.00	374.67	1.07	0.24
138.00	117.00	141.00	-1.21	0.03
16.00	8.00	11.33	-1.45	0.14
82.00	75.00	86.00	-1.16	0.13
4.00	7.00	4.67	2.23	0.12
0.00	0.00	0.33	-5.43	0.08
90.00	89.00	90.67	-1.04	0.73
96.00	105.00	111.00	-1.04	0.72
88.00	101.00	101.67	-1.12	0.21
2397.00	2562.00	2687.67	1.19	0.00
1.00	0.00	0.33	-3.21	0.38

216.00	238.00	249.33	1.25	0.00
4061.00	4276.00	4567.33	-1.46	0.00
2751.00	2677.00	2788.33	1.02	0.53
2670.00	2940.00	2898.00	1.05	0.24
631.00	768.00	732.67	1.08	0.14
3196.00	3527.00	3409.00	1.15	0.00
594.00	795.00	685.33	1.28	0.00
4753.00	4929.00	5041.67	-1.04	0.31
1843.00	1880.00	1972.00	1.21	0.00
4163.00	4109.00	4152.33	-1.05	0.19
98.00	154.00	139.67	-1.03	0.78
8202.00	7961.00	7933.00	1.09	0.04
311.00	416.00	377.67	1.04	0.61
198.00	294.00	261.67	1.05	0.60
898.00	1060.00	1025.00	1.09	0.05
993.00	1061.00	1057.00	-1.08	0.05
477.00	440.00	488.00	-1.21	0.00
641.00	714.00	725.33	1.17	0.00
246.00	327.00	303.33	1.05	0.59
445.00	431.00	467.00	1.22	0.00
710.00	815.00	810.67	1.19	0.00
719.00	713.00	763.33	-1.04	0.49
155.00	202.00	187.67	1.27	0.00
1405.00	1602.00	1560.00	-1.01	0.82
1146.00	1100.00	1156.33	-1.14	0.01
3044.00	3454.00	3354.00	1.15	0.00
3221.00	3405.00	3411.33	1.08	0.03
5859.00	6461.00	6411.67	1.20	0.00
142.00	178.00	163.00	1.36	0.00
214.00	239.00	223.67	-1.02	0.77
52.00	51.00	53.00	1.62	0.00
651.00	734.00	723.33	-1.08	0.12
1826.00	2109.00	2077.67	1.13	0.00
368.00	457.00	446.00	1.08	0.25
102.00	107.00	105.67	1.07	0.50
147.00	181.00	166.33	-1.15	0.06
1565.00	1759.00	1736.67	1.08	0.05
32.00	33.00	29.33	-1.01	1.00
292.00	308.00	318.67	1.00	0.99
960.00	1025.00	987.67	1.18	0.00
761.00	694.00	759.33	1.05	0.35
1930.00	1986.00	2029.00	1.07	0.10
1000.00	965.00	984.00	-1.13	0.00
1685.00	1753.00	1773.00	1.00	0.93
785.00	854.00	791.67	1.22	0.01
0.00	2.00	1.67	-4.03	0.00
520.00	485.00	496.33	-1.02	0.79
75.00	61.00	70.67	-2.48	0.00

918.00	1069.00	1037.33	1.04	0.36
2264.00	2276.00	2267.67	-1.09	0.03
888.00	1071.00	998.00	1.12	0.01
1106.00	1267.00	1200.33	-1.00	0.95
530.00	550.00	567.33	-1.04	0.48
324.00	347.00	340.00	-1.06	0.43
12.00	13.00	17.00	1.29	0.32
1223.00	1326.00	1267.67	-1.10	0.02
121.00	84.00	110.33	-1.19	0.07
877.00	843.00	887.33	-1.49	0.00
566.00	631.00	602.00	-1.10	0.05
22.00	31.00	21.33	-1.25	0.32
282.00	330.00	321.00	1.08	0.25
962.00	1183.00	1137.33	1.16	0.00
1115.00	1184.00	1164.00	-1.02	0.57
61.00	57.00	61.00	-1.27	0.05
674.00	701.00	695.67	-1.16	0.01
1302.00	1388.00	1398.67	-1.04	0.31
98.00	105.00	102.33	1.18	0.10
54.00	72.00	69.00	1.10	0.44
2071.00	2225.00	2250.00	1.07	0.12
32.00	35.00	35.33	-1.22	0.17
21.00	42.00	31.33	-1.19	0.32
1088.00	1142.00	1149.67	1.02	0.66
916.00	1024.00	1027.33	1.09	0.13
647.00	726.00	706.00	1.09	0.05
314.00	332.00	320.00	1.01	0.87
706.00	703.00	746.67	-1.04	0.41
125.00	146.00	143.67	-1.09	0.25
1698.00	1841.00	1829.67	-1.09	0.02
27.00	27.00	28.33	1.58	0.01
594.00	614.00	618.67	1.04	0.37
156.00	208.00	194.67	-1.10	0.24
378.00	435.00	403.67	-1.14	0.02
826.00	835.00	873.00	1.09	0.07
1957.00	1791.00	1929.33	1.02	0.62
2.00	1.00	1.67	-1.39	0.78
597.00	602.00	598.67	-1.06	0.38
25.00	31.00	29.33	2.69	0.00
4583.00	4402.00	4515.00	-1.09	0.04
1050.00	1367.00	1182.33	1.32	0.00
470.00	579.00	558.00	1.54	0.00
6668.00	7061.00	7093.00	1.12	0.00
6.00	12.00	9.33	1.44	0.25
2501.00	2635.00	2523.00	-1.18	0.00
4.00	6.00	5.67	-1.30	0.53
213.00	236.00	230.00	1.03	0.76
325.00	332.00	346.67	-1.07	0.21

2728.00	2564.00	2728.33	1.05	0.34
1743.00	1968.00	1838.33	1.16	0.00
2022.00	2184.00	2153.67	-1.63	0.00
429.00	425.00	450.00	1.20	0.00
1275.00	1262.00	1233.33	-1.01	0.90
100.00	99.00	105.67	1.90	0.00
1.00	3.00	3.33	-1.31	0.68
135.00	138.00	135.00	-1.12	0.18
7.00	6.00	7.33	1.03	1.00
14519.00	14052.00	14043.00	-1.57	0.00
6002.00	6626.00	6765.67	1.18	0.00
5563.00	6251.00	6251.00	1.21	0.00
54.00	60.00	61.67	-1.10	0.40
3427.00	3954.00	3948.00	1.08	0.08
1142.00	1300.00	1318.33	-1.07	0.13
108.00	118.00	113.67	-1.23	0.01
3409.00	3290.00	3296.33	-1.24	0.00
8.00	8.00	8.33	1.23	0.56
42.00	31.00	38.00	-1.51	0.00
47.00	38.00	42.33	1.12	0.47
845.00	923.00	936.33	1.02	0.65
7959.00	7651.00	7764.33	-1.09	0.05
4.00	0.00	3.33	-1.40	0.55
29.00	20.00	24.67	-1.28	0.18
631.00	565.00	592.00	-1.22	0.00
1.00	15.00	8.67	-1.59	0.26
2.00	0.00	0.67	-4.42	0.05
2961.00	3150.00	3067.33	-1.01	0.79
2401.00	2352.00	2394.33	-1.08	0.04
172.00	152.00	168.00	-1.31	0.00
897.00	846.00	873.67	-1.21	0.00
2.00	1.00	2.33	-1.70	0.37
1572.00	1790.00	1627.67	-1.34	0.00
26.00	14.00	19.67	-1.40	0.09
6.00	5.00	5.33	1.56	0.34
1334.00	1534.00	1422.00	-1.09	0.08
15.00	22.00	19.33	-1.47	0.03
394.00	365.00	352.67	-1.29	0.00
3625.00	3430.00	3568.00	-1.10	0.02
42709.00	41137.00	41155.00	1.06	0.21
27.00	23.00	24.33	-1.63	0.00
0.00	1.00	1.33	-4.25	0.01
366.00	390.00	390.00	-1.80	0.00
183.00	173.00	183.33	1.11	0.15
67.00	30.00	49.00	-4.35	0.00
10.00	10.00	13.67	1.22	0.51
88.00	94.00	91.33	1.02	0.81
2790.00	3440.00	3098.67	1.00	0.97

2.00	2.00	1.33	-2.17	0.27
735.00	732.00	758.33	-1.19	0.00
116.00	108.00	114.33	-1.02	0.84
573.00	774.00	673.00	1.38	0.00
480.00	590.00	555.33	1.19	0.00
1212.00	1326.00	1328.00	1.15	0.00
905.00	884.00	923.67	1.06	0.19
744.00	810.00	727.00	1.01	0.87
33.00	36.00	31.00	1.16	0.36
1672.00	2006.00	1879.00	1.01	0.81
218.00	244.00	227.33	-1.16	0.07
235.00	267.00	260.00	-1.17	0.01
125.00	119.00	128.67	1.04	0.68
1685.00	1880.00	1908.33	1.19	0.00
1592.00	1783.00	1663.67	1.13	0.01
406.00	512.00	460.33	-1.10	0.12
1753.00	2017.00	1980.33	1.14	0.00
1849.00	1972.00	1884.67	1.01	0.78
511.00	608.00	555.33	1.11	0.11
625.00	663.00	636.00	1.07	0.21
933.00	959.00	1003.67	1.24	0.00
1232.00	1291.00	1294.67	-1.02	0.62
3427.00	3844.00	3709.33	1.03	0.46
531.00	577.00	576.33	-1.02	0.72
181.00	157.00	166.00	-1.08	0.41
4.00	0.00	1.33	-3.09	0.06
29.00	28.00	31.33	-1.00	1.00
1193.00	1478.00	1396.00	1.11	0.03
967.00	1024.00	1077.00	1.54	0.00
2002.00	2265.00	2135.33	-1.27	0.00
693.00	665.00	718.67	1.04	0.44
11729.00	13091.00	13271.00	1.16	0.00
2890.00	2664.00	2814.33	-1.08	0.05
884.00	856.00	891.33	-1.20	0.00
1100.00	1065.00	1109.00	-1.06	0.18
788.00	978.00	924.33	1.11	0.06
6670.00	7201.00	7154.00	-1.21	0.00
1848.00	2151.00	2064.67	1.16	0.00
275.00	271.00	280.67	1.03	0.73
792.00	756.00	778.00	-1.15	0.00
409.00	454.00	458.67	-1.18	0.00
1274.00	1416.00	1390.33	-1.01	0.79
905.00	1006.00	970.67	-1.04	0.33
4.00	2.00	2.33	-2.79	0.02
81.00	72.00	74.00	-1.09	0.44
274.00	250.00	270.00	-1.14	0.03
12.00	4.00	9.67	1.24	0.50
513.00	443.00	474.00	-1.31	0.00

4035.00	4730.00	4481.33	-1.02	0.68
0.00	1.00	0.33	-5.43	0.08
375.00	411.00	391.00	-1.01	0.89
657.00	647.00	689.00	1.05	0.35
15387.00	17412.00	16973.33	1.10	0.01
24.00	32.00	32.33	1.16	0.41
59.00	82.00	72.00	1.11	0.37
203.00	235.00	231.67	1.14	0.06
7.00	8.00	7.67	-1.01	1.00
889.00	915.00	929.33	1.01	0.90
12.00	11.00	14.67	1.40	0.18
432.00	532.00	493.33	-1.12	0.03
4.00	6.00	5.33	-1.76	0.11
1156.00	1401.00	1322.33	1.00	0.99
15.00	11.00	12.33	1.01	1.00
641.00	759.00	736.00	-1.04	0.46
529.00	528.00	575.67	-1.02	0.72
6972.00	8256.00	7742.67	1.06	0.12
523.00	540.00	528.33	1.04	0.52
3099.00	3663.00	3485.33	1.20	0.00
50.00	50.00	52.00	1.19	0.18
2315.00	2478.00	2410.00	-1.05	0.16
599.00	604.00	612.33	1.00	0.98
0.00	0.00	0.00	-14.45	0.07
164.00	178.00	187.67	-1.11	0.18
1666.00	1690.00	1673.67	-1.01	0.88
1744.00	1801.00	1890.33	-1.07	0.12
23.00	16.00	19.67	1.04	0.86
2746.00	2733.00	2718.33	-1.65	0.00
3107.00	2977.00	3019.00	-1.54	0.00
3786.00	3999.00	3867.67	1.10	0.03
1505.00	1618.00	1548.67	-1.01	0.85
350.00	331.00	345.00	-1.29	0.00
84.00	89.00	85.67	-1.03	0.81
2599.00	2551.00	2670.00	-1.19	0.00
11.00	11.00	11.00	-1.26	0.43
1852.00	2099.00	2002.33	1.18	0.00
366.00	356.00	342.00	-1.22	0.02
53.00	58.00	57.00	-1.36	0.02
312.00	332.00	327.33	-1.14	0.01
412.00	421.00	422.00	-1.20	0.00
411.00	453.00	404.67	-1.03	0.71
2006.00	2238.00	2149.33	1.01	0.77
189.00	216.00	213.67	1.04	0.59
29.00	33.00	26.67	-1.24	0.24
325.00	333.00	315.00	-1.00	0.97
1.00	0.00	0.33	-4.69	0.13
3.00	12.00	5.00	1.35	0.65

1412.00	1588.00	1520.33	1.07	0.07
1175.00	1269.00	1213.67	1.15	0.00
2965.00	3151.00	3075.67	-1.13	0.00
217.00	231.00	228.67	-1.30	0.00
44.00	45.00	53.33	-1.07	0.62
484.00	504.00	477.67	1.59	0.00
4979.00	5097.00	5063.67	1.02	0.66
986.00	1035.00	987.67	-1.06	0.27
955.00	1157.00	1069.67	1.42	0.00
1953.00	2233.00	2115.33	1.19	0.00
654.00	810.00	748.33	1.26	0.00
731.00	818.00	821.00	1.11	0.04
1675.00	1923.00	1799.33	1.03	0.55
3114.00	3290.00	3391.33	1.14	0.00
379.00	360.00	387.67	1.13	0.03
1570.00	1490.00	1593.33	-1.15	0.00
3472.00	3334.00	3427.67	1.58	0.00
11.00	9.00	10.33	-1.47	0.18
644.00	724.00	693.00	1.01	0.79
22.00	28.00	27.33	1.49	0.03
909.00	890.00	910.00	-1.16	0.00
5130.00	5416.00	5209.00	1.07	0.17
8.00	6.00	6.67	1.50	0.30
102.00	69.00	82.33	1.05	0.67
1299.00	1207.00	1270.33	-1.27	0.00
597.00	550.00	604.33	-1.26	0.00
23000.00	26269.00	25027.00	1.14	0.00
4229.00	4137.00	4263.33	1.05	0.25
386.00	381.00	378.67	-1.17	0.01
566.00	485.00	483.67	-1.12	0.29
1141.00	1108.00	1122.00	-1.25	0.00
3192.00	3366.00	3348.33	-1.03	0.43
1423.00	1496.00	1500.00	-1.04	0.33
1503.00	1408.00	1427.67	-1.21	0.00
684.00	659.00	670.00	-1.21	0.00
2227.00	2189.00	2160.67	-1.11	0.04
5.00	7.00	5.33	1.12	0.86
7478.00	6737.00	7143.33	-1.26	0.00
639.00	628.00	584.00	-1.23	0.01
2.00	1.00	2.33	-1.15	1.00
2110.00	2071.00	2119.67	-1.08	0.05
5177.00	5294.00	5325.00	-1.21	0.00
3126.00	3240.00	3217.33	1.19	0.00
924.00	1238.00	1129.33	1.32	0.00
1131.00	1250.00	1233.67	-1.10	0.02
1994.00	2201.00	2133.00	1.18	0.00
1053.00	1112.00	1065.33	1.01	0.85
81.00	90.00	78.00	-1.13	0.29

2528.00	2515.00	2584.33	-1.17	0.00
15.00	6.00	7.67	1.14	0.79
424.00	431.00	419.33	-1.11	0.09
986.00	991.00	1011.33	-1.01	0.87
12.00	15.00	14.00	1.01	1.00
3288.00	3143.00	3189.00	-1.50	0.00
894.00	841.00	866.33	-1.26	0.00
6.00	3.00	3.00	-2.20	0.08
608.00	706.00	682.67	1.19	0.00
1395.00	1559.00	1529.67	1.05	0.20
982.00	1025.00	1080.33	1.02	0.70
542.00	650.00	642.33	1.21	0.00
1577.00	1651.00	1715.33	1.17	0.00
404.00	417.00	442.33	1.09	0.19
1890.00	2039.00	2134.00	1.18	0.00
54.00	61.00	57.67	-1.05	0.73
691.00	954.00	854.33	1.15	0.02
728.00	794.00	833.33	1.36	0.00
622.00	742.00	725.33	1.22	0.00
1675.00	1732.00	1767.00	1.17	0.00
1150.00	1323.00	1328.33	1.25	0.00
256.00	276.00	292.00	-1.03	0.66
596.00	648.00	663.00	1.23	0.00
354.00	371.00	397.00	1.10	0.13
656.00	742.00	739.67	1.10	0.04
275.00	291.00	295.33	1.27	0.00
343.00	414.00	387.67	1.03	0.61
557.00	761.00	695.00	1.06	0.41
341.00	407.00	412.00	1.25	0.00
1047.00	1267.00	1218.00	1.09	0.07
897.00	898.00	960.33	1.16	0.00
1218.00	1205.00	1342.67	1.20	0.00
37.00	44.00	40.33	1.06	0.72
638.00	646.00	691.00	1.13	0.02
1195.00	1299.00	1380.33	1.09	0.11
1162.00	1278.00	1249.33	1.36	0.00
821.00	916.00	938.00	1.25	0.00
974.00	1075.00	1096.33	1.15	0.01
1923.00	2083.00	2124.33	1.20	0.00
3513.00	3974.00	3968.00	1.05	0.26
368.00	410.00	417.67	1.37	0.00
281.00	333.00	315.00	1.13	0.05
1699.00	1894.00	1828.00	1.16	0.00
3247.00	3541.00	3593.33	1.10	0.01
851.00	1001.00	990.00	1.17	0.00
738.00	856.00	847.00	1.18	0.00
1593.00	1709.00	1803.00	1.26	0.00
999.00	1098.00	1098.67	1.31	0.00

867.00	890.00	954.67	1.15	0.02
870.00	915.00	942.00	1.07	0.15
2791.00	2914.00	3000.33	1.05	0.23
2041.00	2361.00	2375.33	1.28	0.00
12.00	23.00	14.67	-1.10	0.77
537.00	614.00	603.33	-1.04	0.39
15052.00	17571.00	16894.33	-1.18	0.00
1.00	8.00	7.67	-1.49	0.16
2.00	1.00	2.33	-1.84	0.27
27.00	34.00	35.00	1.31	0.14
548.00	520.00	570.00	-1.03	0.55
870.00	893.00	925.33	1.07	0.12
967.00	1040.00	1051.33	1.03	0.47
791.00	931.00	842.00	1.25	0.00
2740.00	3038.00	2854.33	1.11	0.02
1778.00	1948.00	1839.67	-1.12	0.01
911.00	1043.00	1079.33	1.11	0.06
4.00	0.00	2.33	-1.01	1.00
268.00	253.00	282.67	1.07	0.36
197.00	197.00	194.67	-1.18	0.03
38.00	49.00	52.00	-1.08	0.57
422.00	476.00	483.33	1.15	0.02
111.00	144.00	134.67	1.35	0.00
344.00	460.00	386.33	-1.06	0.45
128.00	141.00	138.33	-1.31	0.00
87.00	124.00	104.67	1.04	0.77
544.00	608.00	581.67	1.21	0.00
417.00	552.00	507.67	1.31	0.00
536.00	517.00	530.33	-1.01	0.88
13.00	19.00	17.33	1.09	0.77
1309.00	1237.00	1307.00	-1.33	0.00
1077.00	1416.00	1225.00	1.21	0.00
92.00	68.00	81.67	-1.56	0.00
3704.00	3847.00	3810.33	-1.19	0.00
1618.00	1688.00	1663.33	1.10	0.02
1603.00	1657.00	1692.00	1.04	0.35
2395.00	2608.00	2599.67	1.01	0.83
116.00	141.00	123.33	-1.00	1.00
1616.00	1882.00	1777.67	1.01	0.80
2304.00	2449.00	2392.67	1.10	0.01
534.00	576.00	560.67	1.58	0.00
732.00	770.00	790.33	1.27	0.00
514.00	522.00	510.33	-1.31	0.00
91.00	109.00	103.33	1.04	0.68
2250.00	2424.00	2394.33	1.29	0.00
1994.00	1972.00	1980.00	-1.12	0.01
352.00	278.00	308.67	-1.24	0.00
194.00	209.00	200.67	-1.17	0.02

1990.00	2141.00	2154.00	-1.12	0.00
5.00	5.00	4.33	-2.30	0.02
1012.00	1096.00	1045.33	1.02	0.68
114.00	84.00	98.33	1.35	0.01
2087.00	2217.00	2126.00	-1.10	0.06
3465.00	3405.00	3596.00	1.18	0.00
2412.00	2714.00	2500.67	-1.18	0.00
30.00	28.00	26.00	1.03	0.88
2223.00	2344.00	2323.67	-1.03	0.43
455.00	471.00	472.67	-1.34	0.00
845.00	757.00	840.00	-1.08	0.13
10241.00	9935.00	10127.33	-1.16	0.00
2471.00	2728.00	2654.67	1.03	0.42
2884.00	3373.00	3202.33	1.71	0.00
262.00	256.00	282.00	1.18	0.02
1717.00	1799.00	1803.00	-1.03	0.50
1449.00	1351.00	1370.33	-1.13	0.02
377.00	409.00	421.00	-1.16	0.01
502.00	455.00	481.00	-1.03	0.57
1225.00	1394.00	1287.00	1.17	0.00
2521.00	2611.00	2663.00	1.33	0.00
736.00	784.00	765.33	1.38	0.00
509.00	629.00	608.67	1.23	0.00
84.00	87.00	94.00	-1.31	0.00
224.00	242.00	249.00	-1.02	0.79
33.00	31.00	29.67	-1.06	0.78
1125.00	1331.00	1253.67	-1.16	0.00
653.00	641.00	651.33	-1.18	0.00
820.00	891.00	884.67	1.01	0.82
406.00	455.00	463.67	1.21	0.00
1806.00	1801.00	1806.67	-1.01	0.82
614.00	746.00	677.67	-1.02	0.70
1940.00	2019.00	1997.67	-1.15	0.00
441.00	539.00	498.33	1.22	0.00
879.00	1007.00	1021.67	1.02	0.77
248.00	305.00	284.33	-1.13	0.05
681.00	678.00	730.00	1.10	0.06
77.00	89.00	84.67	-2.29	0.00
841.00	839.00	832.00	1.40	0.00
3907.00	4379.00	4372.33	1.21	0.00
824.00	1013.00	936.00	-1.00	0.96
1723.00	1867.00	1898.33	1.13	0.00
248.00	275.00	284.33	-1.09	0.18
5.00	9.00	6.33	1.17	0.74
3.00	1.00	3.00	-1.87	0.21
14.00	17.00	17.67	1.53	0.07
386.00	439.00	397.33	-1.01	0.90
661.00	802.00	743.00	-1.03	0.55

15.00	9.00	12.67	1.07	0.82
1687.00	1856.00	1876.33	1.13	0.00
81.00	113.00	90.67	-2.20	0.00
470.00	469.00	477.33	1.08	0.13
15.00	10.00	9.67	-1.01	1.00
612.00	633.00	635.67	-1.30	0.00
207.00	226.00	222.00	1.10	0.15
4.00	6.00	5.67	-1.36	0.44
981.00	1048.00	1042.00	1.06	0.17
1606.00	1771.00	1790.33	1.13	0.01
85.00	73.00	86.00	1.20	0.10
11.00	18.00	15.67	-1.65	0.01
1573.00	1795.00	1654.33	1.04	0.38
377.00	327.00	373.00	-1.02	0.79
869.00	950.00	915.00	-1.11	0.04
1372.00	1501.00	1503.33	1.20	0.00
1180.00	1221.00	1218.67	1.11	0.01
2032.00	2289.00	2204.67	1.15	0.00
222.00	205.00	197.00	-1.26	0.01
138.00	131.00	129.67	-1.03	0.74
574.00	550.00	558.00	-1.20	0.00
113.00	98.00	96.67	-1.18	0.15
2036.00	2226.00	2095.33	-1.08	0.09
363.00	438.00	399.67	-1.15	0.01
1457.00	1491.00	1565.67	1.27	0.00
213.00	250.00	229.67	1.01	0.90
55.00	54.00	54.00	-1.08	0.59
2674.00	2661.00	2743.67	-1.29	0.00
678.00	795.00	773.00	1.01	0.83
1212.00	1233.00	1293.67	1.16	0.00
369.00	384.00	391.00	1.07	0.21
3.00	5.00	5.00	-2.60	0.00
423.00	462.00	461.67	1.11	0.04
633.00	576.00	625.67	-1.15	0.01
475.00	513.00	498.33	1.09	0.09
3580.00	4236.00	3991.33	-1.18	0.00
18.00	22.00	19.33	-1.29	0.22
107.00	81.00	98.67	1.24	0.06
187.00	131.00	169.67	1.13	0.22
20.00	18.00	19.33	1.06	0.79
96.00	105.00	102.00	-1.24	0.02
30.00	29.00	29.33	1.29	0.14
104.00	128.00	111.67	-1.12	0.25
977.00	995.00	988.67	1.16	0.00
79.00	76.00	78.33	1.14	0.23
2738.00	2647.00	2792.00	-1.17	0.00
12.00	8.00	10.00	-1.01	1.00
557.00	631.00	591.00	-1.34	0.00

1425.00	1368.00	1407.00	1.13	0.00
311.00	355.00	336.67	-1.31	0.00
2801.00	2736.00	2840.33	-1.02	0.52
3.00	3.00	2.67	-1.25	0.82
4.00	4.00	3.33	-1.31	0.68
904.00	919.00	953.33	1.38	0.00
486.00	432.00	456.67	1.11	0.10
225.00	251.00	244.33	1.26	0.00
3.00	2.00	2.33	-1.01	1.00
2266.00	2514.00	2427.67	1.16	0.00
1843.00	2036.00	2049.67	1.31	0.00
571.00	548.00	598.00	1.10	0.06
576.00	566.00	617.00	1.02	0.72
1705.00	1787.00	1851.33	1.16	0.00
414.00	343.00	406.00	-1.06	0.39
491.00	526.00	547.33	-1.03	0.56
263.00	301.00	279.00	1.19	0.01
334.00	389.00	377.33	1.07	0.24
2352.00	2479.00	2471.67	1.44	0.00
457.00	525.00	510.33	1.06	0.25
2874.00	2770.00	2826.67	1.04	0.38
2821.00	2909.00	2851.67	-1.36	0.00
24.00	18.00	22.00	3.06	0.00
107.00	147.00	134.67	1.10	0.31
1425.00	1536.00	1455.00	-1.10	0.05
428.00	422.00	414.67	-1.14	0.07
578.00	524.00	564.00	1.04	0.52
1997.00	1997.00	2053.33	1.10	0.01
3918.00	4248.00	4149.00	1.06	0.10
2380.00	2727.00	2630.67	1.07	0.07
663.00	602.00	657.33	1.08	0.14
120.00	142.00	124.00	1.10	0.41
226.00	319.00	286.00	1.10	0.23
19.00	26.00	21.00	1.02	0.93
1105.00	1219.00	1209.33	1.18	0.00
1665.00	1811.00	1772.33	1.11	0.00
703.00	725.00	747.33	-1.02	0.68
597.00	676.00	692.33	-1.27	0.00
14.00	8.00	8.67	-1.50	0.19
1735.00	1685.00	1782.33	-1.12	0.00
3378.00	3508.00	3559.00	-1.00	0.92
1423.00	1730.00	1660.00	1.23	0.00
1478.00	1645.00	1594.67	-1.02	0.67
763.00	927.00	881.67	1.14	0.05
6035.00	6541.00	6439.67	1.08	0.02
1315.00	1352.00	1310.00	-1.05	0.26
164.00	211.00	187.00	-1.09	0.30
599.00	536.00	554.67	-1.24	0.00

11096.00	11478.00	11497.00	1.04	0.26
1461.00	1601.00	1706.67	1.32	0.00
5712.00	6089.00	5919.33	1.03	0.42
292.00	321.00	317.00	1.09	0.13
1271.00	1354.00	1417.67	1.09	0.05
3080.00	3400.00	3343.67	-1.02	0.52
7490.00	8309.00	8271.67	1.07	0.05
3919.00	4661.00	4482.67	1.15	0.00
1923.00	1620.00	1669.33	-1.56	0.00
21.00	17.00	16.33	-1.01	1.00
179.00	163.00	169.33	-1.08	0.35
1243.00	1359.00	1389.00	1.16	0.00
1568.00	1457.00	1490.00	-1.31	0.00
11614.00	10882.00	10865.00	-1.27	0.00
145.00	109.00	127.67	-1.09	0.43
3.00	6.00	5.33	1.42	0.45
195.00	182.00	190.67	-1.62	0.00
4.00	5.00	4.00	1.07	1.00
0.00	1.00	0.33	-2.48	0.64
155.00	209.00	178.67	1.26	0.01
17.00	8.00	14.33	-1.53	0.06
83.00	61.00	79.67	-1.06	0.65
2.00	1.00	2.33	-1.43	0.63
15.00	20.00	20.00	-1.01	1.00
11.00	12.00	11.00	-1.44	0.16
7288.00	7730.00	7777.67	1.13	0.00
78.00	78.00	83.67	-1.09	0.42
361.00	430.00	379.67	-1.01	0.86
4098.00	4100.00	4128.67	-1.21	0.00
3.00	2.00	3.00	-1.23	0.83
506.00	564.00	557.33	-1.29	0.00
3697.00	3812.00	3855.67	-1.03	0.35
33.00	46.00	40.33	1.13	0.46
2010.00	2280.00	2171.00	1.04	0.29
56.00	72.00	71.33	1.03	0.88
28.00	32.00	36.00	2.16	0.00
171.00	182.00	170.00	-1.01	0.92
289.00	368.00	342.67	-1.17	0.01
200.00	306.00	251.33	1.16	0.15
556.00	514.00	539.67	-1.26	0.00
16.00	34.00	30.33	1.19	0.42
66.00	68.00	69.67	-1.38	0.00
51.00	57.00	58.00	-1.18	0.16
65.00	87.00	80.00	-1.31	0.01
115.00	136.00	133.67	-1.31	0.00
379.00	365.00	406.33	-1.06	0.39
3.00	4.00	3.00	-1.88	0.18
7.00	4.00	10.00	-1.75	0.03

19.00	13.00	12.67	-1.35	0.23
184.00	209.00	196.67	1.18	0.02
3573.00	3452.00	3562.67	-1.13	0.00
25535.00	30050.00	29188.67	1.25	0.00
1508.00	1523.00	1536.67	1.34	0.00
84.00	80.00	91.00	1.13	0.25
482.00	504.00	519.33	-1.24	0.00
274.00	291.00	283.33	-1.06	0.33
565.00	603.00	618.67	-1.02	0.62
1417.00	1539.00	1543.00	1.16	0.00
1711.00	1828.00	1800.00	-1.01	0.89
135.00	107.00	111.00	-1.38	0.00
10.00	15.00	10.00	-1.88	0.01
78.00	77.00	84.67	-1.01	0.90
72.00	111.00	98.67	-1.04	0.74
14.00	10.00	11.33	2.21	0.01
2160.00	2256.00	2259.67	-1.15	0.00
414.00	356.00	412.33	1.05	0.52
274.00	311.00	300.00	1.17	0.02
1438.00	1573.00	1520.33	1.14	0.01
995.00	1044.00	1054.33	1.08	0.06
186.00	158.00	187.33	1.05	0.61
0.00	0.00	0.00	-9.06	0.27
549.00	736.00	669.67	-1.32	0.00
1677.00	1682.00	1655.00	-1.07	0.12
563.00	611.00	615.33	1.15	0.01
13.00	8.00	9.00	-1.86	0.02
963.00	1033.00	996.33	1.32	0.00
507.00	531.00	533.00	1.10	0.11
3338.00	3461.00	3407.67	1.03	0.50
124.00	144.00	130.67	-1.66	0.00
862.00	893.00	885.33	-1.01	0.87
1115.00	1226.00	1213.00	-1.10	0.03
228.00	239.00	237.00	-1.16	0.03
202.00	191.00	195.00	-1.17	0.03
21.00	21.00	20.00	-1.01	1.00
4.00	17.00	10.00	1.02	1.00
4.00	12.00	9.67	1.35	0.34
162.00	161.00	164.67	-1.23	0.00
45.00	42.00	46.67	1.14	0.35
2091.00	2384.00	2270.33	1.27	0.00
151.00	188.00	169.33	-1.12	0.14
484.00	460.00	493.67	-1.06	0.24
2177.00	2393.00	2295.33	1.04	0.27
1049.00	1053.00	1089.67	-1.15	0.00
5716.00	6243.00	6123.67	1.03	0.32
363.00	374.00	363.67	-1.01	0.85
439.00	472.00	473.67	1.20	0.00

449.00	492.00	472.33	-1.04	0.48
1890.00	1743.00	1765.00	-1.05	0.37
64.00	69.00	61.33	-1.41	0.00
138.00	176.00	166.33	1.06	0.50
1622.00	1750.00	1659.67	-1.27	0.00
1516.00	1611.00	1571.33	1.62	0.00
795.00	854.00	815.33	-1.24	0.00
4.00	6.00	7.33	-1.01	1.00
3.00	4.00	2.67	-1.01	1.00
4.00	2.00	3.67	-2.26	0.03
7677.00	8069.00	8029.00	-1.70	0.00
2903.00	2813.00	2833.67	1.11	0.02
522.00	530.00	522.33	-1.01	0.85
1084.00	1018.00	1106.00	-1.11	0.03
124.00	121.00	116.67	-1.18	0.07
1.00	4.00	3.00	-1.01	1.00
102.00	94.00	94.00	-1.48	0.00
1345.00	1569.00	1563.00	1.21	0.00
1938.00	1815.00	1826.33	-1.20	0.00
550.00	570.00	592.33	1.13	0.01
34.00	35.00	36.33	1.19	0.28
1326.00	1544.00	1483.33	1.03	0.43
3.00	2.00	3.00	-1.99	0.13
478.00	540.00	535.00	1.13	0.02
2318.00	2447.00	2496.67	1.00	0.99
792.00	873.00	876.33	1.09	0.06
466.00	505.00	504.33	1.03	0.55
996.00	1090.00	1062.00	1.20	0.00
1999.00	1958.00	2053.33	-1.22	0.00
1092.00	1283.00	1256.67	1.13	0.01
3413.00	3653.00	3572.00	1.03	0.38
139.00	174.00	158.00	1.35	0.00
862.00	893.00	904.00	-1.02	0.70
460.00	487.00	508.67	1.12	0.06
679.00	779.00	788.67	-1.12	0.03
592.00	642.00	667.00	-1.10	0.08
536.00	534.00	570.33	-1.14	0.01
6.00	6.00	5.00	1.06	1.00
766.00	916.00	891.00	1.16	0.00
2131.00	2292.00	2244.33	-1.11	0.00
917.00	1035.00	1006.33	1.20	0.00
853.00	988.00	980.00	1.12	0.02
591.00	644.00	624.67	1.09	0.07
2253.00	2389.00	2409.33	-1.13	0.00
470.00	474.00	488.67	1.18	0.00
25374.00	25568.00	25336.33	-1.10	0.01
105.00	115.00	118.33	1.14	0.16
24.00	23.00	19.33	1.07	0.83

480.00	481.00	514.33	1.07	0.29
3344.00	3499.00	3540.00	-1.08	0.01
1049.00	1079.00	1119.00	1.04	0.44
840.00	922.00	901.33	-2.31	0.00
2347.00	2377.00	2371.00	-1.30	0.00
21.00	20.00	16.33	-2.10	0.00
262.00	263.00	284.67	1.12	0.08
4.00	5.00	6.00	-1.18	0.75
190.00	194.00	194.67	-1.02	0.80
226.00	221.00	223.33	-1.17	0.02
1181.00	1314.00	1237.00	-1.12	0.01
349.00	335.00	345.00	1.06	0.35
800.00	770.00	828.33	1.05	0.34
425.00	469.00	465.33	1.08	0.14
3135.00	3102.00	3202.67	-1.17	0.00
1580.00	1783.00	1743.33	1.27	0.00
4340.00	4909.00	4750.00	1.19	0.00
5625.00	5954.00	5991.00	1.21	0.00
1431.00	1555.00	1552.00	1.20	0.00
559.00	634.00	633.33	1.09	0.08
2792.00	2770.00	2853.33	1.05	0.20
344.00	393.00	392.33	1.31	0.00
4.00	6.00	4.67	-1.15	0.86
323.00	362.00	353.33	1.20	0.00
431.00	526.00	473.67	1.18	0.01
295.00	355.00	338.67	1.22	0.00
35.00	28.00	29.67	1.19	0.34
503.00	532.00	535.00	1.31	0.00
1763.00	1765.00	1783.67	-1.13	0.00
322.00	359.00	346.33	1.05	0.43
122.00	116.00	126.67	-1.07	0.43
46.00	48.00	54.00	1.30	0.06
488.00	505.00	535.33	1.14	0.03
594.00	631.00	677.33	1.13	0.05
2965.00	3333.00	3195.67	1.17	0.00
1435.00	1372.00	1481.00	-1.00	0.92
118.00	118.00	119.00	1.36	0.00
571.00	658.00	613.67	1.09	0.10
3039.00	3316.00	3327.00	1.09	0.01
79.00	91.00	91.00	-1.16	0.11
29.00	28.00	26.00	1.24	0.23
501.00	512.00	514.33	1.02	0.68
14.00	26.00	20.33	-1.13	0.56
900.00	1172.00	1086.33	1.46	0.00
285.00	320.00	327.00	1.15	0.04
3540.00	3792.00	3667.67	-1.16	0.00
9901.00	9911.00	9959.00	-1.28	0.00
1771.00	1892.00	1891.67	-1.13	0.00

602.00	600.00	603.00	1.13	0.01
2490.00	2781.00	2683.00	1.07	0.07
1414.00	1471.00	1504.33	1.07	0.09
3001.00	3099.00	3030.67	1.04	0.38
1718.00	1631.00	1606.00	1.25	0.00
1312.00	1439.00	1390.00	1.05	0.29
3104.00	3190.00	3129.33	-1.01	0.73
4500.00	4636.00	4557.00	1.11	0.00
1887.00	1718.00	1775.33	-1.22	0.00
539.00	610.00	612.67	1.13	0.03
1011.00	1113.00	1088.00	1.14	0.00
1074.00	1238.00	1182.33	1.22	0.00
1576.00	1633.00	1622.00	1.13	0.00
215.00	217.00	251.67	1.06	0.56
758.00	930.00	840.33	1.19	0.00
1640.00	1706.00	1730.33	1.05	0.25
8.00	12.00	13.67	1.19	0.58
1066.00	1178.00	1164.33	1.05	0.33
1737.00	2010.00	1933.00	1.13	0.00
2083.00	2234.00	2237.67	1.13	0.00
3454.00	3637.00	3572.00	-1.08	0.03
1366.00	1508.00	1445.67	1.12	0.01
836.00	960.00	925.33	-1.02	0.63
233.00	253.00	267.67	-1.75	0.00
1193.00	1335.00	1272.00	1.18	0.00
1184.00	1371.00	1357.67	1.31	0.00
1531.00	1663.00	1719.00	1.17	0.00
23.00	7.00	16.33	1.10	0.72
313.00	292.00	329.67	-1.01	0.87
5.00	10.00	9.33	1.31	0.41
378.00	390.00	407.33	-1.02	0.82
18.00	21.00	21.33	-1.09	0.62
854.00	890.00	887.67	1.15	0.00
1699.00	1865.00	1865.33	-1.13	0.00
15.00	10.00	13.67	-1.48	0.08
875.00	877.00	894.67	1.09	0.06
13.00	14.00	13.33	-1.06	0.91
764.00	751.00	731.00	-1.22	0.00
38.00	58.00	46.00	1.64	0.00
55.00	58.00	62.67	1.01	1.00
573.00	550.00	583.67	1.12	0.02
128.00	187.00	148.33	1.26	0.04
27.00	22.00	27.00	-1.53	0.02
1789.00	1684.00	1771.67	-1.22	0.00
585.00	707.00	675.33	1.13	0.02
3.00	3.00	4.33	1.58	0.39
4.00	3.00	5.00	1.62	0.32
82.00	80.00	85.33	1.25	0.04

1737.00	1925.00	1911.67	-1.08	0.06
8855.00	9182.00	9500.67	1.11	0.00
996.00	1054.00	1089.67	1.23	0.00
23.00	18.00	24.33	1.47	0.06
623.00	603.00	604.67	1.09	0.14
160.00	125.00	139.00	-1.62	0.00
1780.00	1816.00	1873.33	-1.02	0.62
3.00	6.00	3.67	1.52	0.49
71.00	95.00	85.67	1.13	0.29
4100.00	4253.00	4311.33	1.10	0.01
1996.00	2214.00	2282.33	1.21	0.00
21.00	18.00	20.33	1.06	0.79
12.00	10.00	15.67	1.08	0.87
704.00	686.00	704.67	-1.03	0.53
8171.00	8748.00	8853.00	1.02	0.60
3147.00	3494.00	3390.00	-1.01	0.81
1504.00	1808.00	1715.00	-1.01	0.85
6.00	6.00	5.00	-1.21	0.73
1452.00	1683.00	1630.00	-1.17	0.00
2501.00	2447.00	2492.00	1.02	0.62
58.00	87.00	75.67	1.48	0.00
1601.00	1790.00	1724.33	1.05	0.20
664.00	683.00	708.67	-1.16	0.00
1089.00	1056.00	1095.67	-1.07	0.14
1055.00	1117.00	1121.33	-1.02	0.67
435.00	491.00	464.00	-1.10	0.10
3.00	8.00	7.00	1.15	0.76
295.00	265.00	305.00	-1.16	0.03
26573.00	30272.00	29265.33	-1.06	0.07
136.00	161.00	154.33	1.15	0.09
252.00	253.00	259.67	-1.08	0.27
2730.00	3091.00	3044.33	1.16	0.00
15.00	28.00	22.67	-1.24	0.25
35.00	36.00	35.33	2.88	0.00
799.00	927.00	881.33	-1.05	0.31
18.00	26.00	19.33	1.96	0.00
227.00	225.00	240.00	-1.01	0.88
731.00	865.00	839.67	-1.02	0.75
127.00	138.00	136.00	1.11	0.19
2067.00	2245.00	2135.67	1.03	0.43
485.00	443.00	466.00	-1.00	0.99
27.00	20.00	24.33	4.71	0.00
300.00	281.00	281.67	-1.11	0.14
13.00	9.00	10.33	2.72	0.00
511.00	564.00	557.33	-1.37	0.00
1.00	5.00	3.00	-2.31	0.05
749.00	761.00	808.67	1.05	0.30
7.00	7.00	5.33	1.21	0.73

0.00	0.00	0.67	-1.87	0.70
10.00	7.00	7.67	-1.36	0.38
38.00	28.00	33.67	-1.09	0.56
39.00	54.00	43.33	1.44	0.02
24.00	25.00	21.00	-1.53	0.02
18.00	19.00	19.00	-1.07	0.86
708.00	737.00	730.00	1.08	0.12
1222.00	1077.00	1134.67	-1.32	0.00
297.00	314.00	315.67	-1.30	0.00
3732.00	4080.00	4020.00	-1.05	0.13
190.00	199.00	202.00	1.48	0.00
1096.00	1161.00	1162.33	1.04	0.33
959.00	1103.00	1081.67	1.15	0.00
705.00	765.00	754.00	1.08	0.13
938.00	1038.00	1034.33	1.22	0.00
982.00	1120.00	1129.00	1.17	0.01
1852.00	1926.00	2018.33	-1.12	0.01
233.00	209.00	237.33	1.01	0.92
854.00	793.00	844.67	-1.06	0.25
5988.00	6422.00	6303.33	-1.16	0.00
1635.00	1555.00	1617.67	-1.07	0.11
128.00	124.00	132.67	-1.02	0.80
402.00	340.00	386.67	1.00	0.98
513.00	529.00	519.33	1.17	0.00
978.00	915.00	957.00	1.12	0.02
943.00	965.00	936.67	1.01	0.88
1206.00	1328.00	1301.33	-1.06	0.15
393.00	374.00	397.00	-1.04	0.48
1015.00	1102.00	1081.00	1.28	0.00
497.00	463.00	487.67	-1.42	0.00
1105.00	1357.00	1179.67	-1.01	0.91
1565.00	1664.00	1669.67	-1.07	0.09
28.00	43.00	39.33	-2.14	0.00
78.00	76.00	78.00	1.12	0.33
956.00	1047.00	1059.33	-1.05	0.31
51.00	37.00	48.33	1.03	0.87
806.00	988.00	949.33	-1.14	0.01
453.00	540.00	499.00	1.08	0.17
10.00	10.00	12.67	1.01	1.00
167.00	161.00	180.33	-1.21	0.01
693.00	810.00	752.33	1.12	0.02
5970.00	6046.00	6048.67	-1.32	0.00
5.00	12.00	10.00	-1.32	0.36
40.00	46.00	44.67	1.11	0.46
1072.00	1223.00	1284.00	1.13	0.04
1326.00	1295.00	1365.67	1.09	0.03
479.00	518.00	527.00	1.45	0.00
1378.00	1425.00	1375.33	1.17	0.00

10.00	1.00	6.00	-2.77	0.00
0.00	0.00	0.00	-11.75	0.13
2644.00	2609.00	2729.67	1.05	0.26
111.00	151.00	146.33	-1.13	0.23
725.00	863.00	792.00	-1.12	0.02
1508.00	1653.00	1573.33	-1.06	0.22
1203.00	1272.00	1266.33	-1.01	0.90
763.00	994.00	878.33	1.11	0.08
663.00	775.00	724.33	1.15	0.01
672.00	692.00	672.33	-1.05	0.38
619.00	663.00	622.67	-1.07	0.25
66.00	73.00	67.67	1.19	0.13
310.00	338.00	342.00	-1.06	0.31
83.00	57.00	69.67	1.15	0.25
36.00	41.00	39.00	-1.65	0.00
2128.00	2441.00	2444.00	1.02	0.69
2028.00	2202.00	2140.00	1.24	0.00
9.00	12.00	10.33	1.60	0.13
1.00	3.00	2.33	-1.57	0.50
1.00	0.00	1.33	-3.33	0.03
1363.00	1395.00	1428.67	1.03	0.51
387.00	400.00	403.00	1.02	0.68
736.00	810.00	782.33	1.15	0.01
476.00	535.00	530.33	-1.07	0.15
1538.00	1584.00	1572.33	-1.08	0.03
402.00	475.00	433.67	-1.10	0.12
194.00	209.00	211.00	-1.39	0.00
417.00	498.00	486.00	-1.02	0.73
50.00	34.00	39.67	1.11	0.52
6.00	6.00	8.67	-1.28	0.46
9.00	7.00	7.67	1.26	0.54
143.00	120.00	120.00	-1.18	0.15
124.00	114.00	119.00	-1.15	0.12
8.00	9.00	7.33	1.03	1.00
1.00	5.00	2.67	-3.43	0.00
2351.00	2618.00	2510.67	-1.13	0.00
1345.00	1441.00	1429.00	-1.08	0.06
3499.00	3723.00	3702.00	-1.11	0.01
1175.00	1193.00	1214.67	-1.01	0.80
2637.00	2733.00	2732.00	1.03	0.44
788.00	825.00	834.33	-1.09	0.04
43.00	64.00	54.00	1.48	0.00
21091.00	20918.00	21336.00	-1.10	0.02
302.00	272.00	301.33	-1.17	0.01
6006.00	6704.00	6566.00	1.20	0.00
134.00	163.00	153.00	1.11	0.24
515.00	453.00	503.67	1.02	0.69
42209.00	45257.00	43499.00	1.14	0.00

4297.00	4331.00	4466.33	-1.06	0.10
157.00	186.00	171.00	1.16	0.08
7935.00	8779.00	8722.33	-1.00	0.94
874.00	940.00	949.67	-1.01	0.91
34.00	33.00	30.33	-1.22	0.21
1.00	1.00	2.33	-1.29	0.81
261.00	340.00	305.67	1.02	0.78
2017.00	1849.00	1890.00	-1.22	0.00
1293.00	1179.00	1264.33	-1.18	0.00
12.00	20.00	20.33	-1.46	0.05
1128.00	1082.00	1116.67	-1.03	0.48
529.00	558.00	565.67	-1.03	0.56
0.00	2.00	1.33	-2.63	0.12
797.00	850.00	847.00	-1.02	0.69
4047.00	4637.00	4381.33	1.03	0.52
1259.00	1366.00	1340.00	1.18	0.00
351.00	375.00	387.67	1.29	0.00
347.00	384.00	377.00	-1.07	0.19
606.00	580.00	560.67	-1.44	0.00
8.00	5.00	8.33	3.93	0.00
550.00	489.00	536.33	1.01	0.80
4284.00	4705.00	4585.33	1.21	0.00
1620.00	1713.00	1764.00	1.10	0.03
1132.00	1144.00	1181.67	1.19	0.00
83.00	90.00	85.00	-1.05	0.65
1006.00	1229.00	1137.67	1.11	0.02
1319.00	1430.00	1418.00	1.16	0.00
2240.00	2361.00	2340.33	-1.01	0.87
158.00	164.00	149.67	-1.03	0.73
692.00	696.00	717.00	1.16	0.00
4.00	6.00	4.00	1.66	0.37
479.00	592.00	563.00	-1.03	0.62
370.00	403.00	397.67	1.31	0.00
69.00	69.00	69.67	-1.16	0.18
2.00	8.00	4.67	-1.72	0.17
3629.00	3765.00	3687.33	-1.27	0.00
0.00	0.00	0.00	-17.12	0.03
1707.00	1751.00	1752.67	-1.65	0.00
2197.00	2191.00	2316.33	-1.38	0.00
904.00	1171.00	1062.00	-1.04	0.40
54.00	49.00	55.67	-1.29	0.03
265.00	285.00	293.00	1.15	0.02
653.00	661.00	677.00	1.10	0.03
308.00	270.00	267.00	-1.25	0.02
30.00	21.00	22.67	1.03	0.94
500.00	480.00	493.33	1.00	1.00
150.00	186.00	162.33	-1.24	0.01
45.00	36.00	40.67	1.24	0.14

791.00	904.00	849.67	1.03	0.57
559.00	541.00	528.67	1.04	0.56
1196.00	1368.00	1298.00	-1.10	0.02
389.00	367.00	374.00	1.34	0.00
3804.00	4061.00	3965.00	1.12	0.01
478.00	502.00	490.00	1.04	0.41
136.00	143.00	134.33	-2.04	0.00
1506.00	1326.00	1293.00	-1.55	0.00
749.00	780.00	777.00	1.04	0.41
683.00	748.00	730.00	1.04	0.35
697.00	772.00	718.00	-1.02	0.79
489.00	568.00	543.00	1.39	0.00
58.00	47.00	48.67	1.03	0.83
201.00	205.00	214.33	-1.35	0.00
965.00	977.00	1030.33	1.06	0.23
733.00	626.00	670.00	-1.38	0.00
1953.00	2074.00	2019.00	-1.07	0.06
14.00	18.00	16.00	-1.12	0.70
133.00	85.00	106.67	-1.07	0.56
34.00	26.00	28.33	1.10	0.61
60.00	89.00	80.00	1.21	0.12
3.00	1.00	3.00	-1.23	0.83
15.00	5.00	8.67	1.35	0.40
664.00	778.00	724.00	1.06	0.21
131.00	132.00	119.33	-1.42	0.00
29.00	30.00	34.33	-1.08	0.61
2934.00	3319.00	3355.33	1.14	0.00
700.00	751.00	773.33	1.07	0.23
3.00	0.00	1.33	-2.86	0.08
5.00	3.00	3.00	-2.09	0.09
3412.00	3688.00	3525.33	1.24	0.00
700.00	694.00	740.33	-1.14	0.02
210.00	246.00	226.67	1.03	0.67
379.00	351.00	370.33	1.09	0.16
772.00	881.00	832.67	-1.09	0.08
180.00	242.00	226.67	1.11	0.19
734.00	791.00	795.33	1.02	0.65
998.00	967.00	1028.33	-1.15	0.00
2170.00	2113.00	2143.00	-1.27	0.00
1338.00	1478.00	1482.33	-1.18	0.00
480.00	600.00	537.00	-1.09	0.13
245.00	227.00	237.33	-1.21	0.00
450.00	526.00	486.67	1.24	0.00
183.00	211.00	197.33	-1.02	0.83
0.00	2.00	2.33	-1.56	0.49
57.00	77.00	67.00	1.22	0.11
1754.00	1844.00	1869.67	-1.23	0.00
921.00	1022.00	1027.00	1.15	0.00

414.00	415.00	432.00	1.02	0.71
4.00	12.00	7.67	-1.06	1.00
1111.00	1127.00	1144.33	-1.06	0.17
22.00	18.00	26.00	-1.11	0.63
1041.00	1132.00	1116.67	-1.03	0.49
416.00	410.00	391.67	-1.08	0.27
531.00	563.00	555.67	-1.07	0.16
17.00	20.00	19.33	-1.33	0.11
8.00	8.00	8.67	-1.24	0.53
143.00	162.00	151.67	1.12	0.16
1552.00	1957.00	1776.00	-1.03	0.51
16.00	44.00	28.67	-1.16	0.43
706.00	789.00	771.00	-1.00	0.99
243.00	281.00	282.00	-1.10	0.17
100.00	107.00	108.00	-1.22	0.02
471.00	614.00	566.67	1.08	0.21
449.00	597.00	530.33	1.59	0.00
127.00	147.00	142.00	1.13	0.14
1397.00	1508.00	1430.00	-1.12	0.01
32.00	16.00	21.67	-1.03	1.00
472.00	539.00	532.67	1.71	0.00
1014.00	948.00	991.00	-1.25	0.00
850.00	871.00	882.33	1.01	0.75
8.00	12.00	9.67	-1.29	0.41
1192.00	1235.00	1284.67	1.35	0.00
252.00	303.00	292.00	1.01	0.87
4846.00	5565.00	5480.00	1.14	0.00
195.00	200.00	218.67	-1.20	0.01
10122.00	11236.00	11333.33	1.07	0.08
5.00	13.00	8.67	-1.28	0.49
6.00	10.00	7.33	-1.78	0.04
602.00	594.00	631.00	-1.08	0.14
294.00	369.00	337.67	-1.23	0.00
1062.00	1067.00	1088.00	1.06	0.12
0.00	0.00	0.67	-2.30	0.46
39.00	40.00	38.67	-1.27	0.08
240.00	281.00	263.00	1.08	0.29
14.00	21.00	17.67	1.27	0.27
9.00	7.00	8.00	1.38	0.36
9.00	16.00	13.00	-2.53	0.00
47.00	50.00	46.67	-1.33	0.02
2.00	3.00	2.67	-1.38	0.65
6.00	4.00	3.67	-1.99	0.09
11.00	10.00	12.00	-1.21	0.51
6.00	4.00	5.67	-1.01	1.00
24.00	17.00	19.67	1.38	0.13
32.00	34.00	33.67	1.12	0.58
50.00	68.00	60.33	1.02	0.93

49.00	63.00	56.67	-1.25	0.06
44.00	43.00	41.67	-1.01	0.95
2.00	3.00	1.67	-1.58	0.59
0.00	2.00	1.67	-2.34	0.15
30.00	39.00	39.33	-1.36	0.02
8.00	4.00	5.67	2.33	0.07
2.00	4.00	4.67	-1.37	0.50
57.00	52.00	56.00	1.31	0.03
29.00	52.00	43.33	1.29	0.09
12.00	12.00	13.33	1.19	0.49
9.00	12.00	11.33	-1.07	0.91
19.00	17.00	15.33	1.37	0.20
16.00	23.00	18.67	-1.79	0.00
51.00	49.00	47.33	-2.33	0.00
154.00	155.00	160.67	-2.09	0.00
4.00	3.00	3.67	-1.99	0.09
20.00	20.00	21.67	-1.11	0.56
9.00	13.00	9.67	-1.25	0.47
8.00	11.00	9.33	-2.08	0.00
12.00	12.00	11.67	-1.18	0.58
48.00	34.00	38.00	1.28	0.12
9.00	16.00	11.33	-1.07	0.91
5.00	2.00	3.67	-3.68	0.00
29.00	38.00	34.33	-1.72	0.00
25.00	18.00	17.33	-1.48	0.05
20.00	9.00	11.00	-1.73	0.04
26.00	28.00	26.67	-1.44	0.02
152.00	94.00	121.67	-1.77	0.00
10.00	16.00	15.67	-1.10	0.71
15.00	10.00	11.67	-2.10	0.00
499.00	437.00	446.67	-1.99	0.00
5.00	5.00	5.00	-2.73	0.00
5.00	6.00	5.00	-3.19	0.00
3451.00	3291.00	3545.33	-1.04	0.34
269.00	311.00	309.33	-1.39	0.00
1664.00	1908.00	1795.67	-1.14	0.00
455.00	456.00	490.67	1.10	0.12
642.00	646.00	681.00	-1.06	0.25
801.00	960.00	857.67	-1.01	0.84
391.00	441.00	422.33	1.31	0.00
1391.00	1518.00	1526.33	1.02	0.67
470.00	479.00	514.67	-1.09	0.20
4299.00	4597.00	4588.33	1.02	0.68
359.00	342.00	348.00	-1.37	0.00
1590.00	1902.00	1783.00	1.01	0.85
1562.00	1654.00	1683.67	1.18	0.00
768.00	843.00	806.67	-1.04	0.41
633.00	881.00	758.33	1.12	0.12

1169.00	1479.00	1394.33	1.41	0.00
2031.00	2474.00	2263.67	1.19	0.00
2122.00	2242.00	2176.00	-1.12	0.00
1965.00	2194.00	2060.00	-1.19	0.00
943.00	966.00	958.33	-1.30	0.00
3386.00	3309.00	3394.67	-1.44	0.00
2233.00	2436.00	2374.33	-1.05	0.19
4822.00	5321.00	5270.67	-1.20	0.00
503.00	576.00	573.00	1.14	0.01
63.00	47.00	53.67	-1.07	0.62
2.00	5.00	3.67	-2.08	0.06
92.00	107.00	107.67	-1.45	0.00
1163.00	1113.00	1165.00	-1.08	0.09
332.00	350.00	352.33	1.19	0.00
1454.00	1453.00	1436.67	-1.26	0.00
452.00	466.00	467.33	-1.04	0.44
535.00	510.00	513.33	-1.08	0.19
663.00	652.00	680.33	1.00	0.95
2719.00	2752.00	2952.00	1.12	0.02
1.00	2.00	1.00	-2.81	0.15
789.00	914.00	871.00	1.24	0.00
5559.00	5733.00	5578.67	-1.14	0.00
1065.00	1108.00	1127.67	1.07	0.09
1657.00	1780.00	1806.33	1.20	0.00
248.00	268.00	290.67	1.28	0.00
2105.00	2609.00	2391.00	-1.15	0.00
148.00	191.00	172.67	1.06	0.51
1084.00	1181.00	1238.00	1.23	0.00
3117.00	3554.00	3557.00	1.18	0.00
4297.00	5150.00	4827.33	1.06	0.16
38.00	41.00	36.00	1.12	0.49
870.00	962.00	957.67	1.08	0.09
243.00	267.00	251.33	-1.05	0.45
377.00	412.00	406.33	1.15	0.01
38.00	50.00	47.33	1.17	0.27
2610.00	2680.00	2647.67	-1.12	0.01
17.00	14.00	15.33	-1.21	0.45
22.00	17.00	16.00	1.10	0.70
680.00	769.00	736.00	1.03	0.46
1246.00	1407.00	1305.33	1.12	0.01
128.00	118.00	129.33	-1.23	0.01
16.00	17.00	14.00	-1.47	0.09
628.00	622.00	619.33	1.28	0.00
453.00	451.00	466.67	-1.16	0.01
192.00	230.00	218.00	1.06	0.45
8.00	8.00	8.67	-1.59	0.10
705.00	820.00	783.00	-1.08	0.10
2632.00	2570.00	2626.67	-1.13	0.00

99.00	114.00	103.67	1.10	0.31
462.00	478.00	490.00	1.08	0.12
10.00	6.00	6.67	1.16	0.75
712.00	808.00	783.00	1.02	0.63
178.00	219.00	201.00	2.13	0.00
761.00	848.00	828.67	-1.12	0.01
69.00	77.00	74.33	1.17	0.18
752.00	835.00	813.67	-1.74	0.00
10.00	13.00	13.00	-2.23	0.00
2101.00	2440.00	2325.33	-1.11	0.01
119.00	121.00	121.00	1.07	0.43
992.00	1015.00	998.33	-1.35	0.00
32.00	37.00	32.67	-1.95	0.00
70.00	81.00	76.00	-1.43	0.00
1163.00	1280.00	1268.00	1.05	0.26
966.00	1045.00	1044.67	1.23	0.00
731.00	928.00	854.00	1.21	0.00
13518.00	15089.00	14383.67	-1.09	0.02
1267.00	1475.00	1378.67	-1.05	0.25
12696.00	13762.00	13471.67	-1.08	0.05
1286.00	1318.00	1334.33	1.04	0.37
13287.00	14411.00	14197.67	1.03	0.41
119.00	137.00	140.67	-1.12	0.19
688.00	749.00	730.67	1.32	0.00
269.00	259.00	273.67	-1.05	0.44
174.00	188.00	187.33	-1.05	0.47
13.00	10.00	12.33	1.58	0.10
646.00	714.00	752.00	-1.27	0.00
495.00	441.00	489.33	1.15	0.03
35.00	40.00	34.33	-1.75	0.00
563.00	610.00	632.00	-1.05	0.34
517.00	497.00	520.00	1.03	0.58
1878.00	1952.00	2010.67	1.14	0.00
3430.00	3997.00	3795.00	-1.14	0.00
660.00	673.00	653.00	-1.19	0.00
634.00	599.00	602.67	-1.30	0.00
0.00	0.00	0.67	-6.55	0.00
2089.00	1972.00	1983.67	-1.21	0.00
544.00	581.00	591.33	1.18	0.00
5390.00	5836.00	5660.67	1.06	0.13
694.00	725.00	688.67	1.07	0.21
415.00	502.00	465.00	1.38	0.00
429.00	504.00	468.33	1.13	0.03
95.00	86.00	97.33	-1.09	0.38
398.00	354.00	386.00	-1.51	0.00
1673.00	1618.00	1731.00	-1.23	0.00
489.00	499.00	525.33	1.14	0.02
889.00	1029.00	1036.00	1.06	0.27

16.00	12.00	11.67	-2.04	0.00
4.00	0.00	2.67	-1.74	0.30
1938.00	1989.00	1914.00	1.04	0.45
2.00	4.00	3.33	-2.48	0.02
21.00	10.00	18.00	-1.33	0.18
1.00	0.00	0.67	-2.29	0.46
4925.00	5100.00	5258.67	-1.19	0.00
1029.00	1063.00	1047.33	-1.22	0.00
5727.00	5817.00	5905.33	-1.28	0.00
3379.00	3723.00	3739.33	1.11	0.01
4.00	3.00	4.00	-1.09	1.00
168.00	173.00	173.33	1.30	0.00
863.00	901.00	940.33	-1.02	0.74
1300.00	1360.00	1349.67	1.15	0.00
217.00	293.00	261.67	1.11	0.17
31.00	31.00	34.00	1.08	0.63
112.00	93.00	105.33	-1.63	0.00
307.00	352.00	340.33	1.01	0.93
2839.00	2816.00	2875.00	1.03	0.48
757.00	710.00	768.67	1.04	0.37
822.00	852.00	868.67	1.04	0.33
635.00	595.00	634.67	-1.12	0.02
339.00	286.00	310.33	-1.28	0.00
366.00	349.00	376.33	1.08	0.24
2381.00	2482.00	2475.33	1.09	0.03
2838.00	3110.00	3063.00	1.13	0.00
292.00	320.00	327.33	1.19	0.05
114.00	103.00	117.33	-1.02	0.82
803.00	823.00	824.33	-1.16	0.00
678.00	763.00	754.67	1.06	0.21
197.00	239.00	207.67	1.10	0.25
428.00	459.00	474.33	1.09	0.12
29.00	33.00	35.67	1.13	0.47
269.00	284.00	288.33	1.02	0.80
398.00	416.00	414.00	1.13	0.04
485.00	480.00	512.33	1.01	0.88
341.00	385.00	388.00	-1.10	0.09
980.00	1023.00	1028.67	1.00	0.92
707.00	750.00	743.00	1.15	0.01
573.00	565.00	562.67	-1.02	0.75
500.00	496.00	514.00	1.07	0.17
1239.00	1320.00	1322.00	-1.14	0.00
329.00	333.00	344.00	2.56	0.00
2767.00	2804.00	2818.00	-1.14	0.00
223.00	266.00	255.33	1.29	0.00
6.00	6.00	6.67	-1.46	0.26
4650.00	4523.00	4622.00	-1.05	0.20
2428.00	2566.00	2671.67	1.18	0.00

1918.00	2247.00	2285.00	1.18	0.00
577.00	674.00	666.67	1.16	0.00
3545.00	4236.00	4214.33	1.13	0.01
0.00	0.00	0.00	-17.09	0.03
388.00	427.00	410.67	-1.06	0.26
1641.00	1616.00	1688.67	1.10	0.02
89.00	121.00	107.33	-1.49	0.00
2078.00	2010.00	2096.00	-1.04	0.34
3383.00	3546.00	3586.00	-1.01	0.89
9671.00	9793.00	9868.00	-1.15	0.00
21053.00	22231.00	22898.67	1.23	0.00
5689.00	6053.00	6048.67	1.30	0.00
19.00	26.00	22.00	-1.07	0.74
1572.00	1611.00	1715.67	1.20	0.00
13.00	11.00	12.00	1.68	0.07
7.00	5.00	6.67	-1.11	0.88
986.00	958.00	992.00	1.24	0.00
440.00	541.00	477.00	-1.17	0.02
718.00	679.00	746.67	1.15	0.01
190.00	158.00	167.00	-1.38	0.00
262.00	281.00	293.00	-1.03	0.63
280.00	304.00	292.00	1.12	0.11
256.00	223.00	239.00	-1.09	0.20
3380.00	3557.00	3517.33	-1.01	0.86
523.00	524.00	526.67	-1.12	0.04
181.00	201.00	189.00	1.01	0.90
10459.00	11421.00	11364.00	1.20	0.00
1842.00	1797.00	1888.33	1.08	0.05
642.00	681.00	686.67	-1.01	0.75
1701.00	1886.00	1852.00	1.19	0.00
132.00	123.00	132.00	-1.25	0.00
1595.00	1887.00	1788.00	-1.06	0.13
37.00	35.00	31.00	-1.24	0.42
709.00	565.00	609.00	-1.90	0.00
32.00	42.00	39.67	-1.07	0.69
762.00	710.00	770.67	-1.10	0.06
419.00	458.00	460.00	-1.48	0.00
110.00	113.00	103.67	-1.06	0.59
1608.00	1499.00	1543.33	1.35	0.00
1067.00	1066.00	1093.33	-1.01	0.83
1432.00	1420.00	1448.67	-1.14	0.00
300.00	293.00	290.33	1.13	0.08
518.00	567.00	559.67	1.05	0.37
930.00	973.00	926.33	1.06	0.26
667.00	800.00	749.00	1.11	0.03
3186.00	3420.00	3515.33	1.17	0.00
5202.00	5469.00	5605.67	1.15	0.00
546.00	510.00	535.67	-1.36	0.00

3178.00	3259.00	3335.33	-1.04	0.25
1259.00	1322.00	1244.33	-1.11	0.09
65.00	59.00	66.33	1.14	0.28
56.00	66.00	68.67	-1.26	0.04
722.00	763.00	758.67	-1.09	0.06
121.00	127.00	131.67	-1.04	0.64
905.00	959.00	936.00	-1.26	0.00
561.00	589.00	589.67	1.03	0.53
1457.00	1734.00	1639.00	1.18	0.00
1004.00	1037.00	1055.33	1.18	0.00
783.00	805.00	781.67	-1.11	0.03
1426.00	1335.00	1378.33	-1.05	0.32
2022.00	2245.00	2145.33	-1.01	0.75
788.00	778.00	816.33	-1.12	0.02
1410.00	1648.00	1605.67	1.07	0.13
1686.00	1770.00	1702.33	-1.10	0.06
635.00	637.00	688.33	1.27	0.00
942.00	1070.00	1007.33	1.12	0.02
115.00	116.00	119.33	-1.15	0.11
1031.00	1123.00	1076.00	-1.32	0.00
12678.00	13545.00	13638.33	1.10	0.00
1712.00	2106.00	1860.67	1.01	0.91
634.00	672.00	655.00	-1.13	0.01
659.00	683.00	671.00	-1.16	0.00
948.00	1075.00	1037.00	-1.07	0.11
20.00	26.00	19.67	-1.34	0.15
679.00	710.00	726.00	-1.09	0.05
6659.00	7220.00	7103.00	-1.16	0.00
179.00	173.00	190.00	-1.25	0.00
1742.00	1639.00	1746.00	-1.13	0.01
258.00	340.00	286.33	-3.44	0.00
92.00	115.00	112.00	-1.05	0.60
324.00	313.00	295.33	-1.32	0.00
558.00	617.00	593.33	-1.11	0.04
53.00	61.00	53.33	-1.21	0.12
943.00	1013.00	1076.00	-1.02	0.69
28.00	32.00	36.67	1.18	0.33
531.00	531.00	533.00	1.05	0.33
866.00	960.00	909.00	1.24	0.00
506.00	513.00	532.00	1.02	0.66
144.00	119.00	134.67	1.07	0.50
10.00	7.00	10.67	-1.99	0.01
627.00	704.00	675.67	-1.11	0.04
1.00	2.00	1.67	-2.33	0.15
987.00	919.00	975.67	-1.09	0.05
308.00	308.00	309.67	1.39	0.00
1426.00	1343.00	1396.00	-1.23	0.00
59.00	68.00	67.00	-1.26	0.06

106.00	118.00	128.00	-1.22	0.02
1756.00	1814.00	1861.00	-1.04	0.35
9.00	4.00	5.33	-1.76	0.11
288.00	319.00	311.00	-1.10	0.08
560.00	625.00	601.33	1.01	0.89
832.00	913.00	960.00	1.07	0.19
479.00	473.00	476.67	1.01	0.91
479.00	540.00	536.00	1.14	0.01
2706.00	2875.00	2772.00	1.02	0.59
209.00	226.00	226.00	-1.06	0.53
26.00	27.00	29.67	1.19	0.33
472.00	487.00	508.67	-1.21	0.00
7058.00	6333.00	6734.00	-1.21	0.00
773.00	821.00	852.33	1.07	0.20
170.00	262.00	221.67	1.07	0.48
502.00	522.00	528.33	-1.04	0.52
187.00	202.00	213.00	1.14	0.09
670.00	677.00	727.67	1.06	0.31
570.00	614.00	630.67	1.06	0.31
935.00	950.00	964.67	-1.23	0.00
424.00	418.00	444.33	1.16	0.01
2507.00	2850.00	2669.00	1.01	0.82
215.00	188.00	221.67	-1.12	0.15
549.00	623.00	606.00	-1.12	0.02
807.00	833.00	779.67	-1.07	0.28
1786.00	1677.00	1740.00	-1.16	0.00
227.00	229.00	252.00	1.14	0.07
977.00	914.00	993.67	-1.02	0.64
999.00	981.00	1019.00	-1.04	0.41
4458.00	4143.00	4329.67	-1.07	0.09
1159.00	1239.00	1249.00	-1.10	0.03
719.00	716.00	758.67	-1.02	0.65
255.00	277.00	273.67	1.01	0.90
160.00	179.00	181.33	-1.01	0.92
755.00	760.00	811.00	1.03	0.62
49.00	44.00	44.67	1.00	1.00
1097.00	1269.00	1157.33	-1.19	0.00
15.00	27.00	19.33	1.27	0.34
590.00	614.00	583.00	1.02	0.68
551.00	526.00	538.33	-1.06	0.39
677.00	722.00	692.67	1.03	0.60
1251.00	1172.00	1231.33	-1.25	0.00
30.00	34.00	33.67	1.87	0.00
169.00	161.00	154.67	1.17	0.10
25.00	15.00	18.33	1.23	0.34
593.00	566.00	553.00	1.03	0.70
1401.00	1297.00	1371.33	-1.16	0.00
62.00	57.00	57.67	-1.01	0.96

1344.00	1409.00	1421.00	-1.13	0.00
1701.00	1987.00	1794.67	-1.07	0.18
7.00	6.00	7.00	-1.35	0.40
22.00	31.00	29.33	1.26	0.23
966.00	1056.00	1037.33	1.18	0.00
147.00	195.00	169.67	1.25	0.01
1375.00	1367.00	1411.33	1.23	0.00
792.00	843.00	856.00	1.04	0.34
389.00	458.00	450.00	1.20	0.00
18.00	9.00	11.67	-1.41	0.18
956.00	963.00	986.00	-1.31	0.00
285.00	311.00	299.00	-1.12	0.06
34.00	29.00	32.67	-2.24	0.00
242.00	252.00	257.67	1.05	0.51
1365.00	1285.00	1378.00	-1.26	0.00
723.00	717.00	758.00	-1.09	0.08
2391.00	2586.00	2556.67	-1.04	0.32
1045.00	1042.00	1048.67	-1.15	0.00
35.00	30.00	31.67	-1.18	0.30
31.00	33.00	29.00	-1.14	0.51
53.00	67.00	56.67	1.48	0.00
1085.00	1120.00	1150.33	-1.16	0.00
696.00	849.00	826.33	1.34	0.00
1526.00	1601.00	1583.67	-1.03	0.46
184.00	162.00	185.33	1.04	0.67
1974.00	2268.00	2159.00	1.15	0.00
290.00	351.00	330.00	1.04	0.53
852.00	882.00	880.33	-1.28	0.00
857.00	847.00	874.67	-1.32	0.00
32.00	30.00	31.67	-1.07	0.73
3588.00	3675.00	3706.67	-1.20	0.00
10.00	12.00	11.67	-1.07	0.91
211.00	180.00	214.33	1.18	0.05
44.00	46.00	46.67	1.04	0.82
4.00	2.00	2.33	1.67	0.56
38.00	51.00	44.67	-1.12	0.42
766.00	863.00	854.00	-1.02	0.65
1673.00	1776.00	1679.67	-1.07	0.21
3773.00	3429.00	3629.00	-1.43	0.00
10.00	3.00	7.00	1.29	0.52
56.00	46.00	50.00	-1.01	0.96
126.00	143.00	128.33	-1.20	0.03
245.00	249.00	253.33	-1.22	0.01
1271.00	1418.00	1395.33	-1.05	0.24
4.00	4.00	4.33	1.07	1.00
51.00	50.00	55.67	1.20	0.17
261.00	295.00	291.00	-1.06	0.30
1143.00	1218.00	1201.33	1.43	0.00

25.00	26.00	26.33	1.03	0.94
197.00	207.00	221.00	1.24	0.01
24481.00	25363.00	25594.67	-1.02	0.50
545.00	594.00	579.67	1.28	0.00
3884.00	3708.00	3932.00	-1.05	0.19
1868.00	2046.00	1920.67	1.03	0.61
10.00	9.00	11.00	1.79	0.05
681.00	803.00	790.00	1.06	0.27
544.00	498.00	543.67	-1.20	0.00
70.00	82.00	72.00	1.56	0.00
1924.00	2069.00	2028.33	1.02	0.54
241.00	313.00	287.00	-1.05	0.44
0.00	0.00	0.00	-11.75	0.13
545.00	521.00	555.67	-1.21	0.00
132.00	147.00	137.33	-1.13	0.12
305.00	342.00	317.33	-1.19	0.01
23.00	6.00	17.33	-1.14	0.67
43.00	43.00	46.67	1.04	0.82
246.00	263.00	264.67	-1.28	0.00
4.00	7.00	7.00	-1.20	0.67
5.00	6.00	4.67	-1.08	1.00
1172.00	1196.00	1242.67	1.18	0.00
64.00	54.00	66.67	1.03	0.82
9.00	5.00	8.00	1.80	0.11
43.00	49.00	54.67	1.04	0.80
552.00	702.00	628.33	-1.17	0.01
891.00	1036.00	979.00	-1.25	0.00
829.00	855.00	843.67	-1.03	0.60
41990.00	46924.00	45649.67	1.09	0.03
14768.00	15160.00	15573.33	-1.46	0.00
11599.00	11641.00	12009.67	-1.04	0.38
7.00	12.00	8.33	1.23	0.56
1690.00	1068.00	1348.00	-1.55	0.00
639.00	690.00	643.33	-1.13	0.06
125.00	105.00	117.67	1.26	0.05
2284.00	2197.00	2299.67	-1.31	0.00
159.00	188.00	175.67	-1.14	0.08
34.00	52.00	41.33	-1.08	0.67
214.00	228.00	232.33	-1.18	0.01
28.00	36.00	36.67	1.93	0.00
521.00	554.00	540.00	-1.40	0.00
3721.00	3658.00	3811.00	-1.19	0.00
13.00	8.00	10.00	-1.38	0.26
13.00	4.00	7.00	1.71	0.20
70.00	65.00	68.67	-1.03	0.79
197.00	229.00	203.67	1.02	0.81
37.00	28.00	32.00	-2.24	0.00
40.00	49.00	45.67	-1.12	0.38

103.00	98.00	111.00	1.05	0.63
1002.00	1042.00	1065.00	-1.19	0.00
3435.00	3481.00	3565.33	-1.22	0.00
541.00	509.00	528.67	1.23	0.00
18738.00	17575.00	17990.67	-1.76	0.00
37.00	39.00	36.67	-1.11	0.46
403.00	531.00	468.67	-1.04	0.50
1375.00	1447.00	1446.67	1.13	0.01
316.00	419.00	362.33	1.14	0.08
194.00	179.00	202.67	1.02	0.81
1044.00	1085.00	1041.67	-1.22	0.00
21.00	16.00	20.00	-2.96	0.00
12.00	7.00	9.00	1.47	0.24
607.00	729.00	649.67	-1.10	0.10
85.00	85.00	93.00	-1.16	0.10
2857.00	3011.00	3010.00	1.07	0.05
121.00	121.00	133.00	-1.06	0.49
293.00	300.00	303.00	-1.11	0.06
942.00	843.00	912.33	-1.13	0.01
609.00	580.00	596.00	1.02	0.73
1786.00	1691.00	1810.33	-1.65	0.00
51.00	35.00	40.00	-1.13	0.45
1546.00	1451.00	1521.67	-1.05	0.23
413.00	420.00	435.33	-1.09	0.12
113.00	116.00	121.00	-1.25	0.01
430.00	527.00	497.33	1.04	0.54
965.00	898.00	985.33	1.02	0.72
144.00	121.00	137.33	-1.09	0.29
544.00	515.00	500.33	-1.49	0.00
2030.00	2019.00	2066.67	-1.18	0.00
357.00	328.00	338.00	-1.11	0.10
223.00	197.00	206.33	1.09	0.25
468.00	475.00	504.33	-1.08	0.16
687.00	718.00	767.67	-1.02	0.77
680.00	716.00	716.00	1.13	0.01
10.00	6.00	7.33	-1.06	1.00
560.00	591.00	582.33	1.02	0.68
1665.00	1666.00	1729.00	1.14	0.00
1347.00	1360.00	1418.00	1.18	0.00
2280.00	2508.00	2402.00	1.31	0.00
440.00	488.00	477.00	1.04	0.41
395.00	500.00	452.33	1.06	0.30
0.00	1.00	0.67	-3.57	0.11
5513.00	5767.00	5694.33	-1.28	0.00
17968.00	20137.00	18896.00	1.00	0.97
5463.00	5743.00	5803.33	-1.10	0.01
1809.00	1797.00	1865.33	1.05	0.31
1724.00	1950.00	1902.67	1.08	0.05

189.00	198.00	197.67	1.17	0.05
7991.00	8771.00	8543.67	1.07	0.05
75.00	89.00	86.00	1.10	0.42
663.00	724.00	724.33	1.08	0.12
5.00	14.00	13.00	-1.07	0.84
80.00	105.00	99.33	-1.43	0.00
3426.00	3453.00	3570.67	1.01	0.86
2.00	1.00	2.00	-2.12	0.17
4177.00	3983.00	4045.00	-1.42	0.00
5501.00	5013.00	5268.67	1.29	0.00
651.00	583.00	642.67	-1.25	0.00
2371.00	2093.00	2156.33	-1.59	0.00
110.00	90.00	103.00	-1.47	0.00
1303.00	1311.00	1302.67	-1.62	0.00
731.00	641.00	714.67	-1.46	0.00
142.00	125.00	132.67	-1.06	0.56
2193.00	2088.00	2158.67	-1.61	0.00
428.00	539.00	470.00	1.25	0.00
1564.00	1932.00	1808.00	1.34	0.00
5.00	3.00	5.00	-1.34	0.51
156.00	173.00	172.67	-1.46	0.00
195.00	220.00	226.67	1.11	0.19
8.00	8.00	7.67	-1.19	0.68
17.00	15.00	16.67	-1.44	0.08
711.00	734.00	739.00	-1.07	0.11
413.00	460.00	471.33	1.11	0.11
891.00	906.00	908.67	-1.03	0.52
440.00	444.00	449.33	-1.06	0.25
1878.00	2048.00	2034.67	-1.01	0.74
1169.00	1358.00	1328.00	-1.02	0.70
1183.00	1531.00	1393.33	1.09	0.08
563.00	571.00	555.67	1.03	0.63
152.00	140.00	152.33	1.02	0.87
304.00	355.00	344.00	1.34	0.00
2.00	6.00	4.33	1.79	0.27
165.00	172.00	163.33	-1.13	0.13
17.00	40.00	32.00	1.49	0.04
63.00	56.00	61.00	1.07	0.55
13.00	19.00	14.33	1.21	0.45
13.00	10.00	11.00	1.05	0.90
616.00	625.00	656.67	1.26	0.00
2.00	3.00	2.67	1.54	0.59
5053.00	6003.00	5651.33	-1.16	0.00
434.00	419.00	453.33	-1.10	0.08
560.00	506.00	534.67	-1.03	0.68
1.00	1.00	1.33	-2.87	0.08
1144.00	1163.00	1206.33	-1.03	0.53
181.00	173.00	170.67	-1.48	0.00

10.00	10.00	9.67	-1.01	1.00
688.00	637.00	678.67	1.23	0.00
8860.00	10764.00	9988.00	1.03	0.48
831.00	898.00	925.67	1.01	0.81
2603.00	3035.00	2971.33	1.40	0.00
1088.00	1117.00	1144.67	-1.29	0.00
199.00	201.00	194.33	-1.55	0.00
340.00	346.00	375.67	1.00	0.99
2811.00	2589.00	2725.00	-1.17	0.00
27.00	29.00	31.33	-1.03	0.89
408.00	512.00	462.00	-1.12	0.05
715.00	666.00	719.00	1.02	0.71
1472.00	1757.00	1661.00	1.09	0.03
191.00	244.00	220.33	-1.27	0.00
1431.00	1818.00	1694.00	1.14	0.01
482.00	486.00	508.67	1.18	0.00
510.00	643.00	591.67	-1.07	0.24
361.00	443.00	419.33	1.12	0.05
8.00	0.00	4.33	-1.47	0.40
3916.00	3743.00	3955.00	-1.13	0.00
2253.00	2310.00	2343.00	1.12	0.01
2984.00	3061.00	2952.00	-1.29	0.00
1559.00	1661.00	1650.33	-1.25	0.00
2772.00	2903.00	2920.33	1.06	0.08
962.00	1016.00	997.00	-1.29	0.00
3079.00	3415.00	3359.33	-1.08	0.02
519.00	632.00	579.00	1.13	0.02
1041.00	1172.00	1144.67	1.21	0.00
454.00	469.00	471.33	1.11	0.04
1438.00	1465.00	1459.67	1.13	0.00
3025.00	3098.00	3236.00	1.17	0.00
479.00	565.00	543.00	1.23	0.00
664.00	715.00	735.33	1.04	0.47
2198.00	2231.00	2353.00	1.08	0.05
1269.00	1224.00	1239.33	-1.10	0.03
1849.00	1914.00	1877.33	1.04	0.40
196.00	150.00	181.67	1.43	0.00
1219.00	1263.00	1310.67	1.26	0.00
612.00	590.00	651.00	1.27	0.00
1677.00	1730.00	1783.67	-1.16	0.00
905.00	1018.00	983.67	-1.11	0.01
265.00	323.00	295.00	-1.03	0.68
808.00	896.00	887.67	-1.07	0.13
829.00	951.00	929.67	-1.07	0.13
915.00	972.00	998.00	-1.06	0.24
1609.00	1769.00	1786.67	1.02	0.67
91.00	113.00	102.33	-1.32	0.00
542.00	603.00	578.33	1.22	0.00

2114.00	1952.00	1963.67	-1.07	0.21
1968.00	2141.00	2101.00	1.05	0.21
1096.00	1268.00	1254.00	1.03	0.53
1811.00	1921.00	1992.00	1.13	0.00
808.00	813.00	857.33	1.21	0.00
5785.00	5069.00	5290.00	-1.23	0.00
3175.00	3731.00	3556.67	1.05	0.24
1490.00	1523.00	1639.33	1.20	0.00
781.00	758.00	784.00	1.23	0.00
1967.00	1982.00	2077.00	1.09	0.03
488.00	559.00	539.33	1.13	0.02
1091.00	1091.00	1154.00	1.19	0.00
1381.00	1491.00	1549.00	1.05	0.31
296.00	333.00	340.33	1.23	0.00
222.00	219.00	234.67	1.20	0.01
30.00	28.00	27.00	-1.26	0.18
217.00	270.00	251.33	-1.10	0.14
848.00	923.00	955.00	1.21	0.00
862.00	980.00	1015.67	1.11	0.07
65.00	44.00	52.33	1.18	0.25
2609.00	2641.00	2597.67	-1.03	0.45
650.00	654.00	649.33	1.04	0.46
1051.00	1061.00	1088.67	1.13	0.01
880.00	863.00	869.00	-1.01	0.92
1455.00	1656.00	1583.00	1.03	0.50
515.00	605.00	562.00	1.14	0.02
810.00	1085.00	957.67	1.09	0.20
294.00	259.00	305.67	1.05	0.52
1081.00	1107.00	1128.00	1.02	0.68
312.00	353.00	363.00	1.29	0.00
2246.00	2171.00	2258.00	-1.14	0.00
978.00	779.00	863.67	-1.29	0.00
1273.00	1092.00	1209.00	-1.21	0.00
1.00	1.00	1.33	-3.79	0.01
57.00	50.00	58.33	-1.86	0.00
2506.00	2483.00	2535.67	-1.11	0.00
495.00	520.00	508.33	-1.01	0.88
41.00	37.00	32.33	1.09	0.68
1212.00	1333.00	1345.67	1.22	0.00
1181.00	1273.00	1253.67	-1.14	0.00
1124.00	1125.00	1156.00	-1.44	0.00
2260.00	2148.00	2294.00	1.04	0.40
668.00	747.00	730.00	1.04	0.44
442.00	499.00	501.67	1.12	0.04
400.00	475.00	480.00	1.10	0.21
652.00	621.00	646.67	1.23	0.00
15.00	12.00	16.33	-1.43	0.12
673.00	705.00	741.67	1.28	0.00

2130.00	2165.00	2219.67	-1.09	0.06
363.00	332.00	367.00	-1.35	0.00
839.00	862.00	909.00	1.03	0.56
8.00	9.00	12.33	1.51	0.19
700.00	856.00	781.33	-1.18	0.00
1995.00	1977.00	2071.67	1.02	0.58
39.00	39.00	39.33	1.35	0.05
1082.00	1208.00	1197.00	1.08	0.05
99.00	110.00	111.33	-1.39	0.00
133.00	153.00	155.33	-1.07	0.37
2849.00	3475.00	3320.00	1.17	0.00
754.00	861.00	815.00	1.20	0.00
186.00	193.00	193.33	-1.25	0.00
297.00	340.00	332.67	1.01	0.91
1646.00	1631.00	1702.67	-1.32	0.00
1653.00	1550.00	1694.00	1.06	0.29
29.00	32.00	32.33	-1.35	0.05
1044.00	1060.00	1078.00	-1.01	0.83
565.00	590.00	568.00	-1.05	0.34
68.00	50.00	59.67	-1.07	0.61
321.00	283.00	299.33	-1.52	0.00
2773.00	2817.00	3007.67	-1.05	0.22
376.00	407.00	405.67	-1.31	0.00
10.00	15.00	14.00	1.04	0.92
415.00	389.00	383.67	1.02	0.73
697.00	771.00	740.00	1.17	0.00
269.00	281.00	291.00	1.34	0.00
693.00	824.00	813.00	1.18	0.00
1343.00	1192.00	1367.00	1.14	0.02
7218.00	7539.00	7443.67	-1.13	0.00
141.00	189.00	165.67	-1.08	0.44
202.00	211.00	214.67	-1.23	0.00
12.00	14.00	11.33	1.24	0.45
3109.00	3360.00	3164.33	-1.34	0.00
30.00	28.00	30.67	1.21	0.30
1142.00	1270.00	1220.33	-1.03	0.42
9805.00	11042.00	11112.67	1.21	0.00
42.00	37.00	38.00	1.46	0.02
0.00	0.00	0.00	-11.74	0.13
10831.00	11240.00	11468.33	-1.13	0.00
782.00	745.00	770.33	1.13	0.03
928.00	1056.00	1045.00	1.24	0.00
1048.00	1147.00	1170.33	1.13	0.01
109.00	119.00	122.67	-1.42	0.00
2620.00	2626.00	2713.33	1.04	0.24
1084.00	1254.00	1181.67	1.11	0.01
754.00	971.00	896.33	1.29	0.00
1013.00	1225.00	1192.67	1.24	0.00

774.00	791.00	829.67	-1.03	0.60
501.00	555.00	566.67	1.14	0.02
755.00	899.00	870.00	1.11	0.03
55.00	50.00	54.33	1.12	0.39
562.00	607.00	595.00	-1.07	0.16
909.00	1019.00	940.00	1.18	0.01
1117.00	1222.00	1196.33	1.08	0.05
634.00	699.00	682.33	1.04	0.35
265.00	305.00	298.33	1.20	0.00
462.00	506.00	472.67	1.14	0.03
2037.00	2016.00	2046.00	-1.10	0.02
3600.00	3825.00	3826.33	1.17	0.00
134.00	153.00	144.33	-1.05	0.58
11.00	31.00	24.33	-1.03	0.89
455.00	503.00	483.67	1.10	0.14
65.00	45.00	48.67	-1.71	0.00
578.00	624.00	612.67	-1.03	0.50
25.00	17.00	21.67	-1.15	0.50
1031.00	943.00	1001.00	-1.06	0.23
786.00	898.00	890.00	-1.07	0.17
2124.00	2100.00	2251.67	1.20	0.00
1985.00	2315.00	2167.33	1.19	0.00
4528.00	4985.00	4888.67	1.20	0.00
101.00	76.00	86.67	1.00	1.00
1449.00	1574.00	1482.00	1.02	0.67
399.00	398.00	386.67	-1.30	0.00
1919.00	1972.00	2042.33	-1.10	0.02
283.00	311.00	304.33	-1.15	0.01
302.00	280.00	292.33	-1.11	0.12
4701.00	5237.00	5175.67	1.15	0.00
9402.00	9538.00	9818.67	-1.18	0.00
2624.00	2593.00	2637.67	1.02	0.70
1415.00	1488.00	1525.67	1.01	0.87
0.00	0.00	1.33	-2.17	0.28
4.00	1.00	3.00	-1.34	0.67
685.00	825.00	762.67	1.10	0.06
358.00	417.00	424.33	-1.07	0.31
1143.00	1117.00	1165.33	-1.23	0.00
74.00	44.00	57.33	-1.07	0.67
18.00	22.00	21.33	1.11	0.66
19.00	11.00	18.33	-1.18	0.40
950.00	974.00	1026.00	1.19	0.00
14.00	13.00	11.33	2.74	0.00
700.00	685.00	707.67	-1.03	0.55
830.00	749.00	814.67	1.44	0.00
128.00	142.00	139.00	-1.04	0.65
597.00	600.00	619.00	-1.21	0.00
255.00	247.00	269.33	1.04	0.59

740.00	783.00	800.00	1.15	0.00
1012.00	1068.00	1073.33	1.11	0.02
319.00	401.00	365.33	1.04	0.56
3299.00	3150.00	3257.67	1.06	0.18
3175.00	3715.00	3574.00	1.08	0.06
1008.00	1066.00	1054.33	1.15	0.00
3259.00	3427.00	3495.67	1.08	0.03
492.00	489.00	501.33	-1.12	0.03
1636.00	1833.00	1800.67	1.18	0.00
8.00	15.00	11.00	-1.26	0.43
1909.00	2007.00	2035.67	-1.14	0.00
321.00	326.00	313.67	-1.10	0.22
125.00	138.00	134.33	-1.05	0.53
621.00	685.00	679.67	1.20	0.00
2034.00	2141.00	2169.33	-1.01	0.72
542.00	648.00	621.00	1.03	0.60
700.00	789.00	766.00	1.00	1.00
1761.00	1938.00	1907.67	1.26	0.00
1073.00	1120.00	1142.33	1.05	0.28
877.00	985.00	965.00	1.12	0.01
566.00	656.00	610.67	-1.19	0.00
1597.00	1805.00	1731.33	1.20	0.00
234.00	283.00	275.33	-1.21	0.00
90.00	125.00	113.67	-1.03	0.77
1368.00	1530.00	1458.67	1.28	0.00
1683.00	1765.00	1805.00	1.13	0.00
2193.00	2398.00	2343.33	1.02	0.51
254.00	201.00	236.67	-1.45	0.00
728.00	814.00	755.67	1.21	0.00
1727.00	1764.00	1788.67	1.03	0.47
1658.00	1822.00	1757.67	1.05	0.22
1148.00	1242.00	1252.67	1.13	0.00
836.00	930.00	935.00	1.15	0.00
2791.00	2789.00	2868.33	-1.02	0.50
1372.00	1374.00	1438.00	-1.02	0.59
2309.00	2483.00	2362.33	1.08	0.07
3472.00	3512.00	3488.33	-1.16	0.00
4655.00	4726.00	4817.00	1.19	0.00
597.00	581.00	583.00	1.09	0.13
1012.00	1203.00	1176.00	1.06	0.21
978.00	1039.00	1063.33	1.06	0.21
547.00	563.00	583.67	1.04	0.45
650.00	637.00	674.33	-1.03	0.49
173.00	176.00	173.33	1.18	0.04
146.00	185.00	174.67	1.33	0.00
756.00	631.00	759.33	1.02	0.80
1.00	4.00	2.00	-2.28	0.12
2112.00	2217.00	2203.33	1.23	0.00

21.00	26.00	21.33	-1.04	0.93
350.00	421.00	421.00	1.01	0.93
1326.00	1356.00	1436.67	1.10	0.04
172.00	135.00	149.00	-1.61	0.00
1413.00	1412.00	1466.33	-1.28	0.00
84.00	80.00	90.33	2.32	0.00
272.00	324.00	303.33	1.06	0.35
14.00	20.00	16.67	-1.34	0.16
8.00	10.00	8.67	-1.36	0.32
63.00	61.00	66.00	1.29	0.03
507.00	613.00	578.00	1.02	0.78
715.00	686.00	685.67	-1.19	0.00
1604.00	1625.00	1634.67	-1.09	0.02
154.00	157.00	159.33	-1.19	0.02
10.00	13.00	13.33	-1.29	0.33
519.00	591.00	570.67	-1.09	0.08
6052.00	6776.00	6823.67	1.24	0.00
1639.00	1801.00	1835.67	1.22	0.00
2839.00	3392.00	3235.67	1.17	0.00
4464.00	4506.00	4661.67	1.09	0.03
5179.00	5540.00	5635.67	1.20	0.00
4314.00	4959.00	4977.33	1.16	0.00
793.00	835.00	867.00	-1.04	0.36
2556.00	2696.00	2745.33	1.30	0.00
133.00	110.00	127.67	-1.01	0.90
851.00	846.00	868.33	1.03	0.50
2664.00	2812.00	2707.00	1.00	0.95
3159.00	3020.00	3103.33	-1.02	0.69
6.00	4.00	3.67	-1.54	0.36
100.00	92.00	93.00	1.08	0.56
126.00	123.00	131.00	-1.32	0.00
253.00	243.00	250.33	-1.40	0.00
140.00	147.00	141.33	-1.47	0.00
285.00	363.00	330.00	1.34	0.00
641.00	620.00	652.00	1.34	0.00
391.00	479.00	455.33	1.30	0.00
2253.00	2822.00	2487.67	-1.02	0.72
548.00	597.00	558.33	-1.06	0.29
499.00	514.00	513.67	-1.04	0.43
1233.00	1242.00	1247.67	-1.11	0.01
691.00	694.00	711.00	-1.05	0.27
1129.00	1270.00	1188.33	1.04	0.38
1479.00	1611.00	1609.33	-1.00	0.93
2437.00	2833.00	2691.67	1.06	0.12
2728.00	3093.00	2887.00	-1.24	0.00
394.00	443.00	416.00	-1.02	0.72
458.00	408.00	381.00	1.02	0.88
32.00	22.00	25.00	-1.32	0.11

150.00	216.00	187.67	1.51	0.00
6537.00	6333.00	6323.00	1.10	0.03
810.00	827.00	839.67	1.08	0.07
263.00	282.00	296.33	1.75	0.00
768.00	759.00	762.33	1.16	0.00
1.00	2.00	1.00	-4.61	0.01
217.00	199.00	208.67	-1.32	0.00
1544.00	1822.00	1682.00	1.14	0.00
91.00	114.00	100.67	1.27	0.01
11214.00	12078.00	11909.67	-1.03	0.43
201.00	200.00	205.33	-1.12	0.09
319.00	345.00	347.00	-1.38	0.00
1915.00	1900.00	1936.33	1.10	0.02
14369.00	15469.00	14655.33	-1.02	0.59
135.00	127.00	124.67	-1.29	0.01
45.00	40.00	39.33	1.03	0.86
33.00	28.00	32.00	1.02	0.94
372.00	405.00	432.33	1.01	0.91
27.00	44.00	38.67	-1.28	0.08
1333.00	1538.00	1443.00	1.04	0.37
153.00	199.00	176.00	-1.16	0.05
331.00	354.00	355.00	-1.30	0.00
103.00	113.00	101.00	1.34	0.01
3405.00	3624.00	3747.67	1.17	0.00
1451.00	1550.00	1525.33	-1.12	0.00
947.00	1097.00	1036.67	1.17	0.00
2651.00	2707.00	2710.33	1.05	0.24
623.00	693.00	697.67	1.01	0.81
849.00	849.00	873.00	1.04	0.33
243.00	276.00	265.00	-1.04	0.56
15207.00	15565.00	15796.00	1.32	0.00
51.00	56.00	50.67	-1.96	0.00
84.00	90.00	91.00	1.10	0.37
2106.00	2138.00	2229.00	1.13	0.00
1.00	3.00	2.33	-1.15	1.00
24.00	36.00	33.67	-1.26	0.11
48.00	51.00	54.33	1.06	0.74
559.00	595.00	596.00	-1.06	0.17
851.00	900.00	951.67	1.10	0.07
1380.00	1396.00	1415.00	-1.02	0.71
84.00	72.00	72.33	1.03	0.80
353.00	314.00	337.00	-1.11	0.08
6.00	3.00	4.67	-1.93	0.06
339.00	320.00	340.33	1.10	0.12
3722.00	3795.00	3856.67	1.11	0.00
1377.00	1642.00	1563.67	1.01	0.86
19.00	11.00	15.00	-1.84	0.00
1166.00	1266.00	1254.67	1.03	0.42

3019.00	3562.00	3403.00	1.04	0.30
1493.00	1894.00	1763.67	-1.03	0.52
748.00	841.00	819.00	1.02	0.71
3596.00	4040.00	3795.00	1.11	0.01
412.00	416.00	444.00	-1.14	0.02
3452.00	4007.00	3664.00	-1.07	0.15
3115.00	3451.00	3301.67	1.05	0.15
25.00	11.00	16.00	-1.07	0.86
915.00	976.00	983.33	1.16	0.00
396.00	451.00	436.67	1.13	0.02
647.00	721.00	706.67	1.13	0.01
589.00	671.00	663.67	1.02	0.70
2724.00	2738.00	2673.00	1.18	0.00
943.00	850.00	889.33	-1.07	0.21
1094.00	1207.00	1189.67	1.29	0.00
815.00	806.00	840.00	1.04	0.39
1358.00	1267.00	1367.33	-1.17	0.00
168.00	248.00	193.00	1.03	0.80
28.00	24.00	23.33	1.41	0.09
226.00	221.00	229.67	1.19	0.01
8.00	8.00	9.00	1.39	0.31
8.00	9.00	8.67	2.10	0.04
74.00	56.00	71.00	1.15	0.25
37.00	53.00	49.00	1.35	0.03
284.00	287.00	296.00	1.17	0.01
118.00	112.00	117.33	-1.03	0.80
1317.00	1494.00	1419.33	1.05	0.27
15.00	20.00	18.00	-1.07	0.85
9616.00	8645.00	9096.00	-1.11	0.02
8286.00	7508.00	7758.00	-1.27	0.00
238.00	224.00	231.33	-1.00	1.00
5.00	13.00	9.00	1.06	0.89
6.00	1.00	6.00	1.11	0.88
16.00	5.00	11.33	-1.28	0.39
56.00	49.00	52.00	-1.38	0.01
58.00	55.00	59.00	-1.04	0.81
1203.00	1333.00	1275.00	-1.10	0.04
1123.00	1196.00	1193.33	-1.05	0.24
4333.00	4178.00	4306.00	-1.41	0.00
10.00	22.00	17.33	-1.19	0.42
621.00	681.00	720.33	-1.05	0.43
0.00	2.00	2.67	-1.62	0.39
6.00	9.00	6.00	1.04	1.00
373.00	322.00	389.00	-1.30	0.00
2.00	3.00	2.33	-1.43	0.63
706.00	737.00	709.00	-1.05	0.34
202.00	229.00	216.33	-1.08	0.23
11.00	5.00	8.67	-1.17	0.70

131.00	136.00	136.33	1.07	0.39
26432.00	27764.00	28004.67	-1.27	0.00
7932.00	8689.00	8593.00	1.19	0.00
36.00	32.00	31.67	1.29	0.15
8.00	10.00	12.00	-1.60	0.04
521.00	510.00	495.33	-1.61	0.00
704.00	755.00	737.67	-1.18	0.00
1400.00	1568.00	1520.33	-1.03	0.40
1234.00	1197.00	1234.67	1.11	0.01
827.00	823.00	894.33	1.10	0.12
33.00	42.00	37.00	-1.08	0.64
13.00	18.00	20.00	3.42	0.00
11.00	9.00	9.00	1.76	0.09
1958.00	2121.00	2139.67	-1.16	0.00
89.00	105.00	103.33	-1.00	1.00
24.00	22.00	24.33	-1.04	0.88
16.00	18.00	22.00	2.30	0.00
44.00	38.00	42.33	1.46	0.01
2500.00	3053.00	2791.00	1.33	0.00
542.00	582.00	594.67	1.11	0.04
3283.00	4094.00	3918.33	1.21	0.00
2139.00	2377.00	2433.00	1.23	0.00
1190.00	1491.00	1446.67	1.12	0.04
1252.00	1495.00	1465.00	-1.01	0.88
2107.00	2525.00	2438.00	1.19	0.00
2036.00	2228.00	2279.67	1.14	0.00
1840.00	2091.00	2035.00	1.12	0.00
4941.00	5115.00	5287.00	1.15	0.00
2224.00	2372.00	2466.33	1.20	0.00
324.00	278.00	307.33	1.13	0.07
2057.00	2266.00	2193.67	1.26	0.00
3946.00	4156.00	4393.00	1.19	0.00
2401.00	2597.00	2672.33	1.15	0.00
2446.00	2684.00	2689.33	1.27	0.00
2005.00	2191.00	2238.67	1.15	0.00
1206.00	1354.00	1347.33	1.17	0.00
1642.00	1885.00	1880.67	1.19	0.00
2063.00	2477.00	2340.00	1.19	0.00
373.00	488.00	466.33	1.45	0.00
1793.00	1899.00	1946.33	1.16	0.00
3513.00	3778.00	3916.33	1.19	0.00
1433.00	1716.00	1610.00	1.11	0.01
2727.00	2904.00	2759.33	1.12	0.01
1125.00	1255.00	1264.00	1.09	0.10
2090.00	2124.00	2160.33	1.14	0.00
1445.00	1754.00	1634.33	1.11	0.02
2056.00	2457.00	2378.67	1.21	0.00
2823.00	3179.00	3153.00	1.24	0.00

11454.00	12407.00	12158.33	-1.12	0.00
2745.00	2735.00	2903.67	1.14	0.00
2802.00	3095.00	3103.67	1.23	0.00
777.00	863.00	859.33	1.12	0.01
612.00	675.00	674.33	-1.01	0.89
1510.00	1697.00	1669.67	1.20	0.00
92.00	81.00	87.67	-1.42	0.00
2147.00	2395.00	2326.67	1.26	0.00
1864.00	2020.00	2084.33	1.13	0.00
1002.00	1030.00	1080.33	1.23	0.00
963.00	1118.00	1105.33	1.03	0.50
1027.00	1132.00	1161.33	1.12	0.02
2368.00	2538.00	2492.67	1.04	0.32
2360.00	2515.00	2430.67	-1.05	0.26
1913.00	1882.00	1994.00	1.01	0.76
833.00	936.00	916.33	1.20	0.00
1099.00	1176.00	1214.00	1.09	0.06
1503.00	1579.00	1671.00	1.08	0.11
124.00	115.00	123.00	-1.30	0.00
354.00	379.00	383.67	1.18	0.00
694.00	793.00	741.33	1.13	0.02
1146.00	1251.00	1285.33	1.20	0.00
11.00	10.00	11.33	-1.52	0.09
661.00	744.00	743.67	1.12	0.02
149.00	131.00	139.33	1.05	0.60
172.00	163.00	173.00	1.26	0.00
2.00	8.00	4.00	-1.58	0.33
1307.00	1538.00	1359.67	-1.12	0.06
7927.00	7943.00	8115.00	1.09	0.01
728.00	985.00	866.67	1.13	0.05
1175.00	1423.00	1279.33	1.04	0.50
25.00	19.00	17.67	1.01	1.00
310.00	278.00	299.67	-1.16	0.02
2249.00	2556.00	2483.33	-1.01	0.82
137.00	130.00	131.67	-1.17	0.06
17.00	14.00	13.00	-1.27	0.41
3786.00	3721.00	3786.00	-1.01	0.81
195.00	173.00	181.00	-1.05	0.57
1290.00	1426.00	1388.67	1.15	0.00
13.00	10.00	11.33	1.59	0.11
5.00	7.00	6.00	1.36	0.48
665.00	806.00	722.33	1.23	0.00
27.00	20.00	23.33	1.06	0.74
54.00	70.00	63.33	-1.79	0.00
4.00	4.00	4.33	-1.85	0.11
211.00	212.00	219.67	1.34	0.00
1397.00	1499.00	1480.00	1.03	0.39
8.00	8.00	8.33	-1.50	0.19

6914.00	7971.00	7726.33	1.27	0.00
1100.00	1223.00	1118.33	-1.10	0.07
2172.00	2072.00	2146.67	-1.14	0.00
14.00	15.00	14.67	-1.10	0.76
908.00	1073.00	1010.33	1.10	0.05
411.00	513.00	475.00	-1.04	0.47
244.00	252.00	235.00	-3.25	0.00
419.00	522.00	472.67	1.03	0.66
0.00	0.00	0.00	-11.74	0.13
42.00	45.00	44.00	-1.40	0.01
3041.00	3084.00	3175.33	-1.15	0.00
86.00	74.00	76.33	1.06	0.58
7.00	6.00	6.67	-1.26	0.56
387.00	427.00	419.33	-1.25	0.00
11784.00	13393.00	13096.33	1.40	0.00
1586.00	1736.00	1737.67	1.15	0.00
1163.00	1302.00	1285.33	-1.01	0.78
6.00	10.00	7.67	-1.19	0.68
263.00	311.00	292.33	-1.02	0.78
2013.00	2494.00	2357.33	1.20	0.00
5030.00	5681.00	5550.67	1.22	0.00
11.00	10.00	10.00	-1.31	0.35
1012.00	994.00	1022.67	-1.06	0.18
697.00	754.00	742.67	1.13	0.01
2145.00	2195.00	2216.67	-1.04	0.25
4505.00	4538.00	4507.00	1.11	0.01
2884.00	3165.00	3088.33	1.18	0.00
857.00	854.00	848.00	-1.04	0.46
4522.00	4146.00	4203.33	-1.15	0.01
1204.00	1256.00	1326.33	-1.14	0.01
1168.00	1423.00	1375.00	1.17	0.00
2.00	1.00	3.67	-1.46	0.45
503.00	549.00	533.33	-1.02	0.68
592.00	550.00	561.33	1.21	0.00
1787.00	1648.00	1709.67	-1.28	0.00
369.00	331.00	337.00	1.00	0.97
331.00	416.00	376.67	1.20	0.00
22.00	18.00	24.00	1.77	0.01
1074.00	1089.00	1097.33	-1.03	0.48
2069.00	2038.00	2118.00	1.08	0.04
494.00	470.00	496.33	-1.13	0.02
55.00	50.00	55.33	-1.24	0.07
163.00	126.00	137.00	-1.17	0.12
545.00	579.00	566.33	-1.19	0.00
15695.00	15009.00	15282.00	-1.11	0.01
1624.00	1405.00	1463.33	-1.06	0.38
68.00	65.00	66.67	1.32	0.02
144.00	131.00	140.00	1.24	0.01

754.00	750.00	740.33	-1.15	0.02
6.00	2.00	3.67	-1.28	0.70
784.00	755.00	758.33	-1.29	0.00
0.00	0.00	0.00	-11.75	0.13
305.00	337.00	320.00	1.08	0.22
295.00	315.00	304.00	-1.32	0.00
5.00	5.00	4.33	1.41	0.53
18.00	10.00	14.67	-1.29	0.29
1865.00	1678.00	1802.33	-1.21	0.00
0.00	0.00	0.33	-4.69	0.13
190.00	215.00	217.00	2.30	0.00
4451.00	4464.00	4498.33	1.22	0.00
172.00	190.00	178.00	1.10	0.27
1150.00	1204.00	1245.67	1.10	0.05
158.00	220.00	202.33	1.28	0.00
374.00	409.00	426.67	1.14	0.03
1164.00	1379.00	1372.67	1.26	0.00
16506.00	15473.00	16205.67	1.18	0.00
7.00	7.00	7.67	1.50	0.26
39914.00	46389.00	43895.67	1.22	0.00
66.00	80.00	84.67	1.27	0.05
2237.00	2291.00	2227.33	1.00	0.99
2802.00	3116.00	2977.33	1.09	0.03
423.00	509.00	468.67	-1.06	0.32
2351.00	2575.00	2517.33	-1.10	0.01
25.00	27.00	29.00	1.24	0.22
833.00	925.00	958.33	1.02	0.75
164.00	162.00	154.33	-1.02	0.82
170.00	181.00	187.67	1.27	0.00
1039.00	1156.00	1123.67	1.04	0.28
599.00	616.00	585.67	1.36	0.00
162.00	148.00	161.33	1.11	0.22
5748.00	5640.00	5743.33	-1.63	0.00
2.00	6.00	3.33	-1.11	1.00
792.00	762.00	814.33	1.09	0.08
2156.00	2087.00	2182.67	-1.14	0.00
1012.00	1019.00	1018.67	1.08	0.11
3.00	3.00	7.00	2.23	0.08
1.00	6.00	2.67	-1.50	0.51
1159.00	1299.00	1286.67	-1.04	0.34
11.00	7.00	7.33	-1.10	0.89
48.00	51.00	50.33	-1.27	0.05
21.00	20.00	19.33	-1.03	1.00
1541.00	1714.00	1666.67	1.07	0.07
324.00	338.00	346.33	1.12	0.06
1154.00	1457.00	1365.33	1.45	0.00
349.00	348.00	345.67	-1.02	0.83
1065.00	1095.00	1127.00	-1.02	0.68

6049.00	5806.00	5982.33	-1.25	0.00
4.00	7.00	5.33	-1.20	0.74
607.00	635.00	641.00	-1.40	0.00
236.00	205.00	232.00	1.25	0.00
224.00	212.00	209.67	1.02	0.80
7965.00	8075.00	8258.33	-1.12	0.00
34.00	25.00	33.33	1.28	0.14
181.00	175.00	195.67	-1.04	0.58
1.00	0.00	0.33	-3.95	0.23
4445.00	4613.00	4741.33	-1.06	0.07
2627.00	2876.00	2961.00	1.13	0.00
4473.00	4486.00	4501.33	-1.12	0.00
191.00	210.00	200.33	1.96	0.00
18.00	12.00	14.00	-1.23	0.44
399.00	444.00	394.33	1.10	0.23
1225.00	1267.00	1269.67	1.08	0.06
554.00	586.00	595.33	1.20	0.00
336.00	357.00	378.00	-1.26	0.00
26.00	19.00	25.00	1.34	0.13
6.00	4.00	3.67	-1.90	0.12
3961.00	4179.00	4166.00	-1.00	0.93
441.00	441.00	452.00	1.23	0.00
2923.00	3343.00	3016.33	-1.00	0.99
832.00	830.00	841.67	-1.10	0.03
518.00	521.00	554.67	-1.18	0.00
218.00	217.00	219.67	1.34	0.00
1799.00	1996.00	1961.67	-1.02	0.55
136.00	143.00	154.67	-1.53	0.00
587.00	666.00	626.00	1.11	0.03
1970.00	2006.00	1964.33	1.14	0.00
27284.00	29244.00	28648.33	-1.39	0.00
3315.00	3371.00	3384.67	-1.13	0.00
1334.00	1434.00	1432.00	-1.13	0.00
1344.00	1542.00	1475.33	1.11	0.02
296.00	347.00	331.33	1.35	0.00
558.00	596.00	587.00	1.05	0.36
1182.00	1183.00	1183.67	-1.01	0.82
2267.00	2334.00	2364.67	-1.09	0.02
1145.00	1081.00	1183.00	-1.04	0.41
2491.00	2896.00	2730.33	1.03	0.44
1136.00	1276.00	1285.67	1.14	0.01
1071.00	1106.00	1148.67	1.18	0.00
22.00	28.00	30.33	-1.03	0.95
290.00	293.00	292.00	1.08	0.23
875.00	961.00	910.33	1.01	0.78
21.00	16.00	22.33	1.32	0.23
2.00	7.00	5.33	-1.32	0.52
1431.00	1239.00	1370.33	-1.23	0.00

1125.00	1305.00	1263.67	1.08	0.07
2853.00	2760.00	2868.00	-1.09	0.03
1583.00	1791.00	1725.33	1.12	0.02
198.00	221.00	221.33	1.09	0.21
1266.00	1489.00	1430.33	1.12	0.02
1883.00	2261.00	2179.00	1.13	0.01
3378.00	3355.00	3587.00	-1.01	0.88
21.00	16.00	20.67	-1.05	0.86
628.00	741.00	696.67	1.06	0.25
1035.00	1173.00	1174.00	-1.16	0.00
642.00	645.00	644.67	-1.05	0.42
1148.00	1319.00	1325.33	-1.03	0.56
27.00	18.00	26.33	-1.47	0.02
134.00	162.00	156.00	-1.01	0.98
205.00	199.00	203.67	1.37	0.00
324.00	363.00	338.33	1.09	0.14
1067.00	1125.00	1144.00	-1.08	0.07
1923.00	2384.00	2180.00	1.17	0.00
393.00	451.00	451.00	-1.02	0.78
23.00	49.00	44.00	1.49	0.03
63.00	69.00	69.67	1.05	0.72
1279.00	1265.00	1297.67	1.01	0.87
896.00	873.00	946.67	1.10	0.07
152.00	120.00	144.33	1.04	0.71
1598.00	1719.00	1750.67	1.01	0.72
1392.00	1326.00	1414.67	1.07	0.11
94.00	55.00	76.33	-1.35	0.04
220.00	221.00	226.00	-1.01	0.87
35.00	24.00	27.00	1.38	0.08
21.00	19.00	25.00	1.25	0.26
7578.00	7779.00	7712.67	1.14	0.00
6.00	2.00	5.00	-1.61	0.21
39.00	41.00	46.67	1.20	0.21
1733.00	1782.00	1825.00	-1.03	0.46
1594.00	1736.00	1667.33	-1.04	0.31
8.00	13.00	11.00	1.35	0.30
722.00	765.00	754.00	1.23	0.00
4.00	8.00	6.33	-1.43	0.32
3.00	1.00	3.00	-1.12	1.00
179.00	159.00	178.33	1.07	0.40
455.00	420.00	446.67	-1.14	0.01
46.00	29.00	31.67	-1.47	0.05
386.00	383.00	427.67	1.25	0.00
1069.00	1245.00	1194.00	1.08	0.11
1227.00	1171.00	1241.33	-1.12	0.01
1903.00	2021.00	2005.00	1.05	0.22
1716.00	1957.00	1859.00	1.01	0.88
43.00	59.00	60.00	1.30	0.05

5987.00	6024.00	6199.67	1.12	0.00
837.00	863.00	892.67	1.10	0.04
600.00	677.00	639.33	-1.34	0.00
518.00	541.00	561.33	-1.07	0.20
31.00	18.00	28.33	2.03	0.00
634.00	583.00	654.00	-1.18	0.01
2417.00	2742.00	2667.67	1.09	0.02
645.00	633.00	676.33	1.09	0.08
697.00	789.00	809.67	1.09	0.14
1175.00	1247.00	1204.67	-1.06	0.15
463.00	487.00	492.33	1.14	0.02
1148.00	1303.00	1268.00	1.06	0.16
523.00	462.00	505.33	-1.00	1.00
6414.00	7801.00	7415.00	1.10	0.02
190.00	226.00	213.33	1.11	0.14
80.00	76.00	79.33	-1.19	0.08
435.00	424.00	448.33	-1.01	0.84
474.00	535.00	524.67	-1.03	0.60
2525.00	2517.00	2620.00	1.08	0.04
5498.00	6216.00	6105.33	1.17	0.00
1566.00	1544.00	1636.00	-1.04	0.29
896.00	873.00	939.00	1.16	0.00
1403.00	1583.00	1496.00	1.27	0.00
736.00	861.00	800.67	1.15	0.01
50.00	65.00	61.33	-1.07	0.54
752.00	872.00	829.00	-1.05	0.28
6296.00	7178.00	6735.00	1.17	0.00
5.00	4.00	5.67	-1.42	0.36
24.00	16.00	23.00	1.54	0.04
2811.00	2753.00	2890.33	1.16	0.00
2698.00	2895.00	2913.00	1.13	0.00
2006.00	2315.00	2177.00	-1.08	0.06
161.00	186.00	178.67	1.71	0.00
387.00	475.00	418.00	1.08	0.27
109.00	80.00	81.67	-1.90	0.00
235.00	207.00	227.67	1.06	0.47
613.00	713.00	713.00	1.16	0.01
275.00	323.00	294.33	1.14	0.05
892.00	960.00	960.00	-1.11	0.01
335.00	400.00	371.33	1.29	0.00
393.00	401.00	406.67	1.11	0.06
464.00	411.00	401.00	-1.45	0.00
276.00	319.00	300.00	-1.01	0.85
11.00	18.00	16.67	1.17	0.54
1332.00	1538.00	1533.67	1.10	0.04
12.00	13.00	14.33	-1.65	0.02
9.00	12.00	10.00	1.28	0.42
26.00	35.00	32.67	1.19	0.32

1764.00	1790.00	1835.67	-1.04	0.34
7.00	19.00	13.00	-1.50	0.09
1521.00	1369.00	1427.00	-1.26	0.00
8325.00	8860.00	8513.33	1.06	0.17
1281.00	1523.00	1425.33	1.12	0.01
982.00	1011.00	1048.33	1.10	0.04
2200.00	2275.00	2282.67	1.01	0.74
374.00	377.00	364.67	-1.05	0.43
22.00	17.00	22.00	-1.21	0.30
540.00	566.00	552.33	-1.02	0.70
2577.00	2664.00	2623.33	-1.08	0.05
925.00	911.00	938.00	1.16	0.00
61.00	62.00	61.67	-1.05	0.74
659.00	715.00	676.00	-1.36	0.00
2886.00	3057.00	3118.67	1.12	0.00
12.00	13.00	10.67	1.02	1.00
8.00	7.00	5.67	-1.01	1.00
9.00	6.00	7.33	-1.15	0.78
6490.00	7364.00	7271.67	1.25	0.00
1494.00	1730.00	1675.33	1.23	0.00
672.00	757.00	718.00	-1.03	0.62
357.00	363.00	355.33	-1.03	0.66
3796.00	4306.00	4056.00	-1.03	0.39
1043.00	1088.00	1122.33	1.01	0.80
1444.00	1613.00	1571.67	-1.07	0.10
979.00	1024.00	1040.00	1.05	0.23
5141.00	5229.00	5350.00	1.01	0.74
866.00	995.00	1015.33	-1.06	0.25
580.00	790.00	717.67	1.19	0.01
1361.00	1530.00	1487.00	1.14	0.00
6045.00	5892.00	5948.00	1.19	0.00
981.00	1058.00	1039.00	1.07	0.12
449.00	501.00	488.67	1.10	0.08
2964.00	3255.00	3273.00	1.65	0.00
476.00	591.00	544.33	1.16	0.01
22.00	32.00	21.67	-1.41	0.07
1440.00	1462.00	1464.00	1.07	0.09
569.00	615.00	576.33	-1.47	0.00
350.00	369.00	368.67	-1.09	0.10
6.00	5.00	6.00	-1.40	0.38
182.00	196.00	200.33	1.23	0.00
572.00	603.00	583.67	-1.17	0.00
183.00	220.00	207.00	1.07	0.37
2734.00	2929.00	2851.00	1.17	0.00
7.00	8.00	6.00	-2.22	0.01
529.00	617.00	607.33	-1.45	0.00
20.00	22.00	29.33	1.17	0.49
91.00	89.00	88.67	-1.26	0.02

198.00	207.00	218.00	-1.38	0.00
58.00	84.00	78.00	1.67	0.00
5.00	1.00	5.33	-1.14	0.87
2530.00	2881.00	2765.00	1.11	0.00
689.00	765.00	749.67	1.09	0.06
3038.00	3268.00	3121.33	1.18	0.00
1494.00	1787.00	1652.33	1.24	0.00
1427.00	1339.00	1384.33	-1.19	0.00
6.00	10.00	5.67	1.28	0.60
13.00	11.00	11.00	1.41	0.24
1703.00	1718.00	1717.67	-1.02	0.68
45.00	45.00	49.67	1.03	0.83
4.00	8.00	5.67	-1.48	0.29
1.00	8.00	4.67	1.06	1.00
5.00	7.00	5.67	1.28	0.59
602.00	588.00	617.67	-1.22	0.00
216.00	235.00	220.67	-1.58	0.00
8.00	4.00	6.67	-1.06	1.00
60.00	75.00	65.00	1.20	0.12
7.00	15.00	11.00	1.62	0.11
3.00	1.00	3.33	-1.40	0.55
686.00	651.00	707.67	1.08	0.11
1.00	0.00	1.00	-3.41	0.06
763.00	751.00	772.00	-1.09	0.06
349.00	415.00	421.67	1.09	0.20
1.00	2.00	2.00	-1.97	0.25
6.00	4.00	7.67	1.33	0.44
1682.00	1788.00	1716.00	-1.18	0.00
88.00	111.00	110.00	-1.13	0.22
1680.00	1699.00	1717.67	-1.02	0.65
517.00	611.00	589.33	-1.10	0.17
826.00	898.00	841.00	1.17	0.01
2338.00	2410.00	2352.00	-1.01	0.87
794.00	890.00	829.33	-1.05	0.29
2653.00	2916.00	2894.00	1.12	0.00
588.00	630.00	632.33	1.08	0.12
2372.00	2584.00	2538.33	-1.10	0.01
3571.00	3825.00	3859.00	1.07	0.05
1023.00	1155.00	1161.33	1.17	0.00
469.00	471.00	471.67	1.23	0.00
2318.00	2423.00	2445.00	-1.40	0.00
524.00	575.00	554.33	-1.06	0.26
19.00	23.00	19.00	-1.17	0.50
5.00	3.00	3.33	-1.11	1.00
87.00	89.00	87.33	-1.11	0.29
57.00	66.00	73.33	1.12	0.37
548.00	631.00	592.33	1.25	0.00
2755.00	2883.00	2804.00	-1.07	0.09

1791.00	1882.00	1903.33	1.02	0.64
2396.00	2487.00	2495.67	1.00	0.91
856.00	987.00	904.00	-1.07	0.21
1150.00	1222.00	1231.00	1.15	0.00
518.00	549.00	553.00	1.02	0.68
1740.00	1784.00	1783.00	-1.00	0.94
2322.00	2567.00	2511.33	1.08	0.05
481.00	571.00	540.33	1.10	0.07
1900.00	1787.00	1829.33	-1.01	0.78
12.00	14.00	14.33	1.09	0.75
1217.00	1370.00	1334.33	-1.04	0.39
736.00	758.00	792.67	-1.18	0.00
18.00	25.00	20.33	-1.21	0.34
4214.00	5091.00	4729.67	1.01	0.80
1806.00	2012.00	1885.67	1.06	0.14
97.00	119.00	110.00	1.04	0.70
944.00	970.00	961.00	1.14	0.01
1639.00	1777.00	1736.00	1.08	0.03
2418.00	2660.00	2642.67	1.28	0.00
2558.00	2532.00	2521.00	-1.27	0.00
738.00	876.00	842.33	1.10	0.07
400.00	420.00	430.00	1.08	0.17
9795.00	11833.00	11356.00	-1.09	0.05
1346.00	1492.00	1497.33	-1.06	0.14
646.00	787.00	743.33	-1.05	0.42
1146.00	1209.00	1236.33	1.09	0.04
164.00	214.00	186.67	1.38	0.00
18.00	22.00	21.67	-1.67	0.00
269.00	294.00	288.00	1.21	0.00
9.00	6.00	6.33	-1.32	0.48
435.00	507.00	494.00	-1.08	0.16
564.00	602.00	614.33	-1.04	0.40
2515.00	2741.00	2728.33	-1.10	0.01
2037.00	2331.00	2242.33	-1.04	0.29
422.00	469.00	458.33	1.01	0.94
1876.00	1952.00	1993.67	1.18	0.00
1660.00	1891.00	1784.67	1.12	0.02
633.00	638.00	636.00	-1.22	0.00
437.00	484.00	450.67	1.03	0.68
25.00	23.00	24.33	1.03	0.88
6110.00	6805.00	6527.33	1.16	0.00
285.00	362.00	345.33	1.02	0.77
575.00	637.00	614.33	1.17	0.00
6.00	3.00	4.67	1.51	0.41
963.00	914.00	937.33	-1.17	0.00
95.00	104.00	107.00	-1.06	0.57
929.00	955.00	943.33	-1.08	0.08
412.00	464.00	480.67	1.17	0.02

622.00	743.00	685.00	-1.01	0.87
370.00	332.00	329.33	1.01	0.91
2031.00	2062.00	2115.00	1.49	0.00
10.00	11.00	11.33	1.15	0.62
23.00	38.00	25.00	-1.12	0.67
871.00	922.00	891.33	1.07	0.16
136.00	155.00	139.00	-1.08	0.37
2345.00	2531.00	2466.33	1.14	0.00
2345.00	2706.00	2631.00	1.17	0.00
690.00	681.00	706.33	1.03	0.48
897.00	936.00	956.67	1.01	0.80
287.00	315.00	300.00	1.12	0.13
667.00	638.00	700.33	1.14	0.01
9977.00	10148.00	10120.33	1.06	0.12
2689.00	3021.00	2873.33	1.12	0.00
4948.00	5158.00	5217.33	-1.18	0.00
636.00	704.00	656.00	1.04	0.47
17.00	27.00	24.33	-1.08	0.65
814.00	964.00	892.00	-1.06	0.22
379.00	400.00	407.33	1.04	0.51
4204.00	4449.00	4517.00	1.10	0.03
8.00	5.00	7.00	1.29	0.52
94.00	55.00	75.00	1.25	0.10
7.00	11.00	12.00	1.01	1.00
24.00	28.00	22.67	-1.78	0.00
3090.00	3380.00	3225.67	1.13	0.00
75.00	67.00	76.33	1.10	0.47
243.00	218.00	234.33	-1.13	0.06
2827.00	3068.00	3022.67	1.15	0.00
1200.00	1240.00	1284.00	1.15	0.01
1366.00	1315.00	1369.67	-1.06	0.22
1.00	4.00	2.67	1.12	1.00
62.00	66.00	71.67	-1.01	0.93
1007.00	1113.00	1058.67	1.17	0.00
1087.00	1093.00	1104.67	1.15	0.00
2969.00	3084.00	3111.33	1.02	0.73
72.00	67.00	76.33	-1.10	0.39
88.00	61.00	72.00	-1.34	0.01
2611.00	2431.00	2649.33	1.03	0.53
632.00	654.00	708.00	1.54	0.00
259.00	266.00	281.67	1.10	0.25
138.00	170.00	158.00	-1.37	0.00
45.00	49.00	44.67	-2.47	0.00
551.00	459.00	500.00	-1.21	0.00
14.00	11.00	12.67	-1.36	0.22
27.00	33.00	34.33	-1.72	0.00
30.00	34.00	31.67	-1.43	0.01
3489.00	3420.00	3614.00	1.03	0.40

1328.00	1508.00	1443.00	-1.07	0.11
22.00	12.00	17.33	-1.88	0.00
3075.00	3201.00	3231.00	-1.07	0.06
2255.00	1986.00	2164.00	-1.17	0.00
39.00	50.00	47.67	-1.47	0.00
979.00	1147.00	1071.00	1.08	0.08
1605.00	1578.00	1604.33	-1.14	0.00
929.00	1039.00	1024.00	-1.03	0.52
522.00	559.00	529.33	-1.23	0.00
1439.00	1360.00	1441.33	-1.00	0.92
1702.00	1916.00	1882.67	1.02	0.65
894.00	847.00	907.67	1.12	0.01
2532.00	2803.00	2661.67	1.13	0.00
522.00	642.00	616.00	1.44	0.00
582.00	772.00	674.00	1.31	0.00
976.00	1154.00	1141.67	1.19	0.00
758.00	852.00	845.00	1.23	0.00
69.00	66.00	72.00	1.04	0.72
101.00	87.00	96.33	-1.22	0.03
447.00	559.00	504.33	1.24	0.00
1541.00	1506.00	1613.67	-1.13	0.01
5.00	4.00	6.67	-1.66	0.11
1702.00	1719.00	1704.00	-1.01	0.83
1282.00	1240.00	1297.00	1.05	0.24
4.00	6.00	6.00	2.46	0.05
853.00	891.00	944.33	1.14	0.01
1792.00	1968.00	1899.67	1.18	0.00
384.00	371.00	392.67	-1.19	0.00
17.00	15.00	16.00	1.81	0.02
513.00	600.00	571.00	1.08	0.29
45.00	54.00	47.67	1.19	0.22
1398.00	1619.00	1523.67	1.27	0.00
571.00	606.00	571.33	-1.04	0.52
3130.00	3174.00	3193.33	1.11	0.00
761.00	849.00	852.67	1.06	0.19
241.00	303.00	261.00	1.07	0.44
2.00	4.00	2.67	-1.62	0.39
31.00	34.00	30.67	-1.05	0.84
54.00	35.00	46.67	1.70	0.00
401.00	405.00	412.67	-1.01	0.90
13.00	16.00	15.00	-1.01	1.00
352.00	353.00	375.67	-1.39	0.00
6256.00	6776.00	6831.33	1.07	0.06
2.00	2.00	2.33	-1.98	0.20
311.00	250.00	255.67	-1.55	0.00
13.00	11.00	10.00	-1.51	0.12
1291.00	1414.00	1412.00	-1.18	0.00
1261.00	1246.00	1241.33	-1.22	0.00

173.00	198.00	196.33	-1.19	0.02
31.00	16.00	24.00	1.15	0.53
69.00	66.00	73.33	-1.23	0.05
311.00	305.00	318.67	1.25	0.00
110.00	125.00	121.33	1.07	0.50
566.00	628.00	631.00	1.13	0.01
2.00	5.00	3.67	-1.46	0.46
2561.00	2808.00	2832.67	1.46	0.00
2785.00	3224.00	3085.00	2.78	0.00
174.00	177.00	178.00	1.08	0.31
1.00	0.00	1.00	-4.92	0.01
4.00	10.00	7.00	-1.06	1.00
1725.00	1842.00	1797.00	-1.04	0.32
518.00	525.00	534.33	1.10	0.08
660.00	806.00	734.33	1.13	0.03
873.00	849.00	899.00	-1.69	0.00
974.00	1019.00	1028.00	-1.25	0.00
85.00	67.00	82.00	-1.61	0.00
8.00	3.00	5.00	-1.47	0.33
1759.00	1979.00	2021.67	1.14	0.00
85.00	107.00	108.00	-1.22	0.03
689.00	728.00	751.67	1.26	0.00
5822.00	6211.00	6247.33	1.16	0.00
100.00	106.00	99.67	-1.79	0.00
466.00	474.00	476.67	-1.08	0.12
291.00	302.00	303.33	1.11	0.07
3064.00	3503.00	3268.33	-1.05	0.23
3151.00	3393.00	3520.00	1.12	0.01
99.00	104.00	112.67	1.19	0.07
598.00	619.00	652.00	1.03	0.56
1.00	7.00	3.33	-1.79	0.20
239.00	269.00	275.67	1.16	0.05
931.00	1036.00	1033.33	1.01	0.86
822.00	852.00	867.67	1.06	0.24
2481.00	2418.00	2613.67	-1.08	0.08
97.00	93.00	108.00	1.02	0.83
8.00	13.00	11.00	1.25	0.45
110.00	124.00	109.67	1.22	0.06
12.00	9.00	8.00	-1.30	0.44
10.00	12.00	12.67	1.95	0.02
959.00	931.00	948.33	-1.18	0.00
1750.00	1756.00	1863.67	-1.03	0.53
428.00	527.00	473.67	1.07	0.25
1149.00	1208.00	1170.67	-1.27	0.00
1634.00	1827.00	1676.33	1.05	0.36
475.00	519.00	512.33	-1.25	0.00
180.00	199.00	203.67	-1.10	0.18
1.00	1.00	1.67	-1.39	0.78

1078.00	1324.00	1170.33	1.05	0.39
20.00	34.00	22.67	-1.25	0.28
60.00	101.00	81.33	1.06	0.70
3.00	3.00	3.33	1.61	0.46
1048.00	993.00	1081.33	-1.29	0.00
2186.00	2324.00	2343.00	1.29	0.00
702.00	647.00	700.00	-1.43	0.00
65.00	68.00	63.67	-1.09	0.45
614.00	786.00	729.33	1.08	0.18
1075.00	1261.00	1205.33	1.21	0.00
1070.00	1260.00	1195.00	1.01	0.84
1529.00	1727.00	1628.33	-1.00	1.00
872.00	921.00	968.33	-1.06	0.23
1104.00	1245.00	1199.67	1.01	0.77
66.00	75.00	75.33	1.16	0.20
506.00	568.00	572.00	1.04	0.54
563.00	582.00	607.00	1.09	0.12
445.00	498.00	481.00	-1.08	0.13
1904.00	2075.00	1954.67	1.04	0.43
15.00	14.00	14.67	1.17	0.53
1129.00	1365.00	1299.67	1.13	0.01
121.00	111.00	113.00	1.20	0.06
1131.00	1236.00	1211.67	1.14	0.00
235.00	278.00	268.67	1.45	0.00
190.00	231.00	212.33	1.26	0.00
697.00	706.00	712.00	1.00	0.97
657.00	723.00	729.00	1.14	0.01
343.00	394.00	384.33	1.22	0.00
5.00	36.00	22.67	1.68	0.18
21.00	81.00	55.33	1.54	0.10
160.00	374.00	278.33	-1.22	0.41
145.00	400.00	282.00	-1.56	0.10
2.00	10.00	6.00	1.93	0.13
11.00	12.00	11.67	1.11	0.72
30.00	23.00	28.33	1.52	0.02
735.00	694.00	702.33	1.05	0.41
282.00	294.00	306.67	1.21	0.01
780.00	990.00	904.67	1.28	0.00
445.00	467.00	493.33	1.16	0.01
6.00	13.00	7.33	-1.10	0.89
1528.00	1544.00	1629.00	-1.13	0.00
89.00	126.00	115.00	1.05	0.66
59.00	76.00	66.67	1.31	0.03
576.00	578.00	580.33	1.10	0.05
1.00	0.00	0.33	-3.21	0.41
5933.00	6416.00	6463.67	1.22	0.00
1766.00	1751.00	1797.67	-1.13	0.00
765.00	728.00	734.33	-1.03	0.61

838.00	1035.00	928.00	-1.17	0.00
859.00	849.00	832.00	-1.13	0.03
3.00	3.00	2.67	-1.50	0.51
242.00	256.00	258.00	1.06	0.34
1890.00	2038.00	2089.00	1.14	0.00
360.00	367.00	372.00	-1.20	0.00
1437.00	1406.00	1435.67	-1.16	0.00
15.00	13.00	14.00	1.12	0.67
1392.00	1363.00	1393.00	-1.06	0.14
204.00	205.00	214.67	1.41	0.00
1053.00	1077.00	1099.67	1.16	0.00
92.00	96.00	91.00	-1.12	0.25
1197.00	1187.00	1188.33	1.12	0.02
750.00	678.00	706.67	1.06	0.27
0.00	1.00	1.33	-1.48	0.76
628.00	704.00	706.67	1.12	0.02
444.00	595.00	503.00	1.38	0.00
1030.00	1206.00	1154.00	-1.07	0.12
83.00	104.00	100.00	1.15	0.18
853.00	944.00	913.67	1.15	0.00
516.00	484.00	515.33	1.14	0.01
134.00	135.00	130.67	1.17	0.09
1615.00	1726.00	1698.33	-1.03	0.48
1081.00	1345.00	1282.33	-1.09	0.09
2907.00	3152.00	3065.33	-1.01	0.73
58.00	43.00	45.00	-1.11	0.53
18.00	28.00	18.67	-1.06	0.88
113.00	102.00	101.33	-1.24	0.02
962.00	913.00	975.00	1.02	0.62
1887.00	1905.00	1988.00	-1.09	0.03
391.00	388.00	397.67	-1.04	0.49
686.00	739.00	719.00	-1.06	0.16
757.00	826.00	791.00	1.12	0.01
6.00	12.00	6.67	-1.46	0.28
844.00	1028.00	990.00	1.04	0.44
162.00	183.00	174.33	-1.06	0.39
652.00	620.00	675.00	-1.08	0.13
1438.00	1489.00	1531.33	-1.07	0.07
803.00	1017.00	906.00	1.04	0.46
552.00	645.00	600.67	1.09	0.08
591.00	670.00	640.67	1.37	0.00
1308.00	1316.00	1302.00	-1.09	0.06
642.00	683.00	697.00	-1.07	0.15
37.00	51.00	46.67	1.11	0.46
4955.00	4519.00	4652.00	-1.30	0.00
487.00	453.00	466.33	-1.07	0.24
962.00	975.00	1002.33	1.17	0.00
2556.00	2718.00	2710.67	-1.12	0.00

675.00	765.00	746.33	1.04	0.39
232.00	290.00	270.33	-1.09	0.18
11.00	12.00	13.00	1.13	0.65
762.00	927.00	872.67	1.07	0.19
0.00	0.00	0.00	-11.75	0.13
1284.00	1436.00	1415.33	-1.29	0.00
469.00	383.00	406.33	-1.26	0.00
454.00	567.00	550.00	1.06	0.35
1687.00	1661.00	1737.33	-1.04	0.39
1075.00	1020.00	1078.00	-1.07	0.10
121.00	128.00	131.00	1.15	0.09
576.00	610.00	636.33	1.06	0.29
450.00	463.00	457.33	-1.00	0.98
747.00	714.00	747.33	1.08	0.09
2274.00	2311.00	2318.67	-1.11	0.01
832.00	934.00	911.00	-1.04	0.36
1630.00	1516.00	1613.67	-1.05	0.28
16.00	23.00	21.00	1.11	0.66
976.00	1137.00	1069.00	1.07	0.14
1293.00	1352.00	1433.33	1.07	0.17
481.00	505.00	505.00	1.03	0.49
629.00	704.00	677.67	1.10	0.06
105.00	139.00	137.00	1.06	0.61
643.00	736.00	682.33	1.07	0.27
2.00	2.00	5.67	-1.25	0.67
36.00	44.00	40.67	-1.17	0.25
1853.00	1991.00	1933.67	-1.14	0.00
358.00	390.00	393.00	1.08	0.19
591.00	680.00	635.67	-1.09	0.08
1450.00	1584.00	1575.67	1.09	0.04
1021.00	992.00	1043.00	-1.07	0.15
3050.00	3184.00	3264.00	1.18	0.00
2.00	0.00	1.33	-1.94	0.39
1.00	3.00	2.67	-1.01	1.00
41.00	37.00	32.00	-1.60	0.01
56.00	76.00	69.67	-1.05	0.63
1459.00	1420.00	1397.67	-1.07	0.19
87.00	96.00	84.67	1.04	0.74
448.00	433.00	490.00	1.16	0.04
1692.00	1744.00	1758.67	-1.04	0.29
1627.00	1767.00	1628.67	1.04	0.52
36.00	40.00	37.33	-1.05	0.81
1137.00	1090.00	1064.33	-1.13	0.04
88.00	81.00	93.67	-1.09	0.40
65.00	71.00	68.00	-1.29	0.02
660.00	709.00	736.00	1.11	0.09
21.00	21.00	21.00	-1.36	0.10
167.00	232.00	195.67	1.30	0.00

22.00	31.00	26.00	1.04	0.88
25.00	8.00	13.00	1.15	0.72
88.00	69.00	81.67	-2.41	0.00
16.00	6.00	10.67	-1.20	0.57
25.00	49.00	38.00	-1.37	0.03
233.00	296.00	266.33	1.08	0.25
281.00	315.00	333.67	1.11	0.15
100.00	105.00	99.67	1.33	0.01
1540.00	1535.00	1569.33	1.15	0.00
666.00	829.00	788.33	1.16	0.01
581.00	662.00	659.67	1.21	0.00
12.00	19.00	14.00	2.16	0.01
1228.00	1347.00	1357.00	-1.02	0.65
1379.00	1452.00	1438.33	-1.17	0.00
487.00	462.00	486.67	1.04	0.45
825.00	929.00	935.67	1.10	0.05
236.00	230.00	259.00	1.06	0.39
1334.00	1437.00	1426.00	1.04	0.36
17.00	33.00	25.67	1.24	0.31
1122.00	1272.00	1239.67	1.20	0.00
1462.00	1650.00	1620.00	1.18	0.00
296.00	297.00	299.33	1.46	0.00
0.00	2.00	1.00	-4.01	0.02
442.00	498.00	476.00	-1.09	0.09
956.00	1025.00	1014.00	1.28	0.00
17200.00	17532.00	19064.67	1.09	0.06
12445.00	13641.00	14047.00	1.17	0.00
17272.00	19342.00	19428.67	1.15	0.00
19810.00	21770.00	22511.33	1.18	0.00
26781.00	25896.00	27512.00	1.15	0.00
29638.00	31874.00	32589.00	1.11	0.00
72.00	57.00	70.00	-1.06	0.62
2270.00	2216.00	2537.67	1.15	0.03
0.00	0.00	1.00	-1.32	1.00
48.00	56.00	48.00	1.19	0.21
9475.00	11028.00	10990.33	1.13	0.00
16476.00	18147.00	18666.33	1.10	0.03
1078.00	1216.00	1213.67	1.11	0.02
11357.00	12153.00	12668.33	1.11	0.02
7455.00	7568.00	7994.33	1.15	0.00
22660.00	25559.00	25622.67	1.13	0.00
36.00	45.00	38.33	1.29	0.11
694.00	792.00	827.33	1.14	0.04
7.00	6.00	6.33	-1.43	0.31
5606.00	6549.00	6420.67	1.19	0.00
3790.00	4288.00	4419.67	1.12	0.03
14220.00	15931.00	16667.33	1.11	0.04
7305.00	8028.00	8433.33	1.08	0.14

86.00	116.00	102.67	-1.11	0.27
147.00	198.00	174.67	1.02	0.87
132.00	105.00	121.67	1.11	0.28
74.00	74.00	80.67	1.19	0.10
7055.00	7706.00	7949.33	1.17	0.00
21274.00	23529.00	24264.67	1.18	0.00
797.00	1023.00	985.33	1.30	0.00
12879.00	14804.00	15048.67	1.17	0.00
13547.00	14904.00	15298.33	1.09	0.03
7596.00	8075.00	8554.00	1.01	0.80
11248.00	12220.00	12668.33	1.11	0.01
39756.00	43586.00	44583.33	1.09	0.04
14989.00	16341.00	16903.67	1.12	0.01
9731.00	10993.00	11668.00	1.15	0.02
11262.00	12557.00	13073.00	1.17	0.00
412.00	339.00	367.33	-1.00	0.97
4904.00	5343.00	5494.00	1.13	0.00
8.00	14.00	12.67	1.69	0.06
7172.00	7838.00	8024.67	1.20	0.00
10239.00	11118.00	11484.33	1.21	0.00
7854.00	8286.00	8708.67	1.17	0.00
391.00	401.00	407.67	-1.06	0.28
1415.00	1676.00	1669.33	1.03	0.55
19051.00	21177.00	22264.67	1.17	0.00
18781.00	20513.00	21621.33	1.14	0.01
5315.00	6351.00	6440.00	1.26	0.00
3961.00	4498.00	4637.67	1.11	0.04
771.00	707.00	770.67	1.41	0.00
31148.00	34068.00	34880.00	1.11	0.01
1038.00	1225.00	1285.00	1.16	0.03
28320.00	32064.00	32465.67	1.16	0.00
17027.00	19288.00	19217.00	1.19	0.00
11321.00	13360.00	13292.00	1.21	0.00
24896.00	27765.00	28138.00	1.13	0.00
1560.00	1444.00	1471.33	1.15	0.01
35761.00	38701.00	39907.00	1.21	0.00
11697.00	12795.00	13170.33	1.17	0.00
34907.00	37027.00	37910.00	1.13	0.00
58.00	45.00	55.33	-1.23	0.10
22256.00	25555.00	25739.67	1.14	0.00
11119.00	12131.00	12500.33	1.17	0.00
8462.00	8888.00	8829.67	-1.03	0.43
13928.00	14025.00	13803.00	-1.03	0.45
634.00	661.00	654.67	1.01	0.79
34.00	33.00	37.33	1.57	0.01
480.00	484.00	532.67	1.08	0.23
1020.00	1209.00	1164.33	1.11	0.02
301.00	360.00	363.33	1.23	0.00

332.00	399.00	368.00	1.19	0.00
4.00	25.00	16.33	1.15	0.77
1307.00	1526.00	1451.33	1.11	0.01
1065.00	1214.00	1215.00	-1.10	0.04
1364.00	1240.00	1316.33	-1.12	0.02
7.00	4.00	4.67	-1.15	0.86
818.00	881.00	902.00	1.25	0.00
65.00	77.00	78.67	1.04	0.77
11784.00	13109.00	13782.00	1.11	0.04
18181.00	19862.00	20599.00	1.14	0.00
8658.00	9729.00	10009.67	1.13	0.01
14776.00	16567.00	17011.33	1.16	0.00
46.00	57.00	53.00	-1.08	0.55
4394.00	4612.00	4845.67	1.11	0.01
10363.00	11861.00	12195.67	1.12	0.02
8.00	11.00	8.33	-1.57	0.11
10790.00	11800.00	12176.00	1.09	0.04
14.00	11.00	13.33	-1.24	0.41
165.00	199.00	197.67	1.20	0.02
76.00	87.00	97.00	1.04	0.78
13714.00	14436.00	14851.33	1.09	0.02
1123.00	1260.00	1232.33	1.10	0.03
30829.00	34299.00	34743.67	1.31	0.00
14385.00	16347.00	16704.00	1.15	0.00
8360.00	9036.00	9718.00	1.07	0.20
13522.00	15299.00	15731.67	1.20	0.00
14231.00	16244.00	16202.00	1.25	0.00
9010.00	10631.00	10848.67	1.15	0.01
193.00	229.00	241.67	1.12	0.19
5673.00	5985.00	6395.67	1.08	0.12
11059.00	12692.00	12811.33	1.15	0.00
851.00	1008.00	1016.67	1.11	0.07
2108.00	2293.00	2328.67	1.12	0.00
6031.00	5931.00	6791.00	1.14	0.04
15.00	33.00	21.33	-1.22	0.37
38390.00	41151.00	42877.67	1.14	0.00
8709.00	9982.00	9920.67	1.17	0.00
37782.00	41498.00	42273.00	1.18	0.00
10099.00	10624.00	11336.67	1.02	0.66
33401.00	36020.00	35524.67	1.19	0.00
356.00	375.00	372.00	1.01	0.90
4719.00	4281.00	4543.33	1.08	0.07
18.00	11.00	15.67	-1.01	1.00
1058.00	1217.00	1142.33	-1.11	0.01
2089.00	2065.00	2118.67	1.10	0.01
66.00	81.00	68.67	-1.09	0.47
101.00	137.00	121.67	-1.20	0.04
784.00	943.00	871.67	-1.06	0.19

555.00	566.00	612.00	1.02	0.79
560.00	585.00	581.00	-1.14	0.00
123.00	129.00	122.67	-1.09	0.36
15681.00	16619.00	17501.67	1.18	0.00
19495.00	21442.00	21932.00	1.14	0.00
378.00	429.00	413.33	1.14	0.02
13398.00	14148.00	14812.67	1.11	0.01
27.00	37.00	35.33	-1.67	0.00
1970.00	2099.00	2230.67	1.13	0.02
33.00	56.00	49.33	-1.06	0.66
1756.00	1645.00	1676.67	-1.06	0.23
948.00	1052.00	1047.33	-1.01	0.89
322.00	377.00	356.33	1.02	0.81
699.00	744.00	753.67	1.07	0.15
642.00	706.00	721.00	1.04	0.41
1751.00	1947.00	1968.00	1.21	0.00
10.00	7.00	10.00	-1.28	0.42
880.00	884.00	906.00	1.13	0.00
276.00	323.00	301.33	-1.17	0.01
670.00	596.00	640.67	-1.11	0.05
11.00	8.00	11.00	-1.01	1.00
310.00	328.00	349.00	1.02	0.77
1528.00	1841.00	1717.67	1.25	0.00
6165.00	6615.00	6441.00	-1.34	0.00
925.00	954.00	936.67	-1.19	0.00
4.00	4.00	4.00	1.18	0.83
1926.00	2073.00	2083.00	1.43	0.00
1922.00	2511.00	2279.67	1.70	0.00
596.00	615.00	612.00	1.18	0.00
19.00	12.00	14.67	1.60	0.07
240.00	312.00	290.67	-1.11	0.14
22.00	12.00	15.33	-1.51	0.05
111.00	88.00	105.33	-1.02	0.84
946.00	1038.00	1030.33	-1.10	0.02
1168.00	1328.00	1279.00	1.09	0.09
3058.00	2971.00	3013.33	-1.06	0.12
876.00	1104.00	1039.67	1.03	0.52
2275.00	2322.00	2277.33	-1.00	0.94
784.00	879.00	866.33	1.09	0.06
737.00	748.00	766.33	1.09	0.07
176.00	182.00	185.00	-1.27	0.00
395.00	405.00	414.00	1.02	0.77
1204.00	1340.00	1307.00	1.04	0.34
1888.00	2000.00	2031.33	1.11	0.01
2.00	4.00	3.33	-1.31	0.68
2800.00	3042.00	3031.67	-1.00	0.97
434.00	464.00	426.00	1.02	0.79
400.00	417.00	409.00	1.03	0.55

58.00	45.00	47.67	-1.56	0.00
655.00	739.00	677.67	-1.05	0.48
97.00	74.00	92.67	-1.08	0.50
4452.00	4895.00	4822.67	1.12	0.00
3492.00	4026.00	3969.33	1.24	0.00
86.00	79.00	80.33	-1.33	0.00
5.00	6.00	5.67	-1.13	0.87
16.00	15.00	14.33	1.36	0.22
1489.00	1587.00	1600.67	1.25	0.00
414.00	438.00	441.67	1.12	0.03
1490.00	1812.00	1754.33	1.08	0.12
1388.00	1538.00	1538.67	-1.16	0.00
1943.00	2074.00	2091.33	1.13	0.00
1495.00	1671.00	1634.67	1.19	0.00
18.00	16.00	18.33	1.18	0.44
1791.00	1962.00	1942.00	1.11	0.00
156.00	144.00	148.00	-1.03	0.76
6.00	19.00	14.00	2.05	0.01
975.00	995.00	1009.67	1.01	0.80
2131.00	2427.00	2428.67	1.12	0.01
336.00	398.00	390.00	1.06	0.37
156.00	197.00	183.00	1.23	0.01
680.00	644.00	658.67	1.63	0.00
88.00	80.00	90.33	-1.51	0.00
6502.00	6392.00	6638.33	1.03	0.45
26566.00	26351.00	27220.33	1.11	0.01
247.00	340.00	296.67	1.23	0.01
48.00	29.00	38.00	1.21	0.24
11.00	3.00	5.67	1.29	0.60
360.00	423.00	400.67	-1.07	0.26
692.00	722.00	746.00	-1.09	0.07
490.00	550.00	529.33	-1.20	0.00
842.00	1001.00	959.33	-1.30	0.00
380.00	364.00	376.00	-1.16	0.01
9.00	8.00	8.67	1.22	0.57
26.00	46.00	35.33	1.36	0.08
2026.00	2170.00	2106.33	-1.02	0.66
2.00	5.00	4.00	1.46	0.51
327.00	391.00	347.00	1.57	0.00
864.00	920.00	914.33	-1.22	0.00
583.00	604.00	622.67	-1.13	0.02
12.00	16.00	15.67	-1.68	0.01
2665.00	2578.00	2685.33	-1.44	0.00
2748.00	3202.00	3071.00	1.13	0.00
2581.00	2835.00	2786.67	1.19	0.00
807.00	925.00	906.33	1.13	0.01
216.00	256.00	250.67	-1.09	0.20
601.00	592.00	634.67	1.25	0.00

291.00	342.00	324.00	1.11	0.18
239.00	311.00	271.33	1.24	0.00
158.00	178.00	169.33	1.11	0.18
340.00	286.00	325.67	-1.07	0.31
4.00	7.00	5.67	-1.36	0.46
627.00	664.00	680.33	-1.01	0.87
2640.00	2798.00	2753.33	1.16	0.00
71.00	74.00	74.00	-1.21	0.06
277.00	310.00	327.00	-1.17	0.03
1783.00	1951.00	2001.00	1.09	0.05
1101.00	1253.00	1269.33	1.13	0.01
7.00	7.00	8.33	-1.05	1.00
3760.00	3844.00	4134.67	1.15	0.00
52.00	52.00	56.33	-1.25	0.05
3.00	5.00	4.67	-1.58	0.25
3.00	2.00	3.67	-1.01	1.00
0.00	2.00	2.33	-1.15	1.00
3830.00	4120.00	4278.00	-1.24	0.00
8.00	13.00	13.33	1.35	0.30
711.00	797.00	790.00	-1.13	0.01
218.00	242.00	231.00	1.03	0.77
80.00	82.00	80.00	-1.25	0.02
567.00	598.00	583.33	-1.17	0.00
584.00	667.00	637.00	1.21	0.00
329.00	332.00	346.00	1.28	0.00
1154.00	1239.00	1221.67	1.08	0.06
3775.00	4422.00	4028.33	-1.19	0.00
2319.00	2550.00	2520.67	1.14	0.00
2923.00	3175.00	3096.00	1.05	0.18
1470.00	1589.00	1535.33	1.00	0.99
69.00	83.00	76.00	-1.19	0.12
408.00	438.00	440.00	1.04	0.44
257.00	225.00	255.00	1.11	0.24
239.00	234.00	237.00	-1.29	0.00
38.00	52.00	44.00	1.10	0.52
17.00	14.00	18.67	1.02	1.00
434.00	544.00	511.33	-1.06	0.28
3011.00	3216.00	3166.00	1.08	0.02
1992.00	1901.00	1924.33	-1.17	0.00
166.00	142.00	153.67	1.06	0.45
1374.00	1429.00	1407.67	-1.07	0.10
561.00	604.00	555.67	-1.03	0.62
262.00	285.00	284.00	-1.31	0.00
527.00	572.00	540.33	-1.01	0.91
771.00	817.00	821.00	1.11	0.02
7.00	12.00	10.00	1.18	0.60
644.00	637.00	649.00	-1.03	0.53
1231.00	1513.00	1489.33	1.15	0.02

72.00	83.00	87.33	-1.46	0.00
194.00	206.00	205.67	1.33	0.00
1046.00	1070.00	1100.67	1.07	0.14
563.00	510.00	525.67	-1.08	0.25
9.00	13.00	11.00	-1.53	0.09
672.00	660.00	671.33	1.07	0.27
1923.00	2223.00	2186.67	1.09	0.03
953.00	991.00	1019.67	1.08	0.08
1732.00	1783.00	1761.33	-1.08	0.04
63.00	73.00	65.33	-1.20	0.12
789.00	777.00	802.00	-1.02	0.66
574.00	727.00	691.33	1.11	0.07
9456.00	10003.00	10144.33	1.04	0.23
391.00	450.00	445.33	1.07	0.24
3383.00	3597.00	3581.67	1.11	0.01
1414.00	1605.00	1504.67	1.04	0.40
2800.00	2981.00	2832.00	1.29	0.00
512.00	579.00	536.00	1.15	0.01
317.00	359.00	349.00	-1.27	0.00
790.00	736.00	829.00	-1.10	0.08
588.00	540.00	562.67	-1.06	0.30
3.00	7.00	6.67	1.50	0.31
876.00	867.00	925.33	-1.14	0.00
287.00	320.00	304.67	1.08	0.20
13.00	14.00	11.67	1.15	0.63
313.00	331.00	339.00	1.03	0.68
1625.00	1975.00	1847.33	1.00	0.96
623.00	768.00	742.67	1.13	0.03
2143.00	2021.00	2117.00	-1.17	0.00
1190.00	1094.00	1135.67	-1.14	0.01
64.00	66.00	69.00	-1.16	0.16
6.00	7.00	6.67	1.16	0.75
1592.00	1691.00	1643.33	1.06	0.15
53.00	37.00	42.33	-1.39	0.02
242.00	228.00	231.67	-1.18	0.03
726.00	796.00	757.00	1.18	0.00
1407.00	1402.00	1425.67	-1.12	0.00
2235.00	2560.00	2379.67	-1.08	0.05
1158.00	1257.00	1237.00	-1.06	0.15
1754.00	1948.00	1899.67	1.02	0.52
255.00	296.00	273.33	-1.14	0.05
1049.00	1104.00	1004.67	1.23	0.00
183.00	197.00	188.33	1.12	0.16
1249.00	1146.00	1213.67	-1.12	0.02
1717.00	1482.00	1663.33	-1.12	0.04
62.00	70.00	72.00	1.46	0.00
664.00	670.00	684.00	1.24	0.00
197.00	187.00	194.67	-1.04	0.62

2492.00	2528.00	2569.67	-1.19	0.00
227.00	234.00	245.67	1.06	0.41
7.00	12.00	11.33	-1.96	0.00
2333.00	2387.00	2398.33	-1.02	0.63
4612.00	4469.00	4607.00	1.06	0.14
782.00	758.00	779.00	-1.21	0.00
2.00	0.00	0.67	-2.29	0.46
124.00	124.00	133.00	-1.05	0.55
26.00	38.00	32.67	-1.00	1.00
11.00	19.00	15.00	-1.24	0.38
2.00	1.00	3.00	-1.45	0.55
0.00	3.00	2.67	1.30	0.79
10.00	12.00	13.33	-1.39	0.13
28.00	33.00	27.67	-1.37	0.05
4.00	0.00	3.00	-1.12	1.00
4.00	0.00	2.67	-1.38	0.65
1.00	3.00	3.00	1.10	1.00
1.00	1.00	2.67	1.29	0.80
9.00	5.00	7.00	1.21	0.63
231.00	190.00	219.00	1.02	0.80
1439.00	1504.00	1495.33	1.22	0.00
10920.00	10336.00	10661.00	1.15	0.00
229.00	204.00	207.33	1.10	0.28
21.00	19.00	20.33	1.00	1.00
928.00	1154.00	1068.67	1.12	0.04
405.00	447.00	449.33	-1.03	0.67
31.00	43.00	42.33	-1.12	0.40
7.00	10.00	6.33	1.85	0.15
377.00	499.00	424.67	1.10	0.19
18.00	20.00	19.67	1.02	0.93
1333.00	1317.00	1369.00	-1.03	0.50
317.00	368.00	358.00	1.25	0.00
174.00	171.00	169.00	1.13	0.11
1.00	2.00	1.00	-1.01	1.00
2.00	6.00	4.00	-1.75	0.17
38.00	38.00	40.67	1.32	0.07
5.00	4.00	4.33	-1.32	0.59
172.00	170.00	162.67	-2.15	0.00
3.00	3.00	2.67	-2.95	0.01
699.00	719.00	702.67	-1.44	0.00
22.00	16.00	18.00	-1.48	0.04
86.00	96.00	101.33	1.13	0.24
14.00	18.00	16.00	1.01	1.00
390.00	410.00	428.00	-1.29	0.00
814.00	864.00	872.67	1.05	0.32
269.00	255.00	269.00	-1.10	0.12
865.00	1091.00	1009.67	1.20	0.00
12.00	25.00	20.00	2.17	0.00

1009.00	1018.00	1036.00	1.14	0.00
1252.00	1271.00	1331.67	-1.03	0.51
6.00	15.00	12.33	1.50	0.23
8158.00	8157.00	8260.00	-1.06	0.10
633.00	679.00	700.33	-1.01	0.88
546.00	601.00	578.33	1.08	0.10
9.00	4.00	7.67	1.19	0.65
57.00	54.00	55.00	1.26	0.07
76.00	91.00	94.33	-1.04	0.73
2906.00	3100.00	3125.67	-1.02	0.54
845.00	892.00	860.00	1.10	0.05
307.00	385.00	355.33	-1.05	0.49
1107.00	1252.00	1231.33	1.09	0.04
8781.00	8620.00	8769.00	1.49	0.00
49.00	55.00	55.67	-1.68	0.00
1025.00	976.00	1019.67	-1.21	0.00
1237.00	1211.00	1276.00	1.31	0.00
2906.00	3232.00	3134.00	1.01	0.75
107.00	108.00	112.33	1.32	0.02
2674.00	2946.00	2963.67	1.07	0.08
29.00	19.00	23.33	1.46	0.17
636.00	744.00	655.00	1.11	0.11
709.00	719.00	728.67	-1.01	0.90
378.00	436.00	428.33	1.18	0.01
4659.00	4456.00	4667.33	-1.01	0.87
2466.00	2844.00	2664.33	1.06	0.12
172.00	172.00	172.33	1.06	0.41
502.00	581.00	580.33	1.09	0.13
143.00	175.00	172.00	1.19	0.03
180.00	181.00	197.67	1.28	0.00
331.00	323.00	326.00	-1.28	0.00
4.00	2.00	2.00	-1.64	0.51
19.00	12.00	14.67	-1.38	0.16
1264.00	1415.00	1401.67	1.19	0.00
1203.00	1132.00	1213.33	-1.06	0.22
841.00	918.00	944.00	-1.35	0.00
21.00	17.00	19.33	-1.22	0.35
619.00	639.00	688.00	1.01	0.83
170.00	145.00	162.67	-1.02	0.88
3334.00	3361.00	3567.33	1.14	0.00
487.00	569.00	548.00	1.21	0.00
2117.00	2101.00	2201.00	1.01	0.72
2210.00	2152.00	2229.00	-1.19	0.00
40.00	55.00	52.00	-1.05	0.68
248.00	217.00	254.33	-1.09	0.25
4246.00	4090.00	4110.67	-1.21	0.00
119.00	124.00	135.67	-1.10	0.28
554.00	506.00	541.67	1.08	0.17

1149.00	1299.00	1279.33	-1.29	0.00
1155.00	1322.00	1241.33	-1.06	0.16
1566.00	1783.00	1656.00	1.01	0.81
1742.00	1829.00	1806.33	1.03	0.49
1849.00	1858.00	1822.33	1.05	0.31
701.00	779.00	710.00	-1.30	0.00
726.00	812.00	810.67	-1.09	0.10
16.00	23.00	17.33	-1.29	0.25
3870.00	3778.00	3886.00	-1.17	0.00
921.00	1042.00	986.33	-1.28	0.00
2186.00	2145.00	2208.67	-1.18	0.00
707.00	644.00	704.33	-1.42	0.00
15395.00	15258.00	15425.67	-1.20	0.00
431.00	385.00	415.00	-1.08	0.18
906.00	1087.00	1062.33	1.08	0.17
886.00	968.00	994.33	1.16	0.00
3355.00	3739.00	3509.33	1.08	0.06
5172.00	5261.00	5296.67	1.07	0.07
1508.00	1608.00	1593.67	1.09	0.02
801.00	800.00	783.33	-1.09	0.15
5.00	4.00	6.00	-1.34	0.45
2058.00	2327.00	2246.67	1.27	0.00
3063.00	3025.00	2969.67	-1.31	0.00
401.00	379.00	395.00	-1.79	0.00
34.00	25.00	33.67	1.51	0.02
538.00	553.00	563.67	1.16	0.00
15.00	25.00	19.67	1.00	1.00
525.00	547.00	577.33	-1.05	0.34
15.00	13.00	15.67	-1.57	0.03
2030.00	2066.00	2107.33	1.22	0.00
1570.00	1837.00	1681.67	-1.21	0.00
77.00	73.00	76.00	-1.96	0.00
1470.00	1802.00	1588.33	1.84	0.00
29.00	25.00	24.67	-1.26	0.21
31.00	19.00	24.67	-2.13	0.00
125.00	134.00	129.00	1.40	0.00
883.00	852.00	871.67	-1.65	0.00
2067.00	1974.00	2074.00	-1.54	0.00
128.00	148.00	139.67	-1.15	0.08
722.00	783.00	768.67	-1.05	0.27
46.00	42.00	47.33	-1.17	0.23
1196.00	1209.00	1159.67	1.38	0.00
16.00	21.00	19.00	-1.23	0.35
23.00	28.00	25.00	-1.12	0.56
59.00	66.00	57.33	-1.85	0.00
241.00	269.00	256.67	1.07	0.31
18.00	9.00	12.00	-1.65	0.03
4188.00	4196.00	4254.00	-2.09	0.00

62.00	83.00	78.00	1.14	0.23
607.00	656.00	618.67	1.07	0.24
817.00	904.00	894.67	1.10	0.03
340.00	342.00	377.33	1.03	0.68
12.00	19.00	16.33	-1.71	0.00
1327.00	1329.00	1335.00	-1.03	0.46
2355.00	2704.00	2614.67	1.06	0.14
275.00	420.00	336.33	-1.01	0.92
48.00	49.00	51.67	-1.11	0.41
14.00	13.00	15.00	-1.10	0.76
1441.00	1604.00	1538.33	1.19	0.00
2681.00	2834.00	2758.00	1.19	0.00
3870.00	4028.00	3997.33	1.28	0.00
7272.00	8148.00	7879.67	1.05	0.13
30.00	55.00	45.33	-1.26	0.08
2926.00	3597.00	3397.00	1.10	0.03
1576.00	1687.00	1700.33	-1.09	0.03
2376.00	2335.00	2385.67	-1.13	0.00
1876.00	2026.00	2047.67	1.13	0.00
1472.00	1546.00	1585.67	1.13	0.00
3090.00	2748.00	2910.67	-1.05	0.36
270.00	301.00	279.33	1.09	0.23
6.00	7.00	7.00	1.09	0.88
21.00	19.00	21.00	-1.16	0.46
330.00	385.00	362.33	-1.26	0.00
7.00	7.00	6.33	-1.27	0.55
395.00	391.00	412.67	1.20	0.00
133.00	127.00	128.67	-1.11	0.21
8606.00	9597.00	9308.00	1.23	0.00
3376.00	3574.00	3745.33	1.06	0.16
211.00	291.00	257.67	1.11	0.18
5604.00	6344.00	6059.67	-1.04	0.30
935.00	874.00	925.33	-5.36	0.00
4481.00	4505.00	4415.33	-1.12	0.01
2.00	6.00	3.67	-1.46	0.45
333.00	352.00	337.67	-1.24	0.00
2981.00	3278.00	3200.33	1.10	0.01
2.00	6.00	6.00	-1.01	1.00
16.00	20.00	23.67	1.88	0.00
87.00	115.00	104.00	2.79	0.00
123.00	117.00	128.67	1.63	0.00
697.00	748.00	769.33	1.02	0.78
22695.00	21655.00	21503.33	1.63	0.00
1846.00	1968.00	2030.00	1.06	0.17
1223.00	1320.00	1303.33	1.14	0.00
1817.00	2118.00	2076.33	1.29	0.00
5.00	5.00	5.33	-1.26	0.62
163.00	159.00	164.33	1.07	0.41

63267.00	61942.00	62961.67	-1.32	0.00
650.00	586.00	601.67	-1.19	0.01
756.00	716.00	770.00	1.24	0.00
7655.00	8427.00	8358.00	-1.22	0.00
124.00	115.00	128.67	1.01	0.90
230.00	258.00	266.67	1.05	0.53
1379.00	1502.00	1457.00	1.12	0.01
200.00	198.00	206.00	1.09	0.21
0.00	8.00	4.33	-2.15	0.03
4.00	8.00	6.00	-2.45	0.00
119.00	148.00	141.67	-1.04	0.68
3931.00	4212.00	4168.00	1.01	0.71
206.00	263.00	237.67	-1.24	0.00
237.00	260.00	238.67	-1.04	0.55
9591.00	10492.00	10483.33	1.18	0.00
88.00	76.00	77.67	-1.54	0.00
1122.00	1017.00	1067.33	-1.18	0.00
897.00	765.00	823.33	-1.23	0.00
1659.00	1628.00	1646.67	-1.25	0.00
962.00	975.00	988.67	1.12	0.01
717.00	792.00	784.67	-1.09	0.05
3826.00	3951.00	4000.67	-1.18	0.00
706.00	731.00	746.67	-1.04	0.37
1340.00	1347.00	1346.00	-1.03	0.48
1309.00	1453.00	1424.33	-1.04	0.28
178.00	164.00	188.33	1.24	0.01
1385.00	1380.00	1424.33	-1.11	0.01
294.00	331.00	326.67	-1.09	0.14
144.00	161.00	161.00	1.02	0.85
27.00	29.00	33.33	1.11	0.59
1572.00	1752.00	1672.67	-1.05	0.21
646.00	613.00	639.67	-1.27	0.00
8101.00	8233.00	8342.33	-1.02	0.62
2863.00	2756.00	2786.67	-1.04	0.42
1574.00	1576.00	1603.00	-1.02	0.60
2800.00	3186.00	3029.00	1.14	0.00
9386.00	11106.00	10489.67	-1.04	0.26
7850.00	8481.00	8338.00	1.06	0.09
7532.00	8040.00	7883.67	1.06	0.09
1569.00	1682.00	1698.00	1.06	0.18
1156.00	1187.00	1246.33	1.13	0.01
2660.00	3023.00	3064.33	1.60	0.00
764.00	816.00	833.00	-1.00	0.93
386.00	384.00	383.33	-1.00	0.96
543.00	552.00	558.33	-1.09	0.08
7808.00	8874.00	8362.00	1.22	0.00
168.00	212.00	195.00	1.23	0.01
14024.00	13367.00	13743.33	1.08	0.04

862.00	969.00	941.67	-1.13	0.00
1537.00	1673.00	1644.33	-1.15	0.00
88.00	105.00	98.00	1.09	0.34
250.00	230.00	252.00	1.35	0.00
5.00	3.00	3.33	-2.48	0.02
1085.00	1085.00	1097.33	1.15	0.00
233.00	211.00	226.33	-1.05	0.47
961.00	851.00	929.67	1.48	0.00
1334.00	1395.00	1431.33	1.20	0.00
299.00	353.00	309.33	-1.16	0.05
720.00	656.00	698.00	1.04	0.48
886.00	944.00	935.33	-1.08	0.06
0.00	1.00	0.67	-3.15	0.19
19.00	26.00	19.67	1.42	0.11
5219.00	6136.00	5842.67	1.50	0.00
3.00	6.00	6.00	-1.23	0.64
369.00	406.00	388.33	-1.82	0.00
3.00	5.00	4.67	2.23	0.12
646.00	728.00	672.33	-1.23	0.00
544.00	563.00	543.67	-1.04	0.52
61.00	50.00	54.67	1.03	0.84
252.00	280.00	254.67	-1.26	0.00
273.00	289.00	293.67	1.14	0.04
423.00	509.00	470.33	1.26	0.00
2318.00	2141.00	2279.67	1.21	0.00
756.00	928.00	836.00	-1.28	0.00
939.00	902.00	955.67	-1.19	0.00
3226.00	3228.00	3332.33	-1.19	0.00
1341.00	1341.00	1417.00	-1.11	0.01
1650.00	1767.00	1782.33	1.05	0.25
397.00	433.00	418.33	-1.07	0.28
1616.00	1599.00	1665.67	-1.18	0.00
53.00	72.00	60.67	-1.19	0.13
2910.00	2810.00	2952.67	-1.47	0.00
0.00	0.00	0.33	-9.11	0.00
16.00	11.00	15.00	1.43	0.15
1169.00	1185.00	1221.33	-1.08	0.05
1.00	6.00	2.67	-1.62	0.39
37.00	39.00	35.67	1.12	0.51
1130.00	1305.00	1237.33	1.11	0.05
255.00	263.00	253.00	-1.02	0.75
2477.00	2651.00	2774.33	-1.01	0.76
237.00	209.00	235.67	-1.07	0.34
8415.00	8312.00	8180.33	-1.26	0.00
2218.00	2272.00	2250.33	-1.89	0.00
1313.00	1482.00	1433.00	1.12	0.01
253.00	321.00	300.00	-1.29	0.00
2897.00	2937.00	2995.33	-1.20	0.00

507.00	518.00	527.67	-1.03	0.57
294.00	234.00	280.00	-1.82	0.00
1285.00	1304.00	1343.00	1.16	0.00
16.00	12.00	13.67	-1.90	0.00
11.00	11.00	14.67	1.20	0.52
37.00	27.00	39.00	1.28	0.12
1479.00	1560.00	1535.67	1.04	0.30
1342.00	1377.00	1431.33	1.04	0.36
4178.00	4323.00	4316.67	-1.16	0.00
4347.00	4350.00	4406.67	-1.20	0.00
825.00	751.00	786.00	-1.53	0.00
658.00	689.00	658.67	-1.09	0.12
92.00	108.00	107.33	-1.17	0.09
714.00	719.00	712.33	-1.24	0.00
7.00	5.00	7.67	-1.19	0.68
238.00	232.00	228.00	1.08	0.33
1580.00	1767.00	1678.33	1.64	0.00
834.00	1066.00	977.67	-1.15	0.01
908.00	698.00	801.67	-1.25	0.00
1180.00	1047.00	1113.00	-1.23	0.00
924.00	951.00	981.67	-1.11	0.01
910.00	765.00	831.00	1.10	0.13
6.00	3.00	4.00	-1.50	0.37
5794.00	5830.00	6062.33	-1.11	0.00
46.00	32.00	39.33	-1.71	0.00
55.00	53.00	50.00	-1.06	0.68
21.00	34.00	23.67	-1.51	0.02
692.00	811.00	745.33	1.18	0.00
20.00	20.00	20.33	-1.29	0.19
1145.00	1242.00	1286.00	1.14	0.01
236.00	256.00	257.00	1.75	0.00
181.00	241.00	207.67	1.19	0.03
110.00	116.00	122.67	1.14	0.13
3355.00	3198.00	3377.67	1.13	0.01
1219.00	1289.00	1314.67	1.03	0.45
443.00	434.00	453.67	1.32	0.00
10157.00	10619.00	10880.67	1.03	0.45
658.00	803.00	734.33	1.07	0.23
540.00	679.00	618.33	1.06	0.35
418.00	409.00	405.33	-1.29	0.00
595.00	631.00	605.33	-1.09	0.09
492.00	549.00	544.00	1.15	0.01
362.00	387.00	382.00	-1.05	0.36
688.00	765.00	722.33	1.09	0.13
36.00	42.00	41.00	1.70	0.00
33.00	23.00	26.00	-1.05	0.83
1307.00	1660.00	1527.00	-1.00	0.99
667.00	710.00	690.67	-1.33	0.00

428.00	437.00	476.00	1.06	0.38
648.00	712.00	717.00	1.14	0.01
342.00	361.00	343.33	-1.38	0.00
420.00	452.00	459.00	1.50	0.00
179.00	145.00	180.00	-2.42	0.00
5.00	5.00	4.33	-2.00	0.06
4669.00	4575.00	4779.67	1.04	0.33
63.00	85.00	72.67	1.03	0.83
455.00	450.00	439.00	-1.14	0.02
635.00	647.00	636.00	-1.08	0.11
1382.00	1593.00	1482.00	-1.04	0.42
1608.00	1702.00	1713.67	1.00	0.95
16.00	15.00	14.00	1.18	0.51
69.00	104.00	84.33	1.10	0.43
306.00	355.00	334.67	1.07	0.23
1461.00	1454.00	1425.33	-1.03	0.60
1948.00	1876.00	1942.00	-1.24	0.00
732.00	743.00	770.00	-1.17	0.00
1539.00	1581.00	1545.33	-1.41	0.00
45.00	52.00	48.00	-1.30	0.03
2825.00	2521.00	2652.67	-1.15	0.00
657.00	591.00	621.00	-1.17	0.00
138.00	133.00	131.33	-1.89	0.00
3.00	1.00	2.00	1.17	1.00
588.00	716.00	689.67	1.05	0.44
556.00	576.00	609.00	-1.07	0.18
340.00	414.00	390.00	-1.10	0.08
15.00	13.00	12.33	1.65	0.08
639.00	640.00	649.67	-1.01	0.89
647.00	673.00	693.00	-1.06	0.34
743.00	934.00	878.00	-1.02	0.74
650.00	591.00	642.67	1.08	0.19
453.00	469.00	452.67	1.07	0.26
609.00	747.00	662.33	-1.07	0.37
303.00	313.00	316.33	1.08	0.25
645.00	819.00	763.00	1.43	0.00
481.00	537.00	519.00	1.22	0.00
10.00	10.00	10.67	-1.23	0.50
1051.00	1132.00	1063.67	-1.20	0.00
1946.00	1859.00	1929.33	1.04	0.35
68.00	57.00	61.33	-1.06	0.61
724.00	908.00	821.33	-1.04	0.55
2.00	3.00	3.67	-1.01	1.00
2537.00	2840.00	2750.00	1.07	0.11
10.00	12.00	11.67	-1.36	0.24
2455.00	2729.00	2721.00	1.14	0.00
24.00	30.00	24.67	1.07	0.75
839.00	829.00	840.67	1.25	0.00

3.00	7.00	4.00	-1.01	1.00
480.00	457.00	486.33	1.07	0.22
903.00	997.00	948.67	1.02	0.71
4283.00	4913.00	4755.67	1.17	0.00
479.00	464.00	494.00	-1.14	0.02
2.00	2.00	4.33	-1.09	1.00
201.00	259.00	237.33	-1.09	0.25
1538.00	1506.00	1555.00	-1.14	0.00
669.00	759.00	696.33	1.05	0.37
1131.00	1008.00	1124.00	-1.07	0.14
2113.00	2157.00	2079.33	1.00	0.94
1416.00	1443.00	1461.67	1.06	0.11
1736.00	1745.00	1830.33	-1.05	0.25
634.00	672.00	671.33	-1.31	0.00
5.00	3.00	3.00	-1.77	0.24
26.00	43.00	34.67	1.13	0.50
501.00	509.00	533.67	1.01	0.94
4376.00	4959.00	4668.33	-1.05	0.19
80.00	72.00	79.33	1.39	0.01
47.00	50.00	44.33	-1.61	0.00
80.00	55.00	62.67	-1.12	0.42
51.00	48.00	49.67	-1.28	0.04
193.00	192.00	189.67	-1.07	0.36
555.00	543.00	565.00	1.11	0.05
466.00	401.00	464.67	-1.20	0.00
25.00	29.00	27.33	-1.01	0.94
75.00	93.00	85.33	-1.29	0.01
200.00	196.00	209.00	1.02	0.78
734.00	896.00	804.67	1.44	0.00
1602.00	1630.00	1612.00	-1.20	0.00
306.00	325.00	306.67	-1.85	0.00
0.00	1.00	3.00	-1.35	0.69
618.00	745.00	693.33	-1.09	0.11
1157.00	1187.00	1196.00	-1.03	0.50
828.00	864.00	864.67	-1.09	0.06
289.00	347.00	329.33	1.28	0.00
232.00	256.00	267.33	1.06	0.40
46.00	37.00	37.00	-1.61	0.00
1388.00	1489.00	1482.00	1.11	0.01
2488.00	2590.00	2568.33	-1.04	0.30
14500.00	15681.00	15504.67	-1.13	0.00
10295.00	11143.00	11142.00	1.25	0.00
1008.00	941.00	960.00	-1.12	0.02
21.00	25.00	23.00	-1.41	0.04
7.00	7.00	5.33	-2.13	0.02
4.00	0.00	1.67	-3.28	0.02
346.00	345.00	361.00	-1.48	0.00
3.00	2.00	2.67	1.12	1.00

60.00	33.00	51.33	1.14	0.45
5.00	3.00	2.67	-1.86	0.22
7.00	9.00	6.00	-2.17	0.01
464.00	456.00	477.33	-1.47	0.00
88.00	81.00	90.00	-1.10	0.34
148.00	176.00	161.33	-1.48	0.00
2343.00	2297.00	2301.67	-1.15	0.00
111.00	126.00	117.33	-1.60	0.00
622.00	631.00	607.00	-1.15	0.02
2.00	2.00	1.33	-2.63	0.13
455.00	475.00	512.00	1.20	0.00
385.00	467.00	442.33	1.18	0.01
1200.00	1303.00	1292.00	1.19	0.00
945.00	1102.00	1027.00	1.08	0.10
959.00	1088.00	1072.33	1.27	0.00
155.00	162.00	172.00	-1.16	0.08
570.00	578.00	585.67	1.27	0.00
416.00	538.00	502.67	1.06	0.31
405.00	431.00	478.00	1.09	0.27
398.00	403.00	398.67	1.19	0.00
124.00	177.00	153.67	1.26	0.02
73.00	75.00	86.67	1.44	0.00
99.00	93.00	95.33	1.00	1.00
928.00	889.00	949.67	1.05	0.37
164.00	176.00	173.67	-1.05	0.49
1535.00	1774.00	1659.00	1.19	0.00
506.00	519.00	511.67	1.08	0.22
388.00	354.00	388.33	-1.45	0.00
263.00	254.00	287.00	1.03	0.70
625.00	705.00	700.33	-1.42	0.00
652.00	762.00	731.33	-1.02	0.76
917.00	1049.00	1039.00	-1.14	0.00
8917.00	9883.00	9999.00	1.12	0.00
335.00	344.00	329.00	-1.00	1.00
1477.00	1712.00	1602.00	1.21	0.00
339.00	420.00	400.33	1.18	0.01
50.00	69.00	58.00	-1.03	0.85
298.00	259.00	289.33	1.10	0.15
2292.00	2637.00	2485.00	1.07	0.10
966.00	1005.00	1019.67	-1.38	0.00
1034.00	1103.00	1092.67	1.04	0.32
2187.00	2210.00	2372.67	-1.09	0.05
296.00	346.00	339.00	1.20	0.01
302.00	360.00	345.33	1.31	0.00
89.00	108.00	103.67	-1.16	0.16
357.00	370.00	362.67	1.13	0.03
733.00	808.00	802.33	1.04	0.40
217.00	199.00	215.00	2.66	0.00

1129.00	1266.00	1220.00	1.03	0.52
4179.00	4499.00	4511.33	1.18	0.00
218.00	238.00	243.00	1.02	0.79
116.00	97.00	107.00	-1.06	0.54
14.00	21.00	22.33	-1.17	0.40
18.00	28.00	25.33	1.44	0.06
5.00	3.00	4.00	-1.18	0.85
136.00	123.00	140.33	-1.24	0.01
395.00	417.00	433.00	-1.06	0.26
1090.00	1130.00	1094.33	-1.02	0.70
7.00	15.00	10.33	-1.05	1.00
774.00	708.00	764.00	-1.49	0.00
14.00	13.00	14.33	-1.34	0.21
271.00	272.00	278.00	-1.27	0.00
1703.00	1538.00	1649.67	-1.01	0.88
66.00	81.00	72.00	1.20	0.11
49.00	62.00	57.00	-1.04	0.77
2766.00	2979.00	2976.33	1.15	0.00
98.00	71.00	87.33	1.15	0.26
2.00	5.00	6.33	-1.59	0.20
28.00	23.00	25.67	1.09	0.63
2026.00	2203.00	2199.33	-1.20	0.00
93.00	59.00	73.67	-1.39	0.01
2.00	1.00	1.67	-1.77	0.43
50.00	75.00	58.33	1.42	0.01
623.00	709.00	651.67	-1.27	0.00
4.00	10.00	8.00	-1.80	0.03
758.00	886.00	839.00	-1.03	0.49
1.00	1.00	1.00	-3.41	0.06
849.00	819.00	871.33	-1.55	0.00
211.00	181.00	199.33	-1.15	0.10
365.00	366.00	365.67	-1.18	0.01
473.00	458.00	451.00	-1.06	0.40
72.00	48.00	53.33	-1.16	0.38
2014.00	2006.00	2014.33	1.09	0.04
1049.00	1092.00	1100.33	-1.04	0.33
2225.00	2409.00	2278.00	-1.03	0.51
2463.00	2752.00	2637.33	1.13	0.00
1286.00	1253.00	1286.00	-1.17	0.00
141.00	167.00	162.00	-1.02	0.85
1595.00	1622.00	1610.33	-1.12	0.01
733.00	841.00	823.00	-1.00	0.97
730.00	781.00	800.67	-1.00	0.96
911.00	977.00	901.67	1.13	0.04
1072.00	1050.00	1097.67	-1.05	0.31
1450.00	1589.00	1541.67	1.07	0.12
831.00	953.00	937.67	1.03	0.58
2502.00	2451.00	2540.67	-1.01	0.81

789.00	895.00	853.67	1.07	0.11
1181.00	1289.00	1223.33	-1.07	0.16
630.00	606.00	654.00	-1.17	0.01
2514.00	2345.00	2478.33	-1.08	0.07
835.00	702.00	755.00	1.08	0.20
797.00	809.00	825.67	1.14	0.00
3.00	0.00	1.33	-2.17	0.27
1347.00	1282.00	1337.33	1.04	0.36
569.00	539.00	567.00	1.00	1.00
1720.00	1685.00	1674.00	-1.05	0.31
278.00	276.00	273.00	1.02	0.73
414.00	425.00	428.33	-1.34	0.00
10.00	10.00	10.33	2.01	0.03
1604.00	1635.00	1666.00	-1.01	0.81
4.00	6.00	6.00	-1.57	0.19
1338.00	1338.00	1330.67	1.14	0.01
985.00	884.00	986.67	-1.14	0.01
307.00	347.00	314.00	1.27	0.00
1086.00	1204.00	1236.33	1.07	0.15
688.00	714.00	718.00	-1.16	0.00
361.00	391.00	380.33	1.02	0.70
455.00	396.00	440.33	-1.98	0.00
1716.00	1811.00	1790.33	1.08	0.07
688.00	699.00	730.00	1.03	0.54
17198.00	17744.00	17298.00	-1.18	0.00
4861.00	4912.00	4856.67	-1.09	0.02
22576.00	25475.00	24137.33	-1.08	0.05
77.00	87.00	81.33	1.40	0.00
7.00	11.00	9.33	-2.09	0.00
1110.00	1340.00	1274.33	1.01	0.84
1097.00	1077.00	1117.33	-1.23	0.00
577.00	673.00	636.67	-1.15	0.01
3270.00	3245.00	3318.67	-1.03	0.47
1485.00	1646.00	1587.33	1.06	0.22
538.00	545.00	557.33	1.02	0.68
2927.00	2810.00	2965.33	-1.20	0.00
5407.00	5302.00	5245.00	-1.13	0.01
785.00	810.00	808.33	1.07	0.14
212.00	257.00	257.33	1.30	0.00
4.00	5.00	5.00	-1.08	1.00
2185.00	2248.00	2188.67	-1.02	0.60
450.00	465.00	447.33	1.09	0.11
1644.00	1413.00	1567.00	-1.03	0.55
12949.00	14027.00	14128.00	-1.13	0.00
1733.00	1632.00	1664.00	-1.32	0.00
118.00	151.00	135.67	-1.32	0.00
1188.00	1104.00	1195.00	1.08	0.09
1202.00	1204.00	1279.67	1.02	0.62

1.00	6.00	2.33	1.36	0.78
6946.00	7298.00	7217.33	-1.08	0.03
1031.00	1092.00	1057.00	1.05	0.24
844.00	829.00	822.67	-1.38	0.00
16.00	16.00	14.33	1.51	0.11
601.00	706.00	646.00	-1.07	0.26
107.00	107.00	107.67	1.15	0.14
610.00	524.00	571.00	-1.26	0.00
813.00	900.00	881.33	1.08	0.08
185.00	187.00	183.33	-1.10	0.18
104.00	126.00	116.33	-1.59	0.00
323.00	286.00	335.67	-1.14	0.08
63.00	44.00	55.67	2.19	0.00
713.00	877.00	829.67	-1.03	0.50
6727.00	6427.00	6842.67	-1.07	0.10
141.00	109.00	127.67	-1.26	0.01
49.00	52.00	52.33	1.09	0.52
11.00	16.00	10.00	1.02	1.00
3058.00	3406.00	3080.33	-1.12	0.08
568.00	543.00	558.33	-1.03	0.59
21.00	32.00	24.33	1.12	0.53
1086.00	1137.00	1146.33	-1.03	0.54
3250.00	3318.00	3412.33	-1.04	0.28
1213.00	1454.00	1298.67	1.02	0.70
2414.00	2474.00	2504.00	-1.12	0.00
2.00	4.00	5.00	-1.08	1.00
4278.00	4398.00	4408.67	-1.08	0.03
200.00	214.00	208.67	-1.37	0.00
949.00	915.00	933.00	-1.33	0.00
1570.00	1477.00	1581.67	1.00	0.96
519.00	476.00	502.00	-1.14	0.02
8680.00	8323.00	8568.33	-1.15	0.00
3162.00	3534.00	3229.00	-1.53	0.00
281.00	289.00	287.67	1.14	0.02
935.00	1052.00	956.67	-1.38	0.00
2.00	7.00	3.33	-1.01	1.00
24136.00	22831.00	23780.67	-1.07	0.12
57.00	53.00	47.67	1.20	0.25
37.00	50.00	44.67	-1.44	0.00
1995.00	2001.00	2042.00	-1.24	0.00
305.00	341.00	328.00	-1.06	0.34
21.00	29.00	25.00	-1.34	0.08
33.00	41.00	35.33	-1.93	0.00
1.00	1.00	2.00	-1.97	0.25
1366.00	1377.00	1384.00	-1.08	0.05
7.00	2.00	4.00	-2.32	0.02
0.00	2.00	0.67	-4.00	0.07
245.00	263.00	257.33	-1.36	0.00

1524.00	1515.00	1548.67	-1.33	0.00
24.00	25.00	24.00	1.27	0.20
424.00	465.00	434.00	1.10	0.10
1002.00	994.00	1037.00	-2.04	0.00
648.00	631.00	646.00	-1.12	0.02
605.00	594.00	595.67	-1.17	0.00
3701.00	3768.00	3774.33	-1.04	0.31
1065.00	1094.00	1090.67	-1.23	0.00
56.00	52.00	53.67	1.16	0.26
468.00	495.00	495.33	-1.04	0.49
0.00	2.00	1.33	-2.17	0.27
49.00	47.00	50.33	-1.39	0.01
1566.00	1699.00	1648.00	-1.03	0.52
329.00	378.00	360.67	1.40	0.00
1567.00	1636.00	1608.33	1.00	0.91
1022.00	1120.00	1084.33	1.28	0.00
326.00	399.00	363.00	-1.09	0.16
5.00	1.00	3.67	-1.90	0.12
10.00	24.00	13.67	1.61	0.15
2791.00	2746.00	2679.00	-1.25	0.00
14.00	5.00	10.33	1.22	0.52
115.00	139.00	135.00	-1.29	0.00
897.00	985.00	974.33	1.25	0.00
1112.00	1152.00	1156.00	1.29	0.00
1497.00	1441.00	1481.33	-1.45	0.00
7.00	6.00	6.00	1.18	0.73
858.00	835.00	839.00	1.18	0.00
1641.00	1992.00	1768.67	1.01	0.93
1101.00	1159.00	1120.33	1.03	0.47
364.00	372.00	370.67	1.06	0.28
823.00	1127.00	1017.33	1.04	0.52
3372.00	3880.00	3696.67	-1.00	0.95
1123.00	1279.00	1213.00	1.01	0.91
585.00	644.00	618.67	-1.05	0.36
91.00	69.00	77.00	-1.21	0.18
19.00	23.00	18.67	-1.07	0.86
247.00	266.00	256.00	1.17	0.02
1001.00	1121.00	1116.67	1.12	0.02
3887.00	4160.00	4100.00	-1.82	0.00
1097.00	1229.00	1165.00	-1.04	0.43
1804.00	2062.00	1906.67	-1.04	0.38
16.00	27.00	22.33	1.27	0.26
97.00	102.00	100.00	-1.61	0.00
151.00	136.00	141.33	1.17	0.06
1263.00	1275.00	1299.67	1.15	0.00
746.00	756.00	784.33	1.11	0.02
1572.00	1731.00	1735.67	1.26	0.00
1021.00	1135.00	1078.33	1.25	0.00

849.00	943.00	916.33	-1.02	0.66
4626.00	4473.00	4502.00	1.04	0.33
2096.00	2339.00	2218.00	1.10	0.01
1502.00	1740.00	1615.67	1.04	0.42
805.00	887.00	858.00	1.07	0.16
3125.00	2929.00	2929.33	-1.05	0.34
2562.00	2495.00	2549.67	-1.14	0.00
3319.00	3474.00	3531.67	1.11	0.00
2739.00	2811.00	2844.33	1.07	0.06
134.00	117.00	134.00	-1.45	0.00
1923.00	2117.00	2145.33	1.12	0.01
4323.00	4395.00	4367.67	-1.01	0.78
1209.00	1360.00	1279.00	1.18	0.00
2696.00	3190.00	2972.67	1.11	0.01
5441.00	6517.00	5999.33	1.27	0.00
982.00	1183.00	1051.33	1.02	0.73
6.00	7.00	8.00	-1.10	0.89
1612.00	1835.00	1759.67	1.01	0.85
2887.00	3266.00	3010.33	1.01	0.84
62.00	73.00	68.67	1.41	0.01
97.00	100.00	110.67	1.49	0.00
1613.00	1500.00	1521.67	-1.24	0.00
3736.00	4117.00	3844.00	-1.05	0.27
56.00	61.00	53.67	-1.47	0.00
38.00	36.00	33.00	1.06	0.74
42.00	46.00	38.67	-1.71	0.00
6.00	9.00	7.67	-1.75	0.05
139.00	161.00	174.00	1.01	0.92
3960.00	3890.00	3958.33	-1.08	0.05
2205.00	2082.00	2203.00	1.19	0.00
2286.00	2267.00	2327.00	-1.11	0.00
8.00	5.00	5.33	1.13	0.86
1037.00	1049.00	1066.33	-1.00	0.97
1688.00	1807.00	1817.67	1.16	0.00
108.00	109.00	113.67	1.16	0.11
94.00	155.00	126.33	1.01	0.94
559.00	653.00	628.67	-1.18	0.00
1025.00	1122.00	1091.33	1.39	0.00
125.00	134.00	139.33	-1.05	0.51
1309.00	1554.00	1486.00	1.18	0.00
9.00	3.00	6.00	-1.18	0.76
401.00	462.00	441.00	1.18	0.00
296.00	335.00	329.00	1.10	0.11
26.00	37.00	29.00	1.45	0.04
96.00	97.00	94.00	1.00	1.00
141.00	195.00	193.33	-1.11	0.34
971.00	958.00	1016.00	1.21	0.00
133.00	150.00	148.00	1.12	0.15

47.00	54.00	51.00	1.16	0.26
1154.00	1378.00	1281.33	1.09	0.06
209.00	221.00	209.00	1.05	0.50
506.00	510.00	537.33	1.05	0.31
40.00	49.00	42.67	-1.08	0.62
502.00	544.00	559.67	-1.41	0.00
1211.00	1195.00	1244.33	-1.09	0.07
229.00	215.00	226.33	1.25	0.00
0.00	0.00	0.67	-8.26	0.00
1431.00	1418.00	1457.00	-1.15	0.00
161.00	198.00	185.67	1.04	0.60
3515.00	4020.00	3944.00	1.38	0.00
7424.00	7405.00	7547.67	-1.06	0.13
1644.00	1773.00	1733.67	1.12	0.00
510.00	597.00	575.67	-1.00	0.98
1093.00	1015.00	1046.33	-1.08	0.13
9496.00	10794.00	10430.33	1.25	0.00
647.00	642.00	659.33	1.10	0.04
476.00	466.00	510.67	1.09	0.21
449.00	467.00	454.67	-1.07	0.29
975.00	1035.00	1045.67	-1.06	0.15
21.00	34.00	35.00	1.04	0.90
1035.00	1139.00	1091.67	-1.10	0.03
0.00	1.00	0.33	-4.68	0.13
52.00	57.00	62.00	-1.30	0.02
1501.00	1629.00	1575.67	1.04	0.33
173.00	207.00	188.33	1.47	0.00
557.00	544.00	551.00	1.09	0.11
551.00	553.00	594.00	1.02	0.73
81.00	70.00	72.67	1.15	0.24
1729.00	1917.00	1867.33	-1.06	0.13
657.00	744.00	727.67	1.05	0.29
688.00	706.00	712.67	1.00	1.00
1205.00	1229.00	1302.00	-1.15	0.00
149.00	182.00	181.00	1.06	0.52
350.00	434.00	416.67	1.04	0.53
199.00	224.00	219.00	-1.42	0.00
66.00	58.00	72.33	1.39	0.01
21.00	23.00	24.67	-1.61	0.00
7385.00	7108.00	7179.33	-1.05	0.29
773.00	946.00	898.00	1.12	0.02
5300.00	5929.00	6006.00	1.06	0.17
286.00	300.00	327.00	-1.08	0.37
416.00	489.00	485.00	-1.03	0.56
735.00	881.00	864.00	-1.04	0.46
1302.00	1347.00	1406.67	1.21	0.00
2103.00	2123.00	2306.33	-1.00	0.96
1733.00	1878.00	1952.67	1.18	0.00

220.00	244.00	243.67	-1.02	0.81
423.00	481.00	485.33	-1.09	0.14
479.00	526.00	507.33	-1.03	0.56
186.00	227.00	216.67	-1.06	0.38
6.00	6.00	8.33	-1.09	0.89
4345.00	5013.00	5006.67	-1.10	0.03
1384.00	1594.00	1463.00	-1.10	0.04
1116.00	1197.00	1267.67	1.05	0.31
1780.00	2011.00	2074.00	1.21	0.00
2892.00	2876.00	3041.33	-1.00	1.00
2068.00	2061.00	2216.33	1.05	0.25
54.00	37.00	51.00	-1.00	1.00
355.00	456.00	422.00	-1.00	0.96
159.00	144.00	155.00	1.09	0.36
34.00	52.00	44.67	1.03	0.86
28.00	23.00	32.33	1.21	0.31
1.00	4.00	2.33	-2.25	0.10
4.00	3.00	4.33	1.07	1.00
4.00	10.00	10.67	1.01	1.00
5.00	9.00	11.33	-1.19	0.51
4.00	4.00	4.00	1.30	0.67
46.00	56.00	50.33	-1.09	0.49
2.00	0.00	0.67	-1.86	0.69
196.00	217.00	207.33	-1.04	0.55
13.00	9.00	9.67	1.89	0.05
16.00	25.00	23.00	-1.28	0.17
5.00	10.00	6.00	1.11	0.87
3.00	3.00	3.00	-1.55	0.41
25.00	51.00	39.00	-1.07	0.69
96.00	117.00	112.67	-1.16	0.08
16.00	11.00	17.00	-1.43	0.07
38.00	40.00	40.33	1.02	0.95
7.00	6.00	6.00	1.26	0.61
1.00	2.00	3.00	-2.09	0.10
10.00	8.00	11.67	-1.39	0.21
118.00	139.00	138.67	-1.05	0.57
105.00	130.00	115.33	1.02	0.82
52.00	69.00	62.00	1.52	0.00
221.00	319.00	274.33	1.09	0.29
26.00	26.00	24.67	1.01	0.94
22.00	37.00	37.00	-1.45	0.01
43.00	37.00	39.33	-1.27	0.07
46.00	40.00	51.00	-1.18	0.21
3.00	4.00	6.33	-1.96	0.03
38.00	35.00	35.67	-1.82	0.00
32.00	33.00	31.33	-1.07	0.68
42.00	51.00	50.33	-1.14	0.31
4.00	7.00	7.33	-1.24	0.58

30.00	36.00	33.00	-1.01	0.95
17.00	18.00	20.00	-1.13	0.55
2.00	4.00	2.00	-1.17	1.00
5.00	2.00	7.33	-1.84	0.06
16.00	28.00	20.67	-1.24	0.27
46.00	37.00	42.33	-1.19	0.20
56.00	48.00	47.67	-1.28	0.06
28.00	15.00	22.33	-1.12	0.64
151.00	184.00	172.00	-1.11	0.22
59.00	74.00	72.00	-1.14	0.21
49.00	57.00	57.00	-1.12	0.34
51.00	39.00	52.00	-1.18	0.20
24.00	37.00	32.33	-1.06	0.74
17.00	43.00	36.00	1.09	0.71
103.00	87.00	103.00	-1.13	0.23
37.00	39.00	37.33	-1.18	0.25
22.00	19.00	19.67	-1.05	0.87
256.00	282.00	290.00	-1.05	0.44
33.00	45.00	38.33	-1.06	0.72
50.00	46.00	48.33	-1.04	0.79
52.00	68.00	60.33	1.02	0.89
10.00	6.00	7.00	-1.77	0.06
41.00	41.00	41.67	1.20	0.24
212.00	217.00	230.33	-1.11	0.10
142.00	151.00	149.33	1.09	0.28
9.00	14.00	10.00	1.34	0.35
32.00	33.00	33.67	1.07	0.70
17.00	8.00	14.00	-1.08	0.84
3.00	3.00	2.33	-1.01	1.00
14.00	7.00	8.67	-1.13	0.80
190.00	205.00	212.33	1.05	0.52
5.00	3.00	5.33	-1.82	0.08
12.00	11.00	8.33	1.37	0.42
59.00	66.00	61.00	-1.36	0.01
70.00	68.00	65.00	-1.06	0.66
64.00	75.00	77.00	-1.12	0.27
36.00	28.00	40.00	1.01	0.96
45.00	64.00	60.00	1.05	0.74
37.00	44.00	42.33	1.00	1.00
61.00	64.00	78.67	-1.25	0.12
7.00	7.00	7.67	-1.27	0.50
79.00	111.00	97.00	-1.21	0.04
3.00	3.00	2.00	-1.65	0.46
0.00	2.00	1.33	-1.48	0.76
2.00	7.00	4.00	-1.58	0.30
8.00	5.00	6.67	-1.26	0.57
9.00	9.00	8.00	-1.22	0.60
2.00	3.00	2.33	-1.56	0.49

7.00	3.00	5.00	-1.21	0.73
17.00	5.00	10.00	-1.11	0.81
34.00	18.00	22.67	-1.73	0.01
13.00	13.00	13.00	2.11	0.01
0.00	3.00	1.67	-1.39	0.78
59.00	44.00	49.00	-1.42	0.01
0.00	4.00	2.00	-1.33	0.79
10.00	5.00	7.67	1.73	0.14
6.00	5.00	7.00	-1.30	0.48
63.00	41.00	49.00	-1.11	0.51
9.00	16.00	10.00	-1.08	0.90
49.00	85.00	69.33	-1.01	0.94
87.00	96.00	98.33	-1.04	0.64
5.00	5.00	5.67	-1.25	0.64
11.00	9.00	16.67	-1.18	0.53
6.00	11.00	9.33	-1.73	0.03
18.00	22.00	19.00	-1.14	0.60
23.00	34.00	27.67	-1.05	0.77
17.00	13.00	17.67	-1.16	0.49
22.00	16.00	22.33	1.16	0.49
8.00	5.00	7.33	-1.74	0.06
32.00	39.00	40.00	-1.17	0.28
16.00	11.00	14.00	-1.04	1.00
24.00	27.00	29.67	-1.13	0.46
14.00	12.00	11.67	-1.38	0.20
6.00	5.00	5.33	-1.45	0.35
22.00	32.00	24.00	-1.07	0.77
51.00	74.00	63.00	-1.01	0.96
13.00	9.00	14.33	-1.32	0.21
23.00	21.00	23.00	-1.22	0.24
164.00	170.00	173.33	1.04	0.66
16.00	19.00	19.33	1.27	0.29
15.00	8.00	12.67	-1.28	0.35
59.00	53.00	60.33	-1.01	0.93
32.00	24.00	29.33	-1.11	0.57
5.00	7.00	9.67	1.35	0.42
52.00	71.00	69.33	1.04	0.77
21.00	24.00	22.00	-1.03	1.00
35.00	33.00	37.00	1.20	0.25
233.00	200.00	216.33	-1.04	0.64
25.00	15.00	20.00	-1.16	0.50
29.00	34.00	34.00	-1.10	0.52
26.00	29.00	29.67	-1.25	0.14
190.00	215.00	228.33	1.18	0.05
26.00	32.00	29.00	-1.09	0.57
6.00	8.00	6.67	1.62	0.22
5.00	5.00	9.00	-1.17	0.62
17.00	34.00	30.00	1.19	0.39

0.00	6.00	3.00	-1.01	1.00
16.00	7.00	13.67	1.01	1.00
3.00	3.00	2.67	-4.28	0.00
28.00	32.00	30.67	1.01	1.00
147.00	146.00	149.67	1.14	0.10
148.00	162.00	141.00	-1.02	0.89
158.00	222.00	190.33	-1.14	0.09
89.00	93.00	89.33	-1.34	0.00
24.00	33.00	28.33	1.37	0.08
170.00	207.00	177.67	-1.05	0.61
78.00	88.00	87.00	-1.15	0.13
45.00	39.00	39.67	1.19	0.25
3.00	8.00	5.33	1.21	0.72
85.00	86.00	86.33	-1.07	0.50
3.00	0.00	1.33	-2.40	0.19
27.00	30.00	31.33	-1.24	0.16
8.00	9.00	11.00	1.08	0.81
1.00	5.00	2.67	-1.01	1.00
27.00	25.00	30.67	-1.16	0.40
26.00	38.00	30.00	1.15	0.42
3.00	2.00	4.33	-1.01	1.00
32.00	38.00	40.67	-1.27	0.07
46.00	80.00	66.00	1.01	0.95
1.00	2.00	2.00	-1.33	0.79
3.00	3.00	2.67	-2.10	0.11
37.00	44.00	39.33	1.05	0.76
11.00	15.00	14.33	1.28	0.32
26.00	32.00	26.67	1.05	0.82
11.00	6.00	9.33	-1.19	0.62
0.00	6.00	2.67	-1.14	1.00
29.00	24.00	29.00	1.10	0.66
10.00	11.00	10.00	1.47	0.21
1.00	5.00	5.67	-1.49	0.31
3.00	9.00	5.00	-1.28	0.62
62.00	39.00	48.67	1.05	0.80
1.00	1.00	3.67	-1.02	1.00
36.00	41.00	37.67	-1.22	0.15
53.00	63.00	63.67	-1.22	0.06
77.00	96.00	91.33	1.05	0.69
30.00	40.00	32.00	1.28	0.13
386.00	427.00	423.00	-1.05	0.37
18.00	26.00	24.00	-1.11	0.54
79.00	67.00	75.00	-1.24	0.04
115.00	124.00	112.67	-1.09	0.37
2.00	0.00	0.67	-2.71	0.30
35.00	55.00	38.33	1.26	0.20
15.00	13.00	18.67	1.04	1.00
6.00	7.00	9.67	1.88	0.07

34.00	43.00	41.00	1.12	0.47
6.00	6.00	8.33	-1.37	0.31
14.00	26.00	17.67	-1.01	1.00
22.00	19.00	17.00	-1.31	0.20
223.00	279.00	267.67	-1.11	0.14
22.00	21.00	21.33	1.07	0.73
1.00	2.00	1.33	-3.10	0.06
19.00	28.00	22.33	1.12	0.55
556.00	537.00	569.33	1.06	0.31
505.00	563.00	552.00	-1.11	0.04
238.00	270.00	271.33	-1.47	0.00
7580.00	7445.00	7477.67	-1.11	0.00
246.00	284.00	280.00	1.30	0.00
531.00	640.00	625.00	1.26	0.00
319.00	329.00	328.00	1.27	0.00
1182.00	1247.00	1271.33	1.11	0.01
624.00	804.00	718.00	1.00	0.96
3719.00	4133.00	4033.00	1.02	0.61
1984.00	2122.00	2201.00	1.14	0.00
987.00	1086.00	1102.67	1.11	0.02
6072.00	6378.00	6591.33	1.21	0.00
1658.00	1957.00	1936.33	1.17	0.00
2136.00	2351.00	2436.67	1.26	0.00
1502.00	1680.00	1688.00	1.40	0.00
1959.00	2259.00	2320.67	1.17	0.00
1793.00	2045.00	2012.00	1.19	0.00
1423.00	1615.00	1662.33	1.34	0.00
1142.00	1419.00	1379.33	1.27	0.00
1028.00	1164.00	1217.00	1.23	0.00
130.00	137.00	138.33	1.14	0.11
129.00	123.00	120.00	1.09	0.37
983.00	1048.00	1018.33	1.11	0.01
1935.00	2225.00	2224.67	1.27	0.00
605.00	658.00	643.00	1.18	0.00
1797.00	2123.00	2014.00	1.09	0.04
2217.00	2528.00	2412.67	-1.12	0.00
47.00	80.00	61.00	1.09	0.51
364.00	357.00	368.00	-1.06	0.31
1116.00	1138.00	1185.67	1.16	0.00
1132.00	1194.00	1164.00	1.04	0.31
2544.00	2746.00	2693.67	1.02	0.66
250.00	339.00	303.00	1.18	0.03
1782.00	2025.00	2027.67	1.10	0.03
361.00	407.00	391.67	1.17	0.01
1653.00	1603.00	1596.67	-1.16	0.00
1534.00	1770.00	1740.67	1.18	0.00
427.00	420.00	418.33	-1.09	0.14
5.00	0.00	2.00	1.44	0.76

342.00	343.00	347.67	1.29	0.00
526.00	578.00	560.00	1.26	0.00
1116.00	1156.00	1179.00	-1.13	0.00
373.00	408.00	382.00	-1.36	0.00
12.00	14.00	10.67	-1.20	0.57
7.00	5.00	4.67	1.15	0.85
1723.00	1902.00	1930.33	1.05	0.26
405.00	389.00	409.00	-1.19	0.01
39.00	45.00	48.33	-1.22	0.11
1146.00	1155.00	1157.33	-1.39	0.00
374.00	417.00	406.00	1.31	0.00
2708.00	3224.00	3101.00	1.12	0.01
1610.00	1808.00	1752.33	1.05	0.25
528.00	586.00	582.00	1.20	0.00
1047.00	988.00	1058.33	-1.04	0.37
1438.00	1599.00	1539.33	-1.15	0.00
2633.00	2895.00	2790.00	1.03	0.42
148.00	186.00	164.33	-1.06	0.45
9.00	14.00	11.33	1.39	0.25
583.00	646.00	629.00	1.20	0.00
67.00	64.00	63.33	-1.04	0.80
1005.00	1032.00	1045.33	-1.07	0.11
1104.00	1276.00	1221.33	-1.08	0.05
604.00	586.00	582.00	-1.04	0.50
318.00	361.00	338.67	-1.18	0.00
56.00	75.00	64.00	-1.12	0.34
2058.00	2399.00	2332.33	1.21	0.00
654.00	663.00	668.67	1.23	0.00
889.00	644.00	719.67	-1.35	0.00
376.00	396.00	365.67	-1.39	0.00
6.00	5.00	4.67	-1.15	0.86
6932.00	7387.00	7022.33	-1.10	0.02
6.00	1.00	3.67	-1.73	0.21
67.00	76.00	81.67	-1.44	0.00
830.00	782.00	835.67	1.04	0.41
5.00	11.00	7.33	-1.51	0.19
239.00	256.00	257.00	-1.70	0.00
519.00	596.00	562.33	-1.38	0.00
1199.00	1176.00	1195.33	1.27	0.00
798.00	793.00	790.00	-1.04	0.46
1152.00	1342.00	1239.67	-1.07	0.15
760.00	772.00	763.33	-1.06	0.31
0.00	4.00	1.67	1.57	0.74
337.00	397.00	368.00	1.25	0.00
1798.00	1723.00	1789.33	1.17	0.00
95.00	107.00	105.00	-1.37	0.00
8.00	13.00	11.67	-1.21	0.51
8.00	10.00	10.67	-1.42	0.18

10.00	15.00	12.33	1.72	0.06
6.00	6.00	5.00	1.34	0.57
512.00	587.00	576.00	-1.04	0.55
8.00	11.00	10.33	-1.62	0.09
4.00	10.00	7.00	-2.15	0.01
35.00	32.00	36.33	-1.50	0.00
2091.00	2287.00	2272.33	1.42	0.00
95.00	98.00	102.67	1.46	0.00
1986.00	1973.00	1974.67	-1.14	0.00
1957.00	1940.00	1988.67	-1.07	0.09
282.00	349.00	326.00	-1.02	0.74
103.00	143.00	126.00	-1.32	0.00
700.00	781.00	766.33	-1.06	0.20
447.00	404.00	448.67	-1.04	0.46
25.00	25.00	29.67	-1.10	0.58
1621.00	1796.00	1688.00	1.12	0.02
344.00	400.00	378.67	1.01	0.86
12.00	4.00	9.00	1.11	0.79
9.00	10.00	8.67	1.42	0.30
19.00	24.00	20.00	1.44	0.08
110.00	148.00	149.33	1.17	0.13
64.00	88.00	80.00	-1.13	0.22
216.00	272.00	236.67	1.43	0.00
292.00	357.00	342.33	1.13	0.05
125.00	104.00	123.00	-1.80	0.00
1258.00	1380.00	1362.67	1.03	0.46
0.00	3.00	3.33	-1.21	0.84
2302.00	2611.00	2560.00	-1.07	0.12
28.00	34.00	35.00	-1.23	0.16
3514.00	3732.00	3597.00	-1.11	0.01
1.00	3.00	2.33	-1.29	0.81
233.00	291.00	286.33	1.11	0.18
26.00	34.00	31.33	1.20	0.31
5446.00	5657.00	5678.33	-1.07	0.14
544.00	584.00	552.00	1.42	0.00
7.00	9.00	8.00	1.80	0.10
963.00	1017.00	955.33	-1.21	0.00
50.00	53.00	55.00	-1.09	0.49
528.00	560.00	576.33	1.09	0.10
1749.00	1662.00	1798.67	-1.23	0.00
67.00	58.00	70.00	-1.08	0.52
139.00	172.00	167.33	-1.15	0.10
233.00	289.00	276.67	1.43	0.00
165.00	180.00	183.67	-1.20	0.02
12.00	6.00	10.67	1.84	0.05
22.00	16.00	19.67	1.35	0.16
211.00	223.00	213.00	1.01	0.92
488.00	549.00	535.67	-1.04	0.39

69.00	65.00	75.33	1.12	0.34
25.00	37.00	32.00	-1.80	0.00
178.00	203.00	190.00	1.03	0.68
8.00	15.00	11.33	1.29	0.38
5056.00	5614.00	5565.67	1.26	0.00
2011.00	2180.00	2145.00	-1.01	0.77
123.00	126.00	125.33	1.23	0.01
5.00	8.00	7.33	1.65	0.18
1963.00	2069.00	2165.67	1.15	0.00
562.00	633.00	631.00	1.15	0.03
3796.00	4057.00	3907.67	1.29	0.00
1150.00	1459.00	1325.33	1.05	0.30
4.00	4.00	4.67	-1.58	0.25
1.00	2.00	2.00	-1.97	0.24
88.00	75.00	90.67	1.11	0.33
917.00	959.00	987.67	-1.08	0.07
7.00	15.00	9.67	-1.33	0.40
130.00	161.00	152.00	-1.09	0.34
2292.00	2221.00	2307.67	-1.41	0.00
2873.00	2586.00	2611.00	-1.50	0.00
128.00	166.00	150.33	1.05	0.64
1792.00	1797.00	1792.67	-1.14	0.00
1550.00	1933.00	1797.67	1.04	0.46
25.00	25.00	26.67	-1.03	0.88
1715.00	1904.00	1896.00	1.07	0.07
2969.00	2949.00	3104.33	-1.10	0.02
2632.00	2757.00	2868.33	-1.29	0.00
608.00	578.00	626.67	-1.01	0.87
404.00	376.00	396.33	-1.13	0.03
921.00	965.00	949.00	-1.06	0.17
1706.00	1864.00	1819.67	-1.06	0.16
26.00	26.00	31.33	-1.00	1.00
735.00	841.00	814.33	-1.09	0.05
608.00	683.00	619.00	1.48	0.00
5.00	1.00	2.67	-2.10	0.11
0.00	1.00	0.67	-2.72	0.29
30.00	19.00	23.67	-1.34	0.10
2562.00	2860.00	2757.67	1.04	0.37
323.00	369.00	352.33	-1.10	0.09
1617.00	1450.00	1623.33	-1.04	0.50
4.00	2.00	4.33	1.07	1.00
14.00	17.00	17.33	-2.12	0.00
1916.00	1908.00	1997.00	-1.32	0.00
168.00	159.00	155.33	-2.44	0.00
3.00	4.00	3.67	-1.99	0.09
6.00	4.00	5.33	-1.51	0.28
1132.00	1270.00	1247.00	1.12	0.01
662.00	763.00	709.00	1.22	0.00

125.00	114.00	126.67	1.55	0.00
1015.00	1073.00	1038.00	1.25	0.00
1378.00	1421.00	1484.67	-1.17	0.00
961.00	988.00	1016.33	1.11	0.01
567.00	569.00	597.67	1.44	0.00
1912.00	2308.00	2052.67	-1.26	0.00
2022.00	2058.00	2076.67	1.18	0.00
287.00	324.00	306.00	1.03	0.65
298.00	263.00	288.67	1.21	0.00
102.00	124.00	113.33	1.49	0.00
5155.00	5045.00	5245.33	1.13	0.00
3006.00	2938.00	3136.67	-1.17	0.00
79.00	68.00	65.67	-1.60	0.00
594.00	569.00	594.33	1.05	0.32
162.00	251.00	206.33	-1.22	0.02
302.00	349.00	329.67	1.32	0.00
823.00	787.00	826.00	-1.10	0.05
240.00	321.00	288.33	-1.01	0.88
580.00	565.00	607.67	-1.14	0.01
5010.00	5073.00	4970.00	-1.15	0.00
0.00	1.00	1.00	-3.11	0.10
3173.00	3131.00	3080.67	-1.42	0.00
3837.00	3601.00	3709.00	-1.03	0.51
2.00	2.00	1.33	-4.02	0.01
155.00	196.00	180.33	-1.14	0.07
2039.00	2273.00	2166.00	1.02	0.66
1215.00	1107.00	1134.67	-1.11	0.05
476.00	399.00	431.00	-1.12	0.08
422.00	555.00	481.33	1.31	0.00
596.00	561.00	569.00	-1.01	0.82
40.00	40.00	41.33	1.11	0.50
581.00	620.00	615.00	-1.12	0.04
1689.00	1770.00	1767.67	1.06	0.14
239.00	292.00	277.00	-1.10	0.14
6501.00	5973.00	6343.33	-1.31	0.00
828.00	917.00	952.33	1.03	0.61
405.00	444.00	427.33	1.07	0.20
1948.00	1819.00	1981.00	-1.14	0.00
3740.00	3114.00	3323.33	-1.34	0.00
446.00	457.00	448.67	1.03	0.56
274.00	243.00	266.00	1.58	0.00
9.00	9.00	8.33	-1.41	0.26
3649.00	3841.00	3763.33	1.01	0.88
2436.00	2322.00	2346.33	1.02	0.62
2866.00	3267.00	3135.00	1.02	0.69
587.00	715.00	646.00	1.08	0.22
1768.00	1703.00	1789.33	1.08	0.05
533.00	483.00	514.33	1.11	0.06

2325.00	2141.00	2225.67	1.01	0.79
1489.00	1393.00	1498.00	-1.23	0.00
83.00	92.00	84.67	-1.26	0.01
57.00	49.00	54.67	-1.15	0.32
30.00	28.00	26.33	-1.34	0.12
6.00	9.00	7.00	-1.72	0.07
8920.00	8939.00	9190.33	1.36	0.00
1394.00	1671.00	1654.67	1.23	0.00
1729.00	1935.00	1922.67	1.18	0.00
1835.00	2017.00	2060.67	1.06	0.16
173.00	171.00	165.33	-1.45	0.00
657.00	736.00	711.00	1.03	0.57
1496.00	1714.00	1655.00	-1.01	0.88
1750.00	1822.00	1854.00	1.05	0.21
2197.00	2227.00	2250.67	1.08	0.07
3004.00	3621.00	3440.67	1.25	0.00
3940.00	4261.00	4158.67	1.14	0.00
825.00	845.00	840.67	1.14	0.01
3124.00	3107.00	3222.33	-1.04	0.30
2563.00	2405.00	2559.33	1.06	0.20
7067.00	7419.00	7272.33	1.21	0.00
976.00	1038.00	1043.33	-1.27	0.00
158.00	201.00	178.33	1.03	0.73
331.00	353.00	364.33	1.07	0.22
2677.00	2775.00	2803.00	-1.16	0.00
25399.00	21471.00	23187.33	-1.40	0.00
6.00	5.00	5.67	-1.48	0.29
17.00	22.00	21.33	1.03	0.93
3019.00	3222.00	3222.67	1.03	0.42
9229.00	10300.00	9944.00	1.15	0.00
3030.00	3738.00	3476.67	1.10	0.02
6652.00	8364.00	7692.67	1.07	0.10
7201.00	8450.00	8079.00	1.07	0.06
5926.00	6615.00	6569.67	1.16	0.00
2523.00	2663.00	2654.33	-1.01	0.87
4740.00	5389.00	5246.33	-1.07	0.05
7211.00	8537.00	8200.00	-1.04	0.31
3482.00	3872.00	3880.67	1.10	0.02
700.00	789.00	763.33	1.03	0.52
2766.00	3015.00	2996.67	1.13	0.00
1886.00	1784.00	1893.33	1.19	0.00
1807.00	2272.00	2103.00	-1.16	0.00
1101.00	1227.00	1214.33	-1.05	0.20
262.00	320.00	316.33	1.09	0.24
3278.00	3844.00	3702.67	1.18	0.00
622.00	711.00	704.67	1.22	0.00
113.00	101.00	120.67	1.12	0.24
1135.00	1157.00	1186.67	1.04	0.32

1058.00	1017.00	1056.67	1.06	0.17
10.00	11.00	11.67	-1.19	0.58
29.00	29.00	28.67	-1.45	0.01
3622.00	3958.00	3633.33	1.11	0.08
1608.00	1497.00	1539.33	-1.23	0.00
565.00	588.00	587.67	-1.15	0.00
173.00	158.00	182.33	-1.16	0.05
742.00	733.00	804.00	1.15	0.01
8.00	5.00	8.67	-1.28	0.45
0.00	3.00	1.33	-3.56	0.02
6911.00	7249.00	7258.67	-1.03	0.39
5969.00	6139.00	6348.00	1.16	0.00
27839.00	27802.00	28188.67	1.39	0.00
2284.00	2200.00	2342.00	1.07	0.17
79.00	93.00	86.00	1.15	0.16
6002.00	6336.00	6274.67	1.17	0.00
685.00	742.00	761.67	1.19	0.00
46.00	42.00	43.67	-1.13	0.38
1673.00	1725.00	1823.33	1.16	0.00
9.00	10.00	9.33	-1.51	0.13
570.00	622.00	602.33	1.23	0.00
3183.00	3359.00	3369.33	1.17	0.00
46.00	46.00	56.33	1.45	0.01
312.00	348.00	357.67	-1.06	0.30
47.00	41.00	53.33	1.01	1.00
16.00	14.00	13.33	2.06	0.01
2133.00	1915.00	1997.33	-1.48	0.00
536.00	598.00	568.67	-1.02	0.65
509.00	549.00	574.67	-1.08	0.14
279.00	270.00	272.67	1.02	0.78
29.00	38.00	32.33	-1.12	0.48
126.00	102.00	113.33	-1.13	0.19
510.00	589.00	554.33	-1.66	0.00
53.00	44.00	52.00	-1.68	0.00
548.00	570.00	556.67	-1.20	0.00
446.00	420.00	433.33	1.14	0.02
14.00	13.00	14.67	1.14	0.59
2.00	1.00	1.33	-3.33	0.03
191.00	175.00	198.33	-1.40	0.00
533.00	480.00	536.67	-1.01	0.84
907.00	944.00	999.33	1.10	0.08
874.00	812.00	855.33	-1.15	0.00
43.00	38.00	38.67	-1.27	0.08
1188.00	1217.00	1221.67	1.06	0.17
8.00	5.00	6.67	-1.96	0.02
222.00	271.00	253.67	1.08	0.29
71.00	108.00	89.67	1.04	0.77
24.00	28.00	28.33	-1.58	0.00

5.00	1.00	2.67	-1.74	0.29
128.00	148.00	131.33	-1.17	0.08
73.00	69.00	70.67	-1.07	0.54
1027.00	1023.00	1020.33	-1.05	0.25
2213.00	2468.00	2311.67	1.02	0.70
254.00	278.00	278.33	1.10	0.16
37.00	19.00	25.33	-1.13	0.59
211.00	194.00	190.67	-1.22	0.04
102.00	77.00	84.67	-1.12	0.34
180.00	176.00	186.67	-1.01	0.96
5.00	6.00	6.33	1.10	0.87
677.00	710.00	723.00	-1.19	0.00
980.00	1084.00	1039.67	1.10	0.03
646.00	757.00	702.33	1.07	0.19
75.00	87.00	80.00	-1.15	0.19
1067.00	1077.00	1097.00	1.10	0.04
846.00	942.00	947.33	-1.01	0.87
95.00	93.00	102.33	1.28	0.02
182.00	164.00	202.33	-1.02	0.83
2301.00	2465.00	2449.33	-1.13	0.00
1570.00	1431.00	1540.00	-1.05	0.28
1245.00	1339.00	1290.00	-1.02	0.69
398.00	423.00	424.00	1.27	0.00
42.00	51.00	46.00	-1.20	0.16
3326.00	3625.00	3572.67	1.10	0.00
210.00	223.00	212.67	-1.11	0.12
504.00	476.00	480.00	-1.01	0.92
538.00	504.00	496.67	-1.50	0.00
1495.00	1624.00	1596.00	1.05	0.26
1049.00	1104.00	1084.67	-1.22	0.00
1114.00	1086.00	1118.67	-1.01	0.87
23.00	17.00	20.67	-1.71	0.00
168.00	162.00	162.67	1.04	0.64
1508.00	1564.00	1550.33	-1.10	0.01
3510.00	3552.00	3628.00	-1.18	0.00
2687.00	2850.00	2811.00	1.11	0.00
666.00	696.00	688.00	1.04	0.41
15.00	13.00	13.00	1.07	0.83
3499.00	3291.00	3437.00	-1.28	0.00
12099.00	11723.00	12419.67	-1.33	0.00
1739.00	2027.00	1965.33	1.55	0.00
1383.00	1425.00	1453.00	1.62	0.00
1022.00	1134.00	1082.67	1.25	0.00
4158.00	4142.00	4144.00	-1.25	0.00
666.00	778.00	742.00	1.24	0.00
1072.00	1014.00	1038.67	-1.20	0.00
365.00	370.00	382.33	1.02	0.78
9563.00	10370.00	10224.00	1.07	0.05

790.00	744.00	788.67	-1.46	0.00
1359.00	1484.00	1442.00	1.09	0.03
712.00	729.00	770.00	1.08	0.13
588.00	681.00	677.33	1.01	0.87
1630.00	1847.00	1775.67	1.14	0.00
398.00	496.00	453.67	-1.23	0.00
1867.00	1927.00	1971.00	1.03	0.48
2499.00	2593.00	2707.00	1.04	0.30
420.00	494.00	444.67	1.20	0.01
565.00	659.00	610.67	1.20	0.00
50.00	46.00	48.00	-1.13	0.39
40.00	42.00	40.67	1.11	0.49
179.00	190.00	189.33	-1.13	0.10
1402.00	1392.00	1414.00	-1.09	0.03
1147.00	1228.00	1226.33	-1.21	0.00
1003.00	1105.00	1116.33	-1.11	0.02
597.00	681.00	638.33	1.24	0.00
1198.00	1217.00	1230.67	1.02	0.62
1613.00	1698.00	1613.00	-1.16	0.00
969.00	990.00	1032.67	-1.35	0.00
10.00	12.00	14.00	1.53	0.13
6.00	5.00	8.67	-1.29	0.46
3023.00	3469.00	3434.33	1.55	0.00
1329.00	1375.00	1398.00	1.38	0.00
1614.00	1461.00	1562.00	1.22	0.00
328.00	276.00	311.67	-1.01	0.90
4489.00	4687.00	4933.67	1.26	0.00
77.00	105.00	89.67	-1.16	0.13
13.00	5.00	7.67	2.03	0.07
295.00	247.00	280.33	-1.22	0.03
3.00	2.00	3.00	1.10	1.00
709.00	698.00	776.33	1.23	0.00
832.00	1006.00	987.00	-1.01	0.89
584.00	633.00	631.33	-1.08	0.11
3443.00	3867.00	3783.33	-1.15	0.00
675.00	683.00	723.67	1.28	0.00
1002.00	979.00	989.67	-1.16	0.00
1207.00	1376.00	1327.33	-1.81	0.00
880.00	950.00	865.67	1.00	0.98
582.00	652.00	625.33	1.06	0.21
1902.00	1835.00	1947.67	1.02	0.67
218.00	193.00	203.67	-1.18	0.02
4887.00	5312.00	5221.00	-1.12	0.00
7517.00	8194.00	7900.00	-1.01	0.73
1845.00	1953.00	1979.00	-1.03	0.37
858.00	966.00	999.00	1.12	0.05
31.00	17.00	24.67	-1.33	0.12
786.00	821.00	810.67	1.15	0.00

3846.00	4503.00	4270.33	-1.11	0.01
31.00	43.00	37.67	-1.09	0.55
425.00	403.00	408.00	-1.24	0.00
464.00	465.00	475.67	1.09	0.12
31.00	23.00	24.67	-1.28	0.15
1.00	6.00	3.00	1.45	0.62
859.00	890.00	922.00	-1.64	0.00
480.00	520.00	517.00	-1.61	0.00
790.00	947.00	882.00	1.06	0.19
1160.00	1225.00	1197.00	-1.09	0.08
815.00	778.00	851.00	-1.09	0.09
467.00	478.00	494.00	-1.12	0.02
797.00	867.00	848.00	-1.10	0.05
853.00	1025.00	979.33	1.14	0.01
345.00	398.00	392.67	1.21	0.00
1355.00	1361.00	1409.00	-1.09	0.02
4.00	4.00	3.33	-1.60	0.33
720.00	886.00	841.00	1.07	0.20
194.00	209.00	212.00	1.09	0.23
720.00	730.00	707.00	-1.12	0.06
270.00	303.00	294.33	-1.02	0.70
279.00	307.00	290.33	4.05	0.00
379.00	508.00	442.33	1.14	0.05
915.00	943.00	976.33	1.10	0.03
2181.00	2578.00	2452.00	1.18	0.00
425.00	512.00	473.00	1.05	0.39
1039.00	1191.00	1176.67	1.20	0.00
1865.00	2032.00	1938.67	-1.03	0.53
104.00	137.00	119.33	-1.37	0.00
1631.00	1739.00	1677.67	-1.13	0.00
1085.00	1134.00	1185.67	1.06	0.18
574.00	501.00	530.33	-1.22	0.00
112.00	118.00	122.00	1.22	0.03
738.00	712.00	755.33	-1.00	0.96
3417.00	3459.00	3461.33	-1.21	0.00
814.00	910.00	906.33	1.29	0.00
207.00	200.00	197.67	1.22	0.01
3.00	3.00	5.33	1.05	1.00
61.00	58.00	60.33	-3.08	0.00
685.00	649.00	683.00	-1.09	0.11
7478.00	6964.00	7476.67	1.09	0.05
1511.00	1724.00	1735.33	1.27	0.00
26.00	27.00	28.67	1.23	0.24
1616.00	1844.00	1835.00	1.24	0.00
1932.00	2217.00	2163.00	1.25	0.00
3766.00	4049.00	3956.33	-1.14	0.00
1579.00	1768.00	1703.00	-1.14	0.01
302.00	354.00	344.67	1.05	0.52

5177.00	5487.00	5350.67	1.12	0.00
1168.00	1231.00	1226.00	1.00	0.95
539.00	651.00	639.67	1.01	0.82
926.00	1035.00	1035.00	1.03	0.47
3012.00	2848.00	2933.33	-1.12	0.01
3755.00	3873.00	3835.33	-1.12	0.00
2338.00	2579.00	2576.33	-1.14	0.00
1137.00	1344.00	1289.33	1.04	0.33
574.00	514.00	577.33	1.04	0.62
402.00	378.00	397.33	1.25	0.00
8213.00	8329.00	8341.00	-1.12	0.01
1327.00	1399.00	1425.33	1.14	0.00
112.00	103.00	115.00	-1.09	0.37
6.00	6.00	6.33	-1.17	0.76
95.00	103.00	93.33	-1.08	0.48
488.00	475.00	489.67	-1.10	0.06
437.00	473.00	481.67	1.14	0.02
479.00	520.00	505.00	1.22	0.00
764.00	811.00	805.00	-1.06	0.24
237.00	218.00	238.33	-1.10	0.15
1091.00	1302.00	1146.33	1.08	0.20
176.00	225.00	213.67	-1.01	0.88
894.00	860.00	871.67	-1.34	0.00
86.00	106.00	105.33	1.34	0.00
121.00	101.00	108.33	1.18	0.09
936.00	966.00	952.00	1.02	0.62
554.00	565.00	575.67	-1.05	0.34
45.00	62.00	53.33	-1.73	0.00
424.00	487.00	475.33	1.54	0.00
1126.00	1138.00	1149.00	1.19	0.00
721.00	863.00	833.00	1.16	0.00
37.00	37.00	41.00	1.30	0.10
151.00	147.00	155.67	-1.11	0.15
1175.00	1381.00	1318.00	1.15	0.00
35.00	44.00	43.33	1.24	0.15
4.00	5.00	4.33	2.07	0.17
28.00	40.00	38.00	1.20	0.26
37.00	40.00	42.67	-1.02	0.91
4.00	7.00	4.33	-1.01	1.00
854.00	810.00	865.33	-1.04	0.43
124.00	104.00	119.00	1.13	0.19
917.00	1021.00	1026.33	1.14	0.01
2644.00	2803.00	2826.00	-1.02	0.53
333.00	409.00	391.67	1.20	0.00
7686.00	8932.00	8278.67	1.13	0.00
1315.00	1424.00	1362.00	-1.09	0.05
424.00	414.00	417.67	1.14	0.02
149.00	127.00	129.00	-1.62	0.00

43.00	42.00	47.33	1.11	0.46
90.00	76.00	77.33	-1.82	0.00
185.00	199.00	201.33	-1.52	0.00
1291.00	1286.00	1294.00	1.11	0.06
10.00	7.00	9.00	1.02	1.00
332.00	331.00	323.00	-1.12	0.09
1420.00	1397.00	1431.00	-1.46	0.00
793.00	806.00	772.33	1.08	0.17
349.00	385.00	377.00	-1.14	0.02
1046.00	862.00	946.00	-1.70	0.00
17.00	10.00	11.33	-1.48	0.13
0.00	1.00	0.67	-5.28	0.01
10.00	2.00	6.33	1.43	0.39
35.00	35.00	41.33	-1.06	0.69
2771.00	3283.00	3114.33	1.11	0.02
11.00	6.00	9.67	1.14	0.69
644.00	766.00	723.00	1.04	0.51
515.00	472.00	506.00	1.01	0.87
894.00	890.00	889.00	1.01	0.82
113.00	109.00	108.67	1.03	0.79
46.00	77.00	61.33	1.26	0.09
1.00	2.00	1.00	-2.21	0.35
5.00	9.00	8.00	1.38	0.36
163.00	161.00	155.00	1.52	0.00
20.00	21.00	27.33	2.67	0.00
667.00	821.00	776.33	1.14	0.02
159.00	188.00	180.67	-1.36	0.00
117.00	119.00	119.33	1.02	0.84
2474.00	2434.00	2501.00	-1.27	0.00
3409.00	3453.00	3538.00	1.05	0.13
1553.00	1338.00	1423.67	-1.34	0.00
542.00	602.00	578.67	-1.06	0.23
1608.00	1710.00	1668.00	1.02	0.57
397.00	471.00	439.00	-1.06	0.30
2096.00	2091.00	2040.67	-1.62	0.00
1131.00	1136.00	1162.00	-1.08	0.05
8335.00	9195.00	8989.00	1.09	0.02
562.00	643.00	634.00	1.10	0.07
6.00	7.00	5.67	-4.57	0.00
283.00	304.00	301.33	1.09	0.17
10.00	17.00	13.67	1.04	0.92
525.00	457.00	504.33	-1.07	0.28
2423.00	2565.00	2605.00	1.13	0.00
1905.00	1908.00	1845.67	-1.20	0.00
724.00	793.00	801.67	1.18	0.00
560.00	699.00	659.67	1.04	0.52
392.00	512.00	478.67	-1.04	0.53
243.00	258.00	245.67	1.32	0.00

16.00	12.00	13.67	1.04	0.92
358.00	397.00	388.00	1.04	0.48
32.00	24.00	31.33	-1.15	0.38
460.00	500.00	518.00	1.24	0.00
2013.00	2150.00	2180.00	-1.11	0.01
1789.00	2169.00	2024.33	-1.01	0.86
3.00	2.00	2.33	-1.70	0.37
1560.00	1611.00	1588.33	-1.06	0.18
278.00	276.00	275.33	1.01	0.94
146.00	117.00	124.33	1.01	0.96
599.00	634.00	641.00	1.08	0.12
240.00	267.00	253.33	1.22	0.00
629.00	658.00	682.33	-1.03	0.50
1497.00	1570.00	1580.67	-1.04	0.25
412.00	461.00	449.67	1.06	0.25
1780.00	2056.00	2002.33	-1.01	0.85
509.00	603.00	558.33	-1.00	0.99
1.00	1.00	0.67	-7.02	0.01
670.00	826.00	780.67	1.18	0.00
43.00	47.00	50.33	-1.50	0.00
4116.00	4347.00	4435.67	-1.00	0.90
688.00	769.00	792.00	1.24	0.00
2.00	6.00	5.00	-1.01	1.00
2852.00	3210.00	3189.33	1.13	0.00
201.00	245.00	236.00	1.04	0.57
655.00	756.00	695.00	-1.28	0.00
1037.00	924.00	978.33	-1.64	0.00
347.00	381.00	376.00	-1.02	0.79
464.00	406.00	444.33	1.01	0.84
701.00	804.00	781.00	1.11	0.02
1726.00	1841.00	1726.00	-1.04	0.53
1570.00	1467.00	1524.33	-1.32	0.00
1852.00	2169.00	2055.67	1.33	0.00
1.00	0.00	0.67	-2.29	0.46
4.00	5.00	6.00	-1.73	0.10
20.00	26.00	23.00	-1.19	0.35
223.00	212.00	215.33	-1.11	0.13
342.00	415.00	368.33	-1.02	0.73
54.00	65.00	65.33	-1.12	0.31
1562.00	1678.00	1622.00	-1.11	0.03
814.00	772.00	805.00	1.03	0.54
4032.00	4492.00	4302.00	-1.02	0.53
12.00	10.00	11.00	1.35	0.30
9601.00	11045.00	10799.33	1.02	0.59
1140.00	1148.00	1174.67	-1.07	0.08
210.00	216.00	214.33	-1.14	0.04
1.00	2.00	4.33	1.56	0.43
5.00	17.00	11.33	-1.49	0.14

100.00	113.00	108.00	-1.10	0.27
203.00	259.00	238.33	1.17	0.03
924.00	1165.00	1140.33	1.18	0.01
946.00	866.00	918.67	-1.15	0.00
335.00	366.00	358.33	1.36	0.00
2600.00	2900.00	2812.33	1.05	0.23
181.00	170.00	183.33	1.14	0.09
1056.00	1037.00	1124.67	-1.14	0.01
1328.00	1368.00	1382.33	-1.10	0.02
271.00	260.00	277.67	1.10	0.26
1133.00	1154.00	1169.67	-1.10	0.02
268.00	274.00	278.33	-1.06	0.37
983.00	896.00	963.67	-1.02	0.65
1790.00	1682.00	1744.67	1.03	0.54
784.00	871.00	810.33	1.04	0.44
1959.00	1746.00	1833.33	-1.30	0.00
445.00	434.00	490.67	-1.07	0.32
60.00	77.00	67.00	1.35	0.01
233.00	210.00	225.67	1.03	0.66
1245.00	1321.00	1295.00	-1.29	0.00
487.00	470.00	474.00	1.06	0.28
433.00	382.00	424.67	1.22	0.00
2683.00	3019.00	2868.00	1.56	0.00
713.00	670.00	719.33	-1.16	0.00
411.00	455.00	439.33	1.11	0.06
1005.00	953.00	974.67	-1.51	0.00
177.00	209.00	197.67	1.09	0.23
446.00	539.00	501.00	1.11	0.06
284.00	372.00	319.67	1.02	0.75
4.00	4.00	3.00	1.11	1.00
967.00	1113.00	1031.00	1.16	0.00
2412.00	2252.00	2294.33	-1.08	0.13
197.00	219.00	210.00	-1.09	0.20
458.00	501.00	491.33	-1.38	0.00
341.00	377.00	354.00	-1.06	0.39
724.00	767.00	751.33	-1.07	0.14
4157.00	3969.00	4091.33	-1.12	0.00
1627.00	1852.00	1862.67	1.31	0.00
1011.00	1201.00	1160.00	1.17	0.00
510.00	551.00	575.33	1.07	0.19
253.00	267.00	264.33	1.08	0.25
950.00	938.00	912.67	-1.08	0.17
711.00	858.00	819.00	1.18	0.00
577.00	662.00	620.00	-1.04	0.46
468.00	522.00	496.00	-1.20	0.00
1014.00	1059.00	1065.33	1.14	0.00
135.00	110.00	125.33	-1.15	0.12
674.00	640.00	698.00	-1.18	0.00

2323.00	2716.00	2575.00	1.12	0.01
1213.00	1338.00	1330.00	-1.02	0.69
1107.00	1100.00	1129.67	1.03	0.49
408.00	466.00	456.67	1.17	0.00
607.00	728.00	695.67	1.24	0.00
1021.00	1003.00	1046.67	-1.11	0.01
3043.00	3518.00	3441.00	-1.02	0.54
797.00	971.00	883.67	-1.00	0.96
71.00	69.00	73.33	-1.21	0.06
342.00	403.00	372.00	1.00	0.96
72.00	85.00	84.67	-1.17	0.17
2099.00	2160.00	2174.33	-1.38	0.00
23.00	40.00	39.00	-1.19	0.29
22.00	31.00	25.00	-1.01	1.00
197.00	181.00	198.67	-1.14	0.06
1206.00	1075.00	1121.67	-1.24	0.00
62.00	70.00	69.67	1.40	0.00
521.00	618.00	567.00	-1.01	0.81
38.00	57.00	48.67	1.24	0.12
5.00	2.00	3.33	-4.43	0.00
3707.00	3983.00	3957.67	1.17	0.00
353.00	328.00	364.33	-1.30	0.00
6.00	7.00	8.33	1.23	0.56
363.00	408.00	387.33	-1.19	0.00
334.00	399.00	377.00	-1.08	0.17
2063.00	2412.00	2361.33	1.08	0.08
1228.00	1510.00	1460.67	1.13	0.01
72.00	81.00	80.00	-1.00	1.00
407.00	451.00	419.33	-1.12	0.08
1288.00	1628.00	1533.00	1.21	0.00
1289.00	1377.00	1435.67	1.09	0.06
1756.00	1907.00	1886.67	-1.06	0.11
59.00	77.00	77.33	-1.18	0.14
3040.00	3523.00	3339.33	1.05	0.21
1983.00	1993.00	1954.00	1.06	0.30
10.00	16.00	12.33	1.04	0.91
2386.00	2384.00	2442.67	-1.09	0.02
2594.00	2474.00	2600.67	1.10	0.01
331.00	411.00	353.67	-1.00	1.00
39.00	26.00	34.67	1.13	0.50
13.00	11.00	12.00	1.36	0.27
482.00	551.00	517.33	-1.02	0.71
914.00	1022.00	1031.00	1.07	0.19
913.00	1001.00	983.00	1.06	0.19
836.00	825.00	891.67	-1.26	0.00
10.00	4.00	9.67	3.93	0.00
88.00	100.00	108.67	1.88	0.00
4178.00	4222.00	4209.67	-1.02	0.54

8.00	2.00	4.33	-1.24	0.72
6402.00	7045.00	7059.00	1.24	0.00
8.00	9.00	9.33	1.44	0.25
303.00	269.00	281.67	-1.04	0.58
76.00	92.00	80.67	-1.31	0.02
361.00	370.00	371.00	-1.15	0.01
214.00	217.00	222.67	1.14	0.05
894.00	928.00	935.00	-1.08	0.08
218.00	241.00	217.33	1.02	0.81
1714.00	1658.00	1706.00	-1.03	0.49
480.00	542.00	522.00	-1.04	0.42
737.00	845.00	808.00	-1.16	0.00
1016.00	1156.00	1150.00	1.10	0.04
306.00	346.00	335.33	1.05	0.50
17.00	21.00	22.33	-1.81	0.00
100.00	111.00	112.67	-1.07	0.44
283.00	305.00	302.00	1.31	0.00
123.00	135.00	128.33	-1.05	0.57
2120.00	2261.00	2150.67	-1.32	0.00
83.00	86.00	88.33	1.05	0.66
244.00	252.00	272.33	-1.30	0.00
874.00	868.00	886.00	1.07	0.21
83.00	93.00	89.67	-1.33	0.00
1042.00	917.00	1002.00	-1.05	0.30
725.00	645.00	683.33	-1.30	0.00
2252.00	2196.00	2359.67	1.07	0.27
7.00	7.00	12.33	-1.59	0.12
10.00	2.00	4.67	1.06	1.00
115.00	137.00	125.67	1.13	0.15
180.00	231.00	211.00	-1.03	0.63
47.00	37.00	43.33	-1.22	0.14
1042.00	1096.00	1100.33	1.02	0.65
43.00	50.00	53.67	-1.15	0.27
218.00	259.00	245.67	-1.60	0.00
680.00	731.00	692.00	-1.49	0.00
1169.00	1190.00	1170.67	-1.06	0.18
205.00	214.00	196.33	-1.27	0.01
1246.00	1220.00	1222.00	-1.20	0.00
656.00	742.00	691.33	1.18	0.00
709.00	779.00	757.33	1.09	0.04
1206.00	1304.00	1286.67	1.20	0.00
265.00	266.00	270.67	1.16	0.02
251.00	269.00	279.00	-1.27	0.00
393.00	419.00	431.00	-1.18	0.00
76.00	59.00	65.00	-1.44	0.00
52.00	51.00	53.67	-1.15	0.25
250.00	289.00	277.33	-1.10	0.12
1068.00	1209.00	1113.00	-1.16	0.00

1570.00	1609.00	1606.67	1.16	0.00
2.00	4.00	3.67	-1.37	0.56
4.00	3.00	5.00	-1.21	0.73
114.00	152.00	131.33	1.71	0.00
23.00	30.00	23.67	-1.48	0.02
1430.00	1375.00	1352.00	1.01	0.82
20.00	15.00	16.33	-1.12	0.71
35.00	34.00	37.67	1.16	0.36
2651.00	2594.00	2641.67	-1.09	0.04
1223.00	1174.00	1250.00	-1.03	0.47
390.00	522.00	484.33	1.35	0.00
12.00	38.00	19.00	1.25	0.52
80.00	85.00	88.67	1.32	0.01
1.00	4.00	2.00	-1.65	0.46
1955.00	2226.00	2128.00	1.23	0.00
350.00	394.00	376.67	1.07	0.24
12.00	12.00	13.00	-1.61	0.03
283.00	299.00	299.00	-1.03	0.67
10.00	5.00	6.00	-1.45	0.31
802.00	796.00	804.33	1.13	0.01
507.00	595.00	580.00	1.08	0.16
670.00	787.00	760.67	1.12	0.03
977.00	1111.00	1058.00	1.13	0.00
1929.00	2140.00	2121.33	1.19	0.00
925.00	1003.00	935.33	1.20	0.00
2431.00	2502.00	2583.33	1.04	0.33
17.00	8.00	10.67	-1.14	0.73
2690.00	2911.00	2888.33	1.01	0.79
826.00	864.00	866.00	-1.10	0.03
13298.00	15379.00	14492.67	-1.24	0.00
6616.00	7133.00	6959.67	1.30	0.00
3.00	3.00	3.67	-2.53	0.01
57.00	69.00	59.00	1.07	0.61
5603.00	5861.00	5755.33	-1.16	0.00
5.00	5.00	5.33	-1.26	0.63
331.00	350.00	360.67	1.15	0.01
1433.00	1483.00	1472.33	-1.18	0.00
971.00	942.00	1009.67	-1.09	0.07
396.00	352.00	369.67	-1.37	0.00
15.00	12.00	15.00	1.11	0.68
22.00	11.00	16.33	1.10	0.80
1044.00	1029.00	1083.00	-2.00	0.00
654.00	640.00	638.00	1.02	0.71
6969.00	6572.00	6638.33	-1.08	0.10
311.00	312.00	297.00	-1.10	0.20
30.00	19.00	25.00	1.08	0.72
1089.00	977.00	1065.00	1.01	0.82
880.00	928.00	955.33	-1.21	0.00

660.00	638.00	688.33	1.45	0.00
33.00	36.00	30.67	-1.20	0.27
18292.00	18211.00	18294.67	-1.01	0.70
3.00	10.00	4.67	-1.29	0.61
7267.00	6795.00	6914.33	-1.35	0.00
774.00	845.00	817.67	1.00	0.98
1516.00	1613.00	1565.33	-1.10	0.02
257.00	329.00	283.33	1.23	0.01
154.00	187.00	161.00	1.17	0.09
673.00	641.00	673.67	1.09	0.08
136.00	164.00	150.00	1.06	0.45
377.00	397.00	392.67	1.06	0.25
1326.00	1371.00	1390.67	1.10	0.02
435.00	552.00	501.00	1.20	0.00
237.00	279.00	277.00	1.14	0.05
275.00	341.00	310.67	1.15	0.03
28.00	36.00	33.33	-1.12	0.43
44.00	55.00	56.00	-1.03	0.85
76.00	65.00	72.00	-1.30	0.01
1341.00	1721.00	1562.67	1.21	0.00
1632.00	1726.00	1666.33	-1.02	0.64
21.00	10.00	13.00	1.38	0.32
819.00	860.00	890.00	-1.00	0.97
17.00	26.00	20.33	-1.18	0.45
7.00	4.00	6.33	1.04	1.00
8.00	10.00	7.33	-2.60	0.00
516.00	571.00	554.33	1.02	0.75
1082.00	1113.00	1121.67	1.01	0.78
12.00	9.00	9.67	-2.56	0.00
417.00	430.00	434.67	1.19	0.01
602.00	736.00	689.33	-1.04	0.41
94.00	76.00	81.33	-1.03	0.79
2599.00	2973.00	2870.67	1.07	0.07
1572.00	1604.00	1574.67	-1.04	0.38
266.00	276.00	286.00	1.07	0.31
1332.00	1470.00	1405.00	1.12	0.01
989.00	1006.00	1009.33	1.25	0.00
955.00	1055.00	1054.67	1.16	0.00
2120.00	2197.00	2210.00	1.06	0.11
315.00	295.00	301.33	1.03	0.71
4068.00	4371.00	4200.00	1.03	0.44
0.00	0.00	0.33	-4.69	0.13
121.00	126.00	123.67	1.31	0.00
1274.00	1166.00	1183.33	-1.68	0.00
1.00	5.00	3.00	-1.34	0.67
284.00	245.00	277.67	-1.51	0.00
1025.00	1194.00	1122.00	1.12	0.01
460.00	500.00	500.33	1.01	0.91

907.00	857.00	941.00	-1.05	0.35
471.00	571.00	522.33	-1.12	0.04
699.00	635.00	690.00	-1.12	0.08
364.00	410.00	415.33	1.02	0.74
2104.00	2721.00	2430.67	-1.09	0.10
7.00	12.00	8.00	-2.09	0.00
3783.00	4349.00	4286.33	1.05	0.24
377.00	322.00	343.33	-1.13	0.11
14.00	9.00	12.67	-2.07	0.00
388.00	409.00	418.33	-1.00	1.00
1.00	0.00	0.67	-3.14	0.18
533.00	567.00	570.67	1.04	0.43
12390.00	12624.00	12692.00	-1.20	0.00
199.00	250.00	230.67	1.27	0.00
359.00	370.00	392.33	1.08	0.18
240.00	270.00	266.00	-1.09	0.18
183.00	196.00	189.67	1.05	0.53
6.00	1.00	5.67	-1.30	0.54
24.00	22.00	23.33	-1.01	1.00
140.00	158.00	154.67	-1.04	0.59
31.00	25.00	28.00	1.24	0.25
1450.00	1600.00	1522.67	1.20	0.00
417.00	491.00	485.33	1.17	0.01
644.00	664.00	660.33	-1.01	0.82
799.00	875.00	897.67	1.22	0.00
1060.00	1181.00	1188.00	1.29	0.00
763.00	751.00	782.33	1.03	0.58
566.00	605.00	624.00	1.16	0.00
1479.00	1484.00	1590.67	1.20	0.00
312.00	378.00	349.33	-1.11	0.10
1522.00	1581.00	1623.67	1.02	0.65
1027.00	1102.00	1073.33	1.14	0.00
181.00	232.00	219.33	1.03	0.68
323.00	383.00	378.00	1.20	0.00
610.00	682.00	711.67	1.14	0.04
1945.00	2100.00	2105.67	1.03	0.50
7532.00	7786.00	8133.00	-1.01	0.91
5391.00	4829.00	5185.33	-1.19	0.00
3657.00	3698.00	3764.33	-1.18	0.00
7.00	8.00	8.00	-3.09	0.00
8.00	13.00	14.33	-1.56	0.07
565.00	631.00	620.67	1.00	0.94
763.00	878.00	814.00	1.08	0.10
37.00	25.00	36.00	-1.08	0.63
224.00	256.00	266.67	1.09	0.27
1339.00	1487.00	1484.00	1.21	0.00
71.00	83.00	74.00	-1.21	0.13
1428.00	1590.00	1584.67	-1.08	0.04

67.00	69.00	68.67	-1.22	0.06
2119.00	2111.00	2087.00	1.28	0.00
412.00	430.00	452.67	1.65	0.00
900.00	998.00	981.67	-1.07	0.09
1247.00	1214.00	1266.00	1.03	0.44
6393.00	6354.00	6539.00	-1.17	0.00
130.00	145.00	140.00	1.22	0.02
2247.00	2292.00	2315.67	-1.11	0.00
2238.00	2265.00	2272.67	1.04	0.28
666.00	633.00	664.00	-1.19	0.00
227.00	290.00	255.00	1.26	0.00
945.00	1012.00	994.00	1.10	0.08
1139.00	1175.00	1148.00	1.04	0.39
2.00	2.00	2.67	-1.74	0.30
2.00	3.00	2.67	-1.13	1.00
13543.00	13021.00	13146.33	-1.50	0.00
409.00	394.00	397.00	1.38	0.00
20.00	25.00	22.33	1.16	0.44
14.00	23.00	20.33	1.02	1.00
9.00	10.00	9.33	-5.73	0.00
582.00	556.00	563.33	1.23	0.00
0.00	0.00	0.33	-6.16	0.04
488.00	446.00	463.67	1.20	0.00
1002.00	1033.00	1017.67	1.08	0.08
570.00	607.00	611.00	1.15	0.00
21439.00	22085.00	23166.33	2.13	0.00
473.00	461.00	471.33	1.40	0.00
45.00	39.00	38.67	-1.69	0.00
35.00	37.00	37.33	1.55	0.01
7.00	6.00	7.67	2.03	0.06
6.00	8.00	5.67	-1.95	0.04
228.00	200.00	217.33	-1.06	0.45
3362.00	3469.00	3467.33	1.19	0.00
2447.00	2342.00	2473.00	-1.08	0.06
3971.00	4287.00	4173.67	-1.07	0.06
10219.00	10982.00	10577.33	1.11	0.00
3960.00	3951.00	4030.33	-1.19	0.00
424.00	523.00	514.67	1.24	0.00
1027.00	1236.00	1270.33	1.21	0.00
2795.00	2760.00	2825.33	1.03	0.47
1120.00	1374.00	1315.67	1.10	0.06
24079.00	23971.00	24494.67	-1.15	0.00
0.00	0.00	0.33	-10.58	0.00
16.00	7.00	15.33	-1.72	0.01
25.00	15.00	24.00	-1.59	0.00
379.00	352.00	365.00	1.39	0.00
0.00	0.00	0.00	-33.26	0.00
412.00	472.00	450.00	1.06	0.27

619.00	776.00	721.33	1.04	0.45
66.00	81.00	77.67	1.17	0.16
256.00	295.00	280.33	-1.15	0.02
2588.00	2645.00	2676.00	1.13	0.00
762.00	875.00	817.00	-1.24	0.00
321.00	273.00	307.67	1.07	0.35
564.00	598.00	593.33	-1.13	0.01
789.00	838.00	819.67	-1.00	0.96
6520.00	6449.00	6533.67	1.03	0.52
4649.00	4575.00	4633.67	1.09	0.02
2041.00	1890.00	2058.00	1.20	0.00
3477.00	3162.00	3402.67	1.01	0.90
2483.00	2899.00	2661.67	1.04	0.36
8.00	18.00	11.33	-1.19	0.55
1312.00	1480.00	1392.00	1.09	0.03
14.00	19.00	14.00	-1.06	0.92
77.00	45.00	57.00	1.01	0.97
4664.00	4754.00	4990.67	-1.09	0.07
18.00	19.00	20.67	-1.36	0.10
1254.00	1312.00	1370.00	1.05	0.28
49.00	38.00	43.33	-1.27	0.08
744.00	685.00	713.67	-1.23	0.00
206.00	188.00	190.67	1.11	0.21
1889.00	2067.00	1933.33	-1.07	0.16
1202.00	1242.00	1291.33	1.10	0.03
525.00	504.00	529.00	1.10	0.13
137.00	181.00	167.00	-1.16	0.05
217.00	337.00	290.00	-1.32	0.00
3015.00	2988.00	3180.67	1.07	0.16
301.00	412.00	373.00	1.06	0.41
582.00	550.00	554.00	-1.16	0.01
1768.00	1765.00	1797.67	-1.09	0.04
253.00	256.00	267.67	1.26	0.00
75.00	83.00	73.67	-1.10	0.46
432.00	468.00	474.00	1.10	0.06
1748.00	1707.00	1750.00	-1.23	0.00
32.00	21.00	27.67	1.67	0.01
13985.00	14696.00	14034.00	-1.03	0.48
254.00	280.00	278.67	1.10	0.11
636.00	622.00	665.00	1.15	0.01
1467.00	1491.00	1483.33	-1.14	0.00
303.00	384.00	351.00	1.17	0.02
6589.00	6580.00	6778.67	1.03	0.49
2572.00	2329.00	2418.33	-1.24	0.00
2162.00	2252.00	2315.33	-1.06	0.13
2.00	2.00	2.67	-1.62	0.40
397.00	525.00	481.67	-1.04	0.47
203.00	221.00	228.00	1.05	0.47

268.00	339.00	298.67	1.09	0.29
436.00	483.00	482.33	1.02	0.76
829.00	837.00	888.67	1.00	1.00
33.00	24.00	29.67	-1.14	0.45
335.00	354.00	377.33	1.12	0.07
124.00	110.00	122.67	-1.04	0.63
202.00	183.00	205.00	1.48	0.00
26.00	33.00	29.33	1.13	0.50
1528.00	1518.00	1580.00	1.08	0.05
954.00	1085.00	1082.67	1.03	0.62
311.00	369.00	362.33	1.53	0.00
1676.00	1883.00	1907.00	1.19	0.00
2242.00	2318.00	2431.33	1.11	0.02
327.00	351.00	370.67	-1.18	0.00
31.00	19.00	27.67	1.51	0.04
438.00	411.00	438.33	-1.22	0.00
2984.00	3167.00	3204.67	-1.13	0.00
2762.00	2774.00	2887.00	1.39	0.00
336.00	315.00	327.00	-1.10	0.13
213.00	228.00	227.00	1.40	0.00
1140.00	1180.00	1184.67	-1.14	0.00
1192.00	1292.00	1228.00	-1.06	0.21
249.00	265.00	283.00	1.01	0.92
1343.00	1441.00	1378.67	-1.19	0.00
1499.00	1710.00	1666.00	1.00	0.97
1868.00	2184.00	2074.67	1.11	0.01
918.00	912.00	983.33	1.09	0.08
587.00	742.00	661.00	1.07	0.26
3.00	5.00	3.33	-1.31	0.68
283.00	290.00	295.00	1.21	0.00
826.00	939.00	882.67	1.11	0.03
259.00	271.00	268.00	1.39	0.00
86.00	102.00	105.33	1.36	0.00
429.00	458.00	471.00	1.15	0.04
463.00	462.00	486.67	1.08	0.19
346.00	437.00	407.00	1.12	0.07
28.00	21.00	28.00	-1.48	0.01
663.00	621.00	662.33	1.09	0.14
828.00	873.00	873.33	1.20	0.00
237.00	302.00	272.33	-1.29	0.00
3753.00	3798.00	3756.33	1.03	0.43
135.00	143.00	149.00	1.32	0.00
462.00	494.00	504.67	-1.05	0.38
36.00	43.00	40.67	1.03	0.88
24.00	11.00	19.67	-1.17	0.51
1735.00	1530.00	1669.33	-1.10	0.06
736.00	713.00	729.33	-1.09	0.07
168.00	167.00	178.00	-1.13	0.09

3521.00	3439.00	3579.00	-1.02	0.68
337.00	392.00	379.33	-1.01	0.91
243.00	267.00	277.33	-1.04	0.59
615.00	728.00	720.00	1.11	0.12
602.00	655.00	634.67	1.10	0.05
10.00	5.00	6.67	1.77	0.16
4.00	3.00	2.33	-1.29	0.81
851.00	773.00	800.33	1.00	0.97
113.00	125.00	116.33	-1.41	0.00
1067.00	1047.00	1080.67	-1.38	0.00
489.00	558.00	553.00	1.02	0.68
417.00	441.00	427.33	1.00	0.98
4100.00	4213.00	4050.00	-1.07	0.12
80.00	92.00	82.67	1.56	0.00
1295.00	1320.00	1345.67	1.40	0.00
880.00	860.00	896.33	1.09	0.05
423.00	455.00	453.67	-1.10	0.09
691.00	657.00	727.67	-1.05	0.37
877.00	988.00	973.00	-1.01	0.76
594.00	641.00	651.00	1.05	0.51
2760.00	2952.00	2825.33	1.11	0.01
1597.00	1655.00	1641.00	-1.56	0.00
150.00	163.00	157.00	-1.34	0.00
22.00	25.00	24.33	1.01	1.00
294.00	279.00	286.67	1.01	0.84
1105.00	1148.00	1204.00	1.05	0.30
27.00	21.00	21.00	1.27	0.25
718.00	742.00	758.33	-1.01	0.79
295.00	294.00	297.33	1.21	0.01
2414.00	2720.00	2685.00	1.14	0.00
409.00	355.00	404.67	-1.12	0.06
38.00	37.00	35.33	-1.22	0.16
142.00	144.00	146.67	1.05	0.54
64.00	52.00	59.33	-1.46	0.00
1001.00	1193.00	1132.67	1.03	0.54
1.00	0.00	1.67	-1.39	0.78
10.00	16.00	12.33	-1.42	0.15
210.00	232.00	230.00	1.03	0.69
535.00	524.00	551.00	1.32	0.00
522.00	572.00	569.00	-1.01	0.78
21.00	9.00	21.33	2.61	0.00
2416.00	2283.00	2322.67	1.02	0.62
1066.00	1013.00	1055.67	-1.05	0.26
2254.00	2239.00	2282.67	-1.13	0.00
87.00	107.00	94.33	-1.29	0.01
0.00	2.00	1.00	-3.41	0.07
640.00	643.00	650.00	1.02	0.66
60.00	76.00	62.00	-1.05	0.71

352.00	353.00	349.33	-1.97	0.00
394.00	396.00	421.33	1.07	0.26
842.00	892.00	939.67	1.15	0.01
6378.00	6182.00	6485.67	-1.06	0.10
16.00	15.00	16.00	-1.06	0.85
556.00	559.00	580.33	1.04	0.47
1285.00	1286.00	1360.00	1.14	0.00
1205.00	1359.00	1329.33	1.07	0.11
255.00	297.00	290.67	-1.15	0.02
19.00	14.00	14.67	-1.13	0.69
5416.00	5624.00	5392.67	-1.23	0.00
6594.00	7175.00	7004.00	1.06	0.06
23.00	23.00	22.00	1.14	0.49
885.00	849.00	887.33	1.10	0.04
1764.00	1734.00	1793.33	-1.17	0.00
1022.00	1117.00	1130.33	-1.04	0.44
910.00	889.00	938.00	-1.06	0.27
1043.00	1222.00	1152.33	1.04	0.37
478.00	531.00	530.33	-1.06	0.26
4167.00	4199.00	4312.33	-1.33	0.00
907.00	987.00	971.00	-1.31	0.00
90.00	94.00	102.67	-1.06	0.58
166.00	191.00	179.33	-1.08	0.30
6.00	16.00	9.33	2.09	0.04
1021.00	1028.00	1058.33	1.18	0.00
1973.00	2185.00	2175.00	1.16	0.00
4.00	1.00	2.33	-2.77	0.04
197.00	182.00	190.00	-1.12	0.13
2.00	3.00	2.67	1.30	0.79
76.00	88.00	82.33	1.04	0.75
425.00	503.00	489.67	1.05	0.40
407.00	544.00	487.00	1.29	0.00
527.00	498.00	540.33	-1.10	0.05
46.00	44.00	46.00	1.53	0.00
27.00	51.00	33.33	-1.24	0.31
664.00	749.00	742.33	-1.09	0.11
2441.00	2460.00	2538.33	-1.03	0.43
56.00	43.00	45.00	-1.66	0.00
289.00	300.00	313.00	1.04	0.52
355.00	399.00	398.00	1.27	0.00
932.00	851.00	911.67	-1.28	0.00
1326.00	1194.00	1292.67	-1.12	0.04
674.00	674.00	678.00	1.24	0.00
268.00	309.00	295.33	-1.16	0.01
681.00	737.00	717.67	-1.01	0.87
1252.00	1383.00	1340.00	1.27	0.00
890.00	1033.00	1022.67	1.18	0.00
645.00	712.00	736.00	1.04	0.46

214.00	170.00	197.67	1.06	0.48
40.00	29.00	32.00	1.20	0.30
289.00	299.00	303.33	1.03	0.58
555.00	625.00	630.00	-1.04	0.42
51.00	72.00	71.00	-1.16	0.23
6.00	11.00	8.00	-1.43	0.26
154.00	134.00	142.67	-1.09	0.34
1577.00	1692.00	1641.67	-1.02	0.62
1368.00	1539.00	1412.67	-1.13	0.01
12.00	10.00	12.33	-1.80	0.01
1540.00	1554.00	1607.00	-1.06	0.17
334.00	330.00	324.67	-1.20	0.00
1619.00	1665.00	1716.67	-1.05	0.32
169.00	181.00	177.33	-1.18	0.02
32.00	31.00	32.33	1.14	0.48
790.00	772.00	802.00	1.37	0.00
568.00	546.00	577.33	1.19	0.00
318.00	326.00	347.67	1.04	0.51
788.00	891.00	837.33	1.13	0.02
178.00	125.00	147.67	-1.78	0.00
1104.00	1255.00	1158.67	1.02	0.72
133.00	146.00	138.00	-1.04	0.63
321.00	321.00	320.33	-1.13	0.07
849.00	893.00	867.00	1.10	0.06
14.00	11.00	14.67	-1.54	0.04
2338.00	2650.00	2486.00	1.39	0.00
47.00	65.00	59.67	1.55	0.00
28.00	27.00	25.67	-1.92	0.00
798.00	840.00	811.33	-1.14	0.02
13.00	22.00	15.00	1.20	0.46
1.00	4.00	2.67	-1.62	0.39
13742.00	14846.00	15765.33	1.34	0.00
6.00	9.00	9.00	1.39	0.31
3777.00	4035.00	4356.33	1.41	0.00
6.00	8.00	5.67	-1.01	1.00
299.00	291.00	291.00	-1.09	0.17
575.00	676.00	623.67	-1.07	0.21
676.00	709.00	686.33	1.26	0.00
1207.00	1192.00	1235.00	1.15	0.00
1134.00	1127.00	1152.33	-1.10	0.02
2057.00	2126.00	2082.33	1.22	0.00
2581.00	2745.00	2743.67	1.10	0.01
3105.00	3783.00	3493.67	-1.01	0.84
1119.00	998.00	1045.67	1.07	0.29
7650.00	7100.00	7177.67	-1.46	0.00
575.00	618.00	616.67	-1.07	0.18
973.00	940.00	991.00	-1.39	0.00
666.00	684.00	684.33	-1.19	0.00

3.00	0.00	1.00	-3.40	0.07
89.00	117.00	107.33	1.18	0.15
281.00	313.00	323.33	1.30	0.00
474.00	440.00	438.33	-1.10	0.14
4584.00	4809.00	4778.67	-1.25	0.00
2.00	3.00	2.00	-2.60	0.06
4327.00	4167.00	4272.67	-1.32	0.00
155.00	150.00	150.00	-1.03	0.72
14.00	7.00	10.67	-1.14	0.72
8804.00	9214.00	9455.00	1.10	0.02
795.00	751.00	801.33	1.15	0.00
2361.00	2343.00	2407.00	-1.17	0.00
768.00	739.00	755.67	-1.23	0.00
694.00	696.00	730.33	1.06	0.33
494.00	590.00	579.00	1.98	0.00
18.00	35.00	26.00	-1.30	0.12
203.00	161.00	193.00	1.13	0.14
5.00	5.00	6.00	-2.12	0.02
32.00	45.00	40.00	-1.22	0.15
19.00	24.00	21.33	-1.15	0.51
5.00	5.00	7.00	2.25	0.05
55.00	73.00	74.67	1.01	1.00
4.00	4.00	4.33	-1.54	0.30
427.00	390.00	386.33	-1.11	0.14
1798.00	1751.00	1826.00	-1.30	0.00
592.00	619.00	631.33	1.09	0.07
5.00	6.00	5.00	1.62	0.31
1673.00	1655.00	1735.33	-1.14	0.00
1142.00	1367.00	1249.33	-1.01	0.81
3511.00	3469.00	3555.67	-1.14	0.00
2435.00	2520.00	2452.33	1.02	0.65
12.00	16.00	14.67	1.11	0.67
3.00	4.00	3.00	-1.55	0.41
56.00	65.00	58.33	-1.11	0.41
5393.00	6024.00	5533.00	1.02	0.79
4007.00	4127.00	4181.67	-1.03	0.44
2316.00	2519.00	2439.00	-1.12	0.00
5532.00	5265.00	5377.33	-1.10	0.02
2669.00	2710.00	2704.67	-1.12	0.00
634.00	659.00	637.00	-1.18	0.00
337.00	327.00	338.33	-1.24	0.00
2.00	4.00	3.33	-1.31	0.68
399.00	393.00	398.00	-1.91	0.00
2149.00	1954.00	2031.00	-1.17	0.00
147.00	166.00	148.67	-1.31	0.00
4.00	5.00	6.33	-1.75	0.08
1241.00	1168.00	1200.00	-1.03	0.49
49.00	54.00	49.33	1.52	0.00

676.00	803.00	764.33	1.16	0.00
798.00	858.00	831.67	-1.03	0.51
19.00	15.00	15.33	1.33	0.23
541.00	534.00	561.33	1.01	0.80
948.00	977.00	1007.33	1.23	0.00
908.00	869.00	887.33	-1.18	0.00
4348.00	4451.00	4615.33	1.15	0.00
1270.00	1329.00	1331.00	1.36	0.00
6317.00	6881.00	6920.33	1.02	0.65
1638.00	1801.00	1804.00	1.20	0.00
471.00	470.00	489.00	1.05	0.33
1288.00	1496.00	1526.00	1.27	0.00
885.00	996.00	1013.33	-1.30	0.00
2035.00	2127.00	2251.67	1.17	0.00
3152.00	3430.00	3335.00	1.10	0.02
1576.00	1496.00	1584.00	1.06	0.27
16.00	21.00	19.33	-1.38	0.08
4375.00	4997.00	4675.00	-1.07	0.07
1512.00	1667.00	1616.33	1.08	0.08
57.00	64.00	55.33	1.02	0.88
1.00	1.00	1.33	-1.24	1.00
4295.00	4805.00	4466.00	1.18	0.00
3483.00	3922.00	3679.00	1.09	0.05
791.00	854.00	817.33	-1.02	0.74
710.00	656.00	709.33	-1.18	0.00
1534.00	1725.00	1632.00	1.03	0.49
778.00	901.00	851.00	1.07	0.15
42.00	45.00	40.00	-1.00	1.00
1744.00	1785.00	1802.00	1.07	0.15
1445.00	1402.00	1419.67	1.13	0.01
1811.00	1871.00	1888.33	1.07	0.07
1038.00	1050.00	1073.33	-1.10	0.05
543.00	463.00	511.00	1.01	0.91
963.00	869.00	915.67	1.18	0.00
1984.00	1786.00	1878.67	1.13	0.02
97.00	100.00	93.67	1.50	0.00
1494.00	1367.00	1469.33	-1.25	0.00
1088.00	1148.00	1126.00	-1.07	0.09
1894.00	1873.00	1901.33	1.46	0.00
1.00	0.00	0.33	-5.42	0.07
2170.00	2137.00	2133.33	-1.10	0.03
1281.00	1370.00	1358.33	-1.10	0.01
651.00	694.00	710.33	-1.06	0.27
236.00	255.00	259.33	1.01	0.89
111.00	170.00	135.00	-1.32	0.00
149.00	150.00	155.00	-1.89	0.00
567.00	552.00	591.00	1.11	0.04
50.00	40.00	51.00	1.19	0.23

7.00	8.00	11.67	-2.22	0.00
898.00	993.00	920.67	1.38	0.00
2160.00	2357.00	2232.00	-2.14	0.00
3366.00	3309.00	3346.33	-1.52	0.00
3174.00	3333.00	3269.67	-1.09	0.02
843.00	991.00	925.33	1.30	0.00
28.00	27.00	29.33	1.29	0.14
4520.00	4635.00	4769.33	1.07	0.04
140.00	160.00	156.00	1.07	0.39
803.00	849.00	867.00	-1.05	0.26
21.00	34.00	28.67	1.43	0.05
10407.00	11218.00	11390.67	1.18	0.00
55.00	36.00	49.67	1.08	0.61
100.00	123.00	118.33	1.21	0.06
6507.00	7080.00	7062.67	1.17	0.00
1328.00	1509.00	1505.00	-1.01	0.78
7086.00	7830.00	7754.00	1.24	0.00
149.00	203.00	184.67	-1.12	0.14
11416.00	12151.00	12281.67	1.15	0.00
444.00	502.00	482.00	1.15	0.01
14185.00	13612.00	14048.33	-1.40	0.00
1996.00	2144.00	2100.67	1.01	0.84
5.00	7.00	4.33	-1.01	1.00
11.00	2.00	7.33	1.03	1.00
4639.00	4968.00	4852.00	-1.21	0.00
1486.00	1502.00	1508.00	-1.12	0.00
4.00	6.00	4.33	1.27	0.69
5.00	8.00	5.00	1.13	0.85
626.00	655.00	668.33	-1.15	0.00
526.00	539.00	545.00	1.21	0.00
687.00	678.00	698.67	-1.08	0.13
830.00	867.00	877.67	1.01	0.84
404.00	400.00	408.00	-1.17	0.01
25140.00	28446.00	28304.67	1.08	0.03
112.00	142.00	144.00	-1.10	0.32
4.00	1.00	2.67	-1.50	0.51
4.00	5.00	6.00	-1.67	0.12
11.00	17.00	17.00	-1.09	0.71
16.00	20.00	14.33	-1.34	0.22
4159.00	4682.00	4399.67	1.04	0.31
1857.00	2489.00	2285.33	1.16	0.01
3759.00	4369.00	4246.67	1.17	0.00
1412.00	1528.00	1568.33	1.02	0.68
684.00	691.00	686.33	-1.02	0.65
238.00	234.00	250.00	-1.11	0.12
99.00	89.00	103.00	1.42	0.01
817.00	900.00	897.00	1.08	0.13
1395.00	1445.00	1415.67	-1.04	0.39

389.00	424.00	404.33	-1.08	0.22
312.00	286.00	297.00	-1.07	0.36
162.00	202.00	187.67	-1.11	0.17
623.00	661.00	678.33	-1.08	0.12
705.00	852.00	808.00	1.13	0.04
334.00	380.00	352.00	-1.16	0.01
2071.00	1924.00	2034.33	-1.39	0.00
429.00	547.00	487.67	1.01	0.93
337.00	414.00	391.67	1.17	0.06
1072.00	1080.00	1108.00	-1.01	0.77
931.00	944.00	928.00	-1.23	0.00
3253.00	3810.00	3461.00	-1.10	0.05
82.00	90.00	88.00	1.13	0.23
4640.00	4361.00	4418.00	-1.45	0.00
175.00	219.00	214.00	1.11	0.22
48.00	39.00	42.33	-1.11	0.49
3580.00	3853.00	3832.67	1.09	0.01
1266.00	1431.00	1474.00	1.17	0.00
799.00	812.00	789.00	-1.12	0.03
713.00	780.00	722.00	-1.05	0.42
294.00	281.00	285.00	-1.27	0.00
615.00	705.00	703.00	1.14	0.02
436.00	429.00	470.67	1.12	0.05
68.00	78.00	71.67	1.07	0.54
633.00	668.00	682.67	1.21	0.00
1125.00	1198.00	1194.33	1.01	0.73
0.00	0.00	0.00	-9.06	0.27
635.00	788.00	728.00	1.11	0.04
382.00	411.00	429.33	1.09	0.16
58.00	62.00	65.33	-1.23	0.07
591.00	598.00	623.67	-1.02	0.70
900.00	1017.00	972.00	-1.03	0.49
352.00	329.00	353.33	-1.13	0.03
236.00	285.00	270.33	-1.06	0.38
1091.00	1157.00	1134.00	-1.20	0.00
225.00	246.00	246.33	-1.04	0.52
23.00	36.00	30.67	-1.06	0.73
9.00	9.00	8.33	1.12	0.78
1271.00	1264.00	1230.67	-1.22	0.00
529.00	650.00	611.33	-1.35	0.00
316.00	335.00	367.00	1.10	0.22
573.00	530.00	587.00	1.09	0.10
490.00	525.00	532.33	-1.38	0.00
4364.00	4460.00	4651.33	-1.34	0.00
16.00	19.00	18.00	1.77	0.03
956.00	985.00	1019.00	1.07	0.14
586.00	554.00	600.00	-1.04	0.46
769.00	803.00	793.33	1.04	0.37

532.00	588.00	604.00	-1.03	0.63
174.00	203.00	211.33	-1.06	0.49
18.00	17.00	20.33	1.07	0.79
275.00	267.00	281.33	1.13	0.06
324.00	318.00	314.67	1.13	0.07
445.00	468.00	458.00	1.16	0.00
3133.00	3125.00	3239.00	-1.28	0.00
6993.00	7425.00	7378.67	1.05	0.13
407.00	444.00	452.00	-1.16	0.01
549.00	616.00	591.67	-1.15	0.00
1206.00	1448.00	1276.00	-1.04	0.54
8.00	6.00	7.33	-1.65	0.09
269.00	347.00	321.67	1.04	0.53
182.00	182.00	184.67	1.20	0.01
1134.00	1264.00	1189.67	1.10	0.04
1204.00	1236.00	1242.67	-1.04	0.36
232.00	257.00	257.33	-1.14	0.04
2069.00	2135.00	2175.00	1.01	0.79
4356.00	4593.00	4507.67	-1.13	0.00
444.00	502.00	504.33	-1.08	0.24
281.00	289.00	295.33	-1.10	0.09
47.00	45.00	48.67	1.36	0.03
853.00	957.00	946.67	1.05	0.28
323.00	320.00	331.67	-1.39	0.00
217.00	219.00	224.33	-1.01	0.86
2237.00	2645.00	2599.67	1.20	0.00
710.00	540.00	562.33	-1.42	0.00
753.00	787.00	779.67	-1.16	0.00
408.00	468.00	438.00	1.16	0.01
150.00	181.00	166.00	1.12	0.14
160.00	161.00	169.00	-1.16	0.03
1794.00	1929.00	1940.33	1.21	0.00
994.00	1030.00	977.67	-1.24	0.00
411.00	377.00	391.00	-1.01	0.83
85.00	80.00	86.67	1.04	0.71
3550.00	3745.00	3761.33	-1.03	0.31
161.00	183.00	180.33	1.46	0.00
9925.00	9339.00	9508.33	-1.44	0.00
2106.00	2108.00	2167.67	-1.18	0.00
1359.00	1559.00	1556.67	-1.03	0.52
1512.00	1524.00	1543.00	-1.14	0.00
5271.00	5637.00	5423.33	-1.04	0.27
1077.00	1244.00	1195.33	1.32	0.00
515.00	541.00	541.00	1.09	0.11
2775.00	2807.00	2981.33	1.03	0.55
724.00	892.00	823.33	1.14	0.04
797.00	927.00	911.00	-1.03	0.60
3446.00	3557.00	3687.67	-1.01	0.70

146.00	179.00	169.00	1.02	0.79
297.00	303.00	311.67	1.03	0.66
769.00	878.00	842.00	1.14	0.00
600.00	768.00	685.67	1.06	0.34
3427.00	3922.00	3899.33	1.14	0.00
364.00	371.00	370.67	-1.04	0.43
925.00	1269.00	1082.67	-1.02	0.76
769.00	866.00	816.00	1.02	0.66
1069.00	1083.00	1104.33	1.04	0.36
423.00	493.00	466.33	1.25	0.00
300.00	275.00	299.00	-1.15	0.03
942.00	1054.00	1019.33	1.06	0.21
1026.00	1246.00	1189.33	1.16	0.00
955.00	1032.00	1074.67	1.05	0.30
463.00	483.00	483.00	-1.03	0.55
656.00	712.00	710.33	-1.06	0.22
239.00	242.00	253.00	1.04	0.52
953.00	1011.00	1020.33	-1.57	0.00
769.00	938.00	843.33	1.04	0.43
2.00	2.00	1.33	-1.71	0.55
1138.00	1219.00	1223.67	1.13	0.02
391.00	428.00	408.67	1.01	0.87
140.00	179.00	155.33	1.07	0.47
706.00	928.00	786.67	-1.12	0.07
26.00	17.00	22.33	-1.11	0.67
31.00	40.00	43.00	1.02	1.00
3117.00	3040.00	3149.33	-1.07	0.08
112.00	127.00	119.33	-1.02	0.85
784.00	717.00	784.00	1.09	0.11
4.00	1.00	2.00	-1.17	1.00
822.00	834.00	853.67	-1.12	0.01
4.00	7.00	4.00	1.07	1.00
2878.00	3206.00	3040.67	1.02	0.67
449.00	415.00	430.67	-1.20	0.00
810.00	870.00	916.67	1.01	0.88
431.00	504.00	487.67	-1.25	0.00
1446.00	1480.00	1517.67	-1.28	0.00
2596.00	2279.00	2382.33	-1.17	0.00
824.00	1061.00	922.00	1.13	0.06
683.00	776.00	730.00	-1.01	0.84
7.00	8.00	11.00	-1.14	0.65
1580.00	1629.00	1641.33	-1.14	0.00
2029.00	1872.00	1980.67	-1.27	0.00
1044.00	1105.00	1123.67	-1.57	0.00
31.00	26.00	30.67	-1.11	0.54
1006.00	1081.00	1050.33	-1.00	0.94
507.00	546.00	554.67	1.05	0.29
933.00	1017.00	999.33	-1.25	0.00

1439.00	1756.00	1670.67	1.18	0.00
591.00	640.00	604.67	-1.14	0.01
531.00	551.00	570.67	-1.04	0.49
568.00	581.00	604.33	1.15	0.00
1369.00	1408.00	1414.00	1.08	0.07
58.00	62.00	64.33	-1.16	0.19
24.00	21.00	19.33	1.54	0.05
186.00	204.00	206.00	-1.24	0.00
442.00	518.00	497.00	1.02	0.69
2.00	9.00	3.67	-2.35	0.02
899.00	866.00	907.67	-1.11	0.01
262.00	351.00	320.67	-1.77	0.00
1918.00	1999.00	2057.33	1.15	0.00
207.00	236.00	232.00	-1.04	0.56
583.00	701.00	661.67	1.18	0.00
24.00	32.00	28.00	-1.28	0.13
3.00	2.00	3.33	-1.11	1.00
82.00	90.00	97.00	1.27	0.03
142.00	141.00	142.00	1.23	0.02
119.00	116.00	122.33	1.21	0.03
2429.00	2276.00	2388.00	-1.71	0.00
100.00	103.00	104.33	-1.31	0.00
1167.00	1090.00	1145.00	-1.01	0.76
3.00	13.00	9.33	2.09	0.04
3833.00	3783.00	3923.67	1.05	0.31
475.00	505.00	506.00	-1.04	0.38
41.00	34.00	41.67	1.92	0.00
4009.00	3941.00	4223.00	1.01	0.76
1761.00	1710.00	1752.67	-1.23	0.00
833.00	898.00	920.33	1.02	0.76
317.00	319.00	328.00	-1.24	0.00
5.00	4.00	4.33	1.16	0.84
614.00	639.00	622.33	-1.27	0.00
2304.00	2243.00	2446.33	1.14	0.00
1231.00	1361.00	1287.00	1.14	0.00
1014.00	1002.00	1037.67	-1.32	0.00
918.00	1001.00	1007.67	-1.11	0.01
4879.00	5206.00	5126.67	1.13	0.00
566.00	597.00	612.67	1.13	0.01
936.00	878.00	931.00	1.17	0.00
622.00	660.00	654.67	1.21	0.00
146.00	143.00	134.67	-1.28	0.02
735.00	751.00	797.33	-1.03	0.60
1.00	1.00	2.33	-1.15	1.00
72.00	68.00	71.67	-1.02	0.90
175.00	211.00	208.67	1.13	0.13
28.00	27.00	28.33	-1.12	0.54
618.00	665.00	651.67	-1.12	0.01

87.00	60.00	77.33	-1.19	0.14
253.00	246.00	232.00	-1.22	0.03
1342.00	1526.00	1491.00	1.16	0.00
386.00	431.00	414.33	-1.09	0.15
824.00	969.00	903.67	1.09	0.07
2116.00	2597.00	2309.00	1.03	0.53
2798.00	3032.00	2980.00	-1.22	0.00
1461.00	1461.00	1510.00	-1.10	0.02
89.00	78.00	83.67	-1.60	0.00
779.00	752.00	767.67	1.03	0.51
387.00	368.00	386.67	1.01	0.80
2.00	1.00	1.00	-1.61	0.73
27.00	23.00	25.33	1.04	0.88
488.00	639.00	568.00	1.04	0.51
513.00	587.00	554.67	1.06	0.26
712.00	635.00	682.67	-1.06	0.26
149.00	191.00	174.33	-1.25	0.01
6.00	4.00	4.33	1.41	0.53
3471.00	3530.00	3539.00	-1.07	0.05
143.00	168.00	146.67	1.11	0.24
71.00	59.00	70.67	-1.06	0.61
961.00	977.00	1002.33	-1.11	0.01
97.00	127.00	122.33	-1.13	0.18
465.00	554.00	502.67	1.28	0.00
4364.00	4634.00	4481.67	-1.07	0.10
428.00	404.00	426.67	-1.18	0.00
0.00	0.00	0.00	-9.06	0.27
129.00	134.00	136.67	1.27	0.00
212.00	250.00	244.67	1.14	0.06
32.00	32.00	32.33	-1.14	0.39
73.00	72.00	74.67	-1.07	0.55
18.00	5.00	14.67	-1.45	0.22
331.00	345.00	332.00	1.04	0.47
4.00	1.00	2.67	-1.98	0.16
381.00	422.00	419.33	-1.11	0.04
969.00	959.00	1030.00	1.15	0.01
502.00	589.00	571.33	-1.03	0.61
1.00	0.00	1.00	-2.51	0.23
415.00	407.00	438.00	-1.08	0.18
448.00	491.00	472.33	1.12	0.04
1208.00	1196.00	1223.67	-1.12	0.01
983.00	1015.00	1010.00	-1.09	0.04
249.00	255.00	253.00	1.11	0.14
807.00	1040.00	950.00	1.16	0.01
2653.00	2791.00	2816.00	1.11	0.00
92.00	114.00	99.00	1.20	0.07
346.00	320.00	311.33	-1.31	0.00
1443.00	1403.00	1474.33	1.05	0.22

3.00	6.00	5.67	-1.59	0.25
115.00	81.00	93.00	-1.79	0.00
1176.00	1238.00	1218.67	-1.22	0.00
829.00	818.00	830.67	-1.13	0.01
147.00	156.00	150.33	-1.50	0.00
4.00	6.00	4.00	-1.09	1.00
164.00	168.00	170.00	-1.38	0.00
30.00	56.00	38.00	1.40	0.10
1044.00	1087.00	1092.67	-1.10	0.03
211.00	182.00	202.00	-1.60	0.00
2470.00	2412.00	2432.33	-1.41	0.00
432.00	494.00	462.33	-1.18	0.02
5865.00	6280.00	6338.00	1.21	0.00
6762.00	7355.00	7276.33	1.11	0.00
6.00	8.00	5.33	1.31	0.58
3.00	6.00	3.33	1.91	0.31
13.00	22.00	18.33	-1.01	1.00
7.00	11.00	8.33	-1.41	0.26
1.00	1.00	0.67	-4.42	0.04
54.00	55.00	60.00	-1.07	0.56
751.00	740.00	774.67	1.04	0.44
4429.00	4947.00	4862.33	1.10	0.02
9501.00	10505.00	10561.00	1.54	0.00
119.00	130.00	132.33	-1.00	1.00
1370.00	1618.00	1564.33	1.13	0.01
760.00	843.00	842.00	1.16	0.02
682.00	738.00	715.00	-1.13	0.01
1655.00	1733.00	1765.00	1.01	0.78
433.00	409.00	399.33	1.04	0.59
393.00	403.00	408.00	-1.15	0.02
41.00	51.00	44.33	1.03	0.87
1574.00	1507.00	1621.67	-1.21	0.00
6291.00	6556.00	6711.33	1.07	0.05
244.00	234.00	251.33	1.64	0.00
5694.00	5974.00	5925.33	-1.14	0.00
721.00	704.00	735.33	-1.12	0.01
662.00	784.00	735.00	-1.04	0.47
530.00	637.00	601.67	1.07	0.19
1808.00	2035.00	1932.33	1.15	0.00
804.00	919.00	873.33	-1.05	0.32
739.00	737.00	781.67	1.13	0.01
223.00	239.00	232.67	-1.16	0.03
1562.00	1743.00	1684.33	1.29	0.00
1418.00	1561.00	1503.33	1.04	0.43
240.00	251.00	273.67	1.09	0.32
336.00	306.00	331.00	1.05	0.44
1181.00	1256.00	1185.33	1.17	0.01
1155.00	1306.00	1205.33	-1.04	0.45

41.00	55.00	50.33	-1.36	0.01
3601.00	3776.00	3768.33	-1.09	0.01
13.00	15.00	16.00	1.08	0.76
1844.00	2049.00	1948.33	-1.02	0.68
3131.00	3463.00	3609.33	1.31	0.00
1007.00	1089.00	1085.67	1.18	0.00
861.00	864.00	852.00	-1.05	0.31
2375.00	2225.00	2366.33	1.22	0.00
1275.00	1325.00	1347.67	1.01	0.78
128.00	118.00	125.67	-1.05	0.59
865.00	914.00	979.33	1.21	0.00
253.00	236.00	232.67	-1.11	0.19
708.00	805.00	770.33	1.18	0.00
244.00	280.00	267.00	1.46	0.00
989.00	1124.00	1125.33	1.19	0.00
782.00	878.00	874.00	1.31	0.00
519.00	523.00	536.67	1.07	0.17
16776.00	18193.00	17758.00	1.21	0.00
1634.00	1606.00	1717.67	1.04	0.42
156.00	183.00	168.67	-1.19	0.02
2357.00	2465.00	2488.00	-1.00	0.97
23.00	20.00	22.67	1.14	0.49
1835.00	2212.00	2126.33	2.17	0.00
7.00	31.00	15.33	1.29	0.49
1078.00	1023.00	1088.67	-1.15	0.00
1202.00	1291.00	1266.00	1.06	0.16
736.00	713.00	752.33	-1.05	0.33
39.00	59.00	56.00	1.05	0.74
1272.00	1301.00	1332.00	1.11	0.02
389.00	475.00	431.00	-1.13	0.03
31.00	36.00	33.67	-1.09	0.57
157.00	137.00	160.67	-1.09	0.36
5495.00	5542.00	5726.67	1.06	0.11
4573.00	5367.00	4983.33	1.19	0.00
7487.00	8851.00	8389.67	-1.06	0.12
14267.00	15689.00	15571.33	1.98	0.00
351.00	358.00	377.33	-1.21	0.00
10398.00	10377.00	10598.00	1.00	1.00
2026.00	2596.00	2382.33	-1.06	0.28
993.00	1198.00	1160.00	-1.00	0.99
1096.00	1247.00	1216.33	1.12	0.01
5536.00	5938.00	6219.33	1.12	0.01
1198.00	1352.00	1243.00	1.06	0.24
190.00	222.00	217.67	1.01	0.94
58.00	58.00	59.33	-1.22	0.10
725.00	801.00	798.00	1.05	0.34
1592.00	1491.00	1575.33	1.06	0.23
41.00	47.00	45.00	1.00	1.00

577.00	551.00	598.00	-1.01	0.92
517.00	519.00	537.33	-1.12	0.02
618.00	687.00	666.33	-1.11	0.02
46.00	48.00	48.00	-1.27	0.05
1160.00	1218.00	1215.67	-1.15	0.00
3956.00	3879.00	3897.00	-1.13	0.00
1438.00	1673.00	1560.33	1.28	0.00
9893.00	11108.00	11196.33	1.12	0.00
7721.00	8100.00	8060.00	1.02	0.52
631.00	722.00	705.33	1.11	0.02
609.00	774.00	718.00	1.07	0.24
1288.00	1485.00	1493.00	1.27	0.00
475.00	608.00	536.00	1.31	0.00
1340.00	1610.00	1559.00	1.13	0.01
4267.00	4568.00	4576.33	1.06	0.07
479.00	484.00	534.67	1.12	0.08
2045.00	2642.00	2476.67	1.19	0.00
581.00	598.00	633.33	1.29	0.00
911.00	1001.00	1012.00	1.18	0.00
47.00	32.00	39.00	1.12	0.46
376.00	461.00	448.00	1.12	0.09
1374.00	1559.00	1487.67	1.25	0.00
3383.00	3689.00	3652.33	-1.02	0.53
2020.00	2052.00	2097.33	-1.28	0.00
2269.00	2199.00	2374.67	1.16	0.00
1383.00	1577.00	1514.00	1.18	0.00
1073.00	1106.00	1148.33	-1.02	0.67
2886.00	3502.00	3244.67	1.11	0.01
1200.00	1300.00	1342.33	1.07	0.15
62.00	76.00	80.00	-1.19	0.13
1420.00	1564.00	1596.00	1.19	0.00
62.00	76.00	66.00	1.32	0.02
1625.00	1838.00	1832.33	1.15	0.00
1380.00	1456.00	1433.67	-1.21	0.00
1628.00	1729.00	1774.00	1.02	0.68
725.00	816.00	783.67	1.14	0.00
35.00	33.00	34.00	-1.36	0.03
1840.00	1890.00	1916.33	1.04	0.32
1743.00	1886.00	1861.33	1.31	0.00
459.00	545.00	541.00	1.42	0.00
75.00	69.00	74.00	-1.20	0.07
1040.00	1182.00	1157.00	1.35	0.00
1048.00	1195.00	1142.67	1.15	0.01
3064.00	3183.00	3206.67	1.08	0.02
2264.00	2489.00	2364.00	1.06	0.19
1073.00	1111.00	1083.67	-1.45	0.00
2717.00	2746.00	2795.67	-1.32	0.00
192.00	201.00	224.67	1.18	0.04

2162.00	2154.00	2142.67	-1.02	0.64
1243.00	1249.00	1258.00	-1.13	0.00
1679.00	1681.00	1669.67	-1.01	0.86
784.00	776.00	785.67	1.02	0.74
643.00	648.00	643.33	-1.08	0.20
1108.00	1090.00	1135.00	1.10	0.03
841.00	898.00	978.00	1.05	0.46
990.00	1148.00	1131.00	1.15	0.00
107.00	103.00	103.67	1.07	0.47
991.00	1132.00	1078.33	1.15	0.00
1369.00	1315.00	1348.67	-1.31	0.00
859.00	987.00	934.67	-1.30	0.00
281.00	266.00	280.67	1.03	0.64
2976.00	3110.00	3112.67	1.12	0.00
2871.00	3142.00	3030.33	1.19	0.00
439.00	536.00	495.67	1.07	0.25
1247.00	1228.00	1270.67	-1.13	0.00
22.00	13.00	18.00	1.44	0.12
1126.00	1195.00	1162.67	1.04	0.41
1295.00	1399.00	1334.00	-1.14	0.00
961.00	926.00	888.00	1.02	0.78
5963.00	5345.00	5483.67	-1.33	0.00
4147.00	4646.00	4302.67	-1.17	0.00
45.00	41.00	38.67	1.17	0.32
457.00	410.00	439.00	1.03	0.69
465.00	491.00	503.33	1.12	0.03
2285.00	2543.00	2415.67	1.05	0.26
1857.00	1883.00	1927.33	-1.01	0.90
295.00	362.00	330.33	-1.13	0.12
186.00	266.00	221.67	1.19	0.04
715.00	839.00	776.33	1.04	0.48
3032.00	3478.00	3323.00	-1.03	0.46
1530.00	1432.00	1570.00	-1.00	0.93
425.00	406.00	387.67	-1.22	0.01
564.00	516.00	557.67	1.07	0.20
12169.00	12970.00	13391.67	1.06	0.11
776.00	935.00	928.33	1.07	0.22
1285.00	1548.00	1438.67	1.26	0.00
493.00	459.00	481.33	1.01	0.94
2342.00	2370.00	2445.33	1.21	0.00
1172.00	1133.00	1197.00	-1.09	0.05
44.00	46.00	46.33	-1.11	0.47
24.00	47.00	33.33	-1.42	0.02
4.00	3.00	4.33	-3.82	0.00
17.00	7.00	11.00	2.15	0.02
18.00	14.00	16.67	-1.48	0.06
279.00	295.00	277.33	1.03	0.73
1300.00	1414.00	1454.67	1.14	0.01

609.00	658.00	661.67	1.21	0.00
1506.00	1641.00	1535.67	1.07	0.19
1742.00	1907.00	1932.00	1.11	0.02
62.00	40.00	50.33	1.24	0.14
366.00	393.00	402.33	1.13	0.03
5549.00	5780.00	5682.00	1.01	0.79
1651.00	1715.00	1675.33	-1.24	0.00
116.00	131.00	114.33	-1.02	0.94
3871.00	3844.00	3761.00	-1.12	0.02
979.00	1121.00	1078.33	-1.09	0.05
1158.00	1322.00	1240.33	-1.07	0.13
258.00	266.00	253.67	-1.00	1.00
2643.00	2984.00	2732.00	-1.18	0.00
1429.00	1479.00	1479.33	1.25	0.00
1940.00	1878.00	1862.00	-1.03	0.57
387.00	402.00	392.00	1.09	0.16
1662.00	1834.00	1770.33	-1.05	0.19
972.00	1124.00	1077.67	1.02	0.58
20.00	31.00	22.67	-1.31	0.17
209.00	221.00	213.00	1.02	0.79
86.00	58.00	69.33	-1.22	0.10
914.00	812.00	903.00	-1.21	0.00
398.00	402.00	390.67	-1.05	0.40
1266.00	1193.00	1333.33	1.04	0.40
98.00	109.00	112.00	1.04	0.66
255.00	271.00	260.67	1.07	0.42
899.00	934.00	941.67	1.02	0.73
461.00	457.00	498.67	1.08	0.21
1472.00	1592.00	1535.33	1.00	0.94
0.00	1.00	1.33	-2.64	0.14
812.00	811.00	814.67	-1.25	0.00
51.00	49.00	55.00	-1.24	0.06
1770.00	1879.00	1858.00	-1.04	0.26
1183.00	1297.00	1271.33	1.02	0.71
2.00	4.00	3.00	-1.66	0.31
23.00	20.00	21.00	-2.08	0.00
512.00	502.00	522.00	1.02	0.77
460.00	435.00	473.33	1.53	0.00
947.00	1108.00	1079.67	1.08	0.08
807.00	952.00	885.00	1.22	0.00
3.00	10.00	6.00	2.16	0.08
3223.00	3315.00	3265.67	-1.01	0.82
1512.00	1719.00	1643.33	1.05	0.21
753.00	922.00	868.33	-1.05	0.34
460.00	533.00	510.33	1.16	0.01
1.00	1.00	1.00	-1.91	0.51
31.00	9.00	26.67	1.13	0.70
2739.00	2969.00	2983.33	-1.01	0.73

188.00	198.00	192.33	1.06	0.41
1730.00	1760.00	1854.33	-1.11	0.01
588.00	709.00	692.67	-1.07	0.22
330.00	344.00	356.00	1.04	0.54
726.00	900.00	882.67	1.26	0.00
1167.00	1414.00	1418.33	1.15	0.01
2696.00	3335.00	3289.33	1.14	0.02
2088.00	2400.00	2369.00	-1.05	0.22
2494.00	2696.00	2715.00	1.04	0.29
1048.00	1127.00	1147.33	1.26	0.00
2934.00	3285.00	3444.33	1.23	0.00
129.00	132.00	133.33	1.13	0.16
1284.00	1281.00	1384.33	1.14	0.01
3273.00	3263.00	3243.00	-1.16	0.00
47.00	34.00	44.67	-1.17	0.29
801.00	823.00	811.00	-1.05	0.26
1602.00	1724.00	1747.00	1.01	0.74
1611.00	1802.00	1741.33	1.18	0.00
1446.00	1636.00	1649.00	1.09	0.06
2713.00	2924.00	2954.67	1.08	0.06
603.00	618.00	675.67	-1.06	0.32
891.00	894.00	938.00	-1.05	0.25
379.00	436.00	437.33	1.07	0.22
823.00	891.00	908.33	-1.01	0.87
1030.00	1007.00	1090.00	1.01	0.80
5.00	0.00	4.00	-1.34	0.58
1320.00	1594.00	1578.67	1.21	0.00
1961.00	2310.00	2160.67	-1.00	0.98
1339.00	1636.00	1520.00	1.26	0.00
2952.00	3306.00	3254.33	-1.01	0.72
1498.00	1478.00	1527.67	-1.01	0.76
799.00	978.00	867.00	-1.09	0.12
4.00	2.00	3.00	-1.88	0.18
1375.00	1443.00	1422.67	1.14	0.00
4791.00	5173.00	5069.00	1.15	0.00
1349.00	1488.00	1425.00	1.05	0.27
1040.00	1160.00	1138.67	1.07	0.13
20.00	17.00	18.00	1.05	0.85
2140.00	2160.00	2177.33	-1.12	0.00
1395.00	1387.00	1440.67	-1.05	0.22
926.00	1001.00	1024.33	-1.04	0.39
5012.00	4628.00	4960.67	-1.04	0.30
2647.00	2708.00	2684.67	-1.09	0.02
974.00	1005.00	1029.33	1.13	0.01
89.00	116.00	111.33	1.05	0.63
17.00	32.00	22.00	1.03	0.87
788.00	954.00	873.33	-1.10	0.06
18.00	23.00	19.00	-1.15	0.54

1204.00	1371.00	1355.33	-1.01	0.73
291.00	313.00	313.33	-1.09	0.14
715.00	773.00	764.00	-1.00	0.96
1770.00	1816.00	1779.33	-1.02	0.59
1527.00	1866.00	1774.33	-1.26	0.00
11.00	21.00	17.33	-1.23	0.34
1854.00	1972.00	1928.33	1.10	0.01
2948.00	3223.00	3123.33	-1.04	0.30
233.00	271.00	248.67	-1.19	0.01
2268.00	2191.00	2191.33	-1.27	0.00
290.00	298.00	292.67	-1.04	0.50
326.00	389.00	348.33	-1.09	0.21
1147.00	1265.00	1251.67	1.10	0.02
32.00	23.00	26.33	-1.14	0.47
1529.00	1649.00	1616.33	-1.01	0.79
2098.00	2186.00	2189.00	-1.06	0.11
591.00	572.00	594.00	-1.04	0.47
607.00	653.00	633.33	-1.29	0.00
494.00	506.00	515.67	-1.05	0.31
20.00	39.00	28.33	1.21	0.35
2137.00	2429.00	2314.67	1.05	0.21
1818.00	2113.00	1989.00	-1.01	0.77
446.00	493.00	463.67	-1.13	0.03
2531.00	2504.00	2564.67	1.01	0.85
43.00	36.00	42.00	1.16	0.32
1331.00	1513.00	1386.00	-1.01	0.87
902.00	1000.00	941.67	-1.11	0.02
31.00	43.00	45.00	1.08	0.68
571.00	667.00	634.67	1.08	0.13
3035.00	3376.00	3260.33	-1.01	0.86
1271.00	1355.00	1323.33	1.03	0.49
3359.00	3400.00	3397.00	-1.15	0.00
695.00	783.00	750.67	-1.11	0.01
920.00	908.00	918.33	-1.03	0.55
961.00	1145.00	1095.67	1.23	0.00
1381.00	1632.00	1572.67	1.07	0.18
794.00	882.00	806.67	-1.05	0.41
705.00	758.00	741.67	1.10	0.03
1885.00	2175.00	2112.33	1.08	0.06
2120.00	2129.00	2040.00	-1.01	0.91
620.00	740.00	707.67	1.16	0.00
917.00	1054.00	1032.33	1.11	0.04
1530.00	1825.00	1746.67	1.11	0.01
1090.00	1223.00	1113.00	-1.68	0.00
791.00	797.00	813.00	1.05	0.28
392.00	348.00	395.00	-1.27	0.00
956.00	1062.00	1014.00	1.16	0.00
529.00	546.00	572.67	1.04	0.50

3.00	6.00	5.33	-1.94	0.04
1262.00	1174.00	1217.67	-1.05	0.26
378.00	399.00	403.33	-1.26	0.00
1568.00	1699.00	1743.00	1.06	0.21
1394.00	1461.00	1497.33	1.08	0.05
701.00	789.00	753.33	-1.02	0.71
15.00	28.00	23.00	1.13	0.56
6.00	4.00	3.67	-1.10	1.00
962.00	998.00	969.00	-1.03	0.56
4035.00	4631.00	4509.00	1.16	0.00
1615.00	1633.00	1685.33	1.07	0.09
6.00	4.00	5.00	-1.28	0.62
135.00	128.00	133.67	-1.28	0.00
182.00	164.00	172.67	-1.01	0.90
5.00	6.00	5.67	1.12	0.86
1477.00	1620.00	1617.67	-1.12	0.01
4157.00	4029.00	4216.67	-1.32	0.00
946.00	940.00	956.00	-1.12	0.01
4.00	5.00	4.00	-1.26	0.71
74.00	50.00	66.00	1.25	0.11
886.00	1026.00	1023.33	1.26	0.00
2274.00	2668.00	2525.00	1.40	0.00
382.00	459.00	419.00	-1.13	0.04
9944.00	9617.00	9845.00	-1.11	0.00
7648.00	8417.00	8155.00	1.07	0.06
623.00	685.00	659.67	-1.04	0.41
262.00	303.00	293.33	1.01	0.91
5620.00	6154.00	6220.00	1.33	0.00
2574.00	2791.00	2851.33	1.16	0.00
1481.00	1847.00	1723.00	1.22	0.00
119.00	87.00	101.67	-1.79	0.00
5801.00	6233.00	6138.67	-1.39	0.00
1218.00	1312.00	1318.33	-1.08	0.05
1248.00	1296.00	1333.67	-1.29	0.00
1474.00	1722.00	1670.33	1.17	0.00
1636.00	1775.00	1735.00	1.00	0.93
1341.00	1432.00	1415.67	1.06	0.16
157.00	158.00	168.33	-1.44	0.00
42.00	65.00	56.33	1.23	0.12
487.00	522.00	470.33	-1.35	0.00
1142.00	1108.00	1118.33	1.01	0.81
1200.00	1390.00	1338.33	1.07	0.13
52.00	57.00	56.00	1.02	0.88
117764.00	127730.00	128065.00	1.22	0.00
92.00	100.00	109.67	1.19	0.11
1173.00	1124.00	1184.00	1.01	0.77
9.00	16.00	11.67	-1.39	0.20
571.00	612.00	609.67	-1.02	0.62

57.00	44.00	54.33	-3.04	0.00
2080.00	2068.00	2177.67	-1.03	0.59
1415.00	1382.00	1396.00	1.07	0.12
1257.00	1273.00	1285.67	-1.16	0.00
118.00	153.00	142.00	1.26	0.01
1198.00	1397.00	1373.67	1.31	0.00
76.00	55.00	66.00	-1.53	0.00
7.00	16.00	10.67	1.26	0.46
3609.00	4014.00	3973.67	-1.01	0.69
7.00	4.00	4.33	-1.47	0.39
347.00	442.00	425.00	1.17	0.02
948.00	892.00	915.67	1.11	0.04
1339.00	1378.00	1348.33	-1.03	0.53
873.00	954.00	940.67	-1.03	0.53
1176.00	1197.00	1206.33	-1.24	0.00
1169.00	1267.00	1234.00	-1.27	0.00
1605.00	1793.00	1686.33	-1.09	0.05
989.00	893.00	937.00	-1.30	0.00
1099.00	1147.00	1178.33	-1.00	0.95
677.00	682.00	682.67	-1.15	0.00
1258.00	1334.00	1395.33	1.02	0.65
1740.00	1934.00	1927.00	1.09	0.04
756.00	823.00	807.67	-1.02	0.66
1980.00	1977.00	2162.33	1.08	0.12
929.00	1153.00	1121.33	1.14	0.02
779.00	837.00	817.00	1.04	0.41
362.00	345.00	352.67	-1.07	0.29
2772.00	2789.00	2859.33	1.10	0.01
1158.00	1323.00	1282.00	1.07	0.13
609.00	687.00	657.00	1.06	0.22
1282.00	1280.00	1300.67	1.02	0.59
594.00	561.00	597.33	1.16	0.00
35.00	45.00	43.00	1.44	0.01
1743.00	1811.00	1757.33	-1.12	0.01
1320.00	1370.00	1357.33	1.00	0.99
706.00	907.00	848.33	1.06	0.32
1900.00	2047.00	2011.67	1.04	0.24
885.00	906.00	882.00	1.03	0.59
574.00	666.00	625.67	1.01	0.86
2229.00	2214.00	2299.00	-1.00	0.92
48.00	48.00	49.67	1.11	0.44
713.00	721.00	718.67	1.05	0.33
1151.00	1252.00	1214.33	1.05	0.28
1024.00	1119.00	1158.00	1.08	0.10
1037.00	1091.00	1086.00	-1.16	0.00
412.00	436.00	456.67	-1.10	0.09
568.00	660.00	622.67	-1.17	0.00
708.00	889.00	827.00	1.30	0.00

508.00	524.00	540.00	1.03	0.55
530.00	560.00	563.00	-1.11	0.04
549.00	566.00	576.67	-1.03	0.52
10.00	11.00	13.67	-1.38	0.12
0.00	4.00	3.00	-1.99	0.13
4.00	7.00	5.33	-1.14	0.87
881.00	1038.00	1015.00	1.34	0.00
579.00	584.00	587.33	-1.15	0.01
905.00	978.00	969.67	1.02	0.67
27.00	25.00	28.67	-1.39	0.03
22.00	16.00	18.67	1.67	0.03
694.00	726.00	738.00	-1.15	0.00
1299.00	1394.00	1395.67	1.08	0.05
9.00	11.00	10.67	1.65	0.10
5.00	7.00	5.67	-1.77	0.10
2.00	6.00	3.67	1.76	0.34
4385.00	4729.00	4631.67	1.04	0.22
612.00	647.00	644.00	1.07	0.15
1962.00	2045.00	1986.33	1.04	0.40
5271.00	5482.00	5501.33	1.02	0.50
256.00	301.00	303.33	1.03	0.67
5.00	4.00	3.00	-1.34	0.67
730.00	820.00	758.33	-1.02	0.73
1968.00	1793.00	1877.33	-1.33	0.00
557.00	603.00	593.00	-1.01	0.92
209.00	204.00	211.67	-1.11	0.13
152.00	189.00	176.67	-1.07	0.47
211.00	175.00	194.67	-1.09	0.39
11.00	9.00	10.67	-1.52	0.11
115.00	98.00	112.67	-1.10	0.28
880.00	1008.00	940.33	-1.01	0.81
102.00	130.00	128.00	-1.33	0.00
2308.00	2369.00	2375.00	1.10	0.01
829.00	774.00	803.00	-1.03	0.59
1697.00	1689.00	1776.00	-1.03	0.53
377.00	451.00	413.33	1.17	0.01
1464.00	1758.00	1659.33	1.10	0.03
885.00	960.00	966.67	1.06	0.18
2045.00	2160.00	2219.00	1.08	0.05
633.00	628.00	634.00	1.02	0.74
1430.00	1508.00	1475.00	-1.18	0.00
1456.00	1503.00	1514.67	1.15	0.00
439.00	537.00	488.33	-1.29	0.00
30.00	28.00	25.33	-1.38	0.05
683.00	800.00	746.00	1.19	0.00
98.00	90.00	93.00	-1.05	0.64
4595.00	4707.00	4729.33	-1.13	0.00
1792.00	2029.00	1946.33	-1.04	0.30

1854.00	2045.00	2039.33	1.15	0.00
789.00	932.00	922.67	1.01	0.92
499.00	608.00	555.33	1.18	0.00
1086.00	1180.00	1192.00	1.16	0.00
1697.00	1919.00	1814.67	-1.23	0.00
439.00	477.00	461.00	1.01	0.92
445.00	485.00	483.33	-1.06	0.34
132.00	160.00	151.00	-1.17	0.05
3011.00	3416.00	3285.00	-1.05	0.19
2127.00	2362.00	2263.67	-1.28	0.00
3281.00	3386.00	3366.00	1.05	0.19
71.00	89.00	71.67	1.16	0.27
2266.00	2383.00	2342.67	-1.06	0.11
878.00	940.00	979.67	1.33	0.00
2024.00	2405.00	2232.00	-1.02	0.66
2293.00	2632.00	2412.67	-1.03	0.51
857.00	915.00	909.00	-1.10	0.02
484.00	394.00	433.33	-1.17	0.02
523.00	526.00	532.33	1.10	0.09
2901.00	3217.00	3063.33	1.08	0.06
415.00	498.00	461.67	-1.28	0.00
932.00	968.00	1004.00	-1.11	0.02
2409.00	2588.00	2678.67	1.09	0.05
133.00	153.00	144.00	1.10	0.32
346.00	388.00	369.00	-1.00	1.00
896.00	928.00	929.00	1.04	0.42
2059.00	2197.00	2191.33	1.11	0.00
315.00	342.00	345.00	1.03	0.61
834.00	819.00	903.33	1.17	0.01
729.00	691.00	698.00	-1.09	0.13
1806.00	1941.00	1979.00	-1.01	0.74
508.00	534.00	544.33	-1.22	0.00
5743.00	5676.00	5835.33	-1.14	0.00
511.00	519.00	525.33	-1.16	0.00
745.00	792.00	796.67	1.12	0.01
649.00	690.00	707.00	1.07	0.18
15.00	6.00	10.33	-1.01	1.00
0.00	0.00	0.00	-17.14	0.03
1026.00	963.00	1024.00	2.69	0.00
300.00	324.00	314.33	-1.15	0.02
836.00	976.00	933.67	1.09	0.06
29.00	25.00	28.33	1.05	0.83
1169.00	1303.00	1263.67	-1.04	0.37
1545.00	1645.00	1627.33	1.01	0.84
2710.00	3029.00	2971.67	1.03	0.38
211.00	287.00	240.67	1.76	0.00
1742.00	1978.00	1985.33	1.07	0.15
72.00	100.00	90.00	1.15	0.20

255.00	259.00	277.33	-1.02	0.77
1649.00	1641.00	1679.67	-1.03	0.42
223.00	236.00	242.67	1.20	0.01
1370.00	1545.00	1587.00	1.06	0.23
463.00	516.00	494.67	1.02	0.66
1273.00	1212.00	1323.67	-1.05	0.27
507.00	527.00	529.67	-1.24	0.00
436.00	439.00	447.00	1.07	0.20
326.00	363.00	361.67	1.08	0.17
1000.00	968.00	989.33	1.01	0.91
366.00	481.00	432.67	1.36	0.00
507.00	596.00	564.33	1.23	0.00
7.00	0.00	4.33	-1.17	0.86
18.00	16.00	19.33	-1.17	0.40
814.00	915.00	871.67	-1.19	0.00
193.00	243.00	217.33	-1.08	0.28
73.00	123.00	94.33	-1.14	0.33
13.00	5.00	7.67	2.47	0.03
106.00	142.00	124.00	1.21	0.05
9499.00	10011.00	9855.67	1.14	0.00
1129.00	1103.00	1116.00	-1.11	0.02
240.00	289.00	274.00	1.01	0.89
652.00	717.00	684.00	-1.06	0.24
901.00	806.00	830.33	-1.09	0.14
456.00	477.00	504.33	-1.12	0.04
1795.00	1906.00	1920.67	-1.02	0.59
54.00	49.00	47.33	1.97	0.00
1090.00	1134.00	1147.67	-1.01	0.73
3995.00	4164.00	4227.33	-1.11	0.00
6089.00	5828.00	5843.67	-1.19	0.00
5.00	4.00	5.67	-1.31	0.53
1260.00	1237.00	1259.33	-2.09	0.00
2.00	0.00	1.00	-3.71	0.04
16.00	21.00	19.00	2.53	0.00
67.00	76.00	73.00	-1.23	0.04
9.00	8.00	9.33	-1.01	1.00
899.00	931.00	957.67	2.02	0.00
4.00	1.00	1.67	-2.89	0.05
463.00	513.00	499.67	1.06	0.29
622.00	605.00	643.33	-1.03	0.58
560.00	544.00	563.33	1.30	0.00
792.00	942.00	889.33	1.01	0.76
2311.00	2588.00	2481.33	1.12	0.00
1702.00	1985.00	1916.00	1.05	0.27
874.00	896.00	927.33	1.17	0.00
39.00	45.00	42.33	1.45	0.01
9.00	18.00	15.00	-1.26	0.29
1156.00	1362.00	1327.00	1.06	0.24

47.00	57.00	88.67	-1.08	0.75
18.00	26.00	20.67	1.09	0.66
7.00	8.00	10.33	-1.18	0.65
3135.00	3332.00	3272.33	1.25	0.00
975.00	1045.00	1010.33	-1.03	0.50
12.00	12.00	10.00	1.47	0.22
780.00	779.00	792.67	-1.35	0.00
799.00	942.00	870.00	1.13	0.03
745.00	813.00	798.33	-1.10	0.04
490.00	521.00	497.67	-1.21	0.00
12.00	12.00	12.67	1.33	0.28
1505.00	1662.00	1591.00	-1.06	0.19
32.00	49.00	50.67	1.01	0.96
6732.00	6772.00	6895.33	1.07	0.08
592.00	622.00	610.67	3.70	0.00
1254.00	1229.00	1181.00	1.02	0.81
253.00	271.00	275.33	-1.13	0.06
16055.00	18720.00	17329.00	-1.35	0.00
19.00	7.00	16.00	1.43	0.16
5.00	1.00	2.33	-1.15	1.00
165.00	185.00	183.33	1.14	0.07
1168.00	1295.00	1245.67	-1.26	0.00
1307.00	1367.00	1375.00	1.04	0.28
596.00	685.00	682.33	-1.01	0.84
9683.00	11335.00	10618.33	-1.02	0.65
1249.00	1364.00	1287.33	1.07	0.17
2829.00	2839.00	2879.67	-1.03	0.47
3414.00	3326.00	3433.33	-1.01	0.89
1501.00	1457.00	1448.33	-1.01	0.78
10012.00	10864.00	10707.00	1.03	0.42
1228.00	1363.00	1274.00	-1.01	0.81
736.00	831.00	809.33	-1.06	0.21
443.00	529.00	488.00	1.06	0.29
792.00	864.00	868.00	1.04	0.44
319.00	397.00	349.33	1.40	0.00
7985.00	8844.00	8556.67	1.13	0.00
6932.00	7775.00	7537.33	1.09	0.01
1019.00	1133.00	1040.00	-1.14	0.02
4445.00	4790.00	4626.67	1.06	0.12
559.00	558.00	549.67	-1.21	0.00
626.00	644.00	664.67	-1.13	0.01
219.00	211.00	216.33	-1.08	0.26
1674.00	1865.00	1787.33	-1.18	0.00
319.00	386.00	377.67	1.11	0.16
253.00	373.00	319.67	1.21	0.02
13698.00	15023.00	14550.33	1.01	0.84
6587.00	7013.00	7089.33	1.04	0.28
778.00	848.00	848.67	-1.00	0.97

246.00	258.00	251.00	1.04	0.55
12947.00	14929.00	14557.33	1.35	0.00
4760.00	5352.00	5385.67	1.18	0.00
27.00	37.00	32.67	1.30	0.11
672.00	682.00	703.67	1.11	0.02
2218.00	2092.00	2185.00	-1.03	0.47
511.00	582.00	573.00	1.25	0.00
3668.00	4141.00	3905.00	1.46	0.00
2385.00	2389.00	2501.33	1.07	0.14
1009.00	974.00	1034.33	-1.27	0.00
475.00	550.00	535.67	1.04	0.47
783.00	760.00	812.67	-1.06	0.22
3389.00	3523.00	3626.67	-1.10	0.03
891.00	921.00	896.00	1.03	0.52
1474.00	1451.00	1502.67	-1.02	0.68
1903.00	1914.00	1932.00	-1.16	0.00
297.00	272.00	292.33	-1.40	0.00
2542.00	2705.00	2731.00	1.01	0.77
2120.00	2213.00	2121.33	-1.09	0.05
4780.00	4988.00	4935.00	1.13	0.00
419.00	536.00	471.33	-1.32	0.00
8.00	9.00	10.33	1.13	0.70
85.00	85.00	78.00	1.46	0.00
193.00	165.00	183.67	-1.19	0.02
6.00	2.00	3.67	-1.28	0.70
585.00	636.00	632.67	1.01	0.91
902.00	844.00	931.67	1.19	0.00
0.00	2.00	1.00	-2.21	0.35
1303.00	1532.00	1420.67	-1.06	0.18
1337.00	1564.00	1516.33	1.03	0.43
1966.00	2187.00	2183.00	1.05	0.21
2271.00	2343.00	2295.33	1.08	0.09
4.00	6.00	9.67	1.66	0.23
6476.00	6786.00	6792.00	1.19	0.00
42.00	56.00	49.67	1.40	0.02
6.00	18.00	11.00	1.35	0.31
3696.00	3975.00	3895.00	1.07	0.07
1046.00	1175.00	1136.00	1.25	0.00
4668.00	5229.00	5109.00	1.13	0.00
11067.00	12292.00	11968.00	1.17	0.00
1462.00	1591.00	1530.33	1.10	0.04
941.00	990.00	989.67	-1.09	0.03
83.00	86.00	84.00	-1.20	0.06
729.00	847.00	817.67	-1.00	1.00
1240.00	1195.00	1174.00	1.06	0.34
0.00	1.00	0.33	-5.43	0.08
1.00	1.00	1.00	-1.01	1.00
11.00	15.00	11.67	1.32	0.32

84.00	95.00	82.67	-1.36	0.00
35.00	49.00	47.00	1.02	0.96
1316.00	1256.00	1311.33	-1.09	0.04
1043.00	1313.00	1218.00	-1.09	0.07
93.00	99.00	93.00	1.40	0.00
232.00	172.00	177.67	-1.29	0.09
222.00	263.00	249.00	1.04	0.59
1090.00	1152.00	1147.33	-1.02	0.61
464.00	545.00	499.67	1.02	0.79
1066.00	1313.00	1182.67	-1.01	0.91
76.00	100.00	86.67	1.03	0.79
17.00	9.00	13.00	1.10	0.76
554.00	599.00	603.67	-1.00	0.99
724.00	846.00	843.00	-1.05	0.32
1076.00	1210.00	1148.33	1.01	0.88
516.00	534.00	519.67	-1.03	0.52
311.00	314.00	316.00	-1.52	0.00
819.00	844.00	853.00	-1.17	0.00
13.00	7.00	10.00	1.40	0.29
564.00	533.00	547.33	1.13	0.03
219.00	215.00	230.00	-1.04	0.57
287.00	286.00	269.67	-1.16	0.06
82.00	76.00	77.33	-1.28	0.01
546.00	592.00	565.67	1.01	0.83
311.00	316.00	322.00	-1.15	0.02
52.00	54.00	49.67	1.11	0.46
4386.00	4747.00	4383.00	1.04	0.45
461.00	506.00	486.67	-1.09	0.07
1838.00	1736.00	1776.00	-1.17	0.00
2718.00	2934.00	2853.00	-1.23	0.00
298.00	401.00	335.67	-1.05	0.54
14.00	15.00	15.33	-1.78	0.00
527.00	570.00	566.00	1.01	0.81
588.00	668.00	630.67	-1.01	0.91
188.00	184.00	197.00	-1.19	0.01
7.00	5.00	5.67	-1.94	0.04
444.00	478.00	474.00	-1.27	0.00
546.00	615.00	617.67	-1.08	0.15
119.00	129.00	128.67	1.16	0.07
421.00	446.00	441.67	-1.21	0.00
284.00	294.00	287.67	-1.03	0.70
974.00	946.00	963.00	-1.06	0.19
550.00	538.00	576.00	-1.12	0.03
97.00	132.00	112.67	-1.01	0.90
332.00	402.00	391.67	1.20	0.01
19.00	12.00	16.00	-1.71	0.01
289.00	349.00	312.33	-1.02	0.87
15.00	21.00	18.67	-1.01	1.00

163.00	174.00	160.00	1.05	0.54
4333.00	4764.00	4667.00	1.03	0.40
210.00	199.00	202.67	-1.10	0.21
921.00	1040.00	942.33	-1.16	0.01
2.00	2.00	3.00	-1.12	1.00
1688.00	1642.00	1613.00	-1.13	0.01
2012.00	2227.00	2169.00	1.04	0.30
2718.00	3282.00	3160.33	1.18	0.00
1816.00	1863.00	1835.00	-1.08	0.06
353.00	395.00	374.00	-1.08	0.18
1275.00	1222.00	1209.00	1.04	0.46
271.00	324.00	300.67	-1.28	0.00
1440.00	1707.00	1630.67	1.06	0.17
3708.00	3835.00	3886.33	1.07	0.03
522.00	576.00	574.33	1.10	0.05
1144.00	1264.00	1177.00	1.01	0.80
263.00	245.00	249.67	-1.10	0.15
814.00	876.00	899.00	1.12	0.02
162.00	184.00	178.00	1.13	0.09
1503.00	1588.00	1542.33	-1.10	0.01
1414.00	1397.00	1434.67	-1.12	0.00
578.00	639.00	646.00	1.01	0.82
243.00	213.00	242.67	1.07	0.37
356.00	340.00	348.67	1.01	0.85
111.00	95.00	98.67	-1.72	0.00
888.00	875.00	954.33	1.12	0.02
354.00	384.00	395.00	1.25	0.00
490.00	554.00	527.67	-1.17	0.00
879.00	999.00	1005.00	1.03	0.57
1234.00	1420.00	1391.00	1.04	0.40
1326.00	1516.00	1481.33	1.26	0.00
789.00	924.00	877.67	1.12	0.02
187.00	210.00	206.00	-1.11	0.11
23.00	20.00	21.33	1.19	0.37
800.00	902.00	815.67	-1.20	0.00
299.00	312.00	302.00	1.06	0.41
49.00	49.00	49.33	-1.65	0.00
355.00	326.00	376.00	1.16	0.09
472.00	510.00	499.00	1.13	0.01
422.00	412.00	438.67	-1.19	0.00
950.00	998.00	1010.33	-1.02	0.66
843.00	988.00	910.67	-1.17	0.00
776.00	723.00	772.33	-1.01	0.88
429.00	453.00	432.00	-1.03	0.71
934.00	978.00	937.67	1.06	0.27
1233.00	1500.00	1358.67	1.11	0.03
487.00	588.00	562.67	1.21	0.00
756.00	760.00	787.33	1.11	0.04

966.00	959.00	990.33	-1.20	0.00
969.00	1018.00	1038.00	1.07	0.13
3566.00	3252.00	3436.00	-1.12	0.01
1926.00	2202.00	2143.67	-1.00	0.97
2889.00	2855.00	2977.33	-1.10	0.01
1855.00	1871.00	1947.67	-1.09	0.03
1274.00	1226.00	1307.00	-1.06	0.17
2429.00	2793.00	2561.33	1.08	0.09
290.00	341.00	333.33	-1.04	0.53
1716.00	1676.00	1690.00	-1.03	0.46
864.00	852.00	861.00	-1.24	0.00
1548.00	1820.00	1765.67	1.18	0.00
333.00	343.00	375.00	1.03	0.70
361.00	406.00	411.33	-1.05	0.46
2178.00	2259.00	2267.67	1.03	0.48
160.00	155.00	167.00	1.24	0.01
1750.00	2010.00	1929.33	-1.06	0.12
925.00	1038.00	1006.00	1.17	0.00
4180.00	4482.00	4733.00	1.11	0.02
278.00	303.00	294.00	-1.10	0.19
3010.00	3315.00	3173.33	-1.17	0.00
25.00	25.00	26.00	-1.23	0.22
178.00	167.00	160.67	-1.33	0.00
1109.00	1089.00	1068.67	-1.22	0.00
20.00	15.00	17.00	-1.19	0.50
272.00	306.00	289.33	1.09	0.20
14.00	37.00	24.00	1.27	0.29
3.00	0.00	2.67	-1.62	0.39
48.00	57.00	47.67	-1.03	0.92
80.00	100.00	94.00	-1.00	0.97
97.00	109.00	114.00	-1.17	0.08
2533.00	2540.00	2570.00	-1.50	0.00
721.00	734.00	752.00	1.15	0.00
115.00	111.00	111.67	-1.12	0.28
687.00	674.00	668.00	-1.35	0.00
1377.00	1574.00	1506.00	1.00	0.99
434.00	400.00	415.33	1.18	0.00
155.00	168.00	165.00	1.18	0.03
254.00	193.00	225.67	1.04	0.60
20.00	19.00	21.00	-1.01	1.00
464.00	461.00	488.00	-1.13	0.01
1687.00	1864.00	1773.33	1.07	0.17
1178.00	1254.00	1283.00	1.04	0.30
184.00	168.00	185.00	1.12	0.13
186.00	201.00	202.67	1.84	0.00
158.00	189.00	181.33	-1.61	0.00
2927.00	3143.00	2981.00	1.05	0.25
1007.00	1079.00	1077.33	-1.02	0.66

375.00	379.00	367.33	-1.21	0.00
831.00	891.00	845.33	-1.06	0.26
442.00	544.00	535.00	-1.04	0.50
415.00	462.00	466.33	-1.20	0.00
925.00	995.00	981.67	-1.24	0.00
1709.00	1904.00	1819.00	-1.09	0.03
23.00	28.00	32.33	-2.34	0.00
984.00	1030.00	1015.67	-1.11	0.01
140.00	176.00	167.67	-1.29	0.00
1304.00	1384.00	1398.33	-1.05	0.26
294.00	445.00	357.67	-1.04	0.66
567.00	682.00	621.67	-1.14	0.01
23.00	25.00	24.67	-1.33	0.09
229.00	200.00	213.33	-1.03	0.73
2485.00	2601.00	2569.00	1.09	0.02
245.00	252.00	257.00	1.05	0.55
259.00	220.00	235.00	1.21	0.02
13.00	11.00	11.33	1.24	0.45
130.00	144.00	131.67	1.19	0.16
20.00	11.00	15.00	-1.15	0.62
3395.00	3585.00	3458.67	-1.10	0.01
244.00	292.00	279.67	-1.20	0.01
106.00	155.00	143.33	-1.08	0.37
161.00	177.00	182.00	-1.12	0.11
576.00	613.00	592.33	1.04	0.42
1755.00	1826.00	1726.00	-1.39	0.00
331.00	377.00	384.00	-1.01	0.92
830.00	950.00	920.00	1.11	0.02
442.00	494.00	459.67	1.07	0.25
306.00	297.00	317.33	-1.03	0.64
1237.00	1207.00	1203.33	-1.40	0.00
21.00	20.00	23.33	-1.49	0.01
2753.00	2584.00	2782.67	-1.14	0.00
2071.00	2322.00	2179.00	1.02	0.67
959.00	1265.00	1129.33	1.15	0.01
1784.00	2138.00	1998.33	-1.02	0.63
1151.00	1125.00	1149.00	-1.20	0.00
1841.00	1973.00	1934.00	-1.03	0.53
327.00	409.00	362.00	-1.13	0.06
600.00	739.00	694.00	-1.13	0.02
840.00	866.00	889.00	1.10	0.04
3.00	5.00	6.00	-1.84	0.05
2197.00	2452.00	2323.67	1.06	0.17
14.00	21.00	14.00	1.43	0.26
605.00	708.00	695.33	1.05	0.40
4343.00	4752.00	4631.67	1.03	0.33
316.00	363.00	350.33	-1.08	0.18
204.00	211.00	213.67	1.18	0.02

2254.00	2283.00	2228.67	-1.04	0.36
630.00	753.00	696.67	1.12	0.03
241.00	250.00	253.67	-1.14	0.03
49.00	61.00	57.67	1.20	0.15
1383.00	1529.00	1474.33	-1.09	0.04
262.00	231.00	233.67	-1.29	0.00
185.00	232.00	223.33	1.16	0.04
1227.00	1470.00	1398.00	1.04	0.40
24.00	24.00	22.67	-1.44	0.04
734.00	914.00	857.00	-1.11	0.04
222.00	222.00	231.00	-1.10	0.18
115.00	111.00	116.33	1.31	0.00
83.00	96.00	96.33	-1.01	0.97
181.00	176.00	183.00	1.29	0.00
81.00	93.00	88.33	1.03	0.81
123.00	185.00	159.67	1.09	0.35
18.00	19.00	16.67	-1.25	0.28
704.00	675.00	708.00	-1.13	0.01
307.00	367.00	345.67	1.01	0.95
2779.00	3146.00	3084.00	1.05	0.16
1040.00	1267.00	1162.67	1.17	0.00
176.00	174.00	179.33	-1.02	0.82
7.00	6.00	5.00	1.14	0.85
271.00	270.00	282.00	1.02	0.75
345.00	381.00	369.67	1.07	0.23
15.00	21.00	18.33	1.13	0.57
119.00	118.00	118.00	-1.49	0.00
90.00	88.00	77.33	-1.22	0.15
302.00	300.00	311.67	1.04	0.55
322.00	309.00	316.00	-1.31	0.00
441.00	473.00	488.00	1.17	0.03
164.00	207.00	193.33	1.01	0.89
171.00	215.00	187.33	-1.30	0.00
310.00	364.00	336.00	-1.11	0.07
218.00	203.00	213.33	1.15	0.05
3101.00	2928.00	3054.67	-1.34	0.00
574.00	623.00	611.00	-1.01	0.87
83.00	79.00	87.00	-1.04	0.73
114.00	103.00	102.33	-1.09	0.41
843.00	1076.00	989.67	1.11	0.04
442.00	499.00	476.33	-1.04	0.42
108.00	103.00	111.67	-1.12	0.21
287.00	377.00	337.00	1.16	0.04
1124.00	1301.00	1264.33	-1.09	0.05
11.00	17.00	14.33	1.09	0.75
395.00	327.00	390.67	-1.04	0.59
4.00	2.00	3.67	-1.99	0.09
3346.00	3693.00	3520.00	-1.01	0.87

213.00	281.00	256.00	-1.13	0.06
596.00	694.00	704.33	-1.04	0.45
287.00	340.00	321.67	-1.11	0.12
206.00	191.00	202.33	-1.13	0.08
34.00	30.00	34.00	-1.00	1.00
343.00	450.00	399.33	1.03	0.71
1268.00	1375.00	1351.67	-1.07	0.09
122.00	97.00	114.33	1.47	0.00
1103.00	1207.00	1179.00	-1.10	0.02
19.00	14.00	15.67	-1.70	0.01
99.00	121.00	112.00	1.45	0.00
50.00	52.00	50.00	1.16	0.29
394.00	444.00	433.33	-1.16	0.00
119.00	140.00	131.67	-1.03	0.71
369.00	388.00	408.00	-1.08	0.17
255.00	267.00	260.33	-1.02	0.74
4.00	2.00	2.00	-2.28	0.12
829.00	909.00	944.33	1.01	0.78
105.00	110.00	108.67	-1.02	0.86
255.00	313.00	308.67	-1.02	0.78
296.00	410.00	354.67	-1.11	0.14
83.00	88.00	86.33	1.41	0.00
611.00	674.00	617.00	-1.15	0.02
853.00	1015.00	979.33	1.34	0.00
1557.00	1769.00	1687.00	-1.01	0.88
1032.00	1158.00	1133.00	-1.14	0.00
436.00	484.00	493.33	1.25	0.00
211.00	245.00	224.33	-1.13	0.12
716.00	700.00	729.67	1.06	0.23
13.00	39.00	26.33	-1.01	1.00
25.00	22.00	25.00	-1.05	0.82
102.00	124.00	110.33	1.02	0.87
15.00	10.00	13.67	-1.36	0.20
743.00	813.00	837.67	1.09	0.08
255.00	308.00	292.67	1.03	0.69
1067.00	1096.00	1118.00	-1.00	0.91
272.00	312.00	309.33	-1.13	0.04
1024.00	1111.00	1120.33	-1.07	0.16
418.00	542.00	472.67	1.05	0.53
336.00	347.00	346.00	1.06	0.33
514.00	619.00	612.67	1.15	0.02
61.00	86.00	69.67	1.17	0.20
606.00	659.00	664.33	1.15	0.01
447.00	489.00	481.33	-1.21	0.00
2039.00	1968.00	2056.33	-1.31	0.00
378.00	400.00	417.67	1.17	0.02
143.00	169.00	164.67	1.17	0.06
231.00	266.00	245.67	1.08	0.27

754.00	890.00	871.67	1.06	0.23
1499.00	1590.00	1533.33	-1.01	0.74
1097.00	1124.00	1145.67	-1.06	0.15
94.00	156.00	121.67	-1.08	0.47
112.00	133.00	121.33	1.03	0.79
119.00	133.00	125.00	1.07	0.43
447.00	519.00	504.67	-1.07	0.23
129.00	142.00	153.00	-1.09	0.28
119.00	124.00	126.00	1.13	0.16
918.00	1152.00	1038.00	1.03	0.56
16.00	17.00	14.33	-1.25	0.37
2000.00	2207.00	2143.33	1.04	0.29
157.00	138.00	143.00	-1.14	0.13
1071.00	1414.00	1266.00	1.24	0.00
252.00	296.00	281.00	-1.15	0.03
236.00	274.00	256.00	-1.02	0.79
14.00	14.00	15.00	-1.06	0.92
1429.00	1426.00	1448.33	-1.09	0.03
968.00	1020.00	1002.67	-1.05	0.24
963.00	1032.00	994.33	-1.13	0.01
398.00	424.00	439.67	-1.11	0.06
372.00	402.00	421.00	1.13	0.09
29.00	26.00	23.33	1.00	1.00
227.00	259.00	250.00	-1.01	0.87
161.00	165.00	168.33	-1.42	0.00
120.00	92.00	101.67	1.03	0.77
888.00	1039.00	993.00	1.20	0.00
161.00	200.00	195.33	1.04	0.62
2.00	8.00	4.33	-1.01	1.00
752.00	893.00	860.67	1.25	0.00
225.00	268.00	264.33	-1.03	0.63
523.00	624.00	592.33	-1.06	0.28
977.00	1087.00	1070.67	-1.21	0.00
1749.00	2077.00	2031.00	-1.38	0.00
326.00	296.00	320.00	1.00	0.99
900.00	1003.00	958.00	-1.20	0.00
703.00	748.00	740.67	-1.07	0.11
160.00	166.00	173.33	-1.11	0.21
178.00	175.00	183.00	1.12	0.14
4.00	0.00	3.00	-1.23	0.83
613.00	696.00	665.67	-1.07	0.12
3.00	2.00	1.67	1.58	0.73
378.00	376.00	392.33	-1.10	0.16
76.00	78.00	78.00	1.12	0.31
103.00	90.00	91.00	1.17	0.15
607.00	693.00	659.33	1.06	0.22
396.00	476.00	419.00	-1.11	0.14
5.00	2.00	3.00	-1.23	0.83

717.00	683.00	733.33	1.03	0.54
200.00	196.00	199.67	1.16	0.03
83.00	123.00	107.00	1.00	1.00
153.00	202.00	174.00	1.55	0.00
1309.00	1463.00	1377.33	-1.17	0.00
1343.00	1522.00	1498.00	-1.10	0.03
50.00	60.00	50.33	1.27	0.11
1738.00	1757.00	1792.67	-1.06	0.12
83.00	66.00	72.00	1.15	0.22
124.00	101.00	117.67	-1.68	0.00
54.00	47.00	51.00	-1.23	0.10
311.00	335.00	353.67	1.09	0.23
717.00	655.00	699.67	-1.14	0.01
9.00	14.00	11.67	-1.10	0.82
254.00	316.00	280.00	1.38	0.00
335.00	319.00	329.33	-1.23	0.00
52.00	51.00	57.00	-1.30	0.02
444.00	365.00	409.67	-1.13	0.07
310.00	326.00	335.33	-1.02	0.71
329.00	355.00	336.67	-1.20	0.00
858.00	944.00	950.67	1.12	0.01
154.00	158.00	167.33	1.07	0.42
88.00	105.00	104.33	-1.18	0.07
124.00	133.00	126.00	-1.41	0.00
29.00	41.00	34.33	-1.08	0.65
326.00	387.00	381.67	-1.06	0.30
30.00	46.00	40.67	-1.15	0.34
57.00	63.00	69.67	-1.07	0.59
175.00	202.00	180.33	-1.03	0.74
547.00	563.00	583.33	1.19	0.00
5.00	1.00	2.33	-1.42	0.64
3.00	5.00	3.00	1.11	1.00
154.00	198.00	161.33	1.09	0.42
254.00	277.00	263.33	-1.03	0.65
160.00	166.00	160.00	-1.05	0.59
20.00	28.00	25.67	-1.34	0.06
246.00	256.00	239.67	-1.17	0.03
118.00	162.00	141.67	-1.39	0.00
439.00	467.00	482.33	1.20	0.00
6.00	2.00	6.67	1.50	0.32
149.00	157.00	166.67	-1.04	0.59
349.00	367.00	345.33	-1.05	0.49
297.00	340.00	307.00	-1.31	0.00
3.00	6.00	4.33	-1.01	1.00
17.00	19.00	21.67	1.60	0.03
627.00	564.00	622.00	1.02	0.76
902.00	962.00	902.33	-1.17	0.00
351.00	329.00	352.00	-1.06	0.37

211.00	239.00	240.67	-1.11	0.11
291.00	328.00	312.67	1.07	0.27
1196.00	1305.00	1238.67	-1.01	0.84
24.00	20.00	20.00	-1.10	0.73
38.00	40.00	37.67	1.05	0.75
947.00	818.00	846.67	-1.23	0.00
58.00	36.00	45.00	-1.04	0.84
19.00	12.00	14.67	-1.74	0.01
109.00	104.00	108.67	1.05	0.58
486.00	463.00	480.00	-1.12	0.03
10.00	4.00	6.67	-1.71	0.09
306.00	281.00	297.67	-1.04	0.56
322.00	356.00	337.33	-1.02	0.76
84.00	84.00	91.33	-1.12	0.27
448.00	465.00	478.00	1.02	0.77
49.00	56.00	52.33	1.26	0.08
28.00	14.00	25.67	1.17	0.47
25.00	34.00	31.00	-1.13	0.46
89.00	91.00	100.00	1.01	0.97
17.00	17.00	19.33	1.17	0.51
90.00	96.00	90.67	-1.20	0.06
931.00	832.00	872.67	-1.16	0.00
110.00	97.00	100.33	1.09	0.37
277.00	308.00	296.67	-1.14	0.04
62.00	38.00	46.33	1.09	0.56
50.00	56.00	60.67	1.28	0.06
313.00	344.00	330.33	1.08	0.20
36.00	34.00	32.67	-1.15	0.44
50.00	41.00	51.67	-1.14	0.32
1008.00	1139.00	1043.67	-1.13	0.03
240.00	274.00	256.00	-1.22	0.00
515.00	575.00	573.67	1.25	0.00
1765.00	2006.00	1919.33	1.02	0.63
2126.00	2076.00	2143.67	-1.18	0.00
424.00	471.00	466.00	-1.09	0.07
1171.00	1313.00	1274.00	-1.08	0.05
1132.00	1158.00	1137.33	-1.22	0.00
80.00	92.00	85.33	1.15	0.18
1085.00	1282.00	1189.67	-1.16	0.00
175.00	160.00	163.33	-1.14	0.16
272.00	433.00	369.00	1.10	0.28
298.00	276.00	288.33	-1.04	0.57
147.00	155.00	156.67	1.07	0.46
53.00	54.00	52.67	1.05	0.75
237.00	216.00	232.67	-1.17	0.03
123.00	115.00	124.67	1.06	0.53
41.00	38.00	39.33	1.25	0.17
14.00	10.00	13.00	1.42	0.19

247.00	293.00	272.00	-1.00	0.97
42.00	36.00	37.00	-1.01	1.00
101.00	134.00	126.33	1.12	0.29
7.00	3.00	5.00	1.06	1.00
5.00	9.00	7.33	1.14	0.76
7.00	12.00	10.67	1.13	0.71
50.00	65.00	60.33	-1.28	0.02
617.00	595.00	619.00	1.10	0.06
48.00	67.00	57.67	-1.17	0.20
63.00	65.00	70.33	-1.04	0.70
428.00	425.00	433.67	-1.09	0.09
218.00	256.00	235.33	1.02	0.78
526.00	622.00	590.00	-1.17	0.00
167.00	156.00	160.67	1.04	0.66
80.00	90.00	84.00	-1.16	0.12
83.00	74.00	79.00	-1.17	0.13
119.00	116.00	131.33	-1.15	0.15
109.00	166.00	132.33	-1.01	0.98
363.00	435.00	442.00	1.05	0.51
602.00	628.00	619.00	-1.04	0.42
36.00	44.00	40.00	-1.01	0.95
571.00	564.00	560.00	-1.05	0.41
5.00	6.00	6.33	-1.07	1.00
163.00	160.00	167.00	-1.03	0.71
60.00	82.00	76.33	-1.13	0.28
136.00	155.00	155.33	1.02	0.87
70.00	105.00	95.00	-1.14	0.19
78.00	85.00	79.67	1.04	0.70
72.00	107.00	95.67	-1.18	0.09
16.00	17.00	14.33	-1.29	0.29
34.00	34.00	38.67	1.31	0.07
76.00	85.00	80.33	-1.16	0.14
20.00	19.00	16.00	-1.20	0.47
407.00	373.00	401.33	-1.02	0.69
16.00	15.00	18.67	1.10	0.71
91.00	79.00	95.67	-1.23	0.04
305.00	344.00	319.67	-1.04	0.53
371.00	346.00	361.67	1.12	0.06
553.00	518.00	568.00	-1.04	0.46
466.00	502.00	508.00	-1.01	0.88
50.00	50.00	53.00	-1.02	0.97
185.00	187.00	215.67	-1.04	0.68
92.00	85.00	93.67	-1.04	0.74
30.00	35.00	35.67	1.02	0.95
118.00	134.00	124.67	-1.01	0.90
865.00	921.00	837.33	-1.29	0.00
253.00	307.00	304.00	1.17	0.05
926.00	969.00	966.00	1.00	0.92

979.00	878.00	906.67	-1.14	0.02
533.00	565.00	561.67	1.20	0.00
397.00	330.00	363.33	-1.19	0.01
167.00	139.00	159.00	1.09	0.38
78.00	88.00	86.33	-1.03	0.84
75.00	82.00	86.33	-1.08	0.39
2573.00	2486.00	2629.00	-1.36	0.00
65.00	52.00	60.67	-1.13	0.29
67.00	88.00	85.33	1.09	0.42
401.00	408.00	409.00	-1.10	0.07
16.00	24.00	18.67	-1.03	1.00
90.00	87.00	81.33	1.06	0.62
39.00	46.00	41.33	-2.04	0.00
1648.00	1502.00	1523.67	-1.31	0.00
12.00	5.00	8.67	1.07	0.89
113.00	115.00	106.33	-1.36	0.00
69.00	61.00	65.67	1.12	0.34
236.00	249.00	247.00	1.07	0.29
105.00	128.00	125.33	-1.10	0.23
172.00	171.00	177.00	-1.06	0.45
360.00	315.00	342.67	-1.32	0.00
175.00	201.00	189.33	-1.06	0.42
121.00	146.00	128.33	-1.07	0.51
2036.00	2117.00	2075.33	-1.15	0.00
986.00	1107.00	1076.67	1.03	0.57
27.00	35.00	31.00	-1.36	0.03
128.00	125.00	128.67	1.02	0.84
22.00	18.00	18.67	-1.12	0.60
9.00	9.00	8.67	-1.01	1.00
13.00	17.00	17.00	-1.11	0.64
367.00	353.00	361.67	1.00	0.98
121.00	108.00	123.33	-1.02	0.89
845.00	853.00	858.33	1.03	0.50
3755.00	4372.00	4065.00	-1.03	0.49
875.00	991.00	996.33	1.10	0.04
113.00	82.00	103.67	-1.10	0.32
1347.00	1508.00	1394.33	1.14	0.01
928.00	812.00	873.00	-1.26	0.00
136.00	159.00	139.67	1.14	0.20
580.00	569.00	575.00	-1.17	0.00
55.00	68.00	68.67	-1.21	0.07
214.00	281.00	232.33	1.00	0.99
2633.00	3066.00	2892.33	-1.17	0.00
37.00	50.00	51.33	-1.30	0.04
23.00	27.00	24.33	1.03	0.87
20.00	26.00	21.00	1.24	0.28
2941.00	3090.00	3105.00	-1.02	0.63
300.00	346.00	323.00	-1.11	0.07

114.00	102.00	101.33	1.03	0.81
126.00	146.00	142.67	-1.02	0.83
91.00	124.00	111.33	1.14	0.18
577.00	649.00	651.67	-1.29	0.00
244.00	241.00	238.33	1.51	0.00
344.00	386.00	376.00	-1.02	0.70
87.00	98.00	88.67	1.16	0.16
354.00	392.00	359.33	1.14	0.04
105.00	144.00	125.33	1.16	0.11
20.00	31.00	23.67	-1.26	0.20
5.00	16.00	10.33	1.38	0.29
84.00	99.00	96.00	1.20	0.06
848.00	856.00	877.00	-1.09	0.12
218.00	248.00	239.00	-1.03	0.63
261.00	257.00	262.00	1.01	0.91
395.00	467.00	451.00	-1.04	0.44
1591.00	1697.00	1811.00	-1.11	0.05
76.00	78.00	72.33	1.45	0.00
639.00	600.00	629.33	-1.06	0.23
1249.00	1408.00	1342.33	-1.08	0.07
50.00	41.00	42.00	-1.30	0.05
972.00	1049.00	1064.33	-1.10	0.02
113.00	96.00	103.00	-1.38	0.00
601.00	761.00	745.33	1.01	0.91
154.00	153.00	148.67	-1.37	0.00
951.00	898.00	981.67	1.00	0.97
193.00	173.00	175.33	-1.11	0.21
9.00	7.00	8.00	-1.22	0.59
1123.00	1218.00	1265.67	1.09	0.06
103.00	142.00	126.33	1.10	0.29
4.00	8.00	8.00	-1.22	0.59
102.00	121.00	118.33	1.04	0.68
896.00	901.00	918.00	-1.06	0.17
38.00	55.00	42.00	1.34	0.07
29.00	25.00	26.00	1.24	0.23
201.00	266.00	225.67	1.12	0.21
199.00	245.00	229.67	-1.13	0.07
32.00	48.00	38.67	-1.04	0.82
142.00	139.00	156.67	1.17	0.08
566.00	653.00	581.67	-1.01	0.83
1.00	0.00	1.00	-1.61	0.73
6.00	3.00	5.00	1.46	0.43
149.00	217.00	181.33	1.08	0.39
3.00	2.00	7.00	1.83	0.25
111.00	103.00	103.00	1.06	0.57
423.00	514.00	492.67	1.02	0.75
1117.00	1186.00	1185.00	-1.02	0.56
727.00	759.00	793.67	-1.02	0.67

214.00	192.00	200.67	-1.11	0.14
130.00	134.00	126.33	-1.13	0.15
201.00	228.00	204.67	1.03	0.67
147.00	153.00	168.33	-1.16	0.08
874.00	949.00	954.33	-1.08	0.09
466.00	497.00	486.33	-1.07	0.18
10.00	13.00	9.67	-1.43	0.20
206.00	234.00	227.00	1.00	0.96
86.00	121.00	91.67	1.02	0.89
238.00	291.00	264.67	1.05	0.49
819.00	1027.00	948.00	-1.16	0.00
2109.00	2019.00	2180.00	1.22	0.00
134.00	130.00	128.00	-1.06	0.50
1724.00	2037.00	1860.67	-1.01	0.80
172.00	164.00	160.67	1.27	0.01
51.00	49.00	45.33	1.03	0.82
176.00	168.00	166.67	1.11	0.17
37.00	27.00	35.00	-1.00	1.00
175.00	158.00	181.33	-1.02	0.79
328.00	328.00	329.33	-1.13	0.03
566.00	591.00	593.33	-1.10	0.03
275.00	243.00	260.00	-1.18	0.01
257.00	300.00	280.33	-1.16	0.01
506.00	488.00	517.33	-1.07	0.20
3.00	12.00	7.33	1.20	0.65
131.00	130.00	142.33	-1.11	0.20
2422.00	2398.00	2465.00	-1.17	0.00
54.00	39.00	46.33	-1.04	0.83
342.00	358.00	339.00	-1.28	0.00
371.00	377.00	379.00	-1.15	0.01
688.00	754.00	771.00	1.07	0.20
11.00	17.00	14.33	1.62	0.06
344.00	379.00	358.00	-1.13	0.03
109.00	101.00	127.00	-1.08	0.50
347.00	378.00	352.00	-1.24	0.00
103.00	102.00	97.33	-1.65	0.00
38.00	46.00	48.33	1.31	0.10
142.00	132.00	146.33	-1.12	0.14
525.00	618.00	563.00	1.05	0.42
457.00	504.00	489.00	-1.03	0.65
180.00	147.00	172.33	-1.23	0.01
98.00	94.00	86.67	-1.16	0.22
173.00	174.00	178.00	-1.24	0.01
9.00	5.00	5.33	1.31	0.59
99.00	111.00	104.33	-1.22	0.02
26.00	25.00	29.00	1.08	0.72
11.00	13.00	13.67	1.06	0.83
55.00	83.00	70.33	-1.01	0.93

82.00	112.00	112.67	1.08	0.48
806.00	837.00	827.67	-1.11	0.02
1362.00	1552.00	1525.33	-1.27	0.00
389.00	462.00	449.33	1.07	0.23
13.00	21.00	20.33	1.25	0.32
42.00	25.00	35.00	-1.01	0.95
47.00	36.00	45.00	1.26	0.12
944.00	1152.00	1096.33	1.07	0.16
1144.00	1324.00	1282.67	-1.00	0.92
399.00	486.00	434.33	-1.11	0.10
3.00	3.00	2.33	-1.70	0.37
138.00	185.00	170.67	1.04	0.66
54.00	57.00	53.00	-1.23	0.08
4.00	14.00	9.67	1.14	0.70
89.00	84.00	77.33	-1.30	0.04
14.00	10.00	10.33	1.27	0.43
970.00	940.00	992.00	-1.35	0.00
420.00	424.00	425.00	-1.09	0.12
6.00	4.00	5.67	-1.48	0.30
67.00	66.00	68.67	1.08	0.49
175.00	191.00	190.67	1.02	0.81
67.00	83.00	73.67	-1.03	0.76
33.00	28.00	35.00	1.11	0.55
109.00	98.00	102.33	-1.23	0.02
252.00	334.00	306.00	1.13	0.12
31.00	44.00	37.67	-1.01	0.95
2066.00	2075.00	2055.00	-1.15	0.00
657.00	645.00	694.67	1.13	0.02
428.00	439.00	469.00	1.16	0.01
2107.00	2378.00	2294.67	1.17	0.00
58.00	57.00	62.33	1.17	0.21
1.00	8.00	4.67	-1.87	0.13
319.00	311.00	304.00	-1.01	0.90
96.00	115.00	97.33	1.10	0.38
25.00	22.00	21.00	1.22	0.32
8.00	11.00	8.33	1.23	0.56
595.00	559.00	559.33	-1.04	0.56
21.00	19.00	17.33	-2.48	0.00
63.00	57.00	67.00	1.48	0.00
6.00	6.00	6.00	-2.72	0.00
1136.00	1164.00	1194.67	1.02	0.67
1250.00	1362.00	1310.33	-1.03	0.54
3920.00	4729.00	4194.67	1.03	0.53
3.00	5.00	3.67	-1.01	1.00
314.00	340.00	339.67	1.06	0.33
436.00	472.00	482.33	-1.04	0.49
489.00	498.00	492.00	1.01	0.82
80.00	57.00	71.00	1.03	0.84

106.00	108.00	109.67	1.09	0.38
102.00	117.00	107.33	1.03	0.75
153.00	195.00	179.00	-1.06	0.42
235.00	227.00	236.00	-1.14	0.04
180.00	201.00	201.67	-1.23	0.00
122.00	102.00	120.67	-1.09	0.36
47.00	71.00	54.67	-1.32	0.03
333.00	386.00	364.67	-1.15	0.01
496.00	529.00	543.67	1.04	0.43
1137.00	1118.00	1105.67	-1.16	0.00
1047.00	1252.00	1187.33	-1.25	0.00
6.00	27.00	21.33	-1.35	0.19
452.00	562.00	533.00	-1.04	0.50
19.00	24.00	19.67	1.10	0.65
402.00	490.00	468.00	-1.19	0.00
206.00	197.00	186.67	-1.06	0.52
173.00	170.00	172.33	1.09	0.24
490.00	479.00	514.33	1.14	0.01
174.00	169.00	170.33	-1.23	0.01
1285.00	1409.00	1391.33	-1.50	0.00
231.00	236.00	259.00	1.51	0.00
1938.00	1894.00	1952.00	-1.31	0.00
190.00	212.00	207.33	-1.26	0.00
376.00	436.00	414.00	1.08	0.15
716.00	769.00	752.67	1.12	0.04
938.00	1090.00	1058.67	1.13	0.01
1221.00	1523.00	1441.00	1.38	0.00
82.00	76.00	78.00	-1.12	0.32
283.00	256.00	276.67	-1.13	0.10
786.00	931.00	868.00	-1.20	0.00
0.00	1.00	0.33	-2.48	0.64
600.00	647.00	645.67	-1.08	0.15
5345.00	5367.00	5447.00	-1.05	0.14
1661.00	1657.00	1659.33	-1.25	0.00
2563.00	2829.00	2730.33	1.08	0.07

FDR p-value correction
0.31
0.13
0.07
0.97
0.34
0.00
1.00
1.00
0.16
0.00
0.00
0.00
0.81
0.89
0.84
0.95
0.13
0.15
0.55
0.77
1.00
0.00
0.11
1.00
0.14
0.00
0.27
0.30
0.88
0.15
0.68
1.00
1.00
0.00
0.91
1.00
0.07
0.00
0.40
1.00
0.46
0.00
0.13

0.00
0.39
1.00
1.00
0.00
1.00
0.00
0.24
0.88
0.24
0.00
0.02
1.00
1.00
1.00
0.97
0.42
0.00
0.45
0.01
1.00
0.54
1.00
0.25
1.00
0.85
0.00
0.00
0.00
0.00
0.09
1.00
0.15
0.66
0.43
0.00
0.00
0.94
0.48
0.77
0.00
0.08
0.00
0.27
0.00
0.87
0.00
0.45

0.74
0.07
0.77
0.00
1.00
0.64
0.00
0.95
0.53
0.39
1.00
0.00
0.00
0.00
0.02
0.73
0.11
0.68
1.00
0.52
1.00
0.93
0.00
1.00
0.00
1.00
0.16
0.15
0.14
0.00
0.64
0.00
1.00
0.52
1.00
0.00
0.94
0.03
0.05
0.03
0.04
0.56
1.00
1.00
0.00
0.26
0.62
0.40

0.60
0.00
1.00
0.00
0.61
1.00
0.00
0.18
0.88
0.00
0.70
1.00
0.00
0.00
1.00
1.00
0.04
1.00
0.52
0.00
0.02
0.19
1.00
0.82
0.08
0.95
1.00
1.00
0.59
0.41
1.00
1.00
0.70
1.00
0.06
0.01
1.00
0.30
0.01
0.94
0.95
0.00
0.00
0.21
0.00
1.00
0.44
0.56

1.00
0.41
0.45
0.00
0.26
0.01
0.00
0.00
0.00
0.08
0.00
1.00
0.00
0.35
0.00
1.00
0.09
0.02
0.06
1.00
1.00
0.34
0.00
0.13
0.00
1.00
0.03
0.22
0.59
0.47
0.00
0.14
0.49
0.71
0.92
0.05
0.26
1.00
1.00
1.00
0.00
1.00
0.00
1.00
1.00
0.30
0.02
0.89

0.00
0.16
0.07
1.00
1.00
0.00
0.19
0.00
0.03
0.00
0.00
0.03
0.03
1.00
1.00
0.00
0.25
0.38
0.00
1.00
0.06
0.95
0.14
1.00
0.25
0.00
0.23
0.84
1.00
0.32
0.16
0.00
0.49
1.00
1.00
0.72
1.00
0.21
0.00
1.00
0.01
0.00
0.00
0.08
0.14
0.06
0.00
0.00

0.89
0.32
0.04
1.00
0.00
0.00
0.49
1.00
0.25
1.00
0.00
0.72
0.88
1.00
1.00
0.78
0.59
1.00
1.00
0.04
1.00
0.13
0.08
0.61
1.00
0.34
1.00
0.86
0.79
0.55
0.00
1.00
0.23
1.00
0.80
0.16
0.31
1.00
0.00
0.75
0.41
0.77
0.00
0.00
0.00
1.00
0.00
0.00

1.00
0.72
1.00
0.73
0.00
0.24
0.00
0.00
0.20
0.00
0.02
0.03
0.00
0.00
0.02
0.42
1.00
0.36
0.42
0.21
0.00
0.18
0.00
0.10
0.87
0.09
0.00
1.00
0.43
0.44
1.00
0.78
0.17
0.28
0.03
1.00
1.00
0.00
0.44
0.05
1.00
0.76
0.11
0.00
0.12
0.02
0.00
1.00

0.07
0.82
0.54
0.24
0.11
0.13
0.20
1.00
0.49
1.00
1.00
0.03
0.16
0.89
1.00
0.24
0.14
0.35
0.42
0.57
1.00
0.27
0.07
0.51
0.00
0.08
1.00
0.98
1.00
0.03
0.00
0.00
0.13
0.05
0.00
0.62
0.00
0.00
0.54
1.00
1.00
0.87
0.10
0.95
0.00
0.00
1.00
1.00

0.67
0.00
0.01
1.00
0.00
0.76
0.00
0.36
0.39
0.97
1.00
0.04
0.19
1.00
1.00
0.54
1.00
0.02
0.51
0.01
0.28
1.00
0.70
1.00
0.97
0.00
1.00
0.00
0.52
1.00
0.25
0.92
0.01
1.00
0.00
0.50
1.00
0.31
1.00
0.01
0.00
0.00
0.86
0.00
0.06
1.00
1.00
0.49

0.38
0.00
0.01
0.15
0.04
0.09
0.00
0.16
1.00
0.50
1.00
0.00
0.07
0.00
0.00
0.05
0.00
1.00
1.00
0.27
1.00
0.64
0.01
0.52
0.42
0.60
0.04
1.00
1.00
1.00
1.00
0.66
0.00
0.00
1.00
1.00
0.02
0.05
0.00
1.00
0.25
0.12
0.54
1.00
1.00
0.13
0.64
0.16

0.77
0.01
0.00
0.00
0.89
0.15
0.38
0.00
0.01
0.00
1.00
1.00
0.54
1.00
0.00
1.00
1.00
1.00
0.00
1.00
1.00
0.50
0.09
0.00
0.00
0.61
0.74
0.69
1.00
0.91
0.46
0.00
0.74
0.49
0.01
0.00
1.00
0.00
1.00
0.00
0.48
0.00
0.14
0.00
0.00
0.00
0.00
1.00

0.00
0.00
0.01
0.01
0.32
0.00
0.01
1.00
0.64
0.19
0.00
1.00
0.00
1.00
0.02
0.51
0.27
0.75
0.75
1.00
0.00
0.01
0.09
0.04
0.00
1.00
0.81
0.03
0.45
0.06
0.45
0.37
0.91
0.51
0.07
1.00
0.38
1.00
0.00
1.00
0.40
0.38
1.00
1.00
0.53
1.00
1.00
0.25

0.51
0.28
0.05
0.00
0.78
0.57
0.26
0.00
0.67
0.07
1.00
1.00
0.00
0.00
1.00
1.00
0.00
0.45
0.25
0.00
0.01
0.00
0.01
0.00
1.00
0.40
1.00
0.56
0.10
0.39
0.05
1.00
1.00
0.01
0.52
0.09
0.02
0.76
0.04
1.00
0.00
0.33
0.39
0.89
1.00
1.00
0.39
1.00

0.00
1.00
0.33
1.00
0.02
0.33
0.11
0.54
1.00
0.18
0.73
0.82
0.09
0.01
0.00
0.19
0.11
0.87
0.78
0.30
1.00
0.41
0.87
0.26
0.34
0.05
0.68
1.00
0.11
0.25
0.00
0.88
0.00
0.19
0.00
0.05
0.01
0.00
1.00
0.00
0.61
1.00
0.00
1.00
1.00
0.00
0.65
0.00

0.00
0.12
1.00
1.00
0.36
1.00
0.16
0.71
0.00
0.00
0.11
0.14
0.00
0.03
0.00
0.00
0.00
0.00
1.00
0.20
0.03
0.00
1.00
1.00
0.01
0.00
0.00
0.97
0.36
0.00
0.30
0.93
0.18
0.00
1.00
1.00
0.00
0.00
0.78
0.31
1.00
0.02
0.00
0.13
0.14
0.88
0.32
0.89

1.00
0.27
0.77
0.66
0.09
0.15
0.10
0.00
1.00
1.00
0.24
0.52
0.00
0.26
1.00
0.00
1.00
0.05
0.11
1.00
0.43
0.00
0.06
0.40
0.14
1.00
0.12
1.00
0.27
0.57
0.04
1.00
0.14
0.21
0.00
0.10
0.82
0.01
0.00
0.00
0.00
0.00
0.00
0.00
0.00
0.00
0.65
1.00

0.18
0.51
0.00
1.00
0.77
1.00
0.01
1.00
1.00
0.00
1.00
0.00
0.27
0.00
0.00
0.30
0.70
0.29
0.71
0.00
1.00
0.34
0.00
1.00
0.14
0.00
0.00
1.00
0.42
1.00
0.91
1.00
1.00
0.00
1.00
0.74
1.00
0.01
1.00
0.00
0.00
1.00
0.17
0.26
1.00
0.00
0.68
1.00

0.70
0.25
0.01
0.84
0.20
1.00
0.73
0.65
1.00
0.13
0.11
0.83
0.99
0.04
0.00
0.25
0.73
0.37
0.00
1.00
0.00
0.52
0.13
1.00
0.21
0.17
1.00
1.00
0.10
0.42
0.02
1.00
0.00
0.70
1.00
0.18
0.47
0.15
0.00
0.93
0.84
0.07
0.00
0.04
0.00
0.00
0.19
0.46

0.17
0.00
0.00
0.07
0.36
0.06
0.01
1.00
0.15
0.94
0.00
0.23
0.00
0.00
0.11
0.75
0.00
0.00
0.04
0.66
0.01
0.00
1.00
1.00
0.00
1.00
0.00
0.00
0.54
0.15
0.01
0.40
0.00
0.00
0.00
1.00
0.36
0.00
0.59
0.00
0.01
0.25
0.00
0.05
0.04
0.00
0.00
0.35

0.00
0.00
0.00
0.00
0.25
1.00
0.59
0.99
0.11
0.00
0.39
0.72
0.67
0.36
0.74
0.84
0.57
0.15
0.00
0.68
1.00
0.00
1.00
0.01
0.52
0.01
0.04
0.00
0.53
0.00
0.00
0.49
0.83
0.00
0.00
0.04
0.84
0.03
0.36
0.02
0.15
0.17
0.41
0.63
0.69
0.01
1.00
0.17

0.00
0.23
0.00
1.00
0.00
0.90
1.00
0.13
0.15
0.02
1.00
1.00
0.28
0.84
0.00
0.01
1.00
0.00
1.00
0.81
0.89
0.04
1.00
1.00
0.00
1.00
0.57
1.00
0.00
0.70
0.42
0.00
0.73
1.00
0.00
0.13
0.00
0.94
0.26
0.84
0.46
0.84
0.97
0.21
1.00
0.21
0.64
0.00

0.00
1.00
0.01
0.54
0.78
0.90
0.19
1.00
0.89
0.00
0.15
0.00
1.00
0.01
1.00
1.00
0.08
0.00
0.14
0.02
0.48
0.16
0.17
1.00
0.11
0.00
0.01
0.14
0.70
1.00
0.14
1.00
0.98
0.18
1.00
0.00
0.72
0.39
0.01
0.01
1.00
0.05
0.08
0.24
1.00
0.01
0.57
1.00

1.00
1.00
0.80
0.00
0.01
0.75
0.25
0.04
0.31
1.00
0.00
0.36
0.39
1.00
0.93
0.88
0.00
0.20
0.93
0.00
1.00
0.54
0.04
0.03
1.00
1.00
1.00
0.97
1.00
1.00
0.01
1.00
0.16
0.61
0.02
0.10
0.00
0.00
0.05
0.02
0.01
0.66
0.52
1.00
0.41
1.00
0.77
0.00

1.00
0.00
1.00
0.00
0.23
0.02
0.00
0.01
0.64
1.00
1.00
0.12
0.53
1.00
0.60
0.25
0.00
0.05
1.00
0.10
1.00
0.18
0.41
0.00
0.01
0.00
1.00
0.05
0.09
0.66
0.35
0.64
1.00
1.00
0.23
0.00
1.00
0.10
1.00
0.63
0.08
0.00
1.00
0.23
0.03
1.00
1.00
1.00

0.00
0.00
0.00
0.13
0.78
0.00
0.63
1.00
0.41
0.03
0.45
0.73
0.01
0.41
0.82
0.02
0.00
1.00
1.00
0.00
1.00
0.00
0.84
0.07
1.00
1.00
0.00
0.00
0.28
0.02
0.01
0.77
0.90
0.08
0.00
0.00
0.37
0.46
0.00
0.00
0.00
0.00
0.88
0.39
0.00
0.01
0.05
0.10

1.00
0.07
1.00
0.00
0.00
0.07
0.63
0.01
0.01
0.85
0.39
1.00
1.00
0.64
1.00
0.09
0.30
0.57
0.05
0.28
0.00
1.00
1.00
1.00
0.00
0.14
0.39
0.07
0.18
0.00
0.34
0.00
0.00
1.00
0.18
0.03
0.94
0.20
0.00
0.01
1.00
0.00
1.00
0.25
0.07
0.00
0.19
1.00

1.00
0.00
0.00
0.02
0.29
0.00
0.00
0.65
0.06
0.00
0.11
0.19
1.00
0.01
0.00
0.00
0.00
0.00
0.68
1.00
0.00
1.00
1.00
0.01
0.00
1.00
0.00
0.00
1.00
1.00
0.03
0.08
1.00
1.00
0.46
0.35
0.00
0.00
0.08
1.00
0.01
0.15
1.00
0.49
0.95
0.64
1.00
1.00

0.00
1.00
1.00
0.51
1.00
0.09
0.15
0.48
1.00
0.84
0.34
0.00
1.00
1.00
0.07
0.00
0.69
1.00
0.03
1.00
1.00
1.00
0.43
0.00
1.00
0.00
1.00
0.20
1.00
0.02
0.97
0.39
1.00
1.00
1.00
0.57
1.00
0.00
1.00
1.00
0.36
0.23
0.82
0.01
1.00
0.00
0.77
1.00

1.00
1.00
0.05
0.95
0.61
0.41
1.00
0.07
1.00
0.00
0.26
0.42
1.00
0.00
0.74
0.02
0.95
0.26
0.14
0.09
0.00
1.00
0.26
0.00
1.00
0.00
1.00
1.00
1.00
0.71
0.02
0.59
0.00
0.96
0.00
0.89
0.56
0.40
0.00
1.00
0.90
0.00
1.00
0.51
0.03
0.00
0.29
1.00

1.00
0.02
0.02
0.80
0.57
0.01
0.54
0.12
0.01
0.51
0.09
0.05
1.00
0.00
0.01
1.00
0.85
0.93
0.41
0.00
0.21
0.54
1.00
0.03
0.37
0.00
0.00
1.00
1.00
0.30
0.99
1.00
0.13
1.00
0.57
0.03
0.33
0.04
0.25
0.84
0.27
1.00
1.00
0.00
1.00
0.41
0.51
1.00

1.00
0.34
0.00
0.02
0.00
1.00
0.27
0.48
1.00
0.14
0.00
0.29
0.15
1.00
0.03
0.00
0.01
0.04
0.16
0.06
1.00
0.04
0.86
1.00
0.05
1.00
0.01
0.00
0.00
0.57
0.68
0.02
1.00
1.00
0.25
1.00
1.00
0.08
0.05
0.31
1.00
0.93
0.16
1.00
0.04
0.16
0.99
0.57

1.00
0.00
0.00
1.00
0.38
0.04
0.57
1.00
1.00
0.00
0.70
0.72
0.31
0.11
0.39
0.00
1.00
0.03
1.00
0.67
0.00
1.00
1.00
0.01
1.00
0.04
0.00
0.01
0.01
1.00
0.01
0.12
0.00
0.17
0.00
0.01
1.00
0.05
0.23
0.52
1.00
1.00
0.00
0.00
1.00
0.28
1.00
1.00

0.00
0.82
0.00
0.14
0.17
1.00
0.02
1.00
0.42
0.82
0.97
0.00
0.89
0.31
1.00
0.29
0.00
1.00
0.07
0.61
0.73
0.53
1.00
0.81
0.01
0.48
1.00
0.35
0.20
1.00
1.00
0.13
0.73
0.00
0.18
1.00
1.00
0.51
1.00
0.00
0.37
1.00
1.00
0.53
0.01
0.24
0.52
0.71

0.06
1.00
0.40
0.02
0.58
0.40
0.42
1.00
1.00
0.33
1.00
1.00
0.40
0.00
0.32
1.00
1.00
0.68
0.80
0.00
0.02
0.12
0.02
0.44
0.00
0.00
0.46
1.00
0.02
0.00
0.01
0.41
1.00
0.25
1.00
0.00
0.61
0.25
0.00
1.00
0.00
0.04
0.00
0.00
0.51
0.25
0.67
0.55

1.00
0.00
0.96
0.57
1.00
1.00
0.37
0.50
0.11
0.85
1.00
1.00
0.56
1.00
0.98
0.00
1.00
0.40
0.00
0.59
0.02
0.04
0.40
0.10
0.01
0.23
0.26
0.01
0.42
0.00
0.01
0.02
1.00
0.06
1.00
0.00
1.00
0.00
0.00
0.70
0.50
0.01
0.00
1.00
0.10
0.90
0.00
0.42

0.52
0.04
1.00
1.00
0.15
1.00
0.61
1.00
1.00
1.00
0.41
0.25
0.74
1.00
1.00
1.00
0.56
0.61
0.00
1.00
1.00
1.00
0.32
1.00
0.92
1.00
0.00
0.40
0.51
0.68
1.00
1.00
1.00
1.00
0.00
0.00
0.00
0.00
0.00
0.06
0.61
0.26
0.77
0.09
0.00
1.00
0.03
1.00

1.00
1.00
1.00
1.00
1.00
0.01
0.16
0.00
1.00
1.00
0.01
0.16
0.32
0.23
0.00
0.12
0.00
0.47
1.00
1.00
0.00
1.00
0.11
1.00
0.69
0.67
0.15
1.00
0.78
0.00
0.38
0.00
0.81
0.51
0.93
0.15
0.02
1.00
0.25
0.33
0.07
0.83
0.63
1.00
0.00
1.00
0.00
0.06

0.04
0.65
0.00
0.07
0.02
0.00
0.04
0.00
0.00
0.84
0.61
0.01
0.01
0.72
0.57
0.28
0.00
0.83
1.00
1.00
0.02
1.00
0.25
1.00
0.46
1.00
1.00
0.12
0.00
0.02
1.00
1.00
1.00
0.34
1.00
0.61
0.81
0.00
1.00
1.00
0.41
0.01
0.06
1.00
0.00
0.89
0.12
0.00

0.00
0.01
0.03
0.02
1.00
0.24
0.06
0.30
0.23
1.00
0.00
0.00
0.32
0.00
0.85
1.00
0.00
0.10
0.51
1.00
0.00
1.00
1.00
0.32
0.38
0.00
0.06
0.02
0.14
0.25
1.00
0.00
0.06
0.23
0.84
0.28
0.01
1.00
0.00
0.50
0.26
0.35
0.00
0.00
0.94
0.00
1.00
0.00

0.83
0.01
0.95
0.31
0.00
0.01
1.00
1.00
0.00
0.25
0.95
0.66
1.00
1.00
0.00
1.00
1.00
1.00
0.14
0.03
0.00
1.00
0.01
0.00
0.05
0.77
0.01
0.48
0.06
1.00
0.36
0.04
0.00
0.00
0.00
0.01
0.26
0.13
0.83
0.00
0.00
0.01
0.03
1.00
0.70
0.30
0.10
0.15

0.00
0.00
1.00
0.12
1.00
0.28
0.11
0.00
0.00
1.00
0.22
0.54
0.40
0.00
1.00
1.00
1.00
0.00
0.01
0.00
0.16
0.31
0.00
0.00
0.14
1.00
0.00
0.40
0.21
0.00
0.00
0.04
0.89
0.35
0.66
1.00
0.00
1.00
0.00
0.00
1.00
1.00
0.91
1.00
0.00
0.14
0.74
0.00

0.82
0.01
1.00
0.53
0.52
0.13
1.00
1.00
0.01
0.21
0.71
0.00
0.18
0.00
0.84
0.42
0.40
0.05
0.00
0.36
0.00
0.00
0.05
0.09
0.00
0.00
0.00
0.00
0.06
0.00
0.15
1.00
0.39
0.00
1.00
1.00
0.00
0.00
0.28
0.00
0.00
0.00
0.24
0.00
0.24
0.26
1.00
0.59

0.05
0.15
0.01
0.79
0.08
1.00
1.00
0.00
0.57
0.02
1.00
0.00
0.70
0.62
0.68
0.55
1.00
0.60
0.22
0.18
0.04
0.69
0.73
0.07
0.37
0.77
1.00
0.13
0.00
0.02
0.81
0.40
0.01
0.00
0.02
0.01
0.59
0.00
0.22
1.00
1.00
0.79
0.17
0.19
1.00
0.57
0.00
0.00

0.33
1.00
0.82
0.00
0.00
1.00
0.91
0.34
0.41
0.00
0.05
1.00
0.77
0.69
0.42
0.54
0.14
0.00
0.00
0.45
0.01
0.11
1.00
0.27
1.00
0.21
0.37
0.32
0.00
0.02
0.10
0.01
0.02
0.00
0.00
0.22
0.07
0.01
1.00
0.00
0.06
0.03
0.33
0.01
0.24
1.00
1.00
1.00

0.09
0.00
0.15
1.00
1.00
0.00
0.89
1.00
0.48
0.00
0.93
0.12
0.45
0.33
1.00
0.00
0.64
0.65
0.29
1.00
1.00
1.00
0.11
0.75
0.26
1.00
0.00
0.38
0.09
0.16
1.00
0.62
1.00
0.02
1.00
1.00
1.00
0.00
0.94
0.15
0.00
0.95
0.00
1.00
1.00
0.00
0.00
0.07

0.00
0.26
0.10
0.01
0.00
1.00
0.18
0.00
0.80
0.00
1.00
0.08
0.12
1.00
1.00
1.00
1.00
1.00
0.85
1.00
0.95
1.00
1.00
1.00
1.00
0.24
0.00
1.00
0.33
0.01
0.00
0.01
0.23
0.16
0.54
0.82
0.45
0.00
1.00
0.00
1.00
0.00
1.00
0.00
0.09
0.00
0.00
0.96

0.52
0.00
1.00
1.00
1.00
0.98
1.00
0.83
1.00
0.62
0.16
0.25
0.97
1.00
1.00
0.04
0.47
0.15
0.30
0.76
0.00
0.54
0.02
0.00
1.00
0.12
0.02
0.00
0.00
0.02
0.02
1.00
1.00
1.00
0.00
0.03
0.11
0.00
0.34
1.00
0.16
0.20
0.77
0.00
0.01
0.01
0.92
0.82

0.08
1.00
1.00
1.00
0.58
0.09
0.02
0.64
1.00
0.42
0.00
1.00
0.00
0.72
0.71
0.00
0.45
1.00
0.01
0.07
1.00
1.00
0.35
0.63
0.05
0.00
1.00
0.46
1.00
0.00
0.00
0.15
0.00
0.09
0.00
0.83
0.04
0.11
1.00
0.00
0.00
0.17
1.00
0.34
0.00
0.03
0.00
0.04

0.11
0.00
0.84
0.00
0.01
0.00
0.03
0.06
0.73
0.00
0.13
0.63
0.26
1.00
0.44
0.77
1.00
0.01
0.57
0.02
0.07
0.46
1.00
0.00
0.68
0.00
1.00
1.00
0.00
0.68
0.97
1.00
0.00
1.00
0.46
0.00
0.00
1.00
1.00
0.38
1.00
0.57
0.60
0.00
1.00
0.10
0.64
0.21

0.09
0.74
1.00
1.00
0.01
0.27
0.77
0.80
0.00
0.00
0.89
0.00
0.03
0.53
1.00
0.00
0.01
0.03
0.24
1.00
0.83
1.00
1.00
0.02
0.02
0.02
0.00
1.00
0.00
0.05
0.00
1.00
0.04
0.04
0.51
0.84
1.00
0.00
1.00
0.29
0.02
1.00
0.43
0.01
0.62
0.00
1.00
0.00

0.00
0.08
0.59
0.79
0.35
0.67
0.00
0.39
0.53
0.02
0.41
0.35
0.07
0.35
0.94
0.05
0.22
0.25
1.00
0.21
0.94
0.99
0.06
0.59
0.37
0.38
0.03
0.00
0.00
1.00
1.00
0.05
0.00
1.00
0.00
0.00
0.01
0.00
1.00
1.00
0.01
0.26
1.00
0.03
0.98
0.11
0.70
1.00

0.00
1.00
0.01
1.00
0.51
1.00
1.00
0.31
0.19
0.05
0.61
0.00
0.00
1.00
0.01
0.64
0.78
0.00
1.00
0.00
0.88
0.68
0.17
0.07
0.00
0.00
0.63
1.00
0.82
0.00
0.00
0.00
0.23
0.69
0.94
0.00
0.00
0.01
1.00
0.49
0.88
0.68
0.27
0.01
1.00
1.00
1.00
0.00

0.18
1.00
0.08
0.72
0.01
1.00
0.16
0.34
0.01
0.81
1.00
0.64
0.41
0.16
0.00
0.00
0.00
0.89
0.05
0.00
1.00
1.00
1.00
0.37
0.53
0.74
0.07
0.01
0.00
1.00
0.08
1.00
0.94
0.94
1.00
1.00
0.97
0.05
0.62
0.00
1.00
0.00
0.55
0.68
1.00
0.72
0.30
0.12

0.01
0.91
1.00
0.54
0.00
0.00
0.00
0.00
0.21
0.89
0.00
0.05
0.16
0.00
0.05
0.01
0.00
0.00
0.94
1.00
0.00
1.00
0.69
1.00
1.00
0.26
0.21
0.11
0.00
1.00
0.00
1.00
0.00
1.00
0.01
0.33
0.00
0.00
0.21
0.06
1.00
0.92
0.06
0.01
1.00
0.06
0.54
0.55

0.03
0.01
0.29
1.00
0.00
0.04
0.00
1.00
0.77
0.05
0.00
0.27
0.02
1.00
0.68
0.12
0.42
0.00
0.55
1.00
0.00
0.02
0.23
0.52
0.74
0.01
0.01
0.00
1.00
0.39
1.00
0.44
0.00
1.00
0.63
0.00
1.00
1.00
0.00
0.88
1.00
0.02
0.01
1.00
0.79
0.00
0.00
0.50

0.06
0.27
0.29
0.45
0.00
0.00
0.13
1.00
0.91
0.63
1.00
1.00
0.44
0.23
0.00
1.00
1.00
0.03
1.00
0.53
0.00
1.00
0.61
0.79
0.00
0.00
0.96
0.22
0.00
1.00
0.40
0.00
1.00
0.00
0.00
0.00
1.00
0.00
1.00
0.00
0.08
1.00
1.00
0.00
0.49
1.00
0.00
0.15

0.00
0.00
0.88
0.03
1.00
0.35
0.12
0.03
0.05
0.19
0.18
1.00
0.44
1.00
1.00
1.00
0.83
0.97
1.00
1.00
1.00
0.10
0.28
1.00
1.00
0.43
1.00
0.35
0.13
0.02
0.02
1.00
0.00
0.02
0.61
0.02
0.68
0.00
0.00
1.00
0.09
0.08
0.02
1.00
0.00
0.11
1.00
0.96

0.03
0.41
0.90
0.00
0.02
0.01
0.00
0.00
0.42
0.21
1.00
0.00
0.03
0.03
0.23
0.61
0.20
0.37
0.00
0.08
0.30
0.49
0.84
0.30
0.07
0.69
1.00
0.33
0.01
0.00
0.82
0.48
0.00
0.17
1.00
0.00
0.09
0.57
0.00
0.00
0.00
0.00
0.00
0.27
0.54
1.00
1.00
0.04

0.91
1.00
0.47
1.00
1.00
0.00
0.00
1.00
0.01
0.05
0.10
0.45
0.68
1.00
0.02
0.04
0.02
0.32
0.49
0.00
0.00
0.00
0.67
0.00
0.00
0.19
0.45
0.22
0.03
0.01
0.44
0.08
1.00
0.00
0.64
0.01
0.58
0.89
0.61
0.88
0.02
1.00
0.57
1.00
1.00
0.00
0.00
0.24

1.00
0.00
1.00
0.00
0.36
1.00
1.00
0.12
0.48
0.43
0.03
0.00
0.00
1.00
0.91
0.56
0.00
1.00
0.12
0.08
0.00
0.70
0.00
1.00
0.00
0.10
0.00
1.00
0.00
0.00
1.00
0.00
0.51
1.00
0.00
1.00
0.20
0.96
0.29
0.60
0.07
0.00
1.00
0.13
0.30
0.32
1.00
0.64

0.00
0.01
1.00
0.00
1.00
0.44
0.18
0.14
0.83
1.00
0.00
0.75
0.00
1.00
0.00
0.00
0.09
0.14
0.82
0.18
1.00
0.07
1.00
0.30
0.08
1.00
0.18
0.98
0.00
0.02
0.90
0.01
0.03
1.00
1.00
0.00
1.00
0.02
0.00
0.31
0.00
0.00
0.99
0.21
0.06
0.18
0.31
0.00

1.00
0.53
0.02
0.62
0.04
0.01
0.00
0.07
1.00
1.00
0.42
1.00
0.78
0.00
1.00
0.00
0.36
0.12
1.00
1.00
0.16
0.33
0.51
0.52
0.00
1.00
1.00
1.00
0.99
1.00
0.12
0.13
0.00
0.28
0.00
0.06
1.00
1.00
0.83
0.54
0.92
0.00
0.74
0.54
0.07
0.03
1.00
0.05

1.00
0.83
0.03
0.33
0.00
0.87
0.41
0.89
0.00
1.00
1.00
0.26
0.00
0.11
0.00
0.09
1.00
1.00
0.00
0.00
1.00
0.00
1.00
1.00
0.11
0.65
0.00
0.68
1.00
0.03
0.00
1.00
1.00
0.01
1.00
0.37
0.27
0.62
0.21
0.69
1.00
0.08
0.14
0.09
1.00
0.08
0.00
1.00

0.00
0.05
0.42
0.00
1.00
0.00
0.00
0.04
0.01
1.00
0.16
0.02
0.00
1.00
0.56
0.13
0.00
1.00
0.06
0.13
1.00
0.00
0.00
0.25
0.40
0.04
0.60
1.00
1.00
0.94
0.00
0.64
0.83
0.47
0.58
1.00
0.42
0.98
0.50
0.01
0.13
1.00
0.40
1.00
0.03
0.91
0.18
0.00

1.00
0.03
0.85
0.00
0.86
0.30
0.07
1.00
1.00
1.00
0.39
1.00
0.03
0.66
0.79
0.00
0.12
0.03
0.00
0.74
0.36
1.00
0.98
0.70
0.00
0.07
1.00
0.02
1.00
0.90
0.82
0.85
0.08
0.40
0.09
0.35
1.00
0.50
1.00
0.00
0.06
0.00
0.60
0.00
0.72
1.00
0.82
0.09

0.00
0.12
0.74
0.11
1.00
0.35
0.00
0.00
0.56
0.21
0.90
0.02
0.09
1.00
0.00
1.00
1.00
1.00
0.00
0.00
0.41
1.00
0.26
0.03
0.92
1.00
0.02
0.20
1.00
0.22
1.00
0.00
0.54
0.74
1.00
0.00
0.41
0.00
1.00
0.11
1.00
0.22
0.43
1.00
0.29
0.09
0.00
0.64

1.00
0.13
0.19
0.01
0.89
0.11
0.36
0.21
0.89
0.77
0.86
0.45
1.00
0.17
1.00
0.11
0.02
1.00
1.00
0.16
1.00
0.20
0.87
0.18
0.03
0.33
1.00
0.00
0.83
0.00
0.04
0.91
0.24
0.54
0.00
0.00
0.00
0.00
0.01
0.89
0.00
0.04
0.80
1.00
0.36
1.00
0.00
0.00

0.00
1.00
0.41
0.00
0.75
0.00
1.00
0.70
1.00
1.00
0.00
1.00
1.00
0.03
0.28
1.00
1.00
0.00
0.76
0.19
0.00
0.01
0.85
1.00
0.15
1.00
1.00
0.15
0.25
1.00
1.00
0.09
0.00
0.46
0.19
1.00
0.45
0.13
0.09
1.00
1.00
0.00
0.12
1.00
0.00
1.00
1.00
1.00

0.01
0.74
0.00
0.08
0.86
0.86
0.00
1.00
0.03
1.00
0.72
0.05
0.00
0.03
0.66
0.46
0.19
1.00
0.10
0.05
1.00
1.00
0.00
1.00
1.00
1.00
0.76
1.00
0.82
0.00
0.01
0.74
0.00
0.07
0.52
0.00
1.00
0.04
0.01
1.00
0.54
0.20
1.00
0.00
0.00
0.00
0.40
1.00

0.32
0.17
0.00
0.00
0.03
0.24
1.00
0.00
0.07
0.52
1.00
0.78
1.00
1.00
0.00
0.00
0.01
0.54
0.00
0.36
0.01
1.00
0.10
1.00
1.00
0.00
0.48
0.73
1.00
0.43
1.00
0.16
0.02
0.59
0.04
0.50
0.37
0.02
0.72
0.84
1.00
1.00
0.61
0.01
0.23
0.97
1.00
0.21

0.14
0.61
1.00
0.00
0.00
0.00
0.04
1.00
0.01
0.27
0.00
0.00
0.23
1.00
0.01
0.15
0.12
0.03
0.10
0.80
0.18
1.00
1.00
0.66
0.89
0.00
1.00
0.00
0.62
0.00
0.11
0.00
0.00
0.89
0.11
0.00
0.01
0.07
0.00
0.00
0.23
0.00
0.84
1.00
0.00
0.18
0.10
1.00

1.00
1.00
0.03
0.00
0.00
0.05
0.79
1.00
0.04
1.00
0.15
0.00
0.39
0.00
1.00
0.74
0.00
0.00
1.00
0.01
0.47
0.92
0.00
1.00
0.91
0.02
1.00
0.56
0.71
0.24
0.00
0.40
0.21
1.00
0.00
1.00
1.00
1.00
1.00
0.00
0.03
1.00
0.73
0.65
0.74
0.28
0.01
1.00

1.00
0.00
0.01
0.13
0.03
1.00
0.55
1.00
0.00
0.00
1.00
0.00
1.00
0.30
0.43
0.00
1.00
1.00
0.56
0.80
0.07
0.00
0.00
0.07
0.83
0.10
1.00
1.00
0.20
0.52
0.07
0.93
0.00
0.09
0.17
1.00
0.00
0.00
1.00
1.00
0.01
0.00
0.00
0.00
0.01
0.00
0.13
0.04

1.00
0.00
0.09
0.00
1.00
0.02
0.51
0.02
0.19
1.00
0.00
0.16
0.07
0.90
0.89
0.85
0.01
0.12
1.00
0.00
1.00
0.03
0.00
0.17
0.79
1.00
0.00
0.40
0.04
1.00
0.02
1.00
0.03
0.00
0.56
0.00
0.00
1.00
0.01
1.00
1.00
0.58
0.65
0.51
0.30
0.15
0.86
0.36

0.00
0.02
0.74
0.00
0.40
1.00
0.70
0.01
0.58
0.02
0.25
1.00
0.01
0.33
0.01
0.32
0.94
0.00
0.13
0.01
0.25
0.67
0.23
1.00
0.00
0.00
0.00
0.16
0.36
0.02
0.00
0.03
1.00
1.00
0.00
0.00
0.00
0.00
1.00
0.57
0.01
0.86
0.82
0.00
0.00
0.01
0.07
0.41

0.37
0.68
1.00
0.89
1.00
0.01
0.43
0.00
1.00
0.30
1.00
0.00
1.00
1.00
0.00
0.06
1.00
0.00
0.25
1.00
1.00
0.06
0.41
0.37
0.11
0.00
1.00
0.00
0.00
0.00
0.03
0.91
0.01
0.00
1.00
0.23
0.66
0.63
0.28
1.00
1.00
0.41
0.26
1.00
0.84
0.00
0.00
0.02

0.00
0.57
0.02
0.00
1.00
0.02
0.00
1.00
0.04
0.16
0.00
0.83
0.00
0.17
0.13
0.84
0.15
1.00
0.75
1.00
1.00
0.33
1.00
0.52
0.33
0.66
0.22
1.00
0.03
0.04
0.00
1.00
0.50
1.00
1.00
0.00
0.59
0.95
0.00
0.64
0.67
0.03
0.81
0.00
0.05
0.05
1.00
0.00

0.74
0.03
0.55
0.57
1.00
1.00
0.00
0.01
0.33
0.36
0.00
0.00
0.21
0.03
0.65
0.06
0.63
0.03
0.60
0.00
0.96
0.02
0.13
0.07
0.00
0.00
0.49
0.31
1.00
0.11
0.08
0.40
0.37
0.00
0.79
0.94
0.30
0.00
0.23
1.00
1.00
0.47
1.00
1.00
0.91
0.04
1.00
0.33

0.00
0.04
0.01
0.00
0.96
1.00
0.00
0.84
0.92
1.00
0.73
0.75
0.16
1.00
0.00
0.00
0.00
1.00
0.84
0.21
0.01
1.00
0.70
0.16
0.53
0.03
0.00
0.03
1.00
0.62
0.14
0.21
0.03
1.00
0.70
0.12
0.04
0.00
0.17
0.11
0.00
0.00
0.35
0.00
1.00
1.00
0.49
0.05

0.16
0.03
0.00
0.00
1.00
0.01
1.00
0.00
0.00
1.00
0.00
0.88
1.00
0.36
0.42
0.59
0.95
0.31
0.00
0.00
0.00
0.93
0.42
0.00
0.00
0.95
1.00
1.00
0.08
0.11
1.00
1.00
0.78
0.00
0.00
0.11
0.75
1.00
0.11
0.00
0.29
0.30
0.00
0.90
0.00
0.00
0.70
0.04

0.00
0.00
0.03
0.01
1.00
0.24
1.00
1.00
1.00
0.39
0.82
0.27
0.70
0.28
0.82
0.00
0.86
0.18
0.65
0.32
0.00
0.00
1.00
1.00
1.00
0.74
0.56
1.00
0.00
0.75
0.35
1.00
1.00
1.00
0.26
1.00
0.00
0.26
0.52
1.00
0.11
0.02
0.82
1.00
1.00
1.00
0.85
0.51

0.00
1.00
0.00
0.34
0.72
0.00
1.00
0.82
0.04
1.00
1.00
0.63
0.01
0.81
1.00
1.00
0.32
1.00
0.00
0.01
1.00
0.83
0.02
0.50
0.01
0.28
0.45
1.00
0.92
1.00
0.00
1.00
1.00
1.00
0.68
0.40
0.62
0.18
0.00
0.73
1.00
0.66
1.00
0.00
0.89
0.04
0.30
0.08

0.86
0.03
0.93
0.00
0.09
0.00
1.00
0.00
0.04
0.99
0.00
0.00
0.01
0.12
1.00
0.94
0.00
0.00
0.00
0.45
1.00
0.75
1.00
0.93
0.35
0.67
0.10
1.00
1.00
1.00
1.00
0.13
0.01
0.00
1.00
0.18
0.32
0.42
1.00
0.66
0.44
0.00
0.18
0.37
1.00
0.28
0.17
1.00

0.63
0.78
0.00
0.62
0.00
0.02
0.82
0.02
0.32
0.03
1.00
1.00
0.65
0.01
0.00
0.26
1.00
0.98
1.00
0.09
0.05
0.07
0.15
0.00
0.01
0.24
0.77
0.00
0.72
1.00
0.97
1.00
0.00
0.77
1.00
0.00
1.00
0.00
0.00
0.02
0.74
0.29
0.00
1.00
0.25
0.00
1.00
0.54

0.16
1.00
1.00
1.00
0.00
0.85
0.45
1.00
0.89
0.04
0.01
0.14
0.00
1.00
0.49
0.77
1.00
0.16
0.08
0.12
0.21
0.00
0.00
0.03
1.00
0.32
1.00
0.02
0.00
1.00
1.00
0.00
0.00
0.65
0.95
1.00
0.38
0.10
1.00
0.18
0.87
1.00
1.00
0.00
1.00
0.00
0.11
1.00

0.50
1.00
0.00
0.00
1.00
0.01
0.00
0.00
0.00
0.63
1.00
0.85
0.60
0.11
1.00
0.27
0.00
0.00
0.82
0.03
0.14
1.00
1.00
0.01
0.00
1.00
0.59
0.00
0.71
0.09
1.00
1.00
1.00
0.11
1.00
0.14
0.75
0.40
0.66
1.00
0.78
0.42
0.00
0.23
1.00
0.69
0.97
0.00

0.10
0.36
0.01
1.00
0.09
1.00
0.35
0.00
0.02
1.00
1.00
0.47
0.00
0.08
1.00
0.29
1.00
0.43
0.00
0.14
0.39
0.04
1.00
0.24
0.72
0.26
0.00
1.00
0.00
0.00
1.00
0.21
0.00
0.29
1.00
0.02
0.07
0.85
0.33
1.00
1.00
0.00
0.24
0.52
0.00
0.00
0.05
0.11

0.00
0.73
1.00
0.00
0.90
0.89
0.11
0.00
0.00
0.01
0.00
0.46
0.21
0.14
1.00
0.33
0.00
1.00
0.00
1.00
0.00
0.48
0.01
0.00
0.01
1.00
0.16
0.05
0.00
0.00
1.00
1.00
0.00
0.01
0.25
0.13
0.00
0.00
1.00
0.45
0.12
0.00
1.00
1.00
0.43
0.02
0.00
0.00

0.52
0.06
0.45
0.88
0.38
1.00
0.29
0.26
0.01
0.00
1.00
0.56
0.26
0.75
0.00
0.58
0.73
0.00
0.48
1.00
0.00
1.00
0.30
0.25
0.00
0.00
1.00
1.00
1.00
0.12
1.00
0.65
1.00
0.01
0.01
0.77
0.01
1.00
0.24
0.00
0.00
0.00
0.98
1.00
1.00
0.03
0.65
1.00

0.86
0.29
0.50
1.00
0.10
0.00
0.00
0.01
0.00
1.00
1.00
0.00
1.00
0.41
0.24
0.08
0.01
0.29
0.51
0.09
1.00
0.21
0.90
1.00
1.00
0.20
1.00
0.09
0.66
0.08
0.02
0.52
1.00
0.00
1.00
0.29
0.72
0.00
0.42
1.00
0.27
0.75
0.24
1.00
0.00
0.00
0.82
0.88

0.00
0.20
0.00
1.00
1.00
0.05
0.00
0.00
0.47
0.45
0.00
1.00
0.00
0.91
1.00
0.03
1.00
0.13
0.05
1.00
0.04
0.30
0.74
0.12
0.06
0.00
0.92
0.01
1.00
1.00
0.11
1.00
0.74
0.51
0.64
1.00
0.68
0.76
0.00
0.18
0.00
0.00
1.00
1.00
0.14
1.00
1.00
1.00

0.54
1.00
1.00
1.00
0.00
0.54
1.00
1.00
0.00
0.00
0.60
0.00
0.17
0.52
1.00
1.00
0.00
0.42
0.16
1.00
0.00
0.39
0.94
0.37
1.00
0.46
1.00
0.18
0.01
0.68
0.01
0.00
0.01
1.00
1.00
0.73
1.00
0.03
0.58
0.00
0.07
0.00
0.03
0.00
1.00
0.10
0.25
1.00

0.11
0.50
0.98
0.34
1.00
1.00
0.00
0.09
1.00
0.21
0.26
0.70
0.00
0.00
1.00
0.01
0.20
0.00
0.00
1.00
0.31
0.89
0.05
1.00
0.19
0.02
0.00
0.18
0.26
0.82
0.01
0.00
0.00
0.75
0.66
1.00
0.00
0.70
0.07
1.00
0.00
0.00
0.42
0.00
0.16
0.00
0.28
0.87

1.00
0.01
0.00
0.07
0.46
1.00
0.05
0.01
0.00
0.00
0.98
0.00
0.28
0.12
0.00
1.00
1.00
0.78
0.19
0.19
0.00
1.00
0.48
0.01
0.00
0.38
0.09
1.00
0.08
0.01
0.74
0.04
0.05
1.00
0.01
0.12
0.03
0.01
0.21
1.00
1.00
0.17
0.00
0.00
1.00
0.45
1.00
0.04

1.00
1.00
0.01
0.22
0.28
1.00
0.00
0.20
0.00
0.71
0.08
0.00
0.00
0.06
0.25
1.00
0.65
0.17
0.09
0.81
0.30
0.81
0.00
0.10
1.00
0.00
0.00
0.54
0.57
0.32
0.00
0.17
0.20
1.00
1.00
0.04
0.01
0.00
0.23
1.00
0.01
0.00
0.07
1.00
0.19
0.00
1.00
0.00

0.42
1.00
1.00
0.66
0.07
1.00
0.79
0.73
1.00
0.78
0.82
0.00
1.00
0.64
0.59
1.00
0.00
0.00
0.73
0.00
1.00
0.00
0.03
0.01
1.00
1.00
1.00
1.00
0.25
0.00
0.00
0.00
0.80
0.12
0.15
1.00
1.00
0.14
0.13
0.98
0.04
0.02
0.23
0.21
0.02
0.00
0.00
0.00

0.62
0.06
0.70
0.80
0.22
0.11
0.27
0.95
0.05
1.00
0.60
1.00
1.00
1.00
0.00
1.00
0.48
0.00
0.00
0.48
0.00
0.92
0.00
0.49
0.22
0.39
0.00
1.00
0.00
0.00
1.00
0.00
0.37
0.12
0.00
0.08
0.23
0.00
0.03
0.00
0.11
0.00
1.00
0.00
0.00
0.04
0.34
0.03

0.00
0.00
1.00
1.00
0.00
1.00
0.51
0.00
0.54
0.74
0.57
0.55
0.33
1.00
0.01
0.00
0.01
0.01
0.00
0.01
0.24
1.00
0.03
0.00
1.00
1.00
0.45
1.00
0.12
1.00
0.00
0.17
0.51
1.00
1.00
0.28
0.00
1.00
1.00
0.89
0.00
0.14
0.79
0.00
0.98
0.15
0.30
0.38

0.78
0.00
0.00
1.00
0.00
0.90
0.51
0.72
0.36
1.00
0.22
0.48
0.61
0.95
0.66
0.65
1.00
0.00
0.04
0.63
0.87
0.74
0.00
0.36
0.68
0.25
1.00
1.00
0.04
0.01
1.00
0.03
0.06
0.17
0.97
0.52
0.00
1.00
1.00
0.10
0.04
0.00
0.00
0.70
0.45
0.27
0.45
1.00

0.01
0.01
0.02
0.31
0.74
0.00
0.07
0.07
1.00
1.00
1.00
0.00
0.84
0.03
0.00
1.00
1.00
1.00
0.43
0.82
0.98
0.04
0.15
1.00
0.87
0.00
1.00
1.00
0.00
0.63
0.32
1.00
0.01
1.00
0.64
0.00
0.00
0.62
0.30
0.03
0.00
0.10
0.27
0.83
1.00
0.06
0.00
0.00

0.14
0.00
0.23
1.00
0.01
0.40
0.00
1.00
0.09
1.00
0.61
1.00
0.63
0.03
0.00
0.42
1.00
0.56
0.02
0.00
1.00
1.00
1.00
1.00
0.57
0.04
0.09
0.00
0.83
1.00
1.00
1.00
0.00
0.00
0.88
0.35
0.02
0.00
0.00
0.18
0.28
1.00
0.93
0.07
1.00
0.55
0.11
1.00

1.00
0.01
1.00
0.58
0.42
0.46
0.00
0.01
0.14
0.04
0.04
0.28
0.00
0.35
0.16
0.00
0.54
0.01
0.14
0.83
0.00
0.00
0.00
1.00
0.06
0.57
0.86
0.31
0.00
0.02
0.00
1.00
1.00
0.39
0.30
0.03
0.03
1.00
0.73
0.00
0.68
0.05
0.21
1.00
1.00
0.68
0.81
0.32

0.63
0.00
0.85
0.00
0.00
0.04
0.19
0.15
0.01
0.59
0.02
0.00
1.00
1.00
0.00
0.05
0.41
0.54
1.00
0.14
0.50
0.24
0.12
0.00
0.00
1.00
1.00
0.14
1.00
0.06
0.00
1.00
0.00
0.84
0.01
1.00
0.01
1.00
0.21
1.00
1.00
1.00
0.80
0.20
1.00
1.00
0.02
1.00

1.00
0.00
1.00
1.00
1.00
1.00
1.00
0.00
1.00
1.00
0.72
0.55
0.64
0.64
0.91
0.44
0.11
0.01
0.01
1.00
0.00
0.00
0.00
0.33
0.00
0.45
0.30
1.00
0.04
0.00
0.66
0.00
0.00
0.03
0.00
0.27
0.17
0.00
0.39
0.05
0.64
0.81
0.44
1.00
1.00
0.20
0.10
1.00

0.00
0.01
0.00
1.00
0.06
0.95
0.15
0.91
0.04
1.00
0.00
0.00
1.00
0.42
0.43
1.00
0.78
0.00
0.98
1.00
0.21
0.00
0.67
1.00
1.00
1.00
0.49
0.00
0.28
1.00
0.00
1.00
0.00
1.00
0.78
0.00
0.00
0.12
0.87
0.00
0.00
0.93
0.03
0.16
0.00
0.37
1.00
1.00

0.50
1.00
0.23
1.00
0.10
0.06
0.02
0.01
0.77
1.00
0.01
1.00
0.00
0.00
0.00
0.00
0.06
1.00
0.00
1.00
0.36
0.73
0.00
0.52
0.05
0.00
0.00
0.00
0.95
1.00
1.00
0.26
0.00
1.00
1.00
0.63
0.00
0.42
0.11
0.00
0.76
0.01
0.06
0.51
0.47
0.63
1.00
1.00

1.00
1.00
0.12
0.18
1.00
0.09
0.49
0.00
0.08
0.00
0.00
0.42
0.87
0.02
1.00
0.05
0.00
0.95
0.44
0.00
0.11
0.00
0.78
0.37
0.00
0.06
0.02
0.00
0.45
0.00
1.00
0.00
0.31
0.03
0.00
0.00
0.02
1.00
1.00
0.99
1.00
0.01
1.00
1.00
1.00
1.00
0.84
1.00

1.00
0.56
0.65
1.00
0.62
1.00
1.00
0.56
0.20
0.40
1.00
1.00
0.61
0.00
0.01
1.00
0.00
0.21
0.00
0.00
0.85
0.72
1.00
0.00
0.06
0.00
1.00
1.00
0.97
0.02
0.10
0.37
0.39
0.00
0.00
0.10
0.00
1.00
1.00
1.00
0.06
0.00
0.71
0.00
0.00
0.00
0.82
1.00

1.00
0.00
0.00
1.00
1.00
1.00
0.00
0.07
1.00
0.00
0.05
0.00
0.00
0.39
0.00
1.00
0.00
0.00
0.95
0.00
0.00
0.45
0.00
0.00
0.00
0.37
0.00
1.00
0.88
0.01
0.05
0.00
0.36
0.00
0.00
0.00
0.12
0.11
0.95
1.00
0.07
1.00
0.11
0.01
0.00
1.00
1.00
0.40

0.02
0.42
0.00
0.00
1.00
0.98
0.88
0.82
0.44
1.00
0.99
0.32
0.28
0.71
0.00
0.38
0.44
1.00
0.21
0.02
1.00
1.00
0.90
0.64
0.36
0.86
0.84
1.00
0.07
0.01
0.26
1.00
0.74
0.07
1.00
1.00
1.00
1.00
0.11
1.00
0.00
0.07
0.00
0.00
1.00
1.00
0.00
0.07

1.00
0.68
0.86
0.02
1.00
1.00
0.00
0.00
0.01
1.00
0.71
0.02
1.00
0.84
1.00
0.24
1.00
0.01
0.00
0.04
0.63
0.02
1.00
0.78
0.01
0.00
0.00
1.00
0.21
0.48
0.41
1.00
0.97
0.13
1.00
1.00
0.28
0.24
0.07
1.00
1.00
0.00
1.00
0.00
0.03
0.13
0.07
0.42

1.00
0.14
0.56
1.00
1.00
0.00
0.91
0.00
1.00
0.00
0.00
0.07
0.24
1.00
0.01
0.06
0.00
0.00
0.38
0.07
1.00
1.00
0.26
0.33
0.99
0.17
1.00
0.57
0.00
0.33
0.46
0.00
1.00
0.61
1.00
0.06
0.00
1.00
0.21
0.00
0.97
0.49
0.00
0.12
0.53
0.41
0.05
0.10

0.42
0.04
1.00
0.03
0.12
0.39
0.33
1.00
0.28
0.24
1.00
0.77
1.00
0.18
0.56
0.00
0.40
0.54
1.00
0.29
1.00
0.87
0.00
0.00
0.02
0.00
0.17
0.56
0.01
1.00
0.00
1.00
0.00
0.10
0.56
0.00
0.06
0.17
1.00
1.00
1.00
1.00
0.87
0.17
0.26
0.09
0.02
0.00

0.39
1.00
0.01
0.01
1.00
0.39
0.00
0.06
0.48
1.00
1.00
0.78
0.74
0.03
0.27
0.00
1.00
0.12
1.00
1.00
0.00
0.13
1.00
0.27
0.20
0.00
0.18
0.00
0.50
0.13
0.00
1.00
0.00
1.00
0.53
1.00
0.00
0.01
0.77
0.42
0.00
1.00
0.04
1.00
0.00
0.00
1.00
0.03

0.00
0.83
0.00
0.00
0.00
0.00
0.99
0.70
0.65
0.83
0.04
1.00
0.02
0.75
0.93
0.98
0.57
1.00
0.69
0.00
1.00
0.17
0.00
1.00
0.74
0.00
0.00
0.06
0.00
1.00
0.00
0.00
1.00
1.00
0.00
0.00
1.00
0.93
0.00
0.00
0.00
1.00
0.03
0.00
0.00
1.00
1.00
0.00

0.72
0.74
0.41
0.94
0.00
0.00
0.68
0.11
0.11
0.00
1.00
0.24
1.00
0.42
0.00
1.00
1.00
1.00
0.45
0.01
0.00
0.52
0.15
0.29
0.02
1.00
1.00
1.00
1.00
0.85
0.00
1.00
0.12
0.00
0.00
1.00
0.05
0.60
0.57
0.00
0.95
0.26
0.01
0.03
0.09
0.41
0.00
0.00

0.00
0.06
0.48
0.03
0.42
1.00
1.00
0.79
0.36
0.61
0.01
0.20
1.00
0.20
0.77
1.00
0.15
0.70
0.49
1.00
0.04
1.00
1.00
0.12
0.71
0.03
0.89
0.88
1.00
0.02
0.08
0.99
0.59
0.42
0.57
0.47
0.39
0.47
0.00
1.00
0.07
0.23
0.66
0.00
0.02
0.00
0.02
1.00

1.00
1.00
0.21
0.89
0.69
1.00
0.00
0.00
0.32
1.00
0.08
1.00
0.00
1.00
1.00
0.35
1.00
0.00
0.00
0.02
0.14
1.00
0.18
1.00
0.47
0.03
0.44
0.00
1.00
1.00
0.56
0.00
1.00
0.44
1.00
1.00
0.00
1.00
1.00
1.00
0.11
0.00
0.00
0.00
0.00
1.00
0.00
0.76

0.00
0.00
0.38
0.54
0.90
0.89
0.54
0.00
0.25
0.01
0.79
0.00
0.68
0.00
0.04
1.00
0.00
0.40
0.00
0.00
0.03
0.52
0.00
0.00
0.37
0.00
1.00
0.30
0.18
1.00
0.01
0.00
0.00
0.83
0.00
0.00
0.19
0.53
0.77
0.06
0.06
0.03
1.00
1.00
0.00
0.35
0.08
0.60

1.00
1.00
1.00
0.02
0.52
0.98
1.00
1.00
0.33
0.47
0.41
0.56
0.76
0.79
0.00
0.45
1.00
0.40
0.63
0.00
0.54
0.00
0.00
0.00
0.01
0.04
0.00
0.00
0.65
1.00
0.51
0.31
0.04
0.00
0.00
0.00
1.00
0.00
0.02
0.16
0.09
0.01
0.81
1.00
1.00
0.06
1.00
1.00

0.37
1.00
0.05
0.52
0.20
1.00
0.00
0.00
1.00
0.44
0.00
0.41
0.59
0.00
0.96
1.00
0.15
0.23
0.10
0.00
0.64
1.00
0.74
0.00
1.00
0.00
0.06
0.67
0.01
0.07
1.00
1.00
0.00
0.97
0.00
0.06
0.00
1.00
0.04
0.90
0.62
1.00
1.00
0.87
0.00
0.01
0.02
1.00

0.45
0.19
1.00
0.01
0.00
1.00
0.01
0.10
0.00
1.00
0.30
0.69
0.44
1.00
1.00
1.00
1.00
0.29
1.00
1.00
1.00
1.00
0.90
0.11
0.78
1.00
0.16
0.24
0.41
1.00
1.00
0.02
0.00
1.00
0.91
1.00
0.24
0.36
0.95
0.01
1.00
0.57
1.00
1.00
0.00
1.00
0.00
0.85

0.62
1.00
1.00
1.00
1.00
1.00
1.00
0.00
0.08
0.10
0.00
0.95
0.00
0.00
0.00
0.03
0.00
1.00
1.00
0.00
0.68
0.42
0.03
0.14
0.10
0.85
0.00
1.00
0.66
0.37
1.00
1.00
0.01
0.01
0.73
1.00
0.00
0.23
0.04
0.00
0.19
1.00
1.00
1.00
0.00
0.11
0.10
0.20

0.01
0.98
0.78
0.00
1.00
0.00
1.00
0.00
0.00
0.10
0.13
0.05
0.00
0.31
0.11
0.09
0.52
0.89
0.10
0.00
0.20
0.07
0.18
1.00
0.22
0.01
0.00
0.70
1.00
0.03
0.00
0.00
0.59
0.00
0.00
0.00
0.29
1.00
0.00
1.00
1.00
0.00
0.00
0.01
0.25
0.36
0.00
0.00

1.00
1.00
0.23
0.00
0.04
1.00
0.00
0.84
0.00
0.61
0.07
1.00
1.00
0.00
1.00
1.00
0.03
0.00
0.00
1.00
0.23
0.73
0.60
0.00
1.00
1.00
0.32
1.00
0.84
0.97
1.00
0.00
1.00
0.68
0.14
1.00
1.00
0.23
0.78
1.00
0.38
1.00
0.09
0.89
0.11
0.70
1.00
0.16

1.00
0.01
0.68
0.22
0.62
1.00
1.00
0.00
0.78
0.33
0.18
1.00
0.82
1.00
0.00
0.00
1.00
0.30
0.00
0.64
1.00
0.98
1.00
0.84
1.00
0.26
0.35
0.13
0.50
1.00
0.90
0.04
0.25
0.01
0.05
0.00
0.00
0.28
0.00
0.00
0.99
0.00
0.00
0.03
1.00
0.00
0.00
0.00

1.00
0.21
0.19
1.00
1.00
1.00
0.54
0.00
1.00
0.02
0.00
0.57
0.97
0.23
0.32
0.81
0.00
0.00
0.00
0.00
0.00
0.00
0.00
0.03
0.01
0.00
0.00
0.00
1.00
0.00
0.00
0.29
0.01
0.09
1.00
1.00
0.84
1.00
1.00
1.00
0.97
0.19
0.00
0.00
0.10
1.00
1.00
0.64

1.00
0.00
1.00
0.01
0.80
0.29
0.00
0.55
0.00
0.00
0.00
0.91
0.01
1.00
0.56
0.06
0.00
0.01
1.00
0.00
0.02
0.72
0.87
0.10
0.25
0.02
0.11
0.16
0.08
1.00
1.00
0.51
0.48
0.81
0.25
1.00
0.98
0.41
0.00
0.00
0.00
0.65
1.00
0.00
0.00
1.00
1.00
0.87

1.00
1.00
1.00
0.04
0.93
1.00
1.00
0.60
0.73
0.01
1.00
0.00
1.00
0.00
0.00
0.00
0.00
0.92
0.00
0.00
1.00
0.02
1.00
0.00
1.00
0.05
0.91
0.01
0.45
0.01
0.64
0.79
0.08
0.83
0.14
0.24
0.12
0.42
0.48
0.00
0.00
1.00
0.20
0.39
0.42
0.00
0.00
0.54

1.00
0.00
1.00
1.00
1.00
0.12
1.00
1.00
0.00
0.00
0.83
0.01
0.43
0.72
0.03
1.00
0.79
1.00
0.46
1.00
0.00
0.78
0.00
0.58
0.47
0.52
0.10
0.48
0.00
0.00
1.00
0.82
0.33
0.23
0.06
0.01
0.06
1.00
1.00
1.00
1.00
0.34
0.78
0.00
1.00
0.96
1.00
0.60

1.00
0.02
1.00
1.00
1.00
0.37
0.00
0.78
0.41
0.05
0.00
0.00
1.00
0.83
1.00
1.00
1.00
0.40
0.06
1.00
1.00
0.25
1.00
1.00
0.42
0.03
0.92
0.78
0.46
0.74
0.25
1.00
0.00
1.00
1.00
1.00
1.00
0.84
0.27
0.30
1.00
0.05
0.00
1.00
0.06
0.89
0.00
0.05

1.00
0.30
0.95
1.00
0.53
1.00
0.68
1.00
0.62
0.68
0.40
0.00
0.00
0.70
1.00
1.00
0.03
0.00
0.59
0.00
0.31
0.01
0.22
0.25
1.00
0.13
1.00
0.31
1.00
1.00
1.00
1.00
0.05
1.00
0.59
0.10
0.25
1.00
0.13
1.00
0.15
0.02
1.00
1.00
1.00
0.06
1.00
0.24

0.02
0.33
1.00
0.18
1.00
0.05
0.74
1.00
0.01
0.00
0.00
0.56
0.00
0.07
1.00
0.38
0.62
0.85
0.68
1.00
0.61
0.00
0.13
1.00
0.05
0.01
1.00
0.61
0.43
0.01
1.00
1.00
1.00
1.00
0.70
0.01
0.00
0.25
1.00
1.00
0.00
1.00
1.00
1.00
1.00
0.35
1.00
1.00

0.15
0.08
1.00
1.00
1.00
0.14
0.00
1.00
0.00
0.28
0.10
0.00
0.73
0.02
1.00
1.00
1.00
1.00
1.00
1.00
1.00
0.93
1.00
0.72
0.18
0.00
0.29
0.30
1.00
0.97
0.00
0.00
0.50
1.00
1.00
1.00
1.00
0.05
0.07
1.00
0.23
1.00
1.00
0.59
0.12
1.00
0.26
0.63

0.00
0.01
0.84
0.28
1.00
1.00
1.00
0.39
1.00
1.00
0.91
0.87
1.00
0.88
0.09
0.00
0.00
0.62
1.00
0.00
1.00
1.00
0.11
0.04
1.00
0.00
0.11
0.07
1.00
0.15
1.00
1.00
0.95
0.00
0.21
0.24
0.00
1.00
0.02
1.00
0.59
0.00
0.10
0.69
0.00
0.85
0.89
0.76

0.08
0.09
0.93
0.06
0.00
0.01
0.63
0.00
0.16
1.00
0.01
0.00
0.00
1.00
0.94
0.84
1.00
0.18
1.00
0.09
0.33
1.00
0.71
0.00
0.44
1.00
0.02
0.44
0.00
0.03
0.54
0.04
1.00
0.08
0.01
0.84
0.01
1.00
0.00
0.46
0.78
0.15
1.00
1.00
1.00
1.00
0.00
1.00

1.00
1.00
0.36
0.70
1.00
0.58
0.04
0.07
0.89
1.00
0.02
0.01
1.00
1.00
0.75
0.70
1.00
0.00
0.35
0.67
0.76
0.00
0.00
0.01
0.14
1.00
0.74
0.29
1.00
1.00
1.00
0.77
1.00
0.15
0.26
0.16
0.12
0.69
0.29
0.93
0.86
0.09
0.96
0.94
0.10
0.99
0.01
0.89

1.00
1.00
0.41
0.99
0.60
1.00
1.00
0.24
0.84
1.00
0.15
0.73
0.00
0.00
0.38
0.00
0.38
1.00
1.00
0.99
0.40
0.46
1.00
0.24
1.00
0.19
0.17
1.00
0.91
0.00
0.51
0.17
1.00
0.00
0.87
0.16
1.00
0.27
0.44
0.86
0.11
1.00
1.00
1.00
1.00
0.95
0.63
0.10

1.00
0.35
1.00
1.00
0.47
0.71
1.00
0.02
1.00
1.00
1.00
0.05
0.70
0.01
0.75
0.81
1.00
1.00
0.53
1.00
0.63
0.70
0.16
0.00
0.00
0.85
0.10
1.00
0.13
0.00
1.00
1.00
1.00
1.00
0.24
0.35
1.00
0.00
1.00
0.27
0.55
1.00
0.49
0.31
0.36
0.27
0.88
0.42

0.00
1.00
0.77
1.00
0.24
1.00
0.23
0.54
0.18
0.03
1.00
1.00
0.89
0.99
0.01
0.92
0.01
0.24
0.04
1.00
1.00
1.00
1.00
1.00
0.56
1.00
0.61
1.00
0.40
1.00
0.27
0.55
0.19
1.00
0.36
1.00
0.89
1.00
1.00
1.00
0.96
1.00
0.75
0.83
1.00
0.17
0.71
0.05

0.89
1.00
1.00
0.76
0.74
0.97
0.27
0.92
0.07
0.64
0.36
0.61
0.11
0.48
1.00
1.00
0.25
0.53
1.00
0.90
0.00
0.64
1.00
1.00
0.50
1.00
0.95
0.00
0.18
1.00
1.00
1.00
0.29
1.00
0.10
0.32
1.00
1.00
0.11
0.39
0.04
1.00
1.00
0.68
0.13
1.00
0.44
1.00

0.11
0.00
1.00
1.00
0.97
1.00
0.04
1.00
0.00
0.76
0.18
0.00
0.00
0.68
1.00
1.00
0.19
1.00
1.00
0.78
0.83
0.00
1.00
1.00
1.00
0.89
0.05
0.89
0.03
1.00
0.03
1.00
0.00
0.35
0.06
0.01
0.84
0.00
0.97
0.61
0.07
1.00
1.00
0.10
1.00
0.21
0.39
0.51

0.00
0.00
0.50
0.00
0.72
0.15
0.34
1.00
1.00
0.75
0.05
1.00
1.00
0.84
0.09
1.00
0.69
0.33
0.56
0.86
1.00
0.10
0.38
1.00
1.00
0.00
0.00
0.70
0.70
0.02
0.89
0.14
0.36
1.00
1.00
1.00
0.43
0.39
1.00
1.00
0.63
0.61
0.69
1.00
1.00
0.86
1.00
0.63

0.44
0.11
0.49
0.97
1.00
0.73
0.00
1.00
0.56
1.00
0.00
0.94
0.06
0.55
0.27
0.52
0.37
0.16
1.00
0.13
1.00
0.01
0.33
1.00
0.60
0.33
0.80
1.00
0.09
0.92
0.61
0.50
0.02
0.01
0.32
1.00
0.73
0.00
1.00
0.00
0.52
1.00
1.00
0.09
1.00
0.01
1.00
1.00

0.11
1.00
1.00
0.00
0.77
0.58
0.76
0.44
1.00
0.11
1.00
0.20
0.97
0.00
0.00
0.10
0.17
0.00
0.00
0.00
0.01
0.91
1.00
0.57
1.00
0.13
0.00
0.12
0.00
0.08
0.00
1.00
1.00
0.29
0.73
1.00
0.01
0.00
0.28
0.95
0.67
0.15
0.00
1.00
1.00
0.11
0.00
0.89

0.07
0.00
0.00
0.55
1.00
0.00
0.00
0.00
0.59
0.05
0.90
0.98
0.00
1.00
0.62
0.35
0.30
1.00
0.49
0.77
0.06
1.00
0.03
0.25
1.00
0.07
0.01
0.35
0.75
1.00
1.00
1.00
1.00
0.46
0.52
0.87
1.00
1.00
1.00
0.24
1.00
0.02
0.00
0.05
1.00
0.29
0.38
1.00

0.98
0.04
1.00
0.11
0.08
0.25
0.18
1.00
0.11
0.00
0.68
0.99
1.00
0.00
0.14
0.33
1.00
0.00
0.00
1.00
1.00
0.00
1.00
0.80
0.79
0.00
0.97
1.00
1.00
0.01
0.30
0.00
0.31
0.18
0.33
0.02
0.60
0.00
0.03
1.00
1.00
0.00
0.03
0.00
0.00
0.65
0.63
0.07

1.00
0.01
0.55
0.00
0.00
0.00
0.00
0.00
0.00
0.36
0.45
1.00
0.10
1.00
1.00
0.36
0.00
0.23
0.00
0.00
0.00
0.46
0.37
1.00
1.00
0.00
0.02
0.26
0.01
1.00
1.00
1.00
0.00
0.00
0.00
1.00
1.00
0.00
0.00
0.01
0.63
0.82
0.84
0.20
0.61
0.11
0.22
0.29

0.00
1.00
0.02
0.69
0.01
0.00
1.00
0.25
0.70
0.72
0.18
0.69
1.00
1.00
0.11
0.00
1.00
0.38
0.00
0.09
0.45
0.00
0.00
0.01
0.07
0.08
0.05
0.72
1.00
1.00
0.41
0.87
0.52
0.00
0.46
0.15
0.17
0.47
1.00
1.00
1.00
0.01
1.00
0.06
0.45
0.07
0.62
0.00

0.36
0.00
0.02
0.00
0.25
0.02
0.01
0.00
1.00
0.81
0.00
1.00
0.21
0.02
0.04
0.00
0.50
1.00
0.33
0.00
0.42
0.64
0.00
0.00
0.19
0.06
0.00
0.14
1.00
0.16
1.00
1.00
1.00
1.00
1.00
0.22
0.00
0.08
0.03
0.28
0.19
0.00
1.00
0.15
0.97
0.02
1.00
1.00

0.00
0.40
0.27
0.07
1.00
0.63
0.00
0.00
0.00
0.00
0.11
1.00
0.00
1.00
0.00
0.00
1.00
0.51
1.00
0.55
1.00
0.40
0.27
0.45
0.00
0.59
0.00
1.00
0.40
0.09
0.81
0.01
0.00
0.90
0.65
0.80
0.07
1.00
1.00
1.00
1.00
0.00
0.07
0.00
1.00
0.00
0.38
0.42

0.03
0.00
1.00
1.00
1.00
0.39
0.00
0.00
0.92
0.09
1.00
0.37
0.14
1.00
0.00
0.56
0.91
0.01
0.02
0.65
0.43
0.04
0.00
1.00
0.00
0.23
0.15
0.42
0.27
0.00
1.00
1.00
0.00
0.04
0.97
0.01
0.00
0.00
0.30
0.04
0.08
0.01
0.89
1.00
0.95
0.30
0.05
0.01

0.38
0.24
0.73
0.98
1.00
0.00
0.00
1.00
1.00
0.00
0.00
1.00
0.00
0.17
1.00
0.37
0.00
0.01
1.00
1.00
0.21
0.62
0.09
1.00
1.00
1.00
0.00
0.00
0.01
0.00
0.07
0.12
1.00
0.22
0.00
0.00
0.01
0.65
0.02
1.00
0.03
0.07
0.11
1.00
1.00
0.15
0.00
0.77

0.00
1.00
0.15
0.64
0.07
0.00
0.14
0.00
0.18
0.00
0.10
0.46
0.44
1.00
0.02
0.51
0.00
0.00
0.00
0.01
0.00
0.50
0.00
1.00
0.06
0.00
0.31
0.00
0.61
0.52
0.25
1.00
0.66
1.00
1.00
0.84
1.00
1.00
0.08
1.00
0.12
0.05
0.16
0.34
1.00
0.04
1.00
1.00

0.00
1.00
0.02
0.04
1.00
0.00
0.50
1.00
0.15
0.08
1.00
0.54
1.00
0.00
0.03
0.00
0.00
1.00
0.62
0.46
0.13
1.00
0.26
0.86
0.00
0.00
0.01
0.21
0.00
0.00
1.00
0.00
0.05
1.00
0.00
0.00
0.00
0.00
1.00
1.00
1.00
0.00
0.46
0.54
0.61
0.39
0.58
1.00

0.08
1.00
0.00
0.70
0.90
0.04
0.45
0.72
0.00
0.08
1.00
0.69
0.02
0.15
0.27
0.14
0.07
0.00
0.89
0.90
0.00
0.09
0.11
0.63
0.00
0.84
0.00
0.00
0.07
0.00
0.01
0.61
1.00
0.22
1.00
0.58
0.12
0.00
0.03
0.00
0.02
0.00
0.29
0.08
0.21
0.00
1.00
0.48

1.00
0.43
0.00
0.50
1.00
0.02
0.93
1.00
0.10
0.45
0.56
0.61
0.01
0.78
0.07
0.18
0.00
1.00
1.00
1.00
0.00
1.00
0.00
0.00
0.00
0.10
0.00
0.00
1.00
0.78
0.91
0.00
1.00
0.05
0.00
0.79
0.00
0.77
0.10
1.00
1.00
0.32
0.76
1.00
1.00
0.64
0.16
0.38

1.00
0.04
0.13
0.00
0.78
0.27
0.00
0.94
1.00
0.65
1.00
1.00
0.28
0.00
1.00
1.00
0.70
0.83
0.38
0.00
1.00
1.00
1.00
0.97
0.54
1.00
0.38
1.00
0.92
0.00
0.00
0.00
0.00
0.08
1.00
0.34
0.70
1.00
0.33
0.66
0.42
0.29
0.79
0.19
0.54
0.99
0.19
0.61

0.70
0.49
0.16
0.39
1.00
0.00
0.04
1.00
0.00
0.42
0.74
0.77
0.89
0.70
0.12
0.97
0.36
0.14
1.00
0.79
1.00
0.36
0.01
0.11
1.00
1.00
0.51
0.46
0.90
0.19
0.38
1.00
0.11
0.14
0.75
0.97
0.02
0.06
1.00
0.91
0.36
1.00
1.00
1.00
0.08
1.00
0.12
0.96

1.00
0.42
0.58
0.08
0.00
1.00
0.75
0.67
0.74
0.39
0.78
0.94
1.00
0.39
1.00
1.00
1.00
0.00
0.01
0.19
1.00
0.03
0.17
1.00
0.57
0.93
1.00
0.30
0.55
0.48
0.26
1.00
0.15
0.04
0.18
0.12
0.18
0.16
1.00
0.02
1.00
0.00
0.01
0.61
1.00
0.42
0.76
0.01

0.67
1.00
0.93
1.00
0.74
0.54
0.16
1.00
1.00
0.51
0.00
1.00
0.16
0.13
0.45
1.00
1.00
1.00
1.00
1.00
1.00
1.00
0.86
0.63
0.64
0.82
0.19
1.00
1.00
0.12
0.00
0.24
0.00
0.33
1.00
0.91
0.03
1.00
0.28
0.00
1.00
1.00
0.08
0.53
0.29
1.00
1.00
0.07

0.00
1.00
1.00
0.28
0.37
0.00
0.00
0.10
0.05
0.51
0.06
0.50
0.90
0.94
0.21
0.13
0.95
0.90
0.00
0.00
0.01
0.00
1.00
0.01
0.39
1.00
0.00
1.00
0.00
0.00
0.64
1.00
1.00
0.94
0.00
1.00
0.34
0.00
0.15
0.00
0.03
0.01
0.07
0.00
1.00
0.00
1.00
0.00

0.00
0.00
1.00
0.31
0.00
1.00
0.00
0.75
0.54
1.00
0.00
0.33
0.01
1.00
0.42
0.00
0.00
0.29
0.00
1.00
0.11
0.55
0.02
0.02
1.00
0.89
0.17
0.00
0.51
0.03
1.00
0.18
1.00
0.00
0.00
0.14
1.00
1.00
1.00
0.00
0.01
1.00
0.23
0.04
1.00
1.00
0.44
0.00

0.69
0.00
0.78
0.00
1.00
0.00
0.00
1.00
0.77
0.00
1.00
0.00
1.00
0.00
0.21
0.00
0.13
1.00
1.00
0.00
0.33
0.84
0.00
0.52
0.00
0.74
0.76
0.00
1.00
0.05
0.50
0.00
0.00
0.01
0.51
0.01
0.03
0.00
0.00
0.00
0.00
0.04
0.00
0.01
1.00
0.05
0.73
0.61

0.00
0.09
0.33
0.25
0.08
0.05
0.04
0.04
0.11
0.14
0.14
0.04
0.08
1.00
1.00
0.00
0.19
1.00
0.52
0.00
0.00
1.00
0.00
0.01
0.00
0.08
0.01
1.00
0.12
0.00
0.12
0.15
1.00
0.39
0.03
0.15
0.45
0.00
0.47
0.89
0.39
0.11
0.00
0.34
0.02
0.00
1.00
0.13

0.00
0.32
0.41
0.23
0.00
0.03
1.00
0.70
0.00
0.00
0.00
1.00
0.49
0.01
1.00
0.00
0.12
1.00
0.28
0.15
0.35
1.00
0.98
1.00
0.89
1.00
0.13
0.02
0.89
1.00
1.00
0.02
1.00
0.19
0.77
1.00
0.67
0.66
0.12
0.43
0.41
0.40
0.28
1.00
1.00
0.60
0.00
0.89

0.02
0.00
1.00
0.65
0.43
0.00
0.00
0.78
0.00
0.54
1.00
0.18
1.00
1.00
0.21
0.19
0.00
0.02
1.00
0.01
0.00
1.00
0.02
1.00
0.05
0.00
0.13
0.00
0.00
1.00
0.00
0.39
0.02
0.66
1.00
0.23
0.21
1.00
1.00
0.01
0.84
0.34
0.03
1.00
0.03
0.02
1.00
0.00

0.85
0.13
0.06
1.00
1.00
0.97
0.78
0.11
0.27
0.00
0.21
0.78
0.68
0.02
1.00
0.20
0.03
0.77
0.34
0.97
0.40
0.52
0.79
1.00
0.41
0.21
1.00
0.92
0.67
0.11
0.07
0.88
0.65
0.10
0.26
1.00
1.00
0.89
0.00
0.17
0.00
0.00
0.00
0.68
0.00
1.00
1.00
0.60

0.82
0.00
0.00
0.01
1.00
0.00
1.00
0.54
1.00
0.00
0.00
0.00
0.92
0.29
0.41
0.07
0.00
1.00
0.01
1.00
1.00
0.20
1.00
0.52
0.00
0.69
0.19
1.00
0.17
0.00
0.00
0.87
0.00
0.32
0.81
0.28
0.13
0.02
0.09
0.59
0.02
0.04
0.00
0.46
0.00
1.00
1.00
1.00

0.71
0.00
1.00
0.00
0.01
0.01
0.56
1.00
0.86
1.00
0.27
0.05
1.00
0.00
0.06
0.39
0.01
1.00
0.38
0.60
0.00
1.00
1.00
1.00
0.93
0.22
1.00
0.13
0.00
0.00
0.98
0.00
0.21
0.00
0.53
0.22
0.00
0.00
1.00
0.01
0.01
1.00
0.80
0.11
0.98
0.15
1.00
0.00

1.00
0.28
1.00
0.84
0.05
0.93
0.88
0.23
1.00
1.00
0.53
0.13
0.36
1.00
1.00
1.00
1.00
0.39
1.00
0.00
0.53
0.48
1.00
0.26
0.54
1.00
0.40
1.00
0.00
0.00
0.12
1.00
0.00
1.00
0.00
0.96
0.00
0.09
0.09
0.07
0.00
1.00
1.00
1.00
0.65
1.00
0.41
1.00

0.27
0.01
0.01
0.00
1.00
0.00
1.00
0.69
0.00
0.00
0.00
0.16
1.00
0.01
0.14
0.00
0.00
0.53
1.00
0.14
0.01
0.51
0.76
1.00
0.00
0.00
0.00
0.68
0.05
0.73
0.00
0.97
0.81
0.00
0.00
0.15
1.00
0.00
0.04
1.00
0.19
0.00
0.00
0.00
0.09
0.00
1.00
0.73

0.00
1.00
0.30
1.00
1.00
0.00
0.00
0.28
0.01
0.57
1.00
0.01
0.00
0.55
0.01
1.00
0.11
0.00
0.00
0.00
0.00
1.00
0.00
0.42
0.16
0.00
1.00
0.92
0.02
0.27
0.02
0.01
1.00
0.11
0.36
0.00
0.00
0.03
0.00
0.68
0.00
0.21
0.00
0.07
0.01
0.00
0.00
0.00

0.08
0.46
0.64
0.00
1.00
0.89
0.00
0.49
0.70
0.44
1.00
0.38
1.00
0.00
0.10
0.05
0.23
1.00
0.86
0.12
1.00
0.10
0.01
1.00
0.00
1.00
0.00
0.00
1.00
1.00
0.00
0.02
0.00
0.00
0.10
0.83
1.00
1.00
1.00
0.07
0.00
0.00
0.00
1.00
0.00
0.04
0.03
0.09

0.01
0.08
1.00
0.03
0.22
0.00
0.00
1.00
0.96
0.00
0.42
0.00
0.95
0.00
0.11
1.00
0.09
0.04
1.00
0.01
0.00
0.00
0.00
0.02
1.00
1.00
0.00
0.00
1.00
0.02
1.00
1.00
0.00
0.00
1.00
0.21
0.24
0.00
0.00
0.00
1.00
0.01
0.54
1.00
0.60
0.27
1.00
1.00

1.00
0.03
0.00
0.42
1.00
0.00
0.46
0.98
0.51
0.03
0.36
0.05
0.88
1.00
0.16
0.00
0.07
0.00
0.06
1.00
0.01
0.46
0.31
0.07
0.00
1.00
1.00
0.00
1.00
0.00
0.58
0.01
0.16
0.03
0.33
0.00
0.61
0.25
0.62
1.00
0.08
0.44
0.66
0.01
0.64
0.00
1.00
0.00

0.02
0.00
1.00
1.00
1.00
0.00
0.33
0.00
1.00
0.00
0.00
0.25
1.00
0.01
0.91
1.00
0.05
0.65
0.00
0.66
0.89
0.00
0.00
0.77
0.19
0.27
1.00
0.06
0.35
0.26
0.44
0.93
0.63
1.00
0.00
0.03
1.00
0.00
0.55
0.02
1.00
0.00
1.00
0.19
0.11
0.69
0.74
0.01

0.68
0.00
0.95
0.42
0.21
1.00
0.20
0.00
0.00
1.00
0.84
0.01
0.00
0.00
0.96
1.00
0.00
1.00
1.00
0.04
0.23
1.00
1.00
1.00
0.49
0.00
0.95
1.00
0.00
1.00
0.00
0.84
1.00
0.73
1.00
0.00
1.00
0.06
0.47
0.00
0.95
0.01
0.48
0.03
0.01
0.90
0.52
0.12

0.63
0.09
0.01
0.00
0.00
0.67
0.00
0.81
1.00
0.00
1.00
0.02
0.05
1.00
1.00
0.06
0.00
1.00
0.09
0.04
0.23
1.00
0.70
0.00
0.40
0.04
0.08
0.00
0.36
1.00
0.00
1.00
0.15
0.12
0.13
1.00
1.00
0.83
0.02
0.83
0.00
0.44
0.65
0.71
0.01
0.78
1.00
0.00

1.00
0.87
0.01
1.00
0.00
0.00
0.00
1.00
1.00
0.15
0.00
0.08
1.00
0.12
0.26
1.00
0.00
0.00
0.00
0.07
0.72
0.97
0.41
0.08
1.00
0.22
1.00
0.00
0.00
0.04
0.89
0.01
1.00
0.23
0.15
0.30
0.04
1.00
0.02
0.02
0.00
0.09
0.25
0.01
0.02
0.07
0.47
1.00

0.73
0.07
0.99
0.00
0.00
0.00
0.29
1.00
1.00
0.10
0.07
0.84
0.82
0.43
0.01
0.00
0.00
0.00
0.00
0.30
0.58
0.00
1.00
0.01
0.03
0.01
0.82
0.00
0.01
0.96
0.97
0.23
0.14
0.20
0.00
1.00
0.02
0.34
0.07
0.37
0.63
1.00
1.00
0.00
0.17
0.01
0.00
0.01

0.06
0.26
0.31
0.89
0.00
0.74
1.00
0.03
0.00
0.15
0.02
0.00
0.02
1.00
0.01
0.66
1.00
0.80
0.02
0.00
0.14
0.04
1.00
0.00
0.00
0.00
0.00
1.00
1.00
0.93
1.00
1.00
0.01
0.01
0.28
0.22
1.00
0.01
0.02
1.00
0.11
0.16
0.11
0.00
0.11
0.90
0.78
0.16

0.24
0.02
0.00
0.23
0.44
0.00
1.00
1.00
0.74
0.05
0.00
1.00
1.00
1.00
1.00
1.00
1.00
1.00
0.00
1.00
0.01
0.56
0.02
0.44
1.00
0.33
1.00
0.13
0.26
0.30
0.70
0.00
0.66
0.00
0.77
0.02
1.00
1.00
0.56
0.97
1.00
0.00
0.44
0.02
0.00
0.20
0.75
1.00

1.00
0.89
1.00
0.09
0.11
1.00
0.39
0.00
0.00
0.42
0.00
0.81
0.01
0.41
0.00
0.04
0.04
1.00
0.67
0.00
0.37
1.00
1.00
0.02
0.09
1.00
0.45
1.00
0.00
0.00
1.00
0.30
0.00
0.80
0.76
1.00
0.04
0.52
1.00
0.07
0.11
0.00
0.86
1.00
0.16
0.14
0.00
0.01

0.00
0.42
0.68
0.63
0.10
0.60
1.00
0.28
0.07
0.88
0.66
0.41
0.78
0.66
0.01
1.00
0.00
0.42
1.00
0.15
1.00
1.00
0.04
0.45
0.15
0.38
0.00
1.00
1.00
1.00
1.00
0.45
0.38
1.00
0.01
0.02
0.24
0.04
1.00
0.98
0.18
0.03
0.09
0.06
0.00
0.66
1.00
0.00

0.35
0.29
1.00
1.00
0.59
1.00
1.00
0.00
0.00
0.21
1.00
1.00
0.40
1.00
1.00
0.00
0.00
0.56
0.00
0.01
1.00
0.00
0.13
0.00
1.00
0.09
0.00
1.00
1.00
0.01
0.87
1.00
0.00
0.52
0.51
0.00
0.14
0.00
0.00
0.91
0.12
0.10
0.15
0.09
1.00
1.00
0.05
0.45

1.00
1.00
0.08
0.00
0.03
0.93
0.00
0.00
0.93
0.83
1.00
0.00
1.00
0.00
0.63
0.00
0.24
1.00
1.00
1.00
0.38
1.00
0.92
0.59
0.00
1.00
0.01
0.64
0.29
0.32
0.00
0.10
1.00
0.49
0.28
0.55
1.00
0.02
0.00
0.00
0.42
0.02
0.00
1.00
1.00
0.37
0.00
0.01

1.00
1.00
0.50
1.00
1.00
0.70
0.49
0.38
1.00
0.48
1.00
0.97
1.00
0.50
0.09
0.58
0.00
0.43
0.07
1.00
0.00
0.00
1.00
0.93
0.00
1.00
0.01
0.06
0.28
1.00
0.18
0.43
0.01
0.40
1.00
0.29
0.73
0.70
0.85
0.00
0.10
1.00
0.31
1.00
1.00
0.41
1.00
1.00

0.24
1.00
1.00
0.46
0.11
0.26
1.00
0.15
0.33
1.00
1.00
0.57
0.01
0.00
0.00
0.31
1.00
1.00
0.01
1.00
0.40
1.00
0.00
0.00
0.21
0.17
0.08
0.00
1.00
0.00
0.00
0.01
0.00
0.83
0.00
0.01
0.39
0.68
1.00
0.00
1.00
0.58
1.00
0.00
1.00
0.00
0.93
0.39

0.00
0.00
0.02
0.00
0.00
0.00
0.55
0.00
0.07
1.00
0.24
0.00
0.30
0.01
0.00
0.97
0.54
1.00
0.11
0.46
0.00
0.01
0.32
0.00
0.01
0.01
1.00
0.00
0.00
0.48
1.00
0.31
0.99
0.04
0.69
0.03
0.99
1.00
1.00
0.06
0.04
0.31
0.00
0.03
1.00
0.33
0.35
0.01

0.77
0.39
1.00
1.00
0.00
0.04
0.53
0.00
0.00
0.07
0.96
0.00
0.00
0.00
0.68
0.00
0.00
0.09
0.68
0.21
0.88
0.94
0.56
0.00
0.98
1.00
0.34
0.00
0.12
0.00
0.82
1.00
0.01
0.01
0.02
0.00
0.02
0.00
0.01
0.41
0.59
0.00
0.12
0.88
0.00
0.00
0.11
0.70

0.01
0.74
1.00
0.10
0.53
1.00
0.00
0.00
0.00
0.04
1.00
0.01
1.00
0.01
0.51
1.00
0.00
1.00
1.00
0.88
0.79
0.10
0.00
0.64
0.14
0.00
0.21
1.00
0.01
0.60
0.68
0.39
1.00
1.00
0.17
1.00
0.32
1.00
0.05
1.00
0.51
0.01
0.00
0.00
0.00
0.69
0.58
0.00

0.01
0.03
0.08
0.15
0.69
0.11
0.00
0.83
1.00
0.00
0.00
0.00
1.00
0.00
0.26
1.00
0.00
0.08
0.05
0.01
1.00
0.37
0.56
1.00
0.17
1.00
0.00
0.19
1.00
0.00
0.03
0.41
0.94
0.00
1.00
0.23
0.00
1.00
0.00
1.00
0.01
0.28
0.87
0.69
0.14
0.00
0.00
0.00

0.68
0.32
0.71
0.15
0.22
1.00
0.00
1.00
0.00
0.00
0.13
0.79
1.00
0.08
0.41
0.22
0.00
0.09
0.36
0.00
0.02
1.00
0.03
0.02
0.36
0.46
0.21
0.00
0.01
0.04
0.00
1.00
0.00
0.16
0.39
1.00
0.81
0.81
0.00
1.00
1.00
0.03
0.17
0.46
0.19
0.00
0.00
0.24

0.11
0.84
0.36
0.30
1.00
0.55
1.00
0.06
1.00
1.00
0.81
0.00
0.00
0.58
1.00
1.00
0.30
0.78
0.76
0.00
0.06
1.00
0.45
0.08
0.72
0.01
0.25
1.00
0.93
0.32
0.12
1.00
1.00
1.00
1.00
1.00
0.00
0.82
1.00
0.90
1.00
0.00
0.00
0.34
0.83
1.00
0.01
1.00

0.01
0.53
0.92
0.63
0.00
0.05
0.00
0.82
0.01
0.53
0.00
0.22
0.00
1.00
0.00
0.28
0.78
0.01
0.74
1.00
0.02
0.00
0.00
1.00
1.00
0.00
1.00
0.00
0.00
1.00
0.00
1.00
0.20
1.00
1.00
0.94
1.00
0.59
0.00
1.00
1.00
0.13
0.04
0.64
1.00
0.51
0.75
0.00

1.00
0.04
1.00
0.00
0.55
1.00
0.21
0.70
0.00
0.00
1.00
0.97
0.42
0.00
0.38
0.04
1.00
1.00
0.00
1.00
1.00
0.00
1.00
0.36
1.00
0.04
0.00
1.00
0.13
0.00
0.89
1.00
0.00
0.24
0.20
0.00
0.30
1.00
0.05
0.00
0.00
0.00
0.69
0.58
1.00
1.00
0.00
0.88

1.00
0.00
0.00
0.01
0.00
1.00
1.00
0.05
0.28
1.00
0.00
0.00
0.66
0.35
0.34
0.19
1.00
0.24
0.05
1.00
0.00
0.99
0.64
0.39
0.04
1.00
1.00
0.74
0.00
0.00
0.34
0.66
0.49
1.00
0.04
1.00
1.00
0.01
0.00
0.00
0.93
0.76
0.38
0.00
1.00
0.03
0.77
0.20

0.20
0.19
0.95
0.40
1.00
0.00
1.00
0.52
0.00
0.00
0.00
0.00
0.00
0.00
0.00
1.00
0.00
0.03
0.00
1.00
0.00
0.54
1.00
0.28
0.37
0.37
1.00
0.67
1.00
1.00
0.27
1.00
1.00
0.00
0.70
0.41
0.16
1.00
0.99
1.00
0.00
1.00
0.00
0.29
1.00
0.29
1.00
0.00

1.00
0.00
1.00
1.00
0.00
0.00
0.00
1.00
0.00
1.00
0.21
1.00
0.14
0.00
0.04
0.01
0.65
0.18
0.92
0.01
0.05
0.00
0.00
0.30
0.00
0.11
0.11
0.00
0.17
0.02
0.00
0.00
1.00
0.20
0.14
0.92
0.00
0.00
0.00
0.00
0.07
1.00
0.42
0.41
0.66
1.00
0.01
0.00

0.58
0.58
1.00
0.03
0.00
0.00
0.66
0.00
0.00
0.13
0.09
0.00
0.77
0.01
0.05
0.53
0.44
0.00
0.26
0.67
1.00
1.00
0.05
1.00
1.00
0.09
0.57
1.00
1.00
0.00
0.00
0.00
0.00
0.07
0.00
0.03
1.00
1.00
0.00
0.01
0.00
0.91
0.97
0.17
0.60
0.00
0.40
0.00

0.22
0.00
1.00
0.56
0.01
1.00
0.21
0.21
0.00
0.88
0.00
0.00
0.01
1.00
0.00
0.73
0.19
1.00
0.83
1.00
0.00
0.61
0.00
1.00
1.00
0.01
0.00
0.02
0.08
0.00
0.97
0.02
1.00
0.00
0.75
0.95
0.00
0.10
0.42
0.02
0.12
0.00
0.03
0.00
0.65
0.08
0.00
0.00

1.00
0.09
0.13
0.90
0.49
0.04
0.21
0.83
0.03
0.13
0.08
0.00
1.00
1.00
0.43
0.00
1.00
1.00
0.64
0.51
0.00
0.00
0.00
1.00
1.00
0.00
0.09
0.06
0.40
0.00
0.00
1.00
1.00
0.71
1.00
0.23
0.76
0.00
1.00
1.00
0.91
0.00
0.02
1.00
0.00
1.00
0.00
1.00

0.02
0.09
1.00
0.52
0.22
0.00
0.12
0.13
0.00
0.97
0.00
0.60
1.00
0.00
1.00
1.00
1.00
0.00
0.72
0.06
0.00
0.00
0.01
1.00
0.00
0.02
1.00
0.00
0.01
1.00
0.61
0.03
0.01
1.00
1.00
0.27
0.00
0.00
0.41
0.58
0.59
1.00
1.00
0.17
0.00
1.00
0.40
0.00

1.00
1.00
0.17
0.00
0.00
0.00
0.84
0.47
0.78
0.15
1.00
0.01
0.09
0.08
0.80
0.29
0.00
0.00
0.00
0.14
0.00
0.01
0.86
0.00
1.00
1.00
1.00
1.00
0.86
1.00
0.00
0.00
0.00
0.00
0.00
0.00
1.00
0.73
0.97
0.08
0.70
0.88
1.00
0.39
0.00
1.00
1.00
0.37

0.00
0.13
0.25
0.00
0.02
0.05
0.00
0.03
0.08
0.96
0.33
0.00
0.08
1.00
0.06
1.00
1.00
1.00
0.29
0.88
0.20
0.00
0.04
0.00
0.02
0.00
0.65
1.00
0.80
1.00
0.00
0.00
0.87
0.01
1.00
0.36
1.00
0.51
0.27
1.00
1.00
0.30
0.24
0.39
0.02
1.00
0.02
0.95

0.75
1.00
1.00
0.05
0.10
0.46
0.47
1.00
0.01
0.09
0.04
1.00
0.00
0.58
0.00
0.91
0.00
1.00
0.31
0.06
0.77
0.16
0.68
0.14
0.06
1.00
0.71
1.00
0.10
0.00
1.00
1.00
1.00
0.89
0.08
1.00
0.16
0.66
0.00
0.95
0.97
0.91
1.00
0.00
1.00
0.82
0.63
1.00

0.90
0.00
0.00
0.47
0.16
0.00
0.00
0.92
0.07
0.39
1.00
0.00
0.33
0.00
1.00
1.00
0.00
0.07
0.00
0.16
0.00
0.00
0.16
1.00
0.00
0.02
0.02
0.00
0.00
0.26
0.00
0.00
0.01
0.00
0.00
0.00
0.00
0.00
0.00
0.00
0.00
0.07
0.05
0.34
0.01
0.11
0.00
0.00

0.00
0.01
0.00
0.07
1.00
0.00
0.00
0.00
0.03
0.00
1.00
0.09
0.78
0.68
1.00
0.00
0.23
0.36
0.01
0.03
0.10
0.00
0.32
0.11
1.00
0.02
0.79
0.23
0.06
0.18
1.00
1.00
0.09
1.00
0.23
0.93
1.00
1.00
0.02
0.38
1.00
0.00
1.00
0.00
0.36
0.00
0.90
0.55

0.00
0.27
0.01
1.00
0.18
1.00
0.00
1.00
0.42
0.04
0.00
1.00
1.00
0.00
0.00
0.01
1.00
1.00
1.00
0.00
0.00
0.84
0.53
0.07
0.67
0.05
0.00
1.00
0.06
0.04
0.01
1.00
1.00
0.01
0.00
1.00
0.02
0.04
1.00
0.18
0.11
0.25
0.39
0.00
0.04
0.89
0.10
0.06

0.09
1.00
0.00
0.42
0.61
0.00
1.00
0.73
0.00
0.42
0.00
0.00
0.69
0.20
0.03
0.14
0.00
0.00
0.69
0.00
0.21
1.00
0.12
0.79
0.05
0.62
1.00
1.00
0.01
0.72
0.00
0.61
0.00
1.00
0.28
0.03
0.36
0.30
1.00
0.82
1.00
0.20
1.00
0.27
0.22
0.00
1.00
1.00

0.00
1.00
0.00
0.02
1.00
0.00
0.43
1.00
0.63
0.25
0.03
0.01
0.00
0.97
0.63
0.24
0.01
0.00
0.41
0.40
1.00
0.00
1.00
0.13
0.01
0.00
1.00
0.00
0.13
0.02
0.00
0.01
0.02
0.08
0.00
0.85
1.00
0.11
0.92
0.98
0.03
0.00
1.00
0.64
1.00
0.63
1.00
0.00

0.27
0.13
0.11
0.60
0.09
0.04
1.00
1.00
0.66
0.01
0.94
1.00
0.08
1.00
0.00
0.44
0.25
0.01
1.00
0.14
1.00
1.00
0.25
1.00
1.00
0.36
0.18
1.00
0.29
0.68
0.00
0.59
0.59
1.00
0.77
0.75
0.00
0.78
1.00
0.92
0.07
0.21
0.03
0.37
0.05
0.61
1.00
0.21

0.01
0.15
0.00
0.56
0.01
0.03
0.11
0.29
0.44
0.47
0.09
0.49
1.00
0.11
0.43
0.28
1.00
1.00
0.17
0.00
0.73
0.02
0.00
0.04
1.00
0.72
0.00
0.85
0.16
0.00
0.01
0.24
0.00
0.69
0.00
1.00
0.05
0.21
0.05
0.00
0.24
0.00
1.00
1.00
0.17
0.08
0.95
0.78

0.82
0.31
0.00
0.51
0.07
0.17
1.00
0.97
0.76
1.00
0.20
0.02
1.00
0.00
0.02
1.00
1.00
1.00
0.00
0.00
1.00
1.00
0.90
1.00
0.35
0.63
1.00
0.67
0.04
0.01
0.00
0.38
0.29
0.00
0.04
0.25
0.33
0.00
0.34
0.88
0.03
0.01
0.88
0.00
0.04
0.00
1.00
0.09

0.00
0.00
1.00
0.02
0.23
0.00
0.00
0.00
1.00
0.66
1.00
1.00
0.74
1.00
1.00
0.00
0.00
1.00
0.40
0.37
1.00
0.38
0.23
0.22
0.56
0.66
0.99
0.01
0.61
1.00
0.50
0.04
1.00
0.74
0.01
0.40
0.07
0.18
0.01
0.00
0.00
0.68
1.00
1.00
0.73
0.87
0.00
0.32

1.00
1.00
0.60
0.01
1.00
1.00
0.21
0.27
1.00
1.00
0.91
0.00
0.82
1.00
0.45
1.00
0.05
0.13
0.00
0.00
0.25
0.50
0.18
0.44
0.95
0.16
0.01
0.01
0.01
1.00
0.48
0.91
0.07
0.73
1.00
0.00
0.08
0.00
1.00
1.00
0.00
1.00
0.01
0.94
0.00
1.00
0.27
0.10

1.00
1.00
0.00
1.00
1.00
0.48
0.88
0.00
0.00
1.00
1.00
0.41
0.07
0.40
0.03
0.00
1.00
1.00
0.62
1.00
0.12
1.00
0.34
1.00
0.00
0.02
1.00
0.23
0.01
0.04
0.60
1.00
1.00
0.01
0.02
1.00
0.91
0.06
1.00
0.00
0.67
0.00
0.00
0.02
0.62
0.00
0.06
0.91

0.36
0.00
0.22
0.01
0.01
0.29
0.01
1.00
0.00
1.00
1.00
0.06
0.01
0.00
0.00
0.00
0.00
1.00
0.12
0.01
0.04
0.36
1.00
0.64
0.19
0.05
0.00
0.02
0.08
0.74
0.62
0.00
1.00
0.02
0.54
0.98
0.90
1.00
0.00
1.00
1.00
0.00
0.24
0.56
0.00
0.39
0.00
0.00

0.08
1.00
0.19
0.01
1.00
0.07
1.00
0.00
0.00
0.77
0.03
1.00
0.78
0.29
0.15
0.00
0.00
0.00
0.81
0.03
0.13
0.00
0.00
0.00
0.40
0.27
0.63
0.04
0.26
1.00
0.56
0.19
1.00
0.66
0.29
1.00
0.99
0.23
0.97
0.10
0.00
1.00
0.66
0.00
0.86
0.00
0.52
1.00

0.91
0.72
1.00
1.00
0.00
0.00
0.00
1.00
0.54
0.00
1.00
1.00
0.64
1.00
0.58
1.00
0.39
0.42
0.97
1.00
0.04
0.23
0.01
0.00
0.01
1.00
0.04
0.00
0.52
0.34
0.94
0.35
0.42
1.00
0.11
0.93
0.03
0.00
0.05
1.00
0.02
1.00
0.12
0.19
0.92
0.00
0.01
1.00

0.02
0.14
1.00
0.83
0.01
0.01
0.01
1.00
0.44
0.00
0.00
0.66
0.08
0.70
1.00
0.11
0.00
0.39
0.53
0.03
0.06
0.33
1.00
0.30
1.00
1.00
1.00
0.11
1.00
0.13
1.00
0.49
0.06
0.72
0.97
0.91
0.42
0.27
1.00
0.30
0.00
0.22
0.46
1.00
0.00
0.64
0.01
0.01

0.90
0.53
1.00
0.54
0.42
0.00
0.02
0.84
0.90
0.33
0.33
0.73
1.00
0.31
0.03
0.85
0.71
1.00
0.45
0.52
1.00
0.24
1.00
0.70
1.00
0.68
0.01
0.54
0.30
0.17
0.46
0.00
0.91
1.00
0.03
1.00
0.55
1.00
0.18
0.74
1.00
1.00
0.18
0.92
0.08
0.30
0.34
0.02

1.00
1.00
0.00
1.00
0.12
0.67
0.45
0.03
0.00
0.03
0.00
0.04
1.00
0.00
1.00
0.22
0.91
0.85
0.76
0.00
0.00
0.00
0.11
0.31
0.00
0.23
0.00
0.00
0.00
0.00
0.03
1.00
0.13
1.00
0.60
0.03
0.13
0.11
0.08
0.00
0.01
0.37
0.15
0.77
0.00
0.12
0.16
0.43

0.70
1.00
0.73
0.34
0.00
0.00
0.00
0.01
0.13
1.00
0.07
0.18
0.03
0.09
0.01
1.00
0.02
0.23
0.00
0.00
0.00
0.71
1.00
0.02
0.05
0.00
0.18
0.00
0.07
0.12
0.00
0.00
0.00
0.02
0.05
0.00
0.00
0.01
0.34
0.03
0.00
0.96
0.99
1.00
0.03
0.64
0.09
0.02

0.03
1.00
0.07
0.18
0.08
1.00
0.00
1.00
0.18
0.02
0.06
0.01
1.00
0.06
0.09
0.37
0.17
0.93
0.11
1.00
0.08
0.12
0.00
0.02
0.57
0.00
0.00
0.05
0.55
0.39
0.01
0.27
0.02
0.15
0.88
0.01
0.00
0.00
1.00
0.00
1.00
0.27
1.00
0.07
0.07
1.00
0.16
0.54

1.00
0.02
0.85
0.00
0.01
0.09
0.06
0.00
0.08
1.00
0.63
1.00
1.00
0.46
0.93
0.00
0.94
0.02
0.06
0.19
1.00
1.00
0.00
0.00
0.00
1.00
0.00
0.00
0.01
0.25
0.43
0.21
1.00
0.09
0.32
0.39
1.00
1.00
0.23
0.27
0.02
1.00
0.82
0.06
1.00
1.00
1.00
1.00

0.00
1.00
1.00
0.01
0.00
0.02
1.00
0.61
0.00
0.12
0.40
0.01
0.01
0.00
0.98
0.02
1.00
0.07
1.00
0.04
0.87
0.05
0.00
0.00
1.00
0.04
0.03
0.65
1.00
0.68
0.25
0.00
0.00
0.04
1.00
0.28
1.00
1.00
0.00
0.00
0.09
0.03
0.00
0.01
0.00
0.04
0.57
0.00

0.54
0.02
0.54
0.76
1.00
1.00
0.00
0.22
0.12
0.21
0.06
1.00
0.02
0.19
0.68
1.00
1.00
0.00
0.75
0.05
1.00
0.09
0.02
0.00
0.00
0.22
0.00
0.00
0.54
1.00
0.39
0.98
0.64
0.01
1.00
1.00
0.72
0.10
0.00
0.99
0.35
1.00
0.00
1.00
0.10
1.00
1.00
0.09

0.00
0.00
0.44
0.67
0.31
0.70
0.14
0.30
0.18
0.39
1.00
0.27
0.63
0.65
0.05
0.92
0.00
0.06
0.00
0.30
0.74
0.77
0.02
0.57
1.00
1.00
1.00
0.13
0.00
0.04
0.49
1.00
0.46
0.08
0.12
0.01
0.02
0.21
0.46
1.00
0.21
0.03
0.49
0.11
0.16
0.01
0.00
1.00

0.00
0.93
0.01
1.00
0.42
0.00
1.00
1.00
1.00
0.89
1.00
1.00
0.42
0.21
1.00
1.00
1.00
1.00
1.00
1.00
0.00
0.01
0.71
1.00
0.16
1.00
0.92
0.46
0.54
1.00
1.00
0.00
0.36
1.00
0.51
0.25
1.00
0.00
0.05
0.00
0.17
0.64
1.00
0.00
0.78
0.39
0.01
0.01

0.03
1.00
0.63
0.34
1.00
0.35
1.00
0.27
1.00
1.00
0.19
1.00
0.18
0.00
0.00
0.00
0.00
1.00
0.10
0.29
0.50
0.37
1.00
0.04
1.00
0.39
0.93
0.41
0.14
0.01
0.00
1.00
0.48
0.00
0.61
0.00
0.84
1.00
1.00
0.01
0.00
1.00
0.00
1.00
0.67
0.00
0.72
0.51

0.00
0.48
1.00
1.00
0.76
0.00
0.35
0.68
0.00
0.00
0.00
0.00
0.00
0.54
0.50
0.01
0.24
0.26
0.09
0.45
1.00
0.00
0.00
0.00
0.10
0.03
1.00
0.82
0.14
0.00
0.00
0.00
0.00
0.59
0.00
0.00
0.00
0.00
0.28
0.70
0.63
0.00
0.84
1.00
0.00
0.77
0.14
0.00

0.64
0.66
0.12
1.00
0.02
1.00
0.44
1.00
0.93
1.00
0.00
0.00
0.00
0.41
0.27
0.13
0.13
0.01
0.02
0.01
0.85
0.63
1.00
1.00
0.00
1.00
0.01
0.60
0.00
0.48
0.52
0.74
0.00
0.04
1.00
0.01
0.07
1.00
0.02
0.00
0.00
1.00
0.00
0.51
0.02
0.00
1.00
0.93

0.00
0.07
0.00
0.00
1.00
1.00
0.06
0.60
0.14
0.01
1.00
1.00
0.01
1.00
0.00
0.00
0.00
0.00
0.00
0.05
0.19
0.00
0.88
1.00
0.71
0.06
0.06
0.45
1.00
1.00
0.58
0.00
1.00
0.95
1.00
0.00
0.68
0.31
0.30
0.52
0.05
0.00
1.00
1.00
0.29
0.00
0.04
0.17

0.02
0.00
0.82
0.00
0.09
0.01
1.00
0.00
0.00
0.19
1.00
0.24
0.54
0.36
0.00
1.00
0.00
0.40
0.00
1.00
1.00
0.01
0.16
0.00
0.00
0.00
0.00
0.00
0.08
0.67
0.72
0.00
0.42
0.00
0.02
0.45
0.19
0.91
1.00
0.21
1.00
1.00
0.82
0.00
0.00
0.04
0.00
0.00

1.00
0.00
0.00
0.01
1.00
0.40
0.75
0.86
0.00
0.00
0.00
0.39
0.30
0.00
1.00
0.80
0.00
0.03
0.01
0.00
0.07
0.41
0.87
0.02
0.00
1.00
0.10
0.01
0.55
0.05
0.00
0.13
0.41
0.03
1.00
0.00
1.00
0.63
0.83
0.00
0.30
0.03
0.85
0.42
0.01
1.00
1.00
0.00

0.89
0.03
0.00
0.00
0.00
0.22
0.80
1.00
0.11
0.38
0.94
1.00
1.00
0.96
0.63
1.00
0.00
0.01
0.00
0.14
0.03
0.03
0.00
1.00
0.98
0.52
0.29
0.28
1.00
0.81
1.00
0.54
0.68
0.88
0.68
0.00
0.00
1.00
0.00
0.84
1.00
1.00
1.00
0.36
0.65
0.01
1.00
0.00

1.00
0.61
1.00
0.00
0.08
1.00
0.67
0.00
0.87
0.44
1.00
0.37
0.66
0.00
0.65
1.00
1.00
0.55
0.03
0.00
0.95
0.17
0.86
0.20
0.02
1.00
0.04
1.00
0.00
0.00
0.00
1.00
0.37
1.00
0.23
0.00
0.91
0.00
0.06
0.76
0.00
0.00
0.11
0.18
0.09
0.10
0.00
1.00

0.99
0.61
0.05
0.00
0.82
0.00
0.01
0.00
0.08
0.41
0.02
0.07
0.00
0.34
0.00
0.29
0.00
0.77
0.70
0.02
0.08
0.01
1.00
0.87
1.00
0.00
0.61
0.00
1.00
0.00
1.00
0.02
0.02
1.00
0.00
0.04
1.00
0.46
0.33
0.00
0.78
0.19
0.03
0.00
0.49
0.14
0.91
0.00

1.00
0.00
1.00
1.00
0.91
0.24
1.00
0.04
0.68
1.00
1.00
0.00
0.59
0.00
1.00
0.36
1.00
0.01
0.68
0.56
1.00
0.00
0.04
0.97
0.06
0.00
0.14
1.00
0.23
0.00
0.33
0.04
0.91
0.88
0.18
0.80
1.00
0.02
0.01
1.00
0.03
1.00
1.00
0.16
0.77
0.40
1.00
1.00

0.38
0.48
0.07
0.25
0.58
0.01
0.70
0.86
1.00
0.77
1.00
0.00
0.13
1.00
0.55
0.05
0.07
0.01
0.46
0.02
1.00
0.00
0.25
1.00
0.00
0.10
0.19
0.01
0.02
1.00
0.00
0.03
1.00
0.62
1.00
0.00
0.05
0.42
0.02
1.00
1.00
0.38
1.00
0.01
0.00
0.01
0.32
1.00

1.00
0.14
0.64
0.00
0.37
0.68
0.44
0.00
0.28
0.53
0.00
0.30
0.00
1.00
0.34
0.04
1.00
1.00
0.30
1.00
1.00
1.00
0.71
1.00
0.01
1.00
0.12
0.00
0.00
1.00
0.09
0.00
0.00
0.11
0.00
1.00
0.40
0.68
0.02
0.00
0.82
0.30
0.00
0.66
0.20
0.10
0.26
0.00

0.00
0.57
0.34
0.00
0.08
0.02
0.77
0.00
0.68
1.00
0.70
0.04
1.00
0.00
1.00
0.00
0.49
0.40
0.45
0.00
1.00
0.01
0.00
0.00
0.00
1.00
0.01
1.00
1.00
0.71
1.00
1.00
1.00
0.86
0.52
1.00
0.08
0.11
0.00
0.96
0.89
0.69
0.00
0.24
0.00
0.10
0.00
0.00

1.00
0.81
0.07
0.95
0.49
0.83
0.00
0.01
0.23
0.00
0.05
1.00
0.00
0.07
0.00
1.00
1.00
1.00
1.00
0.04
0.00
0.00
0.70
0.02
1.00
0.00
0.19
1.00
0.19
0.00
0.03
1.00
1.00
0.00
0.38
1.00
0.00
0.00
1.00
0.00
1.00
0.01
0.38
0.15
1.00
0.82
0.00
0.46

0.69
0.24
1.00
0.77
1.00
0.00
0.27
0.01
0.00
0.00
1.00
0.00
0.42
0.02
1.00
0.42
0.00
0.17
0.59
0.73
0.46
1.00
0.12
0.42
0.10
0.81
0.00
0.38
1.00
0.64
0.41
0.74
1.00
0.02
1.00
1.00
0.00
0.07
0.01
0.73
0.10
0.51
0.88
1.00
1.00
0.00
1.00
0.00

1.00
0.43
1.00
0.89
1.00
0.13
0.16
0.77
0.00
1.00
0.66
1.00
1.00
0.85
1.00
0.77
0.34
1.00
1.00
1.00
1.00
1.00
1.00
1.00
0.22
0.52
1.00
0.94
1.00
0.29
0.26
1.00
1.00
0.33
0.59
1.00
1.00
0.01
0.73
1.00
0.06
0.27
0.59
0.12
0.00
1.00
0.77
1.00

1.00
1.00
1.00
0.23
0.70
0.57
0.24
1.00
0.61
0.59
0.83
0.57
1.00
1.00
0.64
0.67
1.00
0.98
1.00
1.00
1.00
0.22
0.64
0.35
0.71
0.83
1.00
1.00
1.00
1.00
1.00
0.29
0.95
0.03
1.00
0.71
1.00
1.00
1.00
0.39
1.00
0.15
1.00
1.00
0.75
1.00
1.00
1.00

1.00
1.00
0.03
0.05
1.00
0.04
1.00
0.44
1.00
1.00
1.00
1.00
1.00
1.00
1.00
0.14
1.00
1.00
1.00
1.00
0.22
0.72
1.00
1.00
0.57
0.83
1.00
1.00
0.59
0.66
1.00
0.74
0.83
1.00
1.00
0.95
1.00
1.00
0.68
1.00
1.00
1.00
0.44
0.19
1.00
0.62
1.00
0.90

1.00
1.00
0.00
1.00
0.34
1.00
0.32
0.01
0.28
1.00
0.42
0.68
1.00
1.00
0.54
0.48
1.00
1.00
0.91
0.95
1.00
0.27
1.00
1.00
0.38
1.00
0.79
1.00
1.00
1.00
1.00
0.60
0.76
1.00
1.00
1.00
0.46
0.24
1.00
0.42
0.87
1.00
0.16
0.87
0.74
0.57
1.00
0.25

1.00
0.77
1.00
0.58
0.44
1.00
0.22
1.00
0.77
0.15
0.00
0.03
0.02
0.00
0.00
0.06
1.00
1.00
0.02
0.10
0.00
0.01
0.00
0.00
0.02
0.00
0.00
0.00
0.00
0.37
0.87
0.07
0.00
0.00
0.16
0.02
1.00
0.77
0.01
0.77
1.00
0.13
0.14
0.05
0.01
0.00
0.44
1.00

0.00
0.00
0.01
0.00
1.00
1.00
0.68
0.05
0.38
0.00
0.00
0.05
0.66
0.00
0.87
0.00
0.95
0.99
0.68
0.00
1.00
0.37
0.22
1.00
0.02
0.82
0.00
0.00
0.00
0.00
1.00
0.11
0.59
0.00
0.94
0.55
0.00
0.00
0.00
1.00
0.47
0.77
1.00
0.00
0.00
0.00
1.00
0.53

0.22
1.00
1.00
0.32
0.04
0.02
0.00
0.00
0.01
0.32
1.00
0.01
0.57
1.00
1.00
0.10
1.00
1.00
0.76
0.29
0.41
0.62
0.00
0.19
0.00
1.00
1.00
0.39
0.48
0.04
1.00
0.52
0.77
0.43
0.00
0.36
0.00
1.00
0.35
0.00
1.00
0.35
0.00
0.08
0.21
0.48
1.00
0.90

0.82
0.00
1.00
0.89
0.00
1.00
0.07
0.54
0.02
0.12
0.00
0.76
0.68
0.66
0.80
0.26
0.92
0.83
0.00
0.00
1.00
0.02
1.00
1.00
0.26
0.08
0.00
1.00
0.14
0.51
0.49
1.00
0.21
0.00
0.38
0.74
0.34
0.87
0.32
1.00
1.00
0.00
0.00
0.00
0.31
0.71
0.04
0.00

0.00
0.00
0.00
0.07
0.00
0.00
0.00
1.00
0.02
0.00
0.01
0.00
0.00
0.78
0.11
0.00
0.19
1.00
0.06
0.01
0.33
0.00
1.00
0.05
0.26
1.00
0.19
0.29
0.00
1.00
1.00
0.18
0.44
0.44
0.00
1.00
0.57
0.01
0.00
1.00
0.00
0.68
1.00
1.00
1.00
0.60
0.21
0.23

1.00
0.00
0.07
0.78
0.40
0.26
0.00
0.00
0.00
0.48
0.00
1.00
1.00
0.58
0.26
0.00
0.00
0.03
0.74
0.58
0.00
0.00
1.00
0.61
0.00
0.00
0.73
1.00
0.94
0.00
0.10
0.34
0.24
0.00
1.00
0.19
0.77
0.09
1.00
0.00
0.00
0.01
0.57
0.65
0.00
0.00
0.65
0.78

0.50
1.00
0.06
0.30
0.00
0.03
0.19
0.05
1.00
0.10
0.90
0.00
0.00
0.51
0.49
0.00
0.00
0.89
0.00
0.41
0.00
0.00
0.07
0.74
1.00
0.06
0.00
1.00
0.44
1.00
1.00
0.56
0.00
0.00
0.00
0.10
1.00
0.15
0.00
1.00
0.28
0.01
0.29
0.50
0.11
0.73
1.00
0.01

0.74
0.27
1.00
0.67
1.00
0.49
1.00
0.18
0.82
1.00
1.00
0.00
0.12
0.55
0.56
0.18
1.00
0.08
1.00
0.01
0.72
1.00
0.00
0.48
0.03
0.40
1.00
0.00
0.68
0.00
1.00
0.01
1.00
0.07
0.00
0.02
0.93
1.00
0.00
0.00
0.00
0.00
0.00
0.00
0.00
0.00
1.00
0.22

0.00
0.13
0.42
1.00
0.01
0.00
1.00
0.74
0.07
0.01
0.89
1.00
0.35
0.13
0.00
0.08
0.00
1.00
0.02
0.00
0.41
1.00
0.00
0.00
0.00
1.00
0.00
0.41
0.25
0.14
1.00
0.00
1.00
0.36
0.00
0.00
0.01
0.00
1.00
0.60
1.00
0.11
0.00
1.00
0.86
0.18
0.39
0.01

0.03
1.00
0.00
0.39
0.47
1.00
0.00
0.00
0.56
0.30
0.31
0.11
0.21
0.05
0.01
0.10
0.81
0.57
0.63
0.23
1.00
0.00
0.20
0.14
0.00
0.89
0.00
1.00
0.00
0.02
0.52
0.01
0.12
1.00
0.00
0.00
0.07
1.00
0.00
0.37
0.21
0.00
0.66
0.00
0.00
0.00
0.03
1.00

0.01
1.00
1.00
1.00
0.04
0.01
0.00
0.80
1.00
0.00
0.05
0.01
0.87
1.00
1.00
0.22
0.09
0.00
0.64
0.45
0.57
1.00
0.00
0.02
0.32
1.00
0.82
0.00
0.00
0.00
0.02
0.33
0.45
0.01
0.46
0.51
0.68
1.00
1.00
0.96
0.55
0.04
1.00
0.02
0.03
0.20
0.10
0.00

1.00
0.00
0.00
0.24
1.00
0.33
0.00
0.52
0.08
0.00
0.41
0.07
0.90
1.00
0.08
1.00
1.00
1.00
1.00
1.00
0.31
0.84
0.85
0.00
0.00
0.08
0.00
1.00
0.00
0.41
0.00
0.64
1.00
0.74
0.00
0.20
0.11
0.25
0.00
0.50
1.00
0.72
0.01
0.00
0.01
1.00
1.00
0.00

1.00
1.00
0.89
0.01
0.05
1.00
0.87
0.52
1.00
1.00
0.39
0.02
1.00
0.68
0.67
1.00
1.00
0.04
0.02
0.01
1.00
0.00
1.00
0.01
1.00
0.00
0.00
1.00
1.00
0.11
1.00
0.00
0.00
1.00
0.34
0.83
0.42
1.00
0.76
0.12
1.00
1.00
0.75
1.00
0.30
0.18
0.96
0.43

0.70
0.13
0.03
0.02
0.00
0.64
0.30
0.03
0.10
0.68
0.10
0.87
1.00
1.00
0.97
0.00
0.79
0.06
1.00
0.00
0.72
0.01
0.00
0.01
0.23
0.00
0.63
0.23
1.00
1.00
0.02
0.42
0.57
0.00
0.90
0.43
0.03
0.00
0.01
0.55
0.67
0.52
0.01
1.00
0.00
0.01
0.39
0.01

0.06
1.00
1.00
0.02
0.00
0.06
1.00
1.00
0.24
1.00
0.50
0.00
0.73
1.00
0.23
0.00
0.03
1.00
0.40
0.00
0.00
0.00
1.00
0.01
0.52
0.27
0.07
1.00
0.30
0.00
0.24
0.37
0.43
0.60
0.76
1.00
0.09
0.05
1.00
1.00
0.70
1.00
0.55
0.55
0.00
0.00
0.00
1.00

1.00
0.00
0.67
1.00
0.08
0.05
0.19
0.28
1.00
1.00
0.95
0.01
0.18
1.00
0.00
0.97
0.00
1.00
0.00
1.00
0.00
0.60
0.01
0.75
0.00
0.71
0.39
1.00
0.47
1.00
0.44
1.00
0.70
0.00
0.00
0.52
0.05
0.00
0.01
0.18
0.00
0.11
0.00
0.02
0.01
0.67
0.39
0.03

0.00
1.00
1.00
0.00
0.11
1.00
1.00
0.85
0.16
1.00
0.00
1.00
0.06
1.00
0.00
0.64
0.14
1.00
0.76
0.06
0.48
0.12
0.03
0.00
0.01
0.80
1.00
1.00
0.13
0.00
0.00
0.06
1.00
0.00
1.00
0.07
0.00
0.25
0.00
1.00
1.00
0.00
1.00
0.33
0.57
1.00
1.00
0.00

0.00
0.70
1.00
1.00
0.00
1.00
0.08
0.05
0.31
0.30
0.99
0.67
0.10
0.03
0.21
0.14
0.97
1.00
0.05
0.00
1.00
0.78
1.00
1.00
1.00
0.00
1.00
1.00
0.00
0.03
0.94
1.00
0.25
0.89
0.77
0.04
0.00
0.00
0.36
1.00
0.98
0.41
0.02
0.00
1.00
0.00
0.05
1.00

0.84
0.16
0.30
1.00
0.35
0.03
0.65
0.37
0.00
1.00
0.54
0.96
0.00
0.01
0.53
0.53
1.00
1.00
1.00
1.00
0.66
0.00
0.04
1.00
0.00
0.00
1.00
0.02
0.00
0.35
1.00
0.02
1.00
0.03
0.16
1.00
1.00
0.00
0.00
0.00
0.27
1.00
0.34
1.00
0.70
0.00
0.41
0.18

0.24
0.00
0.00
0.31
0.97
0.00
0.11
0.02
0.73
0.00
0.03
0.27
0.90
0.74
1.00
0.00
0.00
0.97
1.00
0.00
0.00
0.18
0.01
0.30
0.02
0.00
0.00
0.00
0.04
0.24
0.16
1.00
0.00
0.23
0.24
0.03
0.00
0.01
0.02
1.00
0.22
0.00
0.01
0.04
0.03
0.00
0.01
0.70

1.00
0.49
0.11
0.01
0.00
0.84
0.05
1.00
1.00
0.10
0.00
1.00
0.85
1.00
0.15
1.00
1.00
0.27
0.35
0.73
0.28
0.00
0.60
0.49
0.13
0.41
0.21
0.02
0.49
0.93
0.05
0.16
0.00
1.00
0.24
0.00
0.05
1.00
0.37
0.05
0.01
0.08
1.00
0.00
0.42
0.91
1.00
1.00

0.73
1.00
1.00
1.00
0.26
1.00
0.00
1.00
0.22
1.00
0.00
0.00
0.09
0.03
0.16
0.02
0.00
0.00
0.42
0.00
0.02
0.60
1.00
0.00
1.00
0.06
0.28
0.69
1.00
0.02
0.14
0.00
0.02
0.16
0.55
0.26
0.06
0.43
0.00
0.00
0.97
0.01
0.88
1.00
1.00
0.22
0.26
0.32

1.00
1.00
1.00
0.40
0.19
0.48
1.00
1.00
0.00
0.00
1.00
1.00
0.40
0.00
0.00
0.20
0.31
0.87
1.00
1.00
0.07
0.00
0.00
1.00
1.00
0.76
0.66
1.00
0.03
0.01
0.23
0.49
1.00
0.01
1.00
1.00
0.46
1.00
0.00
1.00
0.02
1.00
0.68
0.01
0.05
0.25
1.00
1.00

0.00
0.68
0.03
0.35
1.00
1.00
0.02
0.36
0.10
1.00
0.00
0.24
1.00
0.18
0.00
0.99
0.69
0.87
0.68
0.00
0.00
1.00
0.76
0.15
0.00
0.00
0.17
0.41
1.00
1.00
0.91
0.00
0.21
0.02
0.77
0.37
0.96
0.00
1.00
0.01
0.00
0.15
0.00
0.07
1.00
0.00
0.01
1.00

1.00
0.74
1.00
0.94
0.63
0.68
0.82
1.00
0.07
0.04
0.51
0.02
0.78
0.11
1.00
0.00
0.00
1.00
0.10
0.00
1.00
1.00
0.26
0.24
0.18
0.00
0.01
0.00
0.10
1.00
0.90
0.00
0.77
0.00
1.00
0.52
0.59
0.00
0.01
0.10
0.00
0.07
1.00
0.74
0.00
0.53
0.00
0.00

0.25
0.45
0.00
0.45
0.00
0.22
0.00
1.00
1.00
0.11
0.03
0.00
0.00
0.80
0.00
0.39
0.44
0.08
0.46
1.00
0.21
1.00
0.76
0.45
0.00
0.27
0.78
0.01
1.00
0.00
1.00
1.00
0.93
0.92
1.00
0.98
0.01
0.09
0.01
0.01
0.00
1.00
0.00
0.01
0.01
0.29
1.00
0.02

0.01
1.00
0.64
1.00
0.00
0.00
0.00
0.00
1.00
0.00
0.80
0.00
0.00
0.01
0.08
0.69
0.30
0.25
0.28
1.00
1.00
0.00
0.18
1.00
0.01
1.00
0.45
1.00
0.47
0.05
0.25
0.20
1.00
0.02
0.09
0.00
0.00
0.31
0.00
0.27
0.12
0.05
0.70
1.00
0.02
0.00
0.15
0.64

0.00
0.00
0.00
0.00
0.10
0.00
0.44
0.17
0.90
0.68
0.20
0.00
1.00
0.23
0.00
1.00
0.00
0.43
0.00
0.05
0.00
1.00
1.00
1.00
0.00
0.02
1.00
1.00
0.02
0.00
0.41
1.00
0.03
0.12
0.79
1.00
0.40
1.00
0.61
0.77
0.05
0.00
1.00
1.00
0.40
0.05
0.41
0.90

0.60
0.86
0.51
0.38
0.16
0.05
0.00
1.00
0.24
1.00
0.00
0.21
0.63
0.00
0.61
1.00
0.06
0.02
0.12
0.95
0.00
0.10
0.20
1.00
0.00
1.00
0.70
0.17
0.48
0.25
1.00
1.00
0.12
0.89
0.00
1.00
1.00
1.00
0.00
0.00
0.60
0.36
0.00
0.00
0.12
0.43
1.00
0.87

1.00
1.00
1.00
0.22
0.26
0.03
0.00
0.42
0.03
0.02
1.00
0.33
1.00
0.07
0.15
0.85
0.17
1.00
0.01
0.65
0.33
0.13
0.72
0.00
1.00
0.00
0.01
0.00
0.05
0.43
0.14
0.00
0.00
1.00
1.00
0.77
0.00
0.00
0.00
1.00
0.01
0.70
0.00
0.38
1.00
0.18
1.00
1.00

1.00
1.00
0.03
0.82
0.01
0.96
1.00
1.00
0.86
0.00
0.12
0.59
0.03
0.75
1.00
0.62
1.00
0.00
0.97
1.00
0.11
1.00
1.00
0.26
1.00
1.00
0.28
1.00
0.37
1.00
0.04
1.00
1.00
0.01
1.00
0.00
0.00
0.02
0.23
1.00
1.00
0.00
0.00
0.00
1.00
1.00
0.74
0.00

0.00
0.06
1.00
0.03
0.25
0.56
0.20
0.01
1.00
0.11
0.07
0.00
0.00
1.00
0.01
0.42
1.00
0.12
0.10
0.13
0.00
0.01
1.00
0.17
0.77
0.89
0.00
1.00
0.00
1.00
0.00
1.00
0.00
0.03
0.01
0.00
0.06
0.00
0.06
0.01
0.00
0.09
1.00
1.00
1.00
0.41
1.00
0.06

0.43
0.14
0.00
0.45
0.27
1.00
0.00
0.08
0.00
1.00
1.00
1.00
1.00
1.00
0.69
0.69
0.05
1.00
0.21
0.65
1.00
0.07
0.53
0.00
0.34
0.02
0.70
0.03
0.22
0.90
1.00
0.61
1.00
0.49
0.18
0.03
1.00
0.64
0.52
0.16
0.05
0.16
0.43
0.03
0.02
0.27
0.00
0.62

0.67
0.00
0.00
0.03
0.00
1.00
0.00
0.33
0.15
0.00
0.00
0.08
0.00
0.01
1.00
0.77
1.00
0.68
0.17
1.00
0.98
0.10
0.00
1.00
0.05
0.11
0.05
1.00
1.00
0.08
1.00
0.00
0.20
0.00
0.00
0.08
1.00
0.55
0.00
0.78
0.07
0.13
0.00
0.97
0.78
0.99
0.04
1.00

0.07
0.03
1.00
1.00
0.00
0.00
0.77
0.00
1.00
1.00
0.01
0.55
0.00
0.00
0.00
0.00
0.50
0.00
0.95
0.08
1.00
1.00
0.00
1.00
0.01
0.47
0.81
1.00
0.09
0.15
1.00
0.85
0.36
0.00
0.40
0.00
0.01
1.00
0.71
1.00
0.04
0.06
0.64
1.00
0.35
0.82
0.64
1.00

1.00
0.11
0.11
0.20
0.01
0.01
0.00
0.02
1.00
0.11
0.65
0.00
0.00
0.03
0.26
0.30
0.01
0.00
0.00
1.00
0.30
0.00
1.00
0.00
0.01
0.00
1.00
0.05
0.46
0.41
0.00
0.10
0.01
0.00
1.00
0.03
0.14
0.79
0.00
0.00
0.25
0.00
0.04
0.09
0.56
0.00
0.00
0.18

1.00
0.02
1.00
1.00
0.58
0.14
1.00
0.01
1.00
0.03
0.00
0.00
1.00
0.00
0.00
0.67
0.02
0.39
0.94
0.03
1.00
0.00
0.00
0.78
1.00
0.12
0.68
1.00
0.39
0.18
1.00
1.00
1.00
0.07
0.56
0.36
0.61
0.00
1.00
0.00
0.19
1.00
0.09
0.00
0.09
0.22
1.00
0.03

0.00
0.54
0.09
0.43
0.14
1.00
0.00
1.00
0.08
0.19
0.42
1.00
0.02
0.00
1.00
0.48
0.56
1.00
0.50
1.00
0.35
0.00
0.91
0.92
1.00
0.94
1.00
0.59
1.00
0.44
0.00
0.23
0.69
1.00
0.78
0.00
1.00
0.00
0.29
0.00
0.30
1.00
0.60
0.82
0.05
1.00
1.00
1.00

0.92
0.05
0.61
1.00
0.00
0.06
0.08
0.61
0.74
0.00
0.00
0.49
0.05
0.00
0.73
0.68
1.00
0.00
0.23
0.24
0.79
0.67
0.61
1.00
1.00
1.00
0.00
1.00
0.00
1.00
1.00
0.39
0.52
0.01
0.00
0.70
0.41
1.00
0.02
0.62
0.90
0.75
0.10
0.05
1.00
1.00
0.23
1.00

1.00
0.43
1.00
1.00
0.00
0.82
0.05
0.75
0.04
0.00
1.00
0.59
0.09
1.00
1.00
0.37
1.00
0.00
0.76
0.84
0.60
1.00
0.15
1.00
0.79
1.00
0.09
1.00
0.42
1.00
1.00
0.00
0.07
1.00
0.00
0.52
0.93
0.14
0.22
1.00
0.03
0.16
0.08
0.00
0.71
0.00
0.01
1.00

0.18
0.68
0.00
0.59
0.21
1.00
1.00
1.00
1.00
0.00
0.32
1.00
0.01
1.00
1.00
0.04
0.00
0.05
1.00
0.38
0.00
0.00
0.16
0.03
0.24
0.94
1.00
0.00
0.00
0.00
0.00
0.00
0.20
0.00
0.01
1.00
0.49
0.00
0.39
0.00
1.00
0.41
1.00
0.00
0.38
1.00
0.57
1.00

0.00
1.00
0.39
0.00
0.04
0.00
0.00
1.00
1.00
0.89
0.08
0.18
1.00
1.00
0.00
0.00
0.20
0.00
1.00
0.02
1.00
0.17
1.00
0.40
0.08
0.94
0.73
0.07
0.42
0.61
1.00
0.02
0.06
0.05
1.00
0.79
0.66
1.00
1.00
1.00
0.98
0.80
0.72
0.33
0.00
0.31
0.01
0.00

1.00
0.17
1.00
0.40
0.41
1.00
0.00
0.04
1.00
0.13
0.12
0.01
0.19
0.34
0.35
0.83
0.61
0.46
0.92
1.00
1.00
1.00
1.00
0.00
1.00
0.41
1.00
0.90
0.37
0.71
1.00
0.02
0.04
1.00
1.00
0.04
0.15
0.52
0.21
1.00
0.01
0.01
0.00
0.21
0.00
1.00
0.00
0.75

0.00
1.00
0.03
0.00
0.00
1.00
0.82
0.19
0.55
0.00
0.55
0.70
0.36
0.00
1.00
1.00
0.11
0.10
0.32
0.24
0.00
0.08
0.20
0.79
1.00
0.95
0.03
1.00
0.03
0.42
1.00
0.00
0.00
0.02
0.04
0.54
1.00
0.15
0.00
0.11
0.24
1.00
0.88
1.00
0.89
0.00
0.46
0.57

1.00
0.95
0.03
0.63
1.00
0.70
0.00
0.56
0.50
1.00
0.00
0.00
1.00
0.91
0.00
0.71
0.80
0.12
0.21
0.00
0.11
1.00
0.65
0.43
0.16
1.00
0.00
1.00
0.02
0.00
1.00
0.00
0.16
0.00
0.18
1.00
0.00
0.20
0.73
1.00
0.00
1.00
0.01
0.70
0.00
0.07
0.74
0.64

1.00
1.00
1.00
0.00
1.00
0.60
0.00
0.13
0.17
0.00
0.71
0.55
1.00
0.28
0.00
1.00
0.23
0.00
0.49
1.00
0.27
0.00
0.72
1.00
1.00
0.50
1.00
1.00
1.00
0.95
1.00
0.60
0.74
0.97
0.00
0.01
0.05
0.11
0.40
0.00
0.06
0.68
0.00
0.49
0.08
1.00
0.71
1.00

1.00
0.00
0.00
0.37
0.11
1.00
0.00
0.00
0.45
0.00
1.00
0.61
0.13
1.00
1.00
0.00
0.00
1.00
0.19
0.01
0.00
1.00
0.01
0.11
1.00
1.00
0.01
0.84
0.54
0.96
0.59
0.32
0.62
0.00
0.08
0.77
0.26
0.00
0.00
0.00
0.17
0.13
0.25
1.00
0.83
0.28
1.00
0.79

0.01
1.00
0.16
0.25
0.01
0.31
1.00
1.00
1.00
1.00
1.00
1.00
1.00
0.78
1.00
1.00
0.00
0.00
0.74
0.14
1.00
0.24
0.07
1.00
0.09
1.00
0.99
0.27
0.01
0.00
1.00
0.02
1.00
1.00
0.07
0.16
0.00
0.46
0.26
0.00
1.00
0.56
0.13
1.00
0.03
0.04
1.00
1.00

1.00
0.91
0.59
0.06
1.00
0.07
0.76
0.00
0.23
0.54
1.00
0.00
0.50
0.14
0.21
1.00
0.45
0.09
0.32
0.07
0.03
1.00
0.87
1.00
0.00
0.11
0.00
0.02
1.00
0.92
0.00
0.08
0.38
0.88
0.01
0.92
0.00
0.31
0.07
0.01
1.00
0.01
1.00
1.00
0.70
0.13
0.02
0.18

0.00
0.41
0.06
1.00
0.04
0.12
0.50
0.33
1.00
1.00
0.00
0.00
1.00
1.00
1.00
0.05
0.40
0.00
0.11
0.56
0.00
0.60
0.01
0.01
1.00
0.56
0.73
0.90
1.00
1.00
0.28
0.00
0.02
0.71
0.00
1.00
0.03
0.14
1.00
1.00
0.07
0.50
0.75
0.41
0.00
0.00
0.66
1.00

0.01
0.69
1.00
0.00
0.00
0.13
0.00
0.06
0.01
0.68
1.00
0.07
0.32
1.00
0.10
1.00
0.08
1.00
0.49
1.00
0.07
0.03
0.88
0.38
0.94
0.00
1.00
0.10
0.66
1.00
0.00
0.07
0.01
1.00
0.07
1.00
0.00
1.00
0.24
0.09
0.16
0.22
0.51
0.68
0.92
0.81
0.53
0.09

0.86
0.13
0.12
0.45
0.17
0.02
0.16
0.91
0.16
0.17
0.52
0.02
1.00
0.00
1.00
0.84
0.72
0.04
1.00
0.49
0.02
1.00
1.00
1.00
0.64
1.00
0.00
0.45
1.00
0.00
0.12
1.00
0.00
0.27
0.20
0.00
1.00
1.00
0.93
0.18
0.95
0.58
0.15
0.19
1.00
1.00
0.30
1.00

0.24
1.00
0.40
0.30
1.00
1.00
0.31
0.00
0.10
0.05
0.00
0.73
0.02
1.00
0.50
1.00
0.40
1.00
1.00
1.00
0.43
0.01
0.08
0.00
1.00
0.01
0.00
0.39
0.62
1.00
1.00
1.00
0.57
0.30
1.00
1.00
0.16
0.48
1.00
0.81
0.10
0.58
0.04
0.00
0.00
0.11
0.23
0.70

0.63
1.00
0.46
1.00
1.00
0.96
0.63
0.72
0.49
1.00
0.88
0.73
0.41
0.00
0.13
1.00
1.00
0.12
0.65
0.04
0.24
0.30
1.00
1.00
0.00
1.00
0.00
1.00
1.00
0.00
1.00
0.71
0.00
0.00
1.00
0.00
0.38
0.59
0.43
1.00
0.40
1.00
0.47
0.77
0.45
0.61
0.44
1.00

1.00
0.14
1.00
0.00
0.00
0.15
0.38
0.40
0.61
0.00
0.33
0.62
0.04
1.00
0.00
0.01
0.09
0.26
1.00
0.01
0.07
0.95
0.26
0.00
1.00
0.76
0.82
1.00
1.00
0.02
1.00
1.00
0.95
1.00
1.00
0.24
0.14
0.00
0.01
0.79
1.00
1.00
0.00
1.00
0.13
1.00
0.02
0.87

0.36
0.70
1.00
1.00
1.00
0.01
1.00
0.04
1.00
0.14
0.31
1.00
1.00
0.70
1.00
0.29
1.00
1.00
1.00
1.00
0.23
0.03
0.88
0.16
1.00
0.22
0.56
0.98
0.79
0.15
0.01
0.00
1.00
0.00
0.27
0.21
0.00
0.53
0.01
0.49
0.71
1.00
1.00
1.00
0.13
1.00
0.52
0.54

1.00
1.00
0.74
1.00
1.00
1.00
0.11
0.23
0.57
1.00
0.31
1.00
0.02
1.00
0.39
0.41
0.47
1.00
1.00
0.95
1.00
0.92
1.00
1.00
0.71
1.00
0.54
1.00
0.31
0.73
0.27
0.45
1.00
1.00
1.00
0.17
1.00
0.22
1.00
1.00
1.00
1.00
1.00
1.00
1.00
0.00
0.20
1.00

0.09
0.00
0.04
0.88
1.00
0.91
0.00
0.73
0.95
0.26
1.00
1.00
0.00
0.00
1.00
0.01
0.82
0.73
0.63
0.99
0.00
0.95
1.00
0.00
1.00
0.14
1.00
1.00
1.00
1.00
1.00
1.00
1.00
1.00
0.18
0.78
0.06
0.00
0.57
0.01
0.27
1.00
0.00
0.17
1.00
0.71
1.00
0.26

1.00
1.00
0.53
0.00
0.00
1.00
0.48
0.18
0.37
0.58
0.74
0.22
0.39
1.00
1.00
0.98
0.21
0.01
0.64
0.27
0.21
0.11
0.00
1.00
0.00
1.00
0.59
1.00
0.24
0.74
1.00
1.00
0.52
0.26
0.63
0.60
0.26
1.00
0.30
1.00
1.00
0.97
0.90
0.67
1.00
1.00
1.00
1.00

0.43
0.47
1.00
0.30
0.32
0.54
0.57
1.00
1.00
1.00
0.02
0.00
1.00
1.00
0.04
1.00
0.51
1.00
1.00
0.14
0.15
0.05
0.07
0.57
1.00
0.57
0.00
1.00
0.00
0.07
0.56
0.24
0.14
1.00
0.01
0.00
0.35
0.44
0.95
1.00
0.03
0.61
0.03
1.00
0.11
1.00
1.00
1.00

1.00
0.10
0.00
0.64
0.78
1.00
0.39
0.48
1.00
0.34
0.88
1.00
0.29
1.00
0.17
0.97
0.00
0.39
0.75
1.00
1.00
1.00
1.00
0.10
0.39
1.00
0.00
0.10
0.06
0.00
0.60
0.41
1.00
0.89
0.79
1.00
1.00
0.00
0.01
0.00
1.00
1.00
1.00
1.00
0.80
1.00
1.00
1.00

0.89
1.00
0.95
0.18
0.03
0.86
0.12
0.05
0.97
0.01
0.00
0.56
1.00
1.00
0.01
1.00
0.65
0.07
0.05
0.00
0.00
0.00
0.00
0.45
0.15
0.03
0.00
0.79
0.34
0.00
1.00
0.46
0.43
0.00
0.25

Supplementary Table 1: Comparison of gene expression profile between scramble and siARID1A in ES2 cells. Each condition contains triplicate samples.

Supplementary Figure 1. Original WBs. A. Original of WB shown in Figure 1A (boxed area). B. Originals of WBs shown in Figure 2A and 2E. C. Amido black performed on scr. and KD ES-2 cell lysates after Seahorse experiments shown in Figures 2B and 2D showing that equal amounts of cells were subjected to measurement of mitochondrial respiration via SeaHorse. D. Amido black performed on scr. and KD RMG1 cell lysates after Seahorse experiments shown in Figures 2F and 2H (boxed area). E. Original WB shown in Figure 2I (boxed area). F. Amido black performed on empty vector (EV) and overexpressing (OE) TOV-21G cell lysates after Seahorse experiments shown in Figures 2J and 2L.

Supplementary Figure 2. Original WBs. A. Original of WB shown in Figure 1E (boxed area for ES-2, left, and entire WB for RMG1, right). B. Original of WB shown in Figure 4C (boxed area).

Supplementary Figure 3. Colony formation assay. ARID1A-mutated OCCCs derived TOV-21G cell line expressing either Empty Vector (EV) of pcDNA-ARID1A (overexpressing OE) in colony formation assay.
